# Catalytic Asymmetric Transfer Hydrogenation of β,γ-Unsaturated α-Diketones

**DOI:** 10.1021/jacs.4c11070

**Published:** 2024-11-27

**Authors:** Zhifei Zhao, Wennan Dong, Jinggong Liu, Shuang Yang, Andrej Emanuel Cotman, Qi Zhang, Xinqiang Fang

**Affiliations:** † State Key Laboratory of Structural Chemistry, Center for Excellence in Molecular Synthesis, Fujian Institute of Research on the Structure of Matter, University of Chinese Academy of Sciences, Fuzhou 350100, China; ‡ School of Chemistry and Chemical Engineering, Institute of Industry & Equipment Technology, Hefei University of Technology, Hefei 230009, China; § Orthopedics Department, Guangdong Provincial Hospital of Traditional Chinese Medicine, Guangzhou 510120, China; ∥ Faculty of Pharmacy, University of Ljubljana, Aškerčeva Cesta 7, SI-1000 Ljubljana, Slovenia

## Abstract

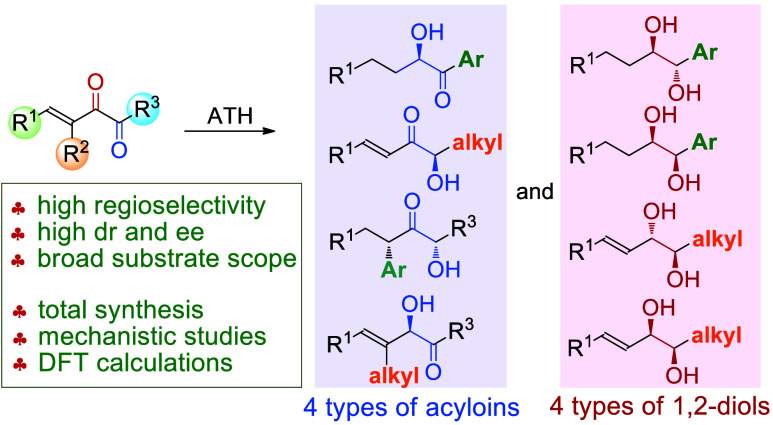

Asymmetric transfer hydrogenation (ATH) has been recognized as a highly valuable strategy that allows access to enantioenriched substances and has been widely applied in the industrial production of drug molecules. However, despite the great success in ATH of ketones, highly efficient, regio- and stereoselective ATH on enones remains underdeveloped. Moreover, optically pure acyloins and 1,2-diols are both extremely useful building blocks in organic synthesis, medicinal chemistry, and materials science, but concise asymmetric approaches allowing access to different types of acyloins and 1,2-diols have scarcely been discovered. We report in this paper the first highly efficient ATH of readily accessible β,γ-unsaturated α-diketones. The protocol affords four types of enantioenriched acyloins and four types of optically pure 1,2-diols in highly regio- and stereoselective fashion. The synthetic value of this work has been showcased by the divergent synthesis of four related natural products. Moreover, systematic mechanistic studies and density functional theory (DFT) calculations have illustrated the origin of the reactivity divergence, revealed the different roles of aromatic and aliphatic substituents in the substrates, and provided a range of unique mechanistic rationales that have not been disclosed in ATH-related studies.

## Introduction

**Scheme 1:**
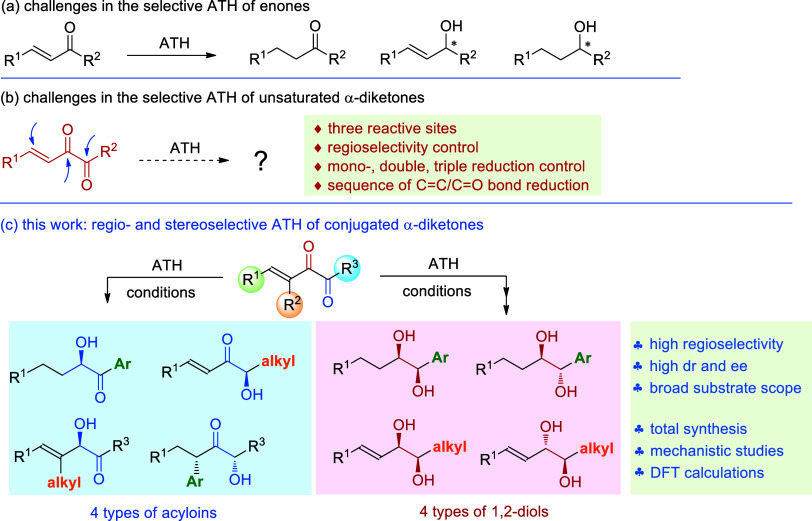
Research Background

Asymmetric transfer hydrogenation (ATH) has attracted the extensive attention of global researchers from both academia and industry,^1−12^ and has been established as a highly robust and practical method for the synthesis of enantioenriched molecules owing to its mild reaction conditions, operational simplicity, and avoidance of the use of special reactors.^13−23^ Through the use of half-sandwich bifunctional catalysts, great success has been achieved in the ATH of various prochiral ketones such as aryl/alkyl ketones, α-functionalized aryl ketones, cyclic ketones with α-substituents, and ynones.^24−47^ But in sharp contrast, the highly selective ATH of enones, especially acyclic enones, still remains underdeveloped. The main challenge lies in the regioselectivity control because the activated double bond is also amenable for the hydrogenation through a 1,4-reduction pathway; in many cases, mixtures of two or three products are obtained, and in some cases, the enantioselectivity control is also a problematic issue (Scheme 1a).^48−60^ These difficulties have been clearly demonstrated in the works from Wills,^50−53^ Deng,^54^ Morris,^55,56^ Costa,^57,58^ and others.

In this context, a more complicated situation will be confronted when the substrate for ATH is a conjugated α-diketone. Such types of compounds can be readily synthesized from commercial reagents but have not been employed as substrates for ATH. The coexistence of both the enone and diketone units undoubtedly makes the control of regioselectivity and the control of mono-, double, and triple hydrogenation in the related ATH an extremely challenging task; in addition, the sequence of C=C bond and C=O bond reduction is also an important issue to be clarified (Scheme 1b). As our continued interest both in ATH^61−66^ and α-diketone chemistry,^67−77^ we report herein the systematic study on the regio-, enantio-, and diastereoselective ATH of conjugated α-diketones (Scheme 1c). The protocol affords four types of enantioenriched acyloins and four types of stereomerically pure 1,2-diols, which are all highly valuable intermediates in organic synthesis, medicinal chemistry, and materials science,^78−83^ but known reports are scarcely able to furnish several types of optically pure acyloins and diols using the ATH protocol. The synthetic value of our method has been demonstrated in the synthesis of sordariol and the other three related natural products. The substitution patterns of the substrates are found to play a vital role in achieving diversified reactivities, and mechanistic studies and DFT calculations have also been executed to understand the unique reaction pathways that have not been revealed in previous investigations.

## Results and Discussion

At the initial stage, we tested the ATH of conjugated α-diketone **1a** using various commercially available half-sandwich-type catalysts **A**–**D**.^1,84−87^ When catalyst **A** was used in HCO_2_H/Et_3_N (1:3 v/v) at 40 °C, diols **3a** and **4a** were both obtained with low stereoselectivity (Table 1, entry 1). A similar result was observed when the ratio of HCO_2_H/Et_3_N was 1:2 (v/v) (Table 1, entry 2). When the relative ratio of HCO_2_H was increased, the reaction was retarded (Table 1, entry 3). Then, we set the ratio of HCO_2_H/Et_3_N to 1:3 (v/v) and surveyed other parameters. The reaction at rt for 19 h afforded again both **3a** and **4a** (Table 1, entry 4), but when the reaction was stopped after 1.6 h, the acyloin product **2a** (99% ee) was produced in 63% yield, together with its regioisomer **2a**′ (16% yield and 44% ee) and small amounts of **3a** and **4a** (Table 1, entry 5). It is noteworthy that the double bonds were hydrogenated en route to **2a** and **2a**′. Reducing the amount of the catalyst to 1 mol % and running the reaction for 4 h afforded **2a** in 74% yield with excellent 99% ee, together with 14% yield of **2a**′ (Table 1, entry 6). Prolonging the reaction time to 6 h led to the formation of the diol **3a** (Table 1, entry 7). We also tested the reaction using a 1:1 ratio of HCO_2_H/Et_3_N but found that the conversion was almost completely inhibited (Table 1, entry 8). Such a result is in accordance with Xiao’s study.^88^ Catalysts **B**–**D** have been surveyed, but did not show better results in terms of regio- and stereoselectivity (Table 1, entries 9–11). Our next goal was to get the diol with high regioselectivities and stereoselectivities. The reactions using 2 mol % of the catalyst **A** at 40 °C with the addition of a series of additives have been examined (Table 1, entries 12–15), and Ti(O^*i*^Pr)_4_ was found to produce a better *syn*/*anti* ratio of the diol product (Table 1, entry 15). The reactions at rt or 50 °C gave inferior outcomes (Table 1, entries 16–17), but using 1.5 mol % of the catalyst and conducting the reaction at 60 °C slightly increased the yield of **3a** (Table 1, entry 18), and by changing the base to diisopropylethylamine (DIPEA) we were able to get **3a** in 84% yield with *syn*/*anti* 84:14 and 94% ee (Table 1, entry 19). Catalysts **B**, **C**, and **D** have also been tested, but all provided **3a** with lower *syn*/*anti* selectivity (Table 1, entries 20–22).

**Table 1:**
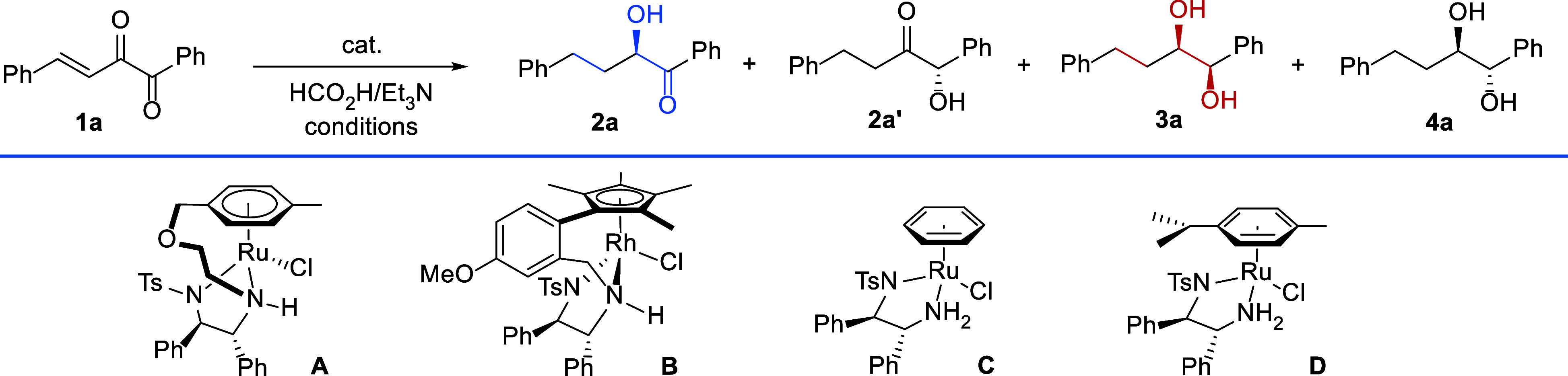
Condition Optimization for the ATH of Conjugated α-Diketones^a^

						yields (%), ee (%)			
entry	cat. (mol %)	HCO_2_H:Et_3_N (v/v, M)	temp (°C)	time (h)	additive	**2a**	**2a′**	**3a**	**4a**
1	**A** (2.0)	1:3	40	12	none	trace	trace	33, –	55, –
2	**A** (2.0)	1:2	40	12	none	trace	trace	32, –	49, –
3	**A** (2.0)	5:2	40	12	none	trace	trace	trace	trace
4	**A** (2.0)	1:3	rt	19	none	trace	trace	34, 61	58, 45
5	**A** (2.0)	1:3	rt	1.6	none	63, 99	16, 40	9, –	6, –
6	**A** (1.0)	1:3	rt	4	none	74, 99	14, 41	7, –	5, –
7	**A** (1.0)	1:3	rt	6	none	45, 99	14, 33	23, –	16, –
8	**A** (1.0)	1:1	rt	4	none	trace	12, –	trace	trace
9	**B** (1.0)	1:3	rt	0.5	none	27, 11	13, 4	34, –	9, –
10	**C** (1.0)	1:3	rt	3	none	17, 92	56, 21	21, –	trace
11	**D** (1.0)	1:3	rt	3	none	48, 97	23, 11	14, –	trace
12	**A** (2.0)	1:3	40	12	LiCl	trace	trace	33, 80	59, 29
13	**A** (2.0)	1:3	40	17	CeCl_3_	trace	trace	36, 61	58, 58
14	**A** (2.0)	1:3	40	17	TiCl_4_	trace	trace	68, 82	29, 68
15	**A** (2.0)	1:3	40	12	Ti(O^*i*^Pr)_4_	trace	trace	73, 90	25, 93
16	**A** (2.0)	1:3	rt	20	Ti(O^*i*^Pr)_4_	trace	trace	66, 90	32, 92
17	**A** (2.0)	1:3	50	12	Ti(O^*i*^Pr)_4_	trace	trace	65, 88	32, 85
18	**A** (1.5)	1:3	60	11	Ti(O^*i*^Pr)_4_	trace	trace	78, 91	20, 95
19^b^	**A** (1.5)	1:3	60	12	Ti(O^*i*^Pr)_4_	trace	trace	84, 94	14, 84
20^b^	**B** (2.0)	1:3	60	10	Ti(O^*i*^Pr)_4_	trace	trace	57, 95	33, 46
21^b^	**C** (2.0)	1:3	60	9	Ti(O^*i*^Pr)_4_	trace	trace	53, 55	44, 94
22^b^	**D** (2.0)	1:3	60	11	Ti(O^*i*^Pr)_4_	trace	trace	58, 74	37, 90

a Reaction conditions: **1a** (0.2 mmol), HCO_2_H/Et_3_N (1:3 v/v, 1 mL), additive (1 equiv), under argon atmosphere. All isolated yields were based on **1a**. The ee values were determined via HPLC analysis on a chiral stationary phase.

b DIPEA (diisopropylethylamine) was used instead of Et_3_N.

**Scheme 2:**
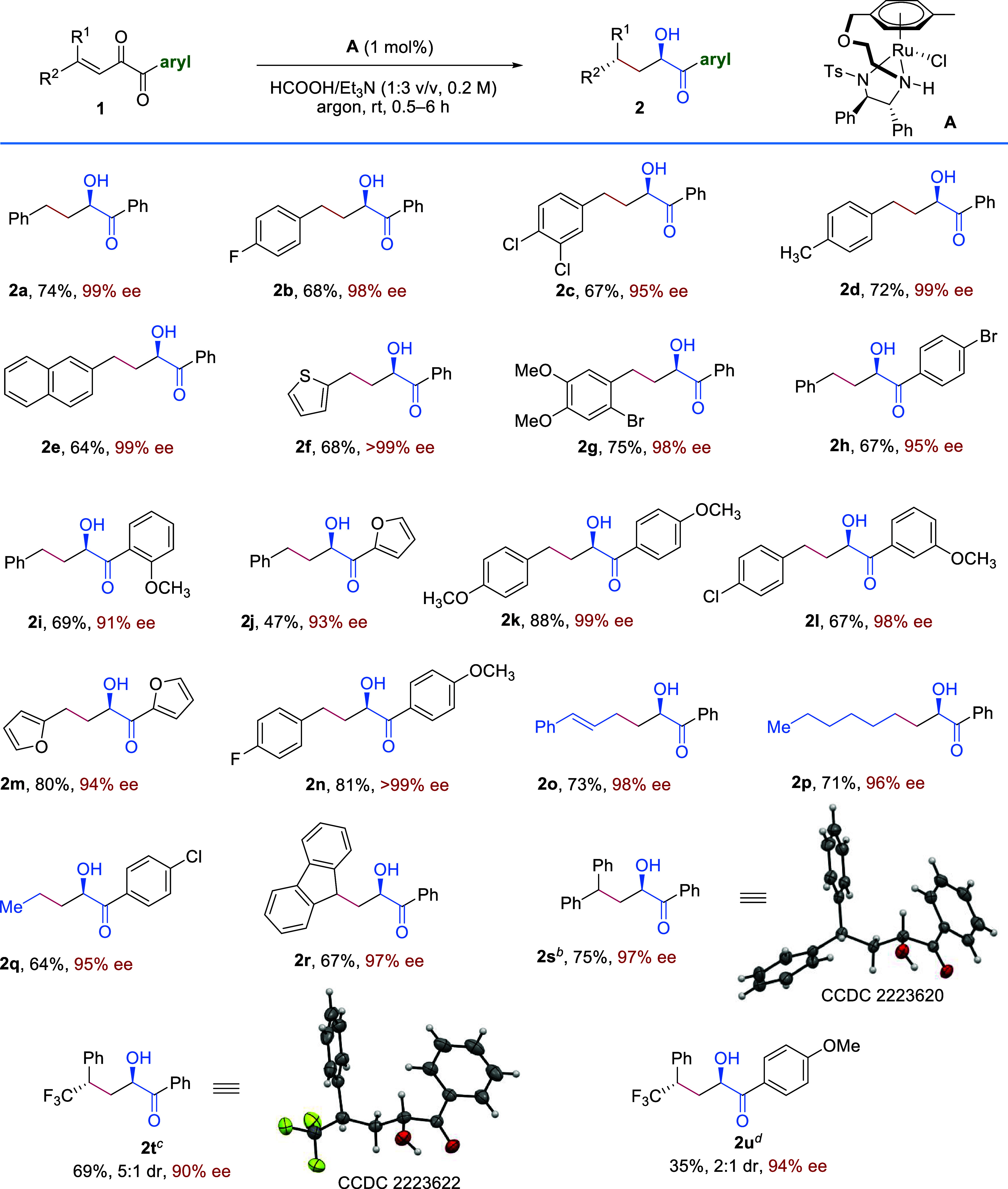
Scope of Substrates with Aryl Ketone Moiety Reaction conditions: **1** (0.2 mmol), **A** (1 mol %), HCOOH/Et_3_N (1:3 v/v, 0.2 M), rt, under argon atmosphere; all isolated yields were based on **1**; ee values were determined via HPLC analysis on a chiral stationary phase.a (0.2 mmol), A (1 mol %), HCOOH/Et_3_N (1:3 v/v, 0.2 M), rt, under argon atmosphere; all isolated yields were based on 1; ee values were determined via HPLC analysis on a chiral stationary phase. **A** (1.5 mol %) was used. HCOOH/DIPEA (1:5 v/v, 0.2 M) and Ti(O^*i*^Pr)_4_ (0.2 mmol) were used. Ti(O^*i*^Pr)_4_ (0.2 mmol) was added.

Having established the optimal conditions for the ATH of aryl vinyl α-diketones to afford both acyloin (Table 1, entry 6) and diol (Table 1, entry 18) products with high regio- and stereoselectivities, we then evaluated the generality of the protocol, with the synthesis of acyloins being checked first (Scheme 2). We were pleased to find that the introduction of both electron-withdrawing and -donating groups into the phenyl ring at the R^2^ position showed little influence on the results, releasing **2b**–**2d** in good yields with excellent ee values (Scheme 2, **2b**–**2d**). When the R^2^ group was a naphthyl, a thienyl, or a 2-Br-4,5-OMe_2_C_6_H_2_ group, the reactions still proceeded smoothly to afford the corresponding products with 98–99% ee (Scheme 2, **2e**–**2g**). Then, the aryl substituents at the ketone moiety were evaluated, and we found that 4-BrC_6_H_4_, 2-OMeC_6_H_4_, and furyl groups all tolerated well (Scheme 2, **2h**–**2j**). The simultaneous change of both the R^2^ and aryl groups at the substrates proved successful, delivering **2k**–**2n** in good to high yields with excellent ee values (Scheme 2, **2k**–**2n**). Notably, the substrates bearing a styryl group or alkyl group worked well under the reaction conditions, producing **2o**–**2q** efficiently (Scheme 2, **2o**–**2q**). Finally, we investigated γ,γ-disubstituted substrates, and the reactions yielded **2r** and **2s** with 97% ee (Scheme 2, **2r** and **2s**). When an γ-CF_3_ group exists in the substrate, the corresponding products with two stereocenters were obtained (Scheme 2, **2t** and **2u**). The absolute configurations of **2s** and **2t** were determined by single-crystal X-ray diffraction (SCXRD) analysis, and the configurations of all of the other products **2** were assigned by analogy.

**Scheme 3:**
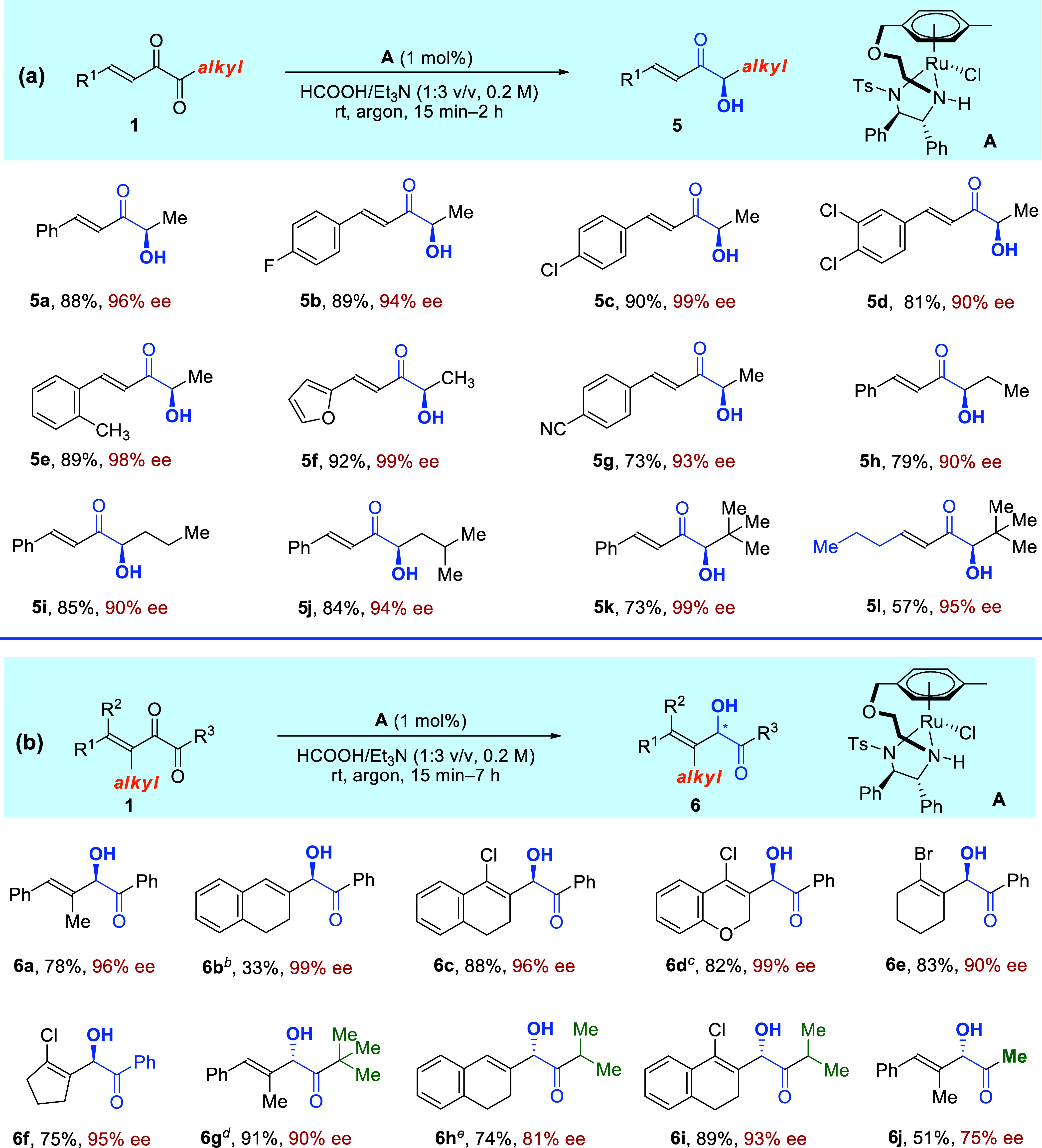
Scope of Conjugated α-Diketones with (a) Alkyl Ketone Units and (b) β-Alkyl Substituents Reaction conditions: **1** (0.2 mmol), **A** (1 mol %), HCOOH/Et_3_N (1:3 v/v, 1.0 mL), rt, under argon atmosphere; all isolated yields were based on **1**; all ee values were determined via HPLC analysis on a chiral stationary phase. **A** (3 mol %). **B** (2 mol %) was used. **C** (5 mol %) was used. **C** (5 mol %), HCOOH/Et_3_N (1:3 v/v, 2.0 mL), and Ti(O^*i*^Pr)_4_ (0.2 mmol) were used.

Having achieved the regio- and enantioselective ATH of vinyl α-diketones bearing aryl ketone moiety, we then started to examine the reactivity of alkyl ketones (Scheme 3a). Somewhat to our surprise, a set of thoroughly different types of products (i.e., α-hydroxy enones) were obtained with high regio- and enantioselectivity under the same conditions. When the alkyl group was set as Me, the R^1^ group could be changed to aryl rings with electron-withdrawing or donating groups and furyl, and in all cases, the corresponding products were generated in high yields and with 90–99% ee (Scheme 3a, **5a–5g**). A variety of other alkyl substituents such as Et, ^*n*^Pr, ^*i*^Bu, and ^*t*^Bu have also been evaluated, and no apparent influence on the outcomes was detected, and **5h**–**5k** were produced efficiently with excellent ee values (Scheme 3a, **5h–5k**). Additionally, the substrate having two aliphatic groups was also tolerable, delivering **5l** with 95% ee, albeit in a moderate yield (Scheme 3a, **5l**). The absolute configuration of product **5b** was determined to be *R-* using the Mosher ester method^89^ (see the Supporting Information for more details). Stereomerically pure α-hydroxy enones have been widely applied in natural product synthesis such as varitriol, varioxirane, microcosamine A, aplykurodinone-1, hyptenolide, malyngic acid, and fulgidic acid, but tedious steps were often employed to construct the α-hydroxy enone units from enantioenriched starting materials.^90−93^ Therefore, our method provides a new choice to make such key moieties using a catalytic method.

Subsequently, conjugated α-diketones with β-alkyl substituents were subjected to the reaction conditions, and again, a new type of products, which are α-hydroxy-β,γ-unsaturated ketones, were furnished (Scheme 3b). The reaction tolerates substrates with trisubstituted alkene units such as phenylpropenyl and benzocyclohexenyl, affording **6a** and **6b** with excellent 96% and 99% ee, respectively (Scheme 3b, **6a** and **6b**). Moreover, tetrasubstituted alkenyls such as various chloro- or bromo-substituted cyclic alkene units were proved compatible, and the reactions produced **6c**–**6f** in high yields with up to 99% ee (Scheme 3b, **6c**–**6f**). Additionally, we were pleased to find that alkyl R^3^ groups were also suitable substituents, and the corresponding products **6g**–**6j** were formed efficiently with good to excellent ee values (Scheme 3b, **6g**–**6j**). The absolute configuration of **6a** was confirmed to be *R*- through the comparison of its HPLC spectrum with that obtained using the established method, and **6g** was found to be *S*-configured using the Mosher ester method (see the Supporting Information for more details). These results indicate that although two probable ATH models are involved in Scheme 3b, the internal carbonyl groups show higher reactivity in both cases. Enantioenriched α-hydroxy allylic ketones are also key intermediates in complex natural product synthesis such as bryostatins, goniodomin A, and amphidinolides G and H, but the related synthetic routes heavily rely on multiple steps using chiral starting materials.^94−96^

**Scheme 4:**
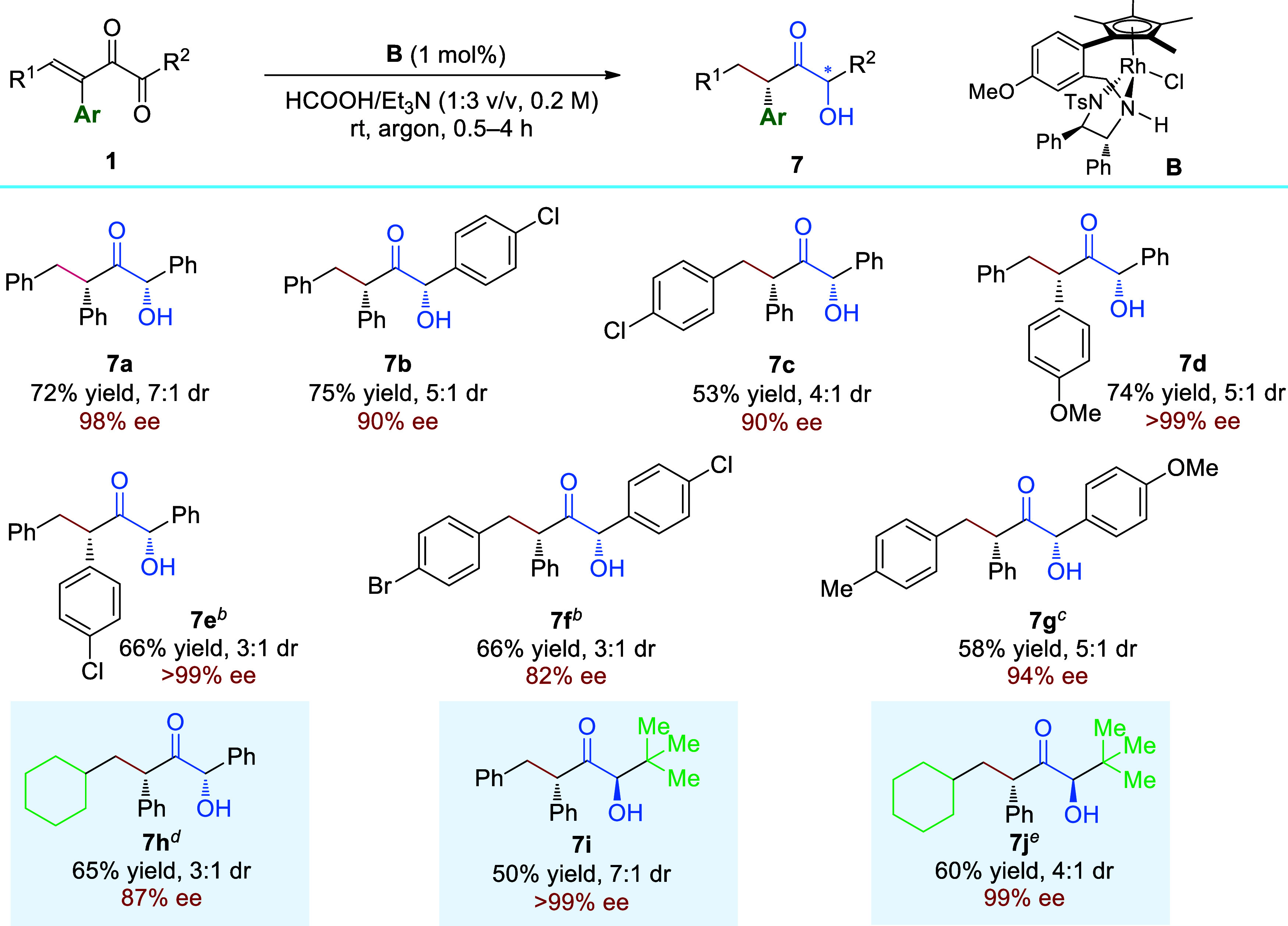
Scope of Conjugated α-Diketones with β-Aryl Substituents Reaction conditions: **1** (0.2 mmol), **B** (1 mol %), HCOOH/Et_3_N (1:3 v/v, 1.0 mL), rt, under argon atmosphere; all isolated yields were based on **1**; ee values were determined via HPLC analysis on a chiral stationary phase. HCO_2_H/DIPEA (1:3 v/v, 1.0 mL) was used. **B** (4 mol %) and HCO_2_H/DIPEA (1:3 v/v, 1.0 mL) were used. The reaction was conducted at 10–15 °C. **B** (5 mol %) was used.

After that, we further tested conjugated α-diketones bearing β-aryl substituents. This time, we were able to obtain the fourth type of products, that is, α-hydroxy-α′-aryl ketones (Scheme 4). Ru catalyst **A** and Rh catalyst **B** show comparable results in the initial screenings, and **B** is slightly better. So, we used **B** to further optimize the reaction conditions (see Supporting Information for more details). The protocol affords the corresponding disubstituted ketones with various aryl groups such as **7a**–**7g** in 53–75% yields with 82 to >99% ee values (Scheme 4, **7a**–**7g**). Moreover, substrates bearing alkyl R^1^ or R^2^ groups also worked well to provide **7h**–**7j** with up to 99% ee (Scheme 4, **7h**–**7j**). It is noteworthy that **7i** and **7j** have an *anti*-configuration, and other products have *syn*-configurations.

**Scheme 5:**
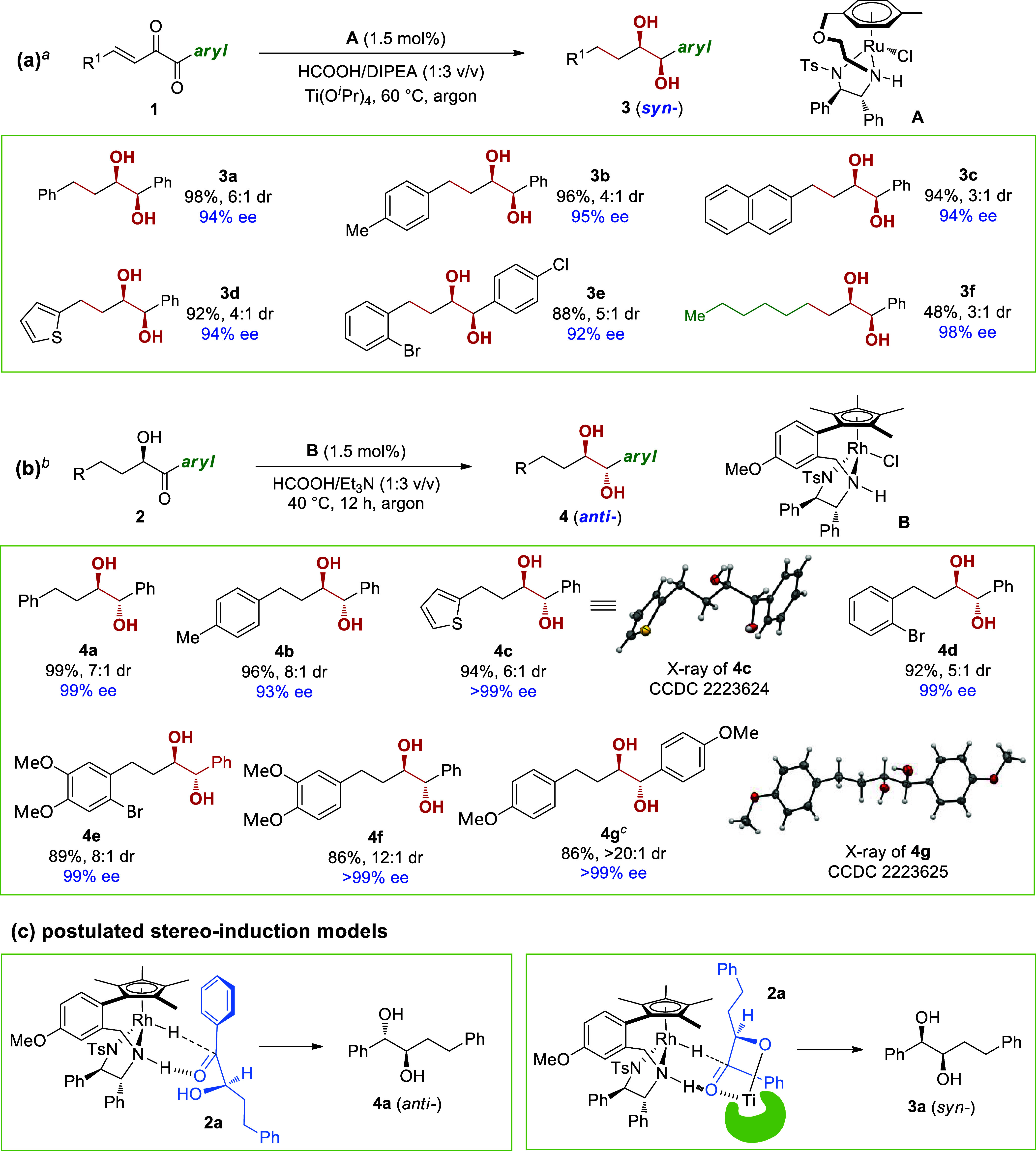
ATH Affording (a) *syn*-Diols and (b) *anti*-Diols Reaction conditions: **1** (0.2 mmol), **A** (1.5 mol %), HCOOH/DIPEA (1:3 v/v, 1.0 mL), 60 **°**C, Ti(O^*i*^Pr)_4_ (0.2 mmol), 12 h, under argon atmosphere; the isolated yields are the combined yields of both diastereoisomers based on **1**; ee values were determined via HPLC analysis on a chiral stationary phase. Reaction conditions: **2** (0.2 mmol), **B** (1.5 mol %), HCOOH/Et_3_N (1:3 v/v, 1.0 mL), 40 **°**C, 12 h, under argon atmosphere; the isolated yields are the combined yields of both diastereoisomers based on **2**; ee values were determined via HPLC analysis on a chiral stationary phase. **B** (3 mol %) was used and the reaction was run at 60 °C.

As has been mentioned in the optimization stage, *syn*-diol **3a** could be targeted with high yield with good dr and excellent ee in the presence of Ti(O^*i*^Pr)_4_ (Table 1, entry 18). The asymmetric synthesis of stereomerically enriched 1,2-diols has been an enduring topic because of their wide applications in active pharmaceutical ingredients production, asymmetric catalysis, and total syntheses of natural products.^97−102^ Considering the high importance of diols in synthetic chemistry, we continued to survey the scope of this diol synthesis method. As has been demonstrated in Scheme 5a, the protocol tolerates substrates having various aryl R^1^ groups including naphthyl and thienyl groups, affording **3a**–**3e** in excellent yields with excellent ee values (Scheme 5a, **3a**–**3e**). A further survey showed that alkyl R^1^ groups were also tolerated and could afford **3f** in moderate yield with 98% ee (Scheme 5a, **3f**). Our next goal is to obtain the corresponding *anti*-diols. Our endeavors toward direct transformation of vinyl diketones **1** to *anti*-diols with high diastereoselectivity failed, but the diastereoselective hydrogenation of enantioenriched α-hydroxy ketones **2** using catalyst **B** was found to generate *anti*-diols **4** efficiently with high dr and excellent ee values (Scheme 5b, **4a**–**4g**). The electronic properties of the phenyl rings within the substrates showed little effect on the outcomes, and the absolute configuration of **4c** and **4g** were confirmed by SCXRD analysis, and correspondingly, the relative configuration of diols **3** could be deduced through the ^1^H NMR spectra comparison. The oxidation of **3a** using activated MnO_2_ afforded (*R*)-**2a**, and therefore, the absolute configuration of **3a** can be deduced to be 1*R*,2*R*. In Scheme 5c we postulate the stereoinduction models for the formation of **4a** and **3a**. The ATH of (*R*)-**2a** using the catalyst **B** undergoes conventional model wherein CH-π attractive interaction contributes to the enantioselective hydrogen transfer, therefore *anti*-**4a** is formed. In contrast, in the presence of Ti(O^*i*^Pr)_4_, monohydrogenated **2a** is generated first, which coordinates with Ti(O^*i*^Pr)_4_ and is attacked by ruthenium hydride via the chelated Felkin-Ahn model to generate *syn*-**3a** as the major one.^103,104^

**Scheme 6:**
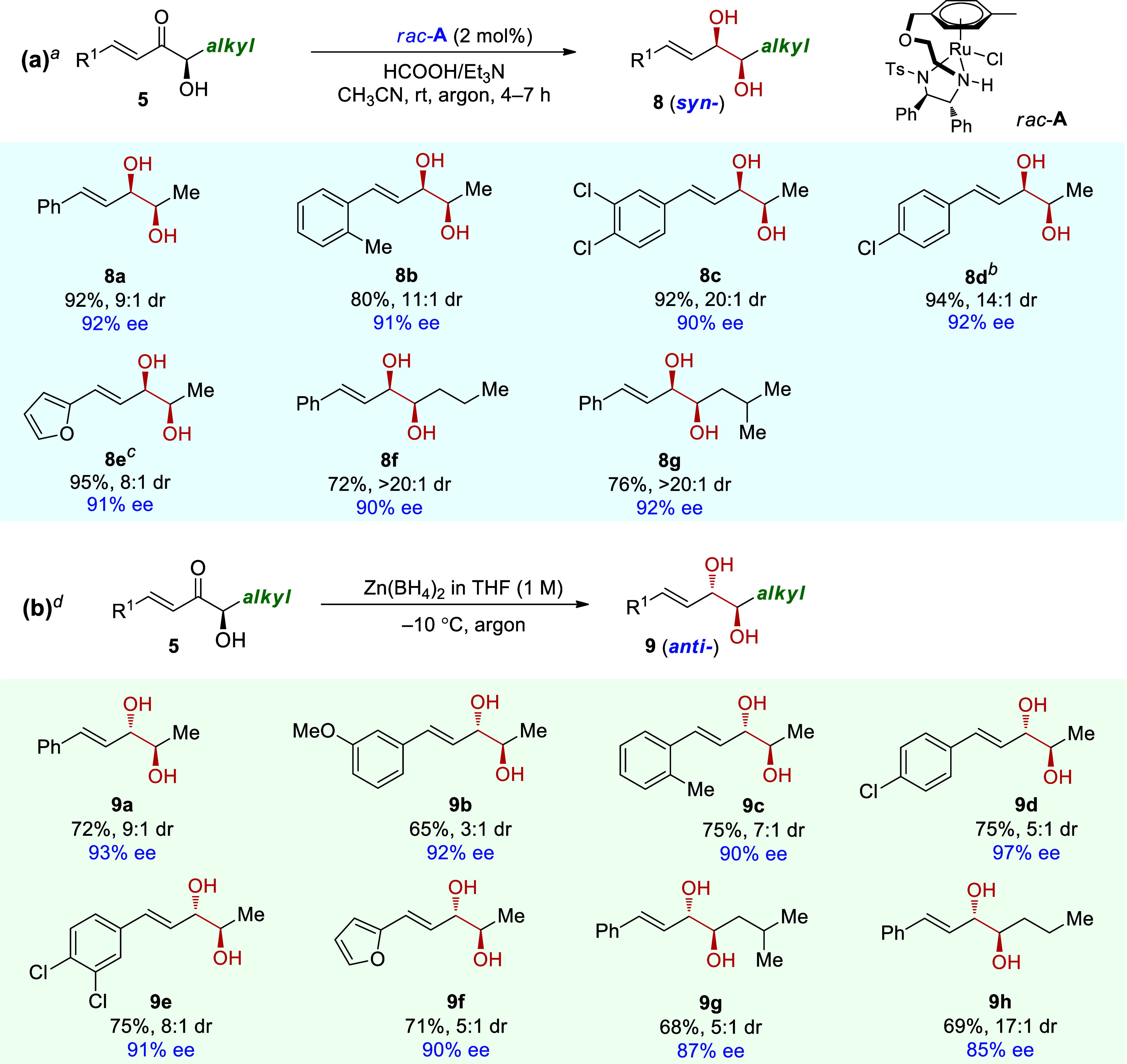
Reduction of α-Hydroxy Enones Affording (a) Vinyl *syn*-Diols and (b) Vinyl *anti*-Diols Reaction conditions: **5** (0.2 mmol), *rac*-**A** (2 mol %), HCOOH/Et_3_N (pH 6–7, 250 μL), CH_3_CN (0.15 M), rt, under argon atmosphere; all isolated yields were based on **5**; ee values were determined via HPLC analysis on a chiral stationary phase. HCOOH/Et_3_N (pH 6–7, 109 μL) was used. *rac*-**A** (1.5 mol %) and HCOOH/Et_3_N (pH 6–7, 2.0 mL) were used. Reaction conditions: **5** (0.2 mmol), Zn(BH_4_)_2_ (1 mmol), THF (1.0 mL), −10 °C, overnight, under argon atmosphere; all isolated yields were based on **5**; ee values were determined via HPLC analysis on a chiral stationary phase.

Inspired by the above results on the synthesis of saturated diols **3** and **4**, we then envisioned to use α-hydroxy enones **5** to produce both *syn*- and *anti*-diols having vinyl units. To our pleasure, we found that racemic catalyst **A** could facilitate the *syn*-hydrogenation of enantioenriched **5** to yield *syn*-**8a**–**8g** with a high dr and excellent ee values (Scheme 6a). In contrast, Zn(BH_4_)_2_ reduction of **5** afforded *anti*-**9a**–**9h** in good yields with a high level of diastereo- and enantioselectivities (Scheme 6b).

It is worthwhile to mention that optically enriched vinyl 1,2-diols are also key synthons in a range of bioactive compound synthesis or act as the key substructures in many natural products such as resolvin E3, decarestrictine D, agropyrenol, sordarial, and muricatacin,^104−111^ but the known methods either use tedious steps to modify molecules from chiral resources^105,110,112−114^ or employ the sequence of asymmetric synthesis of allylic alcohols-asymmetric epoxidation-selective ring opening^104,106,107,110,115,116^ to get the desired vinyl diols. Ideally, the direct Sharpless asymmetric dihydroxylation of 1,3-dienes could also result in the formation of diols **8** or **9**, but the regioselectivity control of such reactions is very challenging and the mixtures of regioisomers are usually observed in the known reports.^117^ Therefore, our method provides a convenient approach to solving the above problem.

**Scheme 7:**
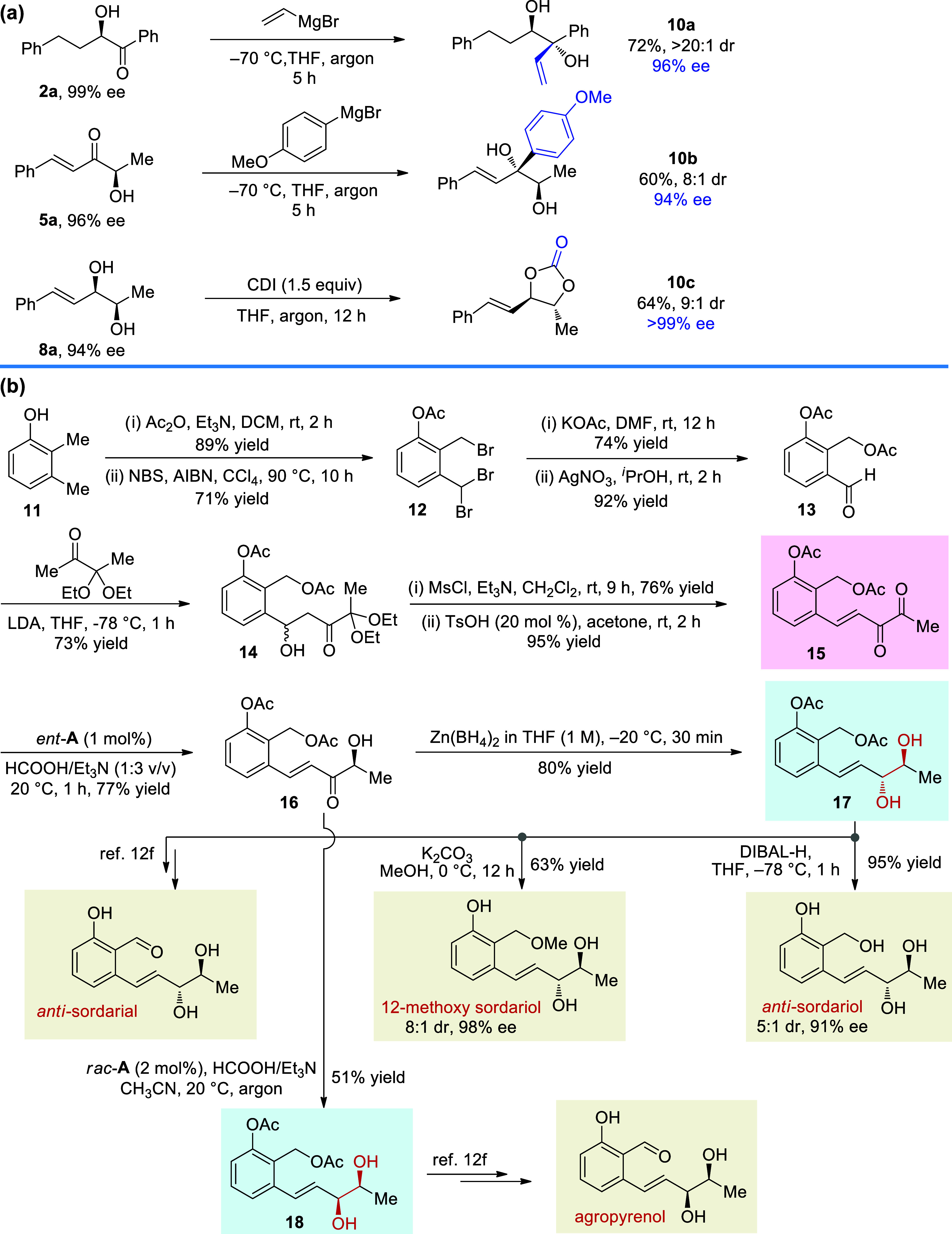
Synthetic Applications

Selected synthetic applications of the reaction are listed in Scheme 7. The addition of vinyl or aryl Grignard reagents to enantioenriched **2a** and **5a** furnished diols **10a** and **10b**, respectively, without erosion of the enantiopurity; moreover, the reaction of diol **8a** with *N*,*N*′-carbonyldiimidazole (CDI) could generate cyclic carbonate **10c** (Scheme 7a). Additionally, bioactive fungal metabolites have been recognized by their potential direct use as agrochemicals or as a lead for natural pesticides.^118,119^ For instance, sordarial, 12-methoxy sordariol, sordariol, and agropyrenol were isolated from different fungi such as *Sordaria macrospora*, *Gelasinospora heterospora*, and *Ascochyta agropyrina var. Nana*.^120,121^ Sordariol is not phytotoxic toward rice roots, but it induces browning on the rice leaves and shows immunosuppressive activity. Agropyrenol is found to exhibit cytotoxicities against certain human cancer cell lines and has antibacterial activity against *Staphylococcus aureus* and *Bacillus subtilis*.^122^ The limited accessibility of these compounds from natural sources makes their corresponding chemical synthesis very desirable. However, to date, only two successful reports of asymmetric total synthesis of these bioactive molecules have been released. Sudhakar and co-workers used chiral starting material to construct the key vinyl diol unit,^109^ and Koóš and Markovič achieved the catalytic asymmetric synthesis of vinyl diols using a multistep protocol from the desymmetric Sharpless asymmetric epoxidation of penta-1,4-dien-3-ol.^107^ In this context, we planned to use our method to address the issue. As shown in Scheme 7b, aldehyde **13** could be obtained starting from 2,3-dimethylphenol **11** in four steps. Then, **13** reacted with the in situ formed enolate to produce alcohol **14**, which allowed access to the desired unsaturated 1,2-diketone **15** after formal dehydration. Now, using the ATH method developed in this work, **15** could be selectively hydrogenated to α-hydroxyketone **16**, and further reduction of **16** afforded the key *anti-*diol **17** and *syn*-diol **18**, respectively. Then, under DIBAL-H reduction or K_2_CO_3_/MeOH conditions, *anti-*diol **17** was able to provide the desired natural products *anti*-sordariol and 12-methoxy sordariol, respectively. Additionally, using known literature methods, **17** and **18** could furnish *anti*-sordarial and agropyrenol, respectively.

## Mechanistic Studies

**Scheme 8:**
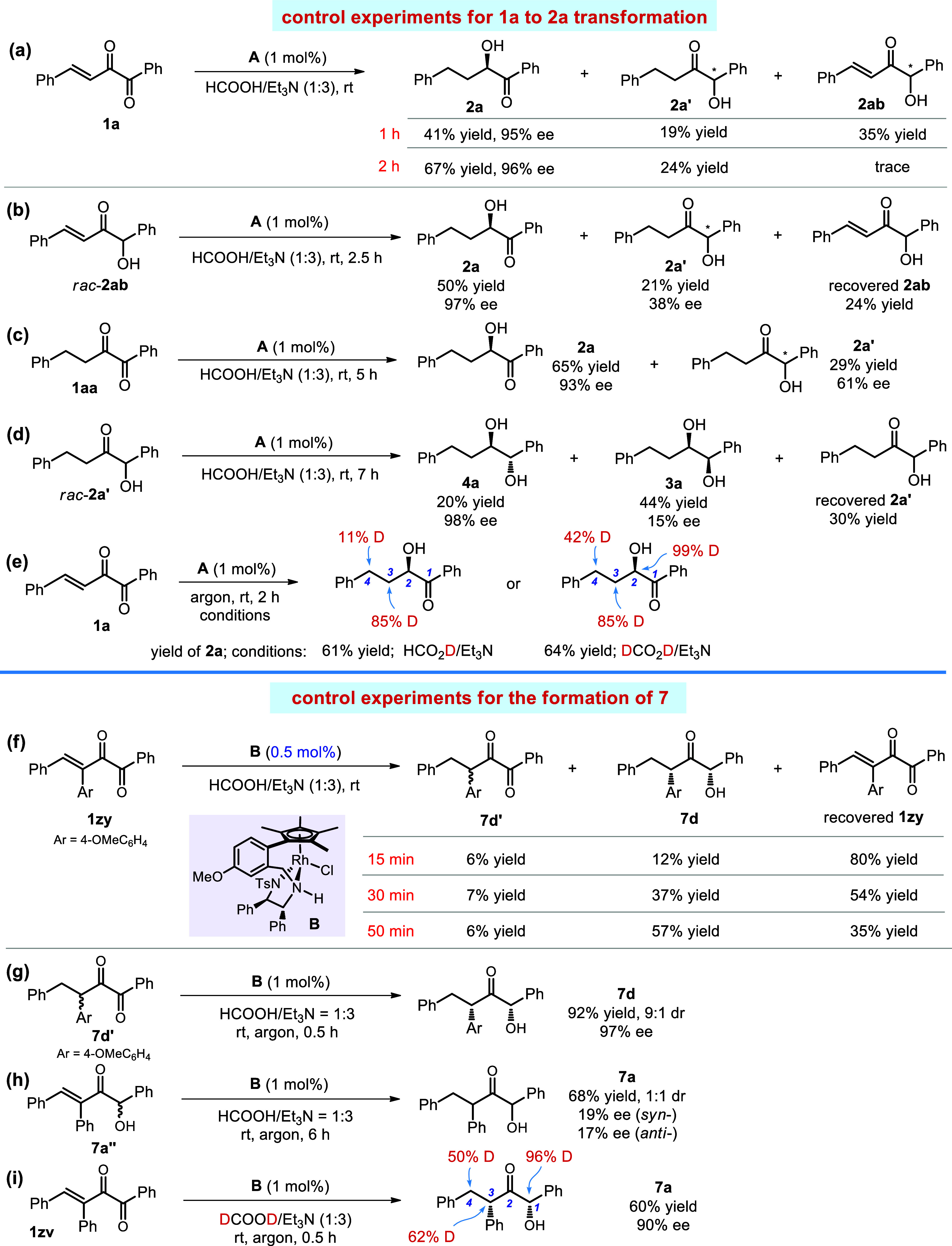
Control Experiments to Probe C=C and C=O Hydrogenation Sequence

Having achieved the divergent asymmetric synthesis of four types of acyloins and four types of 1,2-diols and demonstrated their synthetic applications, we then started to explore the mechanistic insights into the reactions, especially the origin of the diversified reactivities. At the first stage, the hydrogenation sequence of the C=C and C=O bonds for the reactions listed in Schemes 2 and 4 needs to be clarified. For this purpose, we conducted a series of control experiments (Scheme 8). When the ATH of **1a** was stopped at 1 h, we found that **2a** was formed in 41% yield with 95% ee, together with the generation of **2a**′ and **2ab**; then, after 1 more hour, **2ab** was mostly consumed, the yield of **2a** was increased to 67%, and the yield of **2a′** slightly increased (Scheme 8a). The results indicate that **2ab** might be a reaction intermediate toward the formation of **2a**. To prove this hypothesis, we exposed **2ab** to the reaction conditions and found that it delivered both **2a** and **2a**′ with the former as the major product (Scheme 8b). The reduction of diketone **1aa** delivered a similar level of stereo- and regioselectivity as the reduction of **2ab** (Scheme 8c). Additionally, the ATH of racemic **2a**′ was evaluated, and we found that it cannot be converted to **2a**; only the diols **3a** and **4a** were formed (Scheme 8d). Furthermore, the reaction using HCO_2_D afforded **2a** with 11 and 85% D introduced into the 4- and 3-position, and when DCO_2_D was employed, 42, 85, and 99% D were observed at the 4-, 3-, and 2-position of **2a** (Scheme 8e). The results suggest that the 1,4-reduction of **1a** is the major pathway, although other possible processes still exist.

The next goal is to investigate the ATH of β-aryl-substituted unsaturated diketones such as **1zy** (Scheme 8f–i). The reaction-monitoring experiment (Scheme 8f) showed that the C=C bond hydrogenation product **7d**′ is formed in the reaction and **7d**′ is reduced preferentially in the presence of **1zy**. This points to an energetically favorable interaction between catalyst **B** and **7d**′, which is reproduced by DFT calculation (see Figure 4). Subjecting racemic **7d**′ to the standard reaction conditions yielded **7d** in excellent yield with high dr and ee (Scheme 8g), which confirmed the concomitant dynamic kinetic resolution of **7d**′, but **7a″** could not provide the same result because **7a″** led to **7a** with 1:1 dr and low ee values (Scheme 8h). Finally, we conducted the ATH of **1zv** using DCO_2_D and observed 50, 62, and 96% D incorporating into the 4-, 3-, and 1-position of **7a** (Scheme 8i). These experiments clearly show that the C=C bond hydrogenation product is a reasonable intermediate for the formation of the doubly hydrogenated products **7**, and the introduction of a β-aryl substituent significantly enhances the regioselectivity of the corresponding ATH process because C=C bond reduction (1,4-reduction) is more favored.

Density functional theory (DFT) calculations have been widely used to get insightful understanding of the mechanism of ATH.^123−131^ In this regard, we selected four model reactions and conducted systematic DFT calculations to gain more mechanistic insight into the origin of the diversified reactivities.

**Figure 1:**
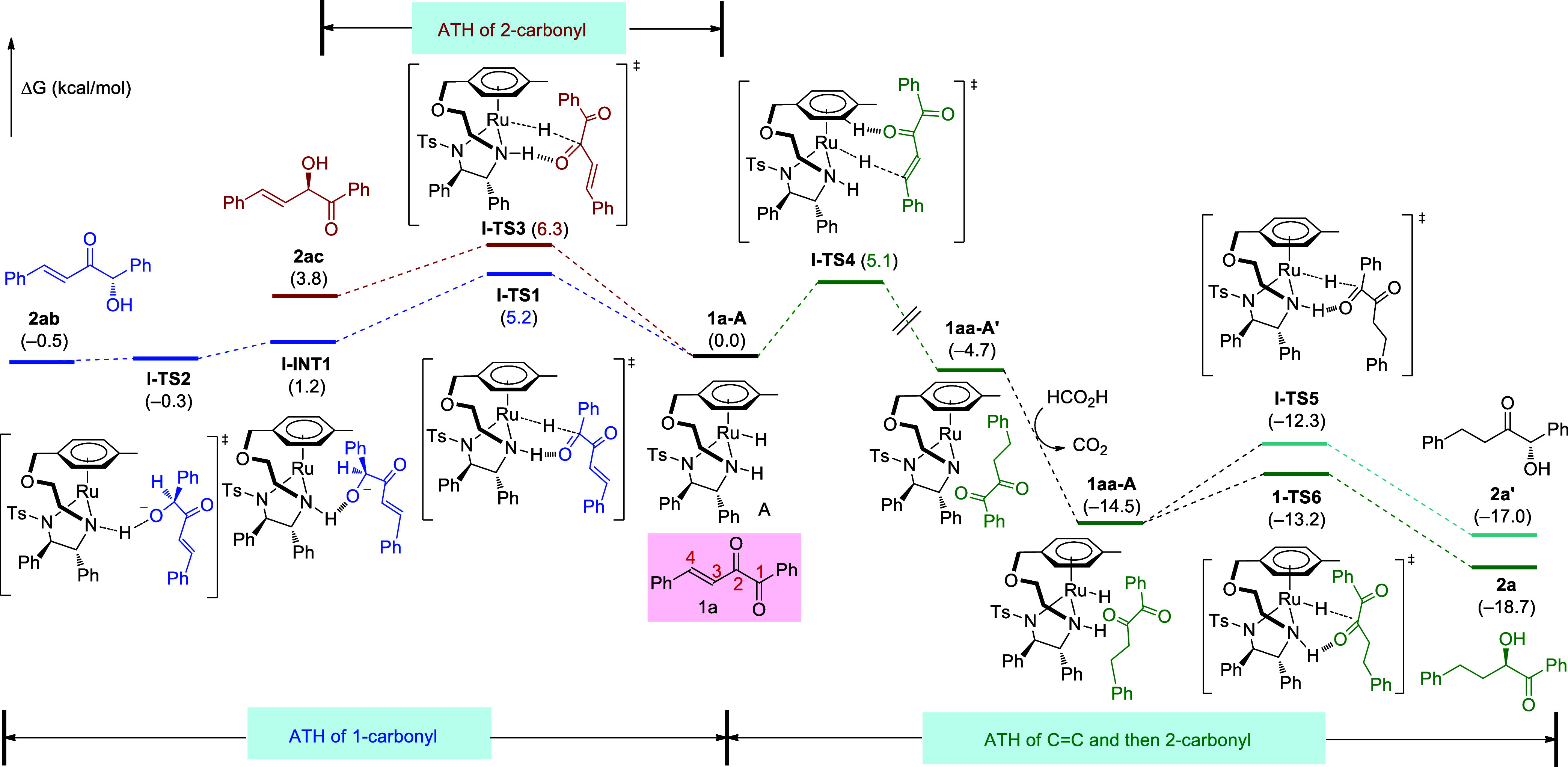
Gibbs free energy profiles of ATH of vinyl α-diketone **1a**.

First, substrate **1a** with three reactive sites (1-carbonyl, 2-carbonyl, and 3,4-C=C) was studied (Figure 1). The optimized transition state for the ATH of 1-carbonyl is **I-TS1** with a free energy of 5.2 kcal/mol. In **I-TS1**, a hydrogen bond interaction exists between the carbonyl oxygen atom and N–H of the catalyst, with an O···H distance of 1.82 Å. The obtained intermediate **I-INT1** then undergoes rapid proton transfer through transition state **I-TS2** with the free energy of −0.3 kcal/mol. The product **2ab** is generated with a free energy of −0.5 kcal/mol. Overall, the total energy barrier of 1-carbonyl ATH is 5.2 kcal/mol, and the energy change is −0.5 kcal/mol. For the ATH of 2-carbonyl, the hydride transfer transition state is **I-TS3**, with the free energy of 6.3 kcal/mol, and the free energy of the corresponding product **2ac** is 3.8 kcal/mol, indicating the process is kinetically and thermodynamically unfavorable than 1-carbonyl ATH. For ATH of 3,4-C=C bond, the hydride transfer transition state is **I-TS4**, with a free energy of 5.1 kcal/mol. The product of double-bond reduction, **1aa-A′**, is then obtained with a free energy of −4.7 kcal/mol via rapid proton transfer and enol-ketone conversion. These two steps are not decisive for the regioselectivity control of the whole reaction, and the calculation results (from **I-TS4** to **1aa-A′**) are displayed in the Supporting Information.

The results indicate that both 1-carbonyl and 3,4-C=C bond reduction have similar energy barriers (5.2 vs 5.1 kcal/mol), but the product of the C=C reduction is thermodynamically more stable. This allows both groups to be reduced at the beginning, but **2ab** undergoes the reverse process to produce the reactant of **1a** again, and as the reaction proceeds, the more stable product **1aa** gradually becomes the main product. This explains the control experiment in which 1-carbonyl reduction product **2ab** first forms and then disappears (Scheme 8a). For the following ATH occurring on **1aa**, the rate-determining hydride transfer transition states of 1-carbonyl and 2-carbonyl are **I-TS5** and **I-TS6**, with free energies of −12.3 and −13.2 kcal/mol, respectively. The related products are **2a**′ and **2a**, with free energies of −17.0 and −18.7 kcal/mol, respectively. Here, ATH of 2-carbonyl becomes more favorable, since 3,4-C=C has been reduced and thus cannot inhibit the reactivity of 2-carbonyl by the conjugation effect. Additionally, the final product of **2a** exhibits a lower energy of 4.2 kcal/mol than **1aa-A**, thus favoring an equilibrium toward **2a**. Overall, substrate **1a** first undergoes simultaneous ATHs of 1-carbonyl and C=C bond, but the inverse process of 1-carbonyl ATH occurs driven by the formation of more stable C=C reduction product. In the subsequent ATH, the 2-carbonyl reduction is kinetically more favorable, finally releasing **2a** as the major product.

**Figure 2:**
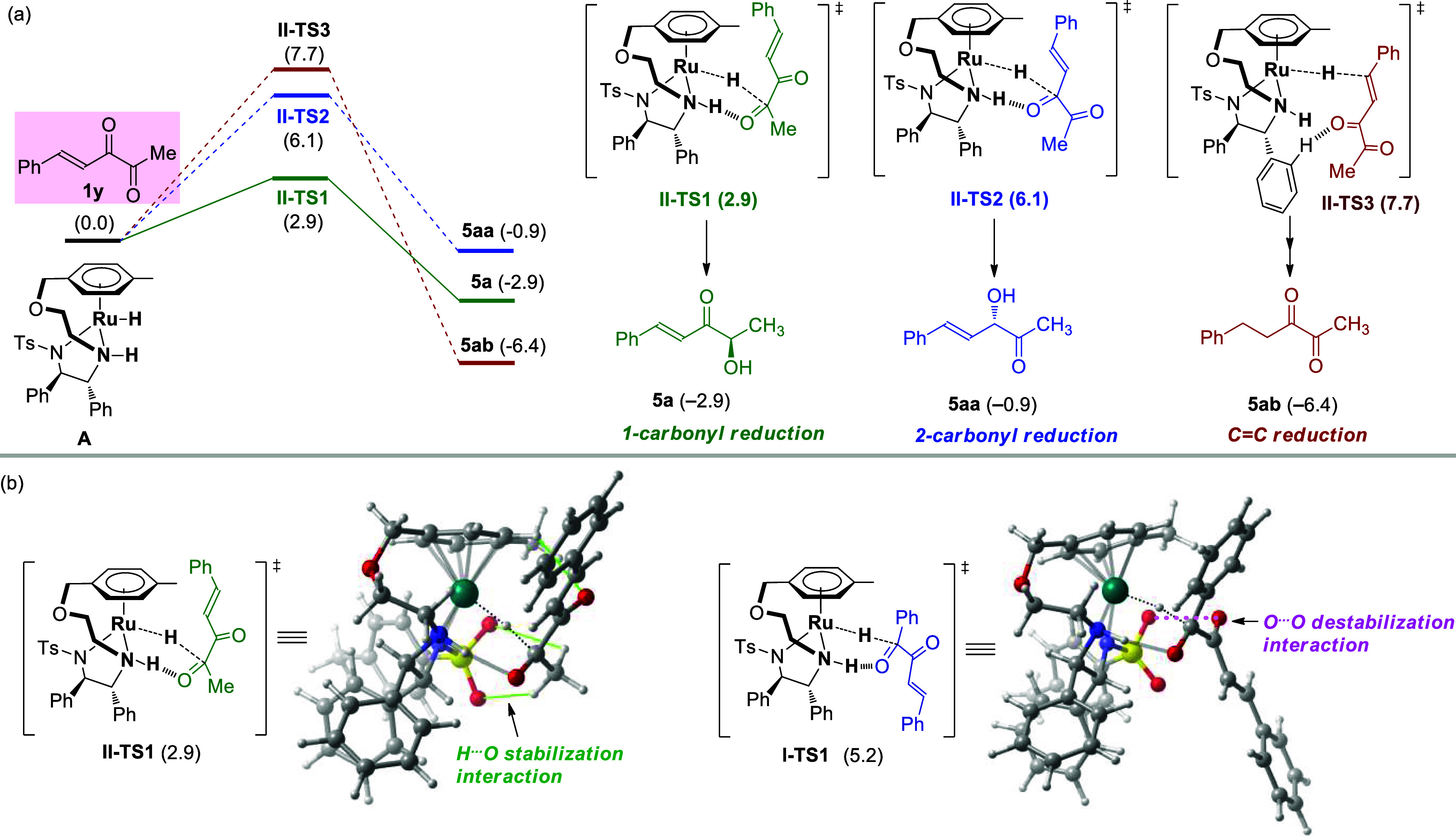
(a) Gibbs free energy profiles of ATH of α-diketones **1y** with the aliphatic ketone unit. (b) Structure and energy comparison of **II-TS1** and **I-TS1**.

Following that, the α-diketone substrate **1y** with a methyl substituent was investigated to corroborate the origin of the regioselectivity observed for the reduction of alkyl ketones (Figure 2). The hydride transfer transition states of the 1-carbonyl, 2-carbonyl, and 3,4-C=C bond are **II-TS1**, **II-TS2**, and **II-TS3**, with free energies of 2.9, 6.1, and 7.7 kcal/mol, respectively. The free energies of the corresponding products are −2.9, −0.9, and −6.4 kcal/mol, respectively. Compared with that in the ATH of **1a** (5.2, 6.3, and 5.1 kcal/mol), methyl substitution significantly enhances the reactivity of the 1-carbonyl group of **1y**. As shown in Figure 2b, in **II-TS1**, the phenyl group of the substrate faces upward, and the methyl group faces downward. Additionally, the H···O stabilizing interaction forms between the α-H of the alkyl ketone and the O atoms of the Ts group (green line). Similar interactions in transition-state geometries have been observed in related studies.^43,44^ In contrast, as to **I-TS1** of substrate **1a** with phenyl ketone units, H···O stabilizing interaction disappears, and the O···O destabilizing interaction between the 2-carbonyl and the Ts group exists (pink line), which makes the ATH of 1-carbonyl of **1a** to be with higher energy barrier than that of **1y**.

**Figure 3:**
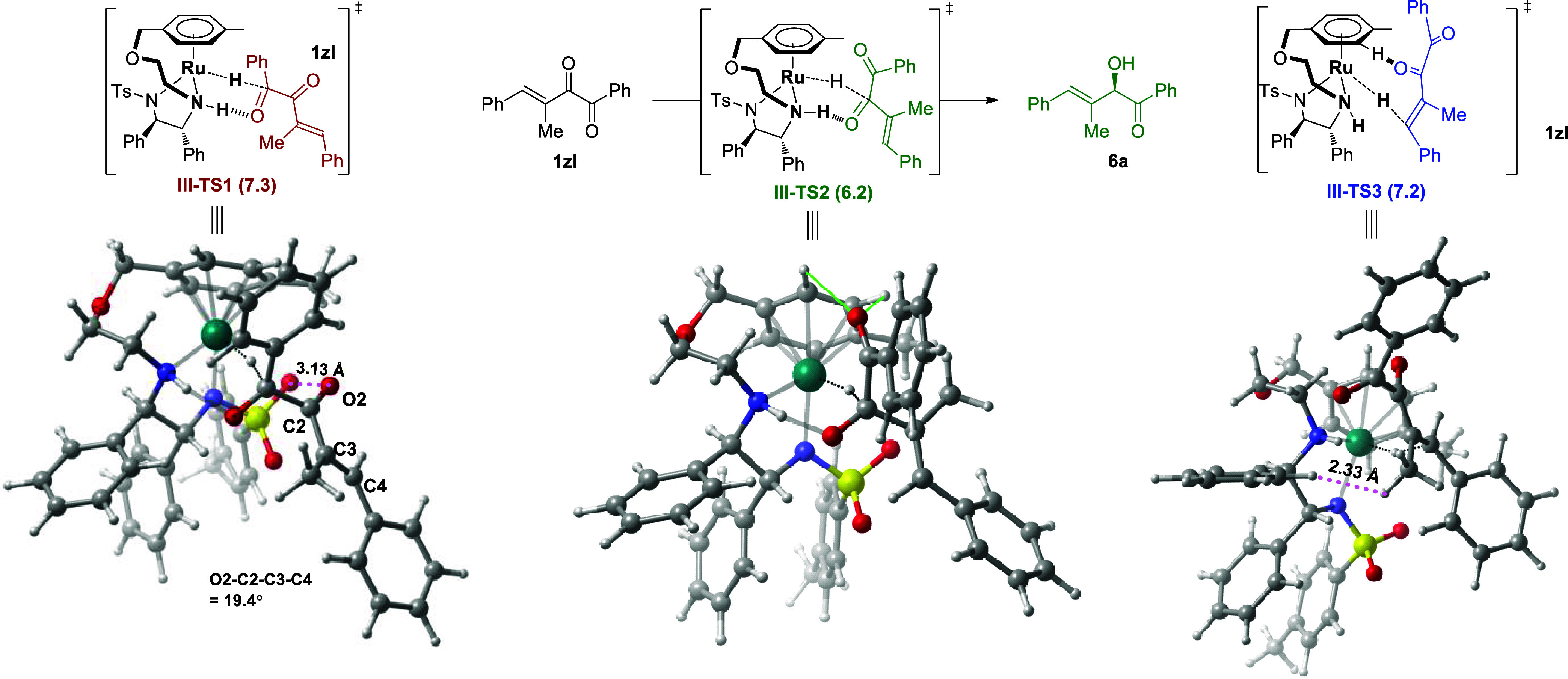
Gibbs free energies and structures of hydride transfer transition states for substrate **1zl**.

The investigation of substrate **1zl** with β-alkyl substituent reveals that the structure distortion and steric hindrance control the regioselectivity. The transition states of hydride transfer to 1-carbonyl, 2-carbonyl, and 3,4-alkenyl of **1zl** are **III-TS1**, **III-TS2**, and **III-TS3**, with free energies of 7.3, 6.2, and 7.2 kcal/mol, respectively (Figure 3). Compared with that of **1a** (5.2, 6.3, and 5.1 kcal/mol, Figure 1), the methyl substitution on the C=C double bond has no obvious effect on the 2-carbonyl group, but impedes the ATH of 1-carbonyl and the C=C bond. For the ATH of 1-carbonyl, the presence of methyl group weakens the conjugation in transition state **III-TS1**, distorting the O2–C2–C3–C4 dihedral angle to 19.4° (vs 2.3° in **I-TS1**), which in turn shortens the O–O distance to 3.13 Å (vs 3.24 Å in **I-TS1**). For ATH of the C=C bond, obvious steric hindrance appears between the methyl group and the benzene ring of the catalyst in **III-TS3**, with an H–H distance of 2.33 Å. Thus, the β-alkyl substitution causes conjugation structure distortion and steric hindrance in the reduction of the 1-carbonyl and C=C bond, which are not present for 2-carbonyl, making the ATH of the 2-carbonyl the most favorable.

**Figure 4:**
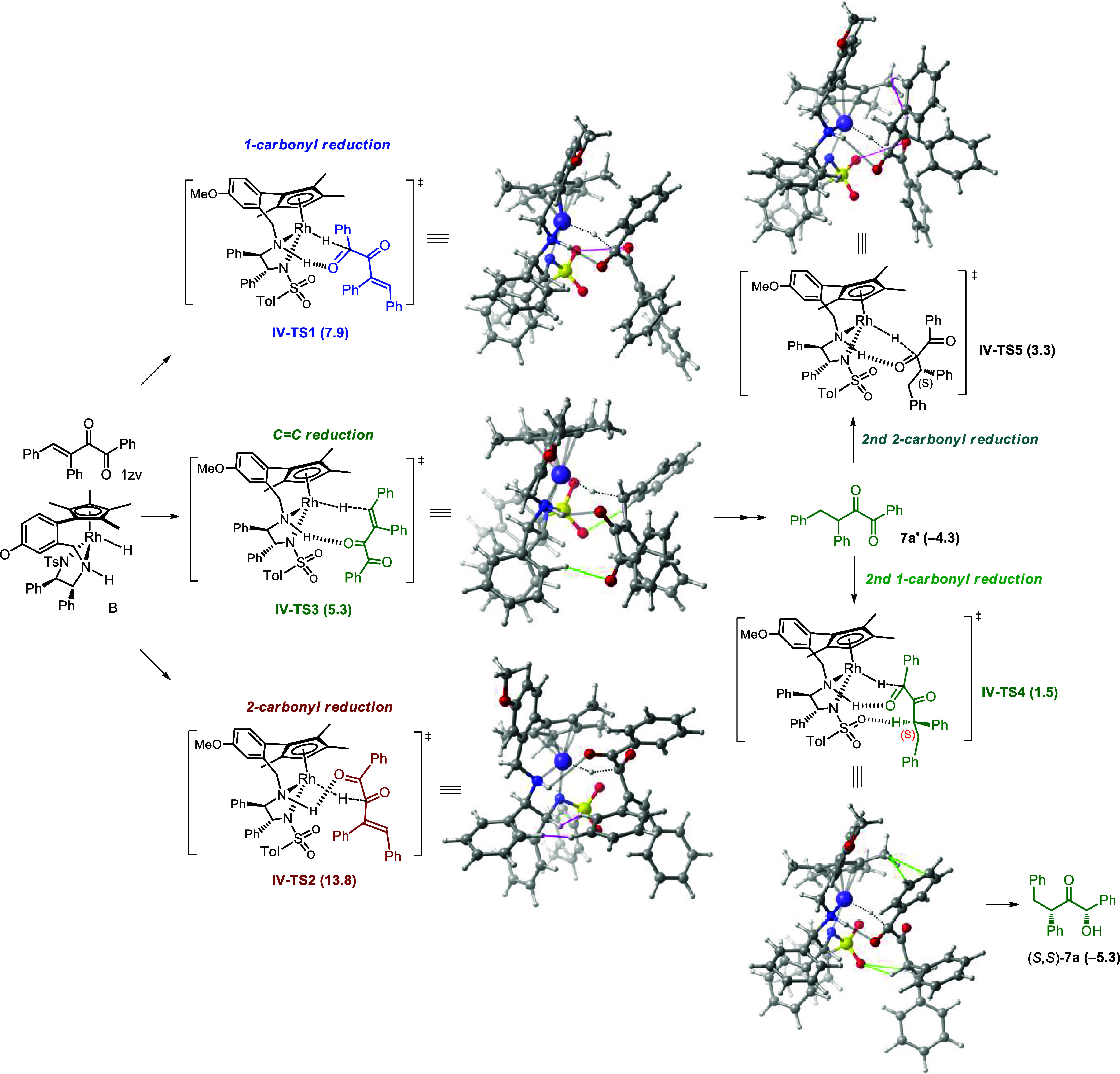
Gibbs free energy profiles of ATH of α-diketones **1zv** with a β-aryl substituent.

In the ATH of substrate **1zv** with a β-phenyl substituent, catalyst **B** was used (Figure 4). The free energies of the hydride transfer transition states of 1-carbonyl, 2-carbonyl, and 3,4-C=C bond are 7.9, 13.8, and 5.3 kcal/mol, respectively. Compared with **1c** with a β-methyl substituent (7.3, 6.2, and 7.2 kcal/mol, Figure 3), the methyl to phenyl substituent change has no discernible effect on 1-carbonyl reduction but reverses the relative kinetics of the 2-carbonyl and C=C double-bond hydrogenation. Comparing the related transition states of **III-TS2** and **IV-TS2**, it can be seen that the huge steric hindrance of the Cp* ring in **IV-TS2** makes the original upward-facing β-substituent (in **III-TS2**) downward facing. This causes steric hindrance between the β-phenyl substituent and the catalyst benzene ring (pink line), rendering the 2- carbonyl reduction unfavorable. In contrast, in **IV-TS3**, the extrusion of the Cp* ring keeps the β-phenyl substituent away, while the O–H stabilization interaction occurs between the 2-carbonyl and the catalyst (green line), thus making the ATH of 3,4-C=C the most favorable. Then, intermediate **7a**′ is generated with a free energy of −4.3 kcal/mol. The subsequent hydride transfers of 1-carbonyl and 2-carbonyl in (*S*)-**7a**′ occur via **IV-TS4** and **IV-TS5**, with free energies of 1.5 and 3.3 kcal/mol. The Cp* ring also plays the decisive role. In **IV-TS4**, the CH-π stabilizing interaction exists between the Cp* and the benzene ring in the substrate, as well as hydrogen bonding between the SO_2_ unit and the acidic CH next to the 2-carbonyl, but in **IV-TS5** it is replaced by H···H destabilization between Cp* and the substrate. Therefore, (*S*,*S*)-**7a** is obtained as the major product. The hydrogenation of (*R*)-**7a**′ is not favorable according to the absolute configuration of the product; therefore, no more calculation was conducted.

Overall, for the ATH of aryl-substituted α-diketone **1a**, 1-carbonyl and 3,4-C=C bond reduction show competitive kinetics but favorable thermodynamics of the latter. Thus, the reverse process of 1-carbonyl ATH occurs to obtain a 3,4-C=C bond reduction product. Therefore, the regioselectivity of 1-carbonyl and 3,4-C=C bond reduction of **1a** is controlled by thermodynamic factors. In contrast, changing the aryl ketone to alkyl ketone in **1y** enhances the 1-carbonyl ATH because the H···O stabilizing interaction between the alkyl and Ts group shows up. Adding β-alkyl substitution to **1a** causes conjugation structure distortion and steric hindrance in the reduction of the 1-carbonyl and C=C bond in **1zl**, thus impeding both the ATH processes. Therefore, 2-carbonyl ATH becomes the most favorable. Changing the β-alkyl to aryl substituent significantly enhances the C=C double-bond reduction of **1zv** because the huge Cp* ring keeps the β-phenyl substituent away and O–H stabilization interaction between 2-carbonyl and the catalyst emerges.

## Conclusions

In summary, the asymmetric transfer hydrogenation of β,γ-unsaturated α-diketones has been systematically studied by using both experimental and DFT methods for the first time. Four types of acyloins and four types of 1,2-diols were obtained with a high level of enantiopurities. The synthetic applications of this protocol have been illustrated in the total synthesis of related natural products. Both the terminal carbonyl substituents and the β-substituents are found to play crucial roles in regioselectivity control. The control experiments and DFT calculations were conducted to reveal mechanistic insights into the reactivity divergence of the reactions in a quantitative fashion. The work provides constructive information on the various selectivity controls in the ATH of substrates bearing multiple reactive sites and will inspire more studies.

## Supporting Information Available

Experimental procedures, materials and methods, characterization data, NMR spectra, HPLC spectra, single-crystal data, and computational details (PDF)
