# Supplementary material for: Catalytic Asymmetric Transfer Hydrogenation of β,γ-Unsaturated α-Diketones
Source: J Am Chem Soc. 2024 Nov 27;146(49):33543–60. doi: 10.1021/jacs.4c11070 (PMC11694242; doi:10.1021/jacs.4c11070)
Supplement: Supplementary file 1 — ja4c11070_si_001.pdf [file ja4c11070_si_001.pdf]

Supporting Information

Catalytic Asymmetric Transfer Hydrogenation of β,γ-Unsaturated α-Diketones

Zhifei Zhao,[1,†] Wennan Dong,[1,†] Jinggong Liu,[3] Shuang Yang,[1] Andrej Emanuel Cotman,[4] Qi Zhang,*[2] Xinqiang Fang*[1]

[1]State Key Laboratory of Structural Chemistry, Center for Excellence in Molecular Synthesis, Fujian Institute of Research on the Structure of Matter, University of Chinese Academy of Sciences, Fuzhou 350100, China.

[2]School of Chemistry and Chemical Engineering, Institute of Industry & Equipment Technology, Hefei University of Technology, Hefei 230009, China.

[3]Orthopedics Department, Guangdong Provincial Hospital of Traditional Chinese Medicine, Guangzhou 510120, China.

[4]Faculty of Pharmacy, University of Ljubljana, Aškerčeva cesta 7, SI-1000 Ljubljana, Slovenia.

†These authors contributed equally to this work.

*Email: xqfang@fjirsm.ac.cn; zhangq@hfut.edu.cn

Table of Contents

**I**	General information	S1
**II**	X-ray crystallographic analysis	S2
**III**	General procedures for the preparation of substrates	S8
**IV**	Conditions optimization of the transfer hydrogenation of **1zv**	S16
**V**	Typical procedure for the transfer hydrogenation	S17
**VI**	Procedures for the derivatizations of products	S20
**VII**	Determination of the absolute configurations	S30
**VIII**	Characterizations of new compounds	S35
**IX**	Reference	S88
**X**	^{1}H NMR and ^{13}C NMR spectra of new compounds	S90
**XI**	HPLC spectra for ee determination	S220
**XII**	Computational methods	S308

I. General information

Commercially available materials purchased from Meryer, Titan, Aladdin, and Adamas were used as received; unless otherwise noted, all reactions and manipulations involving air- and moisture-sensitive compounds were performed using standard Schlenk techniques. All solvents were purified and dried using normal procedures. Proton nuclear magnetic resonance (^{1}H NMR) spectra were recorded on Bruker AVANCE III HD400 (400 MHz) and ECZ600s (600 MHz) spectrometers.

Chemical shifts were recorded in parts per million (ppm, δ) relative to tetramethylsilane (δ = 0.00 ppm) or chloroform [δ = 7.260 ppm (400 M) or δ = 7.248 ppm (600 M)]. ^{1}H NMR splitting patterns are designated as singlet (s), doublet (d), triplet (t), quartet (q), dd (doublet of doublets), m (multiplet), and etc. All first-order splitting patterns were assigned on the basis of the appearance of the multiplet. Splitting patterns that could not be easily interpreted are designated as multiplet (m) or broad (br). Carbon nuclear magnetic resonance (^{13}C NMR) spectra were recorded on Bruker AVANCE III HD400 (400 MHz) and ECZ600s (600 MHz) spectrometers. High resolution mass spectral analysis (HRMS) was performed on Thermo Fisher Scientific LTQ FT Ultra mass spectrometer. The determination of ee was performed via chiral HPLC analysis using Shimadzu LC-20AD HPLC workstation. X-ray crystallography analysis was performed on Agilent SuperNova X-ray diffractionmeter. Optical rotations were measured using a 1 mL cell with a 5 dm path length on an INESA SGW-1 polarimeter and are reported as follows: $[\alpha]^{rt}_{D}$ (cin g per 100 mL solvent). Analytical thin-layer chromatography (TLC) was carried out on WFH-203 F254 pre-coated silica gel plate (0.2 mm thickness). Visualization was performed using a UV lamp or 2,4-dinitrophenylhydrazine or potassium permanganate stain.

II. X-ray crystallographic analysis

Method for single crystal cultivation:

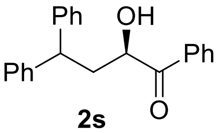

2s

2s: a solid sample (10–20 mg) was dissolved in *i*-PrOH (200–400 μL) in a vial at room temperature, and hexane (500–800 μL) was added into the above solution slowly while keeping the sample all dissolved. Then vial was sealed with a piece of parafilm and stayed quietly for several days to allow the slow evaporation of the solution until a single crystal was obtained.

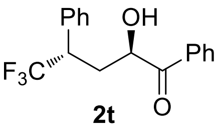

2t

2t: a solid sample (10–20 mg) was dissolved in EtOAc (100–300 μL) in a vial at room temperature, and petroleum ether (400–800 μL) was added into the above solution slowly while keeping the sample all dissolved. Then vial was sealed with a piece of parafilm and stayed quietly for several days to allow the slow evaporation of the solution until a single crystal was obtained.

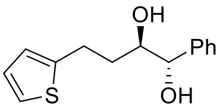

4c

4c: a solid sample (15–20 mg) was dissolved in EtOAc (100–200 μL) in a vial at room temperature, and petroleum ether (400–600 μL) was added into the above solution slowly while keeping the sample all dissolved. Then vial was sealed with a piece of parafilm and stayed quietly for several days to allow the slow evaporation of the solution until a single crystal was obtained.

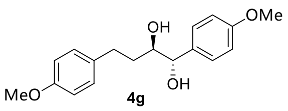

4g: a solid sample (20–30 mg) was dissolved in EtOAc (200–300 μL) in a vial at room temperature, and petroleum ether (300–800 μL) was added into the above solution slowly while keeping the sample all dissolved. Then vial was sealed with a piece of parafilm and stayed quietly for several days to allow the slow evaporation of the solution until a single crystal was obtained.

Table S1. Crystal data and structure refinement for data of compound 2s:

Identification code	**2s**
Empirical formula	$C_{44}H_{40}O_4$
Formula weight	632.805
Temperature/K	149.99(10)
Crystal system	orthorhombic
Space group	$P2_12_12_1$
a/Å	5.74696(9)
b/Å	16.2319(2)
c/Å	18.2433(3)
α /°	90
β /°	90
γ /°	90
Volume/Å^3	1701.81(4)
Z	2
ρ_{calc}g/cm^3	1.235
μ /mm^{-1}	0.611
F(000)	674.0
Crystal size/mm^3	0.05 × 0.03 × 0.03
Radiation	CuKα (λ = 1.54184)
2Θ range for data collection/°	7.28 to 147.76
Index ranges	-6 ≤ h ≤ 5, -17 ≤ k ≤ 19, -22 ≤ l ≤ 21
Reflections collected	8347
Independent reflections	3331 [R_{int} = 0.0274, R_{sigma} = 0.0335]
Data/restraints/parameters	3331/0/218

Goodness-of-fit on F^2	1.039
Final R indexes [I>=2 σ (I)]	R_1 = 0.0321, wR_2 = 0.0806
Final R indexes [all data]	R_1 = 0.0336, wR_2 = 0.0820
Largest diff. peak/hole / e $Å^{-3}$	0.11/-0.19
Flack parameter	0.24(19)

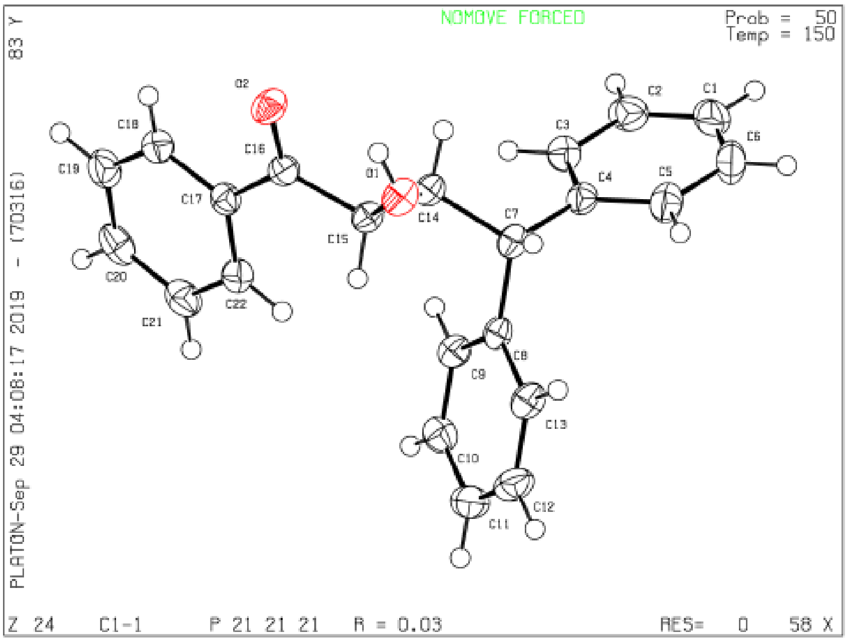

Table S2. Crystal data and structure refinement for data of compound 2t:

Identification code	**2t**
Empirical formula	$C_{17}H_{15}F_3O_2$
Formula weight	308.29
Temperature/K	150.00(10)
Crystal system	monoclinic
Space group	P21
a/Å	9.6721(2)
b/Å	5.82400(10)
c/Å	13.3469(3)
α/°	90
β/°	98.842(2)
γ/°	90
Volume/Å3	742.90(3)

Z	2
ρcalcg/cm3	1.378
μ/mm-1	0.980
F(000)	320.0
Crystal size/mm3	0.1 × 0.01 × 0.01
Radiation	CuKα (λ = 1.54184)
2Θ range for data collection/°	6.702 to 147.952
Index ranges	-11 ≤ h ≤ 11, -7 ≤ k ≤ 6, -16 ≤ l ≤ 16
Reflections collected	6686
Independent reflections	2870 [Rint = 0.0339, Rsigma = 0.0420]
Data/restraints/parameters	2870/1/200
Goodness-of-fit on F2	1.056
Final R indexes [I>=2σ (I)]	R1 = 0.0361, wR2 = 0.0901
Final R indexes [all data]	R1 = 0.0405, wR2 = 0.0922
Largest diff. peak/hole / e Å-3	0.17/-0.20
Flack parameter	-0.10(10)

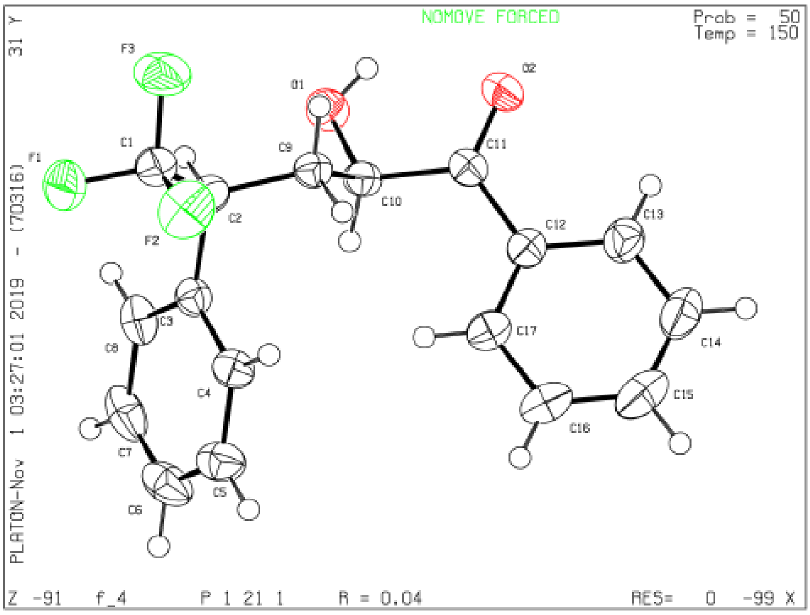

Table S3. Crystal data and structure refinement for data of compound 4c:

Identification code	**4c**
Empirical formula	$C_{14}H_{17}O_2S$

Formula weight	249.36
Temperature/K	149.99(10)
Crystal system	trigonal
Space group	P32
a/Å	13.93586(16)
b/Å	13.93586(16)
c/Å	5.48763(10)
α/°	90
β/°	90
γ/°	120
Volume/Å3	922.96(2)
Z	3
ρcalcg/cm3	1.3458
μ/mm-1	2.226
F(000)	401.1
Crystal size/mm3	0.2 × 0.01 × 0.01
Radiation	CuKα (λ = 1.54184)
2Θ range for data collection/°	7.32 to 148.06
Index ranges	-16 ⩽ h ⩽ 17, -17 ⩽ k ⩽ 16, -6 ⩽ l ⩽ 6
Reflections collected	9069
Independent reflections	2305 [Rint = 0.0540, Rsigma = 0.0436]
Data/restraints/parameters	2305/1/156
Goodness-of-fit on F2	1.032
Final R indexes [I>=2σ (I)]	R1 = 0.0865, wR2 = 0.2265
Final R indexes [all data]	R1 = 0.0876, wR2 = 0.2267
Largest diff. peak/hole / e Å-3	0.61/-0.74
Flack parameter	0.13(6)

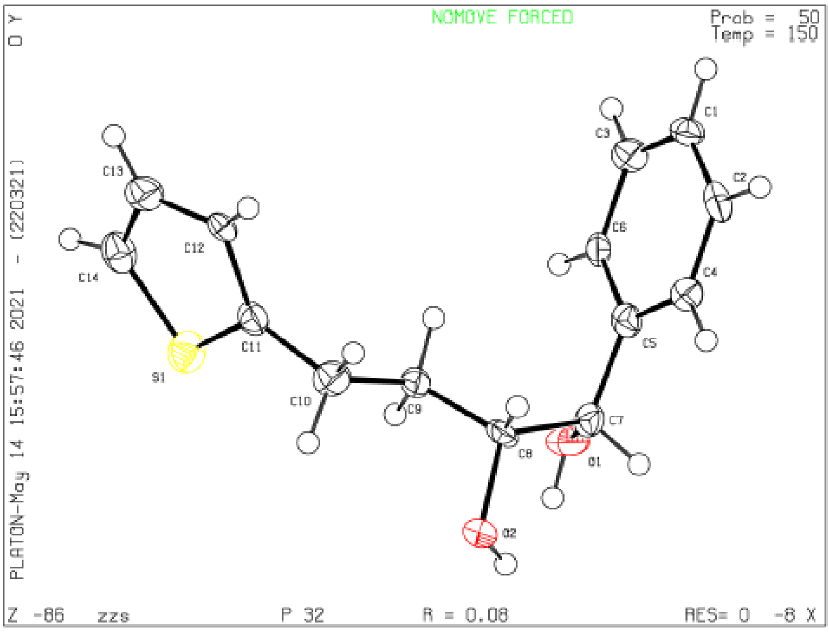

Table S4. Crystal data and structure refinement for data of compound 4g:

Identification code	**4g**
Empirical formula	$C_{18}H_{22}O_4$
Formula weight	302.36
Temperature/K	150(2) K
Crystal system	monoclinic
Space group	C2
a/Å	32.3887(15)
b/Å	5.3199(2)
c/Å	9.0200(4)
α /°	90
β /°	96.207(2)
γ /°	90
Volume/Å3	1545.08(11)
Z	4
ρ calcg/cm3	1.3000
μ /mm^{-1}	0.737
F(000)	648.0
Crystal size/mm^3	0.2 × 0.1 × 0.1

Radiation	CuKα (λ = 1.54184)
2Θ range for data collection/°	7.32 to 148.06
Index ranges	-38 < = h < = 38, -5 < = k< = 6, -10 < = l < = 10
Reflections collected	9435
Independent reflections	2727 [R(int) = 0.0451]
Data/restraints/parameters	2727/6/209
Goodness-of-fit on F2	1.050
Final R indexes [I>=2σ (I)]	R1 = 0.0528, wR2 = 0.1235
Final R indexes [all data]	R1 = 0.0530, wR2 = 0.1236
Largest diff. peak/hole / e Å^{-3}	0.405/-0.247
Flack parameter	0.17(8)

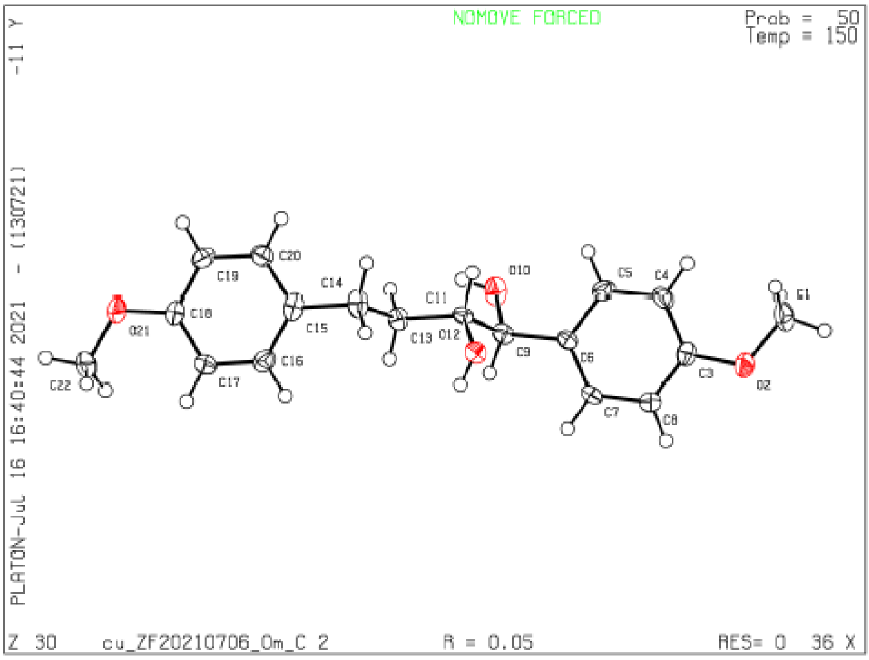

III. General procedures for the preparation of substrates

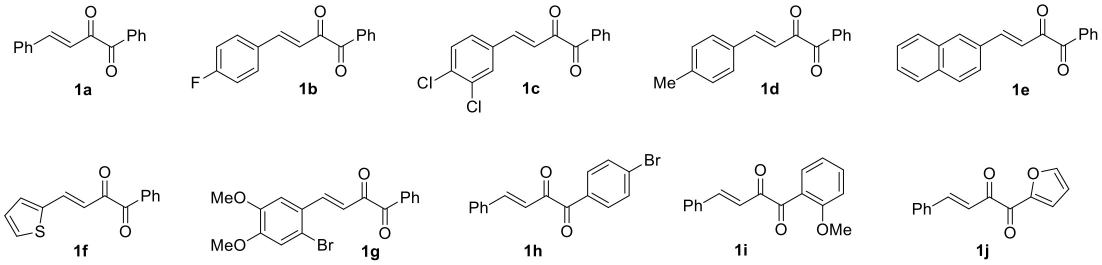

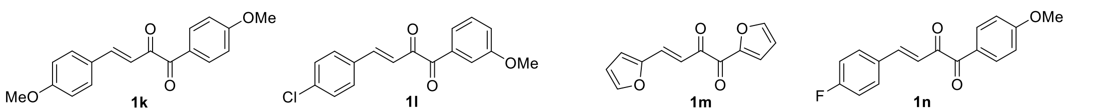

1k 1l 1m 1n

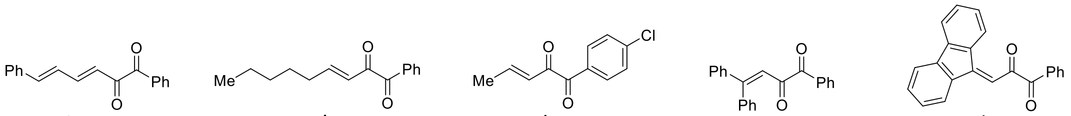

1o 1p 1q 1r 1s

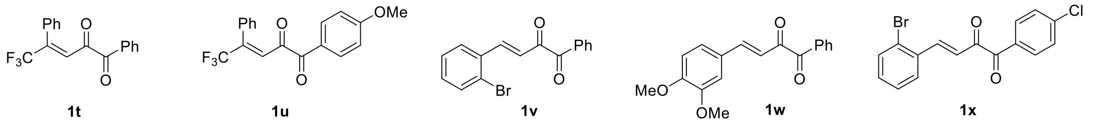

1t 1u 1v 1w 1x

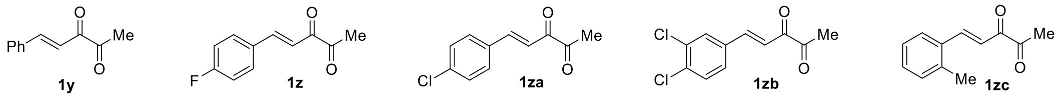

1y 1z 1za 1zb 1zc

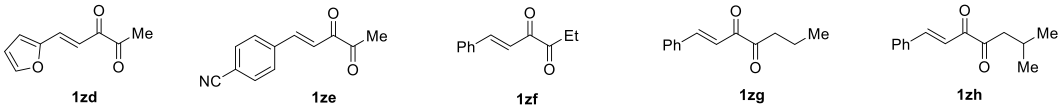

1zd 1ze 1zf 1zg 1zh

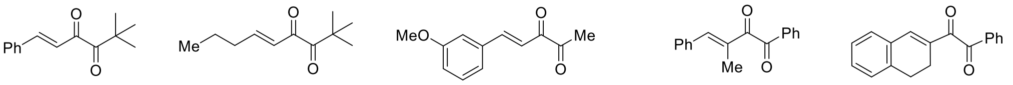

1zi 1zj 1zk 1zl 1zm

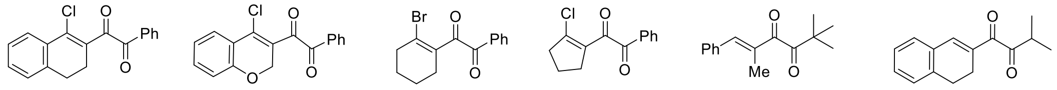

1zn 1zo 1zp 1zq 1zr 1zs

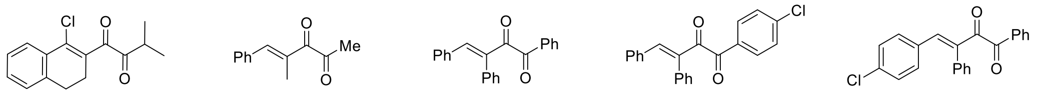

1zt 1zu 1zv 1zw 1zx

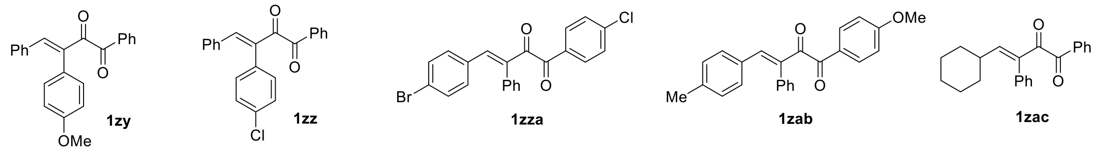

1zy 1zz 1zza 1zab 1zac

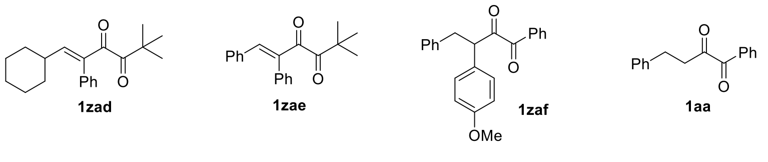

1zad 1zae 1zaf 1aa

1. Substrates **1a**[1], **1b**[1], **1c**[1], **1d**[1], **1e**[1], **1i**[2], **1k**[1], **1m**[3], **1n**[1], **1q**[2], **1zu**[4] are known compounds and were prepared according to the literature reports.

2. Substrates **1f**, **1g**, **1h**, **1p**, **1l**, **1o**, **1v**, **1w**, **1x** were synthesized according to the

following procedure.

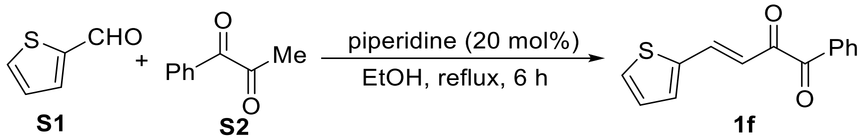

Typically, to a dried 100 mL two-necked flask equipped with a magnetic stirrer was added thiophene-2-carbaldehyde **S1** (1.3 g, 10.0 mmol), 1-phenylpropane-1,2-dione **S2** (1.6 g, 10.0 mmol) and piperidine (0.17 g, 2 mmol, 20 mol%). The mixture was refluxed in EtOH (10 mL) for 6 h and monitored by TLC. The reaction mixture was cooled to room temperature. The solvent was removed and the residue was extracted with ethyl acetate (3 × 100 mL) and saturated brine (3 × 100 mL). The combined organic phase was dried with anhydrous Na_2SO_4 and concentrated under reduced pressure. The residue was purified by chromatography (petroleum ether/ethyl acetate, v:v = 7:1) to afford product **1f** (460 mg, 19% yield).

3. Substrates **1r**, **1s**, **1t**, **1u** were synthesized according to the following procedure.

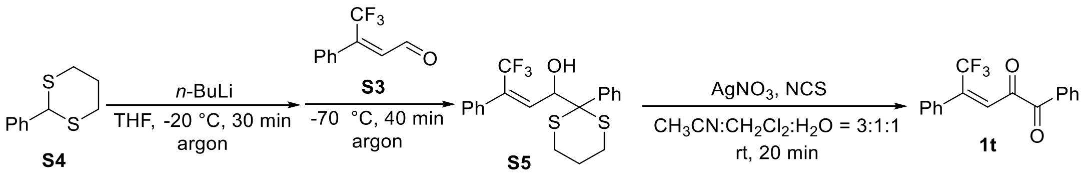

Typically, to a dried 100 mL two-necked flask containing THF (15 mL) at –20 ˚C under argon atmosphere was added 2-phenyl-1,3-dithiane **S4** (1.0 g, 5.1 mmol). To this mixture, *n*-BuLi (3.1 mL, 7.8 mmol, 2.5 M in hexane) was added at –20 ºC. The mixture was stirred at the same temperature for 30 min. After cooling down to –70 ºC, (*Z*)-4,4,4-trifluoro-3-phenylbut-2-enal **S3** (1.0 g, 5.1 mmol) was added dropwise via a syringe. The reaction was stirred for additional 40 min at –70 ºC, and then quenched with saturated aqueous NH_4Cl (10 mL) and warmed to room temperature. The reaction mixture was extracted with ethyl acetate (3 × 50 mL) and saturated brine (3 × 50 mL). The combined organic phase was dried with anhydrous Na_2SO_4 and concentrated under reduced pressure. The residue was purified by chromatography (petroleum ether/ethyl acetate, v:v = 15:1) to afford product **S5** (1.3 g, 64% yield).

To the solution of **S5** (1.3 g, 3.3 mmol) in $CH_3CN/CH_2Cl_2/H_2O$ (3:1:1, 15 mL/5 mL /5 mL) were added the mixture of NCS (1.7 g, 10 mmol) and $AgNO_3$ (2.4 g, 11.3 mmol) at room temperature. The mixture was stirred at room temperature for 20 min. After completion, saturated aqueous $Na_2S_2O_3$ solution (5 mL) was added. Then the reaction mixture was extracted with CH_2Cl_2 (3 × 10 mL) and saturated brine (3 × 10 mL), dried over anhydrous Na_2SO_4. The residue was purified by chromatography

(petroleum ether/ethyl acetate, v:v = 7:1) to afford product **1t** (291 mg, 29% yield).

4. Substrates **1j**, **1aa** were synthesized according to the following procedure.

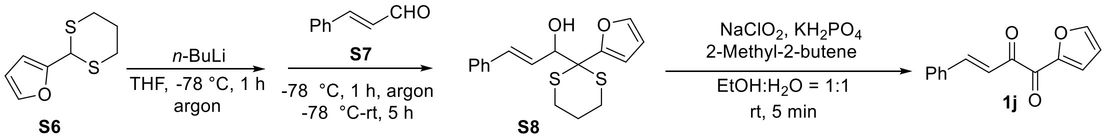

Typically, to a dried 250 mL two-necked flask containing THF (50 mL) at –78 ˚C under argon atmosphere was added 2-(1,3-dithian-2-yl)furan **S6** (1.9 g, 10.0 mmol). To this mixture, *n*-BuLi (4.4 mL, 11.0 mmol, 2.5 M in hexane) was added at –78 ºC. The mixture was stirred at the same temperature for 1 h. Then, cinnamaldehyde **S7** (1.5 g, 11.0 mmol) in 10 mL THF was added dropwise via a syringe. The reaction was stirred for additional 1 h at –78 ºC. Next, the reaction was stirred for additional 5 h at room temperature. The reaction was quenched with saturated aqueous NH_4Cl (15 mL) at 0 ºC and warmed to room temperature. The reaction mixture was extracted with ethyl acetate (3 × 100 mL) and saturated brine (3 × 100 mL). The combined organic phase was dried with anhydrous Na_2SO_4 and concentrated under reduced pressure. The residue was purified by chromatography (petroleum ether/ethyl acetate, v:v = 7:1) to afford product **S8** (2.6 g, 82% yield).

To the solution of **S8** (1.6 g, 5.0 mmol) in EtOH/H_2O (1:1, 25 mL/25 mL) were added the mixture of 2-methyl-2-butene (3.5 g, 50 mmol), $NaClO_2$ (3.4 g, 7.5 mmol) and KH_2PO_4 (1.4 g, 10 mmol) at room temperature. The mixture was stirred at room temperature for 5 min. After completion, saturated aqueous $Na_2S_2O_3$ solution (10 mL) was added. Then the reaction mixture was extracted with CH_2Cl_2 (3 × 30 mL) and saturated brine (3 × 20 mL), dried over anhydrous Na_2SO_4. The residue was purified by chromatography (petroleum ether/ethyl acetate, v:v = 15:1) to afford product **1j** (927 g, 82% yield).

5. Substrates **1y**, **1z**, **1za**, **1zb**, **1zd**, **1zf** are known compounds and were prepared according to the literature reports.[1]

6. Substrates **1zc**, **1ze, 1zk** were synthesized according to the following procedure.

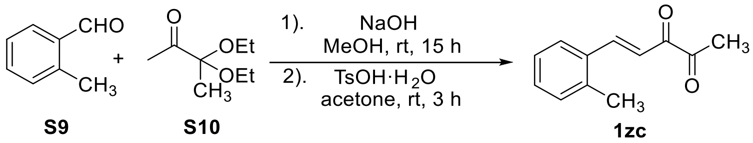

Typically, to a dry 250 mL round bottom flask was added a mixture of 2-methylbenzaldehyde **S9** (2.4 g, 20 mmol) and 3,3-diethoxybutane-2-one **S10** (3.5 g, 20 mmol) in MeOH (40 mL). Then, 23.0 mL of 5.0 M aqueous sodium hydroxide

solution was added. The mixture was stirred for 15 h at room temperature. After completion, MeOH was removed under reduced pressure and the remaining aqueous phase was extracted with CH_2Cl_2 (3 × 200 mL).and saturated brine (3 × 100 mL). The combined organic phase was dried with anhydrous Na_2SO_4 and concentrated under reduced pressure. The product was pure enough used in the next step directly.

In a 250 mL of round bottom flask, the product was dissolved in acetone (80 mL). To this solution, *p*-TSA·H_2O (760.0 mg, 4.0 mmol, 20 mol%) was added and the mixture was stirred for 3 h at room temperature. The solvent was evaporated under reduced pressure. the residue was extracted with CH_2Cl_2 (3 × 100 mL) and saturated brine (3 × 50 mL). The combined organic phase was dried with anhydrous Na_2SO_4 and concentrated under reduced pressure. The residue was purified by chromatography (petroleum ether/ethyl acetate, v:v = 20:1) to afford product **1zc** (527 mg, 14% yield).

7. Substrates **1zj**, **1zh** were synthesized according to the following procedure.

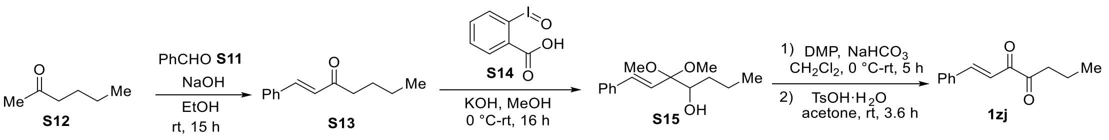

Typically, to a dry 100 mL round bottom flask was added a mixture of benzaldehyde **S11** (4.3 g, 46 mmol) and hexan-2-one **S12** (4.6 g, 46 mmol) in EtOH (14 mL). Then, 23.0 mL of 2.5 M aqueous sodium hydroxide solution was added. The mixture was stirred at room temperature for 15 h. After completion, EtOH was removed under reduced pressure. The reaction mixture was extracted with ethyl acetate (3 × 200 mL) and saturated brine (3 × 100 mL). The combined organic phase was dried with anhydrous Na_2SO_4 and concentrated under reduced pressure. The residue was purified by chromatography (petroleum ether/ethyl acetate, v:v = 30:1) to afford product **S13** (5.4 g, 62% yield).

Typically, to a dried 150 mL two-necked flask containing MeOH (30 mL) at 0 ˚C added KOH (2.5 g, 45.0 mmol). To this mixture, **S13** (2.8 mL, 15.0 mmol) in MeOH (30 mL) was added at 0 ºC. Then, **S14** was added at the same temperature in several portions. The reaction was warmed to room temperature and stirred for additional 16 h. The reaction mixture was extracted with CH_2Cl_2 (3 × 100 mL) and saturated brine (3 × 100 mL). The combined organic phase was dried with anhydrous Na_2SO_4 and concentrated under reduced pressure. The residue was purified by chromatography (petroleum ether/ethyl acetate, v:v = 20:1) to afford product **S15** (1.8 g, 48% yield).

Typically, to a dry 250 mL round bottom flask was added a mixture of **S15** (1.5 g,

5.9 mmol), DMP (2.5 g, 17.7 mmol) and $NaHCO_3$ (0.5 g, 17.1 mmol) in CH_2Cl_2 (40 mL) at 0 ˚C. The mixture was warmed to room temperature and stirred for 5 h. After completion, saturated aqueous $Na_2S_2O_3$ solution (10 mL) was added. Then the reaction mixture was extracted with CH_2Cl_2 (3 × 50 mL) and saturated brine (3 × 40 mL). The combined organic phase was dried with anhydrous Na_2SO_4 and concentrated under reduced pressure. The product was pure enough used in the next step directly.

In a 100 mL of round bottom flask, the product was dissolved in acetone (20 mL). To this solution, *p*-TSA·H_2O (220.0 mg, 1.2 mmol, 20 mol %) was added and the mixture was stirred at room temperature for 3 h. The solvent was evaporated under reduced pressure. The residue was extracted with CH_2Cl_2 (3 × 40 mL) and saturated brine (3 × 20 mL). The combined organic phase was dried with anhydrous Na_2SO_4 and concentrated under reduced pressure. The residue was purified by chromatography (petroleum ether/ethyl acetate, v:v = 50:1) to afford product **1zj** (474.0 mg, 34% yield).

8. Substrates **1zi** and **1zj** were synthesized according to the following procedure.

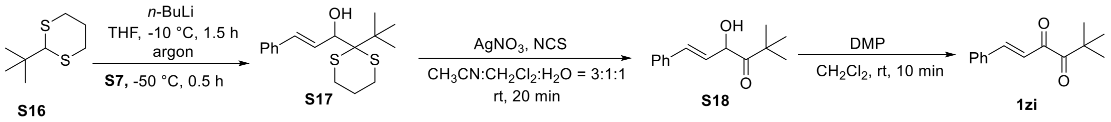

Typically, to a dried 100 mL two-necked flask containing THF (30 mL) at –10 ˚C under argon atmosphere was added 2-*tert*-butyl-1,3-dithiane **S16** (1.8 g, 10.0 mmol). To this mixture, *n*-BuLi (6.0 mL, 15.0 mmol, 2.5 M in hexane) was added at –10 ºC. The mixture was stirred at the same temperature for 1.5 h. Then, cinnamaldehyde **S7** (1.3 g, 10.0 mmol) was added dropwise via a syringe. The reaction was stirred at –50 ºC for additional 30 min. The reaction was quenched with saturated aqueous NH_4Cl (15 mL) at 0 ºC and warmed to room temperature. The residue was extracted with ethyl acetate (3 × 100 mL) and saturated brine (3 × 100 mL). The combined organic phase was dried with anhydrous Na_2SO_4 and concentrated under reduced pressure. The residue was purified by chromatography (petroleum ether/ethyl acetate, v:v = 20:1) to afford product **S17** (1.3 g, 42% yield).

To the solution of **S17** (1.3 g, 4.2 mmol) in $CH_3CN/CH_2Cl_2/H_2O$ (3:1:1, 15 mL/5 mL/5 mL) were added the mixture of NCS (2.2 g, 16.8 mmol) and $AgNO_3$ (3.2 g, 18.9 mmol) at room temperature. The mixture was stirred at room temperature for 20 min. After completion, saturated aqueous $Na_2S_2O_3$ solution (8 mL) was added. Then the reaction mixture was extracted with CH_2Cl_2 (3 × 20 mL) and saturated brine (3 × 20 mL), dried over anhydrous Na_2SO_4. The residue was purified by chromatography (petroleum ether/ethyl acetate, v:v = 20:1) to afford product **S18** (0.4 g, 44% yield).

Typically, in a dry 100 mL round bottom flask was added a mixture of **S18** (391.6 mg, 1.8 mmol) and DMP (1.3 g, 3.1 mmol) in CH_2Cl_2 (12 mL) at room temperature. The mixture was stirred for 10 min. After completion, saturated aqueous $Na_2S_2O_3$ (10 mL) and $NaHCO_3$ solution were added. Then the reaction mixture was extracted with CH_2Cl_2 (3 × 30 mL) and saturated brine (3 × 20 mL). The combined organic phase was dried with anhydrous Na_2SO_4 and concentrated under reduced pressure. The residue was purified by chromatography (petroleum ether/ethyl acetate, v:v = 10:1) to afford product **1zi** (370 mg, 95% yield).

9. Substrates **1zl**, **1zm**, **1zn**, **1zo**, **1zp**, **1zq**, **1zt** were synthesized according to the following procedure.

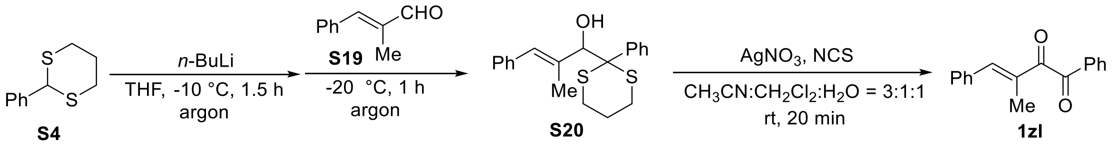

Typically, to a dried 100 mL two-necked flask containing THF (50 mL) at –20 ˚C under argon atmosphere was added 2-phenyl-1,3-dithiane **S4** (2.0 g, 10.0 mmol). To this mixture, *n*-BuLi (6.0 mL, 15.0 mmol, 2.5 M in hexane) was added at –20 ºC. The mixture was stirred at the same temperature for 1 h. Then, (*E*)-2-methyl-3-phenylacrylaldehyde **S19** (1.5 g, 10.0 mmol) was added dropwise via a syringe. The reaction was stirred at –50 ºC for additional 30 min. The reaction was quenched with saturated aqueous NH_4Cl (15 mL) at 0 ºC and warmed to room temperature. The residue was extracted with ethyl acetate (3 × 100 mL) and saturated brine (3 × 100 mL). The combined organic phase was dried with anhydrous Na_2SO_4 and concentrated under reduced pressure. The residue was purified by chromatography (petroleum ether/ethyl acetate, v:v = 15:1) to afford product **S20** (2.1 g, 61% yield).

To the solution of **S20** (1.7 g, 5.0 mmol) in $CH_3CN/CH_2Cl_2/H_2O$ (3:1:1, 12 mL/4 mL/4 mL) was added the mixture of NCS (1.5 g, 11.6 mmol) and $AgNO_3$ (2.2 g, 13.1 mmol) at room temperature. The mixture was stirred at room temperature for 20 min=. After completion, saturated aqueous $Na_2S_2O_3$ solution (5 mL) was added. Then the reaction mixture was extracted with CH_2Cl_2 (3 × 20 mL) and saturated brine (3 × 20 mL), dried over anhydrous Na_2SO_4. The residue was purified by chromatography (petroleum ether/ethyl acetate, v:v = 30:1) to afford product **1zl** (519 mg, 42% yield).

10. Substrates **1zr** and **1zs** were synthesized according to the following procedure.

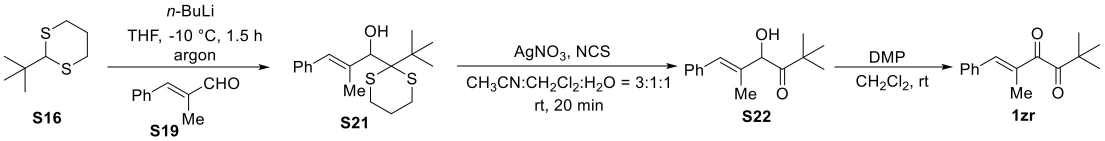

Typically, to a dried 100 mL two-necked flask containing THF (30 mL) at –10 ˚C under argon atmosphere was added 2-*tert*-butyl-1,3-dithiane **S16** (1.8 g, 10.0 mmol). To this mixture, *n*-BuLi (6.0 mL, 15.0 mmol, 2.5 M in hexane) was added at –10 ºC. The mixture was stirred at the same temperature for 1.5 h. Then, (*E*)-2-methyl-3-phenylacrylaldehyde **S19** (1.5 g, 10.0 mmol) was added dropwise via a syringe. The reaction was stirred at –50 ºC for additional 30 min. The reaction was quenched with saturated aqueous NH_4Cl (15 mL) at 0 ºC and warmed to room temperature. The residue was extracted with ethyl acetate (3 × 100 mL) and saturated brine (3 × 100 mL). The combined organic phase was dried with anhydrous Na_2SO_4 and concentrated under reduced pressure. The residue was purified by chromatography (petroleum ether/ethyl acetate, v:v = 20:1) to afford product **S21** (1.3 g, 42% yield).

To the solution of **S21** (1.0 g, 3.1 mmol) in $CH_3CN/CH_2Cl_2/H_2O$ (3:1:1, 15 mL/5 mL/5 mL) were added the mixture of NCS (1.7 g, 12.4 mmol) and $AgNO_3$ (2.4 g, 14.0 mmol) at room temperature. The mixture was stirred at room temperature for 20 min. After completion, saturated aqueous $Na_2S_2O_3$ solution (8 mL) was added. Then the reaction mixture was extracted with CH_2Cl_2 (3 × 20 mL) and saturated brine (3 × 20 mL), dried over anhydrous Na_2SO_4. The residue was purified by chromatography (petroleum ether/ethyl acetate, v:v = 20:1) to afford product **S22** (0.6 g, 83% yield).

Typically, in a dry 100 mL round bottom flask was added a mixture of **S22** (0.6, 2.6 mmol) and DMP (1.8 g, 4.4 mmol) in CH_2Cl_2 (15 mL) at room temperature. The mixture was stirred for 10 min. After completion, saturated aqueous $Na_2S_2O_3$ (10 mL) and $NaHCO_3$ solution were added. Then the reaction mixture was extracted with CH_2Cl_2 (3 × 30 mL) and saturated brine (3 × 30 mL). The combined organic phase was dried with anhydrous Na_2SO_4 and concentrated under reduced pressure. The residue was purified by chromatography (petroleum ether/ethyl acetate, v:v = 20:1) to afford product **1zr** (462 mg, 77% yield).

11. Substrate **1zu** were synthesized according to the following procedure.

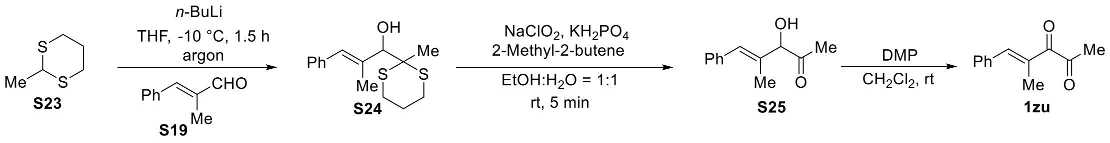

Typically, to a dried 100 mL two-necked flask equipped containing THF (30 mL) at –10 ˚C under argon atmosphere was added 2-methyl-1,3-dithiane **S23** (2.0 g, 15.0 mmol). To this mixture, *n*-BuLi (9.0 mL, 15.0 mmol, 2.5 M in hexane) was added at –10 ºC. The mixture was stirred at the same temperature for 1 h. Then, (*E*)-2-methyl-3-phenylacrylaldehyde **S19** (2.2 g, 10.0 mmol) was added dropwise via a syringe. The

reaction was stirred for additional 1.5 h at –10 ºC. The reaction was quenched with saturated aqueous NH_4Cl (20 mL) at –10 ºC and warmed to room temperature. The reside was extracted with ethyl acetate (3 × 100 mL) and saturated brine (3 × 100 mL). The combined organic phase was dried with anhydrous Na_2SO_4 and concentrated under reduced pressure. The residue was purified by chromatography (petroleum ether/ethyl acetate, v:v = 20:1) to afford product **S24** (3.8 g, 90% yield).

To the solution of **S24** (1.8 g, 6.4 mmol) in EtOH/H_2O (1:1, 25 mL/25 mL) was added the mixture of 2-methyl-2-butene (4.5 g, 64.0 mmol), $NaClO_2$ (4.4 g, 9.6 mmol) and KH_2PO_4 (1.8 g, 13 mmol) at room temperature. The mixture was stirred at room temperature for 5 min. After completion, saturated aqueous $Na_2S_2O_3$ solution (15 mL) was added. Then the reaction mixture was extracted with CH_2Cl_2 (3 × 50 mL) and saturated brine (3 × 30 mL), dried over anhydrous Na_2SO_4. The residue was purified by chromatography (petroleum ether/ethyl acetate, v:v = 9:1) to afford product **S25** (0.2 g, 16% yield).

Typically, in a dry 100 mL round bottom flask was added a mixture of **S25** (0.2, 1.0 mmol) and DMP (0.5 g, 4.4 mmol) in CH_2Cl_2 (5 mL) at room temperature. The mixture was stirred for 10 min. After completion, saturated aqueous $Na_2S_2O_3$ (3 mL) and $NaHCO_3$ solution were added. Then the reaction mixture was extracted with CH_2Cl_2 (3 × 10 mL) and saturated brine (3 × 10 mL). The combined organic phase was dried with anhydrous Na_2SO_4 and concentrated under reduced pressure. The residue was purified by chromatography (petroleum ether/ethyl acetate, v:v = 20:1) to afford product **1zu** (128 g, 62% yield).

12. Substrates **1zv**, **1zw**, **1zx**, **1zy**, **1zz**, **1zaa**, **1zab**, **1zae** were synthesized according to the synthetic procedure of **1zl**.

13. Substrates **1zac** and **1zad** were synthesized according to the synthetic procedure of **1zr**.

IV. Conditions optimization of the transfer hydrogenation of 1zv

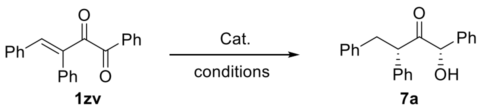

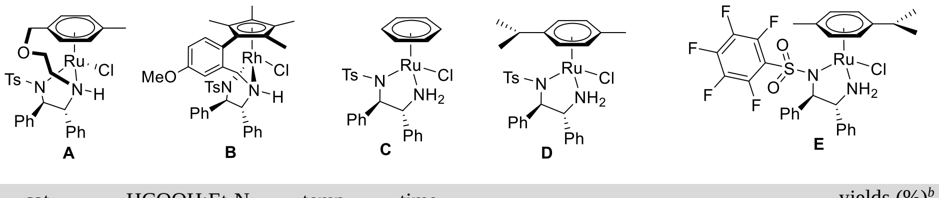

entry	cat. (mol %)	HCOOH:Et_3N (v/v, M)	temp (ºC)	time (h)	additive (equiv)	dr	yields (%)[b], ee (%)[c] 7a
1	**A** (2.0)	1:3 (0.2)	rt	2.7	none	1.6:1	46, 61

2	**B** (2.0)	1:3 (0.2)	rt	2	none	2.3:1	55, 79
3	**C** (2.0)	1:3 (0.2)	rt	1.5	none	1.5:1	37, 52
4	**D** (2.0)	1:3 (0.2)	rt	2	none	1:1	42, 46
5	**E** (2.0)	1:3 (0.2)	rt	2	none	1:1.1	45, 68
6	**B** (1.0)	1:3 (0.2)	rt	0.5	none	7:1	72, 98
7	**B** (1.0)	1:3 (0.4)	10-15	7	none	3:1	65, 87
8	**B** (1.0)	1:3 (0.4)	rt	2	$Ti(O^iPr)_4$ (1)	1.6:1	47, 59
9	**B** (2.0)	1:3 (0.4)	0	6	none	2.2:1	55, 84
10	**B** (1.0)	1:3 (0.2)	rt	2	none	2:1	54, 74
11	**B** (1.0)	1:3 (0.4)	0-15	4	none	2.5:1	54, 84
12	**B** (1.0)	1:3 (0.4)	10	3	none	3:1	39, 87
13	**B** (1.0)	1:3 (0.4)	20	2	none	2.3:1	50, -

[a]Reaction conditions: **1zv** (0.2 mmol), HCO_2H/Et_3N (1:3 v/v), under argon atmosphere. [b]All yields are isolated yields and were based on **1zv**. [c]The ee values were determined via HPLC analysis on a chiral stationary phase.

V. Typical procedure for the transfer hydrogenation

1. Typical procedure of the synthesis of compounds **2**:

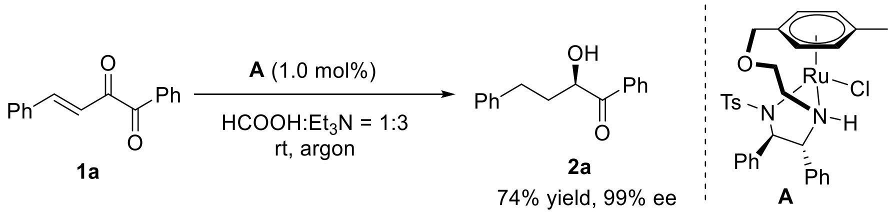

A mixture of unsaturated 1,2-diketone **1a** (47.2 mg, 0.2 mmol) and the Ru catalyst **A** (1.3 mg, 1 mol%) in 1.0 mL formic acid/triethylamine (v:v = 1:3) mixture was stirred at room temperature under argon atmosphere. After completion of the reaction as indicated by TLC, it was extracted with CH_2Cl_2 (3 × 20 mL) and the organic extract was dried over anhydrous Na_2SO_4 followed by the removal of solvent in a rotary evaporator. The residue was purified by chromatography (petroleum ether/ethyl acetate, v:v = 5:1) to afford product **2a** (35.5 mg, 74% yield).

2. Typical procedure of the synthesis of compounds **5**:

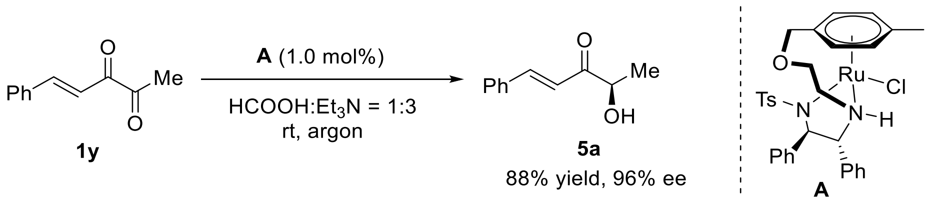

A mixture of unsaturated 1,2-diketone **1y** (34.8 mg, 0.2 mmol) and the Ru catalyst **A** (1.3 mg, 1 mol%) in 1.0 mL formic acid/triethylamine (v:v = 1:3) mixture was stirred at room temperature under argon atmosphere. After completion of the reaction as indicated by TLC, it was extracted with CH_2Cl_2 (3 × 20 mL) and the organic extract was dried over anhydrous Na_2SO_4 followed by the removal of solvent in a

rotary evaporator. The residue was purified by chromatography (petroleum ether/ethyl acetate, v:v = 5:1) to afford product **5a** (31.3 mg, 88% yield, 96% ee).

3. Typical procedure of the synthesis of compounds **6**:

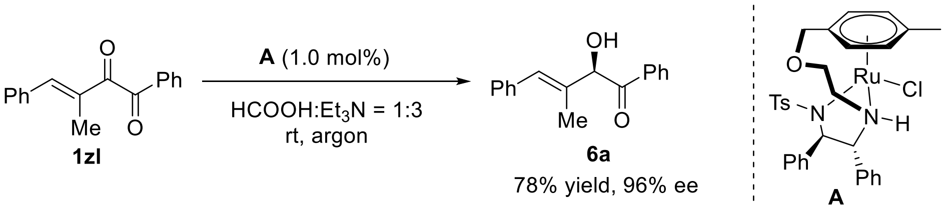

A mixture of unsaturated 1,2-diketone **1zl** (47.2 mg, 0.2 mmol) and the Ru catalyst **A** (1.3 mg, 1 mol%) in 1.0 mL formic acid/triethylamine (v:v = 1:3) mixture was stirred at room temperature under argon atmosphere. After completion of the reaction as indicated by TLC, it was extracted with CH_2Cl_2 (3 × 20 mL) and the organic extract was dried over anhydrous Na_2SO_4 followed by the removal of solvent in a rotary evaporator. The residue was purified by chromatography (petroleum ether/ethyl acetate, v:v = 7:1) to afford product **6a** (39.3 mg, 78% yield, 96% ee).

4. Typical procedure of the synthesis of compounds **7**:

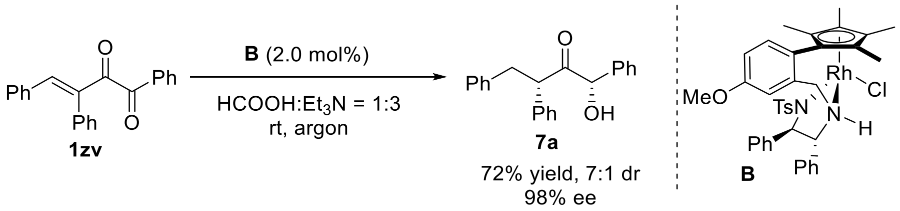

A mixture of unsaturated 1,2-diketone **1zv** (62.8 mg, 0.2 mmol) and the Rh catalyst **B** (3.0 mg, 2 mol%) in 1.0 mL formic acid/triethylamine (v:v = 1:3) mixture was stirred at room temperature under argon atmosphere. After completion of the reaction as indicated by TLC, it was extracted with CH_2Cl_2 (3 × 20 mL) and the organic extract was dried over anhydrous Na_2SO_4 followed by the removal of solvent in a rotary evaporator. The residue was purified by chromatography (petroleum ether/ethyl acetate, v:v = 20:1) to afford product **7a** (45.5 mg, 72% yield, 98% ee, 7:1 dr).

5. Typical procedure of the synthesis of compounds **3**:

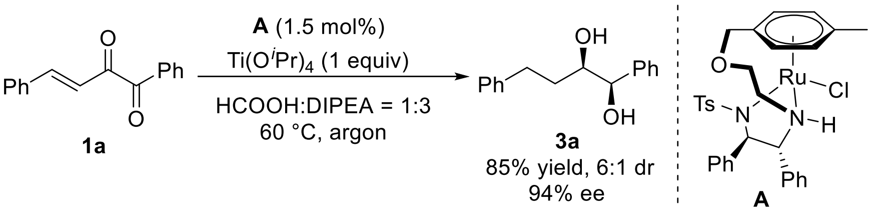

A mixture of unsaturated 1,2-diketone **1a** (47.2 mg, 0.2 mmol), the catalyst **A** (2.0 mg, 1.5 mol%) and Ti(O^{i}Pr)$_4$ (59.2 μL, 1 equiv) in 1.0 mL formic acid/ N,N-diisopropylethylamine (v:v = 1:3) mixture was stirred at 60 ºC under argon atmosphere in oil bath for 12 h. After completion of the reaction as indicated by TLC, it was extracted with CH_2Cl_2 (3 × 20 mL) and the organic extract was dried over anhydrous Na_2SO_4 followed by the removal of solvent in a rotary evaporator. The residue was purified by flash chromatography (petroleum ether/ethyl acetate, v:v = 1:1) to afford product **3a** (41.1 mg, 85% yield, 6:1 dr).

6. Typical procedure of the synthesis of compounds **4**:

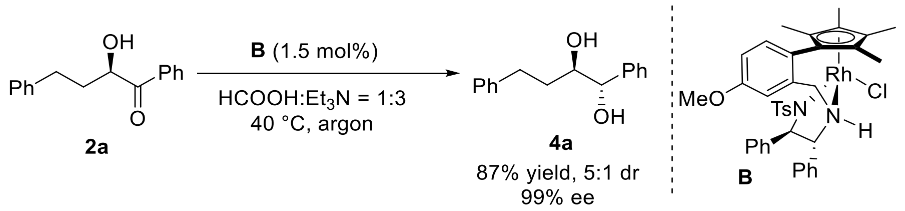

A mixture of **2a** (48.0 mg, 0.2 mmol) and the catalyst **B** (2.3 mg, 1.5 mol%) in 1.0 mL formic acid/triethylamine (v:v = 1:3) mixture was stirred at 40 ºC in oil bath under argon atmosphere. After completion of the reaction as indicated by TLC, it was extracted with CH_2Cl_2 (3 × 20 mL) and the organic extract was dried over anhydrous Na_2SO_4 followed by the removal of solvent in a rotary evaporator. The residue was purified by flash chromatography (petroleum ether/ethyl acetate, v:v = 1:1) to afford product **4a** (42.1 mg, 87% yield, 97% ee, 5:1 dr).

7. Typical procedure of the synthesis of compounds **8**:

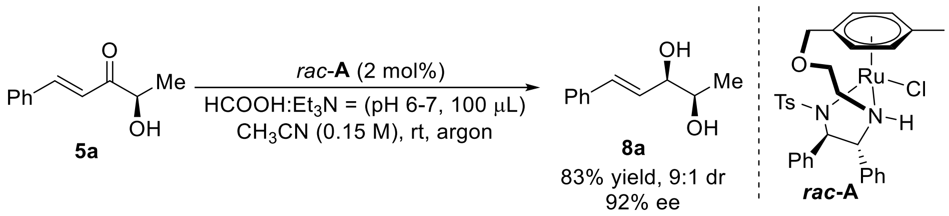

To a mixture of **5a** (35.2 mg, 0.2 mmol) and the racemic catalyst **A** (2.6 mg, 2 mol%) in CH_3CN (0.15 M), =100 μL formic acid/triethylamine (v:v = 1:3) mixture was added and the mixture was stirred at room temperature under argon atmosphere. After completion of the reaction as indicated by TLC, it was extracted with CH_2Cl_2 (3 × 20 mL) and the organic extract was dried over anhydrous Na_2SO_4 followed by the removal of solvent in a rotary evaporator. The residue was purified by flash chromatography (petroleum ether/ethyl acetate, v:v = 1:1) to afford product **8a** (29.6 mg, 83% yield, 92% ee, 9:1 dr).

8. Typical procedure of the synthesis of compounds **9**:

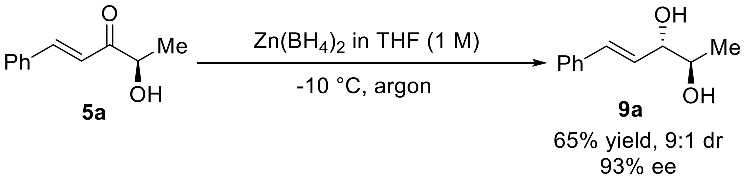

A mixture of **5a** (35.2 mg, 0.2 mmol) was added to $Zn(BH_4)_2$ (1 mL, 1M in THF) and the mixture was stirred at –10 ºC under argon atmosphere. After completion of the reaction as indicated by TLC, it was extracted with CH_2Cl_2 (3 × 20 mL) and the organic extract was dried over anhydrous Na_2SO_4 followed by the removal of solvent in a rotary evaporator. The residue was purified by flash chromatography (petroleum ether/ethyl acetate, v:v = 1:1) to afford product **9a** (23.2 mg, 65% yield, 93% ee, 9:1 dr).

VI. Procedures for the derivatizations of products

1. Typical procedure of the synthesis of compounds **10a**:

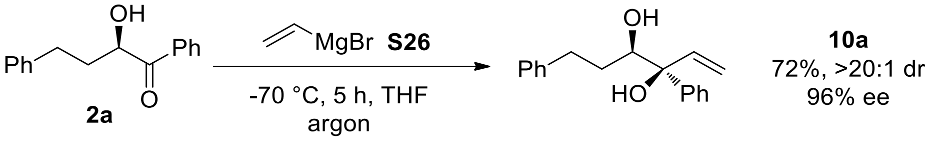

A mixture of **2a** (22.6 mg, 0.094 mmol) in THF (0.5 mL) was added to vinylmagnesium bromide (0.3 mL, 1M in THF) and the mixture was stirred at –70 ºC under argon atmosphere. After completion of the reaction as indicated by TLC, saturated aqueous NH_4Cl (1 mL) solution were added. Then, the mixture was extracted with ethyl acetate (3 × 10 mL) and the organic extract was dried over anhydrous Na_2SO_4 followed by the removal of solvent in a rotary evaporator. The residue was purified by flash chromatography (petroleum ether/ethyl acetate, v:v = 1:1) to afford product **10a** (18.1 mg, 72% yield, 96% ee, >20:1 dr).

2. Typical procedure of the synthesis of compounds **10b**

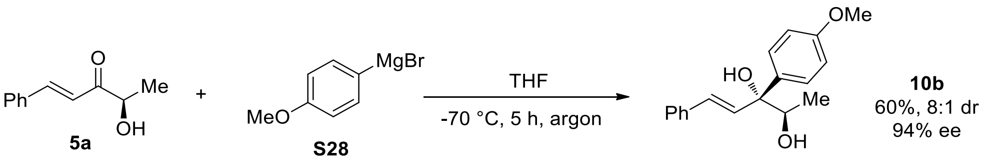

A mixture of **5a** (25.0 mg, 0.14 mmol) in THF (1.0 mL) was added to (4-methoxyphenyl)magnesium bromide **S28** (0.42 mL, 1M in THF) and the mixture was stirred at –70 ºC under argon atmosphere. After completion of the reaction as

indicated by TLC, saturated aqueous NH_4Cl (2 mL) solution were added. Then, the mixture was extracted with ethyl acetate (3 × 10 mL) and the organic extract was dried over anhydrous Na_2SO_4 followed by the removal of solvent in a rotary evaporator. The residue was purified by flash chromatography (petroleum ether/ethyl acetate, v:v = 5:1) to afford product **10b** (23.9 mg, 60% yield, 94% ee, 8:1 dr).

3. Typical procedure of the synthesis of compounds **10c**:

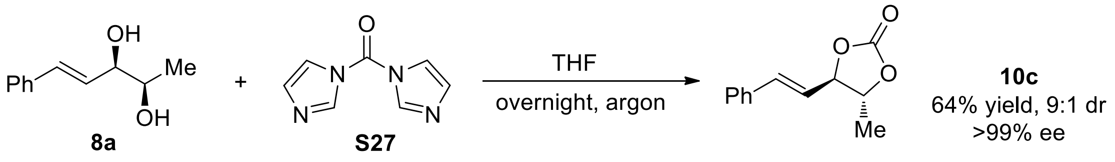

A mixture of **8a** (11.0 mg, 0.062 mmol, 9:1 dr) in THF (0.5 mL) was added to di(1*H*-imidazol-1-yl)methanone **S27** (15.1 mg, 0.093 mmol) and the mixture was stirred at room temperature under argon atmosphere. After completion of the reaction as indicated by TLC, the mixture was concentrated under reduced pressure and purified by flash chromatography (petroleum ether/ethyl acetate, v:v = 8:1) to afford product **10c** (9.0 mg, 64% yield, >99% ee, 9:1 dr).

4. Total synthesis of natural products

4.1 Substrates **15** were synthesized according to the following procedure.

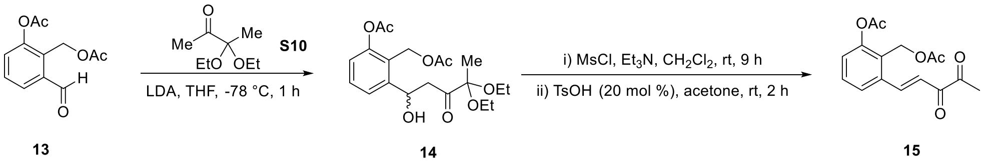

13 is known compound and was prepared according to the literature reports[5].

A solution of LDA was prepared by adding *n*-BuLi (8.8 mL, 22 mmol) dropwise to a cooled (–78 °C) solution of ^{i}Pr$_2$NH (3.2 mL, 23 mmol) in THF (25 mL). The reaction was stirred at –78 °C for 30 min. Then, HMPA (4 mL, 22 mmol) was added dropwise via a syringe. The reaction was stirred at –78 ºC for 2 h. A mixture of ketone **S10** (3.5 g, 20 mmol) in THF (10 mL) was added dropwise to the LDA solution. The reaction mixture was stirred at –78 °C for 2 h. Then, the aldehyde **13** (2.4 g, 10 mmol) was added dropwise. The reaction was stirred for additional 30 min at –78 ºC. The reaction was quenched with saturated aqueous NH_4Cl (30 mL) at –78 ºC and warmed to room temperature. The residue was extracted with ethyl acetate (100 mL) and saturated brine (50 mL). The combined organic phase was dried with anhydrous Na_2SO_4 and concentrated under reduced pressure. The residue was purified by chromatography (petroleum ether/ethyl acetate, v:v = 1:1) to afford product **14** (2.9

g, 73% yield).

To a solution of **14** (2.8 g, 7 mmol) and Et_3N (2.1 mL, 15 mmol) in CH_2Cl_2 (16 mL) was added MsCl (0.7 mL, 6.1 mmol) dropwise at 0 °C. The resulting mixture was warmed to 25 ºC and stirred for 16 h. After the end of the reaction, the reaction mixture was treated with saturated aqueous solution of $NaHCO_3$. The residue was extracted with CH_2Cl_2 (3 × 50 mL) and saturated brine (3 × 50 mL). The combined organic phase was dried with anhydrous Na_2SO_4 and concentrated under reduced pressure. The residue was purified by chromatography (petroleum ether/ethyl acetate, v:v = 3:1) to afford product (2.0 g, 76% yield).

Typically, in a dry 5 mL round bottom flask was added a mixture of product (1.8, 6 mmol) and *p*-TSA·H_2O (118.0 mg, 1.2 mmol, 20 mol%) in acetone (25 mL) at room temperature. The mixture was stirred at room temperature for 4 h. The solvent was evaporated under reduced pressure, the residue was extracted with CH_2Cl_2 (3 × 30 mL) and saturated brine (3 × 20 mL). The combined organic phase was dried with an hydrous Na_2SO_4 and concentrated under reduced pressure. The residue was purified by chromatography (petroleum ether/ethyl acetate, v:v = 3:1) to afford product **15** (1.73 g, 95% yield).

4.2 Formal synthesis of *anti*-sordariol

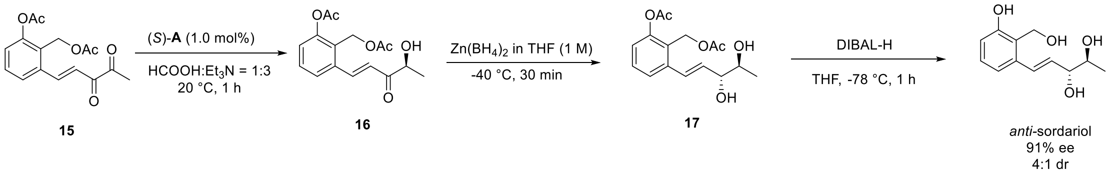

A mixture of unsaturated 1,2-diketone **15** (120 mg, 0.4 mmol) and the Ru catalyst **A** (2.6 mg, 1 mol%) in 1.0 mL formic acid/triethylamine (v:v = 1:3) mixture was stirred at room temperature under 20 °C. The mixture was stirred for 1 h. The reaction mixture was extracted with CH_2Cl_2 (3 × 40 mL) and the organic extract was dried over anhydrous Na_2SO_4 followed by the removal of solvent in a rotary evaporator. The residue was purified by chromatography (petroleum ether/ethyl acetate, v:v = 1:1) to afford product **16** (94.2 mg, 77% yield).

A mixture of **16** (94.2 mg, 0.3 mmol) was added to $Zn(BH_4)_2$ (1.5 mL, 1M in THF) was stirred at –40 ºC under argon atmosphere. The mixture was stirred for 30 min. The reaction mixture was extracted with CH_2Cl_2 (3 × 30 mL) and the organic extract was dried over anhydrous Na_2SO_4 followed by the removal of solvent in a rotary evaporator. The residue was purified by flash chromatography (petroleum ether/ethyl acetate, v:v = 1:3) to afford product **17** (75.9 mg, 80% yield, 4:1 dr).

To a solution of **17** (61.6 mg, 0.2 mmol) in dry THF (3 mL) at –78 ºC was added an excess amount of DIBAL-H (1.0 M solution in toluene, 2.0 mL, 2 mmol) dropwise. The reaction was slowly brought to room temperature and stirred until the completion of the starting material. After completion, saturated aqueous $Na_2S_2O_3$ (10 mL) solution were added. The reaction mixture was extracted with EtOAc, and the organic layer was washed with brine solution, dried over Na_2SO_4, evaporated under reduced pressure. The residue was purified by flash chromatography (CH_3OH/CH_2Cl_2, v:v = 1:8) to afford product *anti*-sordariol (42.7 mg, 94% yield, 4:1 dr, 91% ee).

Table S5-1. NMR data comparison of literature and synthetic natural product (*anti*-sordariol)[6]

**^{1}H NMR (CD_3OD)**		**^{13}C NMR (CD_3OD)**	
**literature**	**synthetic**	**literature**	**synthetic**
7.07 (t, *J* = 8.0 Hz, 1H)	7.07 (t, *J* = 7.8, 1H)	157.3	157.3
7.02 (brd, *J* = 8.1 Hz, 1H)	7.03 (brd, *J* = 8.0 Hz, 1H)	139.6	139.7
7.0 (brd, *J* = 15.0 Hz, 1H)	6.99 (brd, *J* = 9.4 Hz, 1H)	132.5	132.5
6.72 (dd, *J* = 8.0, 1.2 Hz)	6.72 (d, *J* = 7.8 Hz, 1H)	130.6	130.6
6.17 (dd, *J* = 15.7, 6.8 Hz, 1H)	6.18 (dd, *J* = 15.8, 6.8 Hz, 1H)	129.7	129.7
4.78 (s, 2H)	4.78 (s, 2H)	125.2	125.2
4.07 (ddd, *J* = 6.5, 5.0, 1.2 Hz, 1H)	4.07 (ddd, *J* = 6.2, 5.6, 0.6 Hz, 1H)	118.9	118.6
		115.3	115.4
3.77 (dq, *J* = 6.4, 6.5 Hz, 1H)	3.77 (dq, *J* = 11.4, 6.1Hz, 1H)	78.0	78.0
1.20 (d, *J* = 6.4 Hz, 3H)	1.20 (d, *J* = 6.4 Hz, 3H)	71.7	71.7
		56.5	56.5
		18.9	18.9

Table S5-2. ^{1}H NMR spectra comparison of literature and synthetic natural product (*anti*-sordariol).[11]

literature:

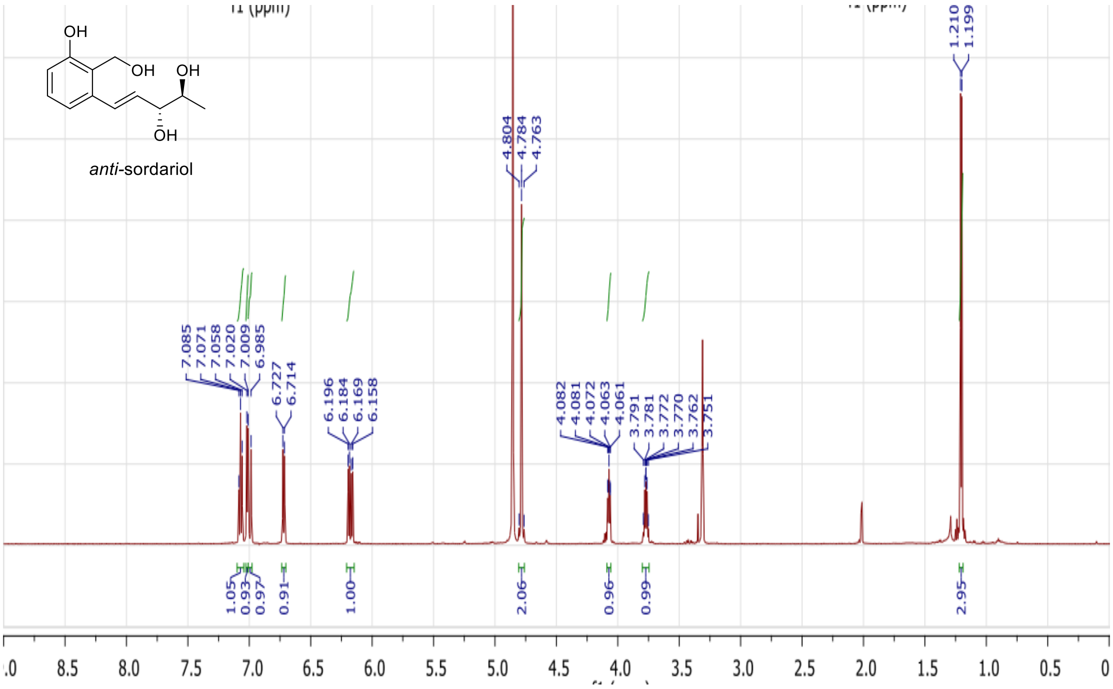

synthetic:

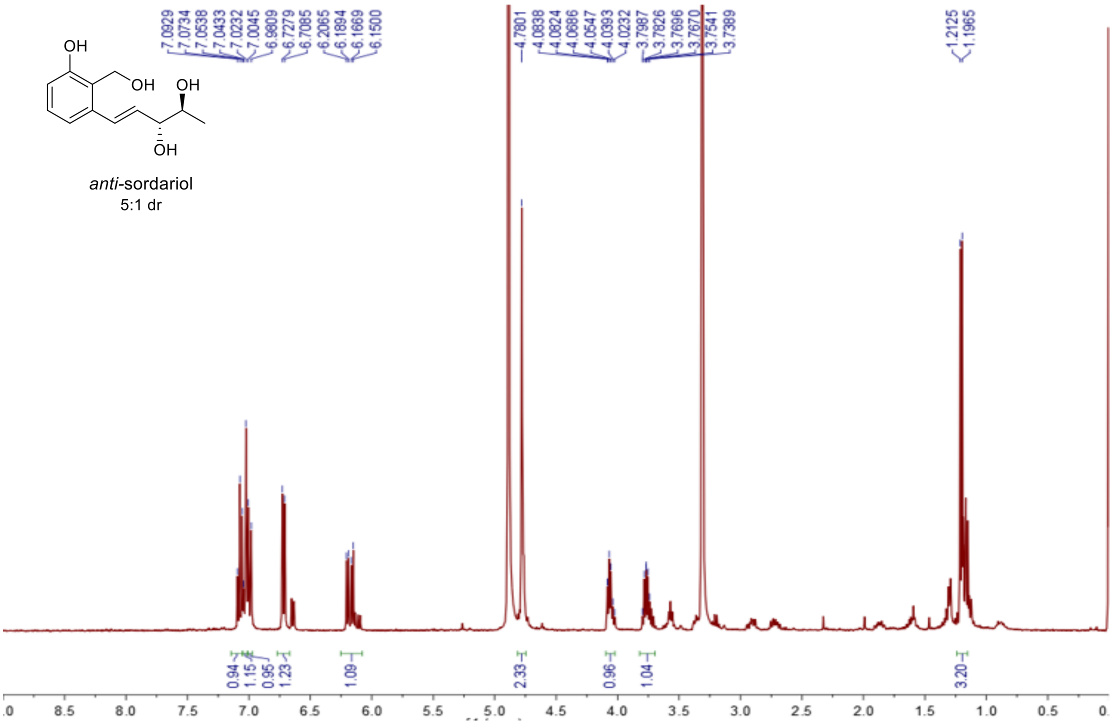

Table S5-3. ^{13}C NMR spectra comparison of literature and synthetic natural product (*anti*-sordariol).[11]

literature:

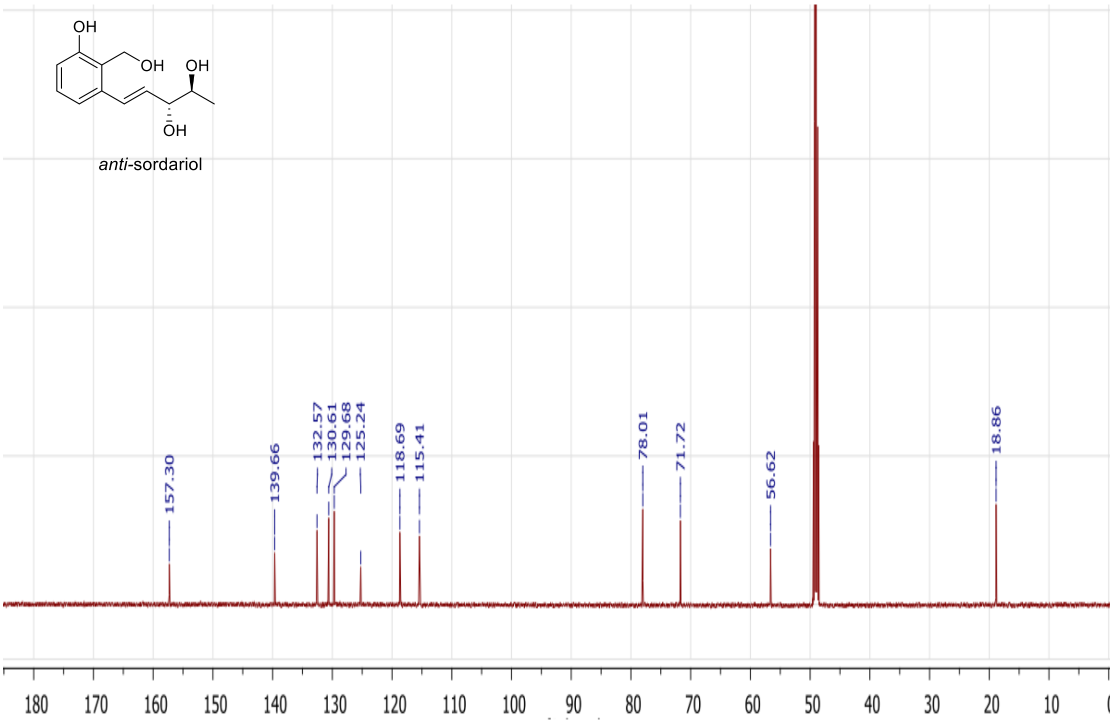

synthetic:

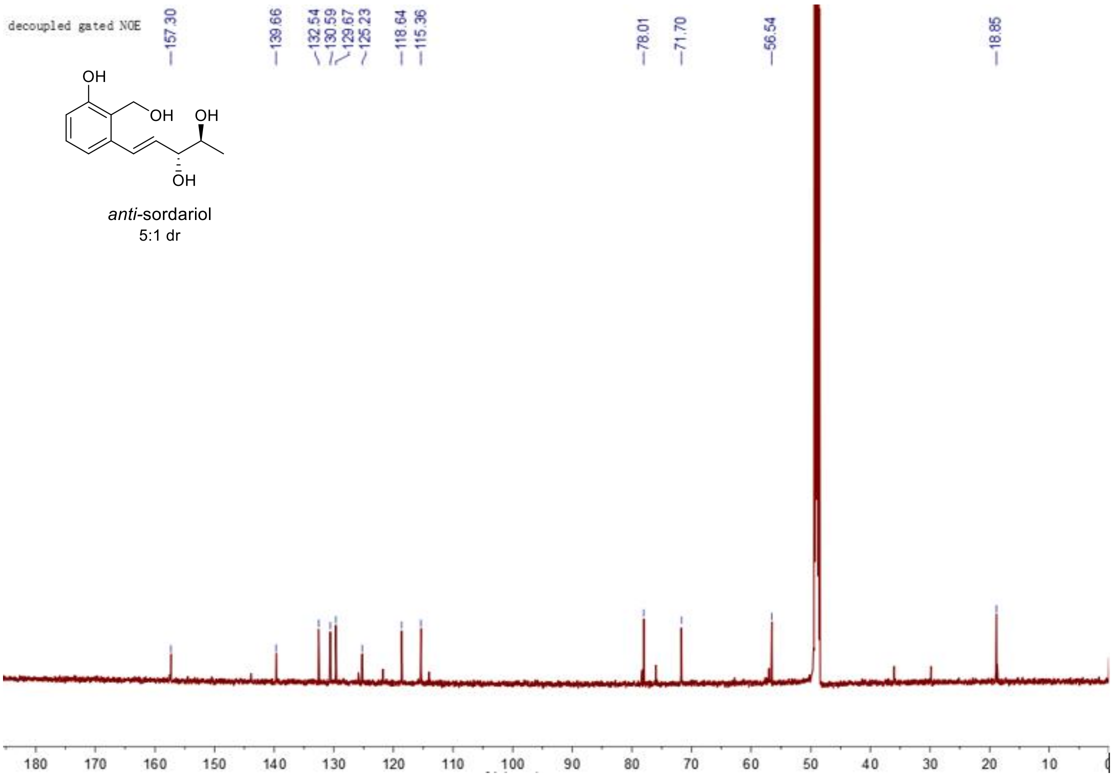

4.3 Formal synthesis of *anti*-sordarial

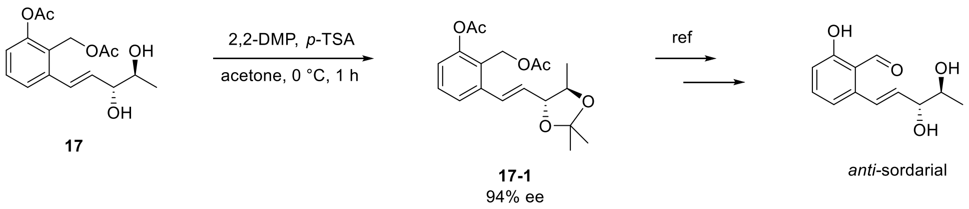

To a solution of **17** (60 mg, 0.2 mmol) dissolved in acetone (2 mL) at 0 °C. 2,2-dimethoxypropane (0.12 mL, 1 mmol) and *p*-TSA (3.4 mg, 0.02 mmol) were then added and the mixture was allowed to slowly warm to room temperature over 1 h. The reaction was quenched with aqueous $NaHCO_3$ (20 mL) and extracted with CH_2Cl_2 (2 × 15 mL). The combined organic extracts were dried over $MgSO_4$, filtered, and concentrated in vacuo. The residue was purified by flash chromatography (petroleum ether/ethyl acetate, v:v = 4:1) to afford product **17-1** (27.1 mg, 39% yield, 5:1 dr, 94% ee).

4.4 Formal synthesis of 12-methoxy sordariol

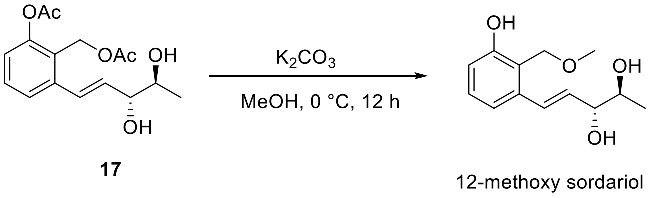

To a solution of **17** (30 mg, 0.1 mmol) in MeOH (1.5 mL) at 0 ºC was added K_2CO_3 (157.9 mg, 1.1 mmol) and stirred at room temperature for 12 h. After completion of the starting material (monitored by TLC), MeOH was evaporated and quenched the reaction with water at 0 °C. The aqueous layer was extracted with ethyl acetate, and the organic layer was washed with brine, dried over Na_2SO_4, and concentrated under reduced pressure. The residue was purified by flash chromatography (petroleum ether/ethyl acetate, v:v = 4:1) to afford product 12-methoxy sordariol (12.1 mg, 51% yield, 8:1 dr, 98% ee).

Table S6-1. NMR data comparison of literature and synthetic natural product (12-methoxy sordariol)[7]

^{1}H NMR (CD_3OD)		^{13}C NMR (CD_3OD)	
**literature**	**synthetic**	**literature**	**synthetic**
7.10 (t, *J* = 7.9 Hz, 1H)	7.10 (t, *J* = 7.8 Hz, 1H)	157.7	157.7
7.03 (d, *J* = 7.1 Hz, 1H)	7.03 (d, *J* = 7.8 Hz, 1H)	140.7	140.7
6.94 (d, *J* = 15.7 Hz, 1H)	6.94 (d, *J* = 15.8 Hz, 1H)	132.8	132.7
6.72 (dd, *J* = 7.9, 1.0 Hz, 1H)	6.72 (d, *J* = 8.0 Hz, 1H)	130.7	130.6
6.19 (dd, *J* = 15.8, 6.8 Hz, 1H)	6.19 (dd, *J* = 15.8, 6.8 Hz, 1H)	130.3	130.3
4.62 (s, 2H)	4.62 (s, 2H)	122.6	122.5
4.07 (ddd, *J* = 6.8, 4.9, 1.4 Hz, 1H)	4.06 (ddd, *J* = 5.7, 5.0, 0.7 Hz, 1H)	118.8	118.7
		115.5	115.3
3.77 (dq, *J* = 4.9, 6.4 Hz, 1H)	3.76 (dq, *J* = 5.0, 6.4 Hz, 1H)	78.1	78.1
3.37 (s, 3H)	3.37 (s, 3H)	71.9	71.8
1.20, (d, *J* = 6.4 Hz, 3H)	1.20 (d, *J* = 6.4 Hz, 3H)	66.5	66.3
		58.1	58.1
		18.9	18.9

Table S6-2. ^{1}H NMR spectra comparison of literature and synthetic natural product (12-methoxy sordariol).[7]

literature:

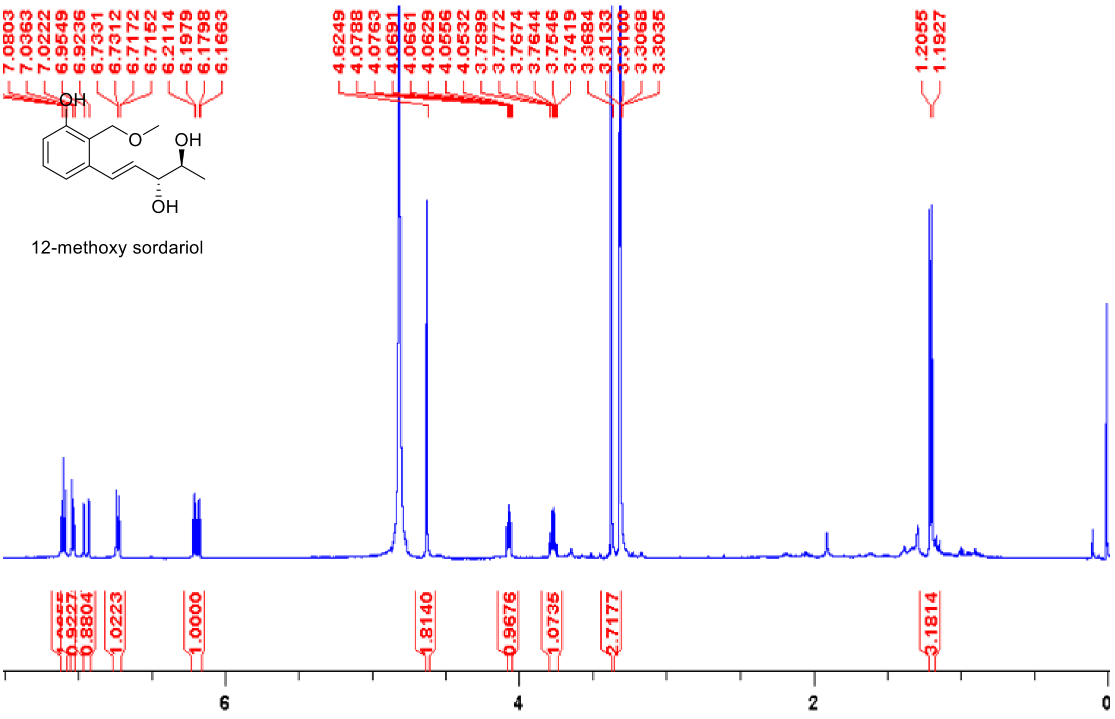

synthetic:

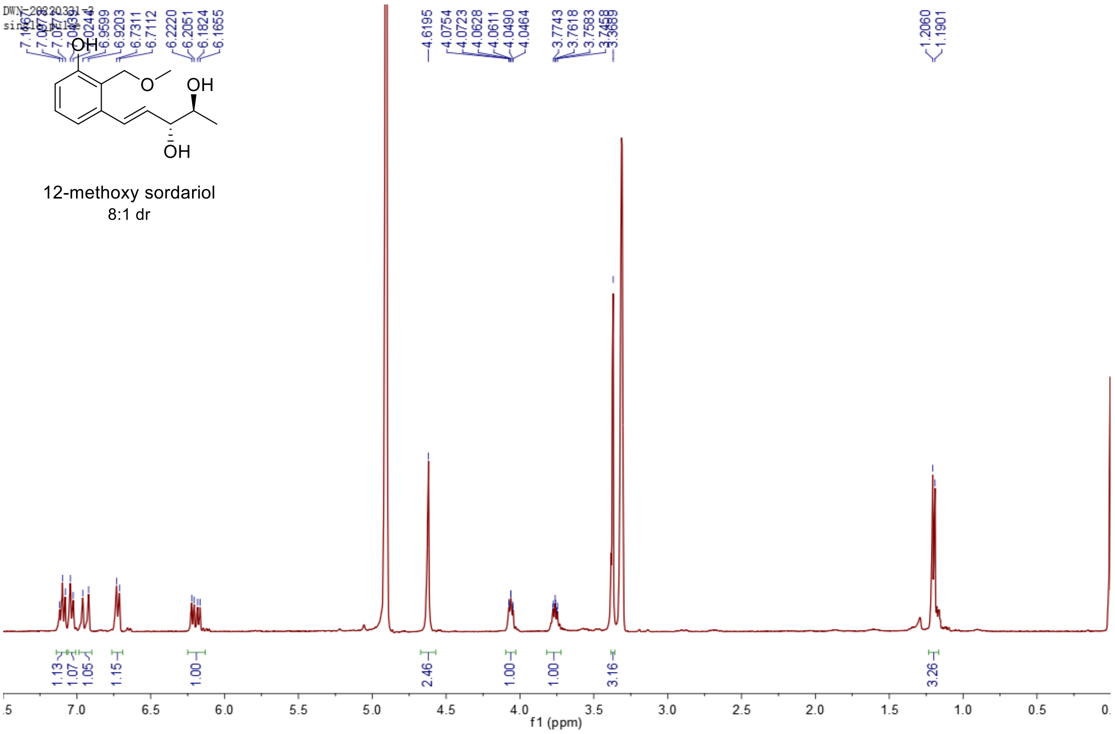

Table S6-3. ^{13}C NMR spectra comparison of literature and synthetic natural product (12-methoxy sordariol).[7]

literature:

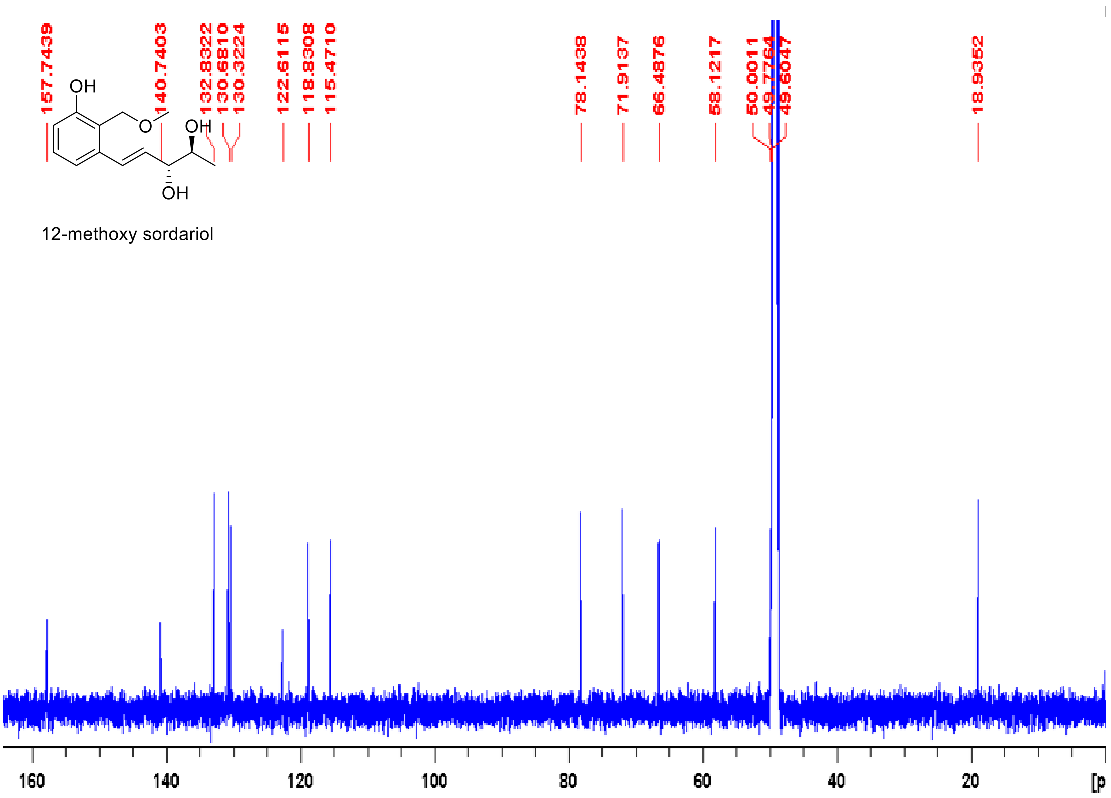

synthetic:

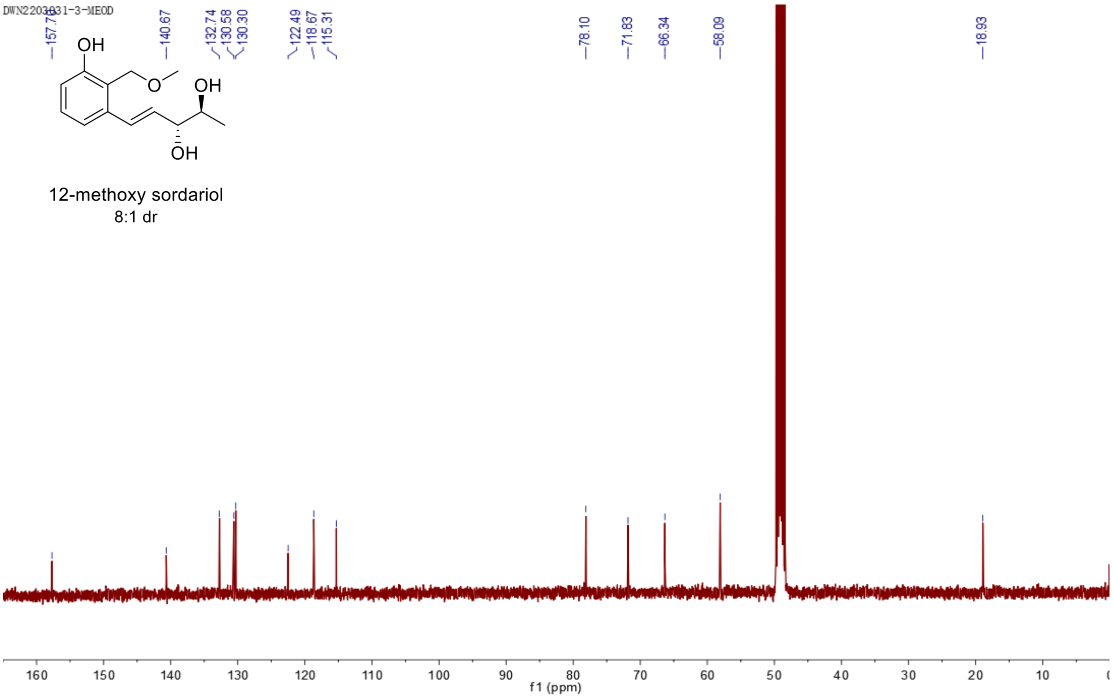

4.5 Formal synthesis of agropyrenol

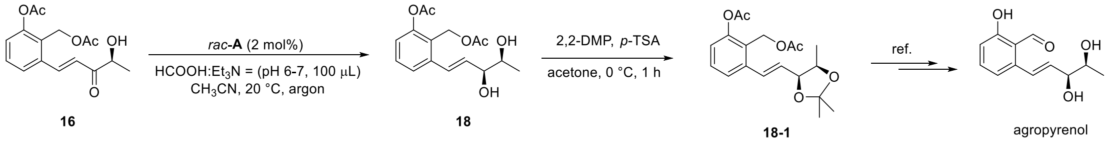

To a mixture of **16** (61 mg, 0.2 mmol) and the racemic catalyst **A** (2.6 mg, 2 mol%) in CH_3CN (0.15 M), 100.0 μL formic acid/triethylamine (v:v = 1:3) mixture was added and the mixture was stirred at 20 °C under argon atmosphere. After completion of the reaction as indicated by TLC, it was extracted with CH_2Cl_2 (3 × 20 mL) and the organic extract was dried over anhydrous Na_2SO_4 followed by the removal of solvent in a rotary evaporator. The residue was purified by flash chromatography (petroleum ether/ethyl acetate, v:v = 1:1) to afford product **18** (31 mg, 51% yield).

To a solution of **18** (30 mg, 0.1 mmol) dissolved in acetone (1 mL) at 0 °C, 2,2-dimethoxypropane (0.06 mL, 0.5 mmol) and *p*-TSA (1.7 mg, 0.01 mmol) were then added and the mixture was allowed to slowly warm to room temperature over 1 h. The reaction was quenched with aqueous $NaHCO_3$ (10 mL) and extracted with CH_2Cl_2 (2 × 5 mL). The combined organic extracts were dried over $MgSO_4$, filtered, and concentrated in vacuo. The residue was purified by flash chromatography (petroleum ether/ethyl acetate, v:v = 4:1) to afford product **18-1** (59.16 mg, 84% yield, 5:1 dr, 91% ee).

VII. Determination of the absolute configurations

VII-1. The products in Scheme 2.

The absolute configurations of **2t** and **2s** were determined by their single crystals and **2a-2s** were identified by analogy.

VII-2. The products in Scheme 3a.

The absolute configuration of **5b** was determined by its Mosher ester analysis, and the absolute configurations of **5a−5l** were identified by analogy.

Procedure: to a solution of **5b** (26 mg, 0.13 mmol) and *R*-MTPA-OH acid (94 mg, 0.4 mmol) in CH_2Cl_2 (2 mL) at room temperature. DCC (84 mg, 0.4 mmol) and 4-dimethylaminopyridine (50 mg, 0.4 mmol) were then added and the mixture stirred at room temperature for 24 h. After completion of the reaction as indicated by TLC, the residue was purified by chromatography (petroleum ether/ethyl acetate, v:v =10:1) to afford Mosher's ester **S29**.

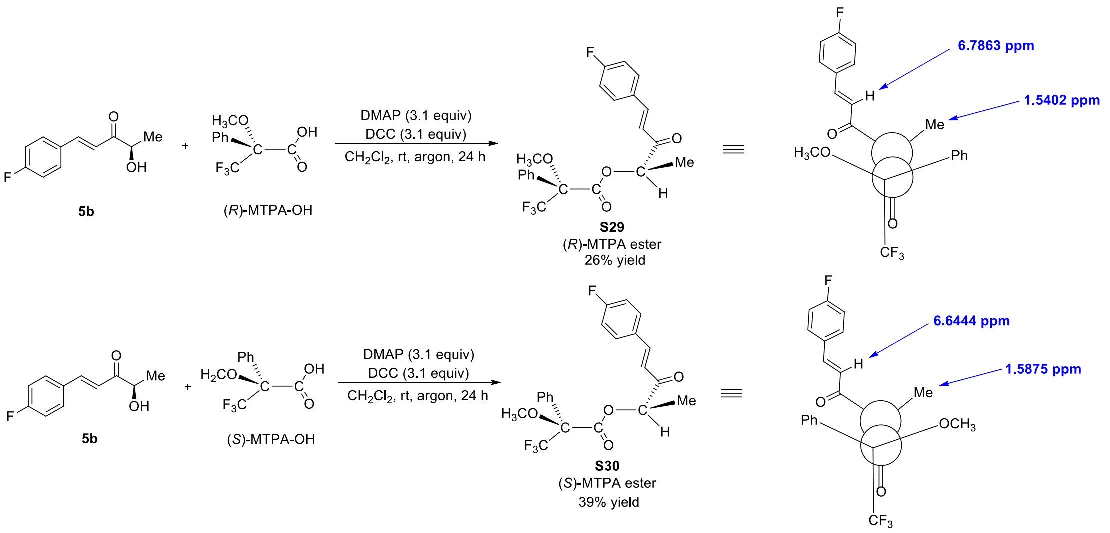

VII-3. The products of 6a−6f in Scheme 3b.

The absolute configuration determination of **6a** was determined by the following method through the HPLC spectrum comparison with the compound obtained using known method. Then **6b**−**6f** were identified by analogy.

Racemic **6a** was protected by MOMCl to afford **S31**, and after $Zn(BH_4)_2$ reduction (*anti*-selective) *rac*-**S32** was produced.[8] Then *rac*-**S32** was resolved using (*S*)-BTM to deliver *opt*-**S32**. The resolution mode has been well-established in literature report.[9] The HPLC spectrum is shown below.

kinetic resolution method to produce *opt*-S32:

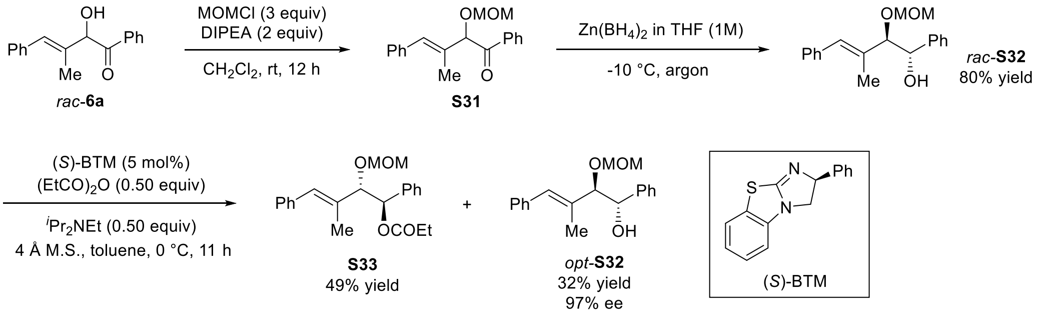

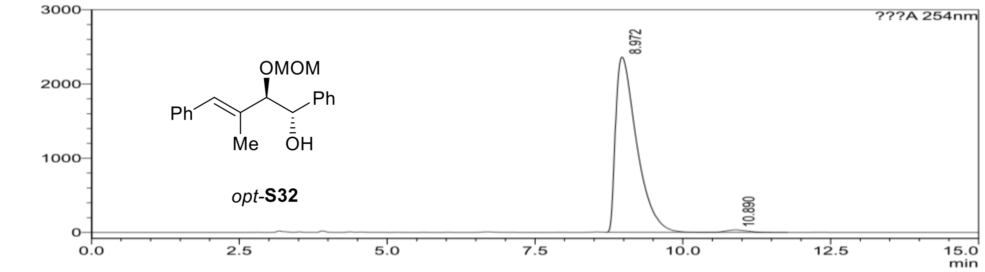

<Peak Table>

???A 254nm

Peak#	Ret. Time	Area	Height	Conc.	Unit	Mark	Name
1	8.972	58518883	2359736	98.567			
2	10.890	850694	33001	1.433		V	
Total		59369577	2392736				

In another method, the enantioenriched **6a** was first obtained via the standard conditions in Scheme 3b using asymmetric transfer hydrogenation, and then protected by MOMCl and then reduced by $Zn(BH_4)_2$ to generate *opt*-**S32**. Through the HPLC spectrum comparison, it can be concluded that **6a** has a (*R*)-configuration.

transfer hydrogenation method to produce *opt*-S32:

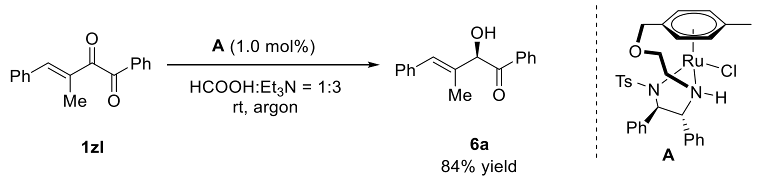

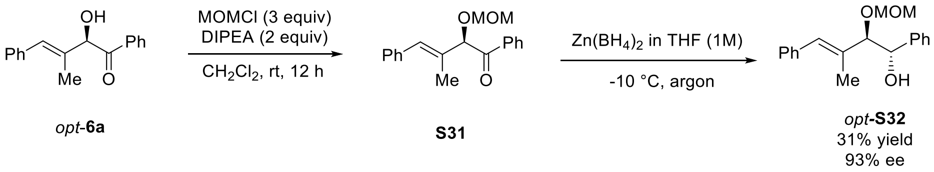

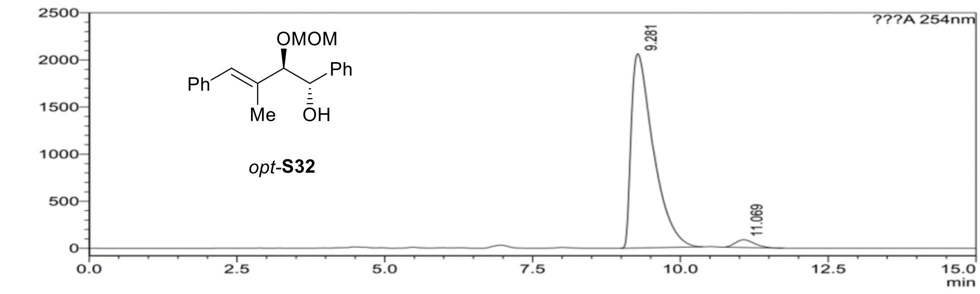

<Peak Table>

???A 254nm

Peak#	Ret. Time	Area	Height	Conc.	Unit	Mark	Name
1	9.281	53897610	2062153	96.650			
2	11.069	1867924	82318	3.350			
Total		55765534	2144471				

VII-4. The products of 6g−6j in Scheme 3b.

The absolute configuration of **6g** was determined via Mosher ester analysis, and **6h−6j** were identified by analogy.

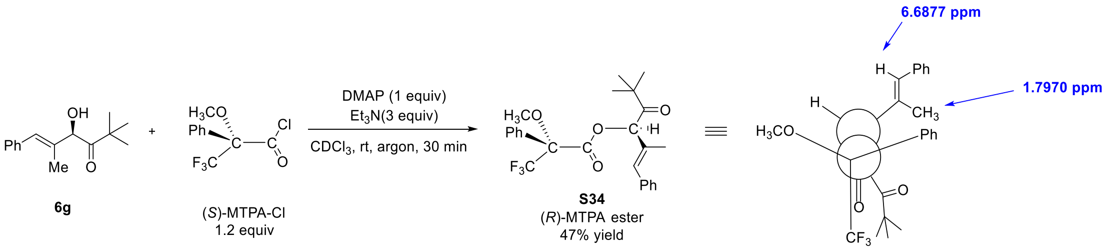

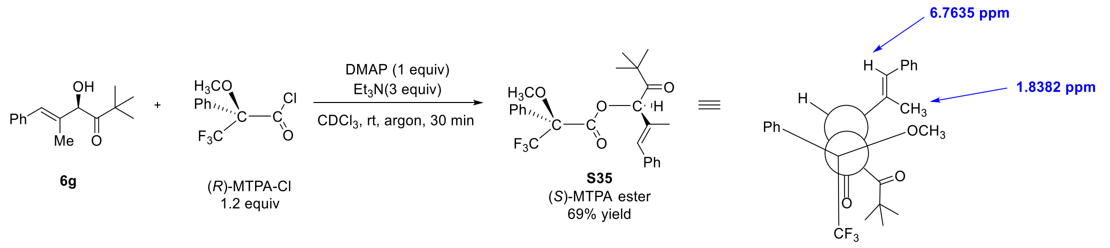

Oxalyl chloride (50 μL, 0.57 mmol) was added to a solution of (*R*)-MTPA (28 mg, 0.12 mmol) and DMF (9 mg, 0.12 mmol) in hexane (5 mL) at room temperature. A white precipitate formed immediately. After 1 h the mixture was filtered and concentrated. A solution of **6g** (23 mg, 0.1 mmol), Et_3N (40 μL, 0.3 mmol) and DMAP (10 mg) in $CDCl_3$ (1 mL) was added to the residue. After completion of the reaction as indicated by TLC, the residue was purified by chromatography (petroleum ether/ethyl acetate, v:v = 5:1) to afford Mosher's ester **S34**.

VII-5. The products of 7a−7j in Scheme 4.

They were identified by the HPLC spectrum comparison with known literature report.[12]

VII-6. The products in Scheme 5.

The absolute configurations of **4c** and **4g** were confirmed by single crystal X-ray analysis, and **4a**, **4b**, **4d**−**4f** were identified by analogy. The relative configuration of **3a** was identified by the NMR spectrum comparison with that of **4a**. The absolute configuration of **3a** was deduced by the following method, and **3b**−**3f** were identified by analogy.

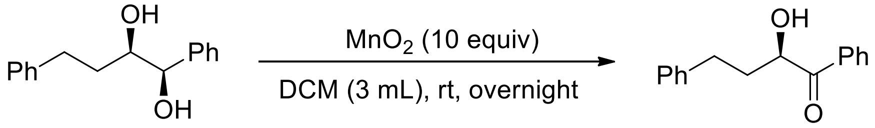

3a

2a, 70% yield
74% ee

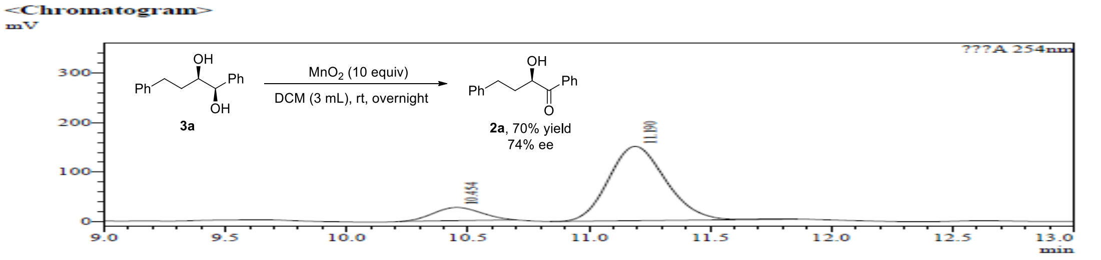

<Peak Table>
???A 254nm

Peak#	Ret. Time	Area	Height	Conc.	Unit	Mark	Name
1	10.454	366938	26695	12.953		M	
2	11.190	2465855	150115	87.047		M	
Total		2832793	176810				

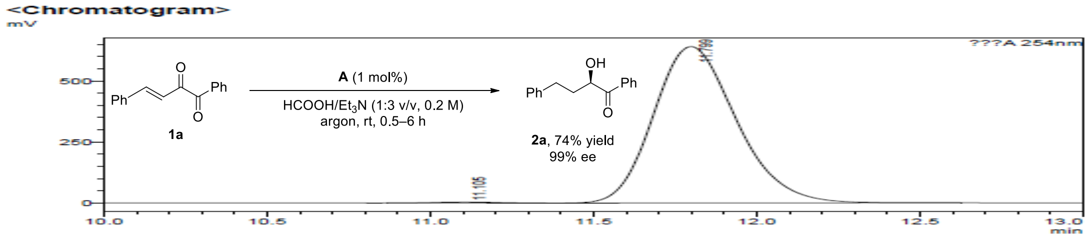

<Peak Table>
???A 254nm

Peak#	Ret. Time	Area	Height	Conc.	Unit	Mark	Name
1	11.105	47941	3376	0.422		M	
2	11.799	11303085	640195	99.578		M	
Total		11351027	643571				

VII-7. The products in Scheme 6.

The $Zn(BH_4)_2$ reduction of *α*-hydroxyketones has been well-established to proceed through a chelation-controlled addition model, and the major products are *anti*-1,2-diols:

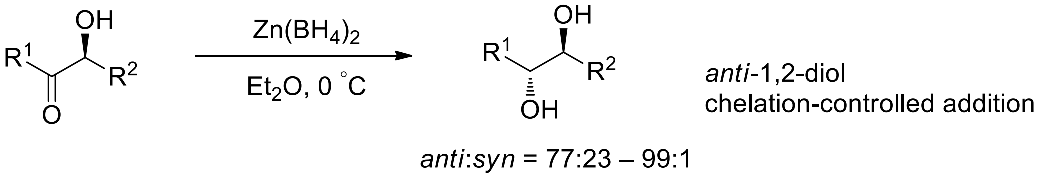

key reference: Nakata, T.; Tanaka, T.; Oishi, T. *Tetrahedron Lett.* **1983**, *24*, 2653–2661.

Therefore, products **9a–9h** have an *anti*-configuration, and the absolute configuration of **9a–9h** can be deduced from the enantiopure starting material **5**.

Then the *syn*-structure of **8a** can be deduced through the comparison of its ^{1}H NMR spectra with that of **9a**. And since products **8** were obtained through hydrogenation of optically pure **5** using racemic **A** catalyst, the absolute configuration of **8** can be deduced.

VII-8. The absolute configuration of 2ab.

The absolute configuration of **2ab** was determined via Mosher ester analysis.

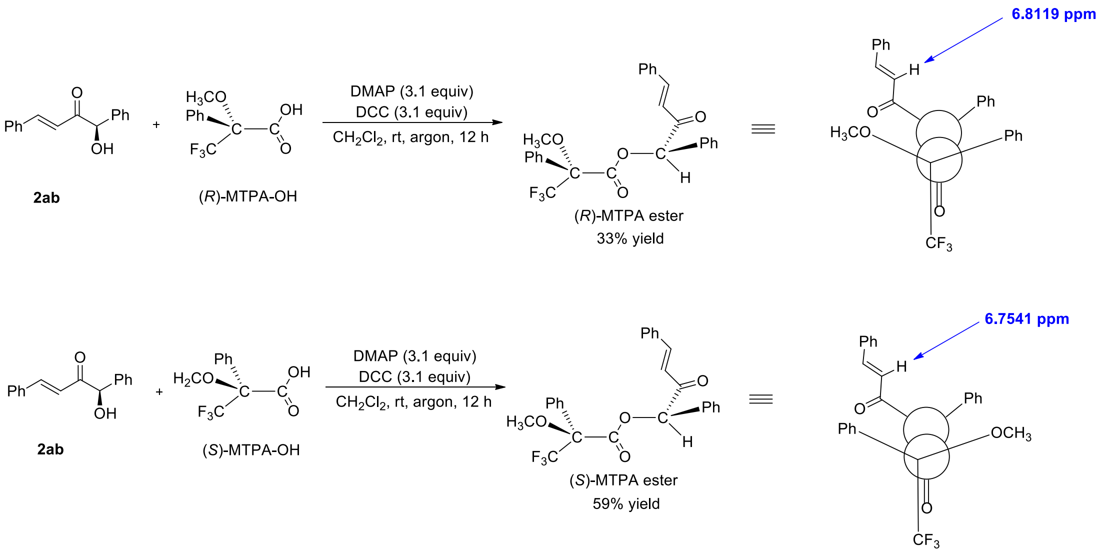

Procedure: to a solution of **1a** (31 mg, 0.13 mmol) and *S*-MTPA-OH acid (94 mg, 0.4 mmol) in CH_2Cl_2 (2 mL) at room temperature. DCC (84 mg, 0.4 mmol) and 4-dimethylaminopyridine (50 mg, 0.4 mmol) were then added and the mixture stirred at room temperature for 12 h. After completion of the reaction as indicated by TLC, the residue was purified by chromatography (petroleum ether/ethyl acetate, v:v =20:1) to afford Mosher's ester.

VIII. Characterizations of new compounds

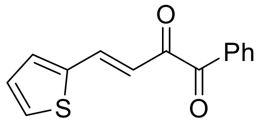

1f

(*E*)-1-Phenyl-4-(thiophen-2-yl)but-3-ene-1,2-dione (1f): Purified by chromatography on silica gel (petroleum ether/ethyl acetate, v:v = 7:1), yellow solid, mp 67–61 ºC, 460 mg, 19% yield. ^{1}H NMR (600 MHz, $CDCl_3$) δ 8.02–8.00 (m, 2H), 7.82 (d, *J* = 16.0 Hz, 1H), 7.65–7.62 (m, 1H), 7.51–7.48 (m, 3H), 7.37–7.35 (m, 1H), 7.08 (dd, *J* = 5.0, 3.7 Hz, 1H), 6.91 (d, *J* = 16.0 Hz, 1H); ^{13}C NMR (151 MHz, $CDCl_3$) δ 193.2, 192.2, 141.0, 139.7, 134.8, 133.5, 132.8, 131.0, 130.3, 129.0, 128.8, 121.1. HRMS (ESI-Quadrupole-Orbitrap) m/z: $[M + H]^+$ Calcd for $C_{14}H_{11}O_2S$ 243.0474, found 243.0473. IR (KBr thin film, cm^{-1}): ν 3080, 3046, 3035, 3026, 1724, 1677, 1604, 1550, 1497, 1442, 913.

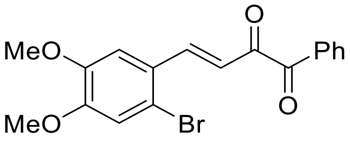

1g

(*E*)-4-(2-Bromo-4,5-dimethoxyphenyl)-1-phenylbut-3-ene-1,2-dione(1g): Purified by chromatography on silica gel (petroleum ether/ethyl acetate, v:v = 5:1), green solid, mp 187–189 ºC, 636 mg, 17% yield. ^{1}H NMR (600 MHz, $CDCl_3$) δ 8.07–8.02 (m, 3H), 7.64–7.61 (m, 1H), 7.51–7.48 (m, 2H), 7.16 (s, 1H), 7.04 (s, 1H), 7.00 (d, *J* = 16.1 Hz, 1H), 3.90 (s, 3H), 3.89 (s, 3H); ^{13}C NMR (151 MHz, $CDCl_3$) δ 193.3, 192.0, 152.5, 148.8, 146.8, 134.8, 132.8, 130.3, 129.0, 126.0, 122.2, 119.2, 115.8, 109.3, 56.4, 56.2. HRMS (ESI-Quadrupole-Orbitrap) m/z: $[M + H]^+$ Calcd for $C_{18}H_{16}O_4Br$ 375.0226, found 375.0225. IR (KBr thin film, cm^{-1}): ν 3081, 3029, 2967, 1736, 1685, 1594, 1583, 1510, 1452, 1030, 866.

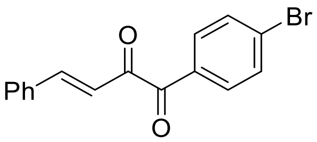

1h

(*E*)-1-(4-Bromophenyl)-4-phenylbut-3-ene-1,2-dione (1h): Purified by chromatography on silica gel (petroleum ether/ethyl acetate, v:v = 30:1), yellow solid, mp 117–120 ºC, 1225 mg, 39% yield. ^{1}H NMR (600 MHz, $CDCl_3$) δ 7.91 (d, *J* = 8.8 Hz, 2H), 7.72 (d, *J* = 16.3 Hz, 1H), 7.65 (d, *J* = 8.7 Hz, 2H), 7.60–7.58 (m, 2H), 7.46–7.39 (m, 3H), 7.16 (d, *J* = 16.3 Hz, 1H); ^{13}C NMR (151 MHz, $CDCl_3$) δ 191.9, 191.8,

149.2, 134.0, 132.4, 131.8, 131.7, 131.6, 130.4, 129.2, 129.0, 122.0. HRMS (ESI-Quadrupole-Orbitrap) m/z: [M + H]$^+$ Calcd for $C_{16}H_{12}O_2Br$ 315.0015, found 315.0014. IR (KBr thin film, cm^{-1}): ν 3092, 3038, 2936, 1709, 1655, 1647, 1589, 1570, 926, 860, 564.

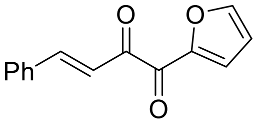

1j

(*E*)-1-(Furan-2-yl)-4-phenylbut-3-ene-1,2-dione (1j): Purified by chromatography on silica gel (petroleum ether/ethyl acetate, v:v = 15:1), yellow solid, mp 250–252 ºC, 927 mg, 82% yield. ^{1}H NMR (400 MHz, $CDCl_3$) δ 7.86 (d, *J* = 16.2 Hz, 1H), 7.77 (s, 1H), 7.70 (d, *J* = 3.6 Hz, 1H), 7.65–7.63 (m, 2H), 7.46–7.42 (m, 4H), 6.63 (dd, *J* = 3.5, 1.4 Hz, 1H); ^{13}C NMR (101 MHz, $CDCl_3$) δ 188.2, 177.9, 149.5, 149.2, 148.2, 134.2, 131.6, 129.1, 129.0, 124.6, 120.1, 113.0. HRMS (ESI-Quadrupole-Orbitrap) m/z: [M + H]$^+$ Calcd for $C_{14}H_{11}O_3$ 227.0703, found 227.0701. IR (KBr thin film, cm^{-1}): ν 3079, 3025, 2942, 1797, 1670, 1640, 1570, 1506, 1467, 1022, 859

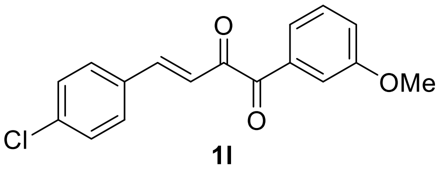

1l

(*E*)-1-(Furan-2-yl)-4-phenylbut-3-ene-1,2-dione (1l): Purified by chromatography on silica gel (petroleum ether/ethyl acetate, v:v = 20:1), yellow solid, mp 131–133 ºC, 990 mg, 33% yield. ^{1}H NMR (600 MHz, $CDCl_3$) δ 7.63 (d, *J* =16.3 Hz, 1H), 7.58–7.51 (m, 4H), 7.42–7.37 (m, 3H), 7.21–7.18 (m, 1H), 7.08 (d, *J* =16.3 Hz, 1H), 3.86 (s, 3H); ^{13}C NMR (151 MHz, $CDCl_3$) δ 193.0, 192.4, 160.1, 147.3, 137.7, 134.0, 132.6, 130.1, 129.5, 123.5, 122.8, 121.9, 113.4, 55.6. HRMS (ESI-Quadrupole-Orbitrap) m/z: [M + H]$^+$ Calcd for $C_{17}H_{14}O_3Cl$ 301.0626, found 301.0625. IR (KBr thin film, cm^{-1}): ν 3073, 2992, 1737, 1723, 1613, 1600, 1571, 1476, 1360, 923, 788.

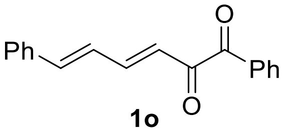

1o

(*3E,5E*)-1,6-Diphenylhexa-3,5-diene-1,2-dione (1o): Purified by chromatography on silica gel (petroleum ether/ethyl acetate, v:v = 20:1), yellow oil, 572 mg, 22.0% yield. ^{1}H NMR (600 MHz, $CDCl_3$) δ 8.02–7.99 (m, 2H), 7.65–7.62 (m, 1H), 7.52–7.44 (m, 5H), 7.38–7.32 (m, 3H), 7.03–6.95 (m, 2H), 6.64 (d, *J* = 15.7 Hz, 1H); ^{13}C NMR (151 MHz, $CDCl_3$) δ 193.5, 193.1, 149.0, 144.2, 135.7, 134.7, 132.9, 130.3, 130.0, 129.04,

129.96, 127.7, 126.6, 125.8. HRMS (ESI-Quadrupole-Orbitrap) m/z: [M + H]$^+$ Calcd for $C_{18}H_{15}O_2$ 263.1067, found 263.1063. IR (KBr thin film, cm^{-1}): ν 3098, 3045, 3025, 1740, 1712, 1697, 1613, 1514, 1436, 874, 851.

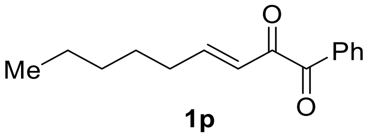

1p

(*E*)-1-Phenylnon-3-ene-1,2-dione (1p): Purified by chromatography on silica gel (petroleum ether/ethyl acetate, v:v = 30:1), yellow oil, 377.1 mg, 41% yield. ^{1}H NMR (600 MHz, $CDCl_3$) δ 7.94–7.92 (m, 2H), 7.63–7.60 (m, 1H), 7.49–7.46 (m, 2H), 6.99 (dt, *J* = 6.8, 16.2 Hz, 1H), 6.46–6.41 (m, 1H), 2.30–2.26 (m, 2H), 1.49–1.44 (m, 2H), 1.31–1.23 (m, 4H), 0.86 (t, *J* = 7.0 Hz, 3H); ^{13}C NMR (151 MHz, $CDCl_3$) δ 193.9, 193.8, 156.1, 134.7, 132.9, 130.1, 128.9, 127.1, 33.3, 31.4, 27.4, 22.5, 14.0. HRMS (ESI-Quadrupole-Orbitrap) m/z: [M + H]$^+$ Calcd for $C_{15}H_{19}O_2$ 231.1380, found 231.1378. IR (KBr thin film, cm^{-1}): ν 3043, 2996, 2980, 1752, 1720, 1604, 1586, 1506, 1428, 1050, 954.

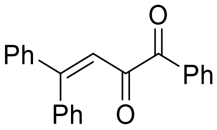

1r

1,4,4-Triphenylbut-3-ene-1,2-dione (1r): Purified by chromatography on silica gel (petroleum ether/ethyl acetate, v:v = 10:1), yellow solid, mp 142–143 ºC, 721 mg, 70% yield. ^{1}H NMR (600 MHz, $CDCl_3$) δ 7.73–7.70 (m, 2H), 7.54–7.51 (m, 1H), 7.42–7.39 (m, 1H), 7.37–7.33 (m, 6H), 7.17–7.16 (m, 1H), 7.11–7.04 (m, 4H), 6.92 (s, 1H); ^{13}C NMR (151 MHz, $CDCl_3$) δ 194.3, 193.0, 160.3, 139.8, 137.9, 134.0, 132.8, 130.6, 130.5, 129.8, 129.4, 129.0, 128.7, 128.3, 128.1, 123.6. HRMS (ESI-Quadrupole-Orbitrap) m/z: [M + H]$^+$ Calcd for $C_{22}H_{17}O_2$ 313.1223, found 313.1222. IR (KBr thin film, cm^{-1}): ν 3093, 3063, 3030, 1735, 1719, 1676, 1608, 1532, 1507, 1433, 764.

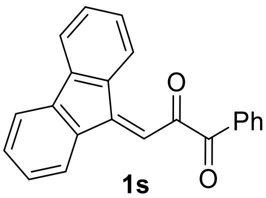

1s

3-(9*H*-Fluoren-9-ylidene)-1-phenylpropane-1,2-dione (1s): Purified by chromatography on silica gel (petroleum ether/ethyl acetate, v:v = 10:1), red solid, mp 167–173 ºC, 307 mg, 30% yield. ^{1}H NMR (400 MHz, $CDCl_3$) δ 9.10 (d, *J* = 7.8 Hz, 1H), 8.11 (d, *J* = 8.1 Hz, 2H), 7.71 (d, *J* = 7.6 Hz, 1H), 7.66–7.63 (m, 1H), 7.60–7.50

(m, 5H), 7.45–7.32 (m, 3H), 7.25–7.22 (m, 1H); ^{13}C NMR (101 MHz, $CDCl_3$) δ 192.8, 190.6, 152.5, 143.2, 141.8, 139.0, 135.4, 134.7, 132.5, 131.9, 130.6, 129.7, 128.9, 128.7, 127.9, 122.3, 120.2, 120.0, 115.3. HRMS (ESI-Quadrupole-Orbitrap) m/z: [M + H]$^+$ Calcd for $C_{22}H_{15}O_2$ 311.1067, found 311.1068. IR (KBr thin film, cm^{-1}): ν 3109, 3097, 3032, 1751, 1737, 1650, 1610, 1590, 1500, 1468, 763.

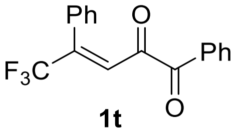

1t

(*E*)-5,5,5-Trifluoro-1,4-diphenylpent-3-ene-1,2-dione (1t): Purified by chromatography on silica gel (petroleum ether/ethyl acetate, v:v = 10:1), yellow oil, 291 mg, 29% yield. ^{1}H NMR (400 MHz, $CDCl_3$) δ 7.68–7.66 (m, 2H), 7.56–7.52 (m, 1H), 7.37–7.32 (m, 2H), 7.22–7.09 (m, 6H); ^{13}C NMR (101 MHz, $CDCl_3$) δ 192.0, 190.2, 144.4 (q, J = 31.7 Hz), 134.6, 131.6, 130.1, 129.9, 129.7, 128.9 (q, J = 5.0 Hz), 128.5, 128.4, 122.4 (q, J = 276.4 Hz). HRMS (ESI-Quadrupole-Orbitrap) m/z: [M + Na]$^+$ Calcd for $C_{17}H_{12}O_2F_3Na$ 327.0603, found 327.0602. IR (KBr thin film, cm^{-1}): ν 3006, 2987, 2843, 2348, 1754, 1737, 1614, 1590, 1503, 1275, 1260.

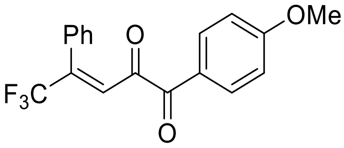

1u

(*E*)-5,5,5-Trifluoro-1-(4-methoxyphenyl)-4-phenylpent-3-ene-1,2-dione (1u): Purified by chromatography on silica gel (petroleum ether/ethyl acetate, v:v = 10:1), yellow oil, 408 mg, 37% yield. ^{1}H NMR (400 MHz, $CDCl_3$) δ 7.68 (d, J = 8.8 Hz, 2H), 7.25–7.10 (m, 5H), 7.10 (s, 1H), 6.82 (d, J = 8.9 Hz, 1H), 3.84 (s, 3H); ^{13}C NMR (101 MHz, $CDCl_3$) δ 192.2, 188.7, 164.8, 143.9 (q, J = 30.3 Hz), 132.5, 130.0, 129.7, 129.0 (q, J = 5.1 Hz), 128.3, 124.7, 122.9 (q, J = 272.7 Hz), 113.9, 55.6. HRMS (ESI-Quadrupole-Orbitrap) m/z: [M + H]$^+$ Calcd for $C_{18}H_{14}O_3F_3$ 335.0890, found 335.0892. IR (KBr thin film, cm^{-1}): ν 3089, 2986,2943, 1754, 1732, 1689, 1615, 1500, 1360, 1057, 954.

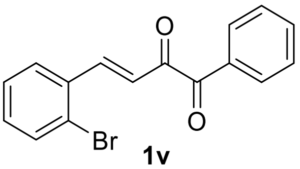

1v

(*E*)-4-(2-Bromophenyl)-1-phenylbut-3-ene-1,2-dione (1v): Purified by chromatography on silica gel (petroleum ether/ethyl acetate, v:v = 20:1), yellow solid,

mp 84–86 ºC, yellow solid, 440 mg, 14% yield. ^{1}H NMR (600 MHz, $CDCl_3$) δ 8.13 (d, *J* =16.3 Hz, 1H), 8.05–8.03 (m, 2H), 7.72 (dd, *J* = 7.9, 1.6 Hz, 1H), 7.66–7.61 (m, 2H), 7.52–7.50 (m, 2H), 7.36–7.34 (m, 1H), 7.28–7.25 (m, 1H), 7.11 (d, *J* = 16.3 Hz, 1H); ^{13}C NMR (101 MHz, $CDCl_3$) δ 192.9, 192.0, 146.8, 134.8, 134.0, 133.7, 132.7, 132.3, 130.3, 128.9, 128.1, 127.9, 126.4, 124.5. HRMS (ESI-Quadrupole-Orbitrap) m/z: $[M + H]^+$ Calcd for $C_{16}H_{12}O_2Br$ 315.0015, found. 315.0017. IR (KBr thin film, cm^{-1}): ν 3022, 2982, 1726, 1708, 1673, 1622, 1564, 1507, 1457, 904, 594.

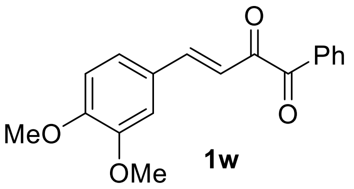

1w

(*E*)-4-(3,4-Dimethoxyphenyl)-1-phenylbut-3-ene-1,2-dione (1w): Purified by chromatography on silica gel (petroleum ether/ethyl acetate, v:v = 6:1), yellow solid, mp 160–161 ºC, 799 mg, 27% yield. ^{1}H NMR (400 MHz, $CDCl_3$) δ 8.04 (d, *J* = 7.3 Hz, 2H), 7.68–7.62 (m, 2H), 7.54–7.48 (m, 2H), 7.19 (dd, *J* = 8.3, 1.8 Hz, 1H), 7.12 (d, *J* = 1.8 Hz, 1H), 7.01 (d, *J* = 16.3 Hz, 1H), 6.89 (d, *J* = 8.3 Hz, 1H), 3.91 (s, 3H), 3.90 (s, 3H); ^{13}C NMR (101 MHz, $CDCl_3$) δ 193.7, 192.9, 152.4, 149.4, 149.2, 134.7, 133.0, 130.3, 129.0, 127.1, 124.4, 120.4, 111.2, 110.0, 56.1, 56.0. HRMS (ESI-Quadrupole-Orbitrap) m/z: $[M + H]^+$ Calcd for $C_{18}H_{17}O_4$ 297.1121, found 297.1120. IR (KBr thin film, cm^{-1}): ν 3047, 3020, 2974, 2359, 1704, 1660, 1583, 1494, 1435, 963, 889.

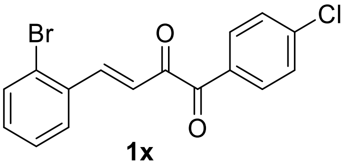

1x

(*E*)-4-(2-Bromophenyl)-1-(4-chlorophenyl)but-3-ene-1,2-dione (1x): Purified by chromatography on silica gel (petroleum ether/ethyl acetate, v:v = 10:1), yellow solid, mp 115–117 ºC, 626 mg, 18% yield. ^{1}H NMR (600 MHz, $CDCl_3$) δ 8.15 (d, *J* =16.3 Hz, 1H), 8.04–8.03 (m, 1H), 7.94–7.92 (m, 1H), 7.73 (dd, *J* = 7.8, 1.5 Hz, 1H), 7.63–7.60 (m, 2H), 7.47–7.44 (m, 1H), 7.37–7.35 (m, 1H), 7.29–7.26 (m, 1H), 7.14 (d, *J* = 16.3 Hz, 1H); ^{13}C NMR (151 MHz, $CDCl_3$) δ 191.2, 190.8, 147.2, 135.3, 134.7, 134.2, 134.0, 133.8, 132.6, 130.3, 130.2, 128.6, 128.2, 128.0, 126.6, 124.0. HRMS (ESI-Quadrupole-Orbitrap) m/z: $[M + H]^+$ Calcd for $C_{16}H_{11}O_2BrCl$ 348.9625, found 348.9626. IR (KBr thin film, cm^{-1}): ν 3086, 3067, 1730, 1679, 1666, 1626, 1593, 1565, 1487, 862, 790.

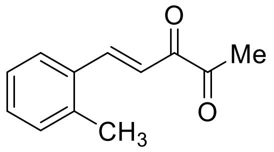

1zc

(*E*)-5-(o-Tolyl)pent-4-ene-2,3-dione(1zc): Purified by chromatography on silica gel (petroleum ether/ethyl acetate, v:v = 30:1), yellow solid, mp 57–59 ºC, 527 mg, 14% yield. ^{1}H NMR (400 MHz, $CDCl_3$) δ 8.17 (d, *J* = 16.0 Hz, 1H), 7.71 (d, *J* = 7.8 Hz, 1H), 7.39 (d, *J* = 16.0 Hz, 1H), 7.34–7.31 (m, 1H), 7.26–7.21 (m, 2H), 2.48 (s, 3H), 2.46 (s, 3H); ^{13}C NMR (101 MHz, $CDCl_3$) δ 199.1, 186.7, 145.2, 139.0, 133.3, 131.2, 131.1, 126.8, 126.5, 118.7, 24.5, 19.8. HRMS (ESI-Quadrupole-Orbitrap) m/z: [M + H]$^+$ Calcd for $C_{20}H_{13}O_2$ 189.0910, found 189.0908. IR (KBr thin film, cm^{-1}): ν 3019, 2951, 2924, 1741, 1698, 1626, 1608, 1539, 1501, 1349, 780.

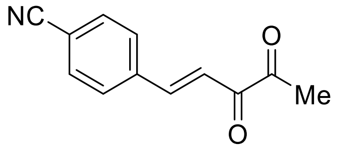

1ze

(*E*)-4-(3,4-Dioxopent-1-en-1-yl)benzonitrile(1ze): Purified by chromatography on silica gel (petroleum ether/ethyl acetate, v:v = 5:1), yellow solid, mp 125–127 ºC, 113 mg, 3% yield. ^{1}H NMR (400 MHz, $CDCl_3$) δ 7.80 (d, *J* = 16.2 Hz, 1H), 7.72 (s, 4H), 7.54 (d, *J* = 16.2 Hz, 1H), 2.47 (s, 3H); ^{13}C NMR (151 MHz, $CDCl_3$) δ 198.3, 186.1, 144.8, 138.6, 132.8, 129.2, 121.0, 118.3, 114.3, 24.3; HRMS (ESI-Quadrupole-Orbitrap) m/z: [M + Na]$^+$ Calcd for $C_{12}H_9O_2NNa$ 222.0525, found 222.0526. IR (KBr thin film, cm^{-1}): ν 3172, 3056, 3025, 2971, 1742, 1711, 1676, 1591, 1579, 831, 774.

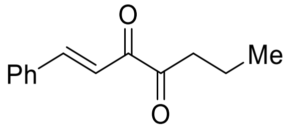

1zg

(*E*)-1-Phenylhept-1-ene-3,4-dione(1zg): Purified by chromatography on silica gel (petroleum ether/ethyl acetate, v:v = 50:1), yellow oil, 405 mg, 34% yield. ^{1}H NMR (600 MHz, $CDCl_3$) δ 7.81 (d, *J* = 16.1 Hz, 1H), 7.63–7.61 (m, 2H), 7.44–7.38 (m, 4H), 2.83 (t, *J* = 7.2 Hz, 2H), 1.71–1.61 (m, 2H), 0.97 (t, *J* = 7.5 Hz, 3H); ^{13}C NMR (151 MHz, $CDCl_3$) δ 201.4, 187.6, 147.7, 134.5, 131.5, 129.1, 129.0, 118.6, 38.7, 16.7, 13.8. HRMS (ESI-Quadrupole-Orbitrap) m/z: [M + H]$^+$ Calcd for $C_{13}H_{15}O_2$ 203.1067 , found 203.1065. IR (KBr thin film, cm^{-1}): ν 3030, 2962, 2875, 1722, 1692, 1673, 1624, 1568, 1454, 1398, 884.

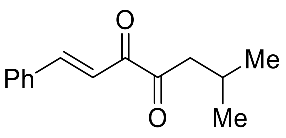

1zh

(*E*)-6-Methyl-1-phenylhept-1-ene-3,4-dione(1zh): Purified by chromatography on silica gel (petroleum ether/ethyl acetate, v:v = 20:1), yellow oil, 778 mg, 61% yield. ^{1}H NMR (600 MHz, $CDCl_3$) δ 7.80 (d, *J* = 16.2 Hz, 1H), 7.62–7.61 (m, 2H), 7.42–7.38 (m, 4H), 2.73 (d, *J* = 6.9 Hz, 2H), 2.22–2.16 (m, 1H), 0.96 (d, *J* = 6.7 Hz, 6H); ^{13}C NMR (151 MHz, $CDCl_3$) δ 201.2, 187.6, 147.7, 134.5, 131.5, 129.1, 129.0, 118.5, 45.5, 24.4, 22.7. HRMS (ESI-Quadrupole-Orbitrap) m/z: $[M + H]^+$ Calcd for $C_{14}H_{17}O_2$ 217.1223, found 217.1221. IR (KBr thin film, cm^{-1}): ν 3062, 2976, 2876, 1726, 1692, 1676, 1584, 1451, 1409, 1363, 764.

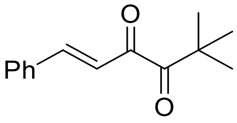

1zi

(*E*)-5,5-Dimethyl-1-phenylhex-1-ene-3,4-dione (1zi): Purified by chromatography on silica gel (petroleum ether/ethyl acetate, v:v = 10:1), yellow oil, 370 mg, 95% yield. ^{1}H NMR (600 MHz, $CDCl_3$) δ 7.57–7.51 (m, 3H), 7.41–7.37 (m, 3H), 6.98 (d, *J* = 16.3 Hz, 1H), 1.29 (s, 9H); ^{13}C NMR (151 MHz, $CDCl_3$) δ 209.1, 192.7, 147.8, 134.2, 131.5, 129.2, 128.9, 122.1, 42.6, 26.3. HRMS (ESI-Quadrupole-Orbitrap) m/z: $[M + H]^+$ Calcd for $C_{14}H_{17}O_2$ 217.1223, found 217.1222. IR (KBr thin film, cm^{-1}): ν 3070, 3023, 2830, 1702, 1681, 1655, 1603, 1561, 1450, 984, 749.

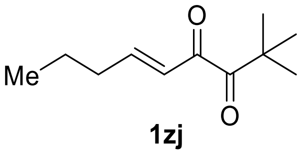

1zj

(*E*)-2,2-Dimethylnon-5-ene-3,4-dione (1zj): Purified by chromatography on silica gel (petroleum ether/ethyl acetate, v:v = 30:1), yellow oil, 250 mg, 47% yield. ^{1}H NMR (600 MHz, $CDCl_3$) δ 6.81–6.76 (m, 1H), 6.25 (d, *J* = 16.1 Hz, 1H), 2.25–2.21 (m, 2H), 1.52–1.46 (m, 2H), 1.22 (s, 9H), 0.91 (t, *J* = 7.4 Hz, 3H); ^{13}C NMR (151 MHz, $CDCl_3$) δ 209.9, 194.1, 154.3, 127.0, 42.4, 35.1, 26.3, 21.1, 13.7. HRMS (ESI-Quadrupole-Orbitrap) m/z: $[M + H]^+$ Calcd for $C_{11}H_{19}O_2$ 183.1380, found 183.1378. IR (KBr thin film, cm^{-1}): ν 3005, 2954, 2929, 1749, 1696, 1667, 1620, 1587, 1487, 1362, 847.

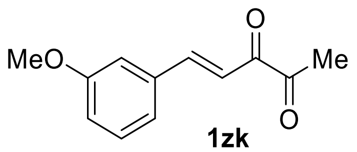

1zk

(*E*)-5-(3-Methoxyphenyl)pent-4-ene-2,3-dione (1zk): purified by chromatography on silica gel (petroleum ether/ethyl acetate, v:v = 10:1), yellow solid, mp 87–89 ºC, 660 mg, 16% yield. ^{1}H NMR (400 MHz, $CDCl_3$) δ 7.80 (d, *J* = 16.1 Hz, 1H), 7.41 (d, *J* = 16.2 Hz, 1H), 7.35–7.30 (m, 1H), 7.22 (d, *J* = 7.6 Hz, 1H), 7.14 (s, 1H), 6.99 (dd, *J* = 8.2, 2.6 Hz, 1H), 3.84 (s, 3H), 2.45 (s, 3H); ^{13}C NMR (101 MHz, $CDCl_3$) δ 198.9, 186.8, 160.0, 147.8, 135.7, 130.1, 121.9, 118.2, 117.6, 113.3, 55.4, 24.4. HRMS (ESI-Quadrupole-Orbitrap) m/z: $[M + H]^+$ Calcd for $C_{12}H_{13}O_3$ 205.0859, found 205.0857. IR (KBr thin film, cm-^{1}): ν 3096, 2947, 2922, 2860, 1756, 1698, 1605, 1536, 1442, 1363, 914.

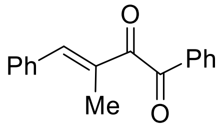

1zl

(*E*)-3-Methyl-1,4-diphenylbut-3-ene-1,2-dione (1zl): Purified by chromatography on silica gel (petroleum ether/ethyl acetate, v:v = 30:1), yellow oil, 519 mg, 42% yield. ^{1}H NMR (400 MHz, $CDCl_3$) δ 7.94 (d, *J* = 7.4 Hz, 2H), 7.65–7.61 (m, 1H), 7.52–7.48 (m, 2H), 7.43–7.33 (m, 6H), 2.26 (s, 3H); ^{13}C NMR (101 MHz, $CDCl_3$) δ 198.1, 196.0, 147.7, 134.9, 134.8, 134.3, 133.5, 130.4, 129.9, 129.8, 129.1, 128.7, 12.3; HRMS (ESI-Quadrupole-Orbitrap) m/z: $[M + H]^+$ Calcd for $C_{17}H_{14}O_2$ 251.1067, found 251.1066. IR (KBr thin film, cm^{-1}): ν 3096, 3045, 2953, 1734, 1665, 1602, 1585, 1516, 1427, 1383, 916.

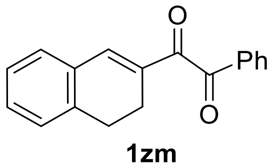

1zm

1-(3,4-Dihydronaphthalen-2-yl)-2-phenylethane-1,2-dione (1zm): Purified by chromatography on silica gel (petroleum ether/ethyl acetate, v:v = 10:1), yellow solid, mp 101–103 ºC, 721 mg, 55% yield. ^{1}H NMR (600 MHz, $CDCl_3$) δ 7.96–7.94 (m, 2H), 7.65–7.63 (m, 1H), 7.52–7.49 (m, 2H), 7.31–7.27 (m, 2H), 7.20–7.13 (m, 3H), 2.94 (t, *J* = 8.1 Hz, 2H), 2.75 (t, *J* = 8.1 Hz, 2H); ^{13}C NMR (151 MHz, $CDCl_3$) δ 195.7, 195.0, 144.3, 138.1, 134.8, 134.4, 133.4, 131.9, 131.2, 130.0, 129.8, 129.1, 128.1, 127.1, 27.2, 20.1. HRMS (ESI-Quadrupole-Orbitrap) m/z: $[M + H]^+$ Calcd for

$C_{18}H_{15}O_2$ 263.1067, found 263.1065. IR (KBr thin film, cm^{-1}): ν 3048, 2968, 2861, 1707, 1675, 1662, 1616, 1552, 1447, 1370, 787.

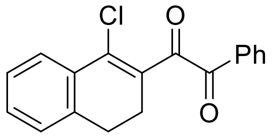

1zn

1-(1-Chloro-3,4-dihydronaphthalen-2-yl)-2-phenylethane-1,2-dione (1zn): Purified by chromatography on silica gel (petroleum ether/ethyl acetate, v:v = 5:1), orange oil, 400 mg, 27% yield. ^{1}H NMR (600 MHz, $CDCl_3$) δ 7.97–7.95 (m, 2H), 7.71 (d, *J* = 7.8 Hz, 1H), 7.63–7.61 (m, 1H), 7.52–7.49 (m, 2H), 7.35–7.33 (m, 1H), 7.28–7.26 (m, 1H), 7.21 (d, *J* = 7.4 Hz, 1H), 2.95–2.92 (m, 2H), 2.86–2.83 (m, 2H); ^{13}C NMR (151 MHz, $CDCl_3$) δ 195.3, 193.6, 141.8, 138.9, 134.4, 132.8, 131.64, 131.59, 131.5, 130.1, 129.0, 127.8, 127.3, 126.8, 27.4, 24.9. HRMS (ESI-Quadrupole-Orbitrap) m/z: $[M + H]^+$ Calcd for $C_{18}H_{14}O_2Cl$ 297.0677, found 297.0678. IR (KBr thin film, cm^{-1}): ν 3109, 2957, 2878, 1717, 1683, 1620, 1554, 1502, 1405, 1364, 781.

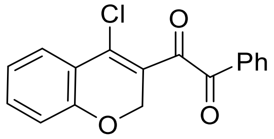

1zo

1-(4-Chloro-*2H*-chromen-3-yl)-2-phenylethane-1,2-dione (1zo): Purified by chromatography on silica gel (petroleum ether/ethyl acetate, v:v = 10:1), yellow solid, mp 135–139 ºC, 715 mg, 48% yield. ^{1}H NMR (600 MHz, $CDCl_3$) δ 7.94–7.92 (m, 2H), 7.67–7.64 (m, 1H), 7.60 (dd, *J* = 1.5, 7.9 Hz, 1H), 7.54–7.51 (m, 2H), 7.38 (ddd, *J* = 8.6, 5.9, 1.6 Hz, 1H), 7.03 (ddd, *J* = 8.5, 6.7, 1.0 Hz, 1H), 6.94 (dd, *J* = 8.3, 0.8 Hz, 1H), 5.17 (s, 2H); ^{13}C NMR (151 MHz, $CDCl_3$) δ 193.1, 192.6, 156.8, 141.0, 134.7, 134.5, 132.6, 130.0, 129.1, 127.3, 123.7, 122.5, 120.8, 116.8, 65.6. HRMS (ESI-Quadrupole-Orbitrap) m/z: $[M + Na]^+$ Calcd for $C_{17}H_{11}O_3ClNa$ 321.0289, found 321.0290. IR (KBr thin film, cm^{-1}): ν 3061, 3021, 2961, 2833, 1738, 1682, 1590, 1558, 1475, 800, 763.

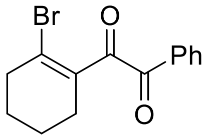

1zp

1-(2-Bromocyclohex-1-en-1-yl)-2-phenylethane-1,2-dione (1zp): Purified by chromatography on silica gel (petroleum ether/ethyl acetate, v:v = 15:1), yellow solid,

mp 93–97 ºC, 599 mg, 41% yield. ^{1}H NMR (600 MHz, $CDCl_3$) δ 7.95–7.93 (m, 2H), 7.61–7.58 (m, 1H), 7.49–7.46 (m, 2H), 2.69–2.65 (m, 2H), 2.52–2.49 (m, 2H), 1.81–1.73 (m, 4H); ^{13}C NMR (151 MHz, $CDCl_3$) δ 195.7, 192.3, 135.6, 134.6, 134.2, 132.9, 130.3, 128.8, 38.1, 28.4, 24.2, 21.2. HRMS (ESI-Quadrupole-Orbitrap) m/z: $[M + H]^+$ Calcd for $C_{14}H_{14}O_2Br$ 293.0172, found 293.0173. IR (KBr thin film, cm^{-1}): ν 3040, 2925, 2907, 1741, 1671, 1586, 1556, 1408, 1235, 917, 715.

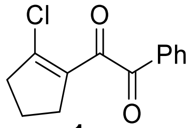

1zq

1-(2-Chlorocyclopent-1-en-1-yl)-2-phenylethane-1,2-dione (1zq): Purified by chromatography on silica gel (petroleum ether/ethyl acetate, v:v = 15:1), brown solid, mp 42–44 ºC, 293 mg, 25% yield. ^{1}H NMR (600 MHz, $CDCl_3$) δ 7.86–7.85 (m, 2H), 7.62–7.59 (m, 1H), 7.49–7.46 (m, 2H), 2.83–2.78 (m, 4H), 2.05–1.99 (m, 2H); ^{13}C NMR (151 MHz, $CDCl_3$) δ 194.1, 192.1, 149.0, 134.9, 134.6, 132.7, 129.8, 129.0, 41.4, 31.3, 20.6. HRMS (ESI-Quadrupole-Orbitrap) m/z: $[M + H]^+$ Calcd for $C_{13}H_{12}O_2Br$ 235.0520, found 235.0518. IR (KBr thin film, cm^{-1}): ν 3066, 2983, 2872, 1801, 1682, 1651, 1598, 1451, 1355, 839, 764.

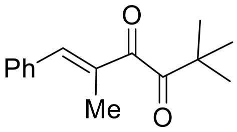

1zr

(*E*)-2,5,5-Trimethyl-1-phenylhex-1-ene-3,4-dione (1zr): Purified by chromatography on silica gel (petroleum ether/ethyl acetate, v:v = 20:1), yellow solid, mp 93–96 ºC, 462 mg, 77% yield. ^{1}H NMR (400 MHz, $CDCl_3$) δ 7.46–7.35 (m, 5H), 7.14 (d, J = 0.9 Hz, 1H), 2.13 (d, J = 1.2 Hz, 3H), 1.29 (s, 9H); ^{13}C NMR (101 MHz, $CDCl_3$) δ 212.6, 198.9, 146.5, 134.9, 134.0, 130.2, 129.7, 128.7, 42.7, 26.6, 11.9. HRMS (ESI-Quadrupole-Orbitrap) m/z: $[M + H]^+$ Calcd for $C_{15}H_{19}O_2$ 231.1380, found: 231.1381. IR (KBr thin film, cm^{-1}): ν 3065, 2973, 2908, 2871, 1805, 1702, 1659, 1599, 1462, 1450, 903.

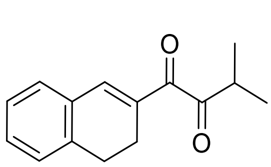

1zs

1-(3,4-Dihydronaphthalen-2-yl)-3-methylbutane-1,2-dione (1zs): Purified by chromatography on silica gel (petroleum ether/ethyl acetate, v:v = 20:1), yellow oil,

362 mg, 61% yield. ^{1}H NMR (400 MHz, CDCl$_3$) δ 7.38 (s, 1H), 7.32–7.29 (m, 1H), 7.24–7.18 (m, 3H), 3.31–3.23 (m, 1H), 2.90 (t, *J* = 8.8 Hz, 2H), 2.63 (t, *J* = 8.8 Hz, 2H), 1.19 (d, *J* = 7.0 Hz, 6H); ^{13}C NMR (101 MHz, CDCl$_3$) δ 207.1, 195.1, 143.4, 138.0, 133.6, 132.1, 131.0, 129.7, 128.0, 127.0, 36.8, 27.1, 20.3, 17.1. HRMS (ESI-Quadrupole-Orbitrap) m/z: [M + H]$^+$ Calcd for $C_{15}H_{17}O_2$ 229.1223, found 229.1222. IR (KBr thin film, cm^{-1}): ν 3080, 3045, 3017, 1794, 1735, 1570, 1556, 910, 907, 880, 678.

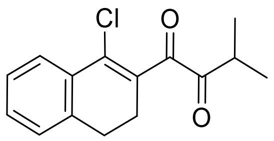

1zt

1-(1-Chloro-3,4-dihydronaphthalen-2-yl)-3-methylbutane-1,2-dione (1zt): Purified by chromatography on silica gel (petroleum ether/ethyl acetate, v:v = 20:1), yellow oil, 393 mg, 69% yield. ^{1}H NMR (600 MHz, CDCl$_3$) δ 7.74 (d, *J* = 7.7 Hz, 1H), 7.35–7.28 (m, 2H), 7.19 (d, *J* = 7.6 Hz, 1H), 3.23–3.18 (m, 1H), 2.89 (t, *J* = 7.6 Hz, 2H), 2.69 (t, *J* = 7.6 Hz, 2H), 1.29 (d, *J* = 7.1 Hz, 6H); ^{13}C NMR (151 MHz, CDCl$_3$) δ 206.4, 195.7, 139.7, 138.4, 131.6, 131.2, 131.1, 127.7, 127.2, 126.5, 37.0, 27.3, 25.2, 18.0. HRMS (ESI-Quadrupole-Orbitrap) m/z: [M + H]$^+$ Calcd for $C_{15}H_{16}O_2Cl$ 263.0833, found 263.0832. IR (KBr thin film, cm^{-1}): ν 3077, 2983, 2964, 1719, 1707, 1647, 1588, 1556, 1453, 835, 719.

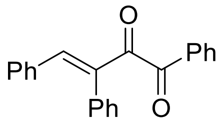

1zv

(*E*)-1,3,4-Triphenylbut-3-ene-1,2-dione (1zv): Purified by chromatography on silica gel (petroleum ether/ethyl acetate, v:v = 20:1), yellow solid, mp 101–105 ºC, 733 mg, 47% yield. ^{1}H NMR (600 MHz, CDCl$_3$) δ 7.98–7.96 (m, 2H), 7.66–7.64 (m, 1H), 7.55 (s, 1H), 7.53–7.51 (m, 3H), 7.43–7.38 (m, 3H), 7.28–7.23 (m, 2H), 7.17–7.14 (m, 2H) 7.07 (d, *J* = 7.6 Hz, 2H); ^{13}C NMR (101 MHz, CDCl$_3$) δ 196.4, 195.4, 147.0, 137.7, 134.8, 133.84, 133.80, 133.4, 131.3, 130.5, 129.78, 129.80, 129.1, 129.0, 128.6, 128.4. HRMS (ESI-Quadrupole-Orbitrap) m/z: [M + H]$^+$ Calcd for $C_{22}H_{17}O_2$ 313.1223, found 313.1224. IR (KBr thin film, cm^{-1}): ν 3028, 3018, 2932, 1727, 1708, 1646, 1594, 1577, 1429, 782, 718.

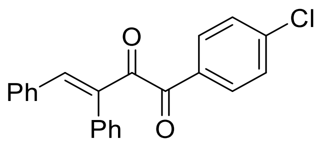

1zw

(*E*)-1-(4-Chlorophenyl)-3,4-diphenylbut-3-ene-1,2-dione (1zw): Purified by chromatography on silica gel (petroleum ether/ethyl acetate, v:v = 20:1), white solid, mp 153–155 ºC, 588 mg, 34% yield. ^{1}H NMR (600 MHz, $CDCl_3$) δ 7.92–7.89 (m, 2H), 7.56 (s, 1H), 7.49–7.48 (m, 2H), 7.43–7.40 (m, 3H), 7.27–7.24 (m, 3H), 7.18–7.15 (m, 2H) 7.08 (d, J = 7.6 Hz, 2H); ^{13}C NMR (151 MHz, $CDCl_3$) δ 195.8, 194.0, 147.2, 141.5, 137.6, 133.81, 133.77, 131.8, 131.4, 131.2, 130.7, 129.9, 129.5, 129.2, 128.7, 128.6. HRMS (ESI-Quadrupole-Orbitrap) m/z: $[M + H]^+$ Calcd for $C_{22}H_{16}O_2Cl$ 347.0833, found 347.0834. IR (KBr thin film, cm^{-1}): ν 3035, 2929, 2880, 2853, 1725, 1702, 1657, 1554, 1417, 910, 714.

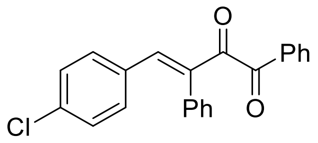

1zx

(*E*)-4-(4-Chlorophenyl)-1,3-diphenylbut-3-ene-1,2-dione (1zx): Purified by chromatography on silica gel (petroleum ether/ethyl acetate, v:v = 20:1), yellow solid, mp 118–124 ºC, 1021 mg, 59% yield. ^{1}H NMR (400 MHz, $CDCl_3$) δ 7.97 (d, J = 7.3 Hz, 2H), 7.68–7.64 (m, 1H), 7.55–7.50 (m, 3H), 7.44–7.40 (m, 3H), 7.27–7.24 (m, 2H), 7.15–7.12 (m, 2H), 6.99 (d, J = 8.6 Hz, 2H); ^{13}C NMR (101 MHz, $CDCl_3$) δ 196.1, 195.1, 145.2, 138.1, 136.5, 134.9, 133.5, 133.3, 132.4, 132.3, 129.8, 129.7, 129.2, 129.1, 128.8. HRMS (ESI-Quadrupole-Orbitrap) m/z: $[M + H]^+$ Calcd for $C_{22}H_{16}O_2Cl$ 347.0833, found 347.0835. IR (KBr thin film, cm^{-1}): ν 3041, 2947, 2922, 2839, 1736, 1723, 1632, 1553, 1439, 886, 723.

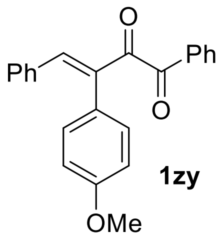

1zy

(*E*)-3-(4-Methoxyphenyl)-1,4-diphenylbut-3-ene-1,2-dione (1zy): Purified by chromatography on silica gel (petroleum ether/ethyl acetate, v:v = 20:1), yellow solid, mp 124–125 ºC, 1112 mg, 65% yield. ^{1}H NMR (400 MHz, $CDCl_3$) δ 7.99–7.95 (m, 2H), 7.67–7.61 (m, 1H), 7.54–7.48 (m, 3H), 7.28–7.10 (m, 7H), 6.94 (d, J = 8.7 Hz,

2H); ^{13}C NMR (151 MHz, $CDCl_3$) δ 196.9, 195.8, 159.8, 147.0, 137.4, 134.9, 134.1, 133.4, 131.3, 131.2, 130.5, 129.9, 129.1, 128.6, 125.8, 114.6, 55.4. HRMS (ESI-Quadrupole-Orbitrap) m/z: $[M + H]^+$ Calcd for $C_{23}H_{19}O_3$ 343.1329, found 343.1331. IR (KBr thin film, cm^{-1}): ν 3039, 2976, 2947, 2900, 1753, 1688, 1610, 1597, 1437, 1361, 903.

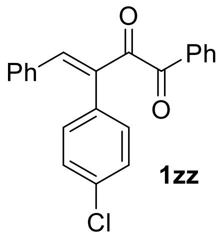

1zz

(*E*)-3-(4-Chlorophenyl)-1,4-diphenylbut-3-ene-1,2-dione (1zz): Purified by chromatography on silica gel (petroleum ether/ethyl acetate, v:v = 20:1), yellow solid, mp 134–135 ºC, 606 mg, 35% yield. ^{1}H NMR (600 MHz, $CDCl_3$) δ 7.97–7.95 (m, 2H), 7.67–7.65 (m, 1H), 7.54–7.51 (m, 3H), 7.42–7.39 (m, 2H), 7.29–7.18 (m, 5H), 7.08 (d, *J* = 7.6 Hz, 2H); ^{13}C NMR (151 MHz, $CDCl_3$) δ 196.1, 195.2, 147.8, 136.6, 135.0, 134.7, 133.5, 133.3, 132.3, 131.4, 131.3, 130.8, 129.9, 129.4, 129.2, 128.7. HRMS (ESI-Quadrupole-Orbitrap) m/z: $[M + H]^+$ Calcd for $C_{17}H_{14}O_2Cl$ 347.0833, found 347.0835. IR (KBr thin film, cm^{-1}): ν3024, 2957, 2916, 1704, 1654, 1616, 1596, 1560, 1481, 793, 691.

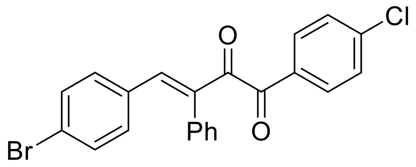

1zaa

(*E*)-4-(4-Bromophenyl)-1-(4-chlorophenyl)-3-phenylbut-3-ene-1,2-dione (1zaa): Purified by chromatography on silica gel (petroleum ether/ethyl acetate, v:v = 15:1), yellow solid, mp 184–187 ºC, 1483 mg, 70% yield. ^{1}H NMR (400 MHz, $CDCl_3$) δ 7.90 (d, *J* = 8.4 Hz, 2H), 7.52–7.47 (m, 3H), 7.47–7.40 (m, 3H), 7.35–7.27 (m, 2H), 7.27–7.19 (m, 2H), 6.93 (d, *J* = 8.4 Hz, 2H); ^{13}C NMR (101 MHz, $CDCl_3$) δ 195.4, 193.6, 145.2, 141.5, 138.0, 133.4, 132.6, 132.5, 131.8, 131.6, 131.1, 129.6, 129.5, 129.2, 128.9, 125.1. HRMS (ESI-Quadrupole-Orbitrap) m/z: $[M + H]^+$ Calcd for $C_{22}H_{15}O_2BrCl$ 424.9938, found 424.9940. IR (KBr thin film, cm^{-1}): ν 3023, 2993, 2961, 1730, 1710, 1682, 1608, 1587, 1489, 821, 754.

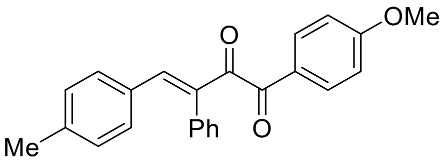

1zab

(*E*)-1-(4-Methoxyphenyl)-3-phenyl-4-p-tolylbut-3-ene-1,2-dione (1zab): Purified by chromatography on silica gel (petroleum ether/ethyl acetate, v:v = 10:1), yellow solid, mp 141–143 ºC, 445 mg, 25% yield. ^{1}H NMR (600 MHz, $CDCl_3$) δ 7.95–7.93 (m, 2H), 7.51 (s, 1H), 7.43–7.38 (m, 3H), 7.27–7.26 (m, 2H), 6.98–6.93 (m, 6H), 3.88 (s, 3H), 2.26 (s, 3H); ^{13}C NMR (101 MHz, $CDCl_3$) δ 196.8, 194.2, 164.8, 147.1, 141.1, 137.0, 134.3, 132.2, 131.3, 131.1, 129.8, 129.2, 129.0, 128.4, 126.6, 114.3, 55.7, 21.5; HRMS (ESI-Quadrupole-Orbitrap) m/z: $[M + H]^+$ Calcd for $C_{24}H_{21}O_3$ 357.1485, found 357.1487. IR (KBr thin film, cm^{-1}): ν 3096, 2996, 2918, 1714, 1668, 1616, 1584, 1451, 1312, 1059, 857.

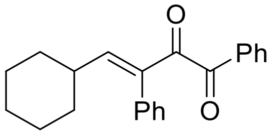

1zac

(*E*)-4-Cyclohexyl-1,3-diphenylbut-3-ene-1,2-dione (1zac): Purified by chromatography on silica gel (petroleum ether/ethyl acetate, v:v = 20:1), white solid, mp 131–135 ºC, 521 mg, 63% yield. ^{1}H NMR (600 MHz, $CDCl_3$) δ 7.93–7.90 (m, 2H), 7.64–7.61 (m, 1H), 7.51–7.48 (m, 2H), 7.43–7.40 (m, 2H), 7.38–7.35 (m, 1H), 7.23–7.21 (m, 2H), 6.62 (d, J = 10.3 Hz, 1H), 2.32–2.27 (m, 1H), 1.63–1.58 (m, 5H), 1.13–1.10 (m, 5H); ^{13}C NMR (151 MHz, $CDCl_3$) δ 196.4, 195.5, 158.3, 138.3, 134.7, 133.4, 129.8, 129.7, 129.0, 128.5, 128.2, 39.0, 31.8, 25.6, 25.0. HRMS (ESI-Quadrupole-Orbitrap) m/z: $[M + H]^+$ Calcd for $C_{22}H_{23}O_2$ 319.1693, found 319.1692. IR (KBr thin film, cm^{-1}): ν 3024, 2974, 2920, 2895, 2840, 2802, 1739, 1650, 1556, 1373, 881.

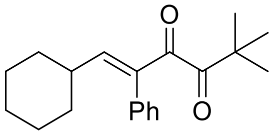

1zad

(*E*)-1-Cyclohexyl-5,5-dimethyl-2-phenylhex-1-ene-3,4-dione (1zad): Purified by chromatography on silica gel (petroleum ether/ethyl acetate, v:v = 20:1), yellow oil, 217 mg, 28% yield. ^{1}H NMR (400 MHz, $CDCl_3$) δ 7.41–7.35 (m, 3H), 7.13 (d, J = 7.1

Hz, 2H), 6.42 (d, *J* = 10.2 Hz, 1H), 2.27–2.22 (m, 1H), 1.68–1.58 (m, 5H), 1.25 (s, 9H), 1.22–1.15 (m, 5H); ^{13}C NMR (101 MHz, $CDCl_3$) δ 212.2, 197.5, 157.1, 137.8, 133.3, 129.6, 128.3, 128.0, 42.6, 38.8, 31.8, 26.6, 25.6, 24.9; HRMS (ESI-Quadrupole-Orbitrap) m/z: $[M + H]^+$ Calcd for $C_{20}H_{27}O_2$ 299.2006, found 299.2003. IR (KBr thin film, cm-1): ν 3088, 3028, 2954, 2947, 2914, 1724, 1680, 1560, 1501, 1393, 784.

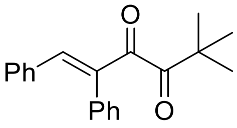

1zae

(*E*)-5,5-Dimethyl-1,2-diphenylhex-1-ene-3,4-dione (1zae): Purified by chromatography on silica gel (petroleum ether/ethyl acetate, v:v = 20:1), yellow oil, 190 mg, 13% yield. ^{1}H NMR (600 MHz, $CDCl_3$) δ 7.43–7.38 (m, 3H), 7.13 (s, 1H), 7.27–7.14 (m, 1H), 7.20–7.16 (m, 4H), 7.08–7.06 (m, 2H), 1.28 (s, 9H); ^{13}C NMR (151 MHz, $CDCl_3$) δ 212.1, 197.5, 145.9, 137.5, 133.90, 133.87, 131.2, 130.4, 129.9, 129.1, 128.6, 42.8, 26.7. HRMS (ESI-Quadrupole-Orbitrap) m/z: $[M + H]^+$ Calcd for $C_{20}H_{21}O_2$ 293.1536, found 293.1534. IR (KBr thin film, cm-1): ν 3060, 3028, 2978, 2885, 1724, 1696, 1655, 1553, 1440, 1359, 760.

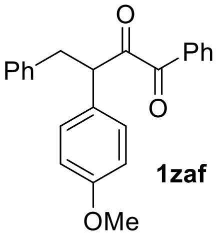

1zaf

3-(4-Methoxyphenyl)-1,4-diphenylbutane-1,2-dione (1zaf): Purified by chromatography on silica gel (petroleum ether/ethyl acetate, v:v = 20:1), yellow oil, 42 mg, 31% yield. ^{1}H NMR (400 MHz, $CDCl_3$) δ 7.71–7.68 (m, 2H), 7.55–7.50 (m, 1H), 7.38–7.34 (m, 2H), 7.24–7.18 (m, 2H), 7.17–7.12 (m, 5H), 6.79 (d, *J* = 8.7 Hz, 2H), 4.81 (dd, *J* = 7.6, 7.6 Hz, 1H), 3.73 (s, 3H), 3.53 (dd, *J* = 14.0, 7.3 Hz, 1H), 3.10 (dd, *J* = 14.0, 7.8 Hz, 1H); ^{13}C NMR (101 MHz, $CDCl_3$) δ 199.7, 192.6, 159.2, 139.0, 134.3, 132.3, 130.3, 130.0, 129.1, 128.6, 128.3, 126.9, 126.3, 114.6, 55.2, 54.1, 37.2. HRMS (ESI-Quadrupole-Orbitrap) m/z: $[M + Na]^+$ Calcd for $C_{23}H_{20}O_3Na$ 367.1305, found 367.1306. IR (KBr thin film, cm^{-1}): ν 3074, 3038, 2923, 1742, 1712, 1635, 1592, 1557, 1506, 1373, 922.

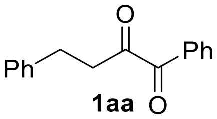

1aa

1,4-Diphenylbutane-1,2-dione (1aa): Purified by chromatography on silica gel (petroleum ether/ethyl acetate, v:v = 10:1), yellow oil, 714 mg, 60% yield. ^{1}H NMR (400 MHz, $CDCl_3$) δ 7.90–7.88 (m, 2H), 7.62–7.58 (m, 1H), 7.46–7.42 (m, 2H), 7.30–7.17 (m, 5H), 3.23 (t, J = 7.2 Hz, 2H), 3.21 (t, J = 7.2 Hz, 2H); ^{13}C NMR (151 MHz, $CDCl_3$) δ 202.4, 192.1, 140.3, 134.7, 131.9, 130.3, 128.9, 128.7, 128.5, 126.5, 40.3, 29.0. HRMS (ESI-Quadrupole-Orbitrap) m/z: $[M + H]^+$ Calcd for $C_{16}H_{12}O_2Br$ 239.1067, found 239.1068. IR (KBr thin film, cm^{-1}): ν 3064, 3031, 2945, 2931, 1713, 1673, 1597, 1580, 1497, 1450, 862.

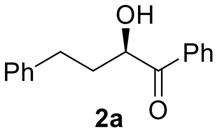

2a

(*R*)-2-Hydroxy-1,4-diphenylbutan-1-one (2a): Purified by flash chromatography on silica gel (petroleum ether/ethyl acetate, v:v = 5:1), colorless oil, 35.5 mg, 74% yield. ^{1}H NMR (600 MHz, $CDCl_3$) δ 7.78–7.57 (m, 2H), 7.60–7.57 (m, 1H), 7.46–7.43 (m, 2H), 7.28–7.25 (m, 2H), 7.20–7.16 (m, 3H), 5.03 (ddd, J = 8.6, 6.4, 3.0 Hz, 1H), 3.78 (d, J = 6.4 Hz, 1H), 2.88–2.76 (m, 2H), 2.18–2.12 (m, 1H), 1.85–1.78 (m, 1H); ^{13}C NMR (151 MHz, $CDCl_3$) δ 202.1, 141.1, 134.1, 133.5, 129.0, 128.8, 128.60, 128.57, 126.2, 72.3, 37.8, 31.4. HRMS (ESI-Quadrupole-Orbitrap) m/z: $[M + Na]^+$ Calcd for $C_{16}H_{16}O_2Na$ 263.1043, found 263.1041. $[\alpha]_D^{27}$: –89.9 (c 0.23, $CHCl_3$); HPLC analysis: 99% *ee* (Chiralcel AD-H, 2:98 ^{i}PrOH/hexanes, 1 mL/min, 254 nm), R_t (major) = 11.8 min, R_t (minor) = 11.1 min. IR (KBr thin film, cm^{-1}): ν 3324, 2942, 2928, 2860, 1734, 1614, 1558, 1520, 1436, 1239, 783.

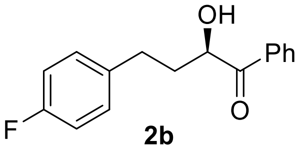

2b

(*R*)-4-(4-Fluorophenyl)-2-hydroxy-1-phenylbutan-1-one (2b): Purified by flash chromatography on silica gel (petroleum ether/ethyl acetate, v:v = 5:1), colorless oil, 35.1 mg, 68% yield. ^{1}H NMR (600 MHz, $CDCl_3$) δ 7.77–7.75 (m, 2H), 7.60–7.57 (m, 1H), 7.47–7.43 (m, 2H), 7.12–7.09 (m, 2H), 6.96–6.93 (m, 2H), 5.01 (ddd, J = 8.6, 6.3, 3.1 Hz, 1H), 3.76 (d, J = 6.3 Hz, 1H), 2.84–2.77 (m, 1H), 2.76–2.71 (m, 1H), 2.14–2.08 (m, 1H), 1.82–1.75 (m, 1H); ^{13}C NMR (151 MHz, $CDCl_3$) δ 202.0, 161.5 (d, J = 243.1 Hz), 136.7, 134.2, 133.5, 130.1 (d, J = 9.1 Hz), 129.0, 128.6, 115.3 (d, J = 21.1 Hz), 72.1, 37.8, 30.5. HRMS (ESI-Quadrupole-Orbitrap) m/z: $[M + Na]^+$ Calcd for $C_{16}H_{15}O_2FNa$ 281.0948, found 281.0950. $[\alpha]_D^{27}$: –13.9 (c 0.64, $CHCl_3$); HPLC analysis: 98% *ee* (Chiralcel AD-H, 5:95 ^{i}PrOH/hexanes, 1 mL/min, 254 nm),

R_t (major) = 8.6 min, R_t (minor) = 7.4 min. IR (KBr thin film, cm^{-1}): ν 3365, 3028, 2927, 2854, 1706, 1604, 1512, 1459, 1378, 1261, 804.

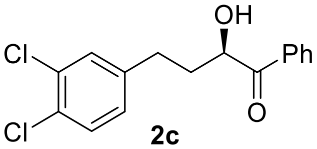

2c

(*R*)-4-(3,4-Dichlorophenyl)-2-hydroxy-1-phenylbutan-1-one (2c): Purified by flash chromatography on silica gel (petroleum ether/ethyl acetate, v:v = 10:1), colorless oil, 41.3 mg, 67% yield. ^{1}H NMR (600 MHz, $CDCl_3$) δ 7.78–7.77 (m, 2H), 7.62–7.59 (m, 1H), 7.49–7.46 (m, 2H), 7.31 (d, *J* = 8.2 Hz, 1H), 7.23 (d, *J* = 2.0 Hz, 1H), 6.99 (dd, *J* = 8.2, 2.0 Hz, 1H), 5.00 (ddd, *J* = 8.5, 6.2, 3.2 Hz, 1H), 3.78 (d, *J* = 6.2 Hz, 1H), 2.82–2.75 (m, 1H), 2.73–2.68 (m, 1H), 2.15–2.09 (m, 1H), 1.82–1.76 (m, 1H); ^{13}C NMR (151 MHz, $CDCl_3$) δ 201.7, 141.3, 134.3, 133.4, 132.4, 130.6, 130.4, 130.2, 129.1, 128.5, 128.3, 72.0, 37.2, 30.4. HRMS (ESI-Quadrupole-Orbitrap) m/z: $[M + Na]^+$ Calcd for $C_{16}H_{14}O_2Cl_2Na$ 331.0263, found 331.0261. $[\alpha]_D^{27}$: –20.1 (*c* 2.64, $CHCl_3$). HPLC analysis: 95% *ee* (Chiralcel AD-H, 5:95 ^{i}PrOH/hexanes, 1 mL/min, 254 nm), R_t (major) = 9.5 min, R_t (minor) = 7.6 min. IR (KBr thin film, cm^{-1}): ν 3380, 3034, 2967, 2914, 1747, 1557, 1458, 1442, 1298, 780, 717.

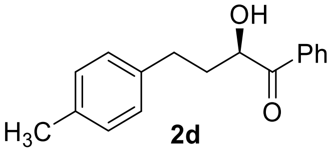

2d

(*R*)-2-Hydroxy-1-phenyl-4-(p-tolyl)butan-1-one (2d): Purified by flash chromatography on silica gel (petroleum ether/ethyl acetate, v:v = 5:1), yellow solid, mp 97–106 ºC, 35.3 mg, 72% yield. ^{1}H NMR (600 MHz, $CDCl_3$) δ 7.78–7.75 (m, 2H), 7.59–7.56 (m, 1H), 7.46–7.42 (m, 2H), 7.11–7.03 (m, 4H), 5.02 (ddd, *J* = 8.7, 6.3, 3.0 Hz, 1H), 3.76 (d, *J* = 6.4 Hz, 1H), 2.83–2.71 (m, 2H), 2.31 (s, 3H), 2.16–2.10 (m, 1H), 1.82–1.75 (m, 1H); ^{13}C NMR (151 MHz, $CDCl_3$) δ 202.1, 138.0, 135.7, 134.0, 133.5, 129.2, 128.9, 128.6, 72.4, 37.9, 30.9, 21.1. HRMS (ESI-Quadrupole-Orbitrap) m/z: $[M + Na]^+$ Calcd for $C_{17}H_{18}O_2Na$ 277.1199, found 277.1201. $[\alpha]_D^{27}$: –14.5 (*c* 2.06, $CHCl_3$); HPLC analysis: 99% *ee* (Chiralcel OJ-H, 5:95 ^{i}PrOH/hexanes, 1 mL/min, 254 nm), R_t (major) = 6.1 min, R_t (minor) = 7.0 min. IR (KBr thin film, cm^{-1}): ν 3402, 3050, 2926, 2911, 1758, 1557, 1446, 1307, 816, 751, 676.

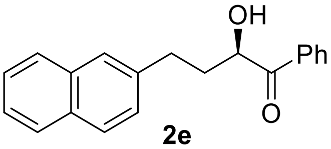

2e

(*R*)-2-Hydroxy-4-(naphthalen-2-yl)-1-phenylbutan-1-one (2e): Purified by flash chromatography on silica gel (petroleum ether/ethyl acetate, v:v = 10:1), yellow solid, mp 62–66 ºC, 35.3 mg, 64% yield. ^{1}H NMR (600 MHz, $CDCl_3$) δ 7.80–7.73 (m, 5H), 7.63 (s, 1H), 7.58–7.55 (m, 1H), 7.46–7.39 (m, 4H), 7.30 (dd, *J* = 8.4, 1.7 Hz, 1H), 5.05 (ddd, *J* = 8.7, 6.4, 3.0 Hz, 1H), 3.81 (d, *J* = 6.4 Hz, 1H), 3.04–2.92 (m, 2H), 2.28–2.22 (m, 1H), 1.93–1.87 (m, 1H); ^{13}C NMR (151 MHz, $CDCl_3$) δ 202.1, 138.5, 134.1, 133.7, 133.5, 132.2, 129.0, 128.6, 128.2, 127.7, 127.5, 127.4, 127.0, 126.1, 125.4, 72.2, 37.6, 31.5. HRMS (ESI-Quadrupole-Orbitrap) m/z: $[M + H]^+$ Calcd for $C_{20}H_{19}O_2$ 291.1380, found 291.1378. $[\alpha]_D^{27}$: –22.8 (*c* 1.05, $CHCl_3$); HPLC analysis: 99% *ee* (Chiralcel AD-H, 10:90 ^{i}PrOH/hexanes, 1 mL/min, 254 nm), R_t (major) = 6.8 min, R_t (minor) = 7.4 min. IR (KBr thin film, cm^{-1}): ν 3312, 3033, 2954, 2883, 1729, 1596, 1556, 1505, 1471, 1270, 728.

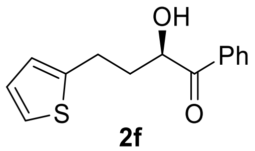

2f

(*R*)-2-Hydroxy-1-phenyl-4-(thiophen-2-yl)butan-1-one (2f): Purified by flash chromatography on silica gel (petroleum ether/ethyl acetate, v:v = 5:1), yellow oil, 33.5 mg, 68% yield. ^{1}H NMR (600 MHz, $CDCl_3$) δ 7.82–7.80 (m, 2H), 7.61–7.58 (m, 1H), 7.48–7.45 (m, 2H), 7.13 (dd, *J* = 5.2, 1.2 Hz, 1H), 6.92 (dd, *J* = 5.2, 3.4 Hz, 1H), 6.83–6.81 (m, 1H), 5.06 (ddd, *J* = 9.0, 6.4, 2.9 Hz, 1H), 3.75 (d, *J* = 6.4 Hz, 1H), 3.15–3.10 (m, 1H), 3.01–2.96 (m, 1H), 2.22–2.17 (m, 1H), 1.87–1.80 (m, 1H); ^{13}C NMR (151 MHz, $CDCl_3$) δ 201.9, 143.7, 134.2, 133.4, 129.0, 128.7, 127.0, 125.2, 123.6, 72.0, 38.0, 25.7. HRMS (ESI-Quadrupole-Orbitrap) m/z: $[M + Na]^+$ Calcd for $C_{14}H_{14}O_2SNa$ 269.0607, found 269.0608. $[\alpha]_D^{27}$: +16.7 (*c* 2.15, $CHCl_3$); HPLC analysis: >99% *ee* (Chiralcel OJ-H, 10:90 ^{i}PrOH/hexanes, 1 mL/min, 254 nm), R_t (major) = 8.3 min, R_t (minor) = 6.9 min. IR (KBr thin film, cm^{-1}): ν 3397, 3053, 2986, 1744, 1556, 1444, 1306, 1227, 777, 720, 678.

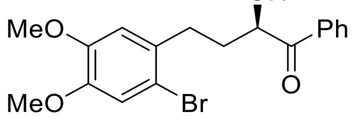

2g

(*R*)-4-(2-Bromo-4,5-dimethoxyphenyl)-2-hydroxy-1-phenylbutan-1-one (2g): Purified by flash chromatography on silica gel (petroleum ether/ethyl acetate, v:v = 3:1), yellow oil, 42.6 mg, 75% yield. ^{1}H NMR (600 MHz, $CDCl_3$) δ 7.83–7.80 (m, 2H), 7.60–7.57 (m, 1H), 7.47–7.44 (m, 2H), 6.94 (s, 1H), 6.71 (s, 1H), 5.04 (ddd, *J* = 8.3, 6.1, 3.1 Hz, 1H), 3.85–3.78 (m, 1H), 3.82 (s, 3H), 3.81 (s, 3H), 2.89–2.77 (m, 2H),

2.23–2.17 (m, 1H), 1.81–1.74 (m, 1H); ^{13}C NMR (151 MHz, $CDCl_3$) δ 201.8, 148.4, 148.1, 134.1, 133.4, 132.3, 128.9, 128.7, 115.6, 114.0, 113.5, 72.8, 56.3, 56.1, 36.1, 31.7. HRMS (ESI-Quadrupole-Orbitrap) m/z: $[M + Na]^+$ Calcd for $C_{18}H_{19}O_4BrNa$ 401.0359, found 401.0357. $[\alpha]_D^{27}$: +17.9 (*c* 0.9, $CHCl_3$); HPLC analysis: 98% *ee* (Chiralcel OJ-H, 15:85 ^{i}PrOH/hexanes, 1 mL/min, 254 nm), R_t (major) = 10.3 min, R_t (minor) = 9.1 min. IR (KBr thin film, cm^{-1}): ν3378, 3022, 2989, 1735, 1557, 1371, 1325, 1137, 895, 750, 686.

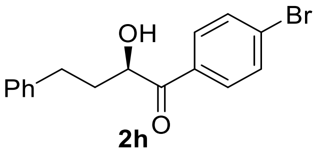

2h

(*R*)-1-(4-Bromophenyl)-2-hydroxy-4-phenylbutan-1-one (2h): Purified by flash chromatography on silica gel (petroleum ether/ethyl acetate, v:v = 10:1), yellow oil, 42.6 mg, 67% yield. ^{1}H NMR (600 MHz, $CDCl_3$) δ 7.60–7.55 (m, 4H), 7.30–7.26 (m, 2H), 7.22–7.13 (m, 3H), 4.95 (ddd, *J* = 8.7, 6.2, 2.9 Hz, 1H), 3.66 (d, *J* = 6.4 Hz, 1H), 2.86–2.75 (m, 2H), 2.13–2.07 (m, 1H), 1.83–1.76 (m, 1H); ^{13}C NMR (151 MHz, $CDCl_3$) δ 201.1, 140.9, 132.3, 132.2, 130.0, 129.3, 128.8, 128.6, 126.3, 72.1, 37.8, 31.3. HRMS (ESI-Quadrupole-Orbitrap) m/z: $[M + Na]^+$ Calcd for $C_{16}H_{15}O_2BrNa$ 341.0148, found 341.0146. $[\alpha]_D^{27}$: –7.8 (*c* 0.65, $CHCl_3$); HPLC analysis: 95% *ee* (Chiralcel AD-H, 5:95 ^{i}PrOH/hexanes, 1 mL/min, 254 nm), R_t (major) = 5.7 min, R_t (minor) = 6.4 min. IR (KBr thin film, cm^{-1}): ν 3396, 3030, 2987, 2904, 2841, 1738, 1614, 1555, 1435, 1361, 750.

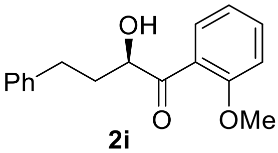

2i

(*R*)-2-Hydroxy-1-(2-methoxyphenyl)-4-phenylbutan-1-one (2i): Purified by flash chromatography on silica gel (petroleum ether/ethyl acetate, v:v = 5:1), yellow oil, 37.3 mg, 69% yield. ^{1}H NMR (400 MHz, $CDCl_3$) δ 7.80–7.78 (m, 1H), 7.52–7.48 (m, 1H), 7.28–7.15 (m, 5H), 7.04–7.00 (m, 1H), 6.92 (d, *J* = 8.4 Hz, 1H), 5.09 (ddd, *J* = 8.9, 5.7, 2.9 Hz, 1H), 3.93 (d, *J* = 5.8 Hz, 1H), 3.65 (s, 3H), 2.88–2.72 (m, 2H), 2.09–1.99 (m, 1H), 1.71–1.61 (m, 1H); ^{13}C NMR (101 MHz, $CDCl_3$) δ 203.4, 158.7, 141.7, 134.8, 131.2, 128.8, 128.4, 125.9, 124.1, 121.0, 111.6, 75.8, 55.3, 36.5, 31.8. HRMS (ESI-Quadrupole-Orbitrap) m/z: $[M + Na]^+$ Calcd for $C_{17}H_{18}O_3Na$ 293.1148, found 293.1146. $[\alpha]_D^{27}$: +29.8 (*c* 1.57, $CHCl_3$); HPLC analysis: 91% *ee* (Chiralcel AS-H, 15:85 ^{i}PrOH/hexanes, 1 mL/min, 254 nm), R_t (major) = 7.2 min, R_t (minor) = 8.1 min. IR (KBr thin film, cm^{-1}): ν 3403, 3065, 2998, 2969, 1731, 1564, 1507, 1344, 1339, 861, 781.

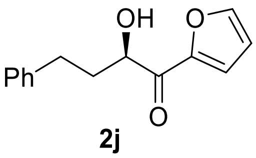

2j

(*R*)-1-(Furan-2-yl)-2-hydroxy-4-phenylbutan-1-one (2j): Purified by flash chromatography on silica gel (petroleum ether/ethyl acetate, v:v = 5:1), yellow solid, mp 70–78 ºC, 21.6 mg, 47% yield. ^{1}H NMR (600 MHz, $CDCl_3$) δ 7.61 (dd, *J* = 1.6, 0.6 Hz, 1H), 7.28–7.25 (m, 2H), 7.19–7.17 (m, 4H), 6.55 (dd, *J* = 3.7, 1.7 Hz, 1H), 4.80 (ddd, *J* = 8.5, 6.6, 3.2 Hz, 1H), 3.54 (d, *J* = 6.6 Hz, 1H), 2.85–2.78 (m, 2H), 2.25–2.19 (m, 1H), 1.94–1.88 (m, 1H); ^{13}C NMR (151 MHz, $CDCl_3$) δ 190.4, 150.2, 147.3, 141.3, 128.7, 128.5, 126.2, 119.2, 112.7, 72.8, 37.5, 31.4. HRMS (ESI-Quadrupole-Orbitrap) m/z: $[M + H]^+$ Calcd for $C_{14}H_{15}O_3$ 231.1016, found 231.1015. $[\alpha]_D^{27}$: +16.1 (*c* 0.61, $CHCl_3$); HPLC analysis: 93% *ee* (Chiralcel OJ-H, 10:90 ^{i}PrOH/hexanes, 1 mL/min, 254 nm), R_t (major) = 7.5 min, R_t (minor) = 8.8 min. IR (KBr thin film, cm^{-1}): ν 3361, 3027, 2924, 2881, 1731, 1591, 1568, 1558, 1463, 1379, 858.

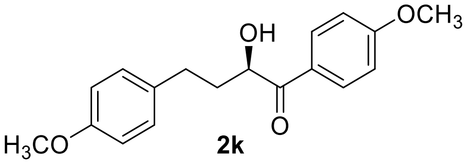

2k

(*R*)-2-Hydroxy-1,4-bis(4-methoxyphenyl)butan-1-one (2k): Purified by flash chromatography on silica gel (petroleum ether/ethyl acetate, v:v = 7:1), yellow solid, mp 68–75 ºC, 52.9 mg, 88% yield. ^{1}H NMR (600 MHz, $CDCl_3$) δ 7.75 (d, *J* = 9.0 Hz, 2H), 7.09 (d, *J* = 8.7 Hz, 2H), 6.90 (d, *J* = 9.0 Hz, 2H), 6.82 (d, *J* = 8.7 Hz, 2H), 4.95 (ddd, *J* = 8.7, 6.5, 2.9 Hz, 1H), 3.86 (s, 3H), 3.80 (d, *J* = 6.5 Hz, 1H), 3.77 (s, 3H), 2.81–2.68 (m, 2H), 2.12–2.06 (m, 1H), 1.80–1.74 (m, 1H); ^{13}C NMR (151 MHz, $CDCl_3$) δ 200.4, 164.2, 158.0, 133.3, 131.0, 129.7, 126.3, 114.1, 113.9, 71.8, 55.7, 55.4, 38.4, 30.5. HRMS (ESI-Quadrupole-Orbitrap) m/z: $[M + Na]^+$ Calcd for $C_{18}H_{20}O_4Na$ 323.1254, found 323.1252. $[\alpha]_D^{27}$: –15.8 (*c* 2.51, $CHCl_3$); HPLC analysis: >99% *ee* (Chiralcel AS-H, 20:80 ^{i}PrOH/hexanes, 1 mL/min, 254 nm), R_t (major) = 10.7 min, R_t (minor) = 6.7 min. IR (KBr thin film, cm^{-1}): ν 3391, 3031, 3014, 2966, 1703, 1553, 1498, 1434, 1361, 580. 550.

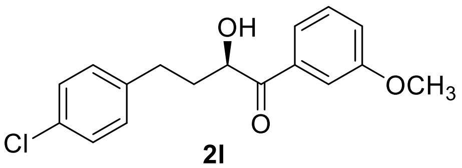

2l

(*R*)-4-(4-Chlorophenyl)-2-hydroxy-1-(3-methoxyphenyl)butan-1-one (2l): Purified by flash chromatography on silica gel (petroleum ether/ethyl acetate, v:v = 6:1), white solid, mp 109–112 ºC, 40.7 mg, 67% yield. ^{1}H NMR (600 MHz, $CDCl_3$) δ 7.36–7.26 (m, 3H), 7.23–7.18 (m, 2H), 7.14–7.05 (m, 3H), 4.97 (ddd, *J* = 8.6, 6.3, 3.1 Hz, 1H), 3.81 (s, 3H), 3.74 (d, *J* = 6.3 Hz, 1H), 2.83–2.78 (m, 1H), 2.75–2.70 (m, 1H), 2.14–2.08 (m, 1H), 1.82–1.76 (m, 1H); ^{13}C NMR (151 MHz, $CDCl_3$) δ 201.8, 160.0, 139.5, 134.7, 132.0, 130.1, 130.0, 128.6, 121.0, 120.6, 112.8, 72.1, 55.5, 37.6, 30.6. HRMS (ESI-Quadrupole-Orbitrap) m/z: $[M + Na]^+$ Calcd for $C_{17}H_{17}O_3ClNa$ 327.0758, found 327.0757. $[\alpha]_D^{27}$: –17.8 (*c* 0.84, $CHCl_3$); HPLC analysis: 98% *ee* (Chiralcel AD-H, 10:90 ^{i}PrOH/hexanes, 1 mL/min, 254 nm), R_t (major) = 7.0 min, R_t (minor) = 6.4 min. IR (KBr thin film, cm^{-1}): ν 3369, 3081, 2964, 2921, 1748, 1554, 1493, 1455, 1306, 930, 859.

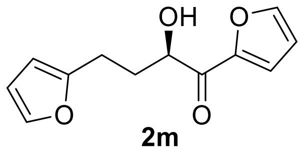

2m

(*R*)-1,4-Di(furan-2-yl)-2-hydroxybutan-1-one (2m): Purified by flash chromatography on silica gel (petroleum ether/ethyl acetate, v:v = 5:1), white solid, mp 103–112 ºC, 35.2 mg, 80% yield. ^{1}H NMR (600 MHz, $CDCl_3$) δ 7.65–7.63 (m, 1H), 7.31–7.30 (m, 1H), 7.27–7.24 (m, 1H), 6.59 (dd, *J* = 3.5, 1.6 Hz, 1H), 6.29 (dd, *J* = 3.0, 1.8 Hz, 1H), 6.05 (dd, *J* = 3.1, 0.6 Hz, 1H), 4.81 (ddd, *J* = 8.6, 6.6, 3.2 Hz, 1H), 3.54 (d, *J* = 6.5 Hz, 1H), 2.93–2.79 (m, 2H), 2.34–2.26 (m, 1H), 1.95–1.86 (m, 1H); ^{13}C NMR (151 MHz, $CDCl_3$) δ 188.3, 153.0, 148.2, 145.5, 139.3, 117.4, 110.8, 108.4, 103.9, 70.8, 32.3, 21.9. HRMS (ESI-Quadrupole-Orbitrap) m/z: $[M + Na]^+$ Calcd for $C_{12}H_{12}O_4Na$ 243.0628, found 243.0629. $[\alpha]_D^{27}$: +4.5 (*c* 0.94, $CHCl_3$); HPLC analysis: 94% *ee* (Chiralcel AS-H, 10:90 ^{i}PrOH/hexanes, 1 mL/min, 254 nm), R_t (major) = 9.2 min, R_t (minor) = 6.2 min. IR (KBr thin film, $cm^{-1)}$: ν 3409, 3160, 3133, 3116, 1726, 1556, 1339, 1141, 915, 897, 749.

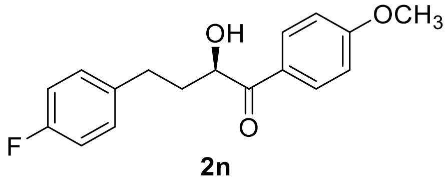

2n

(*R*)-4-(4-Fluorophenyl)-2-hydroxy-1-(4-methoxyphenyl)butan-1-one (2n): Purified by flash chromatography on silica gel (petroleum ether/ethyl acetate, v:v = 5:1), yellow solid, mp 97–103 ºC, 46.7 mg, 81% yield. ^{1}H NMR (600 MHz, $CDCl_3$) δ 7.76–7.45 (m, 2H), 7.13–7.10 (m, 2H), 6.96–6.90 (m, 4H), 4.95 (ddd, *J* = 18.1, 6.4, 3.0 Hz, 1H), 3.86 (s, 3H), 3.83 (d, *J* = 6.4 Hz, 1H), 2.84–2.78 (m, 1H), 2.75–2.70 (m,

1H), 2.13–2.06 (m, 1H), 1.82–1.75 (m, 1H); ^{13}C NMR (151 MHz, $CDCl_3$) δ 200.2, 164.3, 161.5 (d, *J* = 256.7 Hz), 136.9 (d, *J* = 2.6 Hz), 130.9, 130.1 (d, *J* = 9.1 Hz), 126.2, 115.3 (d, *J* = 21.1 Hz), 114.2, 71.7, 55.7, 38.2, 30.5. HRMS (ESI-Quadrupole-Orbitrap) m/z: $[M + Na]^+$ Calcd for $C_{17}H_{17}O_3FNa$ 311.1054, found 311.1052. $[\alpha]_D^{27}$: –9.9 (*c* 0.94, $CHCl_3$); HPLC analysis: 99% *ee* (Chiralcel AS-H, 20:80 ^{i}PrOH/hexanes, 1 mL/min, 254 nm), R_t (major) = 7.1 min, R_t (minor) = 4.8 min. IR (KBr thin film, cm^{-1}): ν 3408, 3028, 2964, 2932, 2825, 1708, 1608, 1556, 1508, 1447, 753.

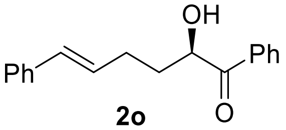

2o

(*S*,*E*)-1-Hydroxy-1,6-diphenylhex-5-en-2-one (2o): Purified by flash chromatography on silica gel (petroleum ether/ethyl acetate, v:v = 15:1), yellow oil, 38.9 mg, 73% yield. ^{1}H NMR (600 MHz, $CDCl_3$) δ 7.90–7.88 (m, 2H), 7.62–7.58 (m, 1H), 7.50–7.47 (m, 2H), 7.31–7.25 (m, 4H), 7.20–7.17 (m, 1H), 6.41 (d, *J* = 15.8 Hz, 1H), 6.19–6.13 (m, 1H), 5.12 (d, *J* = 6.5 Hz, 1H), 3.75 (d, *J* = 6.5 Hz, 1H), 2.48–2.41 (m, 1H), 2.38–2.31 (m, 1H), 2.05–2.00 (m, 1H), 1.73–1.66 (m, 1H); ^{13}C NMR (151 MHz, $CDCl_3$) δ 202.1, 137.5, 134.1, 133.7, 131.3, 129.1, 129.0, 128.7, 128.6, 127.2, 126.1, 72.5, 35.7, 28.5. HRMS (ESI-Quadrupole-Orbitrap) m/z: $[M + Na]^+$ Calcd for $C_{18}H_{18}O_2Na$ 289.1199, found 289.1195. $[\alpha]_D^{27}$: –33.2 (*c* 0.91, $CHCl_3$); HPLC analysis: 98% *ee* (Chiralcel IC, 5:95 ^{i}PrOH/hexanes, 1 mL/min, 254 nm), R_t (major) = 7.5 min, R_t (minor) = 8.7 min. IR (KBr thin film, cm^{-1}): ν 3423, 3030, 2991, 2882, 2871, 1725, 1569, 1505, 1359, 1276, 904.

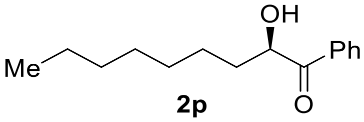

2p

(*R*)-2-Hydroxy-1-phenylnonan-1-one (2p): Purified by flash chromatography on silica gel (petroleum ether/ethyl acetate, v:v = 15:1), yellow oil, 30.1 mg, 71% yield. ^{1}H NMR (600 MHz, $CDCl_3$) δ 7.91–7.89 (m, 2H), 7.62–7.59 (m, 1H), 7.51–7.48 (m, 2H), 5.06 (ddd, *J* = 7.7, 6.4, 3.2 Hz, 1H), 3.68 (d, *J* = 6.4 Hz, 1H), 1.87–1.80 (m, 1H), 1.55–1.45 (m, 2H), 1.40–1.17 (m, 9H), 0.84 (t, *J* = 7.0 Hz, 3H); ^{13}C NMR (151 MHz, $CDCl_3$) δ 202.3, 134.0, 133.8, 129.0, 128.6, 73.2, 36.0, 31.8, 29.4, 29.2, 25.0, 22.7, 14.2. HRMS (ESI-Quadrupole-Orbitrap) m/z: $[M + Na]^+$ Calcd for $C_{15}H_{22}O_2Na$ 257.1512, found 257.1508. $[\alpha]_D^{27}$: –10.7 (*c* 0.46, $CHCl_3$); HPLC analysis: 96% *ee* (Chiralcel AS-H, 2:98 ^{i}PrOH/hexanes, 1 mL/min, 254 nm), R_t (major) = 5.9 min, R_t (minor) = 4.0 min. IR (KBr thin film, cm^{-1}): ν 3407, 2970, 2940, 2867, 2856, 1716, 1597, 1557, 1276, 764, 753.

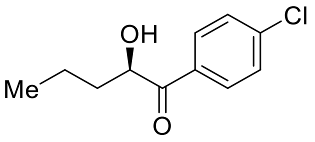

2q

(*R*)-1-(4-Chlorophenyl)-2-hydroxypentan-1-one (2q): Purified by flash chromatography on silica gel (petroleum ether/ethyl acetate, v:v = 10:1), colorless oil, 27.2 mg, 64% yield. ^{1}H NMR (600 MHz, $CDCl_3$) δ 7.85–7.83 (m, 2H), 7.48–7.46 (m, 2H), 5.02 (ddd, *J* = 7.4, 6.5, 3.5 Hz, 1H), 3.61 (d, *J* = 6.5 Hz, 1H), 1.81–1.76 (m, 1H), 1.54–1.36 (m, 3H), 0.91 (t, *J* = 7.1 Hz, 3H); ^{13}C NMR (151 MHz, $CDCl_3$) δ 201.2, 140.5, 132.1, 130.0, 129.4, 73.0, 38.0, 18.3, 13.9. HRMS (ESI-Quadrupole-Orbitrap) m/z: $[M + Na]^+$ Calcd for $C_{11}H_{13}O_2ClNa$ 235.0496, found 235.0496. $[\alpha]_D^{27}$: –18.1 (*c* 0.31, $CHCl_3$); HPLC analysis: 95% *ee* (Chiralcel IC, 5:95 ^{i}PrOH/hexanes, 1 mL/min, 254 nm), R_t (major) = 5.3 min, R_t (minor) = 6.6 min. IR (KBr thin film, cm^{-1}): ν 3374, 2957, 2950, 1734, 1559, 1366, 1276, 904, 765, 679, 562.

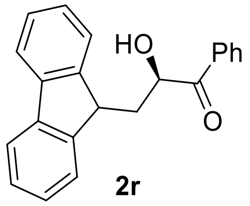

2r

(*R*)-3-(9*H*-Fluoren-9-yl)-2-hydroxy-1-phenylpropan-1-one (2r): Purified by flash chromatography on silica gel (petroleum ether/ethyl acetate, v:v = 8:1), yellow oil, 42.1 mg, 67% yield. ^{1}H NMR (600 MHz, $CDCl_3$) δ 7.85–7.80 (m, 2H), 7.74–7.71 (m, 3H), 7.53–7.49 (m, 1H), 7.44–7.42 (m, 1H), 7.40–7.31 (m, 5H), 7.27–7.23 (m, 1H), 5.44 (ddd, *J* = 11.2, 6.4, 2.8 Hz, 1H), 4.37 (dd, *J* = 10.6, 4.1 Hz, 1H), 3.91 (d, *J* = 6.4 Hz, 1H), 2.25–2.20 (m, 1H), 1.89–1.84 (m, 1H); ^{13}C NMR (151 MHz, $CDCl_3$) δ 201.9, 147.4, 146.5, 141.2, 140.7, 134.1, 133.2, 128.9, 128.7, 127.5, 127.3, 127.1, 125.7, 124.3, 120.3, 120.0, 71.6, 44.2, 40.6. HRMS (ESI-Quadrupole-Orbitrap) m/z: $[M + Na]^+$ Calcd for $C_{22}H_{18}O_2Na$ 337.1199, found 337.1196. $[\alpha]_D^{27}$: –45.7 (*c* 0.74, $CHCl_3$); HPLC analysis: 97% *ee* (Chiralcel AS-H, 10:90 ^{i}PrOH/hexanes, 1 mL/min, 254 nm), R_t (major) = 6.2 min, R_t (minor) =5.2 min. IR (KBr thin film, cm^{-1}): ν 3392, 3041, 3011, 2948, 2839, 1713, 1597, 1553, 970, 765, 751.

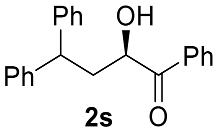

2s

(*R*)-2-Hydroxy-1,4,4-triphenylbutan-1-one (2s): Purified by flash chromatography on silica gel (petroleum ether/ethyl acetate, v:v = 10:1), white solid, mp 109–115 ºC, 47.4 mg, 75% yield. ^{1}H NMR (600 MHz, $CDCl_3$) δ 7.62–7.56 (m, 3H), 7.42–7.36 (m, 6H), 7.29–7.26 (m, 1H), 7.22–7.17 (m, 4H), 7.14–7.11 (m, 1H), 4.80 (ddd, *J* = 10.6, 6.7, 1.9 Hz, 1H), 4.47 (dd, *J* = 11.8, 3.6 Hz, 1H), 3.67 (d, *J* = 6.7 Hz, 1H), 2.55–2.50 (m, 1H), 2.05–2.00 (m, 1H); ^{13}C NMR (151 MHz, $CDCl_3$) δ 202.0, 144.3, 143.3, 134.1, 133.4, 128.9, 128.8, 128.6, 128.5, 127.7, 127.0, 126.4, 71.2, 47.4, 42.5. HRMS (ESI-Quadrupole-Orbitrap) m/z: $[M + Na]^+$ Calcd for $C_{22}H_{20}O_2Na$ 339.1356, found 339.1353. $[\alpha]_D^{27}$: –8.6 (*c* 0.39, $CHCl_3$); HPLC analysis: >99% *ee* (Chiralcel IC, 10:90 ^{i}PrOH/hexanes, 1 mL/min, 254 nm), R_t (major) = 4.7 min, R_t (minor) = 6.0 min. IR (KBr thin film, cm^{-1}): ν 3391, 3033, 3012, 2903, 2844, 1729, 1601, 1556, 1359, 913, 752.

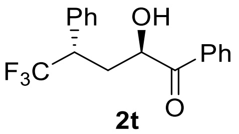

2t

(2*R*,4*S*)-5,5,5-Trifluoro-2-hydroxy-1,4-diphenylpentan-1-one (2t): Purified by flash chromatography on silica gel (petroleum ether/ethyl acetate, v:v = 10:1), white solid, mp 138–140 ºC, 42.7 mg, 69% yield and 5:1 dr (*anti*:*syn*). *anti*–diastereomer: ^{1}H NMR (600 MHz, $CDCl_3$) δ 7.62–7.59 (m, 3H), 7.49–7.42 (m, 7H), 4.56 (ddd, *J* = 11.6, 6.7, 1.7 Hz, 1H), 3.87–3.79 (m, 1H), 3.65 (d, *J* = 6.7 Hz, 1H), 2.33–2.28 (m, 1H), 1.95–1.90 (m, 1H); ^{13}C NMR (151 MHz, $CDCl_3$) δ 201.1, 134.4, 133.6, 132.9, 129.5, 129.2, 129.1, 128.9, 128.6, 126.9 (d, *J* = 271.8 Hz), 69.6, 47.1 (q, *J* = 30.2 Hz), 36.0. HRMS (ESI-Quadrupole-Orbitrap) m/z: $[M + Na]^+$ Calcd for $C_{17}H_{15}O_2F_3Na$ 331.0916, found 331.0913. $[\alpha]_D^{27}$: –22.8 (*c* 0.72, $CHCl_3$); HPLC analysis: 90% *ee* (Chiralcel AD-H, 5:95 ^{i}PrOH/hexanes, 1 mL/min, 254 nm), R_t (major) = 4.0 min, R_t (minor) = 4.8 min. IR (KBr thin film, cm^{-1}): ν 3394, 3048, 2930, 2874, 1748, 1590, 1474, 1325, 1146, 797, 723.

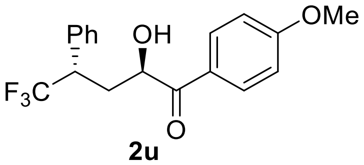

2u

(2*R*,4*S*)-5,5,5-Trifluoro-2-hydroxy-1-(4-methoxyphenyl)-4-phenylpentan-1-one (2u): Purified by flash chromatography on silica gel (petroleum ether/ethyl acetate, v:v = 10:1), white solid, mp 90–93 ºC, 23.7 mg, 35% yield and 2:1 dr (*anti*:*syn*). *anti*–diastereomer: ^{1}H NMR (400 MHz, $CDCl_3$) δ 7.62 (d, *J* = 8.8 Hz, 2H), 7.51–7.42 (m, 5H), 6.91 (d, *J* = 8.8 Hz, 2H), 4.51 (ddd, *J* = 7.9, 6.9, 0.9 Hz, 1H), 3.88 (s, 3H), 3.89–

3.78 (m, 1H), 3.71 (d, *J* = 6.9 Hz, 1H), 2.34–2.27 (m, 1H), 1.97–1.90 (m, 1H); ^{13}C NMR (151 MHz, $CDCl_3$) δ 199.3, 164.5, 133.7, 131.6, 131.0, 129.5, 129.2, 128.9, 127.9, 127.0 (d, *J* = 286.9 Hz), 125.6, 114.3, 69.1, 55.7, 53.5, 47.1 (q, *J* = 30.2 Hz), 36.4. HRMS (ESI-Quadrupole-Orbitrap) m/z: $[M + H]^+$ Calcd for $C_{18}H_{18}O_3F_3$ 339.1203, found 339.1202. $[\alpha]_D^{27}$: –10.3 (*c* 0.91, $CHCl_3$); HPLC analysis: 94% *ee* (Chiralcel AD-H, 2:98 ^{i}PrOH/hexanes, 1 mL/min, 254 nm), R_t (major) = 8.7 min, R_t (minor) = 10.9 min. IR (KBr thin film, cm^{-1}): ν 3364, 3027, 2933, 2810, 1745, 1558, 1361, 1275, 1260, 764, 750.

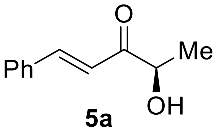

5a

(*S*,*E*)-4-Hydroxy-1-phenylpent-1-en-3-one (5a): Purified by flash chromatography on silica gel (petroleum ether/ethyl acetate, v:v = 5:1), yellow oil, 31.3 mg, 88% yield. ^1H NMR (600 MHz, $CDCl_3$) δ 7.76 (d, *J* = 16.0 Hz, 1H), 7.59–7.57 (m, 2H), 7.44–7.39 (m, 3H), 6.83 (d, *J* = 16.0 Hz, 1H), 4.55 (qd, *J* = 7.1, 5.2 Hz, 1H), 3.70 (d, *J* = 5.2 Hz, 1H), 1.45 (d, *J* = 7.1 Hz, 3H); ^{13}C NMR (151 MHz, $CDCl_3$) δ 201.1, 145.0, 134.1, 131.2, 129.2, 128.7, 120.4, 71.9, 20.6. HRMS (ESI-Quadrupole-Orbitrap) m/z: $[M + H]^+$ Calcd for $C_{11}H_{13}O_2$ 177.0910, found 177.0908. $[\alpha]_D^{27}$: –4.9 (*c* 1.03, $CHCl_3$); HPLC analysis: 96% *ee* (Chiralcel OJ-H, 10:90 ^{i}PrOH/hexanes, 1 mL/min, 254 nm), R_t (major) = 7.5 min, R_t (minor) = 6.9 min. IR (KBr thin film, cm^{-1}): ν 3416, 3021, 2969, 1744, 1598, 1556, 1506, 1449, 1246, 976, 726.

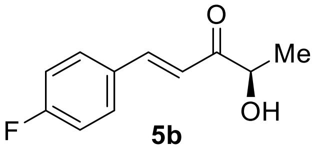

5b

(*S*,*E*)-1-(4-Fluorophenyl)-4-hydroxypent-1-en-3-one (5b): Purified by flash chromatography on silica gel (petroleum ether/ethyl acetate, v:v = 5:1), yellow oil, 38.8 mg, 89% yield. ^1H NMR (400 MHz, $CDCl_3$) δ 7.74 (d, *J* = 16.0 Hz, 1H), 7.61–7.57 (m, 2H), 7.13–7.09 (m, 2H), 6.77 (d, *J* = 16.0 Hz, 1H), 4.55 (qd, *J* = 7.1, 5.1 Hz, 1H), 3.69 (d, *J* = 5.1 Hz, 1H), 1.46 (d, *J* = 7.1 Hz, 3H); ^{13}C NMR (101 MHz, $CDCl_3$) δ 200.9, 164.4 (d, *J* = 252.6 Hz), 143.6, 130.6 (d, *J* = 9.1 Hz), 130.3, 119.9 (d, *J* = 2.0 Hz), 116.3 (d, *J* = 22.1 Hz), 71.9, 20.5. HRMS (ESI-Quadrupole-Orbitrap) m/z: $[M + H]^+$ Calcd for $C_{11}H_{12}O_2F$ 195.0816, found 195.0815. $[\alpha]_D^{27}$: –29.1 (*c* 1.53, $CHCl_3$); HPLC analysis: 94% *ee* (Chiralcel AD-H, 5:95 ^{i}PrOH/hexanes, 1 mL/min, 254 nm), R_t (major) = 9.0 min, R_t (minor) = 9.7 min. IR (KBr thin film, cm^{-1}): ν 3371, 3034, 2894, 1752, 1656, 1544, 1504, 1341, 1217, 934, 823.

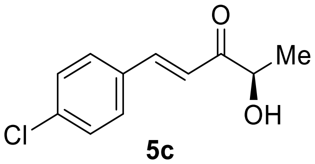

5c

(*S*,*E*)-1-(4-Chlorophenyl)-4-hydroxypent-1-en-3-one (5c): Purified by flash chromatography on silica gel (petroleum ether/ethyl acetate, v:v = 5:1), yellow oil, 37.8 mg, 90% yield. ^{1}H NMR (600 MHz, $CDCl_3$) δ 7.70 (d, *J* = 16.0 Hz, 1H), 7.52–7.50 (m, 2H), 7.39–7.37 (m, 2H), 6.80 (d, *J* = 16.0 Hz, 1H), 4.53 (qd, *J* = 7.1, 5.2 Hz, 1H), 3.65 (d, *J* = 5.2 Hz, 1H), 1.44 (d, *J* = 7.1 Hz, 3H); ^{13}C NMR (151 MHz, $CDCl_3$) ^{13}C NMR (151 MHz, $CDCl_3$) δ 200.9, 143.5, 137.2, 132.6, 129.8, 129.5, 120.7, 72.0, 20.5. HRMS (ESI-Quadrupole-Orbitrap) m/z: $[M + H]^+$ Calcd for $C_{11}H_{12}O_2Cl$ 211.0520, found 211.0518. $[\alpha]_D^{27}$: –10.4 (*c* 0.82, $CHCl_3$); HPLC analysis: >99% *ee* (Chiralcel AD-H, 3:97 ^{i}PrOH/hexanes, 1 mL/min, 254 nm), R_t (major) = 17.0 min, R_t (minor) = 19.1 min. IR (KBr thin film, cm^{-1}): ν 3406, 3037, 2969, 2860, 1735, 1564, 1506, 1359, 1278, 926, 750.

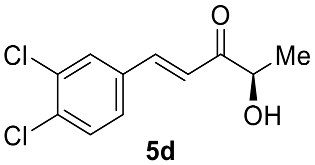

5d

(*S*,*E*)-1-(3,4-Dichlorophenyl)-4-hydroxypent-1-en-3-one (5d): Purified by flash chromatography on silica gel (petroleum ether/ethyl acetate, v:v = 7:1), yellow solid, mp 116–120 ºC, 39.5 mg, 81% yield. ^{1}H NMR (600 MHz, $CDCl_3$) δ 7.66–7.62 (m, 2H), 7.48 (d, *J* = 8.3 Hz, 1H), 7.39 (dd, *J* = 8.3, 2.0 Hz, 1H), 6.81 (d, *J* = 15.9 Hz, 1H), 4.52 (qd, *J* = 7.1, 5.1 Hz, 1H), 3.60 (d, *J* = 5.1 Hz, 1H), 1.44 (d, *J* = 7.1 Hz, 3H); ^{13}C NMR (101 MHz, $CDCl_3$) δ 200.7, 142.0, 135.1, 134.1, 133.5, 131.1, 130.0, 127.6, 121.7, 72.1, 20.3. HRMS (ESI-Quadrupole-Orbitrap) m/z: $[M + Na]^+$ Calcd for $C_{11}H_{10}O_2Cl_2Na$ 266.9950, found 266.9948. $[\alpha]_D^{27}$: –5.7 (*c* 0.98, $CHCl_3$); HPLC analysis: 90% *ee* (Chiralcel AD-H, 10:90 ^{i}PrOH/hexanes, 1 mL/min, 254 nm), R_t (major) = 5.8 min, R_t (minor) = 6.8 min. IR (KBr thin film, cm^{-1}): ν 3385, 3038, 2981, 2852, 1728, 1597, 1556, 1505, 1360, 1243, 936.

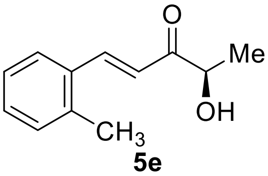

5e

(*S*,*E*)-4-Hydroxy-1-(o-tolyl)pent-1-en-3-one (5e): Purified by flash chromatography on silica gel (petroleum ether/ethyl acetate, v:v = 5:1), yellow oil, 33.8 mg, 89% yield. ^{1}H NMR (600 MHz, $CDCl_3$) δ 8.07 (d, *J* = 15.8 Hz, 1H), 7.60 (d, *J* = 7.8 Hz, 1H), 7.32–7.29 (m, 1H), 7.23–7.21 (m, 2H), 6.76 (d, *J* = 15.8 Hz, 1H), 4.54 (qd, *J* = 7.1, 5.1 Hz, 1H), 3.73 (d, *J* = 5.1 Hz, 1H), 2.45, (s, 3H), 1.45 (d, *J* = 7.1 Hz, 3H); ^{13}C NMR (151 MHz, $CDCl_3$) δ 201.1, 142.5, 138.7, 133.1, 131.1, 130.9, 126.54, 126.50, 121.3, 72.0, 20.6, 19.9. HRMS (ESI-Quadrupole-Orbitrap) m/z: $[M + Na]^+$ Calcd for $C_{12}H_{14}O_2Na$ 213.0886, found 213.0885. $[\alpha]_D^{27}$: –17.8 (*c* 0.48, $CHCl_3$); HPLC analysis: 98% ee (Chiralcel OJ-H, 5:95 ^{i}PrOH/hexanes, 1 mL/min, 254 nm), R_t (major) = 7.5 min, R_t (minor) = 7.2 min. IR (KBr thin film, cm^{-1}): ν 3386, 3083, 3036, 2972, 2869, 2857, 1698, 1612, 1540, 1277, 752.

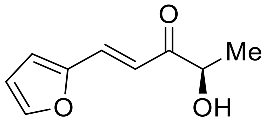

5f

(*S*,*E*)-1-(Furan-2-yl)-4-hydroxypent-1-en-3-one (5f): Purified by flash chromatography on silica gel (petroleum ether/ethyl acetate, v:v = 5:1), yellow oil, 30.1 mg, 92% yield. ^{1}H NMR (600 MHz, $CDCl_3$) δ 7.51–7.48 (m, 2H), 6.72–6.68 (m, 2H), 6.50 (dd, *J* = 3.4, 1.7 Hz, 1H), 4.47 (qd, *J* = 7.1, 5.0 Hz, 1H), 3.72 (d, *J* = 5.0 Hz, 1H), 1.42 (d, *J* = 7.1 Hz, 1H); ^{13}C NMR (151 MHz, $CDCl_3$) δ 200.8, 150.9, 145.6, 130.6, 117.6, 117.3, 112.9, 72.1, 20.5. HRMS (ESI-Quadrupole-Orbitrap) m/z: $[M + Na]^+$ Calcd for $C_9H_{10}O_3Na$ 189.0522, found 189.0521. $[\alpha]_D^{27}$: –23.8 (*c* 0.16, $CHCl_3$); HPLC analysis: 99% *ee* (Chiralcel AD-H, 10:90 ^{i}PrOH/hexanes, 1 mL/min, 254 nm), R_t (major) = 5.8 min, R_t (minor) = 5.5 min. IR (KBr thin film, cm^{-1}): ν 3383, 3023, 2958, 2861, 1719, 1608, 1540, 1498, 1359, 907, 764.

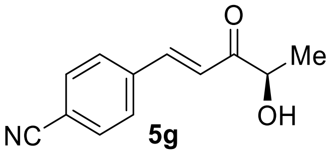

5g

(*S*,*E*)-4-(4-Hydroxy-3-oxopent-1-en-1-yl)benzonitrile (5g): Purified by flash chromatography on silica gel (petroleum ether/ethyl acetate, v:v = 3:1), yellow oil, 29.4 mg, 73% yield. ^{1}H NMR (400 MHz, $CDCl_3$) δ 7.74–7.65 (m, 5H), 6.91 (d, *J* = 10.6 Hz, 1H), 4.54 (dq, *J* = 7.1, 4.9 Hz, 1H), 3.55 (d, *J* = 4.9 Hz, 1H), 1.46 (d, *J* = 7.1 Hz, 3H); ^{13}C NMR (151 MHz, $CDCl_3$) δ 200.7, 142.3, 138.4, 132.8, 128.9, 123.3, 118.3, 114.2, 72.3, 20.3. HRMS (ESI-Quadrupole-Orbitrap) m/z: $[M + H]^+$ Calcd for $C_{12}H_{12}O_2N$ 202.0863, found 202.0861. $[\alpha]_D^{27}$: –40.8 (*c* 0.45, $CHCl_3$); HPLC analysis: 93% *ee* (Chiralcel IC, 15:85 ^{i}PrOH/hexanes, 1 mL/min, 254 nm), R_t (major) = 13.6

min, R_t (minor) = 19.9 min. IR (KBr thin film, cm^{-1}): ν 3404, 3031, 2973, 2865, 1722, 1605, 1528, 1360, 1276, 814, 752.

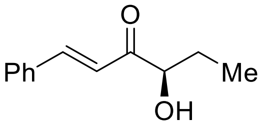

5h

(*S,E*)-4-Hydroxy-1-phenylhex-1-en-3-one (5h): Purified by flash chromatography on silica gel (petroleum ether/ethyl acetate, v:v = 5:1), yellow oil, 30.0 mg, 79% yield. ^1H NMR (600 MHz, $CDCl_3$) δ 7.75 (d, *J* = 16.0 Hz, 1H), 7.59–7.57 (m, 2H), 7.43–7.39 (m, 3H), 6.85 (d, *J* = 16.0 Hz, 1H), 4.45 (ddd, *J* = 6.7, 5.3, 4.1 Hz, 1H), 3.64 (d, *J* = 5.3 Hz, 1H), 2.00–1.92 (m, 1H), 1.71–1.63 (m, 1H), 0.96 (t, *J* = 7.4 Hz, 3H); ^{13}C NMR (151 MHz, $CDCl_3$) δ 200.8, 144.7, 134.1, 131.2, 129.1, 128.7, 120.6, 76.6, 27.4, 8.9. HRMS (ESI-Quadrupole-Orbitrap) m/z: $[M + Na]^+$ Calcd for $C_{12}H_{14}O_2Na$ 213.0886, found 213.0885. $[\alpha]_D^{27}$: –5.1 (*c* 1.13, $CHCl_3$); HPLC analysis: 90% *ee* (Chiralcel AD-H, 10:90 ^{i}PrOH/hexanes, 1 mL/min, 254 nm), R_t (major) = 5.4 min, R_t (minor) = 6.0 min. IR (KBr thin film, cm^{-1}): ν 3370, 3040, 2963, 2885, 1728, 1603, 1557, 1505, 1449, 1359, 921.

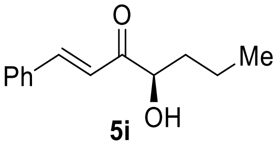

5i

(*S,E*)-4-Hydroxy-1-phenylhept-1-en-3-one (5i): Purified by flash chromatography on silica gel (petroleum ether/ethyl acetate, v:v = 5:1), yellow oil, 34.7 mg, 85% yield. ^1H NMR (600 MHz, $CDCl_3$) δ 7.75 (d, *J* = 16.0 Hz, 1H), 7.60–7.57 (m, 2H), 7.42–7.39 (m, 3H), 6.84 (d, *J* = 16.0 Hz, 1H), 4.47 (ddd, *J* = 7.7, 5.3, 3.5 Hz, 1H), 3.62 (d, *J* = 5.4 Hz, 1H), 1.88–1.82 (m, 1H), 1.59–1.39 (m, 3H), 0.95 (t, *J* = 7.3 Hz, 3H); ^{13}C NMR (151 MHz, $CDCl_3$) δ 200.9, 144.7, 134.1, 131.2, 129.1, 128.7, 120.6, 75.7, 36.5, 18.3, 14.1. HRMS (ESI-Quadrupole-Orbitrap) m/z: $[M + H]^+$ Calcd for $C_{12}H_{17}O_2$ 205.1223, found 205.1219. $[\alpha]_D^{27}$: –14.4 (*c* 0.84, $CHCl_3$); HPLC analysis: 90% *ee* (Chiralcel AS-H, 10:90 ^{i}PrOH/hexanes, 1 mL/min, 254 nm), R_t (major) = 5.0 min, R_t (minor) = 4.6 min. IR (KBr thin film, cm^{-1}): ν 3408, 3029, 2966, 2876, 1732, 1594, 1565, 1494, 1276, 965, 751.

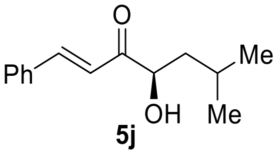

5j

(*S*,*E*)-4-Hydroxy-6-methyl-1-phenylhept-1-en-3-one (5j): Purified by flash chromatography on silica gel (petroleum ether/ethyl acetate, v:v = 10:1), yellow oil, 36.6 mg, 84% yield. ^{1}H NMR (600 MHz, $CDCl_3$) δ 7.74 (d, *J* = 16.0 Hz, 1H), 7.59–7.57 (m, 2H), 7.44–7.39 (m, 3H), 6.81 (d, *J* = 16.0 Hz, 1H), 4.47 (ddd, *J* = 10.1, 5.5, 2.9 Hz, 1H), 3.56 (d, *J* = 5.5 Hz, 1H), 2.02–1.97 (m, 1H), 1.61–1.56 (m, 1H), 1.46–1.41 (m, 1H), 1.04 (d, *J* = 6.6 Hz, 3H), 0.96 (d, *J* = 6.6 Hz, 3H); ^{13}C NMR (151 MHz, $CDCl_3$) δ 201.2, 144.7, 134.1, 131.2, 129.1, 128.7, 120.6, 74.5, 43.6, 25.0, 23.7, 21.6. HRMS (ESI-Quadrupole-Orbitrap) m/z: $[M + H]^+$ Calcd for $C_{14}H_{19}O_2$ 219.1380, found 219.1379. $[\alpha]_D^{27}$: –20.0 (*c* 0.39, $CHCl_3$); HPLC analysis: 94% *ee* (Chiralcel AD-H, 2:98 ^{i}PrOH/hexanes, 1 mL/min, 254 nm), R_t (major) = 9.5 min, R_t (minor) = 11.9 min. IR (KBr thin film, cm^{-1}): ν 3343, 3033, 2944, 2875, 2862, 1699, 1623, 1521, 1276, 764, 751.

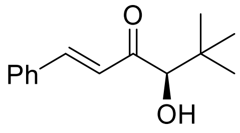

5k

(*S*,*E*)-4-Hydroxy-5,5-dimethyl-1-phenylhex-1-en-3-one (5k): Purified by flash chromatography on silica gel (petroleum ether/ethyl acetate, v:v = 9:1), white solid, mp 99–100 ºC, 31.8 mg, 73% yield. ^{1}H NMR (600 MHz, $CDCl_3$) δ 7.68 (d, *J* = 15.9 Hz, 1H), 7.57–7.55 (m, 2H), 7.41–7.37 (m, 3H), 6.87 (d, *J* = 15.9 Hz, 1H), 4.13 (d, *J* = 6.1 Hz, 1H), 3.48 (d, *J* = 6.1 Hz, 1H), 1.01 (s, 9H); ^{13}C NMR (151 MHz, $CDCl_3$) δ 201.4, 143.4, 134.3, 131.1, 129.1, 128.7, 123.5, 83.5, 36.5, 26.5. HRMS (ESI-Quadrupole-Orbitrap) m/z: $[M + H]^+$ Calcd for $C_{14}H_{19}O_2$ 219.1380, found 219.1381. $[\alpha]_D^{27}$: –85.0 (*c* 1.08, $CHCl_3$); HPLC analysis: >99% *ee* (Chiralcel OJ-H, 8:92 ^{i}PrOH/hexanes, 1 mL/min, 254 nm), R_t (major) = 3.6 min, R_t (minor) = 3.9 min; IR (KBr thin film, cm^{-1}): ν. IR (KBr thin film, cm^{-1}): ν 3400, 3027, 2960, 2878, 1728, 1594, 1557, 1505, 1359, 1277, 919.

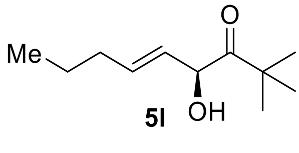

5l

(*R*)-4-Hydroxy-2,2-dimethylnonan-3-one (5l): Purified by flash chromatography on silica gel (petroleum ether/ethyl acetate, v:v = 10:1), colorless oil, 21.0 mg, 57% yield. ^{1}H NMR (600 MHz, $CDCl_3$) δ 7.01–6.94 (m, 1H), 6.27 (dt, *J* = 15.5, 1.6 Hz, 1H), 4.04 (d, *J* = 7.2 Hz, 1H), 3.40 (d, *J* = 7.2 Hz, 1H), 2.25–2.19 (m, 2H), 1.55–1.47 (m, 2H), 0.96 (s, 9H), 0.94 (t, *J* = 7.4 Hz, 3H); ^{13}C NMR (151 MHz, $CDCl_3$) δ 201.4, 148.9, 128.1, 82.7, 36.2, 34.7, 26.5, 21.3, 13.8. HRMS (ESI-Quadrupole-Orbitrap)

m/z: [M + H]$^+$ Calcd for $C_{11}H_{21}O_2$ 186.1536, found 186.1535; $[\alpha]_D^{27}$: –111.5 (*c* 0.25, $CHCl_3$); HPLC analysis: 95% *ee* (Chiralcel AD-H, 2:98 ^{i}PrOH/hexanes, 1 mL/min, 254 nm), R_t (major) = 10.6 min, R_t (minor) = 6.8 min; IR (KBr thin film, cm^{-1}): ν. IR (KBr thin film, cm^{-1}): ν 3386, 3044, 2983, 2951, 2924, 2874, 2862, 2843, 1717, 1601, 1277.

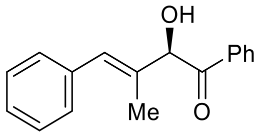

6a

(*R,E*)-2-Hydroxy-3-methyl-1,4-diphenylbut-3-en-1-one (6a): Purified by flash chromatography on silica gel (petroleum ether/ethyl acetate, v:v = 7:1), yellow oil, 39.3 mg, 78% yield. ^{1}H NMR (600 MHz, $CDCl_3$) δ 8.08–8.06 (m, 2H), 7.60–7.57 (m, 1H), 7.47–7.44 (m, 2H), 7.31–7.27 (m, 2H), 7.23–7.20 (m, 3H), 6.82 (s, 1H), 5.52 (d, *J* = 5.4 Hz, 1H), 4.38 (d, *J* = 5.4 Hz, 1H), 1.67 (d, *J* = 1.4 Hz, 3H); ^{13}C NMR (151 MHz, $CDCl_3$) δ 199.9, 136.9, 136.2, 134.3, 133.7, 132.3, 129.00, 128.96, 128.8, 128.3, 127.2, 80.5, 12.8. HRMS (ESI-Quadrupole-Orbitrap) m/z: [M + Na]$^+$ Calcd for $C_{17}H_{16}O_2Na$ 275.1043, found 275.1040. $[\alpha]_D^{27}$: –66.3 (*c* 0.61, $CHCl_3$); HPLC analysis: 96% *ee* (Chiralcel AD-H, 5:95 ^{i}PrOH/hexanes, 1 mL/min, 254 nm), R_t (major) = 13.5 min, R_t (minor) = 11.5 min. IR (KBr thin film, cm^{-1}): ν 3708, 3024, 2966, 2833, 1715, 1681, 1595, 1552, 1455, 1278, 765.

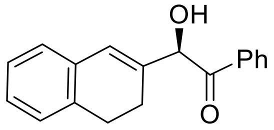

6b

(*R*)-2-(3,4-Dihydronaphthalen-2-yl)-2-hydroxy-1-phenylethan-1-one (6b): Purified by flash chromatography on silica gel (petroleum ether/ethyl acetate, v:v = 10:1), yellow oil, 17.4 mg, 33% yield. ^{1}H NMR (400 MHz, $CDCl_3$) δ 8.04–8.01 (m, 2H), 7.59–7.55 (m, 1H), 7.46–7.42 (m, 2H), 7.16–7.04 (m, 4H), 6.68 (s, 1H), 5.60 (d, *J* = 5.3 Hz, 1H), 4.30 (d, *J* = 5.6 Hz, 1H), 2.83–2.74 (m, 1H), 2.69–2.60 (m, 1H), 2.36–2.28 (m, 1H), 1.99–1.90 (m, 1H); ^{13}C NMR (151 MHz, $CDCl_3$) δ 199.4, 138.2, 135.2, 134.3, 133.7, 133.6, 128.9, 128.8, 127.7, 127.4, 126.60, 126.62, 77.7, 27.9, 22.3. HRMS (ESI-Quadrupole-Orbitrap) m/z: [M + Na]$^+$ Calcd for $C_{18}H_{16}O_2Na$ 253.1199, found 253.1198. $[\alpha]_D^{27}$: –9.3 (*c* 0.92, $CHCl_3$); HPLC analysis: >99% *ee* (Chiralcel IC, 10:90 ^{i}PrOH/hexanes, 1 mL/min, 254 nm), R_t (major) = 7.6 min, R_t (minor) = 6.9 min. IR (KBr thin film, cm^{-1}): ν 3668, 3018, 2832, 1731, 1591, 1554, 1374, 1241, 925, 782, 715.

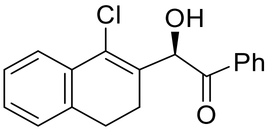

6c

(*R*)-2-(1-Chloro-3,4-dihydronaphthalen-2-yl)-2-hydroxy-1-phenylethan-1-one (6c): Purified by flash chromatography on silica gel (petroleum ether/ethyl acetate, v:v = 6:1), yellow oil, 50.7 mg, 88% yield. ^{1}H NMR (600 MHz, $CDCl_3$) δ 8.04 (d, *J* = 8.0 Hz, 2H), 7.68 (d, *J* = 7.7 Hz, 1H), 7.58–7.55 (m, 1H), 7.45–7.42 (m, 2H), 7.26–7.17 (m, 2H), 7.05 (d, *J* = 7.3 Hz, 1H), 6.27 (d, *J* = 5.0 Hz, 1H), 4.43 (d, *J* = 5.0 Hz, 1H), 2.79–2.73 (m, 1H), 2.56–2.50 (m, 1H), 2.38–2.33 (m, 1H), 1.87–1.82 (m, 1H); NMR (151 MHz, $CDCl_3$) δ 199.5, 136.4, 134.6, 133.2, 133.1, 132.5, 130.1, 129.0, 128.8, 128.6, 127.2, 126.8, 125.3, 74.1, 27.7, 23.4. HRMS (ESI-Quadrupole-Orbitrap) m/z: $[M + Na]^+$ Calcd for $C_{18}H_{15}O_2ClNa$ 321.0653, found 321.0651. $[\alpha]_D^{27}$: –122.0 (*c* 1.82, $CHCl_3$); HPLC analysis: 96% *ee* (Chiralcel OJ-H, 5:95 ^{i}PrOH/hexanes, 1 mL/min, 254 nm), R_t (major) = 5.8 min, R_t (minor) = 6.4 min. IR (KBr thin film, cm^{-1}): ν 3621, 3068, 3029, 2962, 2862, 1718, 1618, 1537, 1363, 775, 669.

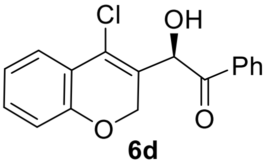

6d

(*R*)-2-(4-Chloro-2H-chromen-3-yl)-2-hydroxy-1-phenylethan-1-one (6d): Purified by flash chromatography on silica gel (petroleum ether/ethyl acetate, v:v = 10:1), yellow oil, 49.2 mg, 82% yield. ^{1}H NMR (600 MHz, $CDCl_3$) δ 8.06–8.05 (m, 2H), 7.62–7.59 (m, 1H), 7.54–7.52 (m, 1H), 7.48–7.45 (m, 2H), 7.19–7.16 (m, 1H), 6.98–6.95 (m, 1H), 6.76 (dd, *J* = 8.2, 0.7 Hz, 1H), 6.17 (d, *J* = 4.8 Hz, 1H), 4.70 (d, *J* = 4.8 Hz, 1H), 4.35–4.31 (m, 2H); ^{13}C NMR (151 MHz, $CDCl_3$) δ 198.5, 154.4, 135.0, 132.9, 131.0, 129.1, 128.7, 127.7, 126.0, 125.5, 121.9, 121.3, 116.0, 72.4, 65.0. HRMS (ESI-Quadrupole-Orbitrap) m/z: $[M + Na]^+$ Calcd for $C_{17}H_{13}O_3ClNa$ 323.0445, found 323.0443. $[\alpha]_D^{27}$: –28.4 (*c* 0.91, $CHCl_3$); HPLC analysis: >99% *ee* (Chiralcel OJ-H, 5:95 ^{i}PrOH/hexanes, 1 mL/min, 254 nm), R_t (major) = 15.6 min, R_t (minor) = 14.9 min. IR (KBr thin film, cm^{-1}): ν 3621, 3074, 3030, 2929, 2861, 1718, 1628, 1618, 1564, 1363, 776.

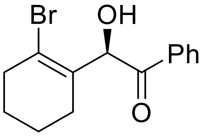

6e

(*R*)-2-(2-Bromocyclohex-1-en-1-yl)-2-hydroxy-1-phenylethan-1-one (6e): Purified by flash chromatography on silica gel (petroleum ether/ethyl acetate, v:v = 10:1), yellow solid, mp 106–112 ºC, 48.8 mg, 83% yield. ^{1}H NMR (400 MHz, $CDCl_3$) δ 8.05–8.02 (m, 2H), 7.65–7.60 (m, 1H), 7.51–7.46 (m, 2H), 6.03 (d, *J* = 4.9 Hz, 1H), 4.29 (d, *J* = 4.9 Hz, 1H), 2.63–2.49 (m, 2H), 2.22–2.12 (m, 1H), 1.72–1.66 (m, 1H), 1.54–1.41 (m, 4H); ^{13}C NMR (151 MHz, $CDCl_3$) δ 199.8, 134.5, 134.0, 133.2, 128.9, 128.7, 125.7, 77.2, 37.0, 25.4, 24.4, 22.0. HRMS (ESI-Quadrupole-Orbitrap) m/z: [M + H]$^+$ Calcd for $C_{14}H_{16}O_2Br$ 295.0328, found 295.0327. $[\alpha]_D^{27}$: –125.3 (*c* 1.67, $CHCl_3$); HPLC analysis: 98% *ee* (Chiralcel AS-H, 10:90 ^{i}PrOH/hexanes, 1 mL/min, 254 nm), R_t (major) = 6.2 min, R_t (minor) = 5.2 min. IR (KBr thin film, cm^{-1}): ν 3749, 3024, 2924, 2831, 1740, 1610, 1553, 1504, 1488, 1364, 865.

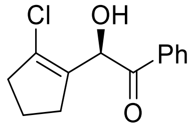

6f

(*R*)-2-(2-Chlorocyclopent-1-en-1-yl)-2-hydroxy-1-phenylethan-1-one (6f): Purified by flash chromatography on silica gel (petroleum ether/ethyl acetate, v:v = 8:1), yellow oil, 354 mg, 75% yield. ^{1}H NMR (600 MHz, $CDCl_3$) δ 8.00–7.98 (m, 2H), 7.62–7.60 (m, 1H), 7.49–7.46 (m, 2H), 5.86 (s, 1H), 4.17 (s, 1H), 2.63–2.40 (m, 3H), 1.93–1.70 (m, 3H); ^{13}C NMR (151 MHz, $CDCl_3$) δ 199.1, 134.5, 133.1, 132.0, 129.1, 128.9, 128.6, 70.1, 38.1, 28.9, 20.5. HRMS (ESI-Quadrupole-Orbitrap) m/z: [M + Na]$^+$ Calcd for $C_{13}H_{13}O_2ClNa$ 259.0496, found 259.0495. $[\alpha]_D^{27}$: –90.9 (*c* 0.86, $CHCl_3$); HPLC analysis: 95% *ee* (Chiralcel OD-H, 1.5:98.5 ^{i}PrOH/hexanes, 1 mL/min, 254 nm), R_t (major) = 5.8 min, R_t (minor) = 5.1 min. IR (KBr thin film, cm^{-1}): ν 3607, 3073, 2922, 2831, 1712, 1679, 1608, 1557, 1456, 1364, 781.

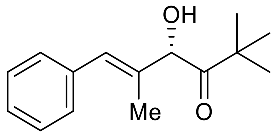

6g

(*R,E*)-4-Hydroxy-2,2,5-trimethyl-6-phenylhex-5-en-3-one (6g): Purified by flash chromatography on silica gel (petroleum ether/ethyl acetate, v:v = 8:1), yellow oil, 42.3 mg, 91% yield. ^{1}H NMR (400 MHz, $CDCl_3$) δ 7.38–7.34 (m, 2H), 7.29–7.26 (m, 3H), 6.68 (s, 1H), 5.04 (d, *J* = 6.2 Hz, 1H), 4.11 (d, *J* = 6.2 Hz, 1H), 1.72 (d, *J* = 1.2 Hz, 3H), 1.25 (s, 9H); ^{13}C NMR (151 MHz, $CDCl_3$) δ 216.0, 136.9, 135.5, 132.2, 128.9, 128.4, 127.2, 79.8, 43.6, 27.2, 13.0. HRMS (ESI-Quadrupole-Orbitrap) m/z: [M + Na]$^+$ Calcd for $C_{17}H_{16}O_2Na$ 255.1356, found 255.1353. $[\alpha]_D^{27}$: –73.6 (*c* 2.00,

$CHCl_3$); HPLC analysis: 90% *ee* (Chiralcel IC, 2:98 ^{i}PrOH/hexanes, 1 mL/min, 254 nm), R_t (major) = 6.5 min, R_t (minor) = 6.0 min. IR (KBr thin film, cm^{-1}): ν 3433, 3075, 3027, 2912, 2832, 1701, 1624, 1541, 1508, 1362, 765.

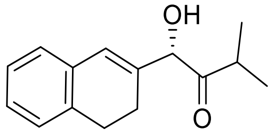

6h

(*R*)-2-(3,4-Dihydronaphthalen-2-yl)-2-hydroxy-1-phenylethan-1-one (6h): Purified by flash chromatography on silica gel (petroleum ether/ethyl acetate, v:v = 5:1), yellow oil, 34.1 mg, 74% yield. ^{1}H NMR (600 MHz, $CDCl_3$) δ 7.18–7.13 (m, 2H), 7.11–7.07 (m, 2H), 6.64 (s, 1H), 4.89 (d, *J* = 4.7 Hz, 1H), 4.01 (d, *J* = 4.7 Hz, 1H), 2.98–2.93 (m, 1H), 2.85–2.73 (m, 2H), 2.36–2.30 (m, 1H), 1.90–1.85 (m, 1H), 1.15 (d, *J* = 7.1 Hz, 3H), 1.05 (d, *J* = 7.1 Hz, 3H); ^{13}C NMR (151 MHz, $CDCl_3$) δ 214.2, 137.1, 135.3, 133.5, 128.9, 127.8, 127.6, 126.7, 126.6, 79.7, 35.6, 27.9, 22.0, 19.8, 18.0. HRMS (ESI-Quadrupole-Orbitrap) m/z: $[M + Na]^+$ Calcd for $C_{15}H_{18}O_2Na$ 253.1199, found 253.1198. $[\alpha]_D^{27}$: –28.1 (*c* 1.57, $CHCl_3$); HPLC analysis: 81% *ee* (Chiralcel IA, 5:95 ^{i}PrOH/hexanes, 1 mL/min, 254 nm), R_t (major) = 8.4 min, R_t (minor) = 7.1 min. IR (KBr thin film, cm^{-1}): ν 3404, 3080, 3032, 2941, 1711, 1619, 1512, 1400, 1360, 1293, 783.

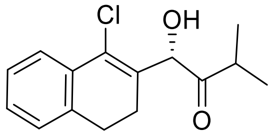

6i

(*R*)-1-(1-Chloro-3,4-dihydronaphthalen-2-yl)-1-hydroxy-3-methylbutan-2-one (6i): Purified by flash chromatography on silica gel (petroleum ether/ethyl acetate, v:v = 5:1), colorless oil, 47.0 mg, 89% yield. ^{1}H NMR (400 MHz, $CDCl_3$) δ 7.70–7.67 (m, 1H), 7.30–7.12 (m, 3H), 5.73 (d, *J* = 4.6 Hz, 1H), 4.06 (d, *J* = 4.6 Hz, 1H), 2.99–2.93 (m, 1H), 2.87–2.68 (m, 2H), 2.47–2.39 (m, 1H), 1.98–1.90 (m, 1H), 1.21 (d, *J* = 7.0 Hz, 3H), 1.06 (d, *J* = 7.0 Hz, 3H); ^{13}C NMR (101 MHz, $CDCl_3$) δ 213.3, 136.3, 132.4, 132.3, 130.3, 128.8, 127.2, 126.9, 125.0, 75.3, 35.8, 27.6, 23.2, 19.7, 17.9. HRMS (ESI-Quadrupole-Orbitrap) m/z: $[M + Na]^+$ Calcd for $C_{15}H_{17}O_2ClNa$ 287.0809, found 287.0808. $[\alpha]_D^{27}$: –16.0 (*c* 1.51, $CHCl_3$); HPLC analysis: 93% *ee* (Chiralcel AD-H, 5:95 ^{i}PrOH/hexanes, 1 mL/min, 254 nm), R_t (major) = 6.2 min, R_t (minor) = 5.9 min. IR (KBr thin film, cm^{-1}): ν 3478, 3028, 2955, 2942, 1733, 1621, 1491, 1362, 782, 715, 689.

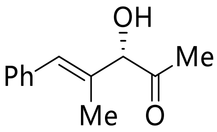

6j

(*R*,*E*)-3-Hydroxy-4-methyl-5-phenylpent-4-en-2-one (6j): Purified by flash chromatography on silica gel (petroleum ether/ethyl acetate, v:v = 5:1), colorless oil, 19.4 mg, 51% yield. ^{1}H NMR (600 MHz, $CDCl_3$) δ 7.38–7.34 (m, 2H), 7.31–7.29 (m, 2H), 7.27–7.24 (m, 1H), 6.72 (s, 1H), 4.67 (s, 1H), 3.99 (s, 1H), 2.24 (s, 3H), 1.72 (d, *J* = 1.4 Hz, 3H); ^{13}C NMR (151 MHz, $CDCl_3$) δ 208.3, 136.7, 134.9, 132.0, 129.0, 128.4, 127.3, 84.3, 25.0, 12.6. HRMS (ESI-Quadrupole-Orbitrap) m/z: $[M + Na]^+$ Calcd for $C_{17}H_{16}O_2Na$ 255.1356, found. $[\alpha]_D^{27}$: –13.7 (*c* 4.23, $CHCl_3$); HPLC analysis: 75% *ee* (Chiralcel IA, 2:98 ^{i}PrOH/hexanes, 0.8 mL/min, 254 nm), R_t (major) = 10.3 min, R_t (minor) = 9.2 min. IR (KBr thin film, cm^{-1}): ν 3459, 3032, 2962, 2835, 1731, 1614, 1523, 1509, 1363, 807, 570.

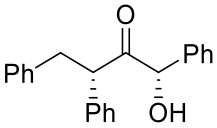

7a

(1*S*,3*S*)-1-Hydroxy-1,3,4-triphenylbutan-2-one (7a): Purified by flash chromatography on silica gel (petroleum ether/ethyl acetate, v:v = 10:1), white solid, mp 125–128 ºC, 42.3 mg, 63% yield and 7:1 dr (*syn*:*anti*). *syn*–diastereomer: ^{1}H NMR (600 MHz, $CDCl_3$) δ 7.34–7.23 (m, 6H), 7.17–7.15 (m, 2H), 7.06–7.02 (m, 5H), 6.72–6.71 (m, 2H), 5.02 (d, *J* = 4.7 Hz, 1H), 4.16 (d, *J* = 4.7 Hz, 1H), 3.96 (dd, *J* = 8.4, 6.6 Hz, 1H), 3.26 (dd, *J* = 13.8, 8.4 Hz, 1H), 2.85 (dd, *J* = 13.8, 6.6 Hz, 1H); ^{13}C NMR (151 MHz, $CDCl_3$) δ 208.1, 138.6, 137.5, 137.3, 129.3, 129.1, 128.8, 128.7, 128.5, 128.2, 128.0, 127.8, 126.2, 78.2, 55.4, 38.7. HRMS (ESI-Quadrupole-Orbitrap) m/z: $[M + Na]^+$ Calcd for $C_{22}H_{20}O_2Na$ 393.1356, found 393.1357. $[\alpha]_D^{27}$: +1.0 (*c* 5.27, $CHCl_3$); HPLC analysis: 98% *ee* (Chiralcel AD-H, 10:90 ^{i}PrOH/hexanes, 1 mL/min, 220 nm), R_t (major) = 4.9 min, R_t (minor) = 5.3 min. IR (KBr thin film, cm^{-1}): ν 3527, 3049, 2973, 2912, 1749, 1601, 1556, 1451, 1358, 908, 751.

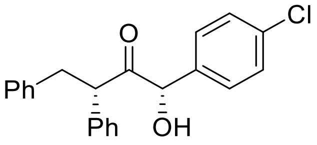

7b

(1*S*,3*S*)-1-(4-Chlorophenyl)-1-hydroxy-3,4-diphenylbutan-2-one (7b): Purified by flash chromatography on silica gel (petroleum ether/ethyl acetate, v:v = 20:1), white solid, mp 119–123 ºC, 44.1 mg, 63% yield and 5:1 dr (*syn*:*anti*). *syn*–diastereomer: ^{1}H NMR (600 MHz, $CDCl_3$) δ 7.36–7.28 (m, 3H), 7.18–7.15 (m, 4H), 7.11–7.05 (m, 3H), 6.90 (d, *J* = 8.3 Hz, 2H), 6.77 (d, *J* = 7.1 Hz, 2H), 4.99 (d, *J* = 3.4 Hz, 1H), 4.15 (d, *J* = 4.0 Hz, 1H), 3.95 (dd, *J* = 9.2, 5.8 Hz, 1H), 3.32 (dd, *J* = 13.7, 9.3 Hz, 1H), 2.82 (dd, *J* = 13.7, 5.7 Hz, 1H); ^{13}C NMR (151 MHz, $CDCl_3$) δ 207.5, 138.4, 137.3, 135.5, 134.6, 129.4, 129.2, 129.0, 128.8, 128.5, 128.3, 128.1, 126.3, 77.6, 55.7, 38.8. HRMS (ESI-Quadrupole-Orbitrap) m/z: $[M + Na]^+$ Calcd for $C_{22}H_{19}O_2ClNa$ 373.0966, found 373.0967. $[\alpha]_D^{27}$: +63.3 (*c* 2.00, $CHCl_3$); HPLC analysis: 90% *ee* (Chiralcel IA, 5:95 ^{i}PrOH/hexanes, 1 mL/min, 220 nm), R_t (major) = 7.9 min, R_t (minor) = 9.4 min. IR (KBr thin film, cm^{-1}): ν 3689, 3047, 2977, 2994, 1742, 1556, 1486, 1344, 925, 716, 690.

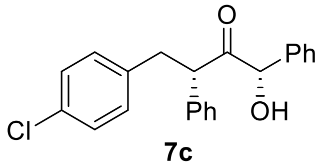

7c

(1*S*,3*S*)-4-(4-Chlorophenyl)-1-hydroxy-1,3-diphenylbutan-2-one (7c): Purified by flash chromatography on silica gel (petroleum ether/ethyl acetate, v:v = 15:1), white solid, mp 102–105 ºC, 29.4 mg, 42% yield and 4:1 dr (*syn*:*anti*). *syn*–diastereomer: ^{1}H NMR (400 MHz, $CDCl_3$) δ 7.33–7.20 (m, 6H), 7.15–7.13 (m, 2H), 6.99–6.49 (m, 4H), 6.62 (d, *J* = 8.3 Hz, 2H), 4.99 (d, *J* = 4.7 Hz, 1H), 4.10 (d, *J* = 4.7 Hz, 1H), 3.90 (dd, *J* = 8.9, 5.8 Hz, 1H), 3.22 (dd, *J* = 13.8, 9.0 Hz, 1H), 2.76 (dd, *J* = 13.8, 6.2 Hz, 1H); ^{13}C NMR (101 MHz, $CDCl_3$) δ 207.6, 137.03, 136.95, 132.0, 130.1, 129.3, 129.0, 128.7, 128.4, 128.3, 128.1, 127.6, 78.2, 55.3, 38.0. HRMS (ESI-Quadrupole-Orbitrap) m/z: $[M + Na]^+$ Calcd for $C_{22}H_{19}O_2ClNa$ 373.0964, found 373.0967. $[\alpha]_D^{27}$: +27.5 (*c* 1.01, $CHCl_3$); HPLC analysis: 90% *ee* (Chiralcel OD-H, 8:92 ^{i}PrOH/hexanes, 1 mL/min, 220 nm), R_t (major) = 5.6 min, R_t (minor) = 9.1 min. IR (KBr thin film, cm^{-1}): ν 3730, 3048, 3026, 2982, 1736, 1600, 1556, 1486, 1359, 903, 765.

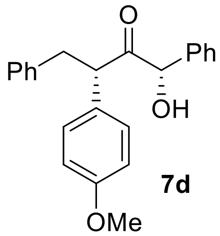

7d

(1*S*,3*S*)-1-Hydroxy-3-(4-methoxyphenyl)-1,4-diphenylbutan-2-one (7d): Purified by flash chromatography on silica gel (petroleum ether/ethyl acetate, v:v = 15:1),

white solid, mp 138–141 ºC, 42.7 mg, 62% yield and 5:1 dr (*syn*:*anti*). *syn*–diastereomer: ^{1}H NMR (400 MHz, $CDCl_3$) δ 7.31–7.27 (m, 2H), 7.26–7.23 (m, 1H), 7.08–7.20 (m, 7H), 6.86 (d, *J* = 8.7 Hz, 2H), 6.73 (dd, *J* = 7.2, 1.4 Hz, 1H), 5.04 (d, *J* = 4.7 Hz, 1H), 4.17 (d, *J* = 4.7 Hz, 1H), 3.90 (dd, *J* = 8.0, 6.9 Hz, 1H), 3.81 (s, 3H), 3.23 (dd, *J* = 13.7, 8.2, Hz, 1H), 2.83 (dd, *J* = 13.8, 6.7 Hz, 1H); ^{13}C NMR (101 MHz, $CDCl_3$) δ 208.2, 159.2, 138.6, 137.3, 129.6, 129.3, 129.0, 128.8, 128.6, 128.1, 127.7, 126.1, 114.6, 77.9, 55.3, 54.4, 38.6. HRMS (ESI-Quadrupole-Orbitrap) m/z: [M + Na]$^+$ Calcd for $C_{23}H_{22}O_3Na$ 369.1461, found 369.1460. $[\alpha]_D^{27}$: +14.1 (*c* 4.80, $CHCl_3$); HPLC analysis: >99% *ee* (Chiralcel AD-H, 2:98 ^{i}PrOH/hexanes, 1 mL/min, 220 nm), R_t (major) = 17.4 min, R_t (minor) = 23.0 min. IR (KBr thin film, cm^{-1}): ν 3452, 3036, 2926, 2878, 1740, 1592, 1556, 1504, 1447, 1357, 818.

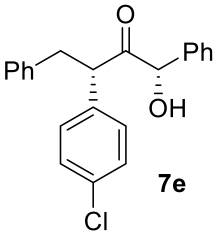

7e

(1*S*,3*S*)-3-(4-Chlorophenyl)-1-hydroxy-1,4-diphenylbutan-2-one (7e): Purified by flash chromatography on silica gel (petroleum ether/ethyl acetate, v:v = 20:1), white solid, mp 117–122 ºC, 35.0 mg, 50% yield and 3:1 dr (*syn*:*anti*). *syn*–diastereomer: ^{1}H NMR (400 MHz, $CDCl_3$) δ 7.31–7.25 (m, 5H), 7.10–7.01 (m, 7H), 6.71–6.69 (m, 2H), 5.01 (d, *J* = 4.6 Hz, 1H), 4.12 (d, *J* = 4.6 Hz, 1H), 3.94 (dd, *J* = 7.9, 7.0 Hz, 1H), 3.19 (dd, *J* = 13.7, 7.9 Hz, 1H), 2.83 (dd, *J* = 13.7, 7.0 Hz, 1H); ^{13}C NMR (101 MHz, $CDCl_3$) δ 207.8, 138.1, 137.0, 135.8, 133.9, 129.8, 129.4, 129.1, 128.8, 128.7, 128.2, 127.7, 126.3, 78.3, 54.6, 38.7. HRMS (ESI-Quadrupole-Orbitrap) m/z: [M + Na]$^+$ Calcd for $C_{22}H_{19}O_2ClNa$ 373.0966, found 373.0967. $[\alpha]_D^{27}$: +83.8 (*c* 0.68, $CHCl_3$); HPLC analysis: >99% *ee* (Chiralcel AD-H, 3:97 ^{i}PrOH/hexanes, 1 mL/min, 220 nm), R_t (major) = 12.2 min, R_t (minor) = 14.6 min. IR (KBr thin film, cm^{-1}): 3748, 3116, 3033, 2872, 2863, 1731, 1605, 1539, 1487, 1276, 765.

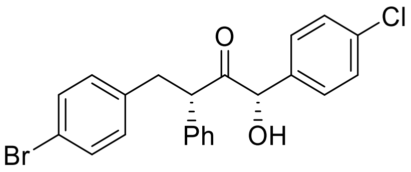

7f

(1*S*,3*S*)-4-(4-Bromophenyl)-1-(4-chlorophenyl)-1-hydroxy-3-phenylbutan-2-one (7f): Purified by flash chromatography on silica gel (petroleum ether/ethyl acetate, v:v = 20:1), white solid, mp 158–163 ºC, 42.8 mg, 50% yield and 3:1 dr (*syn*:*anti*). *syn*–

diastereomer: ^{1}H NMR (400 MHz, $CDCl_3$) δ 7.35–7.27 (m, 3H), 7.19–7.14 (m, 6H), 6.88 (d, *J* = 8.4 Hz, 2H), 6.61 (d, *J* = 8.3 Hz, 2H), 4.96 (d, *J* = 4.6 Hz, 1H), 4.09 (d, *J* = 4.6 Hz, 1H), 3.88 (dd, *J* = 9.5, 5.6 Hz, 1H), 3.24 (dd, *J* = 13.6, 9.5 Hz, 1H), 2.74 (dd, *J* = 13.6, 5.6 Hz, 1H); ^{13}C NMR (101 MHz, $CDCl_3$) δ 207.1, 137.3, 136.8, 135.2, 134.7, 131.2, 130.5, 129.4, 129.1, 128.8, 128.3, 128.2, 120.3, 77.5, 55.4, 38.1. HRMS (ESI-Quadrupole-Orbitrap) m/z: $[M + Na]^+$ Calcd for $C_{22}H_{18}O_2BrClNa$ 451.0071, found 451.0068. $[\alpha]_D^{27}$: –32.8 (*c* 2.17, $CHCl_3$); HPLC analysis: 82% *ee* (Chiralcel IC, 3:97 ^{i}PrOH/hexanes, 1 mL/min, 220 nm), R_t (major) = 6.8 min, R_t (minor) = 7.6 min. IR (KBr thin film, cm^{-1}): ν 3549, 3062, 2973, 2865, 1734, 1590, 1550, 1484, 1362, 929, 764.

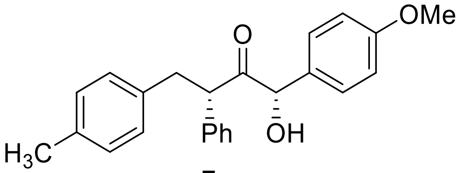

7g

(1*S*,3*S*)-1-Hydroxy-1-(4-methoxyphenyl)-3-phenyl-4-p-tolylbutan-2-one (7g): Purified by flash chromatography on silica gel (petroleum ether/ethyl acetate, v:v = 15:1), white solid, mp 87–97 ºC, 34.8 mg, 48% yield and 5:1 dr (*syn*:*anti*). *syn*–diastereomer: ^{1}H NMR (600 MHz, $CDCl_3$) δ 7.32–7.30 (m, 2H), 7.16–7.14 (m, 2H), 7.08–7.06 (m, 1H), 6.92 (d, *J* = 8.6 Hz, 2H), 6.85 (d, *J* = 7.8 Hz, 2H), 6.75 (d, *J* = 8.6 Hz, 2H), 6.64 (d, *J* = 8.0 Hz, 2H), 4.96 (d, *J* = 4.4 Hz, 1H), 4.09 (d, *J* = 4.6 Hz, 1H), 3.94 (dd, *J* = 8.5, 6.4 Hz, 1H), 3.81 (s, 3H), 3.23 (dd, *J* = 13.8, 8.5 Hz, 1H), 2.81 (dd, *J* = 13.8, 6.4 Hz, 1H), 2.24 (s, 3H); ^{13}C NMR (151 MHz, $CDCl_3$) δ 208.3, 159.8, 137.6, 135.6, 129.3, 129.2, 129.0, 128.8, 128.7, 128.5, 128.3, 127.9, 114.4, 77.6, 55.5, 55.4, 38.2, 21.1. HRMS (ESI-Quadrupole-Orbitrap) m/z: $[M + Na]^+$ Calcd for $C_{24}H_{24}O_3Na$ 383.1618, found 383.1619. $[\alpha]_D^{27}$: +74.0 (*c* 0.98, $CHCl_3$); HPLC analysis: 92% *ee* (Chiralcel OD-H, 8:92 ^{i}PrOH/hexanes, 1 mL/min, 220 nm), R_t (major) = 14.4 min, R_t (minor) = 13.2 min. IR (KBr thin film, cm^{-1}): ν 3458, 3057, 3021, 2940, 2912, 2855, 1702, 1561, 1362, 763, 750.

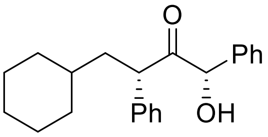

7h

(1*S*,3*S*)-4-Cyclohexyl-1-hydroxy-1,3-diphenylbutan-2-one (7h): Purified by flash chromatography on silica gel (petroleum ether/ethyl acetate, v:v = 20:1), yellow solid, mp 96–100 ºC, 34.8 mg, 49% yield and 3:1 dr (*syn*:*anti*). *syn*–diastereomer: ^{1}H NMR

(600 MHz, $CDCl_3$) δ 7.42–7.39 (m, 2H), 7.36–7.30 (m, 5H), 7.27–7.25 (m, 1H), 7.18–7.16 (m, 2H), 5.05 (d, *J* = 4.6 Hz, 1H), 4.28 (d, *J* = 4.6 Hz, 1H), 3.85 (dd, *J* = 8.8, 6.1 Hz, 1H), 1.92–1.85 (m, 1H), 1.52–1.44 (m, 3H), 1.37–1.31 (m, 2H), 1.01–0.87 (m, 3H), 0.81–0.68 (m, 2H), 0.63–0.56 (m, 1H), 0.52–0.45 (m, 1H); ^{13}C NMR (101 MHz, $CDCl_3$) δ 209.0, 138.5, 137.7, 129.2, 129.1, 128.8, 128.3, 127.8, 127.6, 78.2, 50.4, 40.2, 35.0, 33.2, 32.6, 26.3, 26.03, 26.01. HRMS (ESI-Quadrupole-Orbitrap) m/z: [M + Na]$^+$ Calcd for $C_{22}H_{26}O_2Na$ 345.1825, found 345.1826. $[\alpha]_D^{27}$: +38.9 (*c* 0.50, $CHCl_3$); HPLC analysis: 87% *ee* (Chiralcel AD-H, 1:99 ^{i}PrOH/hexanes, 1 mL/min, 220 nm), R_t (major) = 10.9 min, R_t (minor) = 12.7 min. IR (KBr thin film, cm^{-1}): ν 3481, 3033, 3021, 2954, 2914, 2848, 1739, 1616, 1535, 1276, 764.

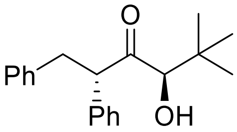

7i

(2*S*,4*S*)-4-Hydroxy-5,5-dimethyl-1,2-diphenylhexan-3-one (7i): Purified by flash chromatography on silica gel (petroleum ether/ethyl acetate, v:v = 20:1), yellow solid, mp 65–67 ºC, 26.1 mg, 44% yield and 7:1 dr (*syn*:*anti*). *syn*–diastereomer: ^{1}H NMR (600 MHz, $CDCl_3$) δ 7.29–7.26 (m, 4H), 7.24–7.18 (m, 3H), 7.16–7.13 (m, 1H), 7.03–7.01 (m, 2H), 4.28 (dd, *J* = 8.0, 7.1 Hz, 1H), 3.61 (d, *J* = 5.6 Hz, 1H), 3.23 (dd, *J* = 13.4, 8.0 Hz, 1H), 2.93 (dd, *J* = 13.4, 7.1 Hz, 1H), 2.50 (d, *J* = 5.8 Hz, 1H), 0.81 (s. 9H); ^{13}C NMR (151 MHz, $CDCl_3$) δ 213.7, 139.0, 137.7, 129.2, 128.81, 128.76, 128.4, 127.3, 126.6, 85.0, 58.4, 41.9, 36.0, 26.2. HRMS (ESI-Quadrupole-Orbitrap) m/z: [M + Na]$^+$ Calcd for $C_{20}H_{24}O_2Na$ 319.1669, found 319.1670. $[\alpha]_D^{27}$: +14.9 (*c* 0.43, $CHCl_3$); HPLC analysis: >99% *ee* (Chiralcel OD-H, 5:95 ^{i}PrOH/hexanes, 1 mL/min, 220 nm), R_t (major) = 3.7 min, R_t (minor) = 3.9 min. IR (KBr thin film, cm^{-1}): ν 3464, 3036, 2953, 2941, 2882, 2837, 1704, 1616, 1597, 1276, 764.

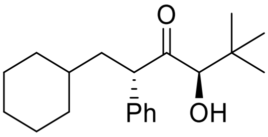

7j

(2*S*,4*S*)-1-Cyclohexyl-4-hydroxy-5,5-dimethyl-2-phenylhexan-3-one (7j): Purified by flash chromatography on silica gel (petroleum ether/ethyl acetate, v:v = 20:1), white solid, mp 74–77 ºC, 29.0 mg, 48% yield and 4:1 dr (*syn*:*anti*). *syn*–diastereomer: ^{1}H NMR (600 MHz, $CDCl_3$) δ 7.31–7.28 (m, 4H), 7.23–7.22 (m, 1H), 4.13 (dd, *J* = 9.1, 5.8 Hz, 1H), 3.91 (d, *J* = 6.8 Hz, 1H), 2.81 (d, *J* = 6.7 Hz, 1H), 1.79–1.70 (m, 3H), 1.66–1.61 (m, 5H), 1.12–1.08 (m, 3H), 0.92 (s. 9H), 0.89–0.80 (m, 2H); ^{13}C NMR

(151 MHz, $CDCl_3$) δ 214.4, 138.0, 128.9, 128.7, 127.1, 84.3, 53.3, 43.1, 36.1, 34.9, 33.9, 32.7, 26.5, 26.4. HRMS (ESI-Quadrupole-Orbitrap) m/z: $[M + Na]^+$ Calcd for $C_{20}H_{30}O_2Na$ 325.2138, found 325.2136. $[\alpha]_D^{27}$: –21.9 (*c* 1.35, $CHCl_3$); HPLC analysis: 99% *ee* (Chiralcel OD-H, 1:99 ^{i}PrOH/hexanes, 1 mL/min, 220 nm), R_t (major) = 8.6 min, R_t (minor) = 11.8 min. IR (KBr thin film, cm^{-1}): ν 3464, 3023, 2947, 2919, 2860, 1734, 1603, 1550, 1506, 1375, 917.

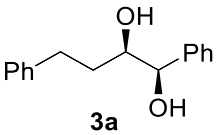

3a

(1*R*,2*R*)-1,4-Diphenylbutane-1,2-diol (3a): Purified by flash chromatography on silica gel (petroleum ether/ethyl acetate, v:v = 1:1), colorless oil, 42.1 mg, 87% yield and 6:1 dr (*syn*:*anti*). *syn*–diastereomer: ^{1}H NMR (400 MHz, $CDCl_3$) δ 7.32–7.19 (m, 7H), 7.13–7.06 (m, 3H), 4.40 (d, *J* = 5.7 Hz, 1H), 3.68–3.65 (m, 1H), 3.04 (d, *J* = 2.5 Hz, 1H), 2.83 (d, *J* = 3.0 Hz, 1H), 2.81–2.75 (m, 1H), 2.58–2.52 (m, 1H), 1.71–1.54 (m, 2H); ^{13}C NMR (101 MHz, $CDCl_3$) δ 141.8, 141.1, 128.6, 128.44, 128.39, 128.2, 126.9, 125.9, 78.0, 75.3, 34.3, 31.9. HRMS (ESI-Quadrupole-Orbitrap) m/z: $[M + Na]^+$ Calcd for $C_{16}H_{18}O_2Na$ 265.1199, found 265.1200. $[\alpha]_D^{27}$: –3.1 (*c* 5.05, $CHCl_3$); HPLC analysis: 94% *ee* (Chiralcel AD-H, 1:99 ^{i}PrOH/hexanes, 1.0 mL/min, 220 nm), R_t (major) = 82.4 min, R_t (minor) = 91.4 min. IR (KBr thin film, cm^{-1}): ν 3498, 3295, 3024, 2876, 1608, 1556, 1503, 1467, 1447, 1362, 762.

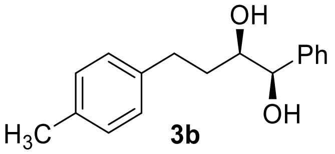

3b

(1*R*,2*R*)-1-Phenyl-4-p-tolylbutane-1,2-diol (3b): Purified by flash chromatography on silica gel (petroleum ether/ethyl acetate, v:v = 1:1), yellow oil, 39.3 mg, 77% yield and 4:1 dr (*syn*:*anti*). *syn*–diastereomer: ^{1}H NMR (400 MHz, $CDCl_3$) δ 7.36–7.28 (m, 5H), 7.06–6.99 (m, 4H), 4.47 (d, *J* = 5.6 Hz, 1H), 3.73–3.66 (m, 1H), 2.82–2.75 (m, 1H), 2.68 (d, *J* = 2.5 Hz, 1H), 2.59–2.52 (m, 1H), 2.51 (d, *J* = 1.6 Hz, 1H), 2.30 (s, 3H), 1.74–1.57 (m, 2H); ^{13}C NMR (101 MHz, $CDCl_3$) δ 141.0, 138.7, 135.3, 129.1, 128.6, 128.3, 128.2, 126.9, 78.0, 75.4, 34.4, 31.5, 21.0. HRMS (ESI-Quadrupole-Orbitrap) m/z: $[M + Na]^+$ Calcd for $C_{17}H_{20}O_2Na$ 279.1356, found 279.1354. $[\alpha]_D^{27}$: –1.7 (*c* 1.73, $CHCl_3$); HPLC analysis: 95% *ee* (Chiralcel OJ-H, 10:90 ^{i}PrOH/hexanes, 1.0 mL/min, 220 nm), R_t (major) = 9.7 min, R_t (minor) = 8.4 min. IR (KBr thin film, cm^{-1}): ν 3439, 3252, 3018, 2931, 2827, 1611, 1509, 1364, 1275, 1260, 765.

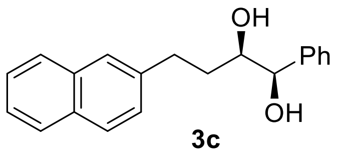

3c

(1*R*,2*R*)-4-(Naphthalen-2-yl)-1-phenylbutane-1,2-diol (3c): Purified by flash chromatography on silica gel (petroleum ether/ethyl acetate, v:v = 1:1), white solid, mp 120–122 ºC, 41.5 mg, 71% yield and 3:1 dr (*syn*:*anti*). *syn*–diastereomer: ^{1}H NMR (600 MHz, $CDCl_3$) δ 7.77 (d, *J* = 7.6 Hz, 1H), 7.72 (d, *J* = 8.0 Hz, 2H), 7.53 (s, 1H), 7.44–7.38 (m, 2H), 7.34–7.28 (m, 5H), 7.24 (dd, *J* = 8.5, 1.6 Hz, 1H), 4.49 (dd, *J* = 6.8, 3.4 Hz, 1H), 3.76–3.72 (m, 1H), 3.00–2.95 (m, 1H), 2.79–2.74 (m, 1H), 2.61 (d, *J* = 3.7 Hz, 1H), 2.53 (d, *J* = 3.5 Hz, 1H), 1.83–1.76 (m, 1H), 1.74–1.69 (m, 1H); ^{13}C NMR (151 MHz, $CDCl_3$) δ 141.1, 139.3, 133.6, 132.1, 128.7, 128.3, 128.0, 127.9, 127.5, 127.3, 126.9, 126.5, 126.0, 125.3, 78.1, 75.3, 34.2, 32.1. HRMS (ESI-Quadrupole-Orbitrap) m/z: $[M + Na]^+$ Calcd for $C_{20}H_{20}O_2Na$ 315.1356, found 315.1353. $[\alpha]_D^{27}$: –7.1 (*c* 3.88, $CHCl_3$); HPLC analysis: 94% *ee* (Chiralcel OJ-H, 15:85 ^{i}PrOH/hexanes, 1.0 mL/min, 220 nm), R_t (major) = 25.8 min, R_t (minor) = 22.5 min. IR (KBr thin film, cm^{-1}): ν 3696, 3342, 3042, 2942, 2824, 1605, 1539, 1489, 1330, 1257, 832.

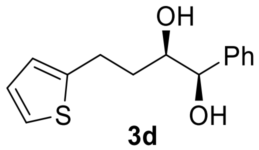

3d

(1*R*,2*R*)-1-Phenyl-4-(thiophen-2-yl)butane-1,2-diol (3d): Purified by flash chromatography on silica gel (petroleum ether/ethyl acetate, v:v = 1:1), yellow oil, 36.7 mg, 74% yield and 4:1 dr (*syn*:*anti*). *syn*–diastereomer: ^{1}H NMR (400 MHz, $CDCl_3$) δ 7.34–7.29 (m, 5H), 7.09 (d, *J* = 5.0 Hz, 1H), 6.89–6.87 (m, 1H), 6.71 (d, *J* = 3.0 Hz, 1H), 4.46 (d, *J* = 6.7 Hz, 1H), 3.75–3.73 (m, 1H), 3.03–2.97 (m, 1H), 2.88–2.82 (m, 1H), 2.80 (bs, 1H), 2.67 (bs, 1H), 1.79–1.63 (m, 2H); ^{13}C NMR (101 MHz, $CDCl_3$) δ 144.6, 140.9, 128.6, 128.2, 126.9, 126.8, 124.3, 123.1, 77.9, 75.0, 34.6, 26.1. HRMS (ESI-Quadrupole-Orbitrap) m/z: $[M + Na]^+$ Calcd for $C_{14}H_{16}O_2SNa$ 271.0763, found 271.0762. $[\alpha]_D^{27}$: –6.8 (*c* 1.62, $CHCl_3$); HPLC analysis: 94% *ee* (Chiralcel OJ-H, 3:97 ^{i}PrOH/hexanes, 0.4 mL/min, 220 nm), R_t (major) = 101.7 min, R_t (minor) = 95.6 min. IR (KBr thin film, cm^{-1}): ν 3625, 3503, 3087, 2945, 2868, 1778, 1589, 1545, 1275, 1260, 750.

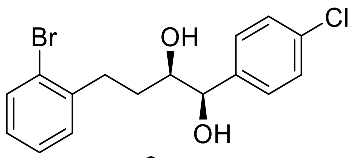

3e

(1*R*,2*R*)-4-(2-Bromophenyl)-1-(4-chlorophenyl)butane-1,2-diol (3e): Purified by flash chromatography on silica gel (petroleum ether/ethyl acetate, v:v = 1:1), white solid, mp 84–86 ºC, 51.7 mg, 73% yield and 5:1 dr (*syn*:*anti*). *syn*–diastereomer: ^{1}H NMR (400 MHz, $CDCl_3$) δ 7.51–7.48 (m, 1H), 7.35–7.32 (m, 1H), 7.28–7.26 (m, 2H), 7.22–7.15 (m, 3H), 7.06–7.02 (m, 1H), 4.48 (dd, *J* = 6.3, 3.2 Hz, 1H), 3.74–3.69 (m, 1H), 2.99–2.91 (m, 1H), 2.78–2.72 (m, 2H), 2.45 (d, *J* = 4.2, 1H), 1.76–1.67 (m, 2H); ^{13}C NMR (151 MHz, $CDCl_3$) δ 143.1, 140.9, 132.9, 130.5, 129.9, 128.4, 127.9, 127.6, 127.1, 125.1, 77.1, 75.3, 32.9, 32.4. HRMS (ESI-Quadrupole-Orbitrap) m/z: $[M + Na]^+$ Calcd for $C_{16}H_{16}O_2BrClNa$ 376.9914, found 376.9913. $[\alpha]_D^{27}$: –10.4 (*c* 0.59, $CHCl_3$); HPLC analysis: 92% *ee* (Chiralcel OJ-H, 5:95 ^{i}PrOH/hexanes, 0.5 mL/min, 220 nm), R_t (major) = 41.0 min, R_t (minor) = 32.7 min. IR (KBr thin film, cm^{-1}): ν 3734, 3418, 3033, 2950, 2853, 1605, 1532, 1276, 1259, 962, 765.

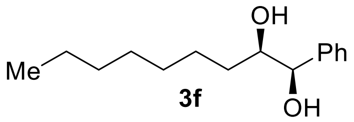

3f

(1*R*,2*R*)-1-Phenylnonane-1,2-diol (3f): Purified by flash chromatography on silica gel (petroleum ether/ethyl acetate, v:v = 3:1), colorless oil, 17.0 mg, 36% yield and 3:1 dr (*syn*:*anti*). *syn*–diastereomer: ^{1}H NMR (600 MHz, $CDCl_3$) δ 7.35–7.33 (m, 3H), 7.32–7.28 (m, 2H), 4.41 (d, *J* = 6.8 Hz, 1H), 3.68–3.62 (m, 1H), 2.82 (bs, 1H), 2.49 (bs, 1H), 1.47–1.39 (m, 1H), 1.37–1.26 (m, 2H), 1.26–1.18 (m, 9H), 0.84 (t, 7.0 Hz, 3H); ^{13}C NMR (101 MHz, $CDCl_3$) δ 141.3, 128.5, 128.1, 126.9, 77.9, 76.0, 32.7, 31.8, 29.5, 29.2, 25.7, 22.6, 14.1. HRMS (ESI-Quadrupole-Orbitrap) m/z: $[M + Na]^+$ Calcd for $C_{15}H_{24}O_2Na$ 259.1669, found 259.1670. $[\alpha]_D^{27}$: +7.8 (*c* 1.50, $CHCl_3$); HPLC analysis: 98% *ee* (Chiralcel OJ-H, 1:99 ^{i}PrOH/hexanes, 0.5 mL/min, 220 nm), R_t (major) = 30.6 min, R_t (minor) = 33.2 min. IR (KBr thin film, cm^{-1}): ν 3702, 3446, 3040, 2946, 2833, 1600, 1554, 1506, 1372, 1259, 751.

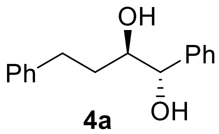

4a

(1*S*,2*R*)-1,4-Diphenylbutane-1,2-diol (4a): Purified by flash chromatography on silica gel (petroleum ether/ethyl acetate, v:v = 1:1), colorless oil, 40.2 mg, 83% yield and 7:1 dr (*anti*:*syn*). *anti*–diastereomer: ^{1}H NMR (400 MHz, $CDCl_3$) δ 7.36–7.33 (m, 4H), 7.32–7.24 (m, 3H), 7.19–7.13 (m, 3H), 4.68 (d, *J* = 3.7 Hz, 1H), 3.87–3.82 (m, 1H), 2.86–2.79 (m, 1H), 2.66–2.58 (m, 1H), 2.40 (bs, 1H), 1.96 (d, *J* = 4.5 Hz, 1H), 1.81–1.73 (m, 1H), 1.66–1.56 (m, 1H); ^{13}C NMR (101 MHz, $CDCl_3$) δ 141.9, 140.3, 128.49, 128.48, 128.4, 128.0, 126.8, 125.9, 77.2, 74.5, 33.4, 32.1. HRMS (ESI-Quadrupole-Orbitrap) m/z: $[M + Na]^+$ Calcd for $C_{16}H_{18}O_2Na$ 265.1199, found

265.1200. $[\alpha]_D^{27}$: +21.9 (*c* 1.45, $CHCl_3$); HPLC analysis: 99% *ee* (Chiralcel OJ-H, 1:99 ^{i}PrOH/hexanes, 0.8 mL/min, 220 nm), R_t (major) = 208.2 min, R_t (minor) = 195.6 min. IR (KBr thin film, cm^{-1}): ν 3565, 3480, 3026, 2931, 2917, 2882, 1595, 1276, 1260, 764, 750.

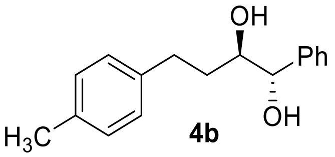

4b

(1*S*,2*R*)-1-Phenyl-4-p-tolylbutane-1,2-diol (4b): Purified by flash chromatography on silica gel (petroleum ether/ethyl acetate, v:v = 1:1), yellow solid, mp 97–98 ºC, 43.5 mg, 85% yield and 8:1 dr (*anti*:*syn*). *anti*–diastereomer: ^{1}H NMR (400 MHz, $CDCl_3$) δ 7.38–7.28 (m, 5H), 7.10–6.99 (m, 4H), 4.68 (d, *J* = 4.5 Hz, 1H), 3.87–3.83 (m, 1H), 2.82–2.75 (m, 1H), 2.62–2.55 (m, 1H), 2.36 (bs, 1H), 2.30 (s, 3H), 1.93 (bs, 1H), 1.88–1.71 (m, 1H), 1.64–1.54 (m, 1H); ^{13}C NMR (101 MHz, $CDCl_3$) δ 140.3, 138.8, 135.3, 129.1, 128.5, 128.4, 128.0, 126.8, 77.1, 74.5, 33.5, 31.7, 21.0. HRMS (ESI-Quadrupole-Orbitrap) m/z: $[M + Na]^+$ Calcd for $C_{17}H_{20}O_2Na$ 279.1356, found 279.1354. $[\alpha]_D^{27}$: +9.4 (*c* 1.31, $CHCl_3$); HPLC analysis: 93% *ee* (Chiralcel AD-H, 5:95 ^{i}PrOH/hexanes, 1.0 mL/min, 220 nm), R_t (major) = 21.2 min, R_t (minor) = 17.9 min. IR (KBr thin film, cm^{-1}): ν 3530, 3495, 3045, 2919, 2871, 1587, 1502, 1430, 1276, 833, 751.

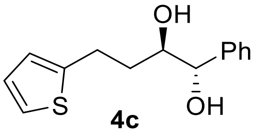

4c

(1*S*,2*R*)-1-Phenyl-4-(thiophen-2-yl)butane-1,2-diol (4c): Purified by flash chromatography on silica gel (petroleum ether/ethyl acetate, v:v = 1:1), white solid, mp 135–139 ºC, 40.2 mg, 81% yield and 6:1 dr (*anti*:*syn*). *anti*–diastereomer: ^{1}H NMR (600 MHz, $CDCl_3$) δ 7.35–7.33 (m, 2H), 7.31–7.29 (m, 3H), 7.08 (dd, *J* = 5.2, 1.1 Hz, 1H), 6.87 (dd, *J* = 5.0, 3.4 Hz, 1H), 6.71 (dd, *J* = 3.2, 0.5 Hz, 1H), 4.46 (d, *J* = 6.6 Hz, 1H), 3.77–3.70 (m, 1H), 3.03–2.98 (m, 1H), 2.86–2.81 (m, 1H), 2.63 (bs, 1H), 2.54 (bs, 1H), 1.80–1.73 (m, 1H), 1.70–1.64 (m, 1H); ^{13}C NMR (151 MHz, $CDCl_3$) δ 144.7, 141.0, 128.7, 128.3, 126.9, 126.8, 124.4, 123.2, 78.0, 75.0, 34.6, 26.1. HRMS (ESI-Quadrupole-Orbitrap) m/z: $[M + Na]^+$ Calcd for $C_{14}H_{16}O_2SNa$ 271.0763, found 271.0762. $[\alpha]_D^{27}$: –44.2 (*c* 0.08, $CHCl_3$); HPLC analysis: >99% *ee* (Chiralcel OJ-H, 3:97 ^{i}PrOH/hexanes, 0.4 mL/min, 220 nm), R_t (major) = 113.7 min, R_t (minor) = 106.6 min. IR (KBr thin film, cm^{-1}): ν 3528, 3410, 3021, 2940, 2875, 1560, 1508, 1428, 1401, 1276, 837.

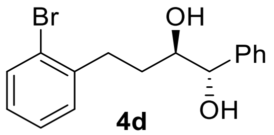

4d

(1*S*,2*R*)-4-(2-Bromophenyl)-1-phenylbutane-1,2-diol (4d): Purified by flash chromatography on silica gel (petroleum ether/ethyl acetate, v:v = 1:1), yellow solid, mp 68–73 ºC, 49.3 mg, 77% yield and 5:1 dr (*anti*:*syn*). *anti*–diastereomer: ^{1}H NMR (400 MHz, $CDCl_3$) δ 7.50 (d, *J* = 7.9 Hz, 1H), 7.36–7.30 (m, 5H), 7.21–7.15 (m, 2H), 7.05–7.01 (m, 1H), 4.68 (d, *J* = 4.5 Hz, 1H), 3.88–3.84 (m, 1H), 2.98–2.91 (m, 1H), 2.79–2.71 (m, 1H), 2.42 (bs, 1H), 2.02 (bs, 1H), 1.85–1.77 (m, 1H), 1.65–1.53 (m, 1H); ^{13}C NMR (151 MHz, $CDCl_3$) δ 141.3, 140.3, 132.9, 130.6, 128.6, 128.1, 127.7, 127.5, 126.9, 124.5, 77.2, 74.6, 32.6, 31.9. HRMS (ESI-Quadrupole-Orbitrap) m/z: $[M + Na]^+$ Calcd for $C_{16}H_{17}O_2BrNa$ 343.0304, found 343.0302. $[\alpha]_D^{27}$: +2.0 (*c* 3.76, $CHCl_3$); HPLC analysis: 99% *ee* (Chiralcel OJ-H, 2:98 ^{i}PrOH/hexanes, 0.5 mL/min, 220 nm), R_t (major) = 149.6 min, R_t (minor) = 111.4 min. IR (KBr thin film, cm^{-1}): ν 3570, 3431, 3014, 2954, 2868, 1600, 1558, 1357, 1236, 742, 696.

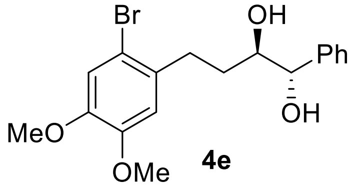

4e

(1*S*,2*R*)-4-(2-Bromo-4,5-dimethoxyphenyl)-1-(4-chlorophenyl)butane-1,2-diol (4e): Purified by flash chromatography on silica gel (petroleum ether/ethyl acetate, v:v = 1:1), colorless oil, 65.4 mg, 79% yield and 8:1 dr (*anti*:*syn*). *anti*–diastereomer: ^{1}H NMR (400 MHz, $CDCl_3$) δ 7.36–7.28 (m, 6H), 6.97 (s, 1H), 6.67 (s, 1H), 4.69 (d, *J* = 4.2 Hz, 1H), 3.92–3.82 (m, 1H), 3.83 (s, 3H), 3.80 (s, 3H), 2.88–2.82 (m, 1H), 2.73–2.65 (m, 1H), 2.40 (bs, 1H), 2.03 (bs, 1H), 1.82–1.75 (m, 1H), 1.60–1.50 (m, 1H); ^{13}C NMR (101 MHz, $CDCl_3$) δ 148.4, 147.8, 140.3, 133.1, 128.5, 128.0, 126.9, 115.5, 114.0, 113.1, 77.1, 74.4, 56.2, 56.1, 32.1. HRMS (ESI-Quadrupole-Orbitrap) m/z: $[M + Na]^+$ Calcd for $C_{18}H_{21}O_4BrNa$ 403.0515, found 403.1514. $[\alpha]_D^{27}$: +2.8 (*c* 6.73, $CHCl_3$); HPLC analysis: 99% *ee* (Chiralcel OJ-H, 15:85 ^{i}PrOH/hexanes, 0.6 mL/min, 220 nm), R_t (major) = 20.2 min, R_t (minor) = 19.4 min. IR (KBr thin film, cm^{-1}): ν 3711, 3440, 3027, 2944, 2875, 1593, 1558, 1507, 1472, 1358, 780.

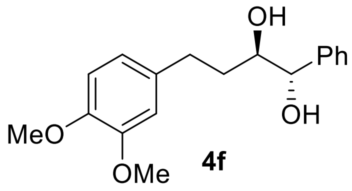

4f

(1*S*,2*R*)-4-(3,4-Dimethoxyphenyl)-1-phenylbutane-1,2-diol (4f): Purified by flash chromatography on silica gel (petroleum ether/ethyl acetate, v:v = 1:1), colorless oil, 47.7 mg, 79% yield and 12:1 dr (*anti*:*syn*). *anti*–diastereomer: ^{1}H NMR (600 MHz, $CDCl_3$) δ 7.38–7.31 (m, 4H), 7.29–7.27 (m, 1H), 6.75 (d, *J* = 8.2 Hz, 1H), 6.67 (dd, *J* = 8.2, 1.9 Hz, 1H), 6.64 (d, *J* = 1.9 Hz, 1H), 4.67 (d, *J* = 4.6 Hz, 1H), 3.85–3.83 (m, 1H), 3.83 (s, 3H), 3.81 (s, 3H), 2.77–2.73 (m, 1H), 2.58–2.53 (m, 1H), 2.45 (bs, 1H), 1.99 (bs, 1H), 1.76–1.70 (m, 1H), 1.61–1.55 (m, 1H); ^{13}C NMR (151 MHz, $CDCl_3$) δ 148.8, 147.2, 140.4, 134.5, 128.5, 128.1, 126.9, 120.3, 111.8, 111.2, 77.2, 74.5, 56.0, 55.9, 33.5, 31.8. HRMS (ESI-Quadrupole-Orbitrap) m/z: $[M + Na]^+$ Calcd for $C_{18}H_{22}O_4Na$ 325.1410, found 325.1409. $[\alpha]_D^{27}$: +4.7 (*c* 2.00, $CHCl_3$); HPLC analysis: >99% *ee* (Chiralcel OJ-H, 10:90 ^{i}PrOH/hexanes, 0.4 mL/min, 220 nm), R_t (major) = 69.2 min, R_t (minor) = 72.9 min. IR (KBr thin film, cm^{-1}): ν 3586, 3454, 2933, 2917, 2871, 2843, 1603, 1501, 1360, 1233, 801.

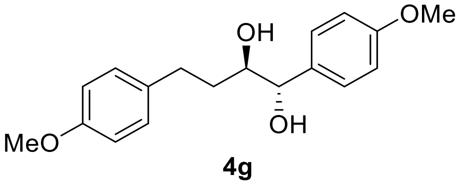

4g

(1*S*,2*R*)-1,4-Bis(4-methoxyphenyl)butane-1,2-diol (4g): Purified by flash chromatography on silica gel (petroleum ether/ethyl acetate, v:v = 1:1), white solid, mp 146–148 ºC, 51.9 mg, 86% yield and >20:1 dr (*anti*:*syn*). *anti*–diastereomer: ^{1}H NMR (600 MHz, $CDCl_3$) δ 7.26–7.24 (m, 2H), 7.06 (d, *J* = 8.7 Hz, 2H), 6.87 (d, *J* = 8.6 Hz, 2H), 6.79 (d, *J* = 8.6 Hz, 2H), 4.58 (d, *J* = 2.9 Hz, 1H), 3.81–3.79 (m, 1H), 3.79 (s, 3H), 3.76 (s, 3H), 2.77–2.72 (m, 1H), 2.59–2.54 (m, 1H), 2.28 (d, *J* = 2.5 Hz, 1H), 1.90 (*J* = 4.7 Hz, 1H), 1.75–1.69 (m, 1H), 1.58–1.51 (m, 1H); ^{13}C NMR (101 MHz, $CDCl_3$) δ 159.3, 157.8, 134.0, 132.4, 129.4, 128.1, 113.8, 113.8, 76.8, 74.4, 55.28, 55.25, 33.8, 31.2. HRMS (ESI-Quadrupole-Orbitrap) m/z: $[M + Na]^+$ Calcd for $C_{18}H_{22}O_4Na$ 325.1410, found 325.1409. $[\alpha]_D^{27}$: –30.0 (*c* 0.23, $CHCl_3$); HPLC analysis: >99% *ee* (Chiralcel OJ-H, 15:85 ^{i}PrOH/hexanes, 0.4 mL/min, 220 nm), R_t (major) = 27.1 min, R_t (minor) = 31.1 min. IR (KBr thin film, cm^{-1}): ν 3564, 3474, 3025, 2936, 2858, 2847, 1606, 1554, 1286, 1231, 788.

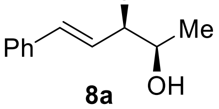

8a

(2*R*,3*R*,*E*)-5-Phenylpent-4-ene-2,3-diol (4a): Purified by flash chromatography on silica gel (petroleum ether/ethyl acetate, v:v = 1:1), colorless oil, 29.6 mg, 83% yield and 9:1 dr (*syn*:*anti*). *syn*–diastereomer: ^{1}H NMR (400 MHz, $CDCl_3$) δ 7.40–7.38 (m,

2H), 7.35–7.32 (m, 2H), 7.29–7.19 (m, 1H), 6.68 (d, *J* = 15.9 Hz, 1H), 6.19 (dd, *J* = 15.9, 5.4 Hz, 1H), 4.04–4.01 (m, 1H), 3.78–3.73 (m, 1H), 2.42 (bs, 2H), 1.23 (d, *J* = 6.3 Hz, 3H); ^{13}C NMR (151 MHz, $CDCl_3$) δ 136.4, 132.9, 128.7, 128.4, 128.1, 126.7, 77.9, 71.1, 19.1. HRMS (ESI-Quadrupole-Orbitrap) m/z: $[M + Na]^+$ Calcd for $C_{11}H_{14}O_2Na$ 201.0886, found 201.0885. $[\alpha]_D^{27}$: +4.8 (*c* 1.33, $CHCl_3$); HPLC analysis: 92% *ee* (Chiralcel AD-H, 6:94 ^{i}PrOH/hexanes, 1 mL/min, 254 nm), R_t (major) = 9.2 min, R_t (minor) = 8.5 min. IR (KBr thin film, cm^{-1}): ν 3568, 3452, 3023, 2975, 2915, 2880, 1595, 1537, 1471, 1151, 767.

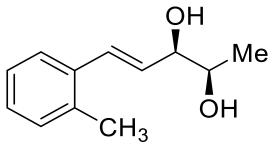

8b

(2*R*,3*R*,*E*)-5-o-Tolylpent-4-ene-2,3-diol (8b): Purified by flash chromatography on silica gel (petroleum ether/ethyl acetate, v:v = 1:1), yellow oil, 28.2 mg, 73% yield and 11:1 dr (*syn*:*anti*). *syn*–diastereomer: ^{1}H NMR (400 MHz, $CDCl_3$) δ 7.27 (d, *J* = 8.0 Hz, 2H), 7.14–7.10 (m, 2H), 6.64 (d, *J* = 15.9 Hz, 1H), 6.13 (dd, *J* = 15.9, 7.1 Hz, 1H), 4.03–3.99 (m, 1H), 3.77–3.70 (m, 1H), 2.41 (bs, 1H), 2.34 (s, 3H), 2.32 (bs, 1H), 1.22 (d, *J* = 6.3 Hz, 3H); ^{13}C NMR (151 MHz, $CDCl_3$) δ 138.0, 133.6, 133.0, 129.4, 127.3, 126.6, 78.0, 71.1, 21.3, 19.1. HRMS (ESI-Quadrupole-Orbitrap) m/z: $[M + Na]^+$ Calcd for $C_{12}H_{16}O_2Na$ 215.1043, found 215.1042. $[\alpha]_D^{27}$: –18.6 (*c* 0.57, $CHCl_3$); HPLC analysis: 91% *ee* (Chiralcel OJ-H, 2:98 ^{i}PrOH/hexanes, 1 mL/min, 254 nm), R_t (major) = 38.0 min, R_t (minor) = 45.2 min. IR (KBr thin film, cm^{-1}): ν 3528, 3416, 3023, 2960, 2875, 1768, 1688, 1608, 1499, 1277, 751.

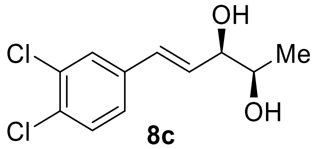

8c

(2*R*,3*R*,*E*)-5-(3,4-Dichlorophenyl)pent-4-ene-2,3-diol (8c): Purified by flash chromatography on silica gel (petroleum ether/ethyl acetate, v:v = 2:1), colorless oil, 43.3 mg, 88% yield and 20:1 dr (*syn*:*anti*). *syn*–diastereomer: ^{1}H NMR (600 MHz, $CDCl_3$) δ 7.46 (d, *J* = 2.0 Hz, 1H), 7.37 (d, *J* = 8.3 Hz, 1H), 7.19 (dd, *J* = 8.3, 2.1 Hz, 1H), 6.59 (d, *J* = 16.0 Hz, 1H), 6.19 (dd, *J* = 15.9, 6.7 Hz, 1H), 4.04–4.01 (m, 1H), 3.76–3.71 (m, 1H), 2.35 (d, *J* = 2.4 Hz, 1H), 2.22 (d, *J* = 2.7 Hz, 1H), 1.23 (d, *J* = 6.4 Hz, 3H); ^{13}C NMR (151 MHz, $CDCl_3$) δ 136.6, 132.8, 131.6, 130.6, 130.5, 129.3, 128.3, 125.8, 76.1, 70.4, 17.8. HRMS (ESI-Quadrupole-Orbitrap) m/z: $[M + Na]^+$ Calcd for $C_{11}H_{12}O_2Cl_2Na$ 269.1007, found 269.0105. $[\alpha]_D^{27}$: +5.3 (*c* 1.13, $CHCl_3$); HPLC analysis: 90% *ee* (Chiralcel IA, 5:95 ^{i}PrOH/hexanes, 1 mL/min, 254 nm), R_t

(major) = 15.9 min, R_t (minor) = 12.8 min. IR (KBr thin film, cm^{-1}): ν 3647, 3475, 3069, 3039, 2976, 2863, 1547, 1463, 1172, 937, 875.

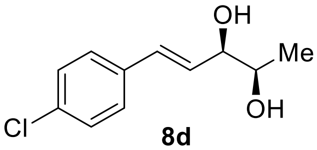

8d

(2*R*,3*R*,*E*)-5-(4-Chlorophenyl)pent-4-ene-2,3-diol (8d): Purified by flash chromatography on silica gel (petroleum ether/ethyl acetate, v:v = 1:1), yellow oil, 37.3 mg, 88% yield and 14:1 dr (*syn*:*anti*). *syn*–diastereomer: ^{1}H NMR (400 MHz, CDCl3) δ 7.32–7.27 (m, 4H), 6.64 (d, *J* = 15.9 Hz, 1H), 6.17 (dd, *J* = 15.9, 6.8 Hz, 1H), 4.04–4.01 (m, 1H), 3.78–3.70 (m, 1H), 2.36 (d, *J* = 3.4 Hz, 1H), 2.30 (d, *J* = 3.4 Hz, 1H), 1.23 (d, *J* = 6.3 Hz, 3H); ^{13}C NMR (101 MHz, $CDCl_3$) δ 134.9, 133.6, 131.6, 129.0, 128.8, 127.8, 77.6, 70.9, 19.1. HRMS (ESI-Quadrupole-Orbitrap) m/z: [M + Na]$^+$ Calcd for $C_{11}H_{13}O_2ClNa$ 235.0496, found 235.0495. $[\alpha]_D^{27}$: –1.5 (*c* 5.00, $CHCl_3$); HPLC analysis: 92% ee (Chiralcel AD-H, 8:92 ^{i}PrOH/hexanes, 1 mL/min, 254 nm), Rt (major) = 8.4 min, Rt (minor) = 7.8 min. IR (KBr thin film, cm^{-1}): ν 3569, 3436, 3084, 3024, 2959, 2874, 1499, 1284, 1213, 928, 714.

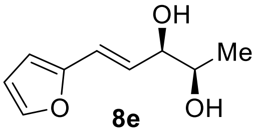

8e

(2*R*,3*R*,*E*)-5-(Furan-2-yl)pent-4-ene-2,3-diol (8e): Purified by flash chromatography on silica gel (petroleum ether/ethyl acetate, v:v = 1:1), white solid, mp 98–150 ºC, 28.2 mg, 84% yield and 8:1 dr (*syn*:*anti*). *syn*–diastereomer: ^{1}H NMR (400 MHz, $CDCl_3$) δ 7.35 (d, *J* = 1.4 Hz, 1H), 6.51 (d, *J* = 15.9 Hz, 1H), 6.37 (dd, *J* = 3.2, 1.8 Hz, 1H), 6.26 (d, *J* = 3.3 Hz, 1H), 6.12 (dd, *J* = 15.9, 6.7 Hz, 1H), 4.01–3.96 (m, 1H), 3.75–3.68 (m, 1H), 2.44 (bs, 2H), 1.22 (d, *J* = 6.3 Hz, 3H); ^{13}C NMR (101 MHz, $CDCl_3$) δ 152.1, 142.2, 126.9, 120.8, 111.4, 108.7, 77.3, 71.0, 19.0. HRMS (ESI-Quadrupole-Orbitrap) m/z: [M + Na]$^+$ Calcd for $C_9H_{12}O_3Na$ 191.0679, found 191.0677. $[\alpha]_D^{27}$: +10.1 (*c* 0.96, $CHCl_3$); HPLC analysis: 91% ee (Chiralcel AD-H, 7:93 ^{i}PrOH/hexanes, 1 mL/min, 254 nm), Rt (major) = 8.6 min, Rt (minor) = 7.9 min. IR (KBr thin film, cm^{-1}): ν 3564, 3454, 3079, 3032, 2952, 2875, 1654, 1608, 1430, 1277, 750.

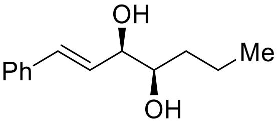

8f

(3*R*,4*R*,*E*)-1-Phenylhept-1-ene-3,4-diol (8f): Purified by flash chromatography on silica gel (petroleum ether/ethyl acetate, v:v = 2:1), yellow oil, 29.7 mg, 72% yield and >20:1 dr (*syn*:*anti*). *syn*–diastereomer: ^{1}H NMR (600 MHz, $CDCl_3$) δ 7.38 (d, *J* = 7.4 Hz, 2H), 7.32–7.29 (m, 2H), 7.27–7.23 (m, 1H), 6.65 (d, *J* = 16.0 Hz, 1H), 6.20 (dd, *J* = 16.0, 7.1 Hz, 1H), 4.08–4.05 (m, 1H), 3.58–3.55 (m, 1H), 2.42 (bs, 1H), 2.33 (bs, 1H), 1.56–1.49 (m, 2H), 1.46–1.37 (m, 2H), 0.92 (t, *J* = 7.0 Hz, 3H); ^{13}C NMR (151 MHz, $CDCl_3$) δ 136.5, 132.7, 128.7, 128.6, 128.0, 126.6, 76.4, 74.6, 35.2, 19.0, 14.2. HRMS (ESI-Quadrupole-Orbitrap) m/z: $[M + Na]^+$ Calcd for $C_{13}H_{18}O_2Na$ 229.1199, found 229.1198. $[\alpha]_D^{27}$: +5.8 (*c* 1.44, $CHCl_3$); HPLC analysis: 90% ee (Chiralcel OJ-H, 1:99 iPrOH/hexanes, 0.8 mL/min, 254 nm), Rt (major) = 64.4 min, Rt (minor) = 72.5 min. IR (KBr thin film, cm^{-1}): ν 3670, 3447, 3077, 2970, 2937, 2881, 2848, 1655, 1568, 765, 681.

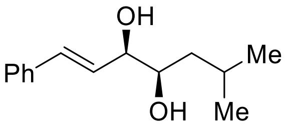

8g

(3*R*,4*R*,*E*)-6-Methyl-1-phenylhept-1-ene-3,4-diol (8g): Purified by flash chromatography on silica gel (petroleum ether/ethyl acetate, v:v = 2:1), white solid, mp 68–75 ºC, 33.5 mg, 76% yield and >20:1 dr (*syn*:*anti*). *syn*–diastereomer: ^{1}H NMR (400 MHz, $CDCl_3$) δ 7.40 (d, *J* = 7.4 Hz, 2H), 7.34–7.31 (m, 2H), 7.27–7.19 (m, 1H), 6.67 (d, *J* = 16.0 Hz, 1H), 6.20 (dd, *J* = 16.0, 7.0 Hz, 1H), 4.07–4.04 (m, 1H), 3.70–3.61 (m, 1H), 2.37 (bs, 1H), 2.25 (bs, 1H), 1.89–1.79 (m, 1H), 1.49–1.42 (m, 1H), 1.32–1.25 (m, 1H), 0.95 (d, *J* = 6.7 Hz, 3H), 0.92 (d, *J* = 6.7 Hz, 3H); ^{13}C NMR (101 MHz, $CDCl_3$) δ 136.4, 132.9, 128.7, 128.6, 128.0, 126.6, 76.8, 72.8, 42.1, 24.5, 23.8, 21.6. HRMS (ESI-Quadrupole-Orbitrap) m/z: $[M + Na]^+$ Calcd for $C_{14}H_{20}O_2Na$ 243.1356, found 243.1354. $[\alpha]_D^{27}$: –4.9 (*c* 0.80, $CHCl_3$); HPLC analysis: 92% ee (Chiralcel OD-H, 2:98 iPrOH/hexanes, 0.9 mL/min, 254 nm), R_t (major) = 42.7 min, R_t (minor) = 39.4 min. IR (KBr thin film, cm^{-1}): ν 3568, 3490, 3073, 3022, 2982, 2940, 2848, 1559, 1277, 991, 766.

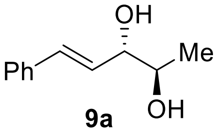

9a

(2*R*,3*S*,*E*)-5-Phenylpent-4-ene-2,3-diol (9a): Purified by flash chromatography on silica gel (petroleum ether/ethyl acetate, v:v = 1:1), colorless oil, 23.1 mg, 65% yield and 9:1 dr (*anti*:*syn*). *anti*–diastereomer: ^{1}H NMR (400 MHz, $CDCl_3$) δ 7.41–7.38 (m, 2H), 7.34–7.22 (m, 3H), 6.66 (d, *J* = 16.0 Hz, 1H), 6.27 (dd, *J* = 16.0, 5.4 Hz, 1H), 4.25 (dd, *J* = 6.7, 3.5 Hz, 1H), 3.99–3.93 (m, 1H), 2.10 (bs, 2H), 1.20 (d, *J* = 6.4 Hz,

3H); ^{13}C NMR (101 MHz, $CDCl_3$) δ 136.4, 133.1, 128.6, 128.0, 127.1, 126.6, 76.6, 70.3, 17.7. HRMS (ESI-Quadrupole-Orbitrap) m/z: $[M + Na]^+$ Calcd for $C_{11}H_{14}O_2Na$ 201.0886, found 201.0885. $[\alpha]_D^{27}$: +14.2 (*c* 0.98, $CHCl_3$); HPLC analysis: 93% *ee* (Chiralcel OD-H, 5:95 ^{i}PrOH/hexanes, 1 mL/min, 254 nm), R_t (major) = 15.0 min, R_t (minor) = 30.0 min. IR (KBr thin film, cm^{-1}): ν 3736, 3477, 3037, 2980, 2853, 1605, 1507, 1356, 1277, 1258, 764.

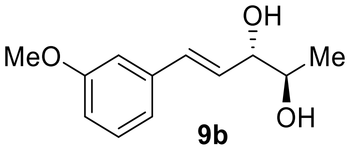

9b

(2*R*,3*S*,*E*)-5-(3-Methoxyphenyl)pent-4-ene-2,3-diol (9b): Purified by flash chromatography on silica gel (petroleum ether/ethyl acetate, v:v = 1:1), colorless oil, 20.2 mg, 49% yield and 3:1 dr (*anti*:*syn*). *anti*–diastereomer: ^1H NMR (400 MHz, $CDCl_3$) δ 7.22 (d, *J* = 7.9 Hz, 1H), 6.99 (d, *J* = 7.6 Hz, 1H), 6.93 (s, 1H), 6.81 (dd, *J* = 8.2, 2.2 Hz, 1H), 6.61 (d, *J* = 16.0 Hz, 1H), 6.25 (dd, *J* = 16.0, 7.0 Hz, 1H), 4.24 (dd, *J* = 6.8, 3.5 Hz, 1H), 3.99–3.92 (m, 1H), 3.81 (s, 3H), 2.38 (bs, 2H), 1.18 (d, *J* = 6.4 Hz, 3H); ^{13}C NMR (101 MHz, $CDCl_3$) δ 159.8, 137.9, 132.9, 129.6, 127.5, 119.3, 113.6, 111.8, 76.5, 70.4, 55.3, 17.7. HRMS (ESI-Quadrupole-Orbitrap) m/z: $[M + Na]^+$ Calcd for $C_{12}H_{16}O_3Na$ 231.0992, found 231.0993. $[\alpha]_D^{27}$: +20.8 (*c* 0.18, $CHCl_3$); HPLC analysis: 92% *ee* (Chiralcel OD-H, 10:90 ^{i}PrOH/hexanes, 0.8 mL/min, 254 nm), R_t (major) = 13.8 min, R_t (minor) = 20.6 min. IR (KBr thin film, cm^{-1}): ν 3544, 3486, 3084, 3037, 2879, 1611, 1506, 1296, 1270, 784, 730.

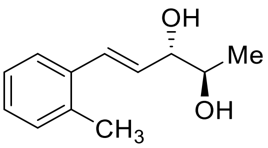

9c

(2*R*,3*S*,*E*)-5-o-Tolylpent-4-ene-2,3-diol (9c): Purified by flash chromatography on silica gel (petroleum ether/ethyl acetate, v:v = 1:1), white solid, mp 88–91 °C, 25.2 mg, 68% yield and 7:1 dr (*anti*:*syn*). *anti*–diastereomer: ^1H NMR (400 MHz, $CDCl_3$) δ 7.29 (d, *J* = 8.0 Hz, 2H), 7.12 (d, *J* = 7.9 Hz, 2H), 6.60 (d, *J* = 16.0 Hz, 1H), 6.20 (dd, *J* = 16.0, 7.2 Hz, 1H), 4.22 (dd, *J* = 7.0, 3.5 Hz, 1H), 3.99–3.91 (m, 1H), 2.47 (bs, 1H), 2.40 (bs, 1H), 2.34 (s, 3H), 1.17 (d, *J* = 6.4 Hz, 3H); ^{13}C NMR (101 MHz, $CDCl_3$) δ 137.8, 133.6, 133.0, 132.8, 129.3, 127.3, 126.5, 126.0, 76.7, 70.4, 21.2, 17.7. HRMS (ESI-Quadrupole-Orbitrap) m/z: $[M + Na]^+$ Calcd for $C_{12}H_{16}O_2Na$ 215.1043, found 215.1041. $[\alpha]_D^{27}$: +6.5 (*c* 2.05, $CHCl_3$); HPLC analysis: 90% *ee* (Chiralcel IA, 1.5:98.5 ^{i}PrOH/hexanes, 0.6 mL/min, 254 nm), R_t (major) = 110.5 min, R_t (minor) =

104.8 min. IR (KBr thin film, cm^{-1}): ν 3708, 3113, 3078, 2977, 1598, 1556, 1339, 1255, 909, 764, 691.

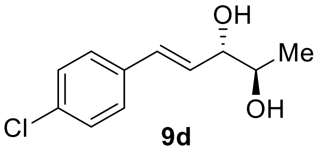

9d

(2*R*,3*S*,*E*)-5-(4-Chlorophenyl)pent-4-ene-2,3-diol (9d): Purified by flash chromatography on silica gel (petroleum ether/ethyl acetate, v:v = 1:1), white solid, mp 96–99 ºC, 26.5 mg, 63% yield and 3:1 dr (*anti*:*syn*). *anti*–diastereomer: ^{1}H NMR (400 MHz, $CDCl_3$) δ 7.33–7.27 (m, 4H), 6.61 (d, *J* = 16.0 Hz, 1H), 6.24 (dd, *J* = 16.0, 6.9 Hz, 1H), 4.25 (dd, *J* = 6.6, 3.5 Hz, 1H), 4.00–3.93 (m, 1H), 2.24 (bs, 1H), 2.14 (bs, 1H), 1.18 (d, *J* = 6.4 Hz, 3H); ^{13}C NMR (101 MHz, $CDCl_3$) δ 134.9, 133.6, 131.7, 128.8, 127.8, 76.4, 70.3, 17.7. HRMS (ESI-Quadrupole-Orbitrap) m/z: $[M + Na]^+$ Calcd for $C_{11}H_{13}O_2ClNa$ 235.0496, found 235.0495. $[\alpha]_D^{27}$: +6.8 (*c* 1.28, $CHCl_3$); HPLC analysis: 97% *ee* (Chiralcel AS-H, 3:97 ^{i}PrOH/hexanes, 0.3 mL/min, 254 nm), R_t (major) = 68.1 min, R_t (minor) = 59.9 min. IR (KBr thin film, cm^{-1}): ν 3669, 3110, 3080, 1600, 1551, 1506, 1357, 1240, 1122, 850, 730.

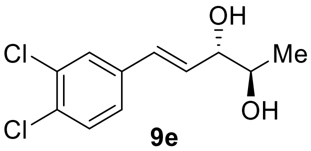

9e

(2*R*,3*S*,*E*)-5-(3,4-Dichlorophenyl)pent-4-ene-2,3-diol (9e): Purified by flash chromatography on silica gel (petroleum ether/ethyl acetate, v:v = 2:1), colorless oil, 33.0 mg, 67% yield and 4:1 dr (*anti*:*syn*). *anti*–diastereomer: ^{1}H NMR (400 MHz, $CDCl_3$) δ 7.46–7.44 (m, 1H), 7.37 (d, *J* = 8.3 Hz, 1H), 7.20–7.18 (m, 1H), 6.55 (d, *J* = 15.9 Hz, 1H), 6.25 (dd, *J* = 16.0, 6.6 Hz, 1H), 4.25 (dd, *J* = 5.9, 3.4 Hz, 1H), 3.99–3.93 (m, 1H), 2.46 (bs, 1H), 2.29 (bs, 1H), 1.17 (d, *J* = 6.5 Hz, 3H); ^{13}C NMR (151 MHz, $CDCl_3$) δ 136.6, 132.8, 131.6, 130.6, 130.5, 129.3, 128.3, 125.8, 76.1, 70.4, 17.8. HRMS (ESI-Quadrupole-Orbitrap) m/z: $[M + Na]^+$ Calcd for $C_{11}H_{12}O_2Cl_2Na$ 269.1007, found 269.0105. $[\alpha]_D^{27}$: +42.0 (*c* 0.27, $CHCl_3$); HPLC analysis: 91% *ee* (Chiralcel IC, 5:95 ^{i}PrOH/hexanes, 1 mL/min, 254 nm), R_t (major) = 8.1 min, R_t (minor) = 9.0 min. IR (KBr thin film, cm^{-1}): ν 3690, 3391, 3079, 2971, 2844, 1609, 1524, 1461, 1299, 1271, 785.

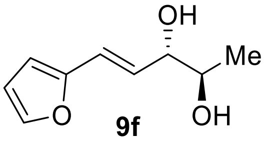

9f

(2*R*,3*S*,*E*)-5-(Furan-2-yl)pent-4-ene-2,3-diol (9f): Purified by flash chromatography on silica gel (petroleum ether/ethyl acetate, v:v = 1:1), yellow oil, 21.2 mg, 63% yield and 5:1 dr (*anti*:*syn*). *anti*–diastereomer: ^{1}H NMR (400 MHz, $CDCl_3$) δ 7.34 (s, 1H), 6.47 (d, *J* = 15.9 Hz, 1H), 6.36–6.35 (m, 1H), 6.25 (d, *J* = 3.2 Hz, 1H), 6.17 (dd, *J* = 16.0, 6.6 Hz, 1H), 4.22 (dd, *J* = 6.4, 3.5 Hz, 1H), 3.95–3.89 (m, 1H), 2.49 (bs, 2H), 1.17 (d, *J* = 6.4 Hz, 3H); ^{13}C NMR (101 MHz, $CDCl_3$) δ 152.2, 142.1, 125.7, 120.9, 111.4, 108.4, 76.0, 70.4, 17.6. HRMS (ESI-Quadrupole-Orbitrap) m/z: $[M + Na]^+$ Calcd for $C_9H_{12}O_3Na$ 191.0679, found 191.0677. $[\alpha]_D^{27}$: +33.7 (*c* 1.13, $CHCl_3$); HPLC analysis: 90% *ee* (Chiralcel OD-H, 6:94 ^{i}PrOH/hexanes, 0.5 mL/min, 254 nm), R_t (major) = 18.0 min, R_t (minor) = 21.5 min. IR (KBr thin film, cm^{-1}): ν 3518, 3450, 3082, 3028, 2947, 1611, 1484, 1406, 1294, 1270, 784.

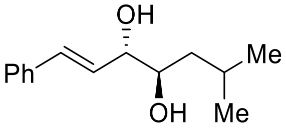

9g

(3*S*,4*R*,*E*)-6-Methyl-1-phenylhept-1-ene-3,4-diol (9g): Purified by flash chromatography on silica gel (petroleum ether/ethyl acetate, v:v = 2:1), white solid, mp 113–119 ºC, 25.1 mg, 57% yield and 5:1 dr (*anti*:*syn*). *anti*–diastereomer: ^{1}H NMR (400 MHz, $CDCl_3$) δ 7.41 (d, *J* = 7.4 Hz, 2H), 7.34–7.31 (m, 2H), 7.27–7.24 (m, 1H), 6.65 (d, *J* = 16.0 Hz, 1H), 6.30 (dd, *J* = 16.0, 7.1 Hz, 1H), 4.24 (dd, *J* = 6.5, 2.9 Hz, 1H), 3.92–3.85 (m, 1H), 2.14 (bs, 1H), 2.02 (bs, 1H), 1.87–1.77 (m, 1H), 1.45–1.37 (m, 1H), 1.28–1.19 (m, 1H), 0.95 (d, *J* = 6.7, 3H), 0.92 (d, *J* = 6.7, 3H); ^{13}C NMR (101 MHz, $CDCl_3$) δ 135.4, 132.1, 127.6, 126.9, 126.0, 125.6, 75.2, 71.4, 40.1, 23.5, 22.6, 20.8. HRMS (ESI-Quadrupole-Orbitrap) m/z: $[M + Na]^+$ Calcd for $C_{14}H_{20}O_2Na$ 243.1356, found 243.1354. $[\alpha]_D^{27}$: +18.5 (*c* 0.22, $CHCl_3$); HPLC analysis: 87% *ee* (Chiralcel OD-H, 6:94 ^{i}PrOH/hexanes, 0.8 mL/min, 254 nm), R_t (major) = 11.4 min, R_t (minor) = 18.5 min. IR (KBr thin film, cm^{-1}): ν 3710, 3102, 3075, 2949, 1601, 1539, 1393, 1356, 1248, 938, 884.

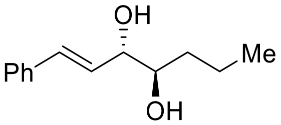

9h

(3*S*,4*R*,*E*)-1-Phenylhept-1-ene-3,4-diol (9h): Purified by flash chromatography on silica gel (petroleum ether/ethyl acetate, v:v = 2:1), white solid, mp 121–124 ºC, 26.8 mg, 65% yield and 5:1 dr (*anti*:*syn*). *anti*–diastereomer: ^{1}H NMR (400 MHz, $CDCl_3$) δ 7.40 (d, *J* = 7.4 Hz, 2H), 7.34–7.30 (m, 2H), 7.27–7.23 (m, 1H), 6.64 (d, *J* = 16.0

Hz, 1H), 6.30 (dd, J = 16.0, 7.1 Hz, 1H), 4.26 (dd, J = 7.1, 2.8 Hz, 1H), 3.83–3.74 (m, 1H), 2.39 (bs, 1H), 2.24 (bs, 1H), 1.58–1.48 (m, 1H), 1.46–1.33 (m, 3H), 0.93 (t, J = 7.1, 3H); ^{13}C NMR (101 MHz, $CDCl_3$) δ 136.5, 133.0, 128.6, 127.9, 127.1, 126.6, 76.0, 74.2, 34.4, 19.1, 14.1. HRMS (ESI-Quadrupole-Orbitrap) m/z: $[M + Na]^+$ Calcd for $C_{13}H_{18}O_2Na$ 229.1199, found 229.1198. $[\alpha]_D^{27}$: +14.5 (c 0.99, $CHCl_3$); HPLC analysis: 85% *ee* (Chiralcel OD-H, 5:95 ^{i}PrOH/hexanes, 0.8 mL/min, 254 nm), R_t (major) = 14.5 min, R_t (minor) = 23.6 min. IR (KBr thin film, cm^{-1}): ν 3646, 3480, 3075, 3032, 2967, 1604, 1519, 1356, 1298, 1270, 783.

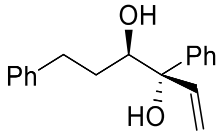

10a

(3S,4R)-3,6-Diphenylhex-1-ene-3,4-diol (10a): purified by chromatography on flash silica gel (petroleum ether/ethyl acetate, v:v = 1:1), white solid, mp 78–80 ºC, 52.8 mg, 82% yield and >20:1 dr (*anti*:*syn*). *anti*–diastereomer: ^{1}H NMR (400 MHz, $CDCl_3$) δ 7.37–7.30 (m, 4H), 7.26–7.21 (m, 3H), 7.17–7.13 (m, 1H), 7.07 (d, J = 7.2 Hz, 2H), 6.30 (dd, J = 17.2, 10.7 Hz, 1H), 5.43 (d, J = 17.2 Hz, 1H), 5.28 (d, J = 10.7 Hz, 1H), 3.85 (d, J = 10.0 Hz, 1H), 2.84–2.77 (m, 1H), 2.60–2.50 (m, 2H), 1.97 (bs, 1H), 1.71–1.62 (m, 1H), 1.54–1.48 (m, 1H); ^{13}C NMR (151 MHz, $CDCl_3$) δ 142.4, 142.3, 141.9, 128.6, 128.5, 128.4, 127.2, 125.9, 125.4, 114.9, 79.1, 75.8, 32.4, 31.5. HRMS (ESI-Quadrupole-Orbitrap) m/z: $[M + Na]^+$ Calcd for $C_{18}H_{20}O_2Na$ 291.1356, found 291.1357. $[\alpha]_D^{27}$: +12.9 (c 0.33, $CHCl_3$); HPLC analysis: 96% *ee* (Chiralcel IA, 5:95 ^{i}PrOH/hexanes, 0.8 mL/min, 220 nm), R_t (major) = 16.2 min, R_t (minor) = 11.5 min. IR (KBr thin film, cm^{-1}): ν 3649, 3350, 3085, 3029, 2860, 1628, 1559, 1507, 1276, 1261,750.

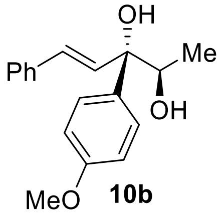

10b

(2*R*,3*R*,*E*)-3-(4-Methoxyphenyl)-5-phenylpent-4-ene-2,3-diol (10b): purified by chromatography on silica gel (petroleum ether/ethyl acetate, v:v = 5:1), colorless oil, 9.0 mg, 74% yield and 19:1 dr (*anti*:*syn*). *anti*–diastereomer: ^{1}H NMR (400 MHz, $CDCl_3$) δ 7.49 (d, J = 8.7 Hz, 2H), 7.40 (d, J = 7.4 Hz, 2H), 7.33–7.29 (m, 2H), 7.24–7.21 (m, 1H), 6.92 (d, J = 8.8 Hz, 2H), 6.77 (d, J = 16.0 Hz, 1H), 6.56 (d, J = 16.0 Hz, 1H), 4.23–4.17 (m, 1H), 3.81 (s, 3H), 2.79 (s, 1H), 1.81 (d, J = 4.4 Hz, 1H), 1.24 (d, J

= 6.4 Hz, 3H); ^{13}C NMR (151 MHz, $CDCl_3$) δ 159.0, 136.9, 135.8, 130.9, 130.3, 128.6, 127.8, 127.4, 126.7, 114.0, 79.1, 73.8, 55.4, 17.1. HRMS (ESI-Quadrupole-Orbitrap) m/z: $[M + Na]^+$ Calcd for $C_{18}H_{20}O_3Na$ 307.1305, found 307.1307. $[\alpha]_D^{27}$: +7.9 (*c* 0.34, $CHCl_3$); HPLC analysis: 94% *ee* (Chiralcel OJ-H, 10:90 ^{i}PrOH/hexanes, 0.8 mL/min, 254 nm), R_t (major) = 27.1 min, R_t (minor) = 24.5 min. IR (KBr thin film, cm^{-1}): ν 3743, 3566, 3032, 2987, 2829, 1617, 1540, 1507, 1364, 1276, 1259.

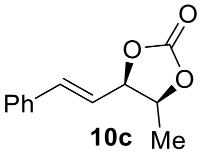

10c

(4*S*,5*R*)-4-Methyl-5-styryl-1,3-dioxolan-2-one (10c): purified by chromatography on flash silica gel (petroleum ether/ethyl acetate, v:v = 8:1), colorless oil, 9.0 mg, 74% yield and 9:1 dr (*syn*:*anti*). *syn*–diastereomer: ^{1}H NMR (400 MHz, $CDCl_3$) δ 7.42–7.29 (m, 5H), 6.77 (d, *J* = 15.8 Hz, 1H), 6.15 (dd, *J* = 15.8, 7.9 Hz, 1H), 4.76 (t, *J* = 7.9 Hz, 1H), 4.56–4.49 (m, 1H), 1.51 (d, *J* = 6.2 Hz, 3H); ^{13}C NMR (101 MHz, $CDCl_3$) δ 154.3, 137.0, 134.9, 129.2, 128.9, 127.0, 121.8, 84.6, 78.7, 18.0. HRMS (ESI-Quadrupole-Orbitrap) m/z: $[M + H]^+$ Calcd for $C_{12}H_{13}O_3$ 205.0859, found 205.0860. $[\alpha]_D^{27}$: +13.7 (*c* 1.14, $CHCl_3$); HPLC analysis: >99% *ee* (Chiralcel IC, 5:95 ^{i}PrOH/hexanes, 1.0 mL/min, 254 nm), R_t (major) = 18.8 min, R_t (minor) = 30.5 min. IR (KBr thin film, cm^{-1}): ν 3629, 3389, 3047, 2868, 1802, 1613, 1558, 1365, 1276, 1189, 764.

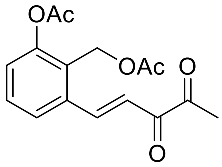

15

(*E*)-2-Acetoxy-6-(3,4-dioxopent-1-en-1-yl)benzyl acetate (15): Purified by chromatography on silica gel (petroleum ether/ethyl acetate, v:v = 3:1), yellow solid, 1.73 g, 95% yield. ^{1}H NMR (400 MHz, $CDCl_3$) δ 8.20 (d, *J* = 16.0 Hz, 1H), 7.65 (d, *J* = 7.8 Hz, 1H), 7.48–7.36 (m, 2H), 7.18 (d, *J* = 8.0 Hz, 1H), 5.24 (s, 2H), 2.46 (s, 3H), 2.36 (s, 3H), 2.04 (s, 3H); ^{13}C NMR (101 MHz, $CDCl_3$) δ 198.5, 186.4, 170.5, 169.4, 150.2, 143.5, 136.6, 130.0, 127.9, 125.2, 124.9, 121.3, 56.8, 24.4, 20.9, 20.7. HRMS (ESI-Quadrupole-Orbitrap) m/z: $[M + Na]^+$ Calcd for $C_{16}H_{16}O_6Na$ 327.0844, found 327.0843. IR: ν 3734, 3628, 2360, 2342, 1734, 1717, 1472, 1457, 1374, 764, 735, 669.

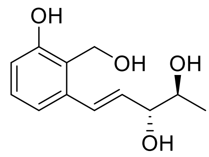

anti-sordariol

(2*S*,3*R*,*E*)-5-(3-Hydroxy-2-(hydroxymethyl)phenyl)pent-4-ene-2,3-diol (*anti*-sordariol) : Purified by chromatography on silica gel ($CH_3OH/CHCl_2$, v:v = 1:7), colorless oil, 42.65 mg, 95% yield and 5:1 dr (*anti*:*syn*). *anti*–diastereomer: ^{1}H NMR (400 MHz, CD_3OD) δ 7.07 (t, *J* = 7.8, 1H), 7.03 (brd, *J* = 8.0 Hz, 1H), 6.99 (brd, *J* = 9.4 Hz, 1H), 6.72 (d, *J* = 7.8 Hz, 1H), 6.18 (dd, *J* = 15.8, 6.8 Hz, 1H), 4.78 (s, 2H), 4.07 (ddd, *J* = 6.2, 5.6, 0.6 Hz, 1H), 3.77 (dq, *J* = 11.4, 6.1Hz, 1H), 1.20 (d, *J* = 6.4 Hz, 3H); ^{13}C NMR (101 MHz, MeOD) δ 157.3, 139.7, 132.5, 130.6, 129.7, 125.2, 118.6, 115.4, 78.0, 71.7, 56.5, 18.9. HRMS (ESI-Quadrupole-Orbitrap) m/z: $[M + Na]^+$ Calcd for $C_{12}H_{16}O_4Na$ 247.0941, found 247.0971[6]. $[\alpha]_D^{27}$: +20.0 (*c* 1.45, $CHCl_3$); HPLC analysis: 91% *ee* (Chiralcel AD-H, 15:85 ^{i}PrOH/hexanes, 1 mL/min, 254 nm), R_t (major) = 8.8 min, R_t (minor) = 7.7 min. IR: ν 3675, 2901, 1684, 1540, 1472, 1251, 1066, 1027, 896, 669, 516.

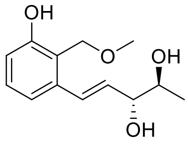

12-methoxy sordariol

(2*S*,3*R*,*E*)-5-(3-Hydroxy-2-(methoxymethyl)phenyl)pent-4-ene-2,3-diol (12-methoxy sordariol): Purified by chromatography on silica gel (petroleum ether/ethyl acetate, v:v = 1:8), colorless oil, 12.1 mg, 51% yield and 8:1 dr (*anti*:*syn*). *anti*–diastereomer: ^{1}H NMR (400 MHz, CD_3OD) δ 7.10 (t, *J* = 7.8 Hz, 1H), 7.03 (d, *J* = 7.8 Hz, 1H), 6.94 (d, *J* = 15.8 Hz, 1H), 6.72 (d, *J* = 8.0 Hz, 1H), 6.19 (dd, *J* = 15.8, 6.8 Hz, 1H), 4.62 (s, 2H), 4.06 (ddd, *J* = 5.7, 5.0, 0.7 Hz, 1H), 3.76 (dq, *J* = 5.0, 6.4 Hz, 1H), 3.37 (s, 3H), 1.20 (d, *J* = 6.4 Hz, 3H); ^{13}C NMR (101 MHz, MeOD) δ 157.7, 140.7, 132.7, 130.6, 130.3, 122.5, 118.7, 115.3, 78.1, 71.8, 66.3, 58.1, 18.9. HRMS (ESI-Quadrupole-Orbitrap) m/z: $[M + Na]^+$ Calcd for $C_{13}H_{18}O_4Na$ 261.1103, found 261.1103[6]. $[\alpha]_D^{27}$: +24.0 (*c* 2.01, $CHCl_3$); HPLC analysis: 98% *ee* (Chiralcel IE, 5:95 ^{i}PrOH/hexanes, 1 mL/min, 254 nm), R_t (major) = 83.8 min, R_t (minor) = 87.7 min. IR: ν 3675, 2988, 2901, 2359, 2342, 1653, 1540, 1507, 1457, 1264, 1066, 896, 703.

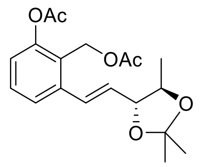

17-1

2-Acetoxy-6-((*E*)-2-((4*R*,5*R*)-2,2,5-trimethyl-1,3-dioxolan-4-yl)vinyl)benzyl acetate (17-1): Purified by chromatography on silica gel (petroleum ether/ethyl acetate, v:v = 4:1), colorless oil, 66.12 mg, 95% yield and 4:1 dr (*anti*:*syn*). *anti*–diastereomer: ^{1}H NMR (400 MHz, $CDCl_3$) δ 7.42–7.27 (m, 2H), 7.02 (d, *J* = 7.1 Hz, 1H), 6.94 (d, *J* = 15.7 Hz, 1H), 6.08 (dd, *J* = 15.7, 7.8 Hz, 1H), 5.16 (s, 2H), 4.70 (t, *J* = 7.0 Hz, 1H), 4.44–4.36 (m, 1H), 2.34 (s, 3H), 2.03 (s, 3H), 1.54 (s, 3H), 1.41 (s, 3H), 1.21 (d, *J* = 6.4 Hz, 3H); ^{13}C NMR (101 MHz, $CDCl_3$) δ 170.8, 169.7, 150.1, 139.4, 130.5, 129.9, 129.5, 125.3, 124.7, 122.2, 108.4, 79.6, 74.5, 57.5, 28.3, 25.7, 21.0, 20.9, 16.3. HRMS (ESI-Quadrupole-Orbitrap) m/z: $[M + Na]^+$ Calcd for $C_{19}H_{24}O_6Na$ 371.1464, found 371.1472. $[\alpha]_D^{27}$: −3.7 (*c* 29.9, $CHCl_3$); HPLC analysis: 94% *ee* (Chiralcel AD-H, 5:95 ^{i}PrOH/hexanes, 1 mL/min, 220 nm), R_t (major) = 5.2 min, R_t (minor) = 6.6 min. IR: ν 3567, 2987, 1739, 1457, 1371, 1224, 1187, 1066, 971, 931, 862, 739, 669.

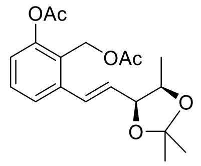

18-1

2-Acetoxy-6-((*E*)-2-((4*S*,5*R*)-2,2,5-trimethyl-1,3-dioxolan-4-yl)vinyl)benzyl acetate (18-1): Purified by chromatography on silica gel (petroleum ether/ethyl acetate, v:v = 4:1), colorless oil, 59.16 mg, 84% yield and 4:1 dr (*syn*:*anti*). *syn*–diastereomer: ^{1}H NMR (400 MHz, $CDCl_3$) δ 7.45–7.30 (m, 2H), 7.05–6.95 (m, 2H), 6.09 (dd, *J* = 15.6, 7.4 Hz, 1H), 5.16 (s, 2H), 4.12 (t, *J* = 7.9 Hz, 1H), 3.92–3.84 (m, 1H), 2.34 (s, 3H), 2.03 (s, 3H), 1.46 (d, *J* = 6.4 Hz, 6H), 1.32 (d, *J* = 6.1 Hz, 3H); ^{13}C NMR (101 MHz, $CDCl_3$) δ 170.7, 169.6, 150.0, 139.0, 130.3, 130.1, 129.8, 125.3, 124.5, 122.2, 108.7, 83.7, 76.8, 57.4, 27.4, 26.9, 20.9, 20.8, 16.5. HRMS (ESI-Quadrupole-Orbitrap) m/z: $[M + Na]^+$ Calcd for $C_{19}H_{24}O_6Na$ 371.1464, found 371.1472. $[\alpha]_D^{27}$: +3.5 (*c* 30.7, $CHCl_3$); HPLC analysis: 91% *ee* (Chiralcel AD-H, 5:95 ^{i}PrOH/hexanes, 0.5 mL/min, 220 nm), R_t (major) = 8.6 min, R_t (minor) = 9.4 min. IR: ν 3675, 2987, 1769, 1457, 1372, 1223, 1187, 1027, 971, 933, 859, 740, 669.

IX. Reference

[1] Liu, J.; Das, D. K.; Zhang, G.; Yang, S.; Zhang, H.; Fang, X. *Org. Lett.* **2018**, *20*, 64−67.

[2] Li, X.; Kong, X.; Yang, S.; Meng, M.; Zhan, X.; Fang, X. *Org. Lett.* **2019**, *21*, 1979−1983.

[3] Liu, W.; Niu, S.; Zhao, Z.; Yang, S.; Liu, J.; Li, Y.; Fang, X. *Org. Lett*. **2020**, *22*, 7572−7576.

[4] Gsco, B.; Eric, L.; Marcus, A. T. *Org. Lett*. **2003**, *5*, 4927−4930.

[5] Knölker, H. J.; Bauermeister, M. *Helv. Chim. Acta.* **1993**, *76*, 2500−2514.

[6] Mahesh, G.; Raghavaiah, J.; Sudhakar, G. *Tetrahedron* **2020**, *76*, 1−37.

[7] Li, T.; Wang, X.; Luo, J.; Yang, M; Kong, L. *Tetrahedron Lett.* **2016**, *57*, 2754–2757.

[8] Lee, S.; Jang, J.; Hwang, G.; Ryu, D. *J. Org. Chem*. **2013**, *78*, 770−775.

[9] Niu, S.; Zhang, H.; Xu, W.; Bagdi, P.; Zhang, G.; Liu, J.; Yang, S.; Fang, X. *Nature Commun*. **2021**, 12: 3735.

[10] Tao, Z.; Robb, K.; Panger, J.; Denmark, S. *J. Am. Chem. Soc.* **2018**, *140*, 15621−15625.

[11] Markovic, M.; Koós, P.; Sokoliová, S.; Bohácikovà, N.; Moncol, J.; Gracza, T. *J. Org. Chem*. **2022**, 10.1021/acs.joc.2c02092.

[12] Chen, T.; Liu, W.; Gu, W.; Niu, S.; Lan, S.; Zhao, Z.; Gong, F.; Liu, J.; Yang, S.; Cotman, A. E.; Song, J.; Fang, X. *J. Am. Chem. Soc.* **2023**, *145*, 585−599.

X. ^{1}H NMR and ^{13}C NMR spectra of new compounds

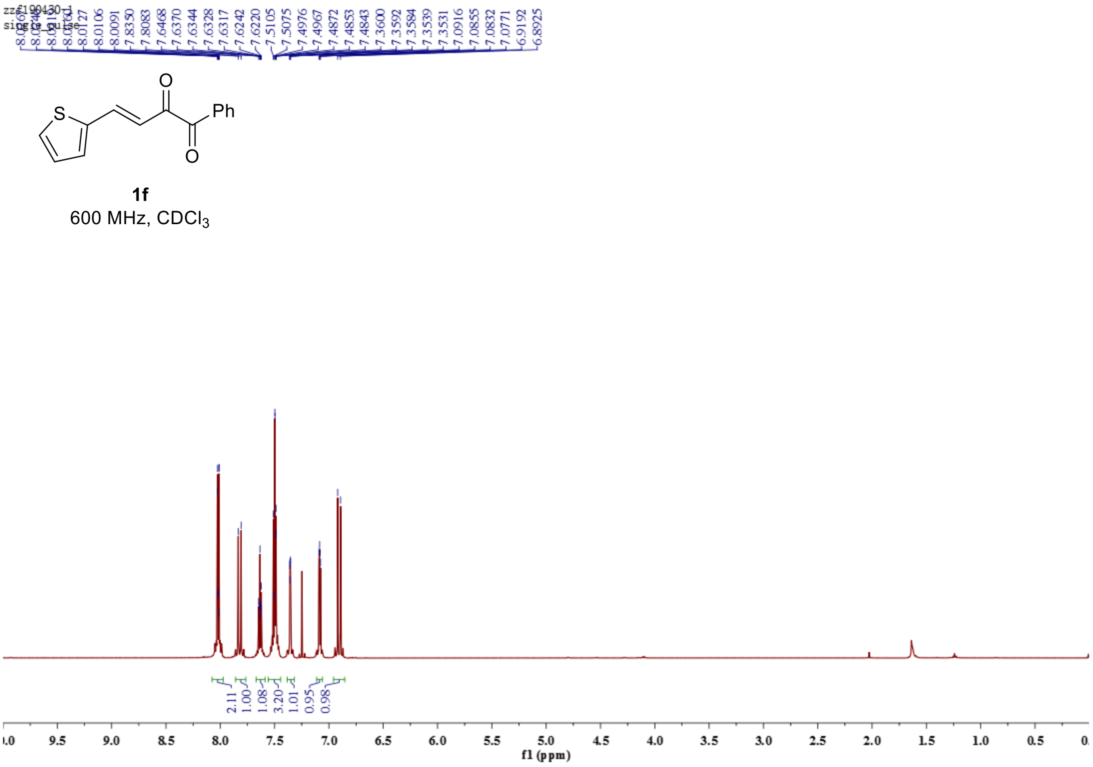

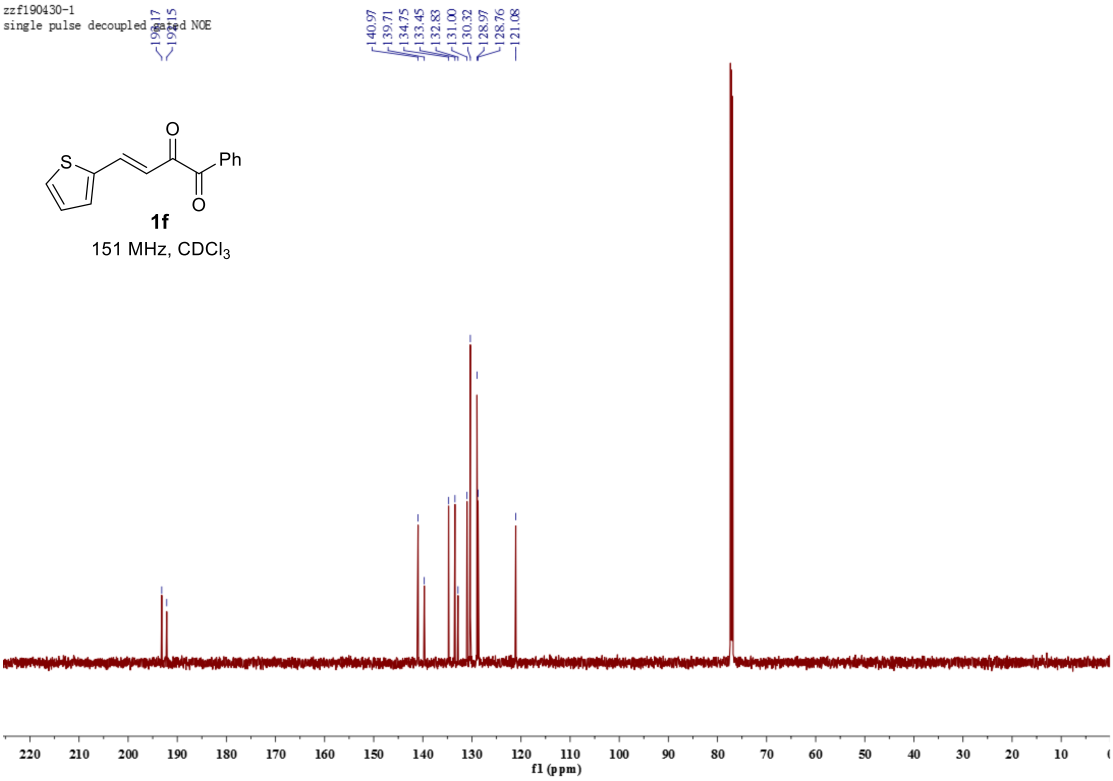

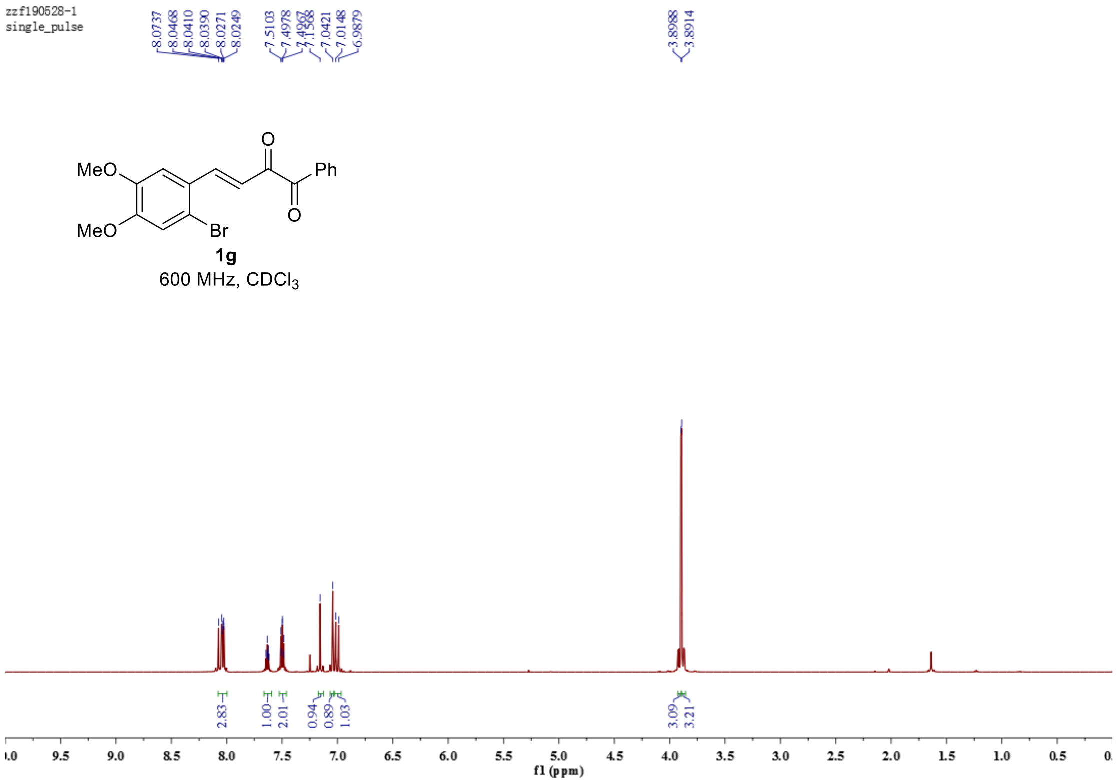

1g
600 MHz, $CDCl_3$

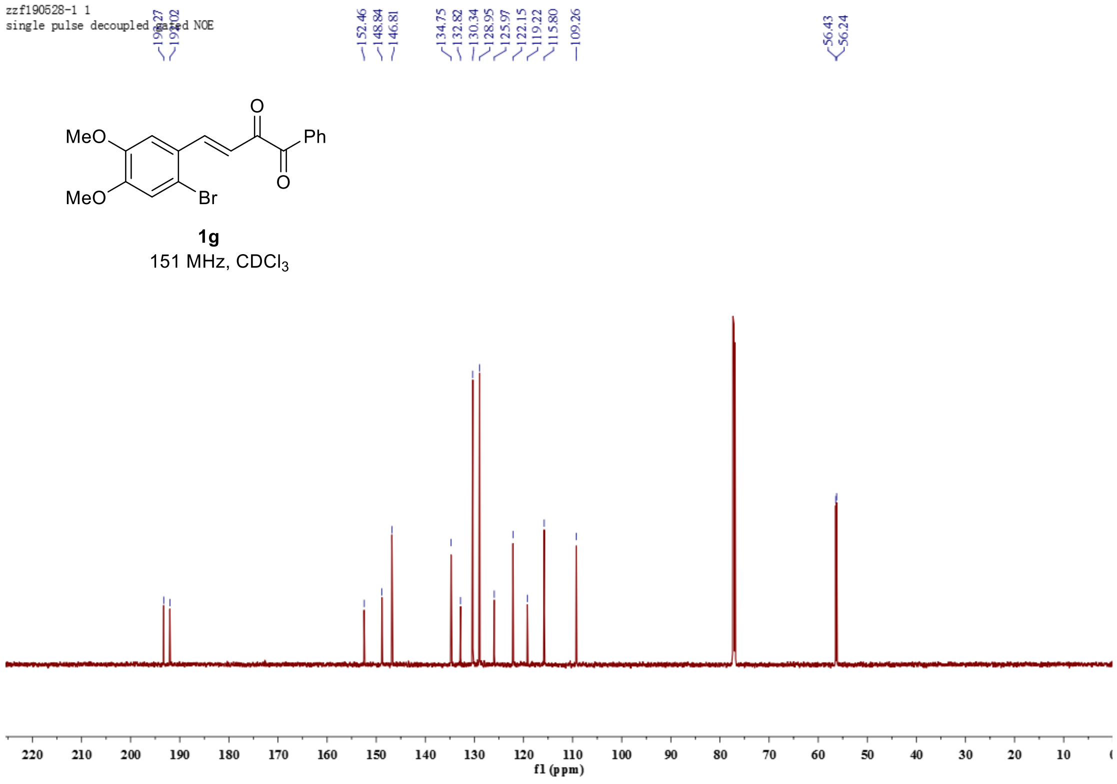

1g
151 MHz, $CDCl_3$

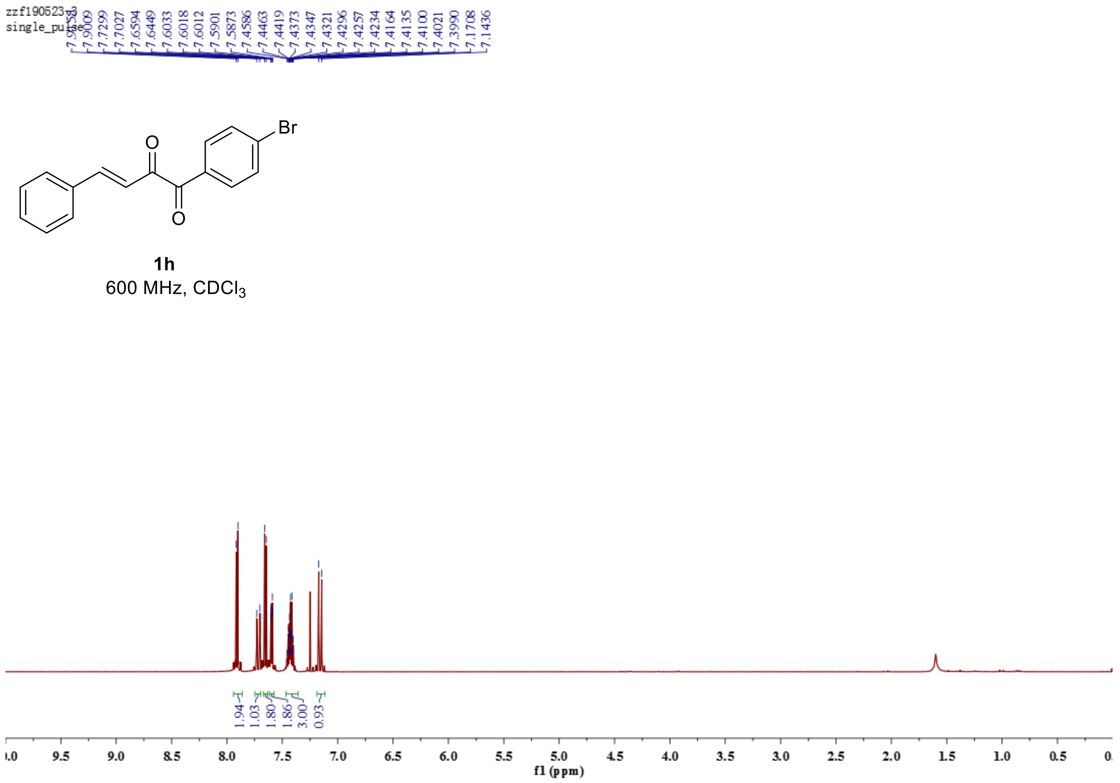
zzf190523-3
single_pulse
7.9009
7.7299
7.7027
7.6594
7.6449
7.6033
7.6018
7.6012
7.5901
7.5873
7.4586
7.4463
7.4419
7.4373
7.4347
7.4321
7.4296
7.4257
7.4234
7.4164
7.4135
7.4100
7.4021
7.3990
7.1708
7.1436
Br
O
O
1h
600 MHz, CDCl3
1.94
1.03
1.80
1.86
3.00
0.93
f1 (ppm)

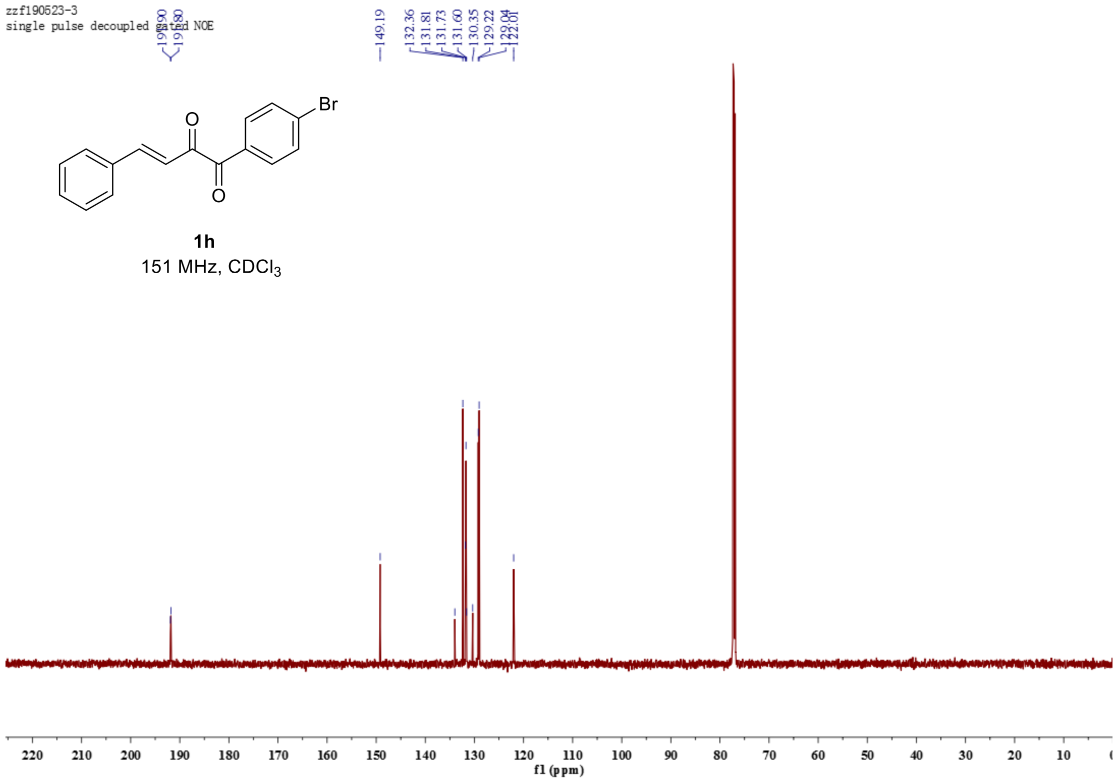
zzf190523-3
single pulse decoupled gated NOE
149.19
132.36
131.81
131.73
131.60
130.35
129.22
122.01
Br
O
O
1h
151 MHz, CDCl3
f1 (ppm)

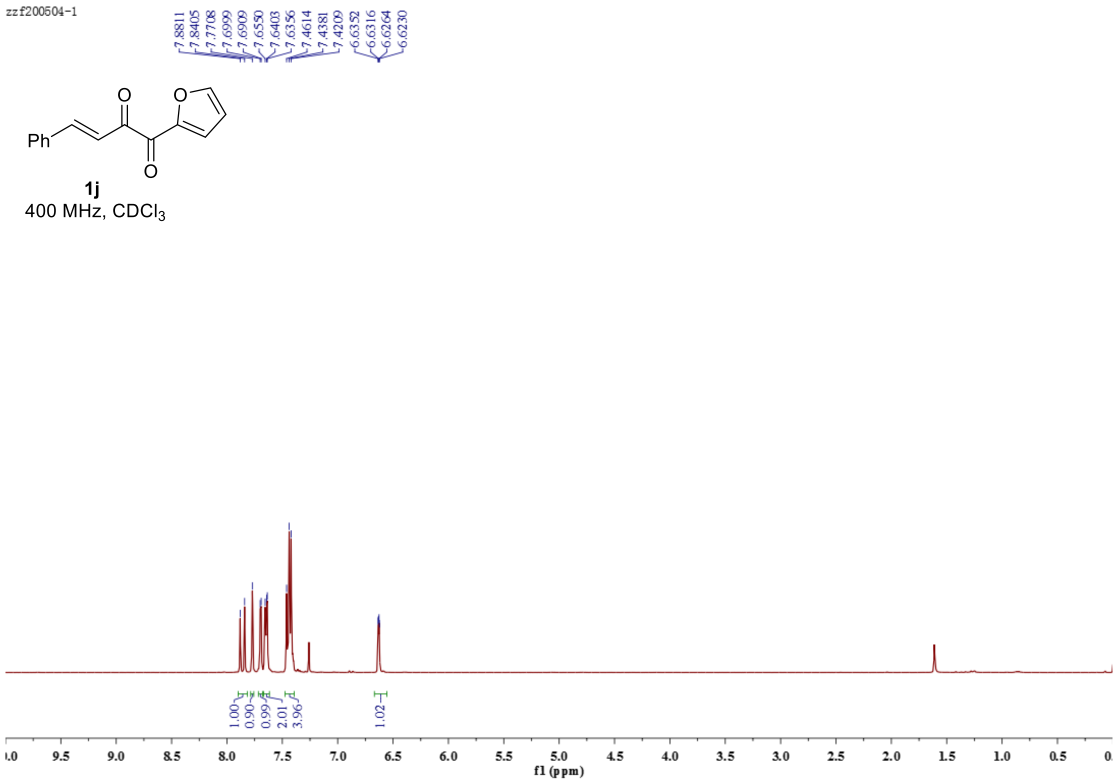

1j
400 MHz, $CDCl_3$

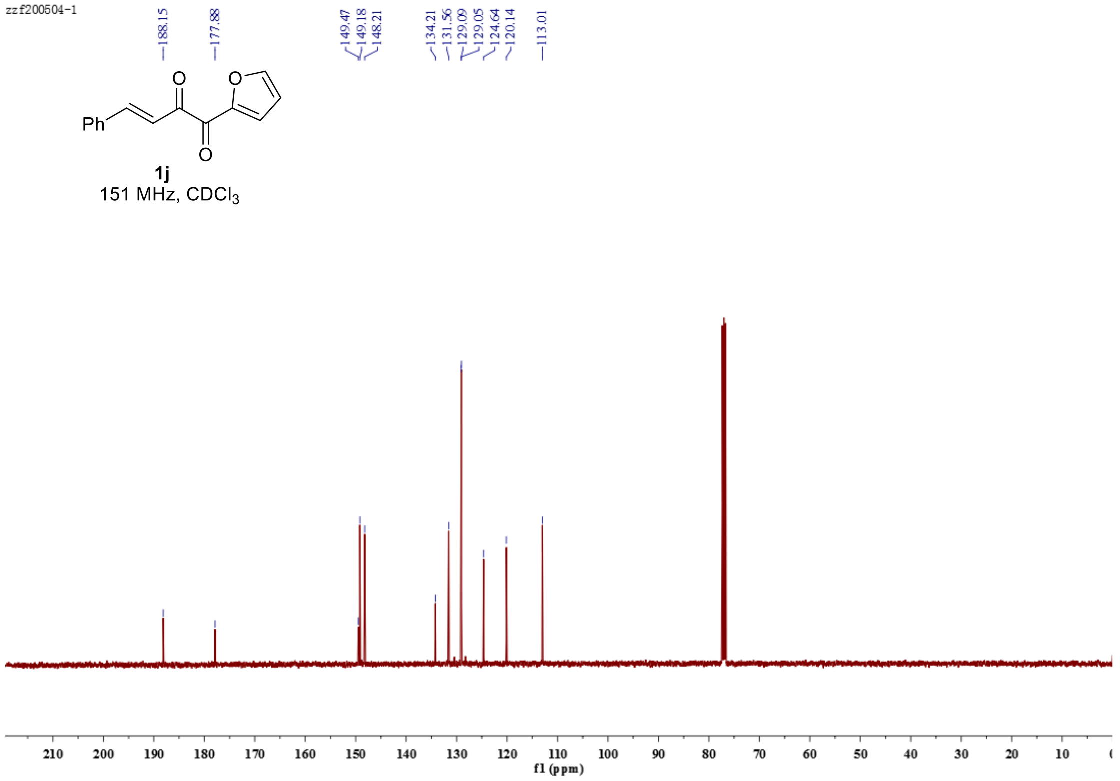

1j
151 MHz, $CDCl_3$

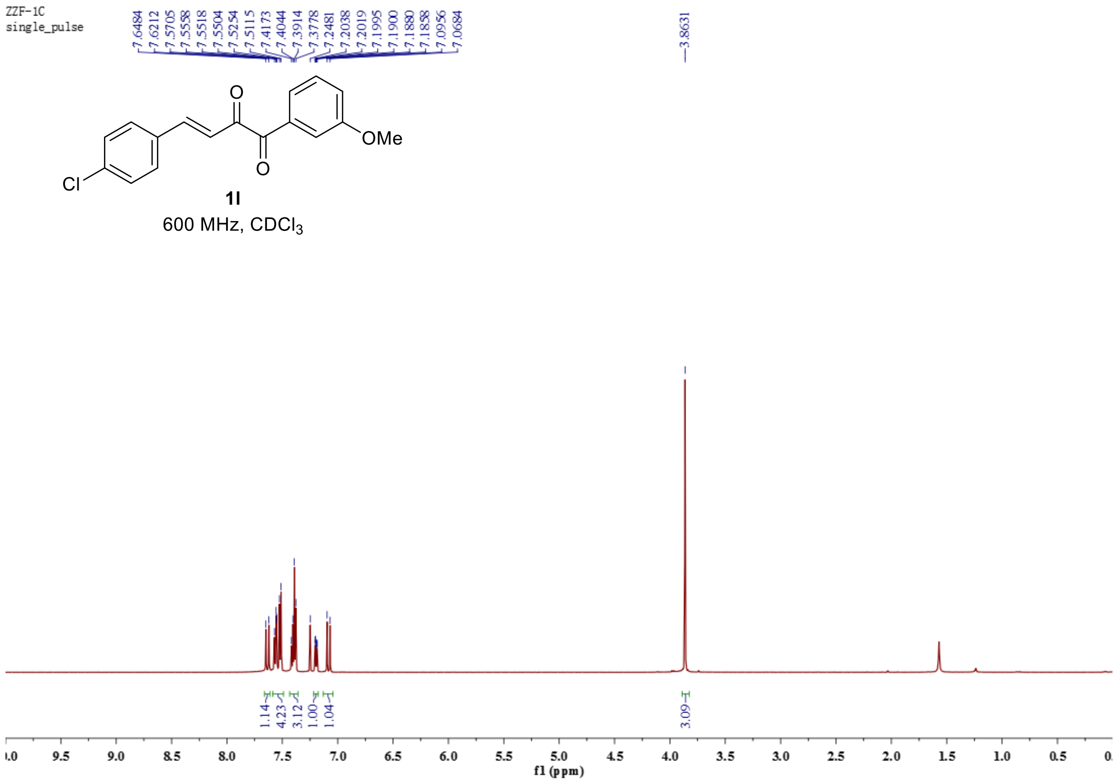
ZZF-1C
single_pulse
1l
600 MHz, CDCl3
OMe
Cl
f1 (ppm)

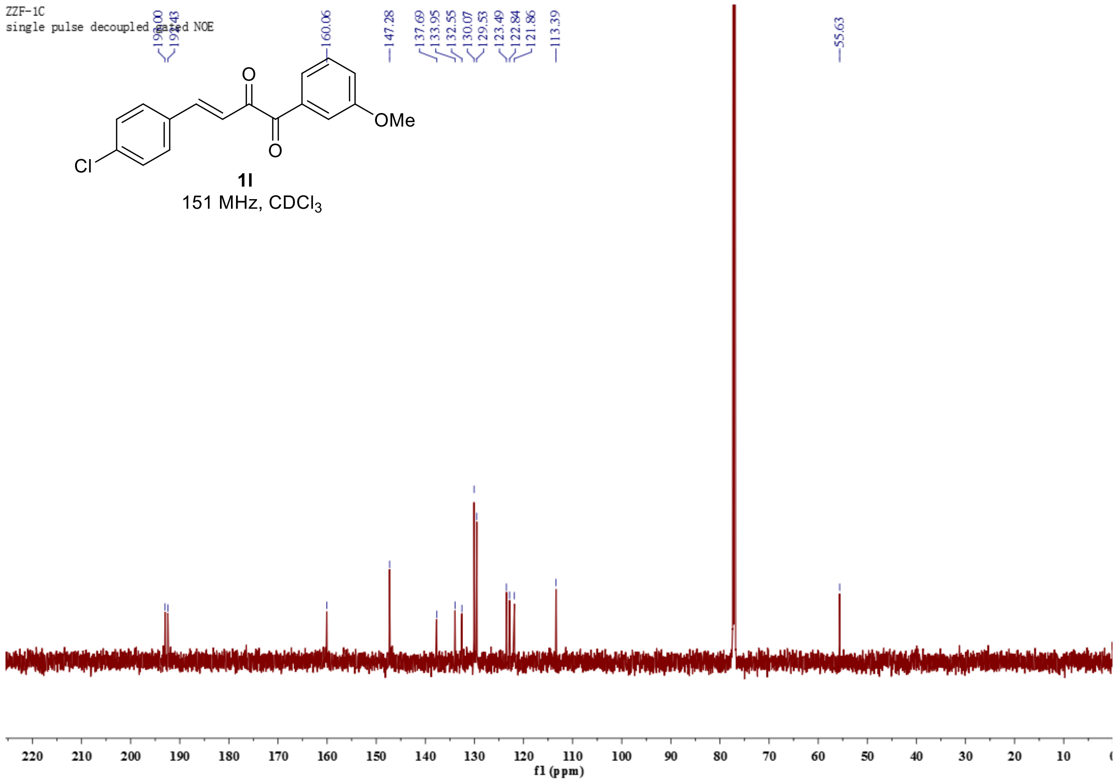
ZZF-1C
single pulse decoupled gated NOE
1l
151 MHz, CDCl3
OMe
Cl
f1 (ppm)

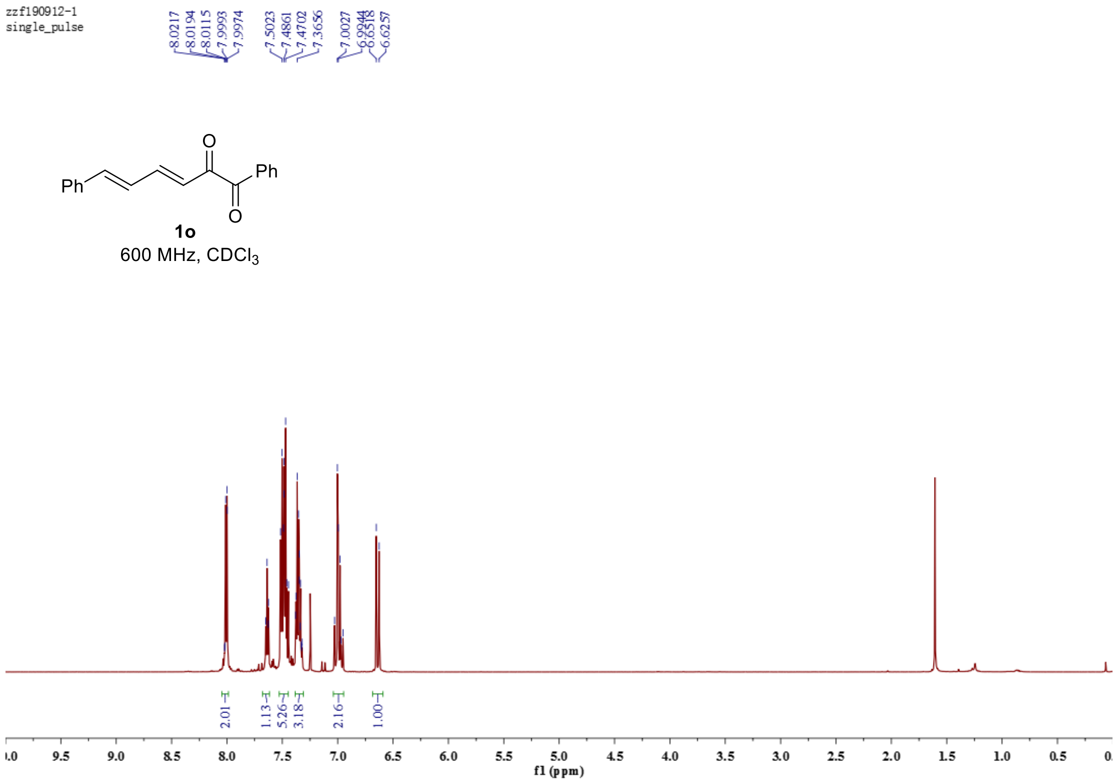

zzf190912-1
single_pulse
8.0217
8.0194
8.0115
7.9993
7.9974
7.5023
7.4861
7.4702
7.3656
7.0027
6.9944
6.6518
6.6257
Ph
O
Ph
O
1o
600 MHz, CDCl3
2.01
1.13
5.26
3.18
2.16
1.00
10.0 9.5 9.0 8.5 8.0 7.5 7.0 6.5 6.0 5.5 5.0 4.5 4.0 3.5 3.0 2.5 2.0 1.5 1.0 0.5 0
f1 (ppm)

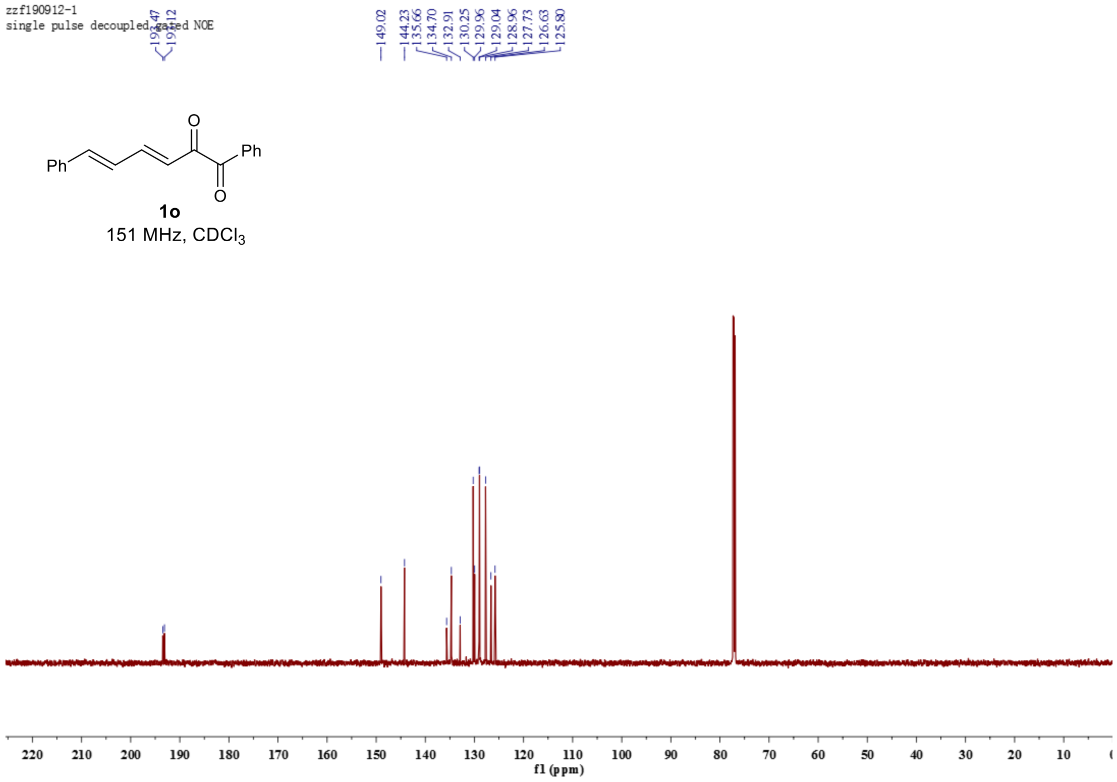

zzf190912-1
single pulse decoupled gated NOE
193.47
193.12
149.02
144.23
135.66
134.70
132.91
130.25
129.96
129.04
128.96
127.73
126.63
125.80
Ph
O
Ph
O
1o
151 MHz, CDCl3
220 210 200 190 180 170 160 150 140 130 120 110 100 90 80 70 60 50 40 30 20 10 0
f1 (ppm)

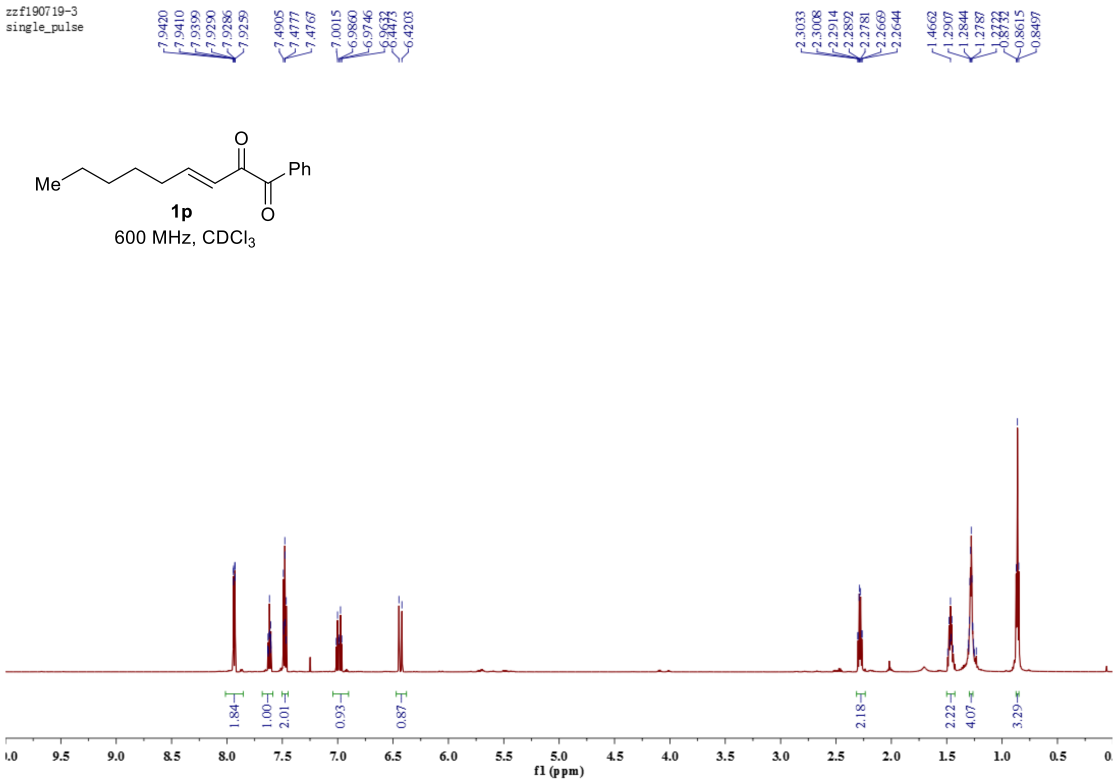

zzf190719-3
single_pulse
Me
O
Ph
O
1p
600 MHz, CDCl3
f1 (ppm)

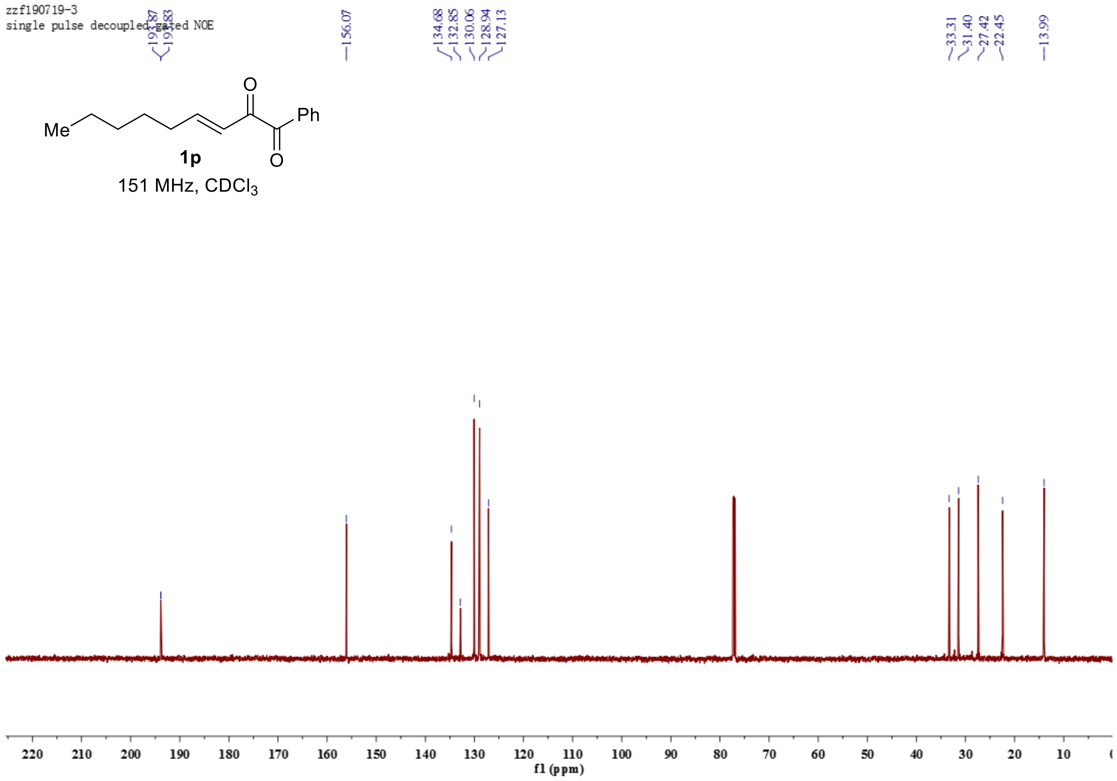

zzf190719-3
single pulse decoupled gated NOE
Me
O
Ph
O
1p
151 MHz, CDCl3
f1 (ppm)

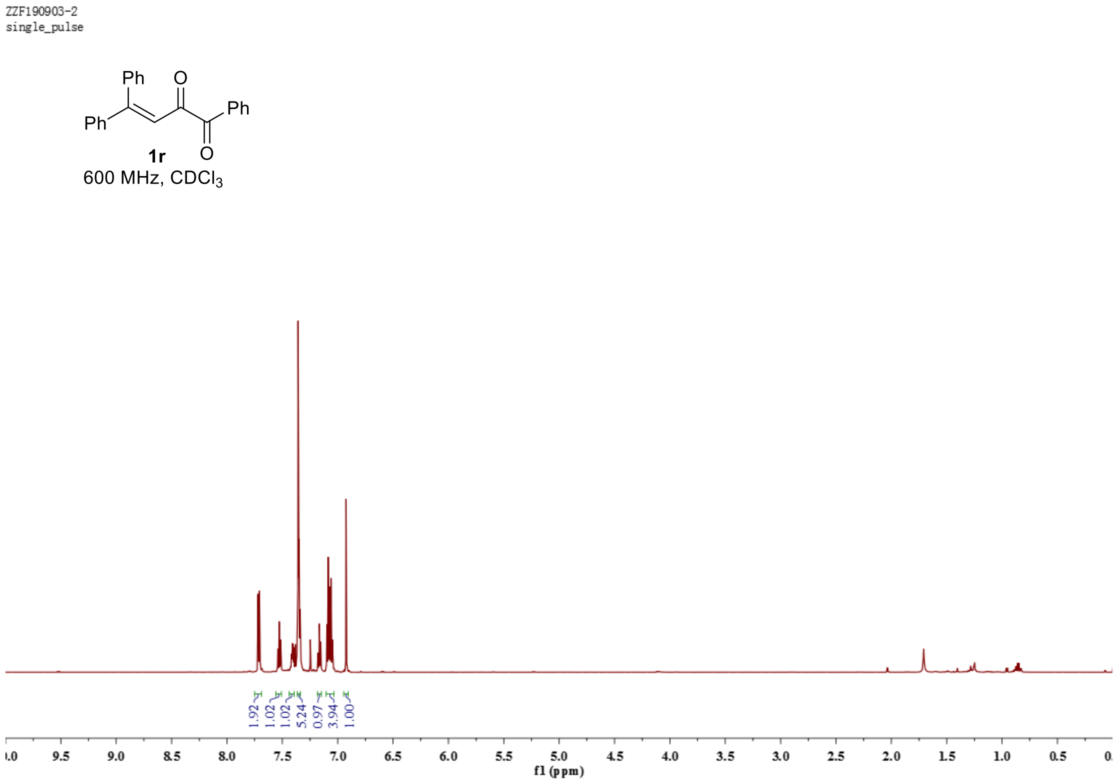
ZZF190903-2
single_pulse
Ph
O
Ph
Ph
O
1r
600 MHz, CDCl3
1.92
1.02
1.02
5.24
0.97
3.94
1.00
f1 (ppm)

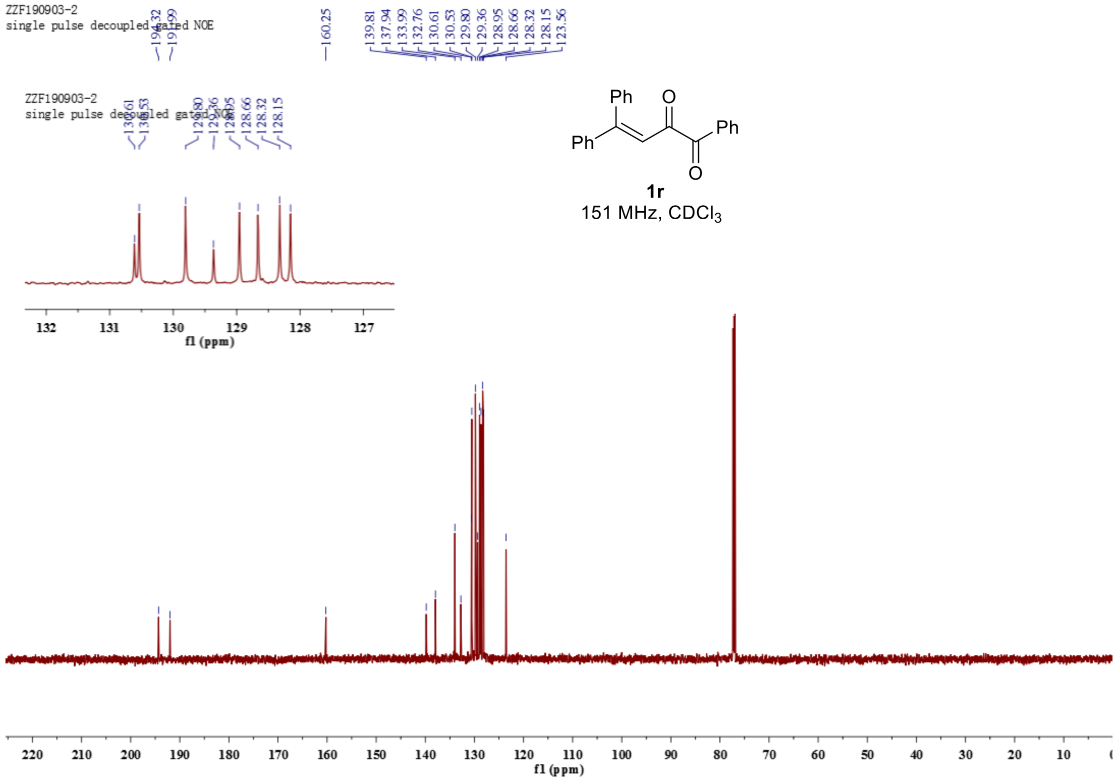
ZZF190903-2
single pulse decoupled gated NOE
194.32
191.99
160.25
139.81
137.94
133.99
132.76
130.61
130.53
129.80
129.36
128.95
128.66
128.32
128.15
123.56
Ph
O
Ph
Ph
O
1r
151 MHz, CDCl3
f1 (ppm)

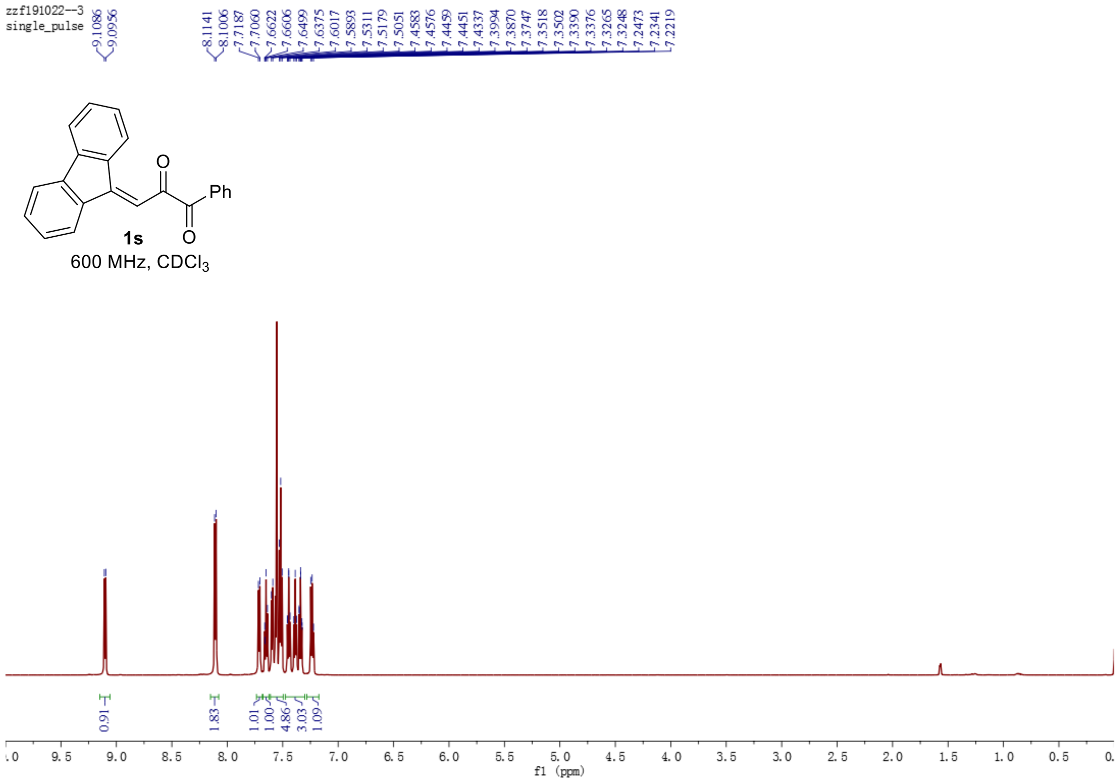
zzf191022--3
single_pulse
9.1086
9.0956
8.1141
8.1006
7.7187
7.7060
7.6622
7.6606
7.6499
7.6375
7.6017
7.5893
7.5311
7.5179
7.5051
7.4583
7.4576
7.4459
7.4451
7.4337
7.3994
7.3870
7.3747
7.3518
7.3502
7.3390
7.3376
7.3265
7.3248
7.2473
7.2341
7.2219
Ph
1s
600 MHz, CDCl3
0.91
1.83
1.01
1.00
4.86
3.03
1.09
f1 (ppm)

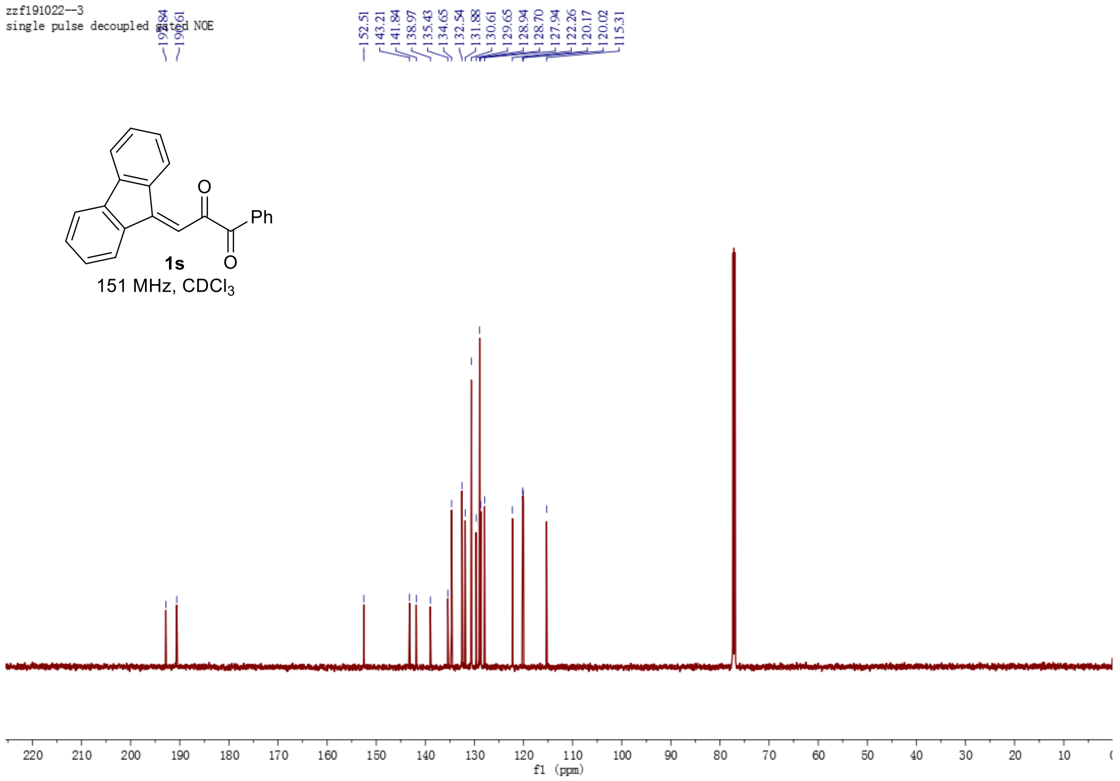
zzf191022--3
single pulse decoupled gated NOE
152.51
143.21
141.84
138.97
135.43
134.65
132.54
131.88
130.61
129.65
128.94
128.70
127.94
122.26
120.17
120.02
115.31
Ph
1s
151 MHz, CDCl3
f1 (ppm)

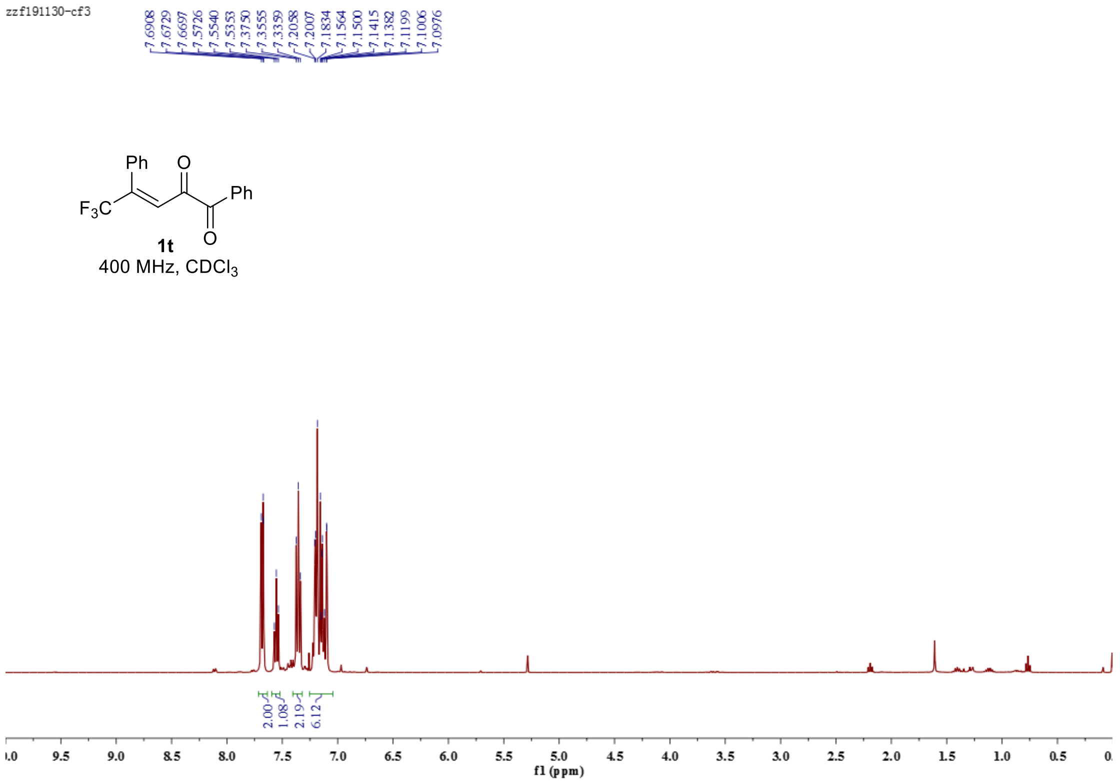
zzf191130-cf3
7.6908
7.6729
7.6697
7.5726
7.5540
7.5353
7.3750
7.3555
7.3359
7.2058
7.2007
7.1834
7.1564
7.1500
7.1415
7.1382
7.1199
7.1006
7.0976
Ph
O
Ph
F_3C
O
1t
400 MHz, $CDCl_3$
2.00
1.08
2.19
6.12
f1 (ppm)

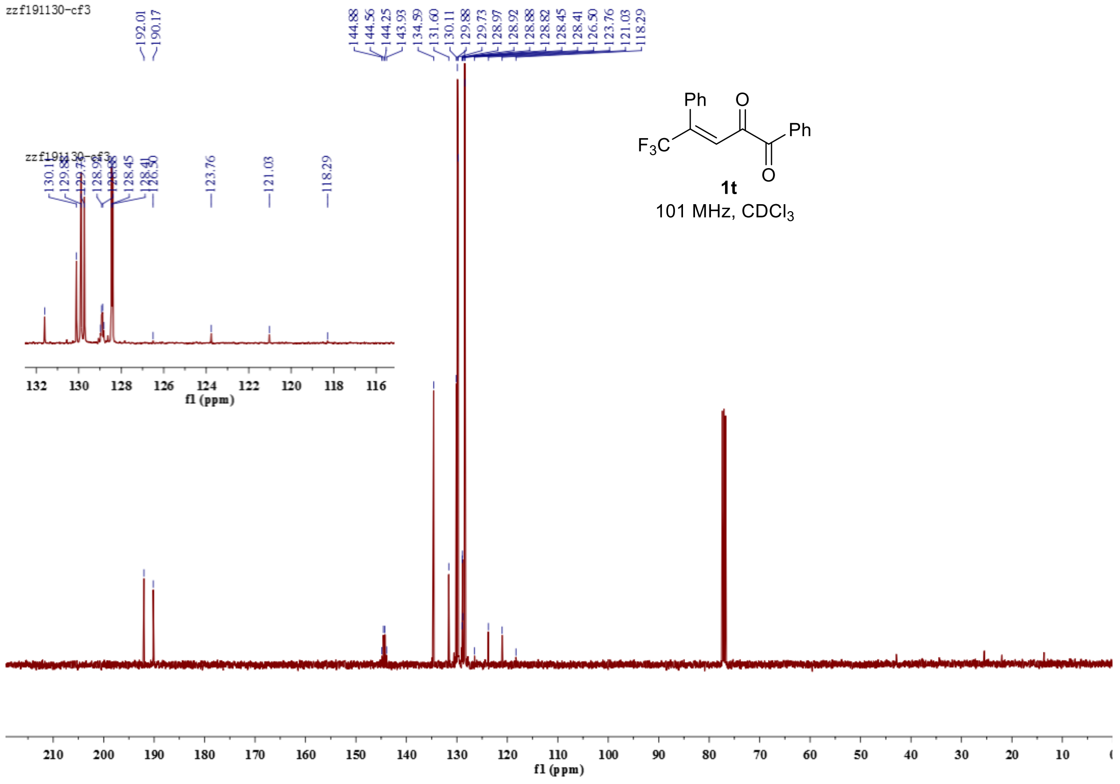
zzf191130-cf3
192.01
190.17
144.88
144.56
144.25
143.93
134.59
131.60
130.11
129.88
129.73
128.97
128.92
128.88
128.82
128.45
128.41
126.50
123.76
121.03
118.29
Ph
O
Ph
F_3C
O
1t
101 MHz, $CDCl_3$
f1 (ppm)

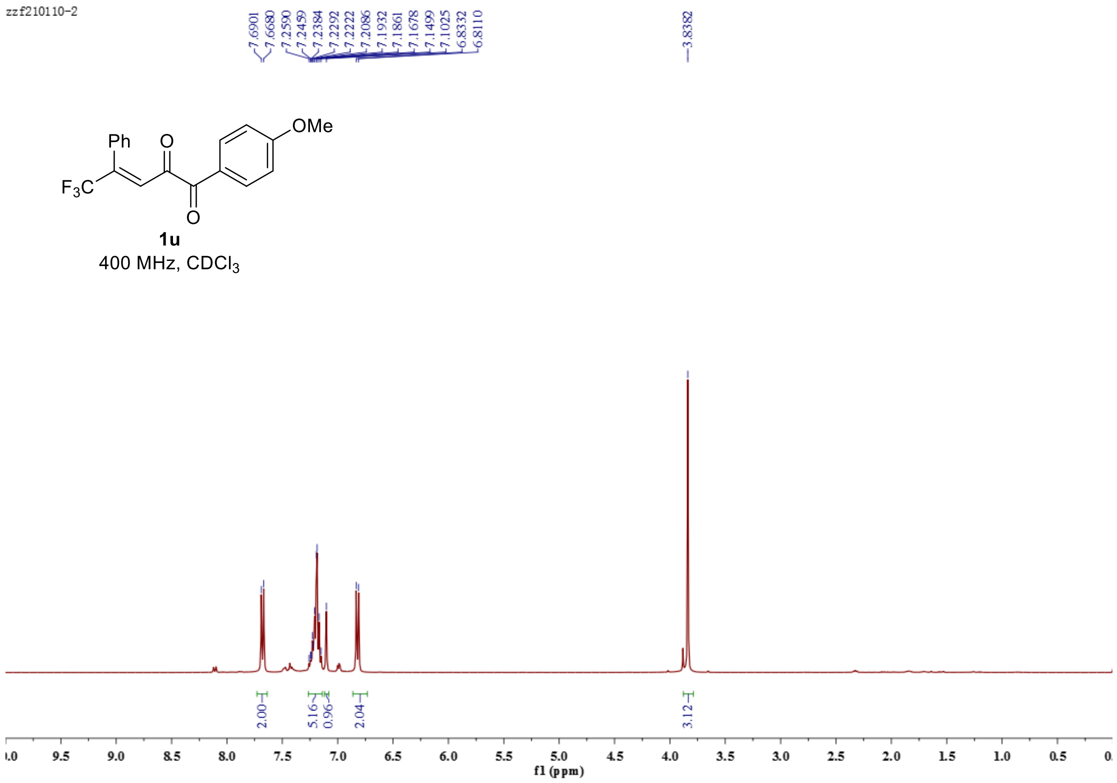
zzf210110-2
7.6901
7.6680
7.2590
7.2459
7.2384
7.2292
7.2222
7.2086
7.1932
7.1861
7.1678
7.1499
7.1025
6.8332
6.8110
3.8382
Ph
O
OMe
F3C
O
1u
400 MHz, CDCl3
2.00
5.16
0.96
2.04
3.12
f1 (ppm)

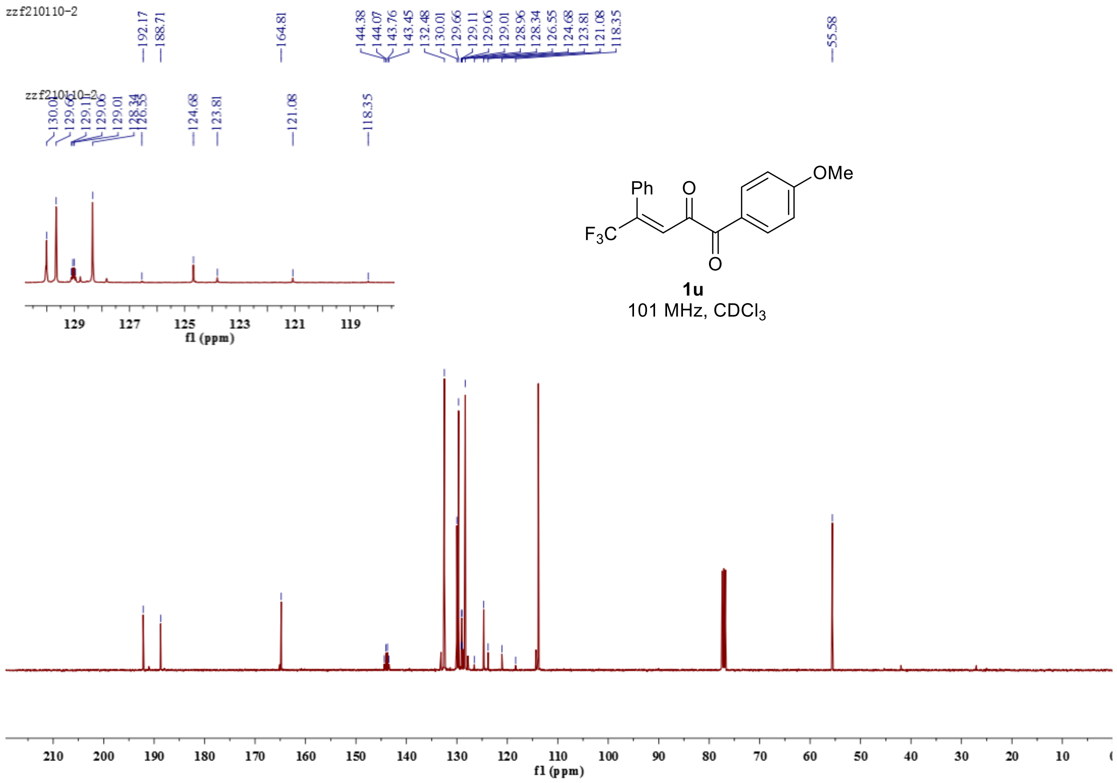
zzf210110-2
192.17
188.71
164.81
144.38
144.07
143.76
143.45
132.48
130.01
129.66
129.11
129.06
129.01
128.96
128.34
126.55
124.68
123.81
121.08
118.35
55.58
Ph
O
OMe
F3C
O
1u
101 MHz, CDCl3
f1 (ppm)

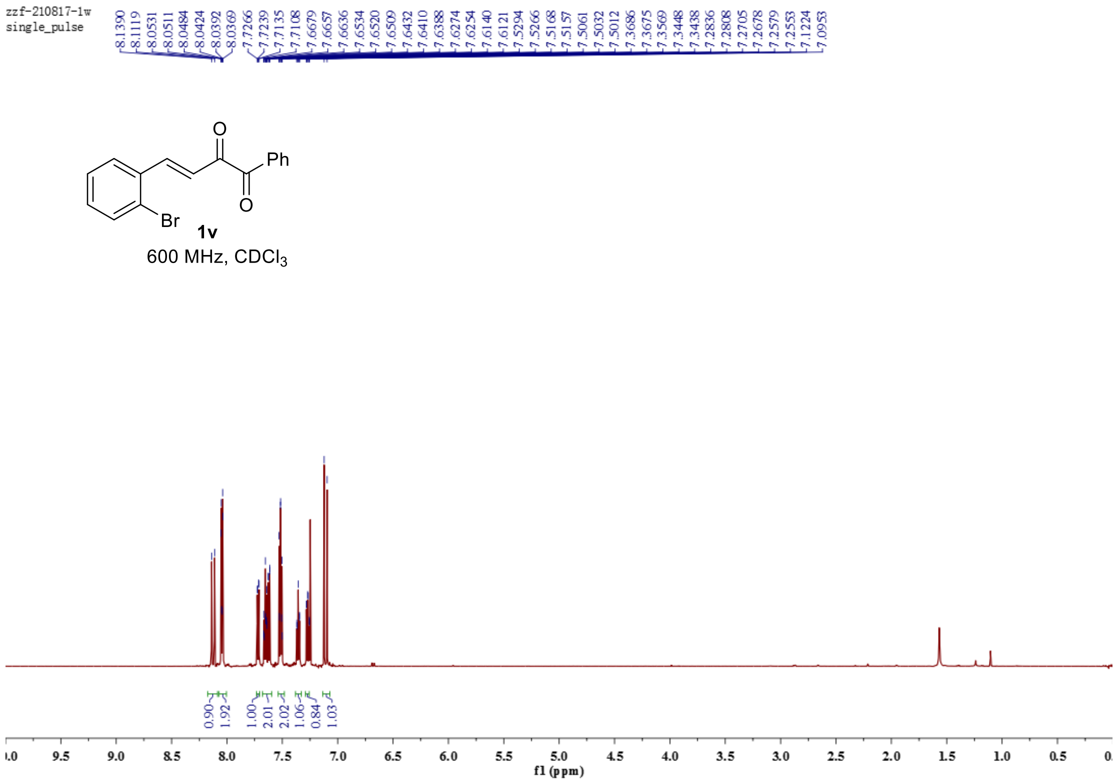
zzf-210817-1w
single_pulse
1v
600 MHz, CDCl3
f1 (ppm)

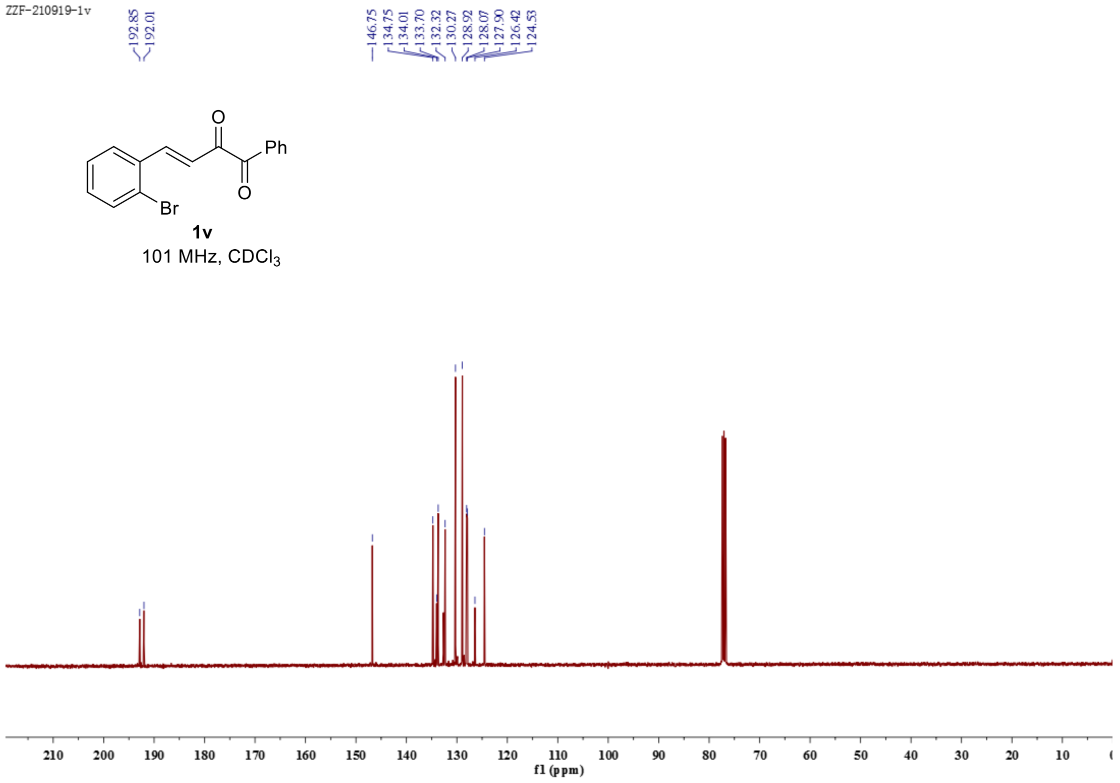
ZZF-210919-1v
1v
101 MHz, CDCl3
f1 (ppm)

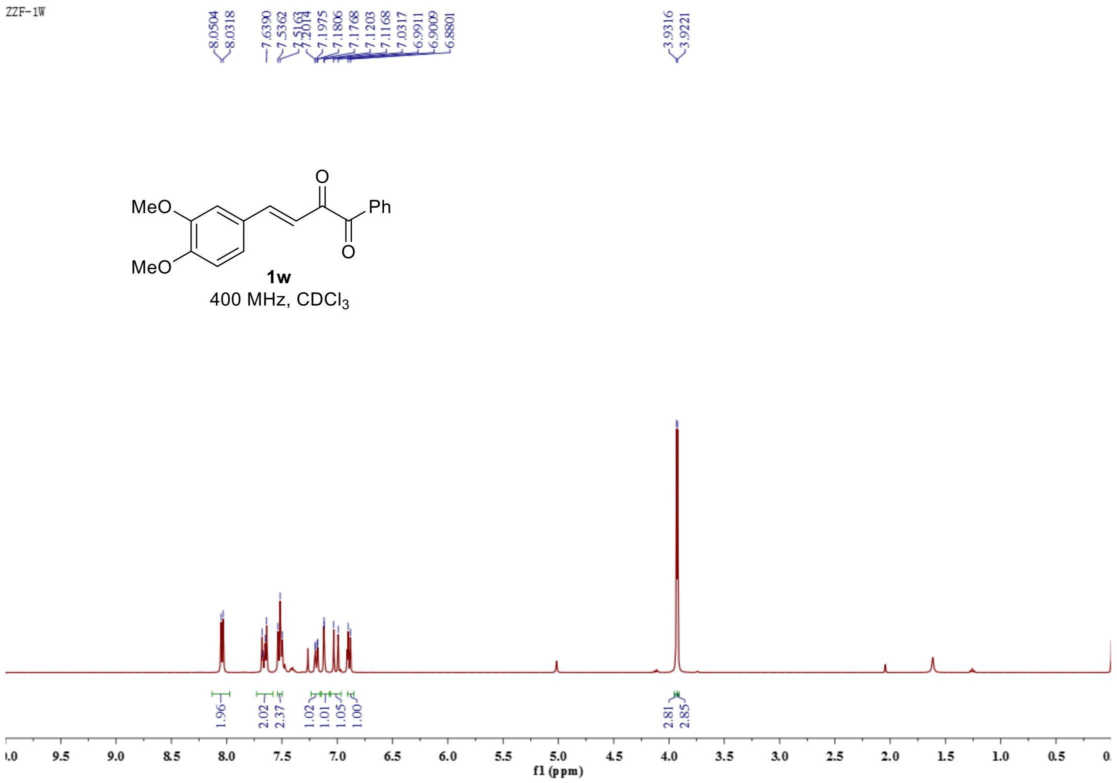
ZZF-1W
8.0504
8.0318
7.6390
7.5362
7.5163
7.2014
7.1975
7.1806
7.1768
7.1203
7.1168
7.0317
6.9911
6.9009
6.8801
3.9316
3.9221
MeO
MeO
O
O
Ph
1w
400 MHz, CDCl3
1.96
2.02
2.37
1.02
1.01
1.05
1.00
2.81
2.85
f1 (ppm)

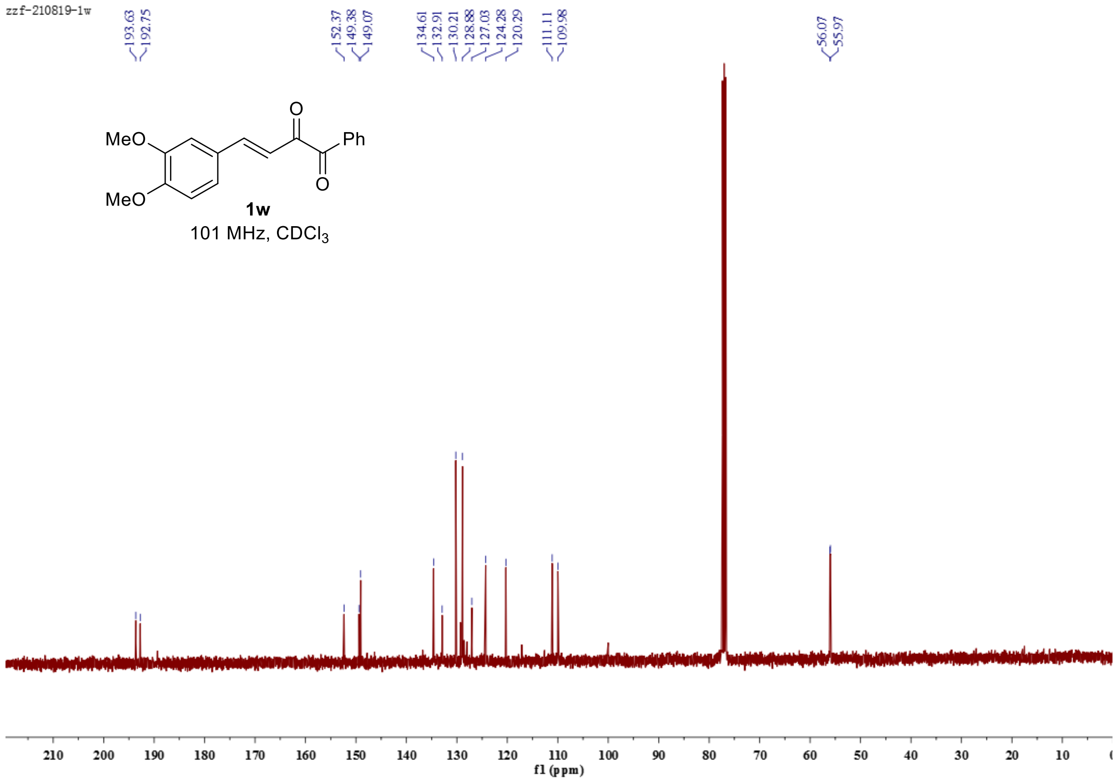
zzf-210819-1w
193.63
192.75
152.37
149.38
149.07
134.61
132.91
130.21
128.88
127.03
124.28
120.29
111.11
109.98
56.07
55.97
MeO
MeO
O
O
Ph
1w
101 MHz, CDCl3
f1 (ppm)

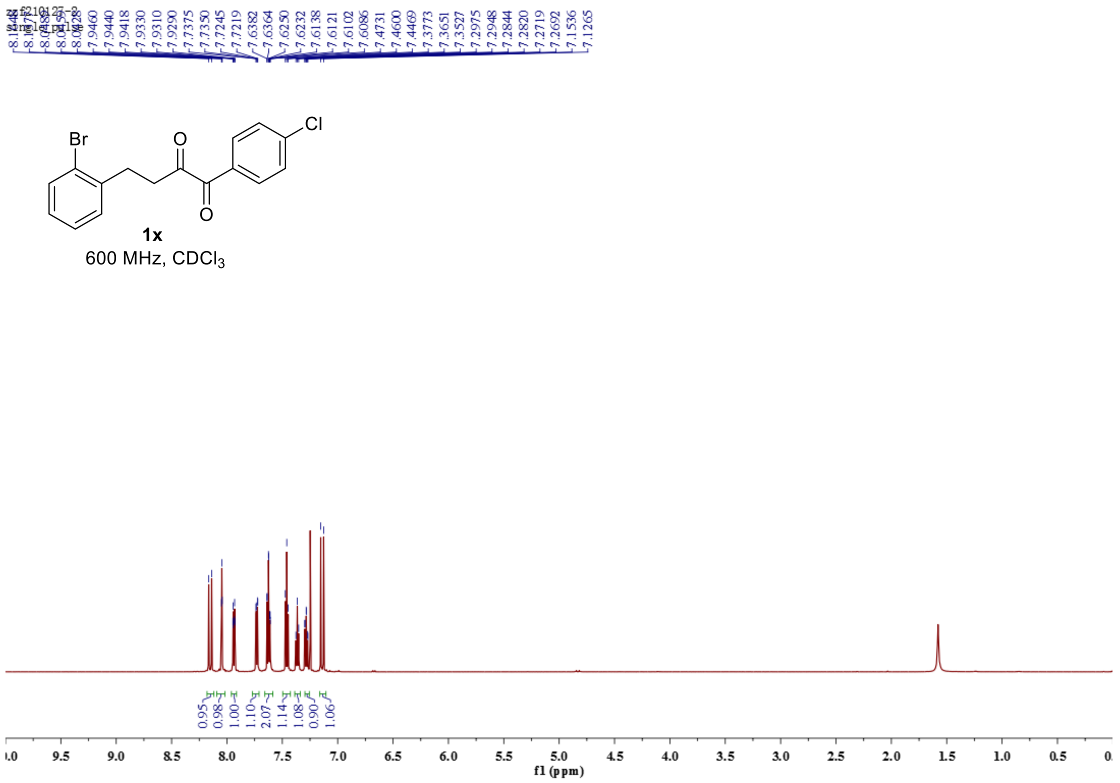
7.9460 7.9440 7.9418 7.9330 7.9310 7.9290 7.7375 7.7350 7.7245 7.7219 7.6382 7.6364 7.6250 7.6232 7.6138 7.6121 7.6102 7.6086 7.4731 7.4600 7.4469 7.3773 7.3651 7.3527 7.2975 7.2948 7.2844 7.2820 7.2719 7.2692 7.1536 7.1265
Br
O
Cl
O
1x
600 MHz, CDCl3
0.95 0.98 1.00 1.10 2.07 1.14 1.08 0.90 1.06
f1 (ppm)

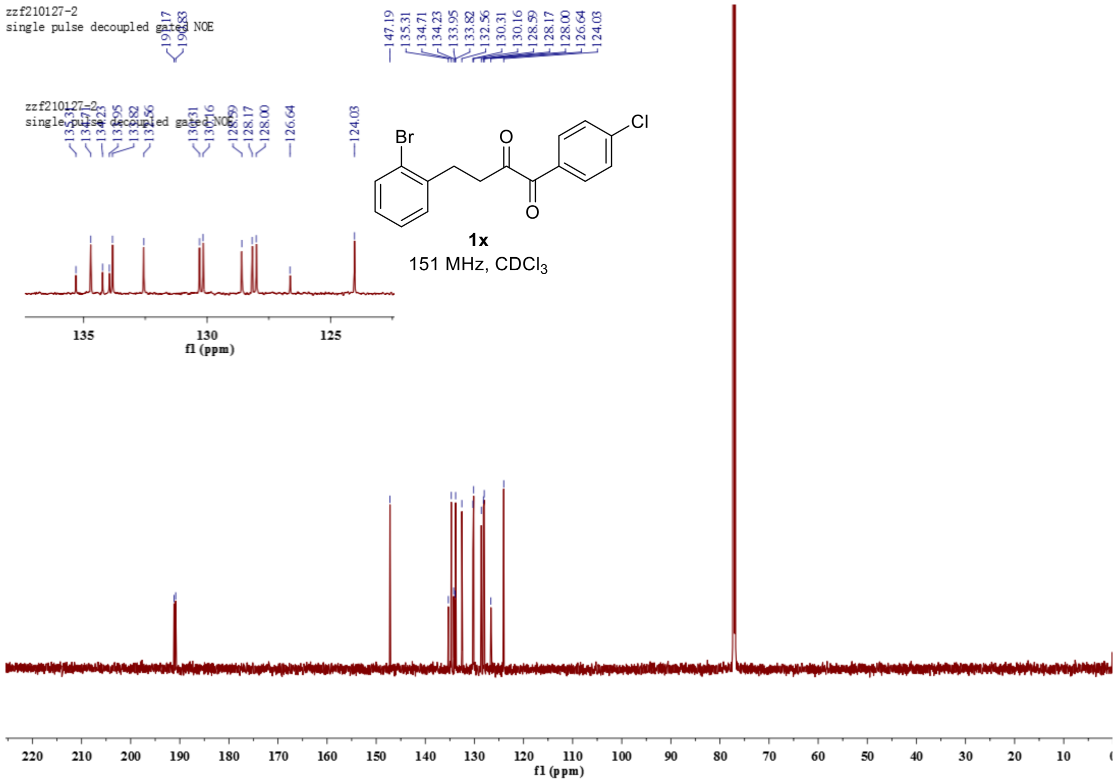
zzf210127-2
single pulse decoupled gated NOE
191.17
147.19 135.31 134.71 134.23 133.95 133.82 132.56 130.31 130.16 128.59 128.17 128.00 126.64 124.03
Br
O
Cl
O
1x
151 MHz, CDCl3
f1 (ppm)

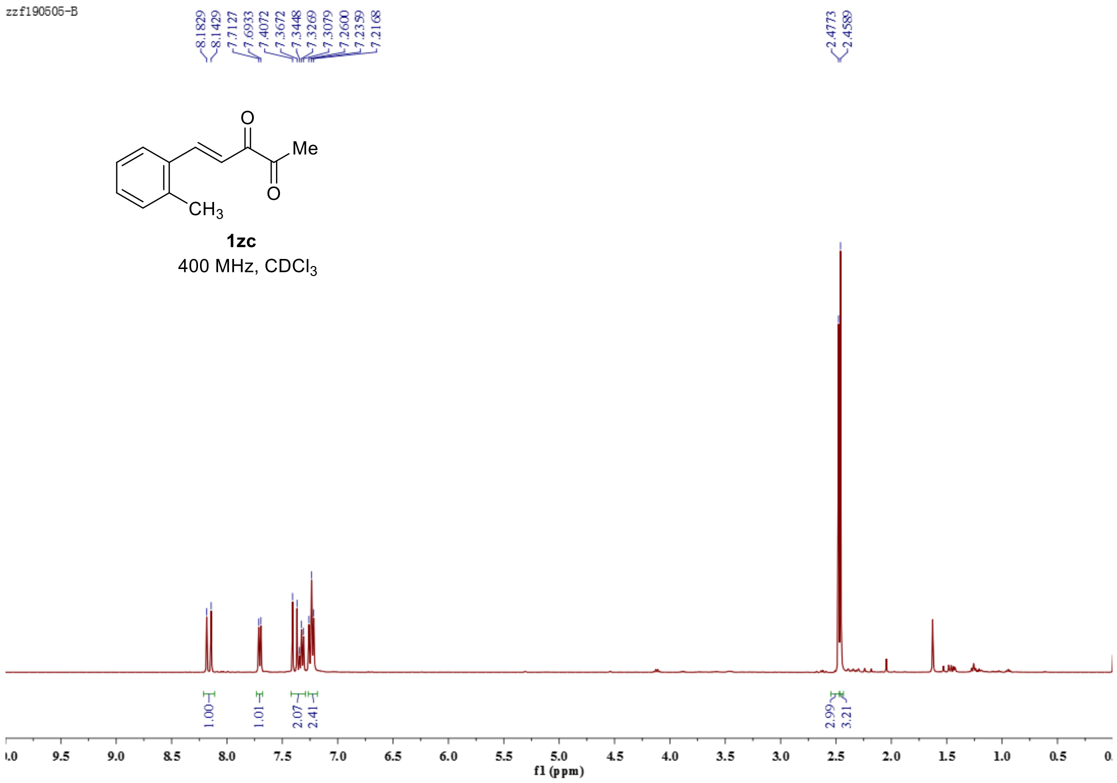
zzf190505-B
8.1829
8.1429
7.7127
7.6933
7.4072
7.3672
7.3448
7.3269
7.3079
7.2600
7.2359
7.2168
2.4773
2.4589
O
Me
O
CH3
1zc
400 MHz, CDCl3
1.00
1.01
2.07
2.41
2.99
3.21
f1 (ppm)

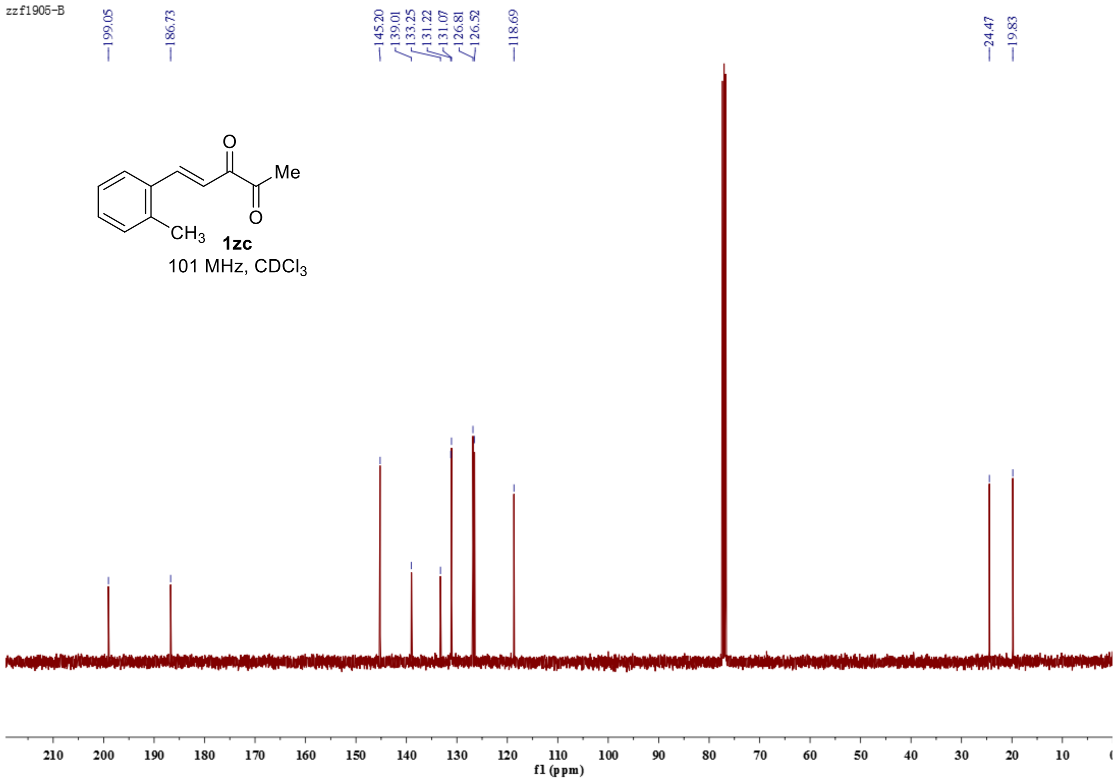
zzf1905-B
199.05
186.73
145.20
139.01
133.25
131.22
131.07
126.81
126.52
118.69
24.47
19.83
O
Me
O
CH3
1zc
101 MHz, CDCl3
f1 (ppm)

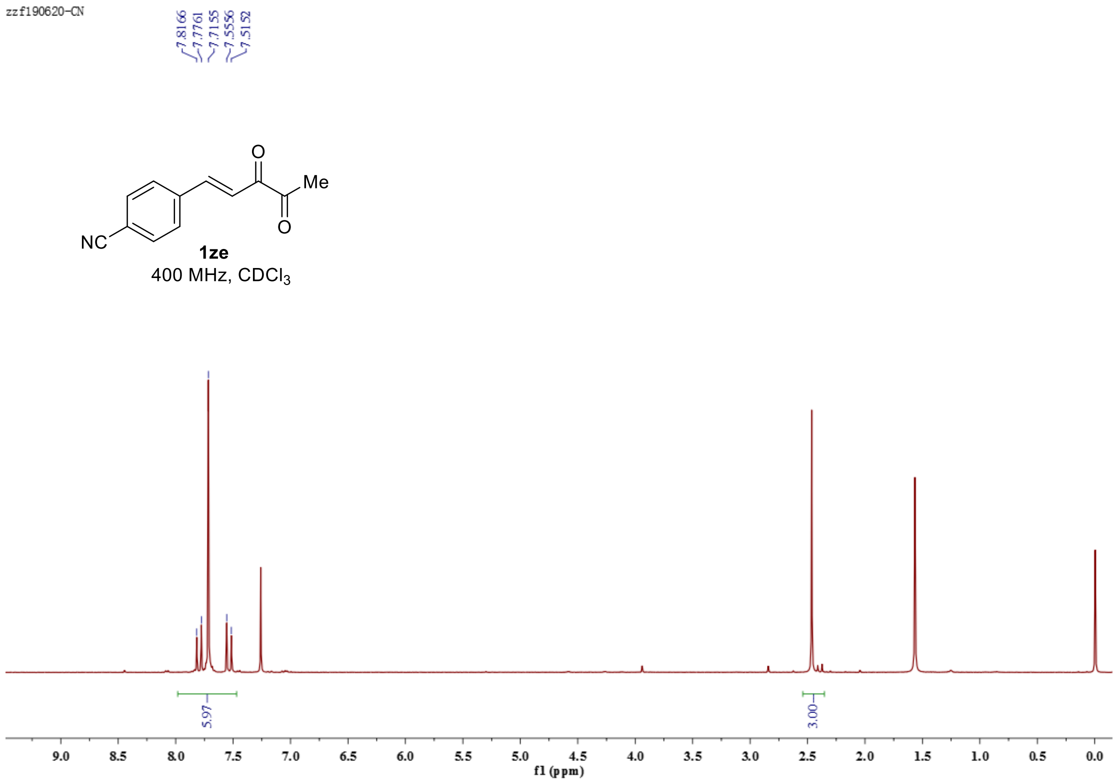
zzf190620-CN
7.8166
7.7761
7.7155
7.5556
7.5152
O
Me
O
NC
1ze
400 MHz, CDCl3
5.97
3.00
9.0 8.5 8.0 7.5 7.0 6.5 6.0 5.5 5.0 4.5 4.0 3.5 3.0 2.5 2.0 1.5 1.0 0.5 0.0
f1 (ppm)

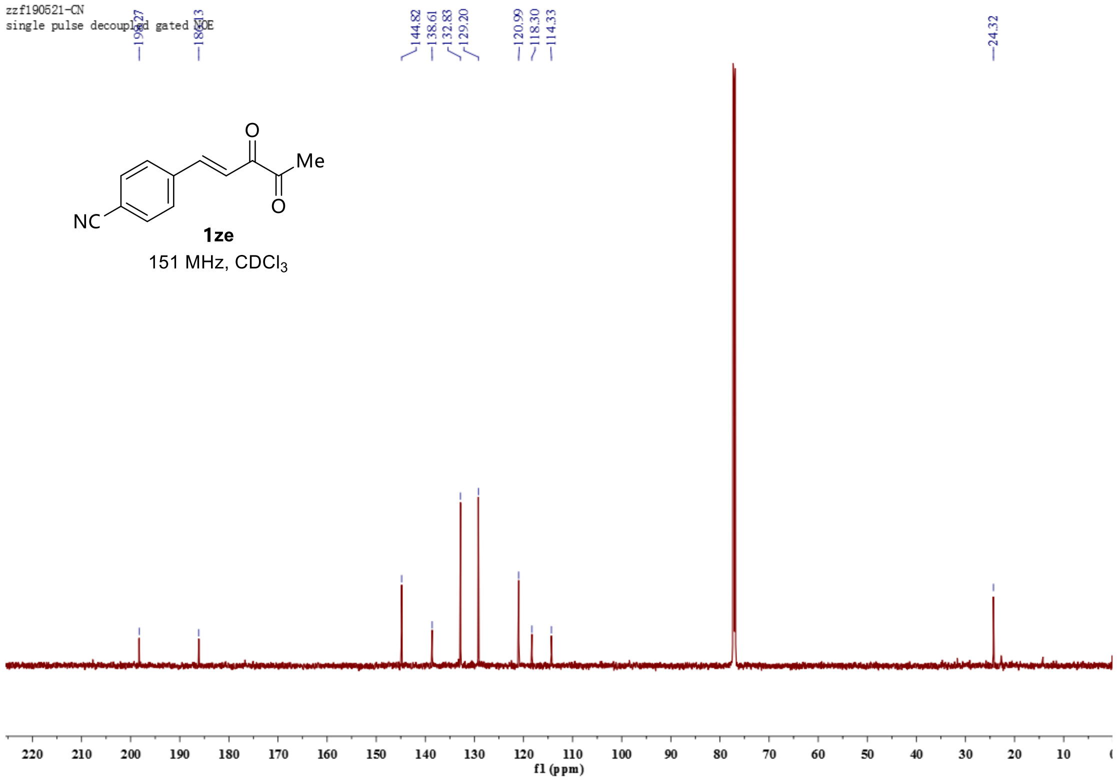
zzf190521-CN
single pulse decoupled gated NOE
144.82
138.61
132.83
129.20
120.99
118.30
114.33
24.32
O
Me
O
NC
1ze
151 MHz, CDCl3
220 210 200 190 180 170 160 150 140 130 120 110 100 90 80 70 60 50 40 30 20 10
f1 (ppm)

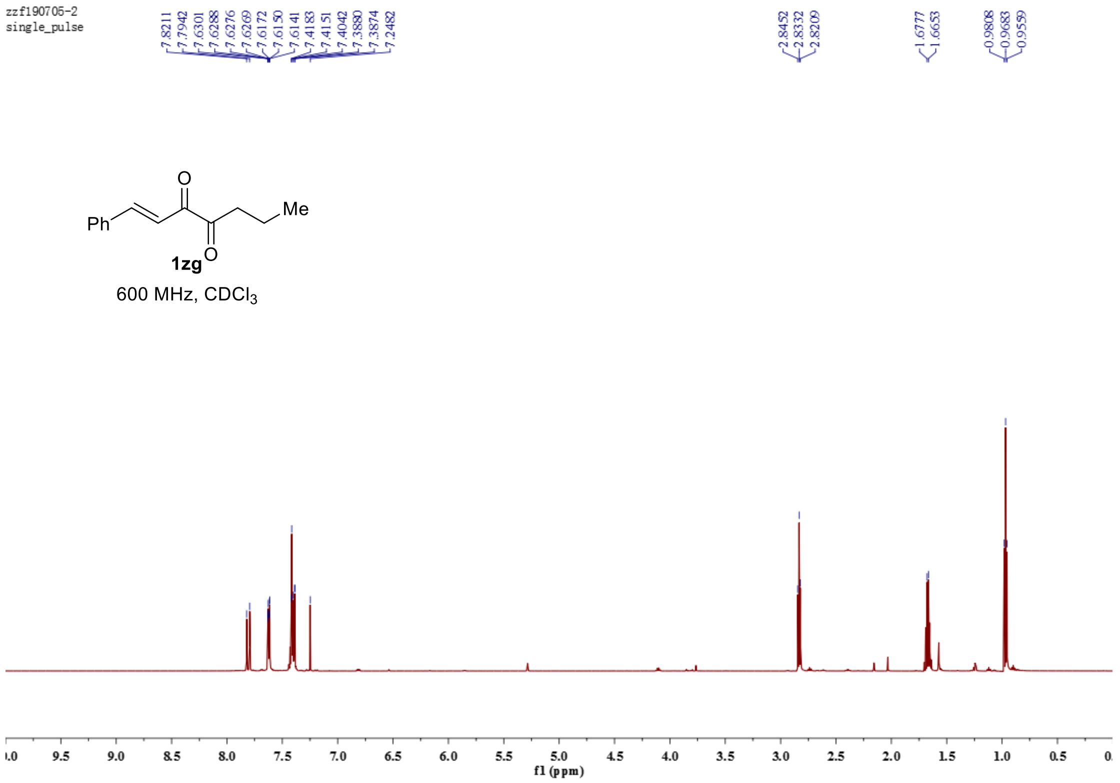
zzf190705-2
single_pulse
7.8211
7.7942
7.6301
7.6288
7.6276
7.6269
7.6172
7.6150
7.6141
7.4183
7.4151
7.4042
7.3880
7.3874
7.2482
2.8452
2.8332
2.8209
1.6777
1.6653
0.9808
0.9683
0.9559
O
Ph
Me
O
1zg
600 MHz, CDCl3
f1 (ppm)

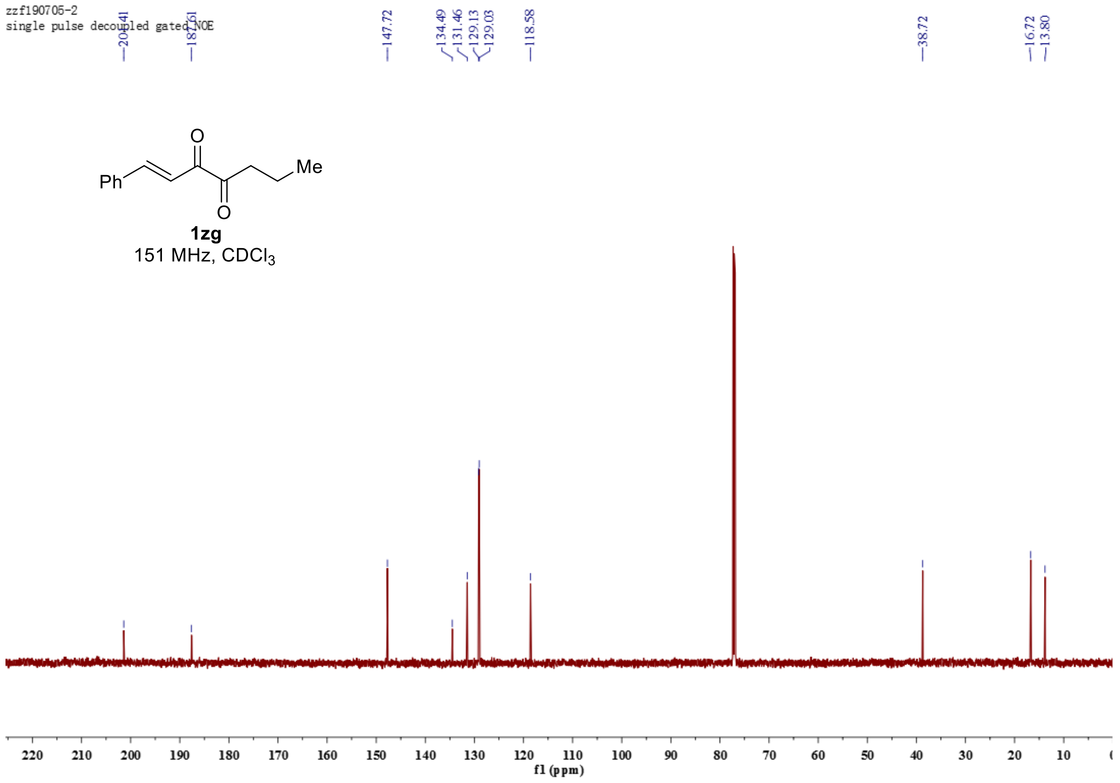
zzf190705-2
single pulse decoupled gated NOE
147.72
134.49
131.46
129.13
129.03
118.58
38.72
16.72
13.80
O
Ph
Me
O
1zg
151 MHz, CDCl3
f1 (ppm)

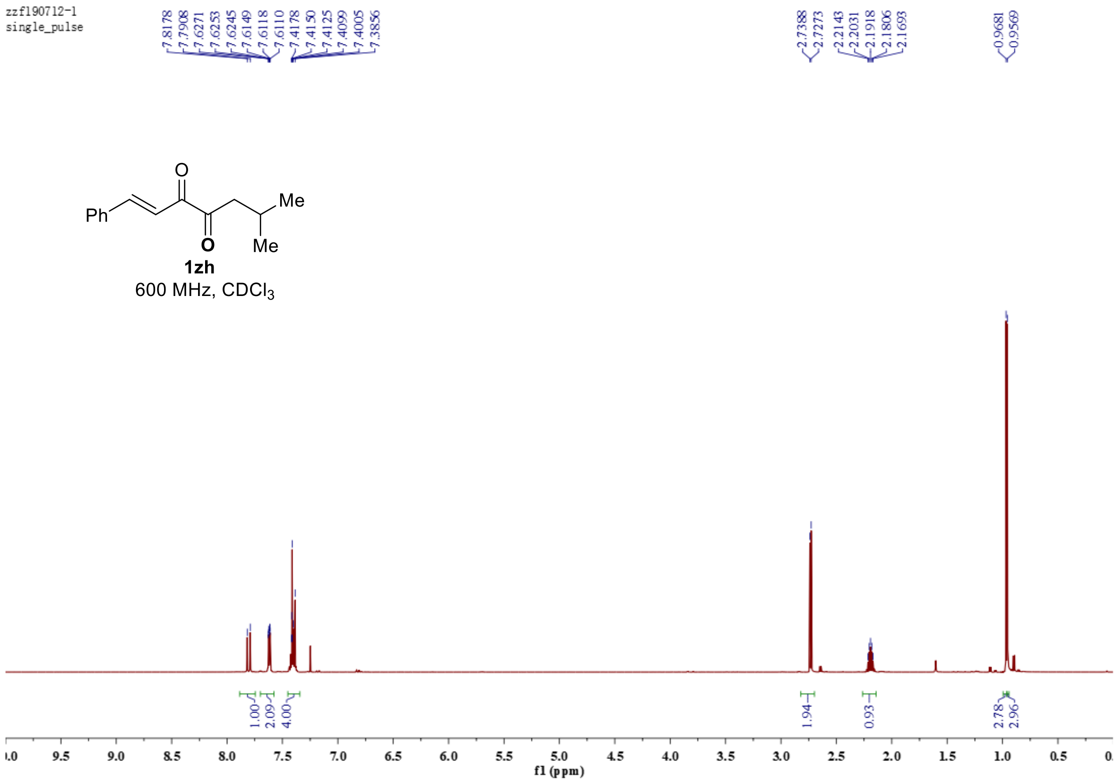
zzf190712-1
single_pulse
7.8178
7.7908
7.6271
7.6253
7.6245
7.6149
7.6118
7.6110
7.4178
7.4150
7.4125
7.4099
7.4005
7.3856
2.7388
2.7273
2.2143
2.2031
2.1918
2.1806
2.1693
0.9681
0.9569
Ph
O
O
Me
Me
1zh
600 MHz, CDCl3
1.00
2.09
4.00
1.94
0.93
2.78
2.96
f1 (ppm)

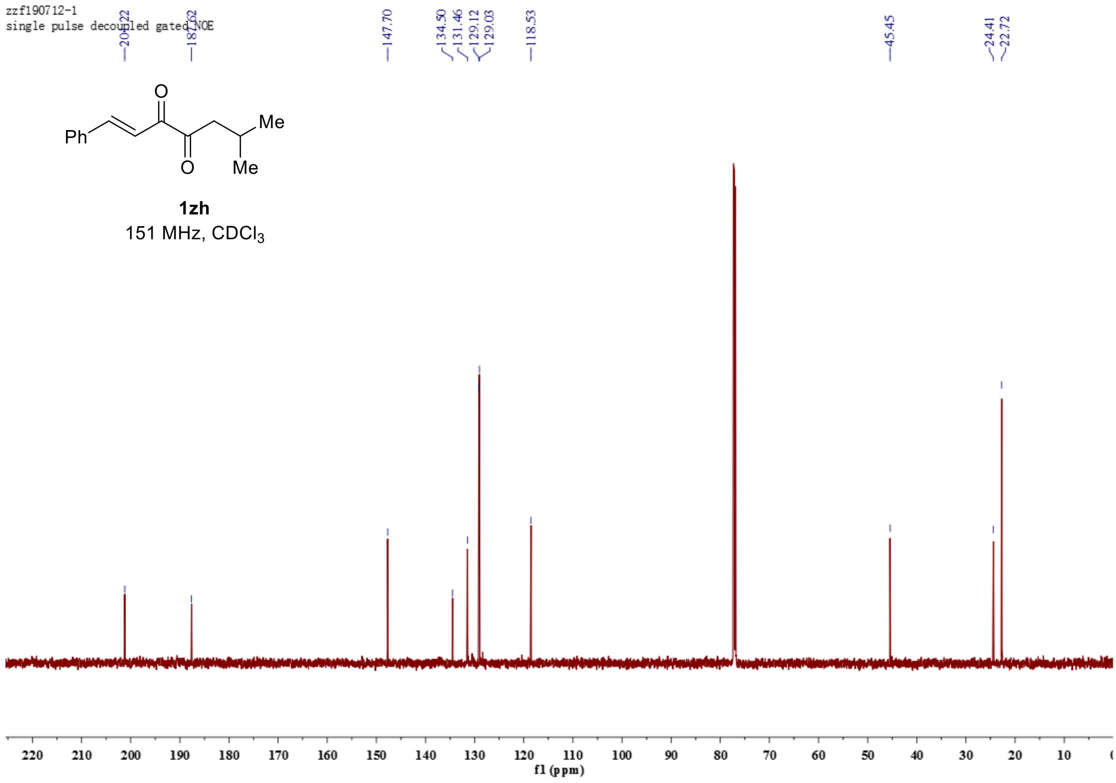
zzf190712-1
single pulse decoupled gated NOE
147.70
134.50
131.46
129.12
129.03
118.53
45.45
24.41
22.72
Ph
O
O
Me
Me
1zh
151 MHz, CDCl3
f1 (ppm)

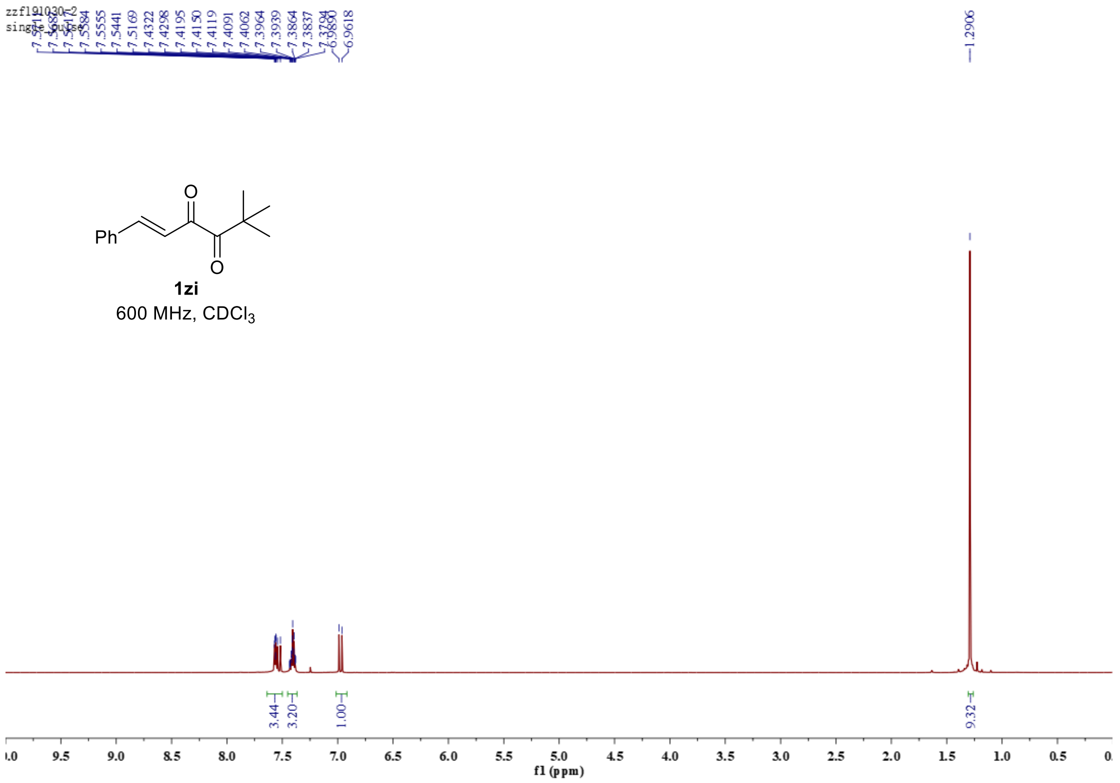
zzf191030-2
single pulse
1zi
600 MHz, CDCl3
1.2906
3.44
3.20
1.00
9.32
f1 (ppm)

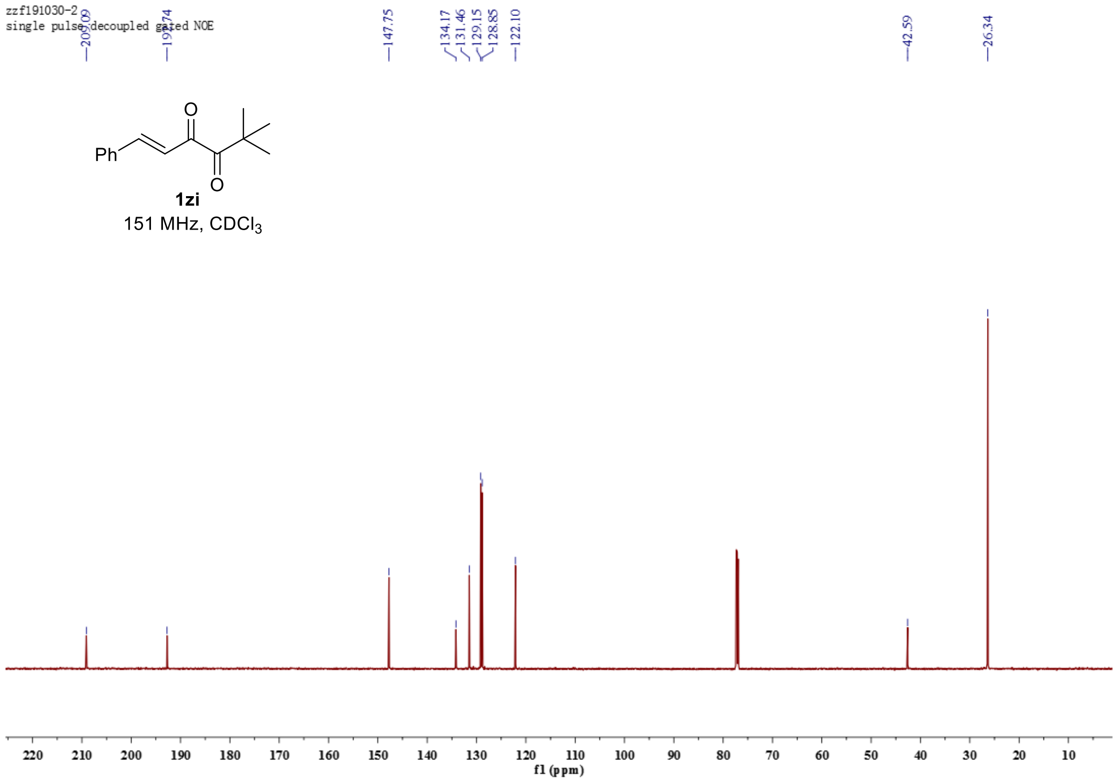
zzf191030-2
single pulse decoupled gated NOE
147.75
134.17
131.46
129.15
128.85
122.10
42.59
26.34
1zi
151 MHz, CDCl3
f1 (ppm)

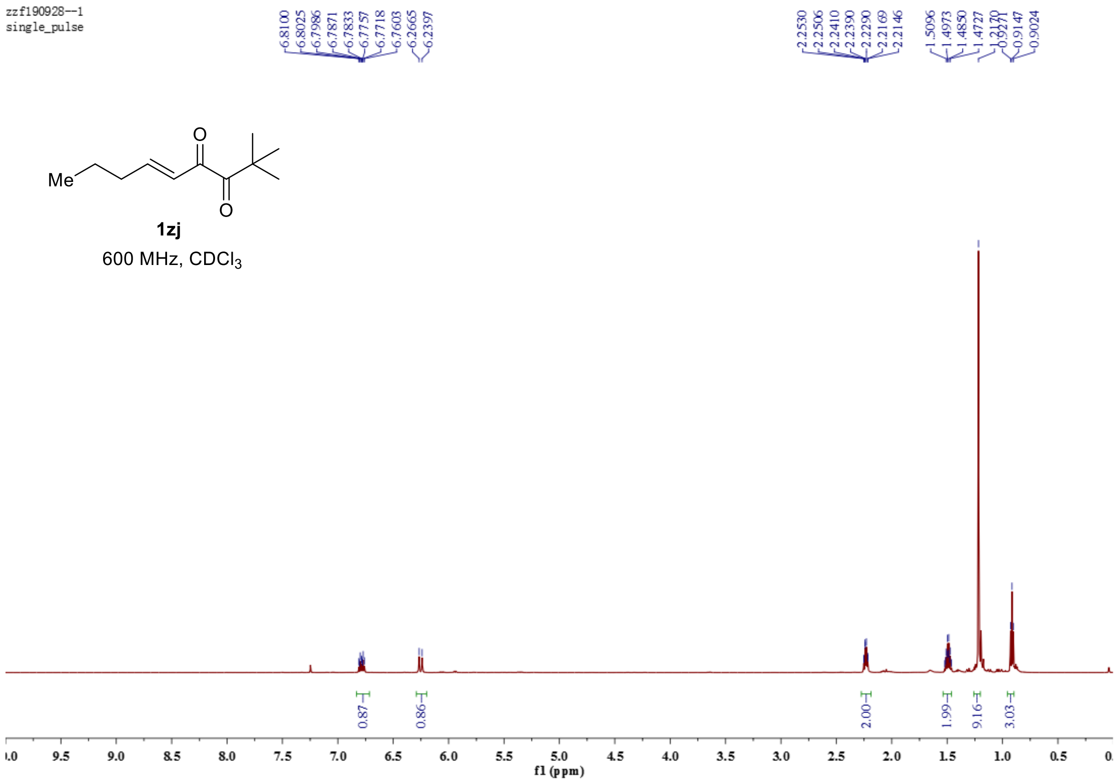
zzf190928--1
single_pulse
6.8100 6.8025 6.7986 6.7871 6.7833 6.7757 6.7718 6.7603 6.2665 6.2397
2.2530 2.2506 2.2410 2.2390 2.2290 2.2169 2.2146
1.5096 1.4973 1.4850 1.4727 1.2170 0.9271 0.9147 0.9024
Me
O
O
1zj
600 MHz, CDCl3
0.87 0.86 2.00 1.99 9.16 3.03
f1 (ppm)

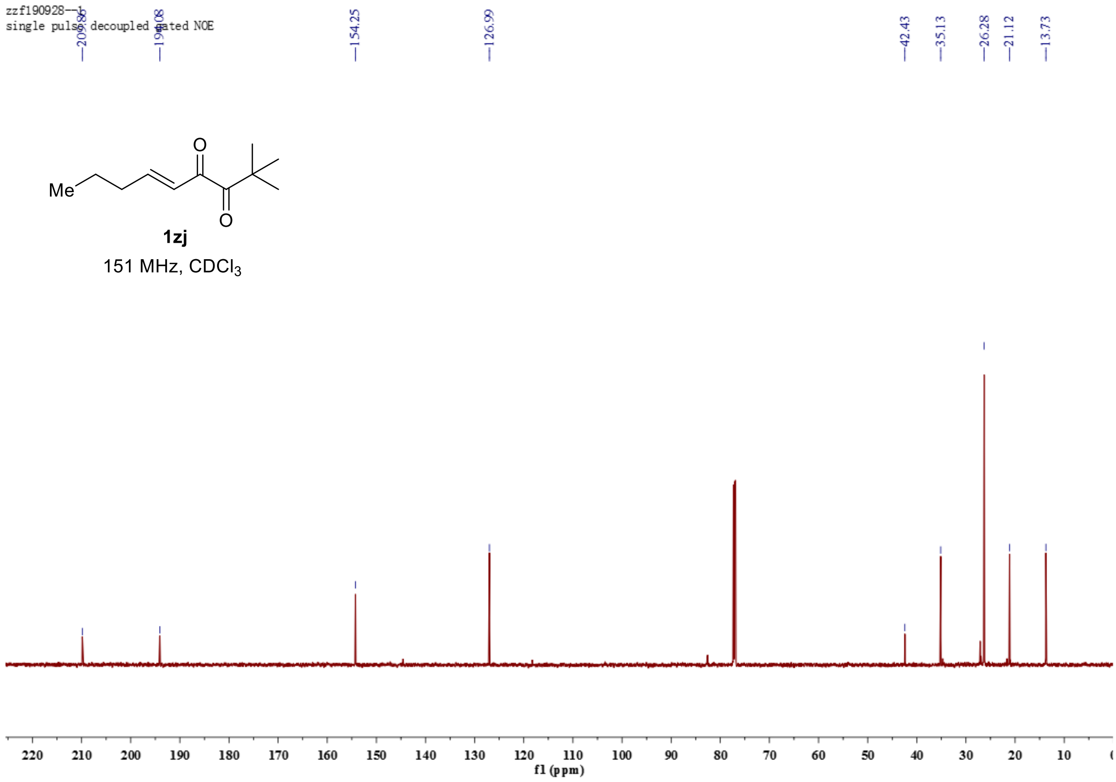
zzf190928--1
single pulse decoupled gated NOE
154.25
126.99
42.43
35.13
26.28
21.12
13.73
Me
O
O
1zj
151 MHz, CDCl3
f1 (ppm)

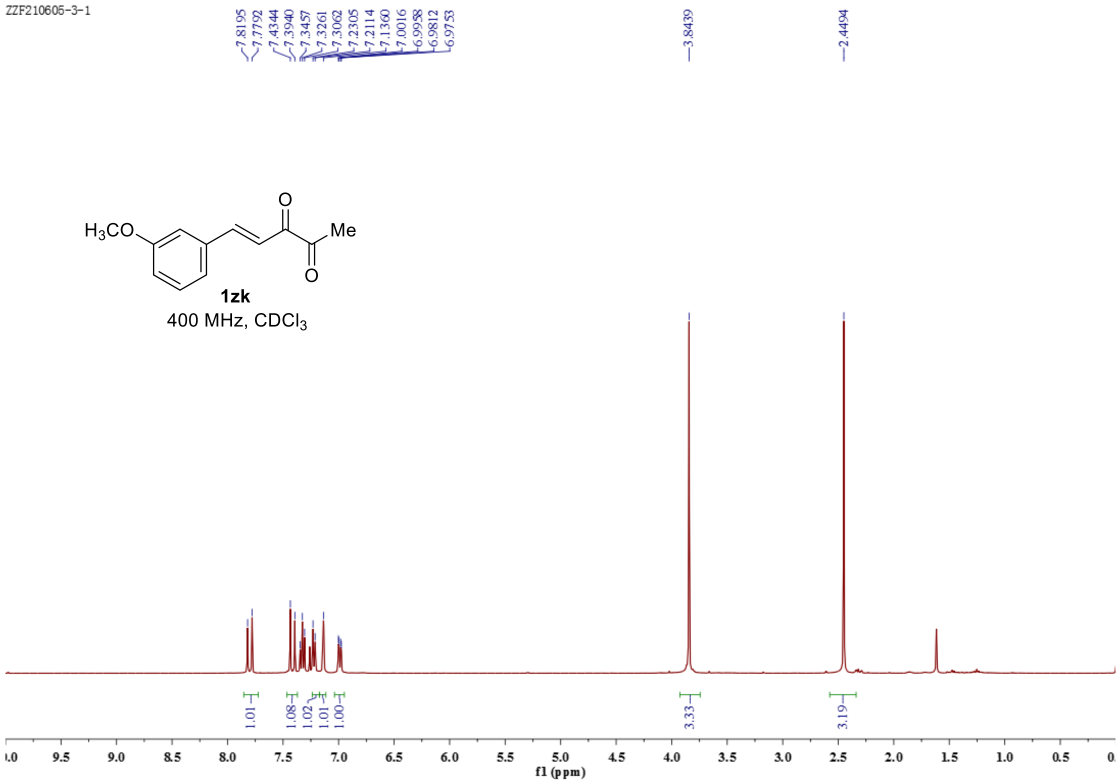
ZZF210605-3-1
7.8195
7.7792
7.4344
7.3940
7.3457
7.3261
7.3062
7.2305
7.2114
7.1360
7.0016
6.9958
6.9812
6.9753
3.8439
2.4494
H3CO
O
Me
O
1zk
400 MHz, CDCl3
1.01
1.08
1.02
1.01
1.00
3.33
3.19
9.5
9.0
8.5
8.0
7.5
7.0
6.5
6.0
5.5
5.0
4.5
4.0
3.5
3.0
2.5
2.0
1.5
1.0
0.5
f1 (ppm)

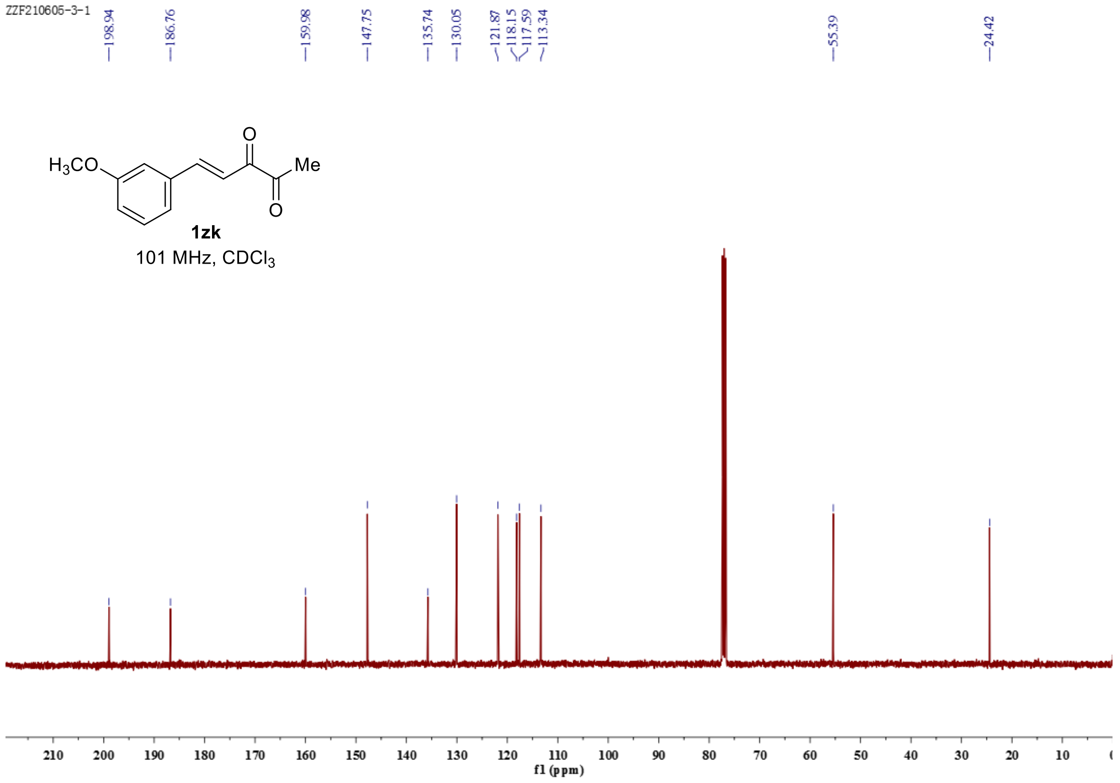
ZZF210605-3-1
198.94
186.76
159.98
147.75
135.74
130.05
121.87
118.15
117.59
113.34
55.39
24.42
H3CO
O
Me
O
1zk
101 MHz, CDCl3
210
200
190
180
170
160
150
140
130
120
110
100
90
80
70
60
50
40
30
20
10
f1 (ppm)

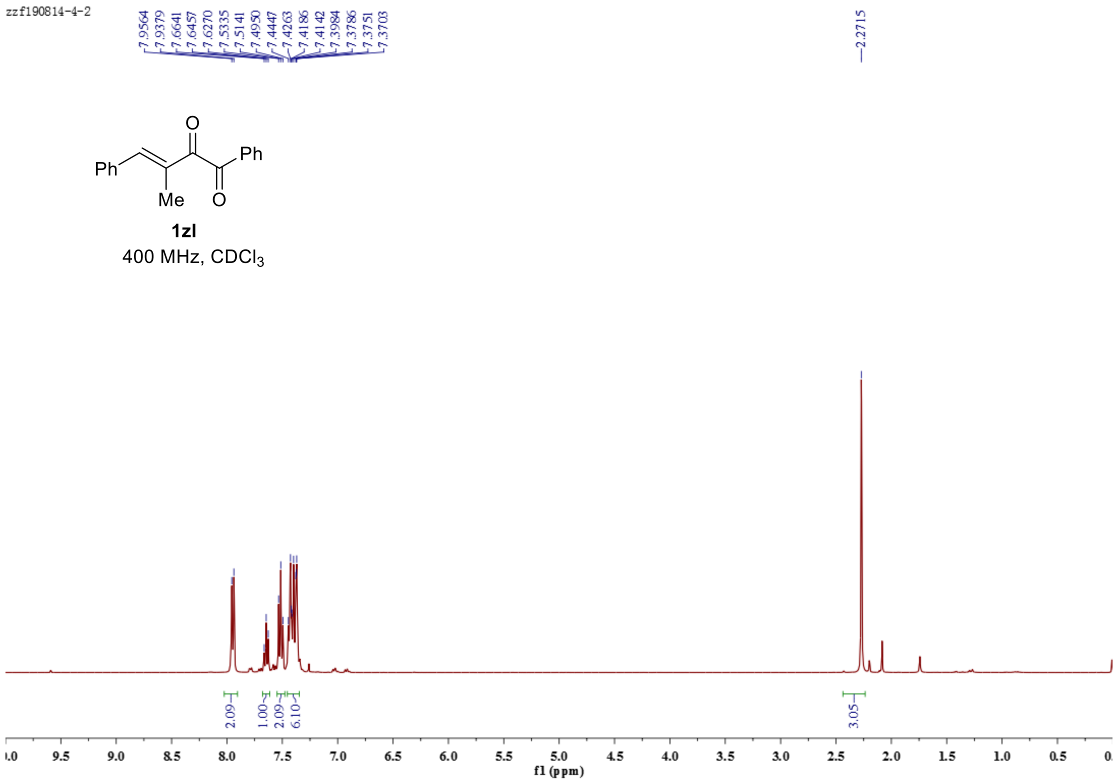
zzf190814-4-2
7.9564 7.9379 7.6641 7.6457 7.6270 7.5335 7.5141 7.4950 7.4447 7.4263 7.4186 7.4142 7.3984 7.3786 7.3751 7.3703
2.2715
Ph
O
Ph
Me
O
1zl
400 MHz, CDCl3
2.09 1.00 2.09 6.10
3.05
f1 (ppm)

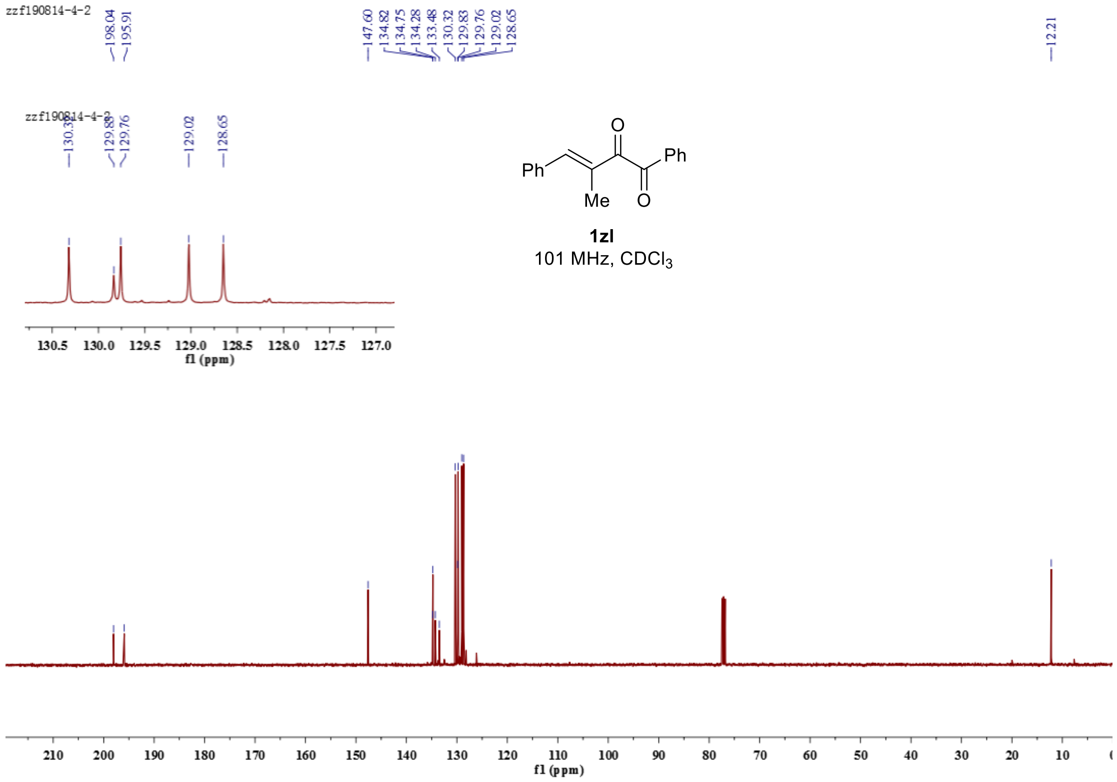
zzf190814-4-2
198.04 195.91
147.60 134.82 134.75 134.28 133.48 130.32 129.83 129.76 129.02 128.65
12.21
130.32 129.83 129.76 129.02 128.65
f1 (ppm)
Ph
O
Ph
Me
O
1zl
101 MHz, CDCl3
f1 (ppm)

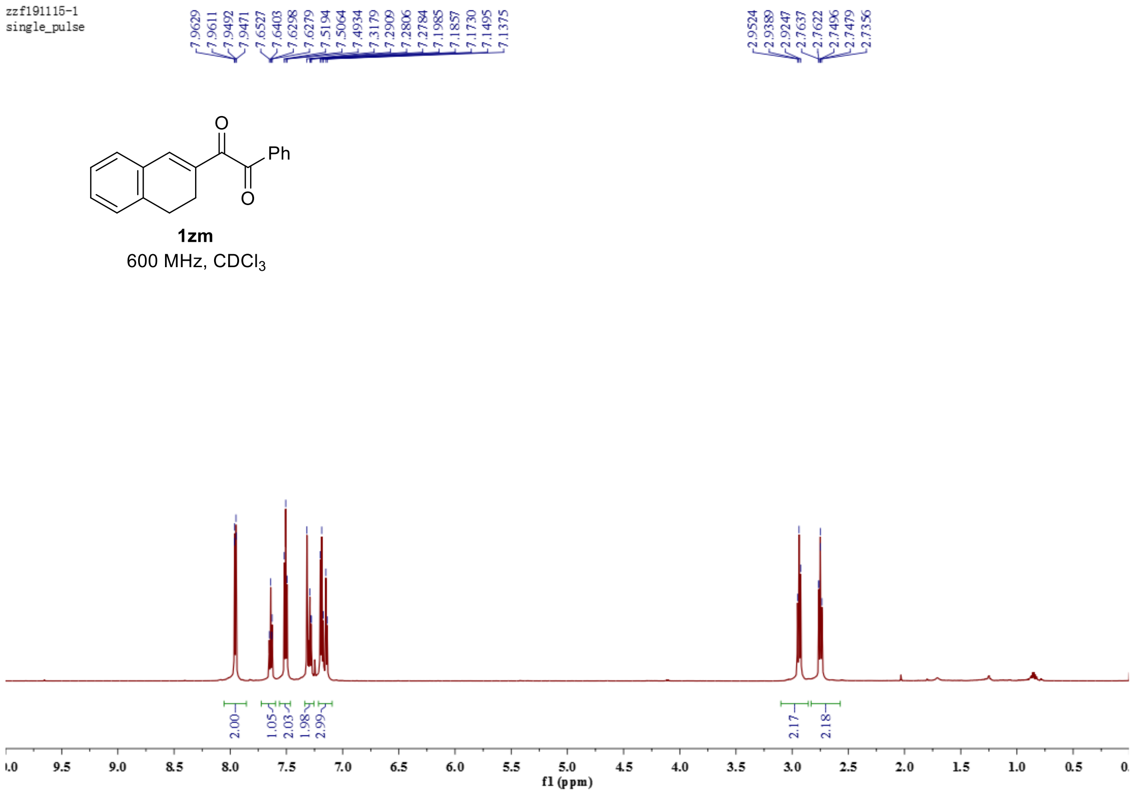

zzf191115-1
single_pulse
7.9629
7.9611
7.9492
7.9471
7.6527
7.6403
7.6298
7.6279
7.5194
7.5064
7.4934
7.3179
7.2909
7.2806
7.2784
7.1985
7.1857
7.1730
7.1495
7.1375
2.9524
2.9389
2.9247
2.7637
2.7622
2.7496
2.7479
2.7356
O
Ph
O
1zm
600 MHz, CDCl3
2.00
1.05
2.03
1.98
2.99
2.17
2.18
f1 (ppm)

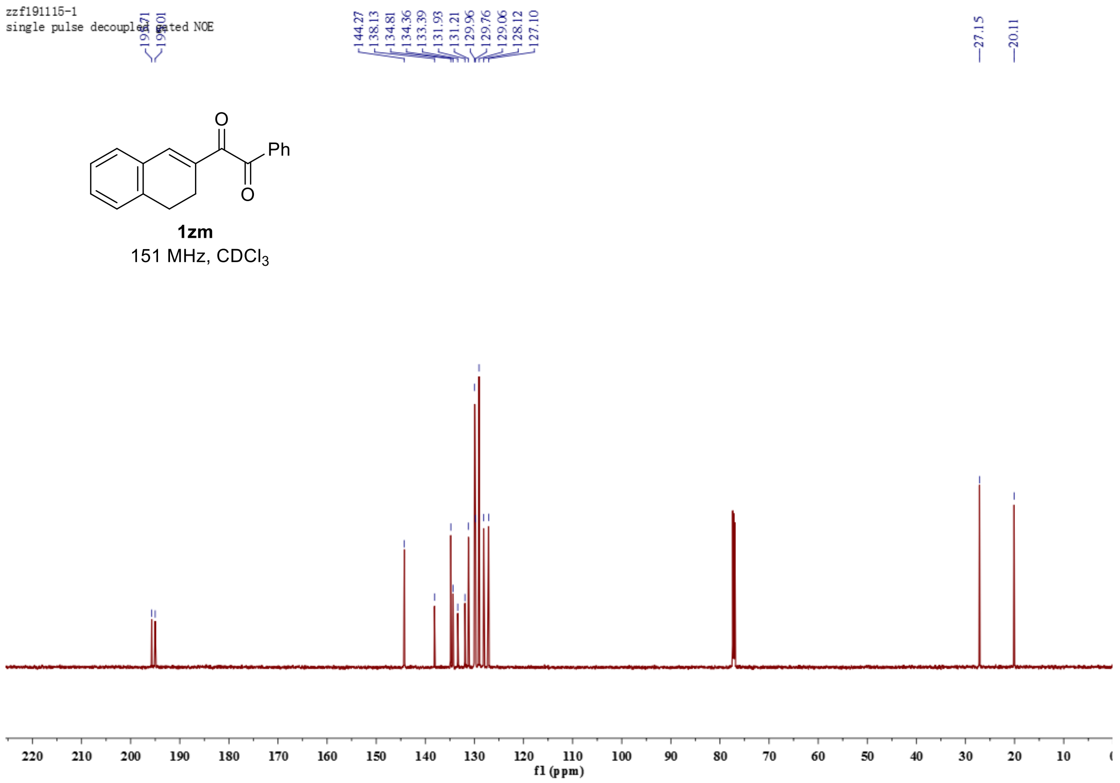

zzf191115-1
single pulse decoupled gated NOE
194.71
194.01
144.27
138.13
134.81
134.36
133.39
131.93
131.21
129.96
129.76
129.06
128.12
127.10
27.15
20.11
O
Ph
O
1zm
151 MHz, CDCl3
f1 (ppm)

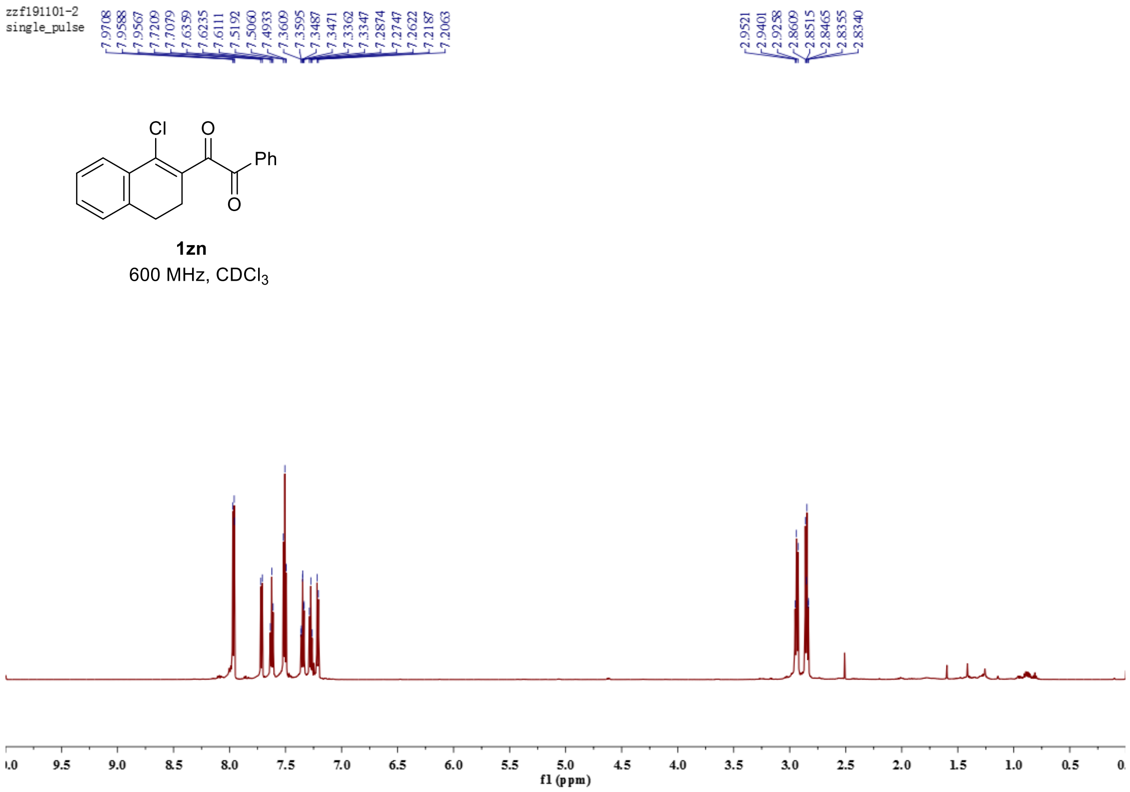

1zn
600 MHz, $CDCl_3$

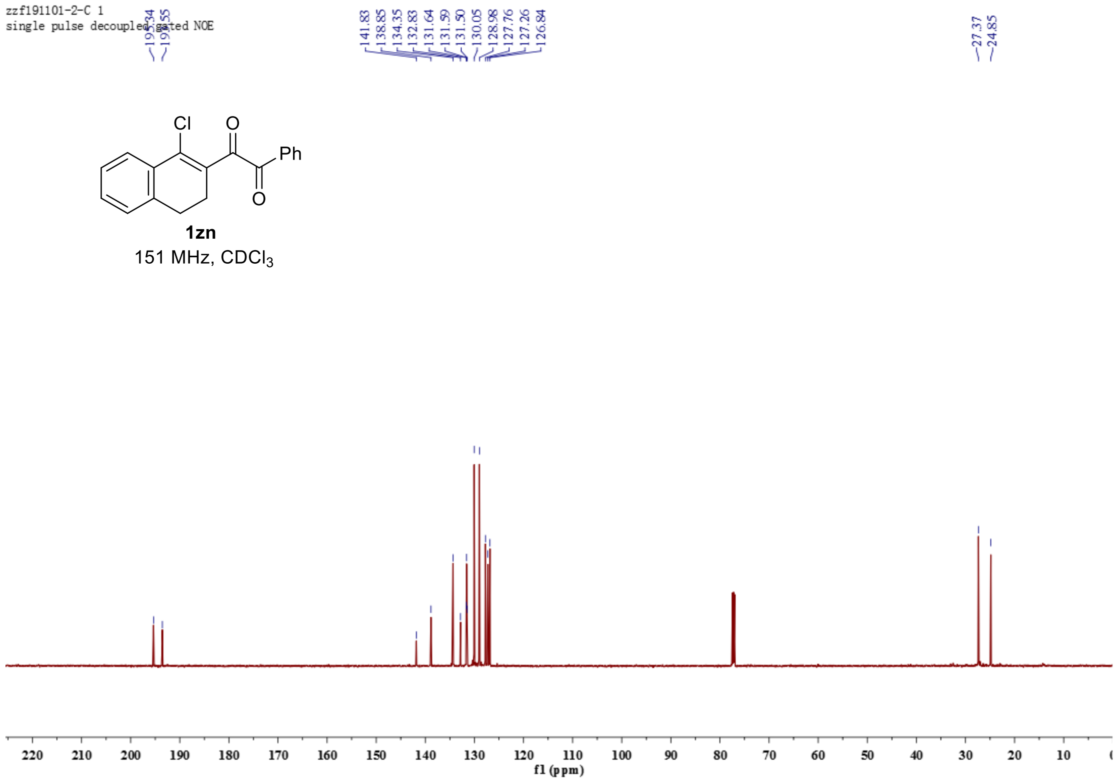

1zn
151 MHz, $CDCl_3$

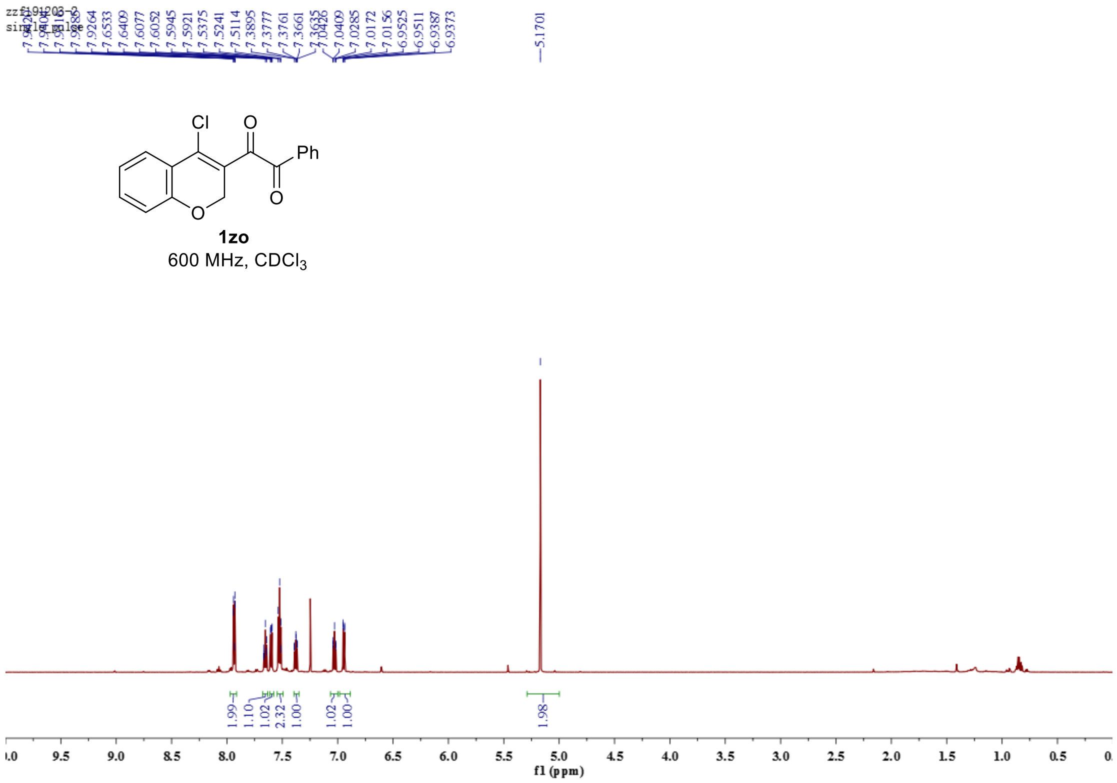
zzf191203-2
single pulse
1zo
600 MHz, CDCl3
f1 (ppm)

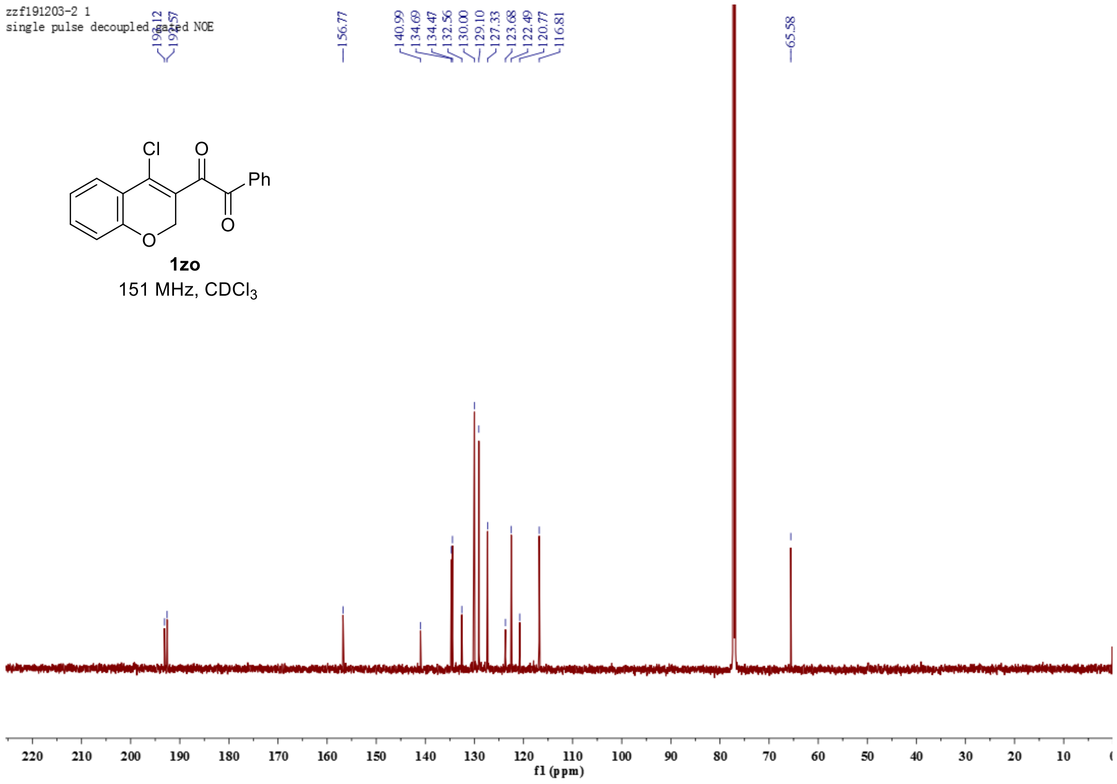
zzf191203-2 1
single pulse decoupled gated NOE
156.77
140.99
134.69
134.47
132.56
130.00
129.10
127.33
123.68
122.49
120.77
116.81
65.58
1zo
151 MHz, CDCl3
f1 (ppm)

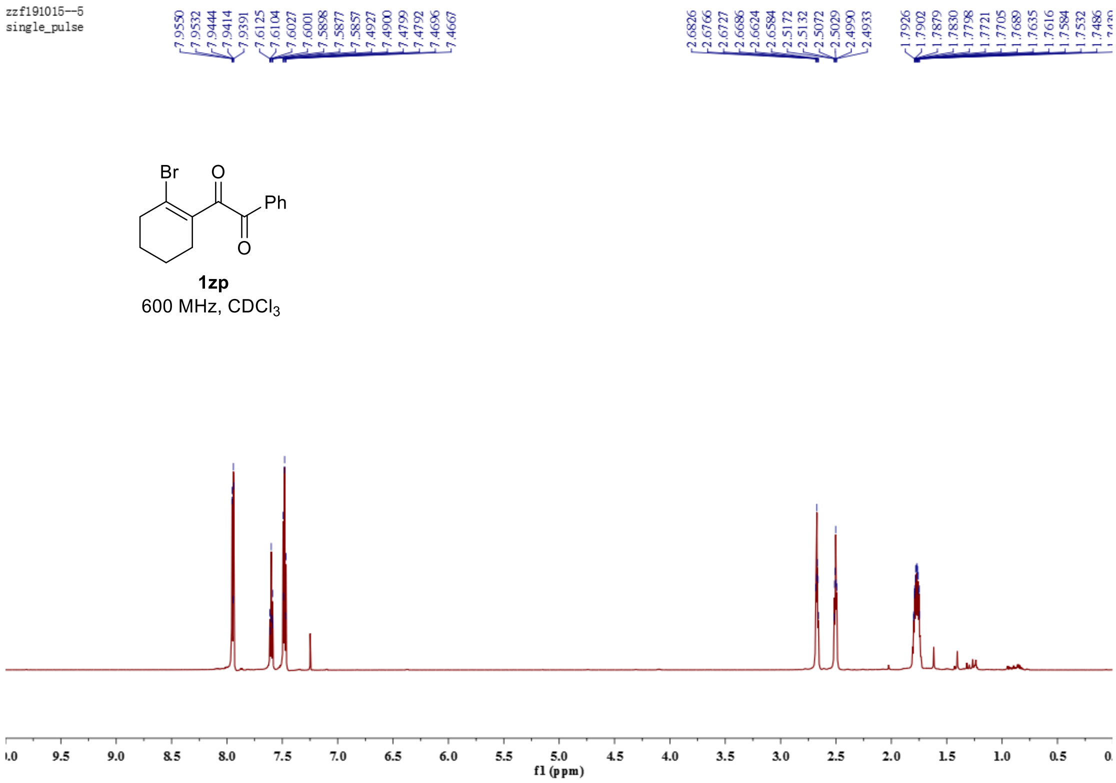
zzf191015--5
single_pulse
7.9550
7.9532
7.9444
7.9414
7.9391
7.6125
7.6104
7.6027
7.6001
7.5898
7.5877
7.5857
7.4927
7.4900
7.4799
7.4792
7.4696
7.4667
2.6826
2.6766
2.6727
2.6686
2.6624
2.6584
2.5172
2.5132
2.5072
2.5029
2.4990
2.4933
1.7926
1.7902
1.7879
1.7830
1.7798
1.7721
1.7705
1.7689
1.7635
1.7616
1.7584
1.7532
1.7486
Br
O
Ph
O
1zp
600 MHz, CDCl3
f1 (ppm)

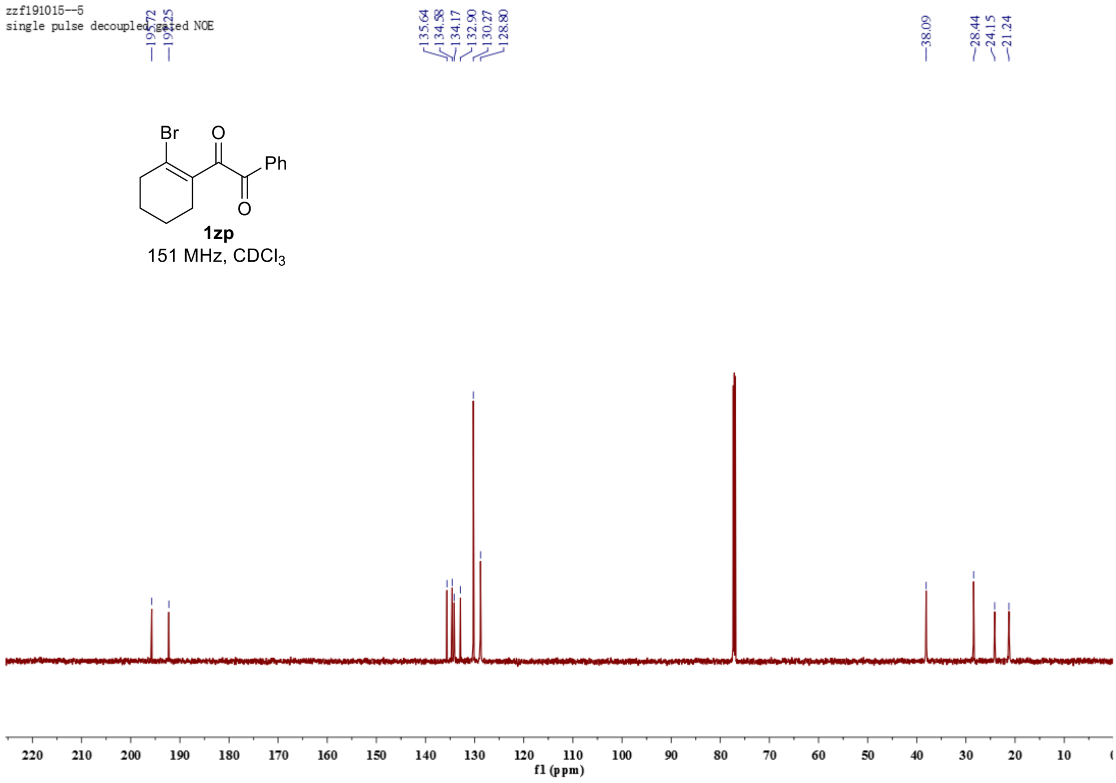
zzf191015--5
single pulse decoupled gated NOE
135.64
134.58
134.17
132.90
130.27
128.80
38.09
28.44
24.15
21.24
Br
O
Ph
O
1zp
151 MHz, CDCl3
f1 (ppm)

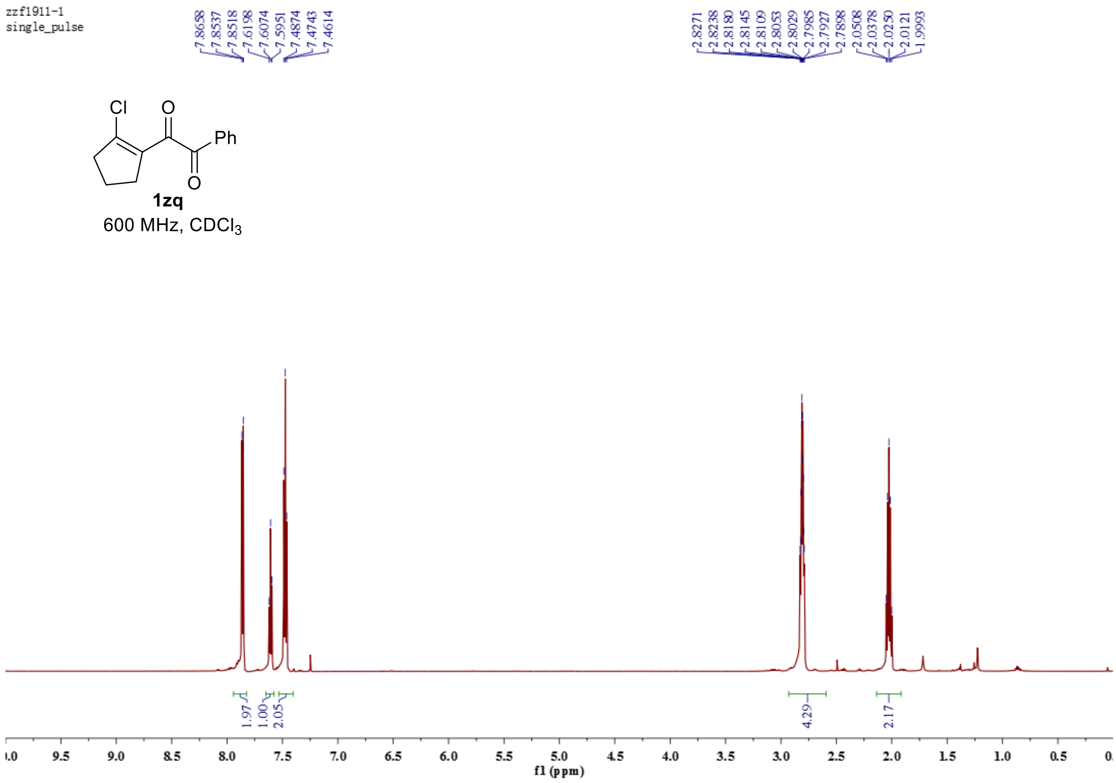
zzf1911-1
single_pulse
1zq
600 MHz, CDCl3
f1 (ppm)

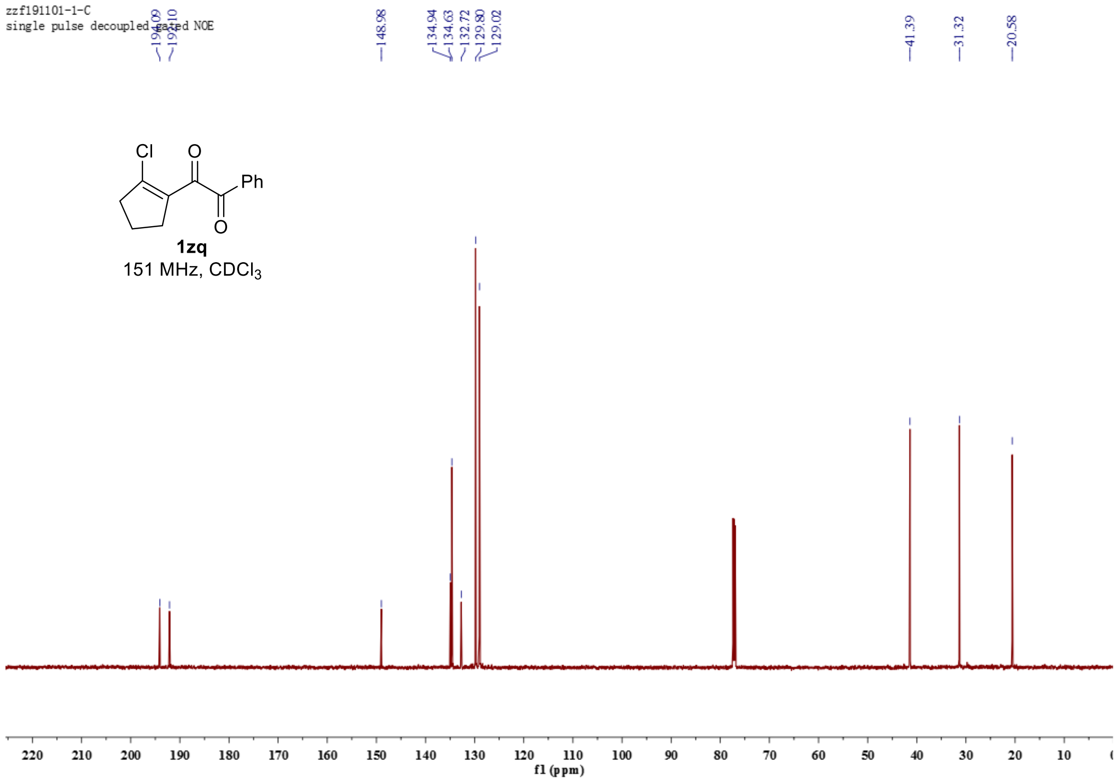
zzf191101-1-C
single pulse decoupled gated NOE
1zq
151 MHz, CDCl3
f1 (ppm)

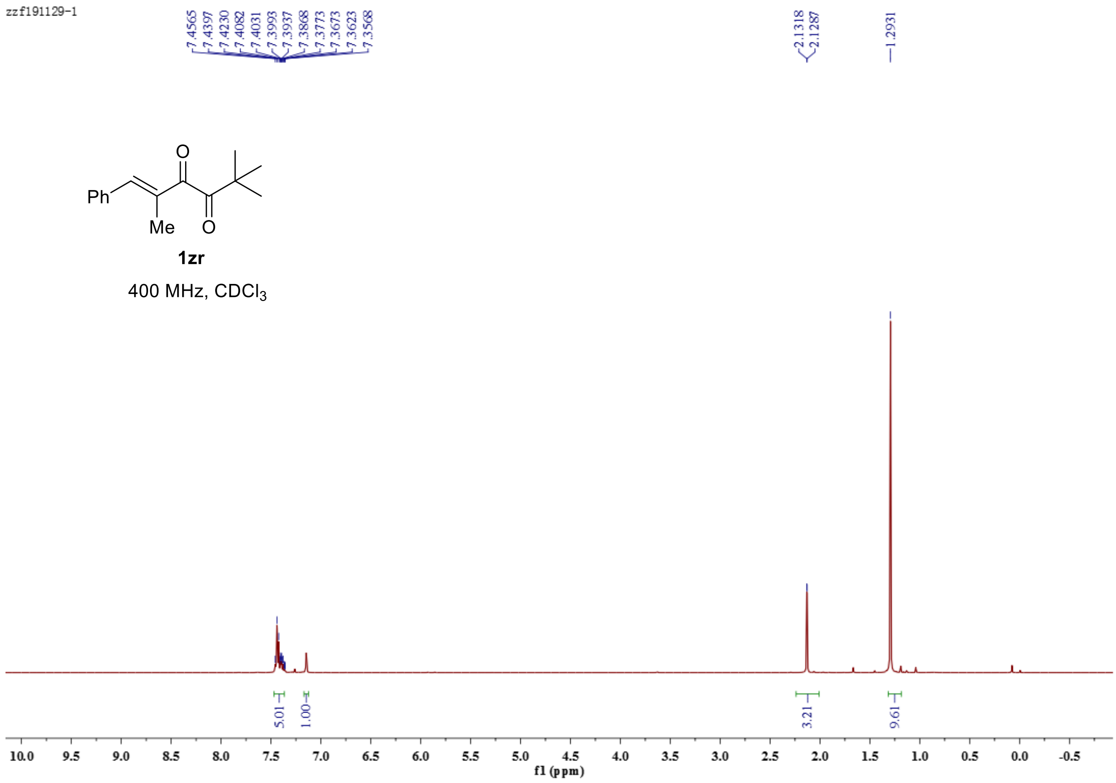

zzf191129-1
7.4565
7.4397
7.4230
7.4082
7.4031
7.3993
7.3937
7.3868
7.3773
7.3673
7.3623
7.3568
2.1318
2.1287
1.2931
Ph
Me
O
O
1zr
400 MHz, CDCl3
5.01
1.00
3.21
9.61
f1 (ppm)

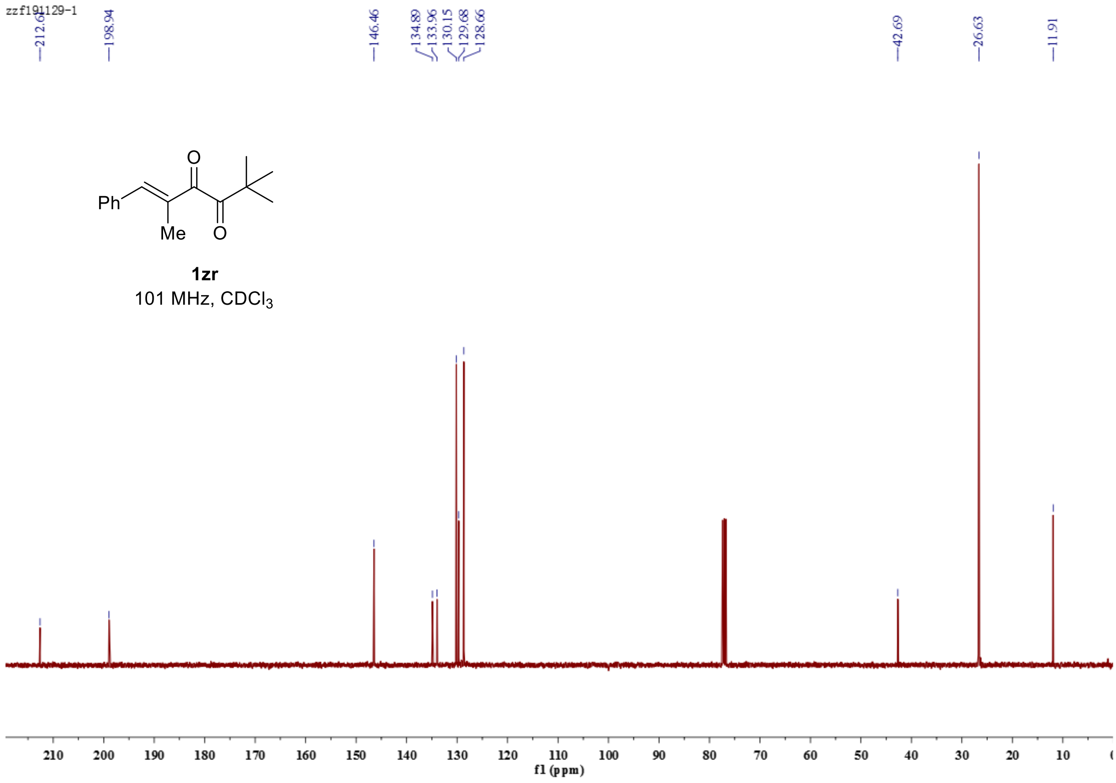

zzf191129-1
212.6
198.94
146.46
134.89
133.96
130.15
129.68
128.66
42.69
26.63
11.91
Ph
Me
O
O
1zr
101 MHz, CDCl3
f1 (ppm)

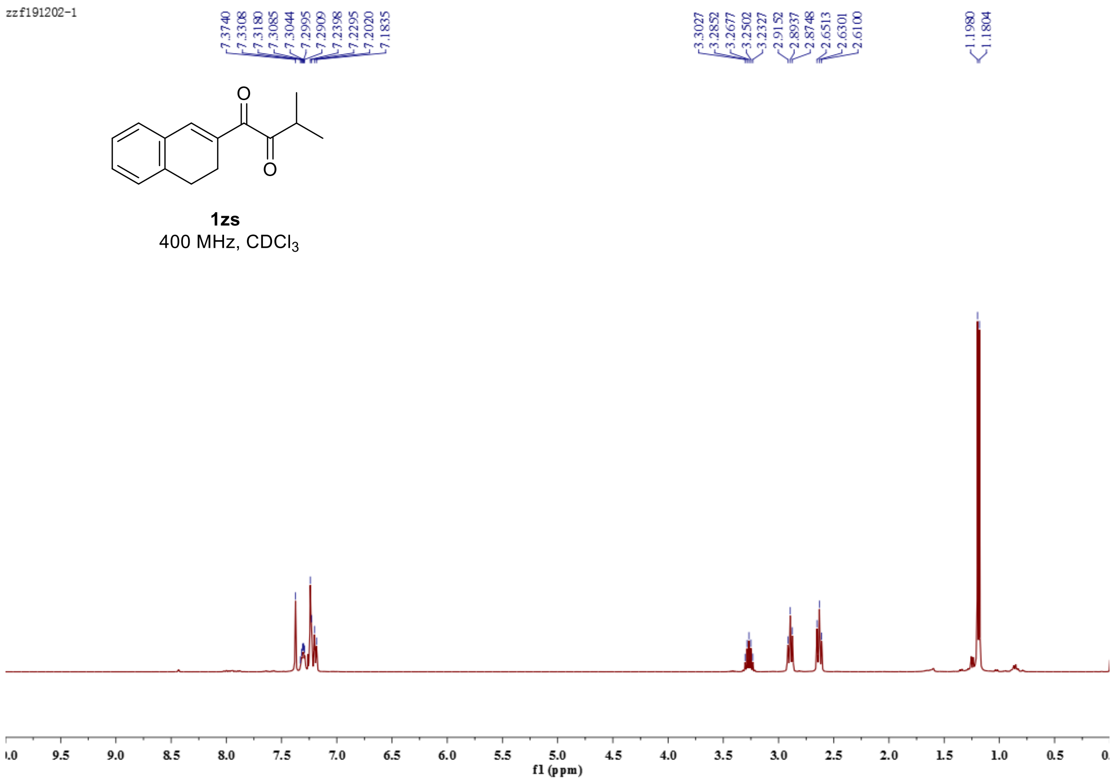
zzf191202-1
7.3740
7.3308
7.3180
7.3085
7.3044
7.2995
7.2909
7.2398
7.2295
7.2020
7.1835
3.3027
3.2852
3.2677
3.2502
3.2327
2.9152
2.8937
2.8748
2.6513
2.6301
2.6100
1.1980
1.1804
1zs
400 MHz, CDCl3
f1 (ppm)

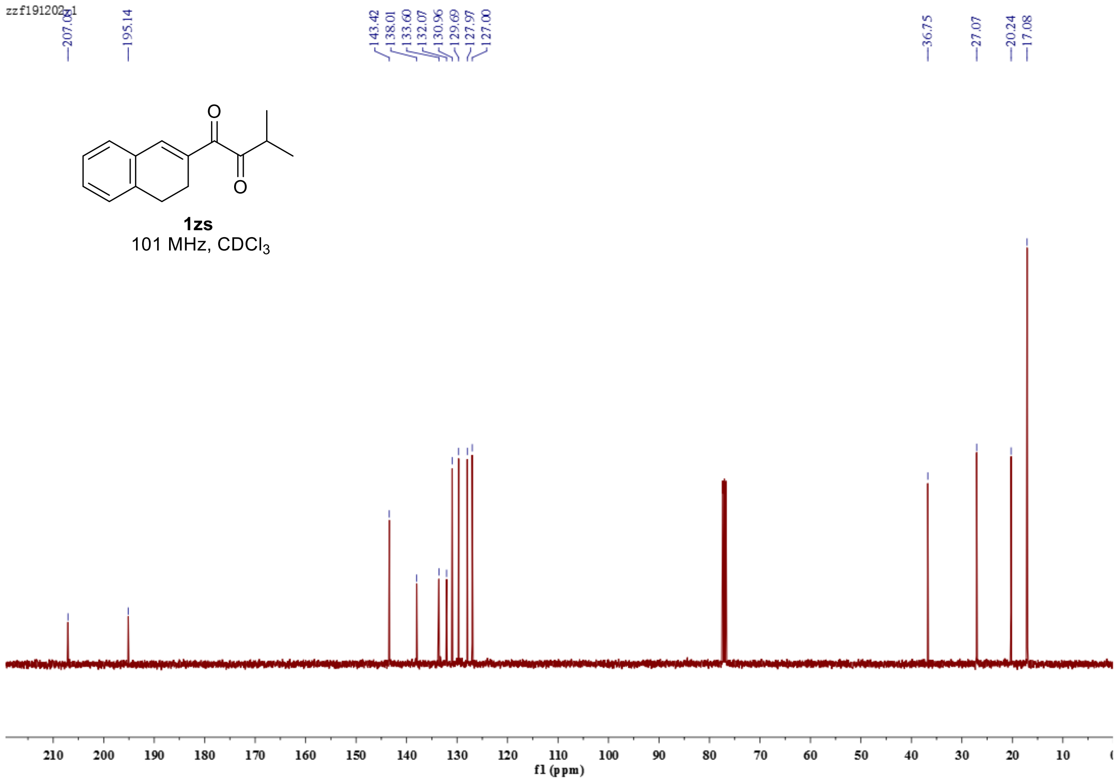
zzf191202-1
207.08
195.14
143.42
138.01
133.60
132.07
130.96
129.69
127.97
127.00
36.75
27.07
20.24
17.08
1zs
101 MHz, CDCl3
f1 (ppm)

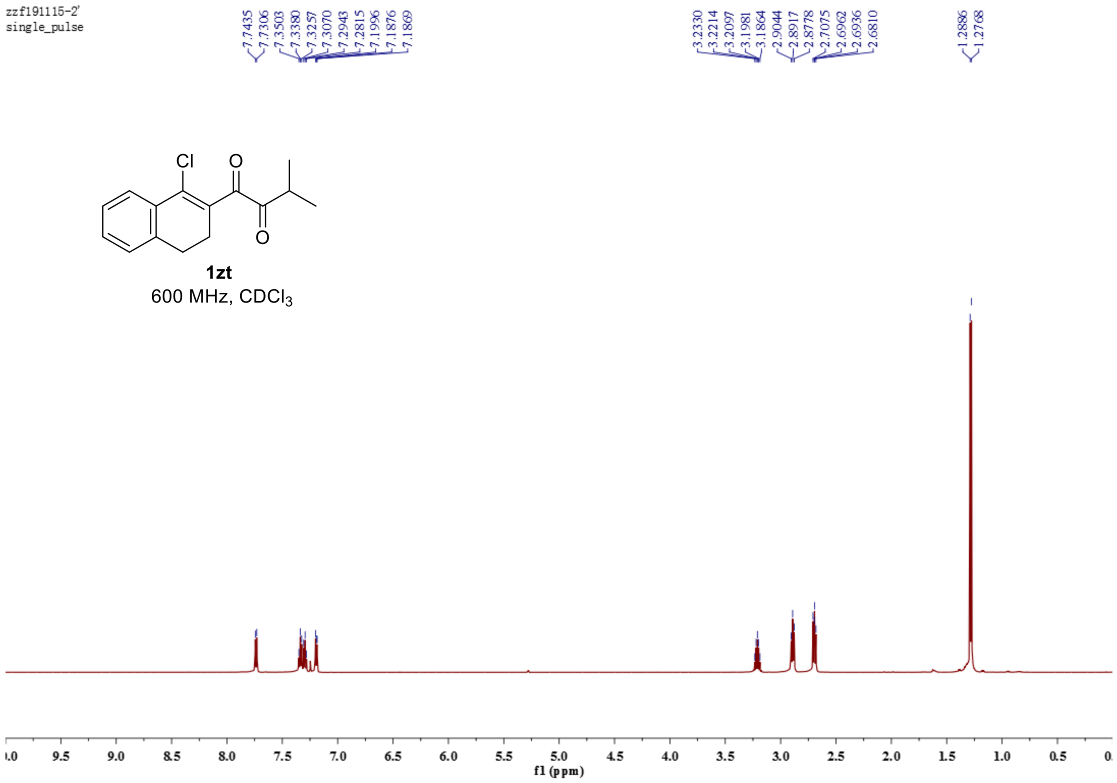
zzf191115-2'
single_pulse
7.7435
7.7306
7.3503
7.3380
7.3257
7.3070
7.2943
7.2815
7.1996
7.1876
7.1869
3.2330
3.2214
3.2097
3.1981
3.1864
2.9044
2.8917
2.8778
2.7075
2.6962
2.6936
2.6810
1.2886
1.2768
Cl
O
O
1zt
600 MHz, CDCl3
f1 (ppm)

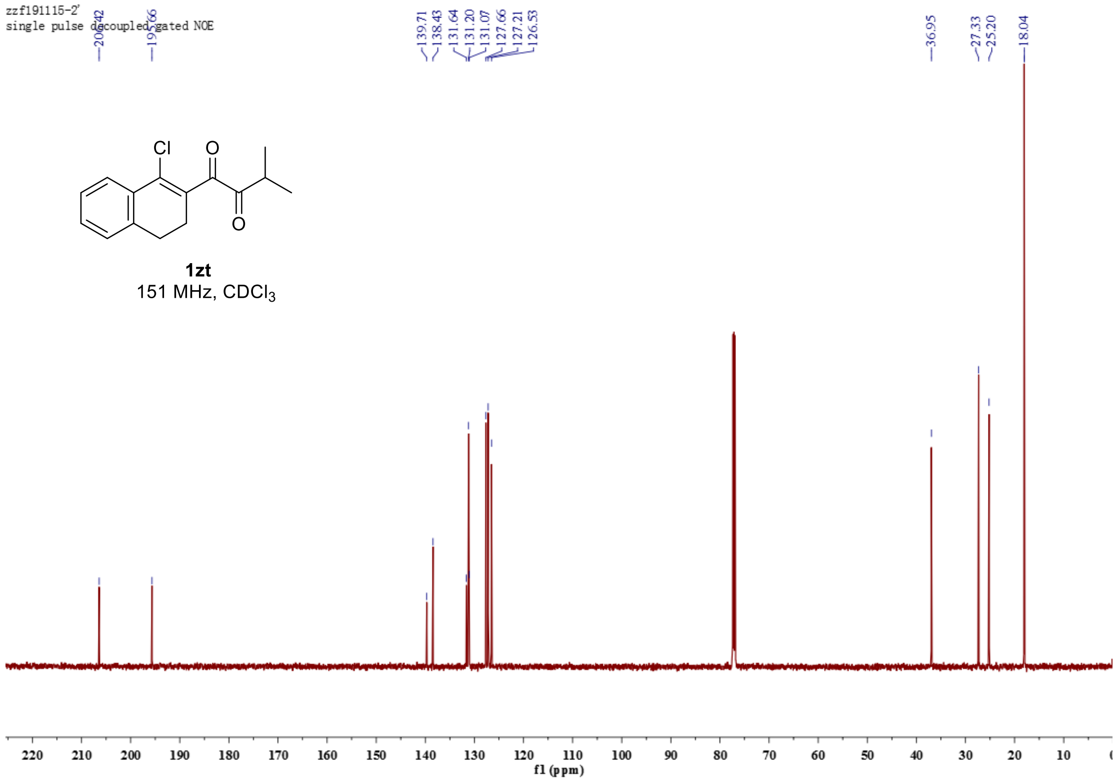
zzf191115-2'
single pulse decoupled gated NOE
139.71
138.43
131.64
131.20
131.07
127.66
127.21
126.53
36.95
27.33
25.20
18.04
Cl
O
O
1zt
151 MHz, CDCl3
f1 (ppm)

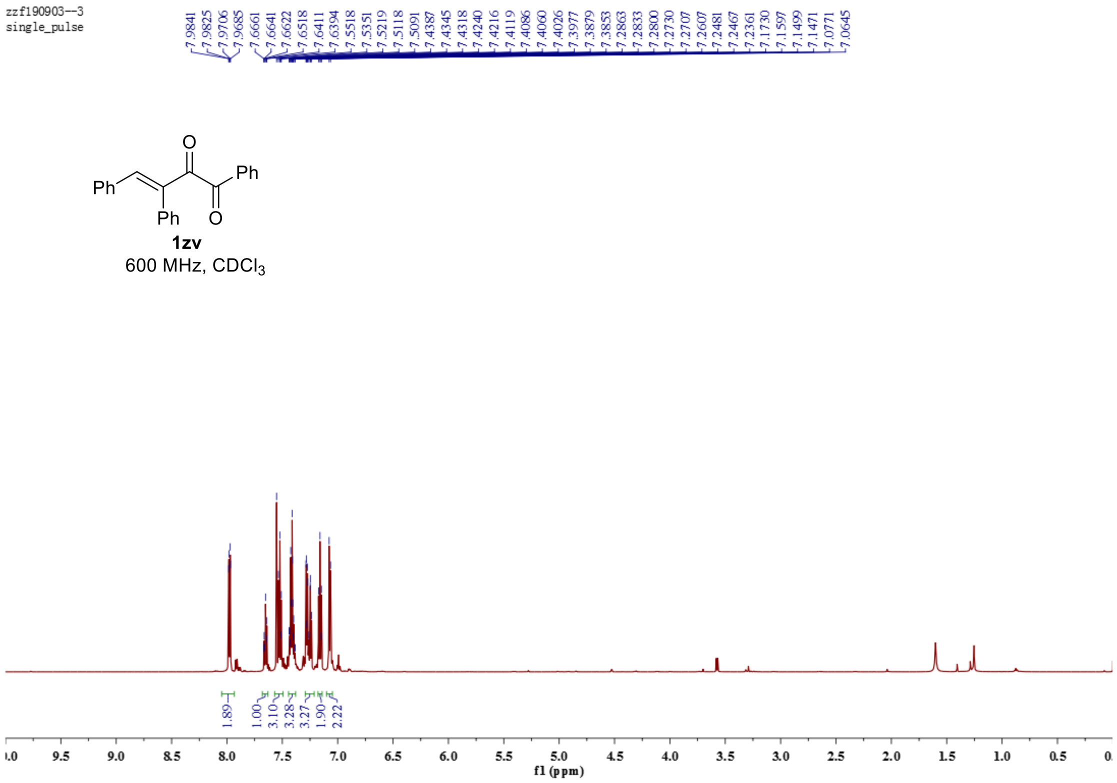
zzf190903--3
single_pulse
7.9841
7.9825
7.9706
7.9685
7.6661
7.6641
7.6622
7.6518
7.6411
7.6394
7.5518
7.5351
7.5219
7.5118
7.5091
7.4387
7.4345
7.4318
7.4240
7.4216
7.4119
7.4086
7.4060
7.4026
7.3977
7.3879
7.3853
7.2863
7.2833
7.2800
7.2730
7.2707
7.2607
7.2481
7.2467
7.2361
7.1730
7.1597
7.1499
7.1471
7.0771
7.0645
Ph
Ph
Ph
O
O
1zv
600 MHz, CDCl3
1.89
1.00
3.10
3.28
3.27
1.90
2.22
f1 (ppm)

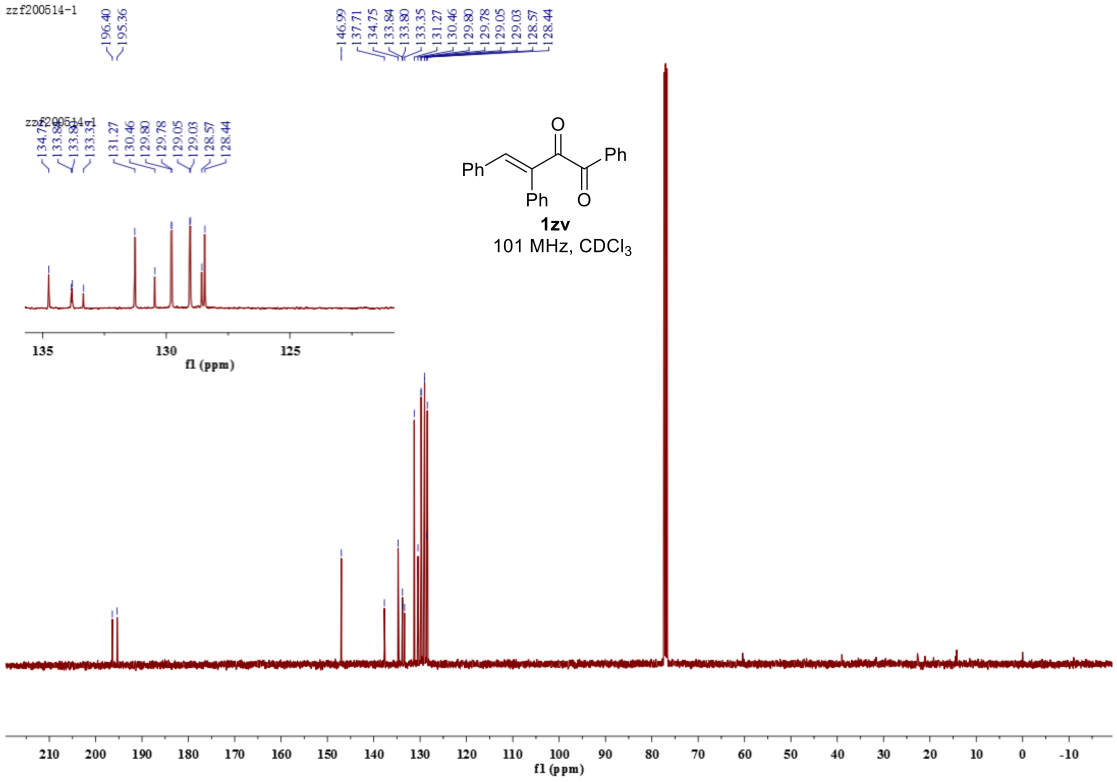
zzf200514-1
196.40
195.36
146.99
137.71
134.75
133.84
133.80
133.35
131.27
130.46
129.80
129.78
129.05
129.03
128.57
128.44
zzf200514-1
f1 (ppm)
Ph
Ph
Ph
O
O
1zv
101 MHz, CDCl3
f1 (ppm)

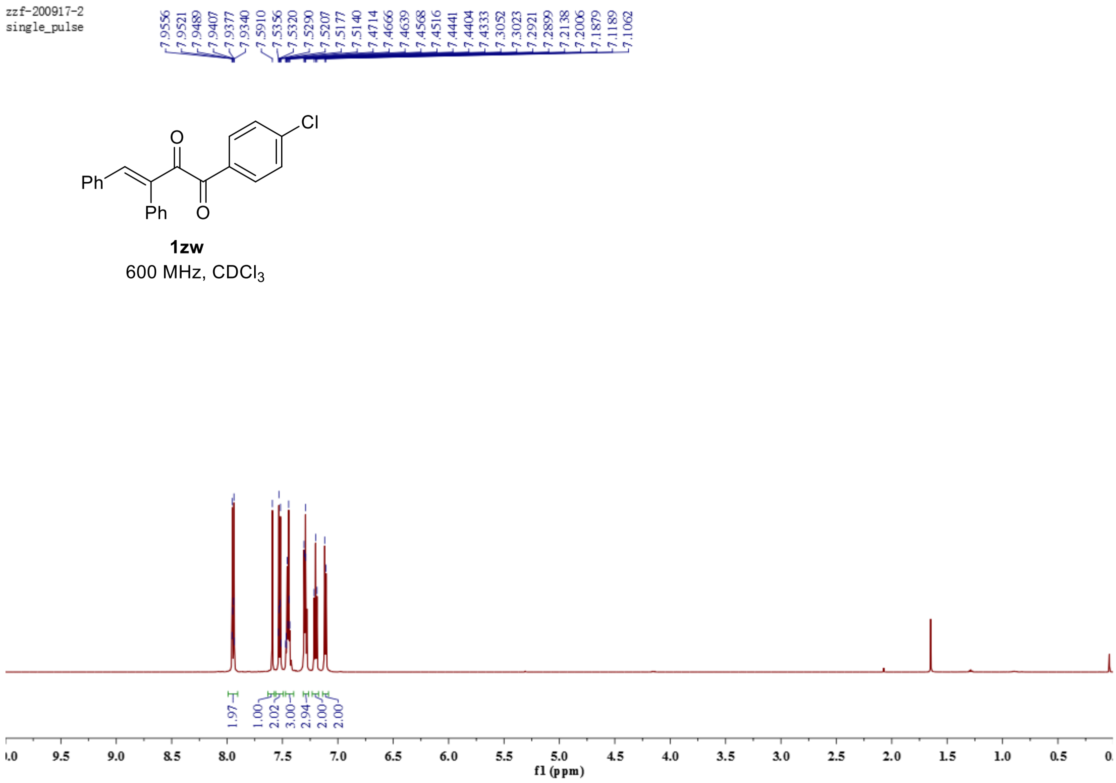

1zw

600 MHz, $CDCl_3$

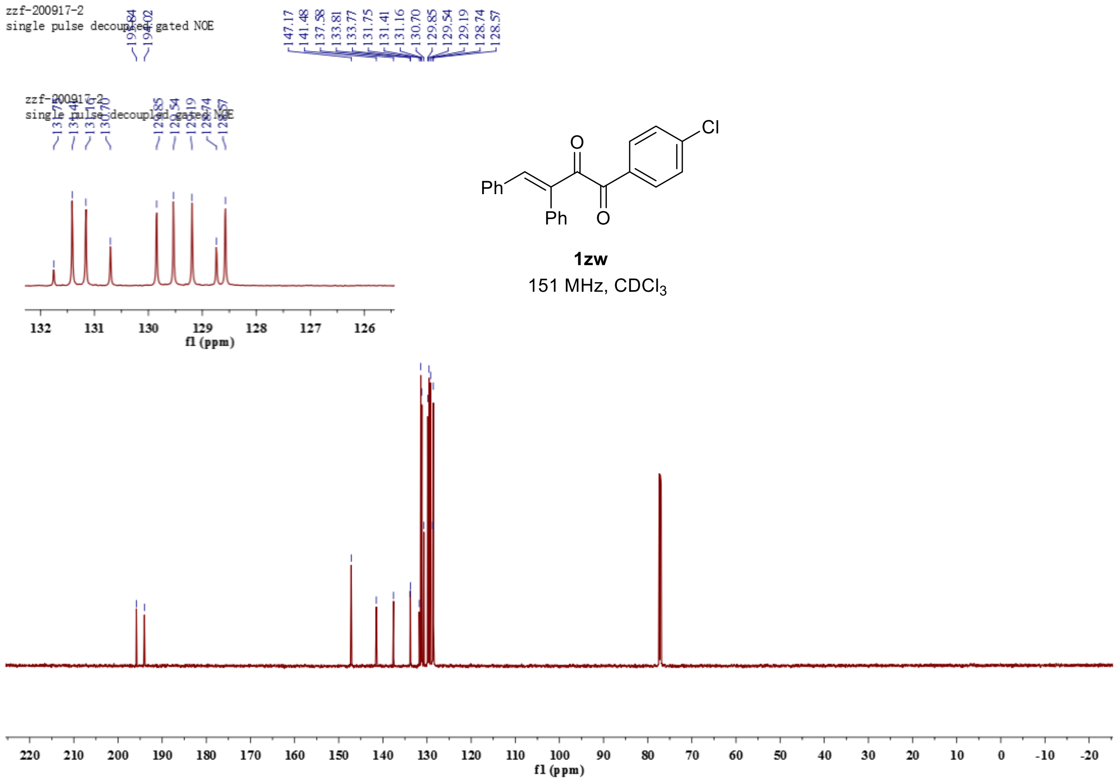

1zw

151 MHz, $CDCl_3$

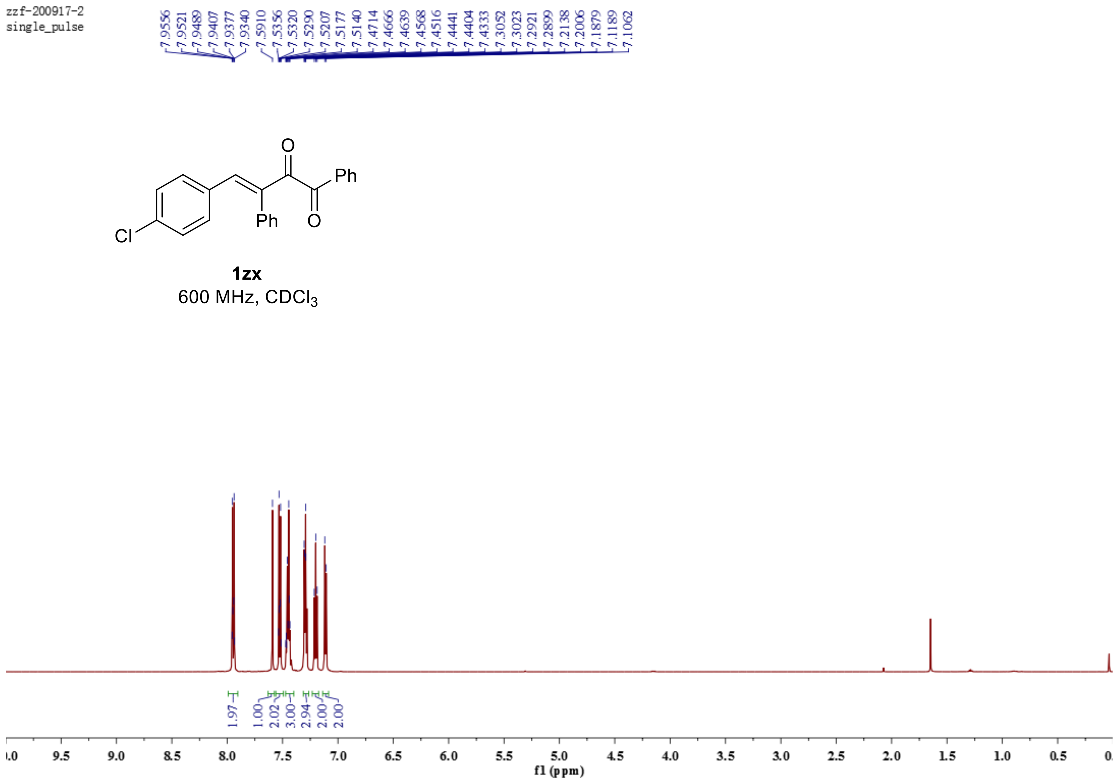
zzf-200917-2
single_pulse
1zx
600 MHz, CDCl3
f1 (ppm)

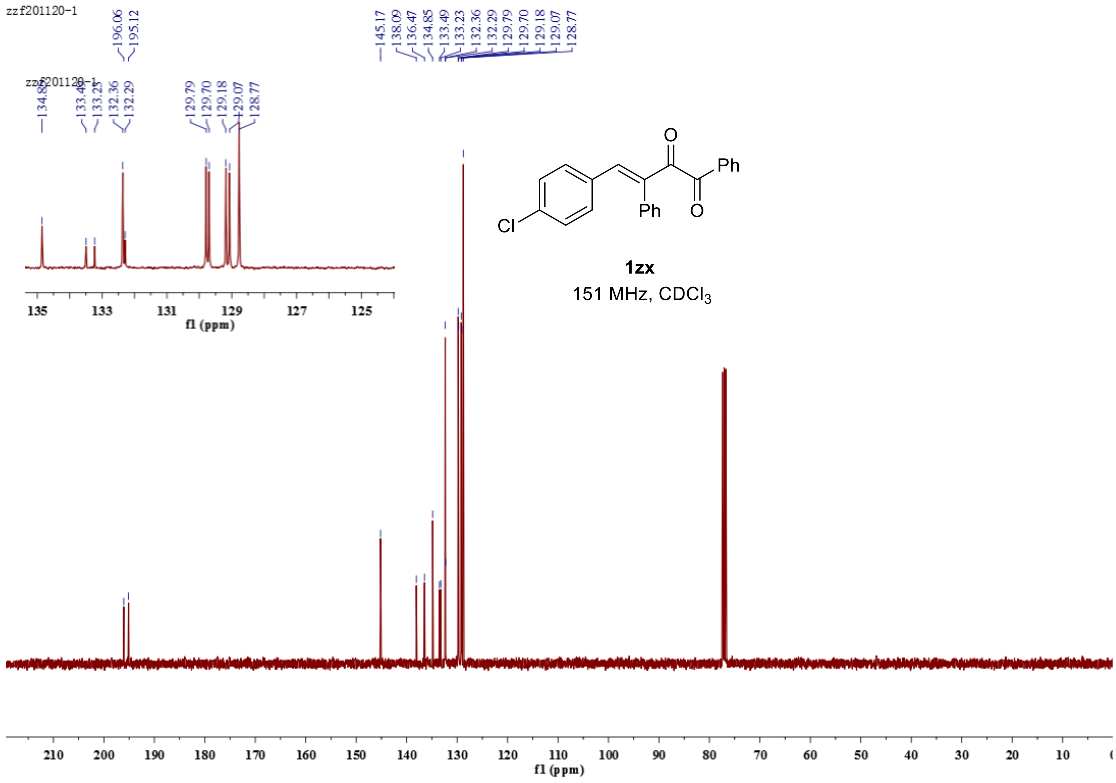
zzf201120-1
1zx
151 MHz, CDCl3
f1 (ppm)

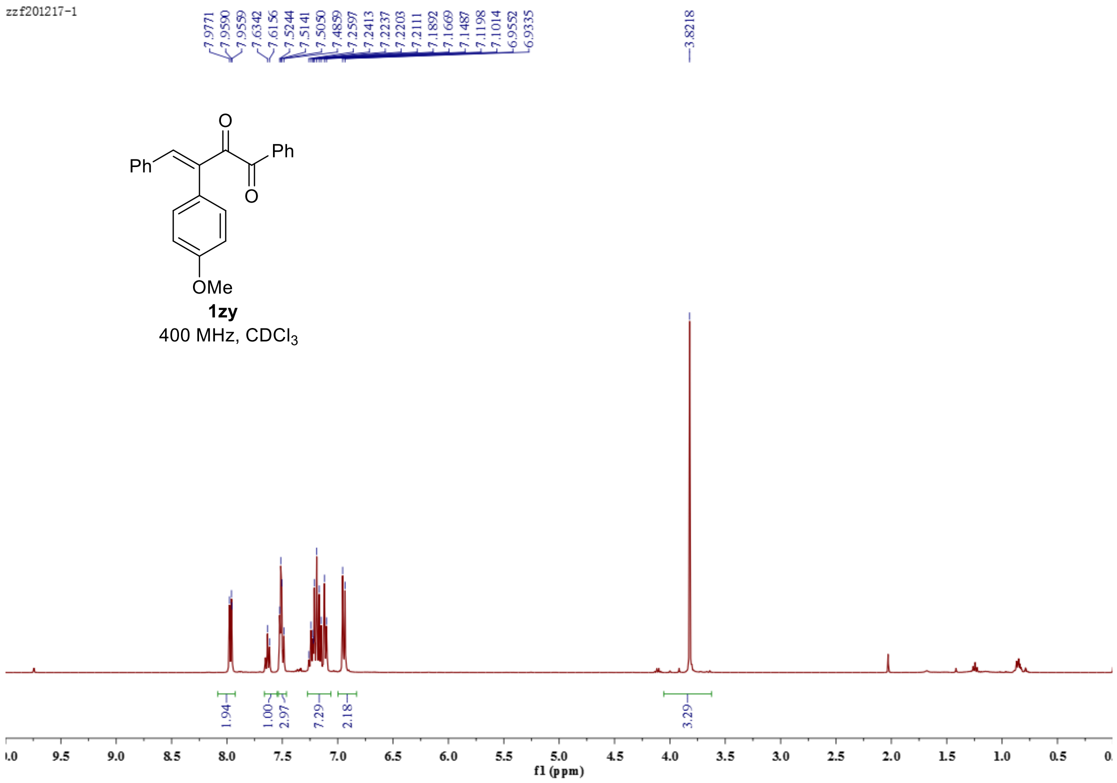
zzf201217-1
1zy
400 MHz, CDCl3
f1 (ppm)

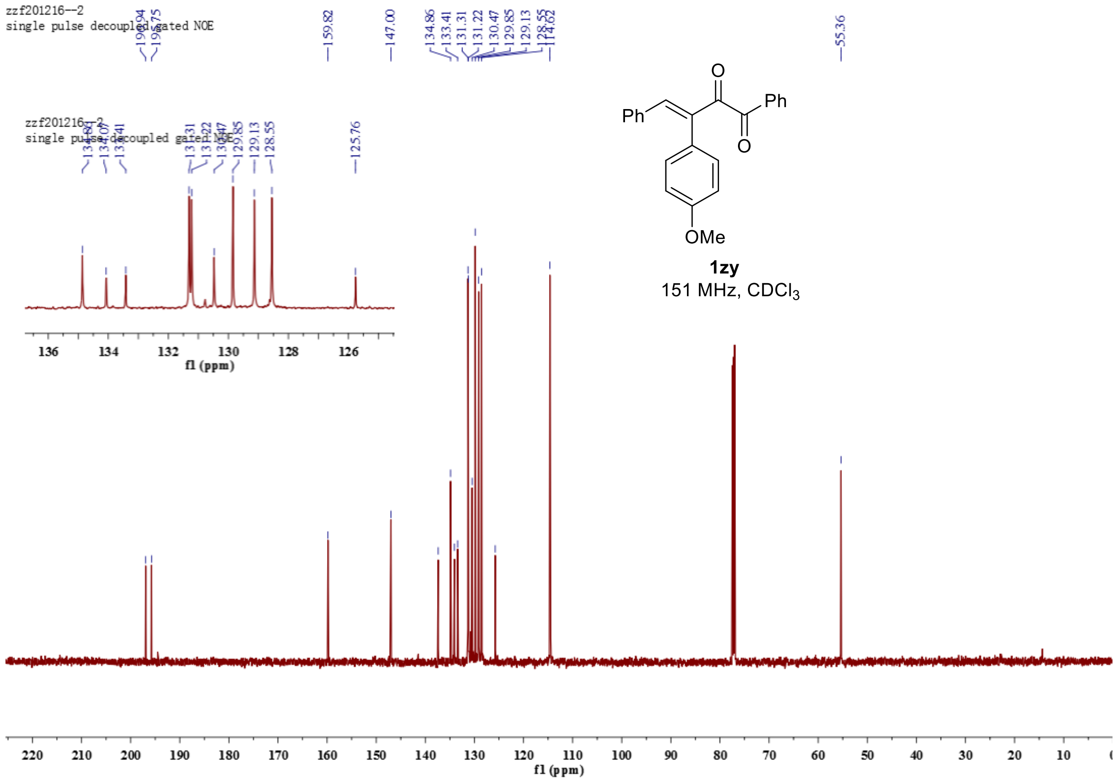
zzf201216--2
single pulse decoupled gated NOE
1zy
151 MHz, CDCl3
f1 (ppm)

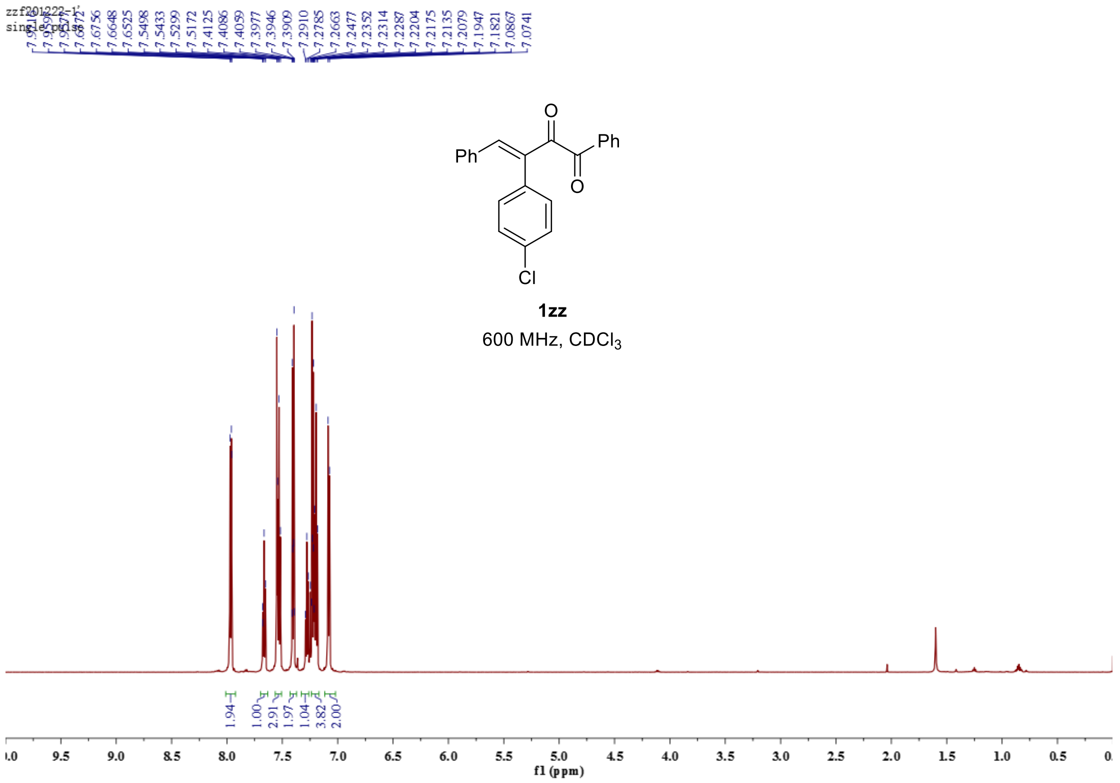

1zz

600 MHz, $CDCl_3$

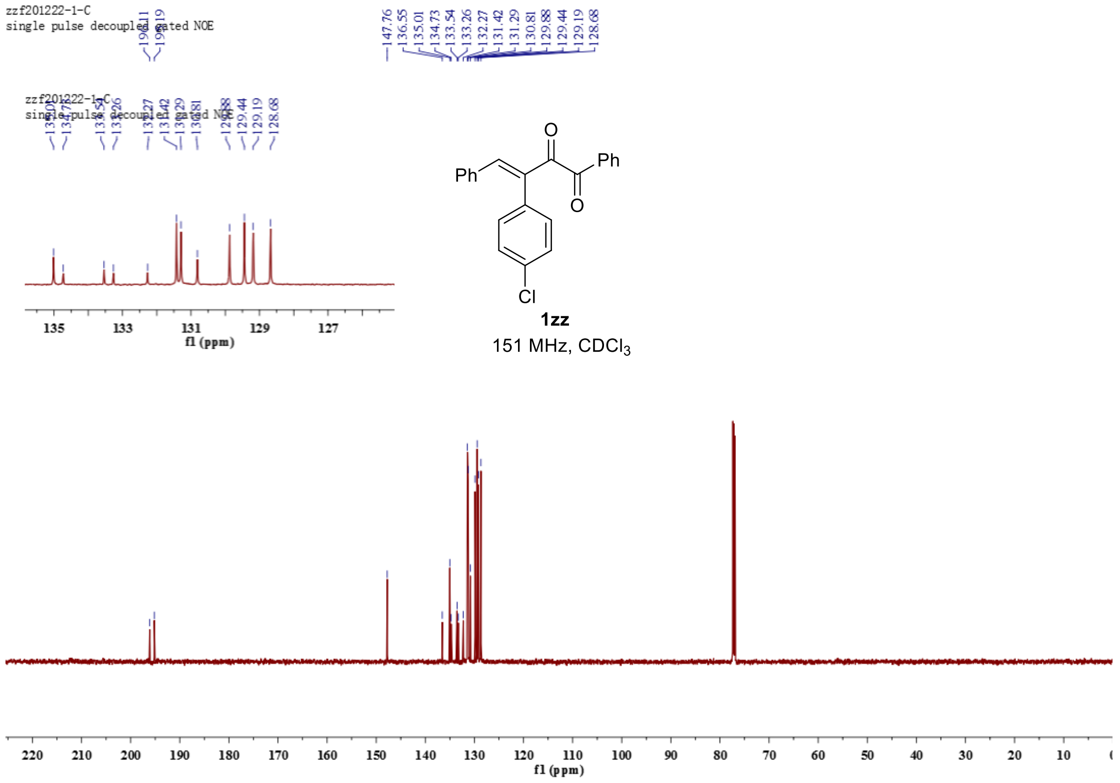

1zz

151 MHz, $CDCl_3$

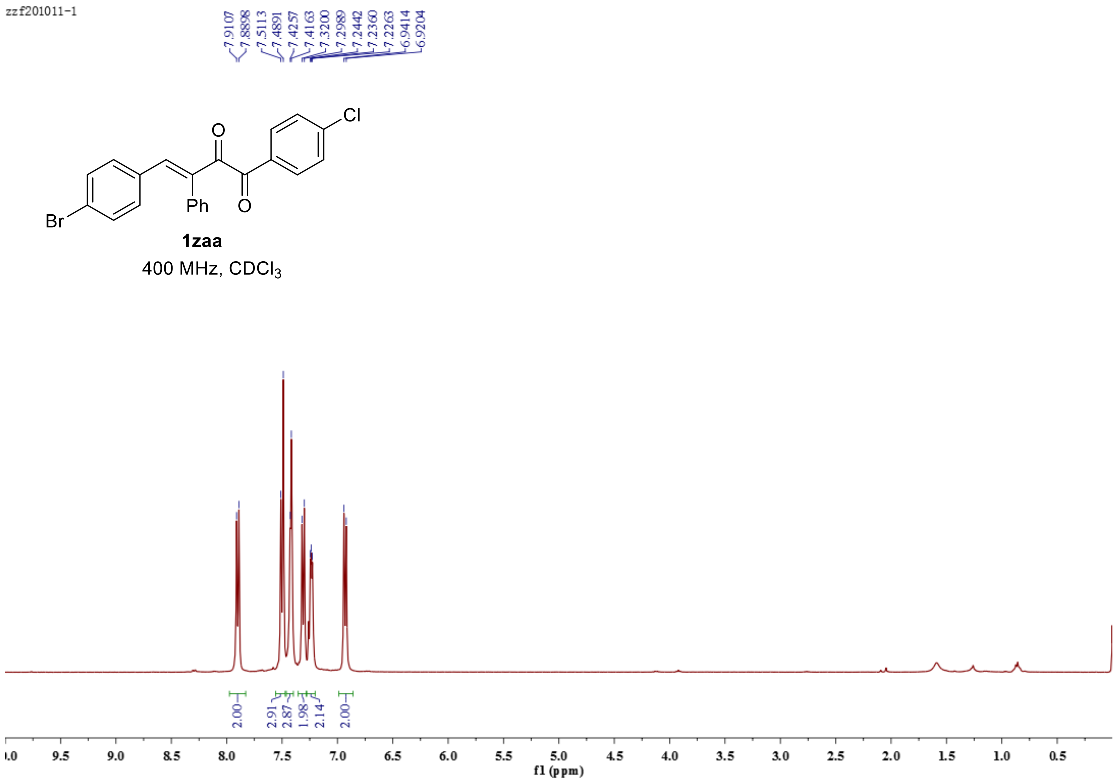
zzf201011-1
7.9107
7.8898
7.5113
7.4891
7.4257
7.4163
7.3200
7.2989
7.2442
7.2360
7.2263
6.9414
6.9204
O
O
Cl
Br
Ph
1zaa
400 MHz, CDCl3
2.00
2.91
2.87
1.98
2.14
2.00
f1 (ppm)

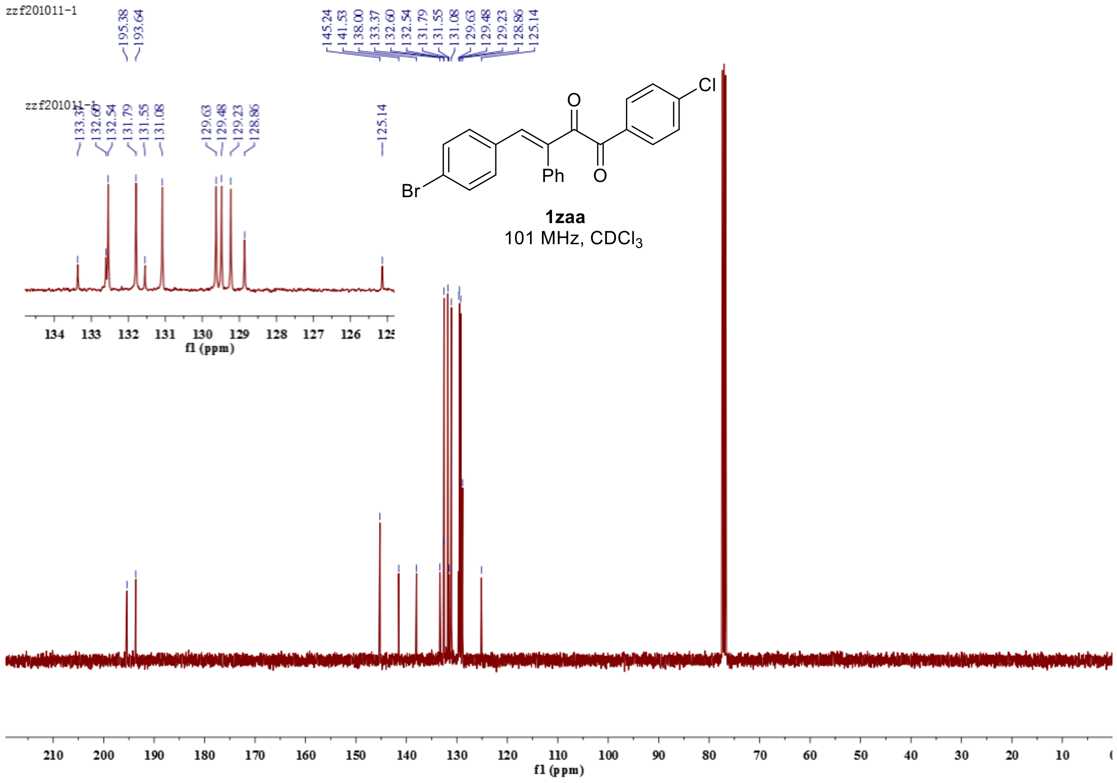
zzf201011-1
195.38
193.64
145.24
141.53
138.00
133.37
132.60
132.54
131.79
131.55
131.08
129.63
129.48
129.23
128.86
125.14
O
O
Cl
Br
Ph
1zaa
101 MHz, CDCl3
f1 (ppm)

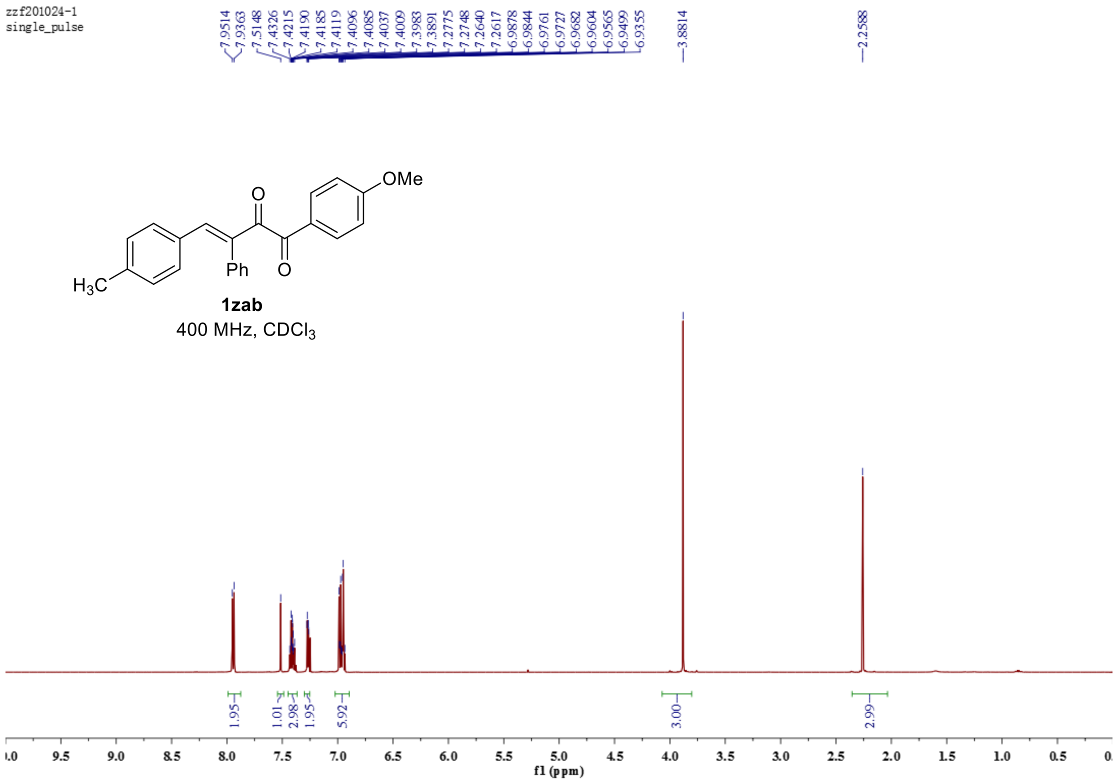
zzf201024-1
single_pulse
7.9514
7.9363
7.5148
7.4326
7.4215
7.4190
7.4185
7.4119
7.4096
7.4085
7.4037
7.4009
7.3983
7.3891
7.2775
7.2748
7.2640
7.2617
6.9878
6.9844
6.9761
6.9727
6.9682
6.9604
6.9565
6.9499
6.9355
3.8814
2.2588
OMe
O
O
Ph
H_3C
1zab
400 MHz, $CDCl_3$
1.95
1.01
2.98
1.95
5.92
3.00
2.99
f1 (ppm)

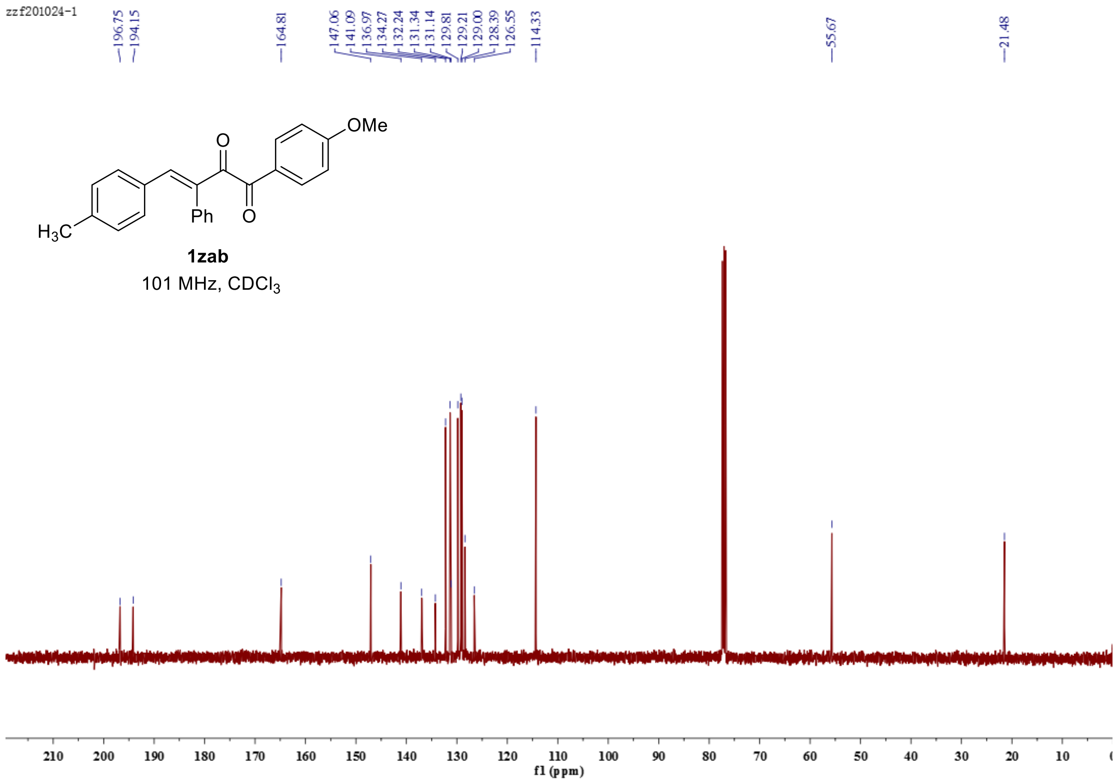
zzf201024-1
196.75
194.15
164.81
147.06
141.09
136.97
134.27
132.24
131.34
131.14
129.81
129.21
129.00
128.39
126.55
114.33
55.67
21.48
OMe
O
O
Ph
H_3C
1zab
101 MHz, $CDCl_3$
f1 (ppm)

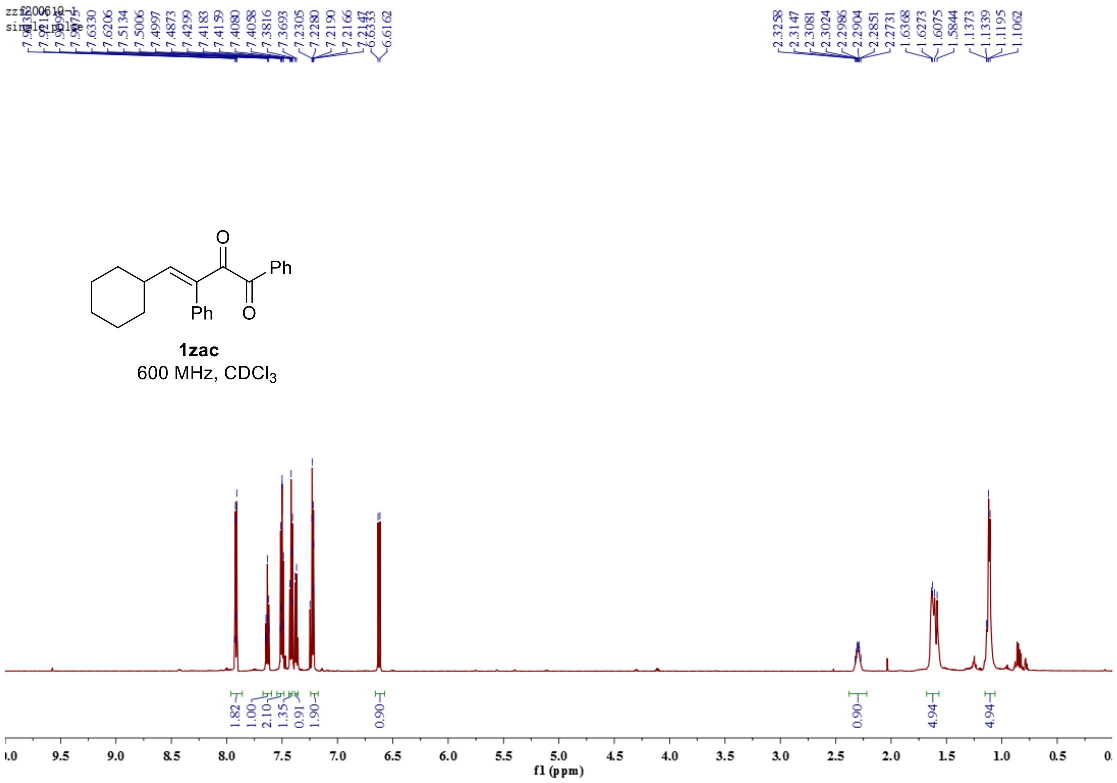

1zac
600 MHz, $CDCl_3$

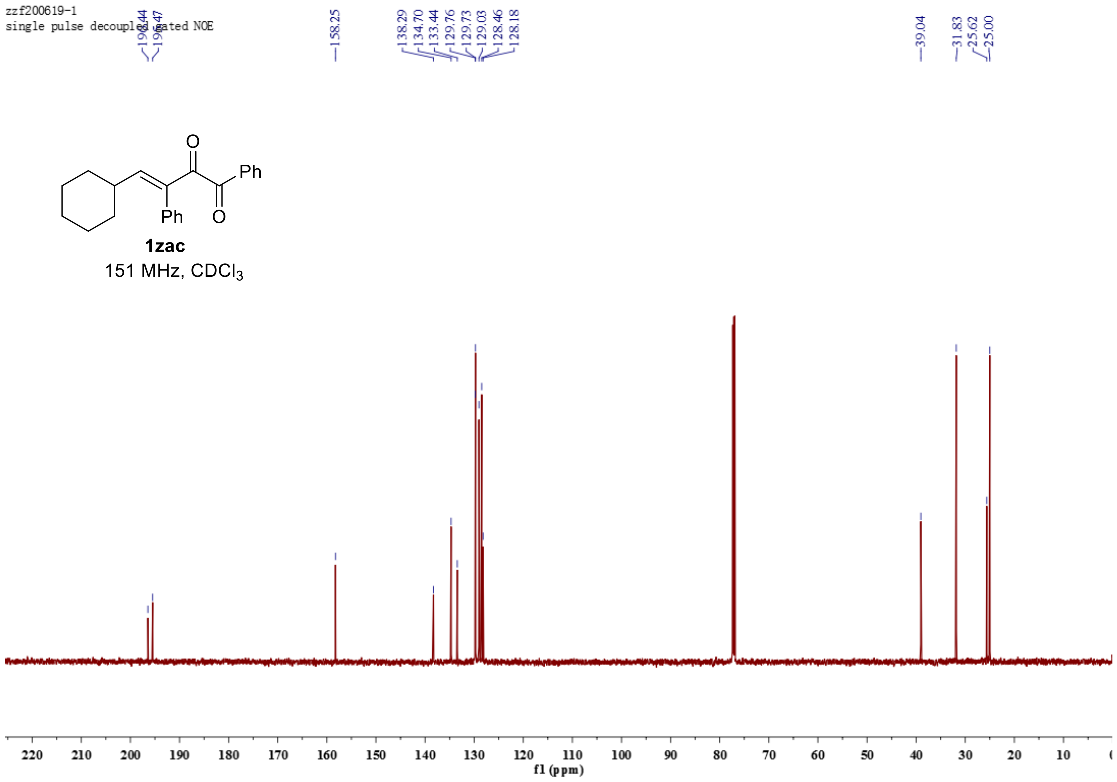

1zac
151 MHz, $CDCl_3$

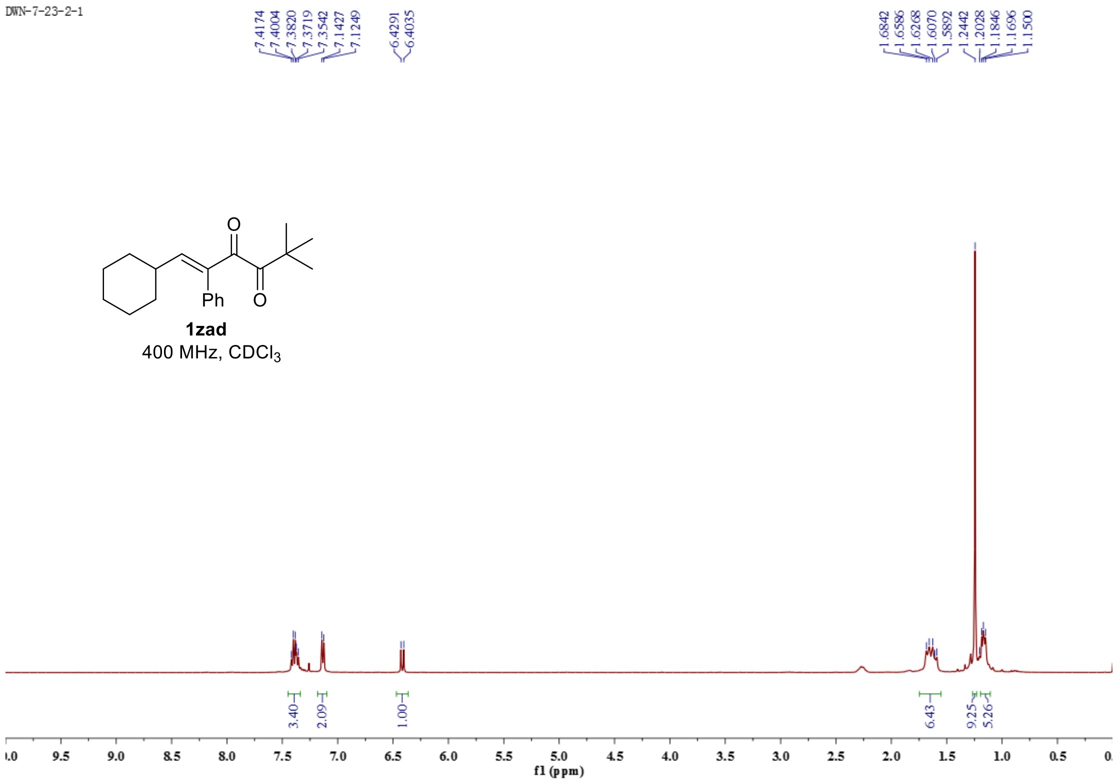
DWN-7-23-2-1
7.4174
7.4004
7.3820
7.3719
7.3542
7.1427
7.1249
6.4291
6.4035
1.6842
1.6586
1.6268
1.6070
1.5892
1.2442
1.2028
1.1846
1.1696
1.1500
Ph
1zad
400 MHz, CDCl3
3.40
2.09
1.00
6.43
9.25
5.26
f1 (ppm)

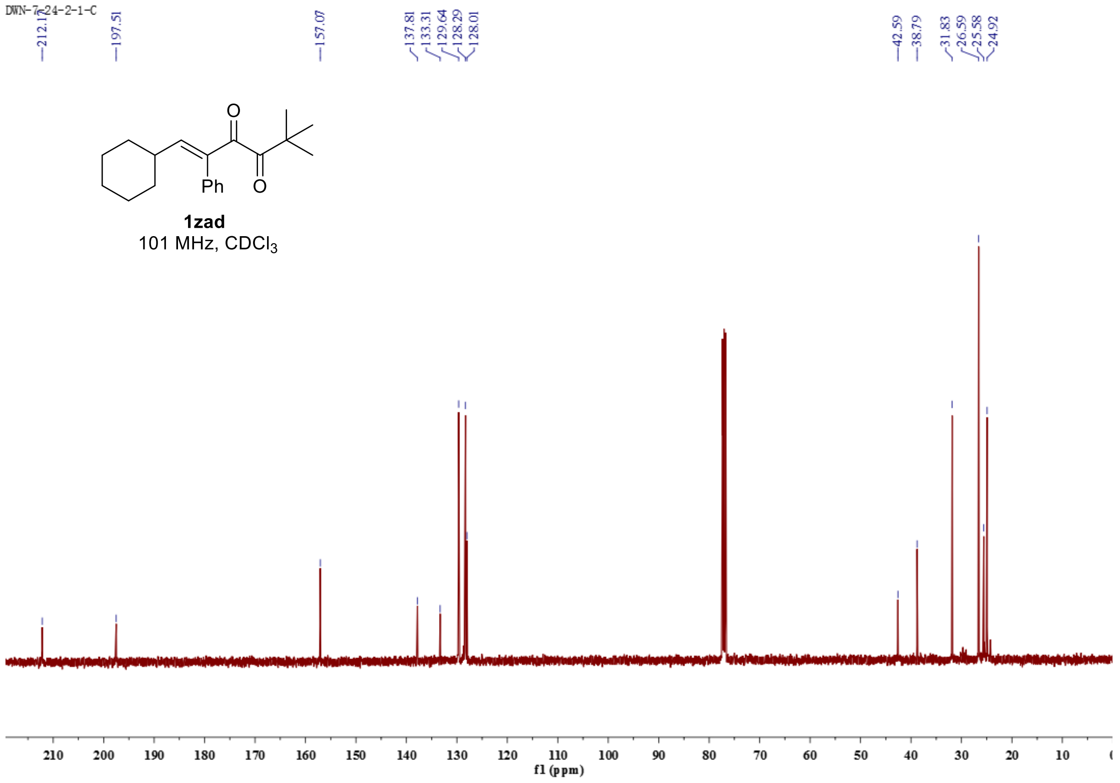
DWN-7-24-2-1-C
212.17
197.51
157.07
137.81
133.31
129.64
128.29
128.01
42.59
38.79
31.83
26.59
25.58
24.92
Ph
1zad
101 MHz, CDCl3
f1 (ppm)

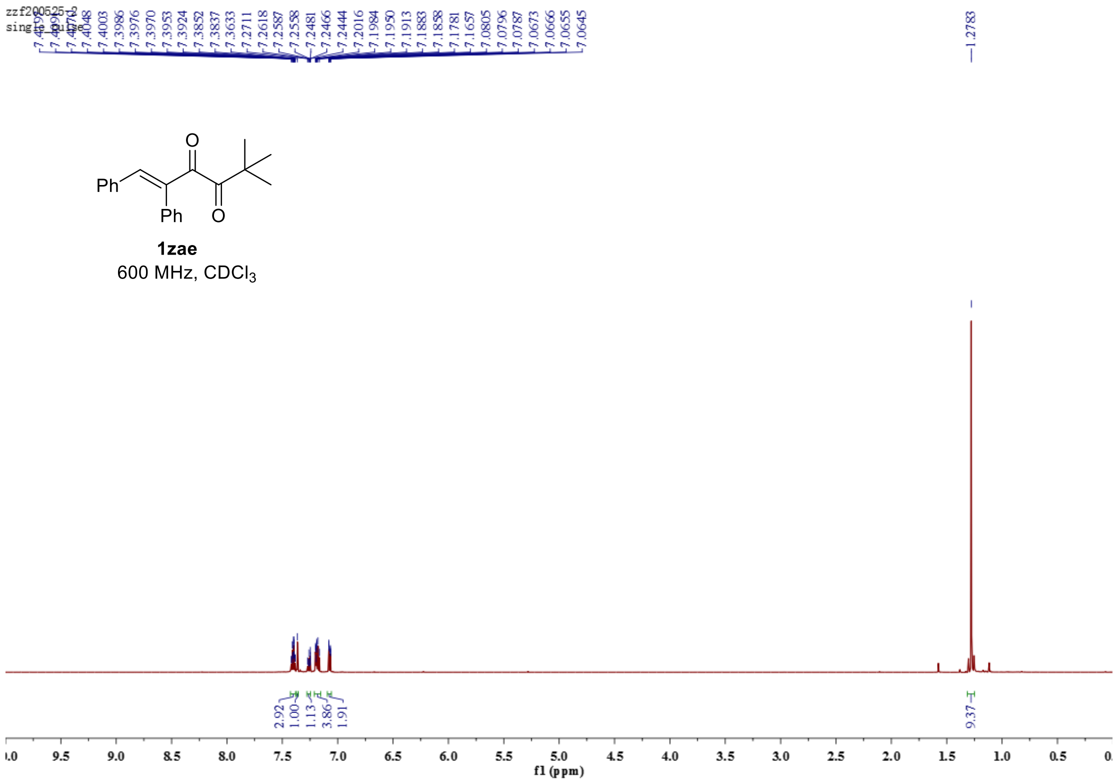
zzf200525-2
single pulse
1zae
600 MHz, CDCl3
f1 (ppm)

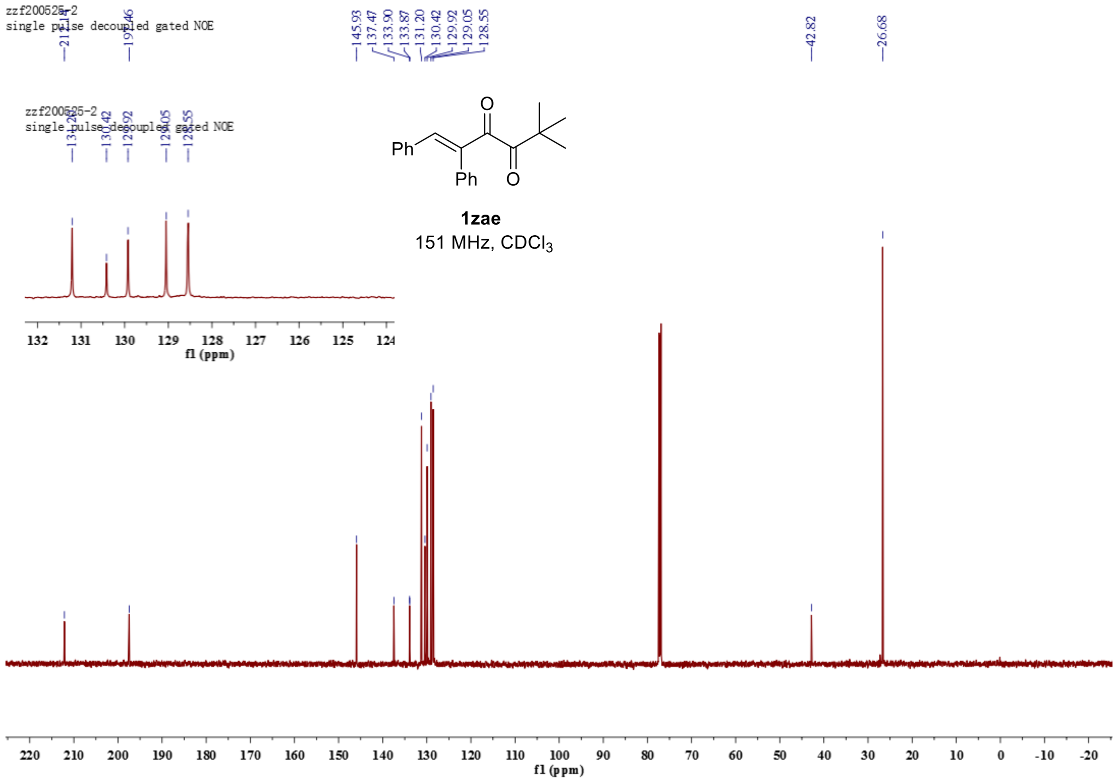
zzf200525-2
single pulse decoupled gated NOE
1zae
151 MHz, CDCl3
f1 (ppm)

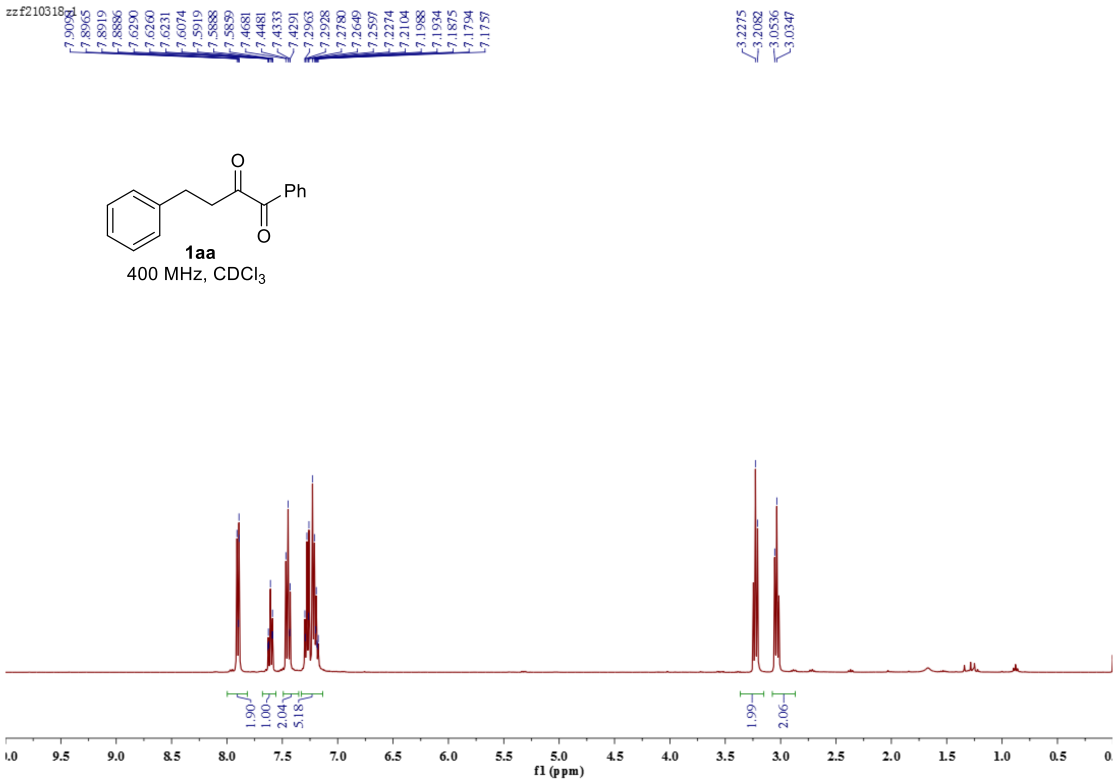
zzf210318-1
1aa
400 MHz, CDCl3
f1 (ppm)

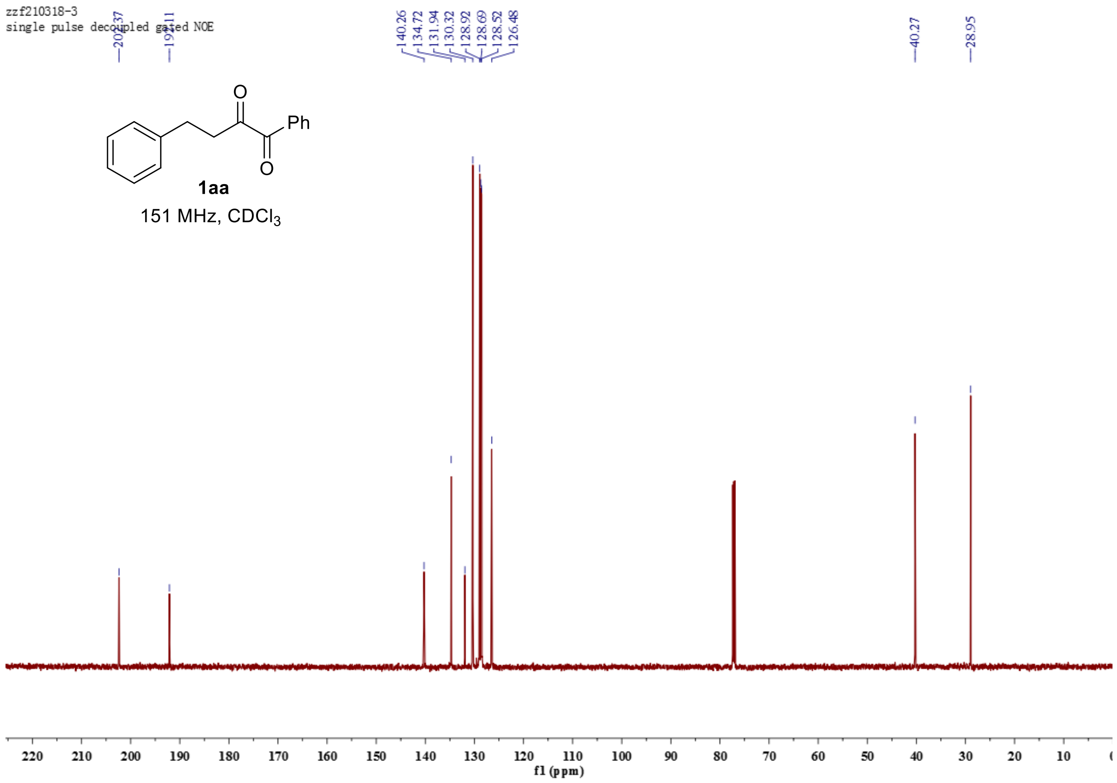
zzf210318-3
single pulse decoupled gated NOE
1aa
151 MHz, CDCl3
f1 (ppm)

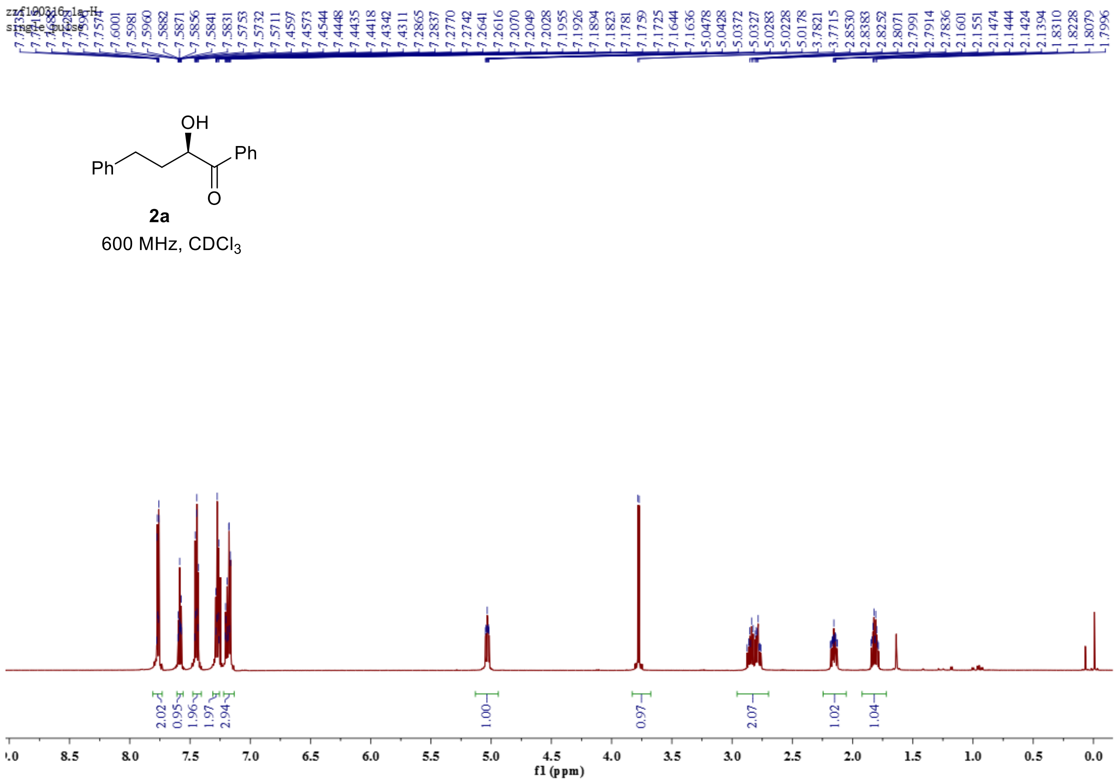
zzf190316-1a-H
single pulse
7.6001
7.5981
7.5960
7.5882
7.5871
7.5856
7.5841
7.5831
7.5753
7.5732
7.5711
7.4597
7.4573
7.4544
7.4448
7.4435
7.4418
7.4342
7.4311
7.2865
7.2837
7.2770
7.2742
7.2641
7.2616
7.2070
7.2049
7.2028
7.1955
7.1926
7.1894
7.1823
7.1781
7.1759
7.1725
7.1644
7.1636
5.0478
5.0428
5.0372
5.0327
5.0283
5.0228
5.0178
3.7821
3.7715
2.8530
2.8383
2.8252
2.8071
2.7991
2.7914
2.7836
2.1601
2.1551
2.1474
2.1444
2.1424
2.1394
1.8310
1.8228
1.8079
1.7996
OH
Ph
Ph
O
2a
600 MHz, CDCl3
2.02
0.95
1.96
1.97
2.94
1.00
0.97
2.07
1.02
1.04
f1 (ppm)

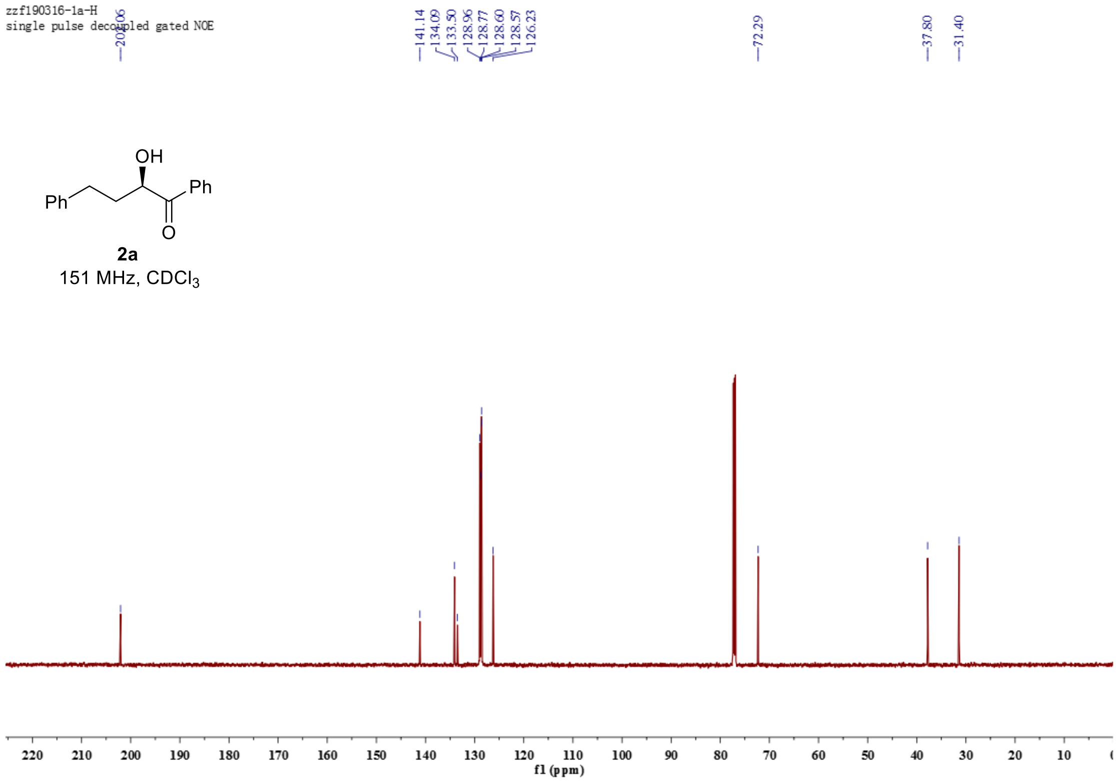
zzf190316-1a-H
single pulse decoupled gated NOE
141.14
134.09
133.50
128.96
128.77
128.60
128.57
126.23
72.29
37.80
31.40
OH
Ph
Ph
O
2a
151 MHz, CDCl3
f1 (ppm)

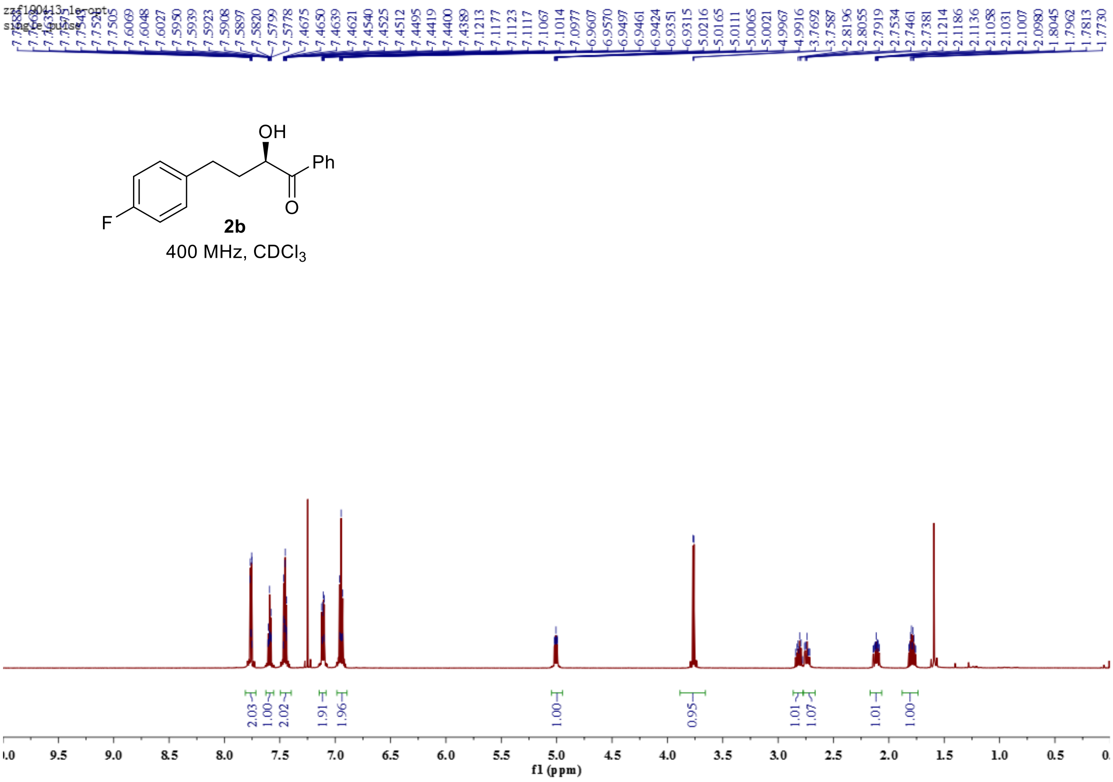
zzf190413-1e-opt
single pulse
7.5505 7.6069 7.6048 7.6027 7.5950 7.5939 7.5923 7.5908 7.5897 7.5820 7.5799 7.5778 7.4675 7.4650 7.4639 7.4621 7.4540 7.4525 7.4512 7.4495 7.4419 7.4400 7.4389 7.1213 7.1177 7.1123 7.1117 7.1067 7.1014 7.0977 6.9607 6.9570 6.9497 6.9461 6.9424 6.9351 6.9315 5.0216 5.0165 5.0111 5.0065 5.0021 4.9967 4.9916 3.7692 3.7587 2.8196 2.8055 2.7919 2.7534 2.7461 2.7381 2.1214 2.1186 2.1136 2.1058 2.1031 2.1007 2.0980 1.8045 1.7962 1.7813 1.7730
OH
Ph
O
F
2b
400 MHz, $CDCl_3$
2.03 1.00 2.02 1.91 1.96 1.00 0.95 1.01 1.07 1.01 1.00
f1 (ppm)

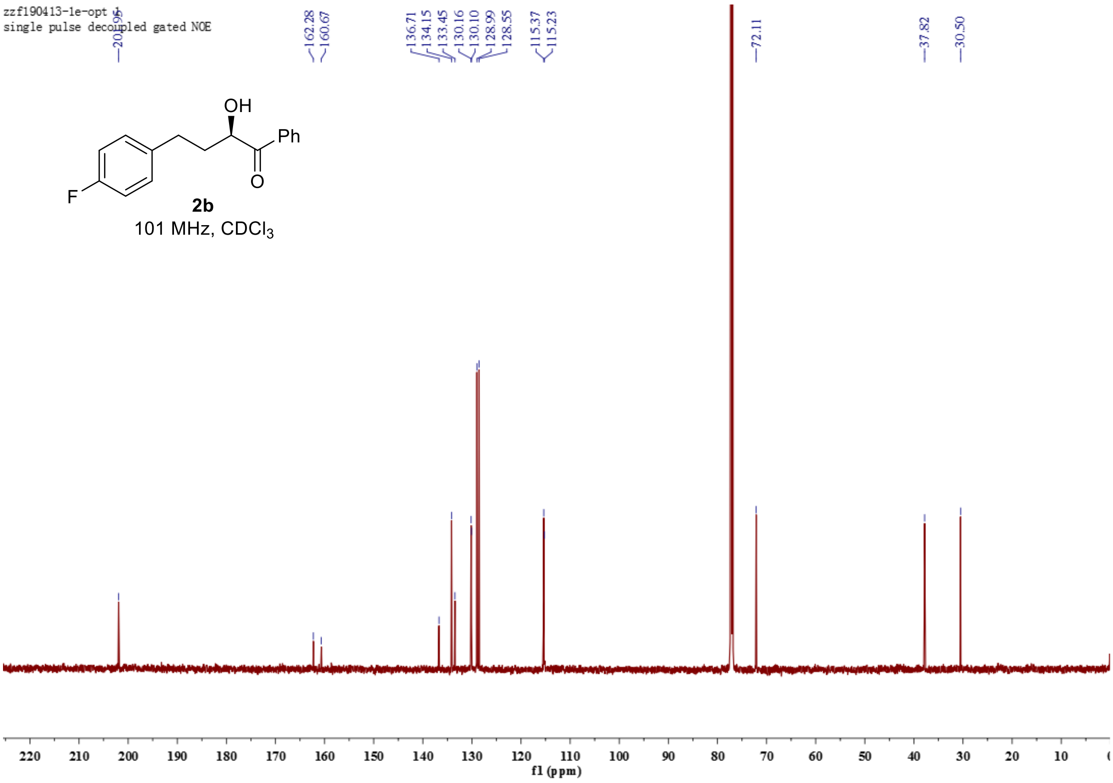
zzf190413-1e-opt
single pulse decoupled gated NOE
162.28 160.67 136.71 134.15 133.45 130.16 130.10 128.99 128.55 115.37 115.23 72.11 37.82 30.50
OH
Ph
O
F
2b
101 MHz, $CDCl_3$
f1 (ppm)

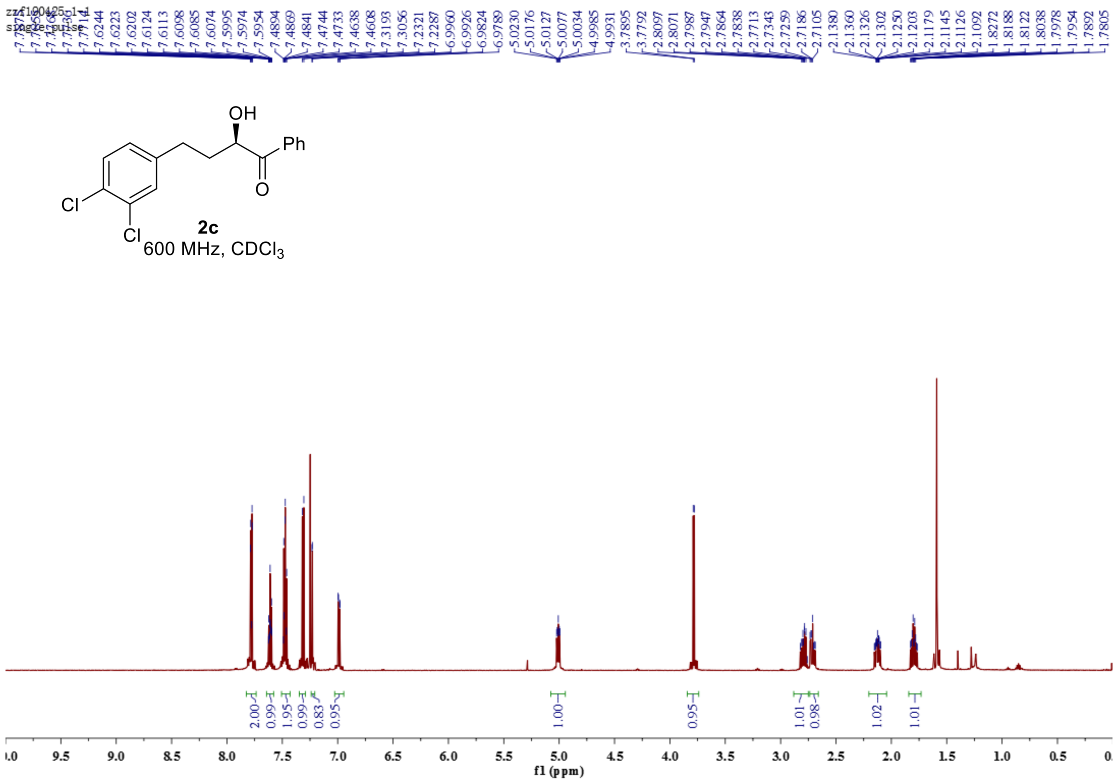
zzf190425-1-1
single pulse
7.6244
7.6223
7.6202
7.6124
7.6113
7.6098
7.6085
7.6074
7.5995
7.5974
7.5954
7.4894
7.4869
7.4841
7.4744
7.4733
7.4638
7.4608
7.3193
7.3056
7.2321
7.2287
6.9960
6.9926
6.9824
6.9789
5.0230
5.0176
5.0127
5.0077
5.0034
4.9985
4.9931
3.7895
3.7792
2.8097
2.8071
2.7987
2.7947
2.7864
2.7838
2.7713
2.7343
2.7259
2.7186
2.7105
2.1380
2.1360
2.1326
2.1302
2.1250
2.1203
2.1179
2.1145
2.1126
2.1092
1.8272
1.8188
1.8122
1.8038
1.7978
1.7954
1.7892
1.7805
OH
Ph
O
Cl
Cl
2c
600 MHz, CDCl3
2.00
0.99
1.95
0.99
0.83
0.95
1.00
0.95
1.01
0.98
1.02
1.01
f1 (ppm)

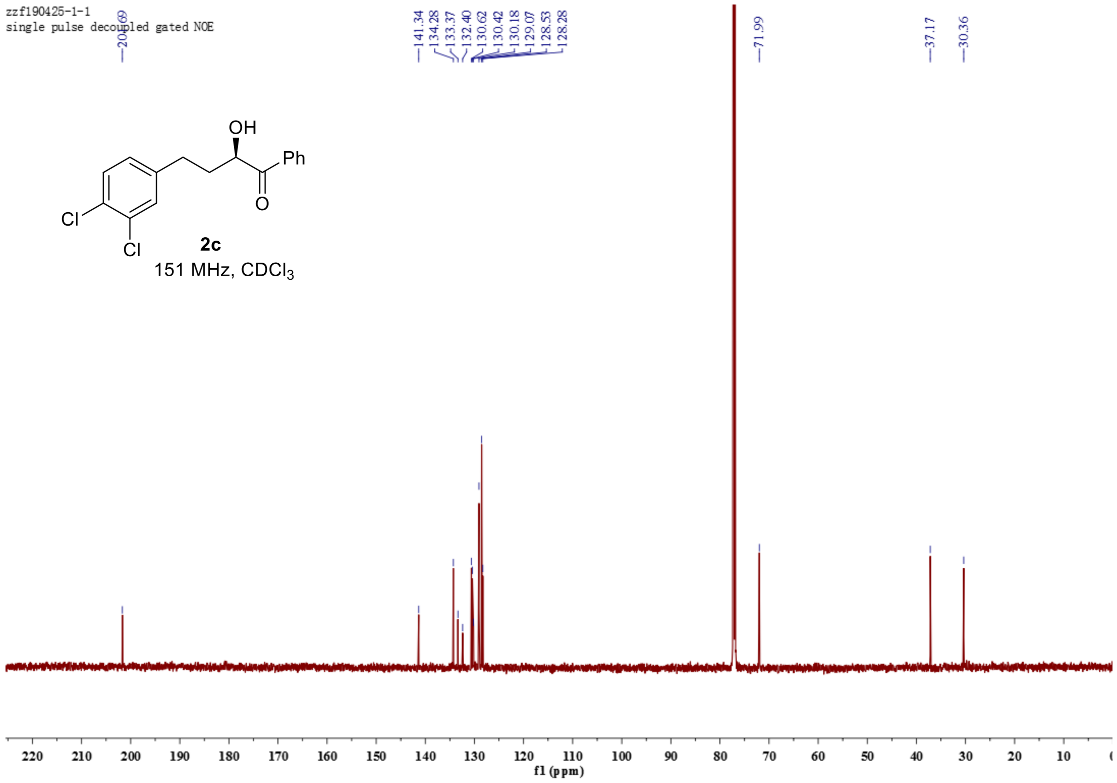
zzf190425-1-1
single pulse decoupled gated NOE
201.69
141.34
134.28
133.37
132.40
130.62
130.42
130.18
129.07
128.53
128.28
71.99
37.17
30.36
OH
Ph
O
Cl
Cl
2c
151 MHz, CDCl3
f1 (ppm)

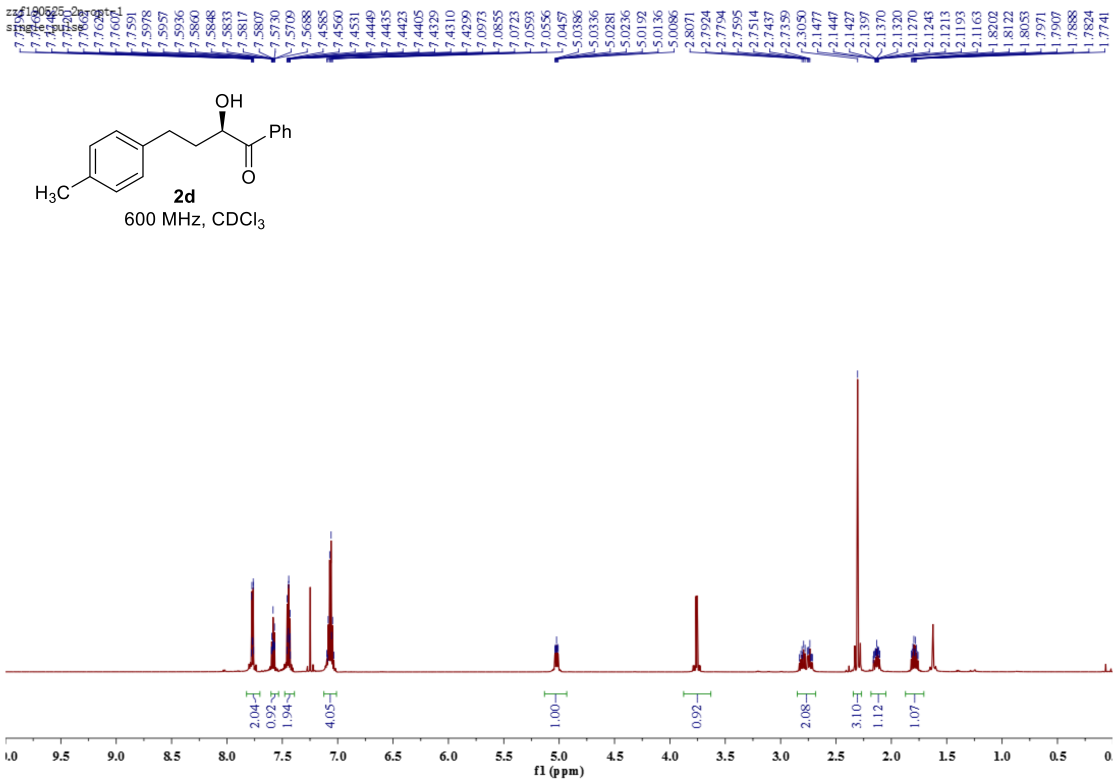
OH
Ph
O
H3C
2d
600 MHz, CDCl3
2.04
0.92
1.94
4.05
1.00
0.92
2.08
3.10
1.12
1.07
f1 (ppm)

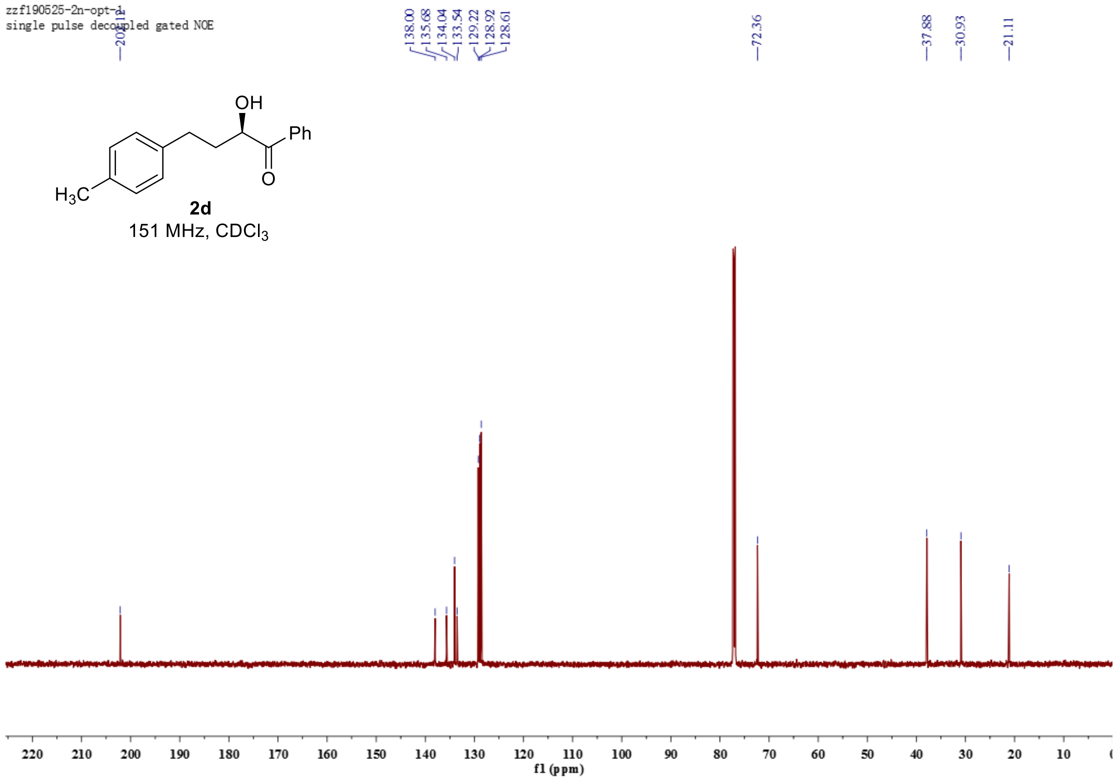
zzf190525-2n-opt-1
single pulse decoupled gated NOE
138.00
135.68
134.04
133.54
129.22
128.92
128.61
72.36
37.88
30.93
21.11
OH
Ph
O
H3C
2d
151 MHz, CDCl3
f1 (ppm)

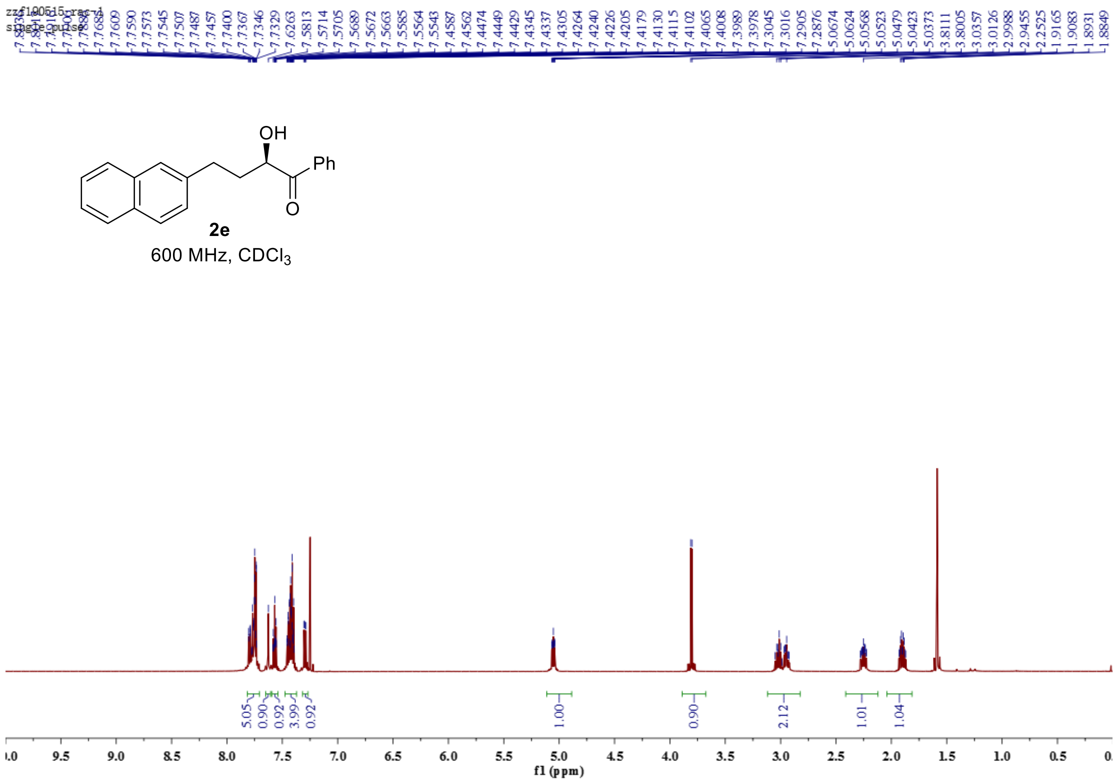
zzf190515-rac-1
single pulse
7.7609 7.7590 7.7573 7.7545 7.7507 7.7487 7.7457 7.7400 7.7367 7.7346 7.7329 7.6263 7.5813 7.5714 7.5705 7.5689 7.5672 7.5663 7.5585 7.5564 7.5543 7.4587 7.4562 7.4474 7.4449 7.4429 7.4345 7.4337 7.4305 7.4264 7.4240 7.4226 7.4205 7.4179 7.4130 7.4115 7.4102 7.4065 7.4008 7.3989 7.3978 7.3045 7.3016 7.2905 7.2876 5.0674 5.0624 5.0568 5.0523 5.0479 5.0423 5.0373 3.8111 3.8005 3.0357 3.0126 2.9988 2.9455 2.2525 1.9165 1.9083 1.8931 1.8849
OH
Ph
O
2e
600 MHz, CDCl3
5.05 0.90 0.92 3.99 0.92 1.00 0.90 2.12 1.01 1.04
10.0 9.5 9.0 8.5 8.0 7.5 7.0 6.5 6.0 5.5 5.0 4.5 4.0 3.5 3.0 2.5 2.0 1.5 1.0 0.5 0
f1 (ppm)

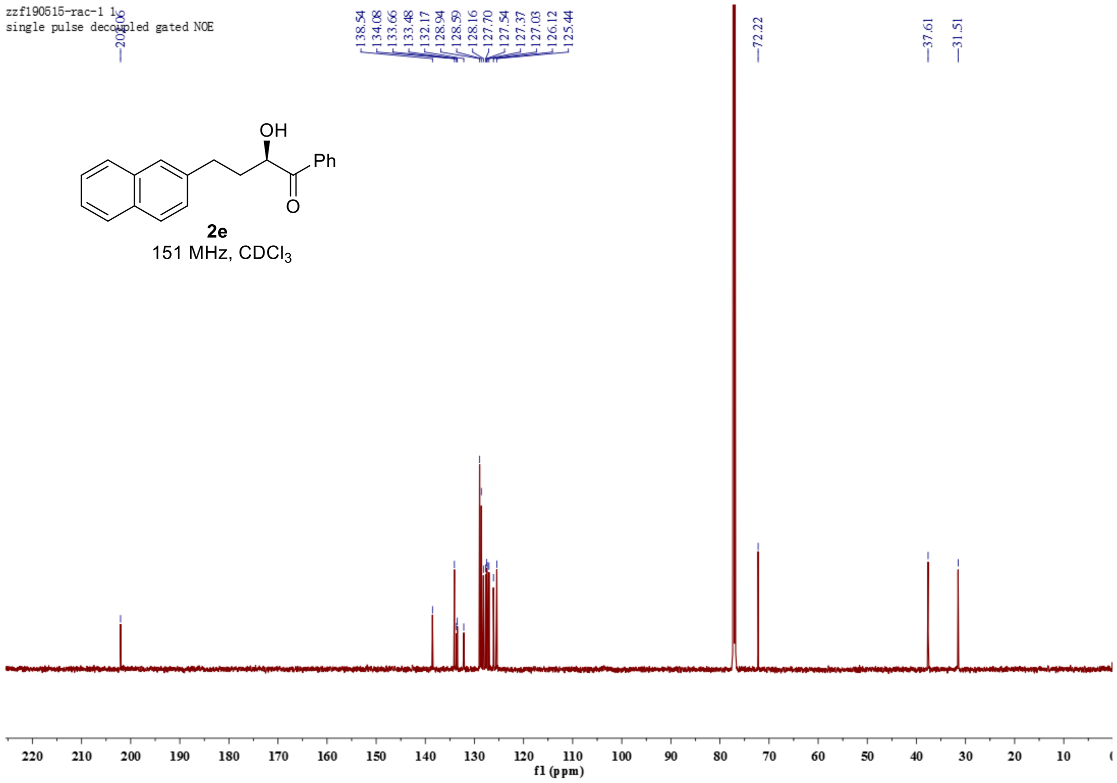
zzf190515-rac-1 1
single pulse decoupled gated NOE
202.06
138.54 134.08 133.66 133.48 132.17 128.94 128.59 128.16 127.70 127.54 127.37 127.03 126.12 125.44
72.22
37.61
31.51
OH
Ph
O
2e
151 MHz, CDCl3
220 210 200 190 180 170 160 150 140 130 120 110 100 90 80 70 60 50 40 30 20 10 0
f1 (ppm)

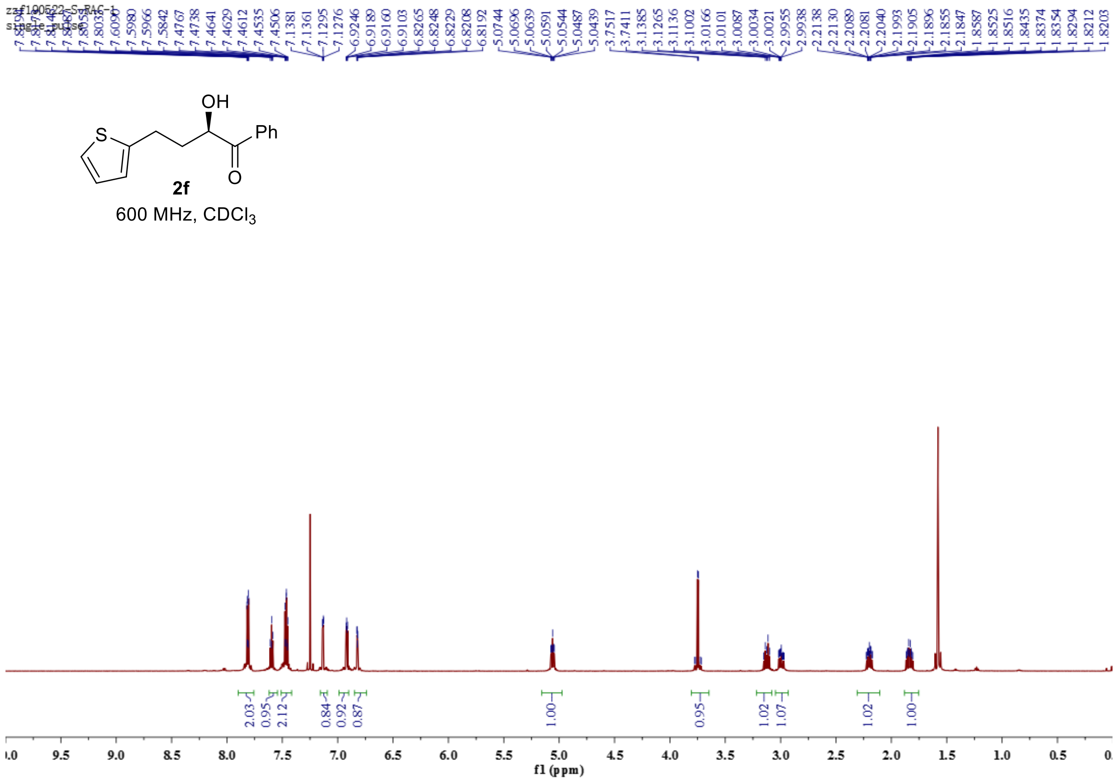

zzf190522-S-RAC-1
single pulse
OH
Ph
S
O
2f
600 MHz, CDCl3
f1 (ppm)

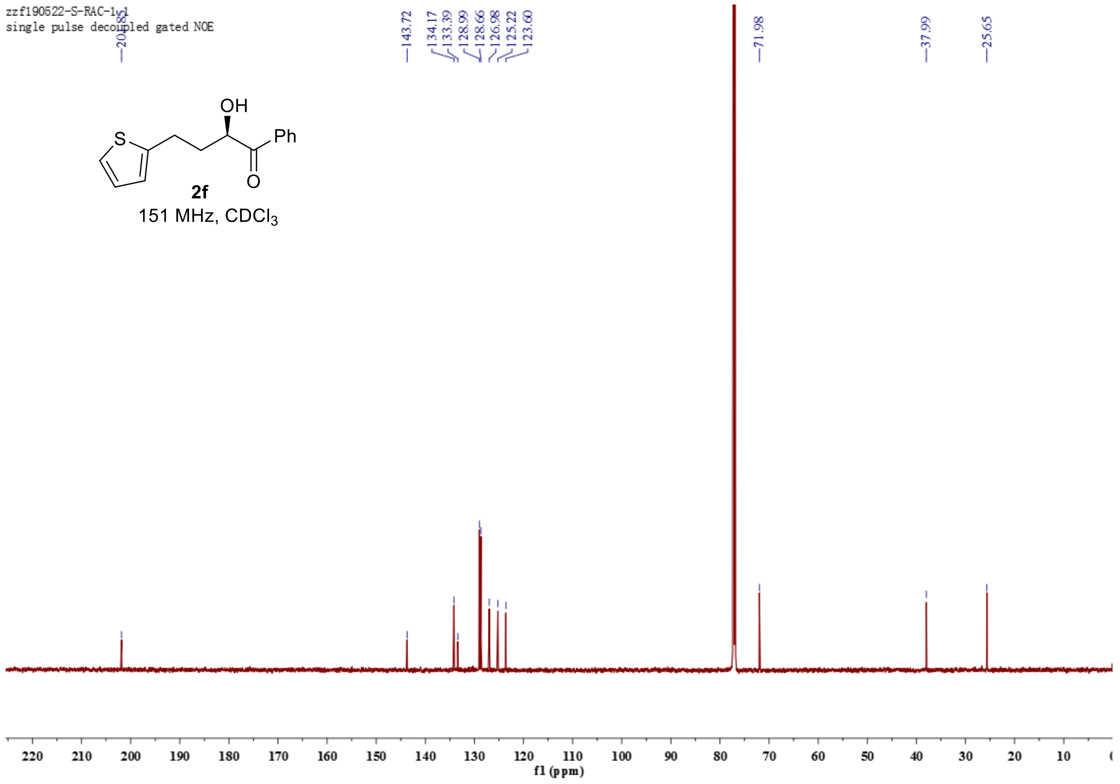

zzf190522-S-RAC-1.1
single pulse decoupled gated NOE
143.72
134.17
133.39
128.99
128.66
126.98
125.22
123.60
71.98
37.99
25.65
OH
Ph
S
O
2f
151 MHz, CDCl3
f1 (ppm)

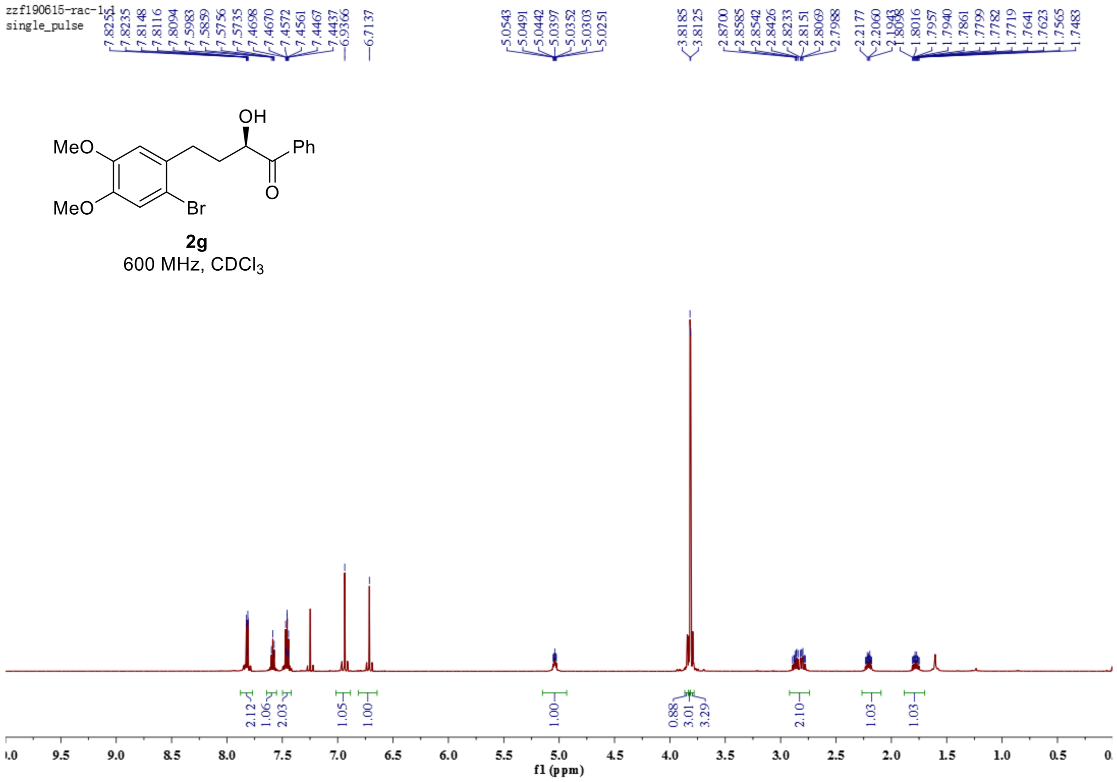
zzf190615-rac-1.1
single_pulse
OH
MeO
Ph
MeO
Br
O
2g
600 MHz, CDCl3
f1 (ppm)

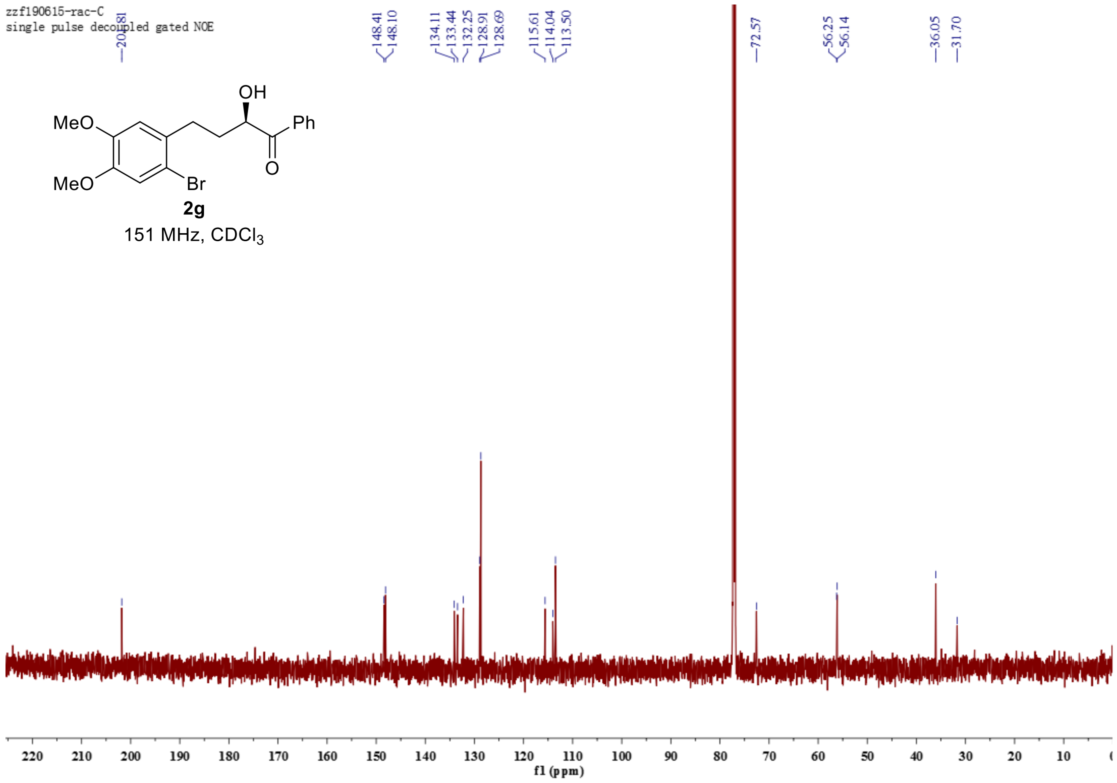
zzf190615-rac-C
single pulse decoupled gated NOE
OH
MeO
Ph
MeO
Br
O
2g
151 MHz, CDCl3
f1 (ppm)

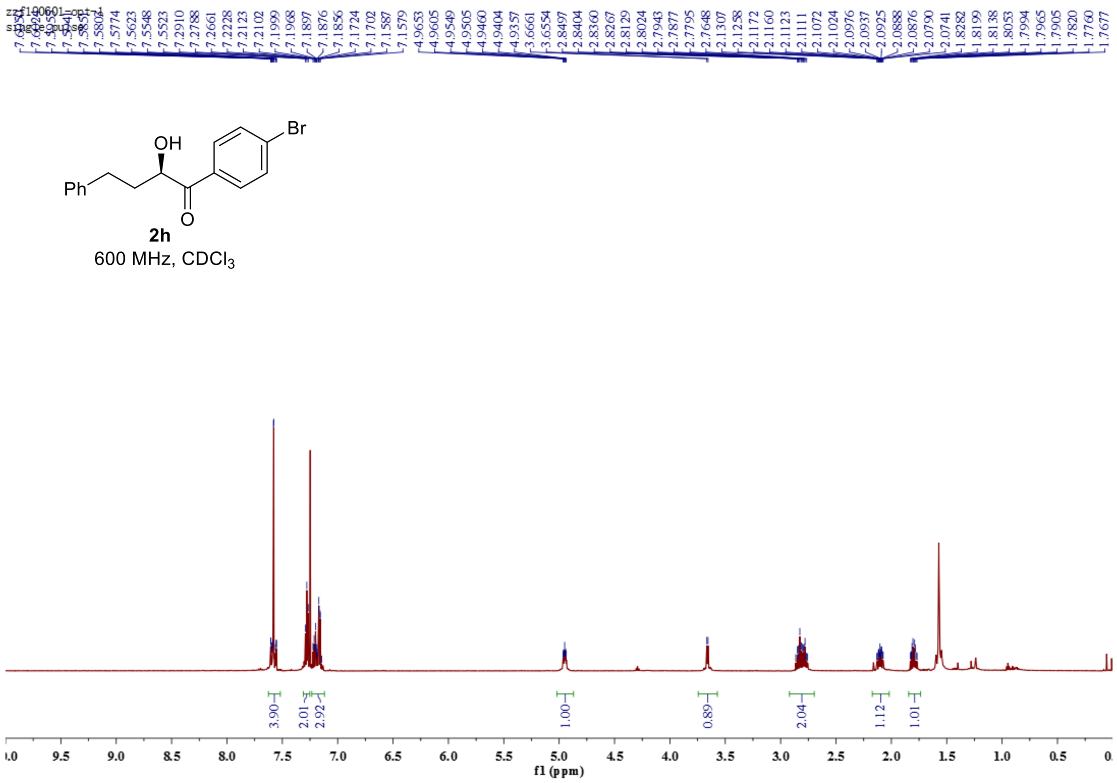

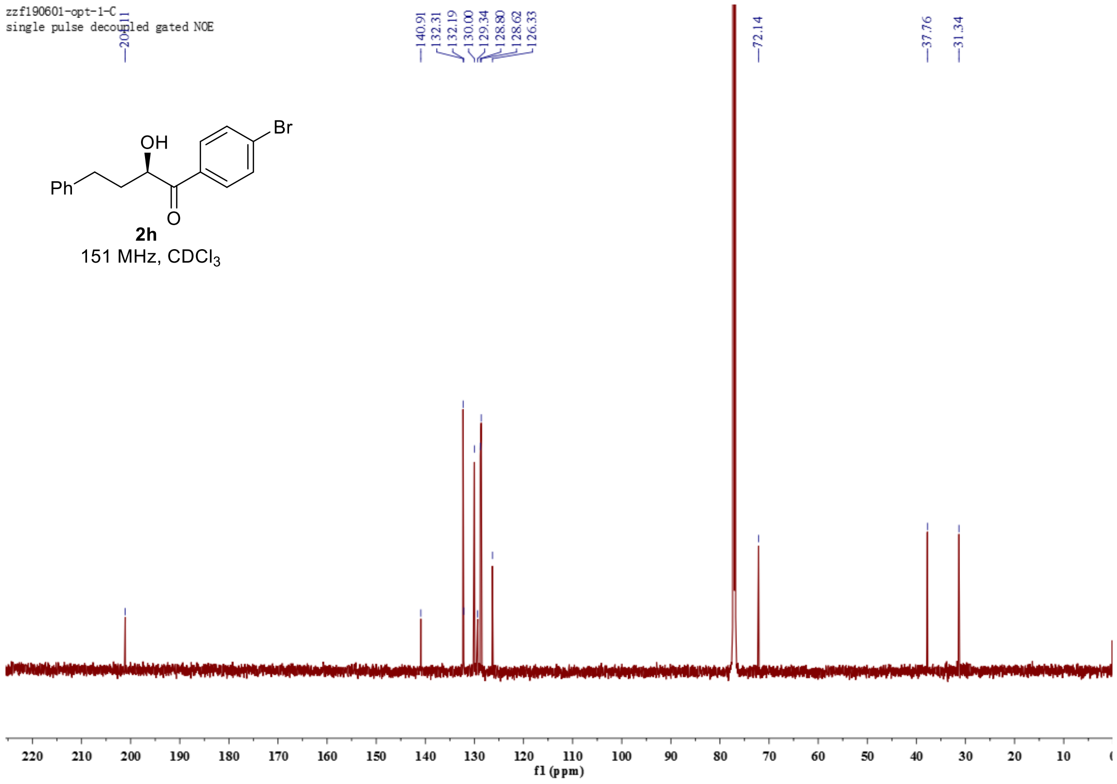

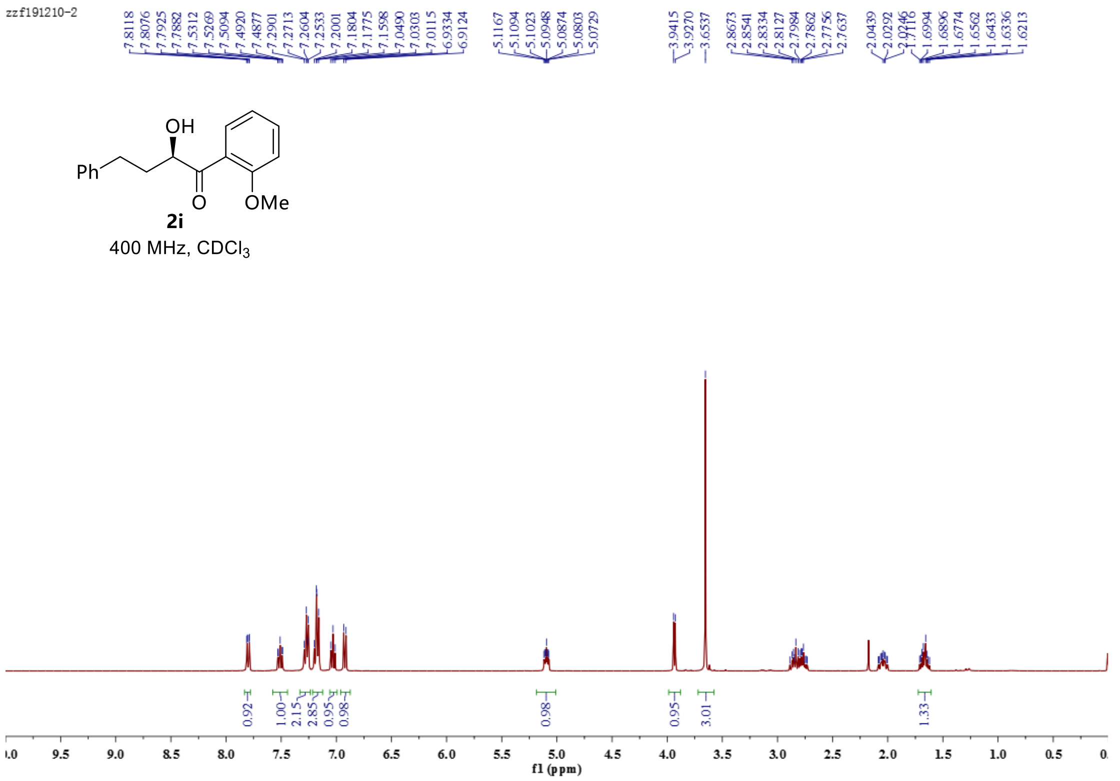
zzf191210-2
7.8118 7.8076 7.7925 7.7882 7.5312 7.5269 7.5094 7.4920 7.4877 7.2901 7.2713 7.2604 7.2533 7.2001 7.1804 7.1775 7.1598 7.0490 7.0303 7.0115 6.9334 6.9124
5.1167 5.1094 5.1023 5.0948 5.0874 5.0803 5.0729
3.9415 3.9270 3.6537
2.8673 2.8541 2.8334 2.8127 2.7984 2.7862 2.7756 2.7637
2.0439 2.0292 1.6994 1.6896 1.6774 1.6562 1.6433 1.6336 1.6213
OH
Ph
O
OMe
2i
400 MHz, CDCl3
0.92 1.00 2.15 2.85 0.95 0.98 0.98 0.95 3.01 1.33
f1 (ppm)

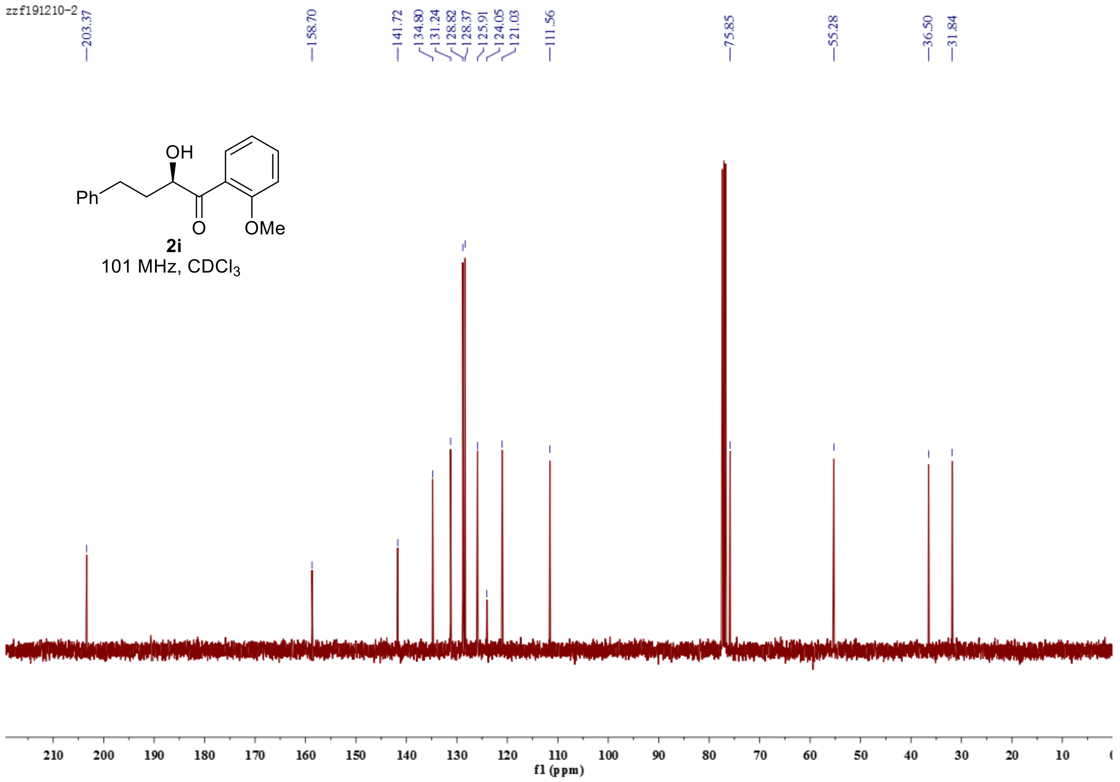
zzf191210-2
203.37 158.70 141.72 134.80 131.24 128.82 128.37 125.91 124.05 121.03 111.56 75.85 55.28 36.50 31.84
OH
Ph
O
OMe
2i
101 MHz, CDCl3
f1 (ppm)

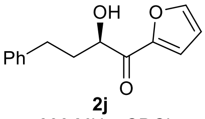

2j

600 MHz, $CDCl_3$

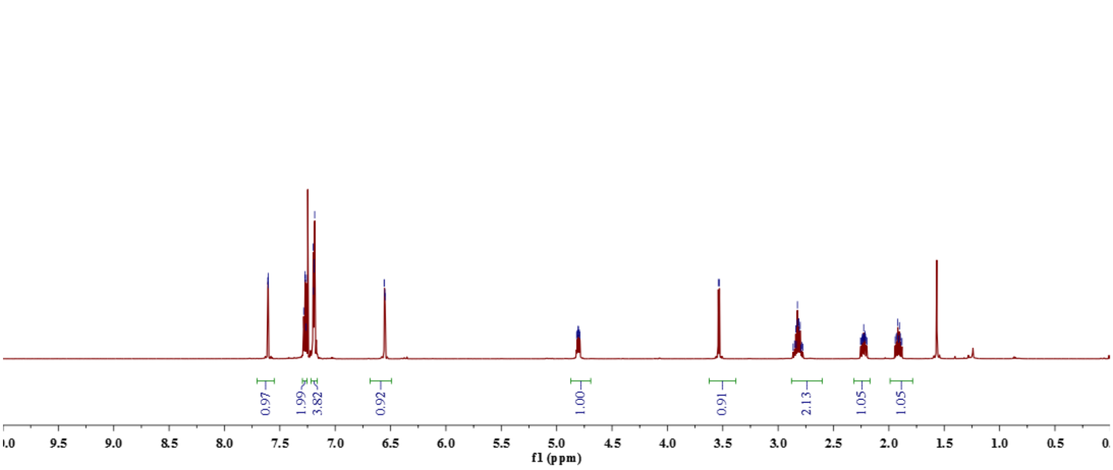

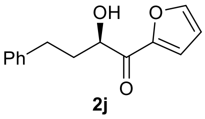

2j

151 MHz, $CDCl_3$

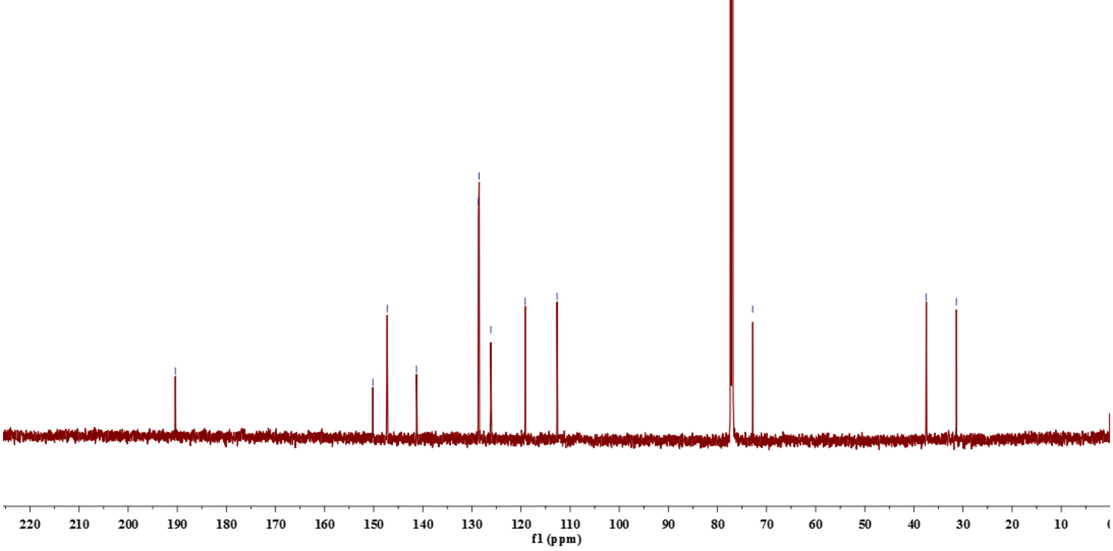

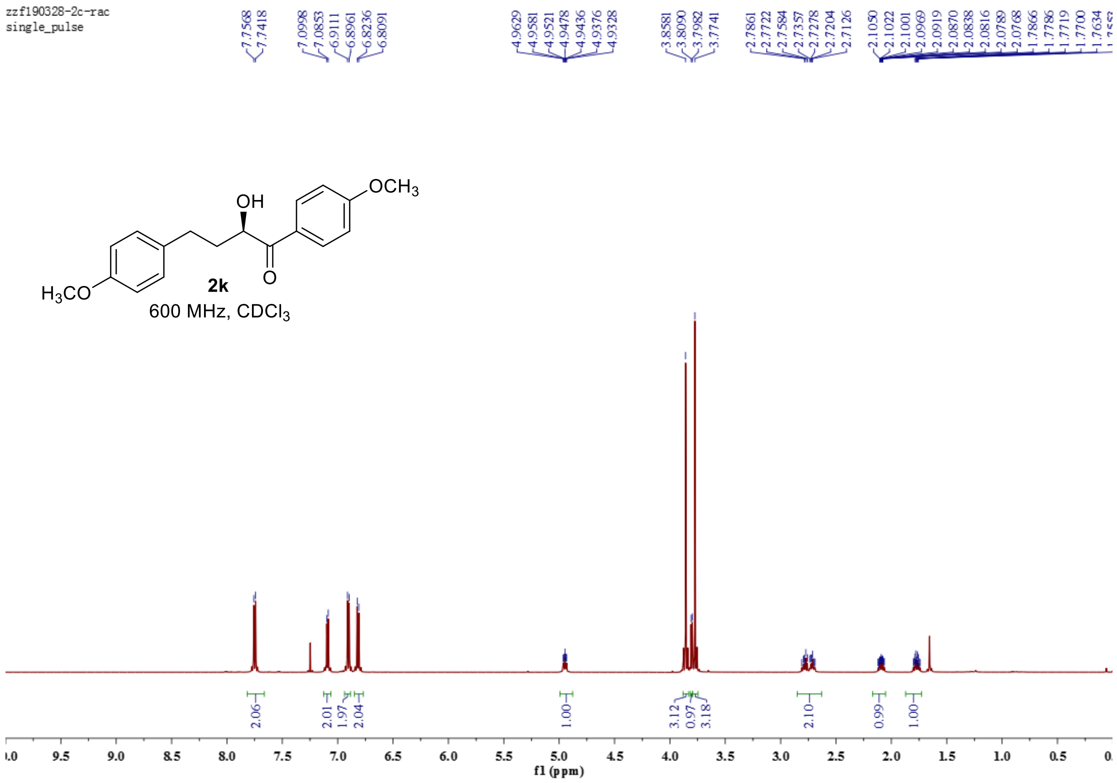
zzf190328-2c-rac
single_pulse
7.7568
7.7418
7.0998
7.0853
6.9111
6.8961
6.8236
6.8091
4.9629
4.9581
4.9521
4.9478
4.9436
4.9376
4.9328
3.8581
3.8090
3.7982
3.7741
2.7861
2.7722
2.7584
2.7357
2.7278
2.7204
2.7126
2.1050
2.1022
2.1001
2.0969
2.0919
2.0870
2.0838
2.0816
2.0789
2.0768
1.7866
1.7786
1.7719
1.7700
1.7634
OH
OCH3
H3CO
O
2k
600 MHz, CDCl3
2.06
2.01
1.97
2.04
1.00
3.12
0.97
3.18
2.10
0.99
1.00
f1 (ppm)

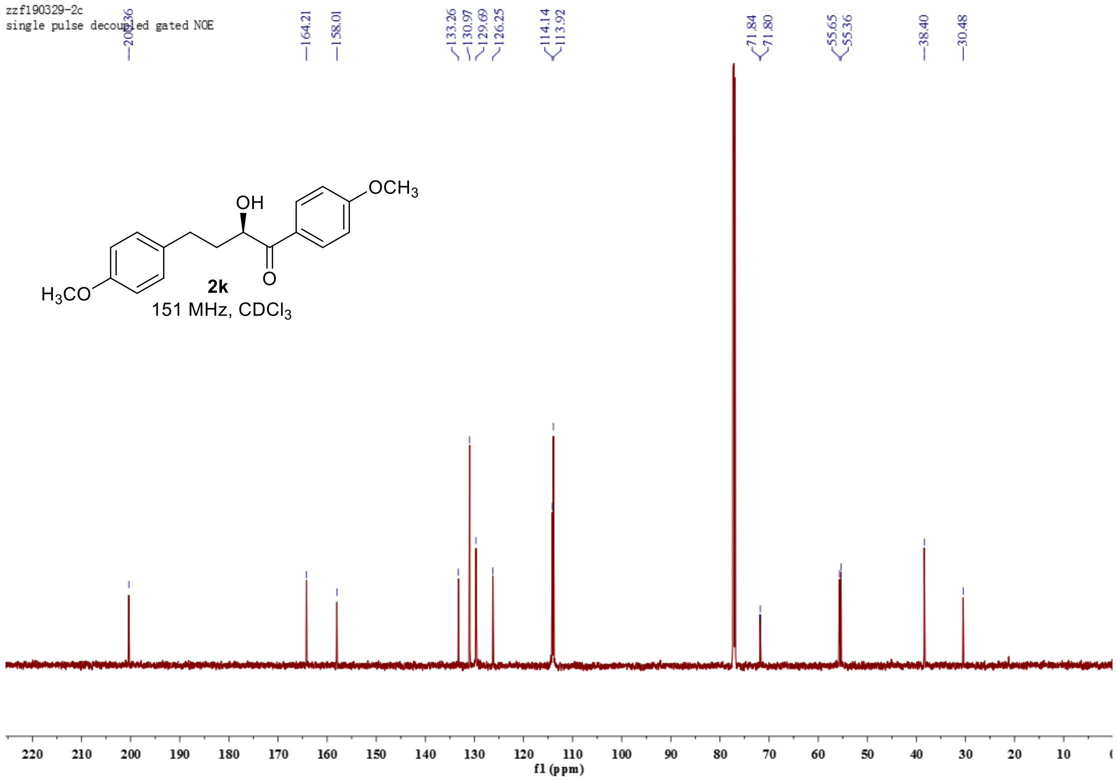
zzf190329-2c
single pulse decoupled gated NOE
164.21
158.01
133.26
130.97
129.69
126.25
114.14
113.92
71.84
71.80
55.65
55.36
38.40
30.48
OH
OCH3
H3CO
O
2k
151 MHz, CDCl3
f1 (ppm)

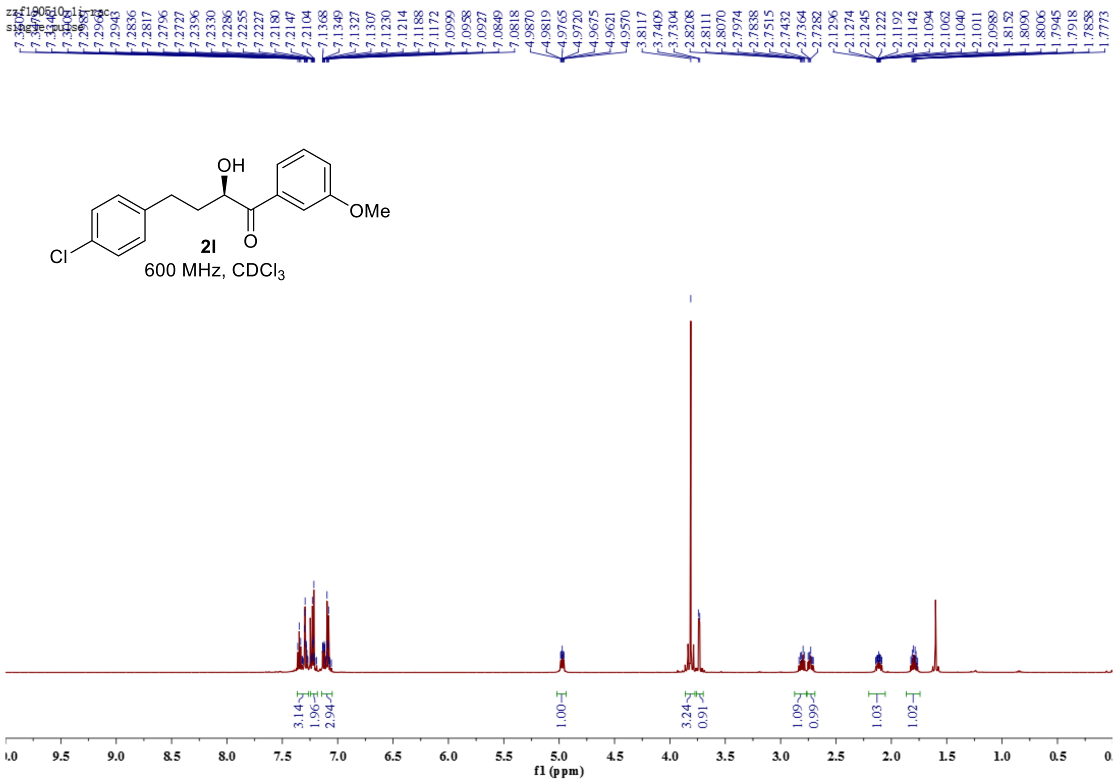
zzf190510-1i-rac
single pulse
7.2943
7.2836
7.2817
7.2796
7.2727
7.2396
7.2330
7.2286
7.2255
7.2227
7.2180
7.2147
7.2104
7.1368
7.1349
7.1327
7.1307
7.1230
7.1214
7.1188
7.1172
7.0999
7.0958
7.0927
7.0849
7.0818
4.9870
4.9819
4.9765
4.9720
4.9675
4.9621
4.9570
3.8117
3.7409
3.7304
2.8208
2.8111
2.8070
2.7974
2.7838
2.7515
2.7432
2.7364
2.7282
2.1296
2.1274
2.1245
2.1222
2.1192
2.1142
2.1094
2.1062
2.1040
2.1011
2.0989
1.8152
1.8090
1.8006
1.7945
1.7918
1.7858
1.7773
OH
OMe
Cl
O
2l
600 MHz, $CDCl_3$
3.14
1.96
2.94
1.00
3.24
0.91
1.09
0.99
1.03
1.02
f1 (ppm)

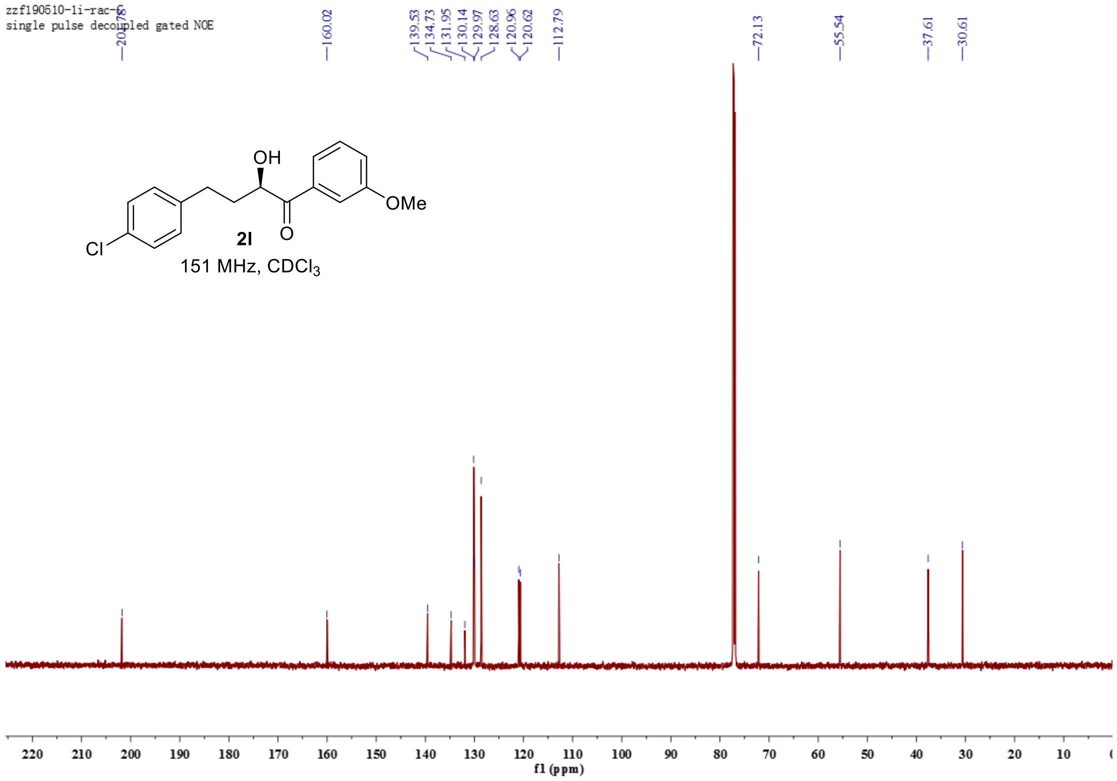
zzf190510-1i-rac-C
single pulse decoupled gated NOE
201.78
160.02
139.53
134.73
131.95
130.14
129.97
128.63
120.96
120.62
112.79
72.13
55.54
37.61
30.61
OH
OMe
Cl
O
2l
151 MHz, $CDCl_3$
f1 (ppm)

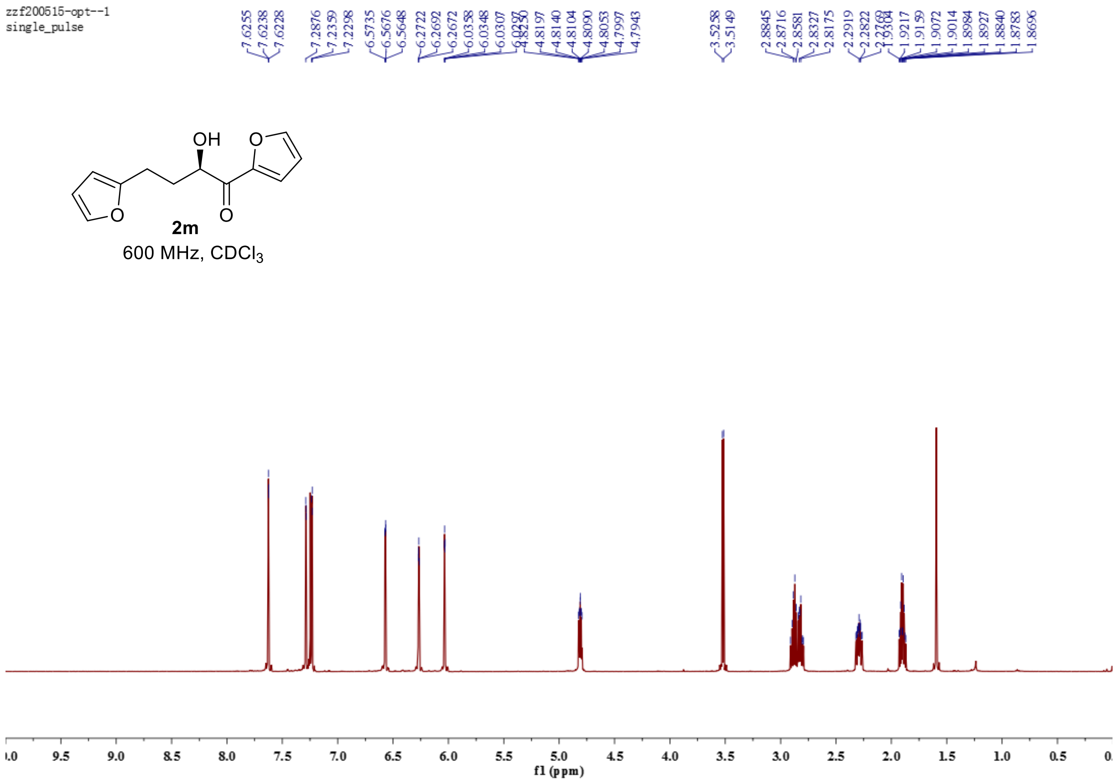

zzf200515-opt--1
single_pulse
OH
O
O
O
2m
600 MHz, CDCl3
f1 (ppm)

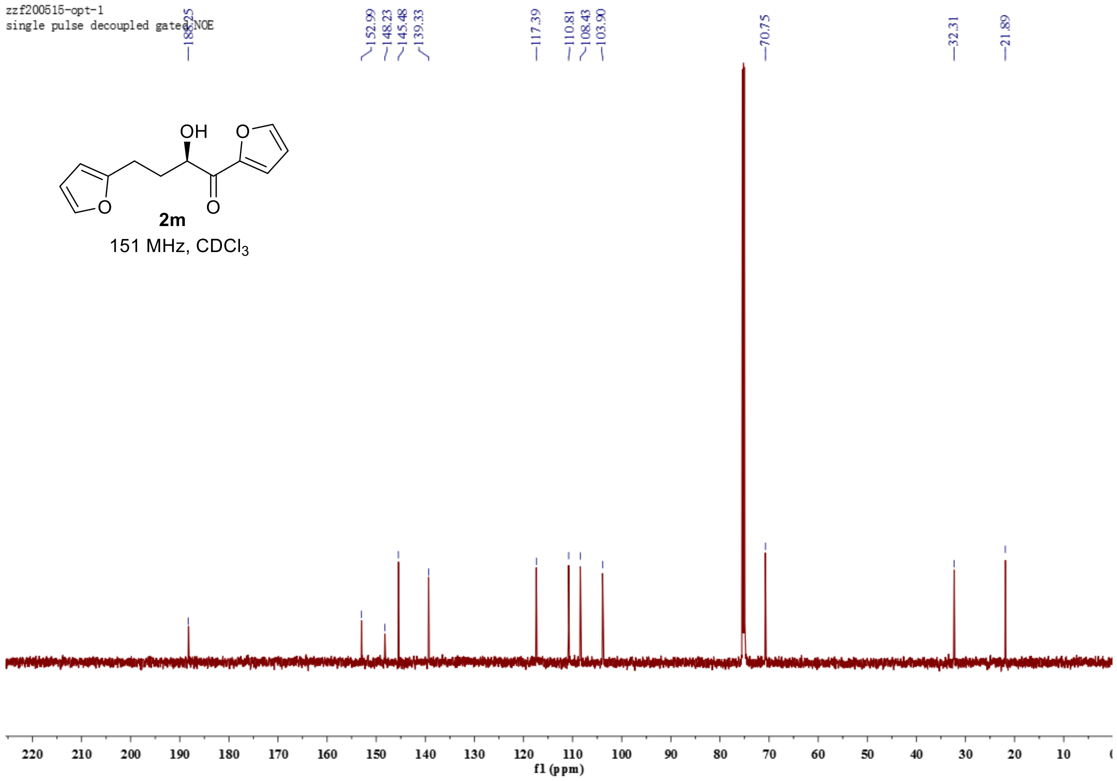

zzf200515-opt-1
single pulse decoupled gated NOE
OH
O
O
O
2m
151 MHz, CDCl3
f1 (ppm)

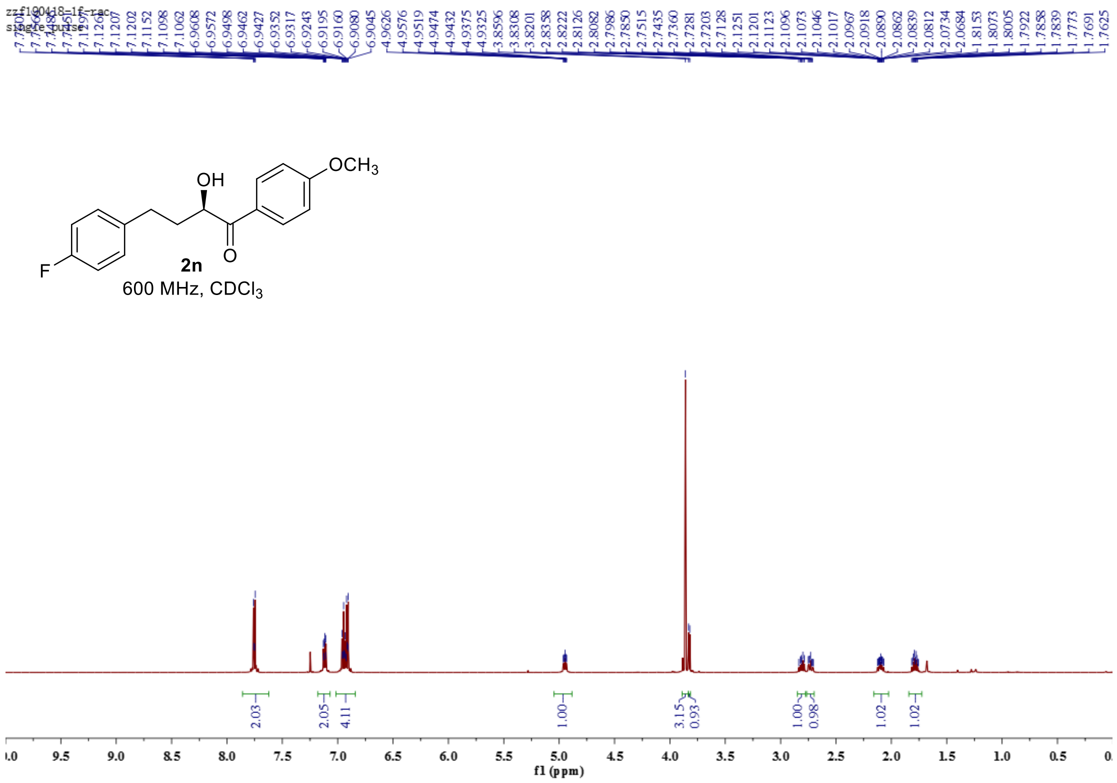
zzf190418-1f-rac
single pulse
OCH3
OH
F
O
2n
600 MHz, CDCl3
f1 (ppm)

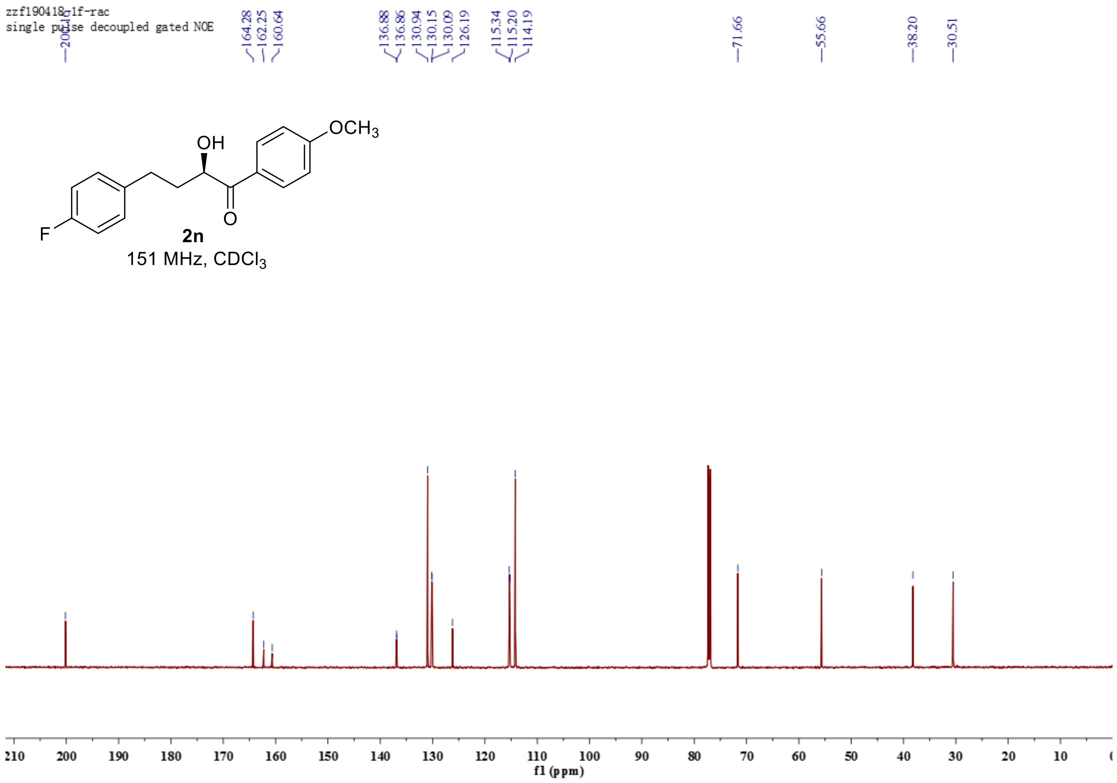
zzf190418-1f-rac
single pulse decoupled gated NOE
OCH3
OH
F
O
2n
151 MHz, CDCl3
f1 (ppm)

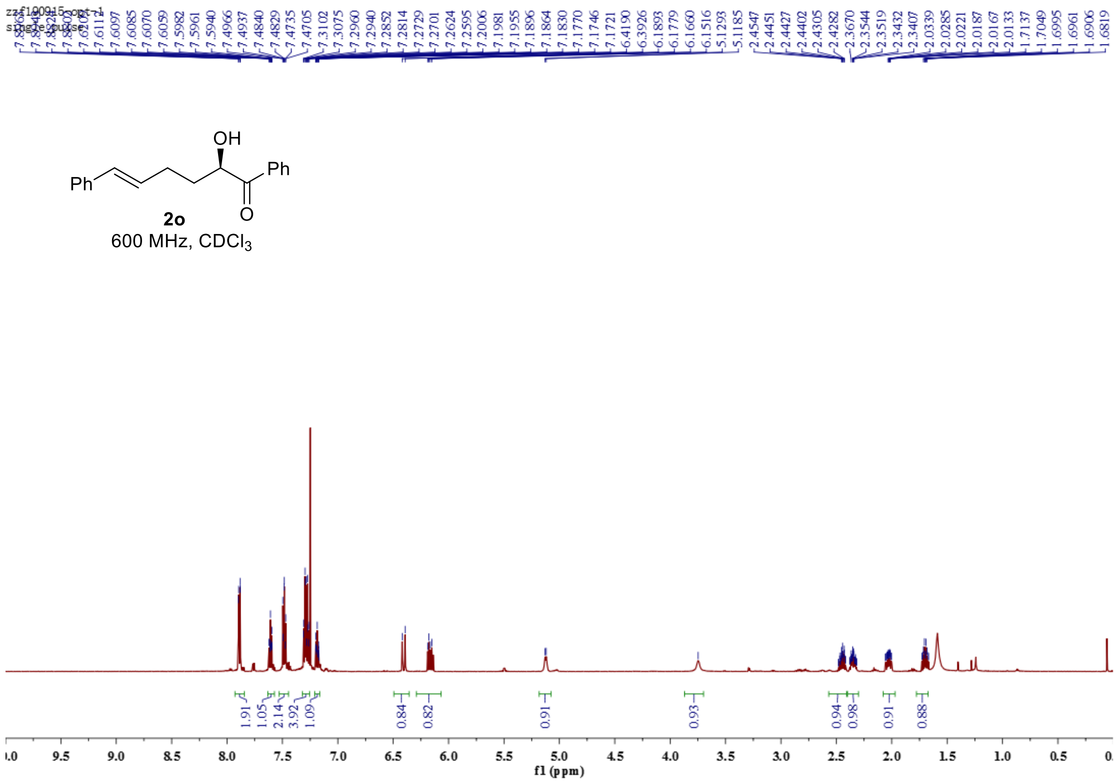

zzf190915-opt-1
single pulse
7.6111
7.6097
7.6085
7.6070
7.6059
7.5982
7.5961
7.5940
7.4966
7.4937
7.4840
7.4829
7.4735
7.4705
7.3102
7.3075
7.2960
7.2940
7.2852
7.2814
7.2729
7.2701
7.2624
7.2595
7.2006
7.1981
7.1955
7.1896
7.1864
7.1830
7.1770
7.1746
7.1721
6.4190
6.3926
6.1893
6.1779
6.1660
6.1516
5.1293
5.1185
2.4547
2.4451
2.4427
2.4402
2.4305
2.4282
2.3670
2.3544
2.3519
2.3432
2.3407
2.0339
2.0285
2.0221
2.0187
2.0167
2.0133
1.7137
1.7049
1.6995
1.6961
1.6906
1.6819
OH
Ph
Ph
O
2o
600 MHz, CDCl3
1.91
1.05
2.14
3.92
1.09
0.84
0.82
0.91
0.93
0.94
0.98
0.91
0.88
f1 (ppm)

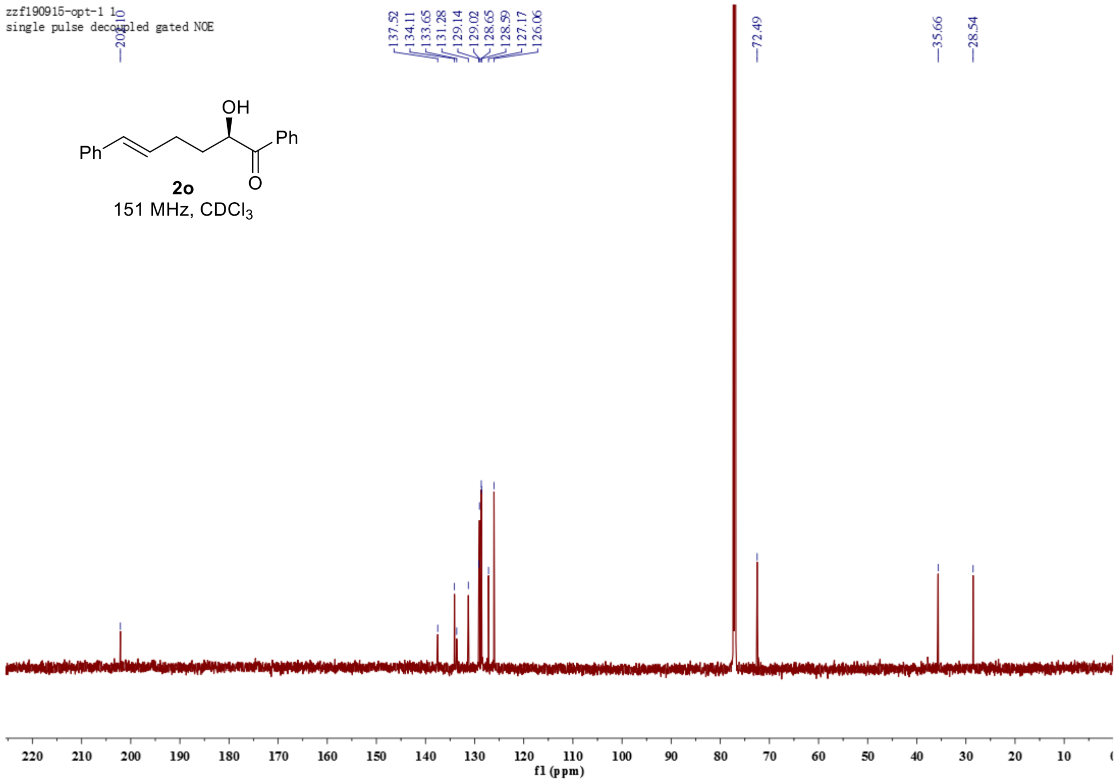

zzf190915-opt-1 1
single pulse decoupled gated NOE
202.10
137.52
134.11
133.65
131.28
129.14
129.02
128.65
128.59
127.17
126.06
72.49
35.66
28.54
OH
Ph
Ph
O
2o
151 MHz, CDCl3
f1 (ppm)

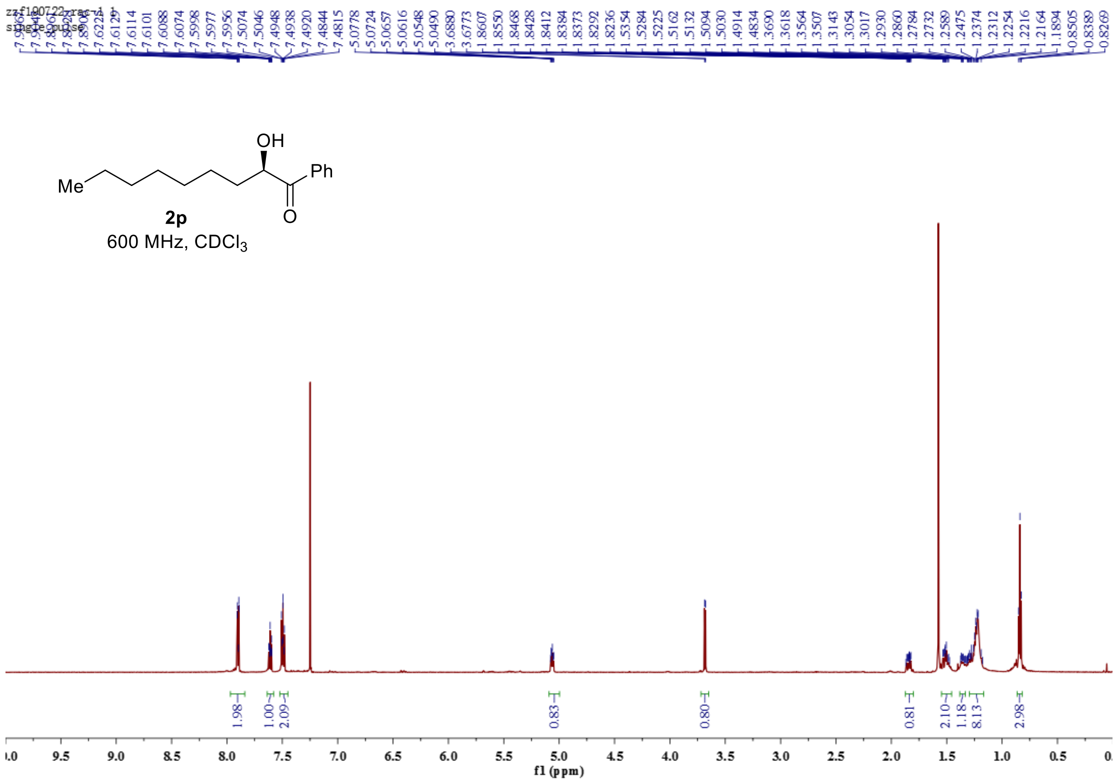
zzf190722-rac-1 1
single pulse
OH
Me
Ph
O
2p
600 MHz, CDCl3
f1 (ppm)

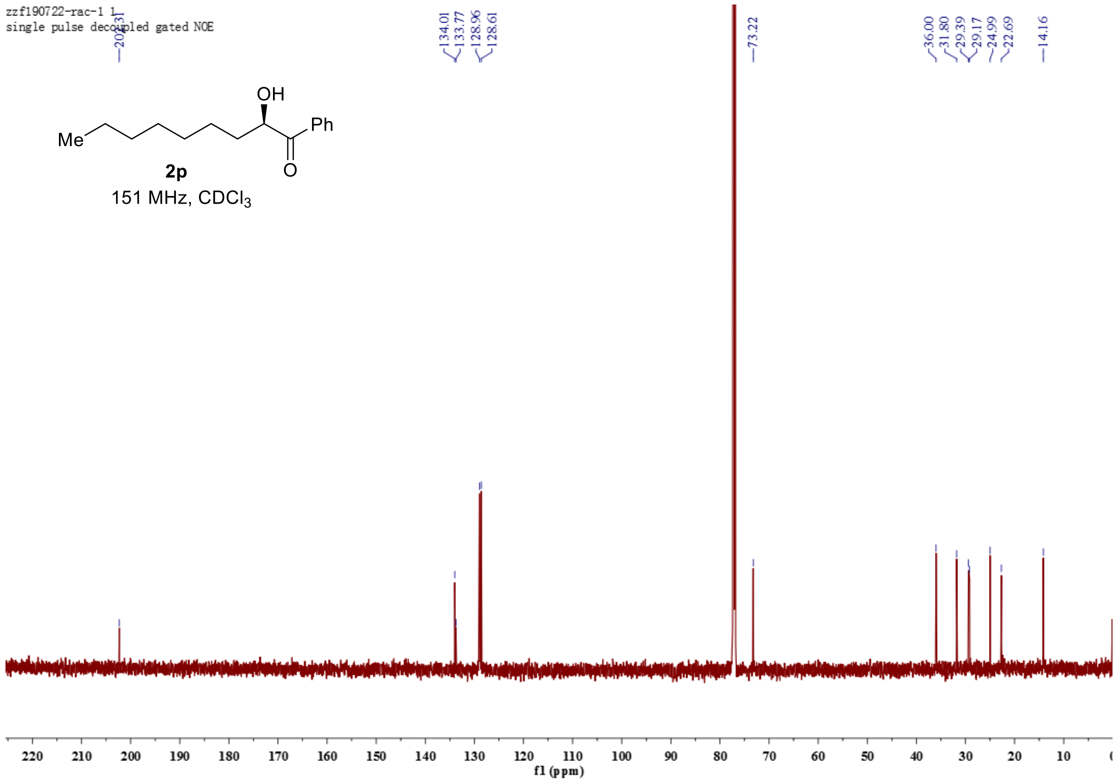
zzf190722-rac-1 1
single pulse decoupled gated NOE
134.01
133.77
128.96
128.61
73.22
36.00
31.80
29.39
29.17
24.99
22.69
14.16
OH
Me
Ph
O
2p
151 MHz, CDCl3
f1 (ppm)

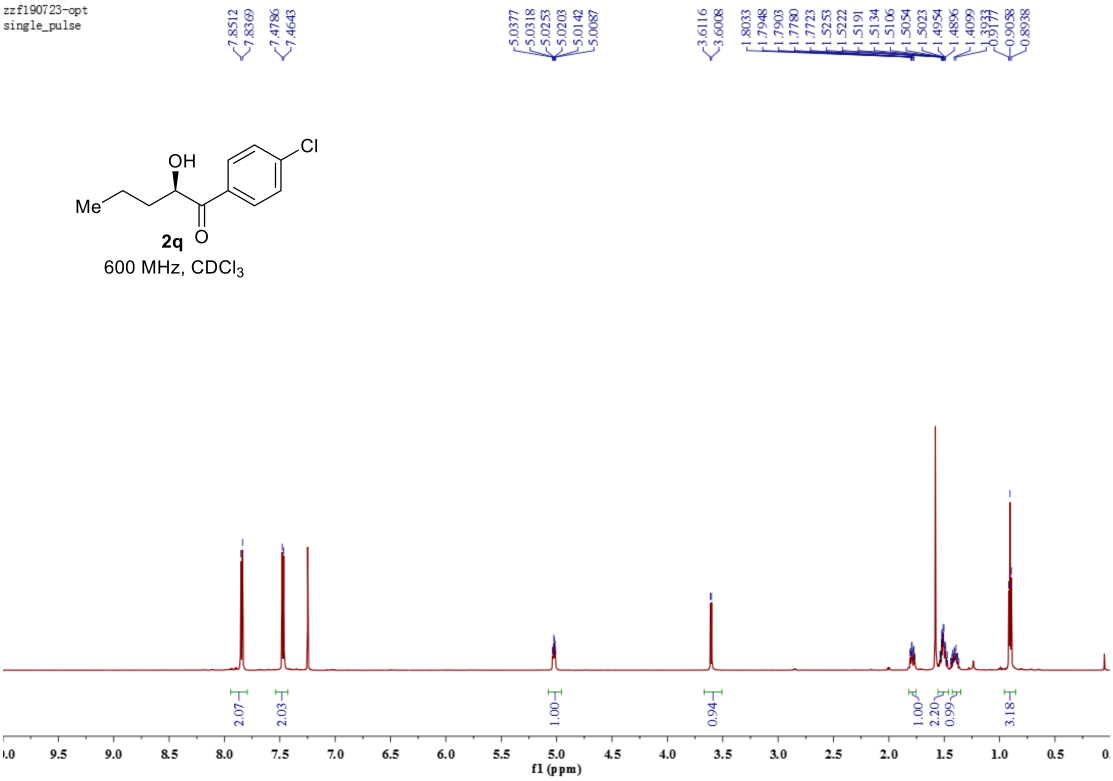
zzf190723-opt
single_pulse
7.8512
7.8369
7.4786
7.4643
5.0377
5.0318
5.0253
5.0203
5.0142
5.0087
3.6116
3.6008
1.8033
1.7948
1.7903
1.7780
1.7723
1.5253
1.5222
1.5191
1.5134
1.5106
1.5054
1.5023
1.4954
1.4896
1.4099
0.9058
0.8938
OH
Cl
Me
O
2q
600 MHz, CDCl3
2.07
2.03
1.00
0.94
1.00
2.20
0.99
3.18
f1 (ppm)

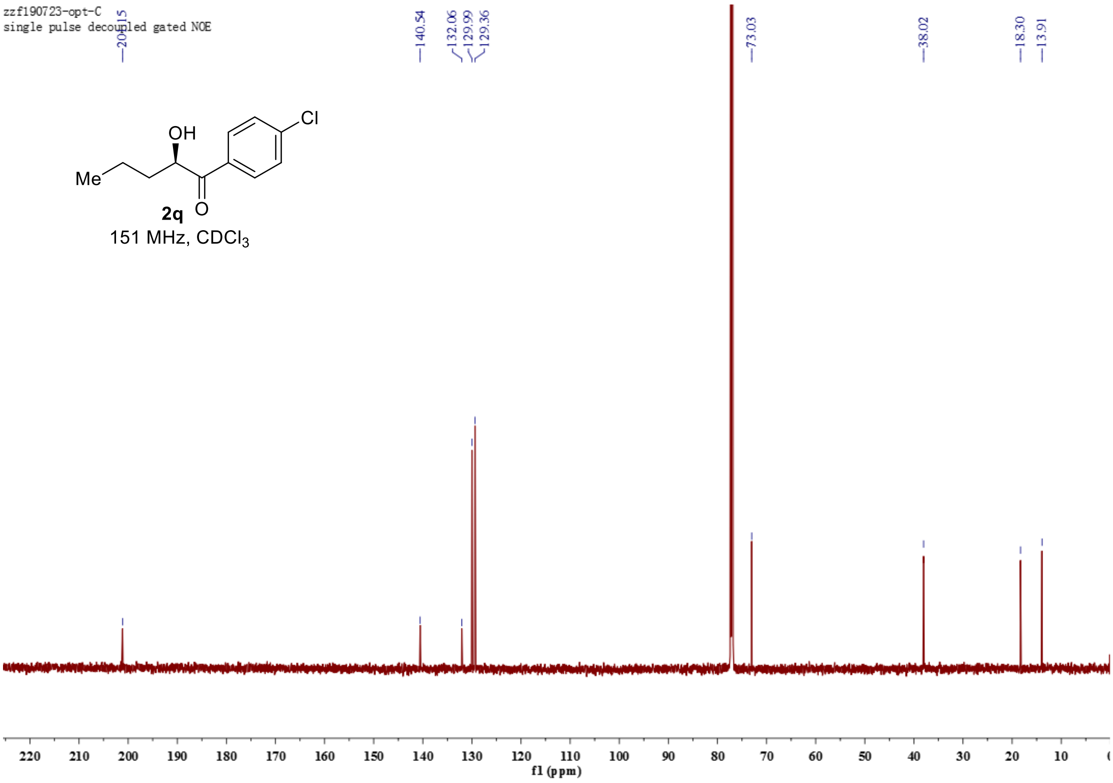
zzf190723-opt-C
single pulse decoupled gated NOE
140.54
132.06
129.99
129.36
73.03
38.02
18.30
13.91
OH
Cl
Me
O
2q
151 MHz, CDCl3
f1 (ppm)

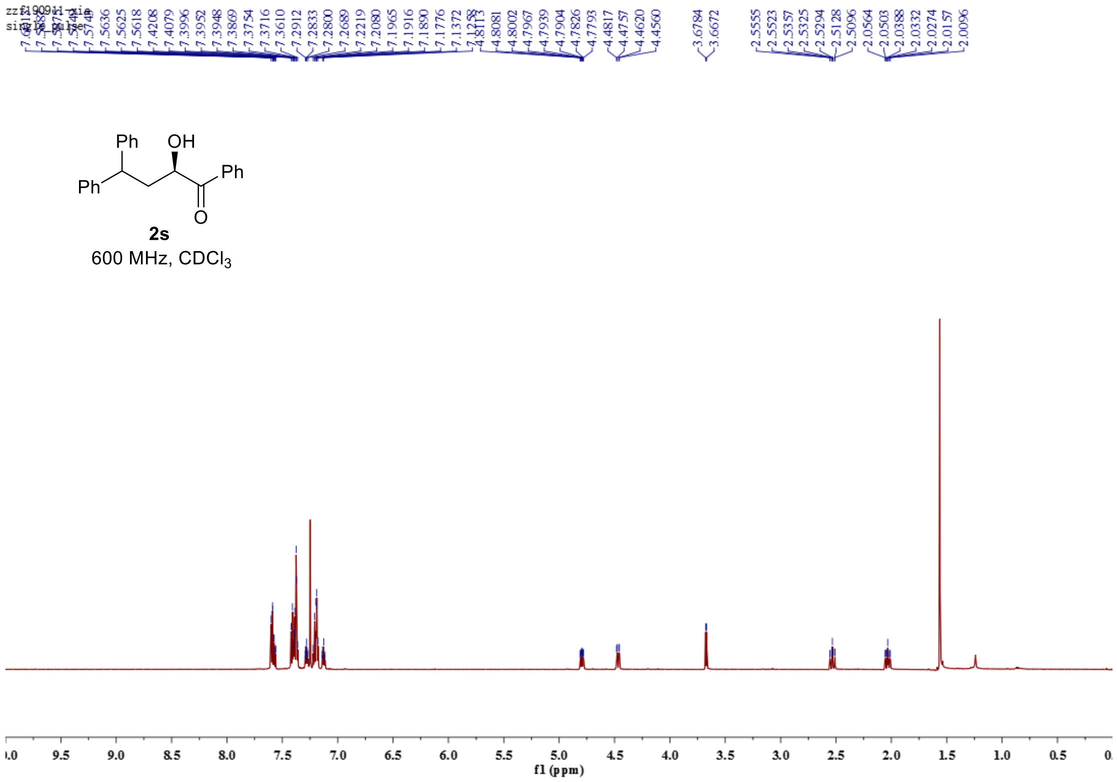
zzf190911-xia
single pulse
2s
600 MHz, CDCl3
f1 (ppm)

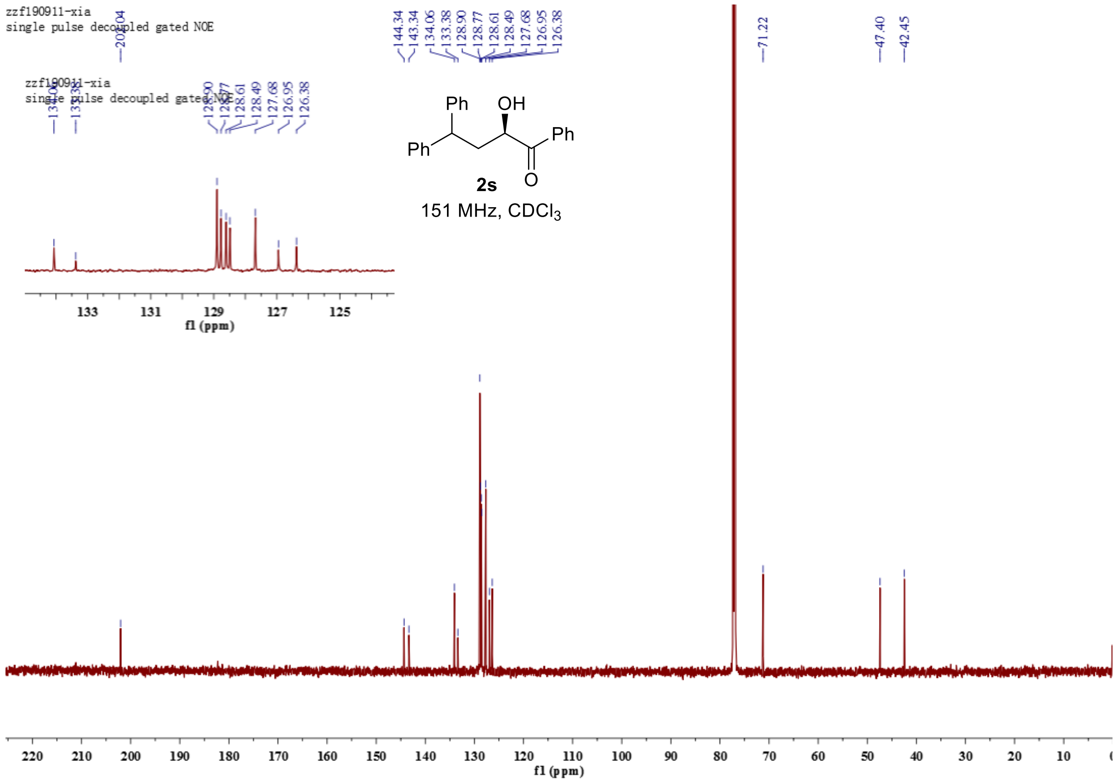
zzf190911-xia
single pulse decoupled gated NOE
202.04
144.34
143.34
134.06
133.38
128.90
128.77
128.61
128.49
127.68
126.95
126.38
71.22
47.40
42.45
2s
151 MHz, CDCl3
f1 (ppm)

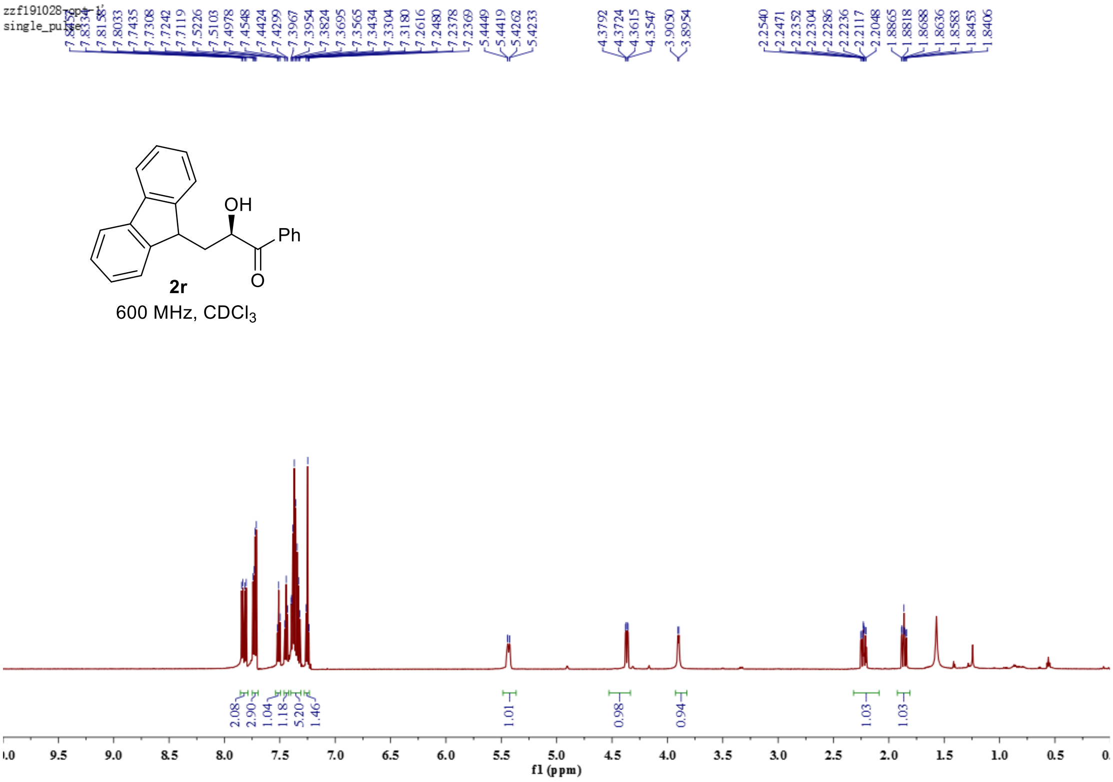
zzf191028-opt-1
single_pulse
2r
600 MHz, CDCl$_3$
2.08
2.90
1.04
1.18
5.20
1.46
1.01
0.98
0.94
1.03
1.03
7.8033
7.7435
7.7308
7.7242
7.7119
7.5226
7.5103
7.4978
7.4548
7.4424
7.4299
7.3967
7.3954
7.3824
7.3695
7.3565
7.3434
7.3304
7.3180
7.2616
7.2480
7.2378
7.2369
5.4449
5.4419
5.4262
5.4233
4.3792
4.3724
4.3615
4.3547
3.9050
3.8954
2.2540
2.2471
2.2352
2.2304
2.2286
2.2236
2.2117
2.2048
1.8865
1.8818
1.8688
1.8636
1.8583
1.8453
1.8406
f1 (ppm)

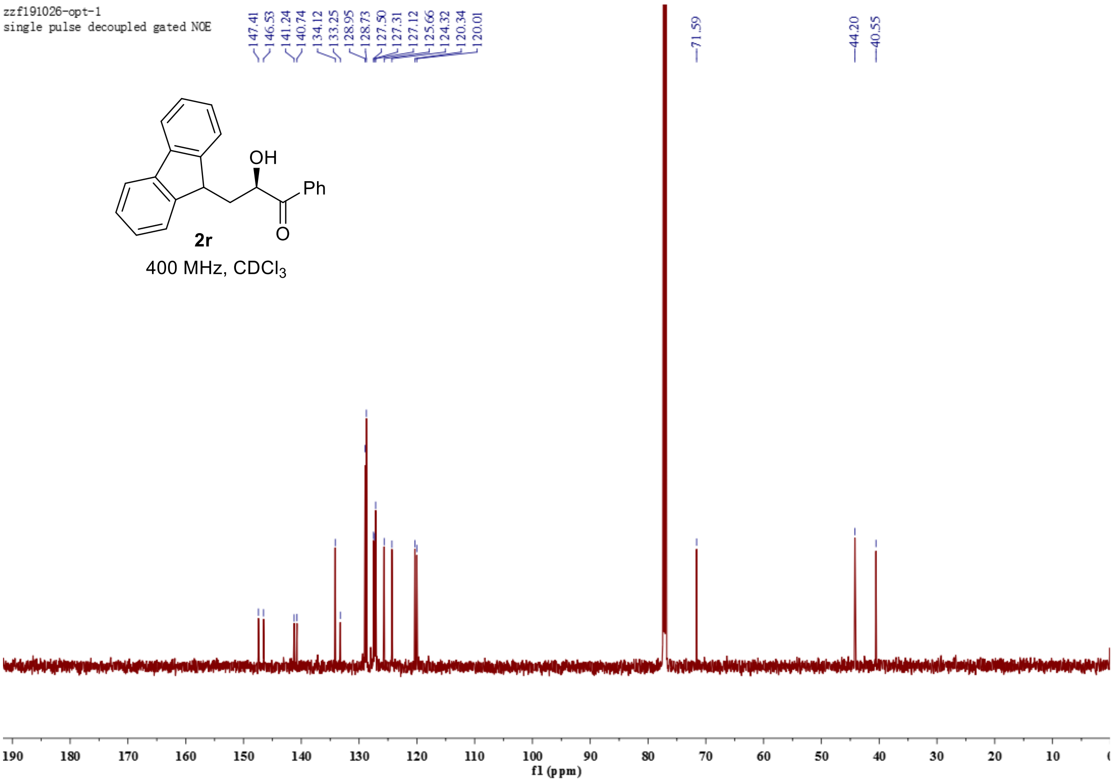
zzf191026-opt-1
single pulse decoupled gated NOE
2r
400 MHz, CDCl$_3$
147.41
146.53
141.24
140.74
134.12
133.25
128.95
128.73
127.50
127.31
127.12
125.66
124.32
120.34
120.01
71.59
44.20
40.55
f1 (ppm)

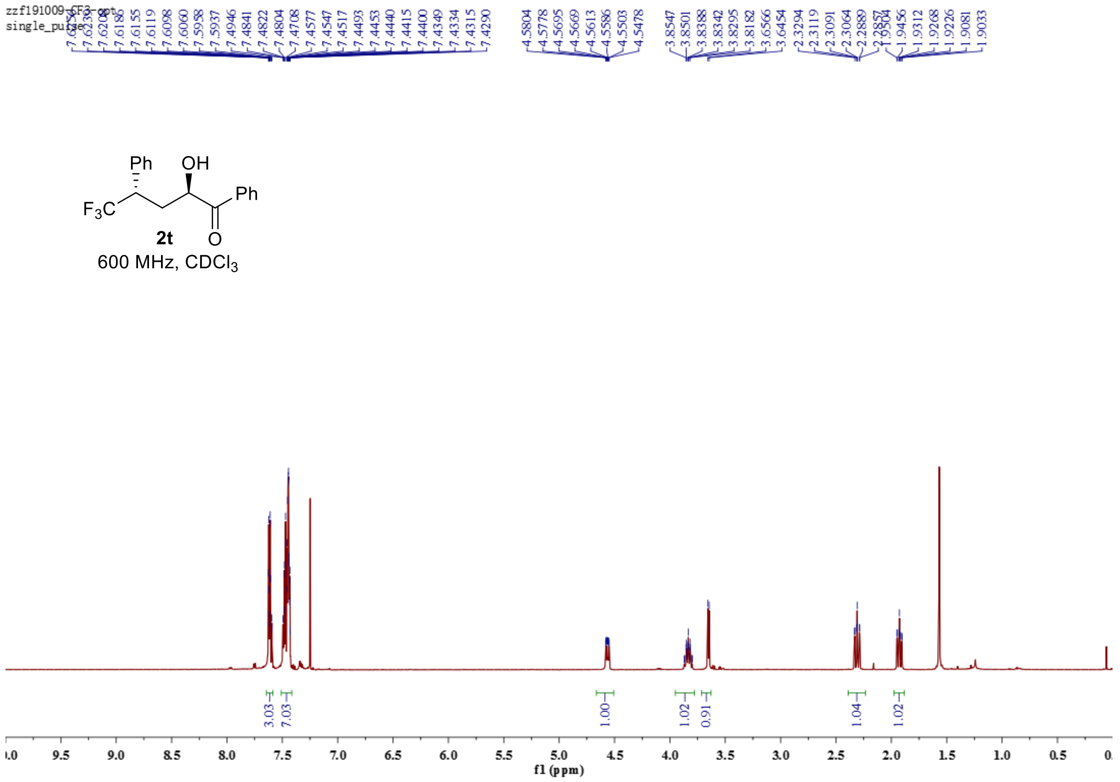

zzf191009-CF3-opt
single_pulse
7.6186
7.6155
7.6119
7.6098
7.6060
7.5958
7.5937
7.4946
7.4841
7.4822
7.4804
7.4708
7.4577
7.4547
7.4517
7.4493
7.4453
7.4440
7.4415
7.4400
7.4349
7.4334
7.4315
7.4290
4.5804
4.5778
4.5695
4.5669
4.5613
4.5586
4.5503
4.5478
3.8547
3.8501
3.8388
3.8342
3.8295
3.8182
3.6566
3.6454
2.3294
2.3119
2.3091
2.3064
2.2889
1.9456
1.9312
1.9268
1.9226
1.9081
1.9033
Ph
OH
Ph
F3C
O
2t
600 MHz, CDCl3
3.03
7.03
1.00
1.02
0.91
1.04
1.02
f1 (ppm)

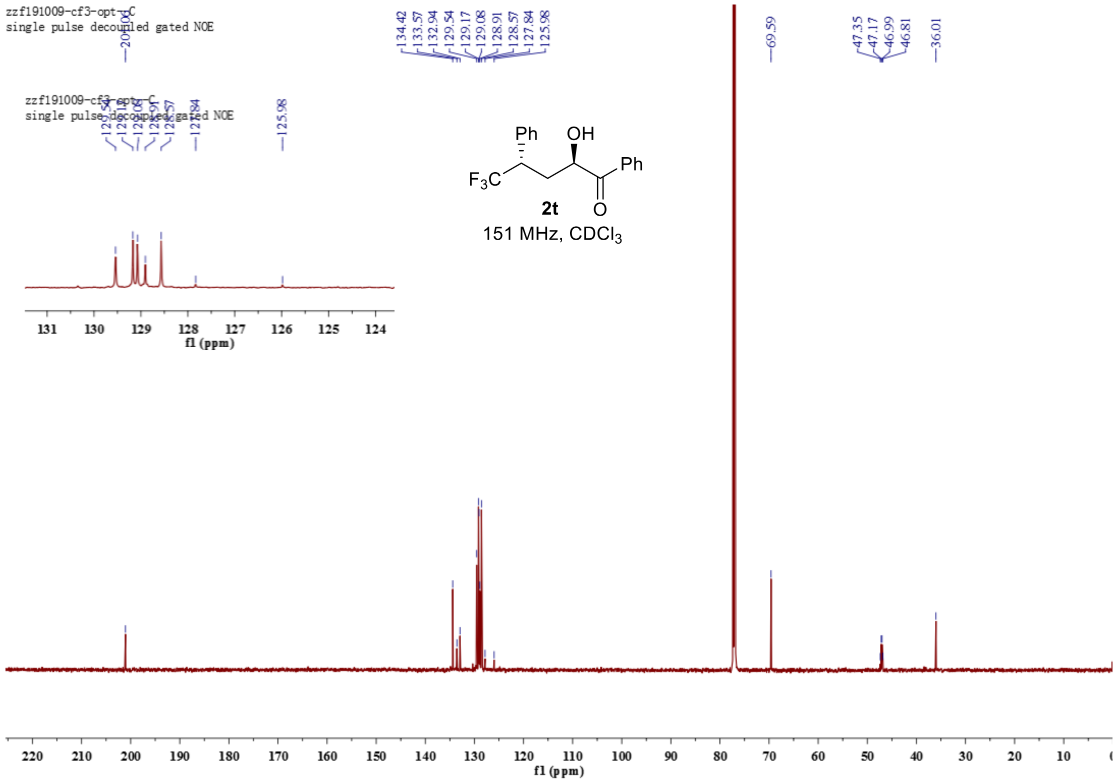

zzf191009-cf3-opt-C
single pulse decoupled gated NOE
134.42
133.57
132.94
129.54
129.17
129.08
128.91
128.57
127.84
125.98
69.59
47.35
47.17
46.99
46.81
36.01
127.84
125.98
Ph
OH
Ph
F3C
O
2t
151 MHz, CDCl3
f1 (ppm)

zzf210112-OMe-opt--1

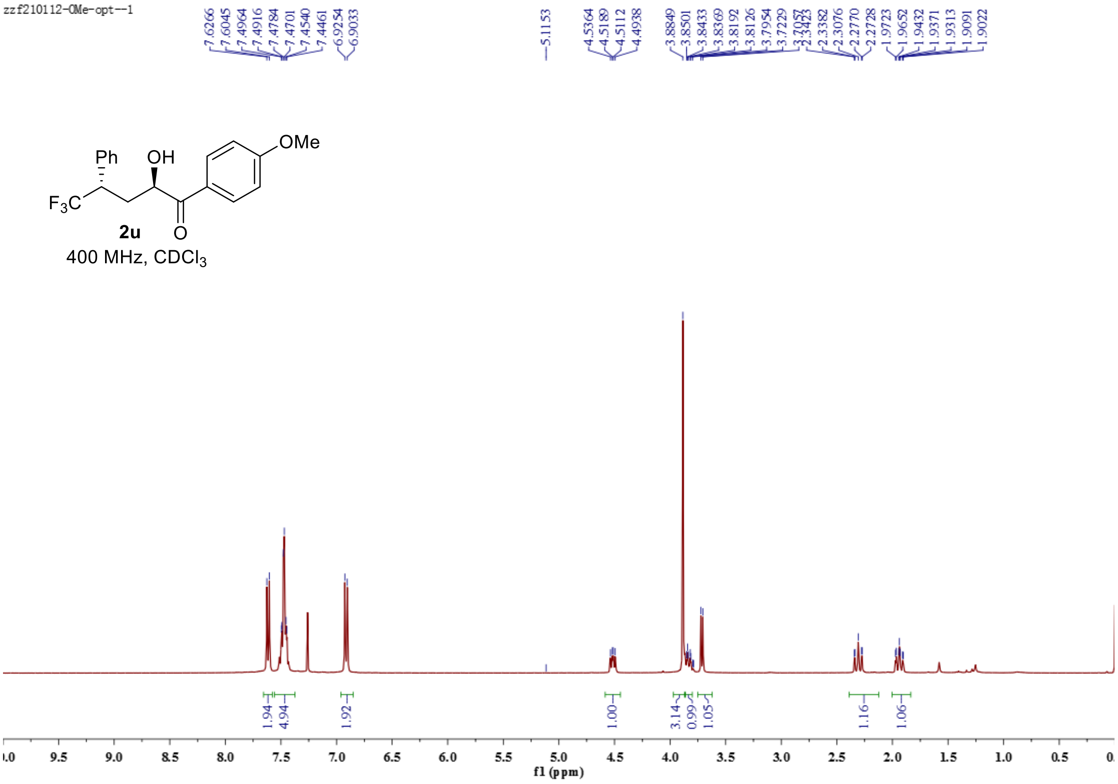

zzf210111-OMe-opt-1
single pulse decoupled gated NOE

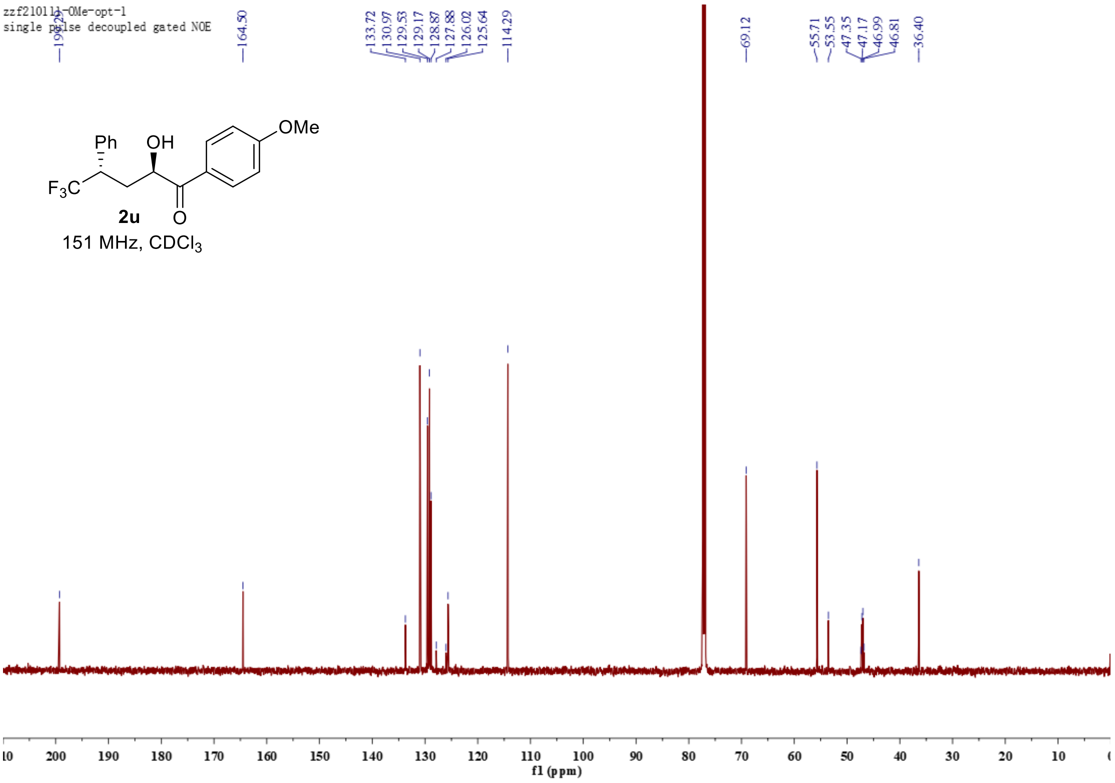

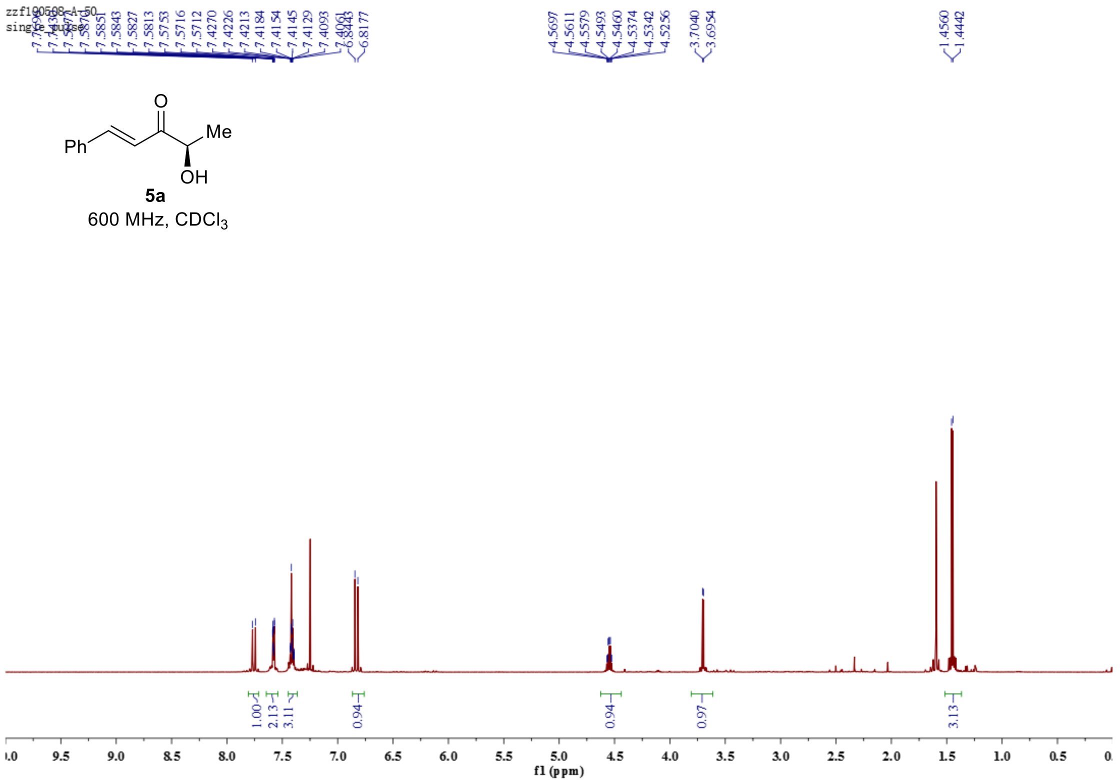
zzf190508-A-50
single pulse
7.5843 7.5827 7.5813 7.5753 7.5716 7.5712 7.4270 7.4226 7.4213 7.4184 7.4154 7.4145 7.4129 7.4093 7.4061 6.8443 6.8177
4.5697 4.5611 4.5579 4.5493 4.5460 4.5374 4.5342 4.5256
3.7040 3.6954
1.4560 1.4442
O
Ph
Me
OH
5a
600 MHz, CDCl3
1.00 2.13 3.11 0.94 0.94 0.97 3.13
9.5 9.0 8.5 8.0 7.5 7.0 6.5 6.0 5.5 5.0 4.5 4.0 3.5 3.0 2.5 2.0 1.5 1.0 0.5
f1 (ppm)

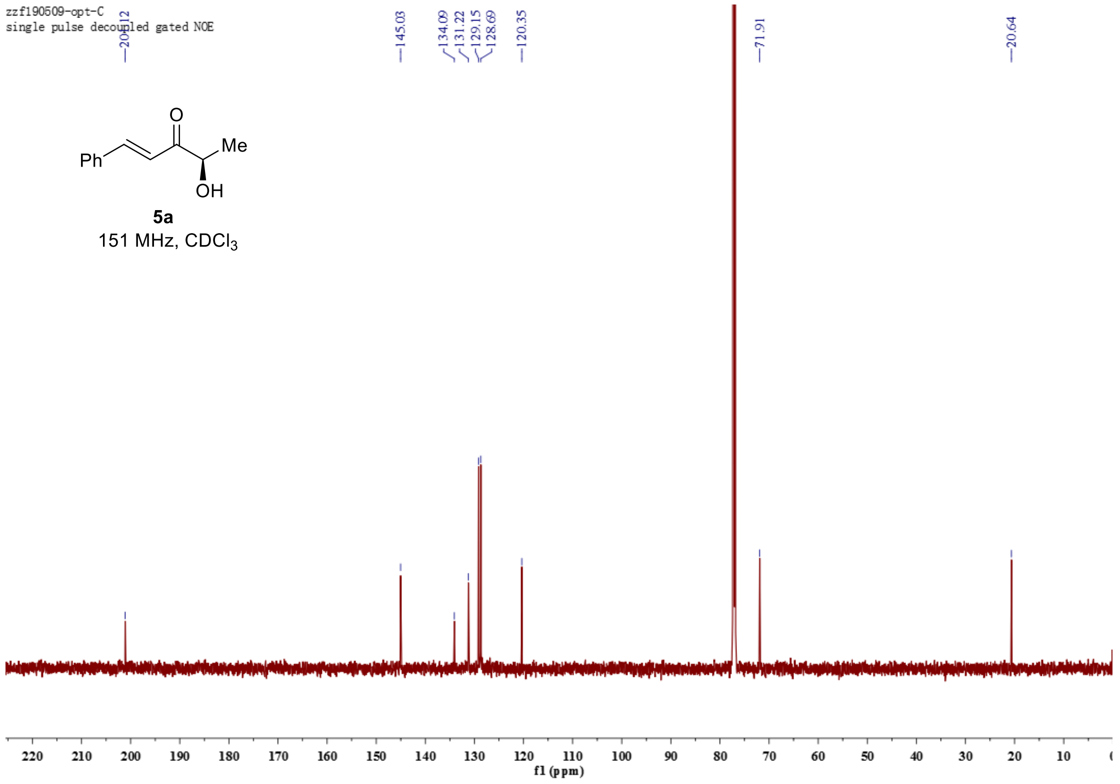
zzf190509-opt-C
single pulse decoupled gated NOE
201.12
145.03
134.09 131.22 129.15 128.69
120.35
71.91
20.64
O
Ph
Me
OH
5a
151 MHz, CDCl3
220 210 200 190 180 170 160 150 140 130 120 110 100 90 80 70 60 50 40 30 20 10
f1 (ppm)

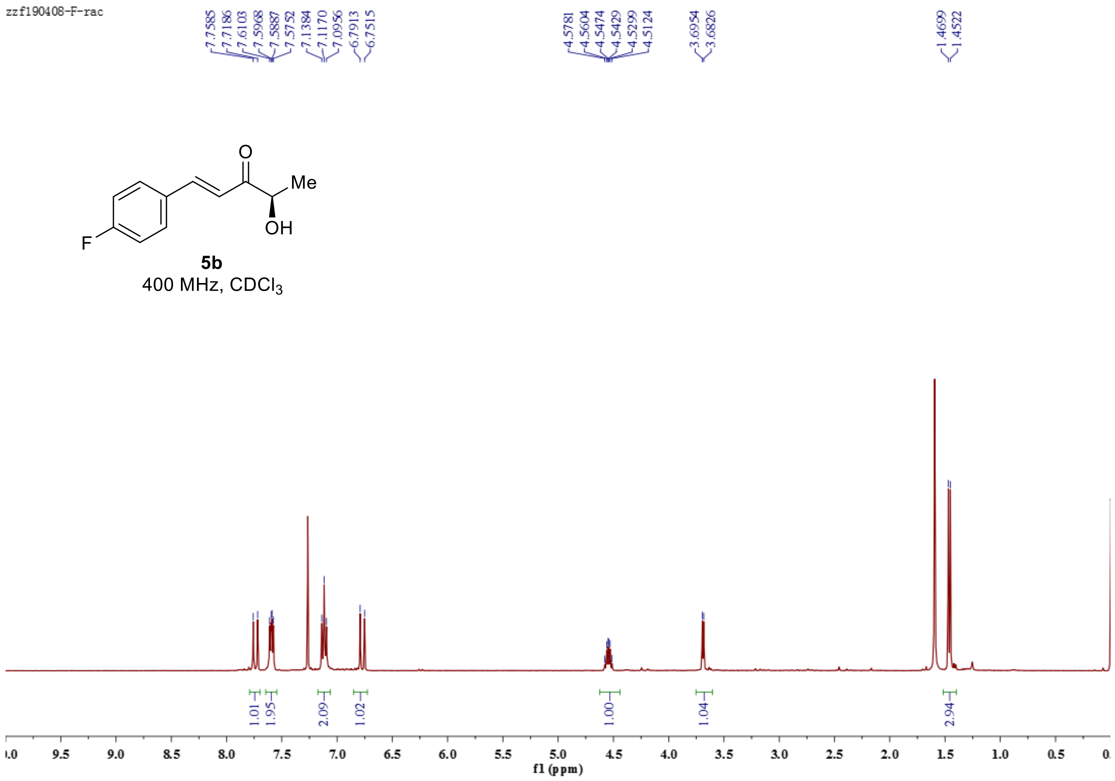
zzf190408-F-rac
7.7585
7.7186
7.6103
7.5968
7.5887
7.5752
7.1384
7.1170
7.0956
6.7913
6.7515
4.5781
4.5604
4.5474
4.5429
4.5299
4.5124
3.6954
3.6826
1.4699
1.4522
O
Me
F
OH
5b
400 MHz, CDCl3
1.01
1.95
2.09
1.02
1.00
1.04
2.94
9.5
9.0
8.5
8.0
7.5
7.0
6.5
6.0
5.5
5.0
4.5
4.0
3.5
3.0
2.5
2.0
1.5
1.0
0.5
f1 (ppm)

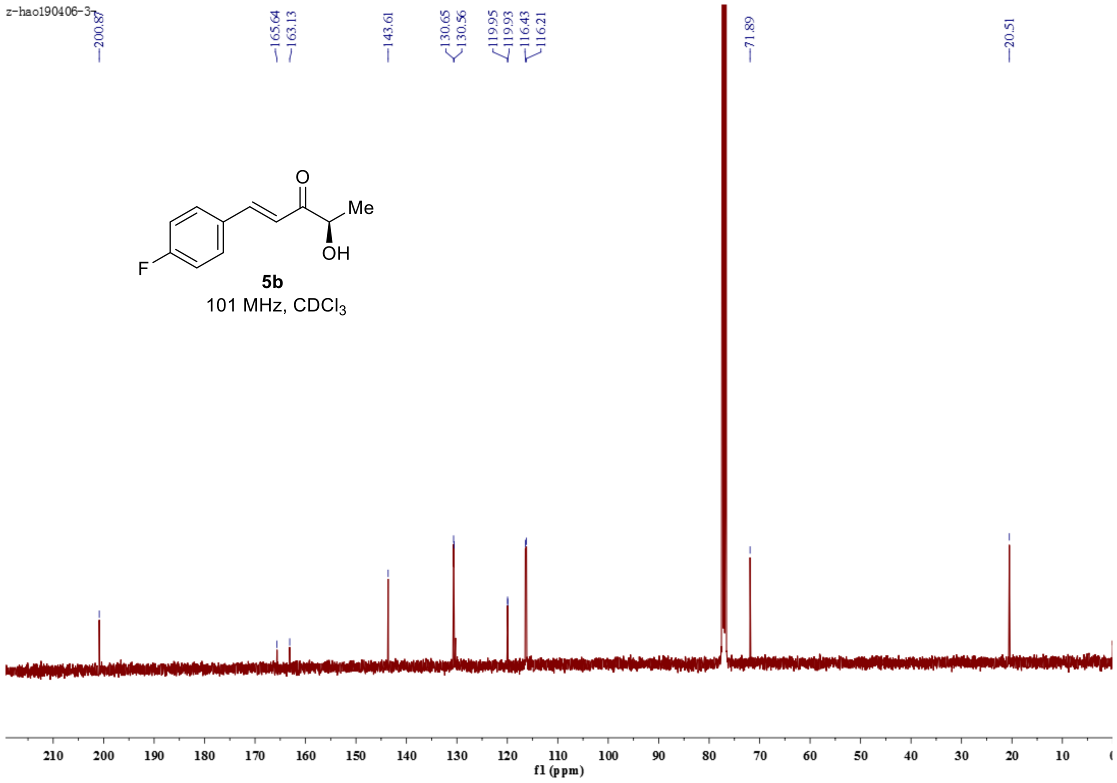
z-hao190406-3
200.87
165.64
163.13
143.61
130.65
130.56
119.95
119.93
116.43
116.21
71.89
20.51
O
Me
F
OH
5b
101 MHz, CDCl3
210
200
190
180
170
160
150
140
130
120
110
100
90
80
70
60
50
40
30
20
10
f1 (ppm)

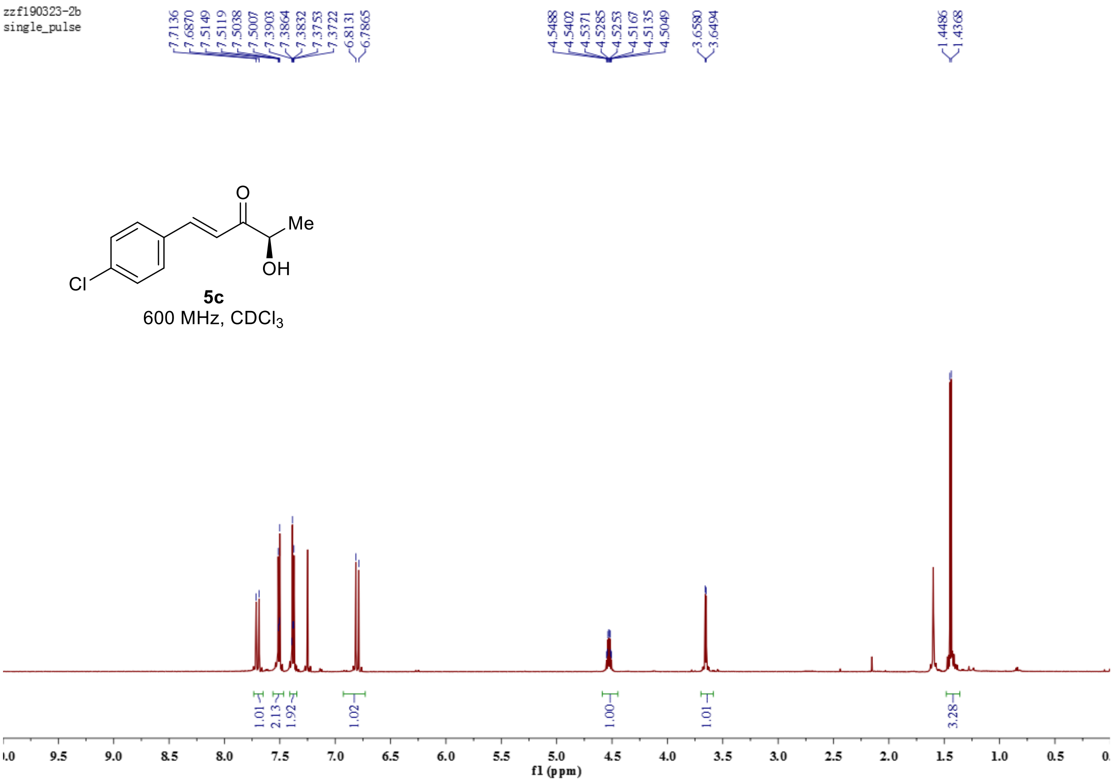
zzf190323-2b
single_pulse
7.7136
7.6870
7.5149
7.5119
7.5038
7.5007
7.3903
7.3864
7.3832
7.3753
7.3722
6.8131
6.7865
4.5488
4.5402
4.5371
4.5285
4.5253
4.5167
4.5135
4.5049
3.6580
3.6494
1.4486
1.4368
O
Me
OH
Cl
5c
600 MHz, $CDCl_3$
1.01
2.13
1.92
1.02
1.00
1.01
3.28
f1 (ppm)

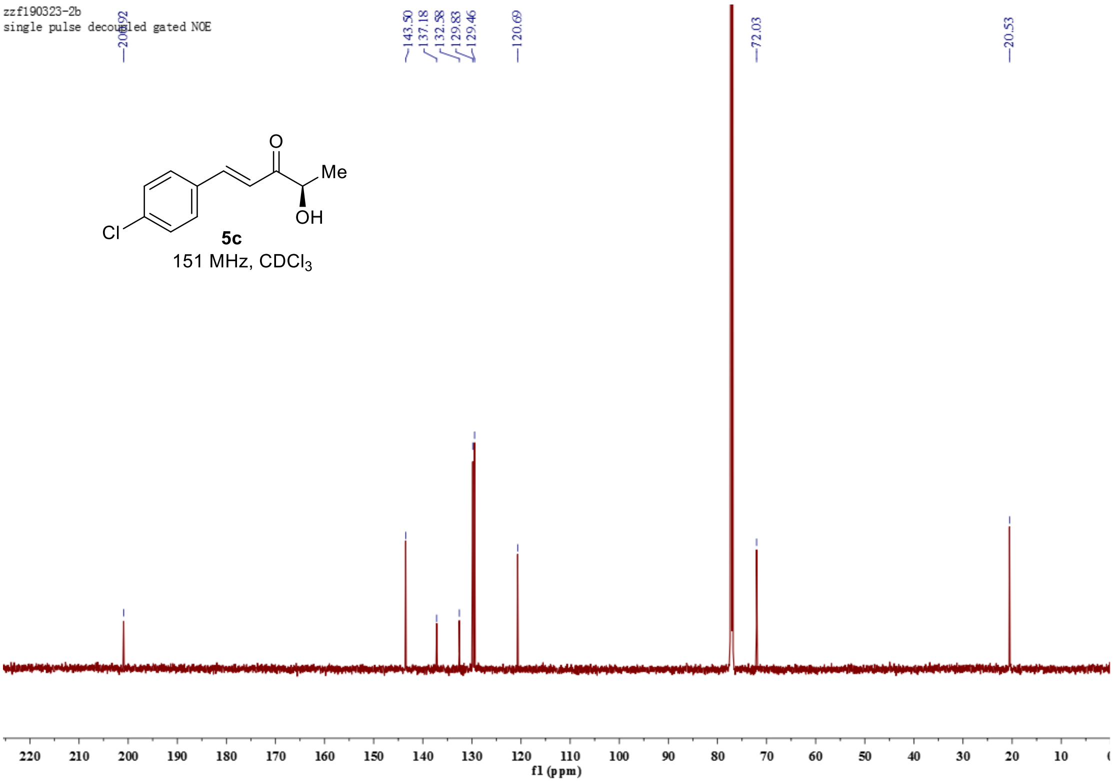
zzf190323-2b
single pulse decoupled gated NOE
200.92
143.50
137.18
132.58
129.83
129.46
120.69
72.03
20.53
O
Me
OH
Cl
5c
151 MHz, $CDCl_3$
f1 (ppm)

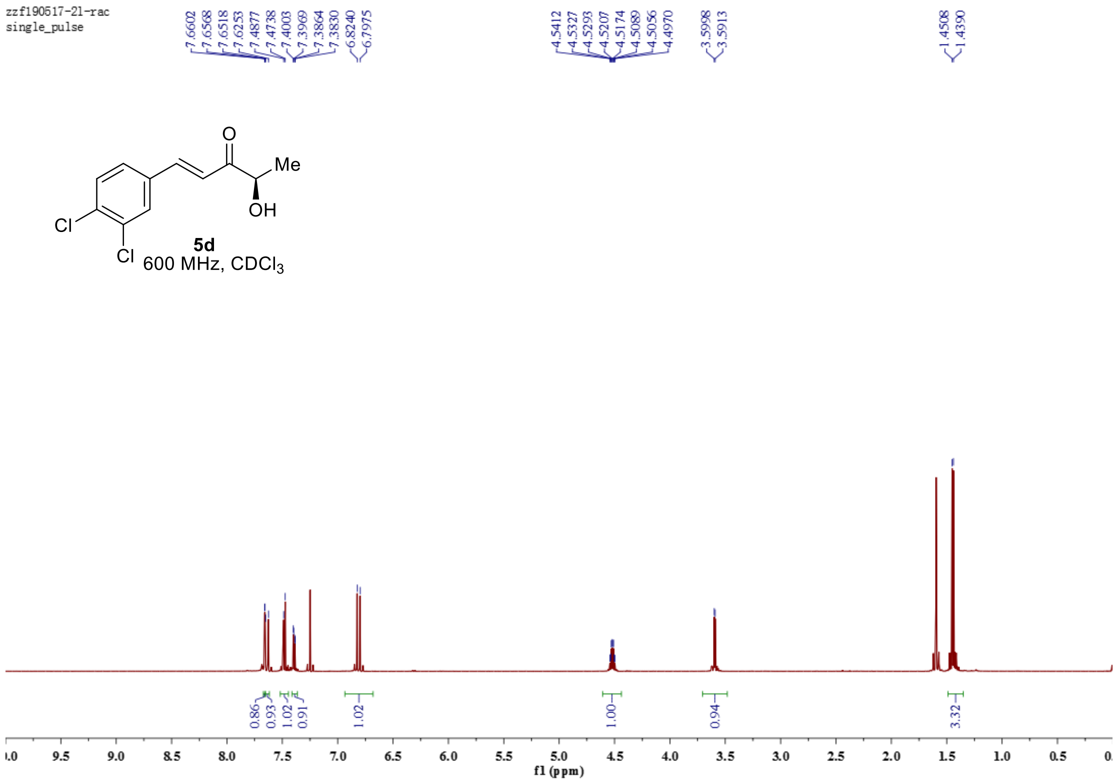
zzf190517-21-rac
single_pulse
7.6602
7.6568
7.6518
7.6253
7.4877
7.4738
7.4003
7.3969
7.3864
7.3830
6.8240
6.7975
4.5412
4.5327
4.5293
4.5207
4.5174
4.5089
4.5056
4.4970
3.5998
3.5913
1.4508
1.4390
O
Me
OH
Cl
Cl
5d
600 MHz, CDCl3
0.86
0.93
1.02
0.91
1.02
1.00
0.94
3.32
f1 (ppm)

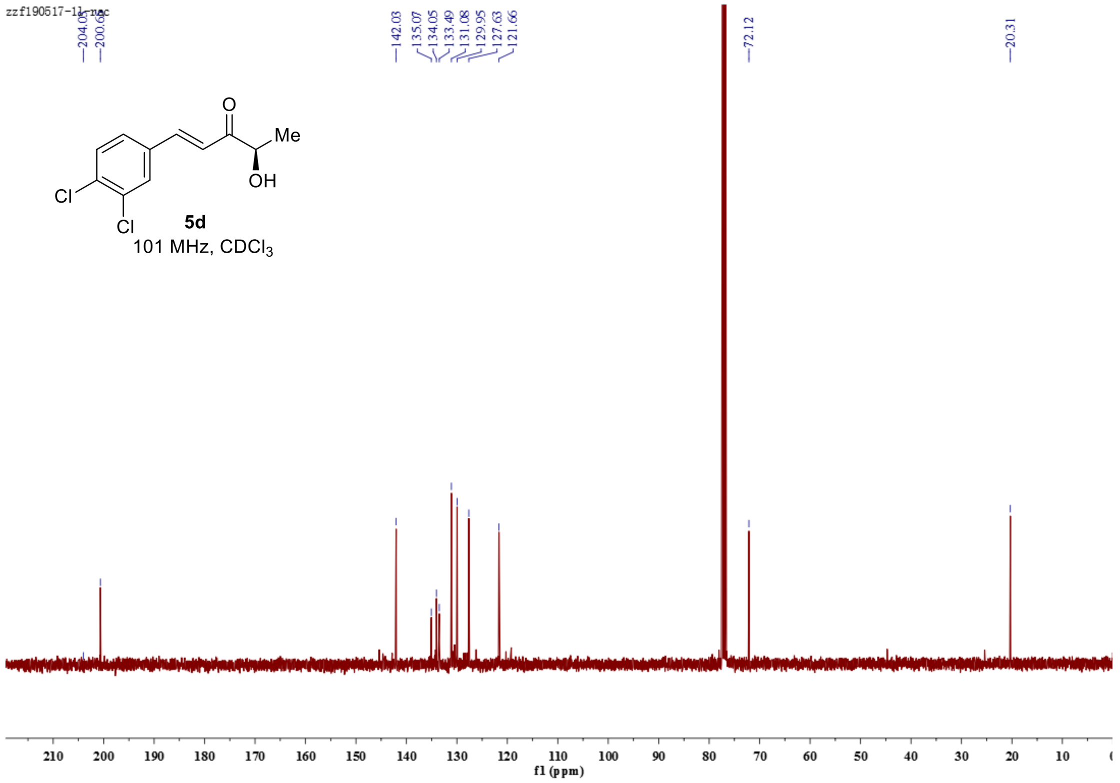
zzf190517-11-rac
204.03
200.68
142.03
135.07
134.05
133.49
131.08
129.95
127.63
121.66
72.12
20.31
O
Me
OH
Cl
Cl
5d
101 MHz, CDCl3
f1 (ppm)

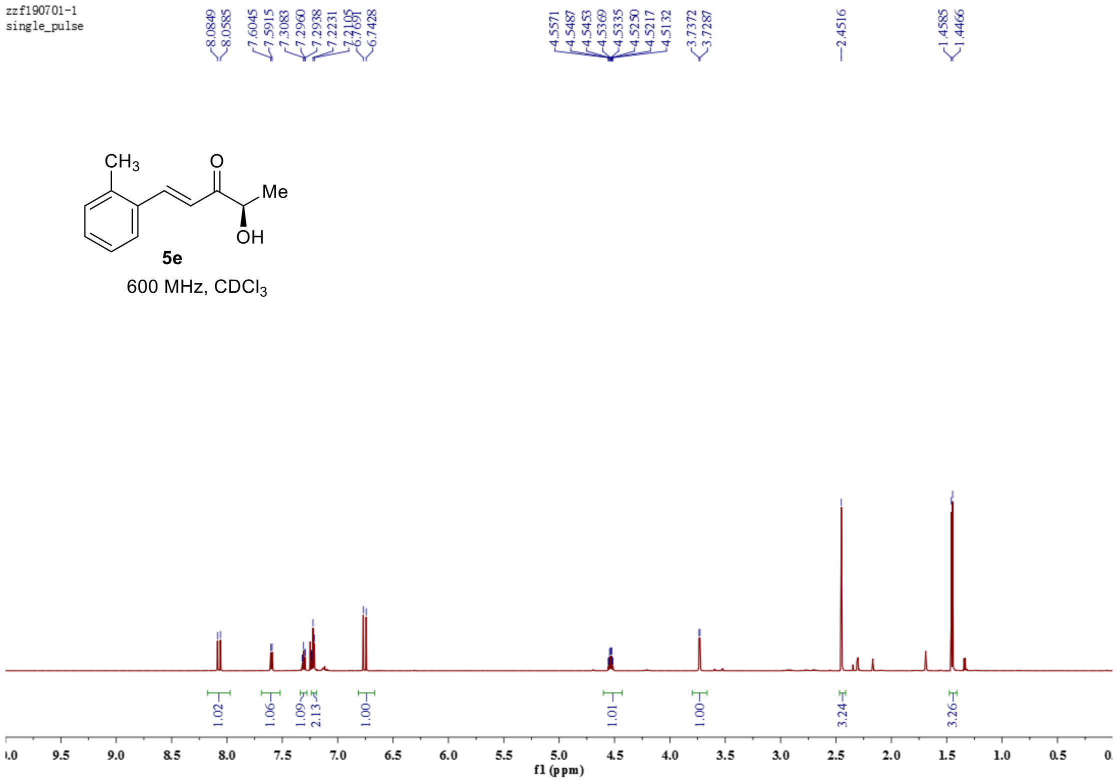
zzf190701-1
single_pulse
8.0849
8.0585
7.6045
7.5915
7.3083
7.2960
7.2938
7.2231
7.2105
6.7691
6.7428
4.5571
4.5487
4.5453
4.5369
4.5335
4.5250
4.5217
4.5132
3.7372
3.7287
2.4516
1.4585
1.4466
CH3
O
Me
OH
5e
600 MHz, CDCl3
1.02
1.06
1.09
2.13
1.00
1.01
1.00
3.24
3.26
f1 (ppm)

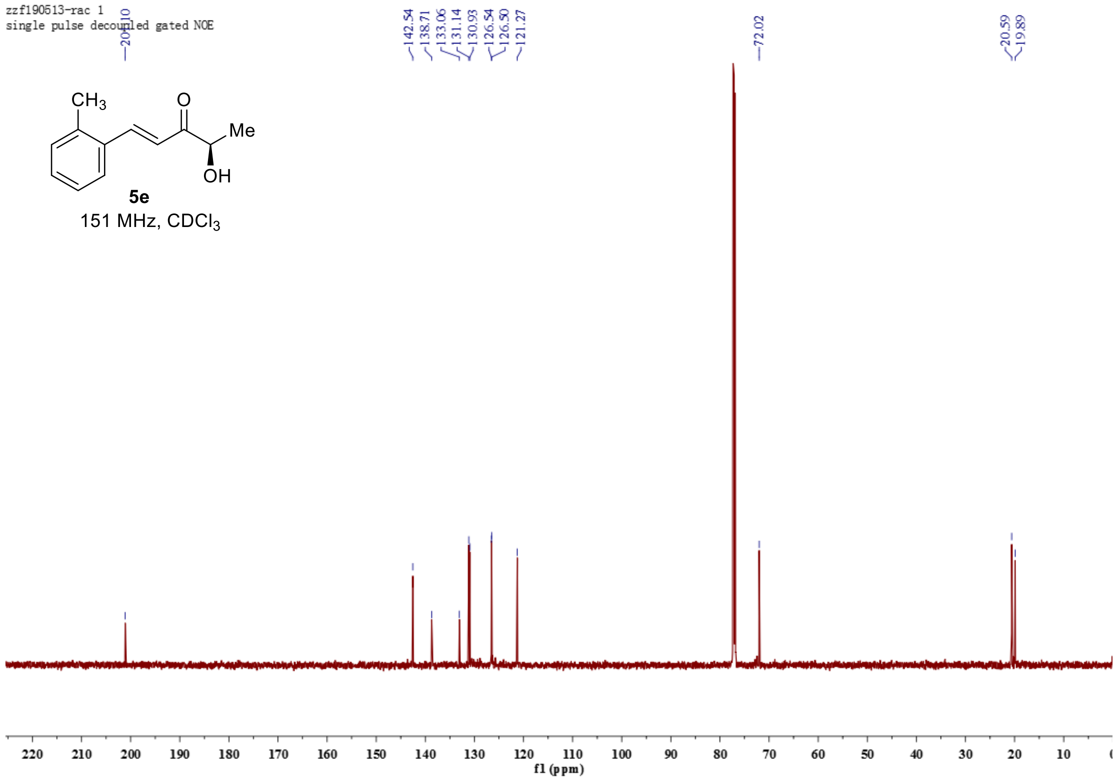
zzf190513-rac 1
single pulse decoupled gated NOE
142.54
138.71
133.06
131.14
130.93
126.54
126.50
121.27
72.02
20.59
19.89
CH3
O
Me
OH
5e
151 MHz, CDCl3
f1 (ppm)

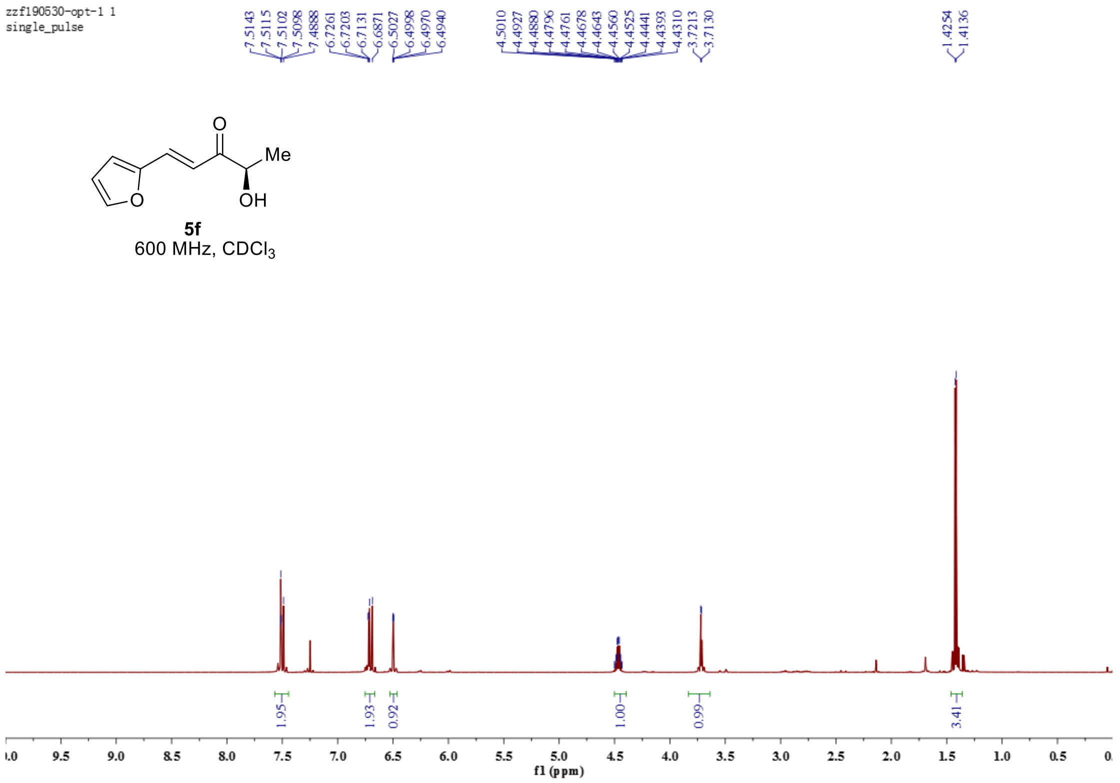

zzf190530-opt-1 1
single_pulse
7.5143
7.5115
7.5102
7.5098
7.4888
6.7261
6.7203
6.7131
6.6871
6.5027
6.4998
6.4970
6.4940
4.5010
4.4927
4.4880
4.4796
4.4761
4.4678
4.4643
4.4560
4.4525
4.4441
4.4393
4.4310
3.7213
3.7130
1.4254
1.4136
O
Me
O
OH
5f
600 MHz, CDCl3
1.95
1.93
0.92
1.00
0.99
3.41
f1 (ppm)

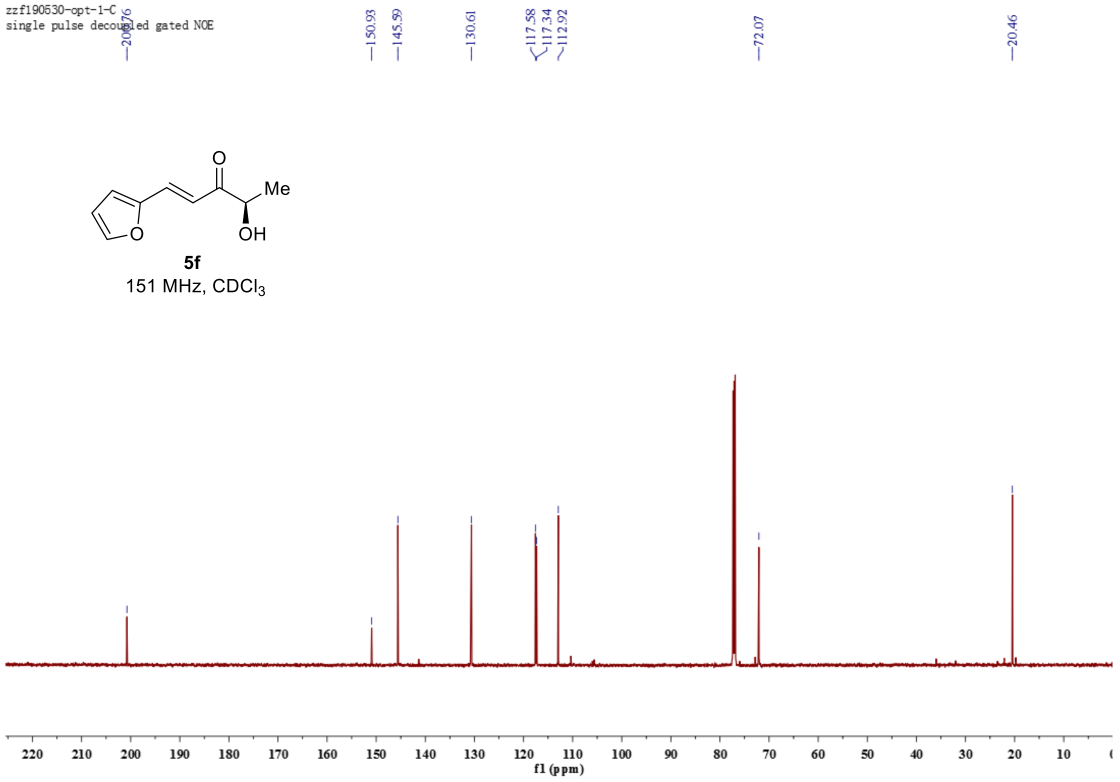

zzf190530-opt-1-C
single pulse decoupled gated NOE
200.76
150.93
145.59
130.61
117.58
117.34
112.92
72.07
20.46
O
Me
O
OH
5f
151 MHz, CDCl3
f1 (ppm)

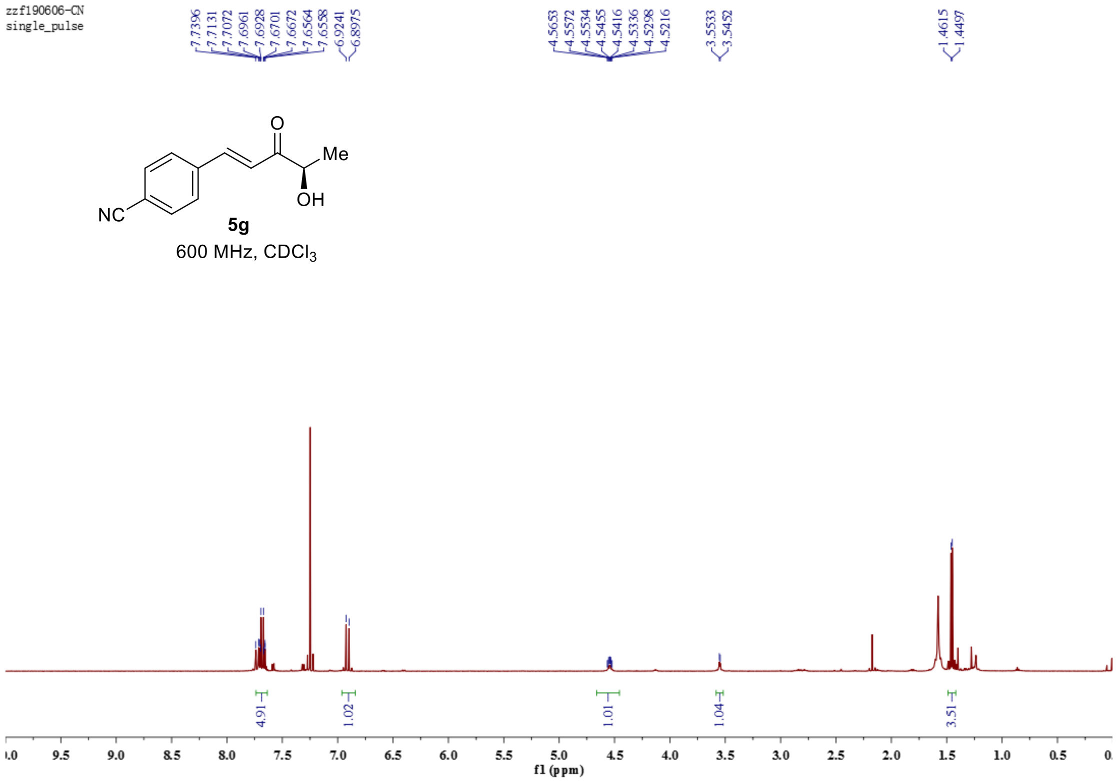
zzf190606-CN
single_pulse
7.7396
7.7131
7.7072
7.6961
7.6928
7.6701
7.6672
7.6564
7.6558
6.9241
6.8975
4.5653
4.5572
4.5534
4.5455
4.5416
4.5336
4.5298
4.5216
3.5533
3.5452
1.4615
1.4497
O
Me
OH
NC
5g
600 MHz, CDCl3
4.91
1.02
1.01
1.04
3.51
f1 (ppm)

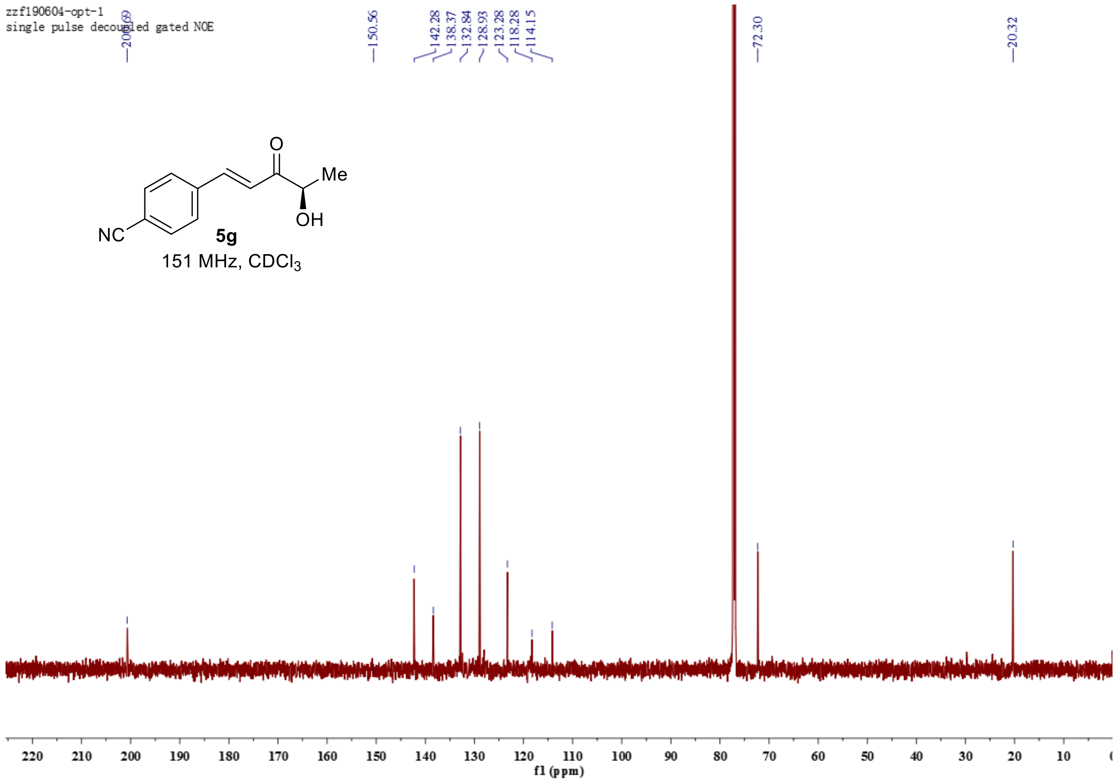
zzf190604-opt-1
single pulse decoupled gated NOE
200.69
150.56
142.28
138.37
132.84
128.93
123.28
118.28
114.15
72.30
20.32
O
Me
OH
NC
5g
151 MHz, CDCl3
f1 (ppm)

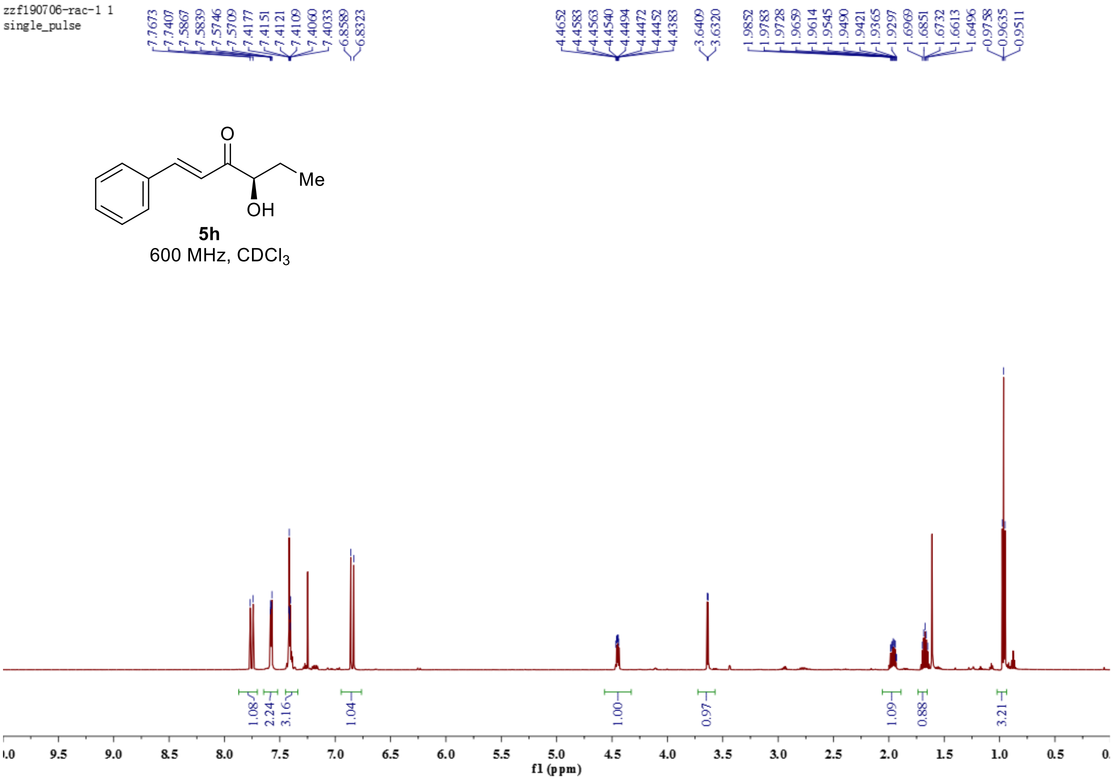
zzf190706-rac-1 1
single_pulse
7.7673
7.7407
7.5867
7.5839
7.5746
7.5709
7.4177
7.4151
7.4121
7.4109
7.4060
7.4033
6.8589
6.8323
4.4652
4.4583
4.4563
4.4540
4.4494
4.4472
4.4452
4.4383
3.6409
3.6320
1.9852
1.9783
1.9728
1.9659
1.9614
1.9545
1.9490
1.9421
1.9365
1.9297
1.6969
1.6851
1.6732
1.6613
1.6496
0.9758
0.9635
0.9511
O
Me
OH
5h
600 MHz, CDCl3
1.08
2.24
3.16
1.04
1.00
0.97
1.09
0.88
3.21
f1 (ppm)

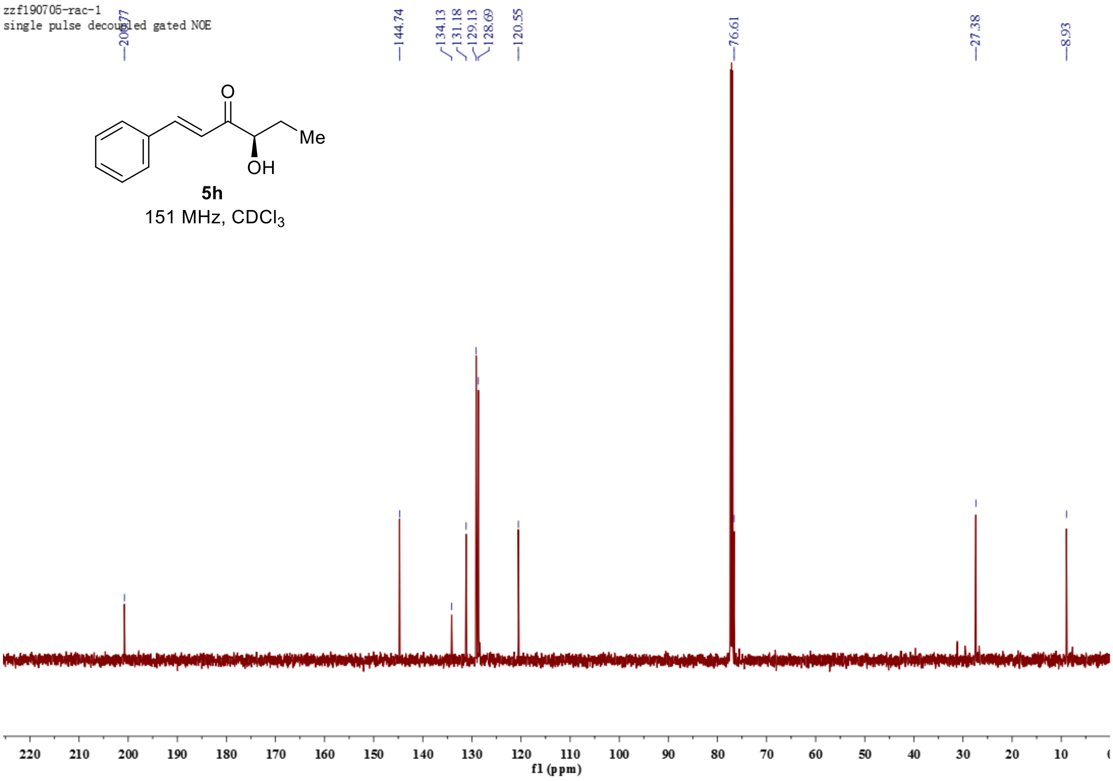
zzf190705-rac-1
single pulse decoupled gated NOE
144.74
134.13
131.18
129.13
128.69
120.55
76.61
27.38
8.93
O
Me
OH
5h
151 MHz, CDCl3
f1 (ppm)

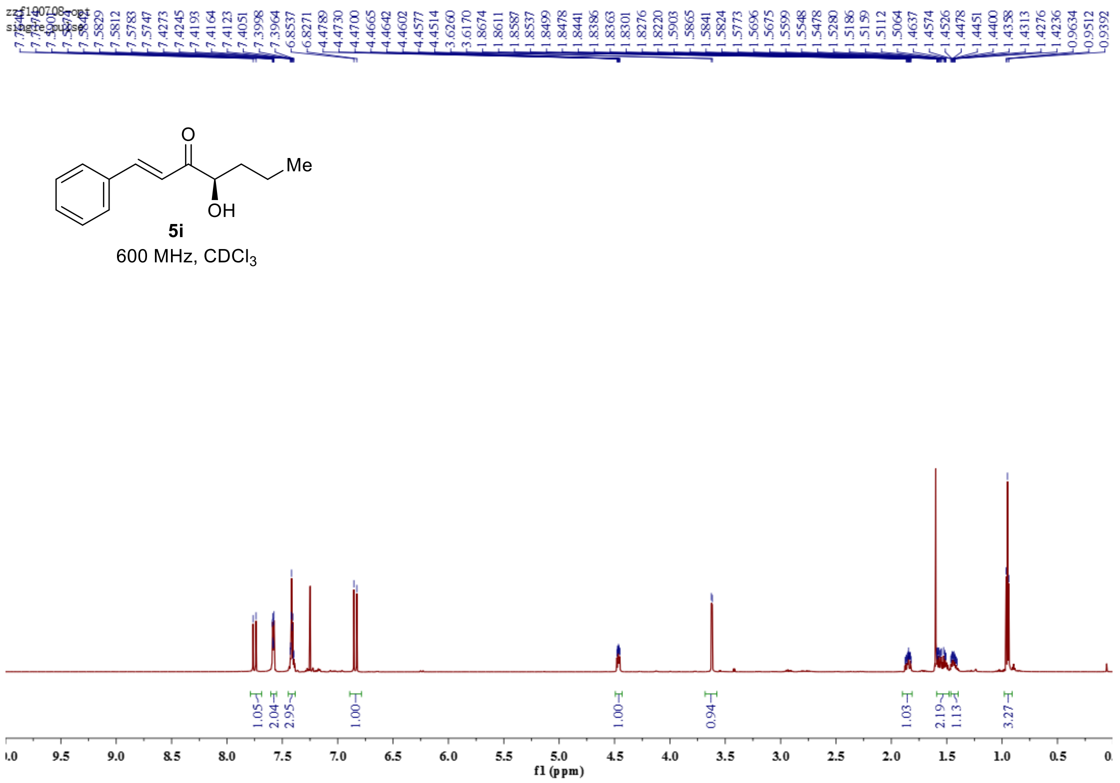

7.5829
7.5812
7.5783
7.5747
7.4273
7.4245
7.4193
7.4164
7.4123
7.4051
7.3998
7.3964
6.8537
6.8271
4.4789
4.4730
4.4700
4.4665
4.4642
4.4602
4.4577
4.4514
3.6260
3.6170
1.8674
1.8611
1.8587
1.8537
1.8499
1.8478
1.8441
1.8386
1.8363
1.8301
1.8276
1.8220
1.5903
1.5865
1.5841
1.5824
1.5773
1.5696
1.5675
1.5599
1.5548
1.5478
1.5280
1.5186
1.5159
1.5112
1.5064
1.4637
1.4574
1.4526
1.4478
1.4451
1.4400
1.4358
1.4313
1.4276
1.4236
0.9634
0.9512
0.9392
O
Me
OH
5i
600 MHz, CDCl3
1.05
2.04
2.95
1.00
1.00
0.94
1.03
2.19
1.13
3.27
f1 (ppm)

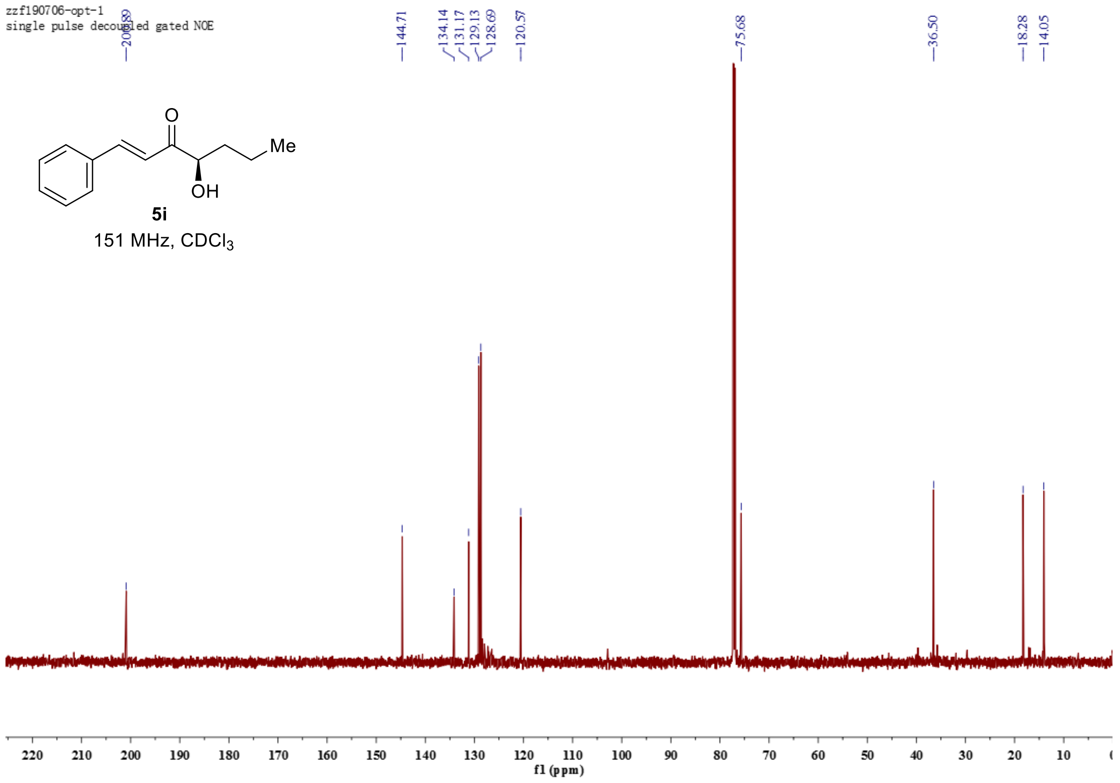

zzf190706-opt-1
single pulse decoupled gated NOE
144.71
134.14
131.17
129.13
128.69
120.57
75.68
36.50
18.28
14.05
O
Me
OH
5i
151 MHz, CDCl3
f1 (ppm)

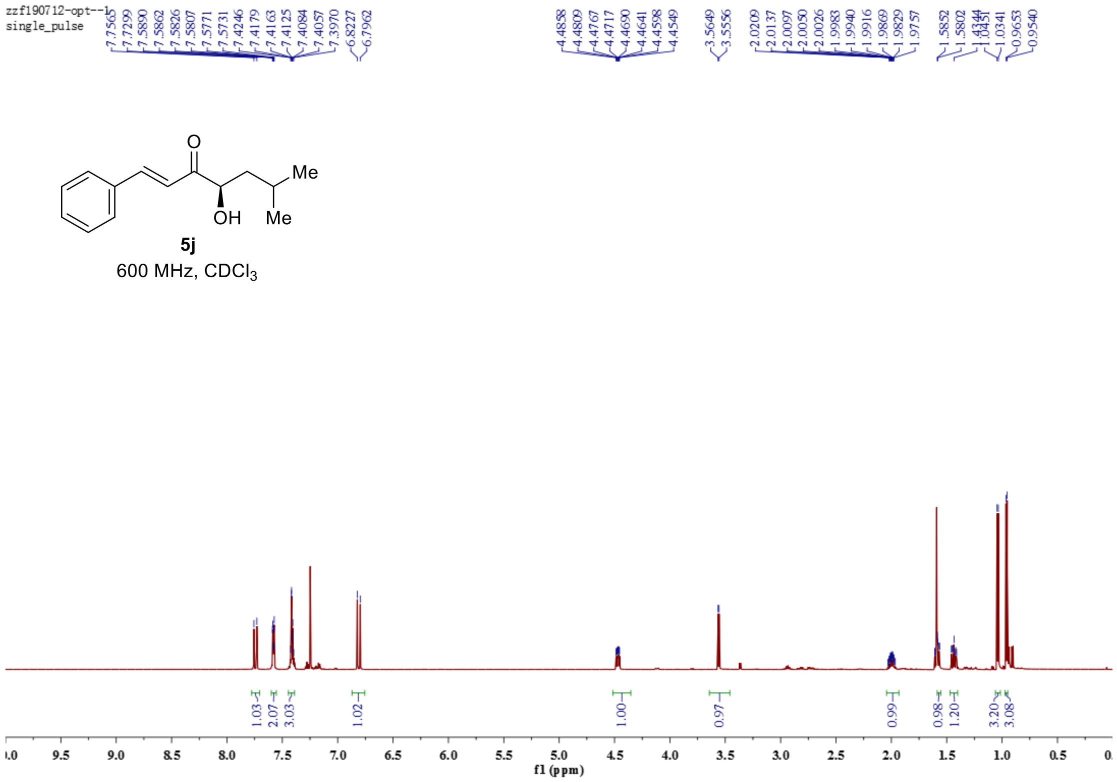
zzf190712-opt--1
single_pulse
7.7565
7.7299
7.5890
7.5862
7.5826
7.5807
7.5771
7.5731
7.4246
7.4179
7.4163
7.4125
7.4084
7.4057
7.3970
6.8227
6.7962
4.4858
4.4809
4.4767
4.4717
4.4690
4.4641
4.4598
4.4549
3.5649
3.5556
2.0209
2.0137
2.0097
2.0050
2.0026
1.9983
1.9940
1.9916
1.9869
1.9829
1.9757
1.5852
1.5802
1.0451
1.0341
0.9653
0.9540
O
Me
OH
Me
5j
600 MHz, $CDCl_3$
1.03
2.07
3.03
1.02
1.00
0.97
0.99
0.98
1.20
3.20
3.08
9.5
9.0
8.5
8.0
7.5
7.0
6.5
6.0
5.5
5.0
4.5
4.0
3.5
3.0
2.5
2.0
1.5
1.0
0.5
f1 (ppm)

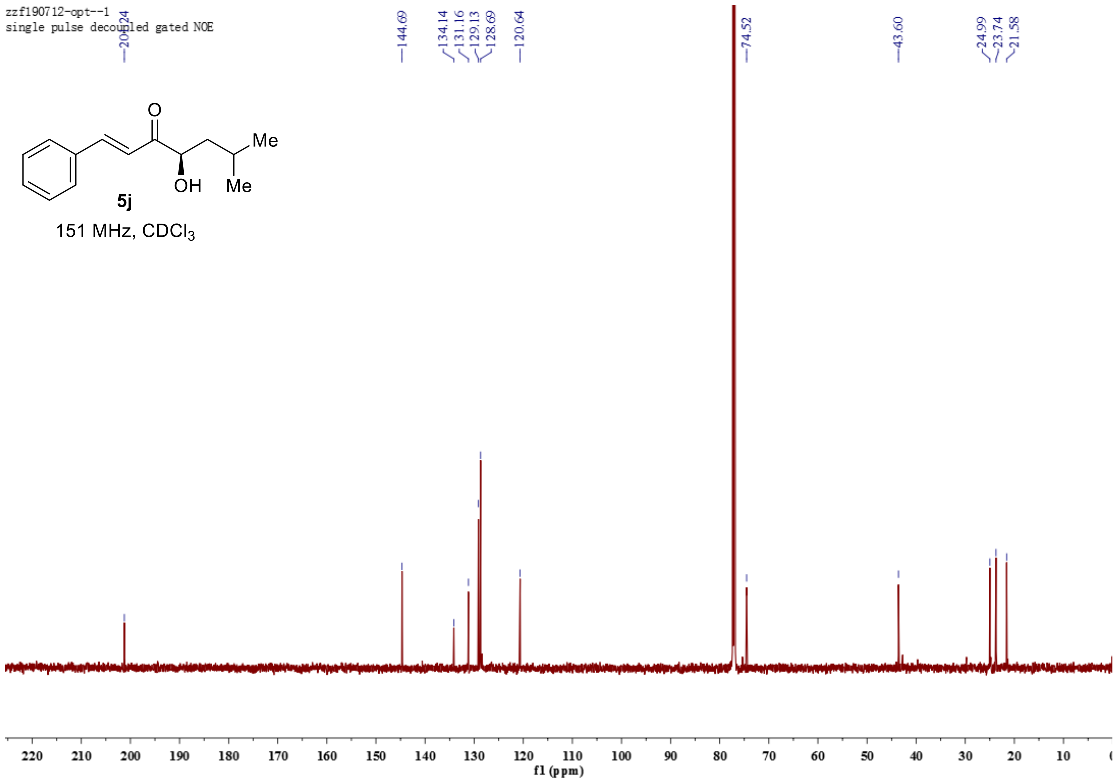
zzf190712-opt--1
single pulse decoupled gated NOE
—144.69
134.14
131.16
129.13
128.69
—120.64
—74.52
—43.60
24.99
23.74
21.58
O
Me
OH
Me
5j
151 MHz, $CDCl_3$
220
210
200
190
180
170
160
150
140
130
120
110
100
90
80
70
60
50
40
30
20
10
f1 (ppm)

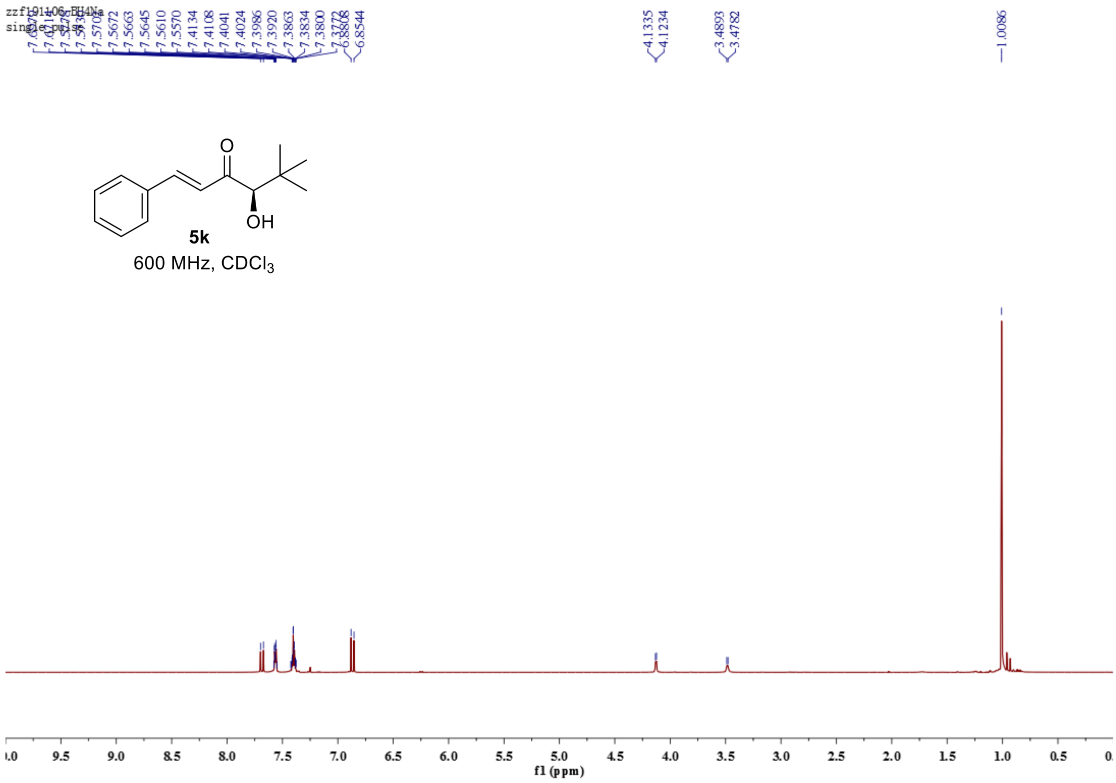
zzf191106-BH4Na
single pulse
4.1335
4.1234
3.4893
3.4782
1.0086
O
OH
5k
600 MHz, CDCl3
f1 (ppm)

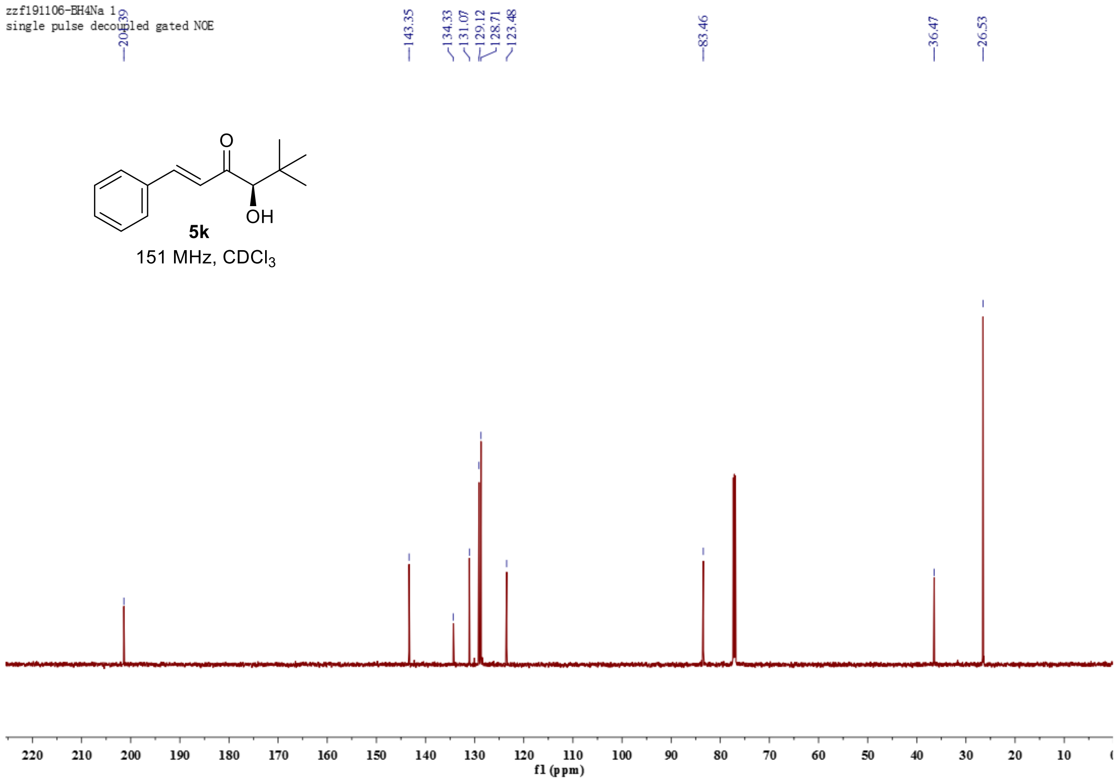
zzf191106-BH4Na 1
single pulse decoupled gated NOE
143.35
134.33
131.07
129.12
128.71
123.48
83.46
36.47
26.53
O
OH
5k
151 MHz, CDCl3
f1 (ppm)

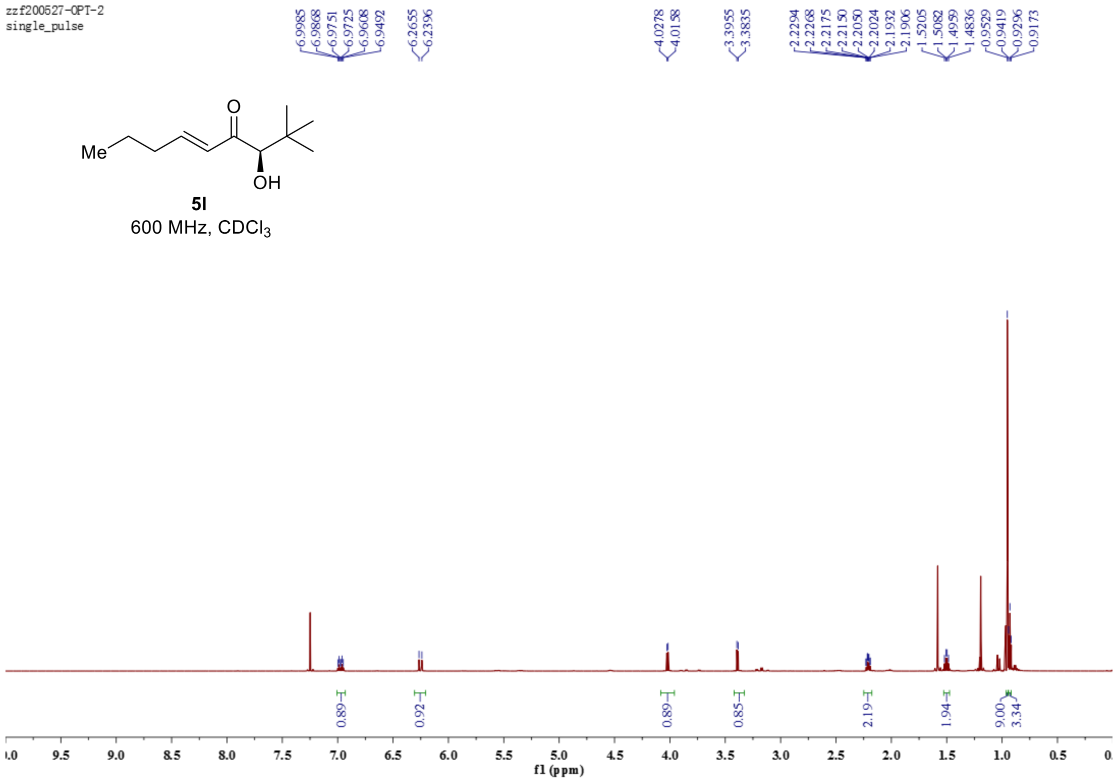
zzf200527-OPT-2
single_pulse
5l
600 MHz, CDCl3
f1 (ppm)

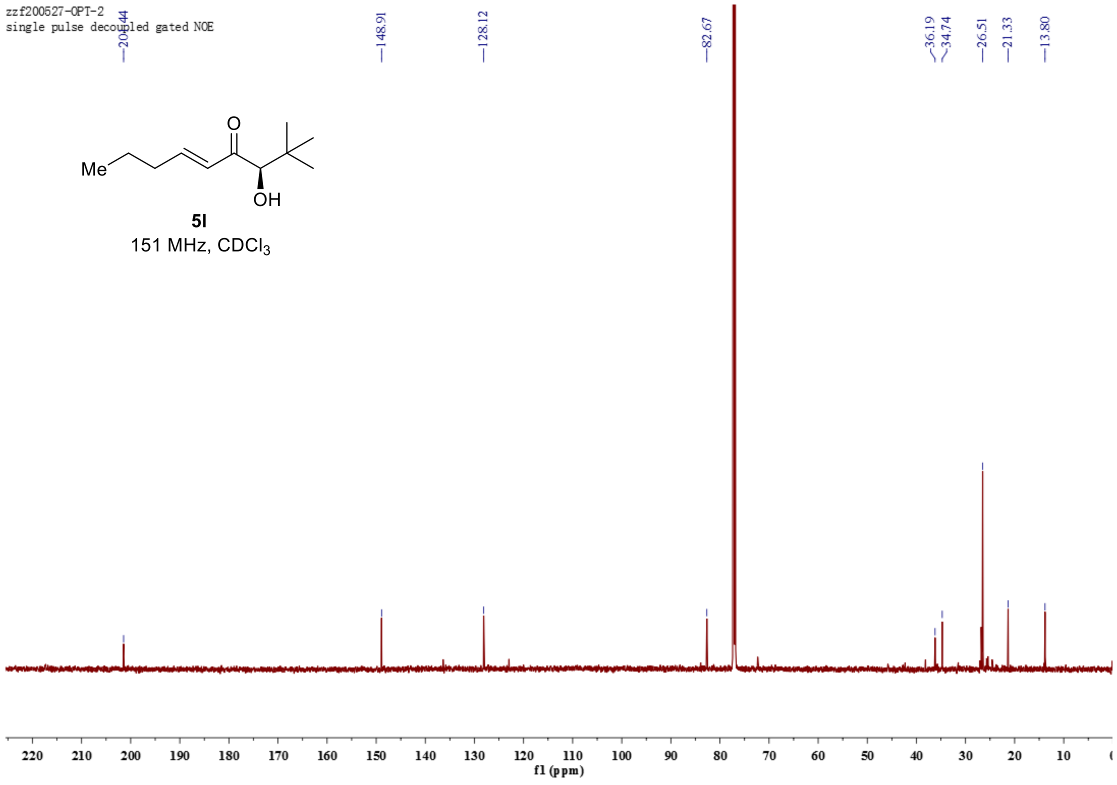
zzf200527-OPT-2
single pulse decoupled gated NOE
5l
151 MHz, CDCl3
f1 (ppm)

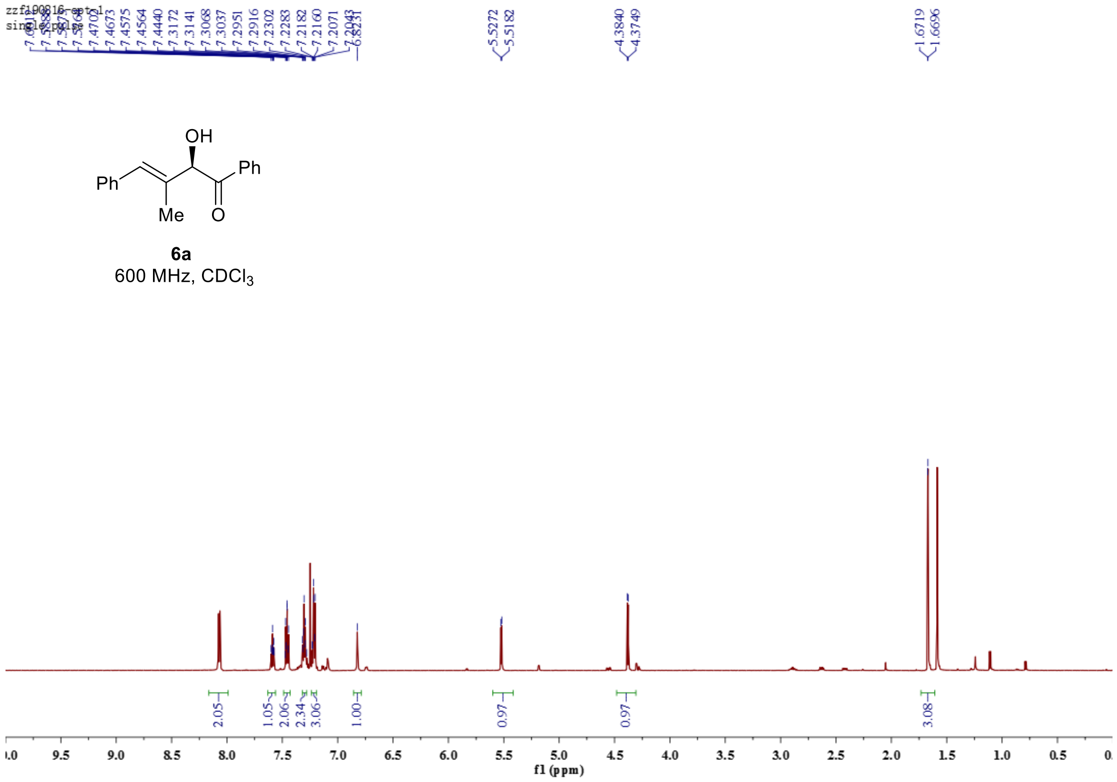
zzf190816-opt-1
5.5272
5.5182
4.3840
4.3749
1.6719
1.6696
OH
Ph
Ph
Me
O
6a
600 MHz, CDCl3
2.05
1.05
2.06
2.34
3.06
1.00
0.97
0.97
3.08
f1 (ppm)

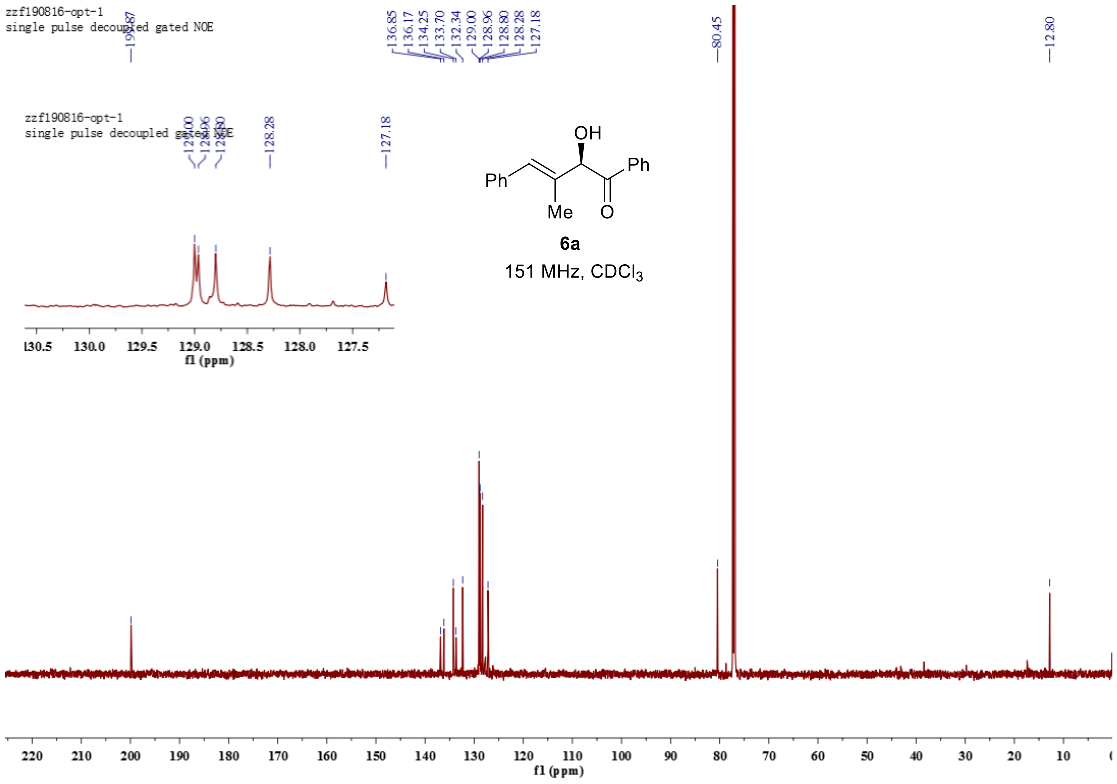
zzf190816-opt-1
single pulse decoupled gated NOE
136.85
136.17
134.25
133.70
132.34
129.00
128.96
128.80
128.28
127.18
80.45
12.80
OH
Ph
Ph
Me
O
6a
151 MHz, CDCl3
f1 (ppm)

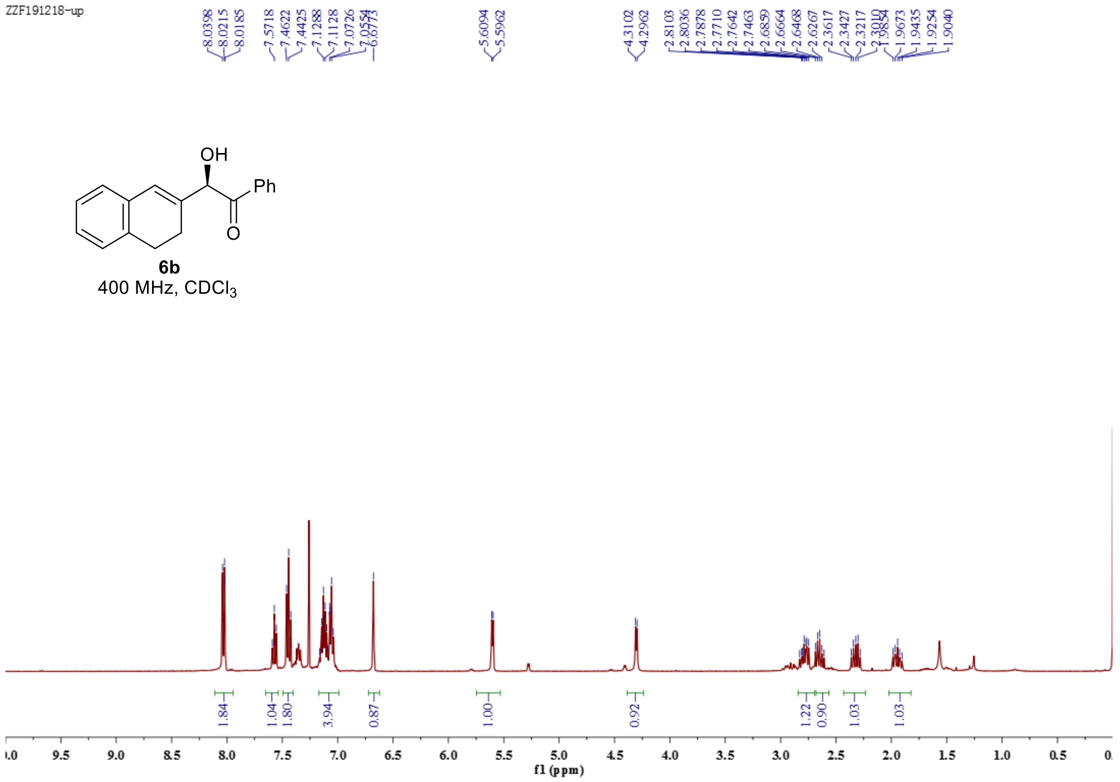
ZZF191218-up
OH
Ph
O
6b
400 MHz, CDCl3
f1 (ppm)

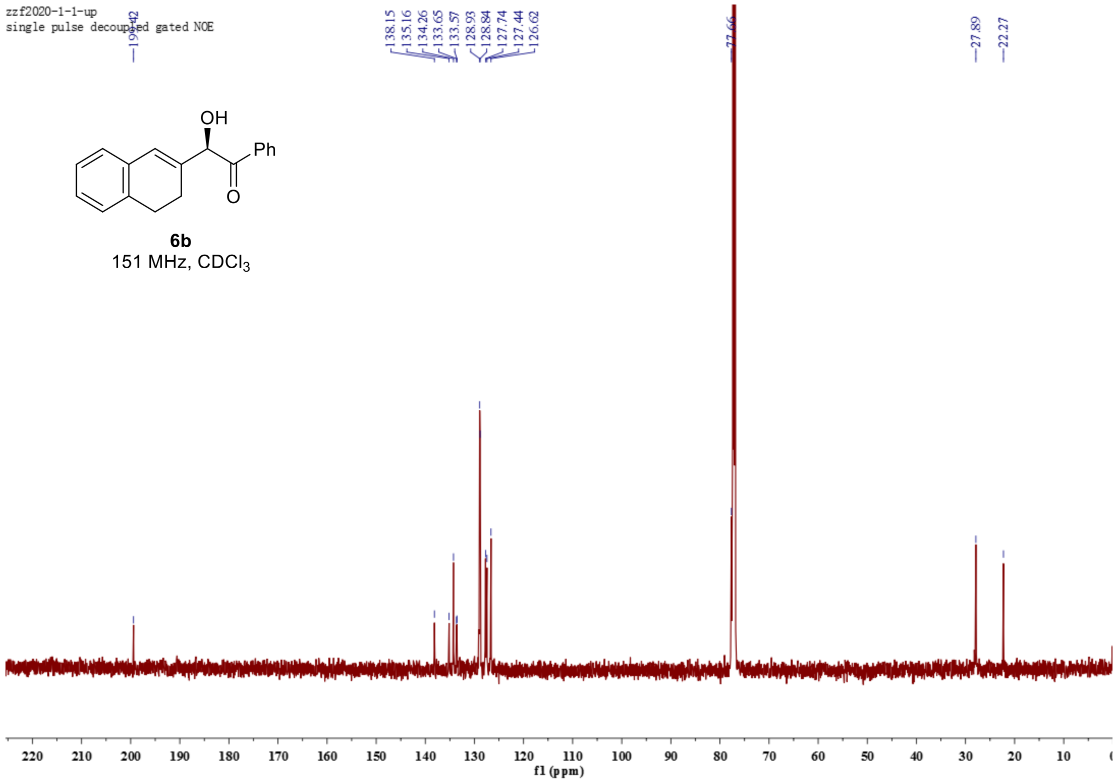
zzf2020-1-1-up
single pulse decoupled gated NOE
OH
Ph
O
6b
151 MHz, CDCl3
f1 (ppm)

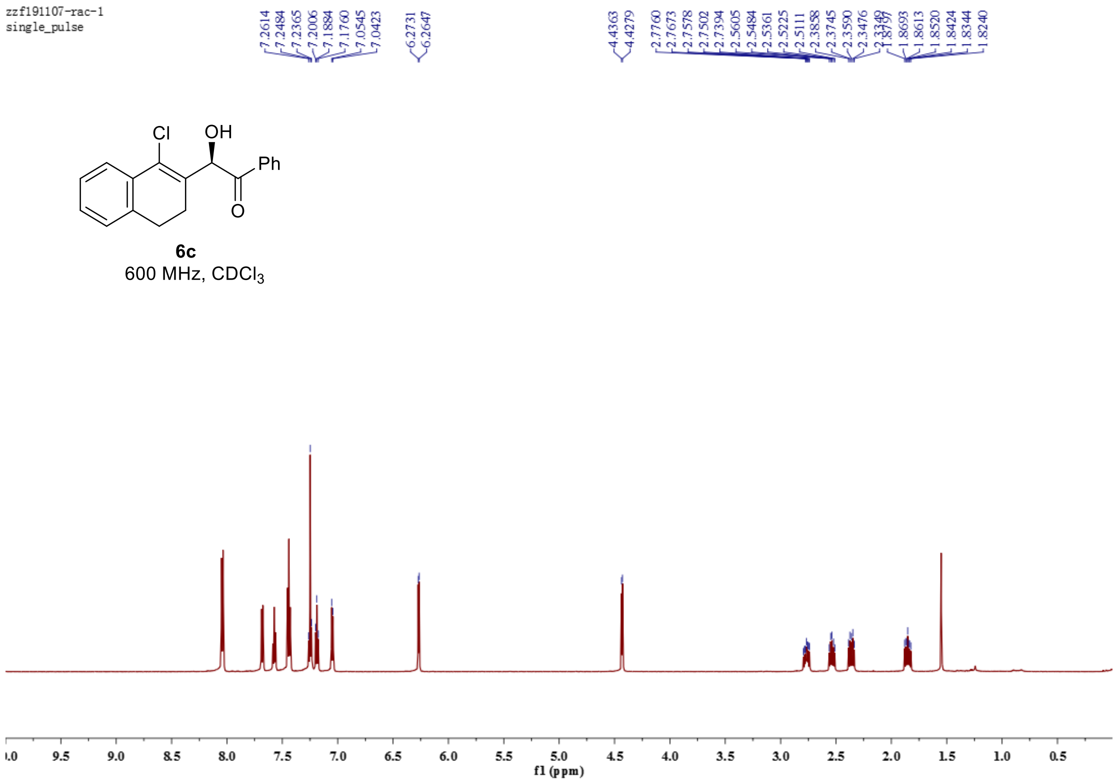

6c

600 MHz, $CDCl_3$

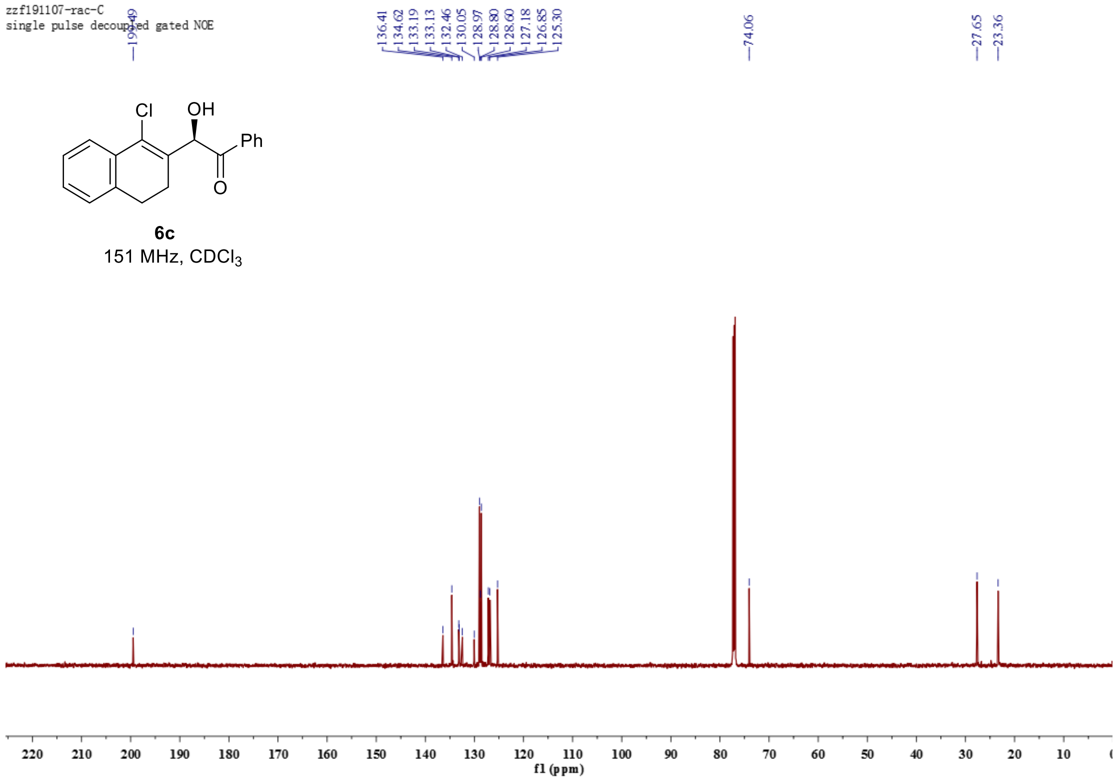

6c

151 MHz, $CDCl_3$

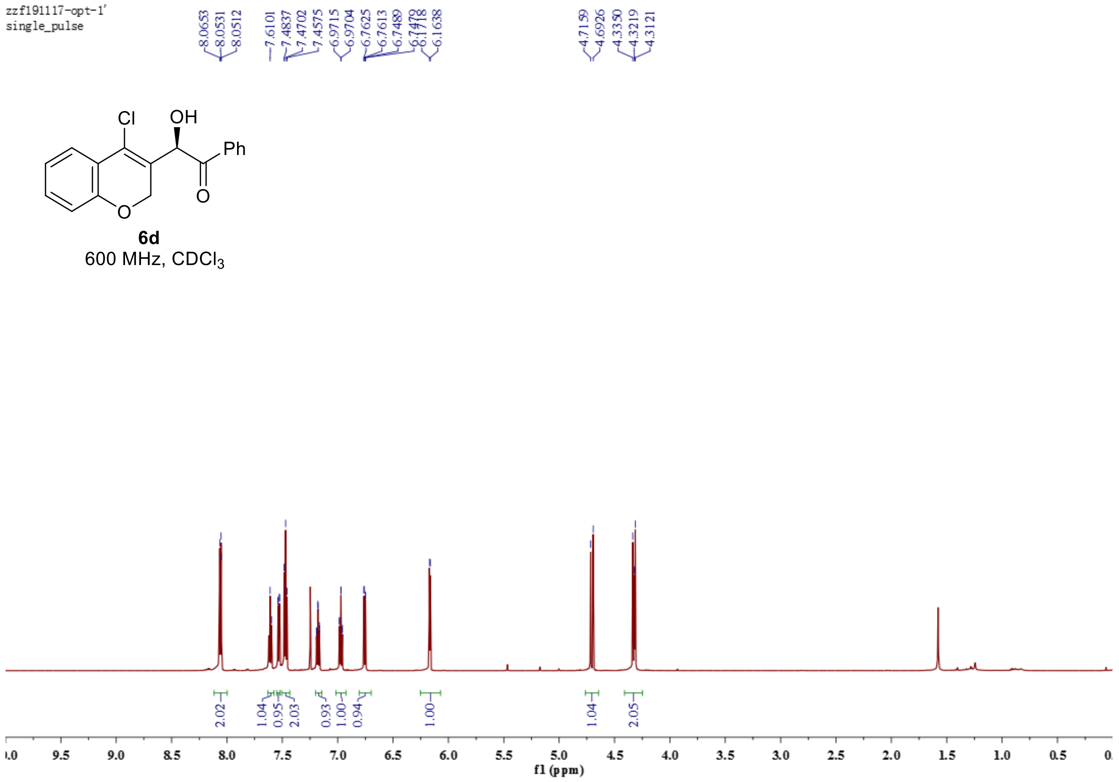
zzf191117-opt-1'
single_pulse
8.0653
8.0531
8.0512
7.6101
7.4837
7.4702
7.4575
6.9715
6.9704
6.7625
6.7613
6.7489
6.1638
4.7159
4.6926
4.3350
4.3219
4.3121
Cl
OH
Ph
O
O
6d
600 MHz, CDCl3
2.02
1.04
0.95
2.03
0.93
1.00
0.94
1.00
1.04
2.05
f1 (ppm)

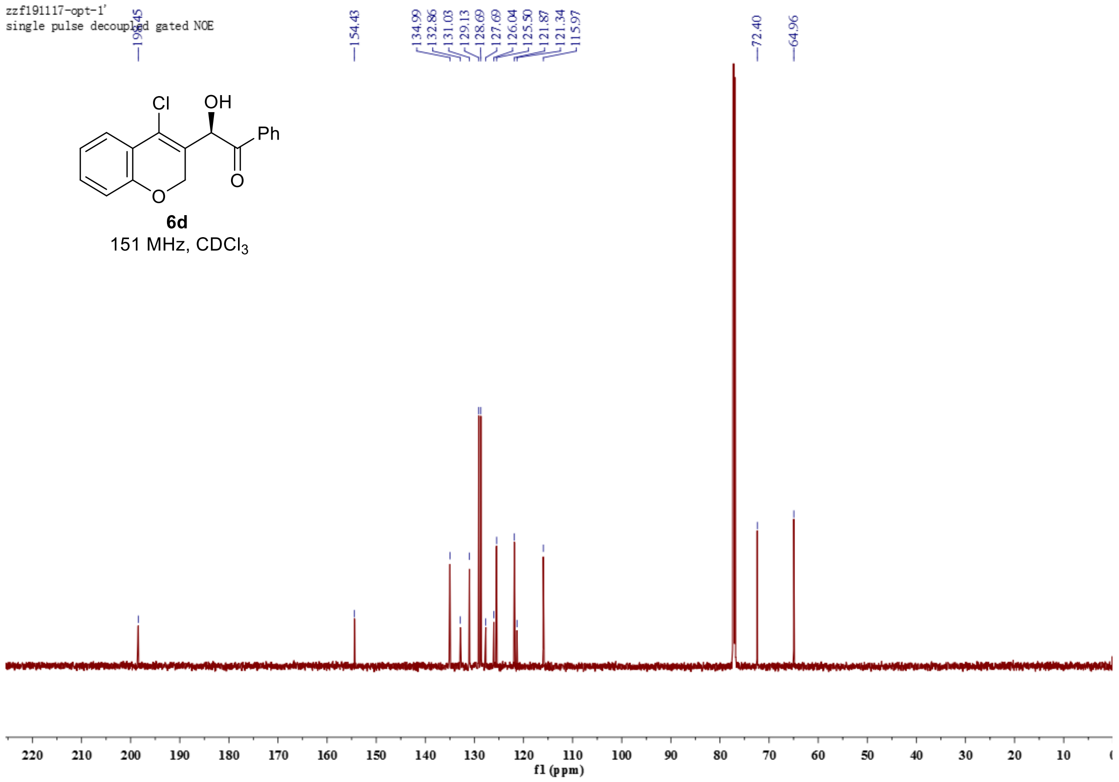
zzf191117-opt-1'
single pulse decoupled gated NOE
154.43
134.99
132.86
131.03
129.13
128.69
127.69
126.04
125.50
121.87
121.34
115.97
72.40
64.96
Cl
OH
Ph
O
O
6d
151 MHz, CDCl3
f1 (ppm)

DWN-20200808-3

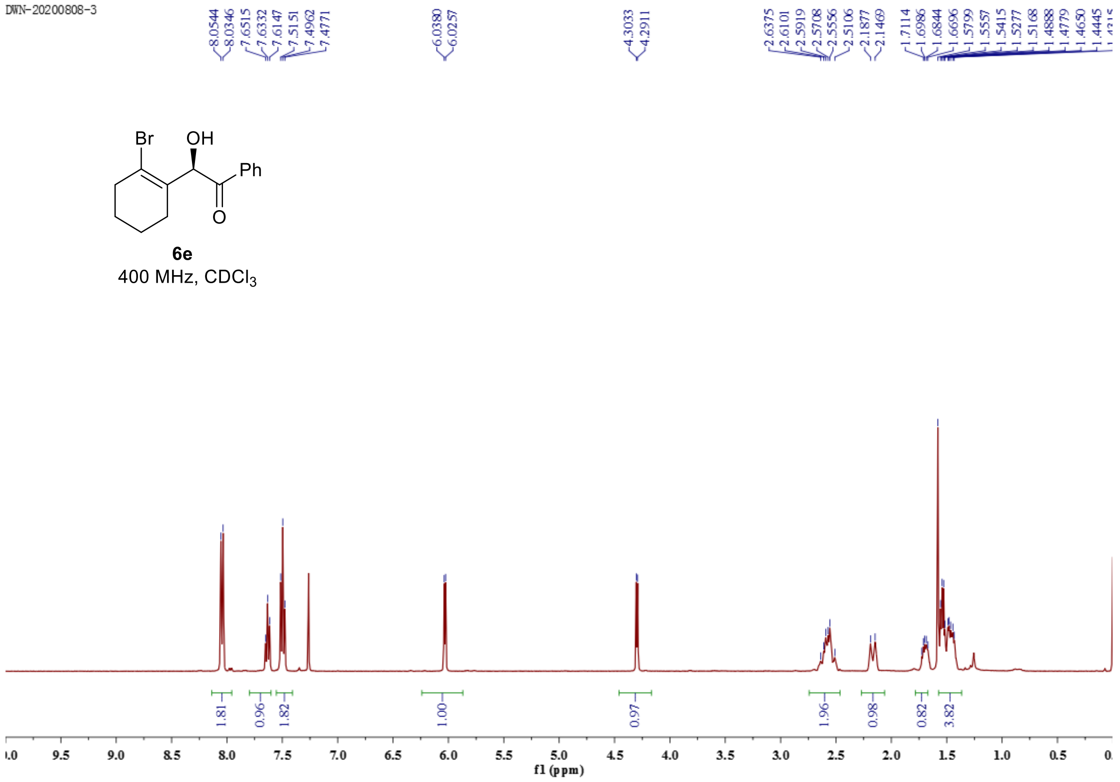

6e
400 MHz, $CDCl_3$

zzf191019-opt-1
single pulse decoupled gated NOE

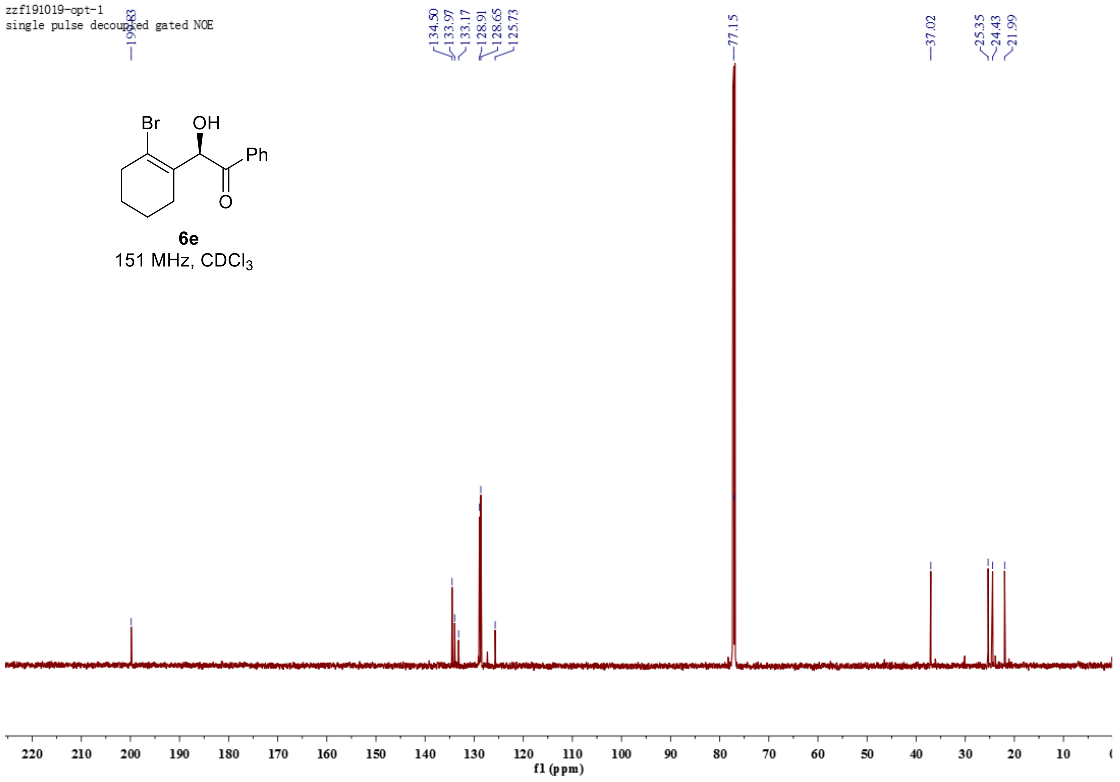

6e
151 MHz, $CDCl_3$

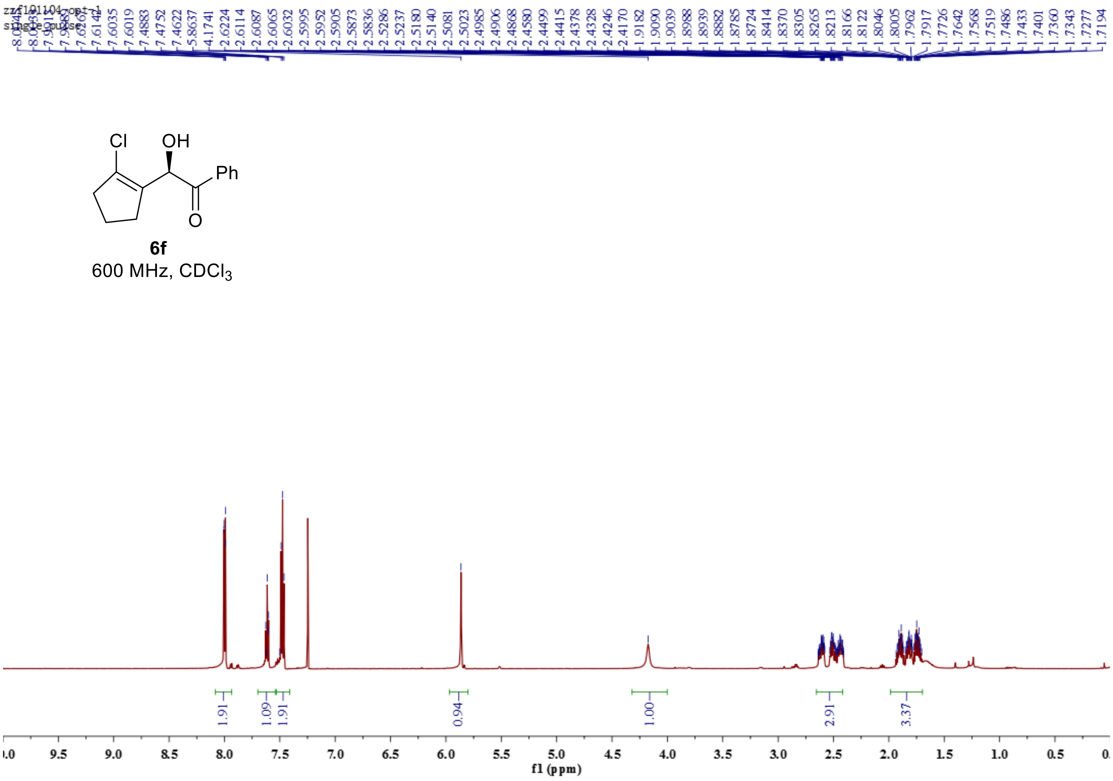
zzf191104-opt-1
single pulse
Cl
OH
Ph
O
6f
600 MHz, CDCl3
1.91
1.09
1.91
0.94
1.00
2.91
3.37
f1 (ppm)

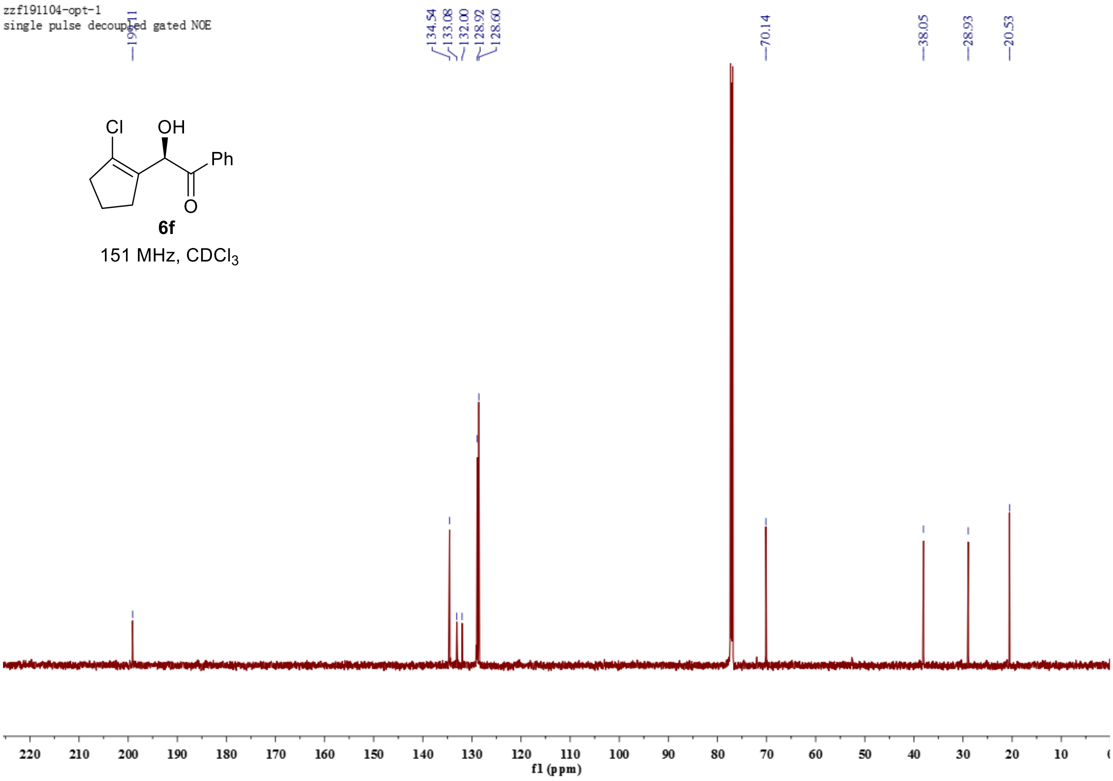
zzf191104-opt-1
single pulse decoupled gated NOE
199.11
134.54
133.08
132.00
128.92
128.60
70.14
38.05
28.93
20.53
Cl
OH
Ph
O
6f
151 MHz, CDCl3
f1 (ppm)

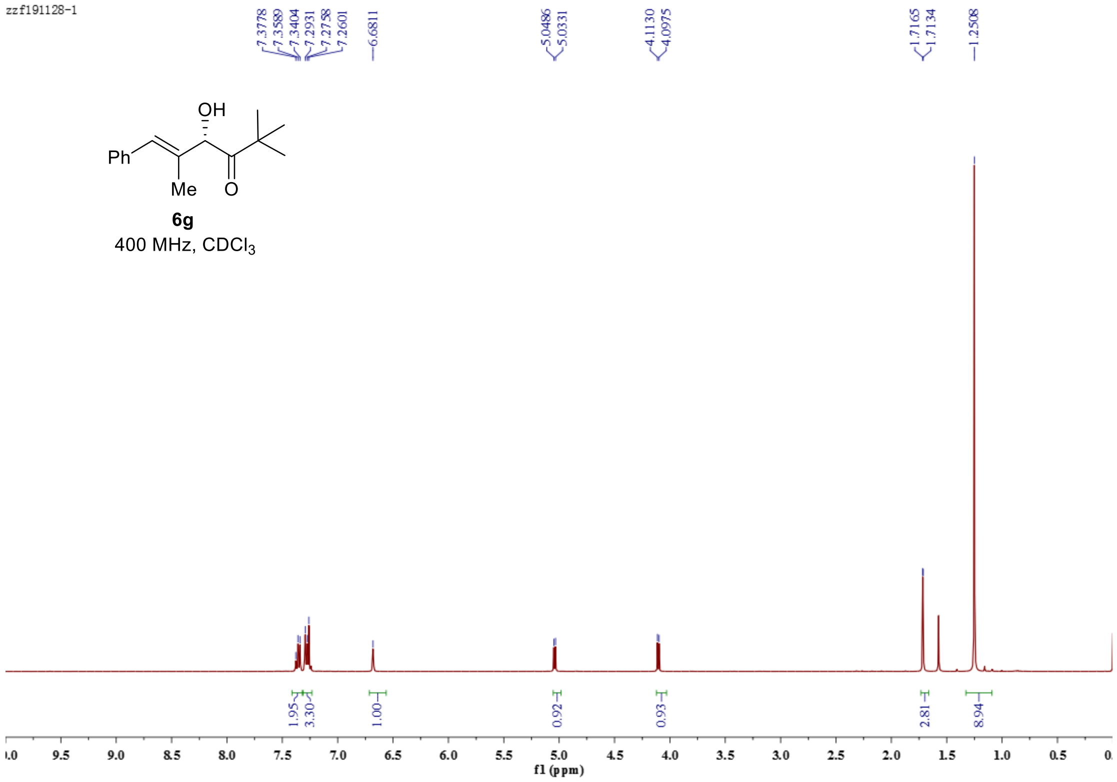
zzf191128-1
7.3778
7.3589
7.3404
7.2931
7.2758
7.2601
6.6811
5.0486
5.0331
4.1130
4.0975
1.7165
1.7134
1.2508
OH
Ph
Me
O
6g
400 MHz, CDCl3
1.95
3.30
1.00
0.92
0.93
2.81
8.94
f1 (ppm)

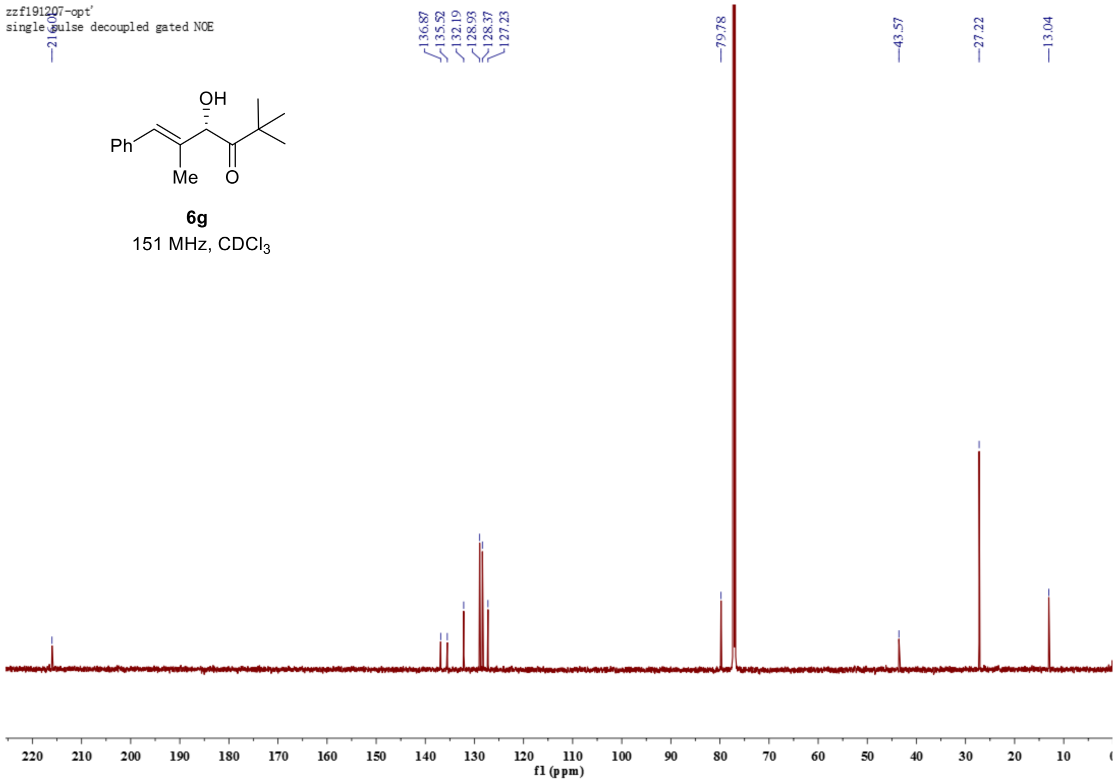
zzf191207-opt
single pulse decoupled gated NOE
136.87
135.52
132.19
128.93
128.37
127.23
79.78
43.57
27.22
13.04
OH
Ph
Me
O
6g
151 MHz, CDCl3
f1 (ppm)

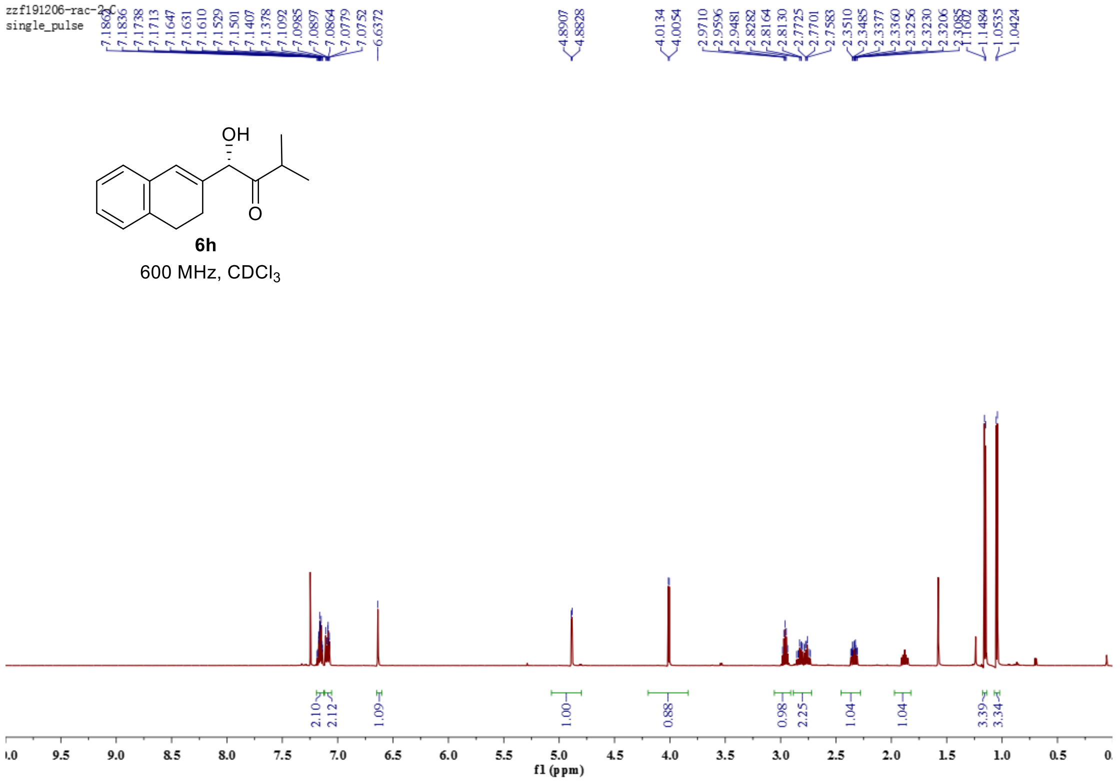
zzf191206-rac-2-C
single_pulse
OH
O
6h
600 MHz, CDCl3
f1 (ppm)

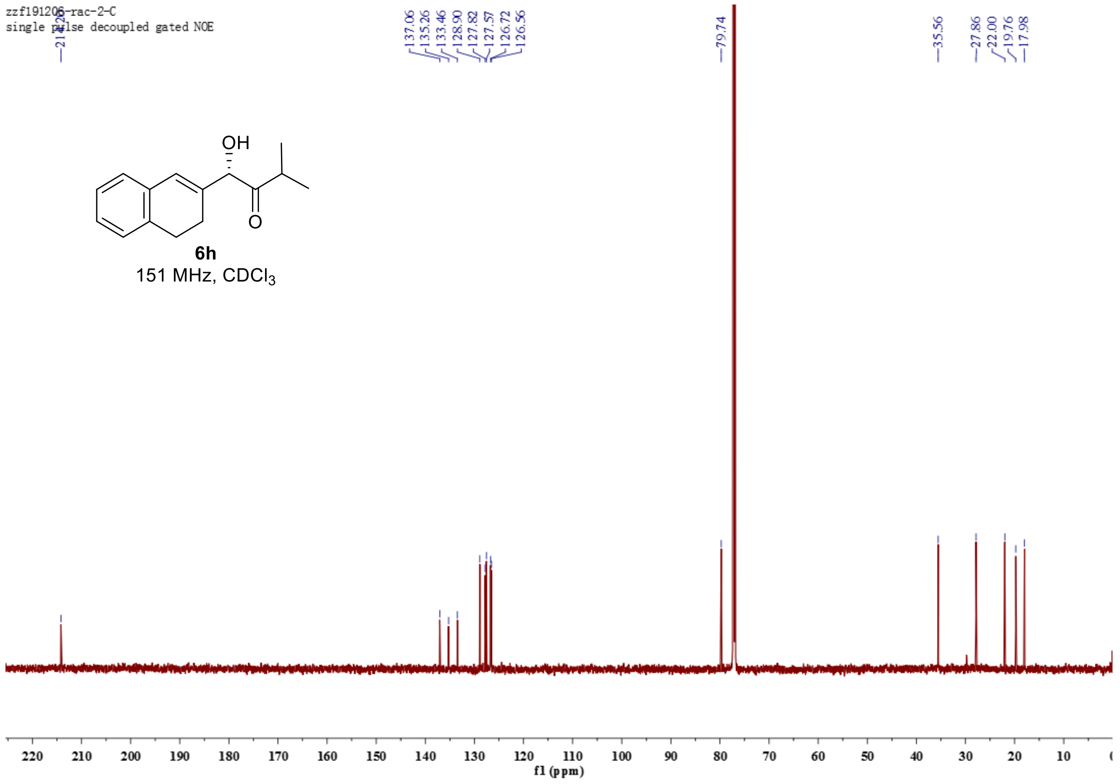
zzf191206-rac-2-C
single pulse decoupled gated NOE
137.06
135.26
133.46
128.90
127.82
127.57
126.72
126.56
79.74
35.56
27.86
22.00
19.76
17.98
OH
O
6h
151 MHz, CDCl3
f1 (ppm)

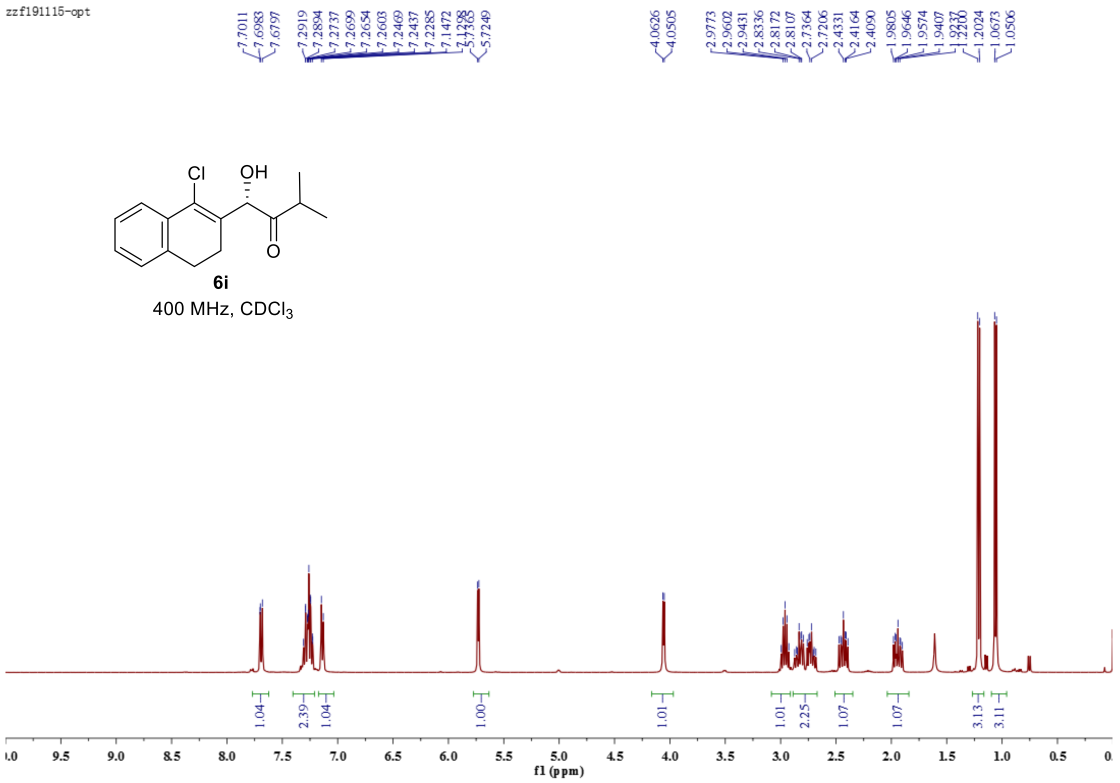
zzf191115-opt
7.7011
7.6983
7.6797
7.2919
7.2894
7.2737
7.2699
7.2654
7.2603
7.2469
7.2437
7.2285
7.1472
7.1258
5.7365
5.7249
4.0626
4.0505
2.9773
2.9602
2.9431
2.8336
2.8172
2.8107
2.7364
2.7206
2.4331
2.4164
2.4090
1.9805
1.9646
1.9574
1.9407
1.9237
1.2200
1.2024
1.0673
1.0506
Cl
OH
O
6i
400 MHz, CDCl3
1.04
2.39
1.04
1.00
1.01
1.01
2.25
1.07
1.07
3.13
3.11
f1 (ppm)

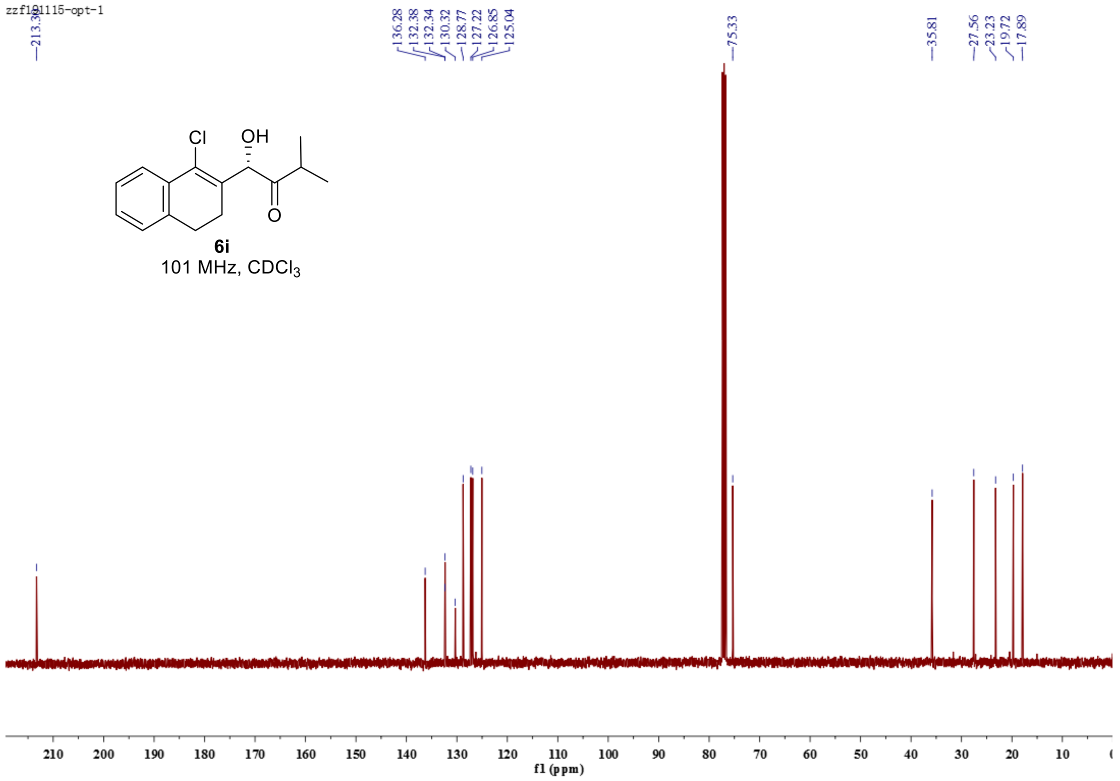
zzf191115-opt-1
213.38
136.28
132.38
132.34
130.32
128.77
127.22
126.85
125.04
75.33
35.81
27.56
23.23
19.72
17.89
Cl
OH
O
6i
101 MHz, CDCl3
f1 (ppm)

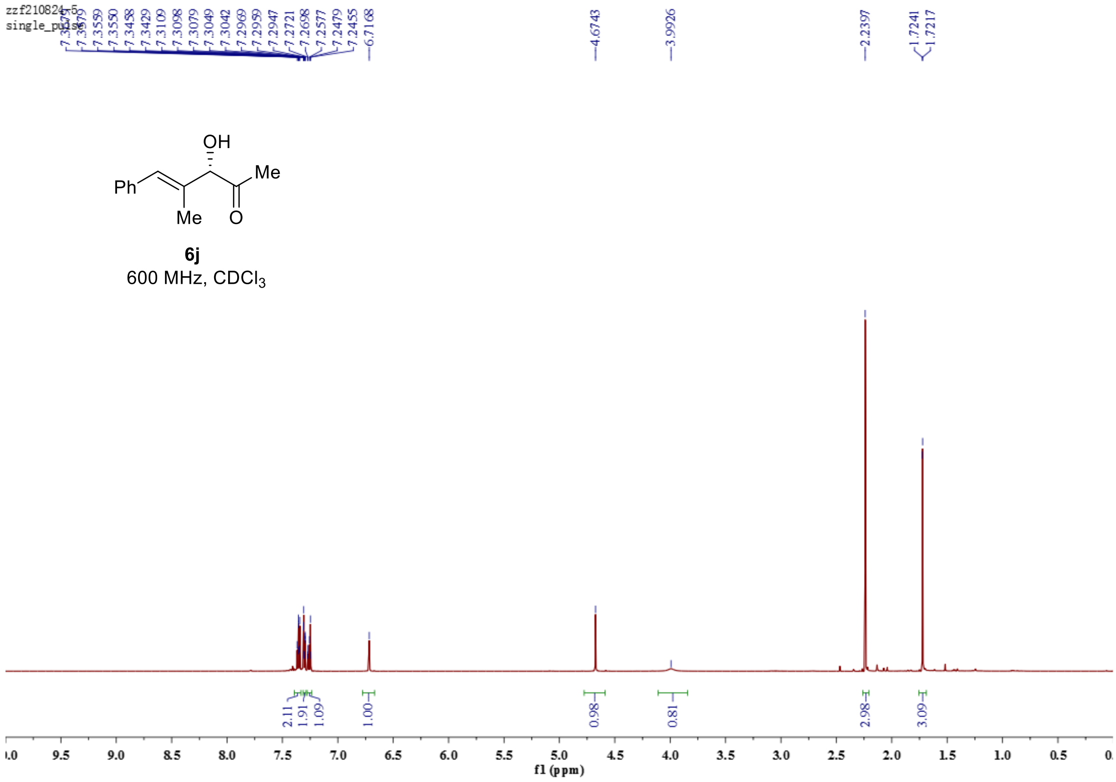
zzf210824-5
single_pulse
7.3559
7.3550
7.3458
7.3429
7.3109
7.3098
7.3079
7.3049
7.3042
7.2969
7.2959
7.2947
7.2721
7.2698
7.2577
7.2479
7.2455
6.7168
4.6743
3.9926
2.2397
1.7241
1.7217
OH
Ph
Me
Me
O
6j
600 MHz, CDCl3
2.11
1.91
1.09
1.00
0.98
0.81
2.98
3.09
f1 (ppm)

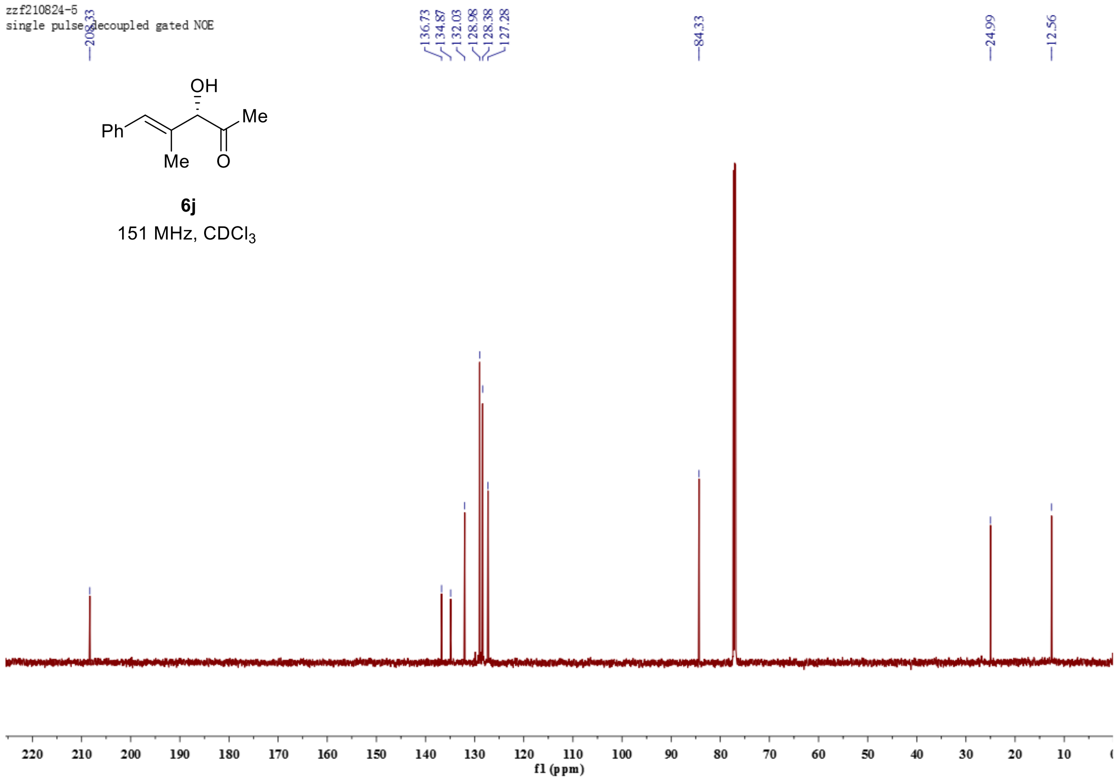
zzf210824-5
single pulse decoupled gated NOE
136.73
134.87
132.03
128.98
128.38
127.28
84.33
24.99
12.56
OH
Ph
Me
Me
O
6j
151 MHz, CDCl3
f1 (ppm)

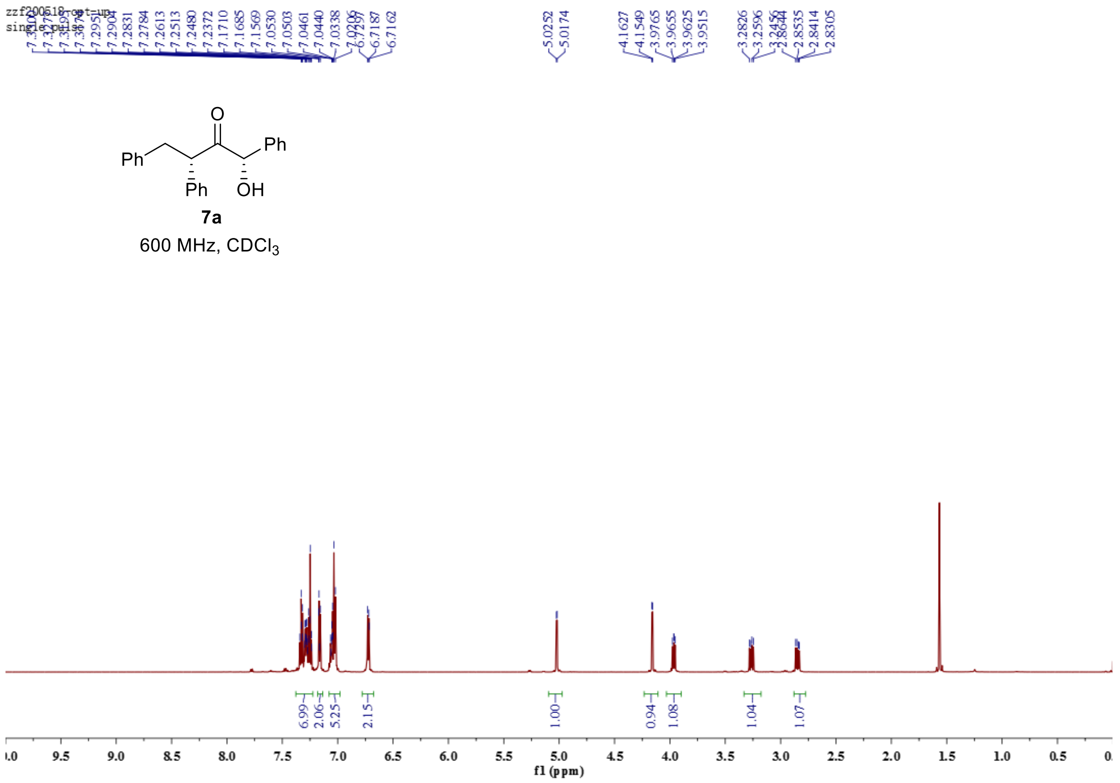

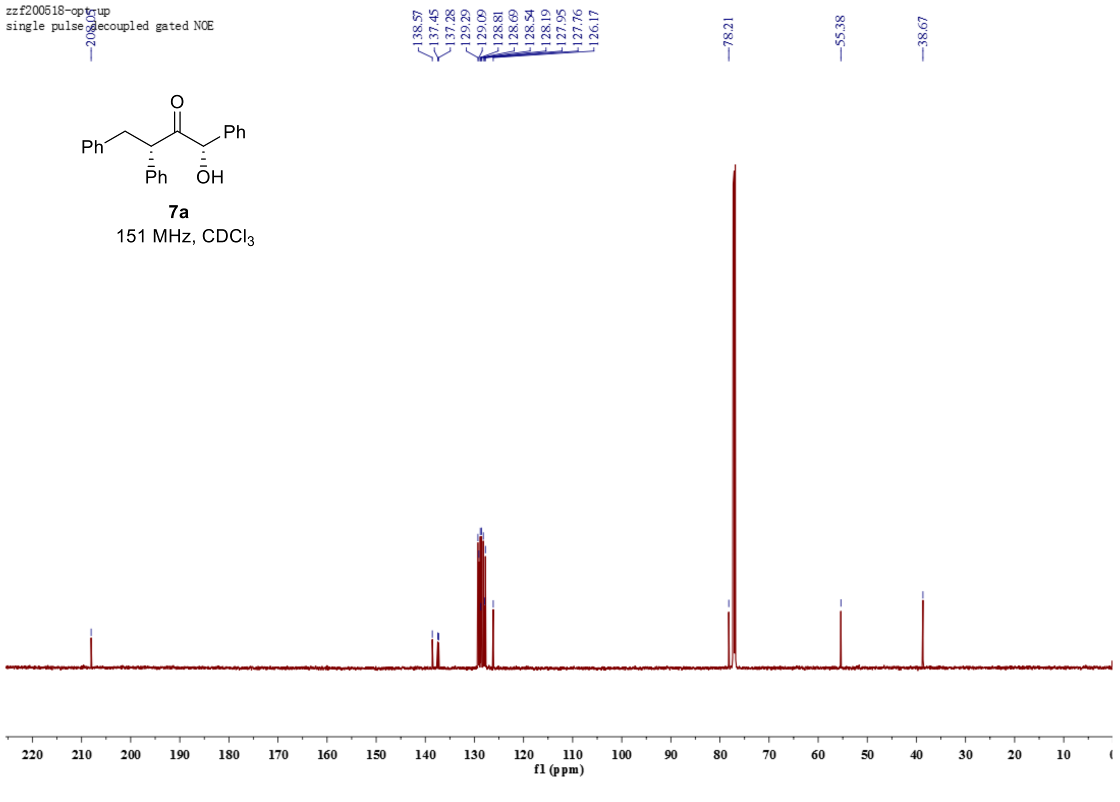

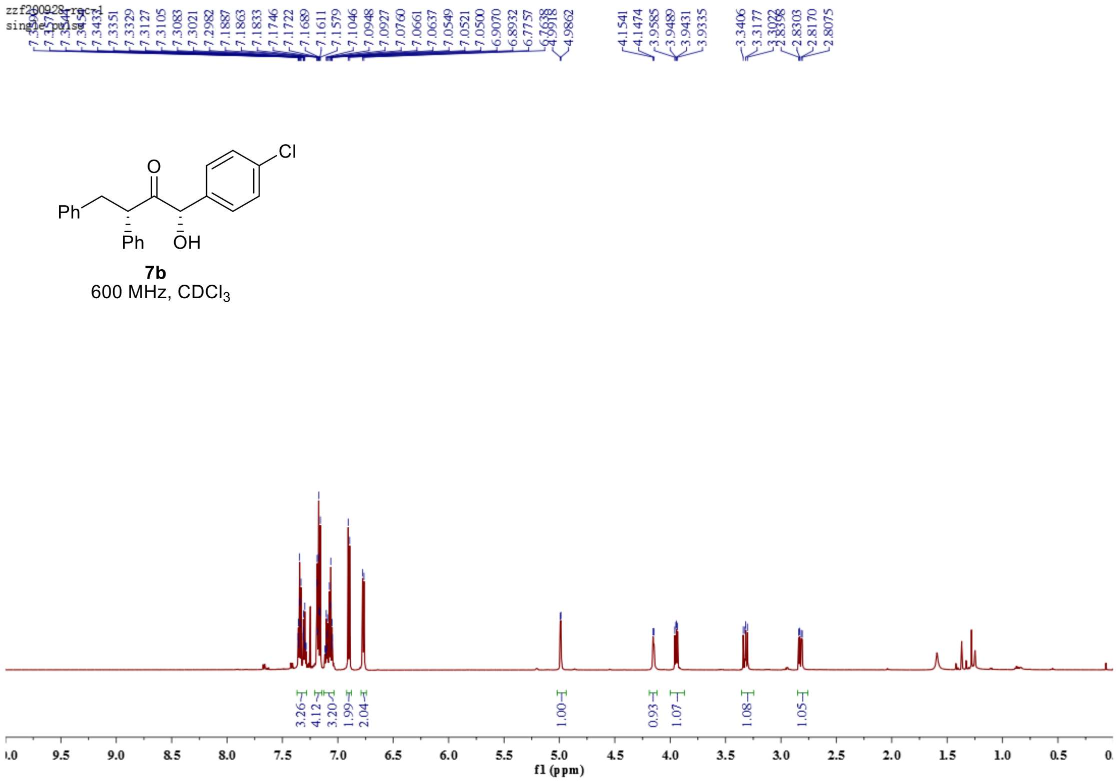

7b
600 MHz, $CDCl_3$

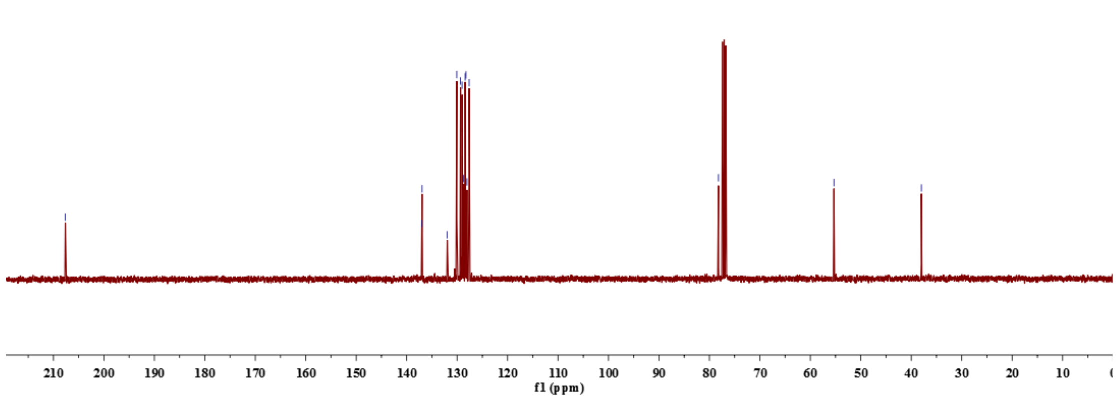

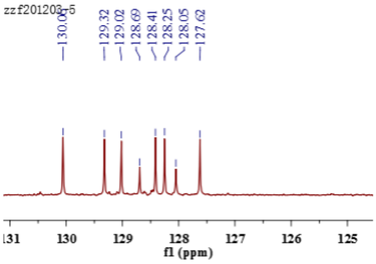

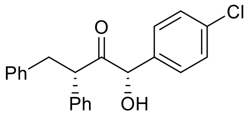

7b
101 MHz, $CDCl_3$

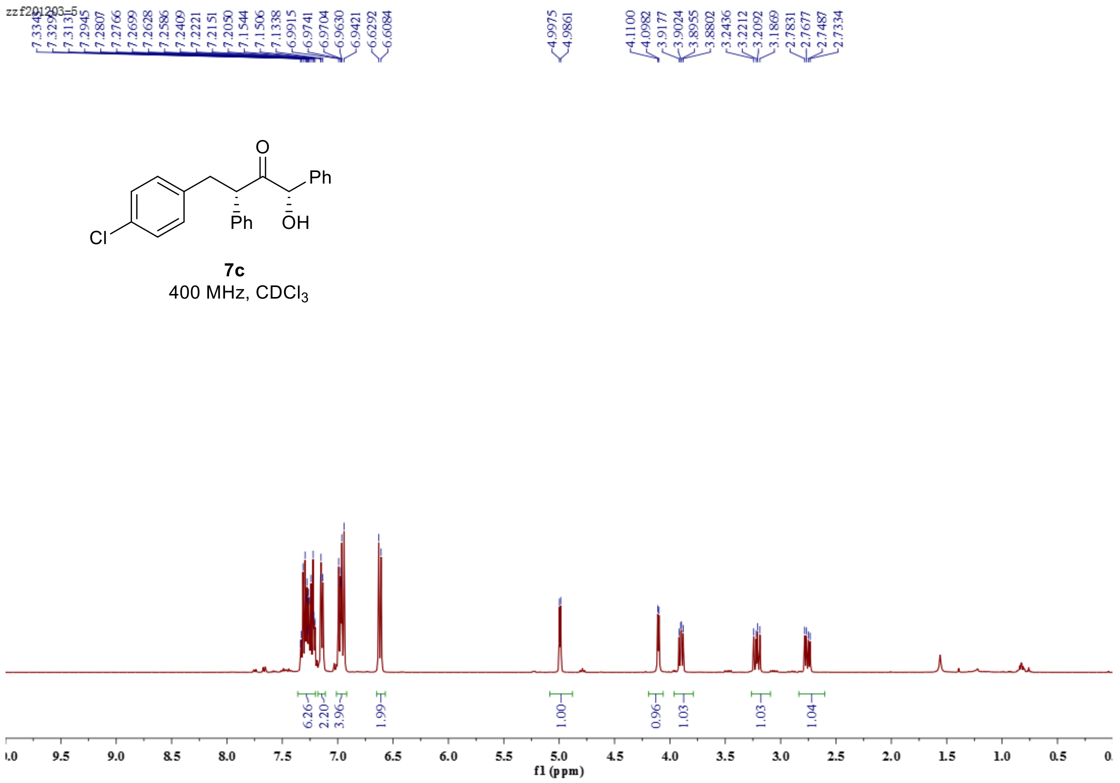

7c
400 MHz, $CDCl_3$

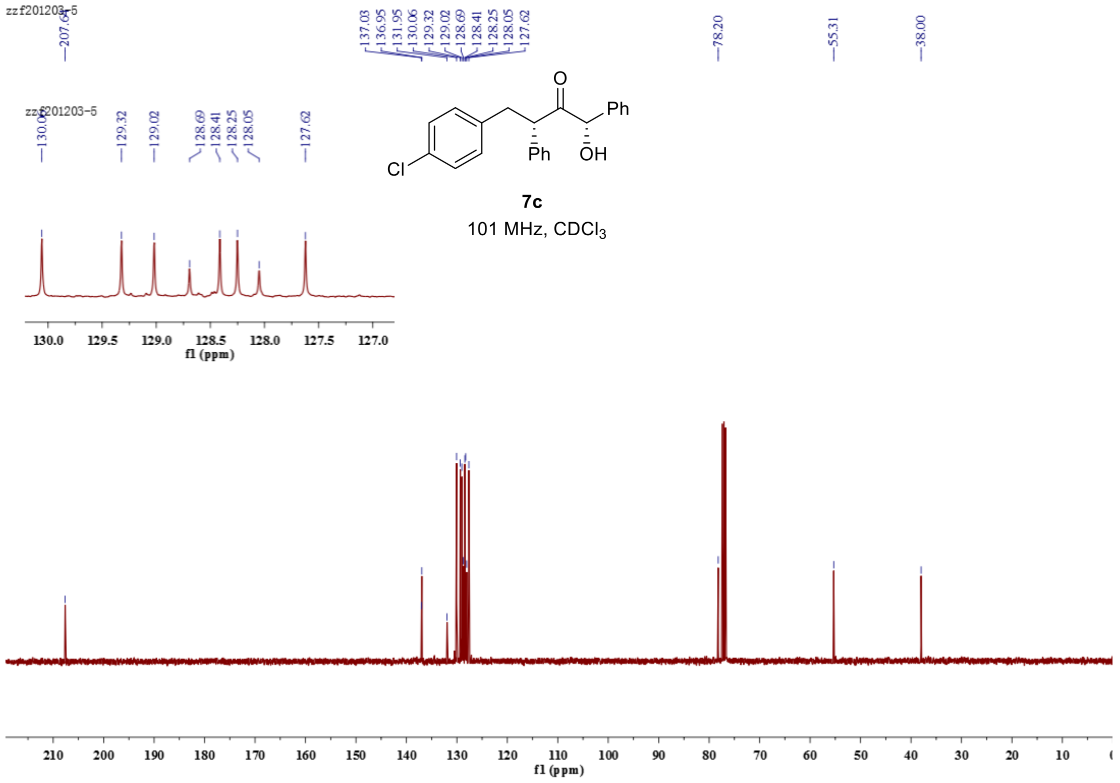

7c
101 MHz, $CDCl_3$

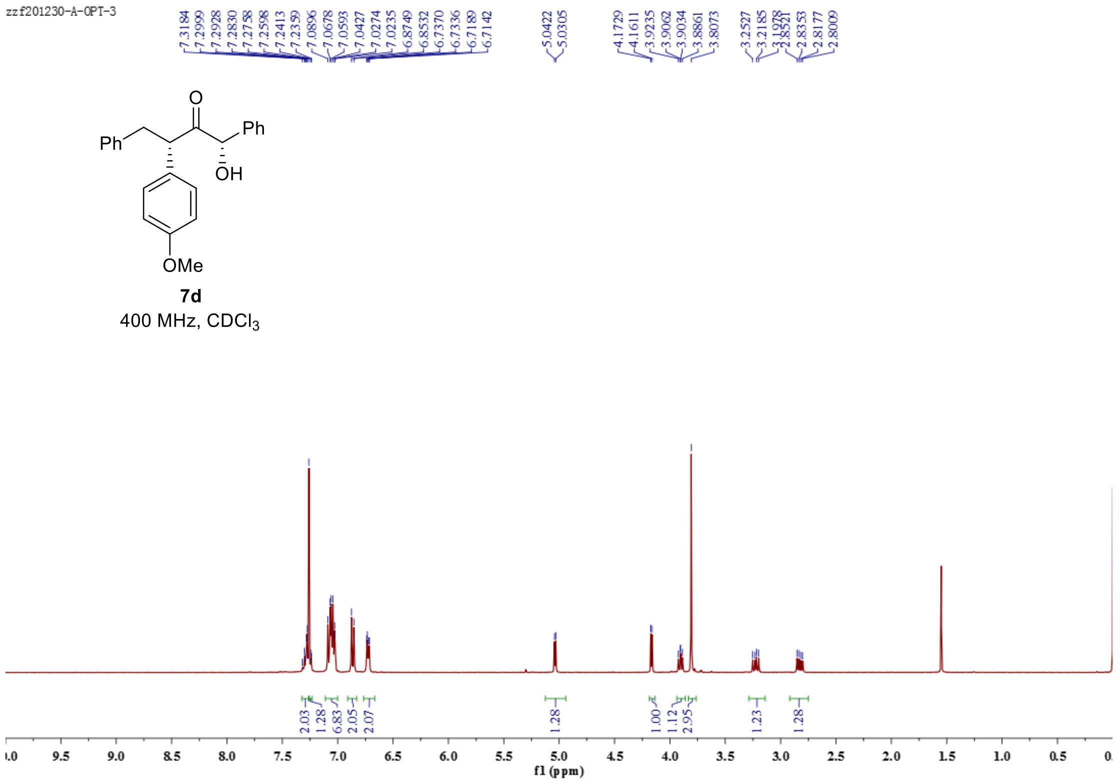
zzf201230-A-OPT-3
7d
400 MHz, CDCl3
f1 (ppm)

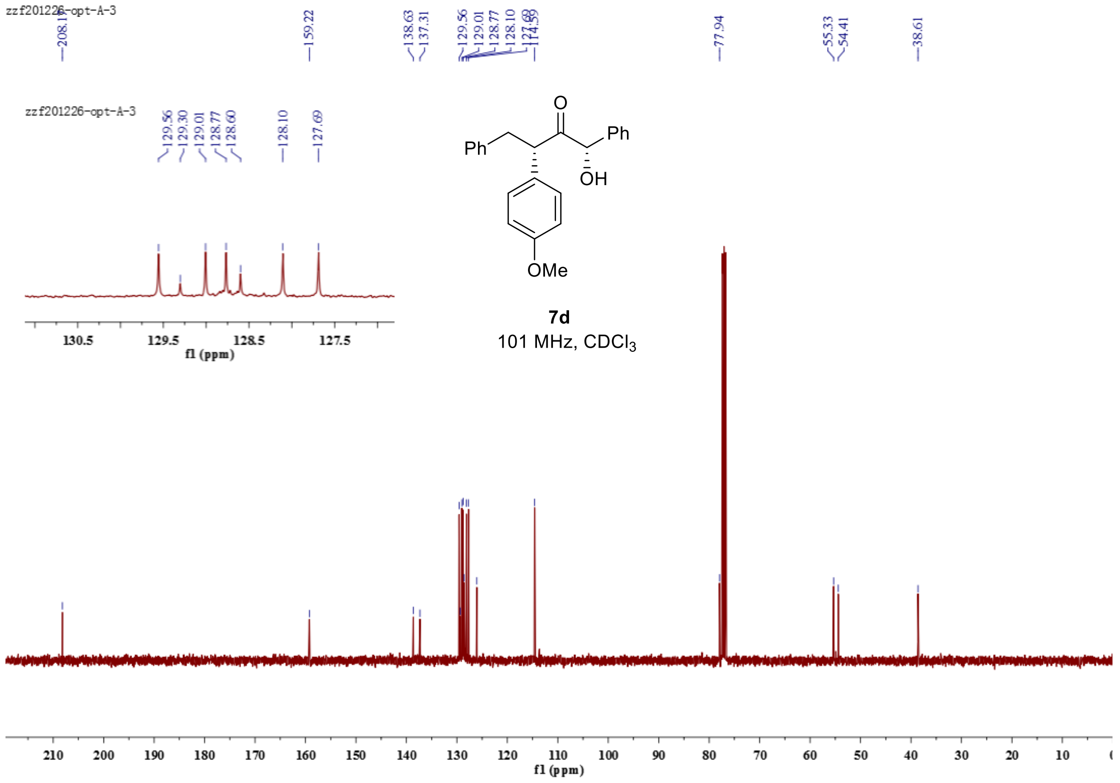
zzf201226-opt-A-3
208.17
159.22
138.63
137.31
129.56
129.01
128.77
128.10
127.69
114.59
77.94
55.33
54.41
38.61
7d
101 MHz, CDCl3
f1 (ppm)

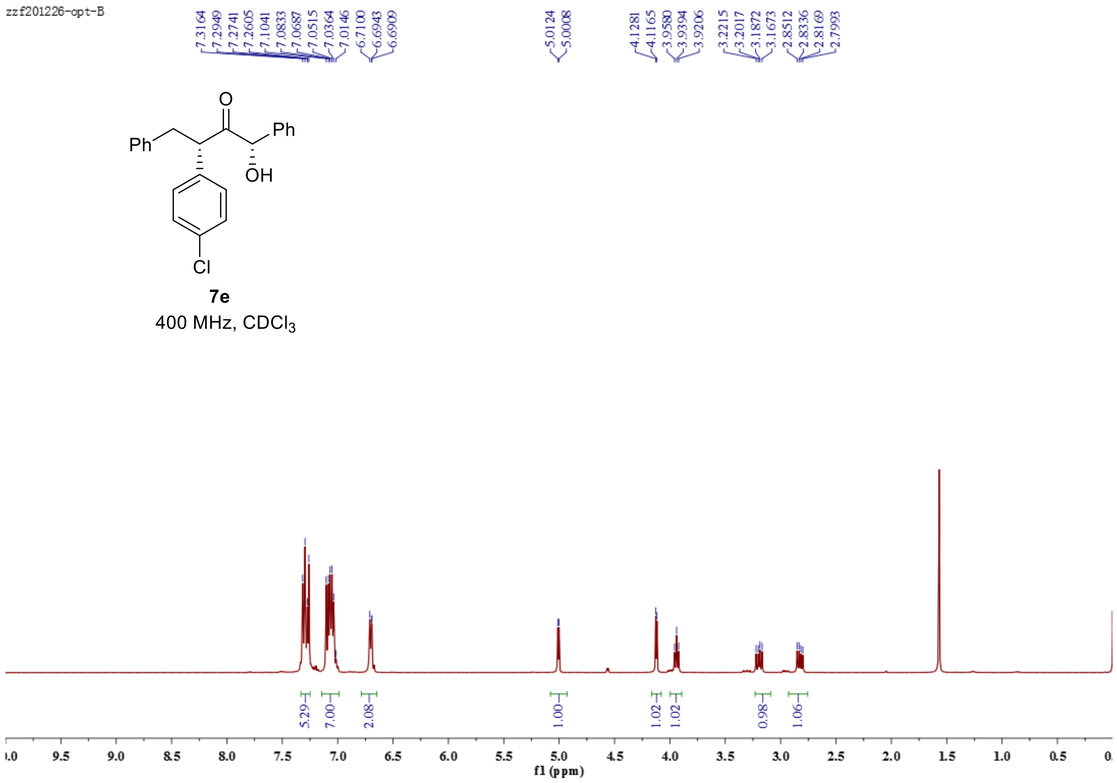
zzf201226-opt-B
7.3164 7.2949 7.2741 7.2605 7.1041 7.0833 7.0687 7.0515 7.0364 7.0146 6.7100 6.6943 6.6909
5.0124 5.0008
4.1281 4.1165 3.9580 3.9394 3.9206
3.2215 3.2017 3.1872 3.1673 2.8512 2.8336 2.8169 2.7993
Ph
O
Ph
OH
Cl
7e
400 MHz, CDCl3
5.29 7.00 2.08 1.00 1.02 1.02 0.98 1.06
f1 (ppm)

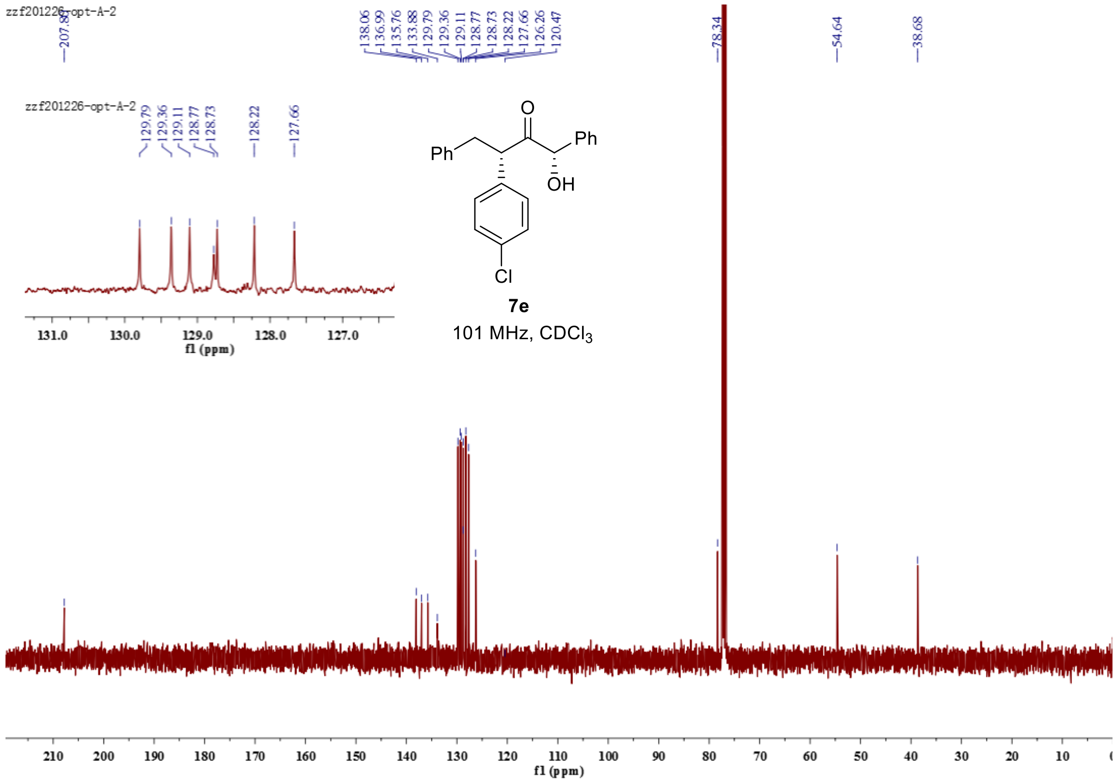
zzf201226-opt-A-2
207.87
138.06 136.99 135.76 133.88 129.79 129.36 129.11 128.77 128.73 128.22 127.66 126.26 120.47
78.34
54.64
38.68
zzf201226-opt-A-2
129.79 129.36 129.11 128.77 128.73 128.22 127.66
f1 (ppm)
Ph
O
Ph
OH
Cl
7e
101 MHz, CDCl3
f1 (ppm)

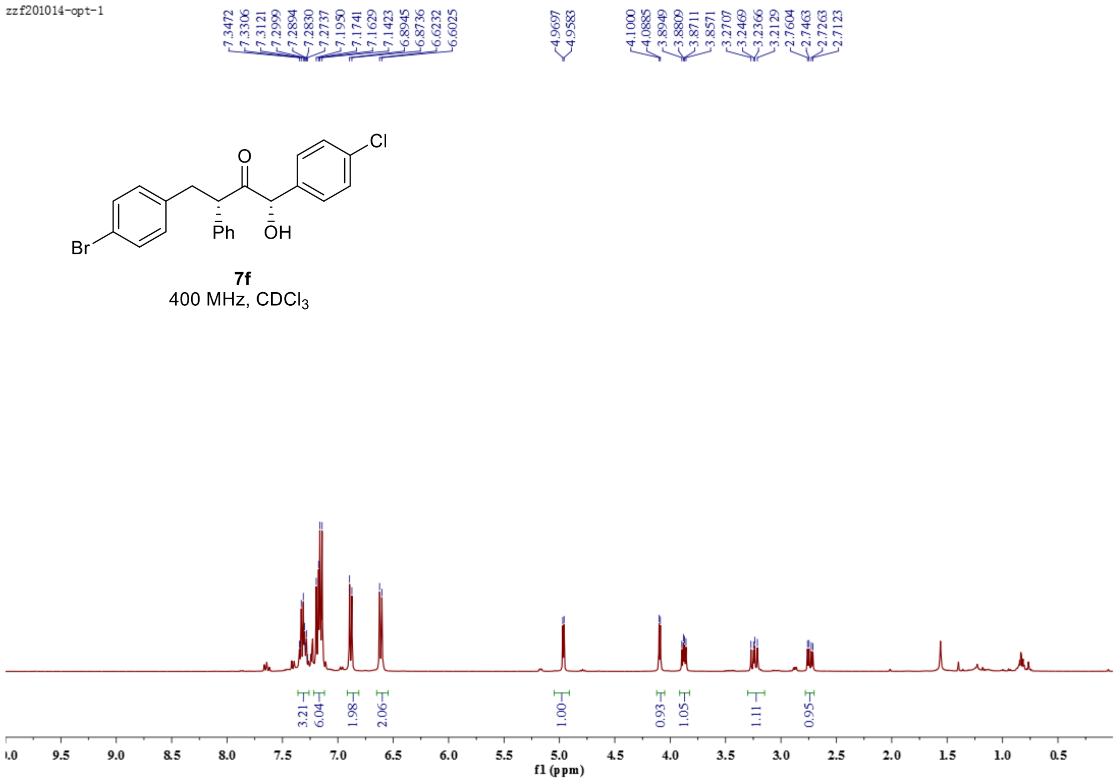
zzf201014-opt-1
7.3472 7.3306 7.3121 7.2999 7.2894 7.2830 7.2737 7.1950 7.1741 7.1629 7.1423 6.8945 6.8736 6.6232 6.6025
4.9697 4.9583
4.1000 4.0885 3.8949 3.8809 3.8711 3.8571 3.2707 3.2469 3.2366 3.2129 2.7604 2.7463 2.7263 2.7123
O
Cl
Br
Ph
OH
7f
400 MHz, CDCl3
3.21 6.04 1.98 2.06 1.00 0.93 1.05 1.11 0.95
f1 (ppm)

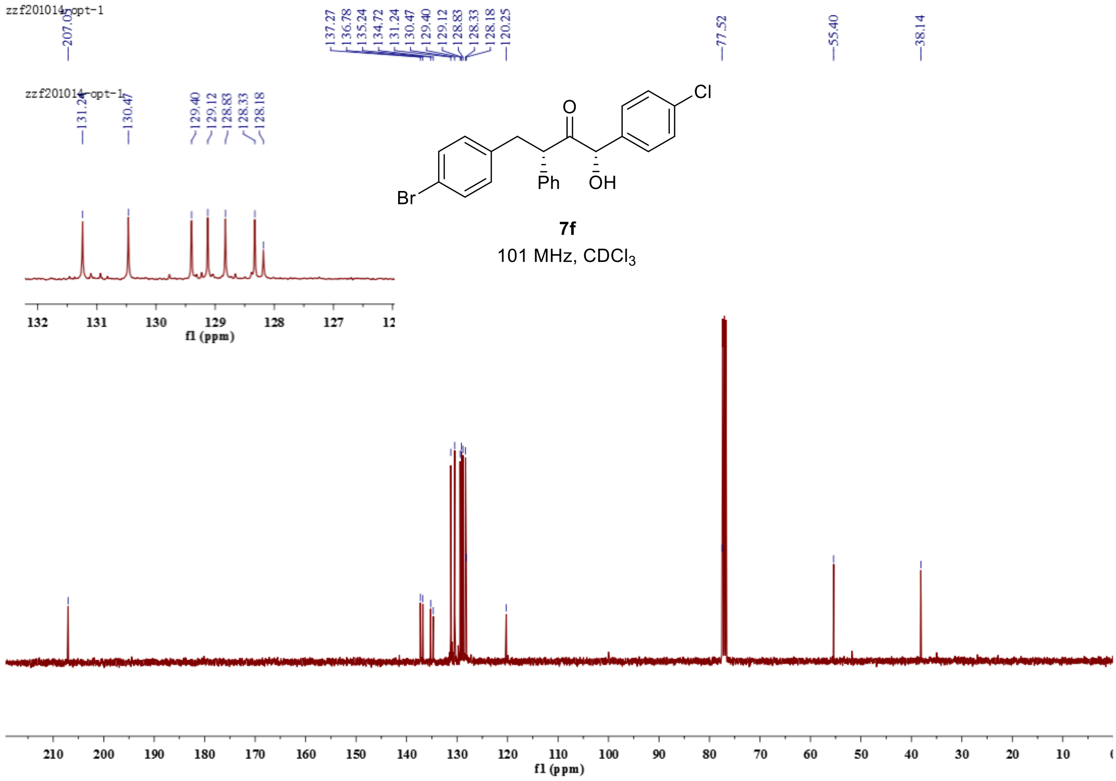
zzf201014-opt-1
207.05
137.27 136.78 135.24 134.72 131.24 130.47 129.40 129.12 128.83 128.33 128.18 120.25
77.52
55.40
38.14
zzf201014-opt-1
131.24 130.47 129.40 129.12 128.83 128.33 128.18
O
Cl
Br
Ph
OH
7f
101 MHz, CDCl3
f1 (ppm)

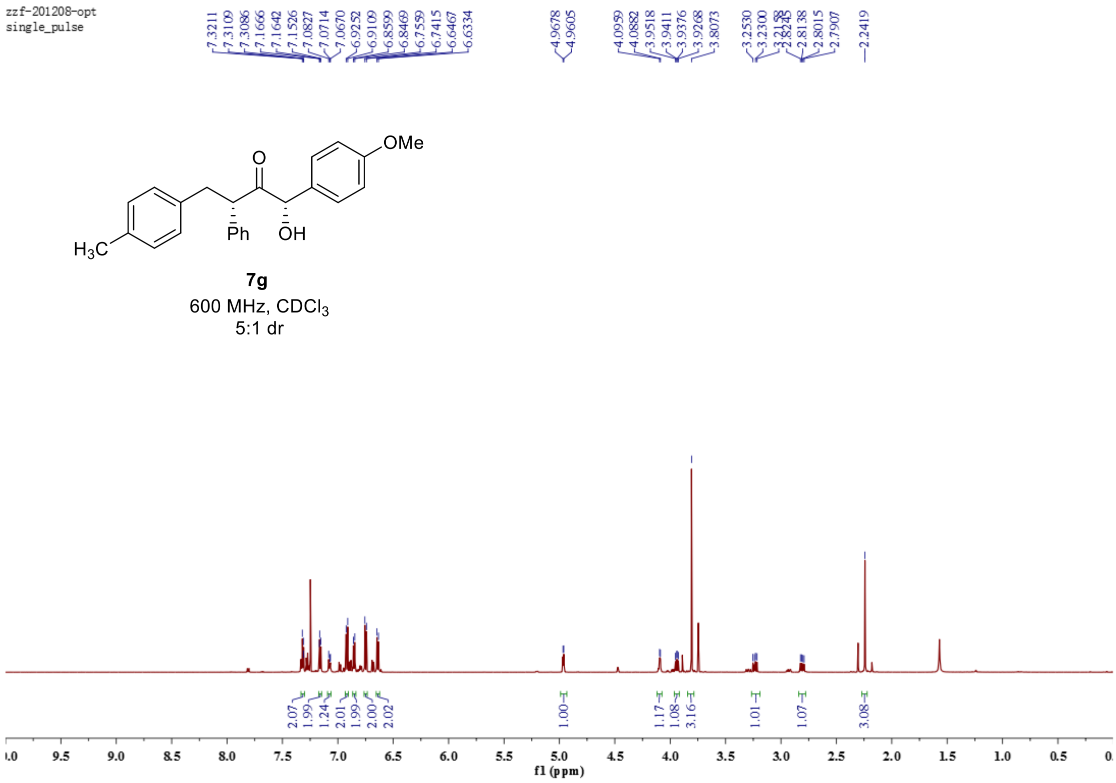

zzf-201208-opt
single_pulse
7.3211 7.3109 7.3086 7.1666 7.1642 7.1526 7.0827 7.0714 7.0670 6.9252 6.9109 6.8599 6.8469 6.7559 6.7415 6.6467 6.6334
4.9678 4.9605
4.0959 4.0882 3.9518 3.9411 3.9376 3.9268 3.8073
3.2530 3.2300 3.2158 2.8245 2.8138 2.8015 2.7907
2.2419
OMe
O
H3C
Ph
OH
7g
600 MHz, CDCl3
5:1 dr
2.07 1.99 1.24 2.01 1.99 2.00 2.02
1.00
1.17 1.08 3.16
1.01
1.07
3.08
f1 (ppm)

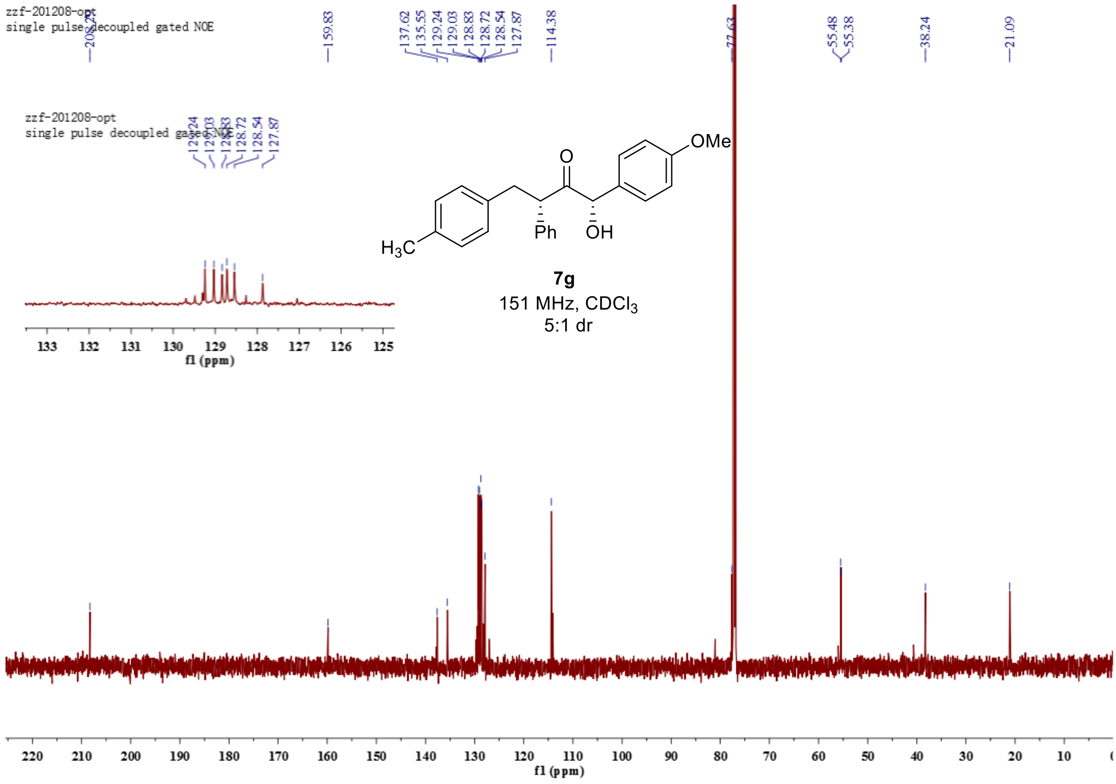

zzf-201208-opt
single pulse decoupled gated NOE
208.28
159.83
137.62 135.55 129.24 129.03 128.83 128.72 128.54 127.87
114.38
77.63
55.48 55.38
38.24
21.09
zzf-201208-opt
single pulse decoupled gated NOE
129.24 129.03 128.83 128.72 128.54 127.87
f1 (ppm)
OMe
O
H3C
Ph
OH
7g
151 MHz, CDCl3
5:1 dr
f1 (ppm)

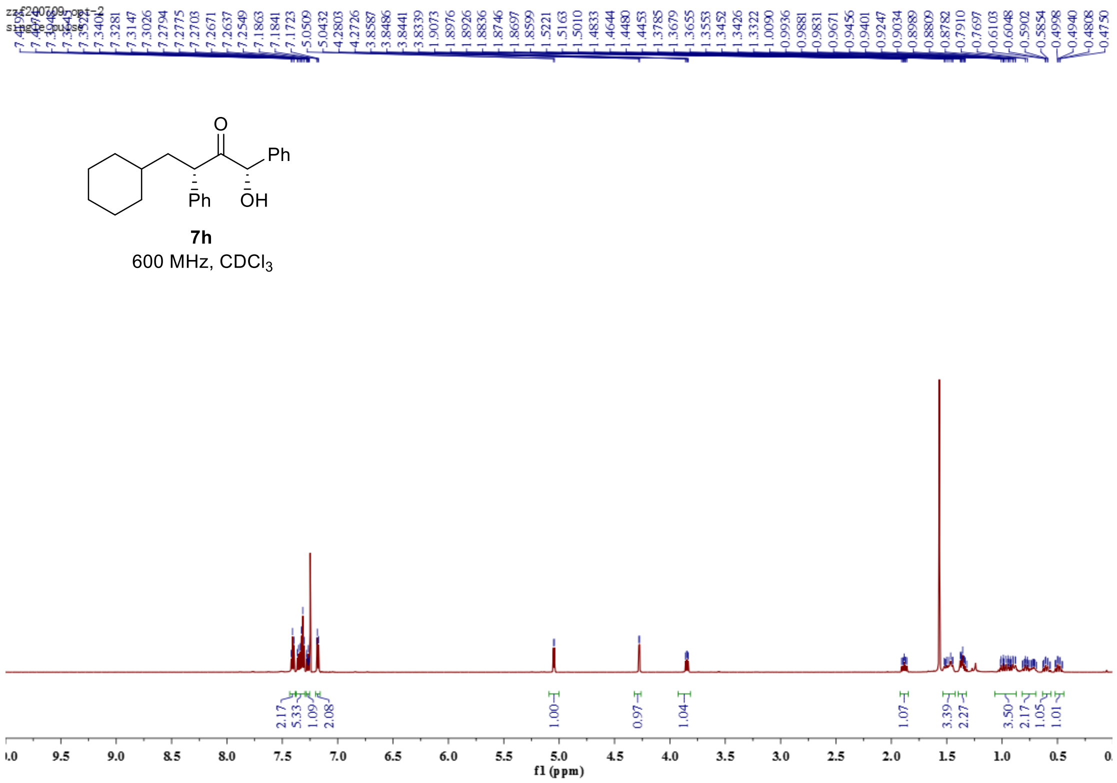
zzf200709-opt-2
single_pulse
7.3404
7.3281
7.3147
7.3026
7.2794
7.2775
7.2703
7.2671
7.2637
7.2549
7.1863
7.1841
7.1723
5.0509
5.0432
4.2803
4.2726
3.8587
3.8486
3.8441
3.8339
1.9073
1.8976
1.8926
1.8836
1.8746
1.8697
1.8599
1.5221
1.5163
1.5010
1.4833
1.4644
1.4480
1.4453
1.3785
1.3679
1.3655
1.3553
1.3452
1.3426
1.3322
1.0090
0.9936
0.9881
0.9831
0.9671
0.9456
0.9401
0.9247
0.9034
0.8989
0.8809
0.8782
0.7910
0.7697
0.6103
0.6048
0.5902
0.5854
0.4998
0.4940
0.4808
0.4750
O
Ph
Ph
OH
7h
600 MHz, CDCl3
2.17
5.33
1.09
2.08
1.00
0.97
1.04
1.07
3.39
2.27
3.50
2.17
1.05
1.01
f1 (ppm)

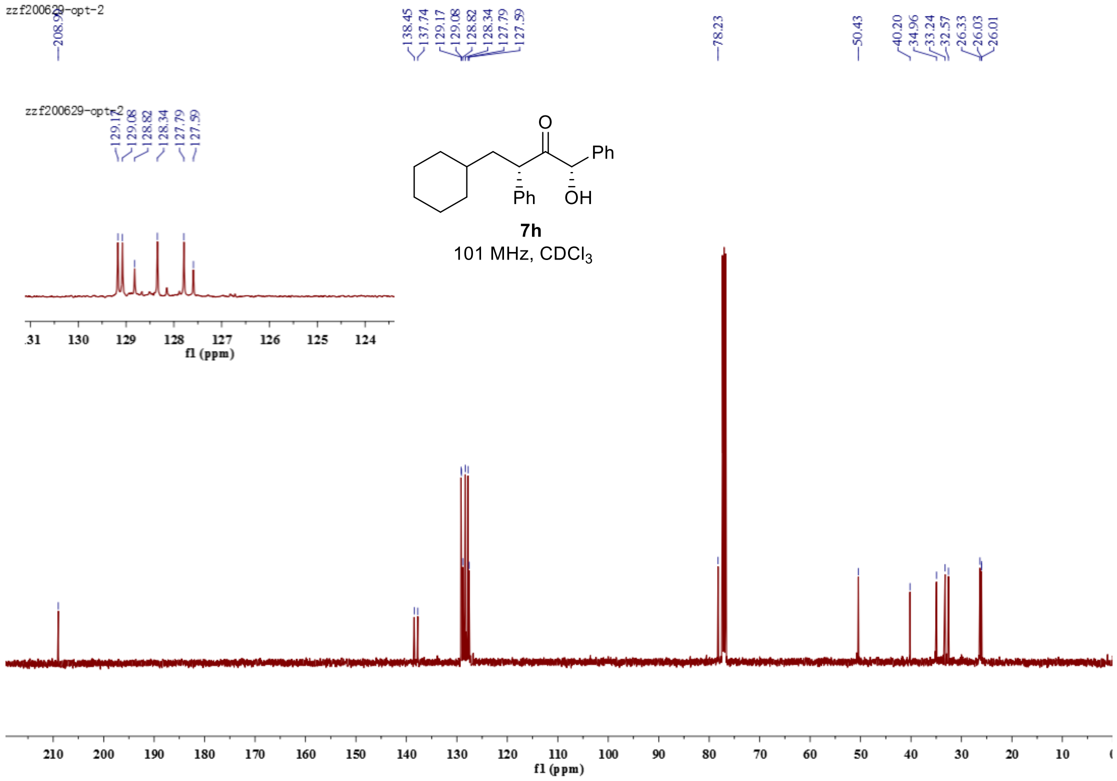
zzf200629-opt-2
208.96
138.45
137.74
129.17
129.08
128.82
128.34
127.79
127.59
78.23
50.43
40.20
34.96
33.24
32.57
26.33
26.03
26.01
zzf200629-opt-2
129.17
129.08
128.82
128.34
127.79
127.59
f1 (ppm)
O
Ph
Ph
OH
7h
101 MHz, CDCl3
f1 (ppm)

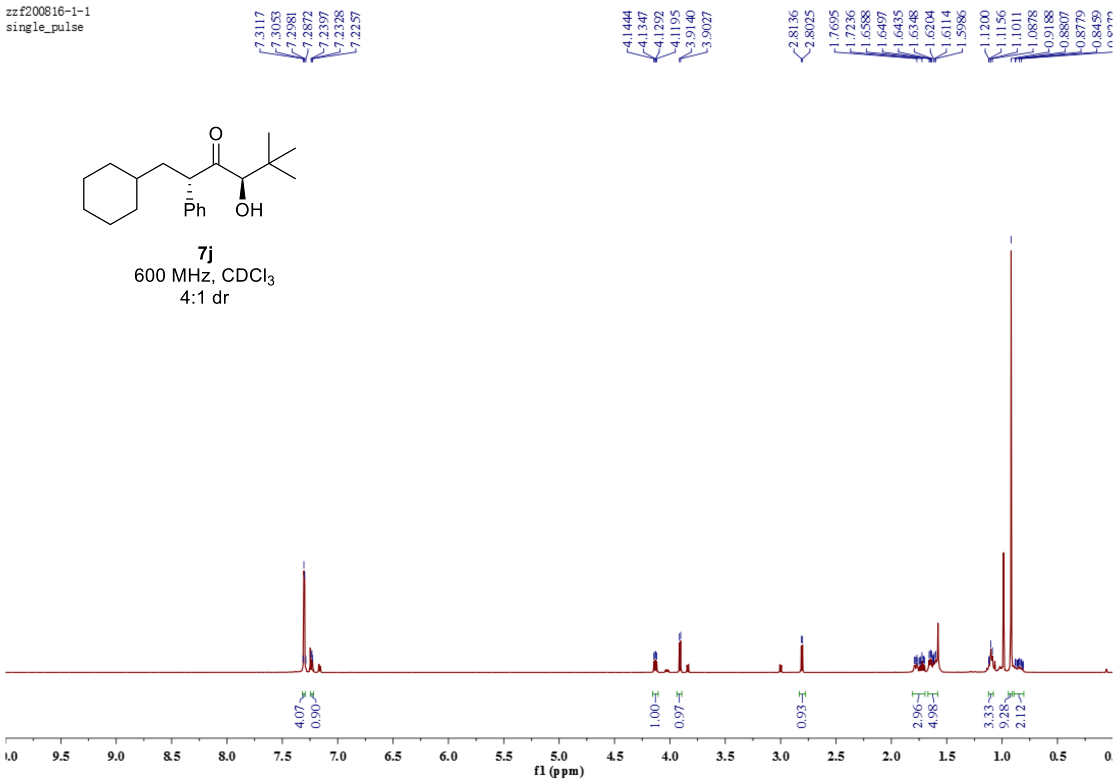
zzf200816-1-1
single_pulse
7.3117
7.3053
7.2981
7.2872
7.2397
7.2328
7.2257
4.1444
4.1347
4.1292
4.1195
3.9140
3.9027
2.8136
2.8025
1.7695
1.7236
1.6588
1.6497
1.6435
1.6348
1.6204
1.6114
1.5986
1.1200
1.1156
1.1011
1.0878
0.9188
0.8807
0.8779
0.8459
Ph
OH
O
7j
600 MHz, CDCl3
4:1 dr
4.07
0.90
1.00
0.97
0.93
2.96
4.98
3.33
9.28
2.12
f1 (ppm)

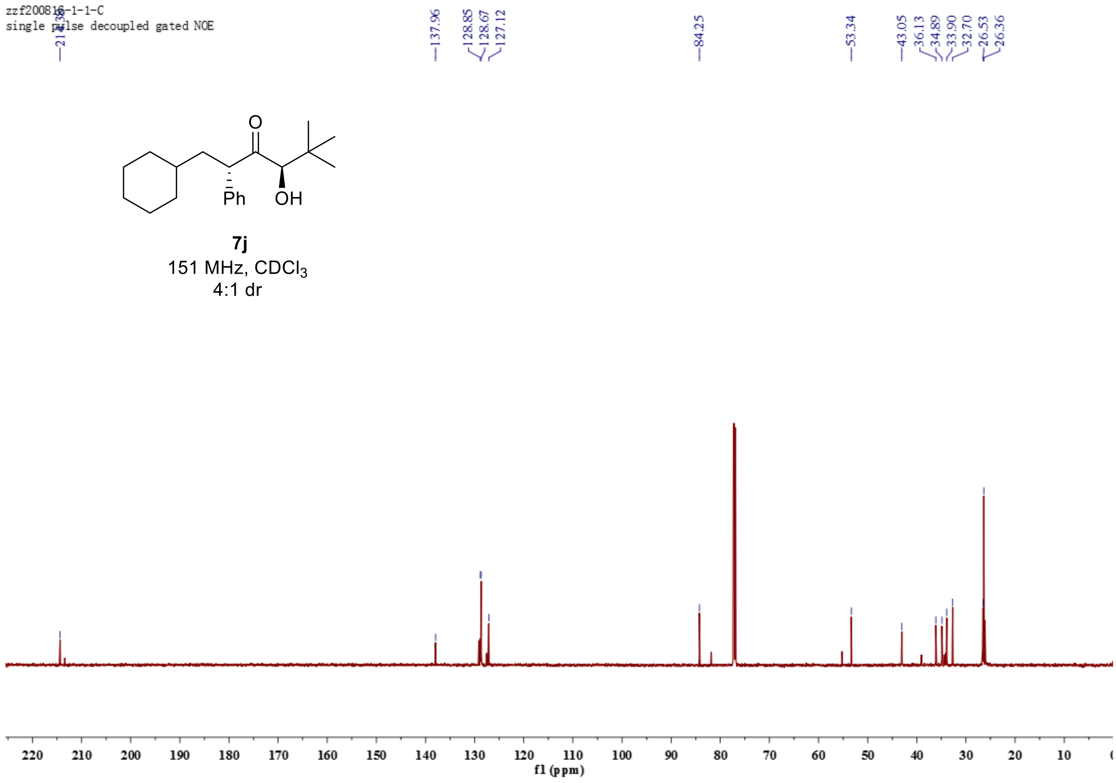
zzf200816-1-1-C
single pulse decoupled gated NOE
137.96
128.85
128.67
127.12
84.25
53.34
43.05
36.13
34.89
33.90
32.70
26.53
26.36
Ph
OH
O
7j
151 MHz, CDCl3
4:1 dr
f1 (ppm)

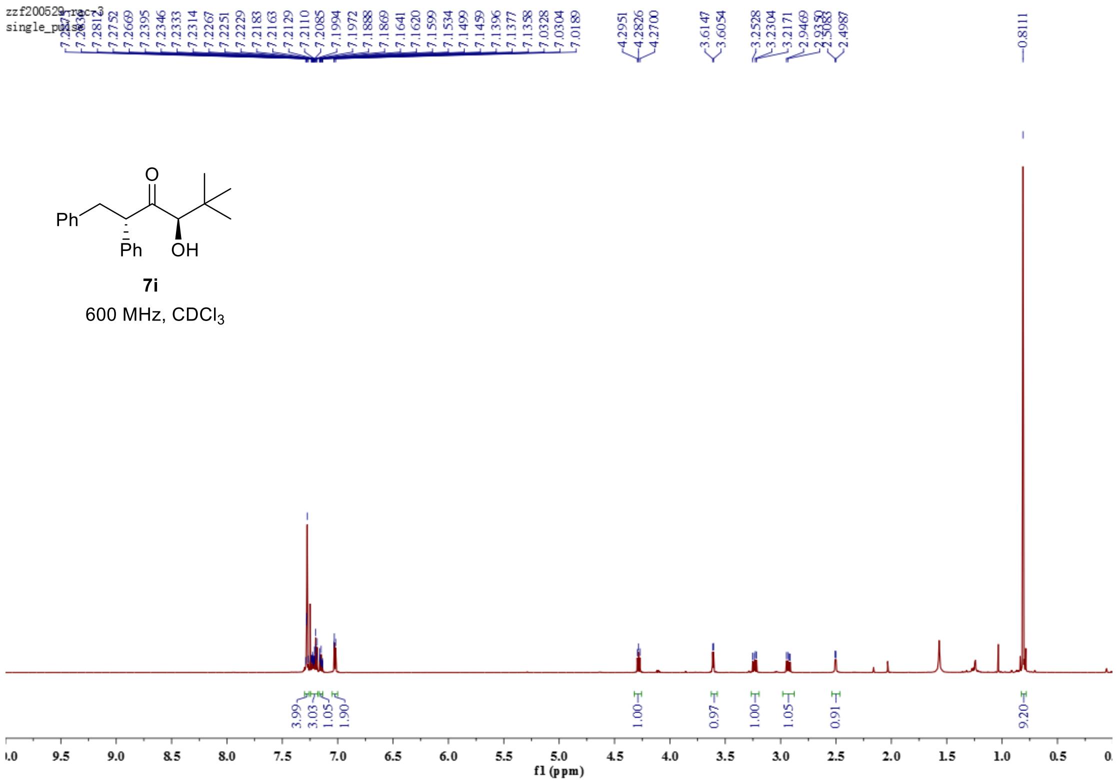
zzf200529-rac-3
single_pulse
7i
600 MHz, CDCl3
f1 (ppm)

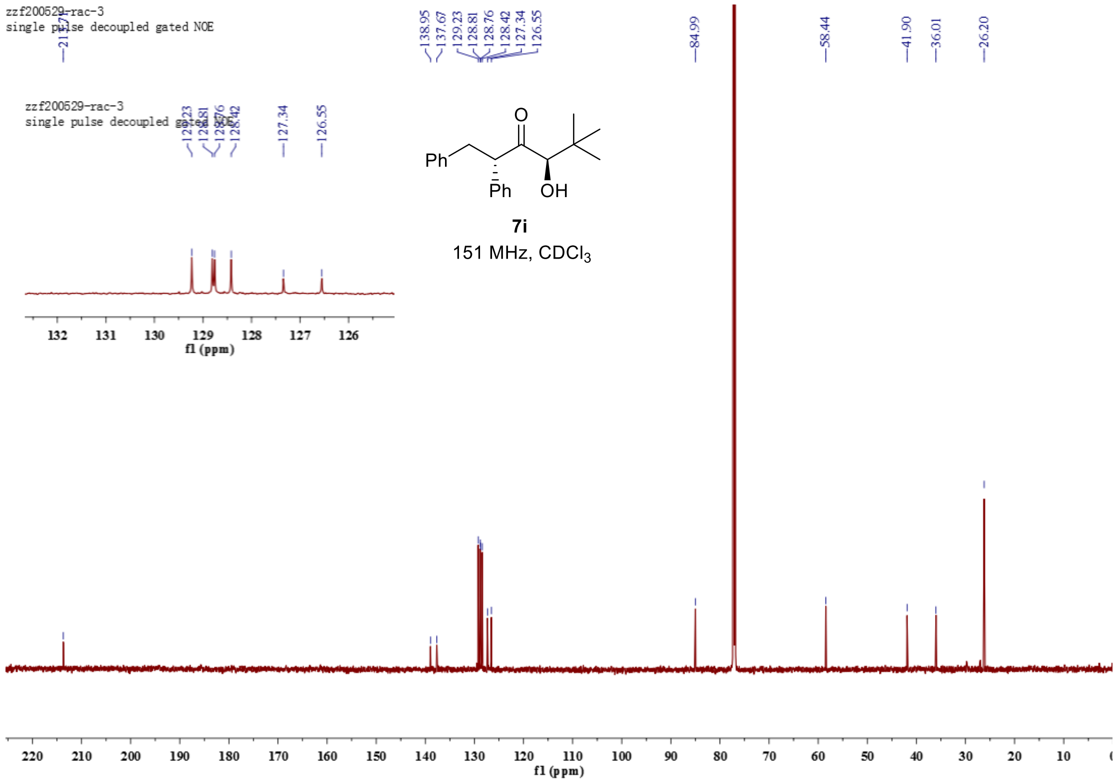
zzf200529-rac-3
single pulse decoupled gated NOE
7i
151 MHz, CDCl3
f1 (ppm)

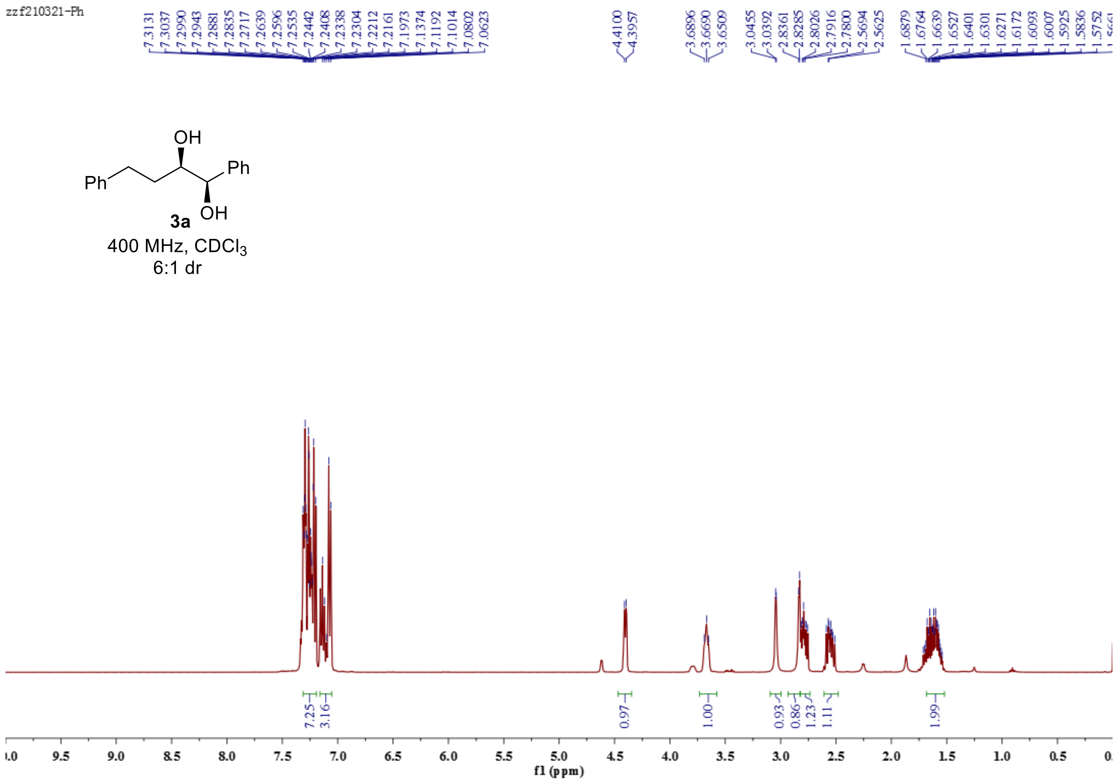
zzf210321-Ph
7.3131
7.3037
7.2990
7.2943
7.2881
7.2835
7.2717
7.2639
7.2596
7.2535
7.2442
7.2408
7.2338
7.2304
7.2212
7.2161
7.1973
7.1374
7.1192
7.1014
7.0802
7.0623
4.4100
4.3957
3.6896
3.6690
3.6509
3.0455
3.0392
2.8361
2.8285
2.8026
2.7916
2.7800
2.5694
2.5625
1.6879
1.6764
1.6639
1.6527
1.6401
1.6301
1.6271
1.6172
1.6093
1.6007
1.5925
1.5836
1.5752
OH
Ph
Ph
OH
3a
400 MHz, CDCl3
6:1 dr
7.25
3.16
0.97
1.00
0.93
0.86
1.23
1.11
1.99
f1 (ppm)

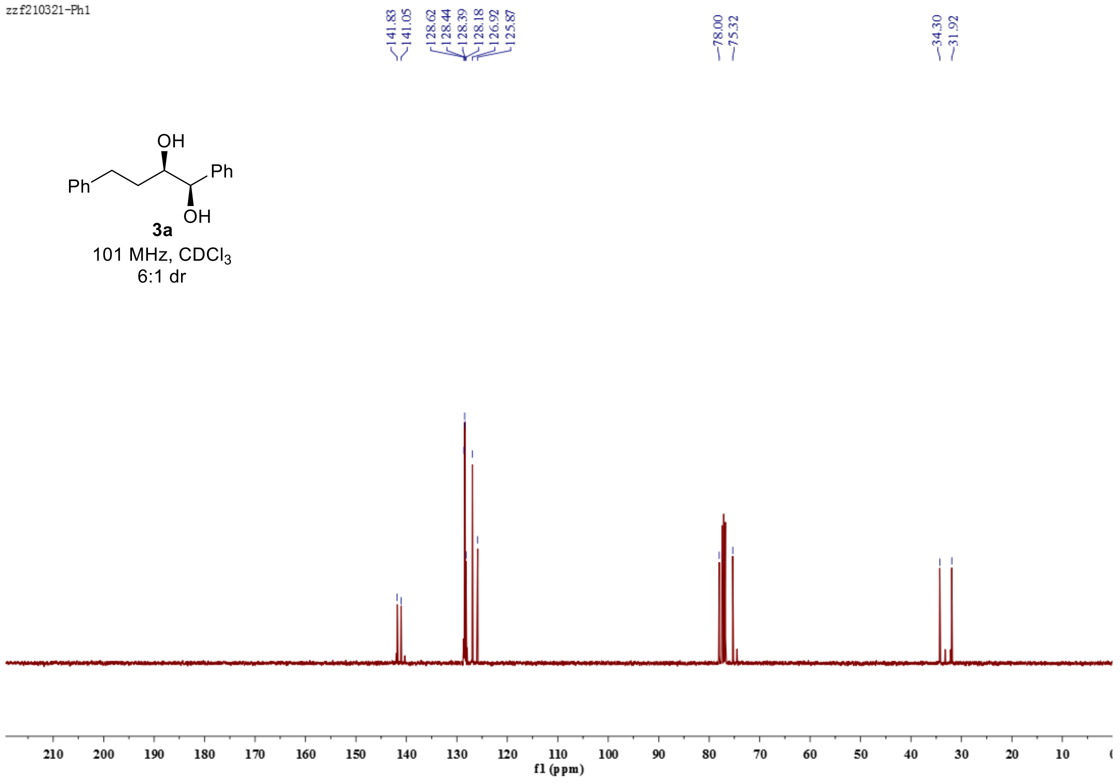
zzf210321-Ph1
141.83
141.05
128.62
128.44
128.39
128.18
126.92
125.87
78.00
75.32
34.30
31.92
OH
Ph
Ph
OH
3a
101 MHz, CDCl3
6:1 dr
f1 (ppm)

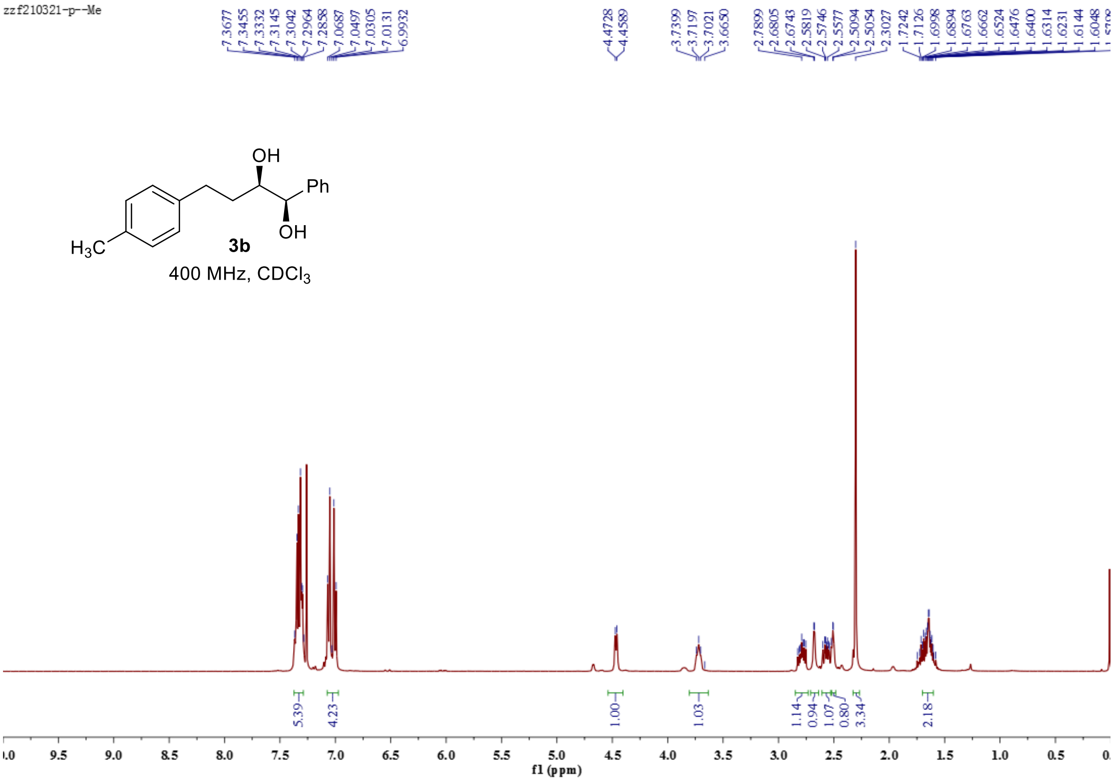
zzf210321-p--Me
7.3677
7.3455
7.3332
7.3145
7.3042
7.2964
7.2858
7.0687
7.0497
7.0305
7.0131
6.9932
4.4728
4.4589
3.7399
3.7197
3.7021
3.6650
2.7899
2.6805
2.6743
2.5819
2.5746
2.5577
2.5094
2.5054
2.3027
1.7242
1.7126
1.6998
1.6894
1.6763
1.6662
1.6524
1.6476
1.6400
1.6314
1.6231
1.6144
1.6048
OH
Ph
OH
H_3C
3b
400 MHz, $CDCl_3$
5.39
4.23
1.00
1.03
1.14
0.94
1.07
0.80
3.34
2.18
f1 (ppm)

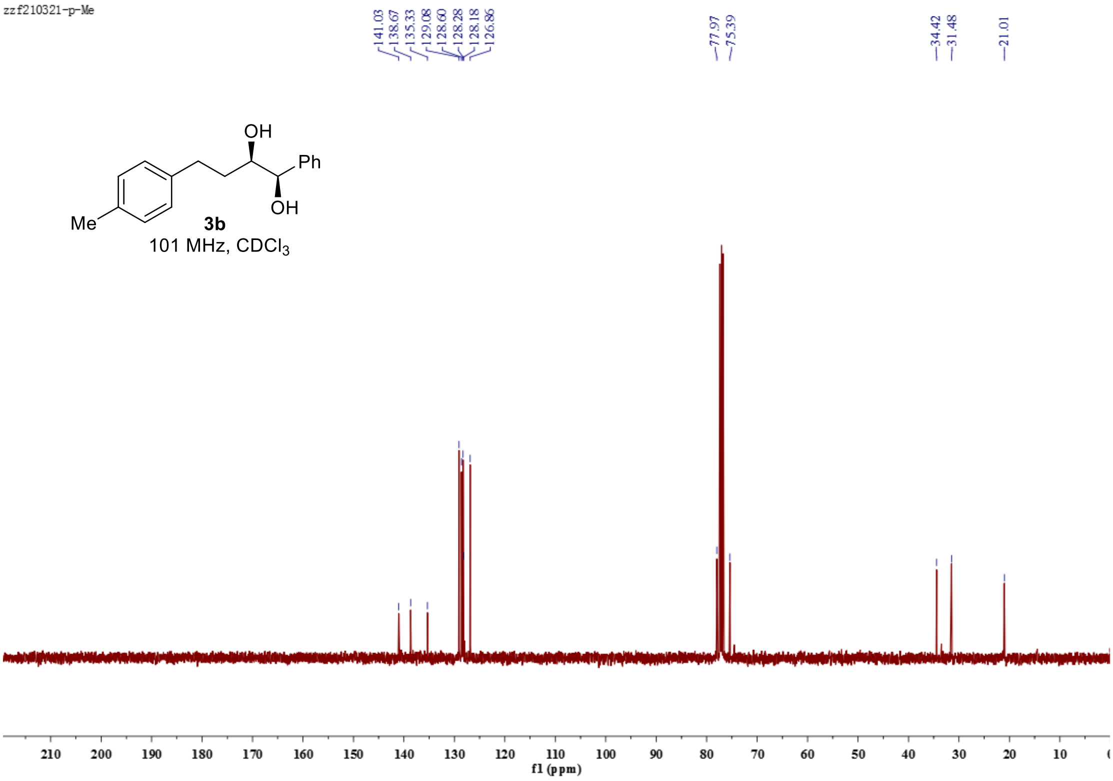
zzf210321-p-Me
141.03
138.67
135.33
129.08
128.60
128.28
128.18
126.86
77.97
75.39
34.42
31.48
21.01
OH
Ph
OH
Me
3b
101 MHz, $CDCl_3$
f1 (ppm)

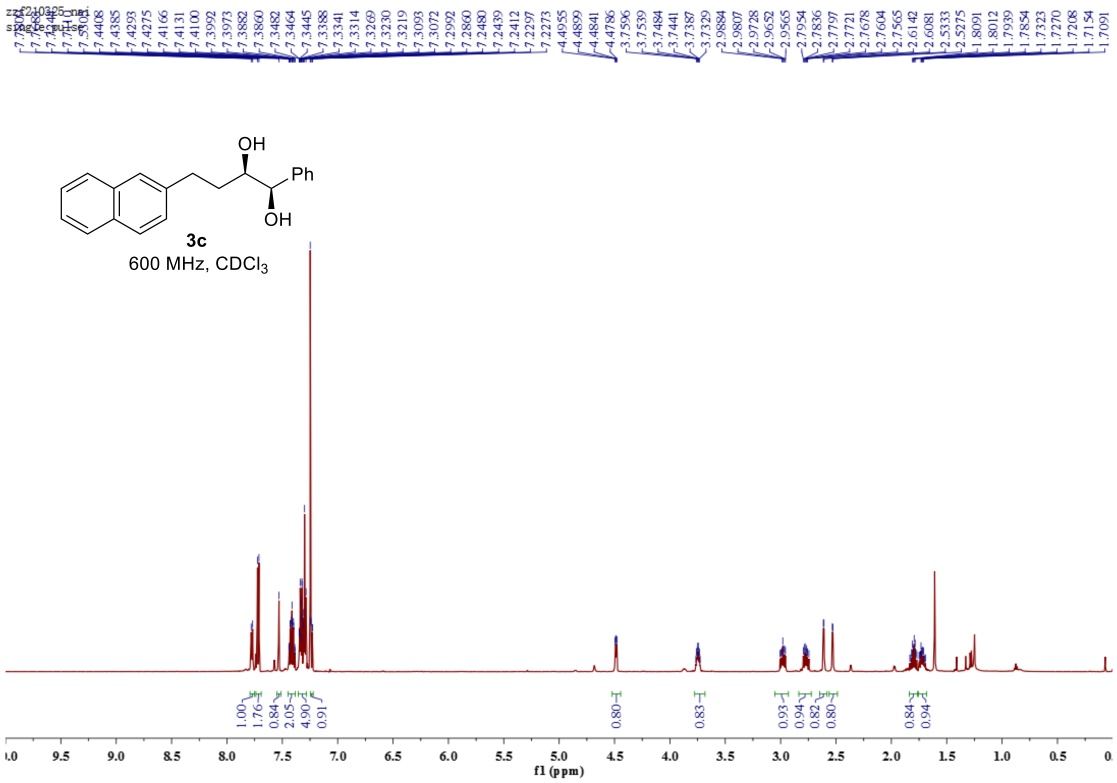

single pulse
7.4408
7.4385
7.4293
7.4275
7.4166
7.4131
7.4100
7.3992
7.3973
7.3882
7.3860
7.3482
7.3464
7.3445
7.3388
7.3341
7.3314
7.3269
7.3230
7.3219
7.3093
7.3072
7.2992
7.2860
7.2480
7.2439
7.2412
7.2297
7.2273
4.4955
4.4899
4.4841
4.4786
3.7596
3.7539
3.7484
3.7441
3.7387
3.7329
2.9884
2.9807
2.9728
2.9652
2.9565
2.7954
2.7836
2.7797
2.7721
2.7678
2.7604
2.7565
2.6142
2.6081
2.5333
2.5275
1.8091
1.8012
1.7939
1.7854
1.7323
1.7270
1.7208
1.7154
1.7091
OH
Ph
OH
3c
600 MHz, CDCl3
1.00
1.76
0.84
2.05
4.90
0.91
0.80
0.83
0.93
0.94
0.82
0.80
0.84
0.94
f1 (ppm)

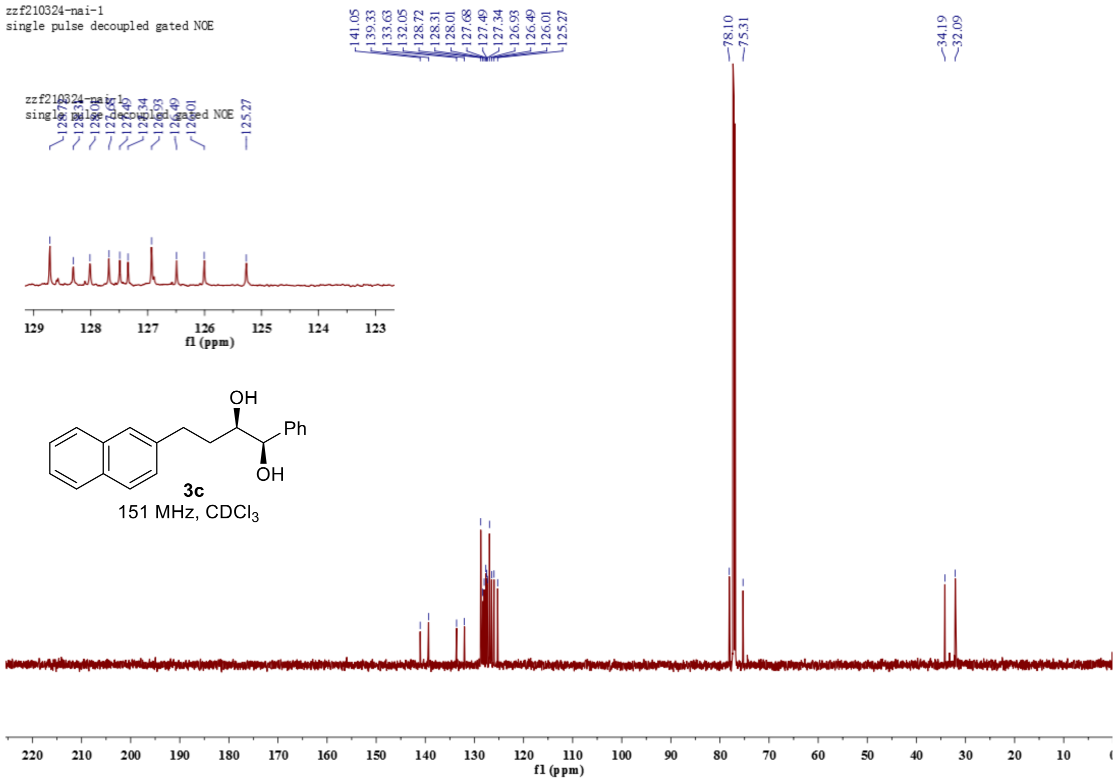

zzf210324-nai-1
single pulse decoupled gated NOE
141.05
139.33
133.63
132.05
128.72
128.31
128.01
127.68
127.49
127.34
126.93
126.49
126.01
125.27
78.10
75.31
34.19
32.09
OH
Ph
OH
3c
151 MHz, CDCl3
f1 (ppm)

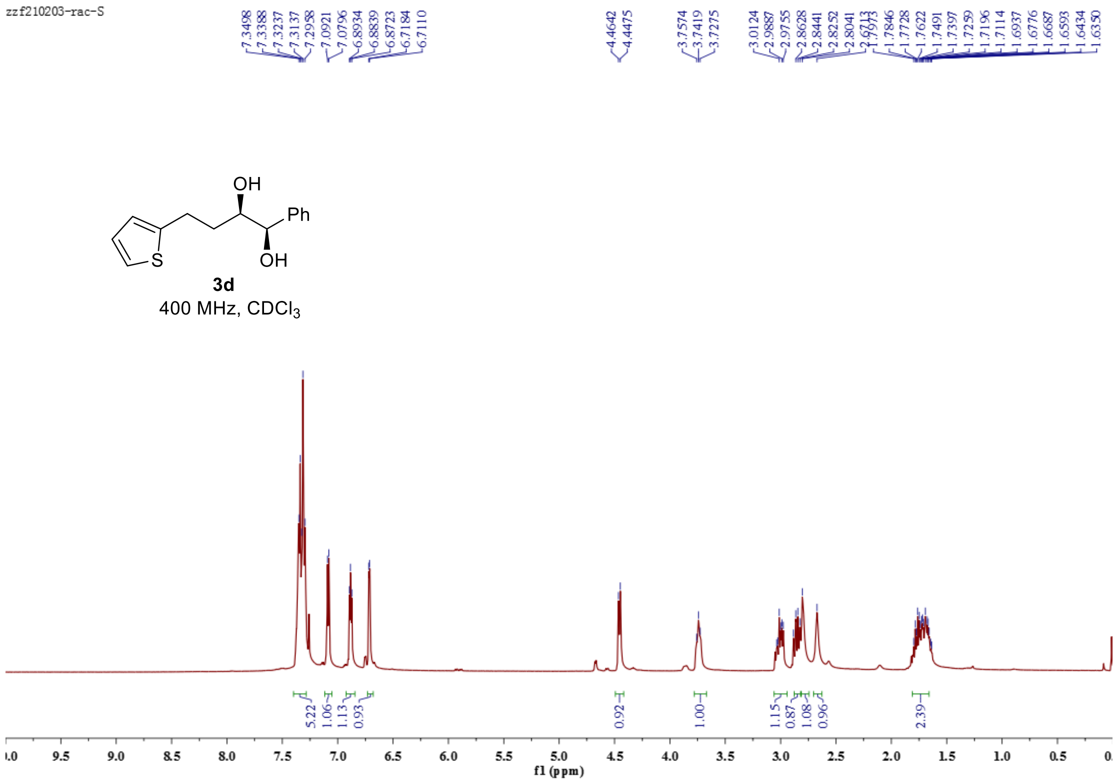
zzf210203-rac-S
7.3498
7.3388
7.3237
7.3137
7.2958
7.0921
7.0796
6.8934
6.8839
6.8723
6.7184
6.7110
4.4642
4.4475
3.7574
3.7419
3.7275
3.0124
2.9887
2.9755
2.8628
2.8441
2.8252
2.8041
[illegible]
1.7846
1.7728
1.7622
1.7491
1.7397
1.7259
1.7196
1.7114
1.6937
1.6776
1.6687
1.6593
1.6434
1.6350
OH
Ph
S
OH
3d
400 MHz, CDCl3
5.22
1.06
1.13
0.93
0.92
1.00
1.15
0.87
1.08
0.96
2.39
9.5
9.0
8.5
8.0
7.5
7.0
6.5
6.0
5.5
5.0
4.5
4.0
3.5
3.0
2.5
2.0
1.5
1.0
0.5
f1 (ppm)

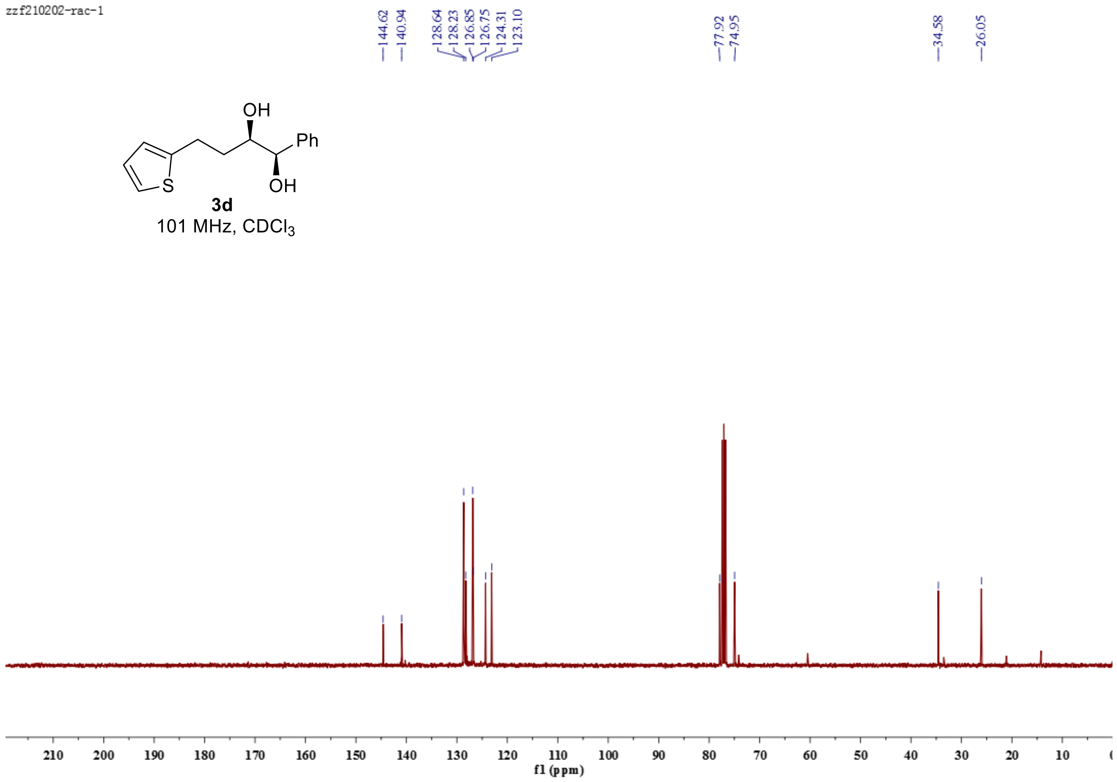
zzf210202-rac-1
144.62
140.94
128.64
128.23
126.85
126.75
124.31
123.10
77.92
74.95
34.58
26.05
OH
Ph
S
OH
3d
101 MHz, CDCl3
210
200
190
180
170
160
150
140
130
120
110
100
90
80
70
60
50
40
30
20
10
f1 (ppm)

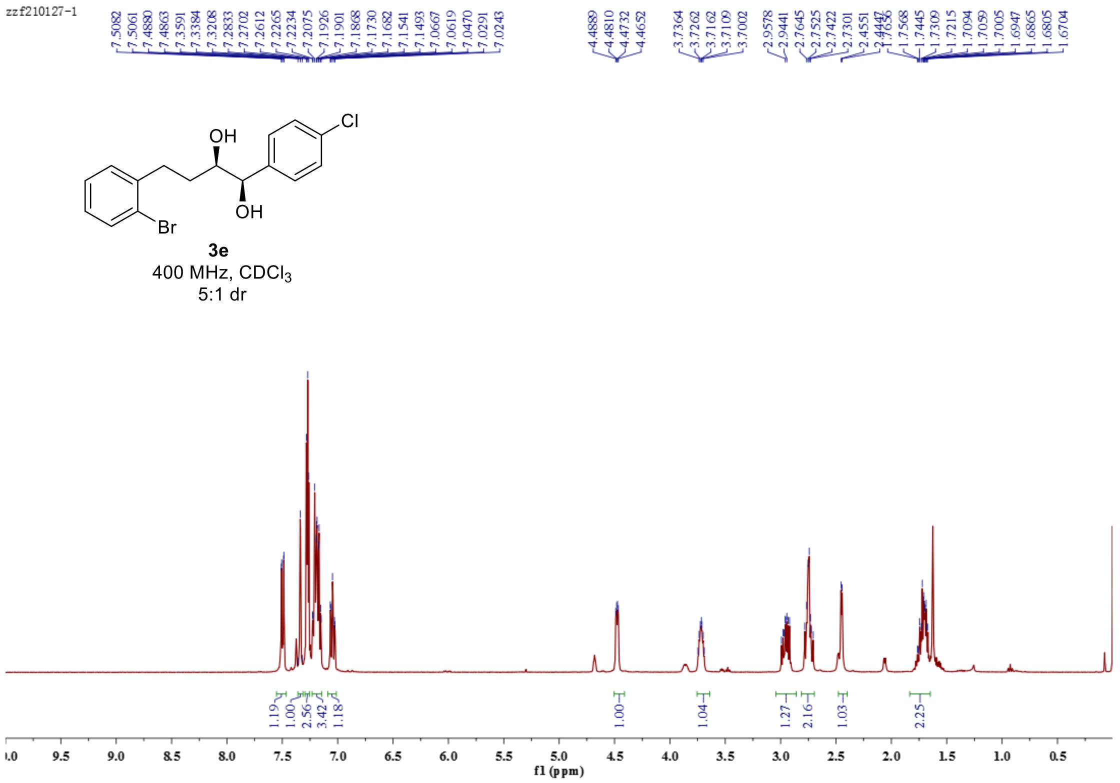
zzf210127-1
7.5082 7.5061 7.4880 7.4863 7.3591 7.3384 7.3208 7.2833 7.2702 7.2612 7.2265 7.2234 7.2075 7.1926 7.1901 7.1868 7.1730 7.1682 7.1541 7.1493 7.0667 7.0619 7.0470 7.0291 7.0243
4.4889 4.4810 4.4732 4.4652
3.7364 3.7262 3.7162 3.7109 3.7002
2.9578 2.9441 2.7645 2.7525 2.7422 2.7301 2.4551
1.7568 1.7445 1.7309 1.7215 1.7094 1.7059 1.7005 1.6947 1.6865 1.6805 1.6704
OH
OH
Cl
Br
3e
400 MHz, CDCl3
5:1 dr
1.19 1.00 2.56 3.42 1.18 1.00 1.04 1.27 2.16 1.03 2.25
f1 (ppm)

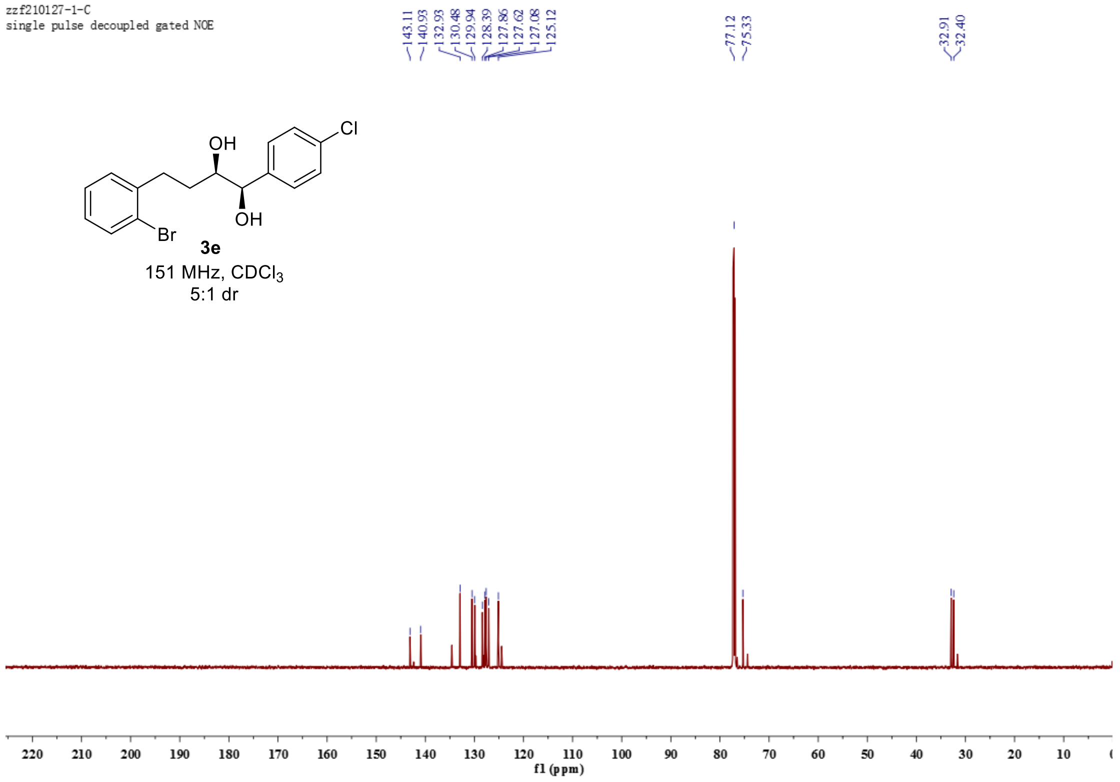
zzf210127-1-C
single pulse decoupled gated NOE
143.11 140.93 132.93 130.48 129.94 128.39 127.86 127.62 127.08 125.12
77.12 75.33
32.91 32.40
OH
OH
Cl
Br
3e
151 MHz, CDCl3
5:1 dr
f1 (ppm)

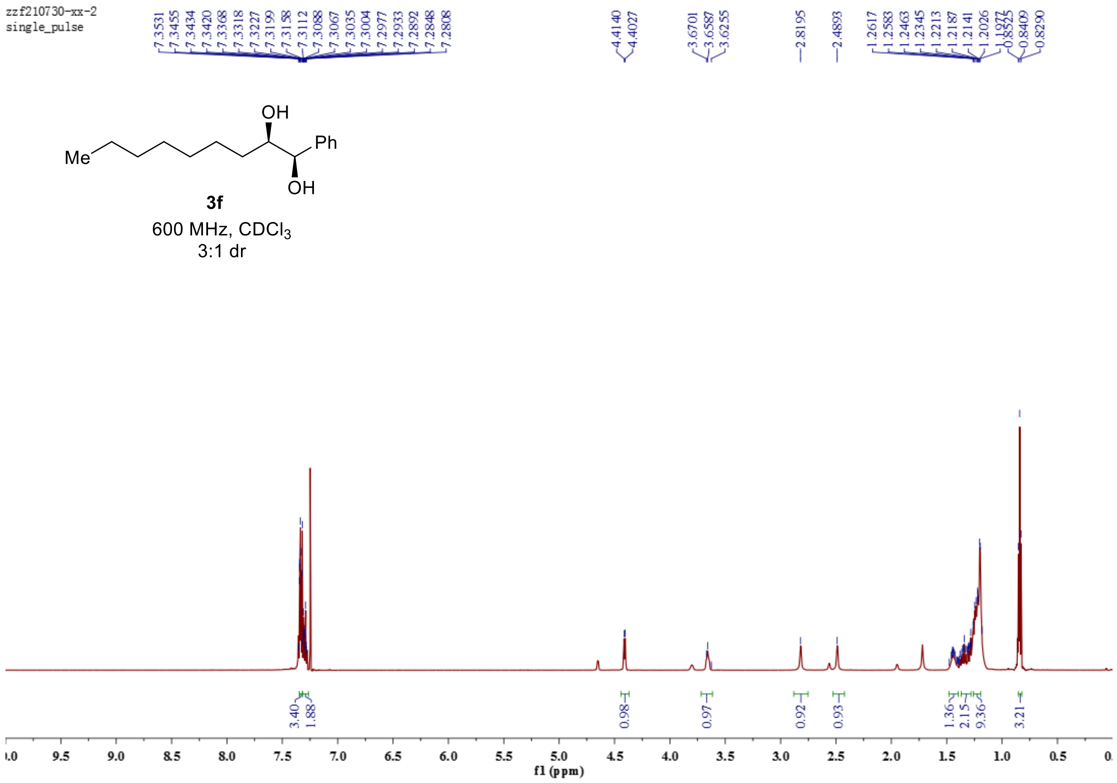

3f

600 MHz, $CDCl_3$

3:1 dr

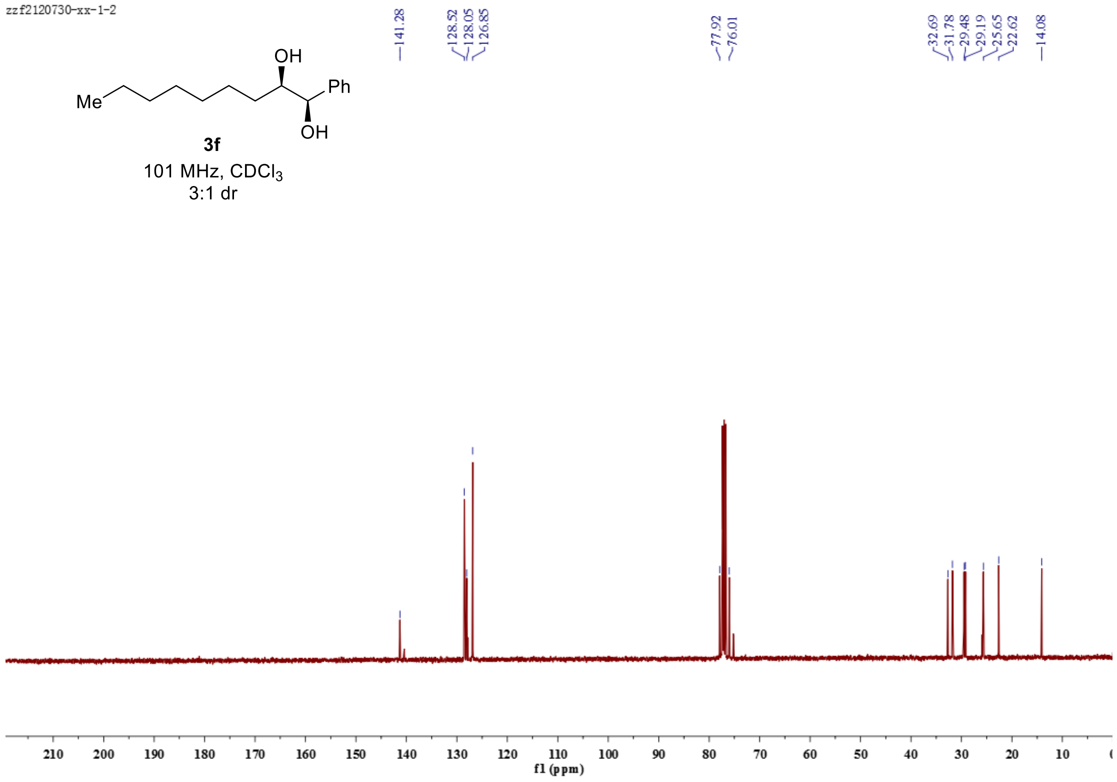

3f

101 MHz, $CDCl_3$

3:1 dr

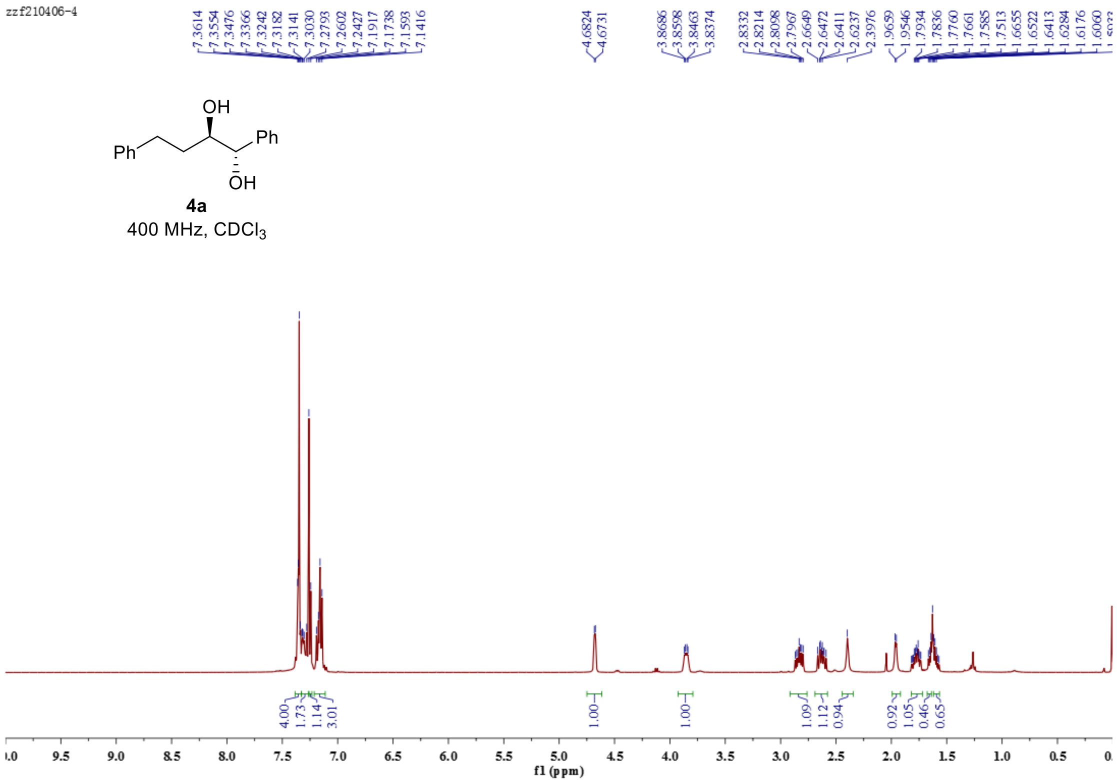
zzf210406-4
7.3614
7.3554
7.3476
7.3366
7.3242
7.3182
7.3141
7.3030
7.2793
7.2602
7.2427
7.1917
7.1738
7.1593
7.1416
4.6824
4.6731
3.8686
3.8598
3.8463
3.8374
2.8332
2.8214
2.8098
2.7967
2.6649
2.6472
2.6411
2.6237
2.3976
1.9659
1.9546
1.7934
1.7836
1.7760
1.7661
1.7585
1.7513
1.6655
1.6522
1.6413
1.6284
1.6176
1.6060
OH
Ph
Ph
OH
4a
400 MHz, CDCl3
4.00
1.73
1.14
3.01
1.00
1.00
1.09
1.12
0.94
0.92
1.05
0.46
0.65
9.5
9.0
8.5
8.0
7.5
7.0
6.5
6.0
5.5
5.0
4.5
4.0
3.5
3.0
2.5
2.0
1.5
1.0
0.5
f1 (ppm)

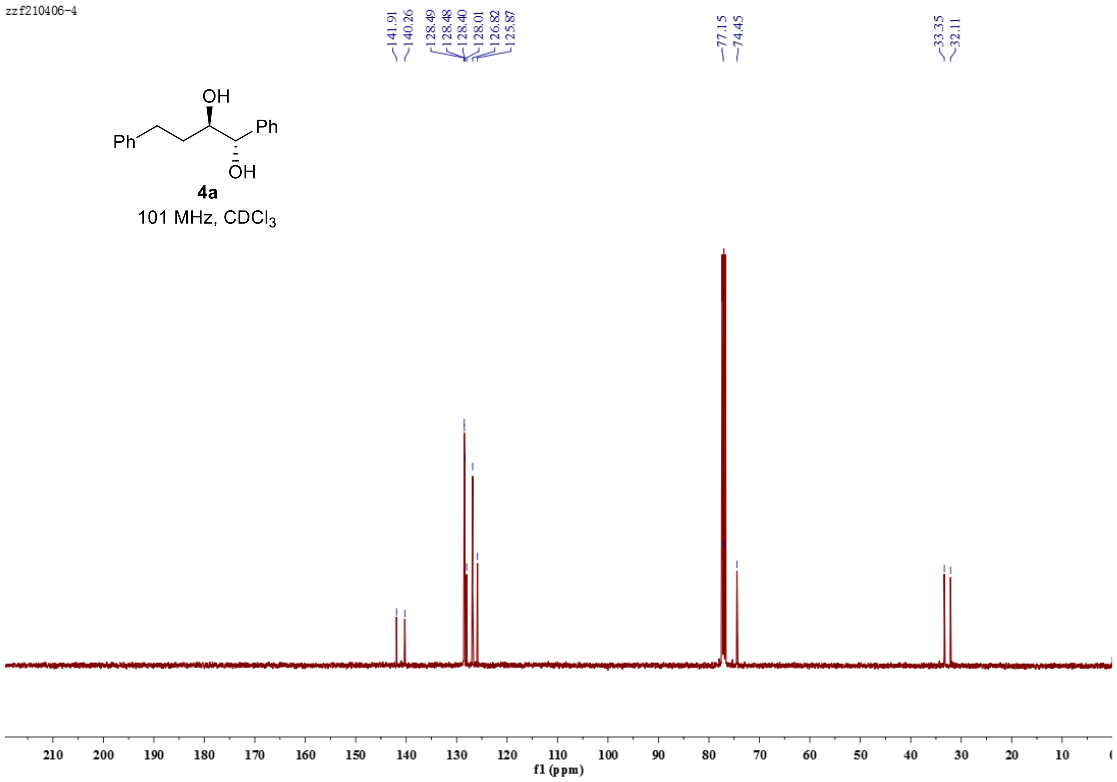
zzf210406-4
141.91
140.26
128.49
128.48
128.40
128.01
126.82
125.87
77.15
74.45
33.35
32.11
OH
Ph
Ph
OH
4a
101 MHz, CDCl3
210
200
190
180
170
160
150
140
130
120
110
100
90
80
70
60
50
40
30
20
10
f1 (ppm)

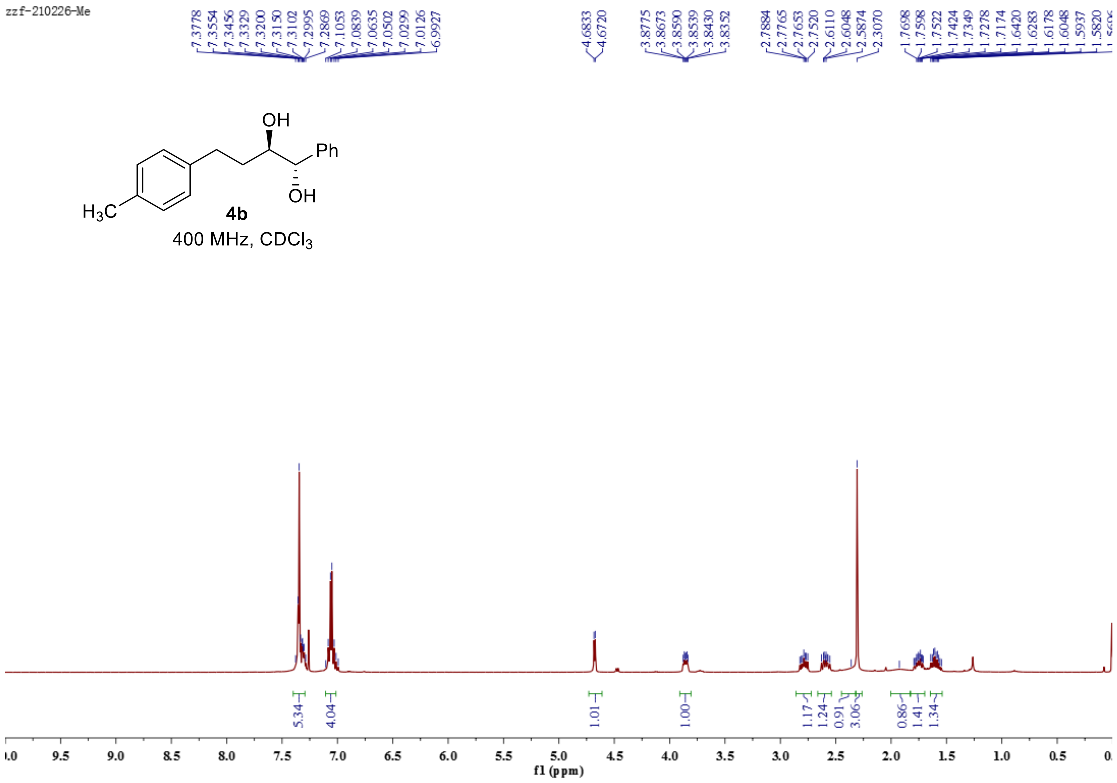
zzf-210226-Me
OH
Ph
OH
H_3C
4b
400 MHz, $CDCl_3$
f1 (ppm)

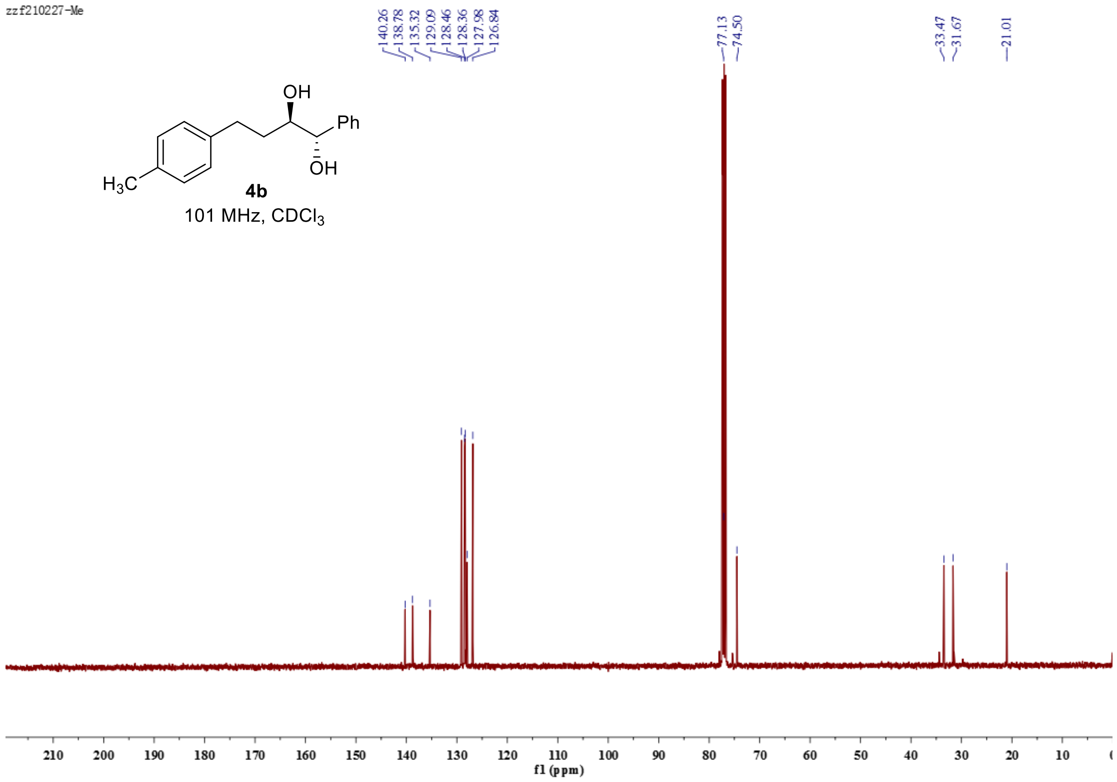
zzf210227-Me
140.26
138.78
135.32
129.09
128.46
128.36
127.98
126.84
77.13
74.50
33.47
31.67
21.01
OH
Ph
OH
H_3C
4b
101 MHz, $CDCl_3$
f1 (ppm)

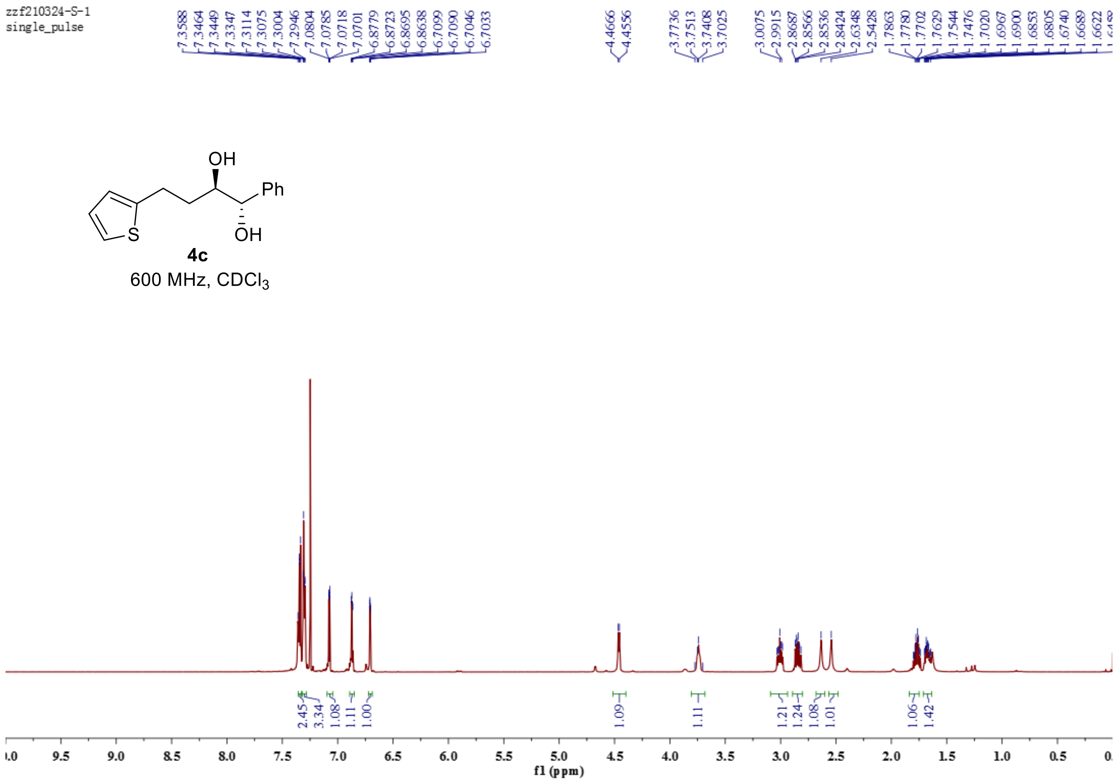
zzf210324-S-1
single_pulse
7.3588 7.3464 7.3449 7.3347 7.3114 7.3075 7.3004 7.2946 7.0804 7.0785 7.0718 7.0701 6.8779 6.8723 6.8695 6.8638 6.7099 6.7090 6.7046 6.7033
4.4666 4.4556
3.7736 3.7513 3.7408 3.7025
3.0075 2.9915 2.8687 2.8566 2.8536 2.8424 2.6348 2.5428 1.7863 1.7780 1.7702 1.7629 1.7544 1.7476 1.7020 1.6967 1.6900 1.6853 1.6805 1.6740 1.6689 1.6622
OH
Ph
S
OH
4c
600 MHz, CDCl3
2.45 3.34 1.08 1.11 1.00 1.09 1.11 1.21 1.24 1.08 1.01 1.06 1.42
f1 (ppm)

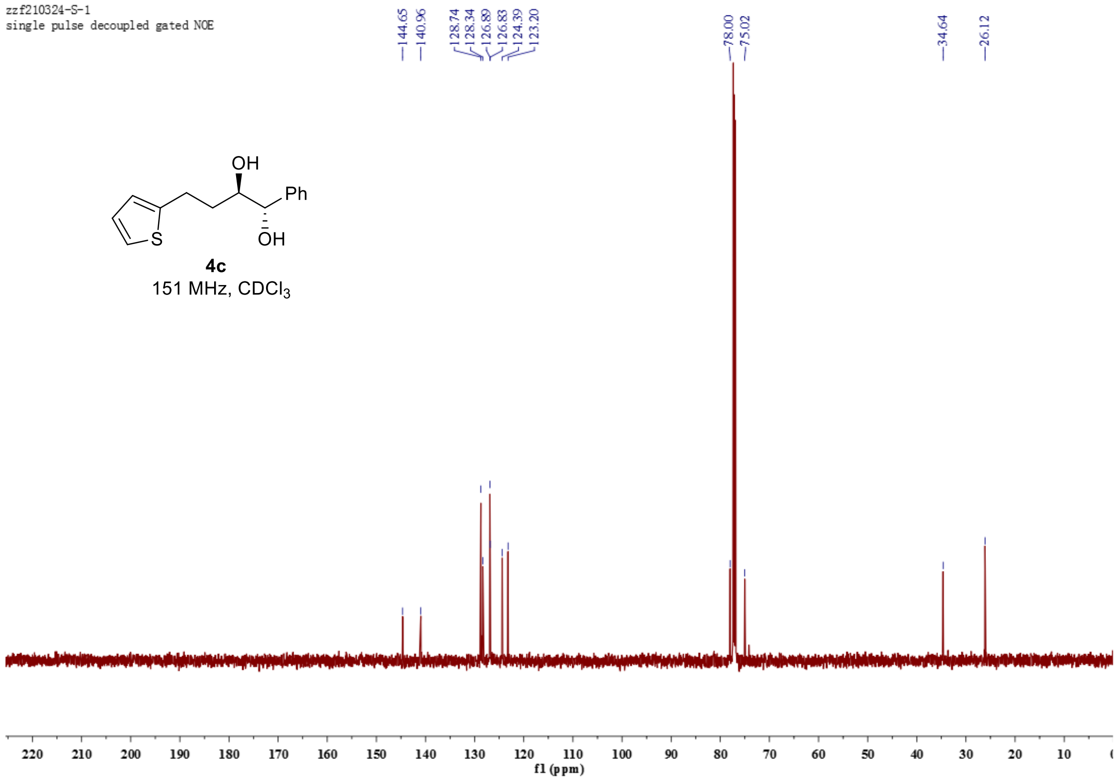
zzf210324-S-1
single pulse decoupled gated NOE
144.65 140.96 128.74 128.34 126.89 126.83 124.39 123.20 78.00 75.02 34.64 26.12
OH
Ph
S
OH
4c
151 MHz, CDCl3
f1 (ppm)

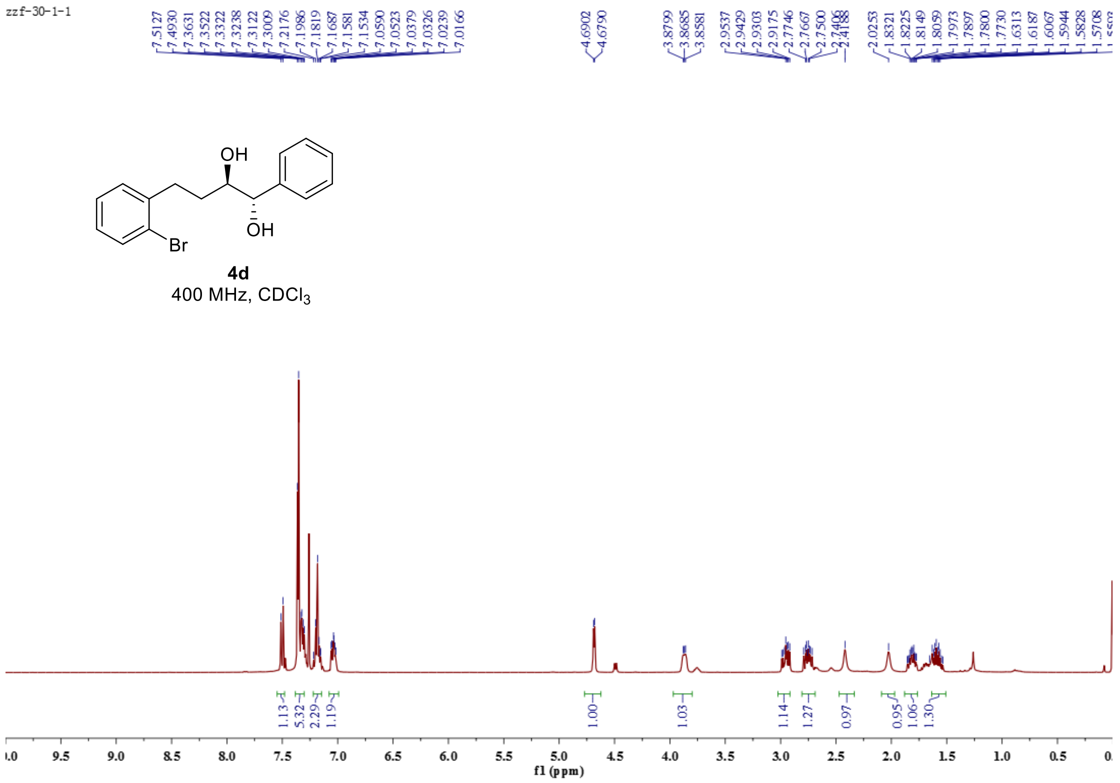
zzf-30-1-1
OH
OH
Br
4d
400 MHz, CDCl3
f1 (ppm)

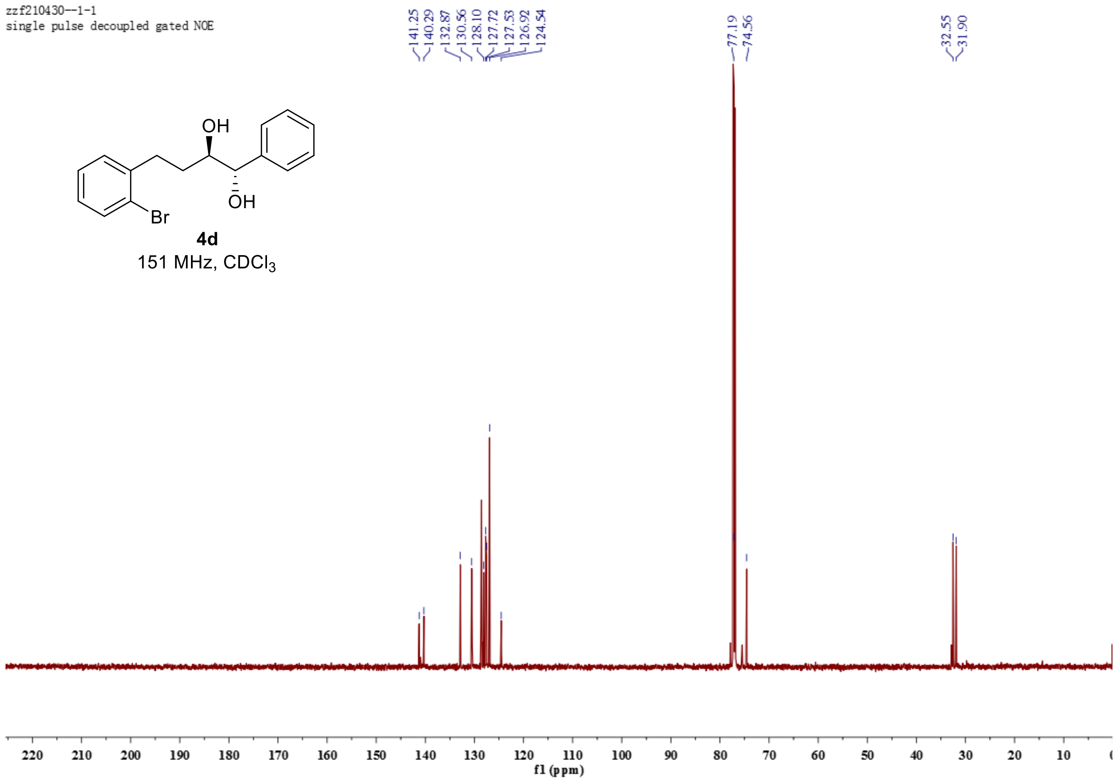
zzf210430--1-1
single pulse decoupled gated NOE
OH
OH
Br
4d
151 MHz, CDCl3
f1 (ppm)

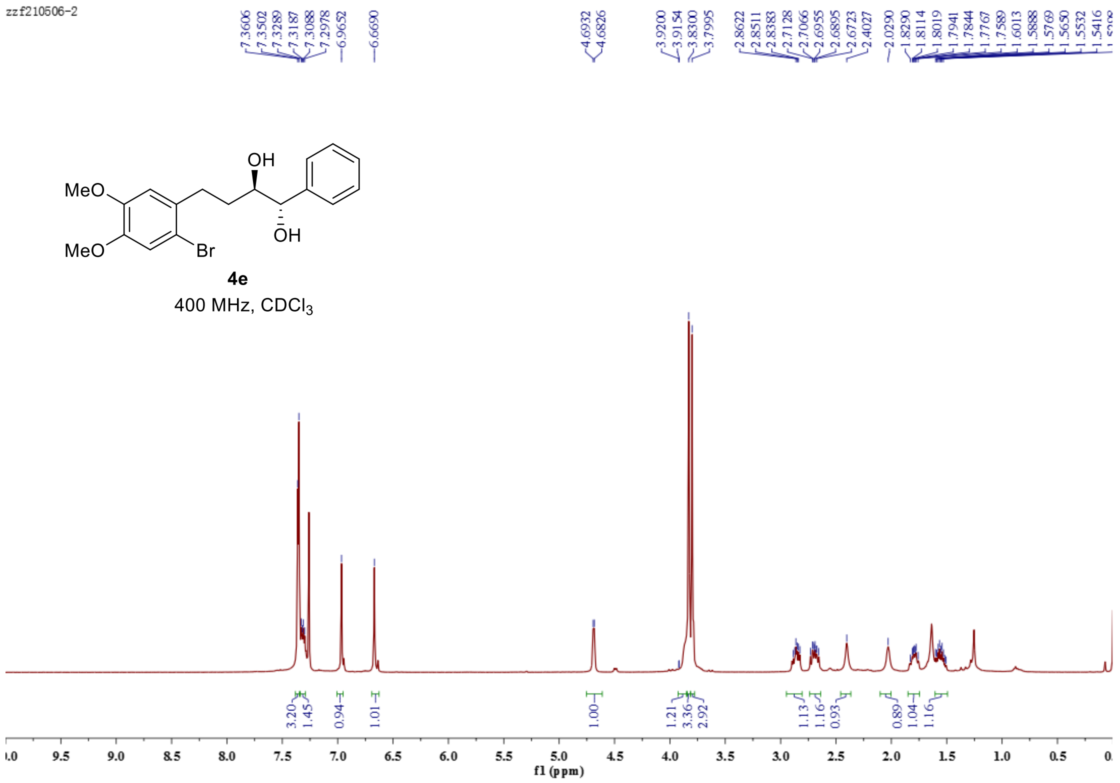
zzf210506-2
MeO
MeO
OH
OH
Br
4e
400 MHz, CDCl3
3.20
1.45
0.94
1.01
1.00
1.21
3.36
2.92
1.13
1.16
0.93
0.89
1.04
1.16
f1 (ppm)

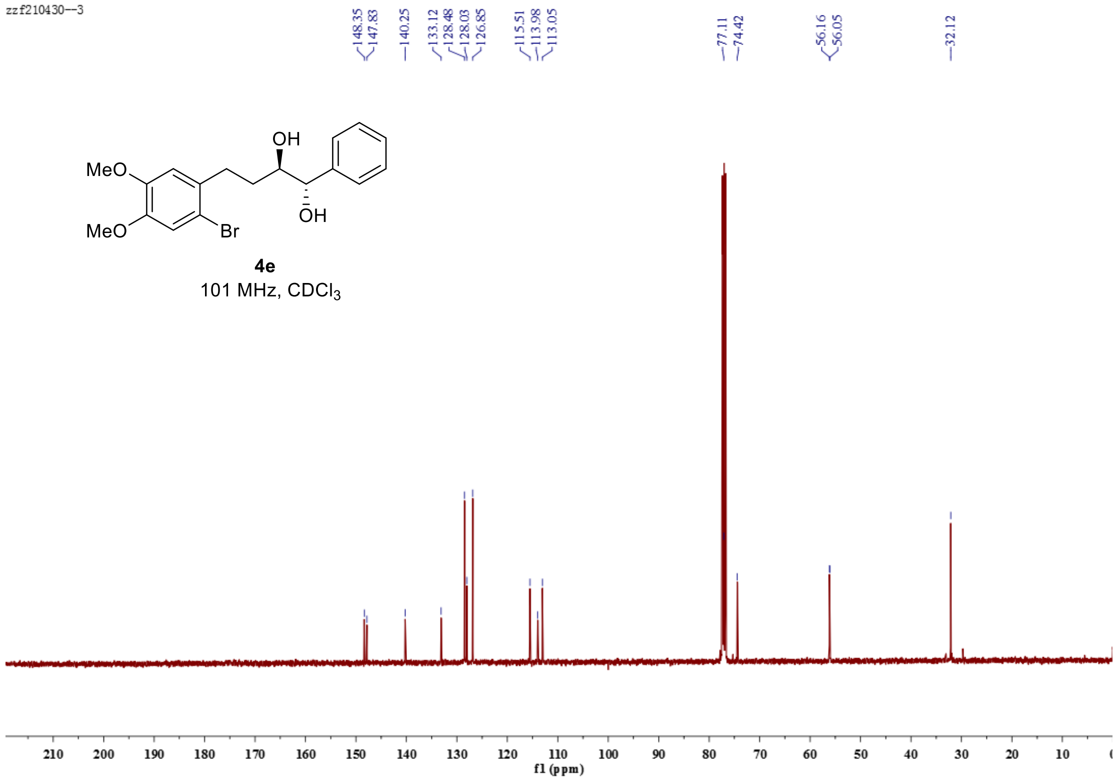
zzf210430--3
148.35
147.83
140.25
133.12
128.48
128.03
126.85
115.51
113.98
113.05
77.11
74.42
56.16
56.05
32.12
MeO
MeO
OH
OH
Br
4e
101 MHz, CDCl3
f1 (ppm)

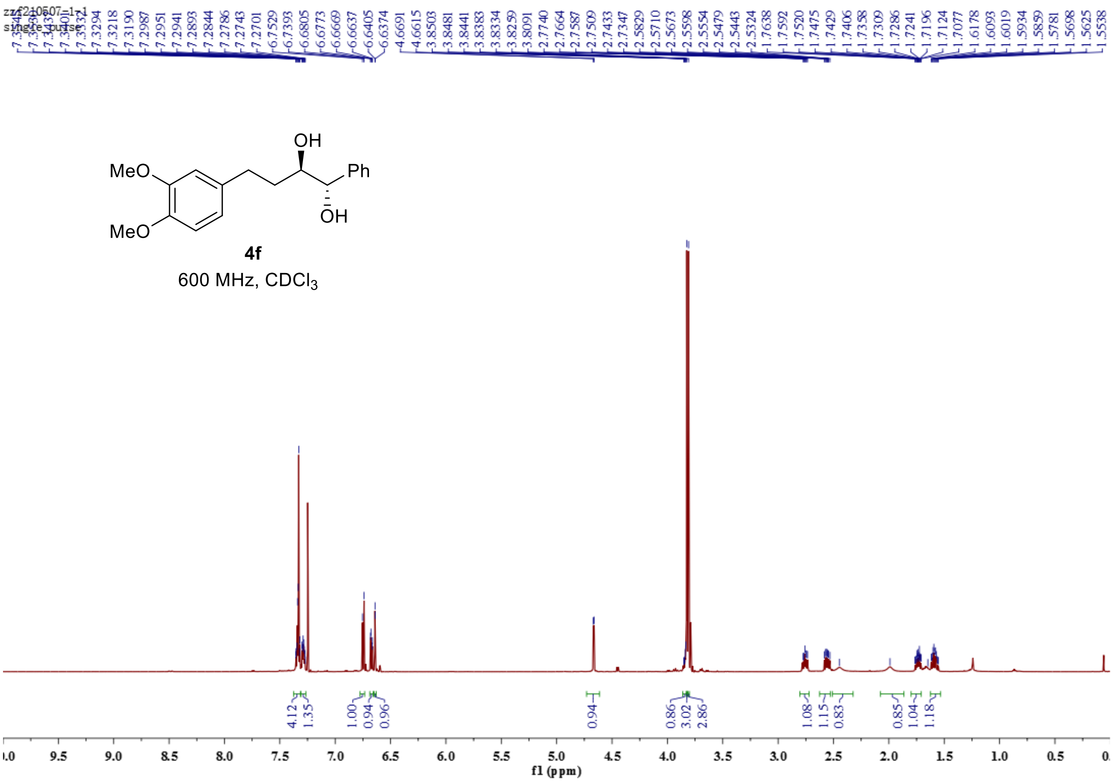

zzf210507-1-1
single pulse
7.3218
7.3190
7.2987
7.2951
7.2941
7.2893
7.2844
7.2786
7.2743
7.2701
6.7529
6.7393
6.6805
6.6773
6.6669
6.6637
6.6405
6.6374
4.6691
4.6615
3.8503
3.8481
3.8441
3.8383
3.8334
3.8259
3.8091
2.7740
2.7664
2.7587
2.7509
2.7433
2.7347
2.5829
2.5710
2.5673
2.5598
2.5554
2.5479
2.5443
2.5324
1.7638
1.7592
1.7520
1.7475
1.7429
1.7406
1.7358
1.7309
1.7286
1.7241
1.7196
1.7124
1.7077
1.6178
1.6093
1.6019
1.5934
1.5859
1.5781
1.5698
1.5625
1.5538
MeO
MeO
OH
OH
Ph
4f
600 MHz, CDCl3
4.12
1.35
1.00
0.94
0.96
0.94
0.86
3.02
2.86
1.08
1.15
0.83
0.85
1.04
1.18
f1 (ppm)

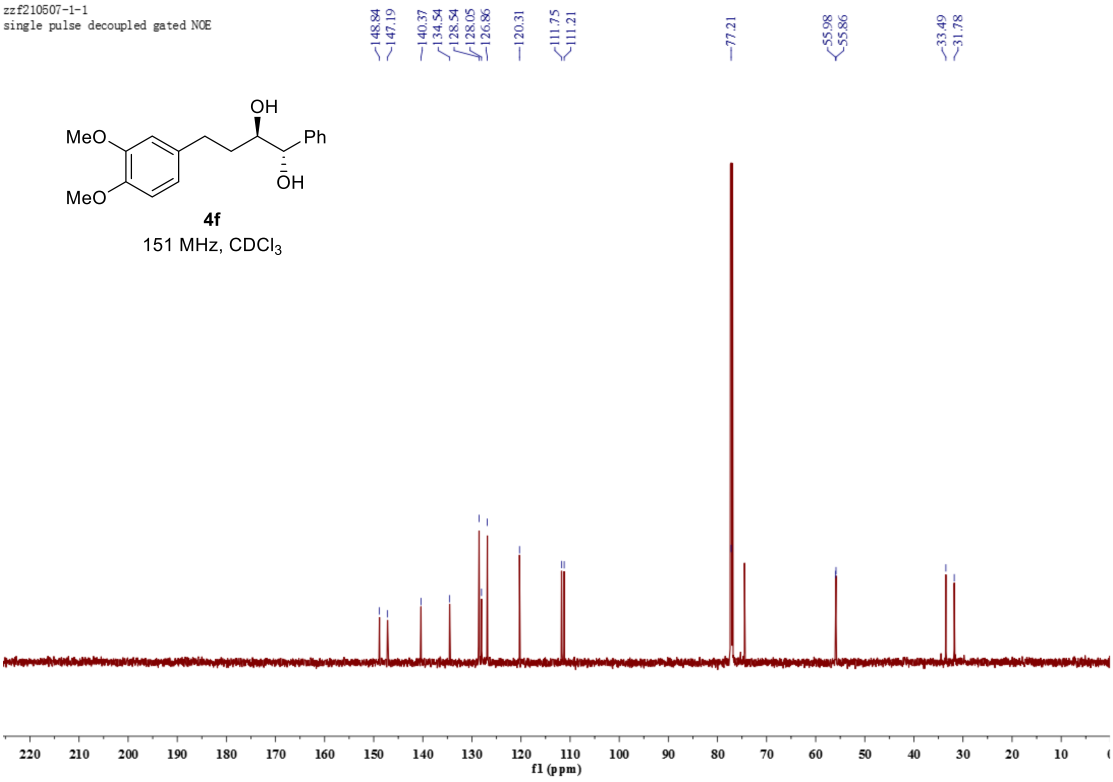

zzf210507-1-1
single pulse decoupled gated NOE
148.84
147.19
140.37
134.54
128.54
128.05
126.86
120.31
111.75
111.21
77.21
55.98
55.86
33.49
31.78
MeO
MeO
OH
OH
Ph
4f
151 MHz, CDCl3
f1 (ppm)

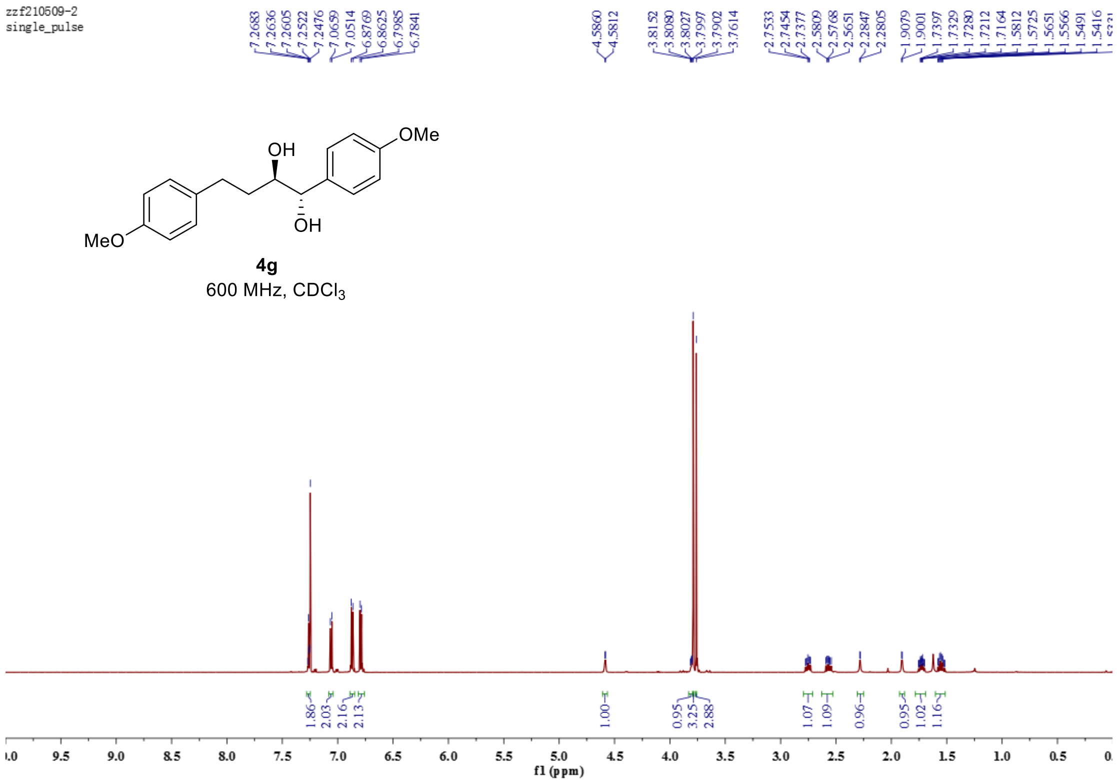
zzf210509-2
single_pulse
OH
OMe
MeO
OH
4g
600 MHz, CDCl3
f1 (ppm)

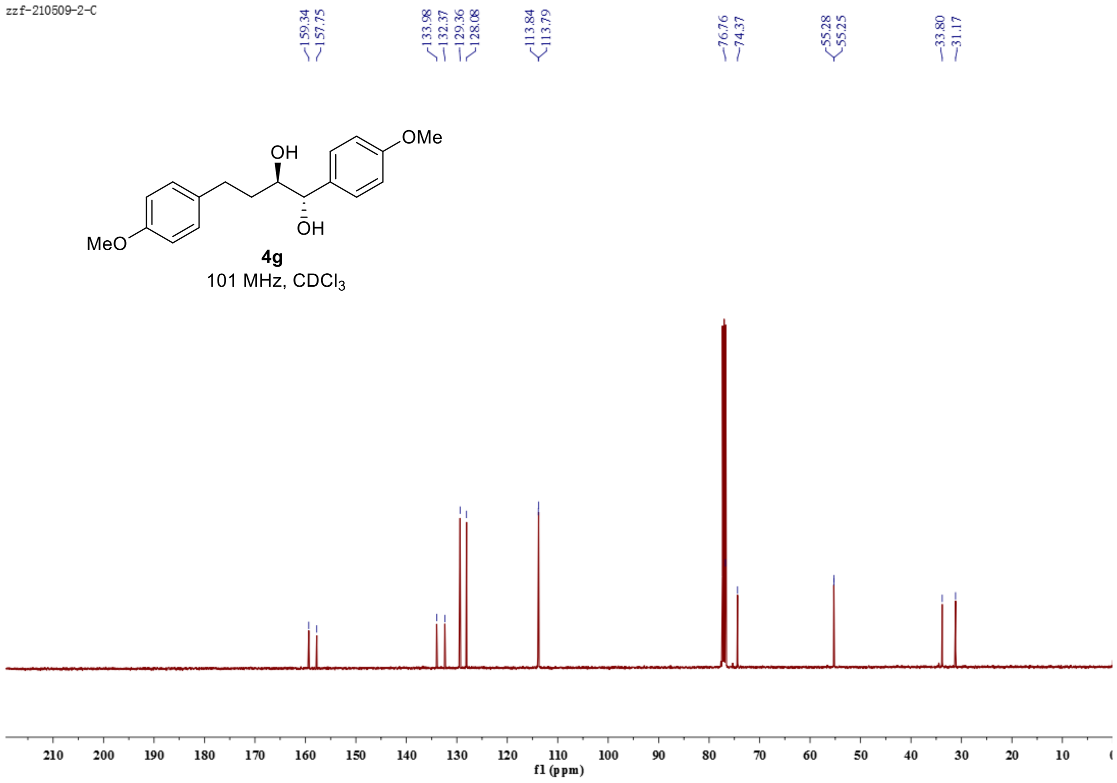
zzf-210509-2-C
159.34
157.75
133.98
132.37
129.36
128.08
113.84
113.79
76.76
74.37
55.28
55.25
33.80
31.17
OH
OMe
MeO
OH
4g
101 MHz, CDCl3
f1 (ppm)

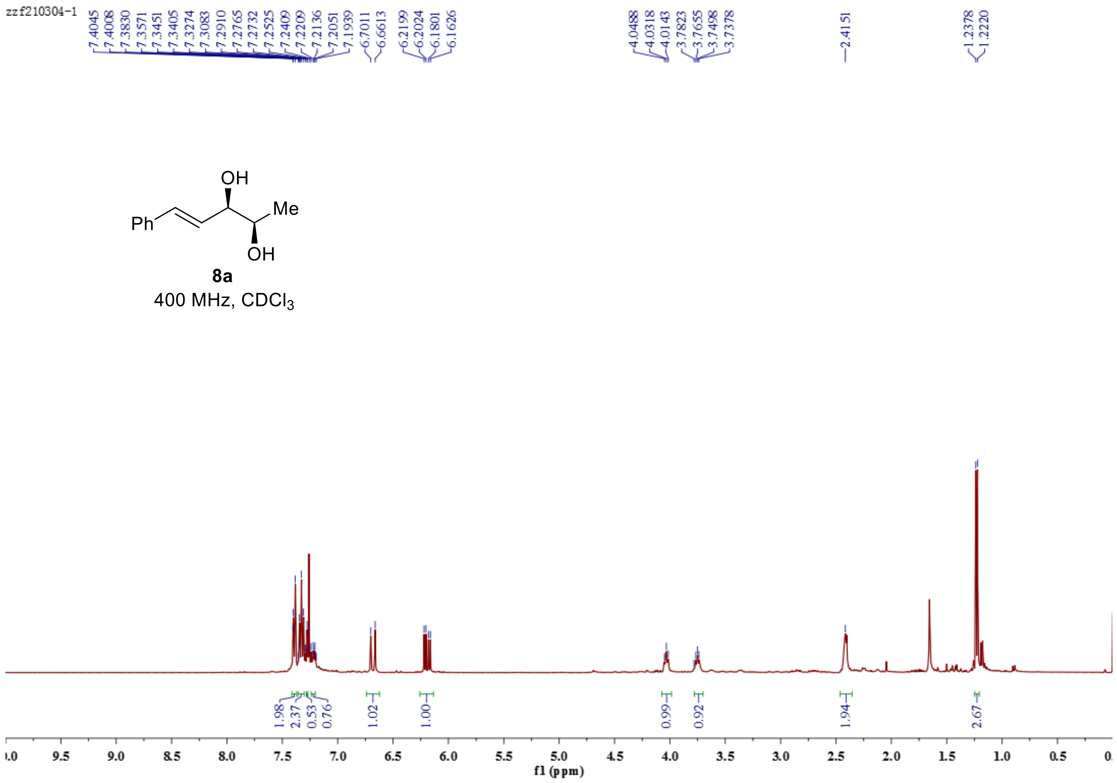
zzf210304-1
7.4045
7.4008
7.3830
7.3571
7.3451
7.3405
7.3274
7.3083
7.2910
7.2765
7.2732
7.2525
7.2409
7.2209
7.2136
7.2051
7.1939
6.7011
6.6613
6.2199
6.2024
6.1801
6.1626
4.0488
4.0318
4.0143
3.7823
3.7655
3.7498
3.7378
2.4151
1.2378
1.2220
OH
Me
Ph
OH
8a
400 MHz, CDCl3
1.98
2.37
0.53
0.76
1.02
1.00
0.99
0.92
1.94
2.67
f1 (ppm)

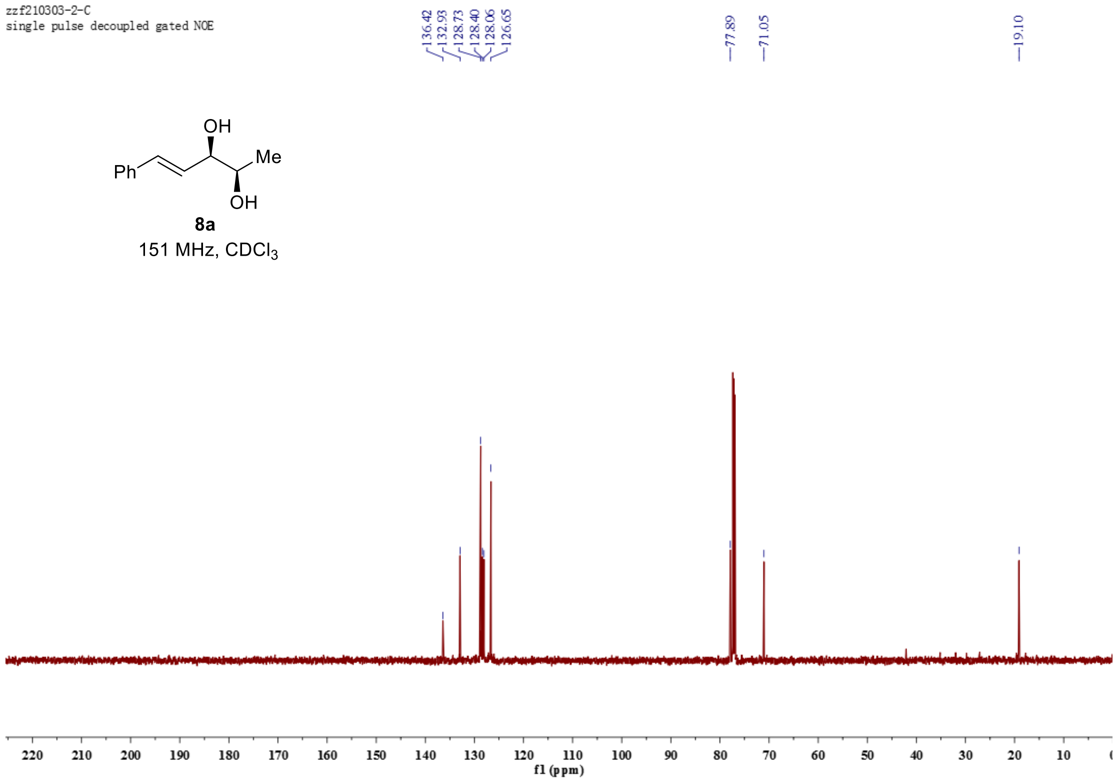
zzf210303-2-C
single pulse decoupled gated NOE
136.42
132.93
128.73
128.40
128.06
126.65
77.89
71.05
19.10
OH
Me
Ph
OH
8a
151 MHz, CDCl3
f1 (ppm)

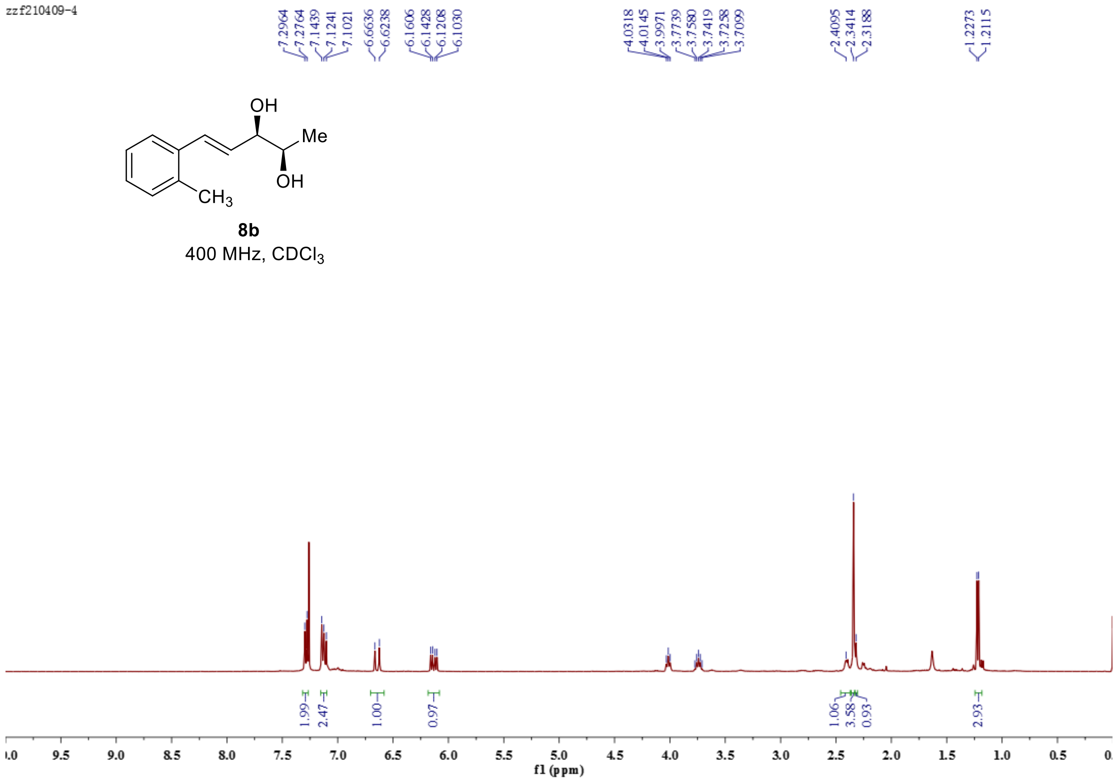
zzf210409-4
7.2964
7.2764
7.1439
7.1241
7.1021
6.6636
6.6238
6.1606
6.1428
6.1208
6.1030
4.0318
4.0145
3.9971
3.7739
3.7580
3.7419
3.7258
3.7099
2.4095
2.3414
2.3188
1.2273
1.2115
OH
Me
OH
CH_3
8b
400 MHz, $CDCl_3$
1.99
2.47
1.00
0.97
1.06
3.58
0.93
2.93
9.5
9.0
8.5
8.0
7.5
7.0
6.5
6.0
5.5
5.0
4.5
4.0
3.5
3.0
2.5
2.0
1.5
1.0
0.5
f1 (ppm)

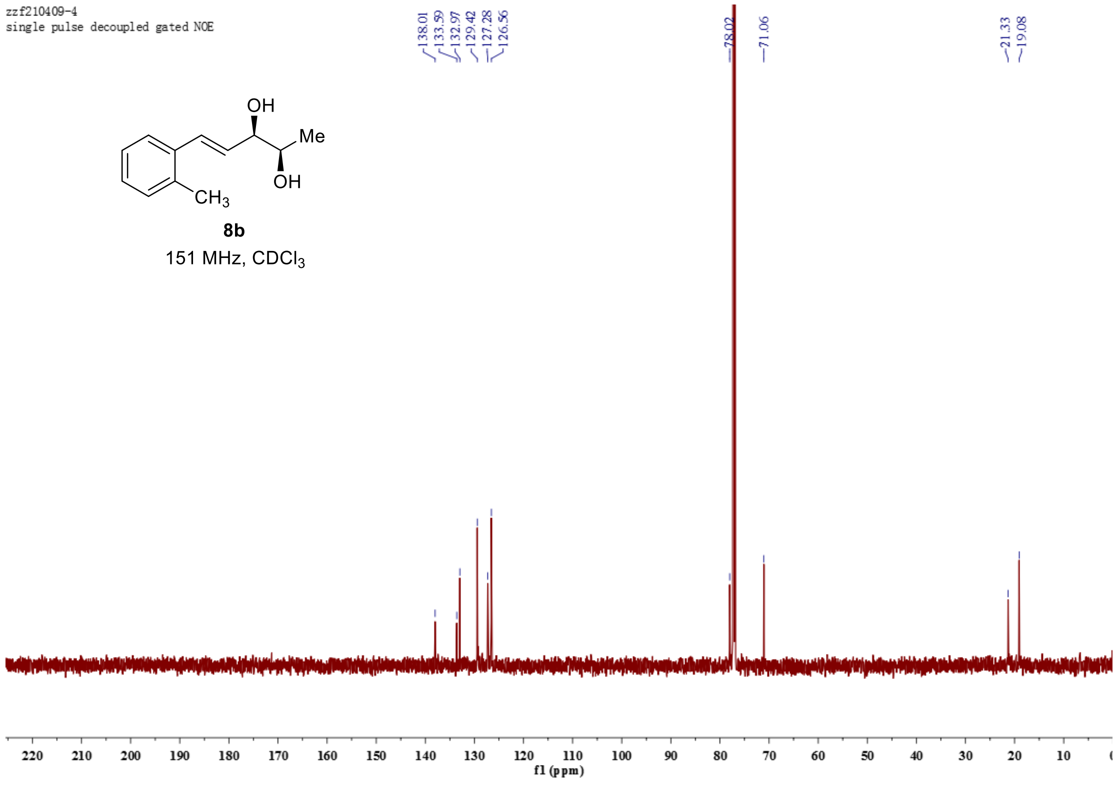
zzf210409-4
single pulse decoupled gated NOE
138.01
133.59
132.97
129.42
127.28
126.56
78.02
71.06
21.33
19.08
OH
Me
OH
CH_3
8b
151 MHz, $CDCl_3$
220
210
200
190
180
170
160
150
140
130
120
110
100
90
80
70
60
50
40
30
20
10
f1 (ppm)

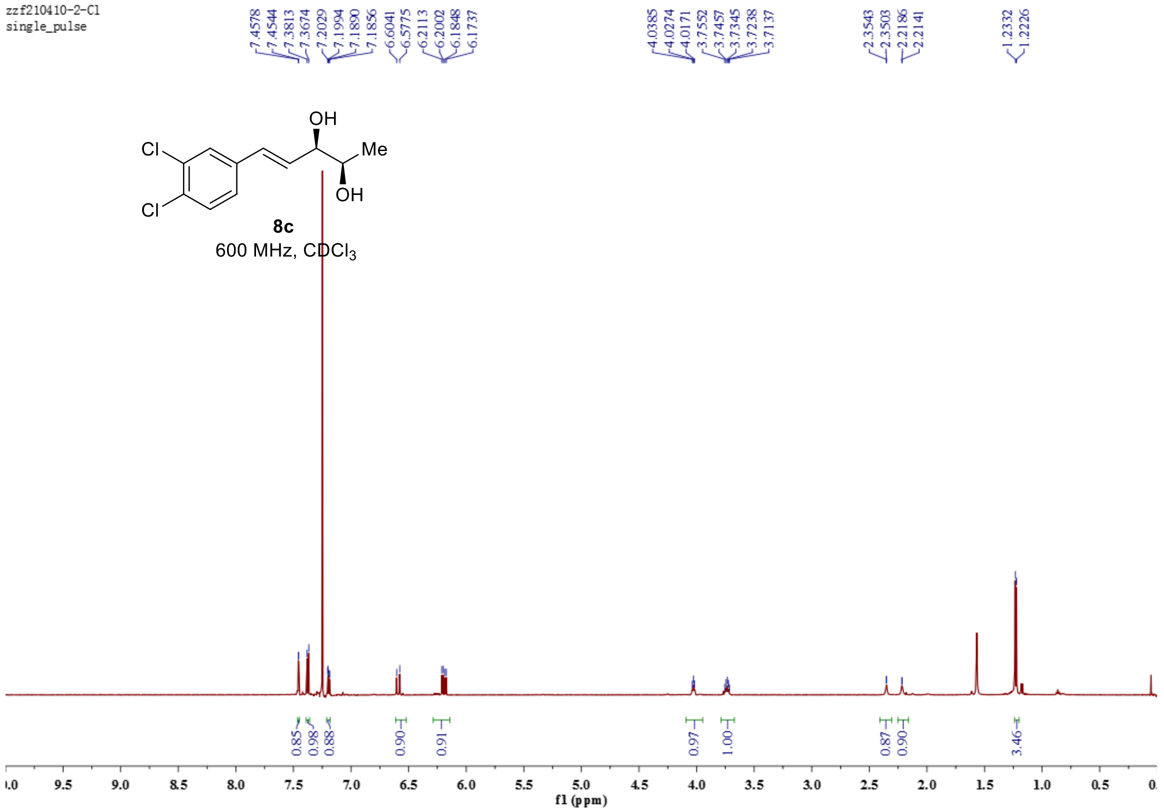
zzf210410-2-Cl
single_pulse
8c
600 MHz, CDCl3
f1 (ppm)

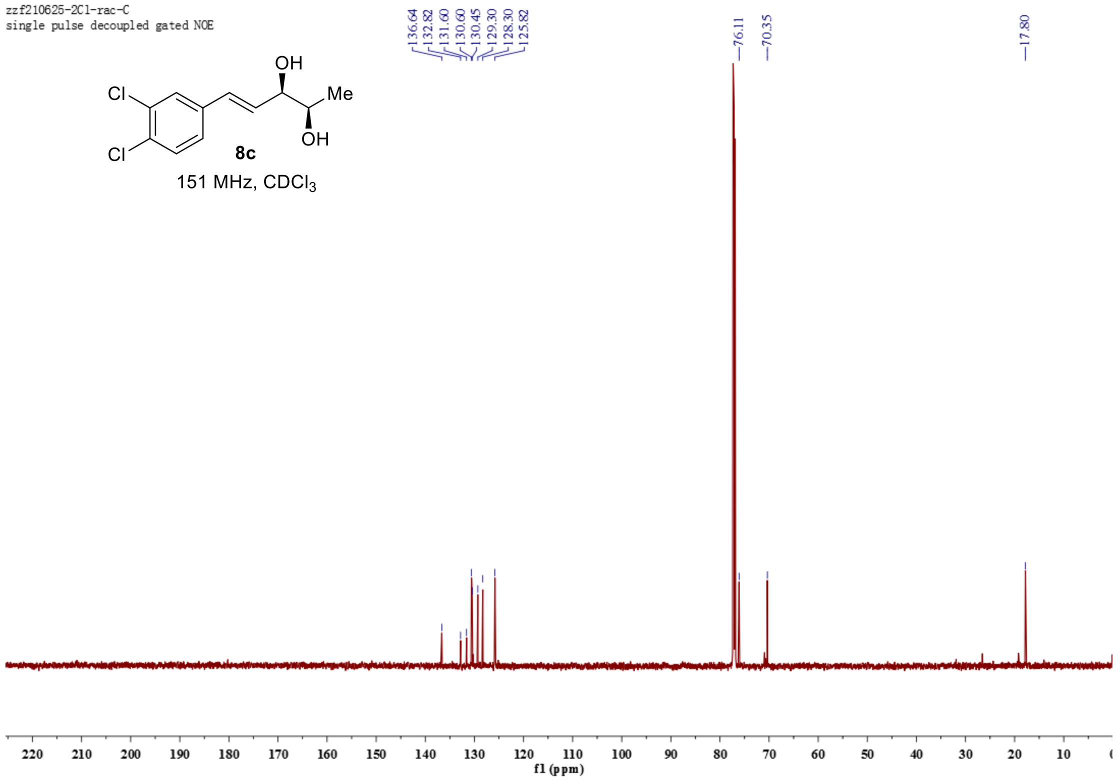
zzf210625-2Cl-rac-C
single pulse decoupled gated NOE
8c
151 MHz, CDCl3
f1 (ppm)

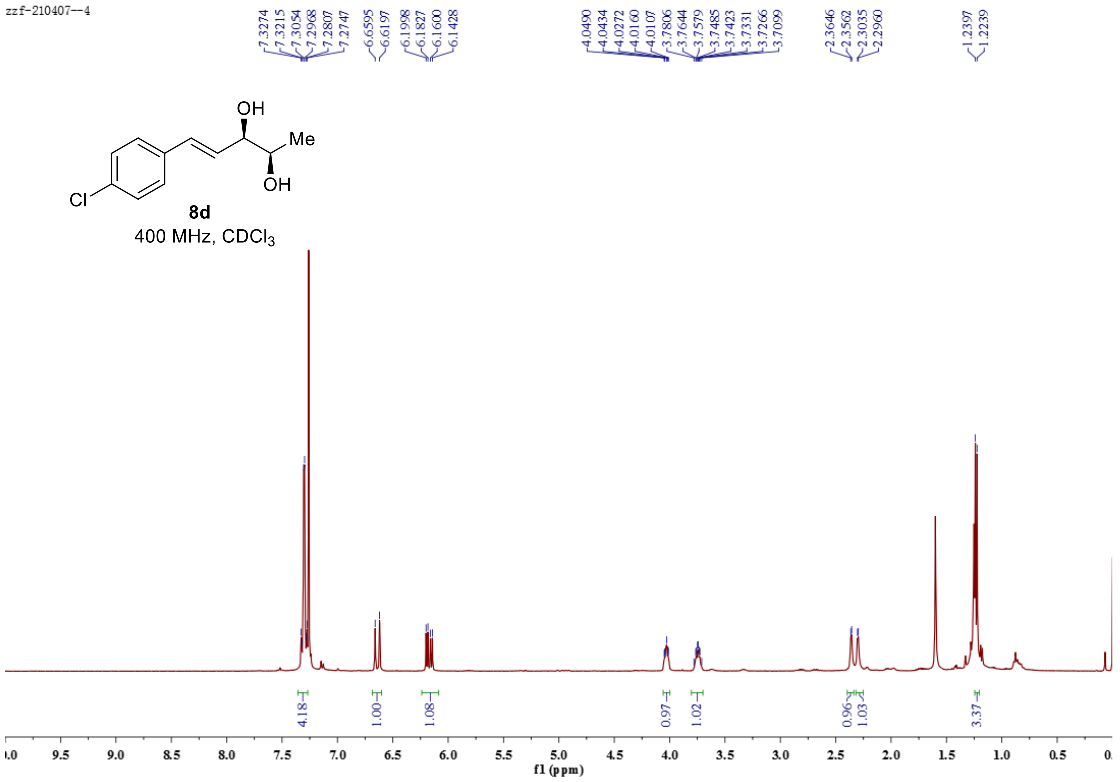
zzf-210407--4
8d
400 MHz, CDCl3
f1 (ppm)

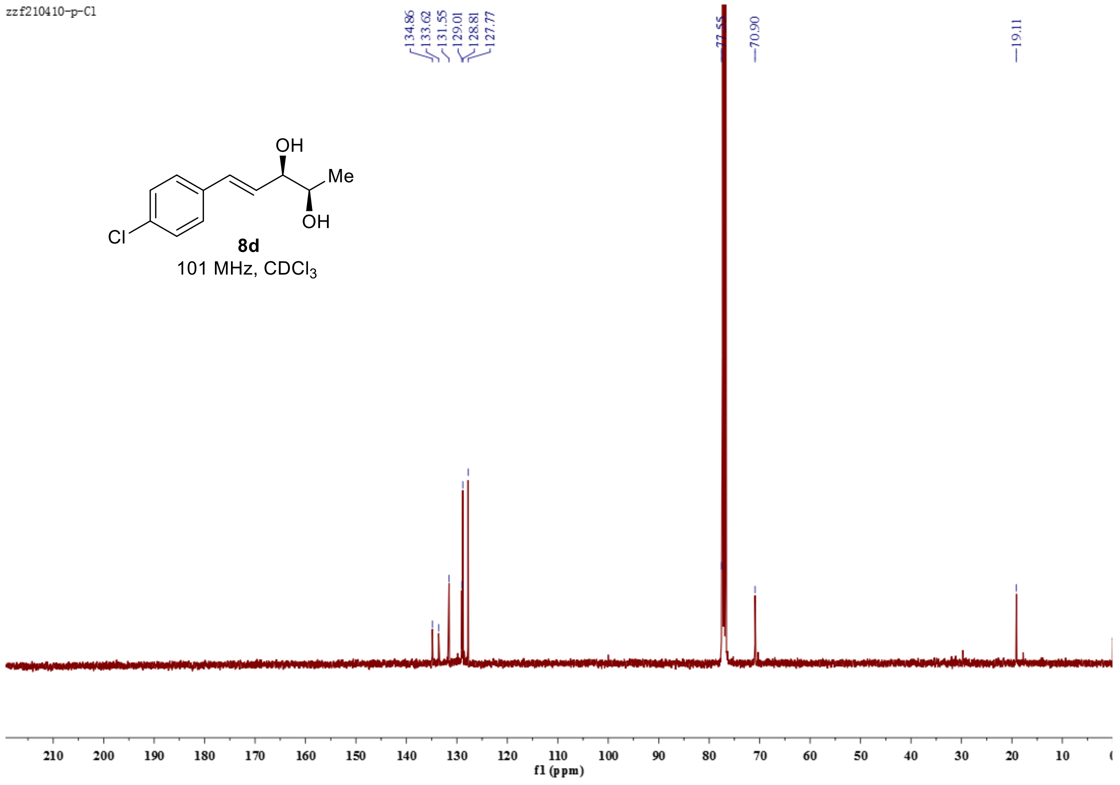
zzf210410-p-Cl
8d
101 MHz, CDCl3
f1 (ppm)

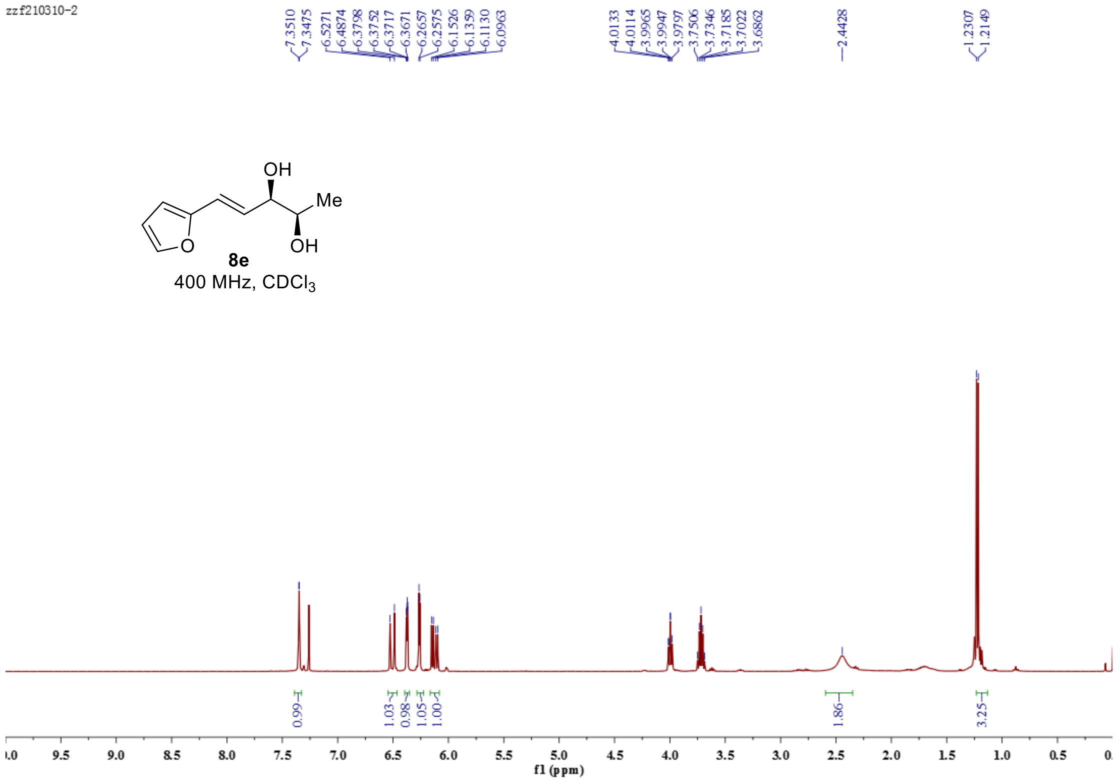
zzf210310-2
OH
Me
OH
O
8e
400 MHz, CDCl3
f1 (ppm)

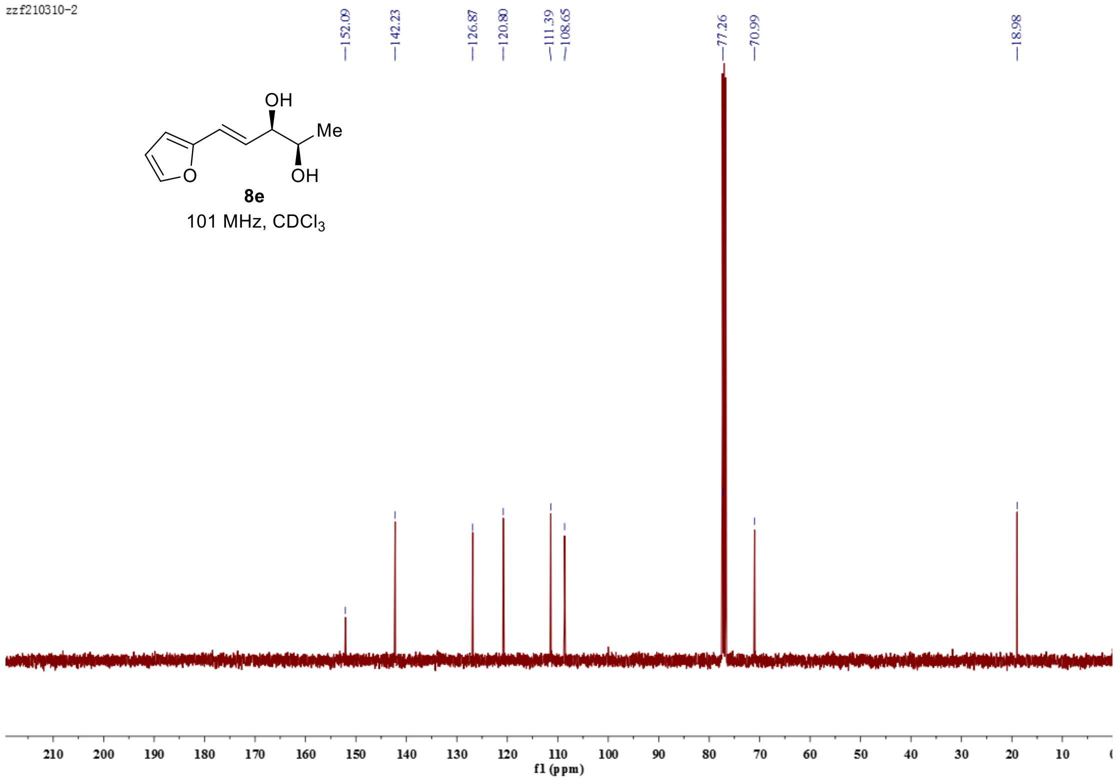
zzf210310-2
152.09
142.23
126.87
120.80
111.39
108.65
77.26
70.99
18.98
OH
Me
OH
O
8e
101 MHz, CDCl3
f1 (ppm)

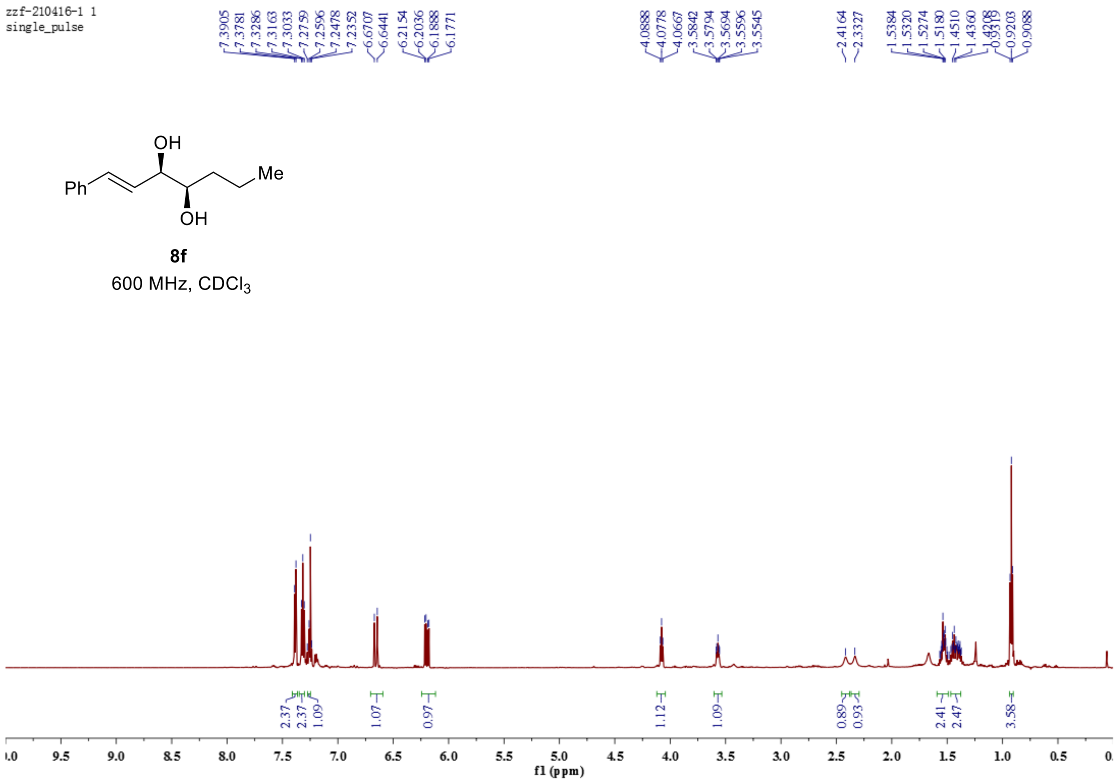
zzf-210416-1 1
single_pulse
7.3905
7.3781
7.3286
7.3163
7.3033
7.2759
7.2596
7.2478
7.2352
6.6707
6.6441
6.2154
6.2036
6.1888
6.1771
4.0888
4.0778
4.0667
3.5842
3.5794
3.5694
3.5596
3.5545
2.4164
2.3327
1.5384
1.5320
1.5274
1.5180
1.4510
1.4360
0.9319
0.9203
0.9088
OH
Ph
Me
OH
8f
600 MHz, CDCl3
2.37
2.37
1.09
1.07
0.97
1.12
1.09
0.89
0.93
2.41
2.47
3.58
9.5
9.0
8.5
8.0
7.5
7.0
6.5
6.0
5.5
5.0
4.5
4.0
3.5
3.0
2.5
2.0
1.5
1.0
0.5
f1 (ppm)

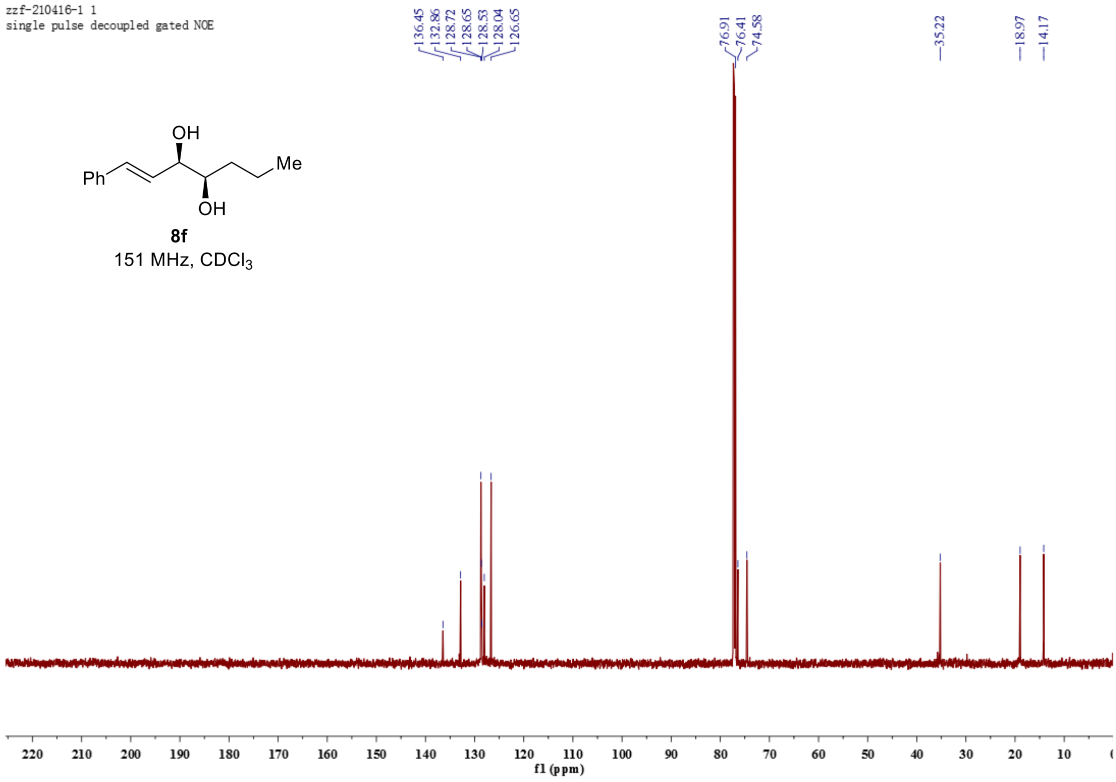
zzf-210416-1 1
single pulse decoupled gated NOE
136.45
132.86
128.72
128.65
128.53
128.04
126.65
76.91
76.41
74.58
35.22
18.97
14.17
OH
Ph
Me
OH
8f
151 MHz, CDCl3
220
210
200
190
180
170
160
150
140
130
120
110
100
90
80
70
60
50
40
30
20
10
f1 (ppm)

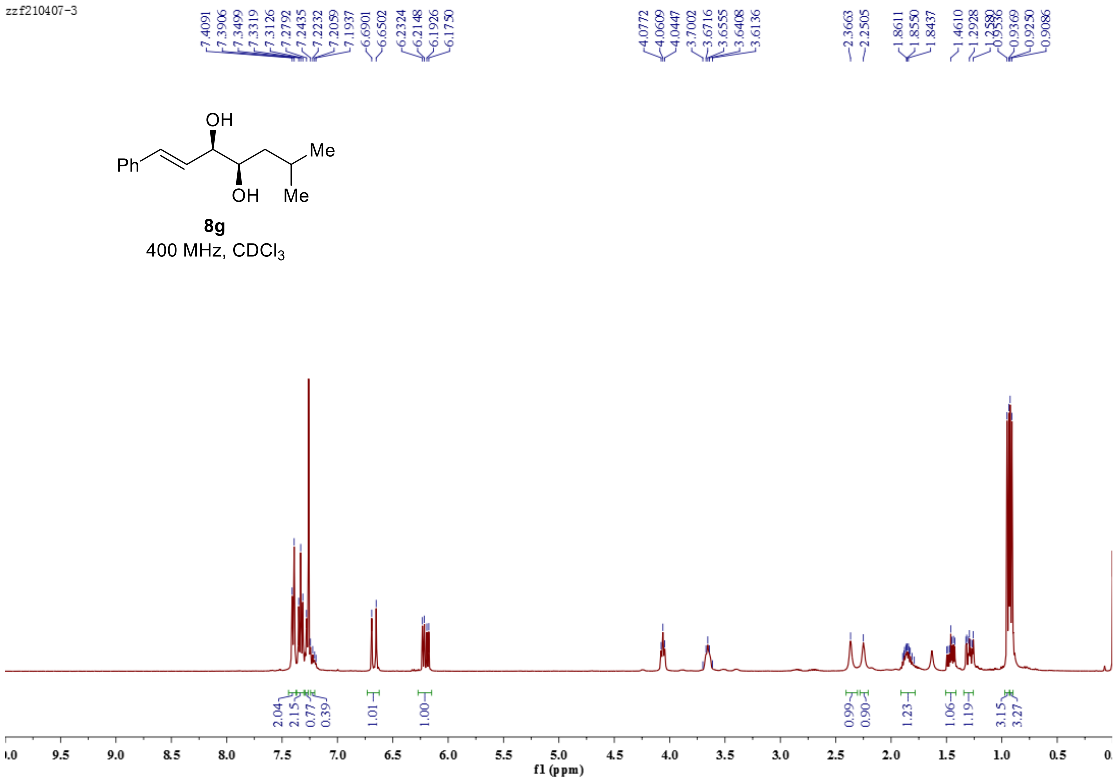
zzf210407-3
7.4091 7.3906 7.3499 7.3319 7.3126 7.2792 7.2435 7.2232 7.2059 7.1937 6.6901 6.6502 6.2324 6.2148 6.1926 6.1750
4.0772 4.0609 4.0447 3.7002 3.6716 3.6555 3.6408 3.6136
2.3663 2.2505 1.8611 1.8550 1.8437 1.4610 1.2928 1.2580 0.9536 0.9369 0.9250 0.9086
OH
Ph
Me
OH Me
8g
400 MHz, CDCl3
2.04 2.15 0.77 0.39 1.01 1.00 0.99 0.90 1.23 1.06 1.19 3.15 3.27
f1 (ppm)

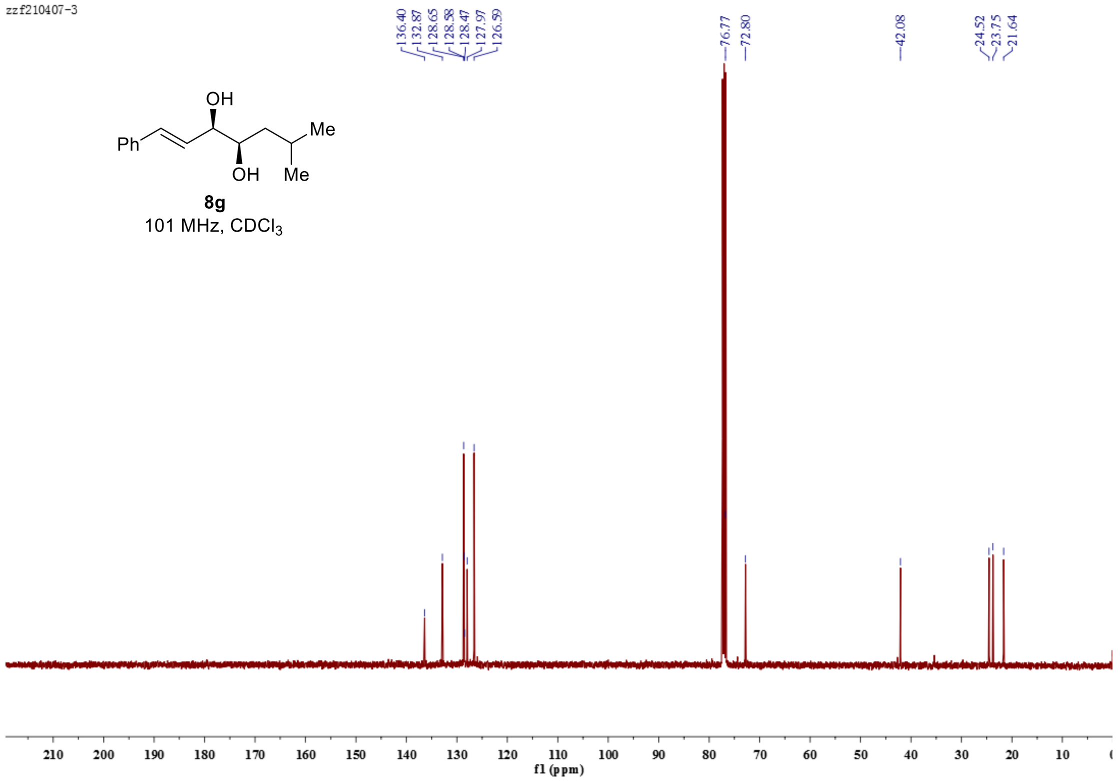
zzf210407-3
136.40 132.87 128.65 128.58 128.47 127.97 126.59
76.77 72.80
42.08
24.52 23.75 21.64
OH
Ph
Me
OH Me
8g
101 MHz, CDCl3
f1 (ppm)

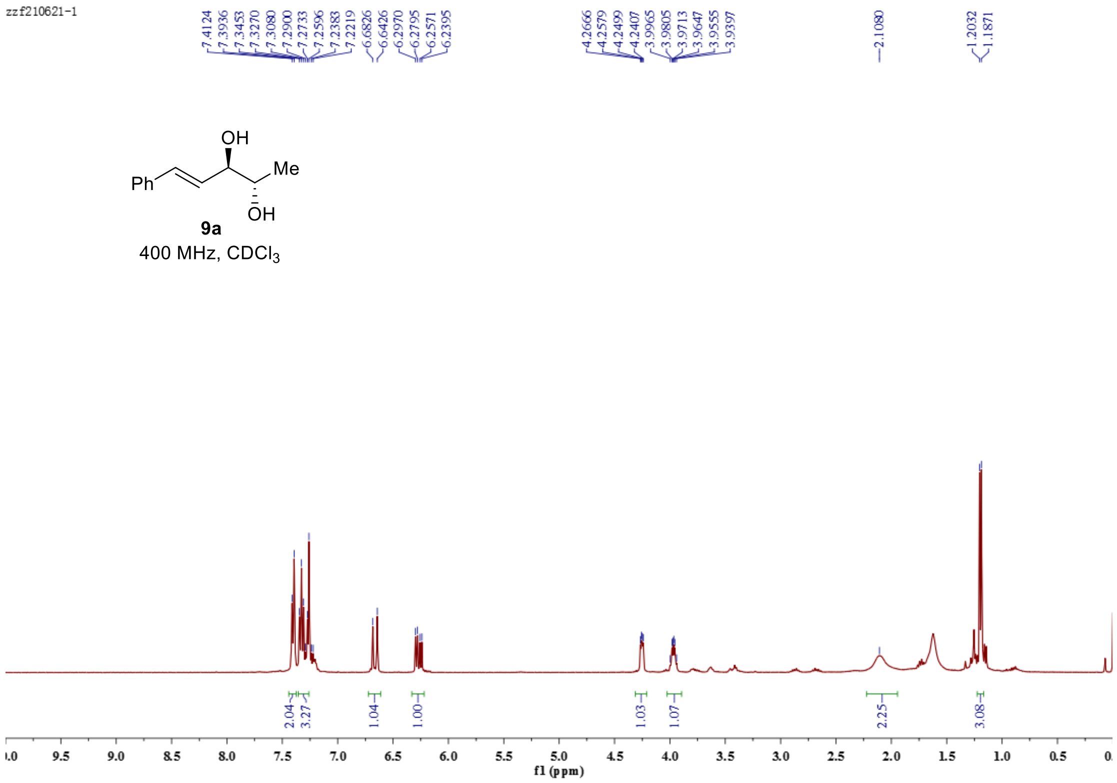
zzf210621-1
7.4124
7.3936
7.3453
7.3270
7.3080
7.2900
7.2733
7.2596
7.2383
7.2219
6.6826
6.6426
6.2970
6.2795
6.2571
6.2395
4.2666
4.2579
4.2499
4.2407
3.9965
3.9805
3.9713
3.9647
3.9555
3.9397
2.1080
1.2032
1.1871
OH
Ph
Me
OH
9a
400 MHz, CDCl3
2.04
3.27
1.04
1.00
1.03
1.07
2.25
3.08
9.5
9.0
8.5
8.0
7.5
7.0
6.5
6.0
5.5
5.0
4.5
4.0
3.5
3.0
2.5
2.0
1.5
1.0
0.5
f1 (ppm)

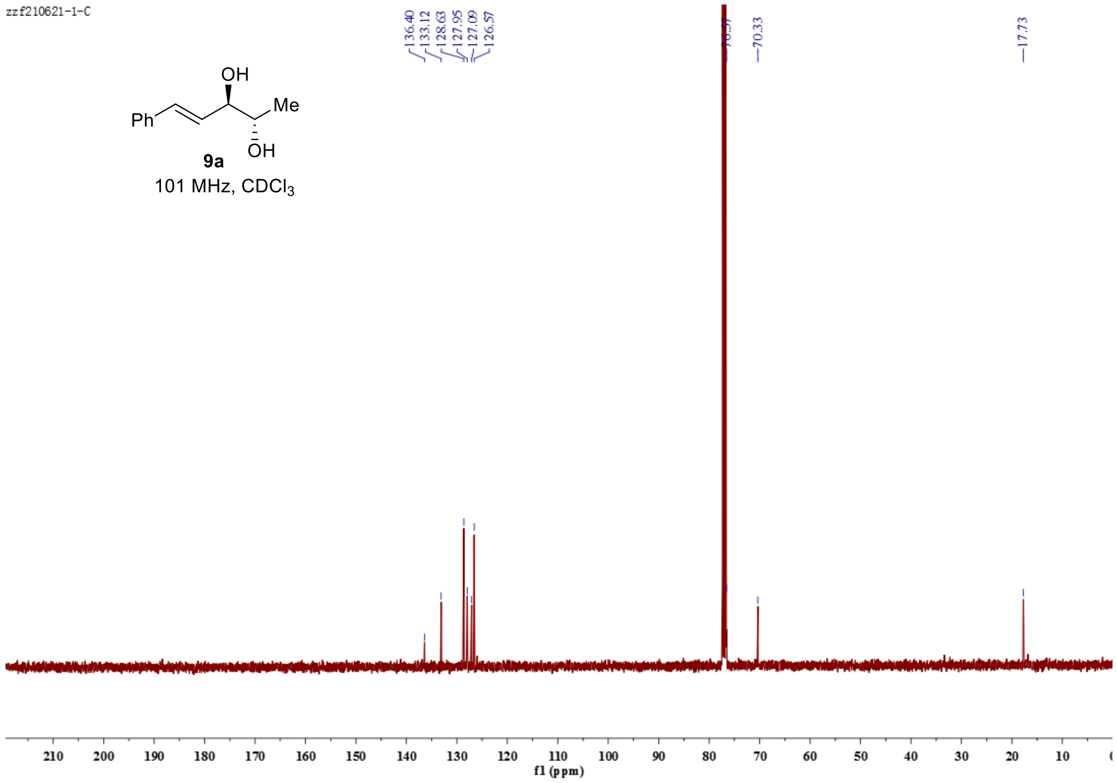
zzf210621-1-C
136.40
133.12
128.63
127.95
127.09
126.57
70.33
17.73
OH
Ph
Me
OH
9a
101 MHz, CDCl3
210
200
190
180
170
160
150
140
130
120
110
100
90
80
70
60
50
40
30
20
10
f1 (ppm)

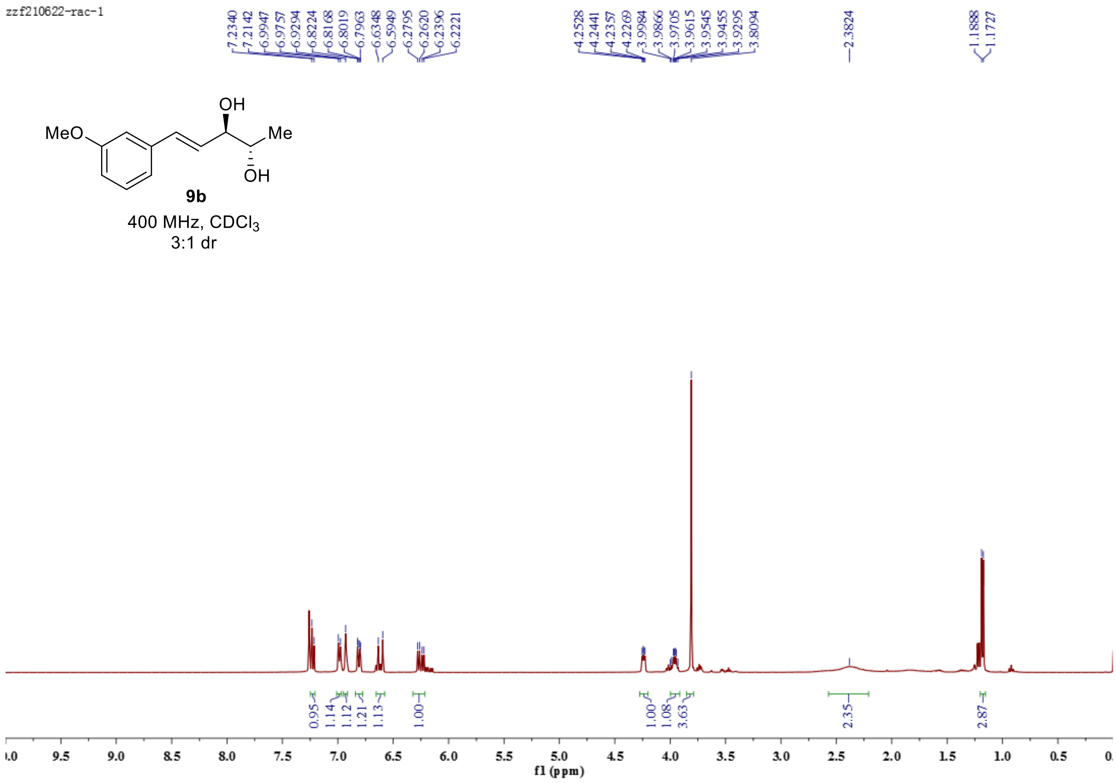
zzf210622-rac-1
7.2340
7.2142
6.9947
6.9757
6.9294
6.8224
6.8168
6.8019
6.7963
6.6348
6.5949
6.2795
6.2620
6.2396
6.2221
4.2528
4.2441
4.2357
4.2269
3.9984
3.9866
3.9705
3.9615
3.9545
3.9455
3.9295
3.8094
2.3824
1.1888
1.1727
OH
MeO
Me
OH
9b
400 MHz, $CDCl_3$
3:1 dr
0.95
1.14
1.12
1.21
1.13
1.00
1.00
1.08
3.63
2.35
2.87
f1 (ppm)

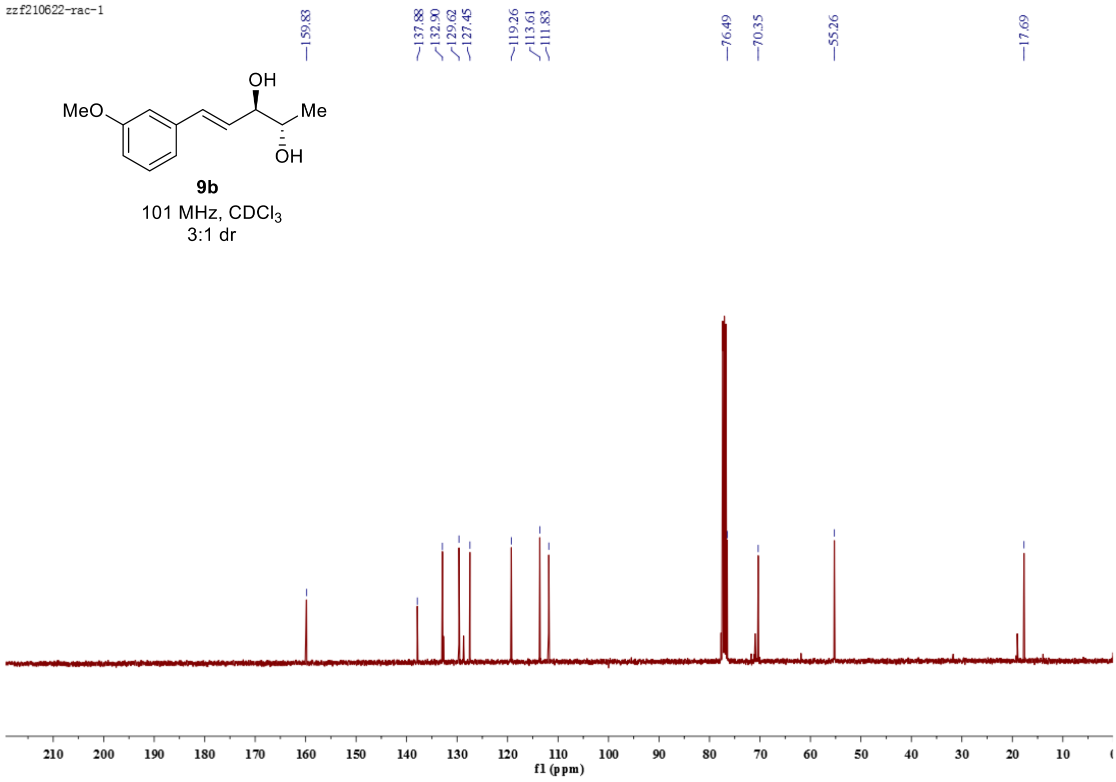
zzf210622-rac-1
159.83
137.88
132.90
129.62
127.45
119.26
113.61
111.83
76.49
70.35
55.26
17.69
OH
MeO
Me
OH
9b
101 MHz, $CDCl_3$
3:1 dr
f1 (ppm)

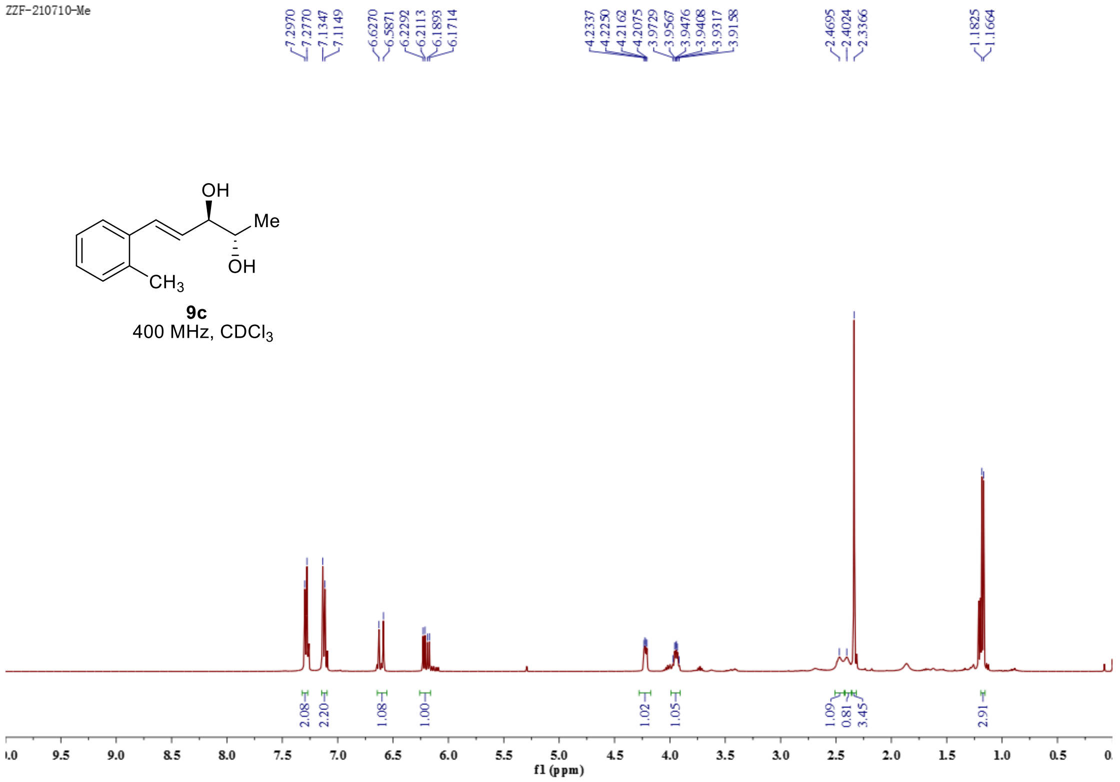
ZZF-210710-Me
7.2970
7.2770
7.1347
7.1149
6.6270
6.5871
6.2292
6.2113
6.1893
6.1714
4.2337
4.2250
4.2162
4.2075
3.9729
3.9567
3.9476
3.9408
3.9317
3.9158
2.4695
2.4024
2.3366
1.1825
1.1664
OH
Me
OH
CH3
9c
400 MHz, CDCl3
2.08
2.20
1.08
1.00
1.02
1.05
1.09
0.81
3.45
2.91
f1 (ppm)

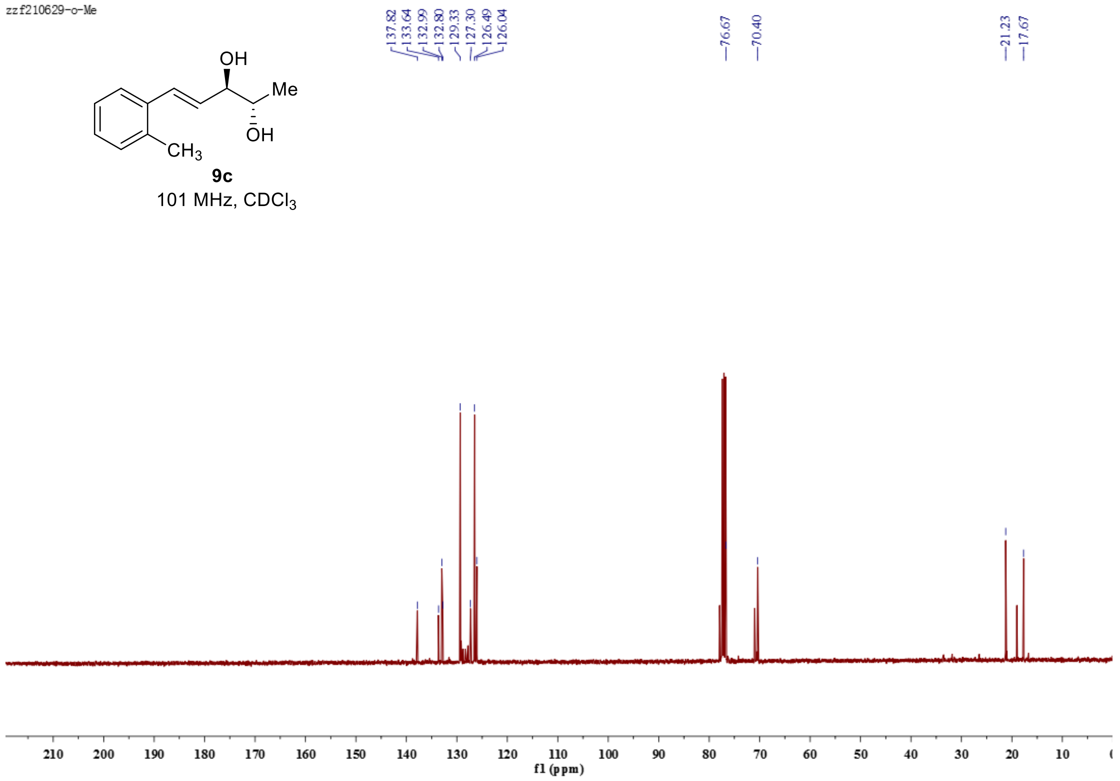
zzf210629-o-Me
137.82
133.64
132.99
132.80
129.33
127.30
126.49
126.04
76.67
70.40
21.23
17.67
OH
Me
OH
CH3
9c
101 MHz, CDCl3
f1 (ppm)

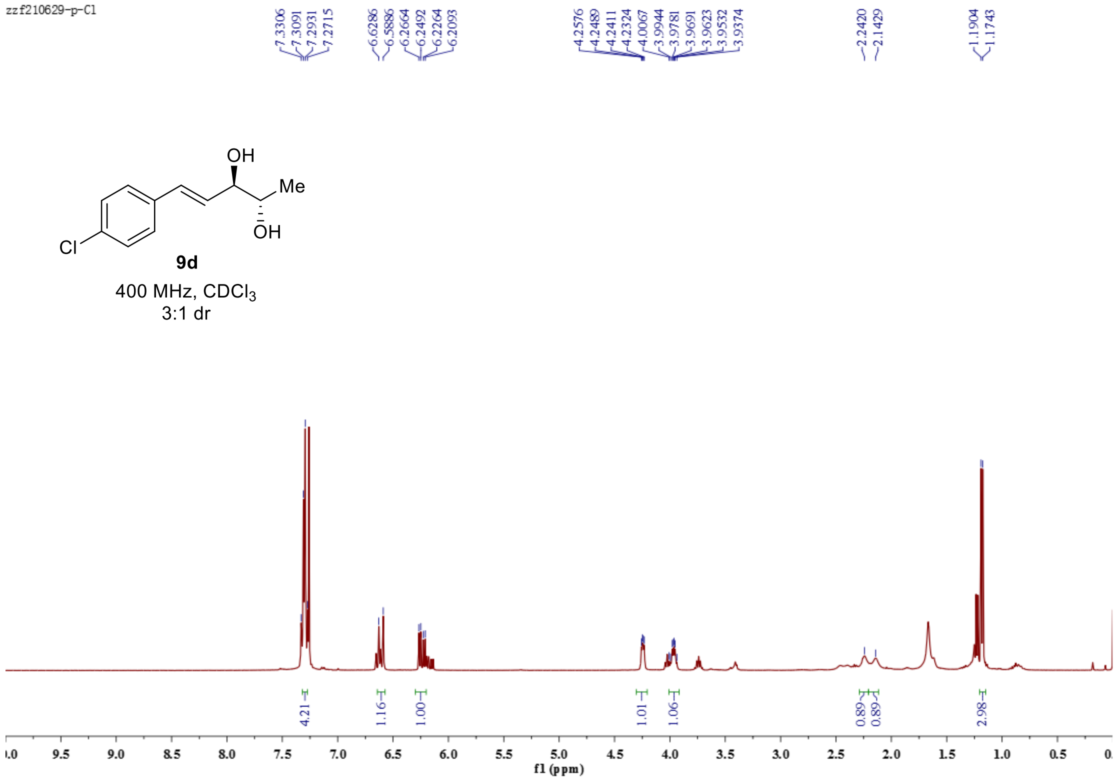
zzf210629-p-Cl
7.3306
7.3091
7.2931
7.2715
6.6286
6.5886
6.2664
6.2492
6.2264
6.2093
4.2576
4.2489
4.2411
4.2324
4.0067
3.9944
3.9781
3.9691
3.9623
3.9532
3.9374
2.2420
2.1429
1.1904
1.1743
OH
Me
OH
Cl
9d
400 MHz, CDCl3
3:1 dr
4.21
1.16
1.00
1.01
1.06
0.89
0.89
2.98
f1 (ppm)

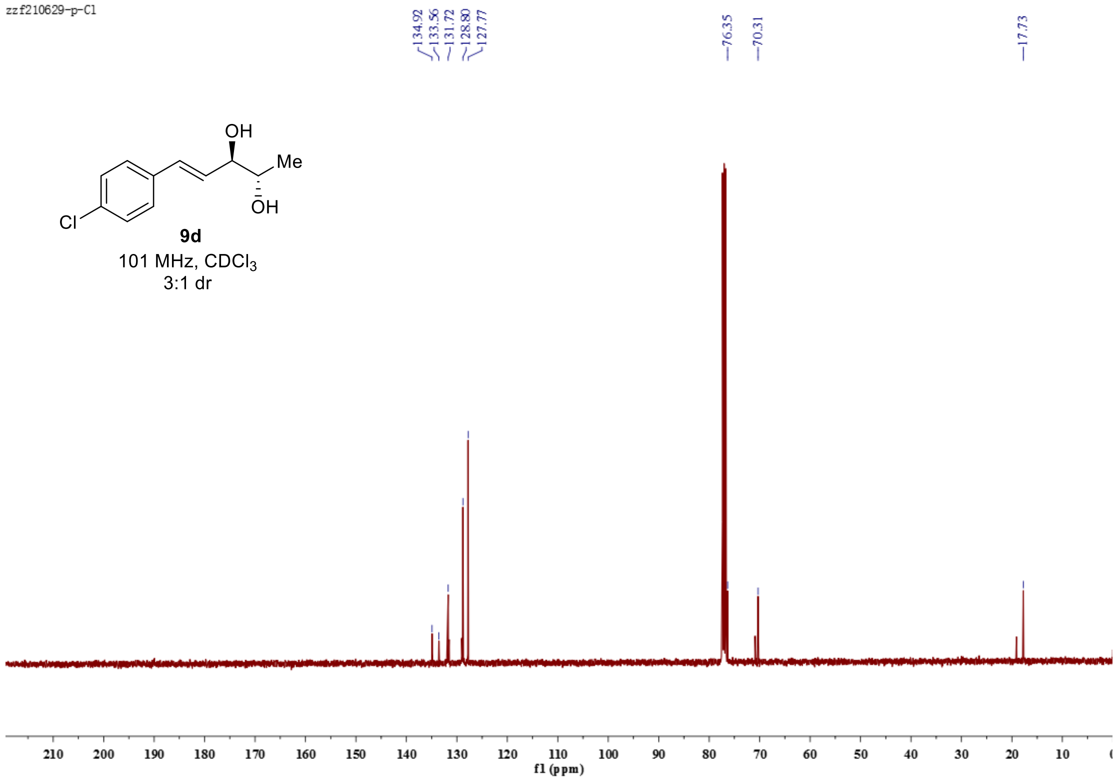
zzf210629-p-Cl
134.92
133.56
131.72
128.80
127.77
76.35
70.31
17.73
OH
Me
OH
Cl
9d
101 MHz, CDCl3
3:1 dr
f1 (ppm)

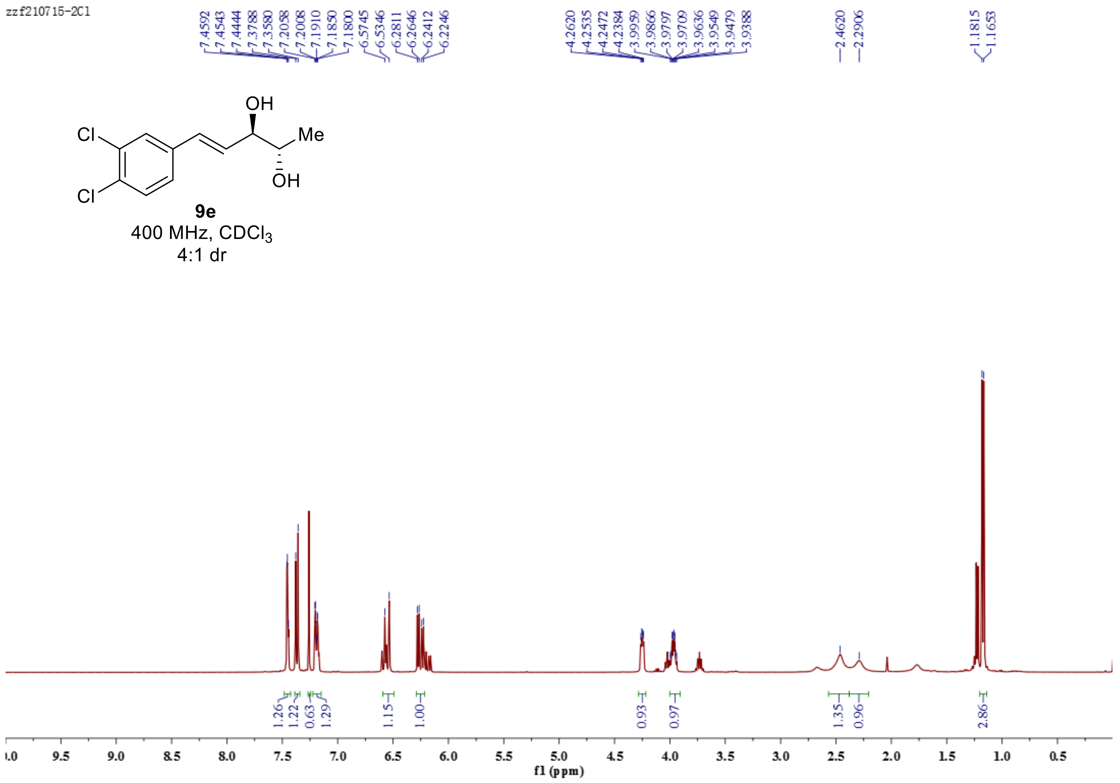
zzf210715-2Cl
7.4592
7.4543
7.4444
7.3788
7.3580
7.2058
7.2008
7.1910
7.1850
7.1800
6.5745
6.5346
6.2811
6.2646
6.2412
6.2246
4.2620
4.2535
4.2472
4.2384
3.9959
3.9866
3.9797
3.9709
3.9636
3.9549
3.9479
3.9388
2.4620
2.2906
1.1815
1.1653
OH
Cl
Me
Cl
OH
9e
400 MHz, CDCl$_3$
4:1 dr
1.26
1.22
0.63
1.29
1.15
1.00
0.93
0.97
1.35
0.96
2.86
f1 (ppm)

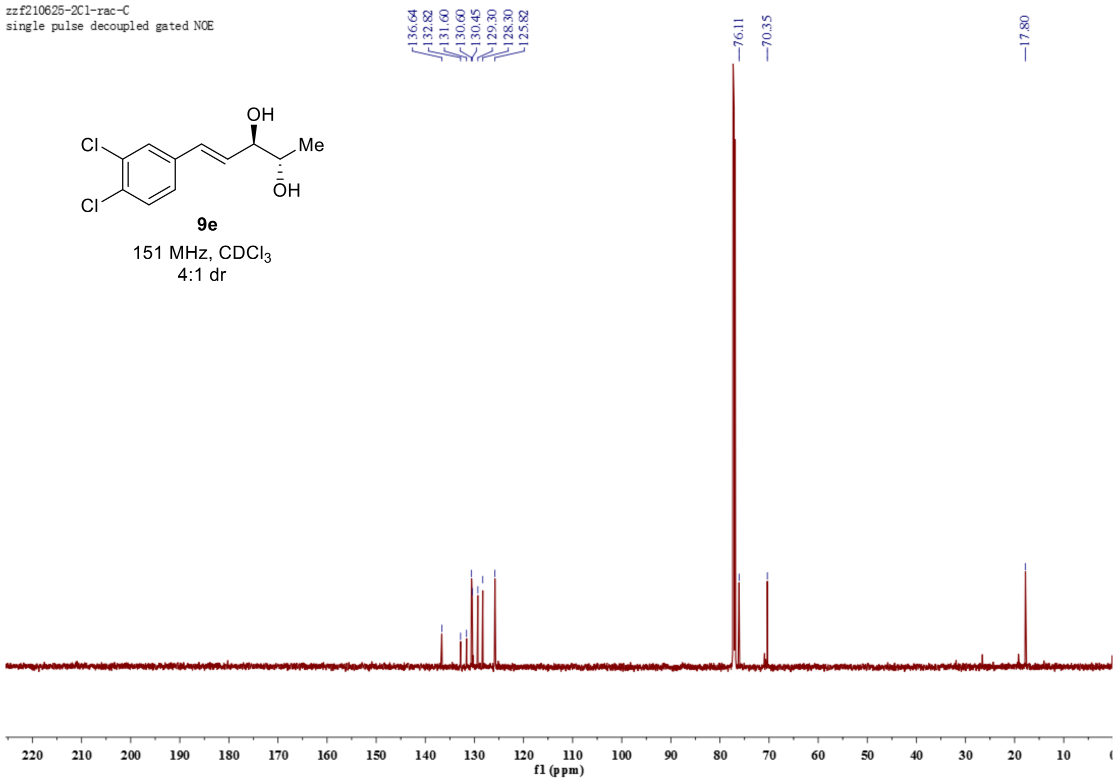
zzf210625-2Cl-rac-C
single pulse decoupled gated NOE
136.64
132.82
131.60
130.60
130.45
129.30
128.30
125.82
76.11
70.35
17.80
OH
Cl
Me
Cl
OH
9e
151 MHz, CDCl$_3$
4:1 dr
f1 (ppm)

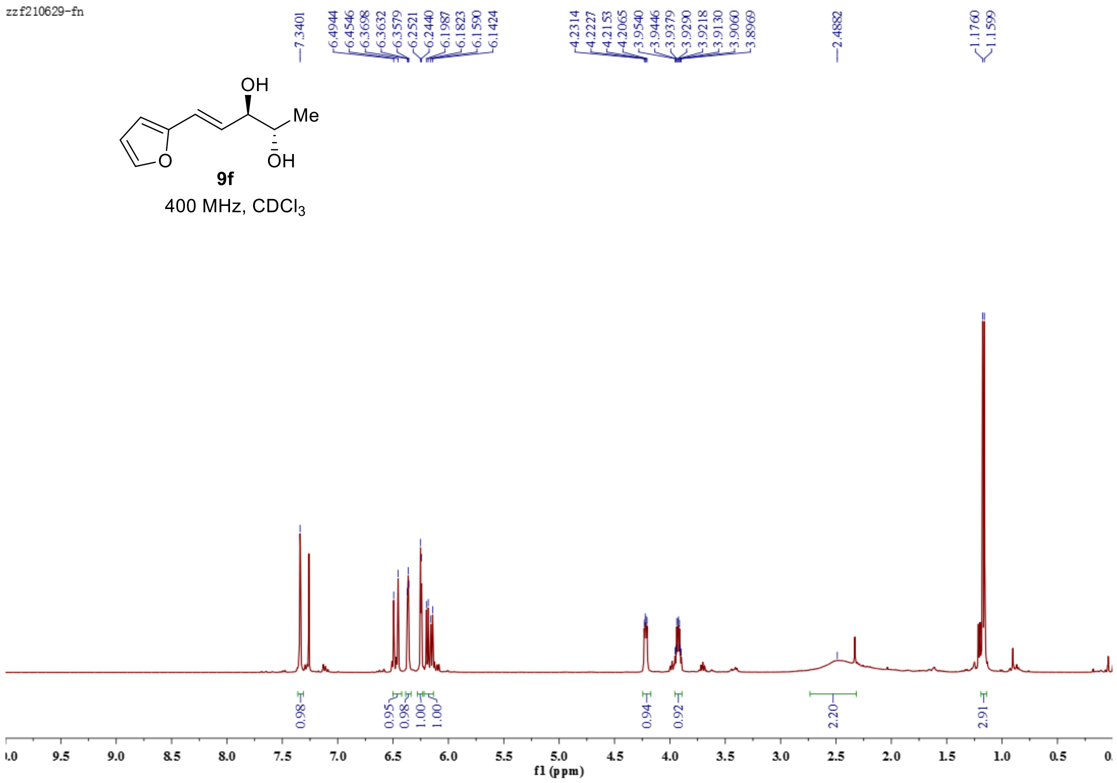
zzf210629-fn
7.3401
6.4944
6.4546
6.3698
6.3632
6.3579
6.2521
6.2440
6.1987
6.1823
6.1590
6.1424
4.2314
4.2227
4.2153
4.2065
3.9540
3.9446
3.9379
3.9290
3.9218
3.9130
3.9060
3.8969
2.4882
1.1760
1.1599
OH
Me
O
OH
9f
400 MHz, CDCl3
0.98
0.95
0.98
1.00
1.00
0.94
0.92
2.20
2.91
f1 (ppm)

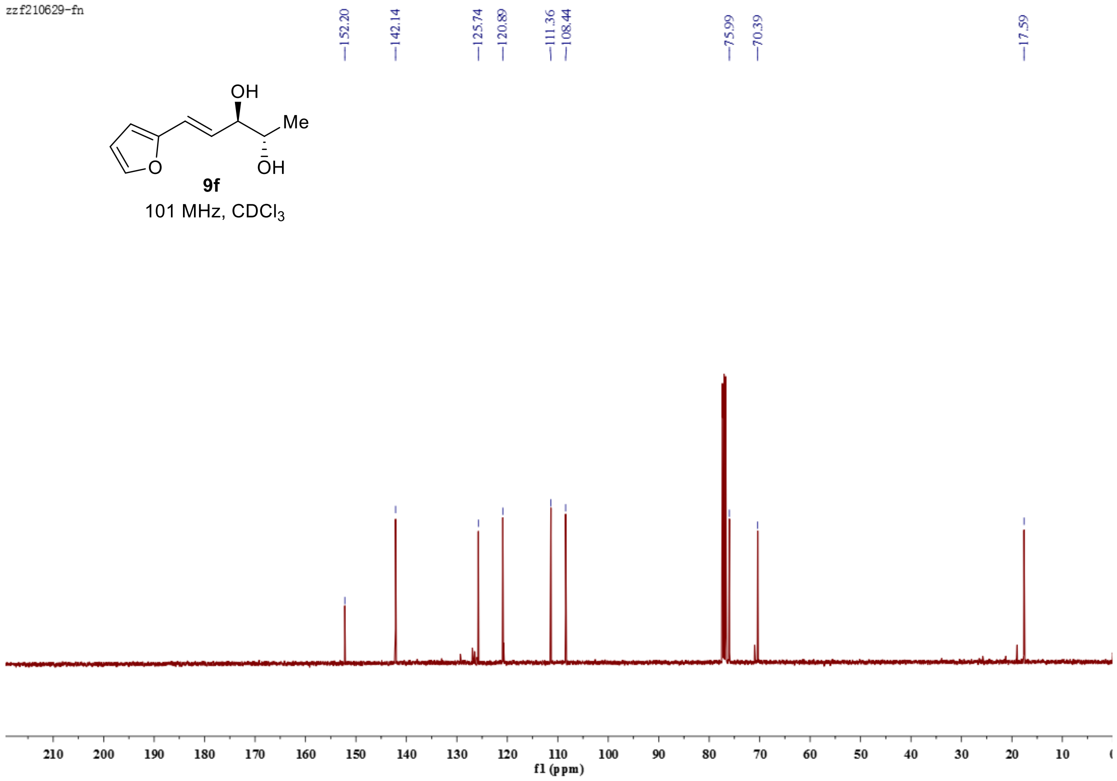
zzf210629-fn
152.20
142.14
125.74
120.89
111.36
108.44
75.99
70.39
17.59
OH
Me
O
OH
9f
101 MHz, CDCl3
f1 (ppm)

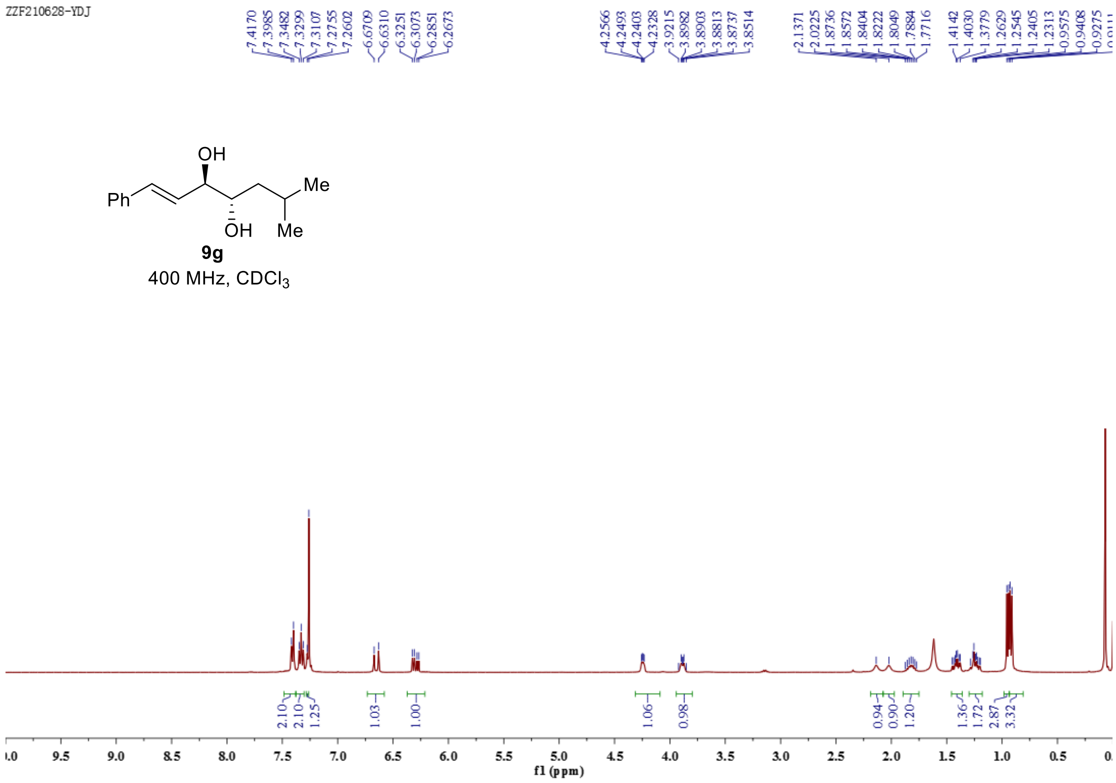

9g

400 MHz, $CDCl_3$

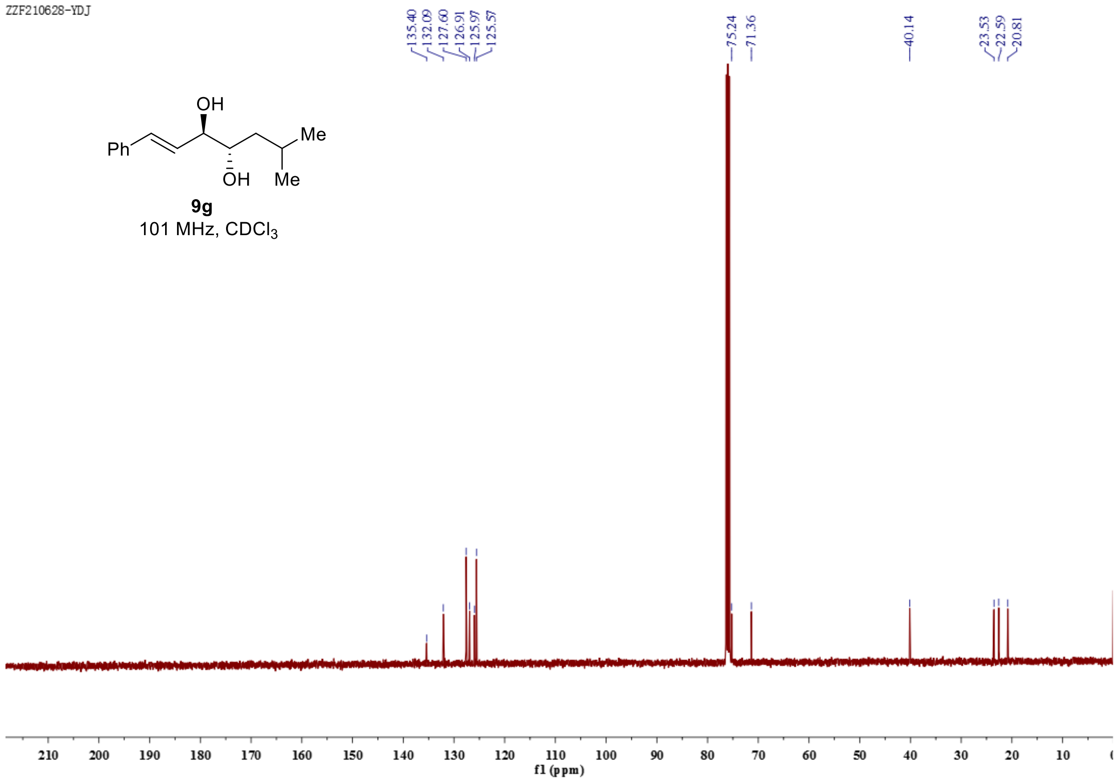

9g

101 MHz, $CDCl_3$

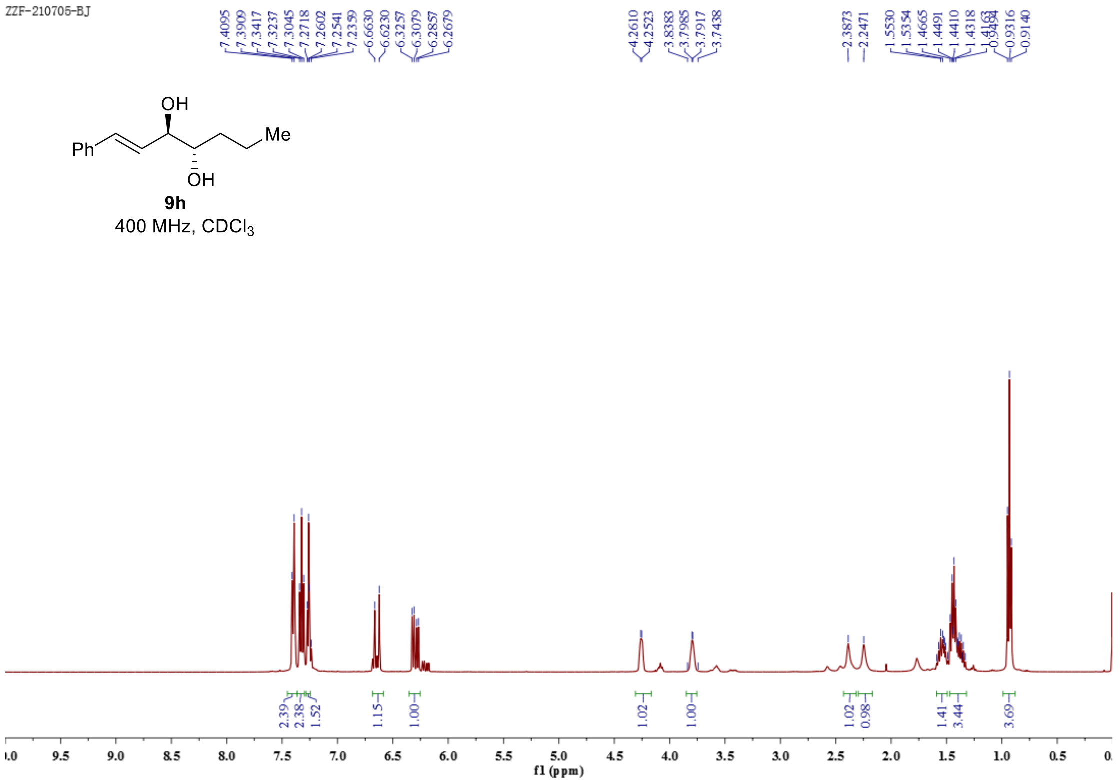
ZZF-210705-BJ
9h
400 MHz, CDCl3
f1 (ppm)

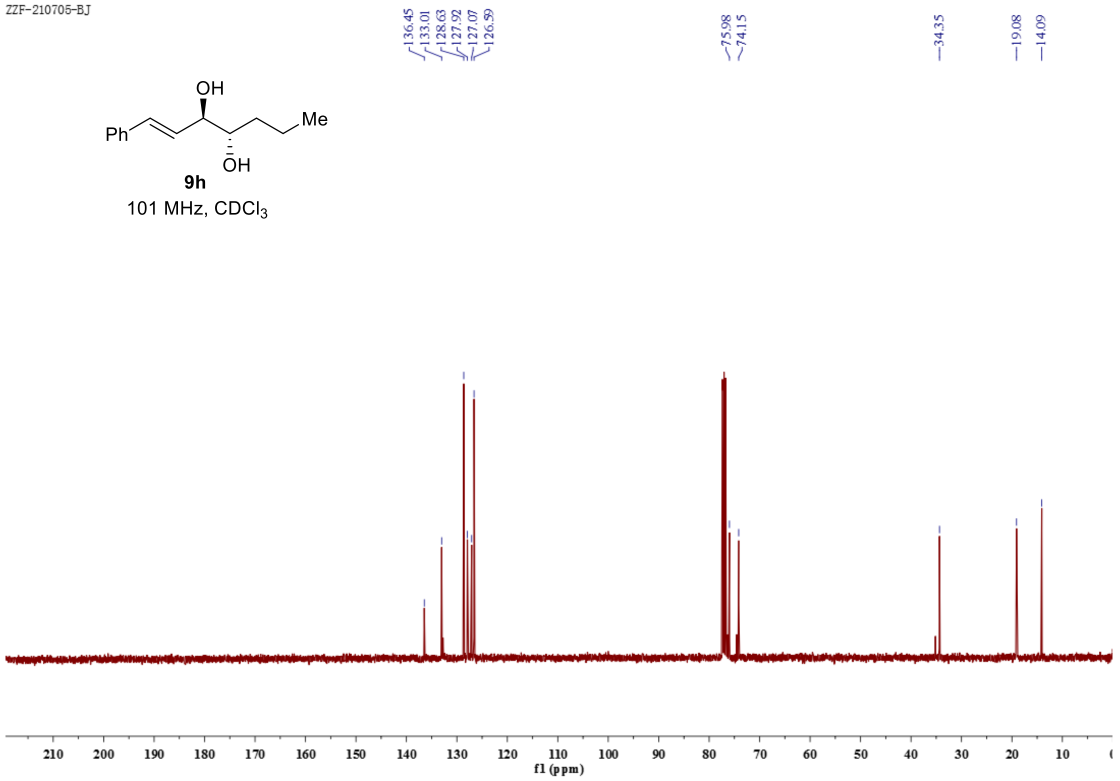
ZZF-210705-BJ
9h
101 MHz, CDCl3
f1 (ppm)

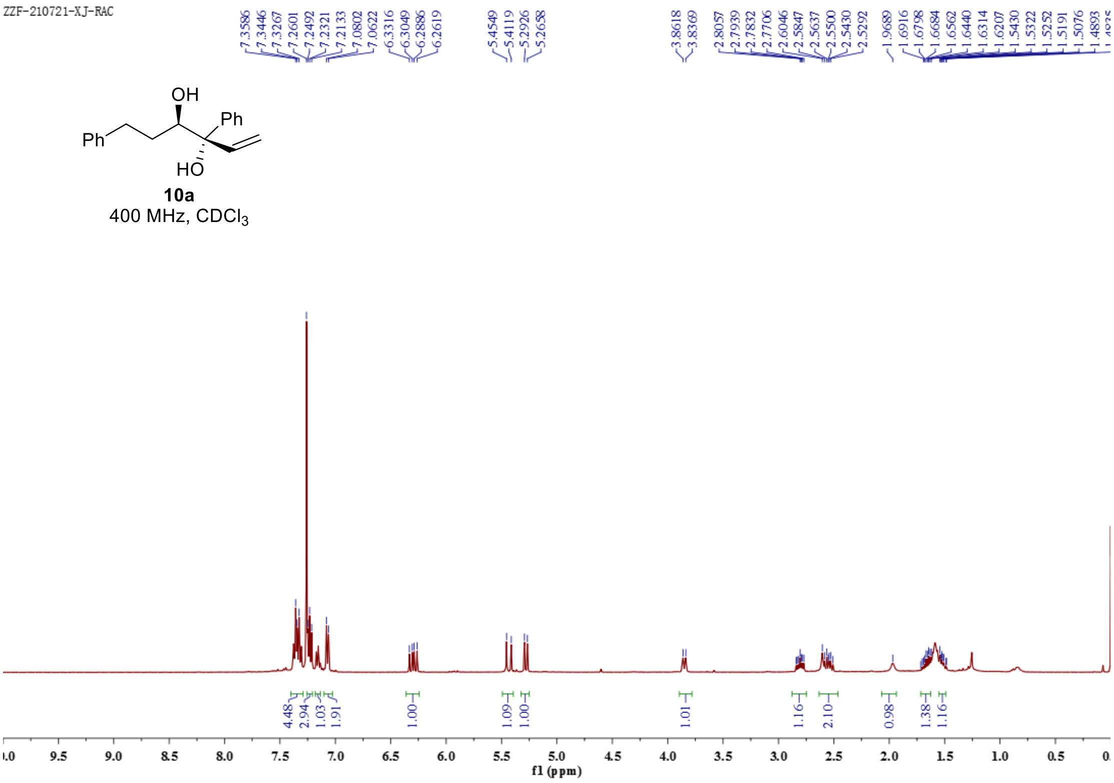
ZZF-210721-XJ-RAC
7.3586
7.3446
7.3267
7.2601
7.2492
7.2321
7.2133
7.0802
7.0622
6.3316
6.3049
6.2886
6.2619
5.4549
5.4119
5.2926
5.2658
3.8618
3.8369
2.8057
2.7939
2.7832
2.7706
2.6046
2.5847
2.5637
2.5500
2.5430
2.5292
1.9689
1.6916
1.6798
1.6684
1.6562
1.6440
1.6314
1.6207
1.5430
1.5322
1.5252
1.5191
1.5076
1.4893
OH
Ph
Ph
HO
10a
400 MHz, CDCl3
4.48
2.94
1.03
1.91
1.00
1.09
1.00
1.01
1.16
2.10
0.98
1.38
1.16
9.5
9.0
8.5
8.0
7.5
7.0
6.5
6.0
5.5
5.0
4.5
4.0
3.5
3.0
2.5
2.0
1.5
1.0
0.5
f1 (ppm)

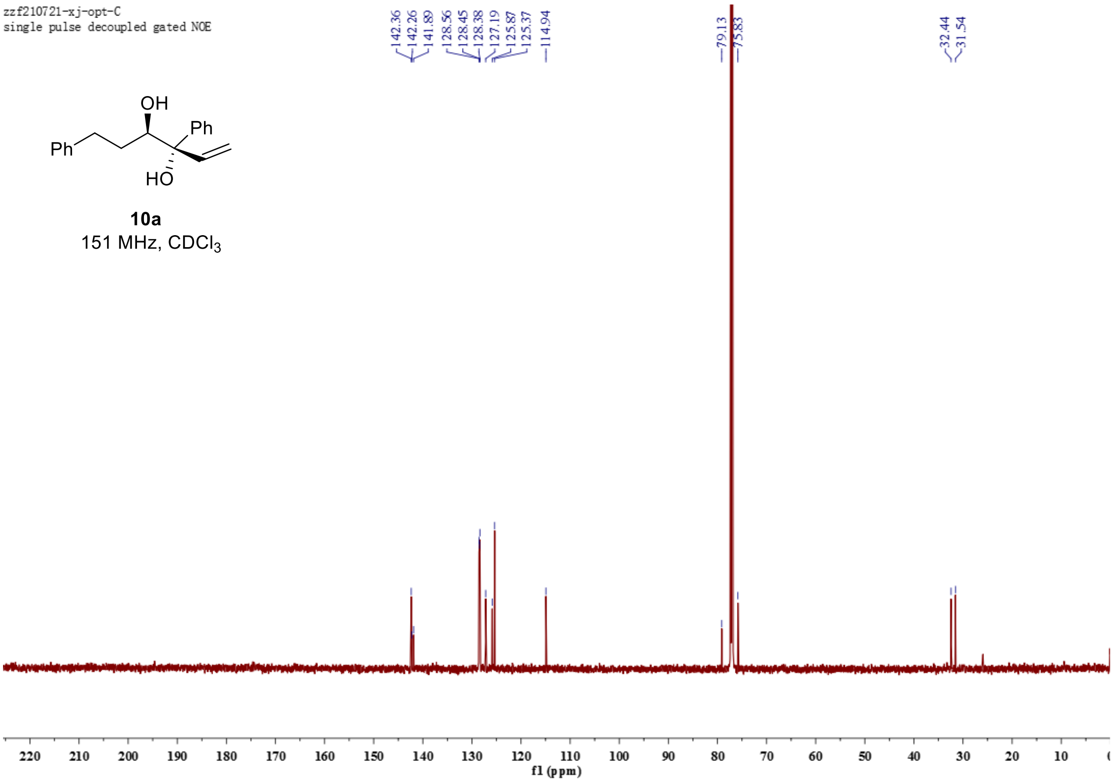
zzf210721-xj-opt-C
single pulse decoupled gated NOE
142.36
142.26
141.89
128.56
128.45
128.38
127.19
125.87
125.37
114.94
79.13
75.83
32.44
31.54
OH
Ph
Ph
HO
10a
151 MHz, CDCl3
220
210
200
190
180
170
160
150
140
130
120
110
100
90
80
70
60
50
40
30
20
10
f1 (ppm)

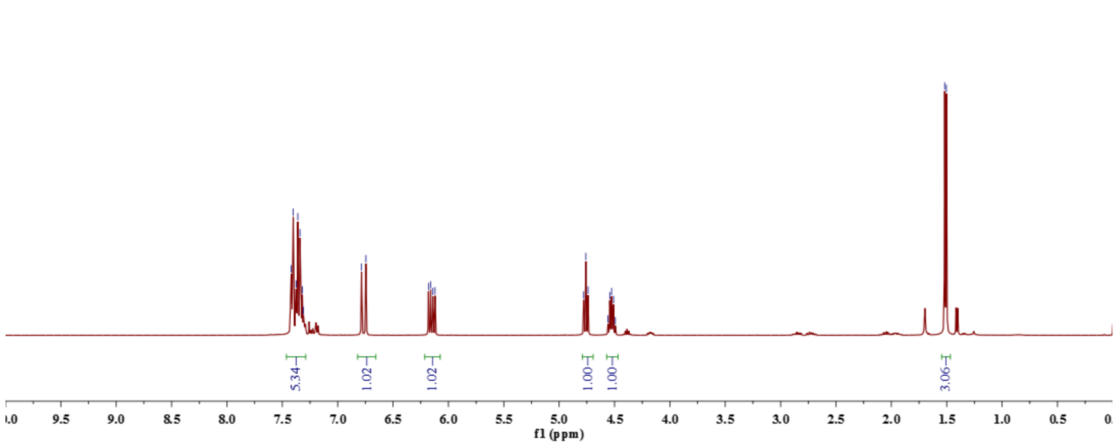

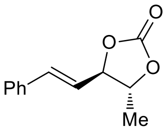

10c

400 MHz, $CDCl_3$

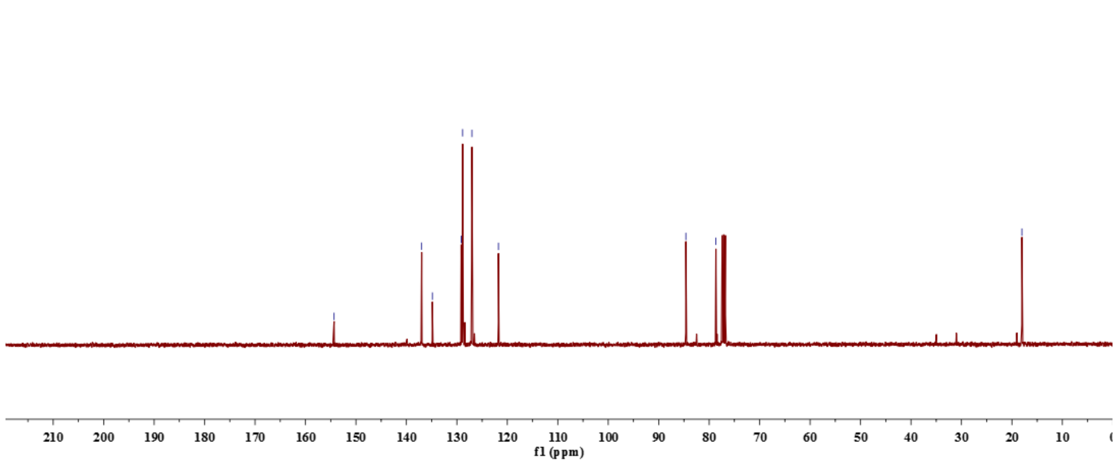

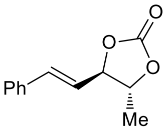

10c

101 MHz, $CDCl_3$

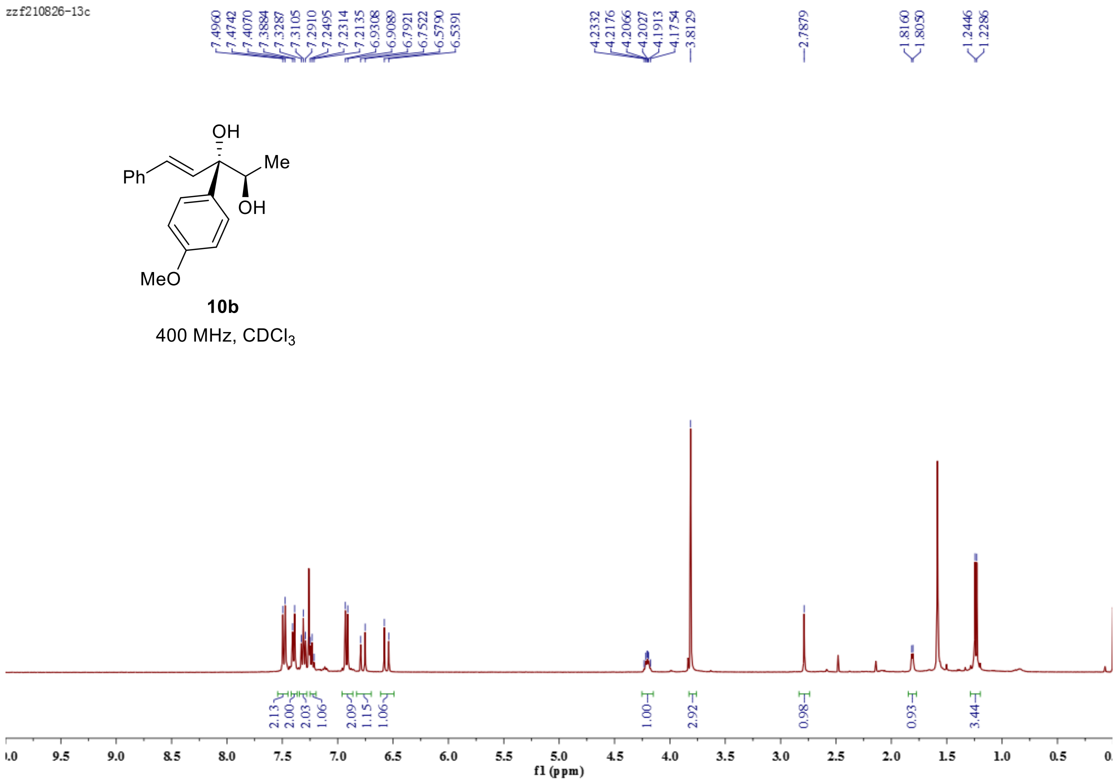

10b

400 MHz, $CDCl_3$

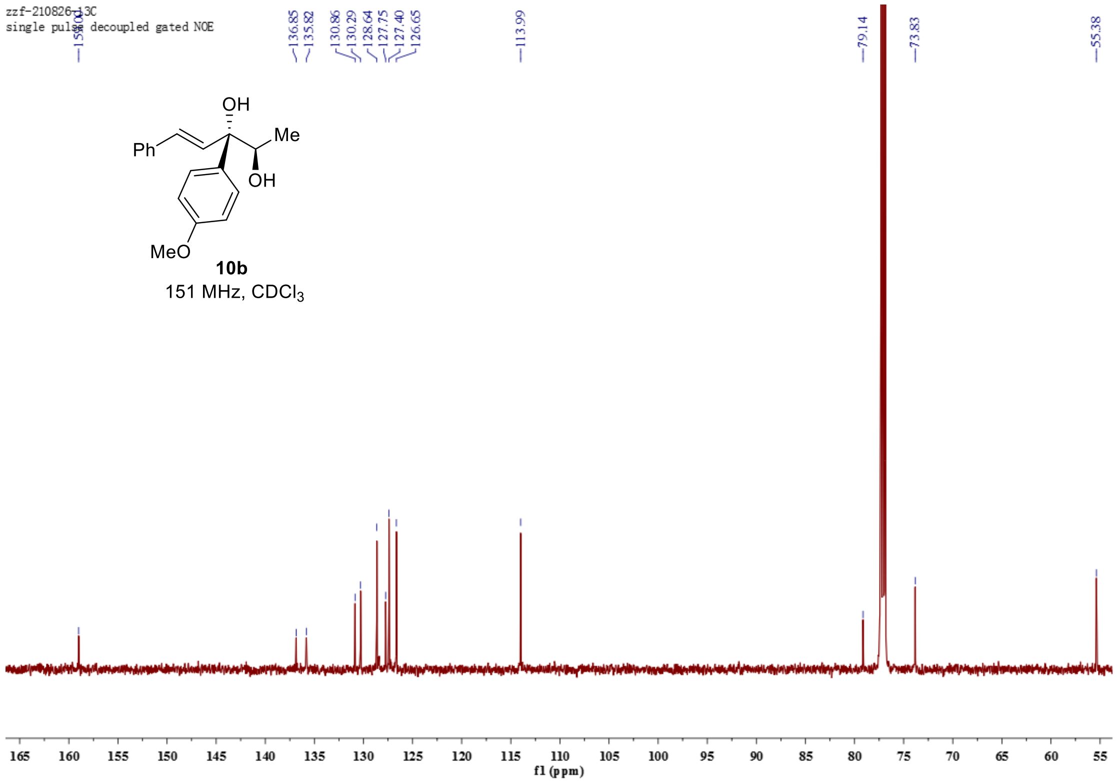

10b

151 MHz, $CDCl_3$

dwn20211129-1

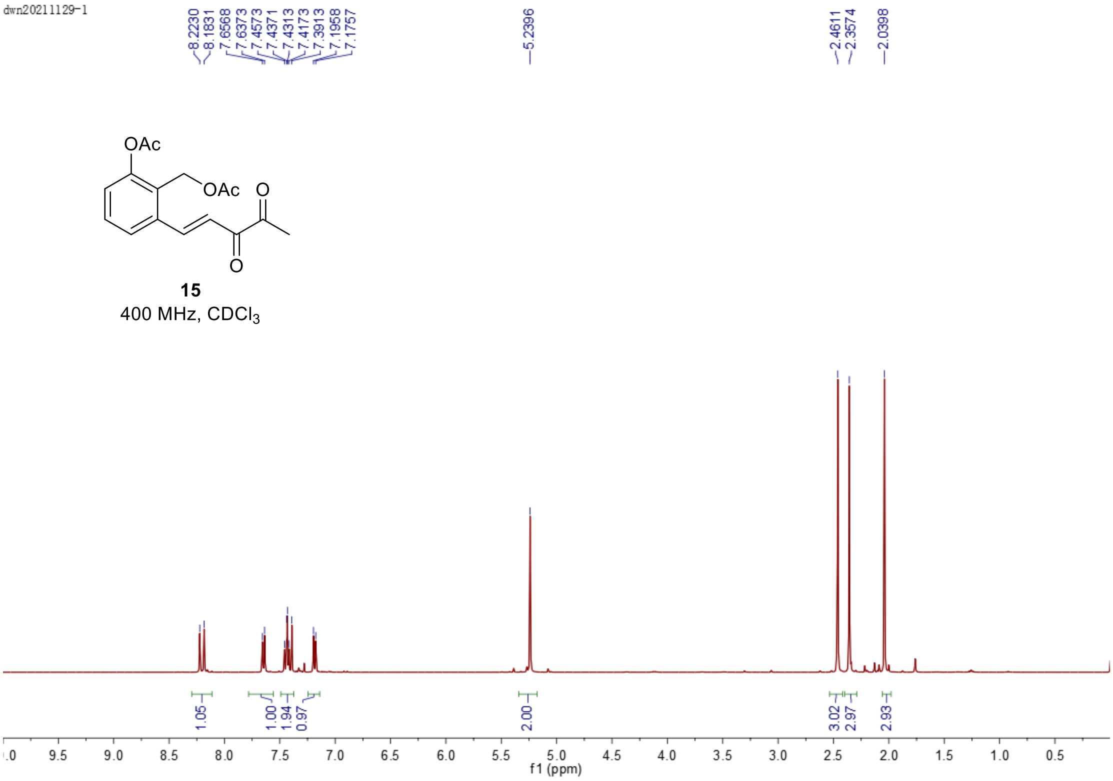

15
400 MHz, $CDCl_3$

DWN-211130-2

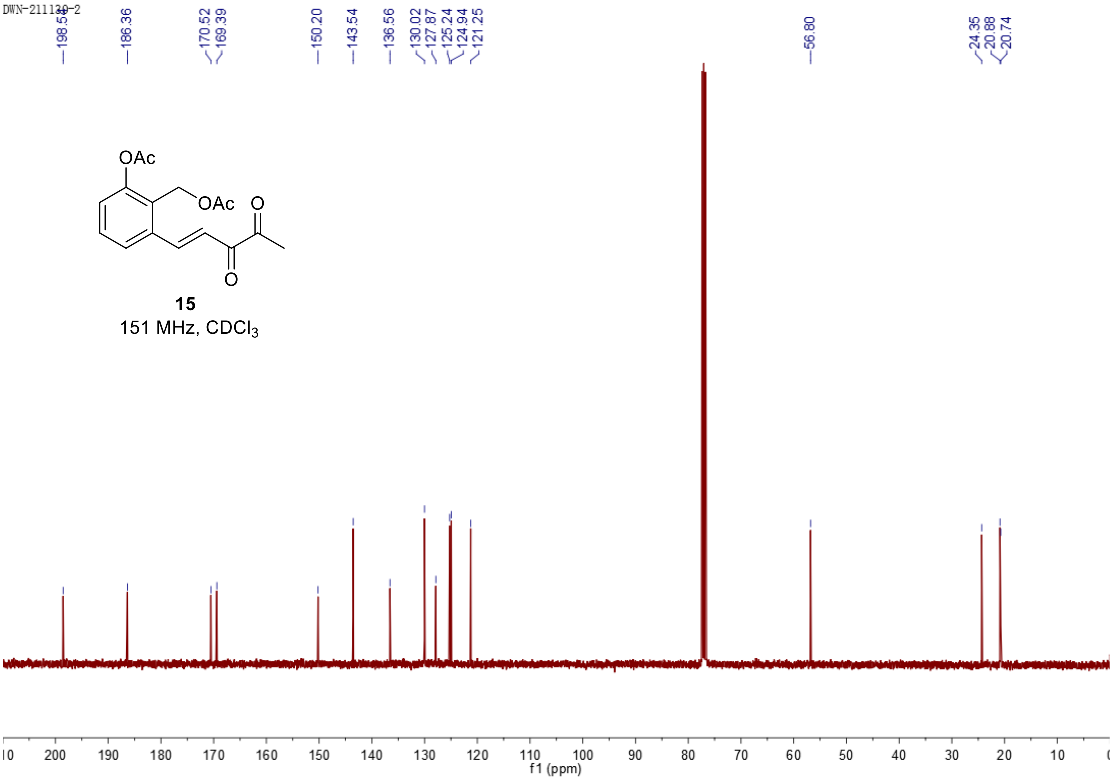

15
151 MHz, $CDCl_3$

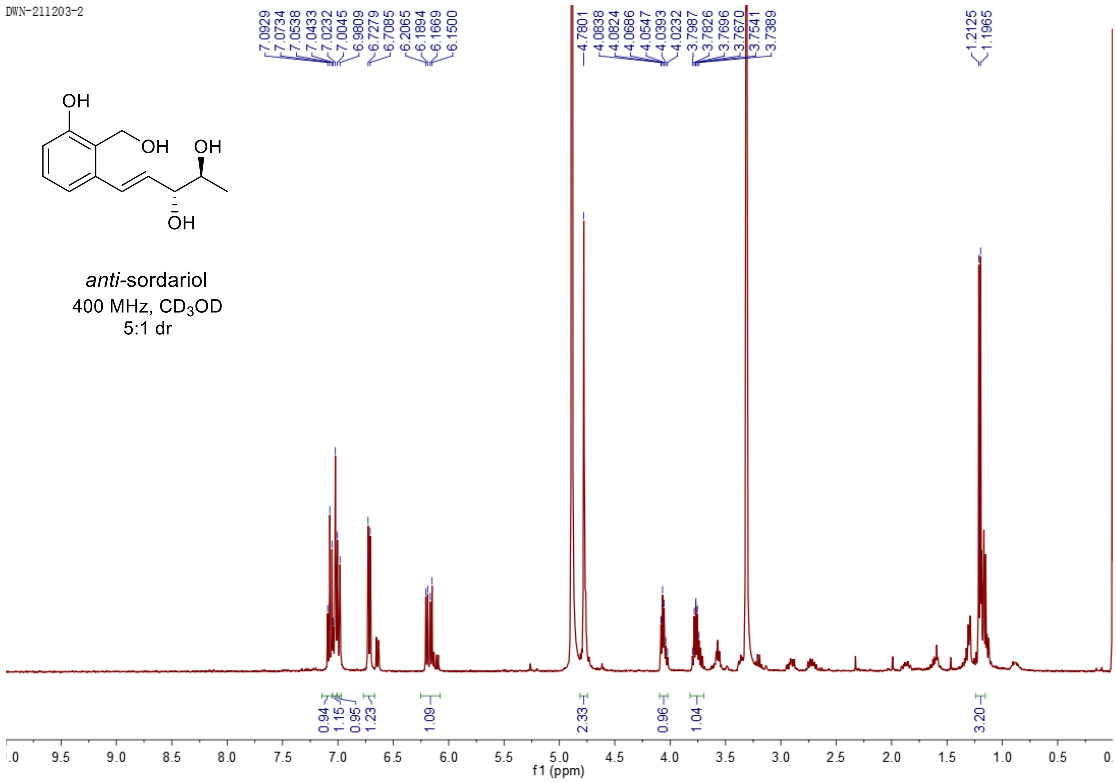
DWN-211203-2
7.0929
7.0734
7.0538
7.0433
7.0232
7.0045
6.9809
6.7279
6.7085
6.2065
6.1894
6.1669
6.1500
4.7801
4.0838
4.0824
4.0686
4.0547
4.0393
4.0232
3.7987
3.7826
3.7696
3.7670
3.7541
3.7389
1.2125
1.1965
OH
OH
OH
OH
anti-sordariol
400 MHz, CD3OD
5:1 dr
0.94
1.15
0.95
1.23
1.09
2.33
0.96
1.04
3.20
f1 (ppm)

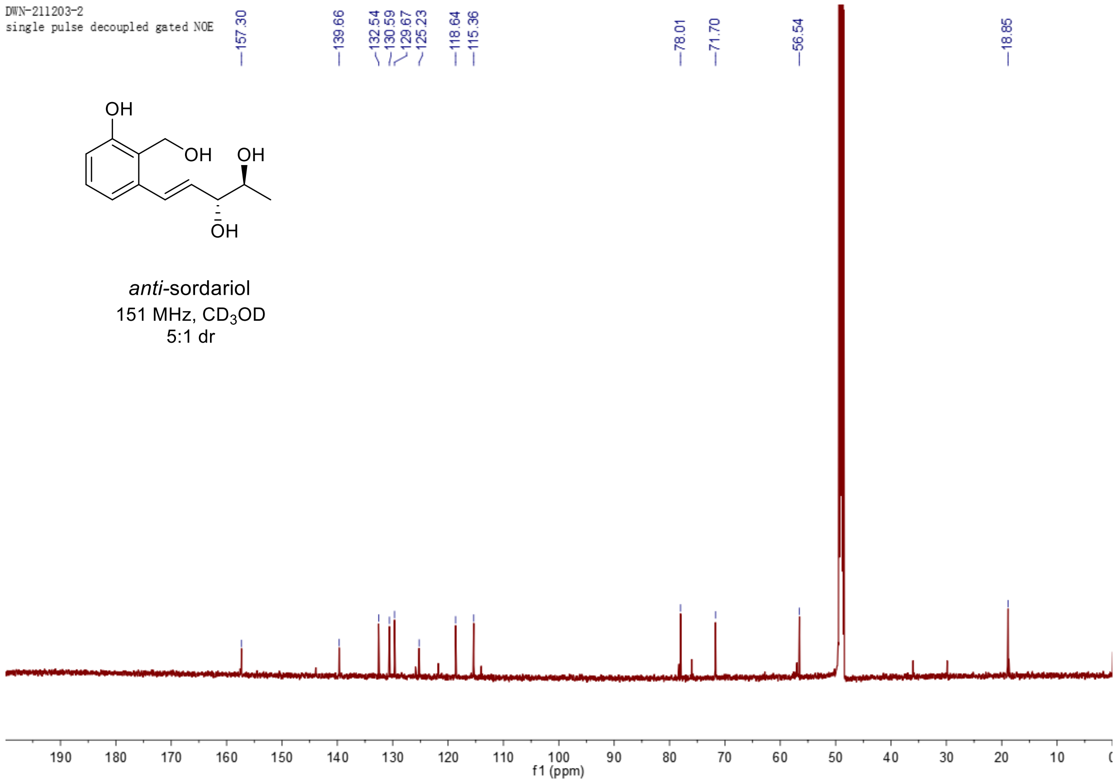
DWN-211203-2
single pulse decoupled gated NOE
157.30
139.66
132.54
130.59
129.67
125.23
118.64
115.36
78.01
71.70
56.54
18.85
OH
OH
OH
OH
anti-sordariol
151 MHz, CD3OD
5:1 dr
f1 (ppm)

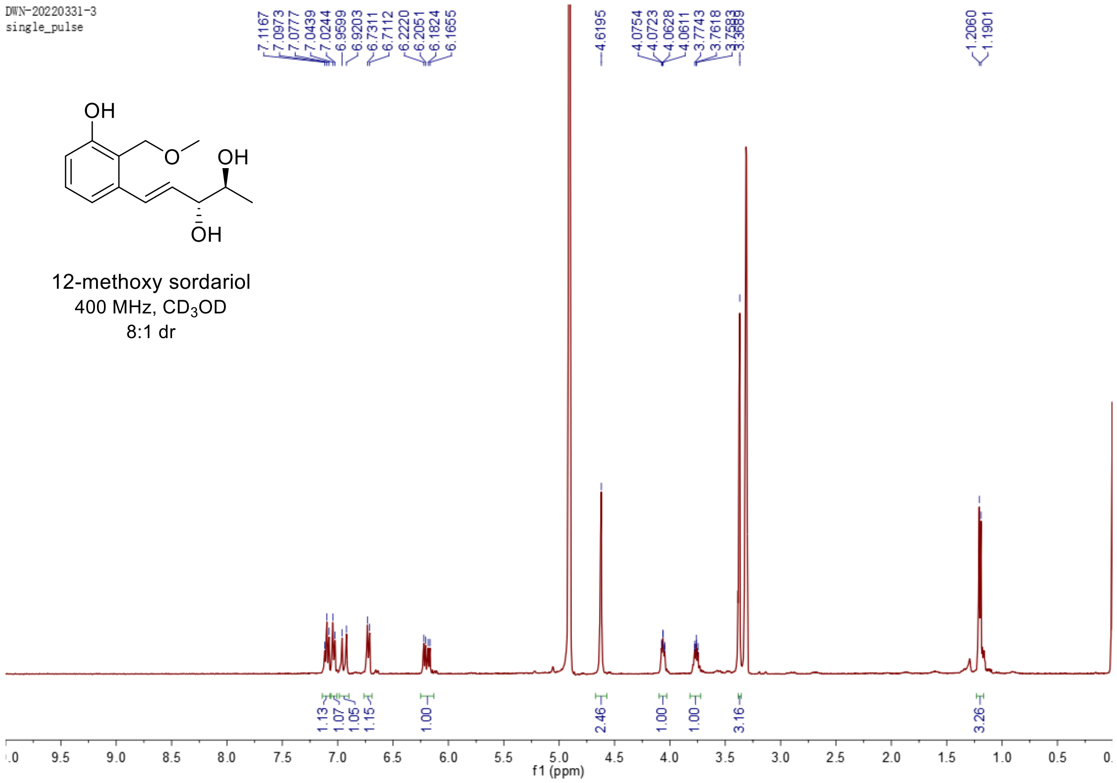
DWN-20220331-3
single_pulse
OH
O
OH
OH
12-methoxy sordariol
400 MHz, CD3OD
8:1 dr
f1 (ppm)

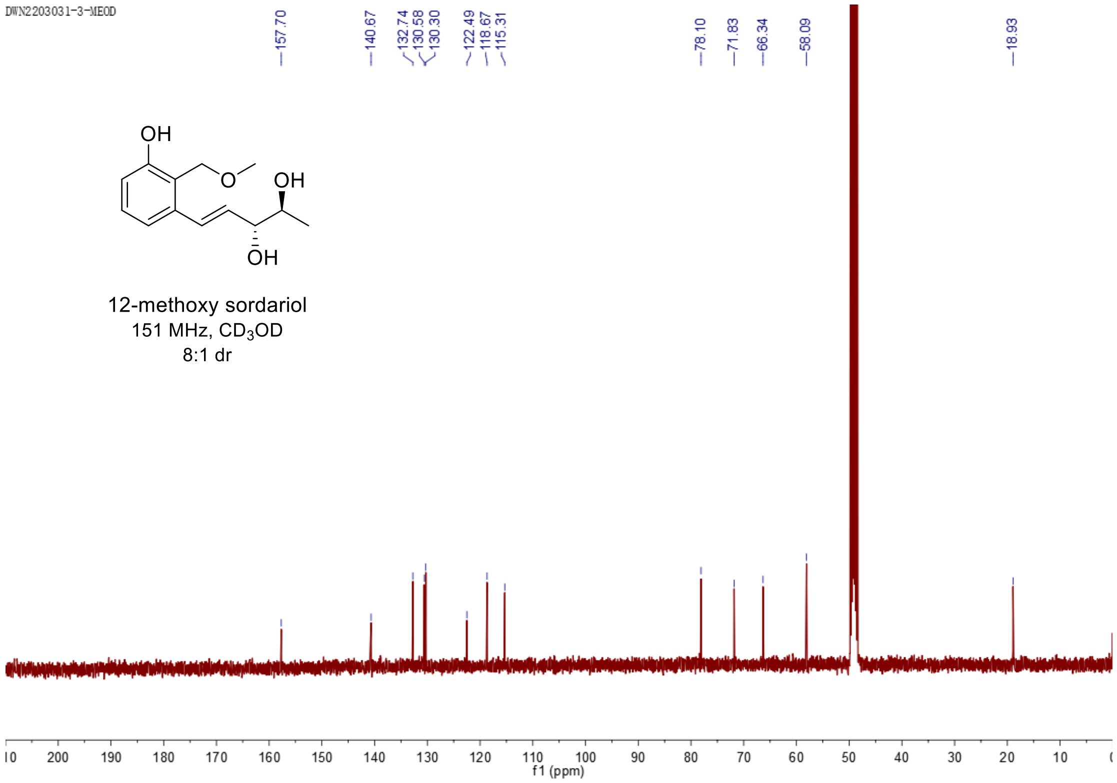
DWN2203031-3-MEOD
OH
O
OH
OH
12-methoxy sordariol
151 MHz, CD3OD
8:1 dr
f1 (ppm)

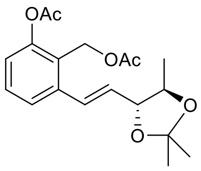

17-1
600 MHz, $CDCl_3$
4:1 dr

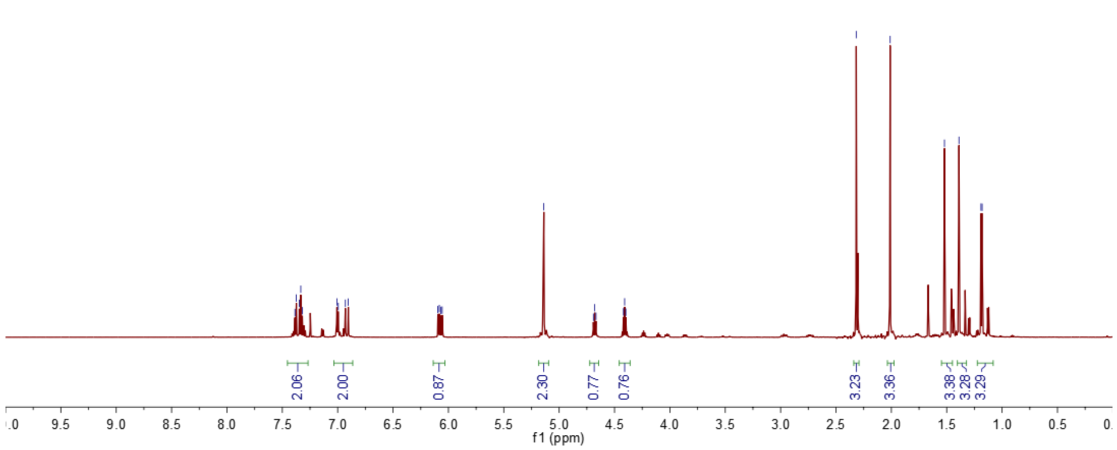

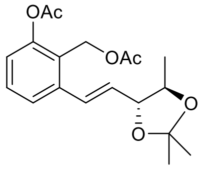

17-1
151 MHz, $CDCl_3$
4:1 dr

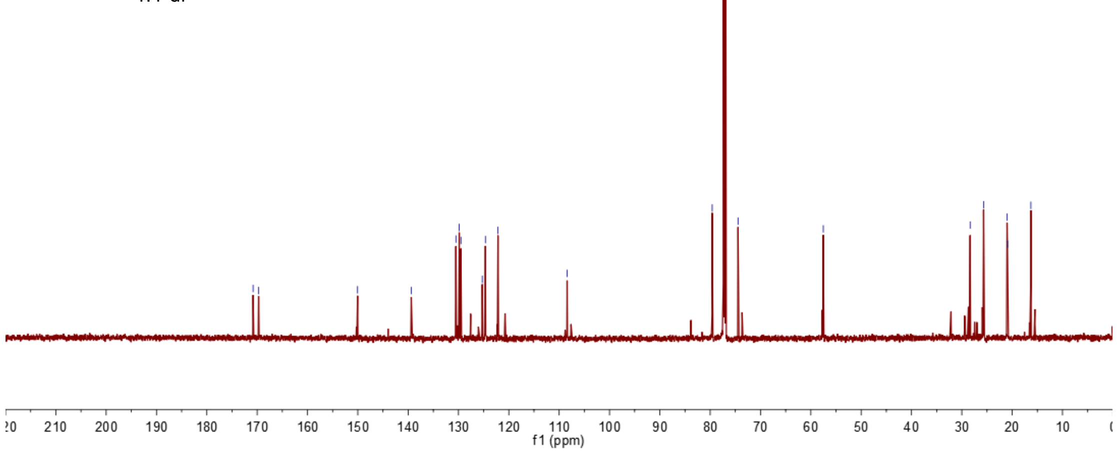

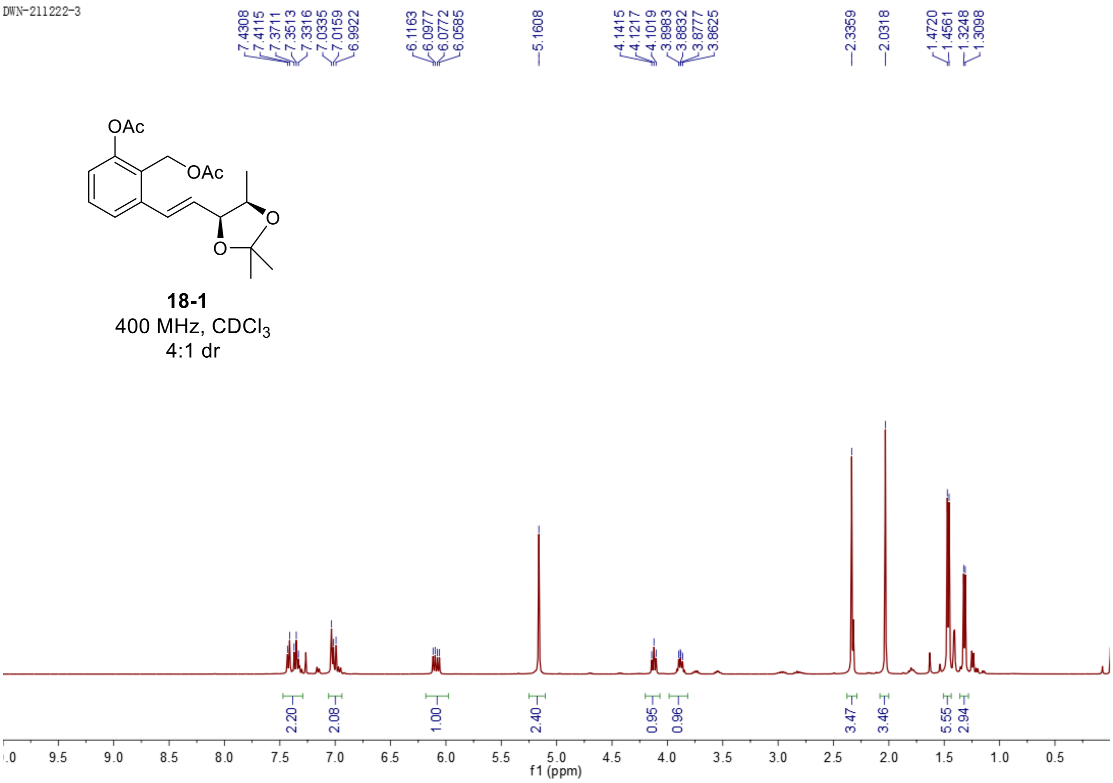

18-1
400 MHz, $CDCl_3$
4:1 dr

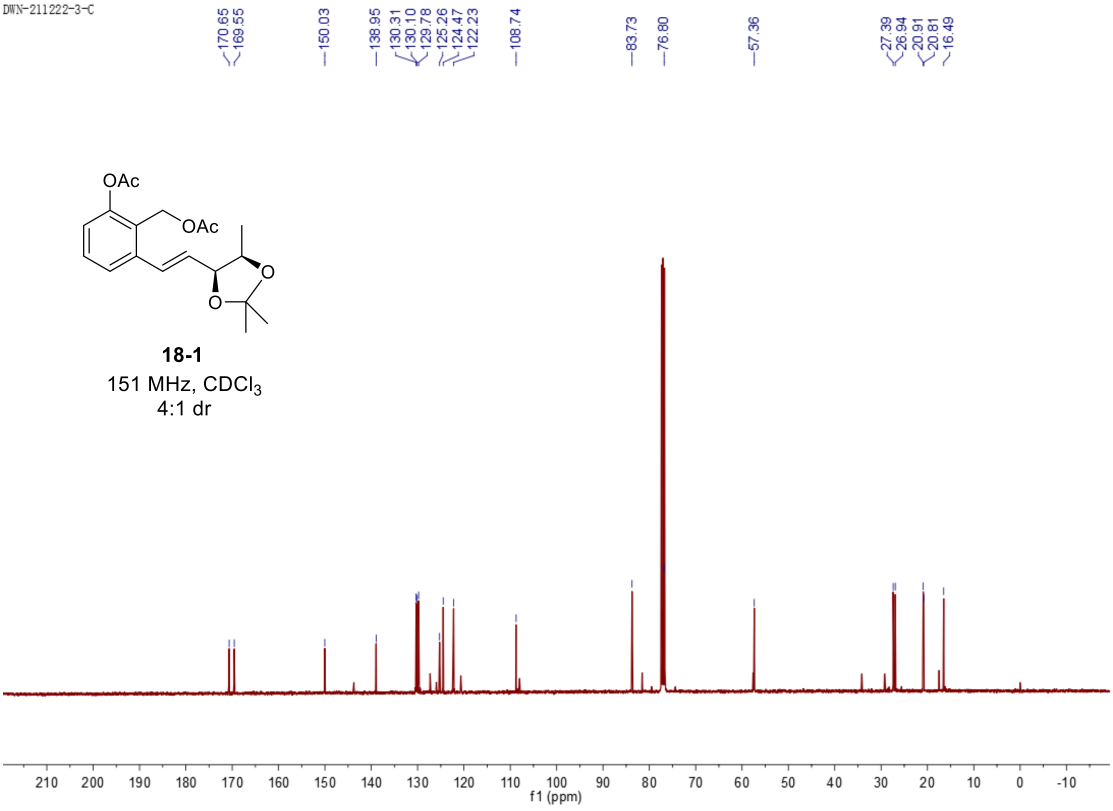

18-1
151 MHz, $CDCl_3$
4:1 dr

XI. HPLC spectra for ee determination

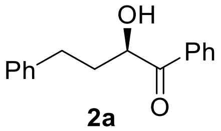

2a

<Chromatogram>

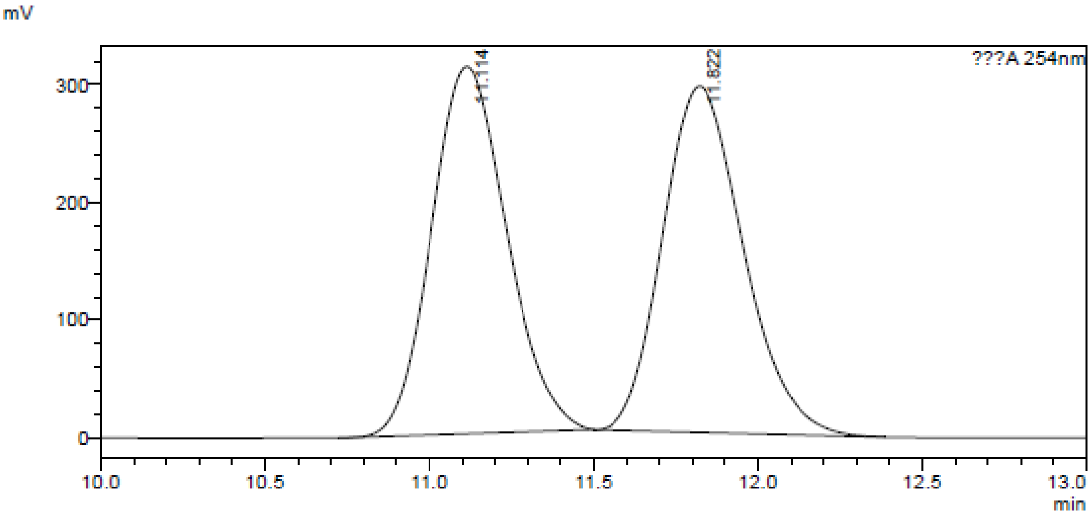

<Peak Table>

???A 254nm

Peak#	Ret. Time	Area	Height	Conc.	Unit	Mark	Name
1	11.114	4958642	311811	49.682		M	
2	11.822	5022135	293759	50.318		M	
Total		9980778	605570				

<Chromatogram>

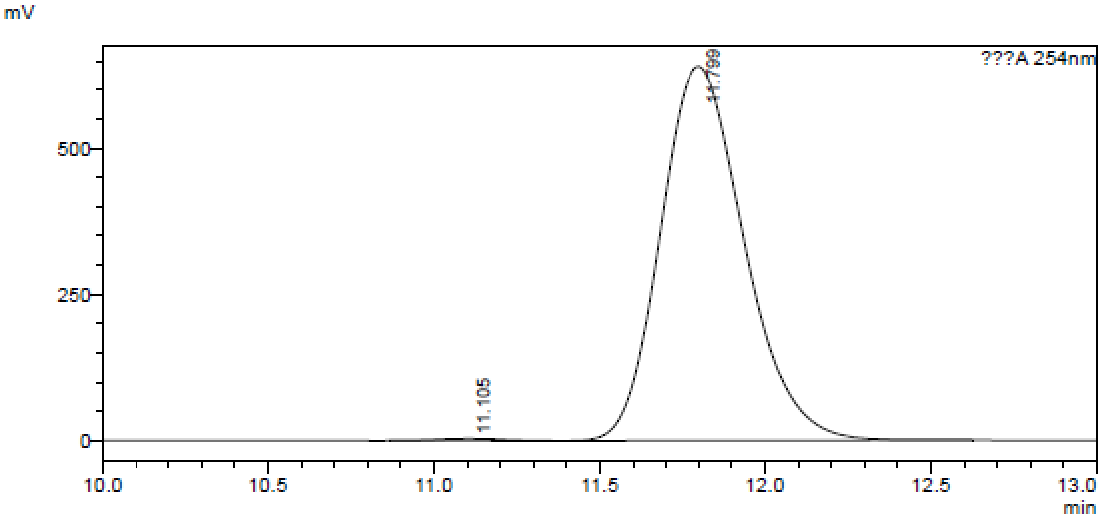

<Peak Table>

???A 254nm

Peak#	Ret. Time	Area	Height	Conc.	Unit	Mark	Name
1	11.105	47941	3376	0.422		M	
2	11.799	11303085	640195	99.578		M	
Total		11351027	643571				

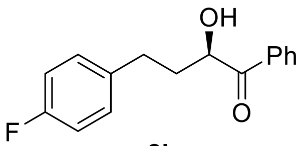

2b

<Chromatogram>

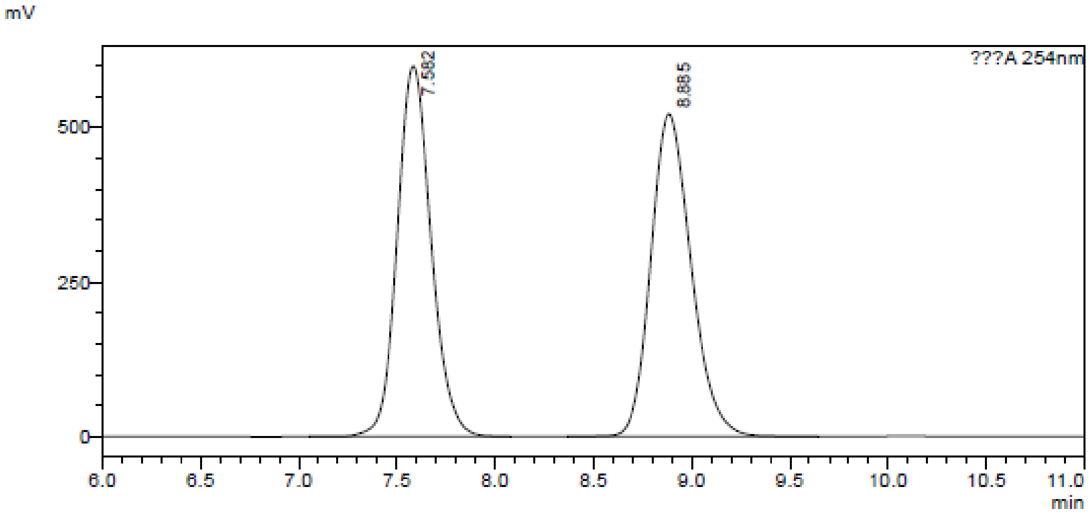

<Peak Table>

???A 254nm

Peak#	Ret. Time	Area	Height	Conc.	Unit	Mark	Name
1	7.582	7146201	597207	49.086		M	
2	8.885	7412306	520070	50.914		M	
Total		14558507	1117277				

<Chromatogram>

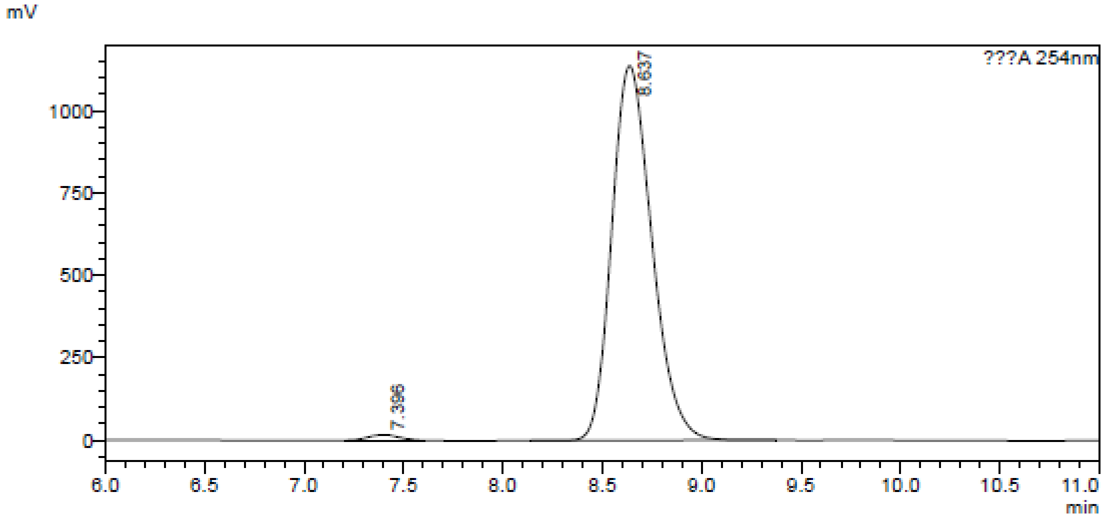

<Peak Table>

???A 254nm

Peak#	Ret. Time	Area	Height	Conc.	Unit	Mark	Name
1	7.396	174838	16773	1.085		M	
2	8.637	15933443	1135199	98.915		M	
Total		16108281	1151972				

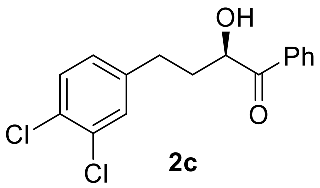

2c

<Chromatogram>

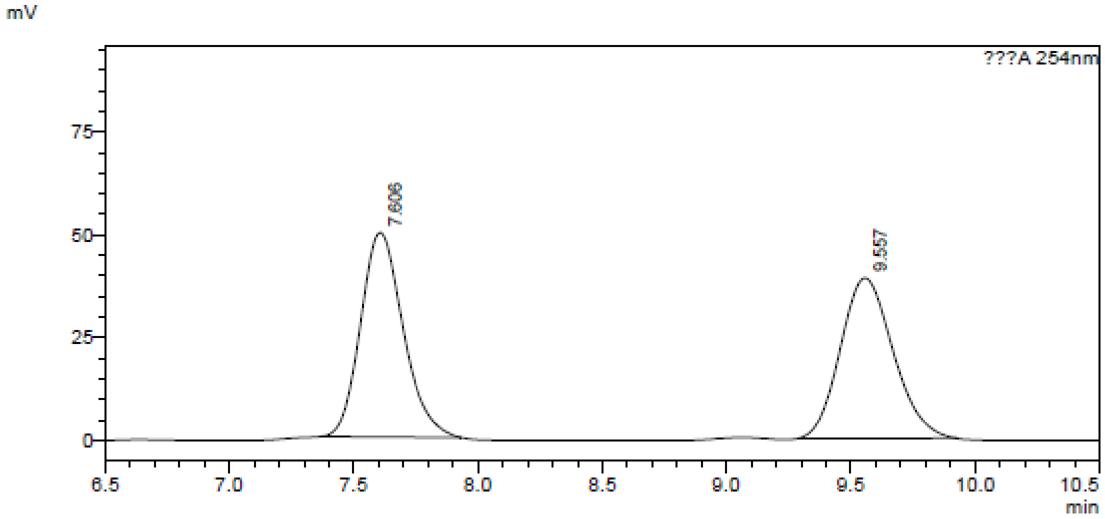

<Peak Table>

???A 254nm

Peak#	Ret. Time	Area	Height	Conc.	Unit	Mark	Name
1	7.606	594818	49779	50.744		M	
2	9.557	577376	38985	49.256		M	
Total		1172195	88763				

<Chromatogram>

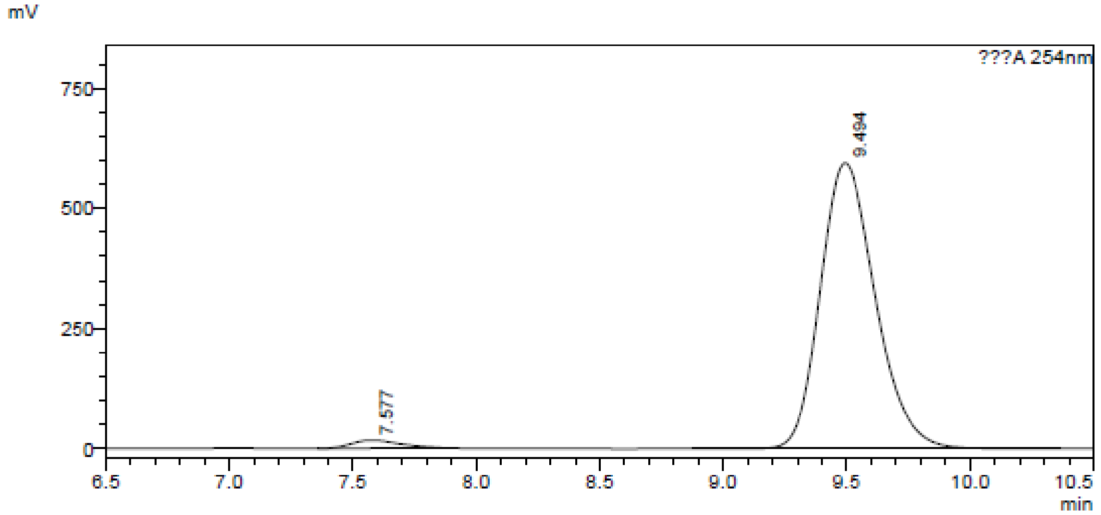

<Peak Table>

???A 254nm

Peak#	Ret. Time	Area	Height	Conc.	Unit	Mark	Name
1	7.577	224166	16631	2.384		M	
2	9.494	9179832	594100	97.616		M	
Total		9403998	610730				

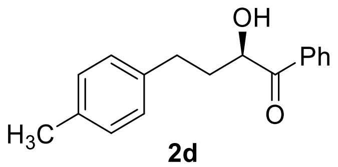

2d

<Chromatogram>

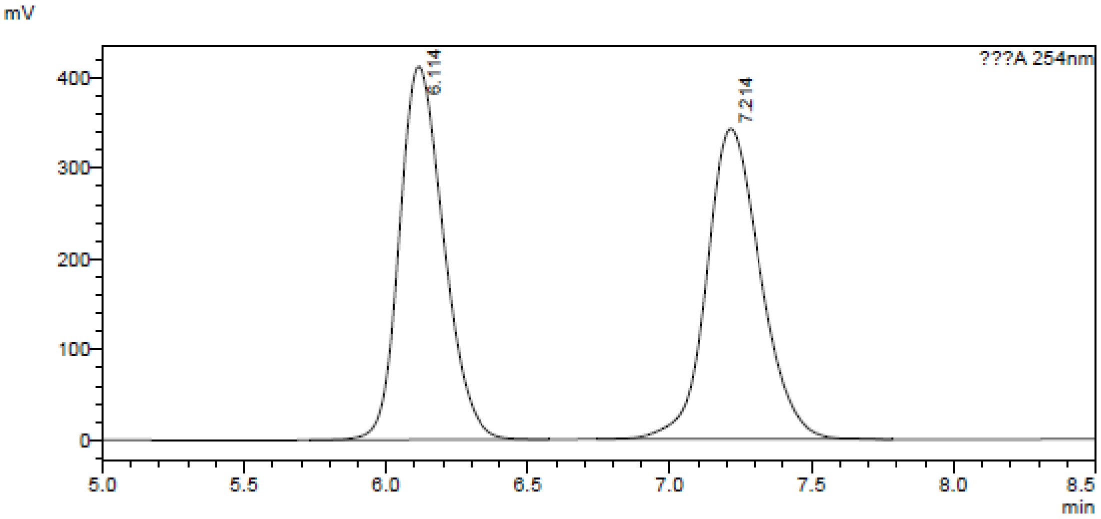

<Peak Table>

???A 254nm

Peak#	Ret. Time	Area	Height	Conc.	Unit	Mark	Name
1	6.114	4446941	411292	49.453		M	
2	7.214	4545382	342446	50.547		M	
Total		8992324	753738				

<Chromatogram>

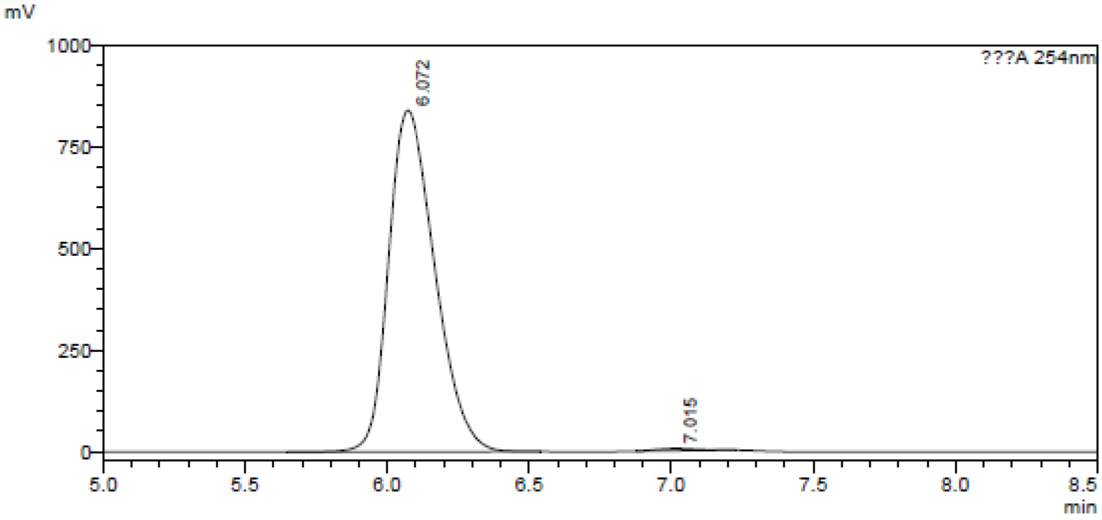

<Peak Table>

???A 254nm

Peak#	Ret. Time	Area	Height	Conc.	Unit	Mark	Name
1	6.072	9558485	839323	99.456		M	
2	7.015	52249	5241	0.544		M	
Total		9610734	844564				

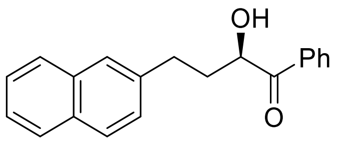

2e

<Chromatogram>

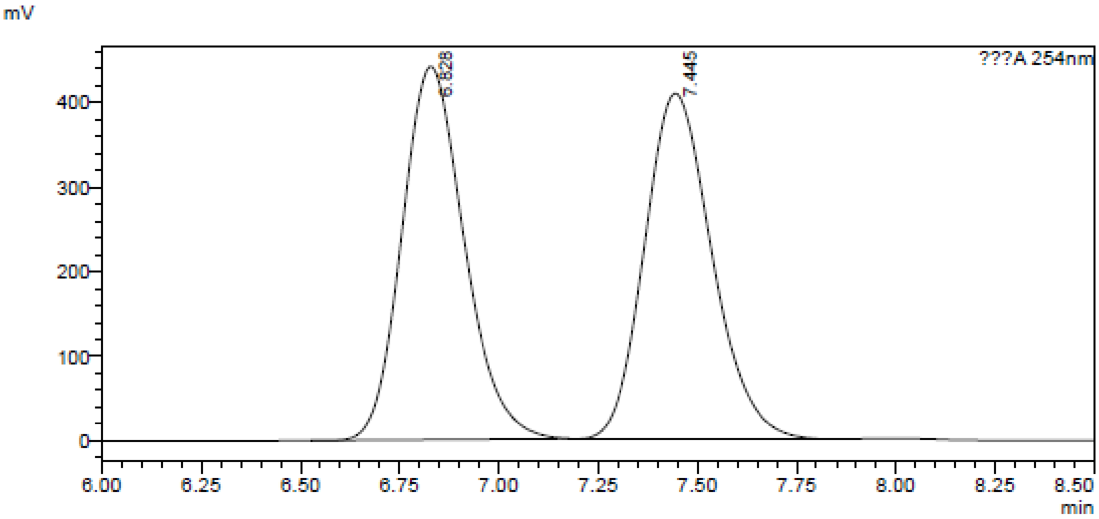

<Peak Table>

???A 254nm

Peak#	Ret. Time	Area	Height	Conc.	Unit	Mark	Name
1	6.828	4826799	441804	49.909		M	
2	7.445	4844321	409272	50.091		M	
Total		9671120	851076				

<Chromatogram>

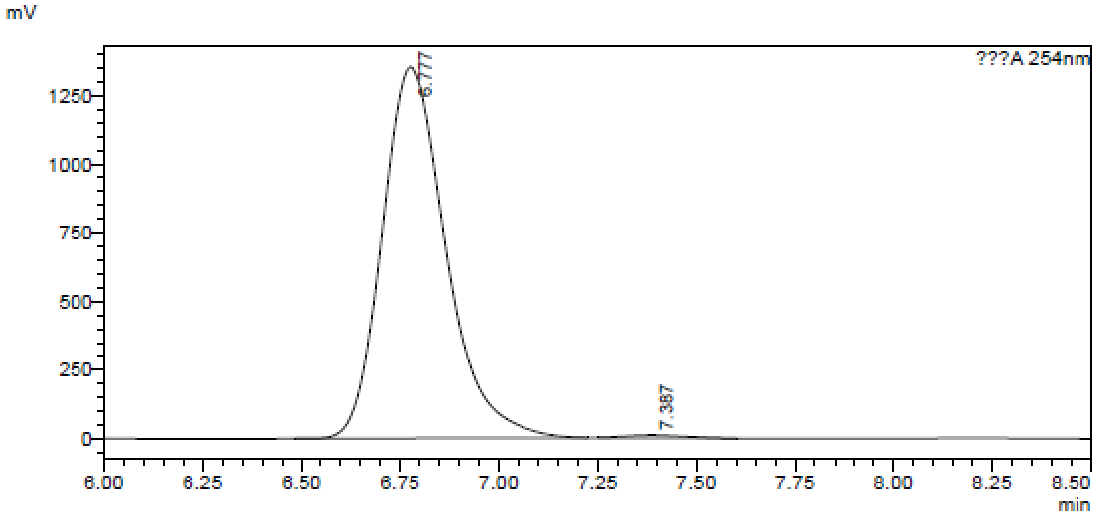

<Peak Table>

???A 254nm

Peak#	Ret. Time	Area	Height	Conc.	Unit	Mark	Name
1	6.777	15236923	1354735	99.340		M	
2	7.387	101178	10233	0.660		M	
Total		15338101	1364968				

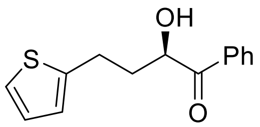

2f

<Chromatogram>

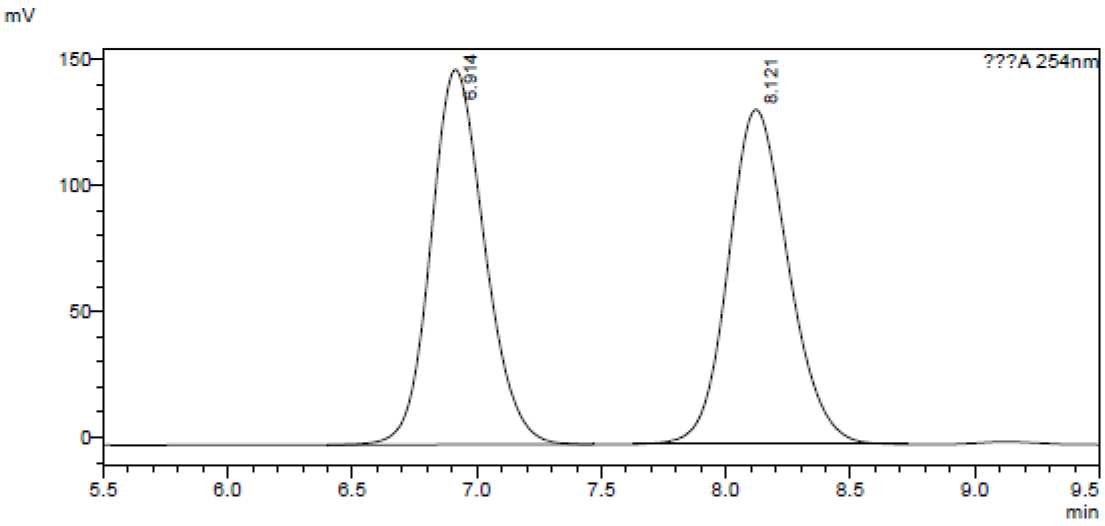

<Peak Table>

???A 254nm

Peak#	Ret. Time	Area	Height	Conc.	Unit	Mark	Name
1	6.914	2237047	148850	50.057		M	
2	8.121	2231912	132735	49.943		M	
Total		4468958	281585				

<Chromatogram>

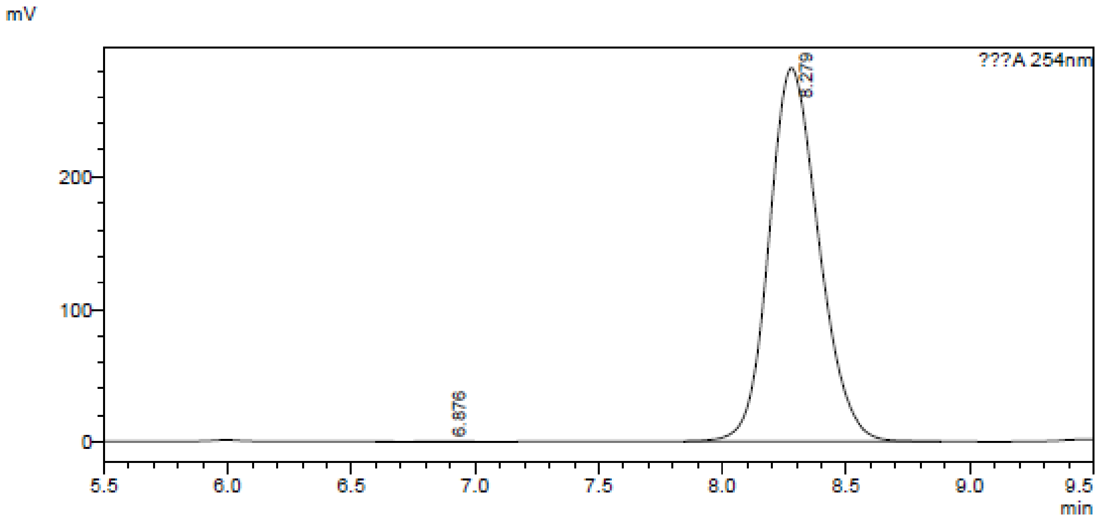

<Peak Table>

???A 254nm

Peak#	Ret. Time	Area	Height	Conc.	Unit	Mark	Name
1	6.876	33	95	0.001		M	
2	8.279	3952906	281466	99.999		M	
Total		3952940	281561				

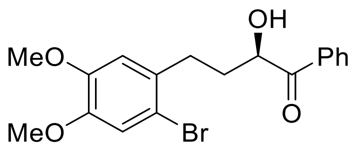

2g

<Chromatogram>

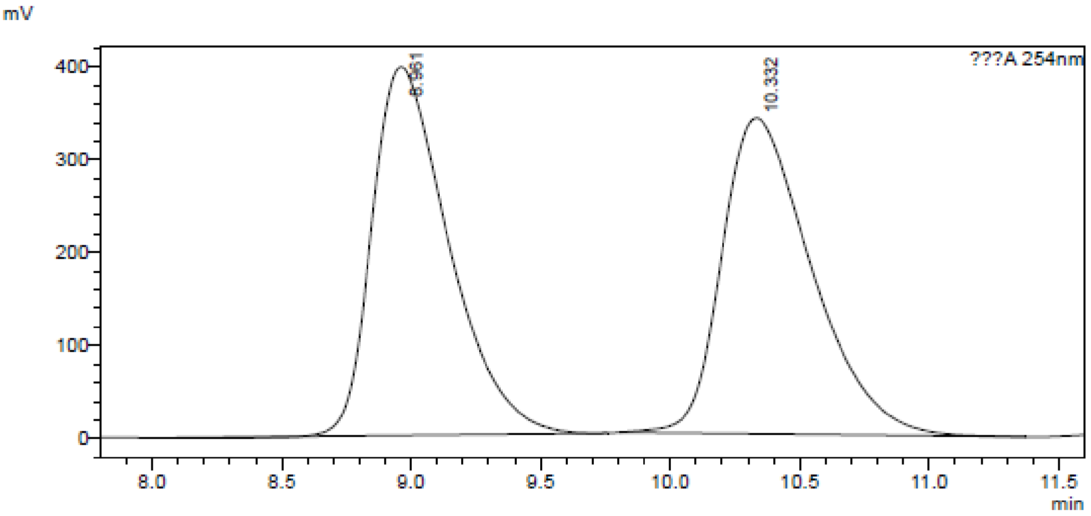

<Peak Table>

???A 254nm

Peak#	Ret. Time	Area	Height	Conc.	Unit	Mark	Name
1	8.961	8153865	396345	49.844		M	
2	10.332	8204887	339492	50.156		M	
Total		16358752	735837				

<Chromatogram>

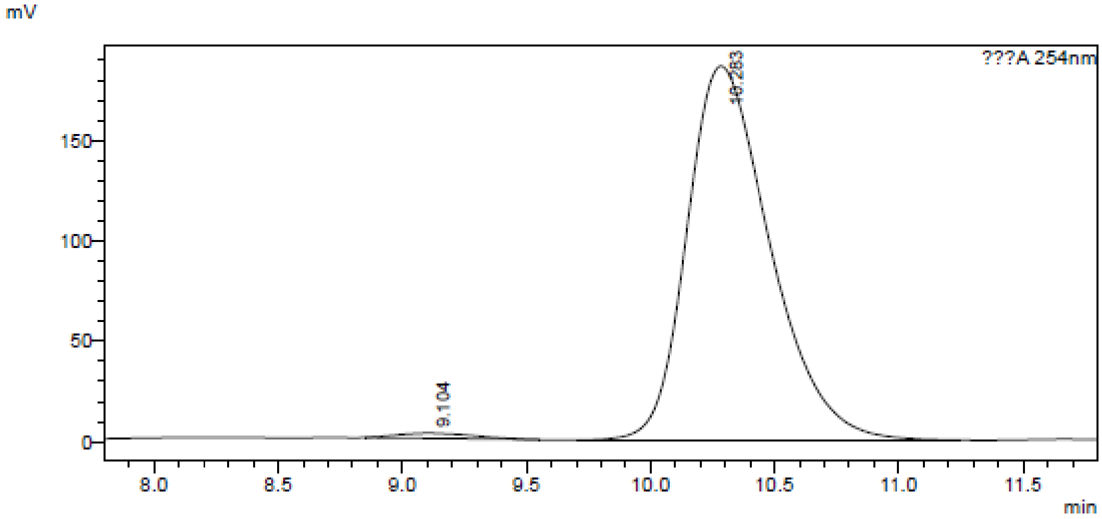

<Peak Table>

???A 254nm

Peak#	Ret. Time	Area	Height	Conc.	Unit	Mark	Name
1	9.104	54204	2520	1.233		M	
2	10.283	4342451	186465	98.767		M	
Total		4396655	188985				

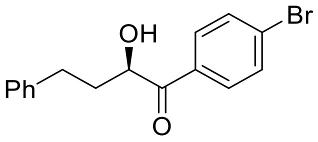

2h

<Chromatogram>

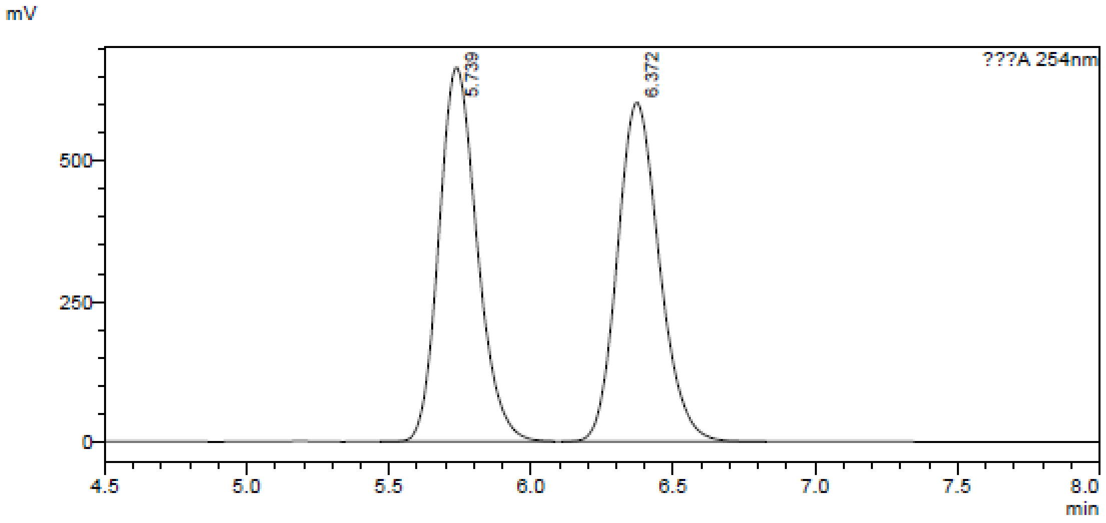

<Peak Table>

???A 254nm

Peak#	Ret. Time	Area	Height	Conc.	Unit	Mark	Name
1	5.739	6205426	664995	50.053		M	
2	6.372	6192166	601729	49.947		M	
Total		12397592	1266724				

<Chromatogram>

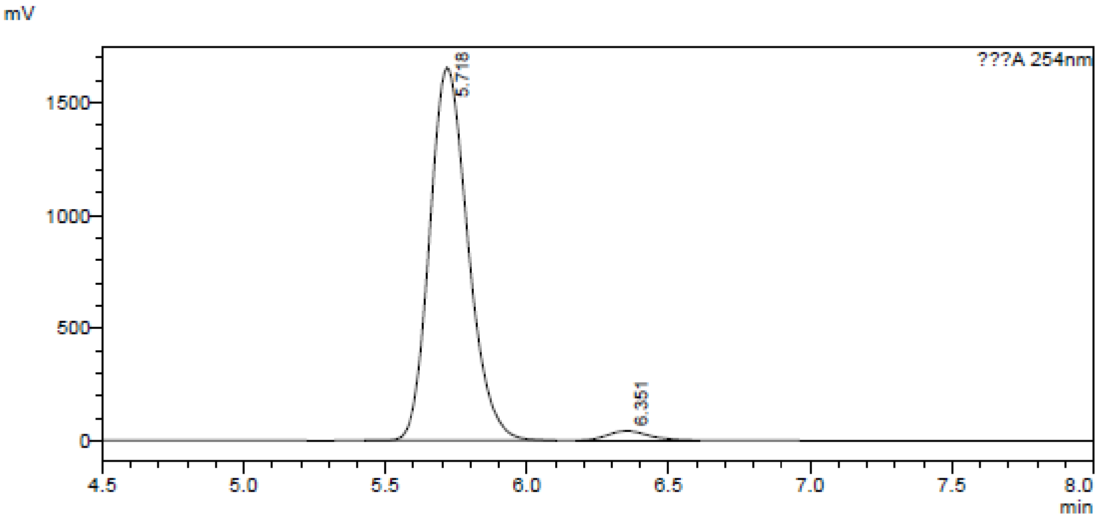

<Peak Table>

???A 254nm

Peak#	Ret. Time	Area	Height	Conc.	Unit	Mark	Name
1	5.718	15518577	1655263	97.420		M	
2	6.351	410910	41078	2.580		M	
Total		15929487	1696342				

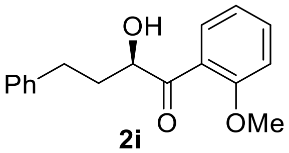

2i

<Chromatogram>

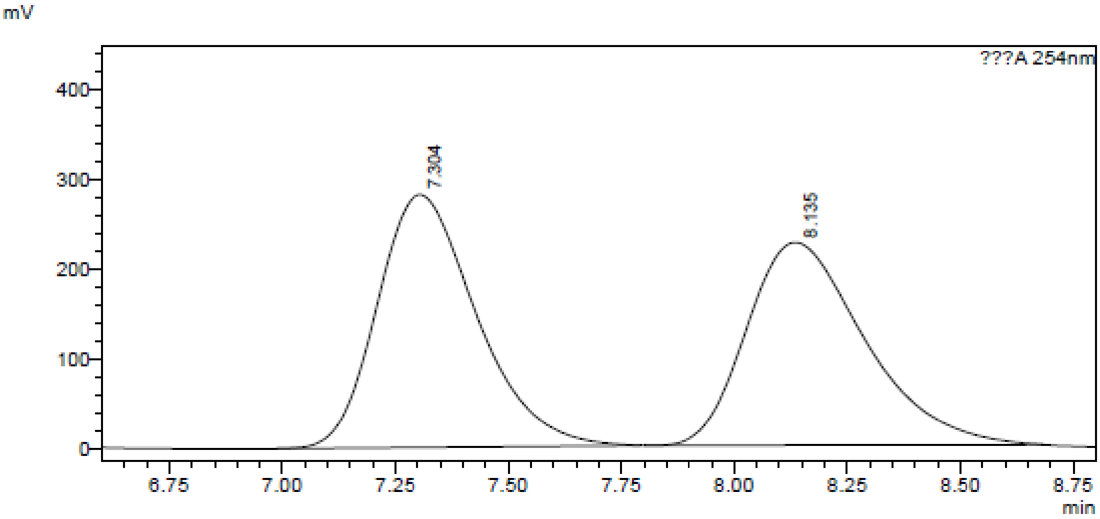

<Peak Table>

???A 254nm

Peak#	Ret. Time	Area	Height	Conc.	Unit	Mark	Name
1	7.304	4317667	281890	51.220		M	
2	8.135	4112064	226037	48.780		M	
Total		8429731	507927				

<Chromatogram>

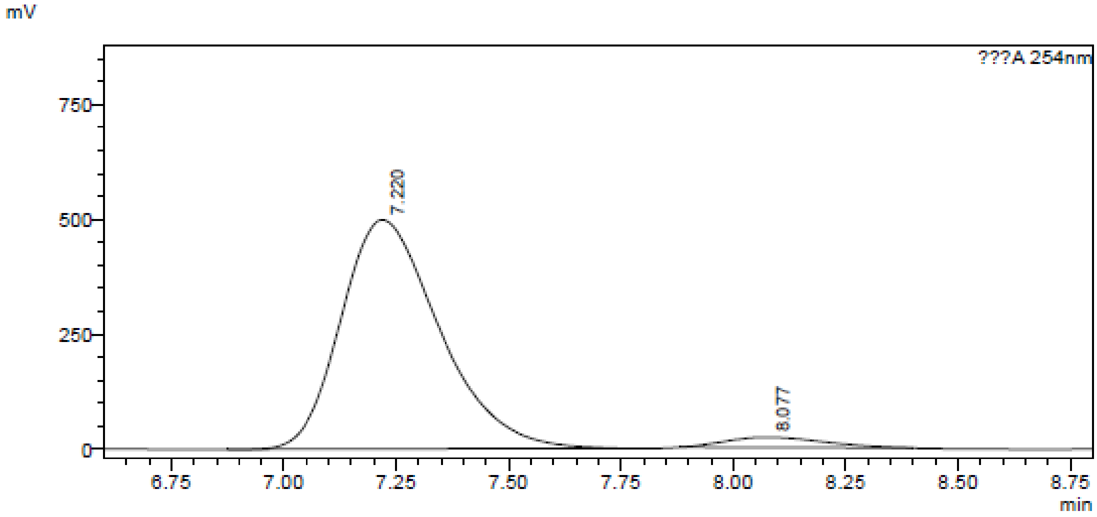

<Peak Table>

???A 254nm

Peak#	Ret. Time	Area	Height	Conc.	Unit	Mark	Name
1	7.220	7594406	498328	95.736		M	
2	8.077	338278	22265	4.264		M	
Total		7932684	520593				

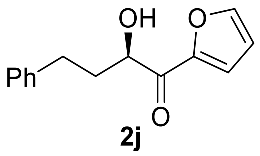

2j

<Chromatogram>

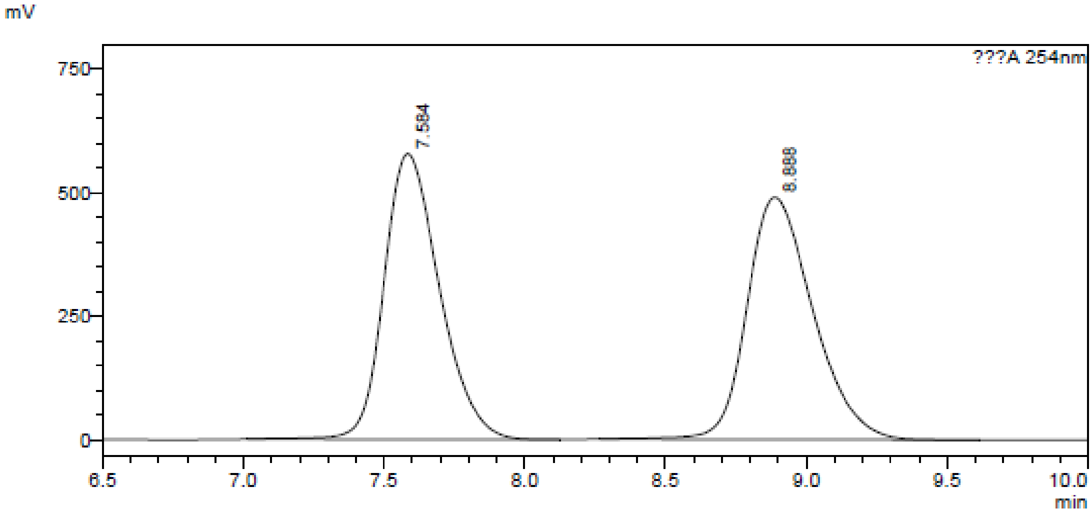

<Peak Table>

???A 254nm

Peak#	Ret. Time	Area	Height	Conc.	Unit	Mark	Name
1	7.584	7910608	577468	49.726		M	
2	8.888	7997809	489386	50.274		M	
Total		15908418	1066854				

<Chromatogram>

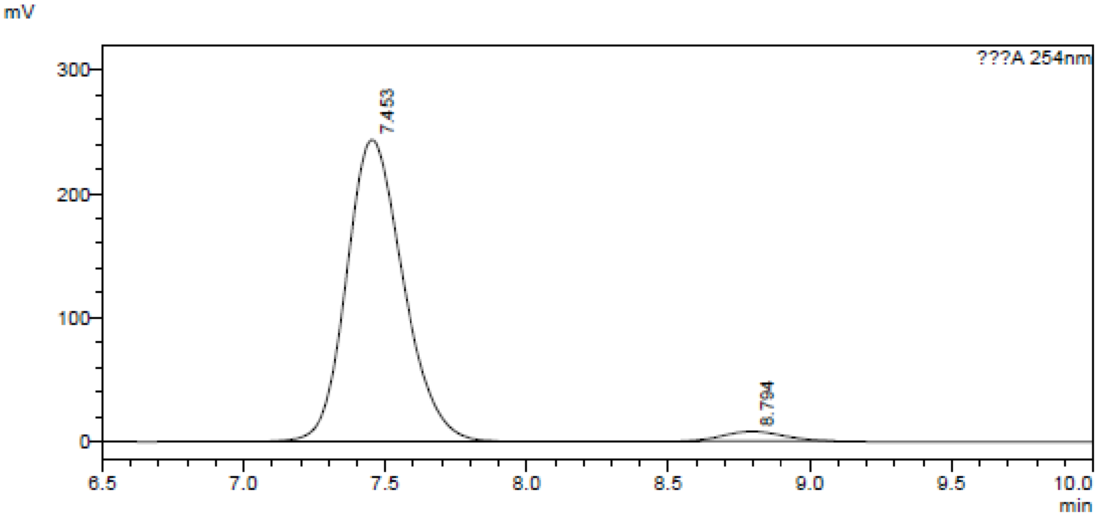

<Peak Table>

???A 254nm

Peak#	Ret. Time	Area	Height	Conc.	Unit	Mark	Name
1	7.453	3398167	243714	96.731		SV	
2	8.794	114846	7766	3.269		M	
Total		3513013	251480				

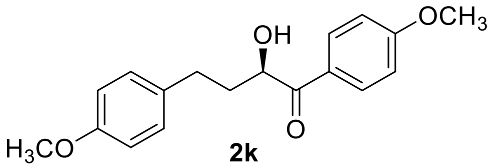

2k

<Chromatogram>

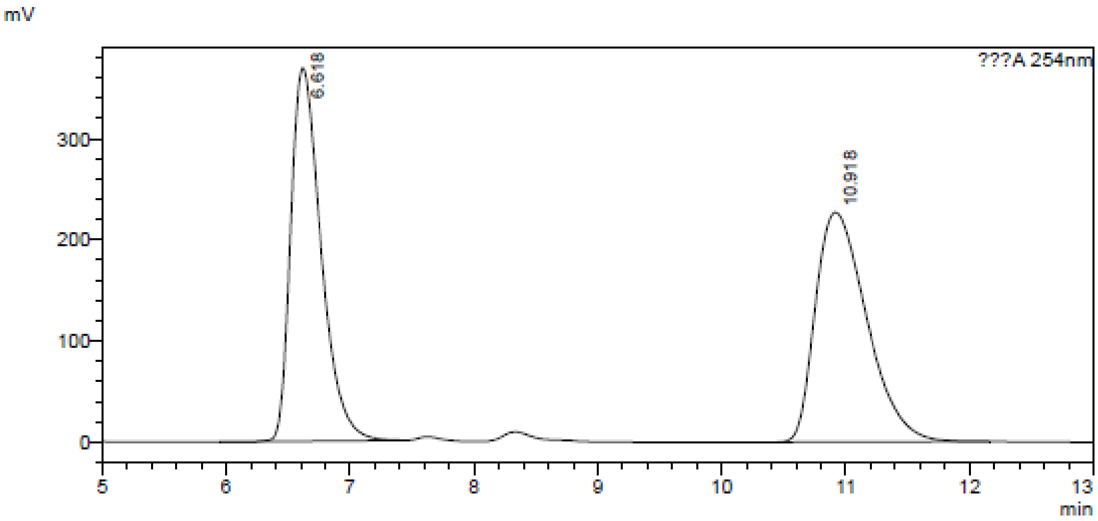

<Peak Table>

???A 254nm

Peak#	Ret. Time	Area	Height	Conc.	Unit	Mark	Name
1	6.618	6300978	368299	49.164		M	
2	10.918	6515214	226627	50.836		M	
Total		12816192	594925				

<Chromatogram>

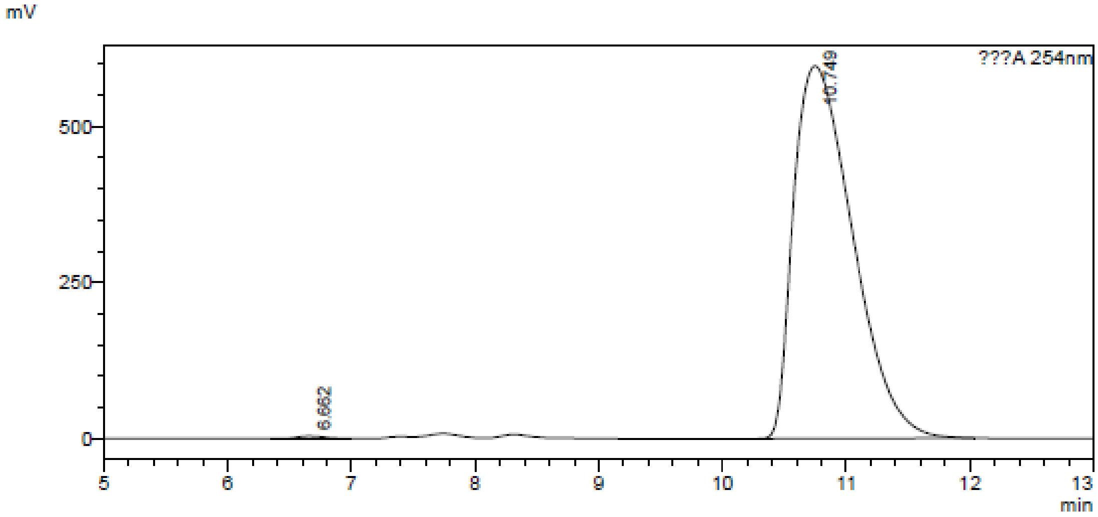

<Peak Table>

???A 254nm

Peak#	Ret. Time	Area	Height	Conc.	Unit	Mark	Name
1	6.662	58522	3853	0.294		M	
2	10.749	19848752	596549	99.706		M	
Total		19907274	600402				

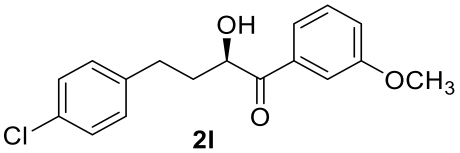

2l

<Chromatogram>

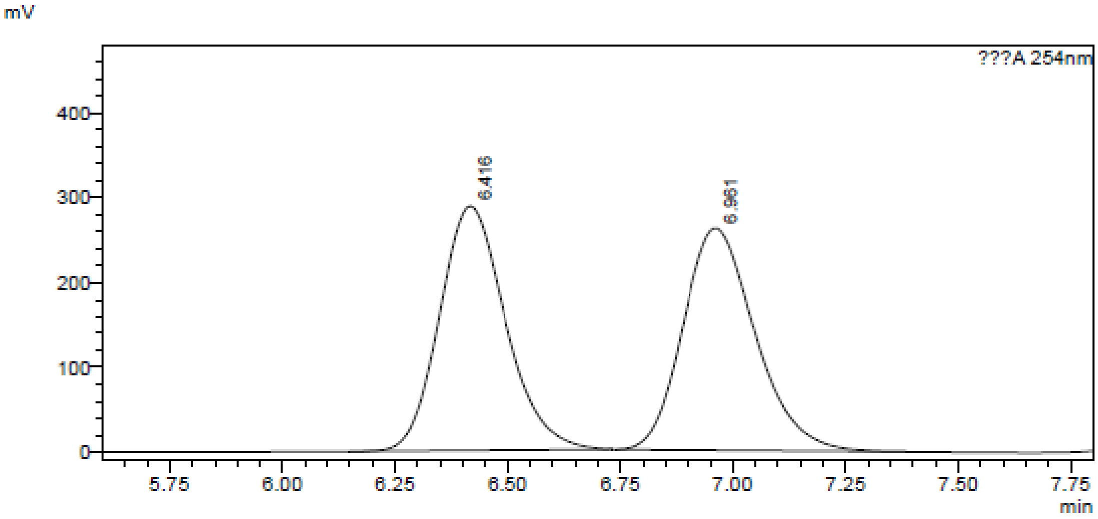

<Peak Table>

???A 254nm

Peak#	Ret. Time	Area	Height	Conc.	Unit	Mark	Name
1	6.416	2948117	288602	50.135		M	
2	6.961	2932294	262082	49.865		M	
Total		5880412	550684				

<Chromatogram>

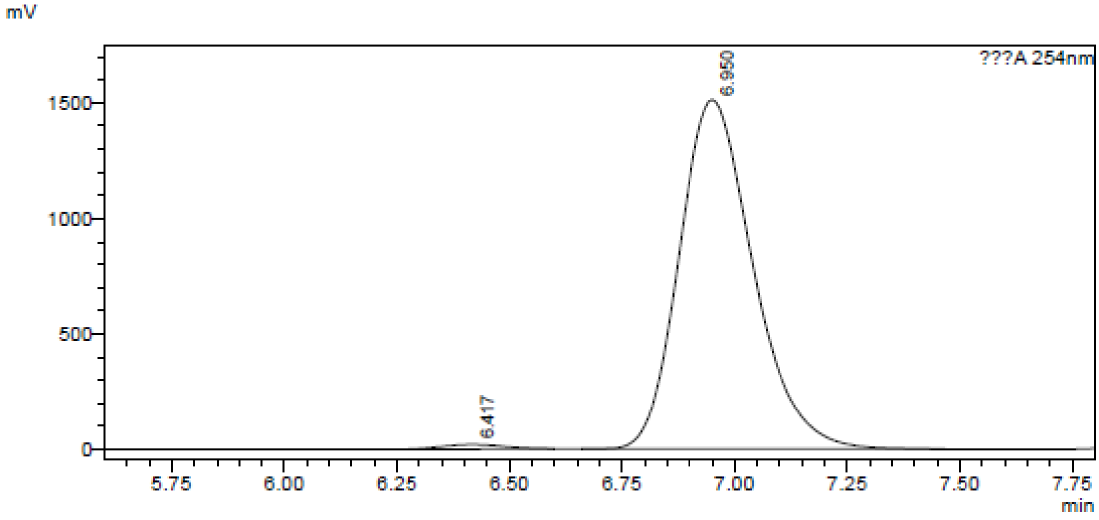

<Peak Table>

???A 254nm

Peak#	Ret. Time	Area	Height	Conc.	Unit	Mark	Name
1	6.417	184965	19584	1.061		M	
2	6.950	17254919	1510900	98.939		M	
Total		17439883	1530484				

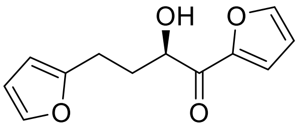

2m

<Chromatogram>

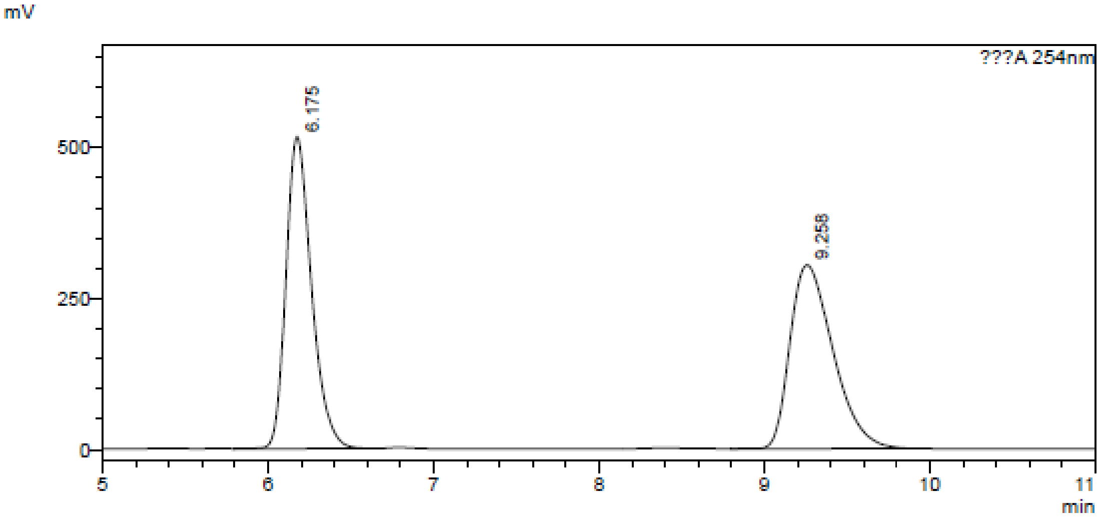

<Peak Table>

???A 254nm

Peak#	Ret. Time	Area	Height	Conc.	Unit	Mark	Name
1	6.175	5535230	516302	50.209			
2	9.258	5489234	304176	49.791		M	
Total		11024464	820478				

<Chromatogram>

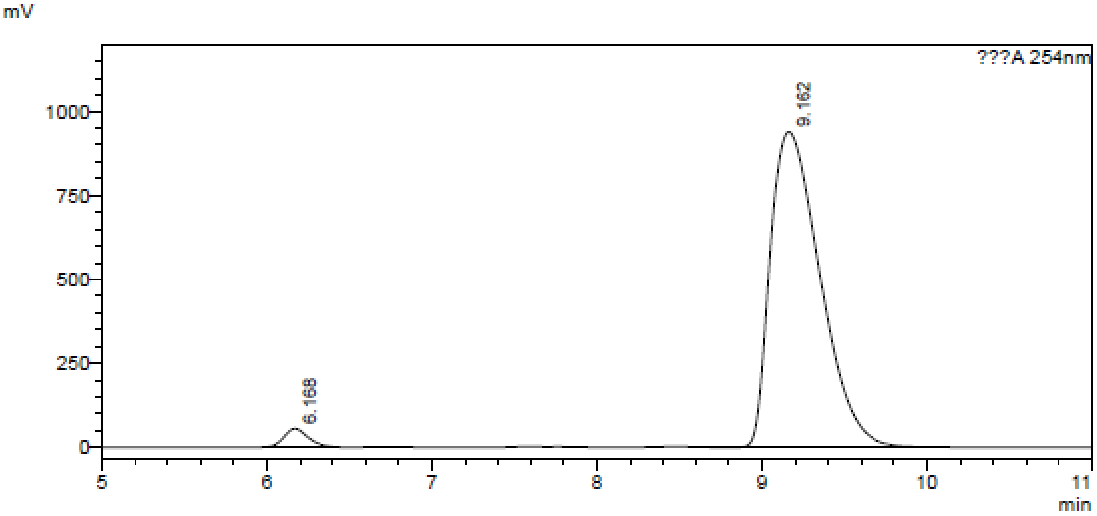

<Peak Table>

???A 254nm

Peak#	Ret. Time	Area	Height	Conc.	Unit	Mark	Name
1	6.168	559161	54804	2.778		M	
2	9.162	19568881	937594	97.222		M	
Total		20128042	992397				

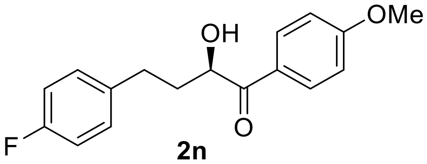

2n

<Chromatogram>

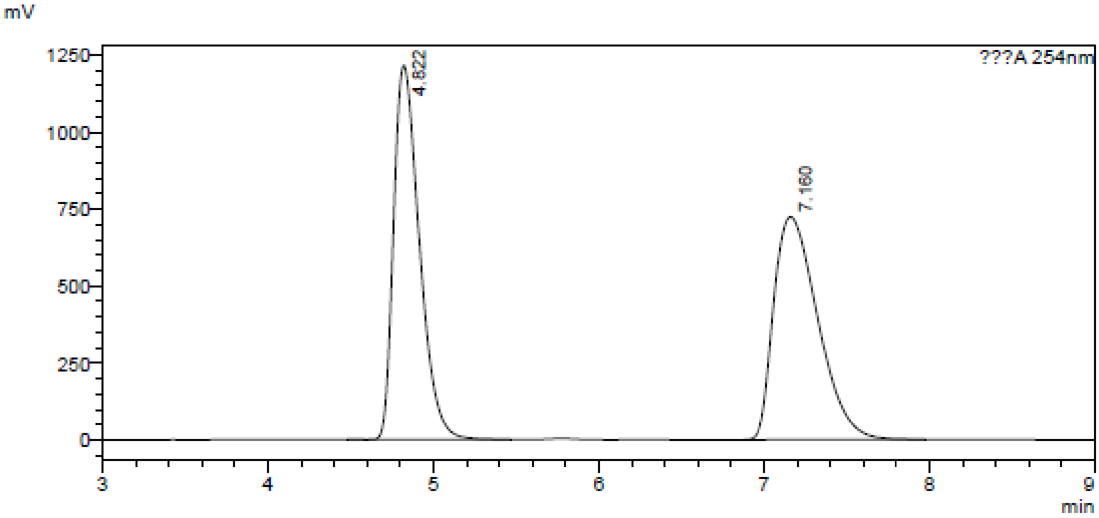

<Peak Table>

???A 254nm

Peak#	Ret. Time	Area	Height	Conc.	Unit	Mark	Name
1	4.822	13135008	1213514	49.533		M	
2	7.160	13382736	724237	50.467		M	
Total		26517744	1937751				

<Chromatogram>

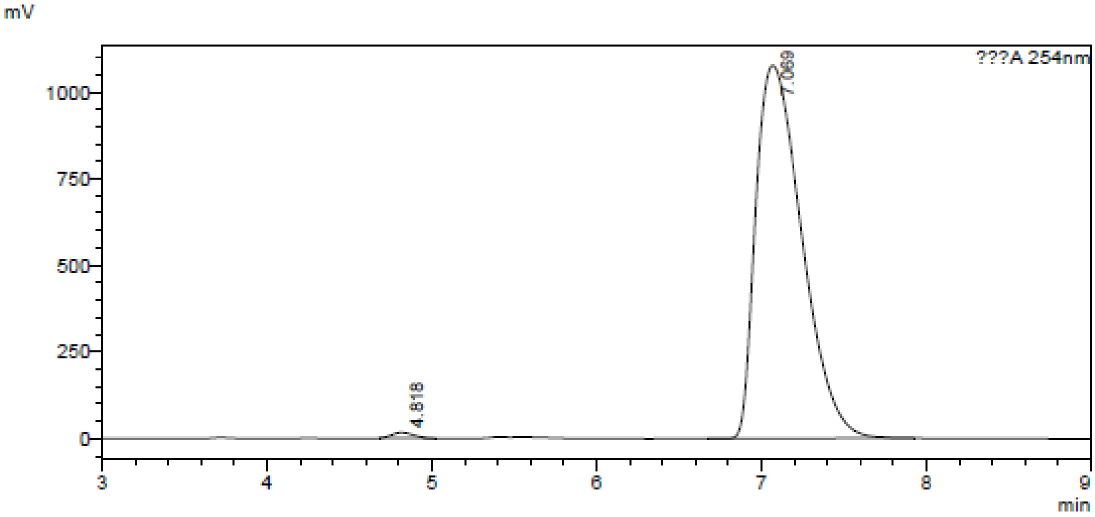

<Peak Table>

???A 254nm

Peak#	Ret. Time	Area	Height	Conc.	Unit	Mark	Name
1	4.818	150330	16436	0.714		M	
2	7.069	20901289	1074760	99.286		M	
Total		21051620	1091196				

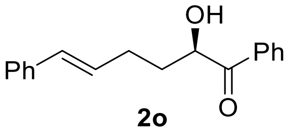

2o

<Chromatogram>

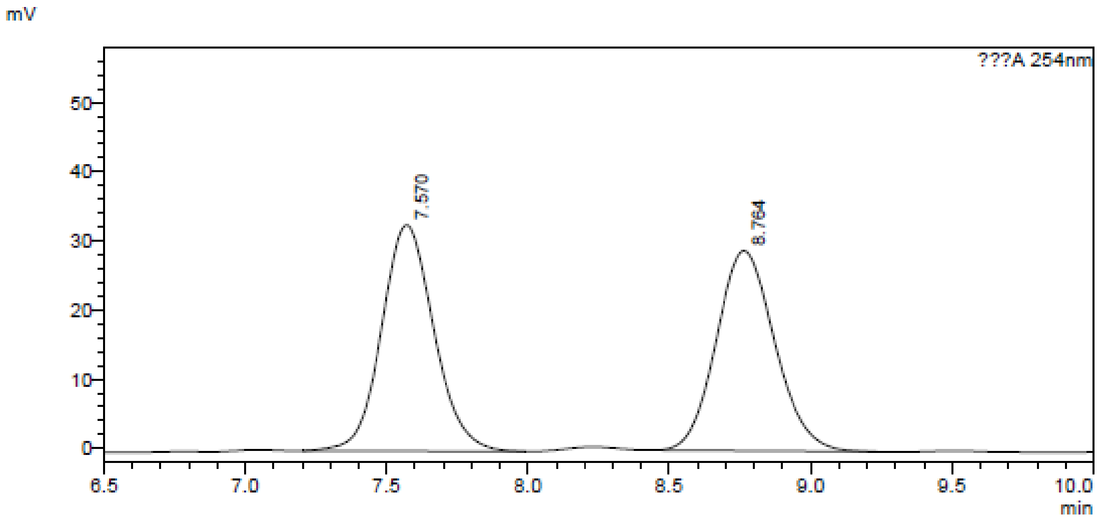

<Peak Table>

???A 254nm

Peak#	Ret. Time	Area	Height	Conc.	Unit	Mark	Name
1	7.570	421087	32648	50.279		M	
2	8.764	416416	28899	49.721		M	
Total		837503	61547				

<Chromatogram>

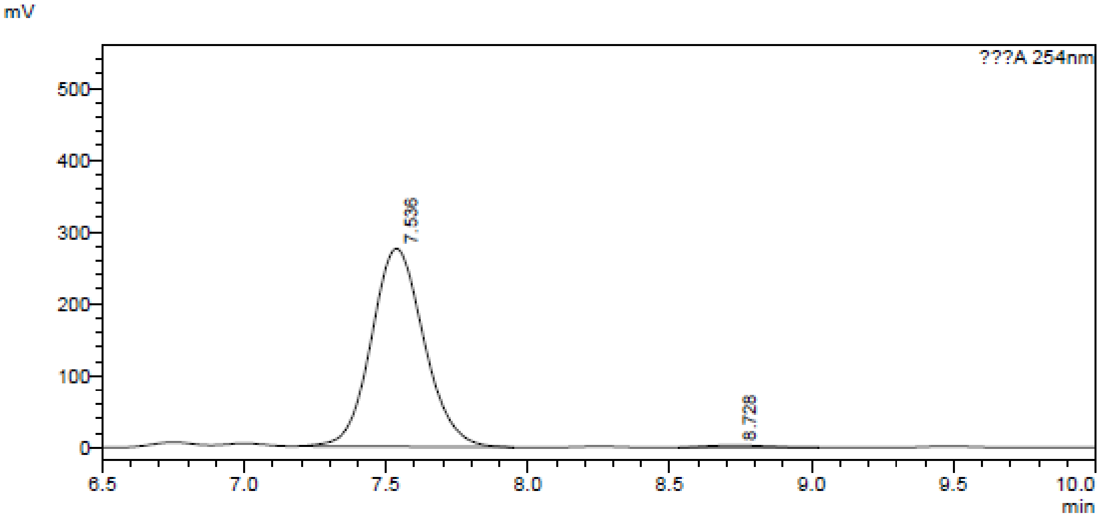

<Peak Table>

???A 254nm

Peak#	Ret. Time	Area	Height	Conc.	Unit	Mark	Name
1	7.536	3550263	275654	98.903			
2	8.728	39371	2942	1.097		M	
Total		3589634	278596				

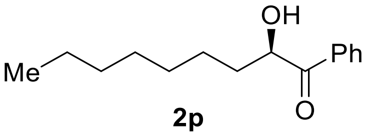

2p

<Chromatogram>

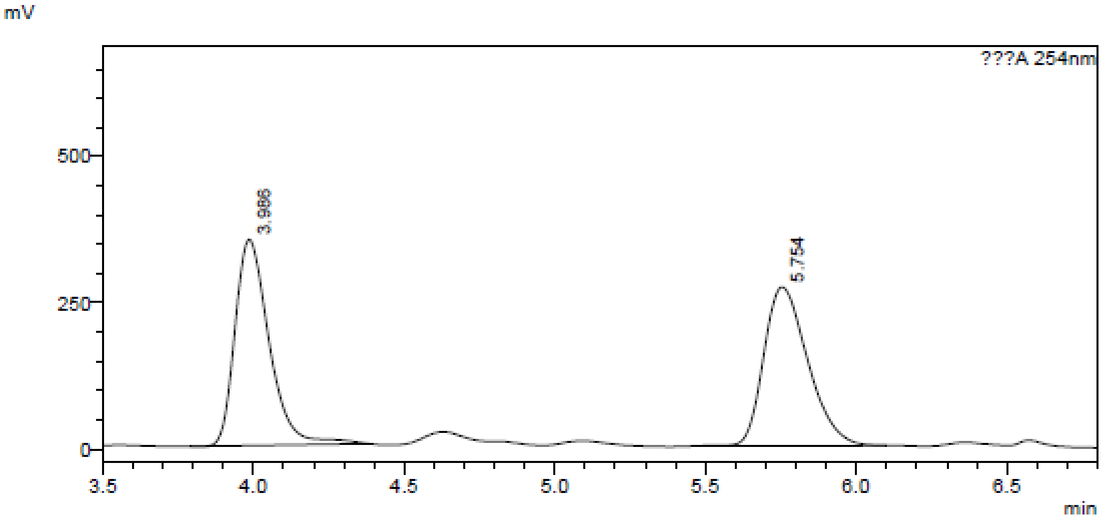

<Peak Table>

???A 254nm

Peak#	Ret. Time	Area	Height	Conc.	Unit	Mark	Name
1	3.986	2779630	352013	50.275		M	
2	5.754	2749200	271204	49.725		M	
Total		5528831	623217				

<Chromatogram>

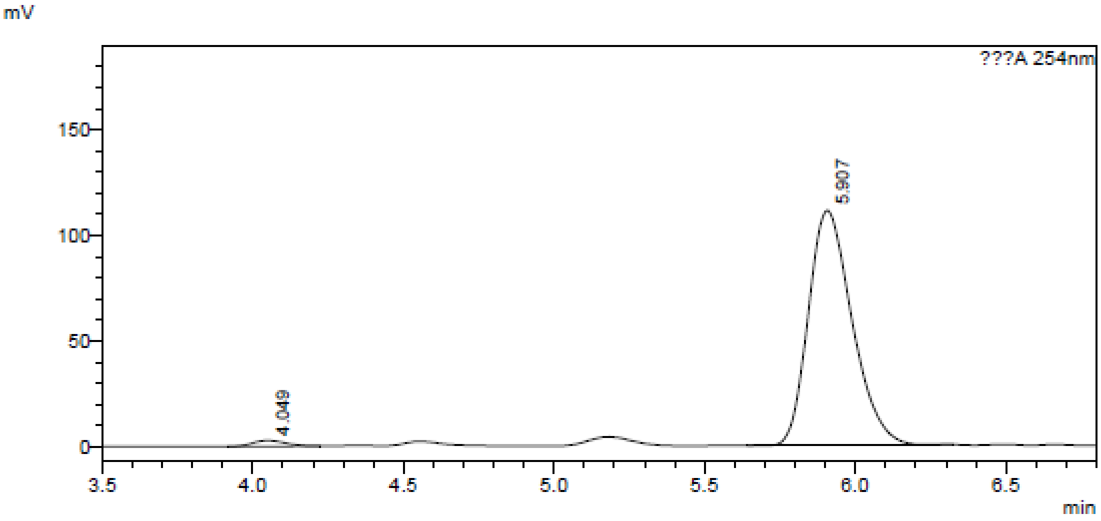

<Peak Table>

???A 254nm

Peak#	Ret. Time	Area	Height	Conc.	Unit	Mark	Name
1	4.049	21276	2839	1.870		M	
2	5.907	1116425	111131	98.130		M	
Total		1137700	113970				

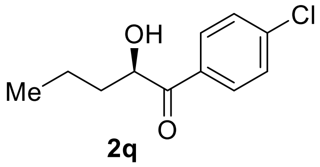

2q

<Chromatogram>

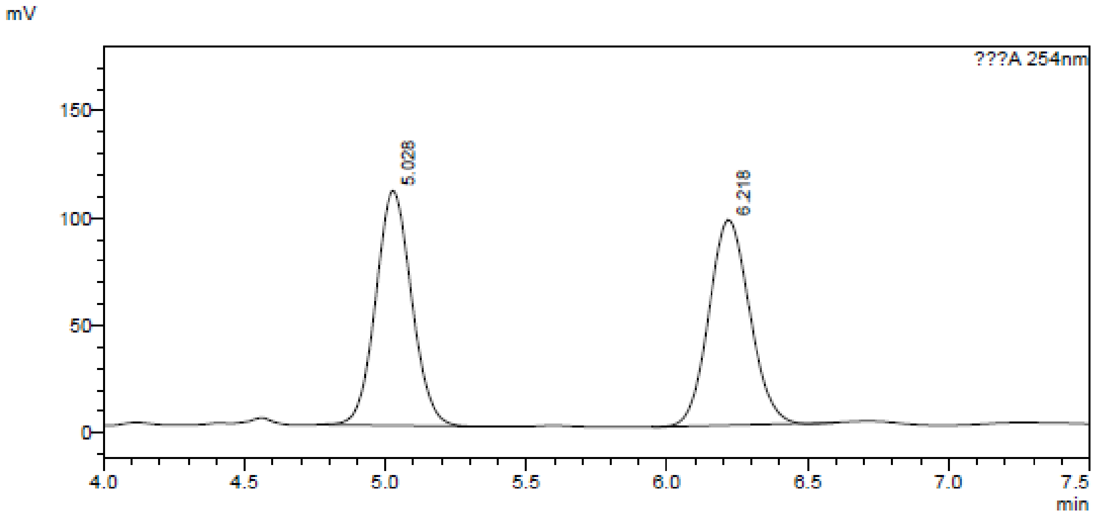

<Peak Table>

???A 254nm

Peak#	Ret. Time	Area	Height	Conc.	Unit	Mark	Name
1	5.028	971468	109298	50.254		M	
2	6.218	961653	95435	49.746		M	
Total		1933121	204733				

<Chromatogram>

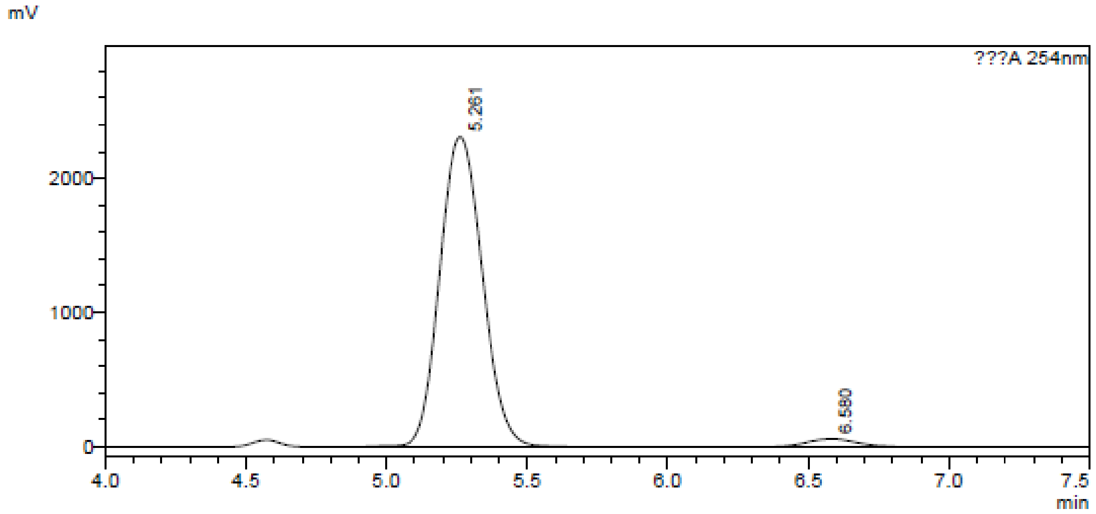

<Peak Table>

???A 254nm

Peak#	Ret. Time	Area	Height	Conc.	Unit	Mark	Name
1	5.261	24311176	2308163	97.355		M	
2	6.580	660481	57024	2.645		M	
Total		24971656	2365187				

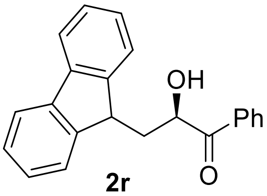

2r

<Chromatogram>

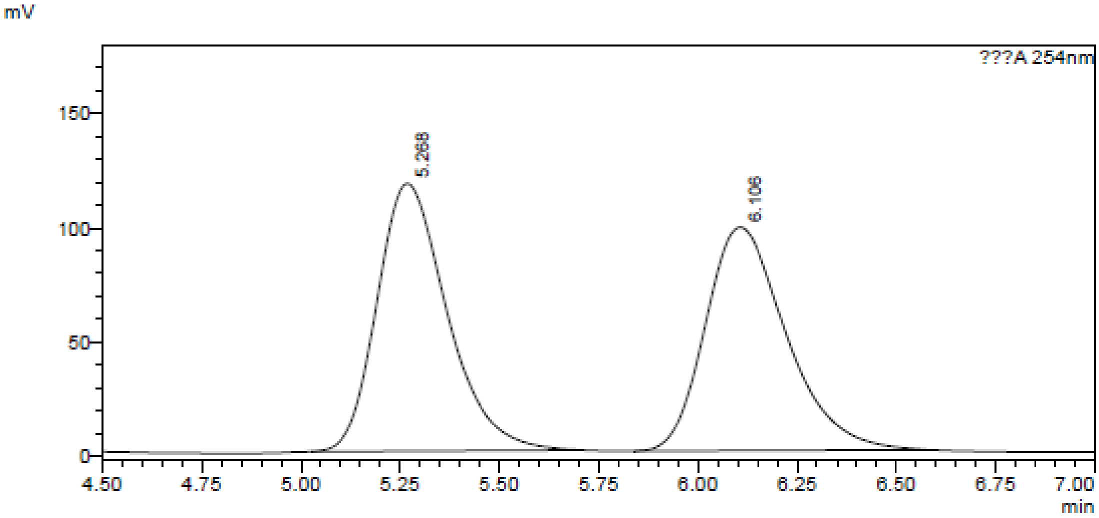

<Peak Table>

???A 254nm

Peak#	Ret. Time	Area	Height	Conc.	Unit	Mark	Name
1	5.268	1439888	117305	50.860			
2	6.106	1391199	98035	49.140			
Total		2831086	215340				

<Chromatogram>

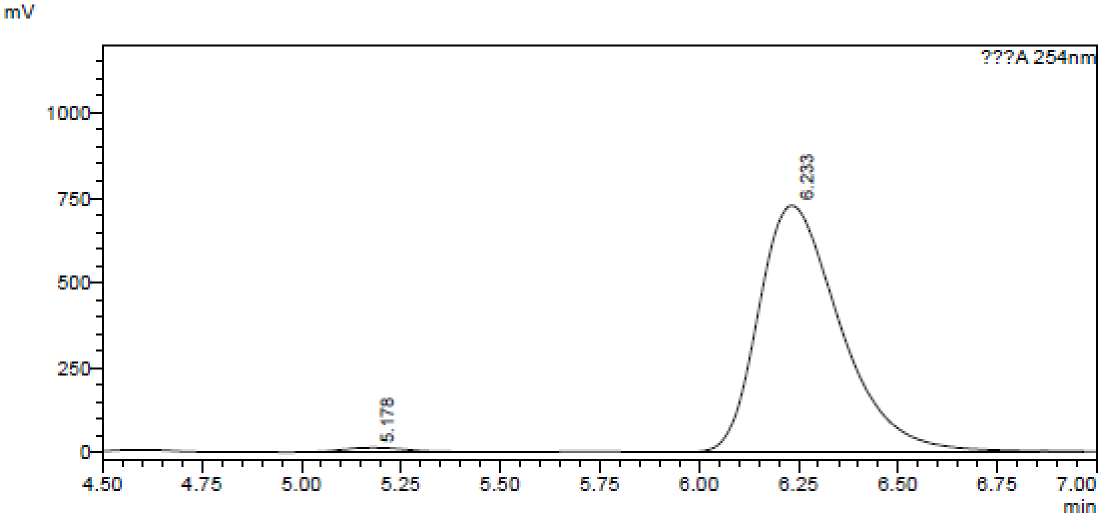

<Peak Table>

???A 254nm

Peak#	Ret. Time	Area	Height	Conc.	Unit	Mark	Name
1	5.178	142136	13210	1.350			
2	6.233	10389170	726844	98.650			
Total		10531306	740054				

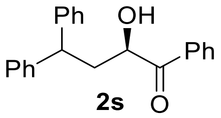

2s

<Chromatogram>

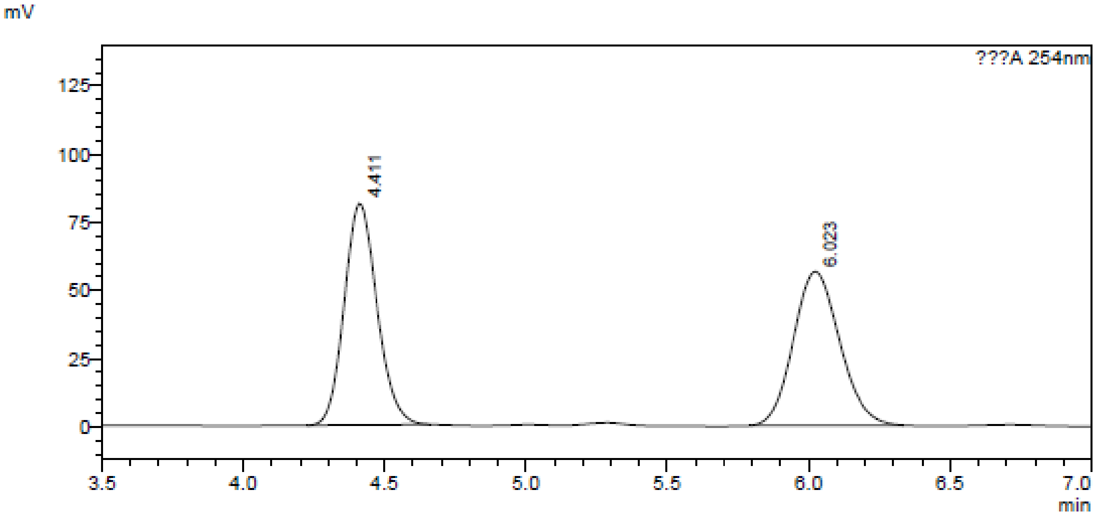

<Peak Table>

???A 254nm

Peak#	Ret. Time	Area	Height	Conc.	Unit	Mark	Name
1	4.411	659085	81237	50.471			
2	6.023	646782	56527	49.529			
Total		1305868	137764				

<Chromatogram>

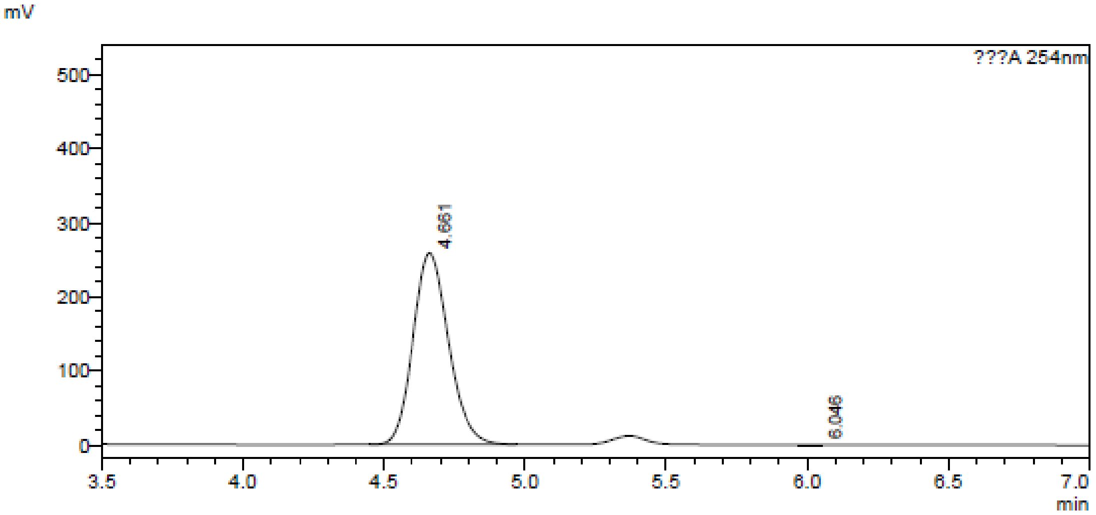

<Peak Table>

???A 254nm

Peak#	Ret. Time	Area	Height	Conc.	Unit	Mark	Name
1	4.661	2262650	258163	99.997			
2	6.046	75	20	0.003		M	
Total		2262725	258183				

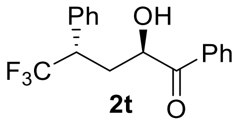

2t

<Chromatogram>

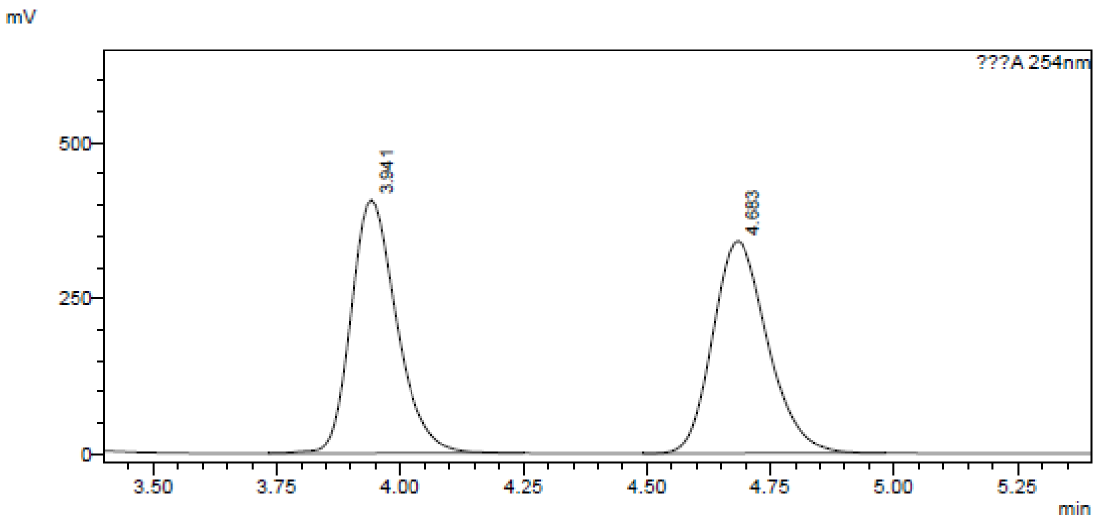

<Peak Table>

???A 254nm

Peak#	Ret. Time	Area	Height	Conc.	Unit	Mark	Name
1	3.941	2618990	408071	49.696			
2	4.683	2651028	342057	50.304			
Total		5270018	750129				

<Chromatogram>

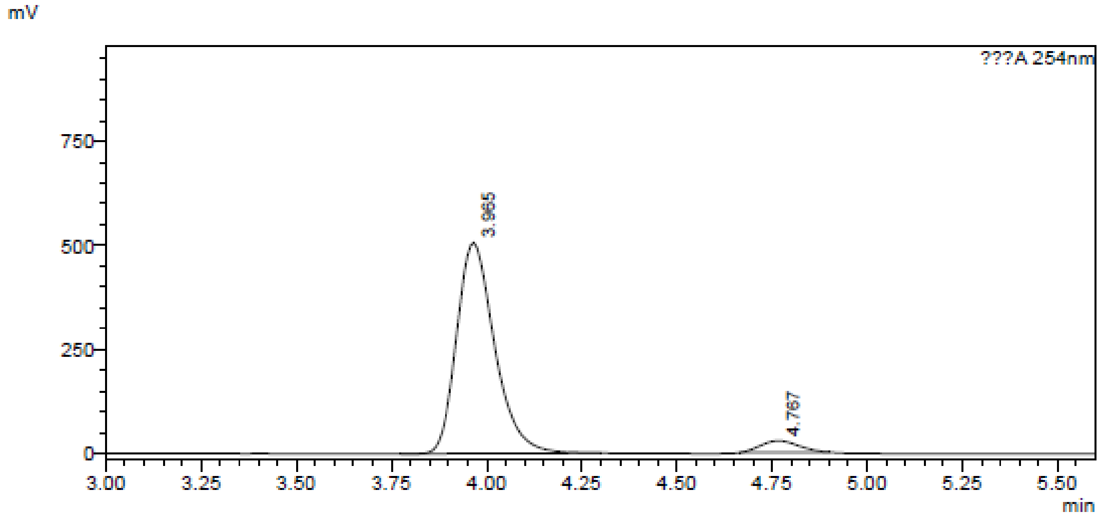

<Peak Table>

???A 254nm

Peak#	Ret. Time	Area	Height	Conc.	Unit	Mark	Name
1	3.965	3505897	506402	94.815		M	
2	4.767	191705	27587	5.185		M	
Total		3697602	533989				

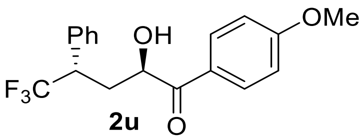

2u

<Chromatogram>

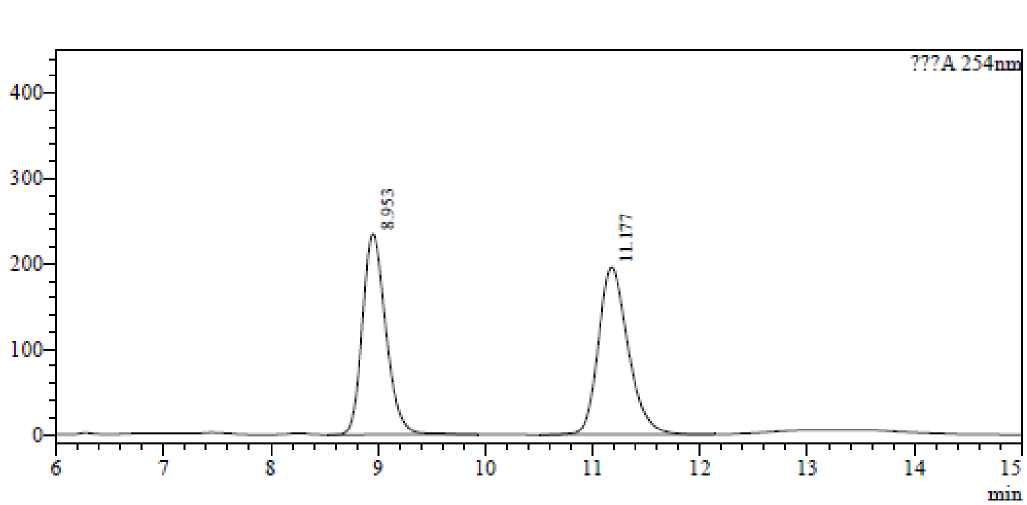

<Peak Table>

???A 254nm

Peak#	Ret. Time	Area	Height	Conc.	Unit	Mark	Name
1	8.953	3582263	235316	48.539			
2	11.177	3797985	195794	51.461			
Total		7380248	431111				

<Chromatogram>

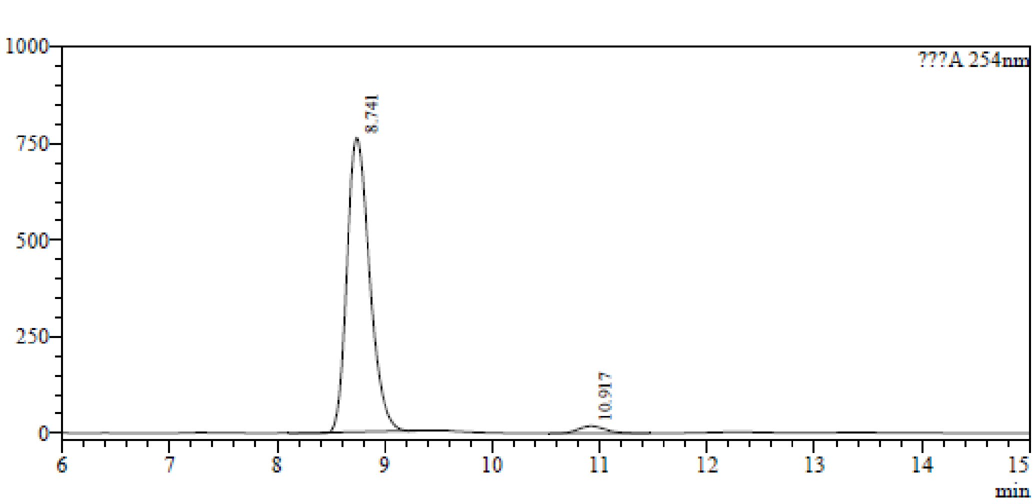

<Peak Table>

???A 254nm

Peak#	Ret. Time	Area	Height	Conc.	Unit	Mark	Name
1	8.741	11129931	761538	97.078		M	
2	10.917	335041	18930	2.922			
Total		11464972	780468				

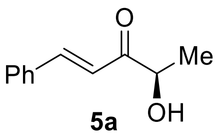

5a

<Chromatogram>

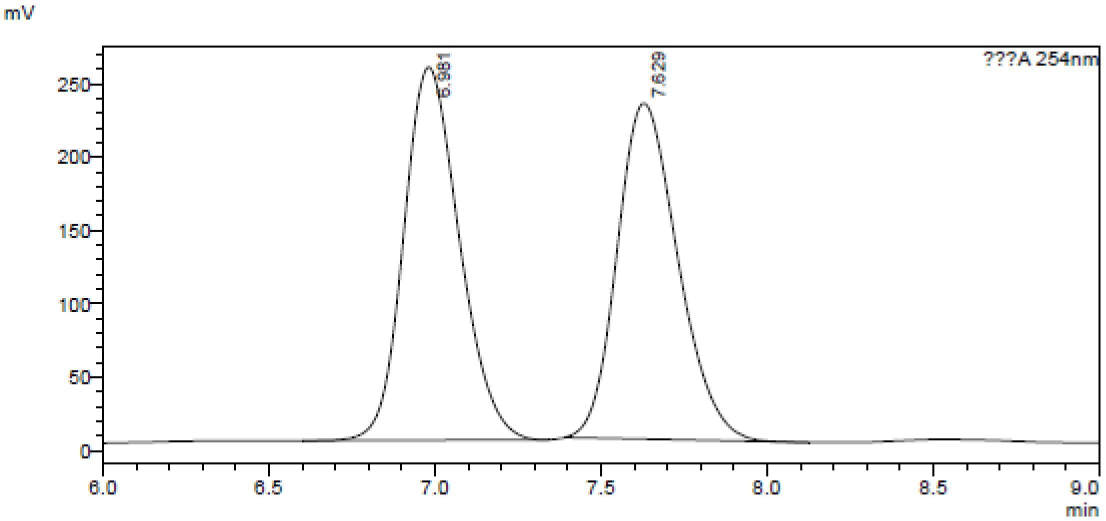

<Peak Table>

???A 254nm

Peak#	Ret. Time	Area	Height	Conc.	Unit	Mark	Name
1	6.981	2969156	254349	50.476		M	
2	7.629	2913111	228757	49.524		M	
Total		5882267	483106				

<Chromatogram>

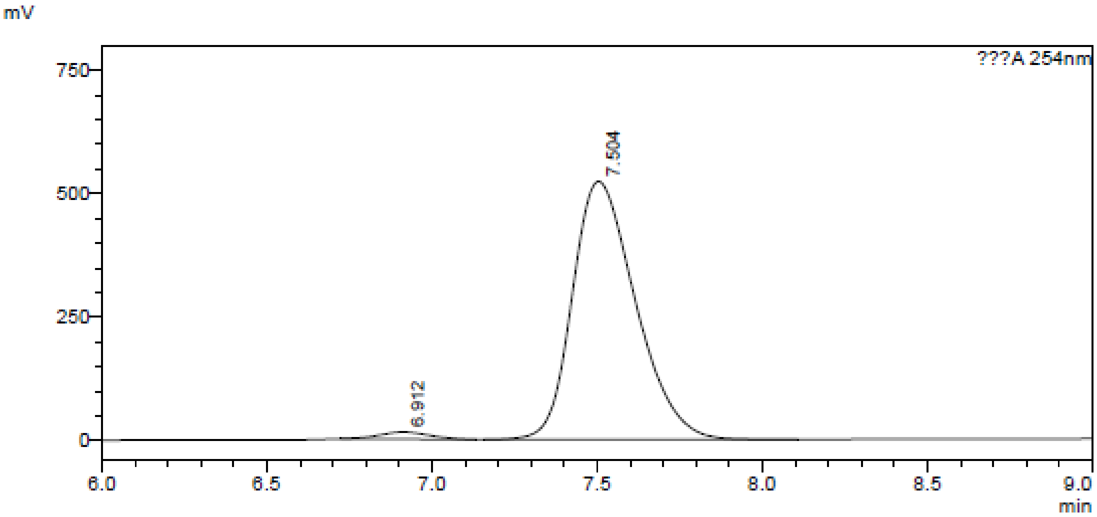

<Peak Table>

???A 254nm

Peak#	Ret. Time	Area	Height	Conc.	Unit	Mark	Name
1	6.912	148552	14132	2.087		M	
2	7.504	6968277	522950	97.913		M	
Total		7116829	537082				

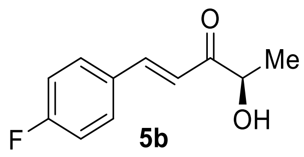

5b

<Chromatogram>

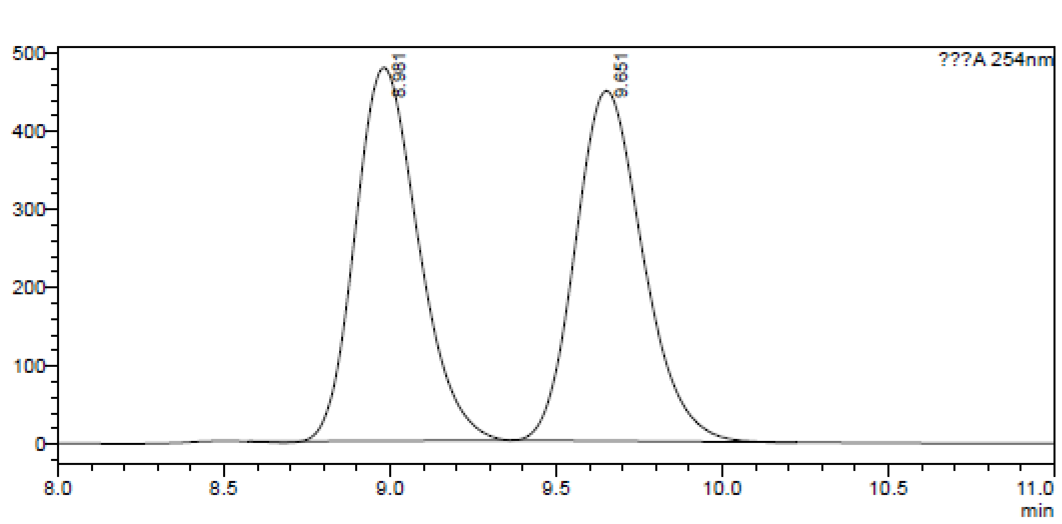

<Peak Table>

???A 254nm

Peak#	Ret. Time	Area	Height	Conc.	Unit	Mark	Name
1	8.981	6300485	477257	49.823		M	
2	9.651	6345140	447732	50.177		M	
Total		12645625	924989				

<Chromatogram>

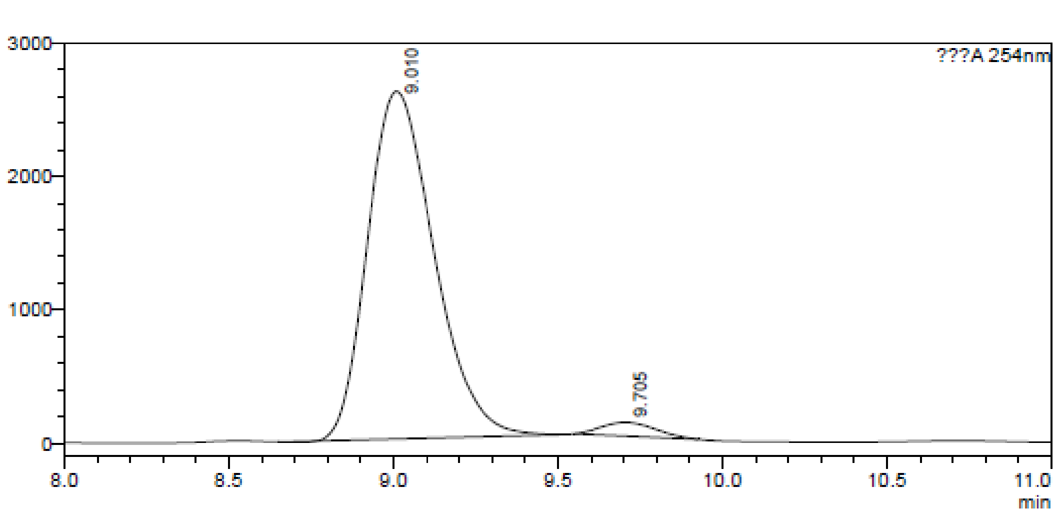

<Peak Table>

???A 254nm

Peak#	Ret. Time	Area	Height	Conc.	Unit	Mark	Name
1	9.010	36653362	2607208	96.920		M	
2	9.705	1164761	101934	3.080		M	
Total		37818123	2709142				

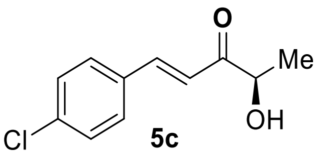

5c

<Chromatogram>

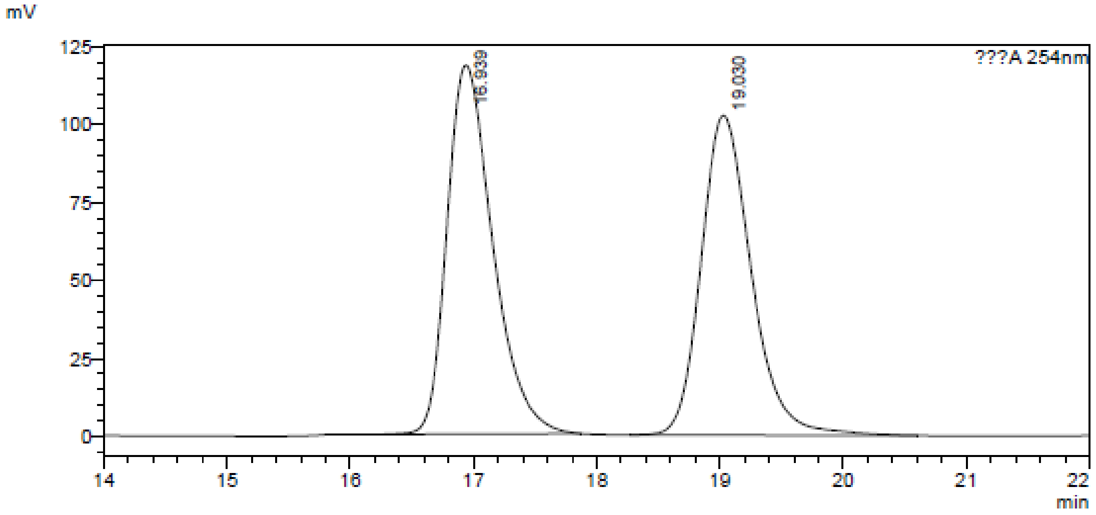

<Peak Table>

???A 254nm

Peak#	Ret. Time	Area	Height	Conc.	Unit	Mark	Name
1	16.939	3055077	118242	51.252		M	
2	19.030	2905769	102467	48.748		M	
Total		5960845	220710				

<Chromatogram>

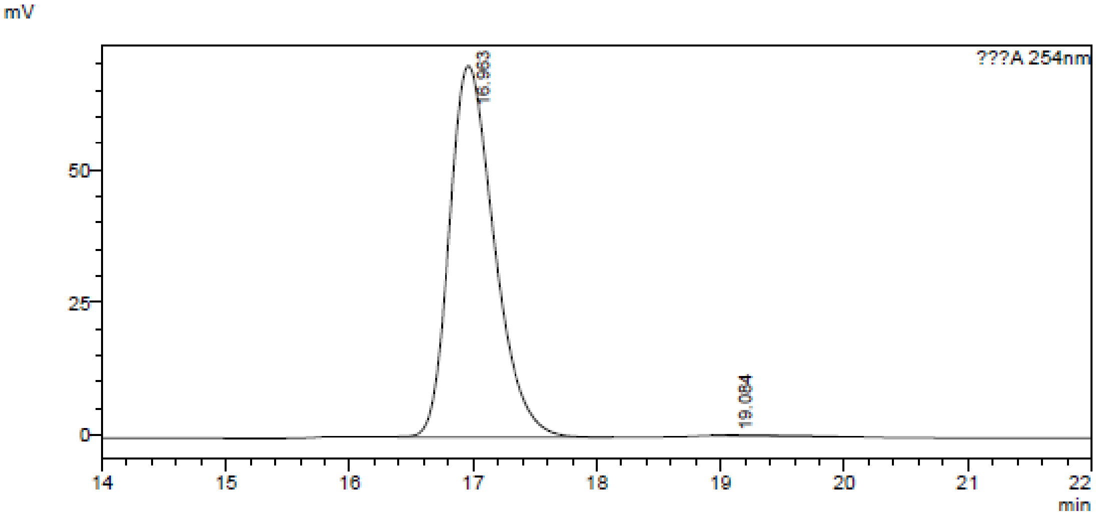

<Peak Table>

???A 254nm

Peak#	Ret. Time	Area	Height	Conc.	Unit	Mark	Name
1	16.963	1764269	69957	99.671			
2	19.084	5818	126	0.329		M	
Total		1770087	70083				

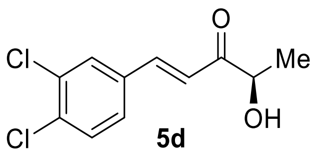

5d

<Chromatogram>

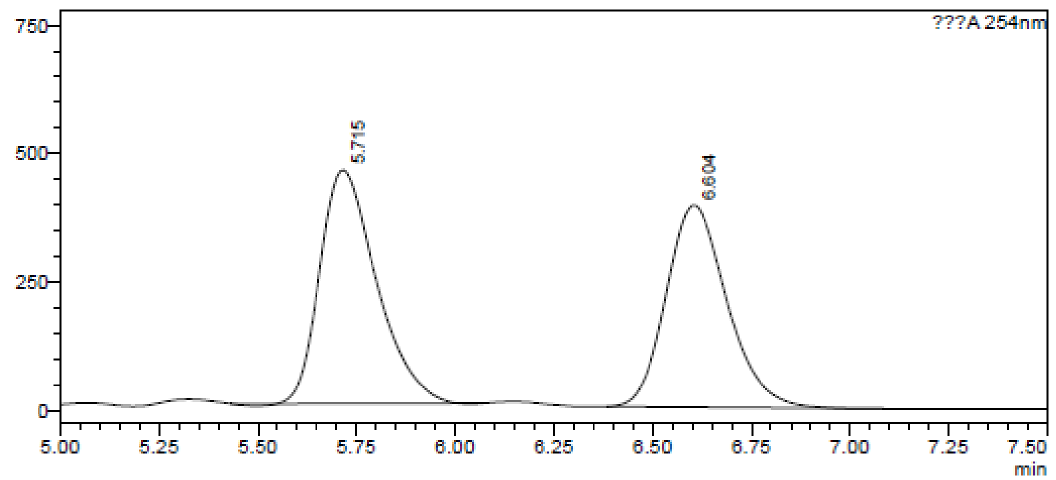

<Peak Table>

???A 254nm

Peak#	Ret. Time	Area	Height	Conc.	Unit	Mark	Name
1	5.715	4526864	454933	51.733		M	
2	6.604	4223564	392644	48.267		M	
Total		8750428	847577				

<Chromatogram>

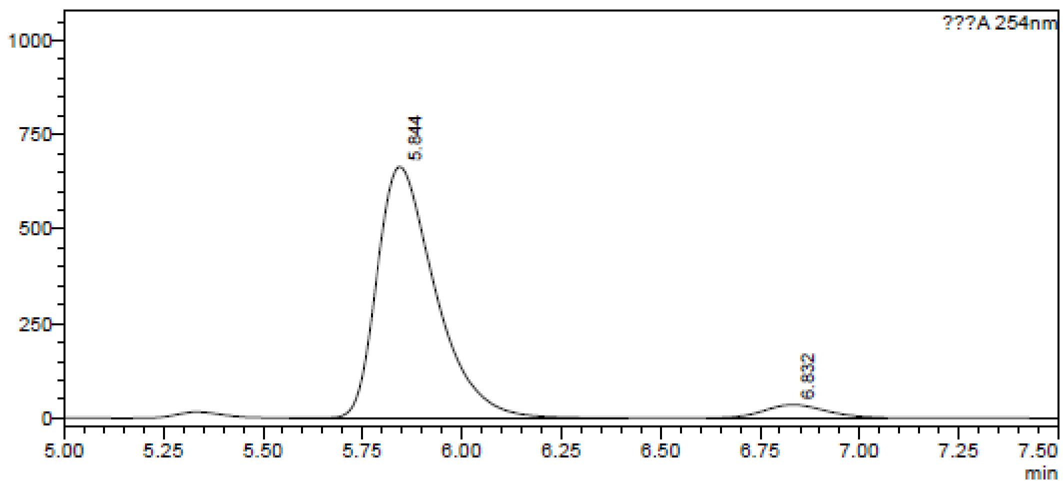

<Peak Table>

???A 254nm

Peak#	Ret. Time	Area	Height	Conc.	Unit	Mark	Name
1	5.844	6802134	665007	94.908		M	
2	6.832	364965	34531	5.092		M	
Total		7167099	699538				

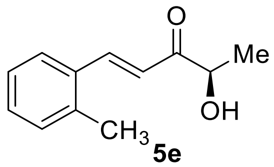

5e

<Chromatogram>

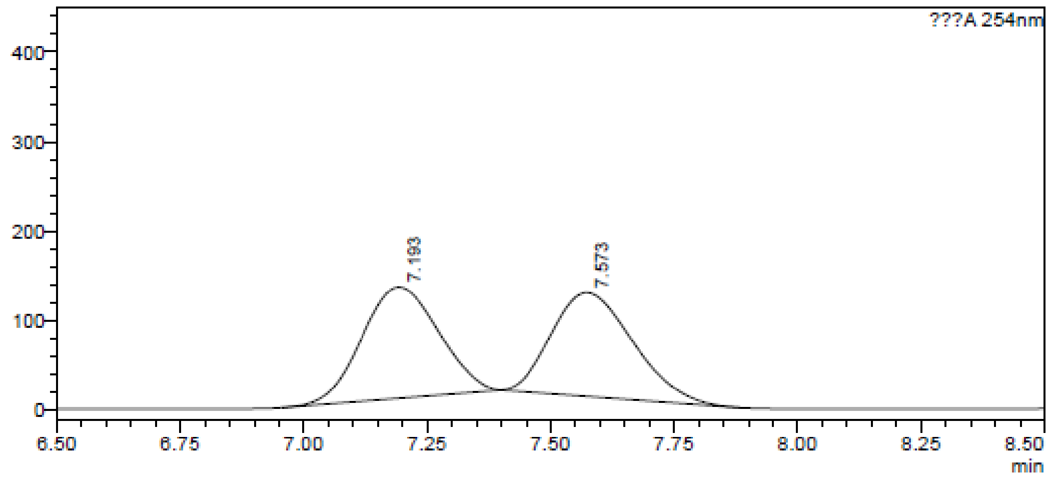

<Peak Table>

???A 254nm

Peak#	Ret. Time	Area	Height	Conc.	Unit	Mark	Name
1	7.193	1347506	124281	50.385			
2	7.573	1326922	116063	49.615		M	
Total		2674428	240344				

<Chromatogram>

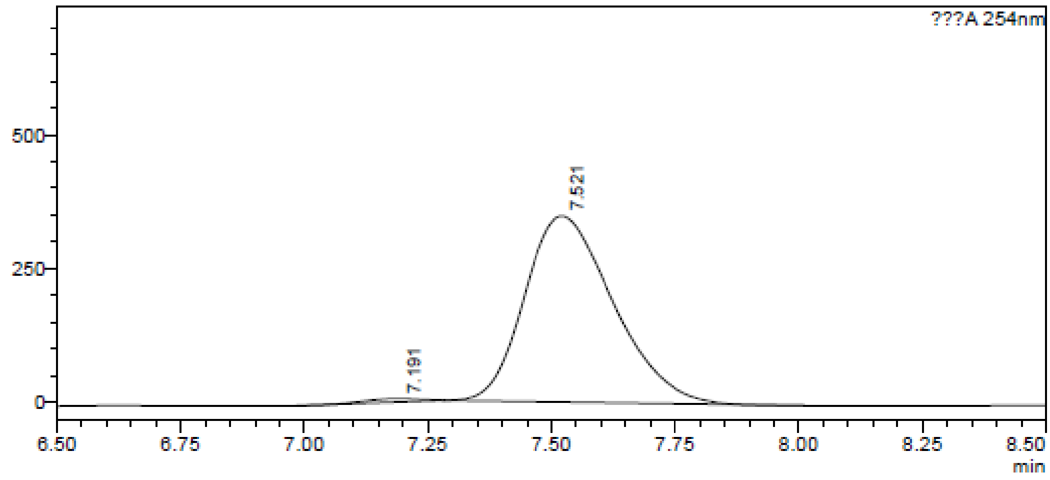

<Peak Table>

???A 254nm

Peak#	Ret. Time	Area	Height	Conc.	Unit	Mark	Name
1	7.191	46851	6507	1.067		M	
2	7.521	4344343	347671	98.933		M	
Total		4391195	354178				

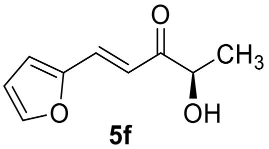

5f

<Chromatogram>

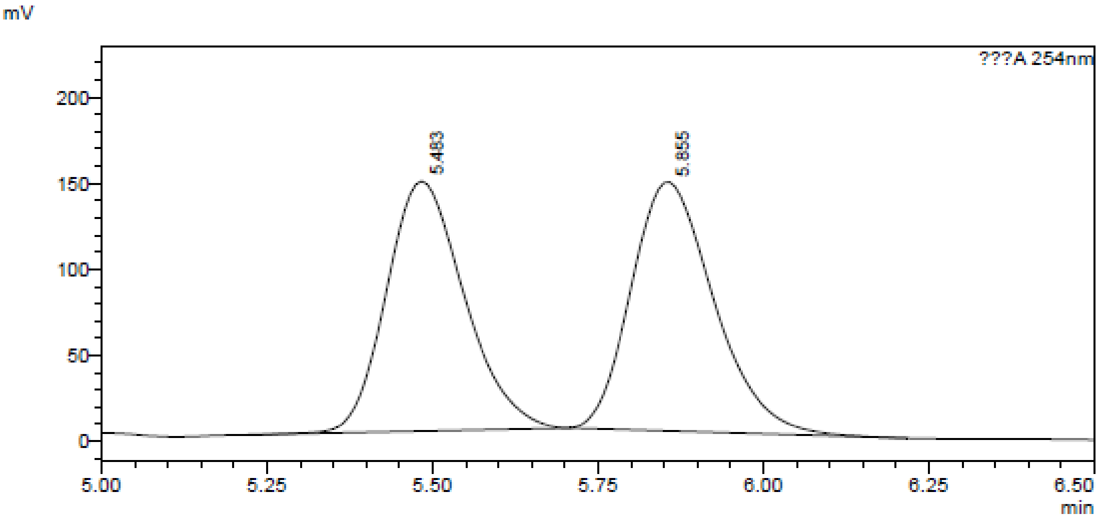

<Peak Table>

???A 254nm

Peak#	Ret. Time	Area	Height	Conc.	Unit	Mark	Name
1	5.483	1193672	145625	48.581		M	
2	5.855	1263395	145006	51.419		M	
Total		2457067	290631				

<Chromatogram>

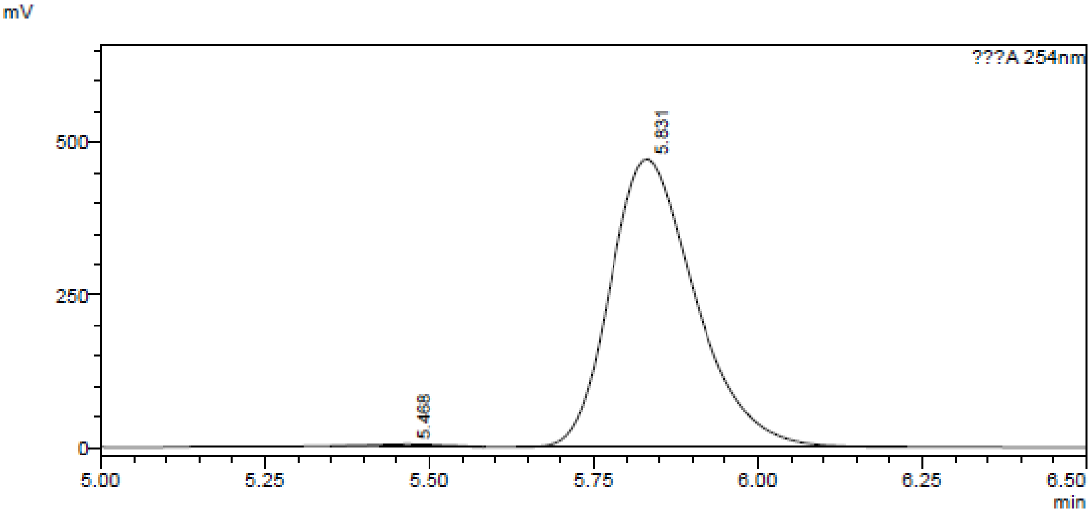

<Peak Table>

???A 254nm

Peak#	Ret. Time	Area	Height	Conc.	Unit	Mark	Name
1	5.468	23848	3474	0.559		M	
2	5.831	4240237	470836	99.441			
Total		4264085	474310				

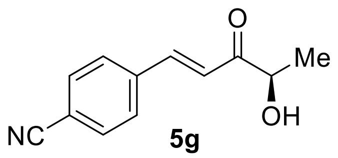

5g

<Chromatogram>

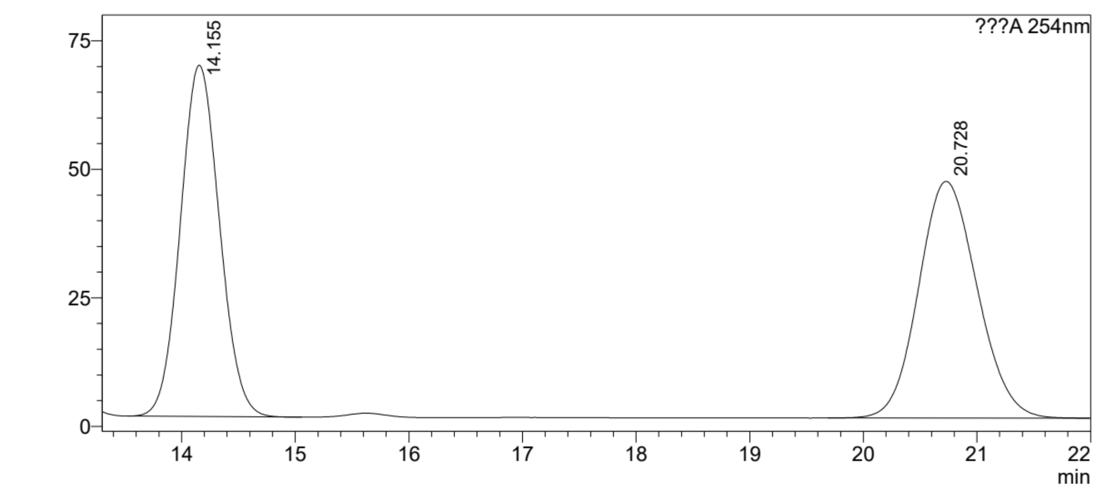

<Peak Table>

???A 254nm

Peak#	Ret. Time	Area	Height	Conc.	Unit	Mark	Name
1	14.155	1639675	68355	49.791			
2	20.728	1653445	46039	50.209			
Total		3293121	114394				

<Chromatogram>

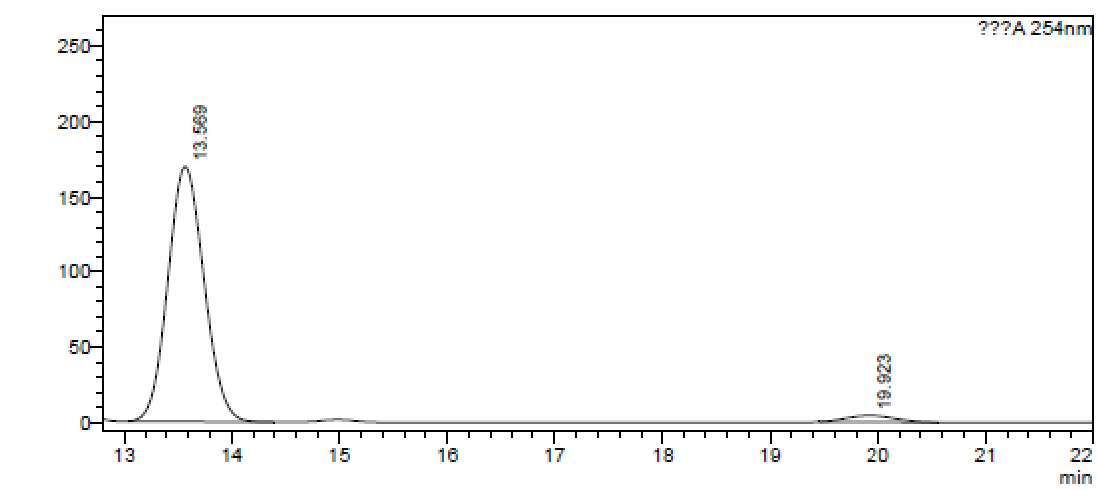

<Peak Table>

???A 254nm

Peak#	Ret. Time	Area	Height	Conc.	Unit	Mark	Name
1	13.569	3939900	169464	96.598		M	
2	19.923	138763	4468	3.402		M	
Total		4078663	173932				

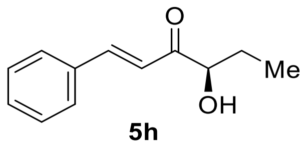

5h

<Chromatogram>

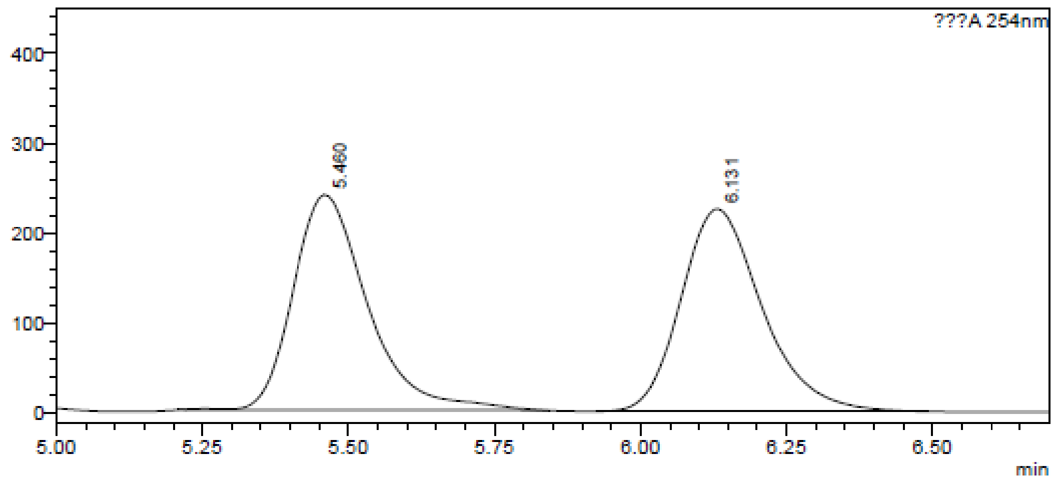

<Peak Table>

???A 254nm

Peak#	Ret. Time	Area	Height	Conc.	Unit	Mark	Name
1	5.460	2132390	239810	49.390		M	
2	6.131	2185063	225725	50.610		M	
Total		4317454	465535				

<Chromatogram>

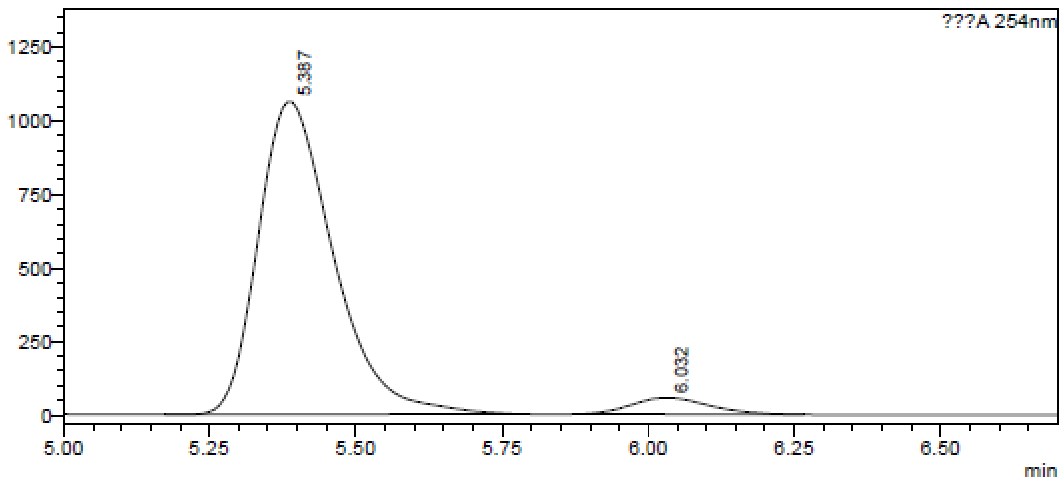

<Peak Table>

???A 254nm

Peak#	Ret. Time	Area	Height	Conc.	Unit	Mark	Name
1	5.387	9518828	1062241	94.832		M	
2	6.032	518765	55867	5.168		M	
Total		10037592	1118109				

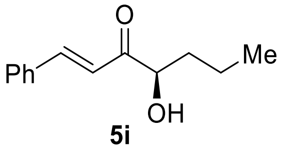

5i

<Chromatogram>

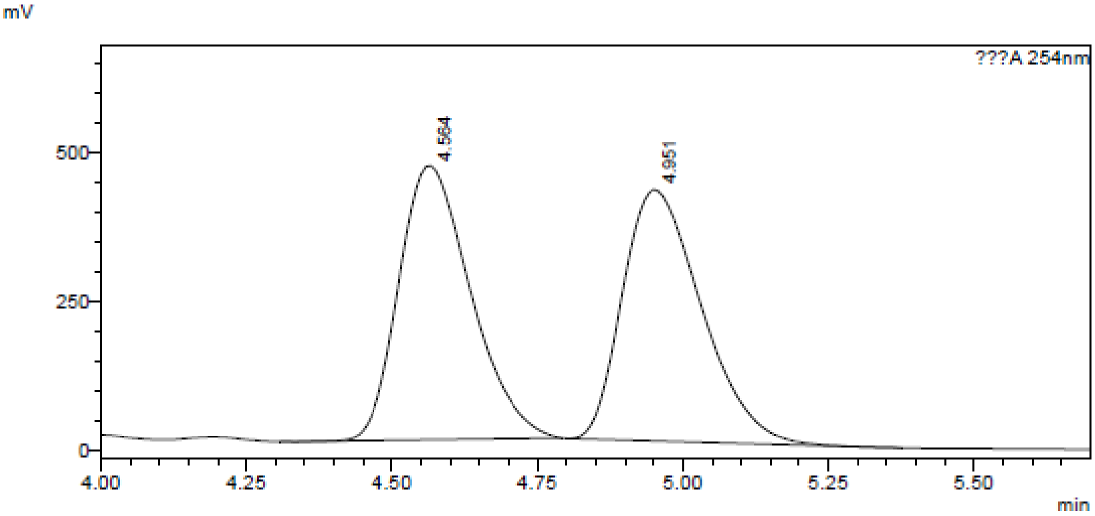

<Peak Table>

???A 254nm

Peak#	Ret. Time	Area	Height	Conc.	Unit	Mark	Name
1	4.564	3950274	460393	49.673		M	
2	4.951	4002261	422774	50.327		M	
Total		7952535	883167				

<Chromatogram>

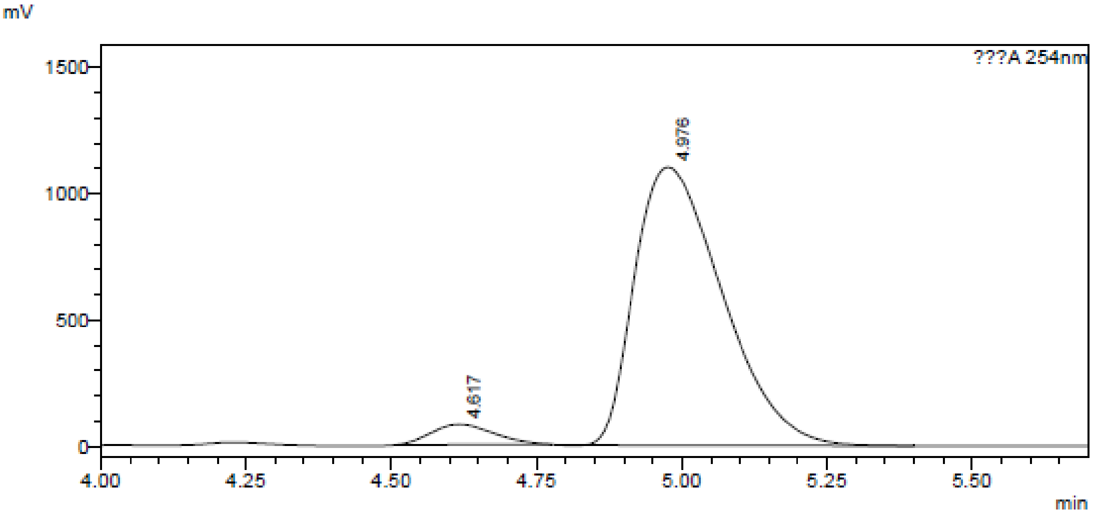

<Peak Table>

???A 254nm

Peak#	Ret. Time	Area	Height	Conc.	Unit	Mark	Name
1	4.617	599794	80411	4.917		M	
2	4.976	11597455	1102388	95.083		M	
Total		12197249	1182799				

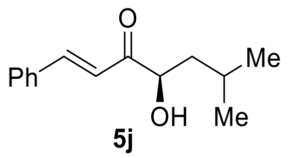

5j

<Chromatogram>

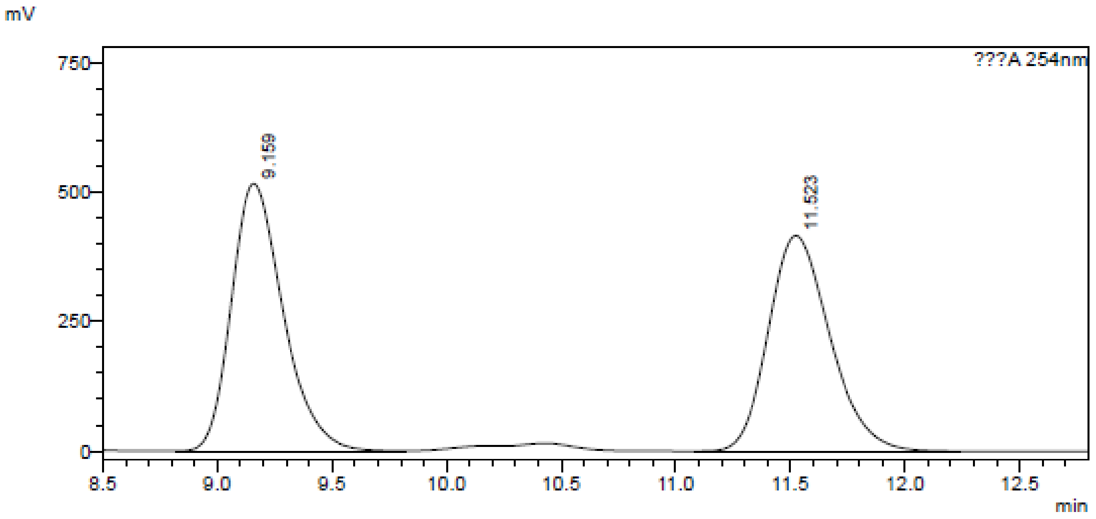

<Peak Table>

???A 254nm

Peak#	Ret. Time	Area	Height	Conc.	Unit	Mark	Name
1	9.159	8131148	516570	51.118		M	
2	11.523	7775536	416178	48.882		M	
Total		15906683	932748				

<Chromatogram>

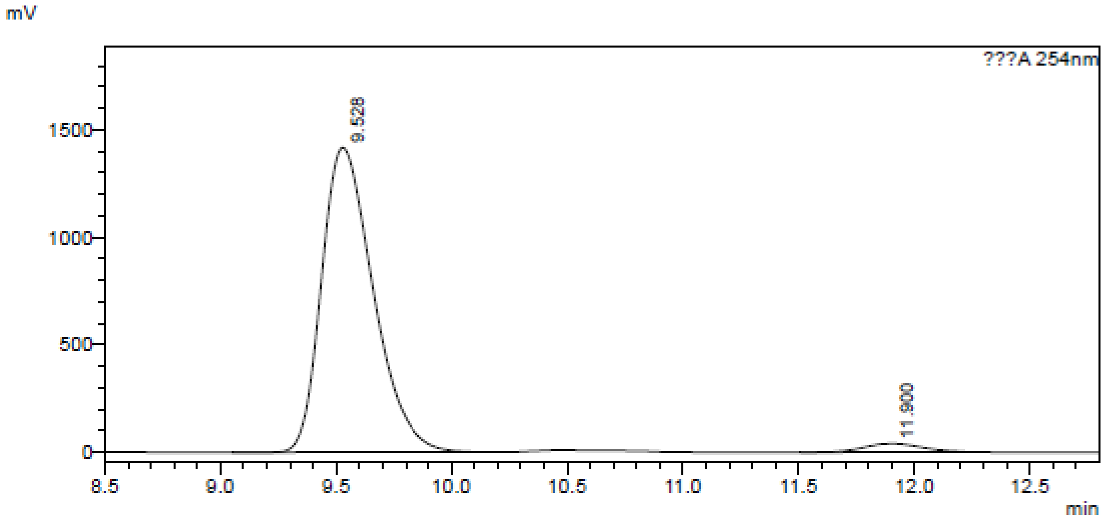

<Peak Table>

???A 254nm

Peak#	Ret. Time	Area	Height	Conc.	Unit	Mark	Name
1	9.528	22020761	1415470	96.988		M	
2	11.900	683955	39712	3.012		M	
Total		22704716	1455182				

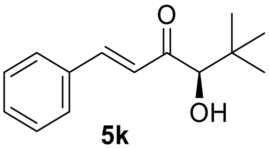

5k

<Chromatogram>

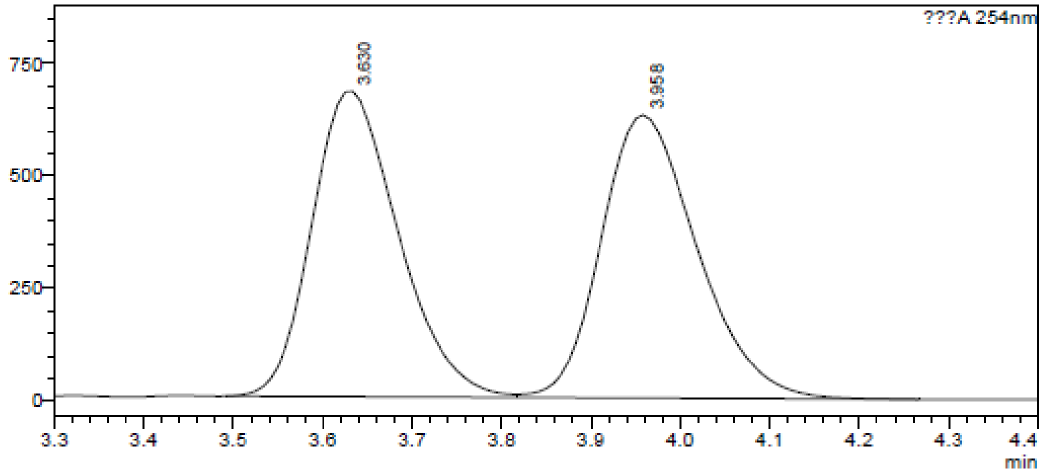

<Peak Table>

???A 254nm

Peak#	Ret. Time	Area	Height	Conc.	Unit	Mark	Name
1	3.630	4574814	678719	49.404			
2	3.958	4685236	629259	50.596		V	
Total		9260050	1307978				

<Chromatogram>

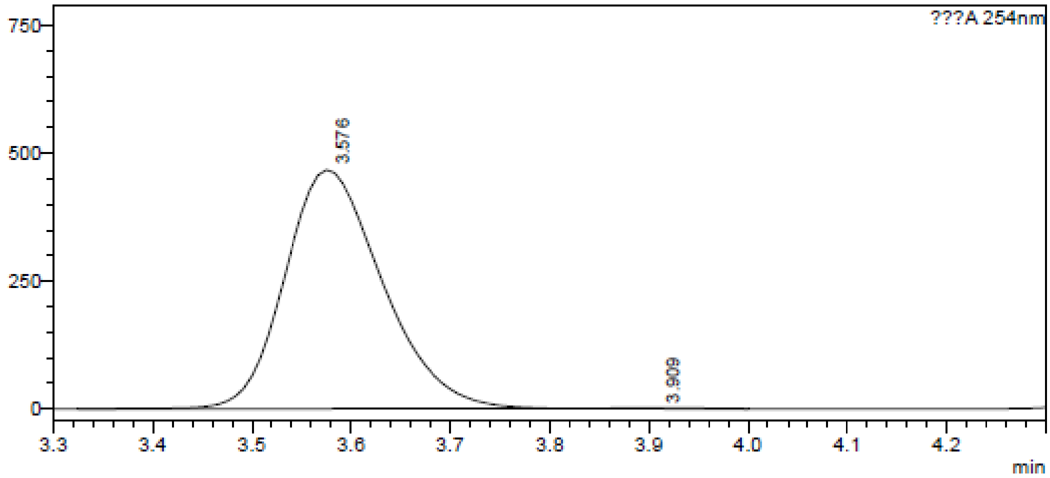

<Peak Table>

???A 254nm

Peak#	Ret. Time	Area	Height	Conc.	Unit	Mark	Name
1	3.576	3202750	467666	99.698			
2	3.909	9699	1823	0.302		M	
Total		3212449	469489				

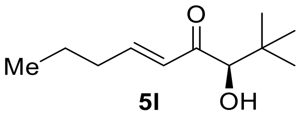

5l

<Chromatogram>

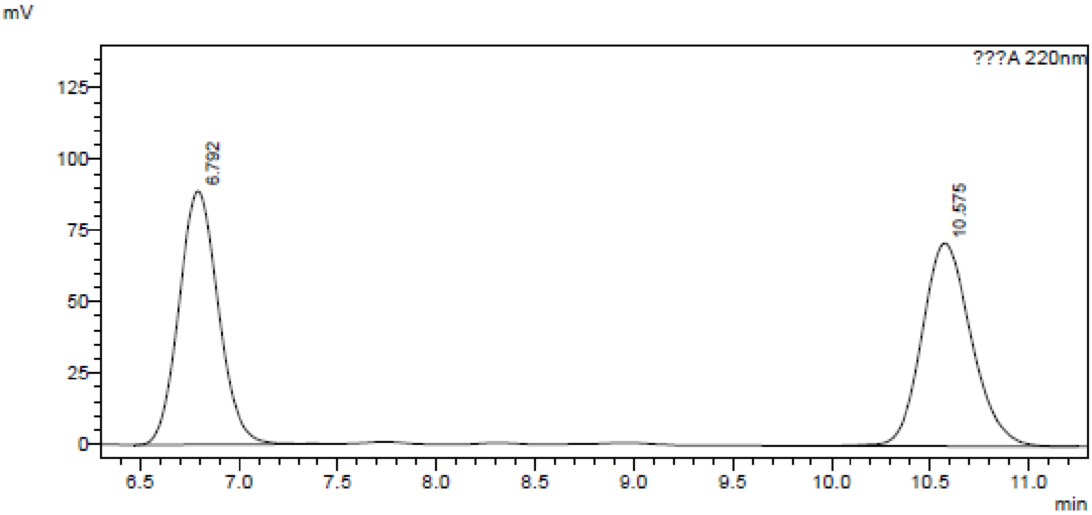

<Peak Table>

???A 220nm

Peak#	Ret. Time	Area	Height	Conc.	Unit	Mark	Name
1	6.792	1233714	88938	49.856		S	
2	10.575	1240853	71229	50.144			
Total		2474567	160168				

<Chromatogram>

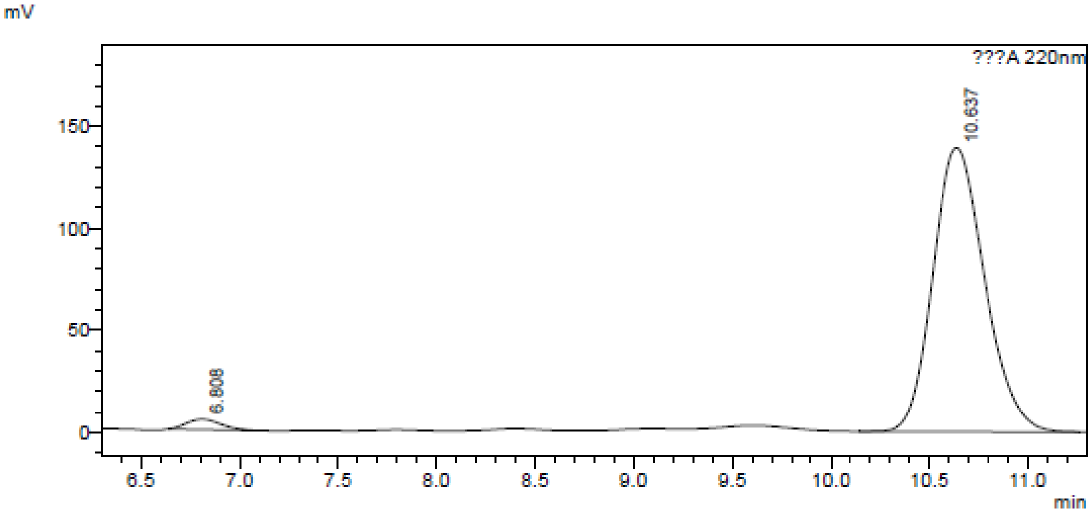

<Peak Table>

???A 220nm

Peak#	Ret. Time	Area	Height	Conc.	Unit	Mark	Name
1	6.808	61505	5088	2.361		M	
2	10.637	2543326	139251	97.639		M	
Total		2604832	144339				

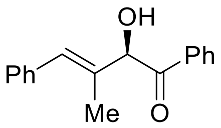

6a

<Chromatogram>

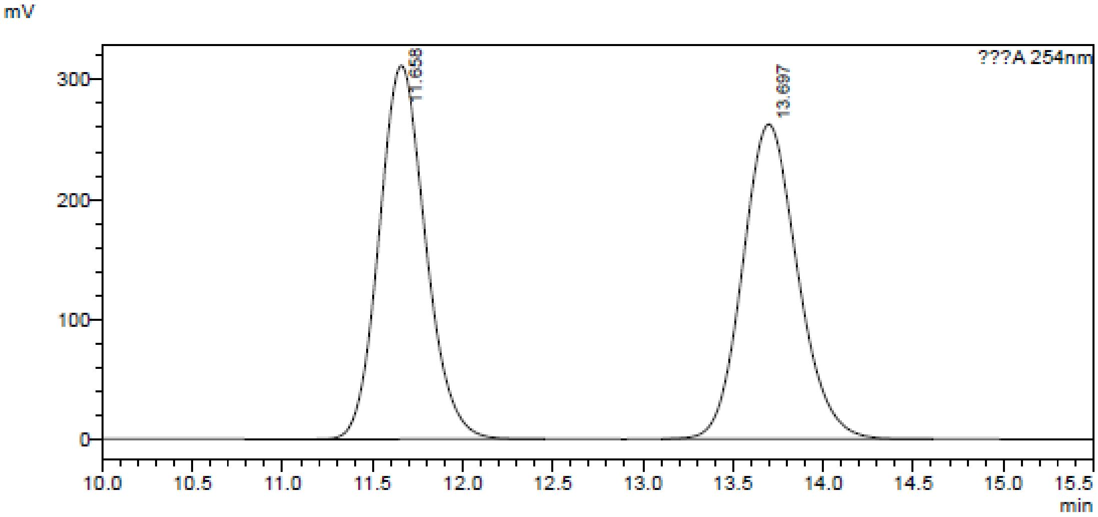

<Peak Table>

???A 254nm

Peak#	Ret. Time	Area	Height	Conc.	Unit	Mark	Name
1	11.658	5605568	311703	50.087		M	
2	13.697	5585989	262385	49.913		M	
Total		11191557	574089				

<Chromatogram>

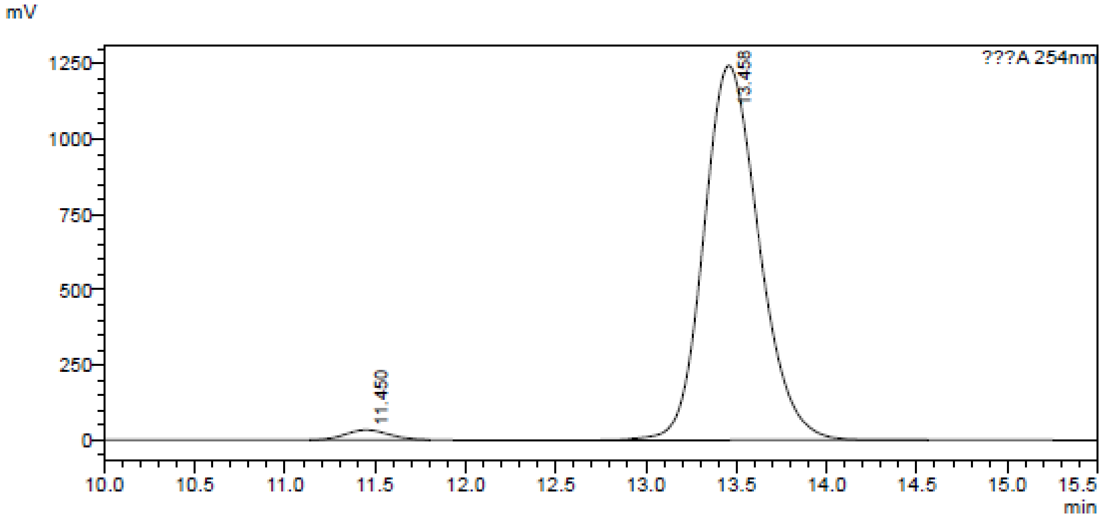

<Peak Table>

???A 254nm

Peak#	Ret. Time	Area	Height	Conc.	Unit	Mark	Name
1	11.450	542558	32446	2.003		M	
2	13.458	26547488	1242454	97.997			
Total		27090045	1274900				

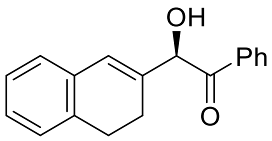

6b

<Chromatogram>

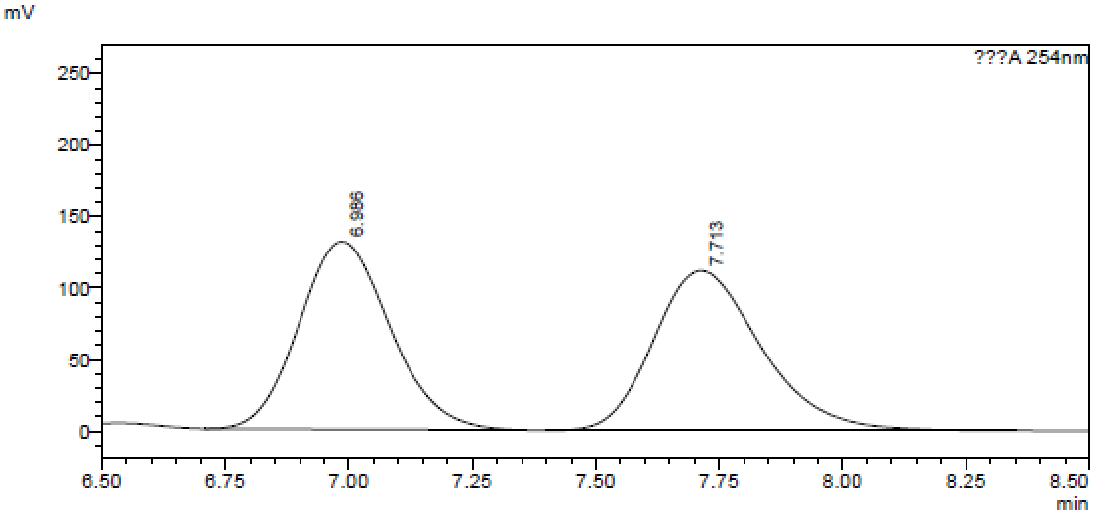

<Peak Table>

???A 254nm

Peak#	Ret. Time	Area	Height	Conc.	Unit	Mark	Name
1	6.986	1691007	131282	49.850		M	
2	7.713	1701204	111688	50.150		M	
Total		3392211	242970				

<Chromatogram>

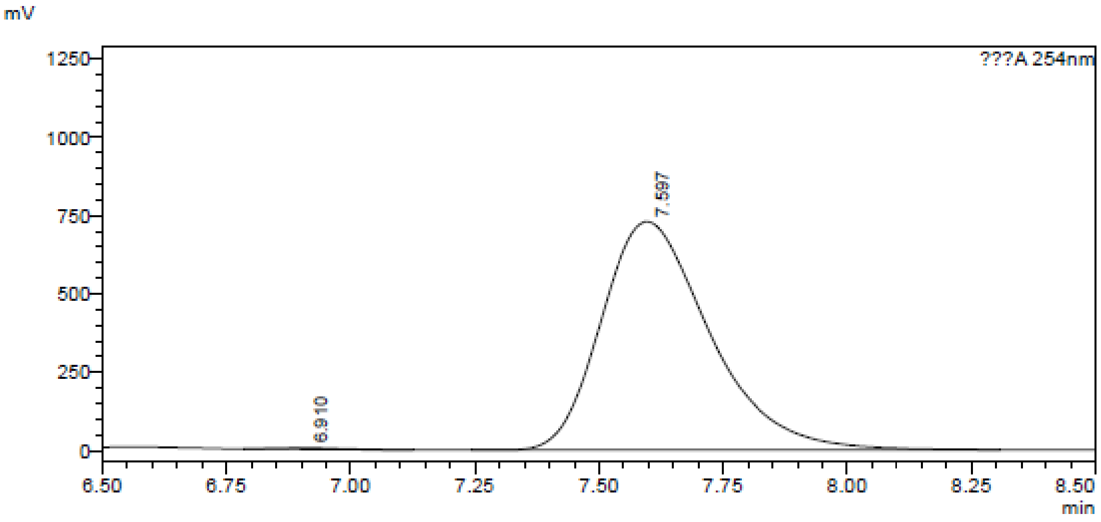

<Peak Table>

???A 254nm

Peak#	Ret. Time	Area	Height	Conc.	Unit	Mark	Name
1	6.910	31929	3258	0.287		M	
2	7.597	11106735	728983	99.713		M	
Total		11138664	732241				

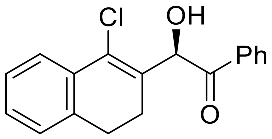

6c

<Chromatogram>

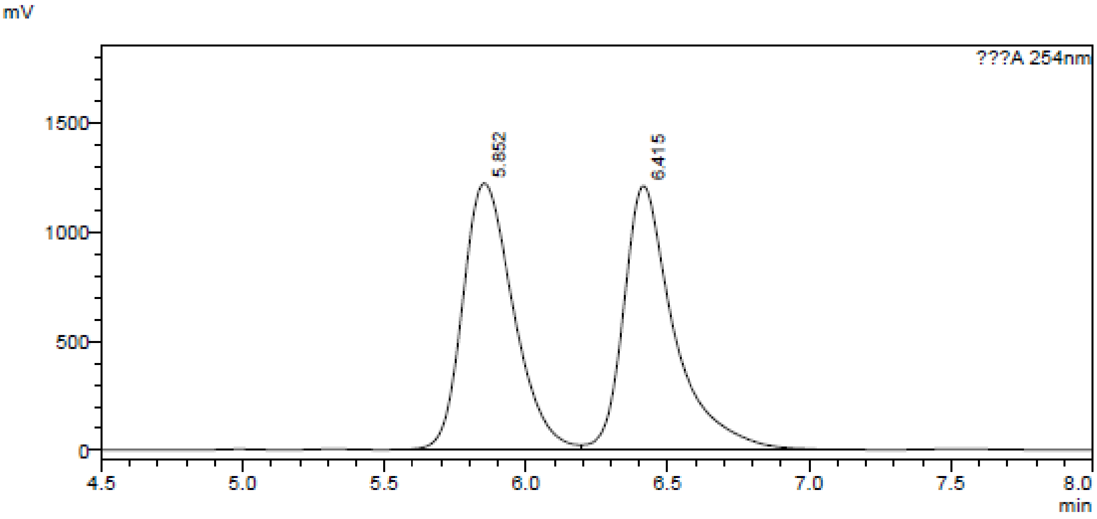

<Peak Table>

???A 254nm

Peak#	Ret. Time	Area	Height	Conc.	Unit	Mark	Name
1	5.852	15175010	1224501	50.408			
2	6.415	14929383	1211772	49.592		V	
Total		30104393	2436273				

<Chromatogram>

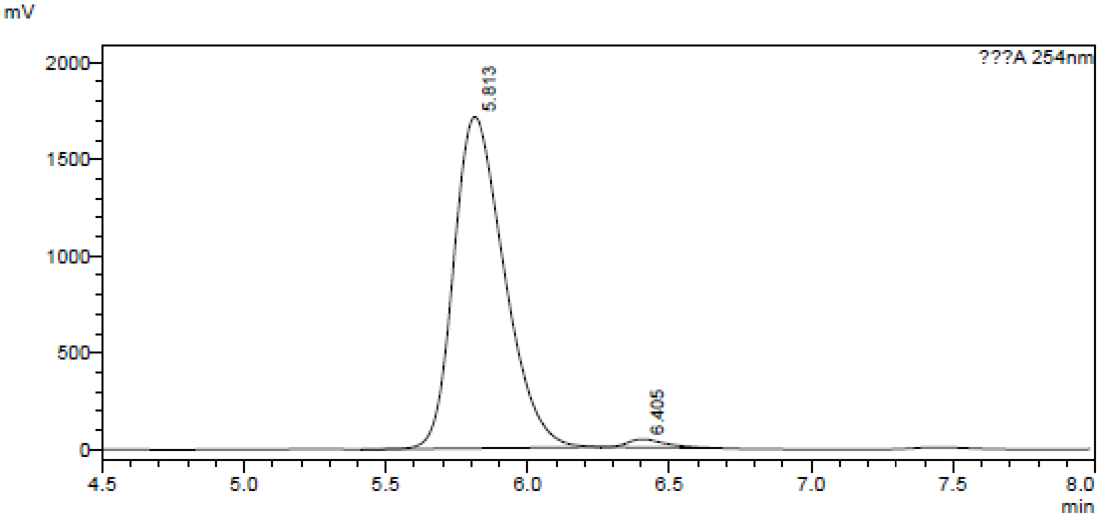

<Peak Table>

???A 254nm

Peak#	Ret. Time	Area	Height	Conc.	Unit	Mark	Name
1	5.813	21298331	1710321	98.184		M	
2	6.405	394017	42627	1.816		M	
Total		21692347	1752948				

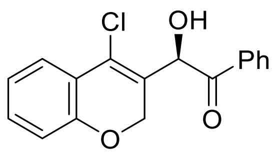

6d

<Chromatogram>

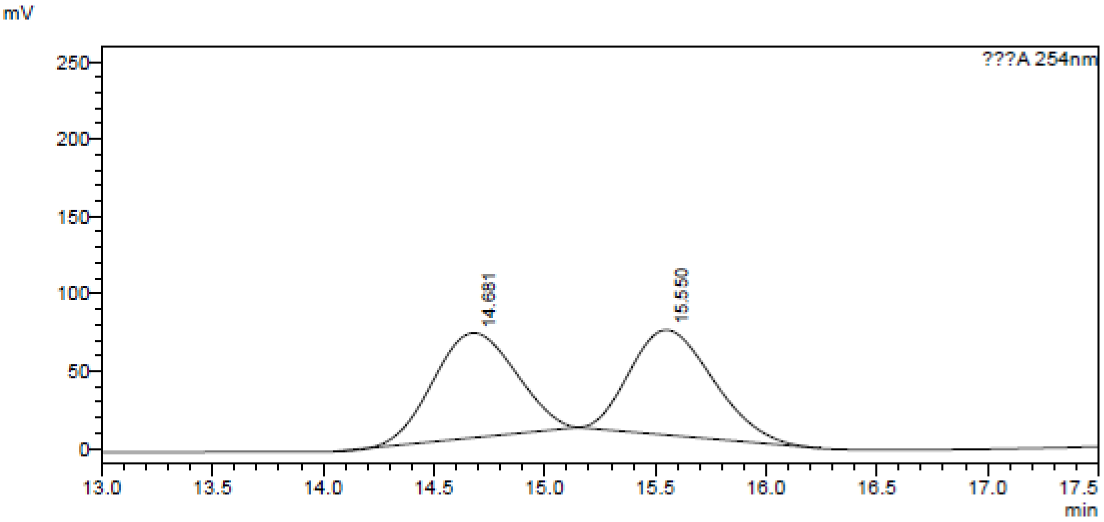

<Peak Table>

???A 254nm

Peak#	Ret. Time	Area	Height	Conc.	Unit	Mark	Name
1	14.681	1760988	67627	49.727		M	
2	15.550	1780317	67833	50.273			
Total		3541305	135460				

<Chromatogram>

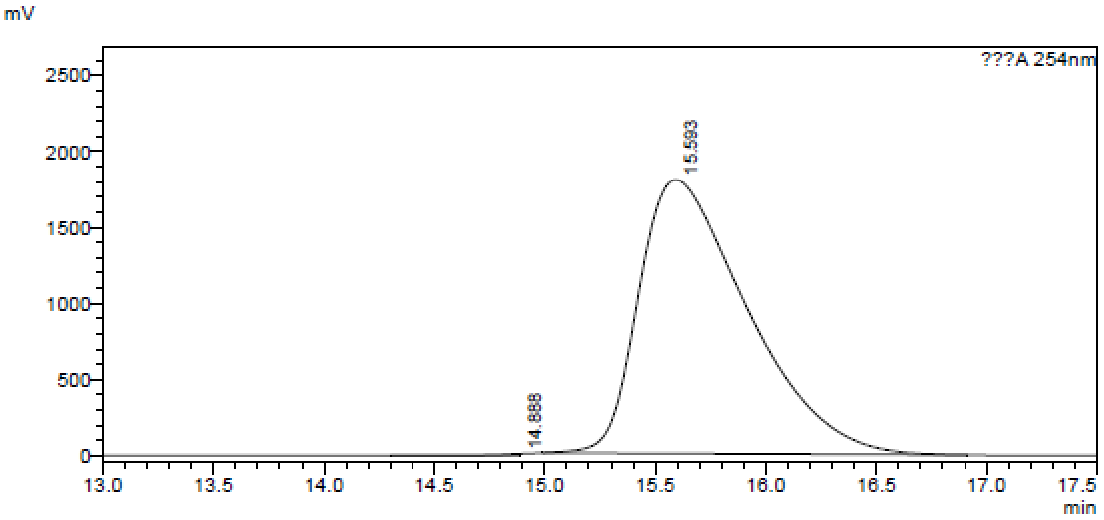

<Peak Table>

???A 254nm

Peak#	Ret. Time	Area	Height	Conc.	Unit	Mark	Name
1	14.888	56125	9350	0.091		M	
2	15.593	61524232	1799296	99.909		M	
Total		61580356	1808647				

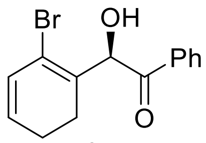

6e

<Chromatogram>

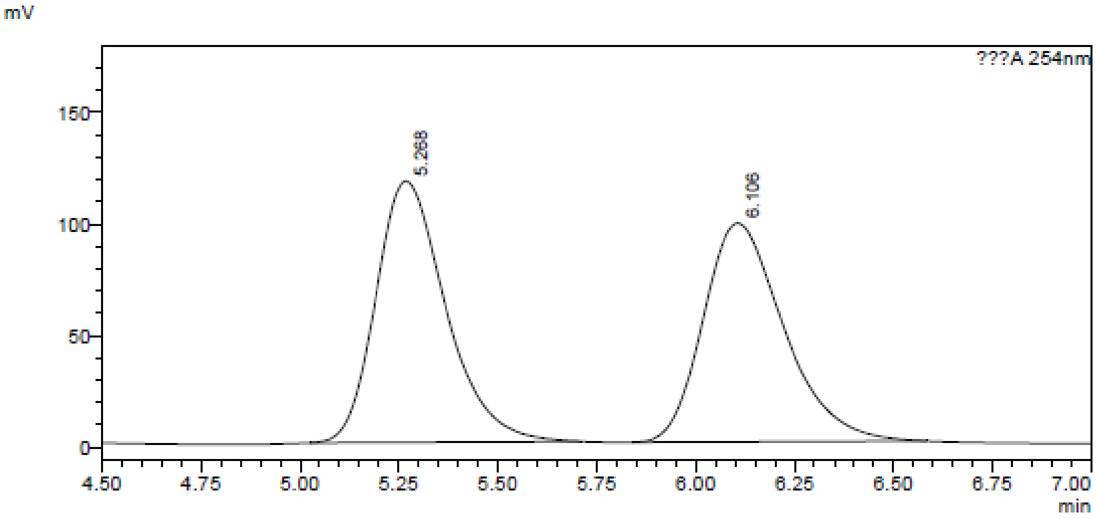

<Peak Table>

???A 254nm

Peak#	Ret. Time	Area	Height	Conc.	Unit	Mark	Name
1	5.268	1439888	117305	50.860			
2	6.106	1391199	98035	49.140			
Total		2831086	215340				

<Chromatogram>

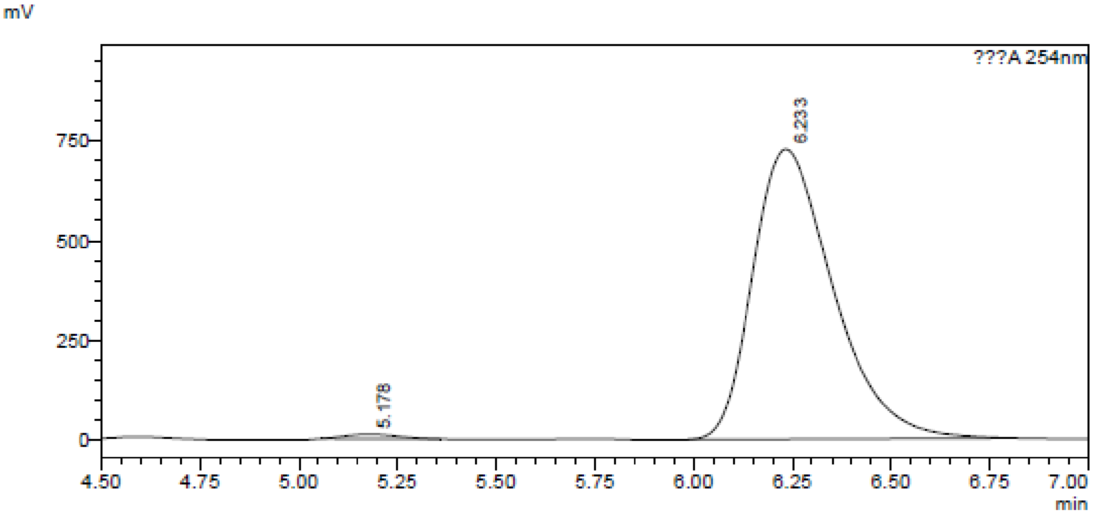

<Peak Table>

???A 254nm

Peak#	Ret. Time	Area	Height	Conc.	Unit	Mark	Name
1	5.178	105100	11247	1.010		M	
2	6.233	10301934	725831	98.990		M	
Total		10407033	737078				

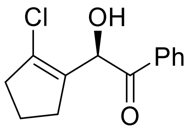

6f

<Chromatogram>

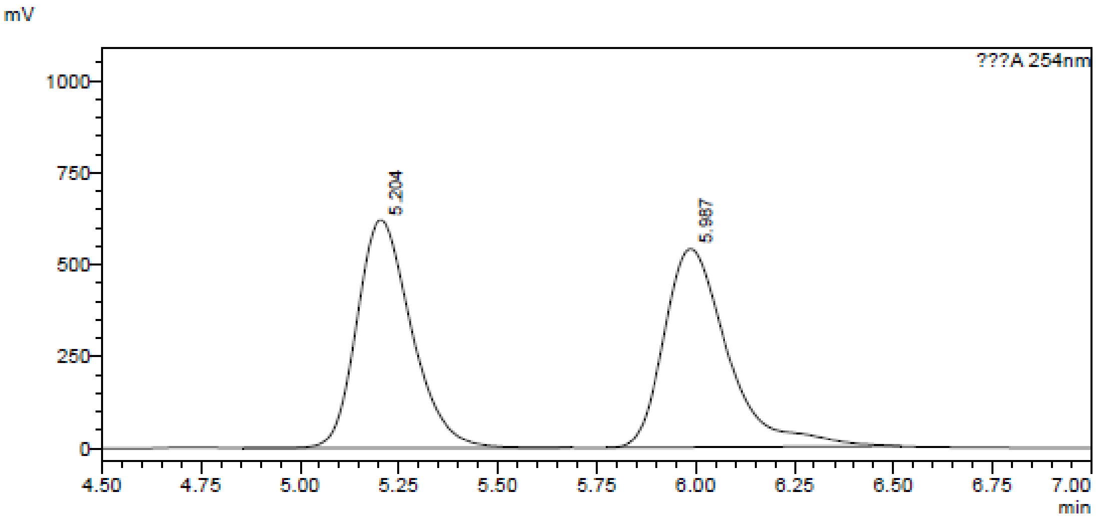

<Peak Table>

???A 254nm

Peak#	Ret. Time	Area	Height	Conc.	Unit	Mark	Name
1	5.204	5969267	620403	49.492		M	
2	5.987	6091864	539747	50.508		M	
Total		12061131	1160150				

<Chromatogram>

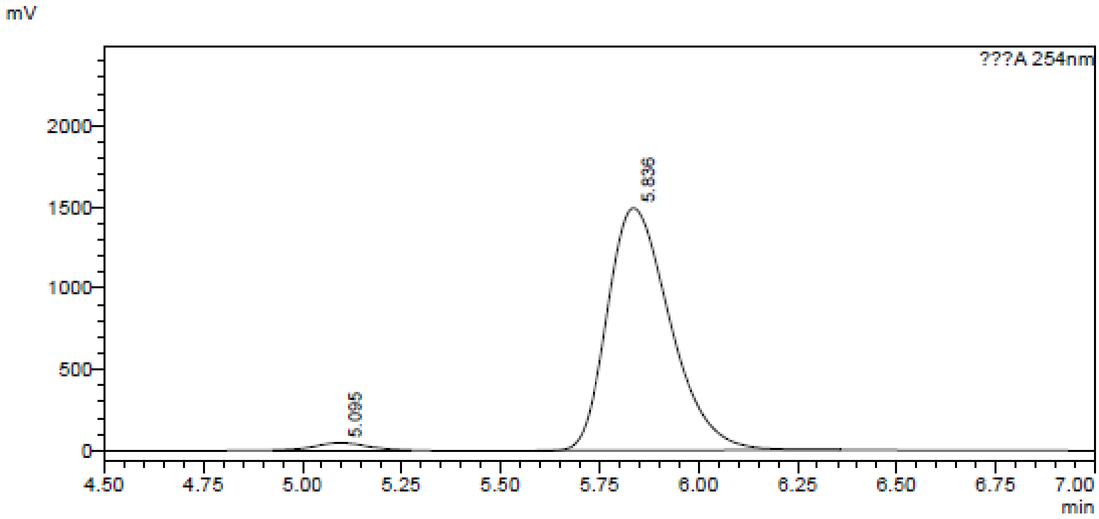

<Peak Table>

???A 254nm

Peak#	Ret. Time	Area	Height	Conc.	Unit	Mark	Name
1	5.095	406663	45247	2.415		M	
2	5.836	16434360	1490328	97.585		M	
Total		16841024	1535575				

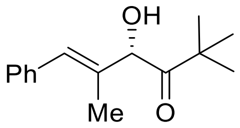

6g

<Chromatogram>

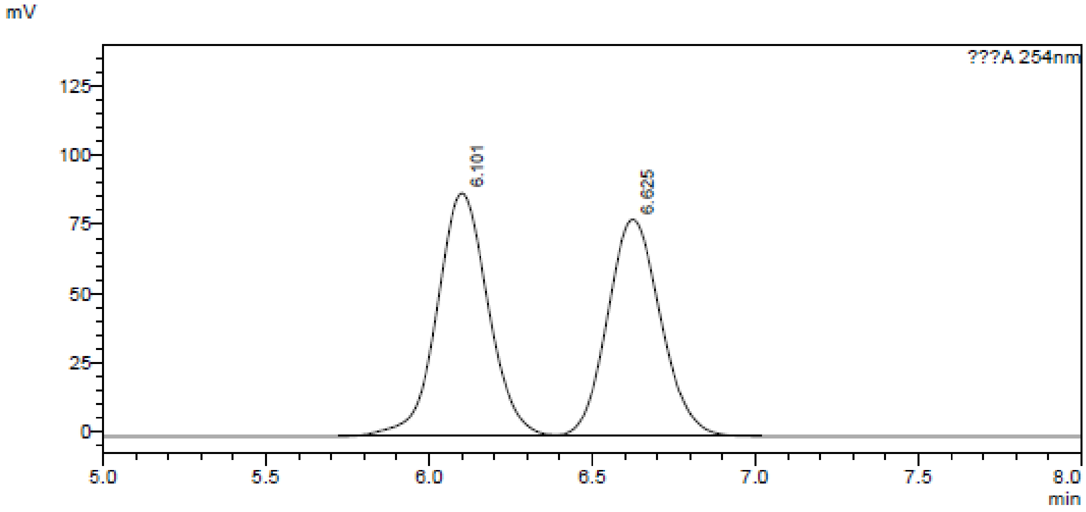

<Peak Table>

???A 254nm

Peak#	Ret. Time	Area	Height	Conc.	Unit	Mark	Name
1	6.101	962658	87973	51.915			
2	6.625	891629	78518	48.085		V	
Total		1854287	166490				

<Chromatogram>

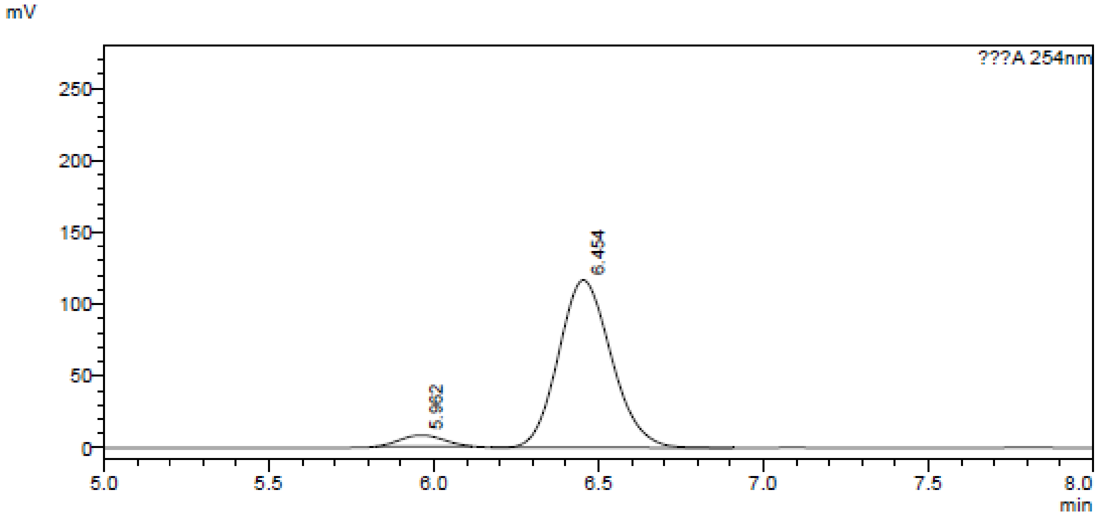

<Peak Table>

???A 254nm

Peak#	Ret. Time	Area	Height	Conc.	Unit	Mark	Name
1	5.962	70288	7756	5.114		M	
2	6.454	1304133	116418	94.886		M	
Total		1374421	124174				

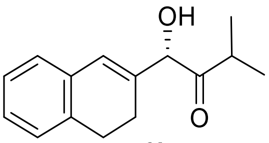

6h

<Chromatogram>

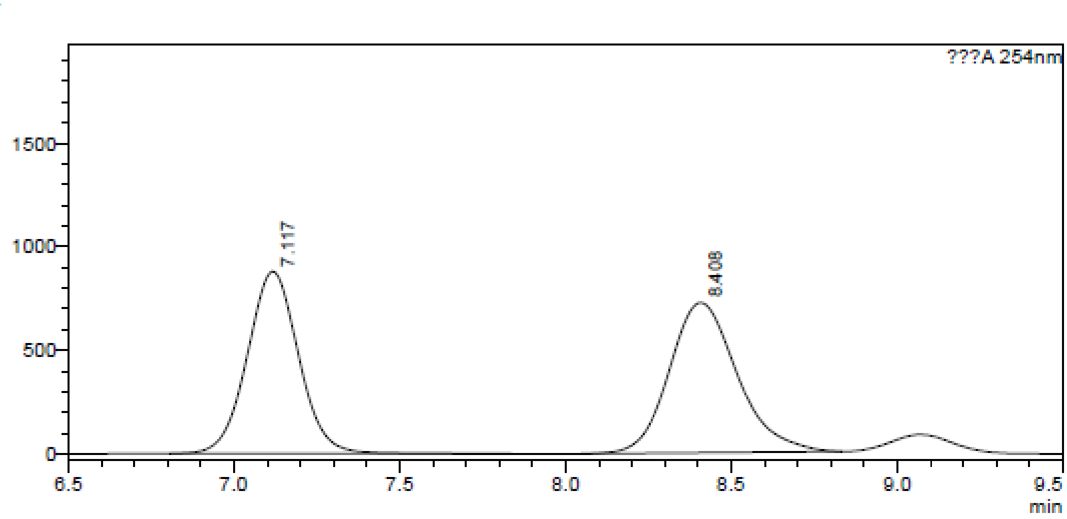

<Peak Table>

???A 254nm

Peak#	Ret. Time	Area	Height	Conc.	Unit	Mark	Name
1	7.117	9446746	878873	47.776			
2	8.408	10326084	723943	52.224			
Total		19772831	1602816				

<Chromatogram>

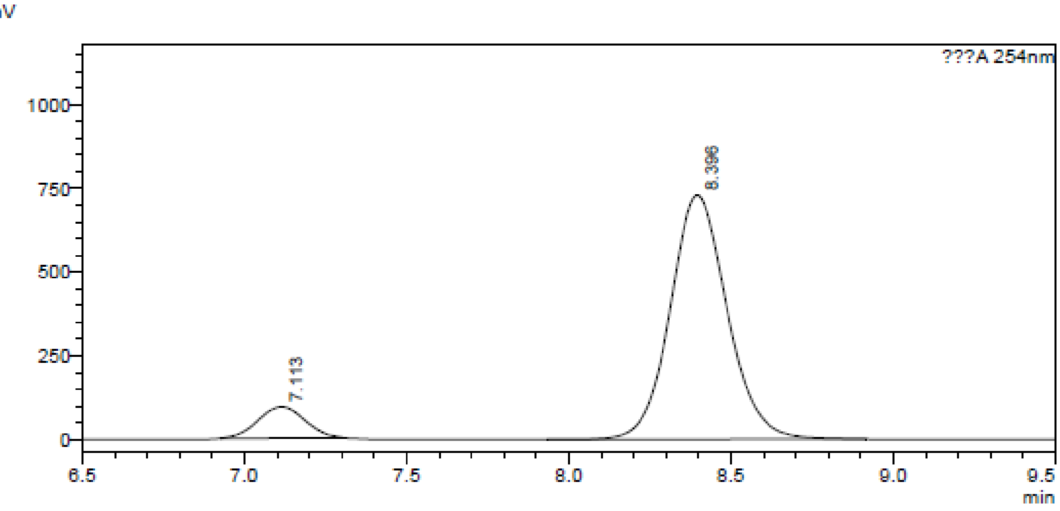

<Peak Table>

???A 254nm

Peak#	Ret. Time	Area	Height	Conc.	Unit	Mark	Name
1	7.113	949140	94258	9.611		M	
2	8.396	8926262	728127	90.389		M	
Total		9875402	822385				

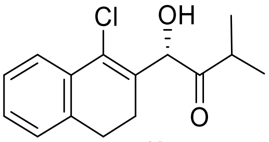

6i

<Chromatogram>

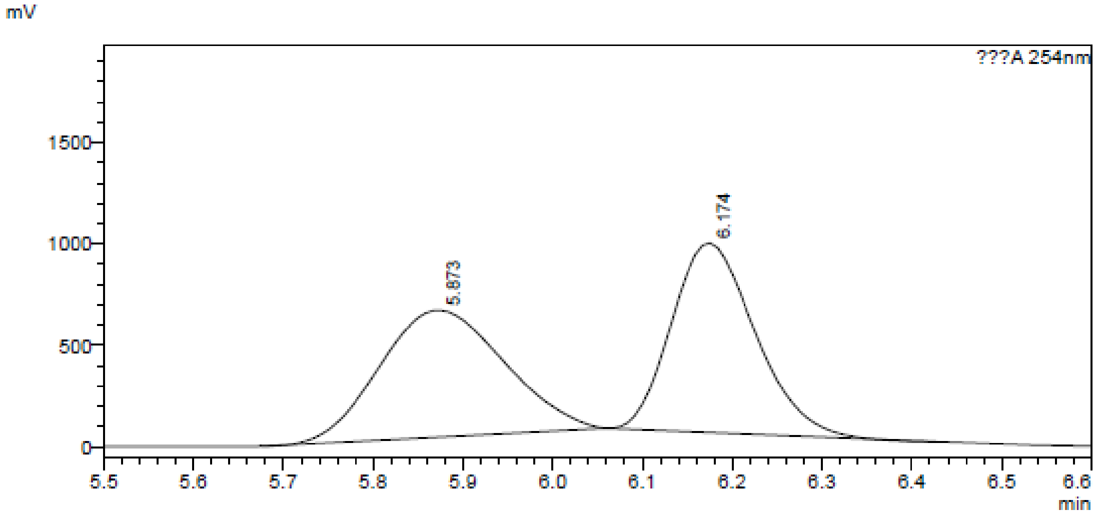

<Peak Table>

???A 254nm

Peak#	Ret. Time	Area	Height	Conc.	Unit	Mark	Name
1	5.873	6044307	624782	50.069		M	
2	6.174	6027691	933398	49.931		M	
Total		12071997	1558180				

<Chromatogram>

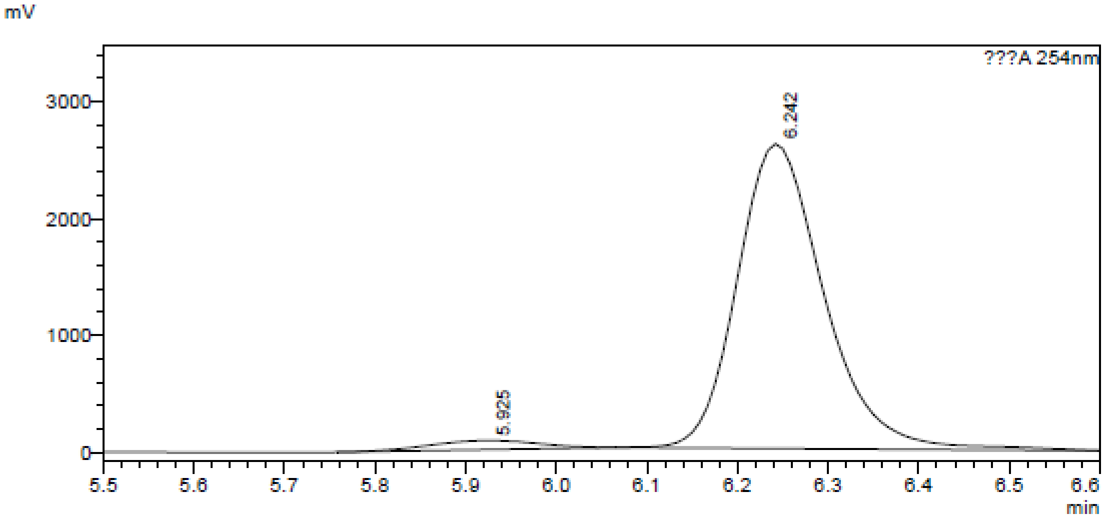

<Peak Table>

???A 254nm

Peak#	Ret. Time	Area	Height	Conc.	Unit	Mark	Name
1	5.925	654303	77519	3.628			
2	6.242	17381755	2605275	96.372		M	
Total		18036058	2682794				

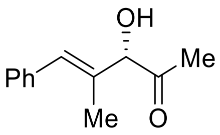

6j

<Chromatogram>

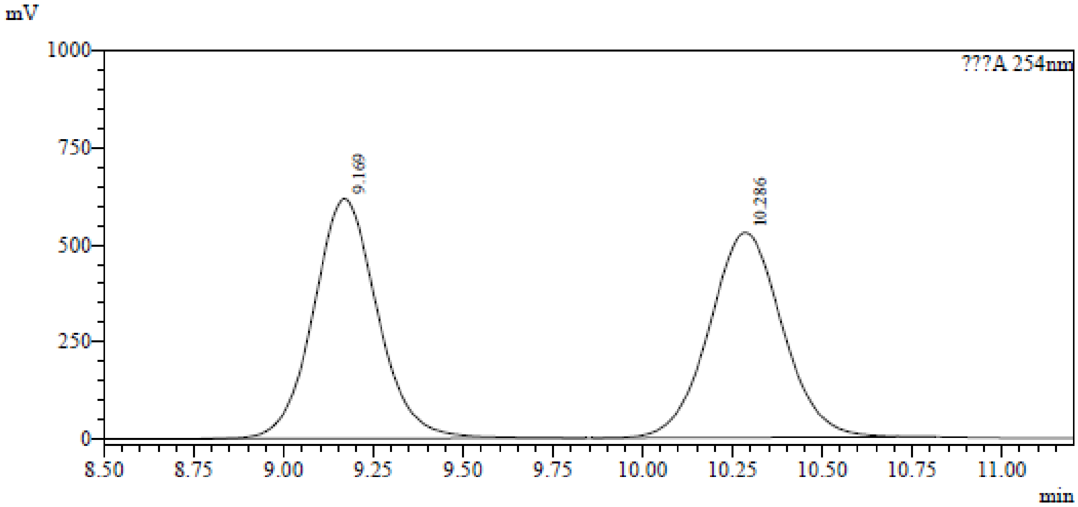

<Peak Table>

???A 254nm

Peak#	Ret. Time	Area	Height	Conc.	Unit	Mark	Name
1	9.169	7695369	618030	50.590			
2	10.286	7515804	528655	49.410		M	
Total		15211173	1146685				

<Chromatogram>

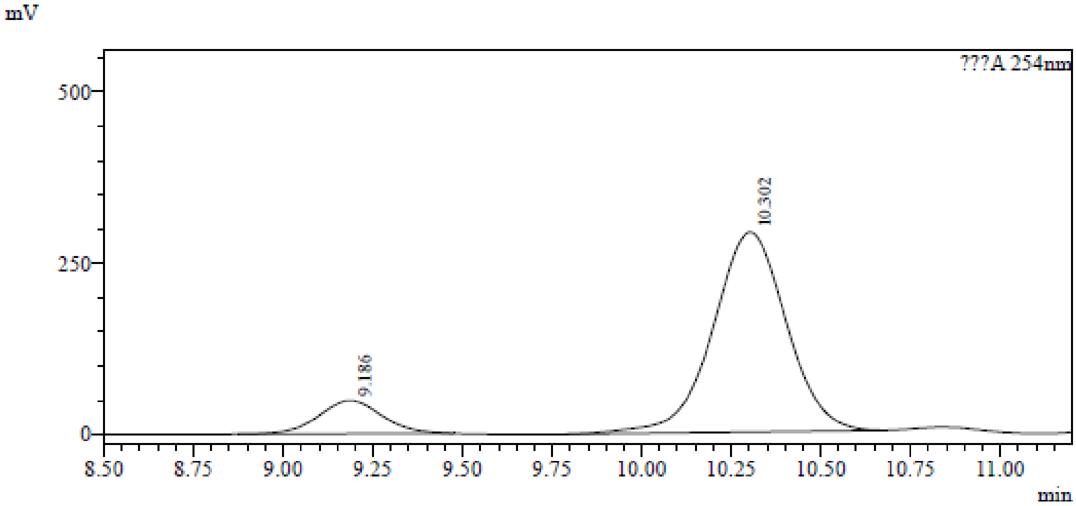

<Peak Table>

???A 254nm

Peak#	Ret. Time	Area	Height	Conc.	Unit	Mark	Name
1	9.186	595928	48264	12.544		M	
2	10.302	4154863	291148	87.456		M	
Total		4750791	339412				

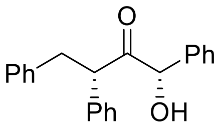

7a

<Chromatogram>

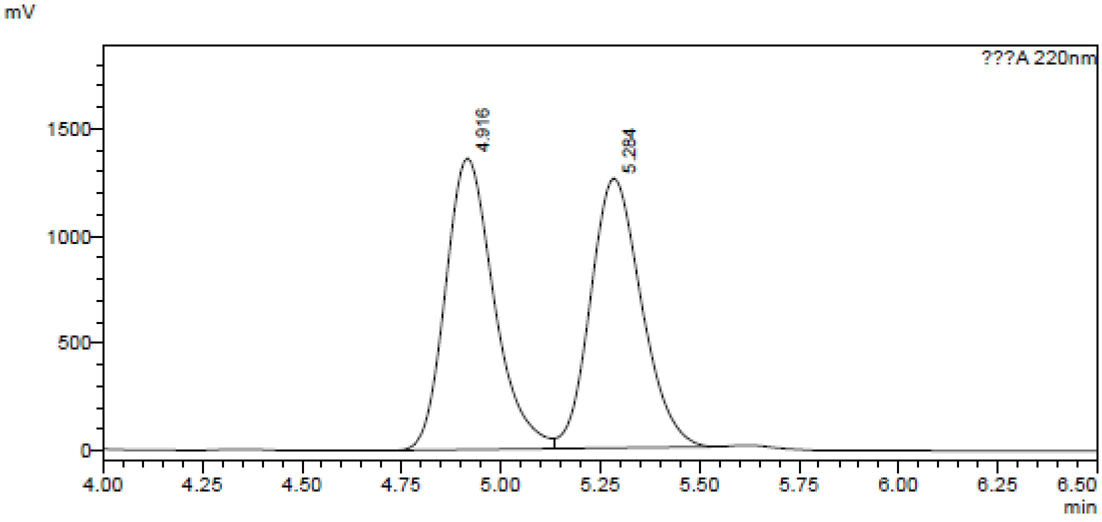

<Peak Table>

???A 220nm

Peak#	Ret. Time	Area	Height	Conc.	Unit	Mark	Name
1	4.916	11489332	1357033	50.674			
2	5.284	11183764	1255719	49.326		V	
Total		22673097	2612753				

<Chromatogram>

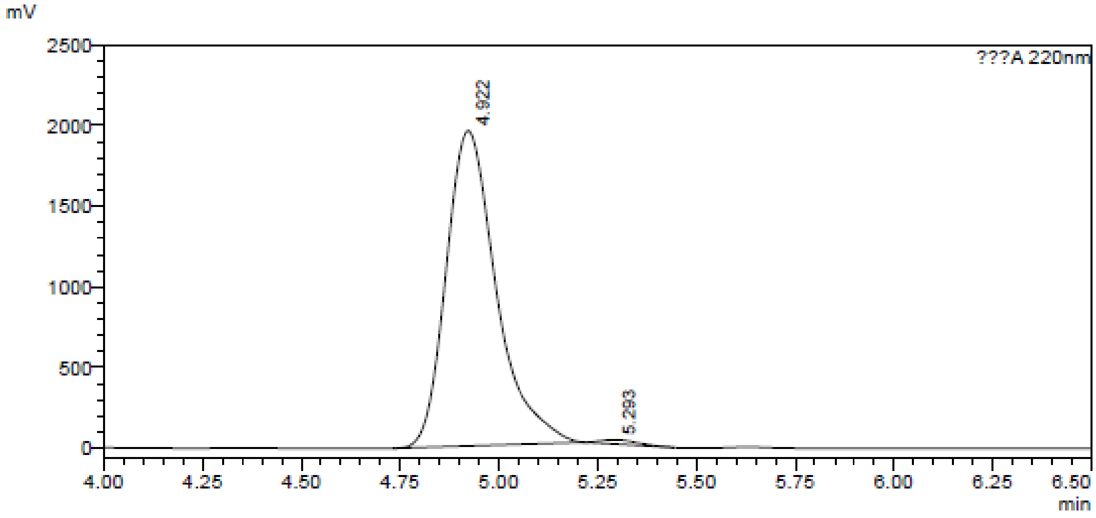

<Peak Table>

???A 220nm

Peak#	Ret. Time	Area	Height	Conc.	Unit	Mark	Name
1	4.922	17315245	1955216	99.078			
2	5.293	161128	26416	0.922		M	
Total		17476373	1981632				

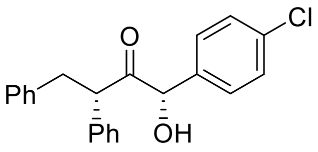

7b

<Chromatogram>

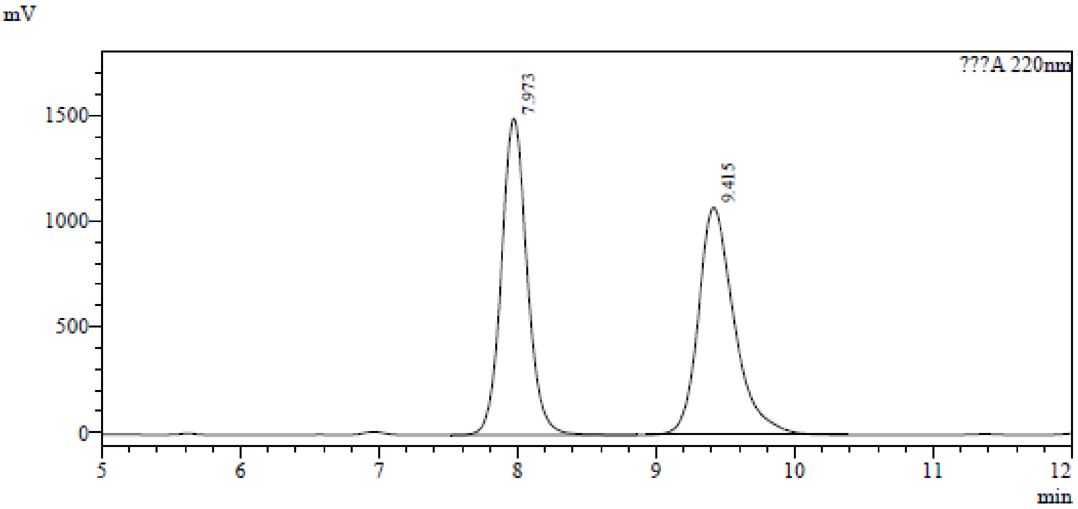

<Peak Table>

???A 220nm

Peak#	Ret. Time	Area	Height	Conc.	Unit	Mark	Name
1	7.973	19316973	1494265	49.858			
2	9.415	19426763	1074012	50.142		M	
Total		38743736	2568277				

<Chromatogram>

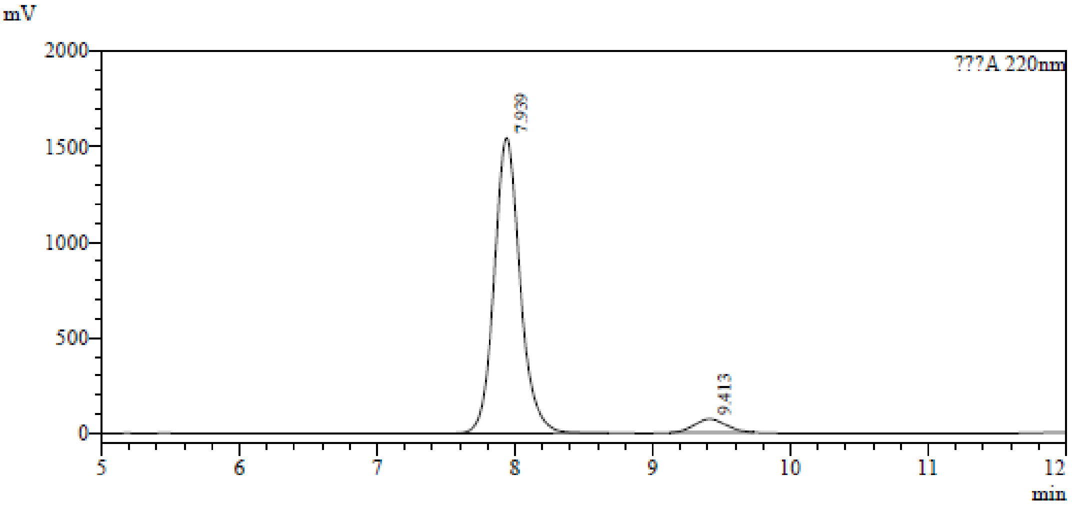

<Peak Table>

???A 220nm

Peak#	Ret. Time	Area	Height	Conc.	Unit	Mark	Name
1	7.939	20265702	1547995	94.768		M	
2	9.413	1118801	70786	5.232		M	
Total		21384503	1618781				

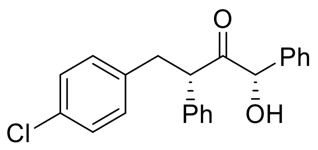

7c

<Chromatogram>

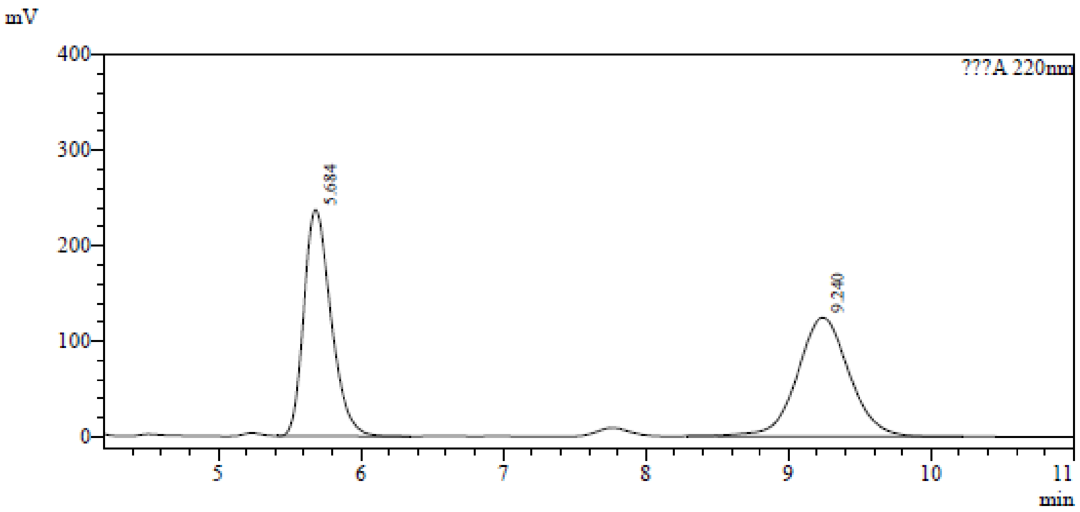

<Peak Table>

???A 220nm

Peak#	Ret. Time	Area	Height	Conc.	Unit	Mark	Name
1	5.684	3146175	236924	50.015			
2	9.240	3144260	124481	49.985		M	
Total		6290435	361405				

<Chromatogram>

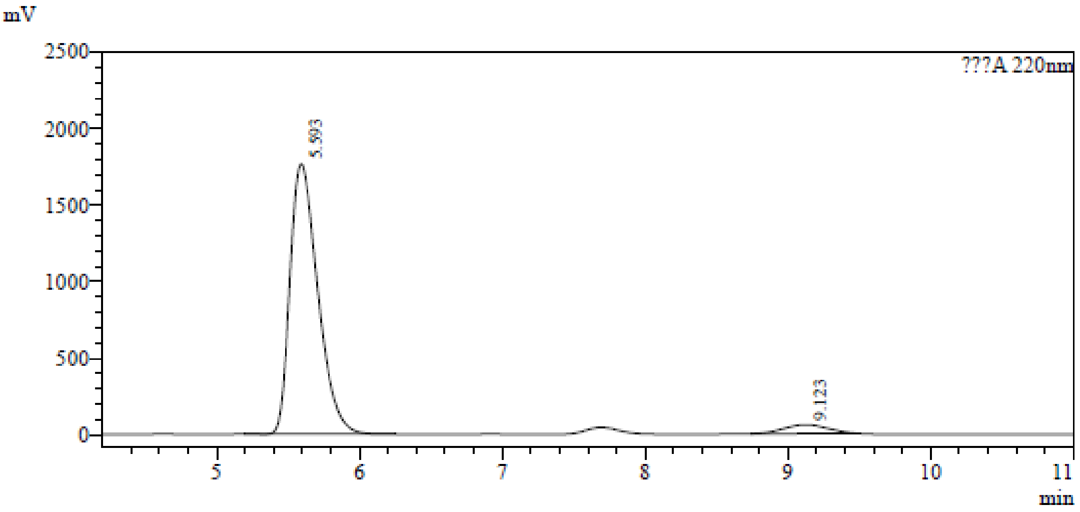

<Peak Table>

???A 220nm

Peak#	Ret. Time	Area	Height	Conc.	Unit	Mark	Name
1	5.593	23837443	1765134	95.069		M	
2	9.123	1236480	57690	4.931		M	
Total		25073922	1822824				

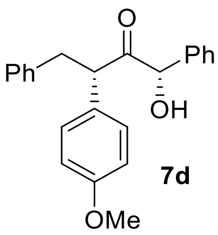

7d

<Chromatogram>

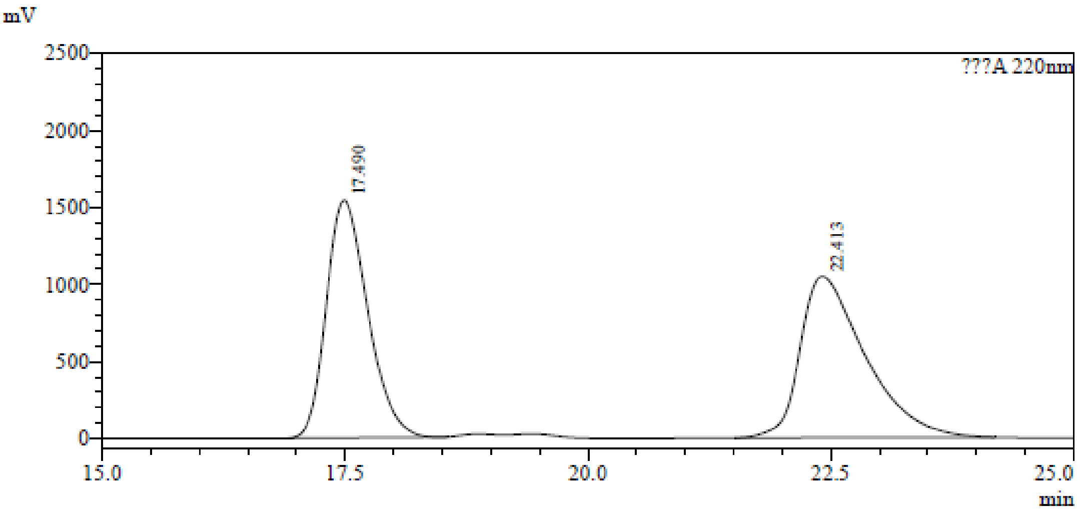

<Peak Table>

???A 220nm

Peak#	Ret. Time	Area	Height	Conc.	Unit	Mark	Name
1	17.490	45813837	1541022	48.348		M	
2	22.413	48944353	1047188	51.652		M	
Total		94758190	2588210				

<Chromatogram>

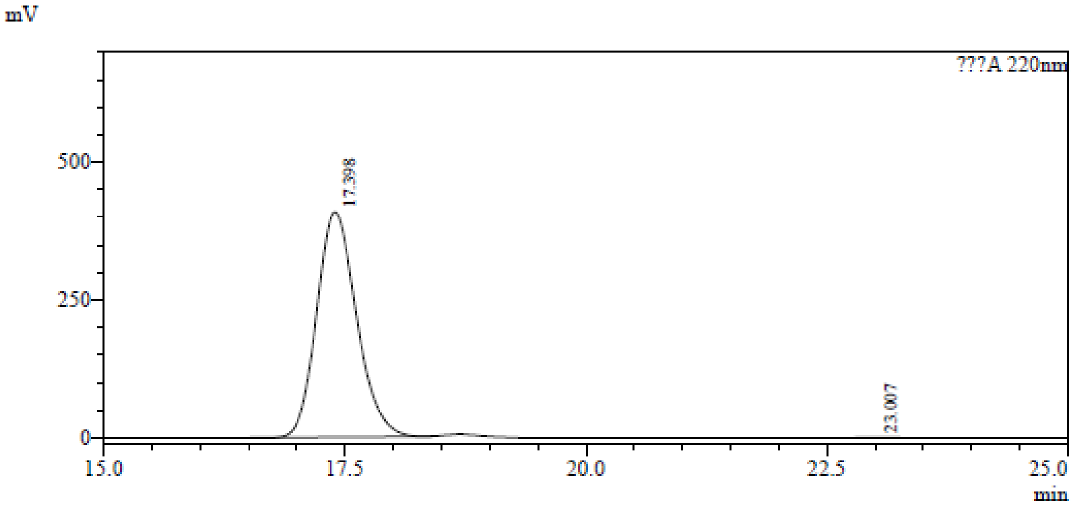

<Peak Table>

???A 220nm

Peak#	Ret. Time	Area	Height	Conc.	Unit	Mark	Name
1	17.398	11749426	408242	99.821		M	
2	23.007	21066	493	0.179		M	
Total		11770492	408736				

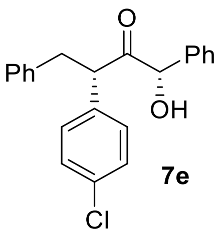

7e

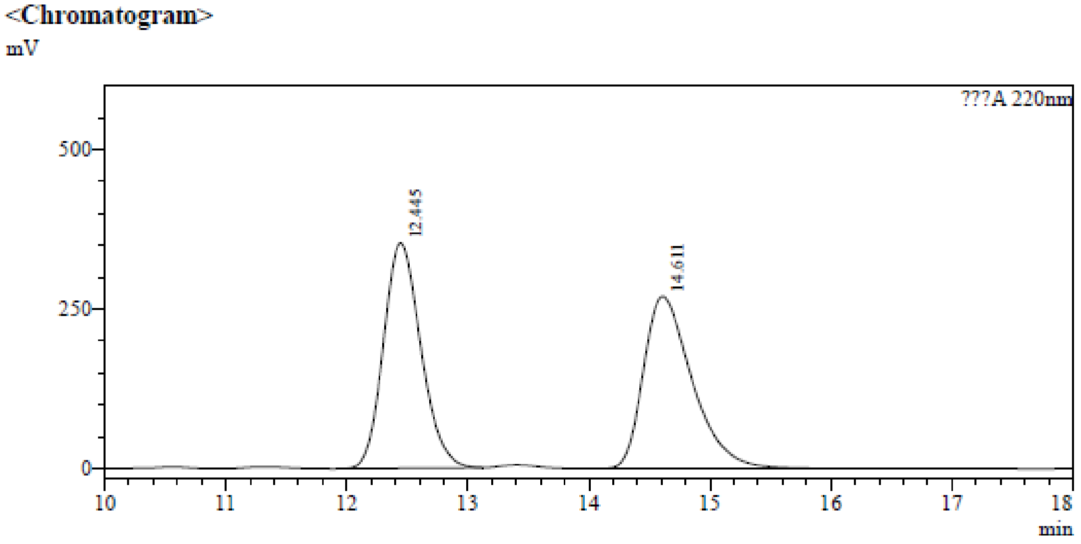

<Peak Table>

???A 220nm

Peak#	Ret. Time	Area	Height	Conc.	Unit	Mark	Name
1	12.445	7606794	353539	49.923			
2	14.611	7630335	270254	50.077			
Total		15237129	623793				

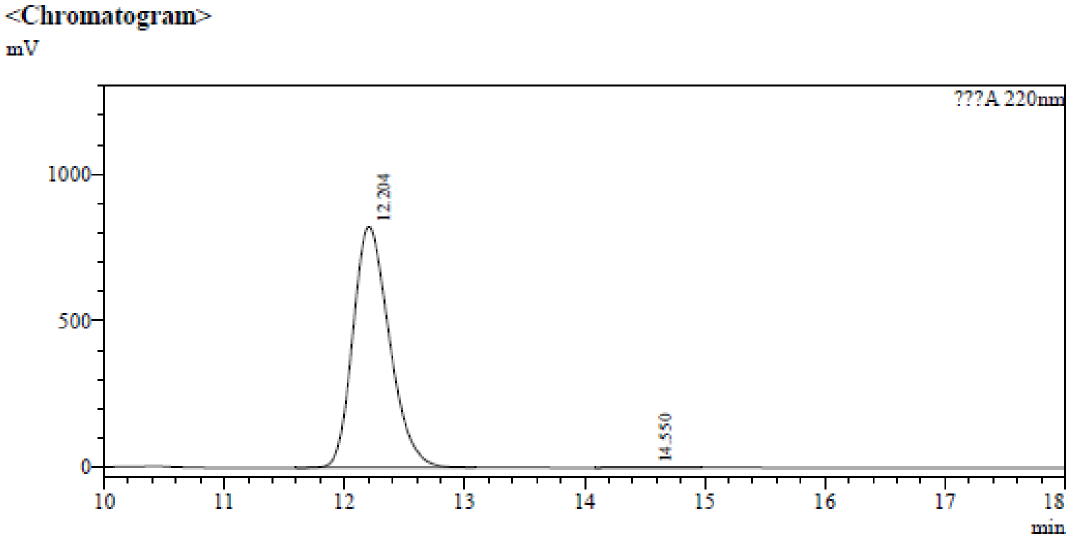

<Peak Table>

???A 220nm

Peak#	Ret. Time	Area	Height	Conc.	Unit	Mark	Name
1	12.204	17372392	820513	99.893		M	
2	14.550	18644	761	0.107			
Total		17391037	821274				

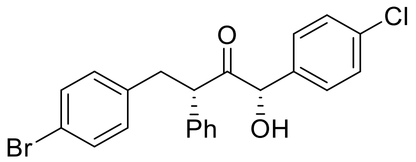

7f

<Chromatogram>

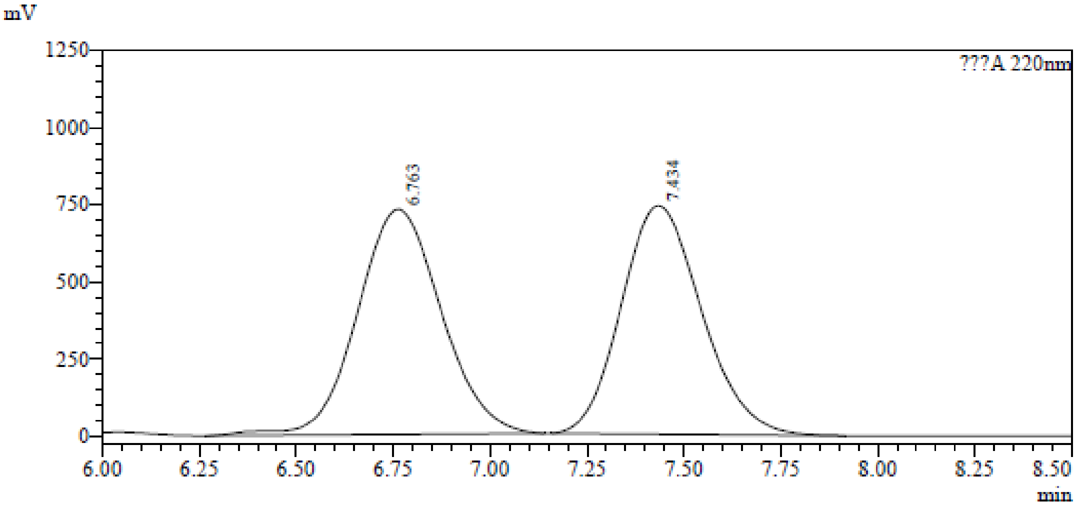

<Peak Table>

???A 220nm

Peak#	Ret. Time	Area	Height	Conc.	Unit	Mark	Name
1	6.763	11007315	729288	50.713			
2	7.434	10697832	740967	49.287		M	
Total		21705147	1470254				

<Chromatogram>

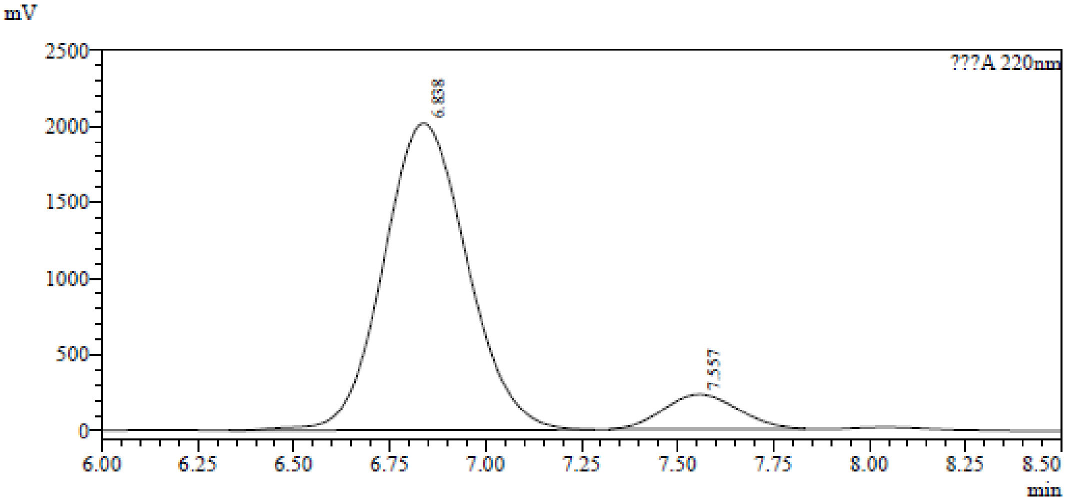

<Peak Table>

???A 220nm

Peak#	Ret. Time	Area	Height	Conc.	Unit	Mark	Name
1	6.838	30601662	2014444	90.900			
2	7.557	3063349	226514	9.100		M	
Total		33665012	2240958				

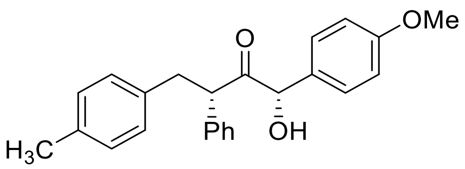

7g

<Chromatogram>

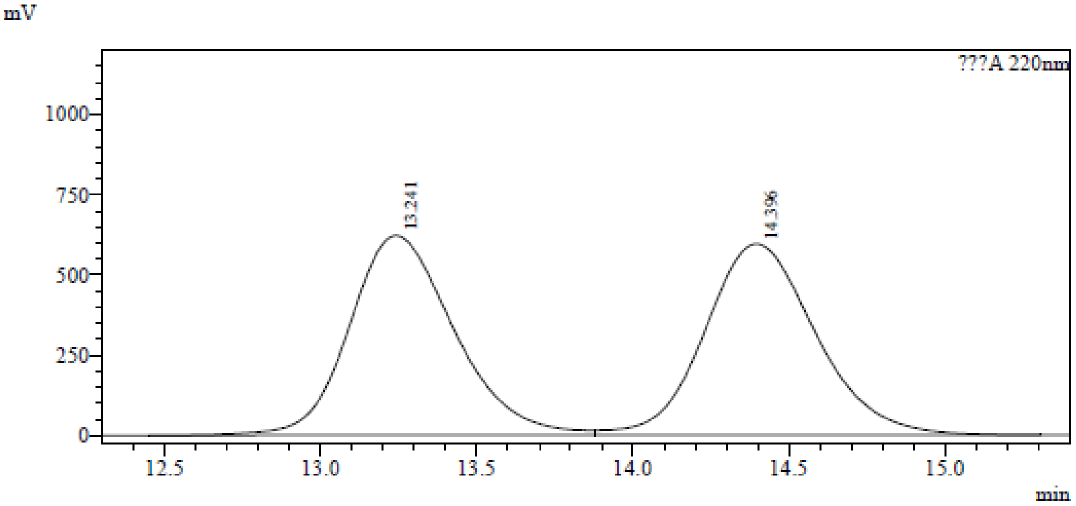

<Peak Table>

???A 220nm

Peak#	Ret. Time	Area	Height	Conc.	Unit	Mark	Name
1	13.241	14557573	622023	49.669			
2	14.396	14751545	596205	50.331		V	
Total		29309118	1218228				

<Chromatogram>

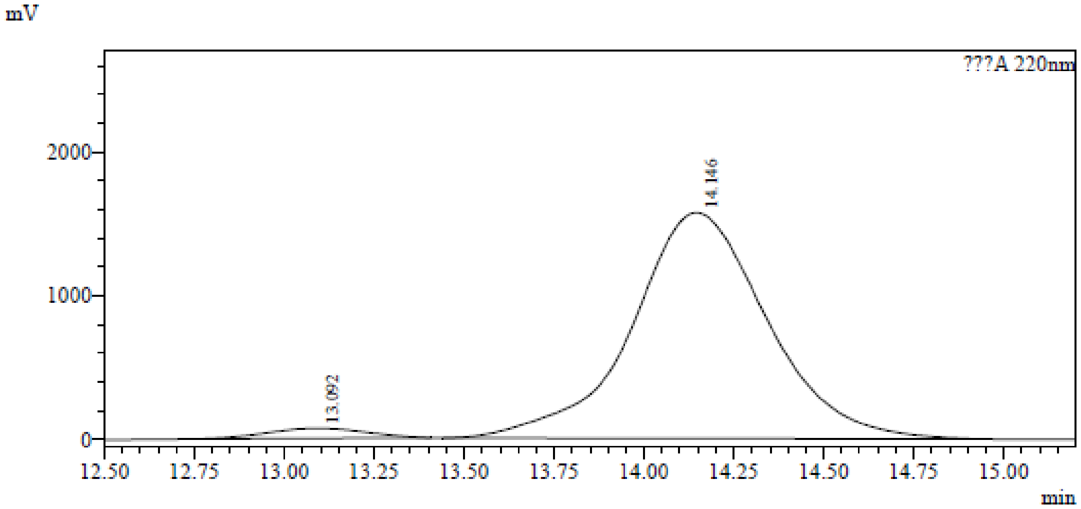

<Peak Table>

???A 220nm

Peak#	Ret. Time	Area	Height	Conc.	Unit	Mark	Name
1	13.092	1321836	68654	3.115		M	
2	14.146	41109566	1566358	96.885		M	
Total		42431402	1635012				

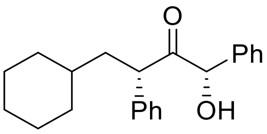

7h

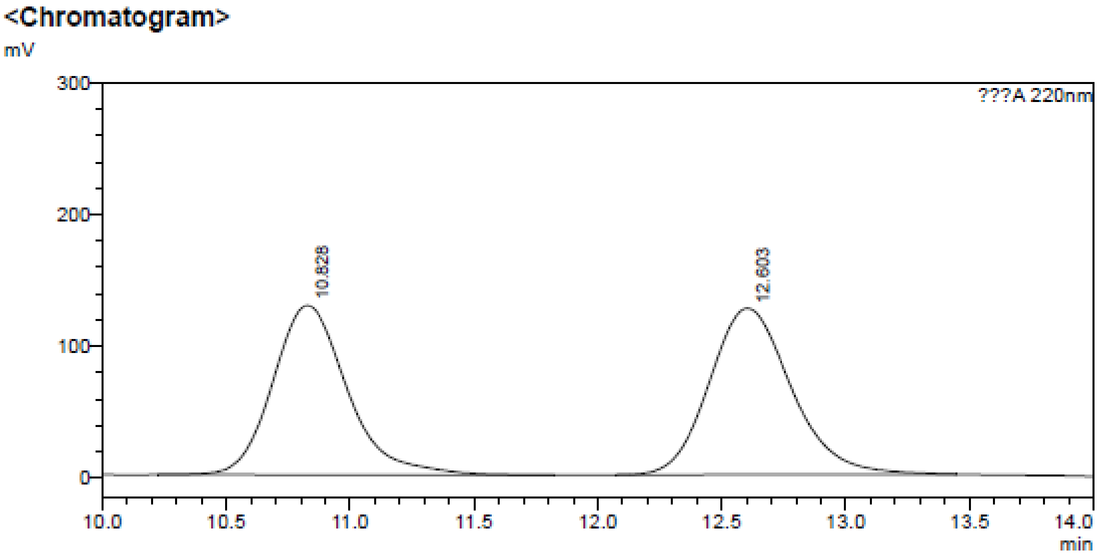

<Peak Table>

???A 220nm

Peak#	Ret. Time	Area	Height	Conc.	Unit	Mark	Name
1	10.828	2688381	128467	48.076		M	
2	12.603	2903526	126034	51.924		M	
Total		5591907	254502				

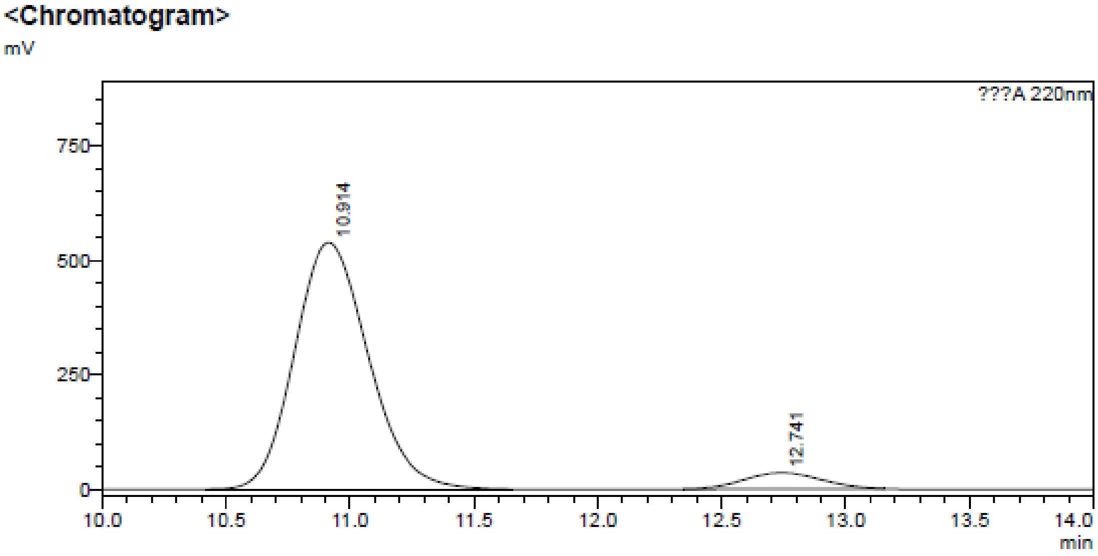

<Peak Table>

???A 220nm

Peak#	Ret. Time	Area	Height	Conc.	Unit	Mark	Name
1	10.914	11174780	538177	93.569		M	
2	12.741	768015	35711	6.431		M	
Total		11942796	573887				

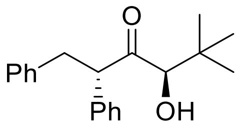

7i

<Chromatogram>

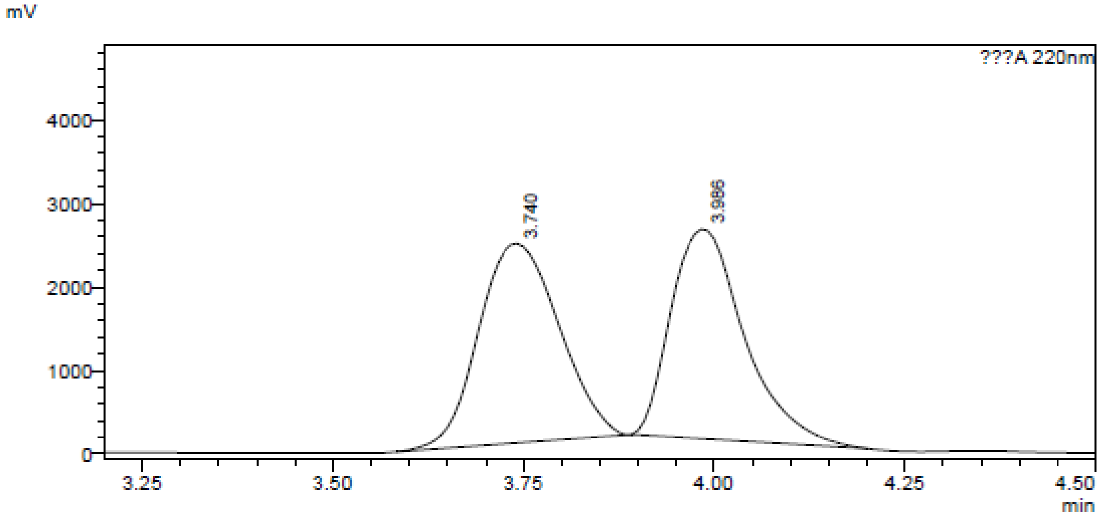

<Peak Table>

???A 220nm

Peak#	Ret. Time	Area	Height	Conc.	Unit	Mark	Name
1	3.740	17623326	2385946	51.122		M	
2	3.986	16849852	2503900	48.878		M	
Total		34473177	4889846				

<Chromatogram>

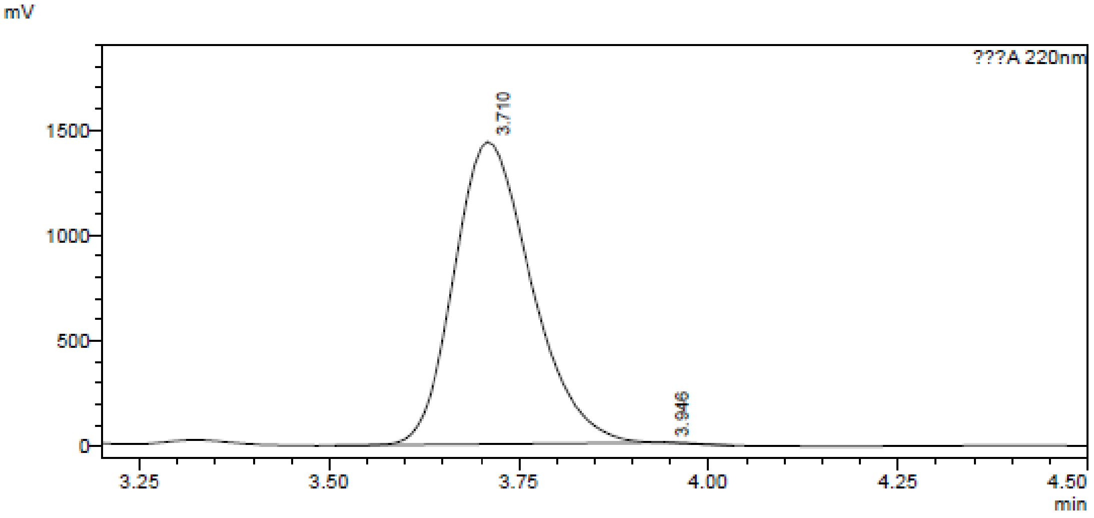

<Peak Table>

???A 220nm

Peak#	Ret. Time	Area	Height	Conc.	Unit	Mark	Name
1	3.710	10188129	1430809	99.978		M	
2	3.946	2285	839	0.022		M	
Total		10190414	1431648				

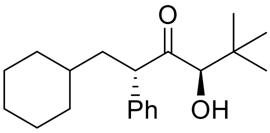

7j

<Chromatogram>

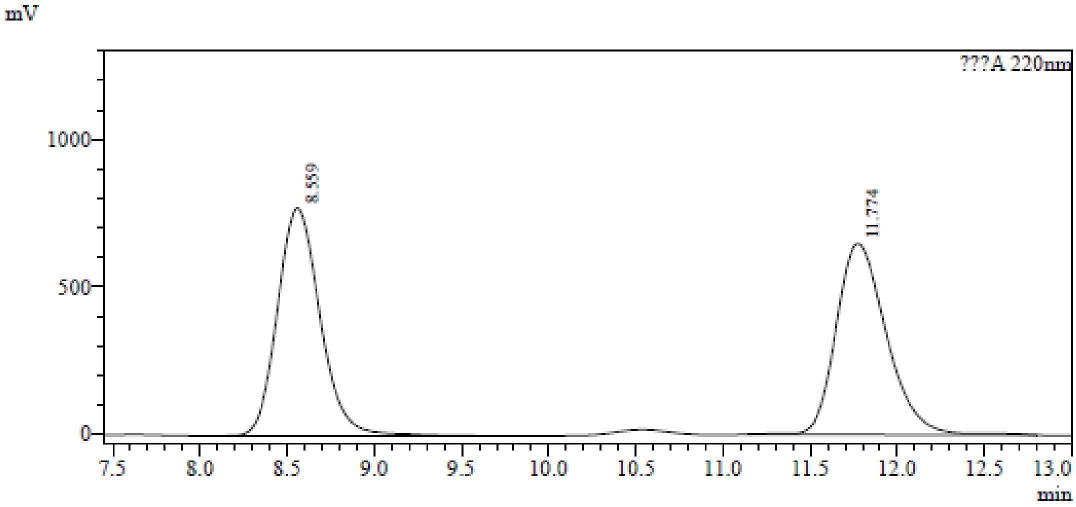

<Peak Table>

???A 220nm

Peak#	Ret. Time	Area	Height	Conc.	Unit	Mark	Name
1	8.559	13178572	771351	50.343			
2	11.774	12998977	647532	49.657		M	
Total		26177549	1418883				

<Chromatogram>

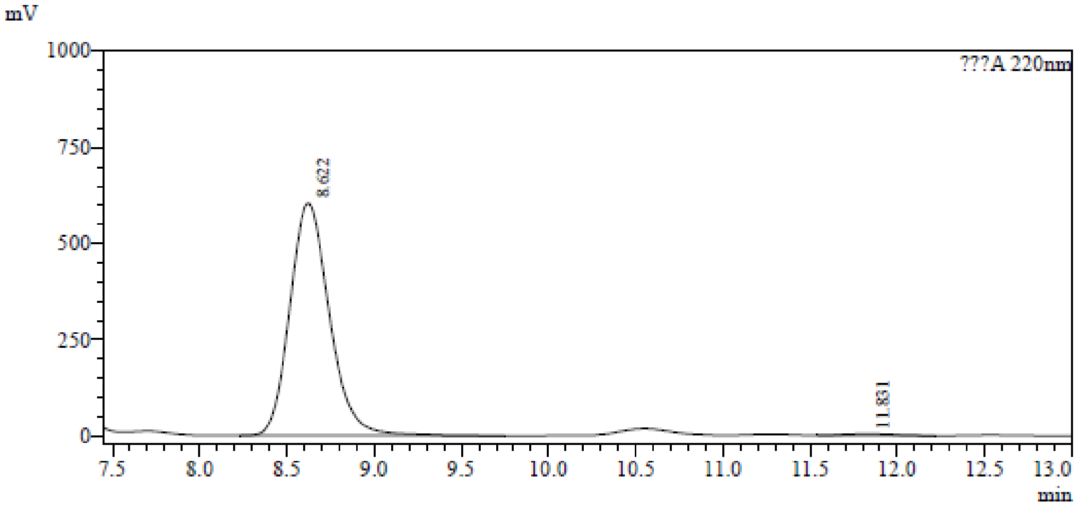

<Peak Table>

???A 220nm

Peak#	Ret. Time	Area	Height	Conc.	Unit	Mark	Name
1	8.622	9551437	603273	99.289			
2	11.831	68399	3906	0.711		M	
Total		9619836	607179				

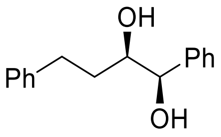

3a

<Chromatogram>

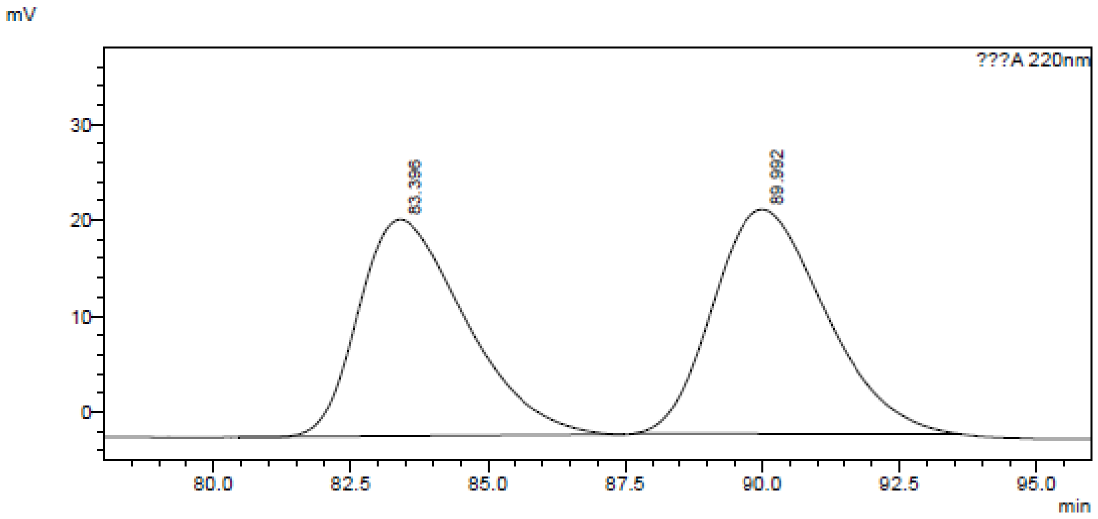

<Peak Table>

???A 220nm

Peak#	Ret. Time	Area	Height	Conc.	Unit	Mark	Name
1	83.396	3055078	22527	47.860		M	
2	89.992	3328263	23345	52.140		M	
Total		6383342	45872				

<Chromatogram>

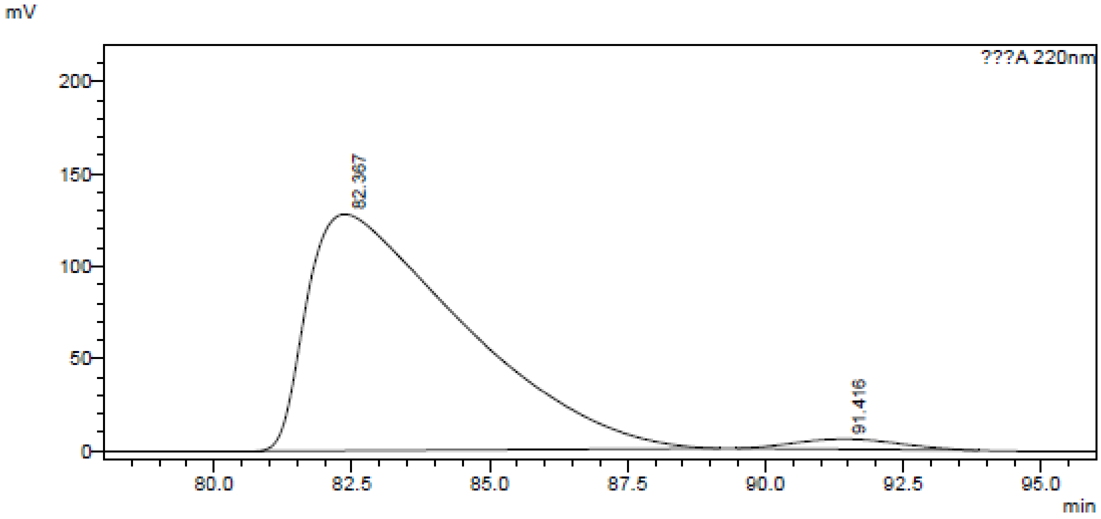

<Peak Table>

???A 220nm

Peak#	Ret. Time	Area	Height	Conc.	Unit	Mark	Name
1	82.367	25260696	128202	97.202		M	
2	91.416	727128	5513	2.798		M	
Total		25987824	133716				

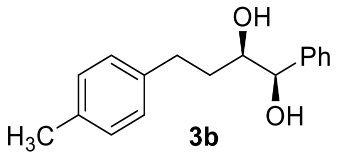

3b

<Chromatogram>

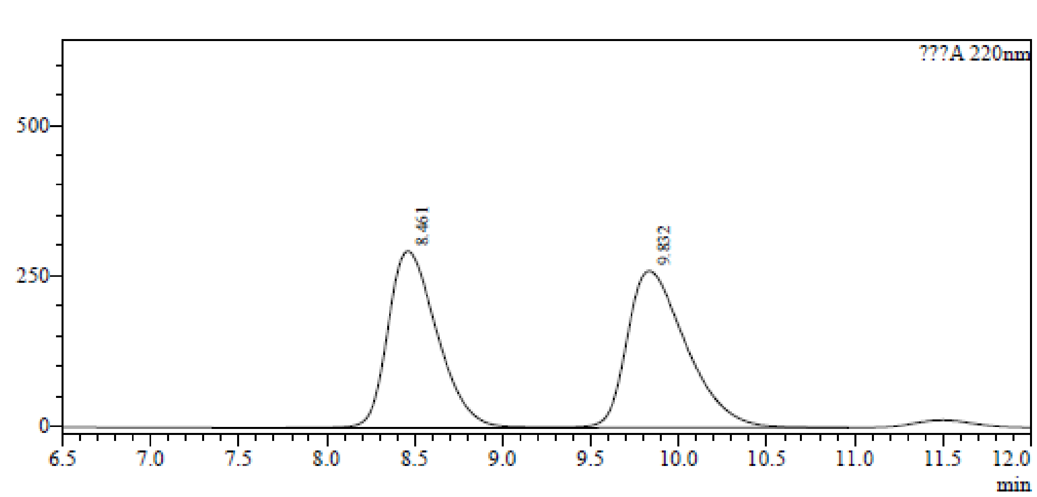

<Peak Table>

???A 220nm

Peak#	Ret. Time	Area	Height	Conc.	Unit	Mark	Name
1	8.461	5712937	293339	48.358			
2	9.832	6100942	260514	51.642		V	
Total		11813879	553854				

<Chromatogram>

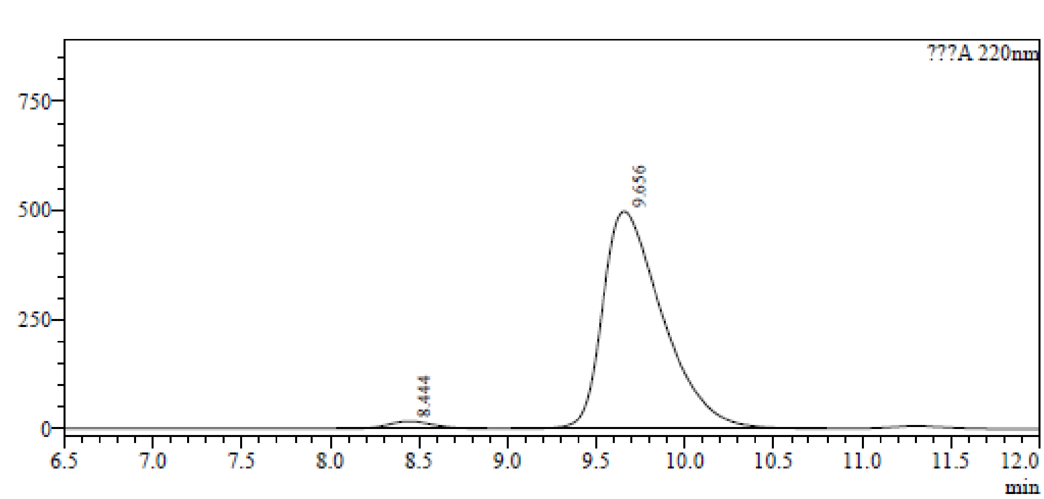

<Peak Table>

???A 220nm

Peak#	Ret. Time	Area	Height	Conc.	Unit	Mark	Name
1	8.444	288515	16829	2.404		M	
2	9.656	11715281	497694	97.596		M	
Total		12003796	514523				

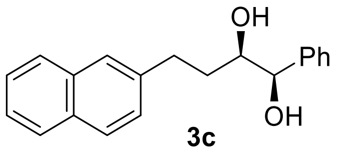

3c

<Chromatogram>

mV

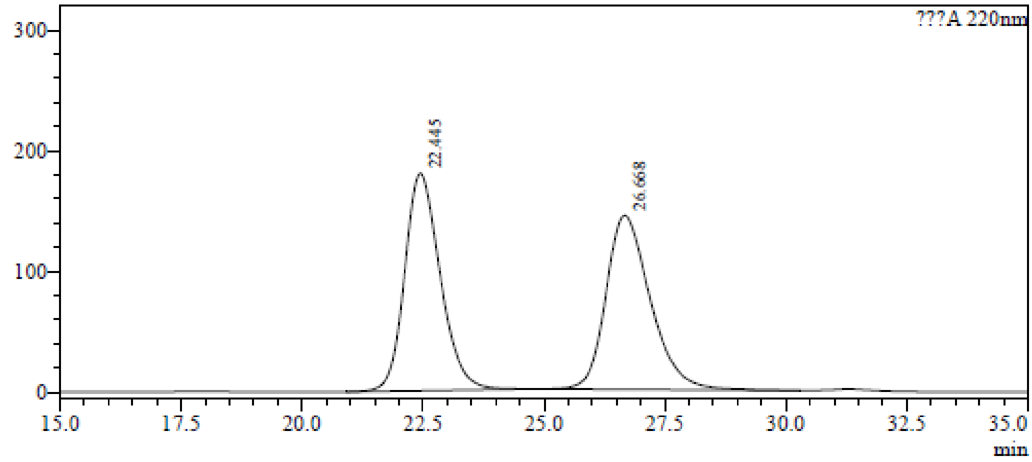

<Peak Table>

???A 220nm

Peak#	Ret. Time	Area	Height	Conc.	Unit	Mark	Name
1	22.445	9218152	180036	50.018			
2	26.668	9211628	144060	49.982			
Total		18429780	324096				

<Chromatogram>

mV

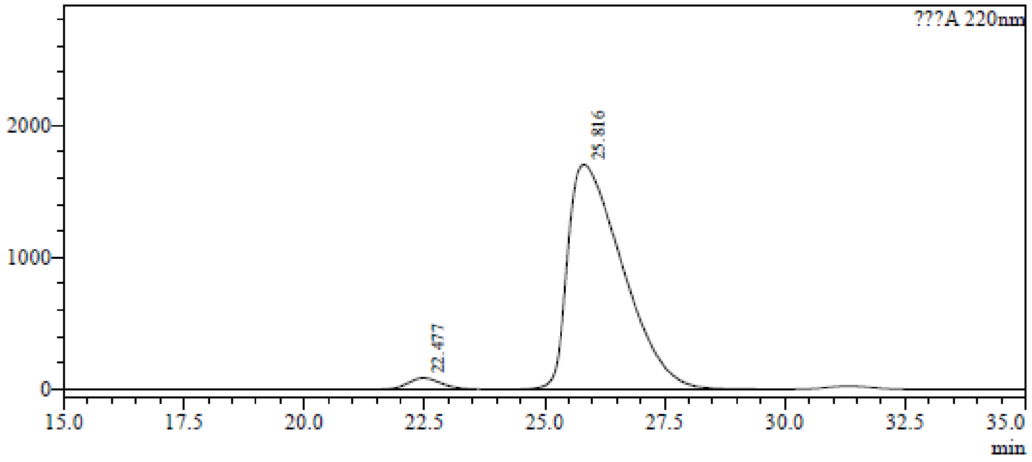

<Peak Table>

???A 220nm

Peak#	Ret. Time	Area	Height	Conc.	Unit	Mark	Name
1	22.477	4062588	85216	2.910		M	
2	25.816	135563822	1700519	97.090			
Total		139626410	1785735				

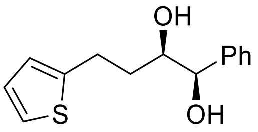

3d

<Chromatogram>

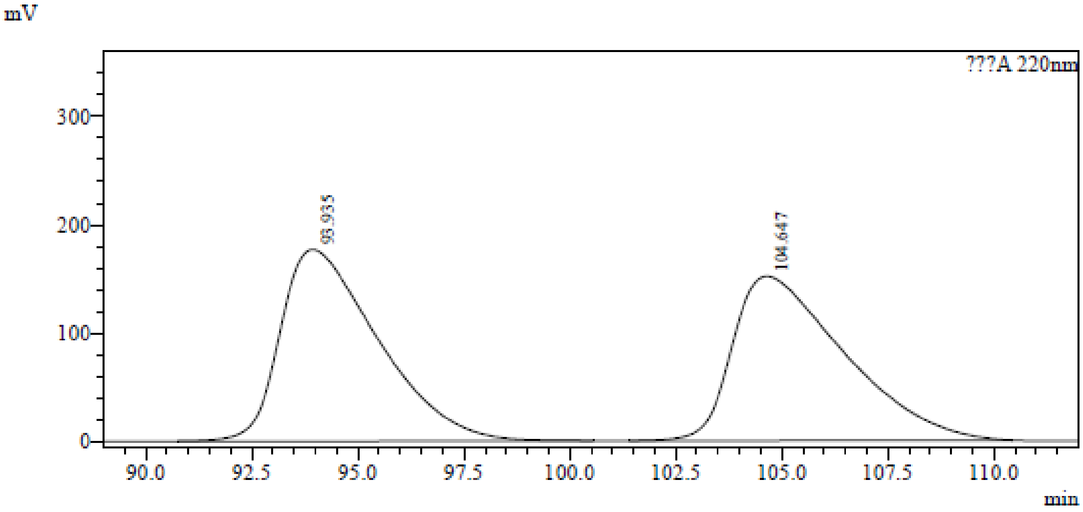

<Peak Table>

???A 220nm

Peak#	Ret. Time	Area	Height	Conc.	Unit	Mark	Name
1	93.935	28332323	176546	50.501			
2	104.647	27769790	151497	49.499		M	
Total		56102113	328043				

<Chromatogram>

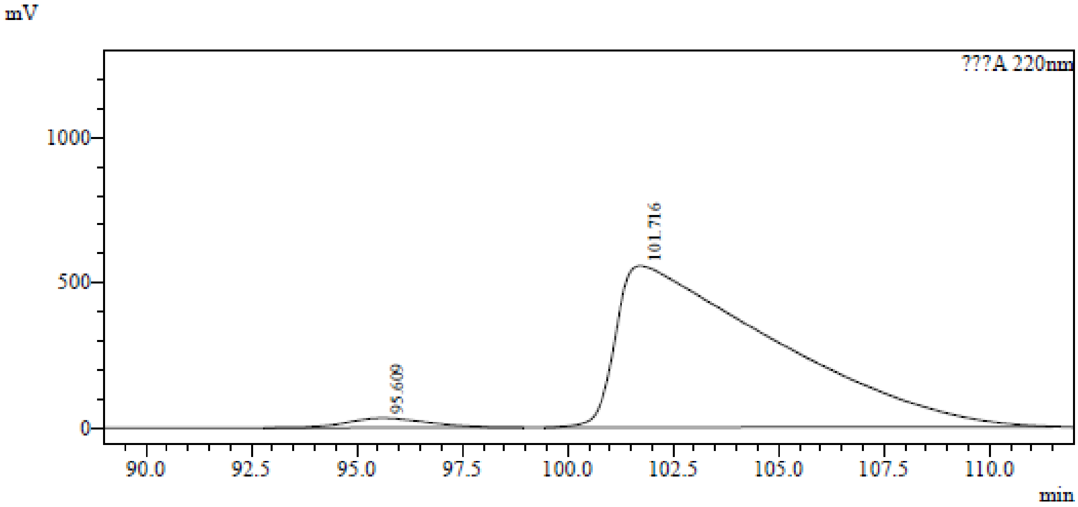

<Peak Table>

???A 220nm

Peak#	Ret. Time	Area	Height	Conc.	Unit	Mark	Name
1	95.609	4487457	33719	2.961			
2	101.716	147052857	557507	97.039		M	
Total		151540315	591226				

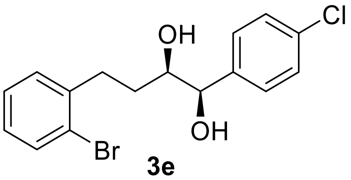

3e

<Chromatogram>

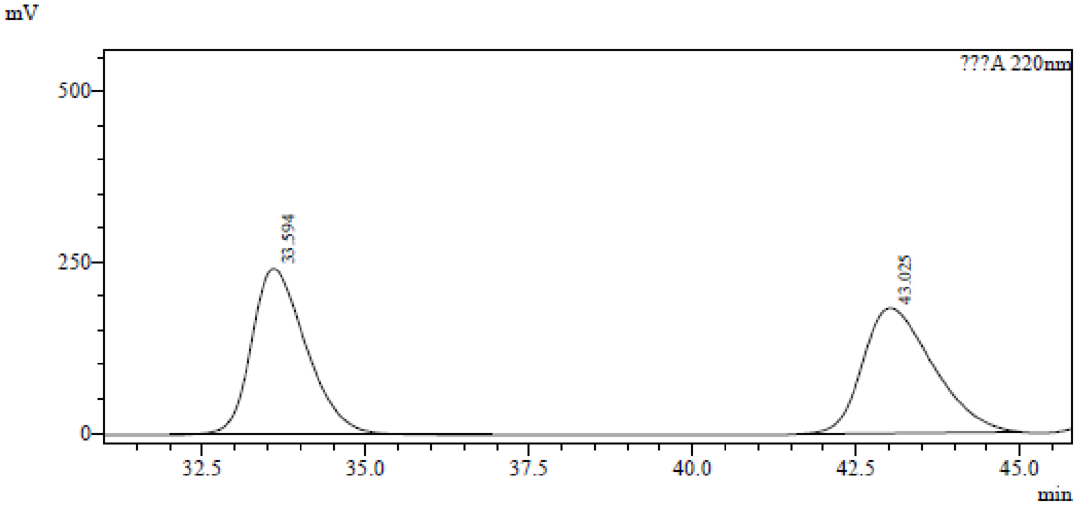

<Peak Table>

???A 220nm

Peak#	Ret. Time	Area	Height	Conc.	Unit	Mark	Name
1	33.594	14176774	242776	50.894			
2	43.025	13678773	182953	49.106		M	
Total		27855547	425729				

<Chromatogram>

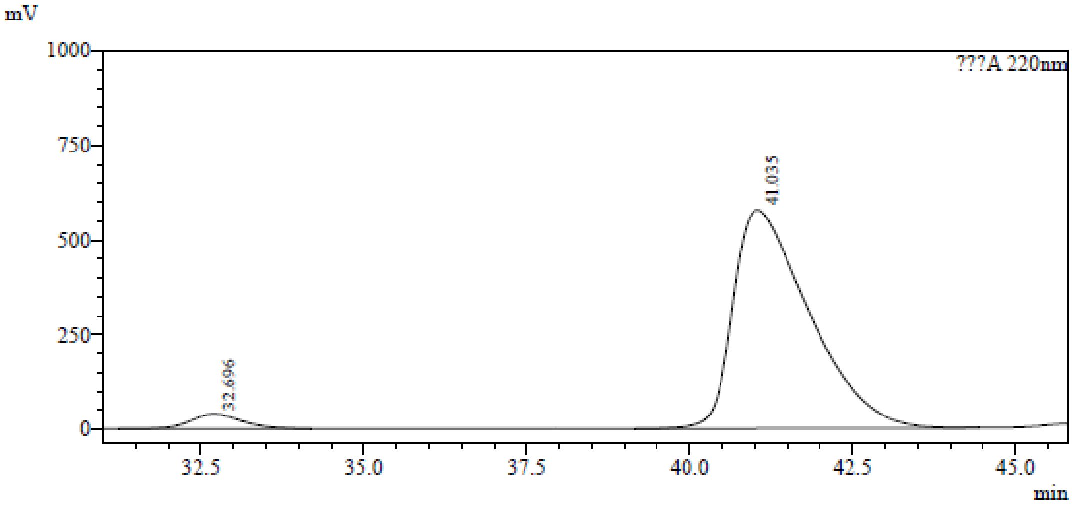

<Peak Table>

???A 220nm

Peak#	Ret. Time	Area	Height	Conc.	Unit	Mark	Name
1	32.696	2049129	37780	4.176		M	
2	41.035	47018436	577061	95.824			
Total		49067564	614841				

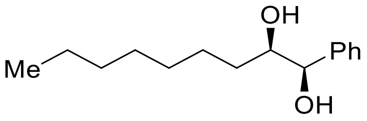

3f

<Chromatogram>

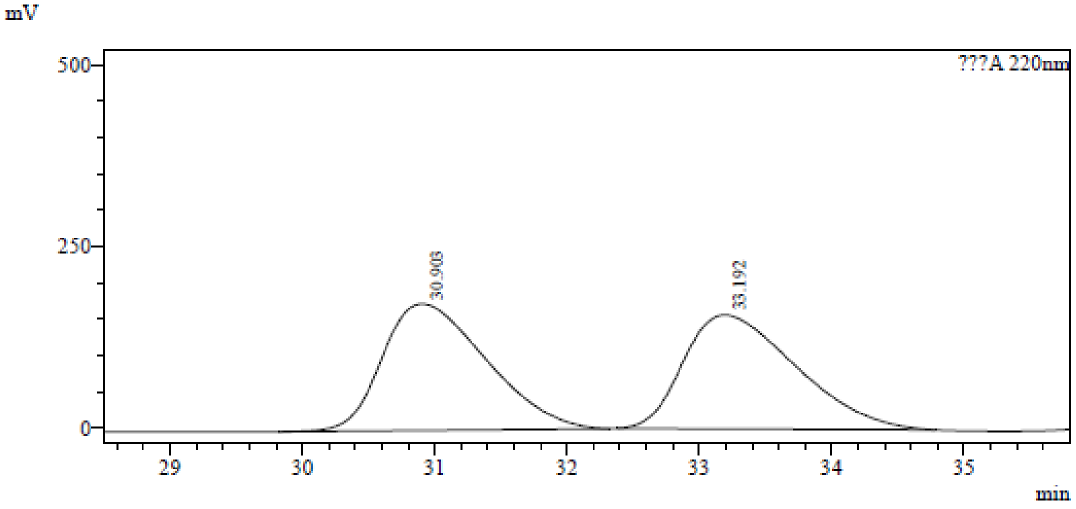

<Peak Table>

???A 220nm

Peak#	Ret. Time	Area	Height	Conc.	Unit	Mark	Name
1	30.903	9474176	174258	50.628			
2	33.192	9238997	156512	49.372		M	
Total		18713172	330770				

<Chromatogram>

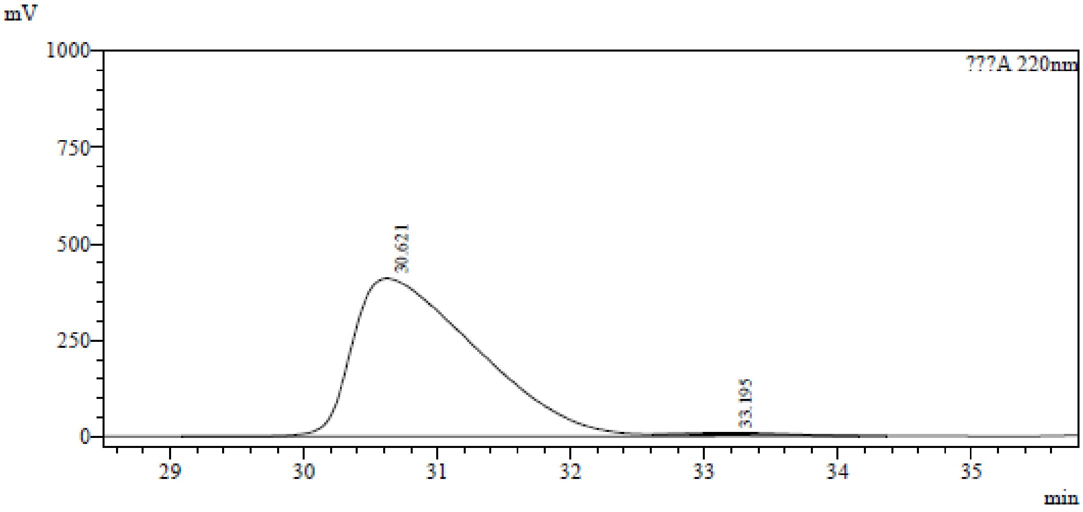

<Peak Table>

???A 220nm

Peak#	Ret. Time	Area	Height	Conc.	Unit	Mark	Name
1	30.621	26679248	409962	99.025		S	
2	33.195	262562	5803	0.975		T	
Total		26941810	415765				

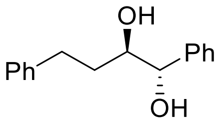

4a

<Chromatogram>

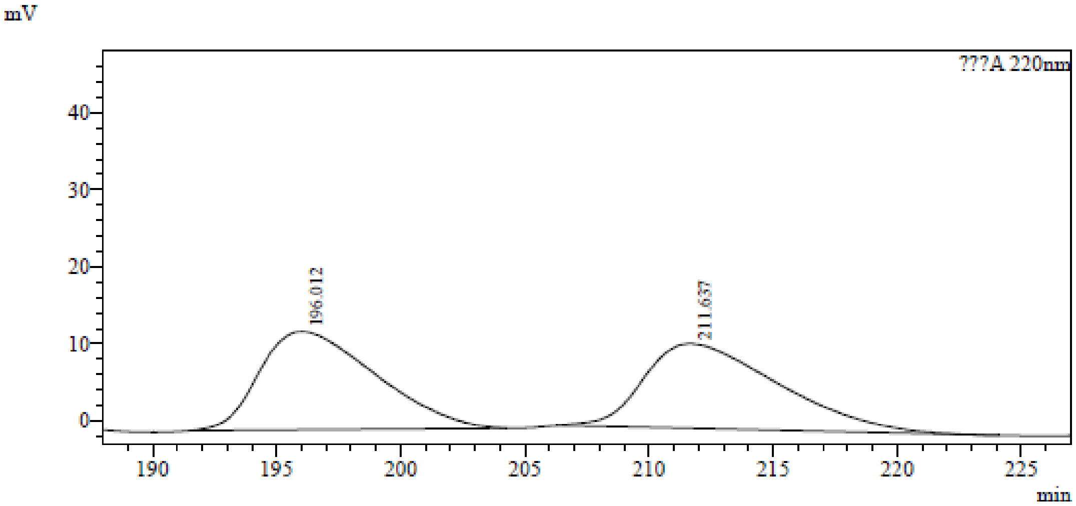

<Peak Table>

???A 220nm

Peak#	Ret. Time	Area	Height	Conc.	Unit	Mark	Name
1	196.012	4138613	12775	49.768			
2	211.637	4177205	11002	50.232		M	
Total		8315818	23777				

<Chromatogram>

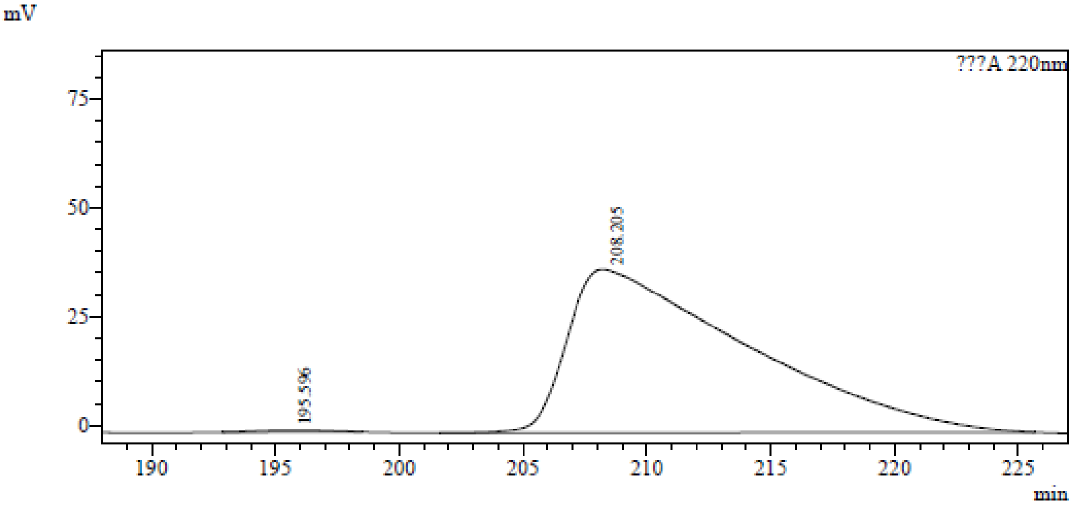

<Peak Table>

???A 220nm

Peak#	Ret. Time	Area	Height	Conc.	Unit	Mark	Name
1	195.596	101845	516	0.532		M	
2	208.205	19026803	37522	99.468		M	
Total		19128647	38038				

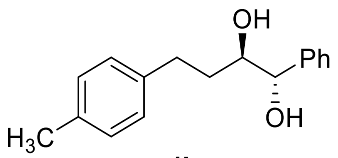

4b

<Chromatogram>

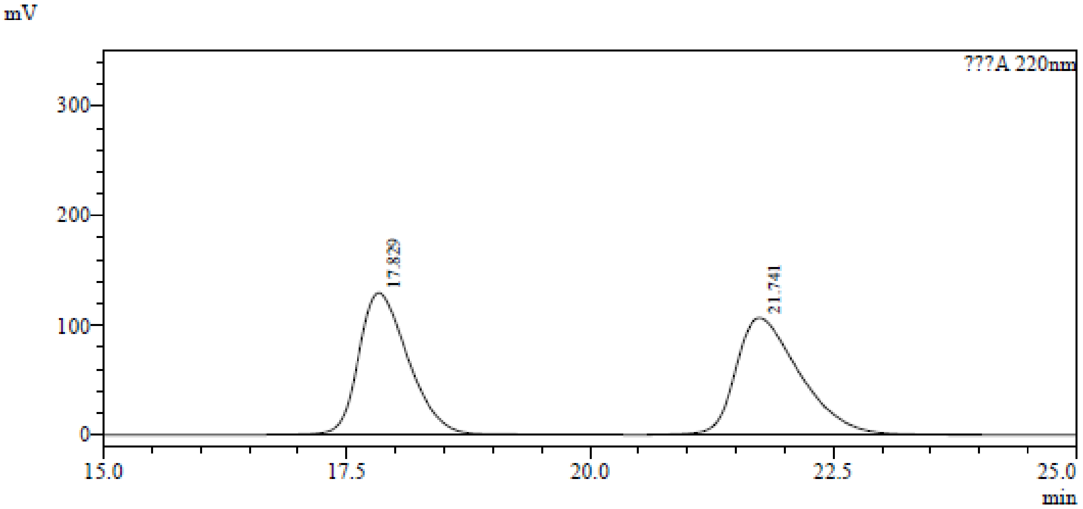

<Peak Table>

???A 220nm

Peak#	Ret. Time	Area	Height	Conc.	Unit	Mark	Name
1	17.829	4580820	129136	48.347			
2	21.741	4894117	106462	51.653			
Total		9474937	235598				

<Chromatogram>

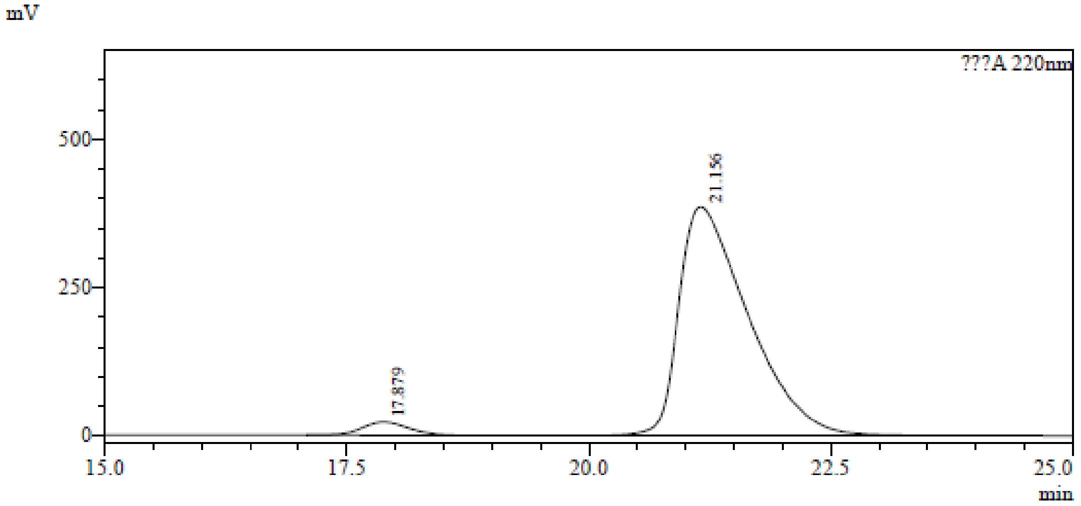

<Peak Table>

???A 220nm

Peak#	Ret. Time	Area	Height	Conc.	Unit	Mark	Name
1	17.879	733673	21805	3.690			
2	21.156	19149278	385308	96.310		M	
Total		19882951	407113				

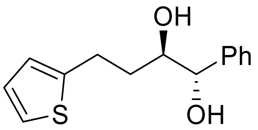

4c

<Chromatogram>

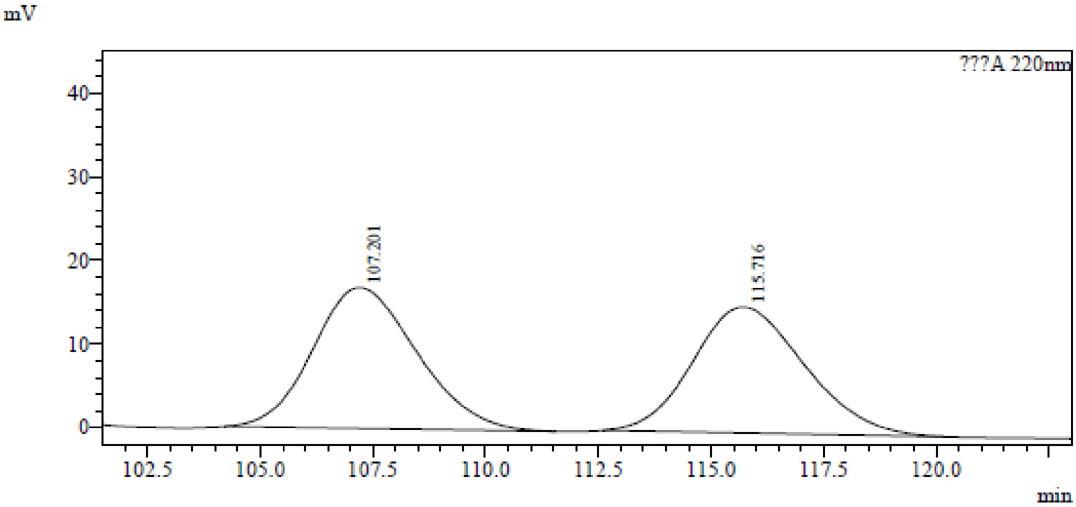

<Peak Table>

???A 220nm

Peak#	Ret. Time	Area	Height	Conc.	Unit	Mark	Name
1	107.201	2701945	16837	50.944			
2	115.716	2601808	15057	49.056			
Total		5303753	31894				

<Chromatogram>

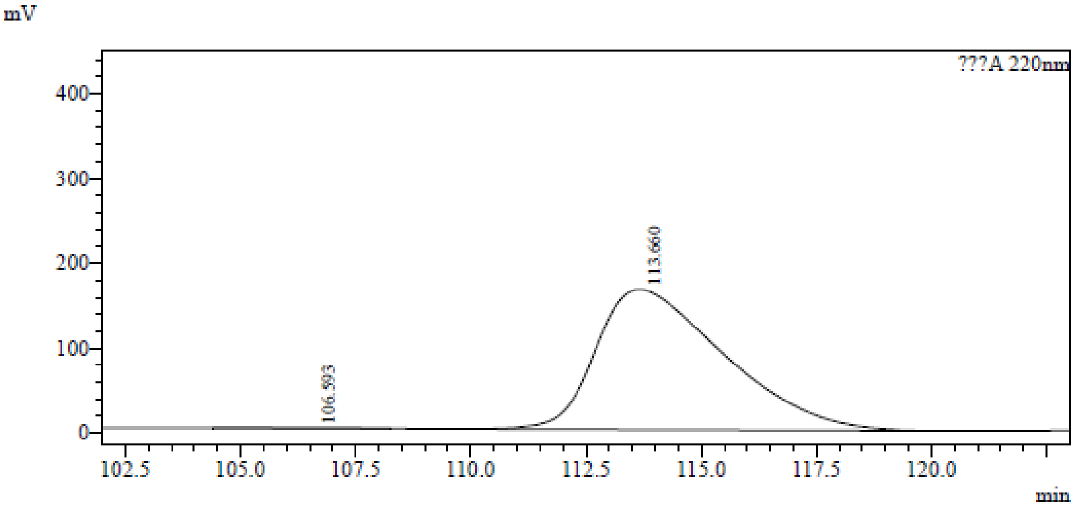

<Peak Table>

???A 220nm

Peak#	Ret. Time	Area	Height	Conc.	Unit	Mark	Name
1	106.593	56038	505	0.175		M	
2	113.660	32021189	165200	99.825			
Total		32077226	165705				

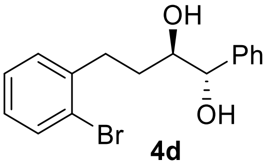

4d

<Chromatogram>

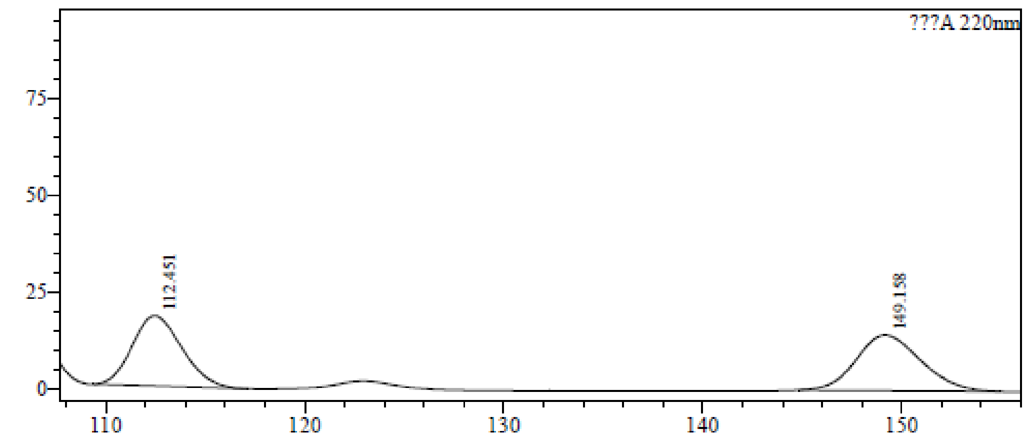

<Peak Table>

???A 220nm

Peak#	Ret. Time	Area	Height	Conc.	Unit	Mark	Name
1	112.451	3165360	18140	49.256			
2	149.158	3261002	14392	50.744			
Total		6426362	32533				

<Chromatogram>

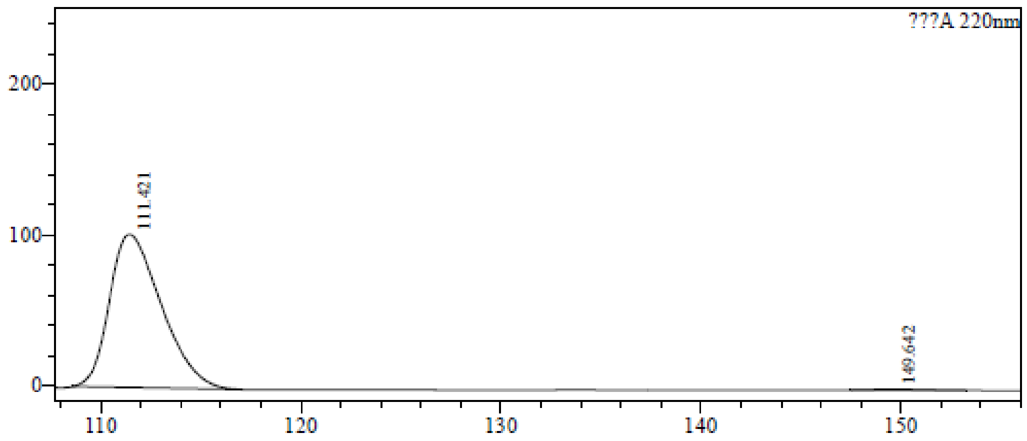

<Peak Table>

???A 220nm

Peak#	Ret. Time	Area	Height	Conc.	Unit	Mark	Name
1	111.421	18275488	101436	99.373		M	
2	149.642	115305	640	0.627		M	
Total		18390792	102076				

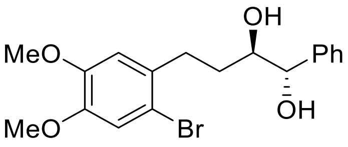

4e

<Chromatogram>

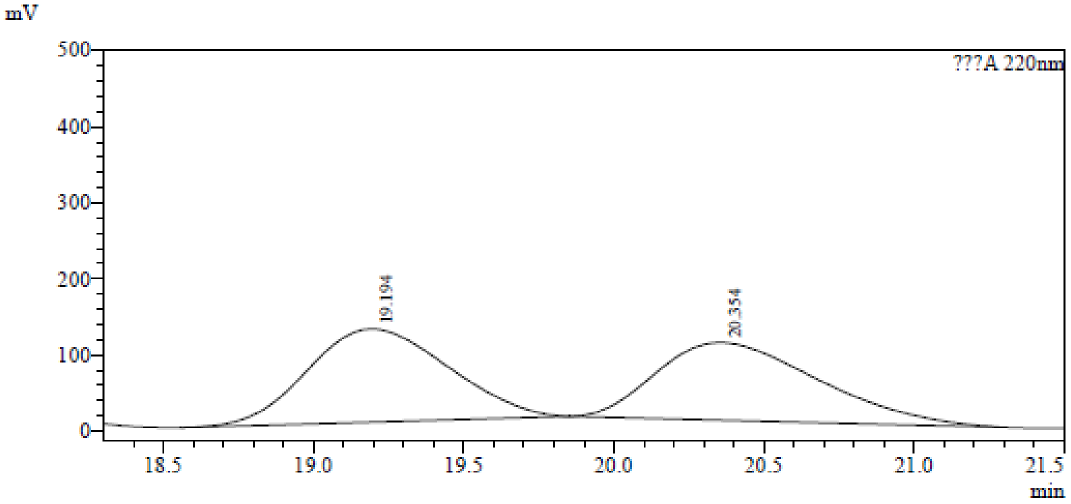

<Peak Table>

???A 220nm

Peak#	Ret. Time	Area	Height	Conc.	Unit	Mark	Name
1	19.194	3969901	122110	50.994		M	
2	20.354	3815138	101734	49.006		M	
Total		7785039	223844				

<Chromatogram>

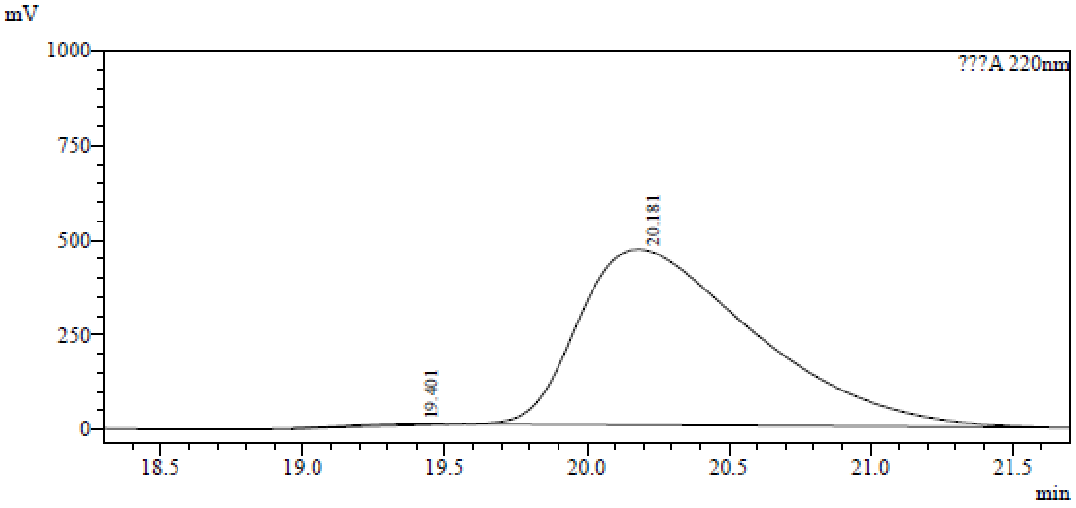

<Peak Table>

???A 220nm

Peak#	Ret. Time	Area	Height	Conc.	Unit	Mark	Name
1	19.401	105776	4544	0.526			
2	20.181	20017236	464311	99.474		M	
Total		20123012	468854				

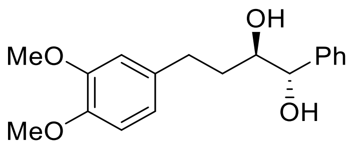

4f

<Chromatogram>

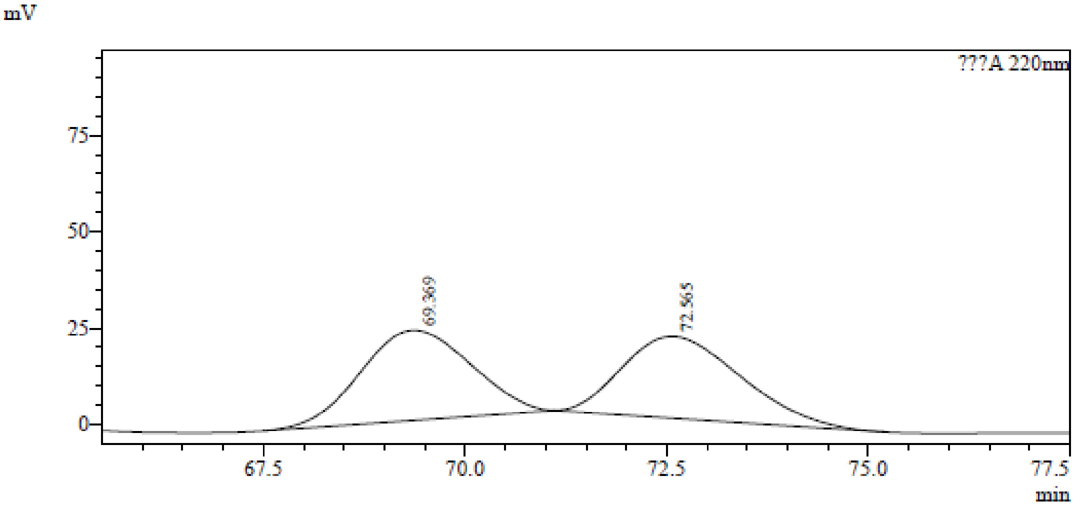

<Peak Table>

???A 220nm

Peak#	Ret. Time	Area	Height	Conc.	Unit	Mark	Name
1	69.369	2219775	23363	50.577		M	
2	72.565	2169137	21183	49.423			
Total		4388912	44547				

<Chromatogram>

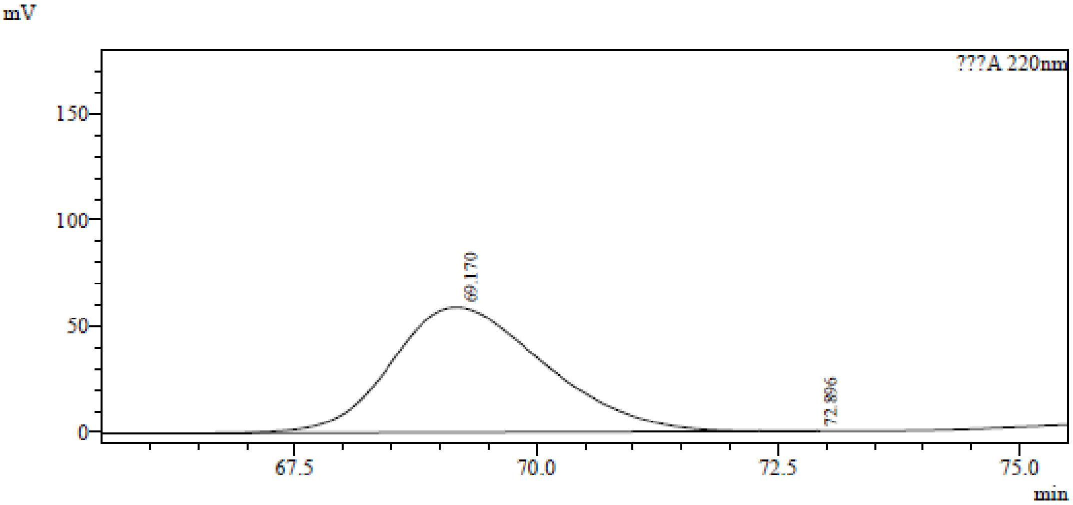

<Peak Table>

???A 220nm

Peak#	Ret. Time	Area	Height	Conc.	Unit	Mark	Name
1	69.170	6533159	58989	99.959			
2	72.896	2708	56	0.041		M	
Total		6535867	59046				

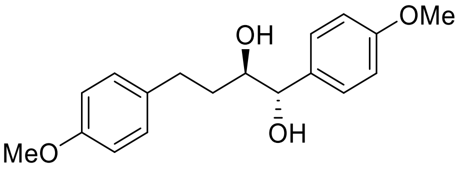

4g

<Chromatogram>

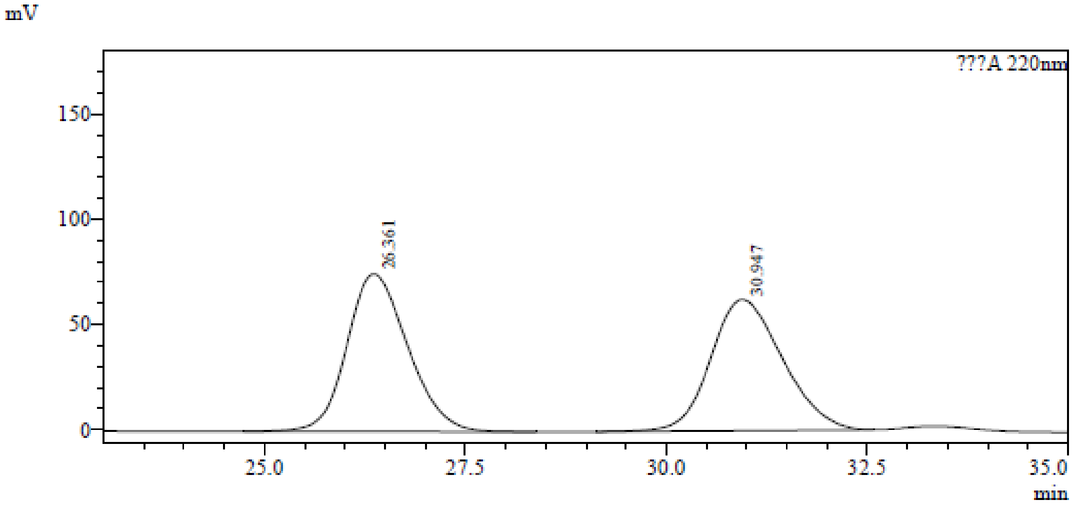

<Peak Table>

???A 220nm

Peak#	Ret. Time	Area	Height	Conc.	Unit	Mark	Name
1	26.361	3967995	74951	50.520			
2	30.947	3886346	62314	49.480			
Total		7854341	137266				

<Chromatogram>

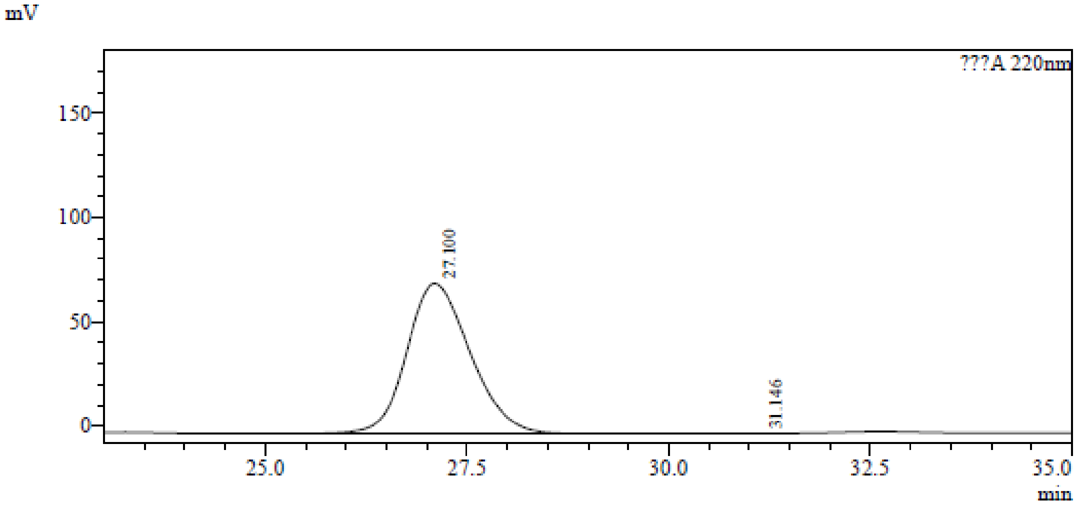

<Peak Table>

???A 220nm

Peak#	Ret. Time	Area	Height	Conc.	Unit	Mark	Name
1	27.100	3943485	71893	99.923			
2	31.146	3042	70	0.077		M	
Total		3946528	71962				

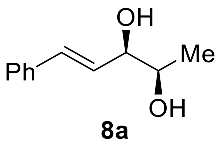

8a

<Chromatogram>

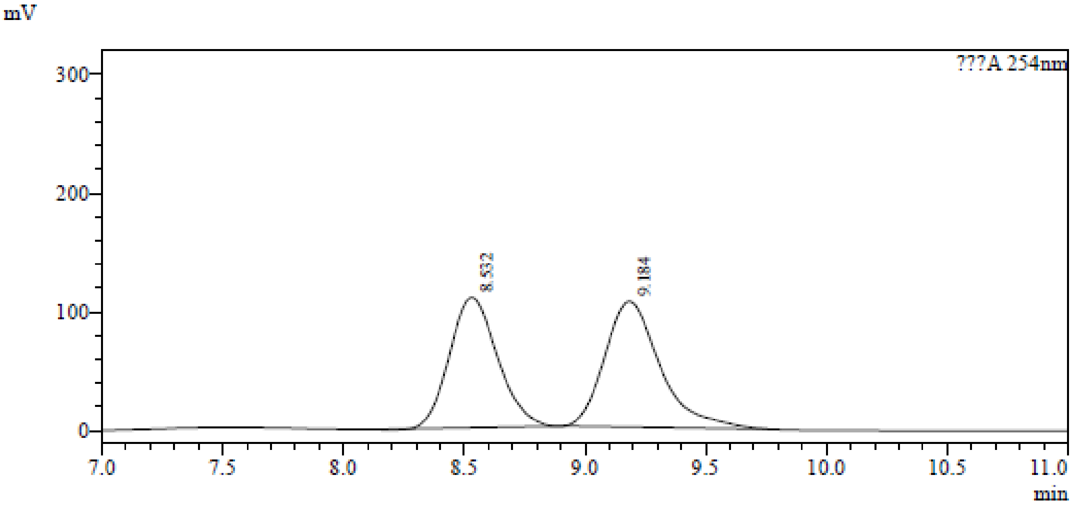

<Peak Table>

???A 254nm

Peak#	Ret. Time	Area	Height	Conc.	Unit	Mark	Name
1	8.532	1516228	109660	47.295		M	
2	9.184	1689696	105747	52.705		M	
Total		3205924	215407				

<Chromatogram>

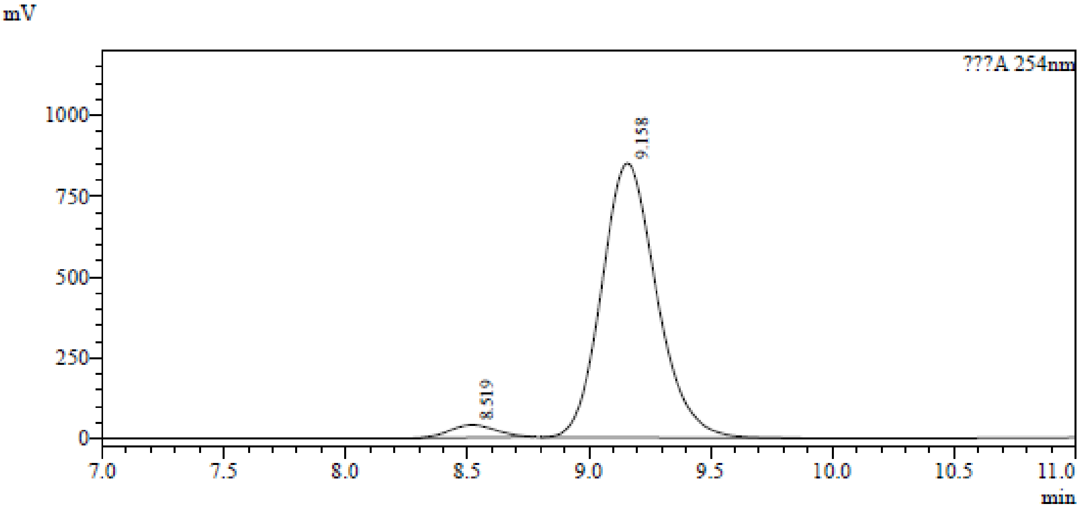

<Peak Table>

???A 254nm

Peak#	Ret. Time	Area	Height	Conc.	Unit	Mark	Name
1	8.519	530867	39941	3.817		M	
2	9.158	13377093	849632	96.183			
Total		13907960	889572				

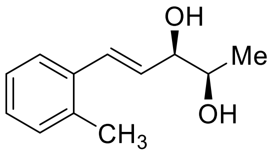

8b

<Chromatogram>

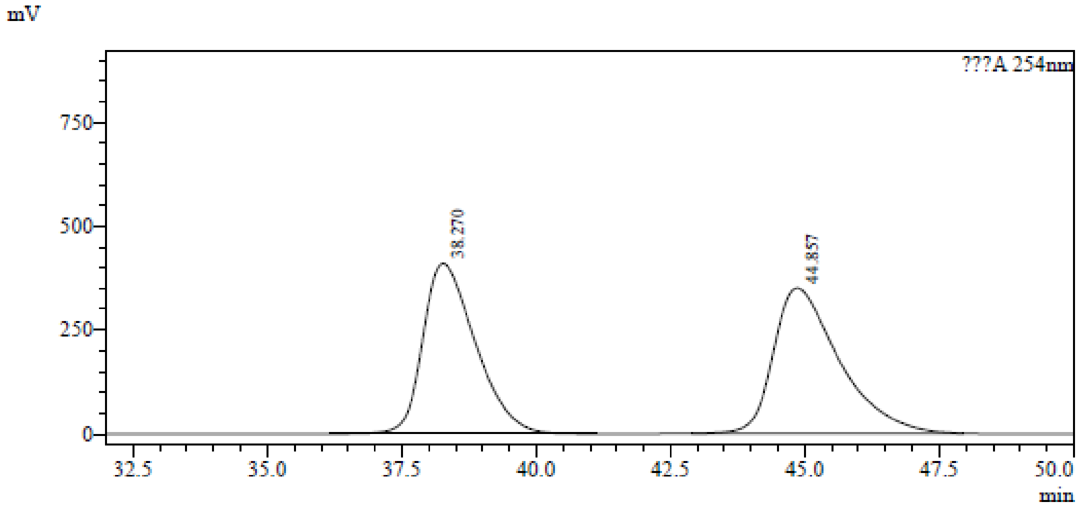

<Peak Table>

???A 254nm

Peak#	Ret. Time	Area	Height	Conc.	Unit	Mark	Name
1	38.270	27880834	409795	48.327			
2	44.857	29810646	349433	51.673		M	
Total		57691480	759228				

<Chromatogram>

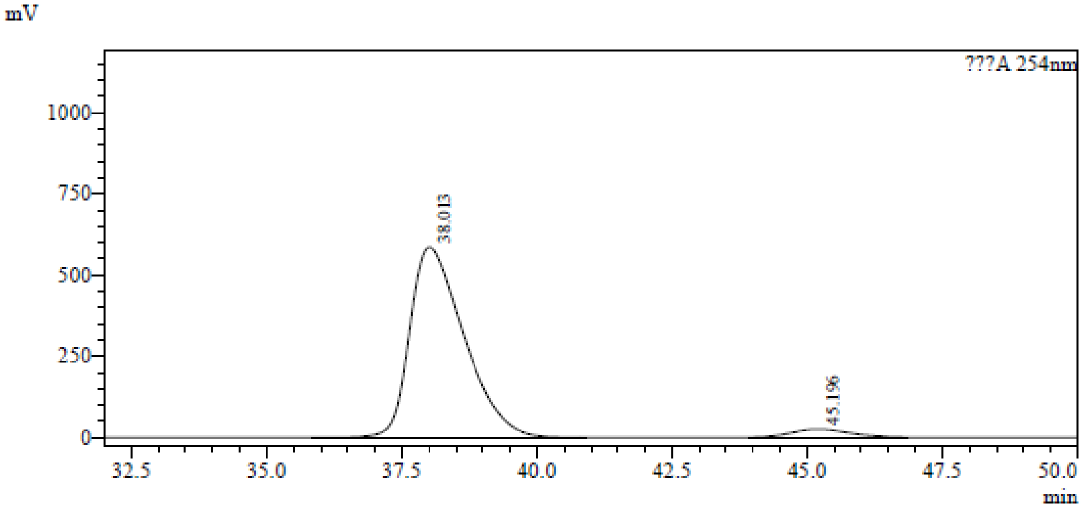

<Peak Table>

???A 254nm

Peak#	Ret. Time	Area	Height	Conc.	Unit	Mark	Name
1	38.013	41070570	587088	95.627		M	
2	45.196	1878231	25696	4.373		M	
Total		42948802	612784				

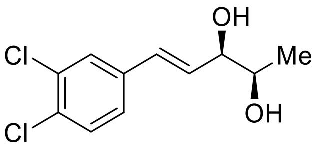

8c

<Chromatogram>

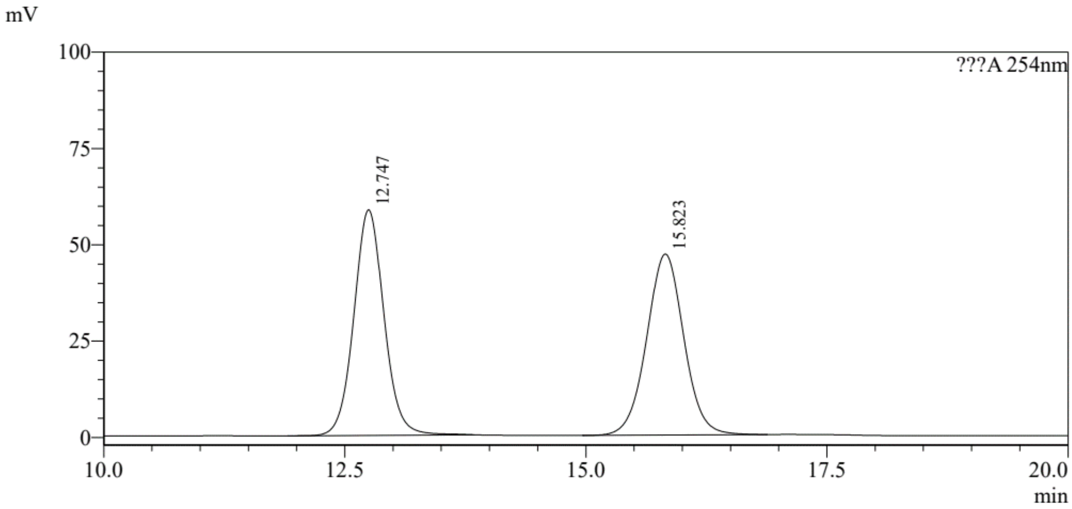

<Peak Table>

???A 254nm

Peak#	Ret. Time	Area	Height	Conc.	Unit	Mark	Name
1	12.747	1274065	58546	50.243		M	
2	15.823	1261757	46989	49.757			
Total		2535822	105535				

<Chromatogram>

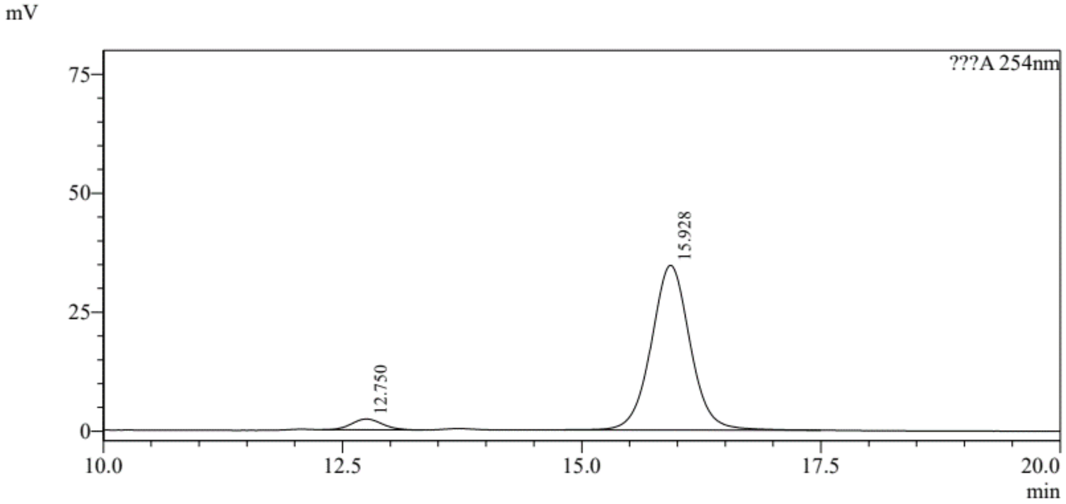

<Peak Table>

???A 254nm

Peak#	Ret. Time	Area	Height	Conc.	Unit	Mark	Name
1	12.750	50809	2281	4.935			
2	15.928	978798	34595	95.065			
Total		1029607	36876				

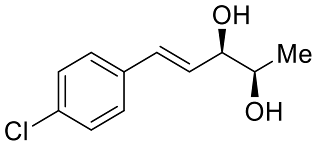

8d

<Chromatogram>

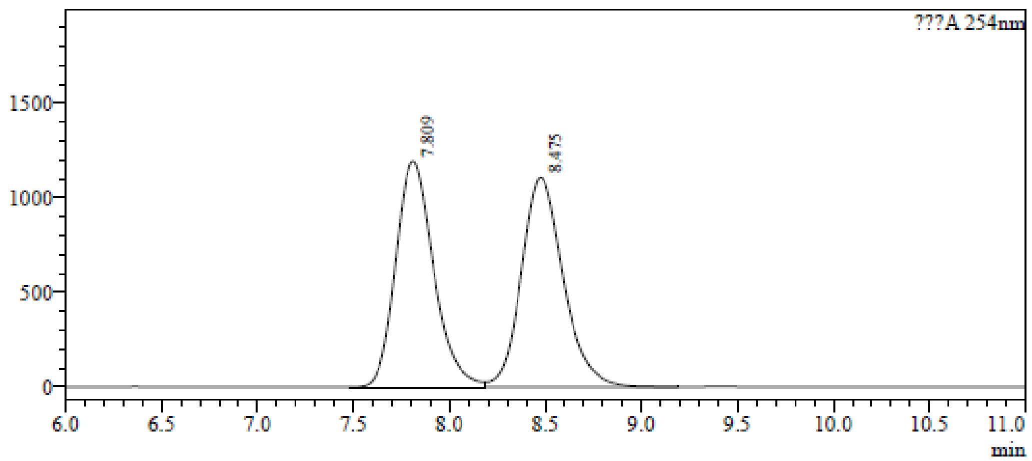

<Peak Table>

???A 254nm

Peak#	Ret. Time	Area	Height	Conc.	Unit	Mark	Name
1	7.809	16452563	1194150	49.442			
2	8.475	16824247	1108027	50.558		V	
Total		33276810	2302176				

<Chromatogram>

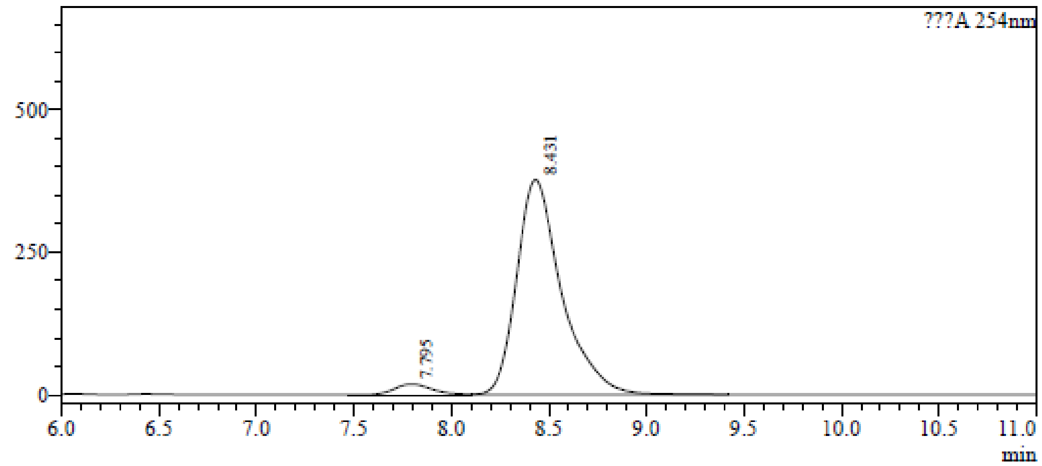

<Peak Table>

???A 254nm

Peak#	Ret. Time	Area	Height	Conc.	Unit	Mark	Name
1	7.795	247282	18486	3.920			
2	8.431	6061154	377383	96.080		V	
Total		6308437	395869				

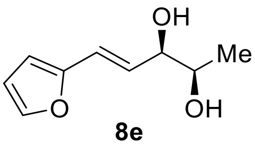

8e

<Chromatogram>

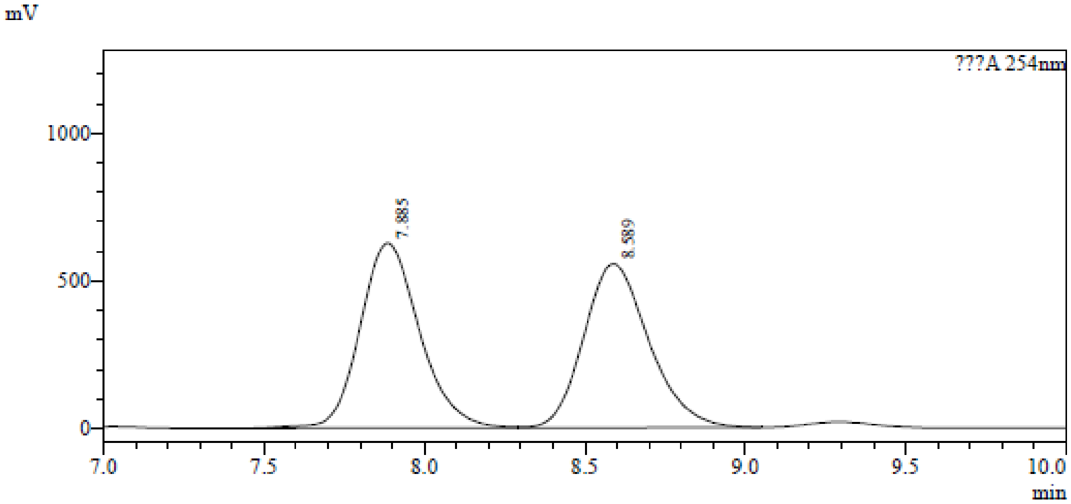

<Peak Table>

???A 254nm

Peak#	Ret. Time	Area	Height	Conc.	Unit	Mark	Name
1	0.096	1067	94	0.007			
2	7.885	8115293	627379	50.705			
3	8.589	7888500	555718	49.288		V	
Total		16004861	1183190				

<Chromatogram>

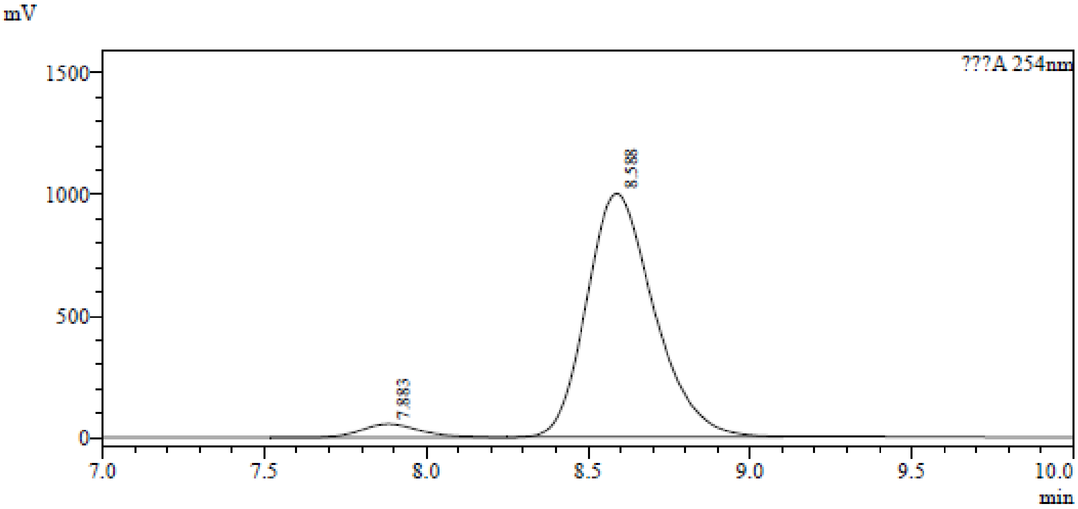

<Peak Table>

???A 254nm

Peak#	Ret. Time	Area	Height	Conc.	Unit	Mark	Name
1	7.883	686492	55429	4.501			
2	8.588	14566274	1002484	95.499			
Total		15252766	1057913				

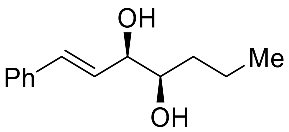

8f

<Chromatogram>

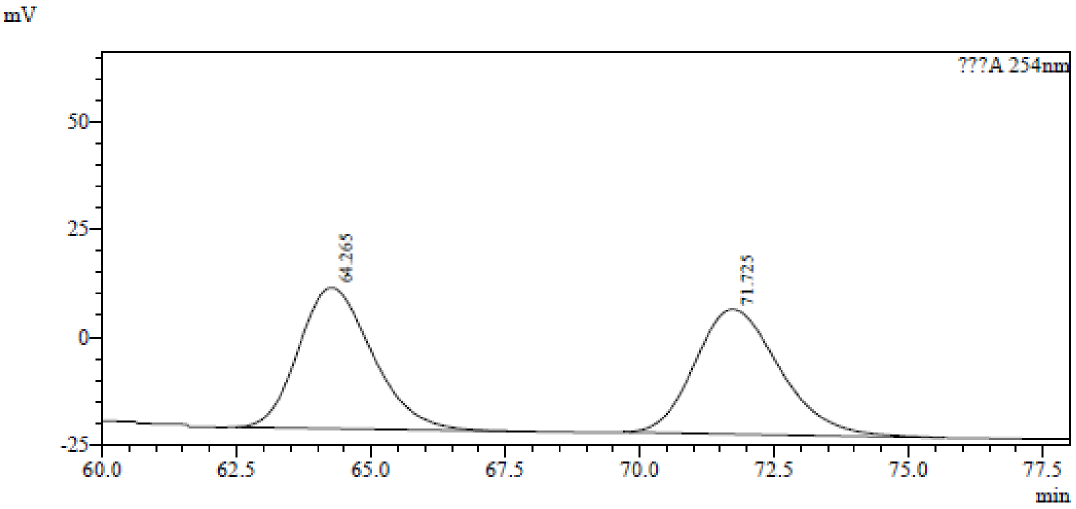

<Peak Table>

???A 254nm

Peak#	Ret. Time	Area	Height	Conc.	Unit	Mark	Name
1	64.265	3123294	32677	49.388			
2	71.725	3200696	28930	50.612			
Total		6323991	61607				

<Chromatogram>

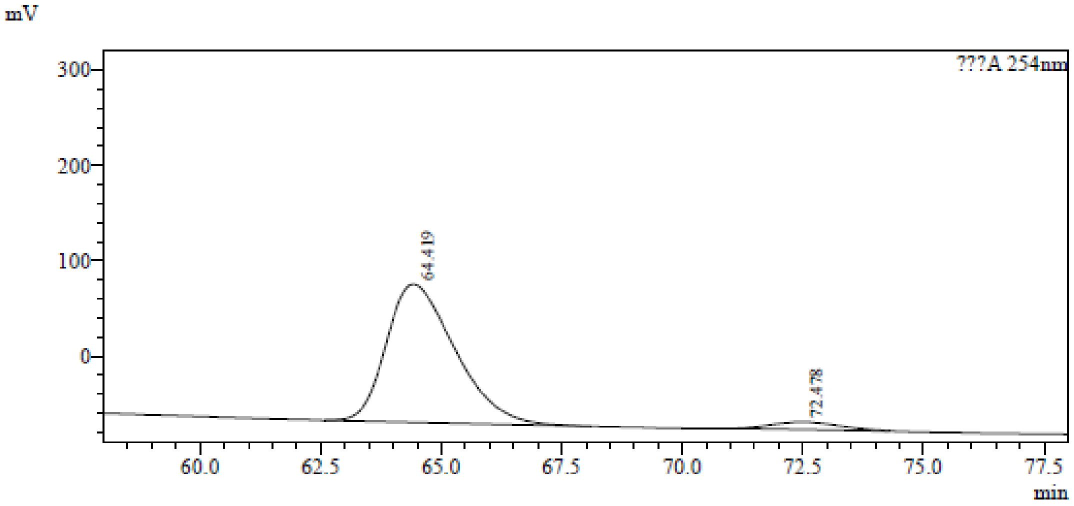

<Peak Table>

???A 254nm

Peak#	Ret. Time	Area	Height	Conc.	Unit	Mark	Name
1	64.419	14346046	144594	94.940			
2	72.478	764647	7912	5.060		M	
Total		15110693	152506				

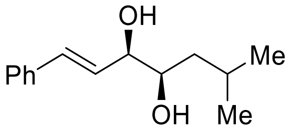

8g

<Chromatogram>

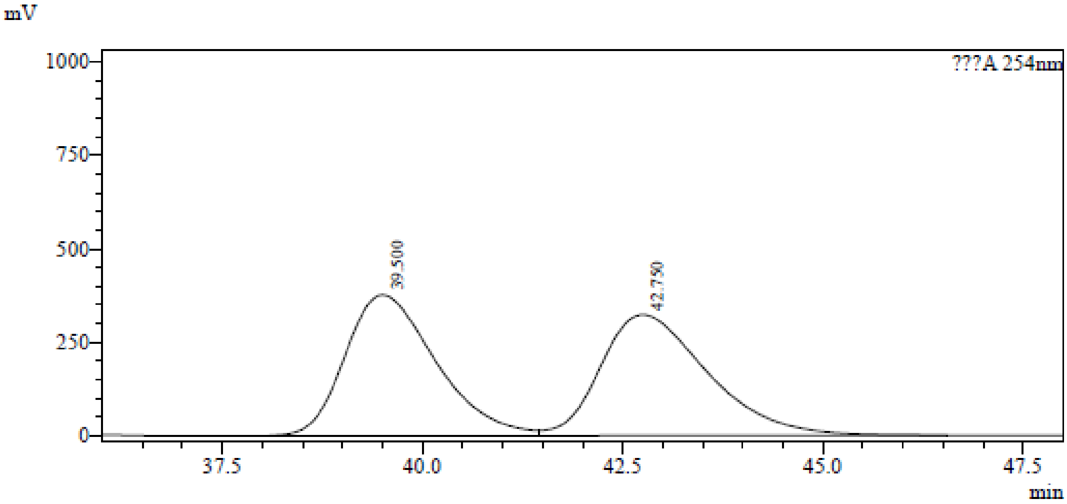

<Peak Table>

???A 254nm

Peak#	Ret. Time	Area	Height	Conc.	Unit	Mark	Name
1	39.500	29624868	376115	49.716			
2	42.750	29962797	323021	50.284		V	
Total		59587664	699136				

<Chromatogram>

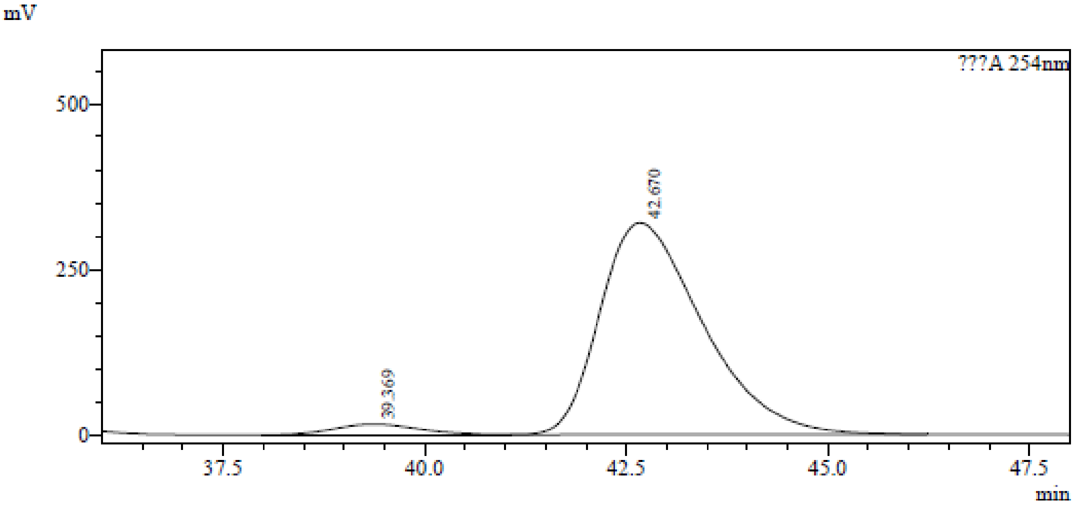

<Peak Table>

???A 254nm

Peak#	Ret. Time	Area	Height	Conc.	Unit	Mark	Name
1	39.369	1177350	16257	3.984			
2	42.670	28373594	319284	96.016		V	
Total		29550944	335541				

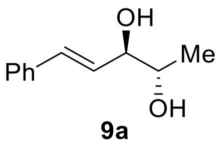

9a

<Chromatogram>

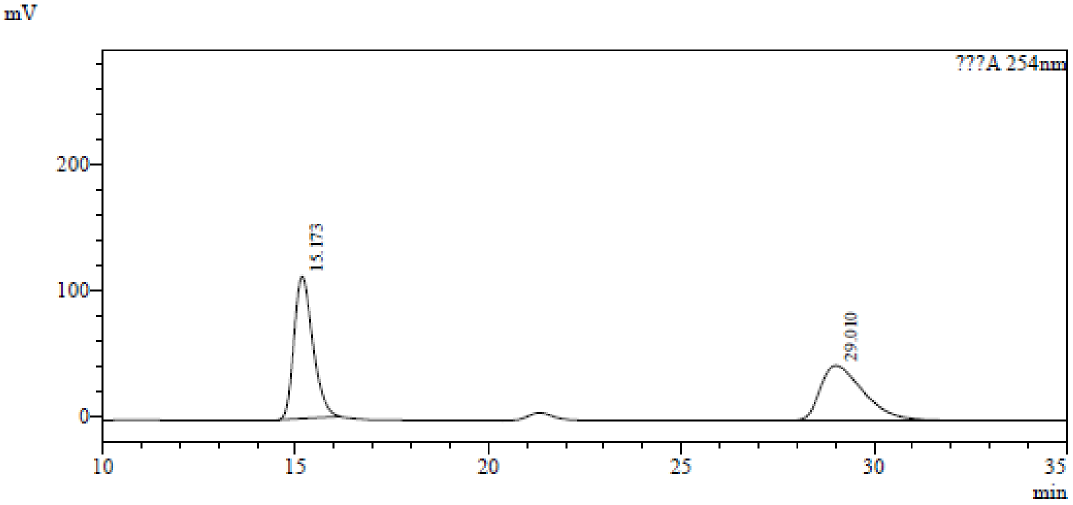

<Peak Table>

???A 254nm

Peak#	Ret. Time	Area	Height	Conc.	Unit	Mark	Name
1	15.173	3842470	112538	52.902		M	
2	29.010	3420861	43720	47.098			
Total		7263331	156258				

<Chromatogram>

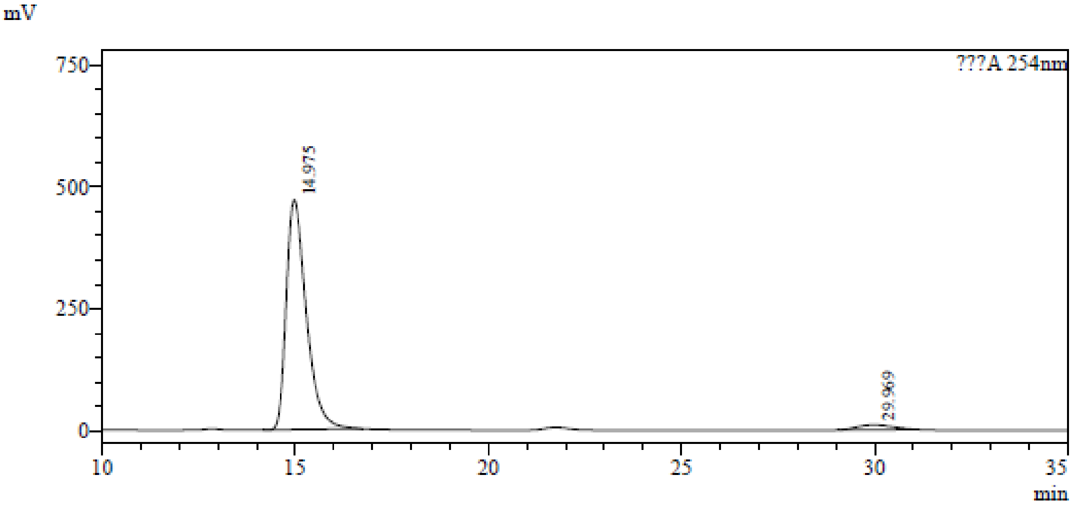

<Peak Table>

???A 254nm

Peak#	Ret. Time	Area	Height	Conc.	Unit	Mark	Name
1	14.975	17002247	471203	96.477		M	
2	29.969	620884	9655	3.523		M	
Total		17623131	480858				

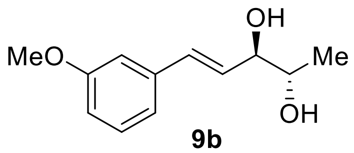

9b

<Chromatogram>

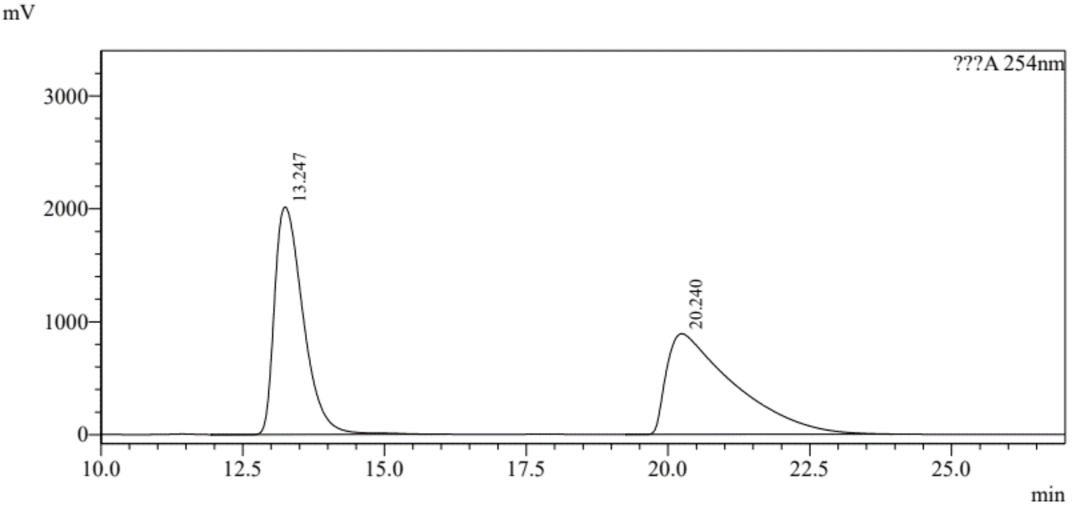

<Peak Table>

???A 254nm

Peak#	Ret. Time	Area	Height	Conc.	Unit	Mark	Name
1	13.247	70932186	2014269	49.045		M	
2	20.240	73693671	892026	50.955		M	
Total		144625857	2906295				

<Chromatogram>

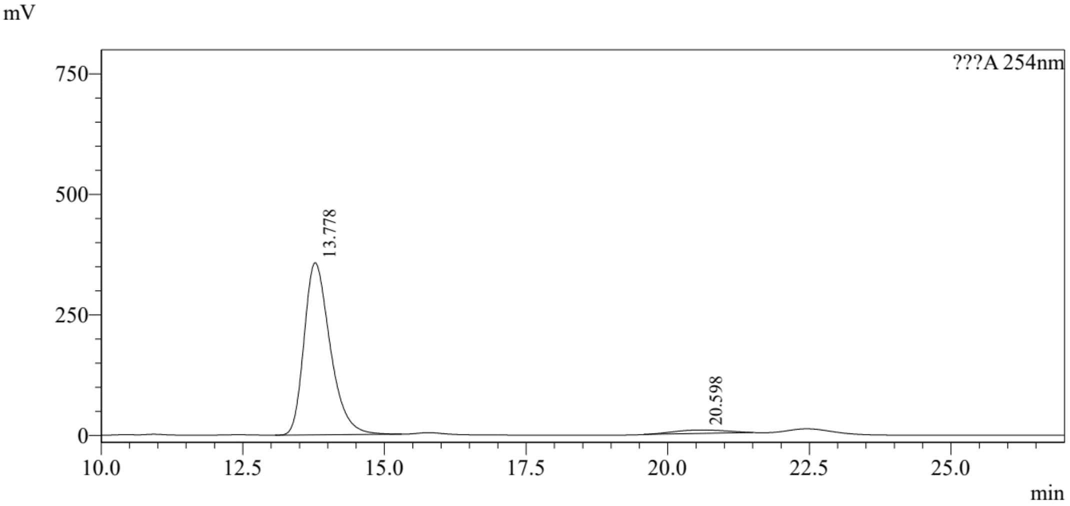

<Peak Table>

???A 254nm

Peak#	Ret. Time	Area	Height	Conc.	Unit	Mark	Name
1	13.778	11479609	357305	96.062			
2	20.598	470636	7219	3.938		M	
Total		11950245	364525				

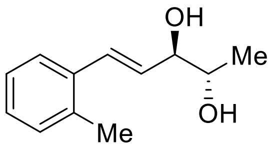

9c

<Chromatogram>

mV

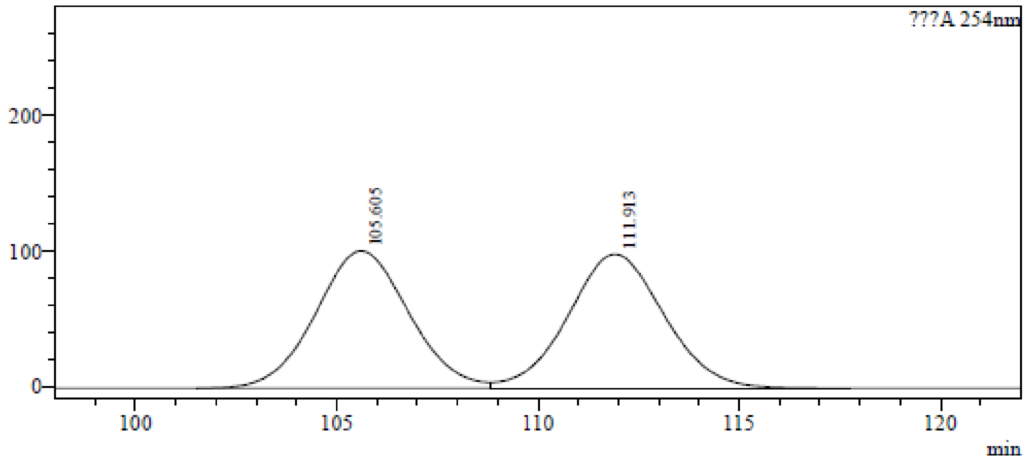

<Peak Table>

???A 254nm

Peak#	Ret. Time	Area	Height	Conc.	Unit	Mark	Name
1	105.605	16336916	101167	49.857			
2	111.913	16430408	98674	50.143		V	
Total		32767324	199841				

<Chromatogram>

mV

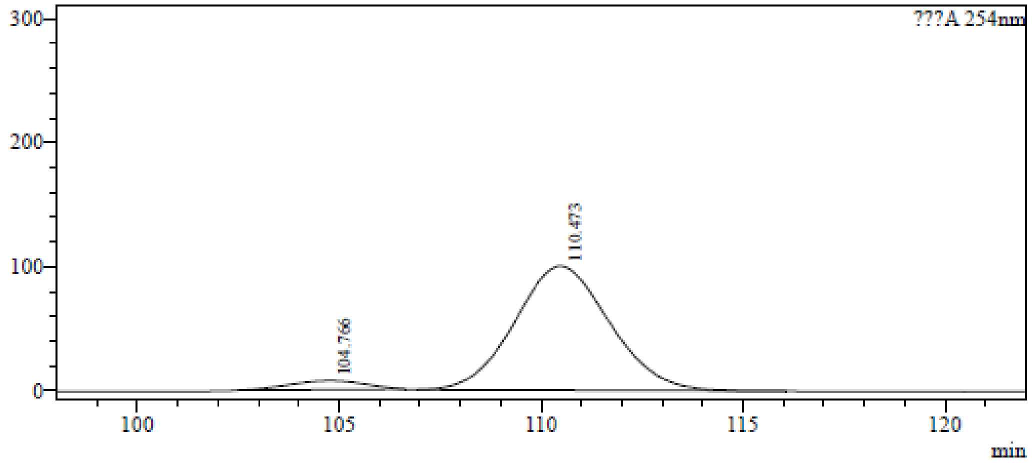

<Peak Table>

???A 254nm

Peak#	Ret. Time	Area	Height	Conc.	Unit	Mark	Name
1	104.766	897201	7069	5.202		M	
2	110.473	16348822	99949	94.798			
Total		17246023	107018				

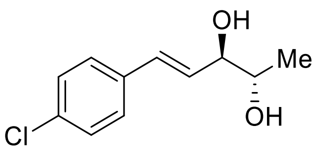

9d

<Chromatogram>

mV

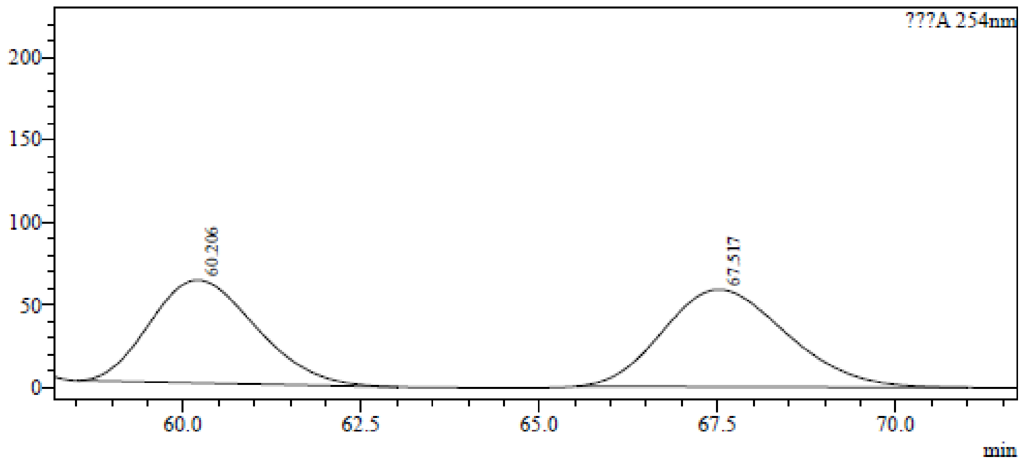

<Peak Table>

???A 254nm

Peak#	Ret. Time	Area	Height	Conc.	Unit	Mark	Name
1	60.206	6450185	62282	47.506		M	
2	67.517	7127365	58846	52.494		M	
Total		13577550	121129				

<Chromatogram>

mV

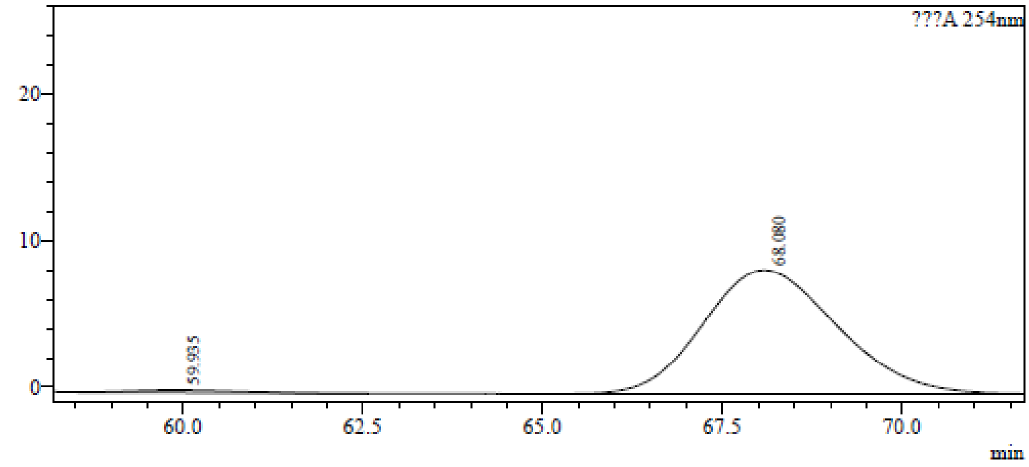

<Peak Table>

???A 254nm

Peak#	Ret. Time	Area	Height	Conc.	Unit	Mark	Name
1	59.935	17222	176	1.541		M	
2	68.080	1100391	8449	98.459		M	
Total		1117613	8626				

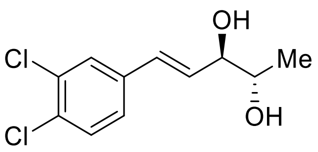

9e

<Chromatogram>

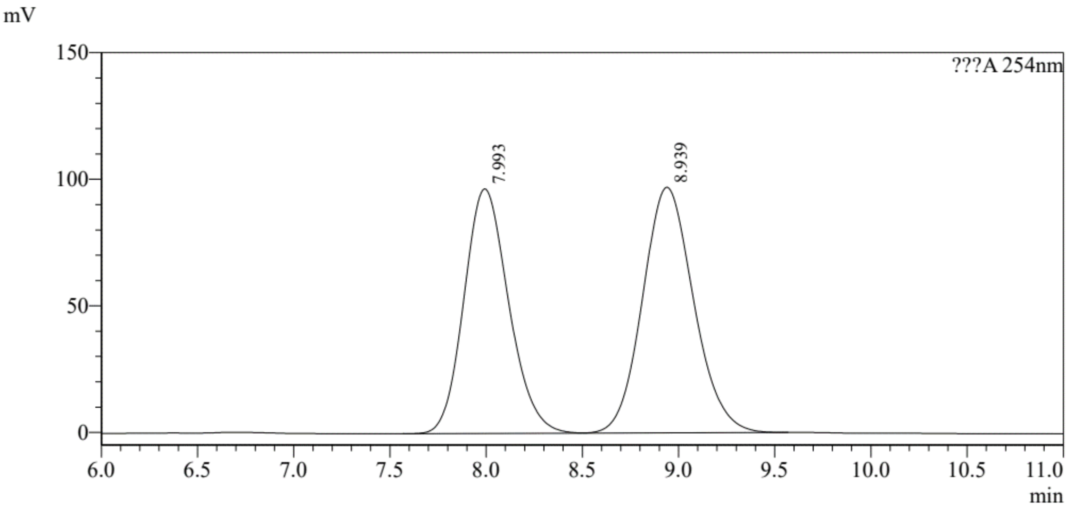

<Peak Table>

???A 254nm

Peak#	Ret. Time	Area	Height	Conc.	Unit	Mark	Name
1	7.993	1562996	96617	46.559			
2	8.939	1794041	96996	53.441		V	
Total		3357038	193612				

<Chromatogram>

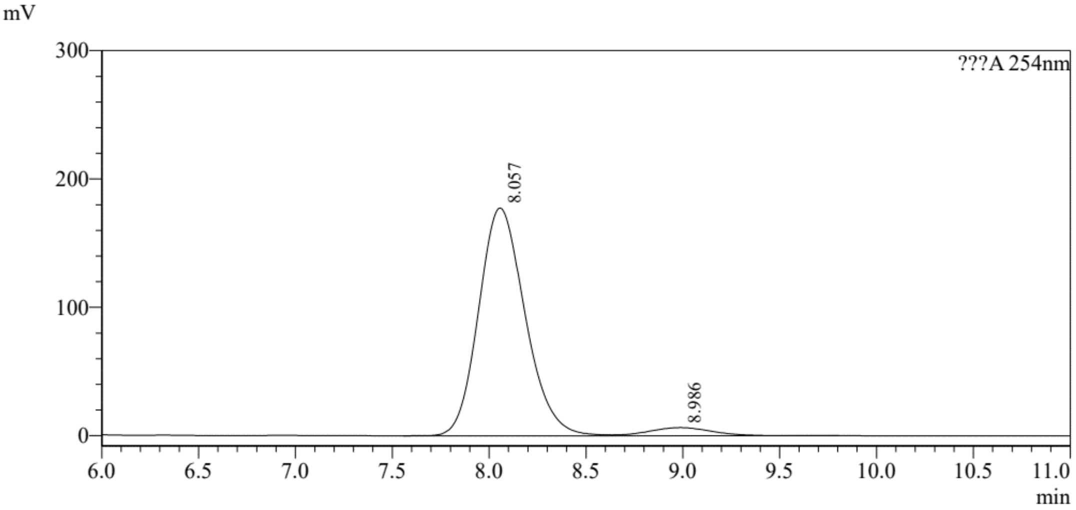

<Peak Table>

???A 254nm

Peak#	Ret. Time	Area	Height	Conc.	Unit	Mark	Name
1	8.057	2960581	177452	95.459			
2	8.986	140833	6312	4.541		V	
Total		3101414	183764				

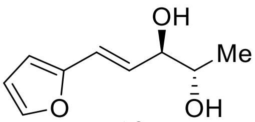

9f

<Chromatogram>

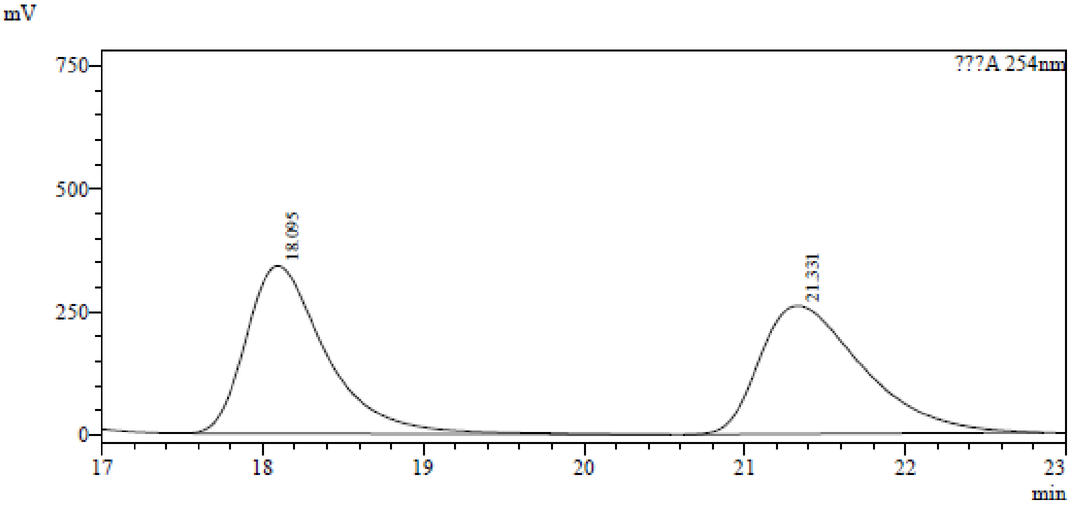

<Peak Table>

???A 254nm

Peak#	Ret. Time	Area	Height	Conc.	Unit	Mark	Name
1	18.095	11856271	340635	50.407			
2	21.331	11664910	260348	49.593		M	
Total		23521181	600983				

<Chromatogram>

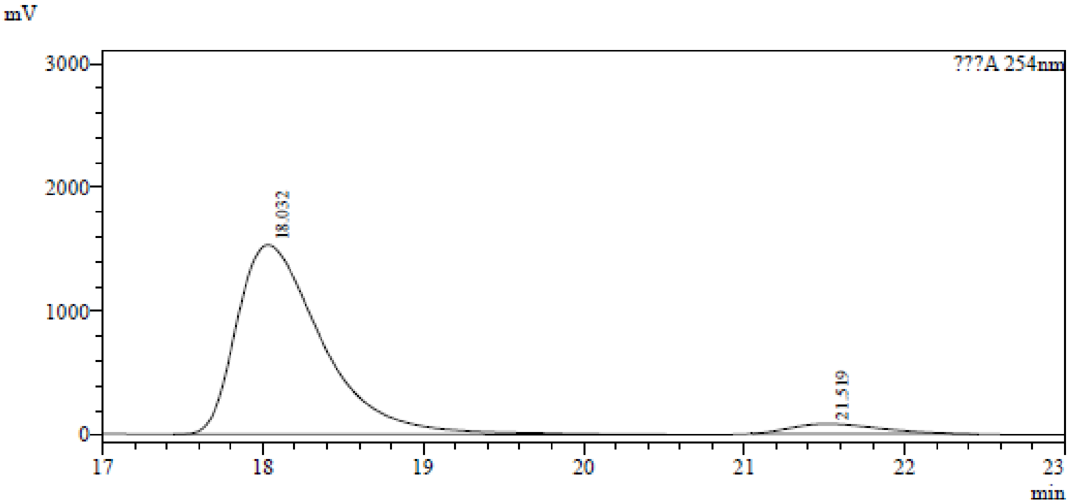

<Peak Table>

???A 254nm

Peak#	Ret. Time	Area	Height	Conc.	Unit	Mark	Name
1	18.032	56719311	1527253	94.792			
2	21.519	3116148	78777	5.208		M	
Total		59835459	1606030				

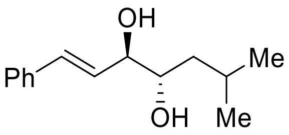

9g

<Chromatogram>

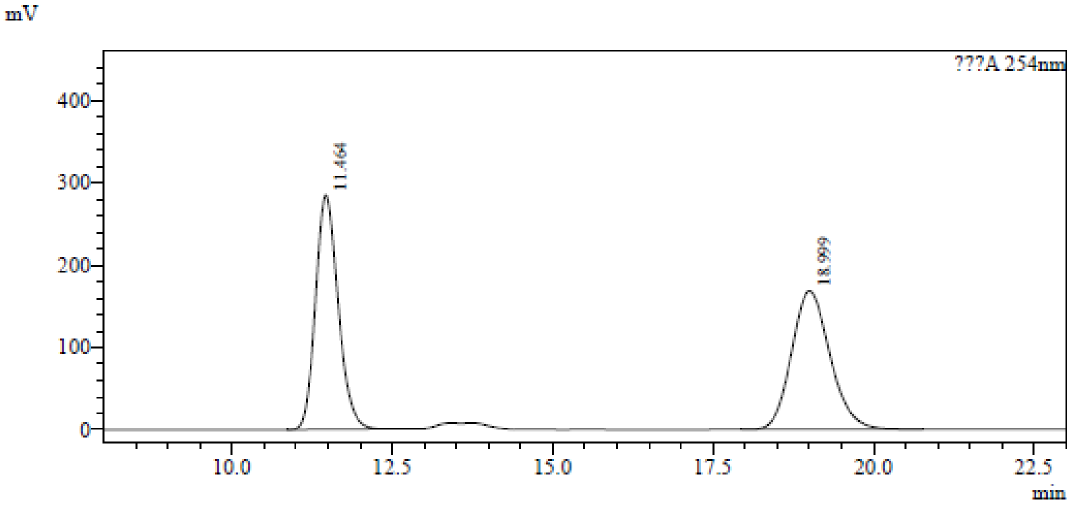

<Peak Table>

???A 254nm

Peak#	Ret. Time	Area	Height	Conc.	Unit	Mark	Name
1	11.464	6997949	284826	49.863			
2	18.999	7036277	168066	50.137			
Total		14034226	452892				

<Chromatogram>

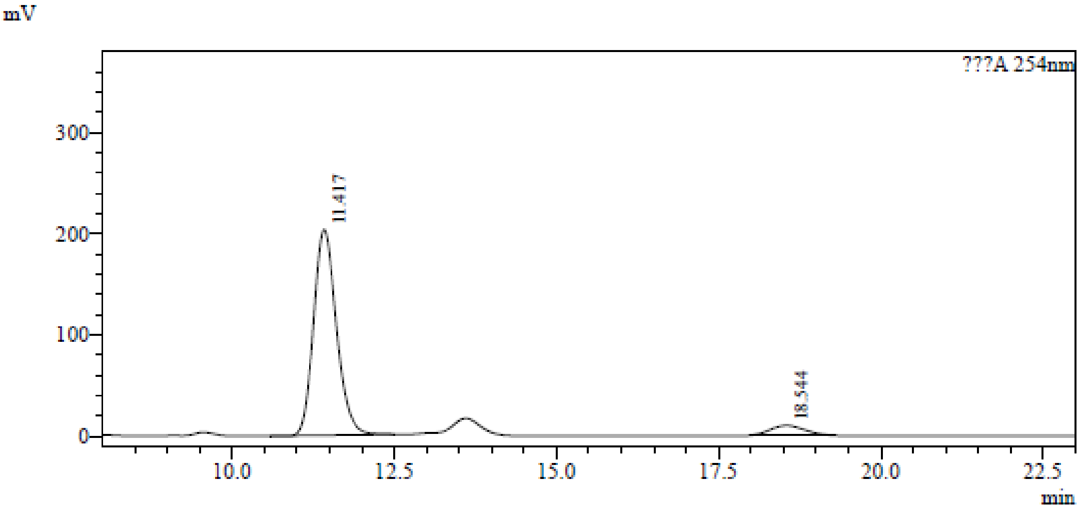

<Peak Table>

???A 254nm

Peak#	Ret. Time	Area	Height	Conc.	Unit	Mark	Name
1	11.417	4978566	203049	93.353		M	
2	18.544	354464	9578	6.647		M	
Total		5333029	212627				

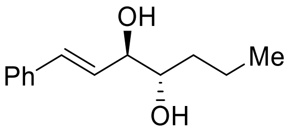

9h

<Chromatogram>

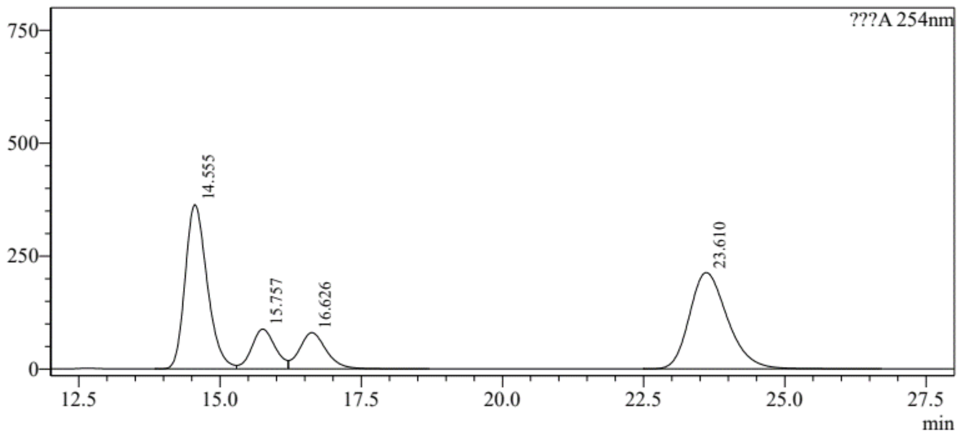

<Peak Table>

???A 254nm

Peak#	Ret. Time	Area	Height	Conc.	Unit	Mark	Name
1	14.555	9895869	363640	39.423			
2	15.757	2548330	88108	10.152		V	
3	16.626	2645918	80267	10.541		V	
4	23.610	10011396	213364	39.884			
Total		25101512	745378				

<Chromatogram>

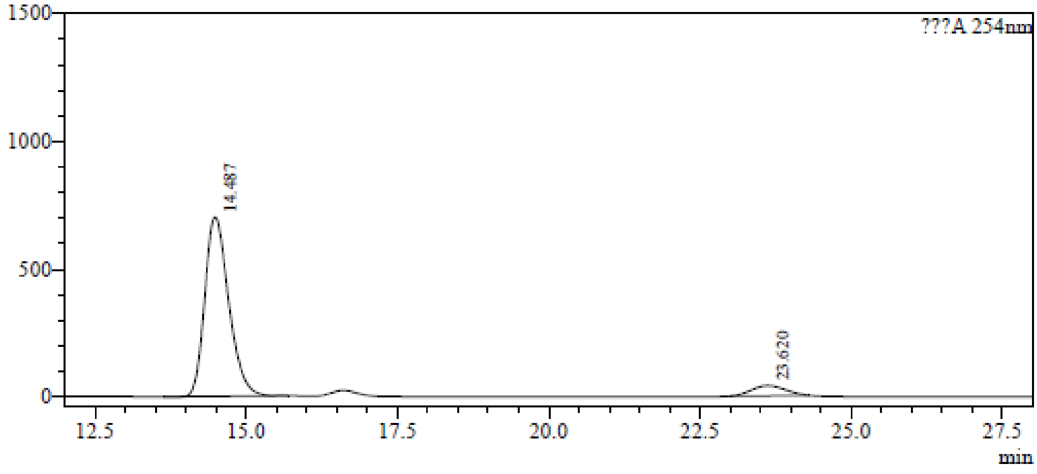

<Peak Table>

???A 254nm

Peak#	Ret. Time	Area	Height	Conc.	Unit	Mark	Name
1	14.487	19405093	702030	92.517		M	
2	23.620	1569638	39634	7.483		M	
Total		20974731	741663				

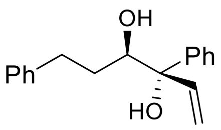

10a

<Chromatogram>

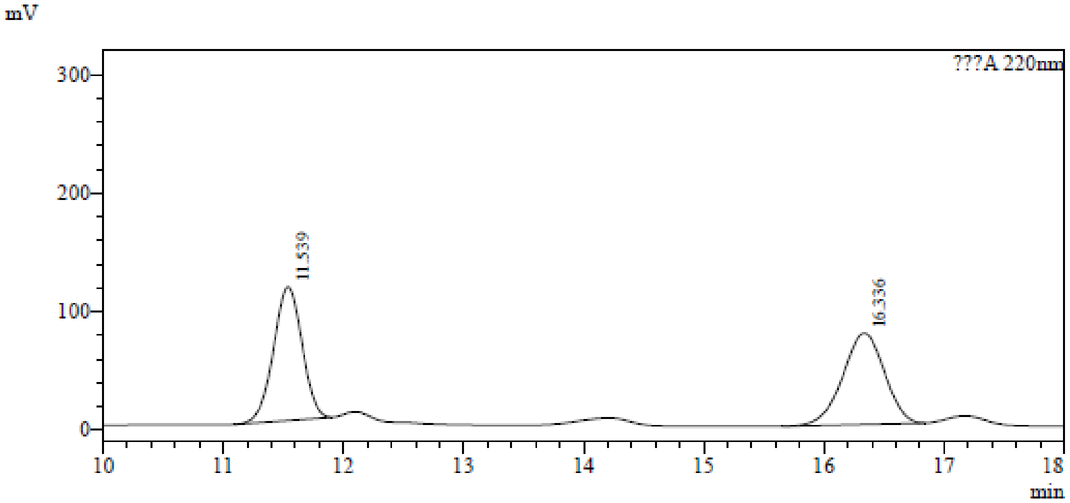

<Peak Table>

???A 220nm

Peak#	Ret. Time	Area	Height	Conc.	Unit	Mark	Name
1	11.539	1868451	112663	50.119			
2	16.336	1859608	76918	49.881			
Total		3728059	189580				

<Chromatogram>

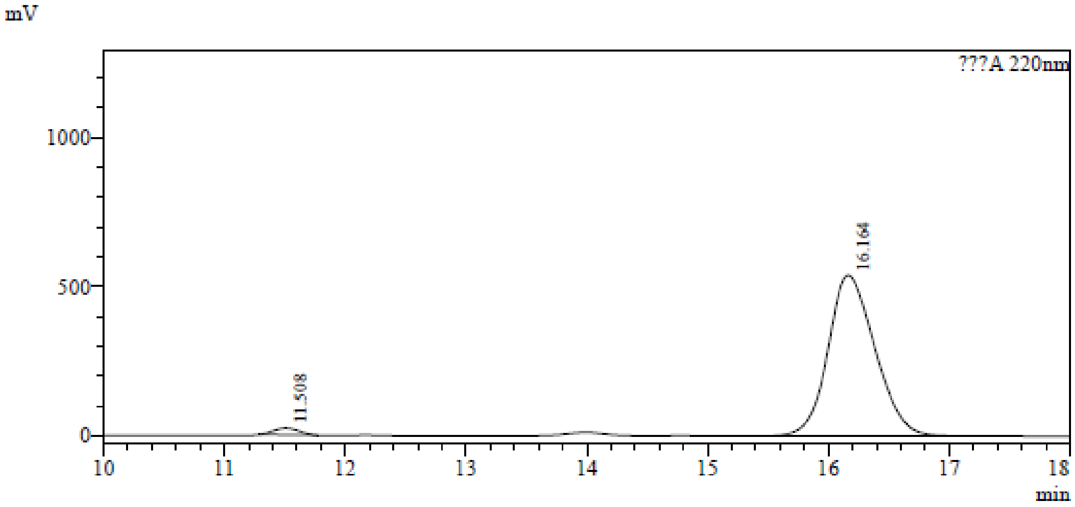

<Peak Table>

???A 220nm

Peak#	Ret. Time	Area	Height	Conc.	Unit	Mark	Name
1	11.508	311828	21631	2.100		M	
2	16.164	14533599	538086	97.900		M	
Total		14845427	559717				

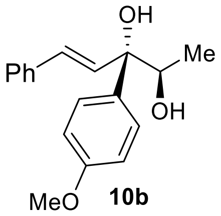

10b

<Chromatogram>

mV

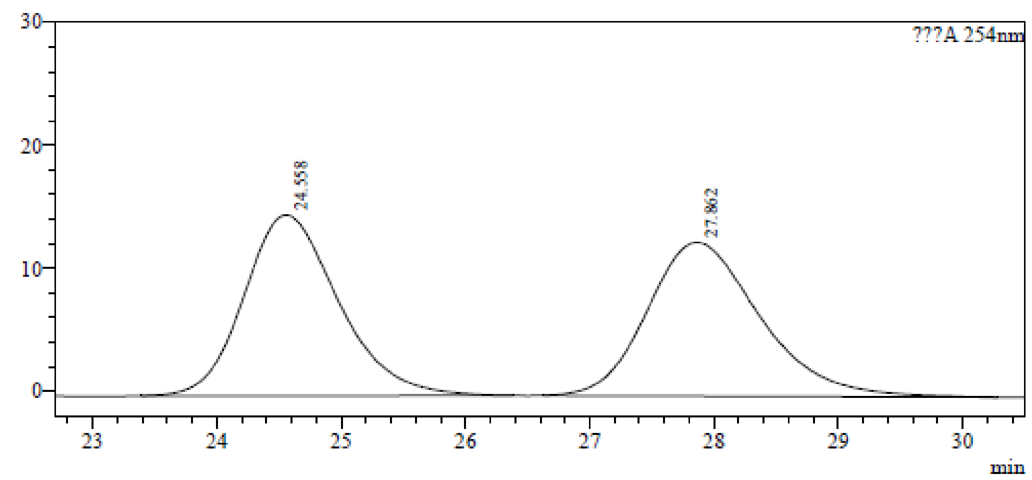

<Peak Table>

???A 254nm

Peak#	Ret. Time	Area	Height	Conc.	Unit	Mark	Name
1	24.558	778347	14692	49.735			
2	27.862	786635	12471	50.265			
Total		1564983	27163				

<Chromatogram>

mV

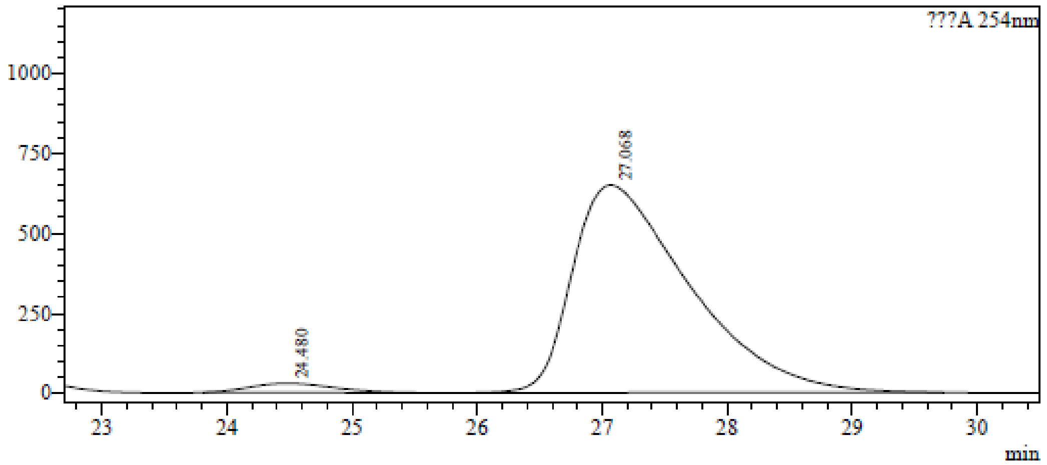

<Peak Table>

???A 254nm

Peak#	Ret. Time	Area	Height	Conc.	Unit	Mark	Name
1	24.480	1315335	28342	3.015		M	
2	27.068	42315678	648663	96.985		M	
Total		43631013	677004				

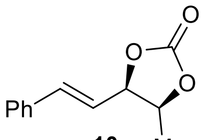

10c

<Chromatogram>

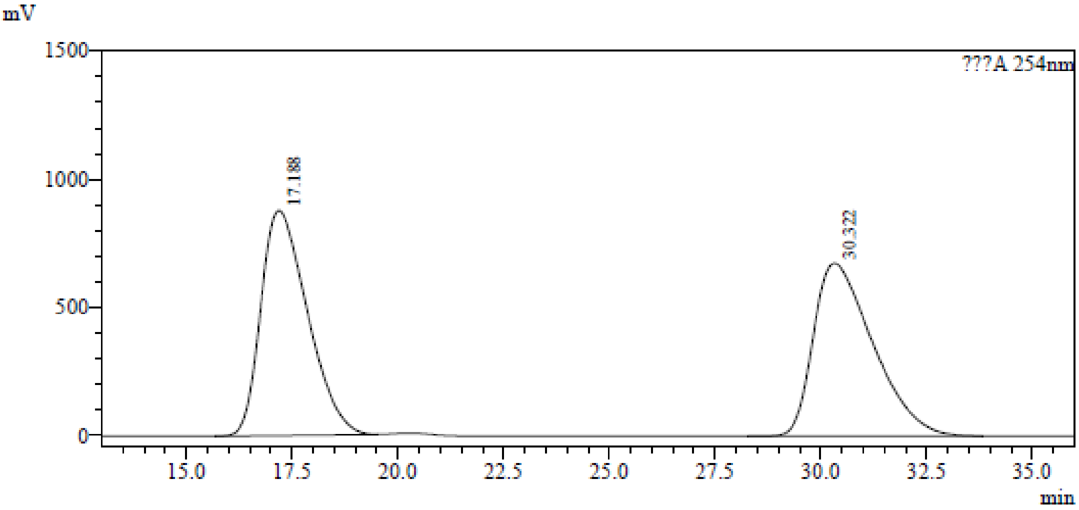

<Peak Table>

???A 254nm

Peak#	Ret. Time	Area	Height	Conc.	Unit	Mark	Name
1	17.188	67512572	878620	50.449			
2	30.322	66309721	674731	49.551		M	
Total		133822292	1553351				

<Chromatogram>

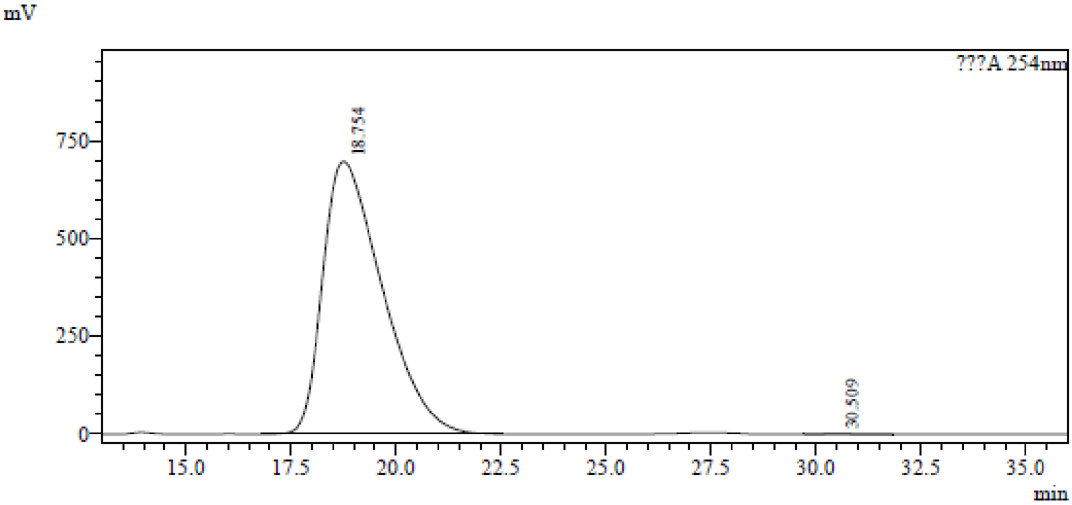

<Peak Table>

???A 254nm

Peak#	Ret. Time	Area	Height	Conc.	Unit	Mark	Name
1	18.754	68684561	696395	99.924		M	
2	30.509	51936	1341	0.076		M	
Total		68736497	697736				

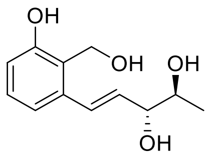

anti-sordariol

<Chromatogram>

mV

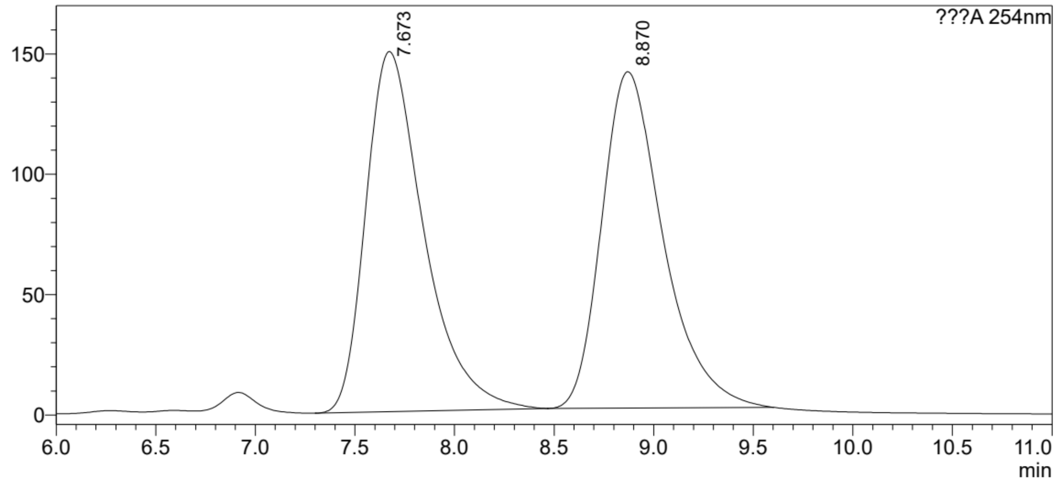

<Peak Table>

???A 254nm

Peak#	Ret. Time	Area	Height	Conc.	Unit	Mark	Name
1	7.673	3037974	149622	50.346			
2	8.870	2996253	139673	49.654		M	
Total		6034226	289295				

<Chromatogram>

mV

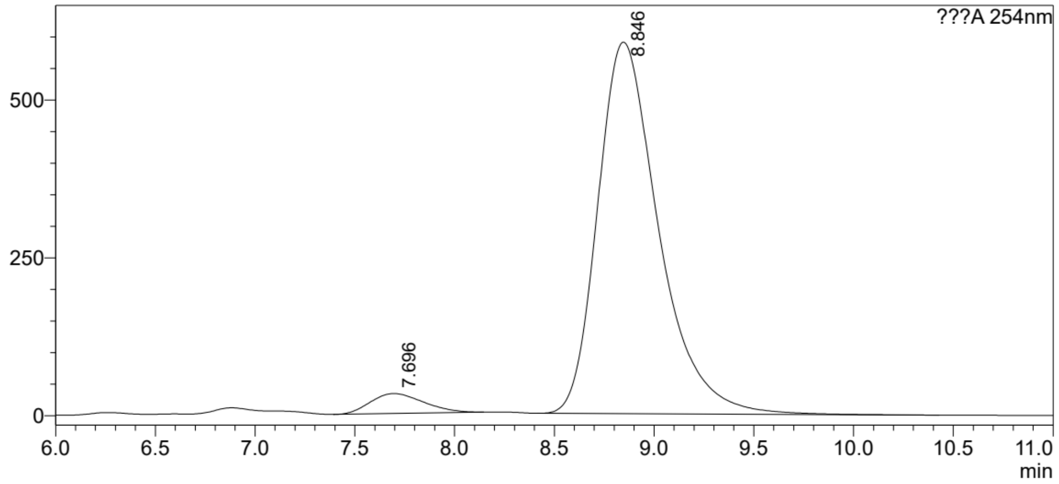

<Peak Table>

???A 254nm

Peak#	Ret. Time	Area	Height	Conc.	Unit	Mark	Name
1	7.696	588080	31567	4.491			
2	8.846	12505951	588665	95.509			
Total		13094031	620232				

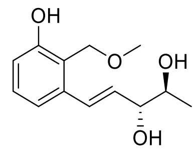

12-methoxy sordariol

<Chromatogram>

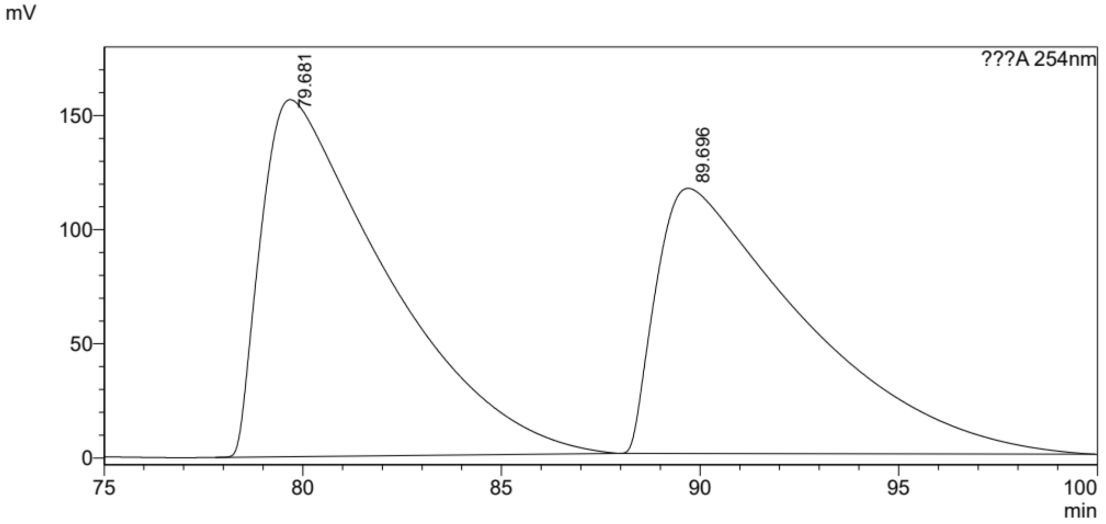

<Peak Table>

???A 254nm

Peak#	Ret. Time	Area	Height	Conc.	Unit	Mark	Name
1	79.681	34319245	156437	53.290			
2	89.696	30081619	116202	46.710		M	
Total		64400864	272639				

<Chromatogram>

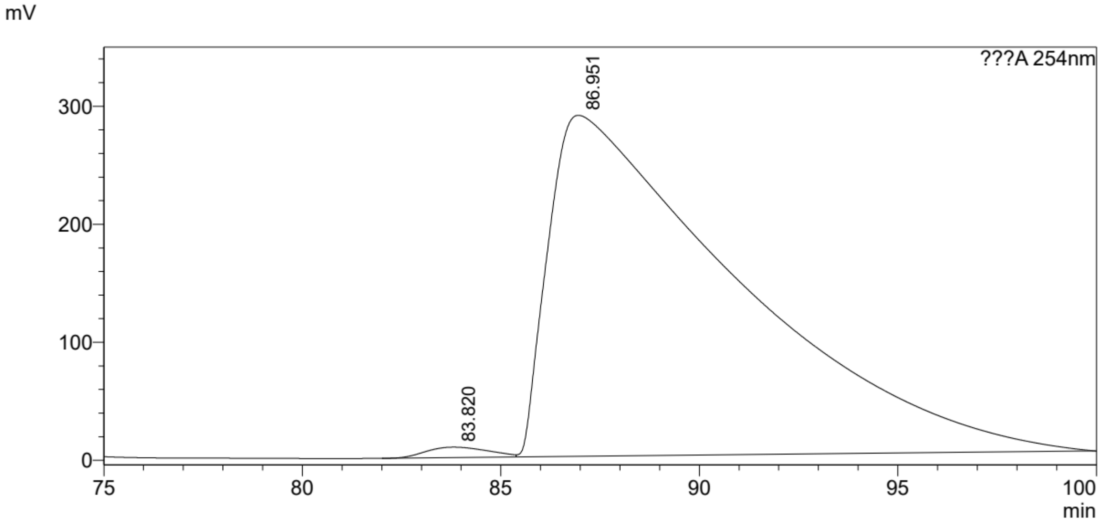

<Peak Table>

???A 254nm

Peak#	Ret. Time	Area	Height	Conc.	Unit	Mark	Name
1	83.820	971285	8891	1.003			
2	86.951	95904082	288924	98.997		V	
Total		96875367	297816				

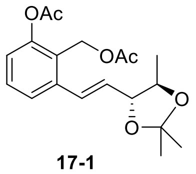

17-1

<Chromatogram>

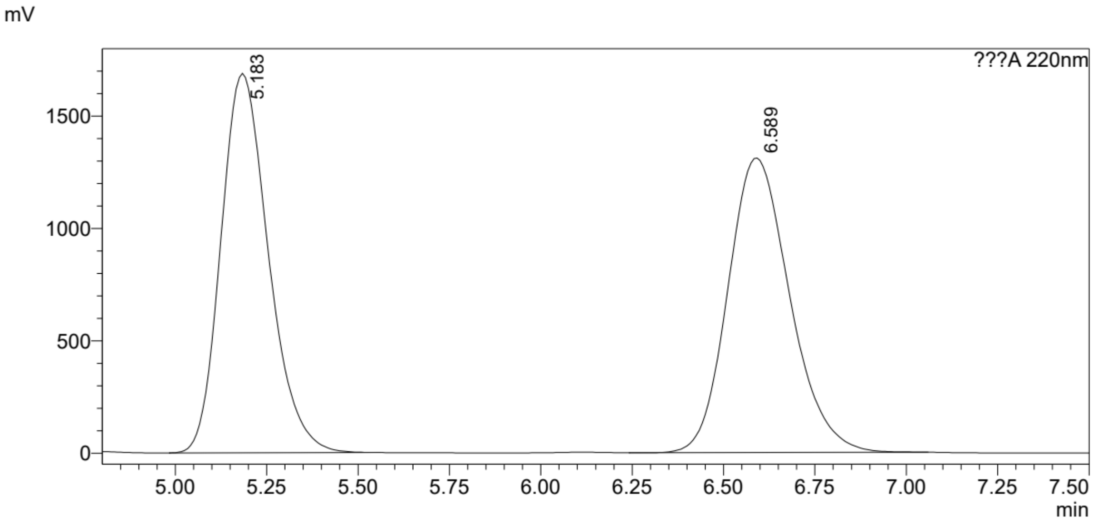

<Peak Table>

???A 220nm

Peak#	Ret. Time	Area	Height	Conc.	Unit	Mark	Name
1	5.183	15259045	1688748	49.727		M	
2	6.589	15426696	1311654	50.273		M	
Total		30685741	3000402				

<Chromatogram>

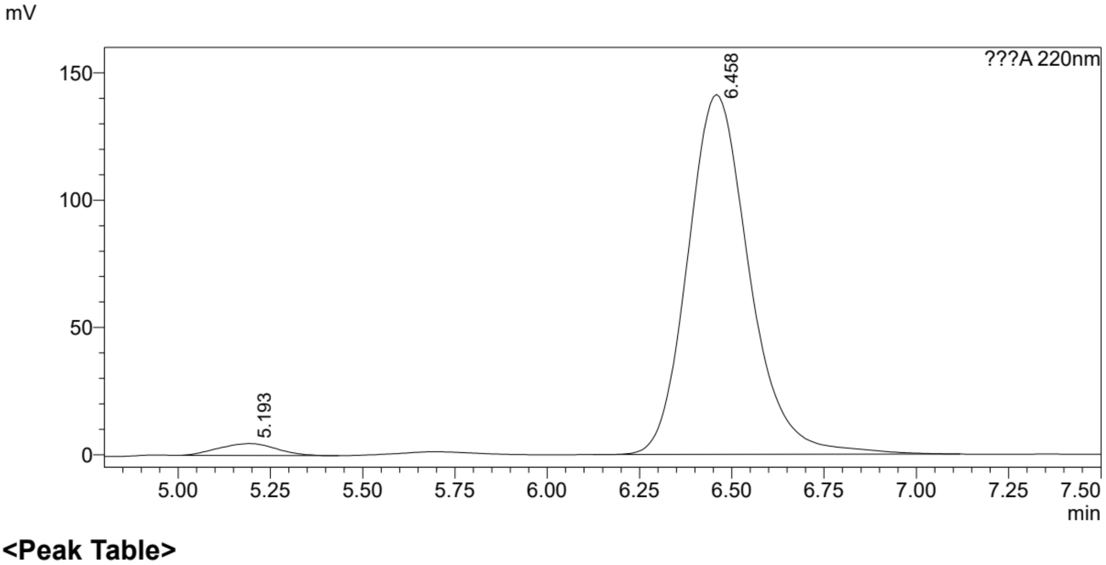

<Peak Table>

???A 220nm

Peak#	Ret. Time	Area	Height	Conc.	Unit	Mark	Name
1	5.193	51487	4708	3.035		M	
2	6.458	1645208	141459	96.965		M	
Total		1696695	146167				

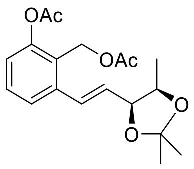

18-1

<Chromatogram>

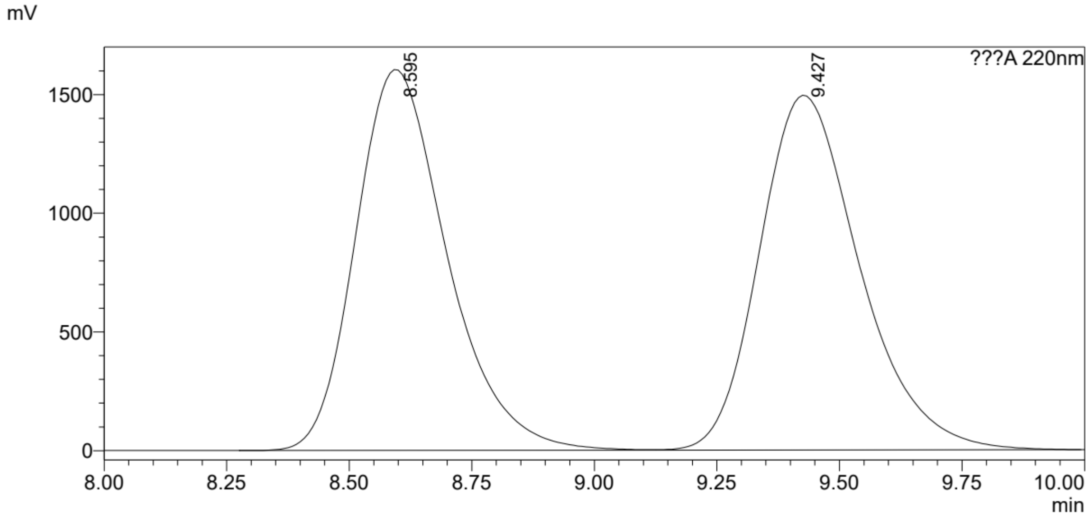

<Peak Table>

???A 220nm

Peak#	Ret. Time	Area	Height	Conc.	Unit	Mark	Name
1	8.595	20848068	1603941	49.528			
2	9.427	21245590	1494043	50.472		V	
Total		42093658	3097984				

<Chromatogram>

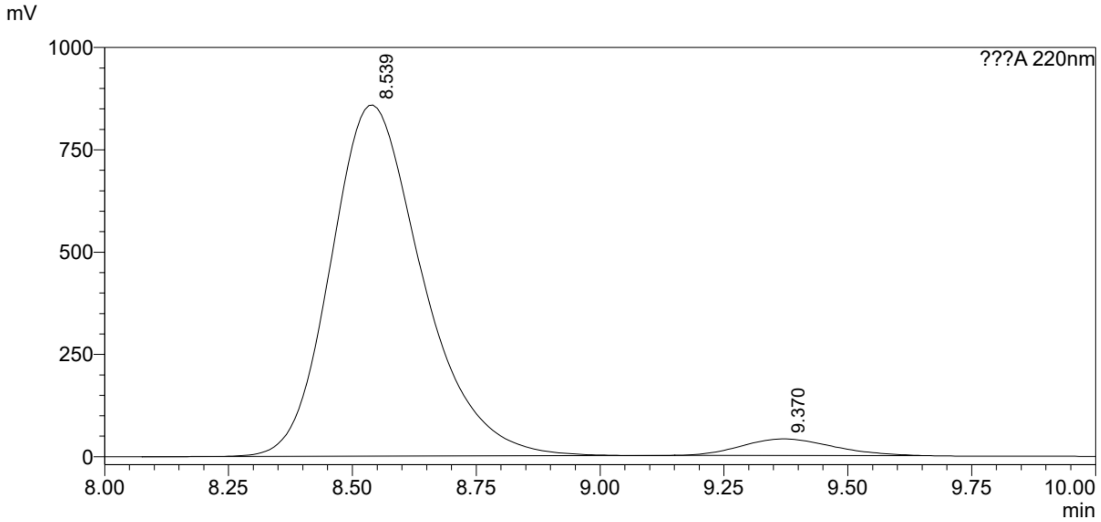

<Peak Table>

???A 220nm

Peak#	Ret. Time	Area	Height	Conc.	Unit	Mark	Name
1	8.539	11032196	858350	95.436		M	
2	9.370	527611	40693	4.564		M	
Total		11559808	899043				

XII. Computational methods

All calculations in this study were performed with the Gaussian 09 program.[13] The B3LYP[14,15]/GEN method (GEN:6-31g* for C, H, O, N, S, LANL2DZ for Ru, Rh) combined with the SMD model[16] were used for geometry optimization in solvent. The B3LYP hybrid functional, corrected with the empirical dispersion Grimme-D3, was used for the solution phase single-point energy calculations. The base set is GEN2 (GEN2: 6-311+g** for C, H, O, N, S, SDD for Ru, Rh).[17] The frequency analysis was conducted at the same level with optimization (zero imaginary frequency for local minima and one for saddle point), to gain the thermodynamic corrections of Gibbs free energy and verify the stationary points to be local minima or saddle points. For transition states, the intrinsic reaction coordinate (IRC) analysis was performed to confirm that they connect the correct reactants and products on the potential energy surface.[18,19] The polarization function was added for Ru (ζ(f) = 1.235) and Rh (ζ(f) = 1.350).[20]

Reference

[13] Frisch, M. J.; Trucks, G. W.; Schlegel, H. B.; Scuseria, G. E.; Robb, M. A.; Cheeseman, J. R.; Scalmani, G.; Barone, V.; Mennucci, B.; Petersson, G. A.; Nakatsuji, H.; Caricato, M.; Li, X.; Hratchian, H. P.; Izmaylov, A. F.; Bloino, J.; Zheng, G.; Sonnenberg, J. L.; Hada, M.; Ehara, M.; Toyota, K.; Fukuda, R.; Hasegawa, J.; Ishida, M.; Nakajima, T.; Honda, Y.; Kitao, O.; Nakai, H.; Vreven, T.; Montgomery, J. A., Jr.; Peralta, J. E.; Ogliaro, F.; Bearpark, M.; Heyd, J. J.; Brothers, E.; Kudin, K. N.; Staroverov, V. N.; Keith, T.; Kobayashi, R.; Normand, J.; Raghavachari, K.; Rendell, A.; Burant, J. C.; Iyengar, S. S.; Tomasi, J.; Cossi, M.; Rega, N.; Millam, J. M.; Klene, M.; Knox, J. E.; Cross, J. B.; Bakken, V.; Adamo, C.; Jaramillo, J.; Gomperts, R.; Stratmann, R. E.; Yazyev, O.; Austin, A. J.; Cammi, R.; Pomelli, C.; Ochterski, J. W.; Martin, R. L.; Morokuma, K.; Zakrzewski, V. G.; Voth, G. A.; Salvador, P.; Dannenberg, J. J.; Dapprich, S.; Daniels, A. D.; Farkas, O.; Foresman, J. B.; Ortiz, J. V.; Cioslowski, J.; Fox, D. J. Gaussian 09, revision D.01; Gaussian, Inc.: Wallingford, CT, 2013.

[14] Becke, A. D. Densityfunctional Thermochemistry. III. The Role of Exact Exchange. *J. Chem. Phys.* **1993**, *98*, 5648–5652.

[15] Lee, C.; Yang, W.; Parr, R. G. Development of the Colic-Salvetti Correlation-energy Formula Into a Functional of the Electron Density. *Phys. Rev. B* **1988**, *37*, 785–789.

[16] Marenich, A.V.; Cramer, C. J.; Truhlar, D. G. Universal Solvation Model Based on Solute Electron Density and a Continuum Model of the Solvent Defined by the Bulk Dielectric Constant and Atomic Surface Tensions. *J. Phys. Chem. B* **2009**, *113*, 6378–6396.

[17] Grimme, S.; Antony, J.; Ehrlich, S.; Krieg, H. A. Consistent and Accurate ab initio Parametrization of Density Functional Dispersion Correction (DFT-D) for the 94 Elements H-Pu. *J. Chem. Phys.* **2010**, *132*, No. 154104.

[18] Fukui, K. A Formulation of the Reaction Coordinate. *J. Phys. Chem.* **1970**, *74*, 4161–4163.

[19] Fukui, K. The Path of Chemical Reactions: the IRC Approach. *Acc. Chem. Res.* **1981**, *14*, 363–368.

[20] Ehlers, A. W.; Bohme, M.; Dapprich, S.; Gobbi, A.; Hollwarth, A.; Jonas, V.; Kohler, K. F.; Stegmann, R.; Veldkamp, A.; Frenking, G. A Set of F-polarization Functions for Pseudo-potential Basis Sets of the Transition Metals Sc-Cu, Y-Ag and La-Au. *Chem. Phys. Lett.* **1993**, *208*, 111–114.

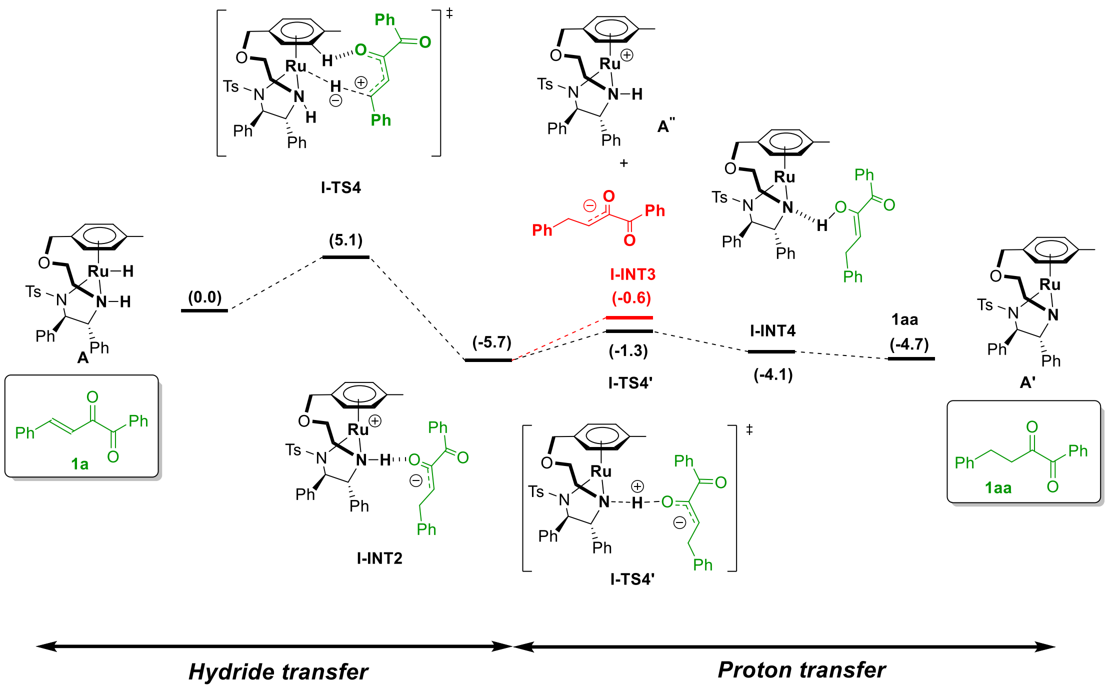

Figure S1. The complete reaction process for the 1,4-addition of **1a**

As shown in Figure S1, substrate **1a** initially undergoes a hydride transfer transition state (**I-TS4**) to form intermediate **I-INT2**, where the substrate part is an enolate anion and the catalyst part is a cation, with the two components interacted through an NH---O=C hydrogen bond. This is followed by a rapid proton transfer transition state **(I-TS4'**), during which the proton from N-H is transferred to O=C, yielding intermediate **I-INT4**. We also investigated the hydrogen bond dissociation in **I-INT2** leading to the formation of catalyst **A''** and enolate anion **I-INT3**. The free energy of **A''+I-INT3** (+0.6 kcal/mol) is higher than that of the proton transfer transition state **I-TS4'** (−1.3 kcal/mol). Therefore, for **I-INT2**, Et_3NHHCO_2 protonation is possible, however the intramolecular proton transfer via **I-TS4'** is more favorable.

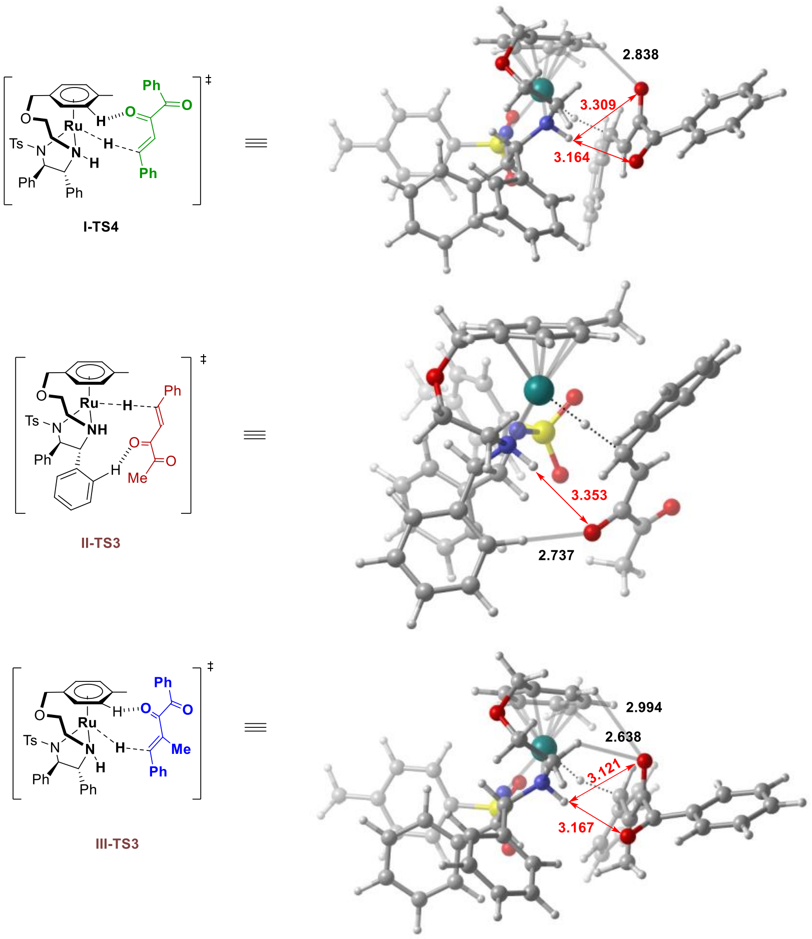

Figure S2. Hydrogen bonding between substrates and catalyst in conjugate reduction transition states.

As shown in Figure S2, theoretically, N-H is indeed a better hydrogen bond donor than C-H. However, in the optimized transition state structures of **I-TS4**, **II-TS3**, and **III-TS3**, the distances between the carbonyl oxygen atom and the N-H bond are significantly large (all bigger than 3 Å). As shown in the following Scheme, in **I-TS4**, the distances between the two O atoms and the NH group are 3.309 Å and 3.164 Å, respectively, while the distance between the O atom and the CH group is only 2.838 Å. This pattern is also observed in **II-TS3** and **III-TS3**. This may be due to the spatial structural factors of both the substrates and the catalyst. If the carbonyl O is forcibly brought closer to the NH bond, other parts of the substrate would encounter significant steric hindrance with the catalyst.

Gibbs Free Energy Corrections and Electronic Energies in Solvate of related compounds and Transition States

	Thermal Correction of Gibbs Free Energies (Hartree)	Electronic Energy (Hartree)
A	0.52477	-2030.579661
1a	0.190301	-767.5947886
I-TS1	0.739426	-2798.190532

I-INT1	0.742629	-2798.200088
I-TS2	0.738212	-2798.198066
2ab	0.21284	-768.8124835
I-TS3	0.739737	-2798.18901
2ac	0.212945	-768.8057779
I-TS4	0.73988	-2798.191144
I-INT2	0.740362	-2798.208814
A’’	0.519728	-2029.84444
I-INT3	0.198181	-768.3338181
I-TS4’	0.736805	-2798.198283
I-INT4	0.737068	-2798.202991
1aa	0.212689	-768.8189034
A’	0.504581	-2029.365163
HCOOH	0.009419	-189.8310715
CO_2	-0.009909	-188.6331121
I-TS5	0.761743	-2799.4193
I-TS6	0.761328	-2799.420336
2a	0.236603	-770.0438552
2a’	0.235468	-770.0399856
1y	0.143138	-575.8042024
II-TS1	0.691654	-2606.402952
II-TS2	0.692098	-2606.398317
II-TS3	0.690073	-2606.393793
5a	0.165664	-577.0256444
5aa	0.162989	-577.0198705
5ab	0.162381	-577.0279471
1zl	0.216943	-806.9210394
III-TS1	0.76692	-2837.514221
III-TS2	0.765599	-2837.514625
III-TS3	0.766005	-2837.513502
B	0.631286	-2317.307409
1zv	0.264667	-998.7144423
IV-TS1	0.920907	-3316.034289
IV-TS2	0.92265	-3316.02662
IV-TS3	0.920987	-3316.038501
7d’	0.287891	-999.9409174
IV-TS4	0.945353	-3317.272036
IV-TS5	0.946896	-3317.270859
7d	0.31049	-1001.161401

Cartesian Coordinates of Intermediates and Transition States

A

Ru -1.89038900 -1.16142000 -0.42342100

N -2.12428700 1.00126600 -0.44600400

N 0.19470500 -0.50191800 -0.48843500

H -2.21081500 1.19221100 -1.44415800

S 1.27755100 -1.35925200 -1.36935500

O	0.79358700	-2.75432600	-1.52072200
O	1.67248700	-0.66934000	-2.63177700
C	2.78274900	-1.52214600	-0.38905900
C	2.71511100	-2.16274600	0.85119900
C	4.00693000	-1.07204500	-0.88648900
C	3.87919700	-2.33503100	1.59850700
C	5.16267400	-1.24910000	-0.12556500
C	5.12002800	-1.88037200	1.12656500
C	0.32258300	0.96677400	-0.69947700
H	0.23077800	1.20138600	-1.76863400
C	-0.86003100	1.65451300	0.01826600
H	-0.78109900	1.44593900	1.08760700
C	1.64439800	1.55363300	-0.20869500
C	2.46712700	2.26473900	-1.09115700
C	2.05270200	1.41286400	1.12633800
C	3.67154000	2.82410600	-0.65493500
H	2.16526900	2.37713300	-2.12955400
C	3.25488800	1.96922500	1.56518200
H	1.43307300	0.85640800	1.82443900
C	4.06964400	2.67683400	0.67520000
H	4.29768000	3.37088500	-1.35563600
H	3.55765400	1.84978000	2.60254400
H	5.00681900	3.10845700	1.01746200
C	-0.85217300	3.16421900	-0.20088500
C	-1.09411700	3.71986100	-1.46711600
C	-0.58116600	4.02800200	0.86947000
C	-1.07155500	5.10344000	-1.65401300
H	-1.30023100	3.07380700	-2.31763700
C	-0.55300700	5.41235500	0.68373800
H	-0.38499900	3.61342000	1.85527100
C	-0.80061700	5.95451300	-0.57907400
H	-1.26305400	5.51606100	-2.64123200
H	-0.33932300	6.06481800	1.52648800
H	-0.78247700	7.03126500	-0.72599600
C	-3.27300600	-1.55681600	1.48055800
C	-3.98060400	-1.78228700	0.25440500
C	-3.50526200	-2.71428400	-0.69147500
C	-2.30378800	-3.45671700	-0.45444500
C	-1.57677100	-3.14582300	0.71972900
C	-2.08259600	-2.25899700	1.72001200
H	-4.89705400	-1.23763900	0.05357900
H	-4.06814000	-2.88409900	-1.60413800
H	-0.62197300	-3.63493800	0.88530000
H	-1.51877600	-2.09053900	2.63208500

C -1.86902100 -4.53809900 -1.40658100
H -2.44863000 -5.45224400 -1.21790900
H -2.04201000 -4.25018300 -2.44872000
H -0.81135400 -4.78060600 -1.28213800
C -3.75601600 -0.51595300 2.47144300
C -3.36655700 1.55889100 0.17253800
H -3.56347200 2.55459300 -0.24564600
H -4.18696400 0.91263200 -0.14174700
C -3.35574000 1.73967500 1.69207000
H -2.69350500 2.56195800 1.97255500
H -4.37728400 2.03097100 1.97968500
O -2.91732600 0.64150000 2.48601100
H 4.05654300 -0.58492900 -1.85400800
H 3.82273200 -2.83204700 2.56381000
H 6.11407500 -0.89280200 -0.51422900
H 1.76539600 -2.51981400 1.23831500
C 6.37948900 -2.06601400 1.93589500
H 7.10218600 -2.69908000 1.40502000
H 6.87590100 -1.10581100 2.12437700
H 6.16940400 -2.53426400 2.90268800
H -1.82431600 -0.96341700 -2.02353200
H -4.79539600 -0.23672500 2.26097500
H -3.71540500 -0.93346000 3.48300800

1a
C 1.69300000 0.71302900 -0.47252500
O 1.46223300 1.88688000 -0.74501500
C 0.52714700 -0.28460700 -0.63695800
O 0.74252000 -1.39740200 -1.11453900
C 3.04126400 0.24095500 -0.06328900
C 3.27669700 -1.04843900 0.44727000
C 4.11033300 1.15226600 -0.15238800
C 4.55943200 -1.41445400 0.85542100
H 2.46420800 -1.76043800 0.52617700
C 5.38965300 0.78017600 0.24772400
H 3.91859300 2.14783000 -0.53981200
C 5.61613800 -0.50573700 0.75305600
H 4.73409500 -2.41060000 1.25215300
H 6.21023400 1.48803400 0.16997200
H 6.61470900 -0.79685800 1.06788400
C -0.79863200 0.22965700 -0.27783200
C -1.89131300 -0.56285600 -0.37856500
H -0.85069200 1.25120200 0.08210200
H -1.73198300 -1.57530500 -0.74898300

C -3.27220100 -0.22961900 -0.04632100
C -4.25791900 -1.22437700 -0.20834900
C -3.66892600 1.03974700 0.42634400
C -5.59405100 -0.96500500 0.09253600
H -3.96321000 -2.20529500 -0.57281900
C -5.00314400 1.29646200 0.72579600
H -2.93192000 1.82624100 0.55707000
C -5.97031900 0.29653300 0.56076200
H -6.33965900 -1.74448700 -0.03771800
H -5.29447600 2.27854300 1.08814400
H -7.01088000 0.50292600 0.79617000

I-TS1
C 1.89348100 -1.71670000 0.49194500
O 1.77895600 -1.19432900 1.64522100
C 0.75855500 -2.68643600 0.05010500
O 0.51522900 -2.90608000 -1.13819700
C 0.03915500 -3.32441100 1.15843100
C -0.98532900 -4.17482000 0.91230900
H -1.24906300 -4.34295500 -0.13148100
C -1.79871100 -4.89640600 1.88417500
C -2.87910500 -5.66762800 1.41008100
C -1.55812300 -4.85850400 3.27425900
C -3.69700100 -6.37238900 2.29121500
H -3.07295000 -5.70475300 0.34093500
C -2.37365300 -5.56584700 4.15159800
H -0.72837700 -4.27852100 3.66681100
C -3.44642100 -6.32329700 3.66464000
H -4.52725800 -6.95915200 1.90768700
H -2.17515700 -5.52962500 5.21938900
H -4.08093600 -6.87325200 4.35457000
Ru 1.29629300 0.91566300 -1.23548300
N 1.58765100 1.50426200 0.82234300
N -0.74485600 0.91243000 -0.47156200
H 1.73712000 0.57962300 1.26368400
S -1.86944700 -0.11709600 -1.06366600
O -1.40914100 -0.62973700 -2.37760500
O -2.27779900 -1.14700200 -0.06617700
C -3.36012100 0.82276400 -1.45140700
C -3.28940900 1.85907600 -2.39043500
C -4.58012800 0.47329400 -0.87582500
C -4.44751000 2.54760300 -2.73776800
C -5.73401100 1.17654300 -1.23286800
C -5.68926000 2.22071400 -2.16418100

C	-0.83834700	1.12650000	0.99963100
H	-0.69121000	0.17525100	1.52677800
C	0.32378300	2.06329700	1.39884400
H	0.16466100	3.02820300	0.91270100
C	-2.16358100	1.72525000	1.46757000
C	-2.94457000	1.04957800	2.41316500
C	-2.61240600	2.96529500	0.98715900
C	-4.14843000	1.59463600	2.86916800
H	-2.61084400	0.08651100	2.79129100
C	-3.81341400	3.51242200	1.43979200
H	-2.02580600	3.50215000	0.24652100
C	-4.58695100	2.82770800	2.38265700
H	-4.74250900	1.05429800	3.60202200
H	-4.14633700	4.47370500	1.05620500
H	-5.52335800	3.25372500	2.73393700
C	0.38508800	2.27704000	2.90925300
C	0.73712300	1.24007200	3.78798100
C	0.05300500	3.52863400	3.44746600
C	0.76007200	1.45588000	5.16777600
H	0.99707700	0.25998600	3.39872500
C	0.07077200	3.74387800	4.82749400
H	-0.22887800	4.33990500	2.78087600
C	0.42592900	2.70704400	5.69288000
H	1.03687000	0.64208800	5.83331700
H	-0.19235100	4.72128700	5.22384100
H	0.44295700	2.87219100	6.76706700
C	2.49578600	2.66184500	-2.17665600
C	3.29419700	1.47848000	-2.18835400
C	2.81021500	0.30555700	-2.80347800
C	1.55459500	0.28164100	-3.48976600
C	0.76525400	1.45121200	-3.42938700
C	1.22991400	2.64594400	-2.80608000
H	4.25718100	1.46867300	-1.68935800
H	3.40873400	-0.59892600	-2.76625400
H	-0.22817000	1.43596200	-3.86480600
H	0.60204600	3.53078100	-2.79232100
C	1.12373200	-0.94430100	-4.24751400
H	1.70748100	-1.02814200	-5.17421400
H	1.29432200	-1.85408800	-3.66410000
H	0.06658300	-0.89612000	-4.51729000
C	2.98085200	3.91896400	-1.47936500
C	2.80615000	2.30810300	1.14001200
H	3.05779800	2.17769100	2.20052800
H	3.62824400	1.88014400	0.56356700

C	2.70206600	3.81712600	0.91238300
H	2.04253100	4.27237900	1.65398100
H	3.70874300	4.23271400	1.06869300
O	2.18593200	4.25101500	-0.34403700
H	-4.63165300	-0.33502800	-0.15495600
H	-4.39063100	3.35336200	-3.46635600
H	-6.68323900	0.90364300	-0.77837800
H	-2.34060900	2.13128700	-2.84326900
C	-6.93373800	2.98118300	-2.54900800
H	-7.81340300	2.60474800	-2.01752400
H	-6.83206700	4.04987800	-2.32047000
H	-7.13018300	2.90420300	-3.62603200
H	1.49120700	-0.64223200	-0.53388500
H	4.03623100	3.81675400	-1.19957400
H	2.89592900	4.76103300	-2.17476900
C	3.29159800	-2.03842400	-0.01246000
C	3.54927600	-2.87709200	-1.10938000
C	4.37688400	-1.50588500	0.70220800
C	4.86390900	-3.16780000	-1.48171800
H	2.72881100	-3.31102800	-1.66852400
C	5.68794300	-1.79082600	0.32385300
H	4.18056800	-0.87834400	1.56550000
C	5.93675100	-2.62395000	-0.77155900
H	5.04754700	-3.82460700	-2.32810600
H	6.51614300	-1.37114100	0.88920200
H	6.95830500	-2.85298400	-1.06356200
H	0.36466600	-3.07830300	2.16248400

I-INT1

C	-3.03437900	-0.28821600	-0.40632000
O	-2.62542100	0.04179600	0.86150200
C	-2.72416200	0.84437900	-1.40879100
O	-2.28512400	0.59363400	-2.54008700
C	-2.94563200	2.21858700	-0.92652400
C	-2.59444500	3.29109900	-1.66915100
H	-2.11539500	3.09584300	-2.62849500
C	-2.77797600	4.70403800	-1.34144400
C	-2.32306500	5.67164100	-2.25898500
C	-3.39462700	5.14680800	-0.15239500
C	-2.47629800	7.03335800	-2.00170700
H	-1.84579400	5.34363700	-3.17935100
C	-3.54746300	6.50629800	0.10266600
H	-3.75728900	4.42474300	0.57309200
C	-3.08981900	7.45515800	-0.81983400

H	-2.11790700	7.76364900	-2.72234700
H	-4.02596000	6.83087700	1.02296900
H	-3.21191100	8.51568000	-0.61587600
Ru	-0.05593200	-2.15507400	-0.43054500
N	-0.53201200	-1.47065600	1.53550900
N	0.93939700	-0.32449900	-0.37082000
H	-1.41365300	-0.88685300	1.28884700
S	1.34242600	0.53837900	-1.72070800
O	0.93785800	-0.24852800	-2.90677700
O	0.80109500	1.91689300	-1.61679700
C	3.13157500	0.68573300	-1.83486000
C	3.91484600	-0.47133800	-1.89437800
C	3.71766400	1.94813800	-1.93003400
C	5.29521000	-0.35305100	-2.03623900
C	5.10223100	2.04804300	-2.07396300
C	5.91285900	0.90555500	-2.12491100
C	0.80738500	0.48092700	0.88511300
H	-0.05871700	1.14454500	0.78617700
C	0.51747700	-0.52318700	2.02159300
H	1.42317200	-1.11283600	2.18336600
C	2.02597900	1.32707000	1.24369300
C	1.89178000	2.71279900	1.39531000
C	3.28262700	0.74414300	1.46852900
C	2.98614900	3.50290600	1.75657900
H	0.92354100	3.17667100	1.22460300
C	4.37834100	1.53069500	1.82706600
H	3.41190900	-0.32834600	1.35099400
C	4.23372200	2.91380500	1.97214100
H	2.86219700	4.57720400	1.86759000
H	5.34576100	1.06333800	1.99291900
H	5.08742100	3.52638000	2.25079700
C	0.17329300	0.18322400	3.33116100
C	-1.01675100	0.91008000	3.49882400
C	1.08786000	0.13664900	4.39351100
C	-1.27948800	1.56767000	4.70277900
H	-1.74138700	0.95513000	2.69122700
C	0.82801000	0.80045700	5.59479800
H	2.01612600	-0.41724100	4.27664300
C	-0.35851200	1.51937700	5.75277700
H	-2.20657300	2.12379000	4.81856300
H	1.55261500	0.75468400	6.40390200
H	-0.56537400	2.03607100	6.68656800
C	0.33916400	-4.29452800	0.02422400
C	-1.01467800	-4.23651400	-0.39611100

C -1.34723900 -3.60710700 -1.61072300
C -0.33957100 -3.18988500 -2.54837400
C 1.00198200 -3.28129800 -2.14805200
C 1.34339500 -3.75640900 -0.84195700
H -1.80274700 -4.56835000 0.27126100
H -2.39266400 -3.46483700 -1.86493600
H 1.78164300 -2.89251500 -2.79246300
H 2.38048600 -3.75806800 -0.52232700
C -0.75560700 -2.63546500 -3.88032400
H -1.22949700 -3.42926100 -4.47228300
H -1.48907900 -1.83161100 -3.75686300
H 0.09883800 -2.24828700 -4.43850200
C 0.71629100 -4.84540300 1.38694500
C -0.93336300 -2.49152300 2.53536000
H -1.46177900 -2.00461200 3.36601000
H -1.65713500 -3.14832000 2.04910300
C 0.21021200 -3.29404200 3.16415100
H 0.78431000 -2.66661200 3.84953800
H -0.24183900 -4.10400400 3.75499800
O 1.18660200 -3.83401400 2.27209700
H 3.10288200 2.84056000 -1.88965700
H 5.90362200 -1.25304200 -2.08157000
H 5.55724900 3.03252000 -2.15067400
H 3.46036200 -1.45501700 -1.82564700
C 7.41130600 1.02375600 -2.24685200
H 7.69872700 1.93107300 -2.78891000
H 7.88216500 1.07534900 -1.25547400
H 7.84122900 0.16030500 -2.76570600
H -2.47056900 -1.15708800 -0.82015900
H -0.13726300 -5.37857500 1.82229100
H 1.53775700 -5.55936400 1.26566500
C -4.53078300 -0.64415200 -0.52474600
C -5.07932000 -1.10519300 -1.73291400
C -5.36945400 -0.51067200 0.58693300
C -6.43501100 -1.42586500 -1.82298600
H -4.44114700 -1.20849200 -2.60830200
C -6.72747800 -0.83153900 0.49986600
H -4.94020700 -0.15793900 1.52000000
C -7.26566400 -1.28906500 -0.70508800
H -6.84433600 -1.78335900 -2.76504100
H -7.36512900 -0.72315900 1.37444400
H -8.32135900 -1.53961500 -0.77388600
H -3.39625600 2.31319000 0.05594200

I-TS2

C	-2.88063000	-0.46979700	-0.36919400
O	-2.45213600	-0.17717400	0.91662600
C	-2.57099900	0.68504300	-1.34366100
O	-2.14093800	0.44728400	-2.48024800
C	-2.79867000	2.04998300	-0.84381100
C	-2.44420800	3.13161800	-1.57266000
H	-1.96472300	2.94686200	-2.53363900
C	-2.62885100	4.54000300	-1.22978400
C	-2.18716400	5.51668300	-2.14427700
C	-3.23577900	4.96980500	-0.03093400
C	-2.34556200	6.87538700	-1.87522700
H	-1.71730900	5.19805900	-3.07166400
C	-3.39316800	6.32636500	0.23590900
H	-3.58651400	4.24040300	0.69308000
C	-2.95005500	7.28444800	-0.68424600
H	-1.99826700	7.61315000	-2.59359900
H	-3.86386200	6.64161300	1.16339100
H	-3.07610700	8.34268300	-0.47113200
Ru	0.10194500	-2.24021800	-0.39230400
N	-0.40106300	-1.51637300	1.51428700
N	1.11861200	-0.40534000	-0.35850400
H	-1.40834300	-0.86122300	1.20074900
S	1.50781000	0.44613700	-1.71416200
O	1.11426700	-0.35331500	-2.89659000
O	0.95756200	1.82371500	-1.62951100
C	3.29664500	0.60826400	-1.82550000
C	4.08870700	-0.54327600	-1.87606600
C	3.87532400	1.87410000	-1.91942600
C	5.46939000	-0.41605400	-2.00719900
C	5.26042000	1.98344800	-2.05191700
C	6.07932700	0.84654100	-2.09298000
C	0.96903700	0.41602200	0.88322300
H	0.11419100	1.09036300	0.75993900
C	0.64113400	-0.57628900	2.01805400
H	1.54628500	-1.16036400	2.21447500
C	2.18908900	1.25511200	1.25354900
C	2.06695600	2.64398000	1.38703500
C	3.43725000	0.66376700	1.50309700
C	3.16344300	3.42807200	1.75538000
H	1.10605500	3.11559800	1.19681800
C	4.53500200	1.44393200	1.86933600
H	3.55768800	-0.41119500	1.39969900
C	4.40192100	2.83002000	1.99662400

H	3.04820200	4.50481100	1.85171700
H	5.49529200	0.96926800	2.05504100
H	5.25729200	3.43766400	2.28097600
C	0.27783200	0.14946900	3.31458400
C	-0.89202600	0.91550300	3.44609600
C	1.15756000	0.08134200	4.40514800
C	-1.17134500	1.58536100	4.63951600
H	-1.59185000	0.97880300	2.61864500
C	0.88205300	0.75606300	5.59701900
H	2.07174400	-0.50090300	4.31886400
C	-0.28641300	1.51066000	5.71853300
H	-2.08390200	2.17042800	4.72483900
H	1.58037700	0.69019300	6.42765200
H	-0.50611800	2.03558700	6.64478800
C	0.46777100	-4.37438400	0.08801700
C	-0.87834100	-4.30446600	-0.35261500
C	-1.18404300	-3.68577800	-1.58168900
C	-0.16052000	-3.29949000	-2.51358800
C	1.17290600	-3.40189700	-2.09159900
C	1.48980200	-3.85984500	-0.77331100
H	-1.68129900	-4.61910700	0.30520600
H	-2.22418100	-3.53695200	-1.85386100
H	1.96792100	-3.03272800	-2.72914800
H	2.52268000	-3.86890500	-0.44013400
C	-0.55249900	-2.76361400	-3.86064900
H	-1.01010500	-3.56569400	-4.45440300
H	-1.29304000	-1.96268200	-3.76078300
H	0.31071000	-2.37766500	-4.40620900
C	0.81691800	-4.90957800	1.46438800
C	-0.83184800	-2.50722000	2.52273500
H	-1.37101500	-1.99882100	3.33534800
H	-1.55719200	-3.16723800	2.04192600
C	0.27990200	-3.32134700	3.20069500
H	0.84476100	-2.69152300	3.89175300
H	-0.20006400	-4.11593500	3.79131200
O	1.27490400	-3.89041500	2.34687500
H	3.25397600	2.76229200	-1.88685100
H	6.08440000	-1.31191200	-2.04548500
H	5.70920800	2.97093100	-2.12715100
H	3.63981100	-1.52976800	-1.80866600
C	7.57812500	0.97497100	-2.20042700
H	7.86465100	1.88225200	-2.74308300
H	8.03856700	1.03388400	-1.20456000
H	8.01959800	0.11275100	-2.71162000

H -2.32542500 -1.33043100 -0.79346500
H -0.04802700 -5.43268900 1.88984400
H 1.63701600 -5.62929900 1.36975400
C -4.37628100 -0.80855000 -0.46081600
C -4.94301300 -1.23185100 -1.67428500
C -5.19790700 -0.70119500 0.66656600
C -6.30136700 -1.54273600 -1.75428300
H -4.31731400 -1.31414900 -2.56055400
C -6.55868000 -1.01221900 0.58774400
H -4.75922600 -0.37845000 1.60547700
C -7.11563400 -1.43284000 -0.62192200
H -6.72473200 -1.87218600 -2.70014100
H -7.18317000 -0.92510500 1.47388500
H -8.17349800 -1.67569300 -0.68307900
H -3.25736000 2.13569700 0.13578100

2ab
C -1.90994200 -0.92054200 1.21653300
C -0.68207200 -1.33245400 0.38616800
O -0.80421400 -2.28176400 -0.39315900
C -2.84092900 -0.09271500 0.33257900
C -2.86073200 1.30431400 0.42084700
C -3.66592100 -0.73229900 -0.60345300
C -3.70239500 2.05118900 -0.40808600
H -2.22504400 1.80547800 1.14438500
C -4.50197900 0.01501500 -1.43391000
H -3.65370600 -1.81667200 -0.67912300
C -4.52368200 1.41003300 -1.33781100
H -3.71318800 3.13522200 -0.32662100
H -5.14152300 -0.49187800 -2.15207200
H -5.17859200 1.99182200 -1.98132000
C 0.55174300 -0.55098000 0.52822000
C 1.64619000 -0.86700900 -0.20240500
H 0.54299400 0.27170100 1.23359600
H 1.55744600 -1.71540200 -0.88048200
C 2.94757100 -0.20779900 -0.19934600
C 3.95024000 -0.70738200 -1.05467400
C 3.25104400 0.90367800 0.61529900
C 5.21324000 -0.11979500 -1.09875800
H 3.72784700 -1.56367100 -1.68680000
C 4.51268500 1.48821900 0.57003700
H 2.49881300 1.31046200 1.28454600
C 5.49786800 0.97981300 -0.28574000
H 5.97327200 -0.51857800 -1.76516500

H 4.73264100 2.34354400 1.20329000
H 6.48132200 1.44114600 -0.31681500
H -2.41693200 -1.85594300 1.48964300
O -1.47345400 -0.22790800 2.37638600
H -2.26157800 0.01242600 2.89413300

I-TS3
C -2.89811700 1.98665700 -0.13717600
O -3.14396500 2.25968400 -1.31018800
C -1.40387200 1.89939000 0.32262700
O -1.13926300 1.56870900 1.53161000
C -0.56143000 2.92260900 -0.37862200
C 0.46294900 3.52631200 0.24831800
C 1.35476900 4.55689800 -0.29282700
C 2.46223800 4.95820800 0.47882800
C 1.16482500 5.16538700 -1.55033200
C 3.35376600 5.92397700 0.01202200
H 2.62207600 4.50004500 1.45211600
C 2.05436500 6.13090900 -2.01491000
H 0.31439200 4.88583900 -2.16596100
C 3.15376500 6.51450900 -1.23783200
H 4.20380800 6.21466700 0.62410500
H 1.89011500 6.58921200 -2.98694000
H 3.84557900 7.26819600 -1.60477900
Ru-0.99831600 -0.91302700 -1.02886800
N -1.15242500 -1.18303800 1.10820300
N 1.08423200 -0.84023900 -0.38727100
H -1.14061500 -0.18969600 1.41823200
S 2.16288200 0.19346100 -1.05450900
O 1.57706100 0.74871800 -2.30007200
O 2.68033200 1.17635200 -0.06268400
C 3.61357600 -0.73451700 -1.60229600
C 3.49154700 -1.64757000 -2.65292700
C 4.85694500 -0.50739900 -1.00829000
C 4.61535000 -2.34467200 -3.09308900
C 5.97241100 -1.21498500 -1.45717500
C 5.87153300 -2.14883100 -2.49883300
C 1.29879700 -1.04759600 1.07136100
H 1.31523700 -0.07933800 1.58579000
C 0.09063400 -1.83309700 1.63016900
H 0.10706300 -2.84671400 1.22225700
C 2.59737000 -1.77587800 1.41030300
C 3.50547800 -1.20329400 2.30937900
C 2.89902400 -3.02767700 0.85316700

C	4.69085700	-1.86239000	2.64770400
H	3.28389600	-0.23143800	2.74386200
C	4.08307500	-3.68711800	1.18632500
H	2.21142600	-3.48446800	0.14617700
C	4.98406400	-3.10610300	2.08477400
H	5.38432200	-1.40118400	3.34651000
H	4.30432300	-4.65539600	0.74413500
H	5.90639600	-3.62017400	2.34318300
C	0.15764900	-1.91594000	3.15306200
C	-0.02800700	-0.78430700	3.96292600
C	0.45669600	-3.13988100	3.76796000
C	0.07355200	-0.88131000	5.35248400
H	-0.25487200	0.17825200	3.51253000
C	0.56616200	-3.23657600	5.15715500
H	0.61299400	-4.02297500	3.15311100
C	0.37240600	-2.10653000	5.95442400
H	-0.07790400	0.00417000	5.96455000
H	0.80102300	-4.19455100	5.61399500
H	0.45326300	-2.17892200	7.03592100
C	-2.48087200	-2.58352500	-1.56051000
C	-3.07100700	-1.33985500	-1.90771800
C	-2.39028900	-0.46005800	-2.78602400
C	-1.16015400	-0.81784800	-3.40232200
C	-0.56711400	-2.04195100	-2.99424200
C	-1.21629100	-2.93754100	-2.10768700
H	-4.01809900	-1.04653700	-1.46818100
H	-2.82201700	0.51306500	-2.99454500
H	0.41341700	-2.30315800	-3.37745700
H	-0.74403800	-3.87331800	-1.83004600
C	-0.54387700	0.05507900	-4.46037600
H	-1.02831100	-0.14711600	-5.42554800
H	-0.68630300	1.11695500	-4.23759700
H	0.52434000	-0.14105500	-4.57409700
C	-3.18346400	-3.54505900	-0.62660000
C	-2.42788300	-1.73966700	1.64974200
H	-2.55254700	-1.40694700	2.68805700
H	-3.23368200	-1.28302200	1.07241200
C	-2.57058500	-3.26030600	1.67362200
H	-1.87014100	-3.70819600	2.38121900
H	-3.58778000	-3.47196300	2.03661300
O	-2.34310800	-3.95039400	0.44668900
H	4.95390300	0.21570700	-0.20633400
H	4.51448600	-3.05131000	-3.91329200
H	6.93779400	-1.03419500	-0.99013600

H	2.53425400	-1.80970500	-3.13615200
C	7.07734300	-2.93367200	-2.95207900
H	7.97413500	-2.30491800	-2.99383800
H	7.29274700	-3.75501900	-2.25494200
H	6.92050000	-3.37522500	-3.94162600
H	-1.11323300	0.77766400	-0.53033000
H	-4.11978000	-3.11180600	-0.25673700
H	-3.43625600	-4.45058500	-1.19159900
C	-4.01545200	1.75808400	0.82500000
C	-3.91216800	1.96521300	2.21263300
C	-5.25604500	1.37063900	0.28044500
C	-5.02750000	1.78011700	3.03124600
H	-2.97060500	2.28434100	2.63974900
C	-6.35877000	1.16636000	1.10420700
H	-5.33879000	1.22997400	-0.79298300
C	-6.24608300	1.37233500	2.48374800
H	-4.94313400	1.95597900	4.10022700
H	-7.30632000	0.85309900	0.67443500
H	-7.10792000	1.21913400	3.12795200
H	-0.84296800	3.16111300	-1.39907700
H	0.67730200	3.21443200	1.26935000

2ac

C	-1.54033300	0.56855400	-0.07833300
O	-1.09453200	1.66443700	0.25145000
C	-0.58286800	-0.51578300	-0.61972100
O	-0.78238600	-1.67898100	0.17754600
C	-2.99996300	0.27915900	0.00297200
C	-3.58127500	-0.88383600	-0.53346700
C	-3.82706600	1.23752000	0.61719300
C	-4.96003300	-1.07762000	-0.45586600
H	-2.96349100	-1.63967100	-1.00437600
C	-5.20145500	1.03697500	0.70027200
H	-3.37566400	2.13667500	1.02501300
C	-5.77109200	-0.12196400	0.16201600
H	-5.40160500	-1.97684500	-0.87645800
H	-5.83023200	1.78145600	1.18095000
H	-6.84468100	-0.27942300	0.22403300
C	0.84519100	-0.03507000	-0.65294900
C	1.83738900	-0.60129300	0.04828300
H	1.02791700	0.80451700	-1.32015500
H	1.58903000	-1.44270300	0.69372300
C	3.25915200	-0.22037200	0.04673400
C	4.16378000	-0.99488000	0.79687600

C 3.76779200 0.88304800 -0.66709200
C 5.52430700 -0.68654200 0.83231400
H 3.78989200 -1.84977500 1.35603000
C 5.12593500 1.19164000 -0.63134100
H 3.09835500 1.50778800 -1.25195200
C 6.01251500 0.40907300 0.11734600
H 6.20129600 -1.30215300 1.41934900
H 5.49572200 2.04868100 -1.18885200
H 7.07121100 0.65361200 0.14234200
H -0.37033200 -2.42928100 -0.28499700
H -0.90392300 -0.70818100 -1.65804200

I-TS4
C 4.71052600 0.58137800 0.43612200
O 4.21204800 1.60879900 0.90360800
C 3.78634900 -0.61111200 0.16181100
O 3.91688600 -1.22614200 -0.92873200
C 2.79758400 -0.86924400 1.14427400
C 1.89065700 -1.93445200 0.98830600
H 2.19616100 -2.69981700 0.27740900
C 1.06899600 -2.45252500 2.11182600
C 0.56136900 -3.76148700 2.03166700
C 0.80252600 -1.69981000 3.26988200
C -0.17783000 -4.30870800 3.07991800
H 0.76092600 -4.35499200 1.14288800
C 0.06080600 -2.24597800 4.31645000
H 1.17261700 -0.68327700 3.36040100
C -0.43071600 -3.55186300 4.22688900
H -0.55441800 -5.32528600 3.00228600
H -0.13322200 -1.65077000 5.20488100
H -1.00677500 -3.97548300 5.04533900
Ru 0.01579800 -0.98914700 -1.47329200
N 0.76157900 0.91741900 -0.77375200
N -1.59965500 -0.34038100 -0.16596100
H 1.33963800 0.61926400 0.02083300
S -2.54539100 -1.41619200 0.62383700
O -2.35022100 -2.76281000 0.03041000
O -2.39134400 -1.33285500 2.10292400
C -4.26797000 -1.00679800 0.28200200
C -4.71921000 -0.98686700 -1.04057800
C -5.14985100 -0.75436800 1.33438600
C -6.05810200 -0.70155900 -1.30362300
C -6.48609100 -0.46744800 1.05466900
C -6.96317700 -0.43626100 -0.26408700

C	-1.27457800	0.87527000	0.62985900
H	-0.71977200	0.59446200	1.53473700
C	-0.34915800	1.76274600	-0.23103800
H	-0.91993700	2.10128900	-1.09795100
C	-2.49020600	1.69381200	1.06581200
C	-2.71467200	1.94791600	2.42453200
C	-3.38424500	2.22812100	0.12518700
C	-3.80693800	2.71495400	2.83929300
H	-2.03236900	1.53603900	3.16398900
C	-4.47685000	2.99316300	0.53579500
H	-3.23463500	2.03520100	-0.93385600
C	-4.69217700	3.23976800	1.89544500
H	-3.96556000	2.89958800	3.89890000
H	-5.16130000	3.39700700	-0.20611400
H	-5.54375500	3.83522100	2.21479300
C	0.15601500	2.97871300	0.54015100
C	1.03371600	2.84090500	1.62675600
C	-0.26140500	4.26591700	0.17380500
C	1.49023100	3.96378800	2.31977400
H	1.36981500	1.85543200	1.93713600
C	0.18942000	5.39045100	0.86955000
H	-0.94753000	4.38796200	-0.66074700
C	1.06944100	5.24224800	1.94377400
H	2.17364900	3.83818000	3.15560200
H	-0.14581000	6.38000800	0.56955700
H	1.42516400	6.11569700	2.48411800
C	0.32858800	-0.56604200	-3.74097100
C	1.40385500	-1.36859700	-3.24920900
C	1.13595000	-2.59447300	-2.60533400
C	-0.19933800	-3.10194500	-2.49140100
C	-1.24771800	-2.26388100	-2.93249500
C	-0.99843300	-1.01808000	-3.58012900
H	2.42559700	-1.01659900	-3.33479400
H	1.96149800	-3.17326900	-2.20314700
H	-2.27367200	-2.58023000	-2.77750000
H	-1.82712100	-0.40962200	-3.92772800
C	-0.45267800	-4.47764100	-1.93929300
H	-0.28760000	-5.22571000	-2.72697500
H	0.23096300	-4.71214000	-1.11730900
H	-1.47961500	-4.58539500	-1.58302600
C	0.59180900	0.77462800	-4.39745700
C	1.70095400	1.66315400	-1.67157800
H	2.29807600	2.35852300	-1.06880200
H	2.39157200	0.92373200	-2.07872200

C 1.06877100 2.49926700 -2.78520500
H 0.54923800 3.36718000 -2.37356700
H 1.90012300 2.87608800 -3.40005100
O 0.10157700 1.86069000 -3.61466200
H -4.79377700 -0.77225100 2.35868800
H -6.40550300 -0.68287700 -2.33372100
H -7.16808700 -0.26432600 1.87713700
H -4.03519700 -1.18105600 -1.86144700
C -8.41441600 -0.14105400 -0.55074600
H -9.05252800 -0.99110300 -0.27388700
H -8.76665300 0.72374400 0.02394700
H -8.57978900 0.06419600 -1.61331600
H 0.79397000 -1.50199700 -0.01682100
H 1.66123400 0.89697900 -4.60669500
H 0.05341800 0.81253000 -5.35052600
C 6.15497500 0.51082000 0.09607600
C 6.79488500 -0.68779500 -0.26855300
C 6.91243400 1.69299900 0.19323800
C 8.16351500 -0.69543500 -0.53600400
H 6.22757800 -1.60807100 -0.34233300
C 8.27627300 1.68201800 -0.07989700
H 6.41405900 2.61282300 0.48339600
C 8.90424000 0.48604700 -0.44662000
H 8.65249100 -1.62480700 -0.81468100
H 8.85204700 2.60062400 -0.00752900
H 2.75526700 -0.21600600 2.00772500
H 9.96996100 0.47584200 -0.65977800

I-INT2
C 4.37807700 -0.31650300 1.28915900
O 4.52897700 -0.41276200 2.51680600
C 3.19564300 -0.95347200 0.59546200
O 2.66409600 -0.31323500 -0.42281000
C 2.79452500 -2.16576800 1.08571100
C 1.67896300 -2.95538900 0.45507200
H 1.64830200 -2.70804500 -0.61930100
C 1.77777800 -4.46095700 0.62263100
C 2.99840000 -5.13606400 0.46427500
C 0.63514000 -5.21999400 0.91764700
C 3.07470200 -6.52432800 0.59634700
H 3.89635100 -4.56589100 0.23664000
C 0.70607000 -6.60943600 1.04874800
H -0.31987000 -4.71364800 1.04563600
C 1.92790300 -7.26799700 0.88989300

H	4.03120800	-7.02642800	0.47020400
H	-0.19294000	-7.17599300	1.28027400
H	1.98668400	-8.34843200	0.99552600
Ru	-0.34739500	0.57672400	-1.88882400
N	0.68672000	1.61882400	-0.31315500
N	-1.36358800	-0.08780400	-0.25461800
H	1.41023700	0.88115300	-0.12485600
S	-2.34049900	-1.43880300	-0.23017500
O	-2.21450000	-2.09719000	-1.54634600
O	-2.02377500	-2.25561000	0.96739200
C	-4.05982900	-0.93867500	-0.09805900
C	-4.62058800	-0.13702100	-1.09974800
C	-4.83687400	-1.41124800	0.95828900
C	-5.96798300	0.19976200	-1.02326900
C	-6.18874200	-1.06468500	1.01639900
C	-6.77511700	-0.25707500	0.03379800
C	-0.98727300	0.45993600	1.09203500
H	-0.31306000	-0.25846500	1.57011100
C	-0.22335300	1.78242700	0.85854100
H	-0.95335200	2.54012700	0.56657800
C	-2.15114000	0.72471000	2.04563400
C	-2.20352000	0.06609400	3.28060300
C	-3.15334600	1.65778200	1.73679500
C	-3.23338700	0.32887900	4.18776900
H	-1.43477600	-0.65992800	3.53253900
C	-4.18441500	1.92023000	2.63963100
H	-3.13912700	2.17655800	0.78216500
C	-4.22772700	1.25628200	3.86925900
H	-3.25785600	-0.19229900	5.14133000
H	-4.95431400	2.64302300	2.38211200
H	-5.03053100	1.46157200	4.57270400
C	0.48839100	2.25945900	2.12020000
C	1.49350200	1.49140100	2.72799600
C	0.14003000	3.48869700	2.69598800
C	2.14033100	1.94805900	3.87739600
H	1.77269600	0.52907600	2.30992500
C	0.78099500	3.94412000	3.85123300
H	-0.64219900	4.09141000	2.24097700
C	1.78593900	3.17622100	4.44320600
H	2.92141900	1.34115600	4.32780100
H	0.49537100	4.89896900	4.28509700
H	2.28887600	3.53055300	5.33929200
C	-0.09813000	2.06541800	-3.53762200
C	1.02141900	1.20092200	-3.59229500

C	0.83762500	-0.19993400	-3.62011400
C	-0.46229200	-0.78116400	-3.82500900
C	-1.57367600	0.07695100	-3.78210200
C	-1.40260000	1.47979900	-3.55689100
H	2.02203100	1.60246100	-3.47680100
H	1.70150600	-0.85074300	-3.53279700
H	-2.57470000	-0.33719300	-3.82344800
H	-2.27349900	2.11815900	-3.44725700
C	-0.58388900	-2.25939800	-4.04951700
H	-0.17292300	-2.49848100	-5.03956300
H	-0.00463800	-2.82298700	-3.31124800
H	-1.62255500	-2.59130600	-4.01507300
C	0.05346200	3.55712500	-3.30537600
C	1.45228100	2.84323500	-0.66726200
H	2.09334700	3.12480800	0.17834600
H	2.12090100	2.57187200	-1.48548200
C	0.62786300	4.08392900	-1.01537500
H	0.10226000	4.45843300	-0.13367200
H	1.34169400	4.85931600	-1.33057800
O	-0.39258700	3.93515400	-2.00403000
H	-4.39569800	-2.03587200	1.72721900
H	-6.40388400	0.82476900	-1.79906100
H	-6.79448800	-1.43194100	1.84079900
H	-4.01774800	0.22426000	-1.92730800
C	-8.23534700	0.11371900	0.09302900
H	-8.77718400	-0.27811100	-0.77732700
H	-8.71320100	-0.28148800	0.99476500
H	-8.36812700	1.20278100	0.08655000
H	0.69854300	-2.63526500	0.84044300
H	1.09247800	3.86467300	-3.47060000
H	-0.57836600	4.09510500	-4.01972100
C	5.38459700	0.45426200	0.48933800
C	5.53889100	0.28858200	-0.89851700
C	6.26022100	1.31630900	1.17191900
C	6.54522500	0.97140100	-1.58185900
H	4.87078200	-0.37264800	-1.43814400
C	7.25369500	2.01105800	0.48577900
H	6.15019500	1.43432500	2.24568600
C	7.39980200	1.83805600	-0.89429500
H	6.66218500	0.82751900	-2.65282200
H	7.91702900	2.68257600	1.02461000
H	3.29331300	-2.57165400	1.96120500
H	8.17792600	2.37487000	-1.43092800

A''

Ru	-1.52075300	-1.38514000	-0.24553400
N	-2.26538300	0.65218300	-0.22884500
N	0.15070900	-0.36411200	-0.77000700
H	-2.53091300	0.76252800	-1.21314500
S	1.47494700	-1.03944500	-1.54076800
O	1.14210900	-2.44895700	-1.82472500
O	1.84552100	-0.18374900	-2.69154100
C	2.87187300	-1.08024400	-0.41735500
C	2.77797400	-1.82094500	0.76697400
C	4.05431600	-0.42835200	-0.76385300
C	3.87994400	-1.88608000	1.61328000
C	5.15095300	-0.50881900	0.09703600
C	5.08330500	-1.23176400	1.29478000
C	0.08570200	1.13581700	-0.81552000
H	-0.09363200	1.43630200	-1.85347600
C	-1.12056800	1.57657600	0.04268600
H	-0.86019100	1.42212500	1.09123900
C	1.33430500	1.86244500	-0.32127600
C	2.01022300	2.74230700	-1.17589900
C	1.79481800	1.71196900	0.99576500
C	3.12533200	3.45592000	-0.72921400
H	1.66173800	2.86827900	-2.19789300
C	2.90979300	2.42176000	1.44336100
H	1.29068500	1.02894900	1.67407500
C	3.57867000	3.29660900	0.58196500
H	3.63751500	4.13484900	-1.40614500
H	3.25647500	2.29145200	2.46527200
H	4.44647400	3.84994300	0.93153900
C	-1.46682100	3.04497900	-0.16868200
C	-1.94034500	3.51449500	-1.40364900
C	-1.30532300	3.95632000	0.88332800
C	-2.24841400	4.86465000	-1.57826400
H	-2.07288200	2.82689400	-2.23624600
C	-1.60740800	5.30905600	0.70813100
H	-0.93498900	3.60551900	1.84335200
C	-2.08124500	5.76622700	-0.52314500
H	-2.61604900	5.21266000	-2.54002800
H	-1.47427600	6.00260700	1.53430200
H	-2.31929500	6.81768500	-0.66112800
C	-2.78136600	-2.25796700	1.38746700
C	-3.50717400	-2.43128900	0.18493400
C	-2.89473000	-3.03151800	-0.93628600
C	-1.61194800	-3.67245300	-0.83510800

C	-0.89392600	-3.51404400	0.36345800
C	-1.44092400	-2.75141700	1.44227500
H	-4.49009300	-1.98542200	0.07838100
H	-3.42546400	-3.05814700	-1.88262400
H	0.11678000	-3.89792400	0.43916600
H	-0.84456200	-2.57003700	2.33099200
C	-1.08665900	-4.45774200	-1.99974000
H	-1.67723700	-5.37905900	-2.09347900
H	-1.19818200	-3.90151900	-2.93610400
H	-0.03855900	-4.72923800	-1.86781100
C	-3.33132400	-1.43516900	2.53763300
C	-3.49515500	0.94198700	0.56561500
H	-3.87623400	1.93372100	0.29322800
H	-4.24739400	0.21582200	0.25487100
C	-3.31539900	0.94267700	2.08495000
H	-2.72020600	1.80294800	2.40099500
H	-4.31848300	1.05527300	2.52124900
O	-2.65505900	-0.18345300	2.66099000
H	4.11820700	0.13832900	-1.68617800
H	3.80884100	-2.45696700	2.53598700
H	6.07307300	0.00100300	-0.16996300
H	1.85963600	-2.33687800	1.02981800
C	6.26719800	-1.31883600	2.22428600
H	6.60802300	-2.35674700	2.33093300
H	7.10855100	-0.72218300	1.85876200
H	6.00710100	-0.96369900	3.22929700
H	-4.41048400	-1.28787900	2.41563900
H	-3.16188100	-1.97408100	3.47545100

I-INT3

C	-1.58116300	-1.01242000	0.42391700
O	-1.52274400	-1.70367000	1.45293500
C	-0.44453200	-0.98377900	-0.58223100
O	-0.72843500	-0.73833700	-1.82582600
C	0.80699000	-1.20057500	-0.04870600
C	2.05410100	-1.13302700	-0.88648000
H	1.76309000	-0.75502900	-1.87821100
C	3.15842500	-0.25754100	-0.31322600
C	2.88646800	1.04768400	0.12828800
C	4.47822100	-0.72219500	-0.22242600
C	3.89976700	1.86291500	0.63521600
H	1.86633400	1.42191900	0.07685100
C	5.49731000	0.08852300	0.28738300
H	4.71034800	-1.73219500	-0.55516700

C	5.21224600	1.38595800	0.71800100
H	3.66530000	2.87146400	0.96831700
H	6.51260000	-0.29643400	0.35020200
H	6.00168000	2.01840600	1.11657100
H	2.47787300	-2.13544100	-1.06438600
C	-2.81870600	-0.19558100	0.18176300
C	-2.85311500	0.91243800	-0.68354000
C	-3.97196400	-0.51273600	0.92085400
C	-4.01193200	1.68129000	-0.79825200
H	-1.97437800	1.15805000	-1.26741600
C	-5.13458500	0.24358600	0.79103600
H	-3.94248300	-1.36196500	1.59695000
C	-5.15604200	1.34675600	-0.06834700
H	-4.02337500	2.54144700	-1.46288400
H	-6.02142400	-0.02172800	1.36093900
H	0.90500500	-1.42245900	1.01015300
H	-6.06006600	1.94224900	-0.16830200

I-TS4’

C	-4.22407600	0.13667700	1.44900500
O	-4.43481200	0.43993600	2.63089400
C	-3.12374300	0.80463900	0.67333100
O	-2.57164500	0.11973000	-0.35225000
C	-2.76907100	2.06416300	1.02377900
C	-1.69762900	2.85978500	0.33460300
H	-1.53692900	2.44099200	-0.66929100
C	-1.97211600	4.34975100	0.23193700
C	-3.23614700	4.83588100	-0.13555200
C	-0.94647400	5.27530000	0.47499700
C	-3.46860100	6.20792500	-0.25633500
H	-4.04407000	4.13302200	-0.32796100
C	-1.17477600	6.64838500	0.35383900
H	0.03999600	4.91368100	0.75915300
C	-2.43824600	7.12012300	-0.01085500
H	-4.45517900	6.56478100	-0.54240700
H	-0.36608400	7.34870000	0.54877400
H	-2.61927200	8.18817200	-0.10222200
Ru	0.21509200	-0.53930200	-1.82190900
N	-0.65693800	-1.50413000	-0.19543300
N	1.33027200	0.25293700	-0.26216900
H	-1.67631600	-0.57993300	-0.11337100
S	2.21458700	1.64167000	-0.35407300
O	2.02524800	2.22021900	-1.70258800
O	1.90214800	2.52370400	0.80071600

C	3.96589600	1.24268000	-0.24829400
C	4.52701500	0.37152400	-1.19009600
C	4.75875600	1.83469500	0.73347200
C	5.88780800	0.08861400	-1.13003500
C	6.12375300	1.53964600	0.77833000
C	6.70949700	0.66537900	-0.14559200
C	0.98728900	-0.21660500	1.11609200
H	0.27586500	0.49282800	1.55369700
C	0.28570500	-1.58132500	0.95081300
H	1.06096000	-2.31451200	0.69663600
C	2.16381700	-0.35603100	2.08035100
C	2.17959500	0.37986400	3.27233700
C	3.21984200	-1.24543800	1.82729100
C	3.22293400	0.23576700	4.19066500
H	1.36931700	1.07407900	3.48127200
C	4.26473500	-1.39045400	2.74131200
H	3.23633600	-1.82053400	0.90558200
C	4.26967300	-0.65030200	3.92721700
H	3.21653900	0.81546000	5.11029100
H	5.07586600	-2.08161300	2.52643300
H	5.08303800	-0.76433500	4.63929100
C	-0.35972600	-2.04084200	2.25869300
C	-1.35511000	-1.28448700	2.89509300
C	0.04806800	-3.24118100	2.85743400
C	-1.94021600	-1.72436700	4.08389200
H	-1.67425100	-0.34043200	2.46722600
C	-0.52956800	-3.68215200	4.05140200
H	0.82800400	-3.83556300	2.38741800
C	-1.53035500	-2.92700800	4.66644200
H	-2.71665300	-1.12427700	4.55207800
H	-0.19777100	-4.61605900	4.49820100
H	-1.98521100	-3.26987700	5.59228600
C	0.02274300	-2.13320600	-3.35750100
C	-1.13127000	-1.32035000	-3.46453600
C	-0.99609600	0.08142600	-3.60793300
C	0.27338200	0.69237000	-3.89377100
C	1.41464100	-0.11469700	-3.78845000
C	1.30317400	-1.49780800	-3.44088100
H	-2.11571800	-1.74901300	-3.31342600
H	-1.88408400	0.70436700	-3.56803200
H	2.39922200	0.33111400	-3.87664200
H	2.20170800	-2.09081400	-3.30347600
C	0.33901600	2.14989700	-4.24718400
H	-0.02870700	2.28740100	-5.27304900

H	-0.29989400	2.74916400	-3.59041000
H	1.35948500	2.53370900	-4.19449400
C	-0.06608600	-3.61520400	-3.04928500
C	-1.37980300	-2.76768700	-0.43609100
H	-1.99265100	-3.01338100	0.44386600
H	-2.08521700	-2.59089100	-1.25126800
C	-0.54282900	-4.02291800	-0.72202700
H	0.02068300	-4.32519000	0.16380800
H	-1.24431400	-4.83685900	-0.96215700
O	0.44153400	-3.92202500	-1.75362100
H	4.31903500	2.50975400	1.45953600
H	6.32190100	-0.59208000	-1.85866500
H	6.73980400	1.99771400	1.54797400
H	3.91195000	-0.08994600	-1.95669900
C	8.18317900	0.34860700	-0.10061400
H	8.69796000	0.73796100	-0.98871300
H	8.66100500	0.78434400	0.78241400
H	8.35546900	-0.73461300	-0.08282200
H	-0.74173900	2.72336800	0.85960900
H	-1.09989400	-3.96359100	-3.16087700
H	0.55444600	-4.16493500	-3.76521100
C	-5.06735300	-0.91482200	0.80202200
C	-5.31351500	-0.94901600	-0.58151500
C	-5.70178300	-1.85379600	1.63367700
C	-6.17775000	-1.90467000	-1.11636800
H	-4.84296700	-0.22294100	-1.23429600
C	-6.54640200	-2.82112200	1.09444400
H	-5.51549300	-1.81881000	2.70312000
C	-6.78823500	-2.84624900	-0.28295800
H	-6.37461800	-1.91463300	-2.18510300
H	-7.01989200	-3.55157800	1.74499100
H	-3.28948100	2.52303400	1.86023700
H	-7.45294400	-3.59557900	-0.70500300

I-INT4

C	3.85511100	1.96265400	-0.07778600
O	4.11446300	2.91283000	-0.82402300
C	2.66827600	2.03634900	0.83394200
O	2.01191100	0.87764100	1.18001600
C	2.31580700	3.22301800	1.36212500
C	1.17361200	3.44887500	2.31614800
H	1.57483900	3.50029100	3.34039200
C	0.42011900	4.73564400	2.01784500
C	0.98464700	5.98225300	2.32807900

C	-0.84432100	4.70383700	1.41418400
C	0.30559900	7.16731600	2.03828300
H	1.96184700	6.02457000	2.80499700
C	-1.52813000	5.88883000	1.12604900
H	-1.30212100	3.74560800	1.18062400
C	-0.95455900	7.12442300	1.43406600
H	0.75820900	8.12383300	2.28837200
H	-2.51043900	5.84429000	0.66192300
H	-1.48566300	8.04617400	1.21024800
Ru	0.42038300	-1.97540700	0.92386100
N	1.13335400	-1.21517000	-0.76400500
N	-1.24573100	-0.77086500	0.30177200
H	1.88046300	0.26346400	0.41111500
S	-2.31360700	-0.11284100	1.35267400
O	-2.17988700	-0.80293000	2.65657300
O	-2.20481500	1.37338600	1.37871500
C	-3.98911300	-0.48926800	0.81069200
C	-4.38643600	-1.82894600	0.72149000
C	-4.89118700	0.53764200	0.53976500
C	-5.69141400	-2.12990300	0.34470200
C	-6.19795800	0.21820800	0.16133200
C	-6.61916100	-1.11297100	0.05662700
C	-0.94366500	0.07797200	-0.88668200
H	-0.49389900	1.01815700	-0.54548100
C	0.11442600	-0.68712900	-1.70243800
H	-0.39050000	-1.53680000	-2.18527400
C	-2.14420400	0.42267100	-1.76352300
C	-2.46282900	1.76322700	-2.01590400
C	-2.92924500	-0.57679900	-2.35947900
C	-3.54076300	2.10236900	-2.83877900
H	-1.86274800	2.54878200	-1.56303000
C	-4.00722100	-0.24222000	-3.18018300
H	-2.70660200	-1.62344000	-2.17021400
C	-4.31770800	1.09980600	-3.42246200
H	-3.77224800	3.14895000	-3.02074800
H	-4.60661700	-1.02968300	-3.63039400
H	-5.15828800	1.35995000	-4.06082300
C	0.69109500	0.18212600	-2.81898400
C	1.37954800	1.37146300	-2.53908300
C	0.53459000	-0.19677400	-4.15912700
C	1.91028600	2.15325700	-3.56628500
H	1.49864500	1.69408100	-1.51085200
C	1.05629900	0.58684400	-5.19267500
H	-0.00352000	-1.11153500	-4.39695000

C	1.75005000	1.76281800	-4.89929200
H	2.44925700	3.06531100	-3.32146500
H	0.92222700	0.27523900	-6.22560400
H	2.16128600	2.37073700	-5.70114900
C	1.36181700	-3.97762600	0.89519200
C	2.20380800	-3.12627100	1.65242000
C	1.67451000	-2.39228300	2.74808000
C	0.36293400	-2.64415400	3.25816200
C	-0.47131300	-3.48331600	2.49807800
C	-0.00893100	-4.09678400	1.29676000
H	3.22699700	-2.95165400	1.33861200
H	2.30839100	-1.67028900	3.25396400
H	-1.51217600	-3.59947900	2.77999200
H	-0.69085500	-4.69711200	0.70293000
C	-0.08663200	-1.99286200	4.53587700
H	0.41555400	-2.47388200	5.38614200
H	0.17904400	-0.93067000	4.55685600
H	-1.16478200	-2.08711900	4.67881100
C	1.84657300	-4.67016400	-0.36502400
C	2.26496100	-1.86489700	-1.42975000
H	2.73528100	-1.17571600	-2.14511100
H	3.02439800	-2.08318700	-0.67625600
C	1.94620400	-3.14204600	-2.22882500
H	1.33085100	-2.90827500	-3.10164600
H	2.90113000	-3.55577900	-2.58914600
O	1.21512500	-4.15758400	-1.53738700
H	-4.57903700	1.57327900	0.61759100
H	-5.99861600	-3.17075600	0.27308000
H	-6.89934200	1.02073800	-0.05299900
H	-3.68542000	-2.62929700	0.93973000
C	-8.03110800	-1.45829200	-0.34601100
H	-8.55448800	-1.98454800	0.46285700
H	-8.60774400	-0.56137300	-0.59304800
H	-8.04325700	-2.12288900	-1.21894900
H	0.48891300	2.59524800	2.28780000
H	2.93778900	-4.59495200	-0.44402300
H	1.58311800	-5.73206500	-0.31171700
C	4.72547800	0.75149000	-0.07817300
C	4.86738200	-0.07319500	1.05127100
C	5.48565200	0.48041700	-1.22950000
C	5.75773700	-1.14752600	1.02527100
H	4.30207200	0.13591500	1.95290900
C	6.35407200	-0.60768200	-1.26057900
H	5.37955100	1.12695000	-2.09538200

C 6.49330400 -1.42225500 -0.13152800
H 5.88011300 -1.76792900 1.90891700
H 6.92658800 -0.81922700 -2.15953600
H 2.92923900 4.08038600 1.09933900
H 7.17768300 -2.26632000 -0.15176900

1aa
C -1.74626900 0.71451500 0.49514500
O -1.52450700 1.87419800 0.83776900
C -0.56948300 -0.27771000 0.60583900
O -0.76662300 -1.38964300 1.07271300
C -3.08188500 0.24865600 0.06171800
C -3.28687400 -0.99994400 -0.55366500
C -4.17340900 1.11931700 0.24312600
C -4.56432600 -1.36612700 -0.97491000
H -2.45715900 -1.67927000 -0.71239600
C -5.44729500 0.74310300 -0.16737400
H -4.00627000 2.08281100 0.71491700
C -5.64406400 -0.50138200 -0.77782600
H -4.71755900 -2.32810700 -1.45569800
H -6.28739200 1.41495000 -0.01547600
H -6.63918500 -0.79456100 -1.10162300
C 0.78278900 0.24304200 0.19888000
C 1.91198300 -0.73912700 0.50132900
H 0.72140100 0.47819800 -0.87500500
H 1.68598200 -1.69948700 0.01926500
C 3.30447600 -0.29472500 0.08599400
C 4.38315400 -1.16591500 0.31887400
C 3.57479300 0.94185300 -0.51516900
C 5.68527100 -0.81530800 -0.03482600
H 4.19481000 -2.13138900 0.78481500
C 4.88083900 1.29729800 -0.87247000
H 2.76978300 1.64392400 -0.71029800
C 5.94091600 0.42293700 -0.63398600
H 6.50168400 -1.50760400 0.15652300
H 5.06400100 2.26296900 -1.33766300
H 6.95499700 0.69963400 -0.91102700
H 0.92688500 1.21028900 0.69684700
H 1.91335100 -0.95170500 1.57889000

A’
Ru-1.98092500 -0.88324600 -0.16923600
N -1.90715000 1.01568100 0.19988100
N 0.18053800 -0.53338400 -0.27174000

S	1.16361200	-1.54135900	-1.10537100
O	0.59995500	-2.91130400	-1.03799000
O	1.47822200	-1.02837100	-2.46987400
C	2.73915200	-1.67755100	-0.24082100
C	2.75337400	-2.17862100	1.06352000
C	3.93312800	-1.36495200	-0.89302000
C	3.97227600	-2.34488800	1.71967800
C	5.14422400	-1.53680700	-0.22319800
C	5.18581600	-2.02632400	1.09109200
C	0.42759800	0.91502700	-0.53860900
H	0.22276900	1.12076400	-1.59689600
C	-0.60664600	1.69925700	0.29234700
H	-0.28209500	1.69202200	1.34402000
C	1.83573300	1.42375700	-0.23925600
C	2.58841500	2.02067000	-1.25894200
C	2.38999400	1.35323800	1.04857600
C	3.86476300	2.53146100	-1.00584000
H	2.17200800	2.08517000	-2.26132400
C	3.66503400	1.85894500	1.30475200
H	1.82782600	0.88973600	1.85456500
C	4.40821000	2.44957200	0.27743500
H	4.43212500	2.99050700	-1.81166600
H	4.07941700	1.79198500	2.30764500
H	5.40125900	2.84363300	0.47823700
C	-0.66101800	3.15928800	-0.15629900
C	-1.11931700	3.51090100	-1.43554500
C	-0.23851600	4.17829100	0.70726500
C	-1.14944500	4.84683700	-1.83933300
H	-1.46047300	2.73792500	-2.11928500
C	-0.26468600	5.51674500	0.30494900
H	0.11563900	3.92204200	1.70320800
C	-0.72062500	5.85513500	-0.97049400
H	-1.50797200	5.10113000	-2.83374900
H	0.06899200	6.29238300	0.98982100
H	-0.74420200	6.89515900	-1.28569300
C	-3.66691200	-1.49482400	1.10946100
C	-4.19000700	-1.23212900	-0.18221500
C	-3.64842500	-1.88739700	-1.32364700
C	-2.70252800	-2.94550500	-1.19552200
C	-2.20174700	-3.21846900	0.09606500
C	-2.62836400	-2.47788700	1.22976700
H	-4.93822300	-0.45838100	-0.31818500
H	-4.00314700	-1.60890100	-2.31149100
H	-1.40267400	-3.94000300	0.21444900

H -2.18408200 -2.67502700 2.20015200
C -2.26790800 -3.72154800 -2.40855400
H -3.03378600 -4.46596200 -2.66504100
H -2.14587900 -3.06533200 -3.27708400
H -1.32783500 -4.24999200 -2.23603800
C -4.11009600 -0.71117700 2.33271800
C -2.99837900 1.69041900 0.89903600
H -2.94013300 2.77766600 0.75792500
H -3.94522100 1.37639700 0.45278900
C -3.04418100 1.46013600 2.42010300
H -2.16164700 1.89365300 2.90001800
H -3.93367900 1.98190500 2.80657300
O -3.05713700 0.09960700 2.85620000
H 3.91659600 -0.98975900 -1.91053600
H 3.98085800 -2.72933900 2.73661800
H 6.07231200 -1.28873100 -0.73332600
H 1.82627600 -2.43261900 1.56907100
C 6.50465800 -2.20760200 1.80069600
H 7.13628700 -2.94017200 1.28179400
H 7.07035400 -1.26811800 1.83558500
H 6.36286000 -2.55544500 2.82881000
H -4.99539800 -0.10699300 2.10089800
H -4.37969200 -1.41226900 3.12976700

HCOOH
C -0.12765200 0.40227200 -0.00003200
O -1.14213400 -0.26405200 0.00001700
O 1.11658500 -0.09132100 0.00000000
H -0.09729000 1.49989200 0.00007300
H 1.06759400 -1.07053800 -0.00001600

CO_2
C 0.00000000 0.00000000 -0.00007300
O 0.00000000 0.00000000 1.16918200
O 0.00000000 0.00000000 -1.16912700

I-TS5
C 2.10769500 -1.73460100 0.20041200
O 1.74753400 -1.43789100 1.37870700
C 1.18909700 -2.72081600 -0.58403900
O 1.14773400 -2.75850400 -1.80369000
C 0.38086200 -3.64894500 0.29244100
C -0.51822300 -4.60858300 -0.49933500
H 0.10792400 -5.26591500 -1.11360400

C	-1.40645300	-5.43311200	0.40831700
C	-1.01407000	-6.70639500	0.84566500
C	-2.63662300	-4.92451700	0.85399900
C	-1.82677800	-7.45343400	1.70270800
H	-0.06523000	-7.11845800	0.50817200
C	-3.45193200	-5.66781100	1.71014200
H	-2.95573800	-3.93796900	0.52398400
C	-3.04972100	-6.93646500	2.13758300
H	-1.50615200	-8.44066100	2.02687300
H	-4.40365700	-5.25787000	2.03978200
H	-3.68547200	-7.51769500	2.80081100
Ru	1.44586300	1.09697900	-1.18954700
N	1.59059500	1.37222400	0.94994800
N	-0.62731000	0.85845400	-0.55128500
H	1.72475100	0.39648300	1.26349600
S	-1.62283700	-0.20335200	-1.29620100
O	-1.03684900	-0.57333400	-2.60797500
O	-2.00412500	-1.34240400	-0.40940100
C	-3.16374000	0.64103200	-1.70334400
C	-3.12832900	1.76490300	-2.53407900
C	-4.38179500	0.13746000	-1.24557600
C	-4.32120700	2.38634100	-2.89685700
C	-5.56825000	0.77297700	-1.61561300
C	-5.55975300	1.90607300	-2.44135500
C	-0.82926300	0.92478200	0.92349300
H	-0.69895800	-0.07131800	1.36338700
C	0.27733500	1.82924100	1.50963100
H	0.12409700	2.84632400	1.14323800
C	-2.20208500	1.44782700	1.34047400
C	-3.02342100	0.67986700	2.17506500
C	-2.65990600	2.70485800	0.91682000
C	-4.27663400	1.15110400	2.57671700
H	-2.68241200	-0.29742700	2.50787500
C	-3.91042400	3.17819300	1.31550500
H	-2.04098900	3.31098200	0.26050400
C	-4.72424400	2.40158700	2.14671500
H	-4.90202500	0.53907600	3.22194100
H	-4.25119800	4.15341500	0.97650600
H	-5.69931400	2.77000800	2.45535500
C	0.23246300	1.84298100	3.03556800
C	0.55116400	0.70541300	3.79460000
C	-0.16969400	3.00638300	3.70734000
C	0.47631900	0.73840300	5.18890600
H	0.86017700	-0.21195900	3.30133900

C	-0.25124800	3.03832000	5.10144600
H	-0.42816000	3.89328100	3.13394000
C	0.07406700	1.90352700	5.84729300
H	0.72991800	-0.15060500	5.76091300
H	-0.56698500	3.94998400	5.60226000
H	0.01540500	1.92586100	6.93244700
C	2.57828400	3.02865300	-1.77061100
C	3.44499000	1.91006700	-1.95773600
C	3.04436300	0.83362100	-2.77766000
C	1.81318100	0.86335000	-3.50445200
C	0.95848200	1.96608100	-3.27656400
C	1.33415300	3.05809500	-2.44333300
H	4.39141100	1.86446900	-1.42950800
H	3.69311300	-0.03134800	-2.86899200
H	-0.01554600	1.98028300	-3.75444200
H	0.65555200	3.89257700	-2.30154500
C	1.47318000	-0.22818200	-4.48250400
H	2.10042900	-0.12711200	-5.37838600
H	1.65791600	-1.21933200	-4.05779100
H	0.42828100	-0.17286400	-4.79563600
C	2.97487200	4.17274000	-0.85664300
C	2.77273000	2.13262300	1.45690500
H	2.97872900	1.83411200	2.49283800
H	3.62900700	1.81784900	0.85755300
C	2.63779100	3.65656800	1.47118900
H	1.95208500	3.97244600	2.25983200
H	3.62994800	4.06574100	1.71305800
O	2.13304300	4.27097000	0.28800700
H	-4.40259700	-0.74039200	-0.60907700
H	-4.29044400	3.25865100	-3.54531800
H	-6.51545200	0.37700700	-1.25698200
H	-2.18234600	2.15674600	-2.89452900
C	-6.84447000	2.60432200	-2.81259500
H	-7.66503300	1.89121400	-2.94865700
H	-7.15184300	3.30434500	-2.02350600
H	-6.73285500	3.18199400	-3.73617800
H	1.72845600	-0.52183700	-0.70632600
H	4.02618600	4.07709800	-0.55972800
H	2.86330000	5.11482000	-1.40474200
C	3.59526700	-1.82220900	-0.09752600
C	4.12169800	-2.30477800	-1.30734700
C	4.48044800	-1.44496900	0.92521200
C	5.50328000	-2.39162000	-1.48943900
H	3.45844800	-2.61385300	-2.10637200

C 5.85971400 -1.52829900 0.73891200
H 4.07421000 -1.09854800 1.86996300
C 6.37670100 -2.00026600 -0.47133300
H 5.89655500 -2.76950200 -2.42958800
H 6.53113700 -1.23443000 1.54164700
H 7.45149400 -2.06978300 -0.61700000
H 1.08387300 -4.19763500 0.93603800
H -0.20551700 -3.02382000 0.97633700
H -1.13615500 -4.02128800 -1.18769900

I-TS6
C -2.51139600 2.45548300 -0.21687800
O -2.51817600 3.01083400 -1.31453500
C -1.13562800 2.14823000 0.44826200
O -1.09923500 1.69154600 1.62948900
C 0.00411700 3.05637900 -0.02027900
C -0.00544400 4.37757300 0.78535200
H -0.93843100 4.92152100 0.59343100
C 1.17677300 5.26179900 0.44976000
C 1.00593100 6.46083500 -0.25598800
C 2.47835500 4.89185000 0.82876900
C 2.09920000 7.27360800 -0.57054900
H 0.00548500 6.76342900 -0.55812200
C 3.57228700 5.70052800 0.51667900
H 2.63592700 3.96191100 1.37148000
C 3.38705700 6.89618100 -0.18493300
H 1.94265800 8.20126400 -1.11588200
H 4.57103900 5.39807900 0.82251000
H 4.23918800 7.52650800 -0.42658800
Ru -0.89383200 -0.59277000 -1.14206700
N -1.26516400 -1.05839200 0.93199000
N 1.12589600 -0.75520300 -0.33752200
H -1.30828300 -0.10850500 1.34128700
S 2.35038300 0.16218400 -0.91073100
O 1.93190300 0.78330500 -2.19201600
O 2.88945600 1.09636100 0.11971000
C 3.72153100 -0.92989900 -1.34214900
C 3.51864400 -1.95528500 -2.27072500
C 4.98571900 -0.71617300 -0.79063200
C 4.58700900 -2.77213600 -2.63483900
C 6.04492000 -1.54500700 -1.16306200
C 5.86584800 -2.58486000 -2.08623500
C 1.18998100 -0.97875400 1.13389900
H 1.17596400 -0.01646100 1.65973300

C	-0.08340400	-1.74939300	1.54308200
H	-0.04228200	-2.74232400	1.09069400
C	2.42973000	-1.74381300	1.59510300
C	3.29683100	-1.17338300	2.53553000
C	2.71797700	-3.02676300	1.10558900
C	4.42863100	-1.86410900	2.97772100
H	3.08759200	-0.17786000	2.91891700
C	3.84810800	-3.71863800	1.54345000
H	2.06250400	-3.48391900	0.36908600
C	4.70856400	-3.13876000	2.48115100
H	5.09099100	-1.40391300	3.70671000
H	4.05831900	-4.71102900	1.15243100
H	5.58962400	-3.67715400	2.82107700
C	-0.18081200	-1.89538700	3.05945100
C	-0.37600500	-0.78251500	3.89343300
C	-0.03951300	-3.15954500	3.64956100
C	-0.43714000	-0.93696200	5.28006400
H	-0.48012300	0.20992100	3.46388800
C	-0.09478700	-3.31412900	5.03679700
H	0.12259100	-4.02955900	3.01806700
C	-0.29676200	-2.20204800	5.85684100
H	-0.59185600	-0.06517200	5.91074400
H	0.01914800	-4.30284200	5.47389500
H	-0.34244500	-2.31930500	6.93645500
C	-2.21494000	-2.28109200	-2.03778900
C	-2.92048700	-1.04235800	-2.12403100
C	-2.33668100	0.06065300	-2.77804400
C	-1.06594400	-0.03939900	-3.42801600
C	-0.36812900	-1.25950600	-3.29521200
C	-0.93558200	-2.38628600	-2.62865900
H	-3.89272400	-0.93919100	-1.65430900
H	-2.86315700	1.00909700	-2.80065200
H	0.63146300	-1.33986400	-3.70866400
H	-0.37568300	-3.31293200	-2.55635200
C	-0.53160000	1.11971400	-4.22299300
H	-1.01055300	1.14062900	-5.21136300
H	-0.75568200	2.07138600	-3.72914400
H	0.54775900	1.04274200	-4.37209800
C	-2.81447700	-3.46378700	-1.30132800
C	-2.57591800	-1.70282900	1.24904200
H	-2.84066400	-1.49173400	2.29319100
H	-3.32584400	-1.21305800	0.62612000
C	-2.63575300	-3.22455800	1.09358100
H	-2.07267800	-3.71173700	1.89207100

H -3.69024000 -3.51628500 1.20826900
O -2.10479400 -3.77949600 -0.10769300
H 5.14112200 0.08632100 -0.07796300
H 4.42541800 -3.56758700 -3.35830900
H 7.02800700 -1.37620700 -0.72980400
H 2.53731700 -2.12567800 -2.70214000
C 7.01241600 -3.48998500 -2.46113500
H 7.95922600 -2.93990900 -2.50026400
H 7.13680300 -4.29462000 -1.72341200
H 6.84867700 -3.96334300 -3.43500800
H -0.86093300 0.99882300 -0.50834200
H -3.87398400 -3.27969800 -1.08660100
H -2.74780700 -4.34955800 -1.94200000
C -3.79910100 2.09089700 0.44550300
C -3.96108200 1.96086000 1.83753900
C -4.92205800 1.92383700 -0.38961300
C -5.21478200 1.65803100 2.37142700
H -3.11540800 2.11337800 2.49432100
C -6.16502100 1.60115000 0.14678800
H -4.80547300 2.04565500 -1.46189500
C -6.31414200 1.46712600 1.53165000
H -5.33150900 1.57293000 3.44840900
H -7.01853200 1.46040600 -0.51077300
H -7.28539800 1.22150700 1.95301300
H 0.94341400 2.53184100 0.16140600
H -0.07403700 3.26289500 -1.09025000
H 0.00706600 4.12421000 1.85332900

2a
C 1.93732800 -1.32157300 -0.13449000
O 2.24112400 -2.24844900 -0.88980600
C 0.68297700 -1.50714700 0.72652700
C 2.73296500 -0.07028200 -0.09226900
C 2.49072400 0.94954800 0.84514800
C 3.77748500 0.08558400 -1.02255900
C 3.27391500 2.10321500 0.84334000
H 1.70092200 0.84977100 1.58226800
C 4.55691700 1.23760300 -1.02107100
H 3.96415500 -0.70584500 -1.74157100
C 4.30444800 2.25006300 -0.08863000
H 3.08065100 2.88639300 1.57083000
H 5.35951200 1.35044800 -1.74459500
H 4.91195300 3.15116700 -0.08764100
C -0.49028300 -0.69685500 0.13134400

C -1.77563300 -0.84101500 0.96947000
H -0.21408100 0.36144400 0.05913900
H -1.57698800 -0.48092700 1.98730800
C -2.93800200 -0.07551000 0.37423400
C -3.79030700 -0.67665500 -0.56449500
C -3.16942800 1.26450100 0.72043300
C -4.84214300 0.03882600 -1.14179000
H -3.62971500 -1.71655500 -0.84175900
C -4.22045000 1.98392400 0.14649600
H -2.52247900 1.74636800 1.45083300
C -5.06068100 1.37318100 -0.78851000
H -5.49316000 -0.44695600 -1.86471200
H -4.38509500 3.02015800 0.43202600
H -5.88033800 1.93072100 -1.23463200
H -0.66794500 -1.05676800 -0.89083600
H -2.02954800 -1.90406800 1.04655300
H 0.87716000 -1.17452600 1.75272000
O 0.36966200 -2.89088900 0.77919400
H 0.79971300 -3.28057800 -0.01010500

2a’
C -1.83220100 -1.31429800 0.72138500
C -0.67255900 -1.27659200 -0.28887900
O -0.86212500 -1.70864300 -1.41783900
C -2.86488700 -0.25515800 0.33941900
C -2.91656400 0.96975400 1.01495600
C -3.75791400 -0.49676400 -0.71394900
C -3.85535400 1.93778200 0.64726400
H -2.22926200 1.15965500 1.83391600
C -4.69049200 0.47325400 -1.08386800
H -3.72215100 -1.44615400 -1.24197000
C -4.74283600 1.69365200 -0.40299000
H -3.89126600 2.88303600 1.18303400
H -5.38095100 0.27331900 -1.89935100
H -5.47319200 2.44662200 -0.68755500
C 0.64443400 -0.68303400 0.14853100
C 1.70097300 -0.67351600 -0.96641600
H 0.44730600 0.33212200 0.52203800
H 1.30985000 -0.11283600 -1.82303800
C 3.01321500 -0.06985900 -0.51347500
C 3.26479200 1.30127100 -0.67073200
C 3.99951700 -0.86280200 0.09251200
C 4.46687300 1.86571300 -0.23706900
H 2.51351700 1.93072400 -1.14352700

C 5.20300200 -0.30286300 0.52783300
H 3.82496500 -1.92952100 0.21810800
C 5.44120100 1.06452300 0.36432000
H 4.64408600 2.92973800 -0.37421400
H 5.95668500 -0.93590500 0.99004500
H 6.37917900 1.50059900 0.69862100
H -2.28865000 -2.30856100 0.61692500
O -1.30271200 -1.13766100 2.02524700
H -2.03475600 -1.23028700 2.65920000
H 0.99457500 -1.24598000 1.02410200
H 1.86155500 -1.70252000 -1.30866100

1y
C 3.37707800 0.51978000 0.00015800
O 3.16835100 1.72163500 0.00073900
C 2.18899100 -0.47321200 -0.00009000
O 2.42800400 -1.67926000 -0.00037100
C 0.84927700 0.11898400 -0.00000700
C -0.25136800 -0.66919300 -0.00014100
H 0.79267600 1.20160100 0.00019700
H -0.08533600 -1.74643900 -0.00021400
C -1.64889700 -0.25137900 -0.00011300
C -2.64622900 -1.24789100 0.00033200
C -2.04994800 1.10194800 -0.00049000
C -3.99813600 -0.90843600 0.00045300
H -2.34807100 -2.29339100 0.00059700
C -3.39988400 1.43843900 -0.00039400
H -1.30284700 1.89009800 -0.00090200
C -4.37859200 0.43603500 0.00009400
H -4.75286300 -1.69011100 0.00081100
H -3.69408200 2.48443700 -0.00070500
H -5.43153300 0.70460000 0.00016600
C 4.75376400 -0.08732400 -0.00017100
H 4.88335800 -0.72931700 0.87908000
H 4.88312000 -0.72904300 -0.87964500
H 5.50838600 0.70206600 -0.00012300

II-TS1
C -1.68488500 -0.29102800 -2.39198100
O -1.65839500 0.97880800 -2.28935400
C -3.02120400 -1.01305500 -2.07557700
O -3.15742500 -2.20503100 -2.36840600
C -4.07929600 -0.20098300 -1.46400600
C -5.27570700 -0.75154300 -1.15207500

H	-3.85632000	0.84592400	-1.29442500
H	-5.40726300	-1.81034700	-1.37576400
C	-6.43105900	-0.09926600	-0.54406800
C	-7.57481100	-0.87886200	-0.27839400
C	-6.45857700	1.27120400	-0.20859000
C	-8.70715000	-0.31389100	0.30552100
H	-7.56551500	-1.93553400	-0.53437900
C	-7.59031400	1.83317500	0.37354000
H	-5.59324700	1.89675800	-0.40600900
C	-8.71798600	1.04403600	0.63278700
H	-9.57942200	-0.93081800	0.50375000
H	-7.59790400	2.89008400	0.62611600
H	-9.59933500	1.48836700	1.08757100
C	-0.85898700	-0.96459300	-3.48184000
H	-0.76639100	-2.04221000	-3.33363300
H	-1.36589700	-0.79054300	-4.44155100
H	0.13465700	-0.51590500	-3.52888500
Ru	-0.52895500	-1.07404900	0.42710200
N	-0.74112600	1.07270600	0.37760000
N	1.43733600	-0.44497300	-0.26657000
H	-1.11627000	1.20441400	-0.58020900
S	2.29791000	-1.36920700	-1.30812100
O	1.70543300	-2.72932800	-1.35586400
O	2.50373900	-0.70017000	-2.62430900
C	3.94979700	-1.60539900	-0.62181300
C	4.09611900	-2.21938000	0.62560700
C	5.07230900	-1.22420000	-1.35835200
C	5.37418700	-2.43800900	1.13591800
C	6.34550300	-1.44824100	-0.83276800
C	6.51944100	-2.05305200	0.42018800
C	1.54959800	1.01540000	-0.53669900
H	1.21190100	1.23706200	-1.55661500
C	0.59652400	1.74437900	0.43508600
H	0.97046200	1.59725300	1.45039400
C	2.96299200	1.57413900	-0.37896300
C	3.56258600	2.27535800	-1.43240800
C	3.67446100	1.42435300	0.82106500
C	4.84527800	2.81350200	-1.29589700
H	3.02222600	2.39676700	-2.36806300
C	4.95575900	1.95946700	0.96056500
H	3.23005400	0.87586300	1.64716300
C	5.54620600	2.65635400	-0.09848600
H	5.29545100	3.35244300	-2.12584600
H	5.49438600	1.83256800	1.89642800

H	6.54496600	3.07179600	0.00959600
C	0.53587500	3.24115800	0.14152700
C	-0.03817400	3.73653800	-1.04074200
C	1.09105400	4.15211300	1.05153400
C	-0.06411600	5.10954300	-1.29488900
H	-0.47019400	3.05284400	-1.76677400
C	1.07129000	5.52518200	0.79466100
H	1.54916200	3.78306400	1.96582300
C	0.48955300	6.00830800	-0.37914400
H	-0.51618700	5.47684900	-2.21275400
H	1.50796300	6.21447500	1.51287400
H	0.46899800	7.07625900	-0.58061600
C	-1.21872100	-1.36888500	2.62300800
C	-2.30292000	-1.69430300	1.75121400
C	-2.13591700	-2.66015600	0.74087400
C	-0.91175000	-3.38816400	0.59366100
C	0.15780000	-3.02946800	1.44202900
C	0.01149000	-2.04516800	2.46582100
H	-3.24661300	-1.16728500	1.84130000
H	-2.95376800	-2.86422800	0.05810500
H	1.12121200	-3.50824000	1.30524300
H	0.85387000	-1.79807800	3.10382900
C	-0.80535900	-4.49205000	-0.42224200
H	-1.24994900	-5.41096600	-0.01627900
H	-1.35260700	-4.23819800	-1.33614600
H	0.23480300	-4.70412400	-0.67887800
C	-1.37500900	-0.29521900	3.68295800
C	-1.75688500	1.67134600	1.29459000
H	-2.07174400	2.64609700	0.89957700
H	-2.63048000	1.01832400	1.26277200
C	-1.30560500	1.92444600	2.73586700
H	-0.62011000	2.77351200	2.77671300
H	-2.20399800	2.19929400	3.30838500
O	-0.60840600	0.87119600	3.39765200
H	4.95311900	-0.75591100	-2.32910700
H	5.48466100	-2.91766700	2.10549300
H	7.21721000	-1.14940400	-1.41020900
H	3.22544100	-2.52020100	1.19999900
C	7.89952000	-2.26360800	0.99212700
H	8.62047600	-2.52820700	0.21067900
H	8.26789900	-1.34805300	1.47488300
H	7.90298600	-3.05557800	1.74828600
H	-0.99795700	-0.88460400	-1.21994900
H	-2.43412500	-0.04103700	3.81104300

H -1.00315900 -0.68387200 4.63689800

II-TS2
C -1.47997400 -0.91625900 -2.99073500
O -1.55462300 -2.13291600 -2.90329800
C -2.11964200 0.00804600 -1.91054100
O -1.81665100 1.24364200 -1.92077800
C -3.50124000 -0.43545900 -1.54258600
C -4.46811000 0.46306800 -1.27807000
H -3.68148100 -1.50582800 -1.55527600
H -4.18737100 1.51571100 -1.30709100
C -5.87420900 0.19986300 -0.95780200
C -6.71880400 1.29663800 -0.69758600
C -6.42885400 -1.09544500 -0.89812500
C -8.06553100 1.11161700 -0.38540200
H -6.30731200 2.30234900 -0.74237500
C -7.77337000 -1.27906200 -0.58614200
H -5.80797900 -1.96324600 -1.10163400
C -8.59858000 -0.17803600 -0.32770200
H -8.69728700 1.97364900 -0.18741800
H -8.18221100 -2.28539600 -0.54538300
H -9.64745900 -0.32735600 -0.08545900
C -0.87368800 -0.21473700 -4.17393900
H -1.67469900 0.29535300 -4.72643700
H -0.16676800 0.55584000 -3.85522800
H -0.38319400 -0.93910300 -4.82791700
Ru-0.73639100 -0.87061300 0.80754400
N -0.74114400 1.28548900 0.71328200
N 1.20205200 -0.46431500 -0.08327200
H -1.18143400 1.43474700 -0.21303900
S 1.84180900 -1.48774800 -1.18977000
O 1.14719400 -2.79462500 -1.09758000
O 1.90953500 -0.87925400 -2.54868600
C 3.54458800 -1.84682900 -0.71830900
C 3.79461600 -2.46959200 0.50779400
C 4.59259500 -1.56141700 -1.59480000
C 5.10390300 -2.79834100 0.85421500
C 5.89804500 -1.89380600 -1.23194400
C 6.17641000 -2.51919800 -0.00776000
C 1.43320400 0.97257700 -0.40404600
H 1.01496100 1.20910500 -1.38972700
C 0.65947100 1.81272500 0.63513500
H 1.10684500 1.63883800 1.61607800
C 2.90514100 1.38117100 -0.40997600

C	3.46077700	1.98097100	-1.54681000
C	3.72043900	1.18922400	0.71588700
C	4.80091100	2.37792200	-1.56484800
H	2.84080700	2.13339700	-2.42691400
C	5.05960500	1.58153100	0.70042100
H	3.31132200	0.71829100	1.60563900
C	5.60512500	2.17735100	-0.44121500
H	5.21479500	2.83991000	-2.45771900
H	5.67811400	1.42182700	1.58014500
H	6.64857100	2.48180600	-0.45342000
C	0.73236100	3.30476300	0.32039800
C	0.09252800	3.85584100	-0.80179800
C	1.48103100	4.15322500	1.14867200
C	0.19452500	5.22108300	-1.07785500
H	-0.49077400	3.22248300	-1.46450300
C	1.58864400	5.51784100	0.86984700
H	1.99082800	3.74113600	2.01608700
C	0.94223000	6.05691200	-0.24428200
H	-0.31040500	5.63116800	-1.94888300
H	2.17604400	6.15696800	1.52418900
H	1.02026700	7.11891000	-0.46235600
C	-1.30332300	-1.03957600	3.04356700
C	-2.46253300	-1.30111800	2.25237300
C	-2.43511500	-2.31102700	1.26941900
C	-1.29160200	-3.15411700	1.09077300
C	-0.14394100	-2.85066800	1.85452100
C	-0.14271200	-1.82080100	2.84094700
H	-3.35134400	-0.69147900	2.37547300
H	-3.31020600	-2.46780300	0.64747100
H	0.76676100	-3.41352900	1.68003300
H	0.75517300	-1.62236900	3.41712900
C	-1.33741900	-4.30504300	0.12266800
H	-2.00044100	-5.08878600	0.51256700
H	-1.72920500	-3.99277400	-0.85056500
H	-0.34678600	-4.74113800	-0.02505400
C	-1.30255200	0.07242000	4.07550900
C	-1.60289500	2.01195400	1.69488800
H	-1.84531400	3.00523600	1.29536800
H	-2.54144100	1.45876300	1.76091400
C	-1.00322700	2.24865100	3.08288600
H	-0.20671200	2.99440500	3.03541700
H	-1.80616700	2.66542400	3.70903300
O	-0.40897800	1.12931100	3.73622600
H	4.39149800	-1.08059000	-2.54580900

H 5.29567100 -3.28111200 1.80942500
H 6.71298600 -1.66600700 -1.91512700
H 2.98093800 -2.69081200 1.19195700
C 7.58949000 -2.90106000 0.35752900
H 7.90329300 -3.80594600 -0.18051300
H 8.29849600 -2.10843100 0.09207800
H 7.68670900 -3.10477300 1.42880600
H -1.36379900 -0.66886800 -0.78926900
H -2.31968800 0.45497600 4.22196800
H -0.95337600 -0.33418300 5.03081700

II-TS3
C 0.02657100 -0.93459300 4.78295600
O -0.18719700 -2.08735300 5.14399700
C 0.93424200 -0.63126400 3.58101100
O 1.13256400 0.58047900 3.29734800
C 1.47647700 -1.74944900 2.90530600
C 2.32412600 -1.59377400 1.79422000
H 1.20014400 -2.73398700 3.26246300
H 2.71219600 -0.58344500 1.66212400
C 3.29192300 -2.65180700 1.39332700
C 4.47484500 -2.27493300 0.73513000
C 3.08417200 -4.01332400 1.67442300
C 5.42646500 -3.22819000 0.37014800
H 4.65202600 -1.22417200 0.51715500
C 4.03285600 -4.96737800 1.30532900
H 2.17911300 -4.33260800 2.18224500
C 5.20716100 -4.57937600 0.65207400
H 6.33906500 -2.91537800 -0.13058600
H 3.85602700 -6.01586400 1.53068600
H 5.94597400 -5.32411200 0.36820100
C -0.57590500 0.25133000 5.48710300
H 0.21130600 0.93700600 5.82142700
H -1.21167400 0.81296700 4.79234800
H -1.16828600 -0.08555900 6.34122100
Ru 0.79223700 -1.27328800 -1.06288500
N 1.59033400 0.74535400 -0.83956200
N -0.85338500 -0.23244600 -0.09437600
H 2.04518200 0.68689800 0.07227900
S -1.89263300 -0.99976300 0.90517800
O -1.69422800 -2.46473100 0.77332100
O -1.85394500 -0.46318900 2.29485400
C -3.57353100 -0.70067800 0.31725700
C -3.91941600 -1.03259700 -0.99521400

C	-4.53428200	-0.18794700	1.19160600
C	-5.23069600	-0.84139800	-1.42905300
C	-5.84120600	-0.00059900	0.74232500
C	-6.21277700	-0.32317400	-0.57152300
C	-0.55041700	1.16881000	0.30699600
H	-0.07647600	1.17151900	1.29551200
C	0.47469200	1.73632500	-0.69963800
H	-0.00228300	1.80099200	-1.67968900
C	-1.76589000	2.09185100	0.37634500
C	-2.06915100	2.74686200	1.57686900
C	-2.58037600	2.32416900	-0.74188900
C	-3.16423600	3.61019600	1.66480300
H	-1.44117700	2.57506800	2.44792000
C	-3.67641900	3.18438500	-0.65706700
H	-2.36526900	1.82221200	-1.68153400
C	-3.97314400	3.82951500	0.54755800
H	-3.38512100	4.10826400	2.60565000
H	-4.29954500	3.35153300	-1.53213100
H	-4.82751700	4.49846000	0.61338200
C	0.95400000	3.12460600	-0.28482400
C	1.71251400	3.32066000	0.88029200
C	0.61153800	4.24216900	-1.05881900
C	2.12544100	4.60180900	1.25178300
H	1.97518800	2.47554900	1.51178700
C	1.01904500	5.52510100	-0.68537000
H	0.01431800	4.10717500	-1.95727100
C	1.78169600	5.70793900	0.46998000
H	2.71274700	4.73524000	2.15666900
H	0.74218100	6.37892400	-1.29850700
H	2.10283400	6.70444400	0.76202800
C	1.28194900	-1.47097300	-3.33428800
C	2.24090200	-2.20944500	-2.57653600
C	1.81685300	-3.21327000	-1.68308300
C	0.43501900	-3.55483600	-1.54362500
C	-0.49808500	-2.77245600	-2.26128800
C	-0.09092900	-1.76514400	-3.18303800
H	3.29701400	-1.97857900	-2.66896400
H	2.55532600	-3.74991200	-1.09781800
H	-1.55684900	-2.95697600	-2.11483800
H	-0.83418300	-1.20165100	-3.73767300
C	0.01553100	-4.72097400	-0.69135500
H	0.20418300	-5.65794300	-1.23302300
H	0.58623800	-4.76088600	0.24155000
H	-1.04935000	-4.67979600	-0.45150000

C 1.72377700 -0.37656600 -4.28546000
C 2.66789600 1.16268500 -1.79137500
H 3.25636300 1.96959800 -1.33650100
H 3.33354400 0.30524600 -1.90549400
C 2.21123500 1.69116900 -3.15254000
H 1.73797200 2.66910600 -3.04578200
H 3.12161300 1.83040300 -3.75456300
O 1.26489000 0.90906500 -3.87739500
H -4.26127800 0.06814400 2.20937200
H -5.49384700 -1.09783400 -2.45233400
H -6.58414300 0.40341900 1.42634700
H -3.17638400 -1.42361800 -1.68297900
C -7.63198600 -0.11961900 -1.04063500
H -8.32785200 -0.76296400 -0.48666100
H -7.95944300 0.91512300 -0.88027800
H -7.73886900 -0.34805600 -2.10581400
H 1.46744900 -1.48273800 0.54752400
H 2.81440700 -0.38366900 -4.39766100
H 1.28264400 -0.56679400 -5.27012000

5a
C 3.31460400 0.50783300 -0.30259400
O 4.49857500 -0.25897500 -0.45135500
C 2.12139900 -0.43366500 -0.13671700
O 2.35222500 -1.63895200 0.02769200
C 0.77521600 0.13774700 -0.17508000
C -0.31542400 -0.64050100 0.01569900
H 0.69584000 1.20325700 -0.36951000
H -0.13900900 -1.69808200 0.21003500
C -1.71699200 -0.23640400 -0.00421100
C -2.69811800 -1.21466600 0.25414100
C -2.13692400 1.08417100 -0.27043900
C -4.05295700 -0.88794900 0.25242100
H -2.38625100 -2.23598400 0.45853000
C -3.49000200 1.40723300 -0.27404900
H -1.40377500 1.85832600 -0.47662900
C -4.45248800 0.42430800 -0.01138800
H -4.79501600 -1.65537000 0.45507900
H -3.79900900 2.42812900 -0.48171400
H -5.50808700 0.68259300 -0.01485800
C 3.43699100 1.45363700 0.90083100
H 2.56576000 2.11125400 0.98976000
H 3.54157200 0.87831700 1.82790100
H 4.32736200 2.07904000 0.77625700

H	3.15564600	1.10392200	-1.21363600
H	4.22673400	-1.18245000	-0.26360200

5aa

C	3.18203400	0.47969800	0.23955000
O	4.19851400	-0.01227200	0.71669700
C	2.26718300	-0.42051300	-0.60690800
O	2.82206200	-1.72605300	-0.65902400
C	0.86621500	-0.46890700	-0.05451200
C	-0.20175200	0.00677200	-0.71481600
H	0.77578700	-0.94158800	0.92262000
H	-0.03818400	0.44497700	-1.70012200
C	-1.60436900	-0.00200500	-0.27186600
C	-2.59212500	0.46945600	-1.15674300
C	-2.01395900	-0.46440300	0.99471700
C	-3.94032100	0.47408500	-0.79727500
H	-2.29402100	0.83236800	-2.13788900
C	-3.36008200	-0.46039400	1.35297700
H	-1.27729900	-0.82758500	1.70572800
C	-4.33055200	0.00777000	0.45977300
H	-4.68433700	0.84202200	-1.49926200
H	-3.65508600	-0.82198200	2.33486100
H	-5.37957400	0.00962000	0.74394100
C	2.79864300	1.91444500	0.44995900
H	2.37506300	2.35173200	-0.46021700
H	2.02478600	1.96749900	1.22716000
H	3.66861600	2.48819900	0.77990500
H	2.24612900	0.02182700	-1.61565600
H	3.64802900	-1.67650900	-0.13160000

5ab

C	3.40792700	0.52937800	0.00719000
O	3.19449900	1.73099500	-0.02765100
C	2.20645100	-0.44588700	-0.01763300
O	2.42277000	-1.64747900	-0.02902200
C	0.83272200	0.16871800	-0.02497400
C	-0.28060400	-0.87600200	-0.04106000
H	0.76516400	0.82794700	0.85200600
H	-0.14531100	-1.55032700	0.81504600
C	-1.69855200	-0.32989600	-0.01703400
C	-2.76825100	-1.24175300	0.00929700
C	-1.99973000	1.03876600	-0.01901100
C	-4.09149800	-0.80362100	0.03323600
H	-2.55598300	-2.30922900	0.01210600

C	-3.32721900	1.48300400	0.00552800
H	-1.20126700	1.77470600	-0.03846600
C	-4.37772200	0.56588400	0.03149200
H	-4.90033300	-1.53013300	0.05387300
H	-3.53447000	2.55048300	0.00426600
H	-5.40831100	0.91112600	0.05090000
C	4.77679200	-0.07899600	0.07448500
H	4.86495000	-0.70616800	0.97014600
H	4.94103600	-0.73569900	-0.78847400
H	5.53183400	0.71006200	0.09284700
H	-0.15705800	-1.51124100	-0.92853900
H	0.77969400	0.84277000	-0.89141900

1zl

C	1.84305600	0.74340300	0.68696500
O	1.78436600	1.88148200	1.14043300
C	0.57791100	-0.12006000	0.83590700
O	0.67049700	-1.07598900	1.60309000
C	3.08292100	0.15058000	0.12939900
C	3.10124400	-1.13638200	-0.43545100
C	4.25932800	0.92126900	0.13883000
C	4.28196600	-1.64391800	-0.97707500
H	2.20046000	-1.74101200	-0.44856600
C	5.43613600	0.41017600	-0.39958800
H	4.23179700	1.91639900	0.57174100
C	5.44848800	-0.87398300	-0.95801400
H	4.29269100	-2.63936700	-1.41164900
H	6.34363500	1.00731000	-0.38779100
H	6.36753800	-1.27293600	-1.37883200
C	-0.67623400	0.32289400	0.17113900
C	-1.79304200	-0.36188000	0.53262500
H	-1.62108500	-1.17449600	1.23726000
C	-3.18842600	-0.21889200	0.12205200
C	-4.04725700	-1.31445200	0.36381700
C	-3.74286700	0.94104300	-0.46194500
C	-5.39131500	-1.27236500	0.00285800
H	-3.64126100	-2.20688000	0.83378700
C	-5.09205800	0.98460300	-0.81101800
H	-3.13221500	1.82240800	-0.61526800
C	-5.91906200	-0.12089300	-0.58967900
H	-6.02867200	-2.13212700	0.19088700
H	-5.50141900	1.88936500	-1.25232100
H	-6.96912700	-0.08085300	-0.86636300
C	-0.59362000	1.42560700	-0.85541300

H -1.31507400 1.26637300 -1.66193000
H -0.79027500 2.41385800 -0.42077900
H 0.40107100 1.46750800 -1.31081900

III-TS1
C 2.13281900 -1.65264400 0.39761700
O 1.78668000 -1.25984900 1.55338400
C 1.21576000 -2.70584100 -0.29734900
O 1.00729000 -2.65533800 -1.50708600
C 0.61810400 -3.75876700 0.58055800
C -0.46745900 -4.38447000 0.05446300
H -0.73833700 -4.08163200 -0.95489900
C -1.33178500 -5.42828500 0.60708700
C -2.08168400 -6.19729300 -0.30964200
C -1.49494900 -5.69408100 1.98320500
C -2.92439300 -7.21747700 0.12335600
H -1.98768600 -5.98966300 -1.37277100
C -2.35184100 -6.70605200 2.41460500
H -0.98330200 -5.08907700 2.72186000
C -3.06078800 -7.47815900 1.49015100
H -3.48121700 -7.80330300 -0.60323900
H -2.47041600 -6.88841400 3.47952200
H -3.72366900 -8.26842400 1.83252100
Ru 1.46749300 1.07396600 -1.17053700
N 1.57702100 1.48750600 0.94387000
N -0.62076400 0.88034700 -0.56028200
H 1.74212000 0.53730400 1.31913600
S -1.62080400 -0.18913000 -1.29183900
O -1.04831800 -0.56111500 -2.60884400
O -1.99642800 -1.32136900 -0.39723600
C -3.16149000 0.65502200 -1.70216700
C -3.12453800 1.77428100 -2.53915400
C -4.38058700 0.14739800 -1.25241600
C -4.31730700 2.38989400 -2.91235000
C -5.56706200 0.77658400 -1.63339500
C -5.55714400 1.90673600 -2.46315900
C -0.82768300 0.96947300 0.91302600
H -0.66108500 -0.01189200 1.37444400
C 0.24524200 1.93075900 1.47031700
H 0.06441200 2.92364500 1.05396800
C -2.21690900 1.45215300 1.32522700
C -3.01488300 0.66112500 2.16101800
C -2.71181400 2.69521400 0.90210400
C -4.28150700 1.09473000 2.56262800

H	-2.64416400	-0.30501700	2.49458500
C	-3.97556600	3.13163200	1.30145500
H	-2.11210300	3.32037200	0.24586600
C	-4.76624200	2.33134800	2.13251700
H	-4.88811400	0.46446800	3.20826400
H	-4.34483800	4.09659300	0.96296200
H	-5.75170900	2.67068400	2.44154200
C	0.18596600	2.01947500	2.99336600
C	0.50175800	0.92349000	3.81268100
C	-0.22918900	3.21291900	3.60188800
C	0.40976100	1.02639600	5.20250200
H	0.82047400	-0.01576400	3.36972300
C	-0.32539900	3.31532800	4.99164200
H	-0.48730100	4.06796200	2.98172500
C	-0.00415400	2.22118400	5.79751400
H	0.65958900	0.16812500	5.82126700
H	-0.65118000	4.24969600	5.44156900
H	-0.07583400	2.29745300	6.87940100
C	2.58216200	2.98144900	-1.86928400
C	3.47332800	1.86903300	-1.94881900
C	3.11541100	0.72724400	-2.69558500
C	1.90444100	0.67670200	-3.45618400
C	1.02259900	1.77449500	-3.33230700
C	1.35743800	2.93380200	-2.57439900
H	4.40665900	1.88196200	-1.39579900
H	3.78287900	-0.12852300	-2.70509800
H	0.05899800	1.73185400	-3.82879700
H	0.65896400	3.76175100	-2.51187700
C	1.61388900	-0.48989600	-4.36043200
H	2.19874600	-0.39250800	-5.28519200
H	1.89064000	-1.43957200	-3.89323000
H	0.55698900	-0.52997200	-4.63309200
C	2.93326300	4.19923300	-1.03608700
C	2.72605200	2.31901300	1.41188800
H	2.92296700	2.10194100	2.46989000
H	3.60092800	1.98588500	0.85072500
C	2.55112000	3.83637600	1.31551100
H	1.83771000	4.18764500	2.06370900
H	3.52680500	4.28502000	1.55480000
O	2.06473000	4.35907400	0.08140300
H	-4.40179600	-0.72834700	-0.61284100
H	-4.28569800	3.25920600	-3.56483700
H	-6.51534500	0.37782000	-1.28067700
H	-2.17719200	2.16637000	-2.89697900

C -6.84212500 2.59853200 -2.84570800
H -7.65835900 1.88141900 -2.98702800
H -7.15917000 3.29849800 -2.06041300
H -6.72601000 3.17503100 -3.76948800
H 1.74670400 -0.51333600 -0.57704700
H 3.97959900 4.15038300 -0.71166600
H 2.81044500 5.09635300 -1.65255500
C 1.25497100 -4.08448100 1.91083100
H 2.32071500 -3.83918300 1.91247200
H 0.79858600 -3.53738000 2.74416200
H 1.16306700 -5.15459700 2.12249800
C 3.61231500 -1.76552000 0.07648100
C 4.09618000 -2.40244100 -1.07812400
C 4.52924800 -1.25378900 1.00826500
C 5.47072400 -2.51053600 -1.29834900
H 3.40478500 -2.81679200 -1.80379300
C 5.90129200 -1.35588900 0.78183600
H 4.15516200 -0.78831000 1.91448600
C 6.37658600 -1.98383800 -0.37387500
H 5.83330100 -3.00974900 -2.19310100
H 6.60048100 -0.95541600 1.51148300
H 7.44590500 -2.06869600 -0.54885100

III-TS2
C -3.18973100 1.73901300 0.15588600
O -3.72307800 1.01875800 -0.67994400
C -1.67339800 1.68196800 0.50698300
O -1.45267500 1.18553200 1.65805400
C -0.83699800 2.80732900 -0.04290000
C 0.05249400 3.36371200 0.81039000
H 0.05315000 2.96848700 1.82493500
C 0.99503800 4.46533400 0.57803600
C 1.29960300 5.32663000 1.65212000
C 1.64659500 4.68164700 -0.65251300
C 2.18376500 6.39164000 1.49159500
H 0.82499500 5.15832500 2.61611800
C 2.54072800 5.74150100 -0.80798300
H 1.48542900 3.99580100 -1.47710700
C 2.80553800 6.60612300 0.25762400
H 2.39406500 7.04998100 2.33069400
H 3.03973800 5.88659000 -1.76283800
H 3.50072200 7.43215400 0.13216200
Ru -0.85995400 -0.97545000 -1.19377600
N -1.29092200 -1.50154600 0.85186100

N	1.10072500	-0.83687800	-0.23234000
H	-1.38374400	-0.56217200	1.27578900
S	2.17594100	0.33957400	-0.59545700
O	1.72811000	1.02068500	-1.83784400
O	2.47047800	1.21421300	0.57380800
C	3.75877500	-0.41453500	-1.03329300
C	3.85970300	-1.17212700	-2.20319600
C	4.87972500	-0.21491200	-0.22479200
C	5.08432200	-1.73796100	-2.55352200
C	6.09808600	-0.79062400	-0.58612600
C	6.22305900	-1.55870600	-1.75300000
C	1.12597200	-1.23379300	1.20140500
H	1.00918100	-0.34637200	1.83544900
C	-0.09241600	-2.14759500	1.47199900
H	0.05463500	-3.09426200	0.94699500
C	2.40983500	-1.94606800	1.62218500
C	3.14335300	-1.48399400	2.72190700
C	2.86874700	-3.07941400	0.93400300
C	4.31216700	-2.13505300	3.12726800
H	2.79977600	-0.60396900	3.26036700
C	4.03629000	-3.73049400	1.33446100
H	2.31771400	-3.44474600	0.07138600
C	4.76282300	-3.25978600	2.43331800
H	4.86989700	-1.76010300	3.98184500
H	4.38105000	-4.60540700	0.78883500
H	5.67291100	-3.76634900	2.74447600
C	-0.23880600	-2.42415800	2.96594100
C	-0.62098000	-1.41819400	3.86791900
C	0.05408300	-3.70023100	3.46746500
C	-0.71486500	-1.68955500	5.23479200
H	-0.84745600	-0.41767000	3.50863100
C	-0.03283000	-3.97061200	4.83514800
H	0.35867900	-4.48743800	2.78197700
C	-0.41952000	-2.96504600	5.72365300
H	-1.01562300	-0.90015700	5.91894500
H	0.20086300	-4.96616600	5.20378900
H	-0.49046800	-3.17260900	6.78823200
C	-2.08201600	-2.69832000	-2.13183200
C	-2.73196400	-1.47100500	-2.42482200
C	-2.02056000	-0.43591800	-3.08263000
C	-0.68879500	-0.62108800	-3.54467900
C	-0.05051400	-1.84182100	-3.19716100
C	-0.73038200	-2.88793900	-2.52805200
H	-3.75366000	-1.30684700	-2.10272100

H -2.51365900   0.51494800   -3.25670500
H   0.99173300   -1.98220500   -3.46573800
H -0.21712600   -3.81503100   -2.29658800
C -0.00934900   0.40895500   -4.40417300
H -0.23306000   0.20634600   -5.46081600
H -0.36689600   1.41842600   -4.18048200
H   1.07592200   0.38637200   -4.28241400
C -2.81548400   -3.81385300   -1.41741200
C -2.59254500   -2.18151800   1.12395300
H -2.89682100   -1.97505600   2.15821000
H -3.33016300   -1.70538300   0.47415400
C -2.62228500   -3.70169800   0.97216200
H -2.02671800   -4.18101800   1.75125500
H -3.66806400   -4.01215100   1.11746300
O -2.12240600   -4.23998300   -0.24951500
H   4.80183400   0.38052600   0.67762200
H   5.15699000   -2.32619300   -3.46507100
H   6.96744200   -0.63495000   0.04856200
H   2.99618200   -1.31697500   -2.84340600
C   7.55313200   -2.15052600   -2.14804500
H   8.19731500   -1.39209900   -2.61365000
H   8.09300900   -2.53797800   -1.27663200
H   7.43165400   -2.96588800   -2.86864400
H -1.22516700   0.59906000   -0.55278000
H -3.84169400   -3.51024500   -1.17975100
H -2.87037600   -4.67988900   -2.08768800
C -1.10337800   3.24479700   -1.45909300
H -0.49648300   2.67572000   -2.17319000
H -2.15218500   3.08703300   -1.73576800
H -0.87752300   4.30593300   -1.60057400
C -4.00424300   2.72138800   0.93785000
C -3.54289600   3.36046600   2.10210600
C -5.29543400   3.01267500   0.45983700
C -4.36194200   4.26934900   2.77150200
H -2.56046400   3.13195900   2.49899900
C -6.10518200   3.92770100   1.12568700
H -5.64648100   2.51824900   -0.44058500
C -5.63940600   4.55814600   2.28445900
H -4.00200100   4.75235500   3.67571900
H -7.09759100   4.15117500   0.74384700
H -6.27131000   5.27245700   2.80561600

III-TS3
C 4.64652100   0.78367600   0.63698100

O	4.12918500	1.88084300	0.86160000
C	3.73525900	-0.40546100	0.33130400
O	3.85020800	-0.82976700	-0.85214900
C	2.79877500	-0.88623500	1.29480200
C	2.00949100	-1.97714600	0.85159400
H	2.42716500	-2.46086200	-0.02753900
C	1.31863900	-2.93693300	1.75297400
C	1.51673100	-4.31105700	1.51820200
C	0.51078000	-2.55520500	2.83796100
C	0.94666400	-5.27196100	2.35396300
H	2.13915900	-4.62410800	0.68361600
C	-0.07209100	-3.51687500	3.66395100
H	0.30783300	-1.50702000	3.02100000
C	0.14869300	-4.87710400	3.43084100
H	1.12365700	-6.32698600	2.16160200
H	-0.70276500	-3.20113400	4.49089100
H	-0.30257400	-5.62325300	4.07965600
Ru	-0.06207300	-0.94144200	-1.39631400
N	0.73440400	1.00589700	-0.84389600
N	-1.59886900	-0.18436000	-0.03761700
H	1.29967700	0.76741700	-0.02361100
S	-2.50327900	-1.16632100	0.90008300
O	-2.31084000	-2.56839300	0.45097500
O	-2.30014400	-0.90907000	2.35344100
C	-4.24816400	-0.81996400	0.59004200
C	-4.75785400	-0.95117200	-0.70430400
C	-5.09121400	-0.47306800	1.64808600
C	-6.11372400	-0.72257500	-0.93550100
C	-6.44489500	-0.24519800	1.40065100
C	-6.97960400	-0.36548600	0.10920500
C	-1.27551400	1.12242800	0.59345000
H	-0.70336800	0.96360800	1.51769300
C	-0.37219400	1.90620400	-0.38427200
H	-0.95903700	2.13225000	-1.27671800
C	-2.49730100	1.97391600	0.93981400
C	-2.70370400	2.41227600	2.25357400
C	-3.41827600	2.35379000	-0.04852200
C	-3.80352400	3.21214800	2.57714300
H	-2.00000900	2.12199100	3.02986500
C	-4.51784300	3.15128600	0.27087200
H	-3.28191200	2.01570800	-1.07230100
C	-4.71461200	3.58328000	1.58630700
H	-3.94760800	3.54170600	3.60309800
H	-5.22233300	3.43535100	-0.50685700

H	-5.57161400	4.20392300	1.83548200
C	0.12063900	3.21348100	0.23032900
C	1.04565200	3.22523800	1.28486100
C	-0.37313700	4.43848100	-0.24098300
C	1.47741900	4.43089300	1.84172600
H	1.44372000	2.29336700	1.67433600
C	0.04735400	5.64536300	0.32280900
H	-1.09830000	4.44777200	-1.05099400
C	0.97951500	5.64538900	1.36294900
H	2.20198400	4.41928700	2.65193700
H	-0.34956500	6.58387200	-0.05565000
H	1.31429100	6.58351700	1.79795100
C	0.15367100	-0.63041700	-3.69442400
C	1.21398100	-1.45745100	-3.21206600
C	0.92091100	-2.64552700	-2.50972700
C	-0.42664600	-3.08861000	-2.32066900
C	-1.45579300	-2.22057600	-2.74970200
C	-1.18452500	-1.01560000	-3.45951900
H	2.24610800	-1.15900700	-3.35885000
H	1.73702300	-3.24982300	-2.12589700
H	-2.48565200	-2.49154100	-2.54245700
H	-2.00212400	-0.38991900	-3.80265000
C	-0.71911000	-4.42885700	-1.70389600
H	-0.55558200	-5.22067700	-2.44772000
H	-0.06018100	-4.63551600	-0.85463800
H	-1.75591900	-4.49577500	-1.36510700
C	0.44785100	0.66110700	-4.43276500
C	1.68310000	1.66171600	-1.80243100
H	2.31573500	2.36884900	-1.25196800
H	2.33801300	0.87645000	-2.17991000
C	1.05384900	2.45617700	-2.94728100
H	0.58842900	3.37078100	-2.57495900
H	1.87944400	2.75468600	-3.61069900
O	0.03063600	1.81132900	-3.70075100
H	-4.69258600	-0.37454800	2.65177700
H	-6.50450400	-0.82170800	-1.94525500
H	-7.09669100	0.02914700	2.22710000
H	-4.10773900	-1.21590800	-1.53206900
C	-8.44767400	-0.12466600	-0.14036200
H	-9.06278700	-0.89147900	0.34863000
H	-8.76687400	0.84446600	0.26212400
H	-8.68057300	-0.14173900	-1.20985100
H	0.84579700	-1.42704700	0.01367000
H	1.51359200	0.72449900	-4.68277300

H -0.11956300 0.66752500 -5.36974100
C 2.70068800 -0.28938000 2.67764500
H 1.78631200 0.30262000 2.82110900
H 2.69146200 -1.07100800 3.44735600
H 3.54430100 0.37148800 2.89329600
C 6.11778100 0.61612800 0.53253700
C 6.71376800 -0.64950300 0.39222600
C 6.93318200 1.75838100 0.62101700
C 8.10217800 -0.76544200 0.34135800
H 6.09803800 -1.54028700 0.32297400
C 8.31822600 1.63919100 0.56524700
H 6.46587700 2.73210700 0.73140400
C 8.90449200 0.37596300 0.42632400
H 8.55878300 -1.74545100 0.23473700
H 8.94271800 2.52591300 0.63056600
H 9.98643900 0.28204000 0.38498300

B
N -1.24294300 0.90053000 -0.11111900
N 1.20257200 -0.42144900 -0.31889200
H -1.61095600 1.25483500 -0.99564900
S 2.32291200 -1.25829400 -1.16860200
O 1.96152200 -2.69925000 -1.11621900
O 2.57291400 -0.69717100 -2.52764000
C 3.88930600 -1.16592300 -0.28295500
C 3.96186400 -1.66041900 1.02228500
C 5.03010300 -0.67310200 -0.91897100
C 5.18269200 -1.63972800 1.69502600
C 6.24365000 -0.65812300 -0.23161200
C 6.34147700 -1.13683900 1.08368700
C 1.18844600 1.04169100 -0.60496200
H 0.98196100 1.21079900 -1.66970800
C 0.01793300 1.64754400 0.20581500
H 0.21404200 1.46076800 1.26547400
C 2.48549600 1.76952500 -0.26280000
C 3.17504900 2.47223400 -1.25896000
C 3.00147700 1.77227300 1.04218900
C 4.35743200 3.15730600 -0.96467300
H 2.78744000 2.47703000 -2.27480000
C 4.18261900 2.45331100 1.33908400
H 2.48558900 1.22878700 1.82914600
C 4.86586500 3.14798800 0.33560600
H 4.88005600 3.69437200 -1.75233100
H 4.57000300 2.44275400 2.35479000

H	5.78596300	3.67839300	0.56760800
C	-0.14246100	3.14704700	-0.00848300
C	-0.43970800	3.67252900	-1.27628100
C	0.00145100	4.03158700	1.06898900
C	-0.58916700	5.04844700	-1.45873400
H	-0.55451000	3.00828600	-2.12992800
C	-0.14382700	5.40931000	0.88759200
H	0.23063400	3.63847700	2.05645700
C	-0.43988400	5.92135500	-0.37731700
H	-0.81937200	5.43839400	-2.44685600
H	-0.02766700	6.08012800	1.73488600
H	-0.55457000	6.99259100	-0.52095000
C	-2.27999400	1.13400500	0.94024200
H	-2.39113500	2.21339400	1.08829000
H	4.96901500	-0.30307600	-1.93663200
H	5.23577000	-2.02150100	2.71165200
H	7.12994000	-0.27016300	-0.72845900
H	3.07754300	-2.05328800	1.51534900
C	7.66190200	-1.10975400	1.81285000
H	8.42458800	-1.68664300	1.27445900
H	8.04371900	-0.08479600	1.90344400
H	7.57289400	-1.52641000	2.82114100
H	-0.73080100	-0.82148100	-1.97740900
H	-1.87295100	0.72208800	1.86821400
C	-3.64718300	0.55060900	0.64029400
C	-3.87646500	-0.83064200	0.40534000
C	-4.73243900	1.42696900	0.65201800
C	-5.19411900	-1.25894400	0.19911400
C	-6.04486400	0.97694400	0.44717000
H	-4.57738200	2.48801200	0.82713600
C	-6.28027300	-0.38114400	0.22051200
H	-5.38260700	-2.31444400	0.02293600
H	-7.28181400	-0.76386600	0.06098500
O	-7.01293800	1.94066300	0.48927600
C	-8.36996600	1.54598300	0.28145200
H	-8.95903500	2.46203200	0.35906200
H	-8.50956200	1.10713500	-0.71374200
H	-8.70126000	0.83466000	1.04712100
C	-2.77726400	-1.82633700	0.33397800
C	-1.91564500	-2.24787900	1.43921600
C	-2.46204800	-2.62891400	-0.82195500
C	-1.01454400	-3.19848000	0.92215700
C	-1.31016400	-3.42255600	-0.48315800
C	-2.03900700	-1.83378200	2.87836500

H -2.76542500 -1.02592000 3.00253500
H -2.38936300 -2.67548900 3.49210200
H -1.08544200 -1.50259200 3.30510200
C 0.05225200 -3.90798600 1.69861300
H -0.30105900 -4.89903000 2.01820300
H 0.95231000 -4.06419200 1.09636300
H 0.33320500 -3.35231600 2.59876800
C -0.74280600 -4.53055400 -1.32238600
H -0.77257000 -4.28918200 -2.38959500
H 0.29414900 -4.75283500 -1.05872000
H -1.32286700 -5.45421800 -1.17922600
C -3.23732900 -2.70565400 -2.10585800
H -2.58631500 -2.93505700 -2.95457400
H -3.99526300 -3.49971500 -2.04907800
H -3.75817800 -1.76819500 -2.32043100
Rh -0.84075200 -1.17783800 -0.44030400

1zv
C -1.92113700 -0.45798900 1.01716400
O -1.81001600 0.14174000 2.08036000
C -0.69225100 -1.26381800 0.55783700
O -0.81584700 -2.48556900 0.55985100
C -3.19437100 -0.54651400 0.26094500
C -3.28023400 -1.19819500 -0.98146300
C -4.33648600 0.07207800 0.79999300
C -4.49196600 -1.22813900 -1.67165900
H -2.40860100 -1.68424300 -1.40717600
C -5.54460200 0.03621900 0.11064100
H -4.25718400 0.57602600 1.75809400
C -5.62336800 -0.61397700 -1.12702300
H -4.55414300 -1.73221900 -2.63182700
H -6.42491400 0.51337000 0.53215300
H -6.56652000 -0.64021000 -1.66613100
C 0.58513800 -0.54592500 0.28952600
C 1.69748100 -1.32666700 0.23712700
H 1.52428400 -2.37796400 0.46469800
C 3.09154000 -1.00596300 -0.06710000
C 3.50882300 0.13601300 -0.78385600
C 4.07480300 -1.92926300 0.34862300
C 4.85890200 0.34851900 -1.05223300
H 2.77492700 0.84396400 -1.14973100
C 5.42613500 -1.70497300 0.09402800
H 3.76570800 -2.82423100 0.88294200
C 5.82307700 -0.56298600 -0.60725900

H 5.16130500 1.22791500 -1.61442000
H 6.16691900 -2.42344400 0.43403400
H 6.87520100 -0.38821400 -0.81547300
C 0.53762000 0.93622200 0.13007200
C -0.17392600 1.51801700 -0.93177300
C 1.17185200 1.77793400 1.05992700
C -0.23713600 2.90767800 -1.06953700
H -0.67046500 0.88079700 -1.65908700
C 1.10678700 3.16443300 0.92250200
H 1.71184100 1.33996000 1.89477200
C 0.40332700 3.73425000 -0.14400400
H -0.78644100 3.34144600 -1.90099800
H 1.60149000 3.80124900 1.65132300
H 0.35345500 4.81469200 -0.24973800

IV-TS1
H 0.20248900 -0.68463100 0.29900500
N 0.83130000 1.33611000 -1.31663400
N -1.51325900 1.62595700 0.13955700
H 1.50017800 1.04849100 -0.58692900
S -2.30891900 1.25888800 1.51705800
O -2.96880300 -0.05873300 1.32616400
O -1.45672800 1.40260600 2.73309600
C -3.67234000 2.42111400 1.74290600
C -4.70014300 2.46651200 0.79795000
C -3.72827800 3.22033400 2.88668600
C -5.78170700 3.32289200 1.00112300
C -4.81501700 4.07396700 3.07430100
C -5.85795400 4.14144000 2.13804500
C -0.46777200 2.67250300 0.31628500
H 0.21406100 2.39030400 1.12593500
C 0.35087200 2.72127700 -0.99141000
H -0.32779900 2.99242300 -1.80527100
C -1.03053900 4.05428600 0.63919900
C -0.59415700 4.73676900 1.78153100
C -1.97985000 4.66885700 -0.19123800
C -1.09609200 6.00336100 2.09441200
H 0.14040500 4.27066900 2.43385900
C -2.48387200 5.93260600 0.11861900
H -2.33654100 4.15091400 -1.07767100
C -2.04485100 6.60390700 1.26456300
H -0.74833400 6.51629100 2.98766000
H -3.22166000 6.39396300 -0.53321100
H -2.44016700 7.58704100 1.50712500

C	1.47849200	3.74613500	-0.94477600
C	2.55483500	3.62109800	-0.05157100
C	1.44513300	4.84873400	-1.80926100
C	3.56849800	4.58131800	-0.02683600
H	2.60852800	2.77172700	0.62378000
C	2.45631300	5.81222300	-1.78139800
H	0.61829800	4.95482600	-2.50773100
C	3.52221200	5.68047200	-0.88899600
H	4.39561200	4.47079600	0.66990700
H	2.41117900	6.66091900	-2.45920500
H	4.31183300	6.42718000	-0.86641000
C	1.49987900	1.32617000	-2.65347400
H	2.40856500	1.93107300	-2.58600200
H	-2.93344300	3.17362300	3.62284400
H	-6.57997200	3.35467400	0.26353600
H	-4.85314500	4.69472600	3.96659600
H	-4.66109100	1.84498900	-0.09064400
C	-7.02304800	5.07520300	2.35208000
H	-7.50321300	4.89906300	3.32277800
H	-6.69578700	6.12308900	2.34508900
H	-7.78162000	4.95625100	1.57185400
H	0.82180700	1.85186400	-3.33200800
C	1.87446600	-0.01471100	-3.26079600
C	0.95768300	-1.07329100	-3.50430800
C	3.19283100	-0.15462800	-3.69472800
C	1.41285400	-2.20390800	-4.19482000
C	3.63420200	-1.30538300	-4.36420700
H	3.91538100	0.63640900	-3.51359400
C	2.73147300	-2.33596300	-4.63422600
H	0.71546500	-3.01286100	-4.39414000
H	3.03409500	-3.23258600	-5.16257400
O	4.95770400	-1.31785800	-4.70094800
C	5.48329500	-2.48067000	-5.34427700
H	6.54787000	-2.28405100	-5.48595400
H	5.35551000	-3.37240800	-4.71971600
H	5.01114000	-2.64361100	-6.32046900
C	-0.42652800	-1.07652400	-2.97090200
C	-1.50772600	-0.14403000	-3.27526100
C	-0.93792800	-2.05276500	-2.04069400
C	-2.62642300	-0.51017000	-2.46664400
C	-2.27895400	-1.66636900	-1.68085400
C	-1.52515900	0.89995100	-4.35095000
H	-0.54680800	1.00690200	-4.82608100
H	-2.23770700	0.60875800	-5.13499900

H	-1.84185400	1.88022500	-3.97841100
C	-3.95954700	0.16570800	-2.49276700
H	-4.59603600	-0.29914800	-3.25930700
H	-4.47782300	0.07050600	-1.53507800
H	-3.86969100	1.22763800	-2.74032700
C	-3.19753800	-2.47151000	-0.81121500
H	-2.67145800	-2.87495700	0.05983400
H	-4.04016200	-1.87725300	-0.44984600
H	-3.60620700	-3.32202100	-1.37520800
C	-0.25491400	-3.29168500	-1.55789500
H	-0.47850000	-3.48678000	-0.50568100
H	-0.61576800	-4.15497100	-2.13517600
H	0.82744800	-3.23708500	-1.68330500
Rh	-0.79461700	-0.03110500	-1.11562600
C	1.47019500	-0.69696800	1.32353900
C	0.56557900	-1.09016900	2.53078400
O	-0.41501700	-1.81325800	2.36283100
C	2.26375500	-1.84007400	0.72444900
C	2.02967000	-3.18893600	1.04240100
C	3.33005900	-1.52107200	-0.13315700
C	2.84242200	-4.19092400	0.50724200
H	1.21933900	-3.46481800	1.70807200
C	4.12711800	-2.52375700	-0.68138900
H	3.53313200	-0.47913400	-0.35666200
C	3.88661900	-3.86463200	-0.36072300
H	2.65438900	-5.22914900	0.76804500
H	4.94183600	-2.25984200	-1.35094300
H	4.51350500	-4.64710800	-0.78021200
C	0.95857300	-0.58948100	3.88562300
C	0.02078300	-0.75797800	4.85733800
H	-0.92323900	-1.17953000	4.51663200
C	0.04860200	-0.45653300	6.28870600
C	1.22349400	-0.31031900	7.05729500
C	-1.19520300	-0.35178500	6.94780100
C	1.14780400	-0.05240300	8.42386800
H	2.19508900	-0.42430700	6.59188500
C	-1.26742500	-0.07591600	8.31125900
H	-2.10924100	-0.48193900	6.37367900
C	-0.09409500	0.07588200	9.05498100
H	2.06342600	0.04610900	9.00103300
H	-2.23662700	0.01188900	8.79484200
H	-0.14495700	0.28293200	10.12068400
C	2.27156800	0.08863400	4.09368600
C	3.47755600	-0.62567300	4.03255500

C	2.31293700	1.46858900	4.35461300
C	4.69809900	0.02399600	4.23641400
H	3.45989300	-1.69353200	3.82989700
C	3.53167300	2.11661100	4.55496100
H	1.38217700	2.02939800	4.38710500
C	4.72891900	1.39561400	4.49813800
H	5.62425900	-0.54338400	4.19004400
H	3.54768400	3.18549200	4.75280800
H	5.67855700	1.90090000	4.65437900
O	1.94479300	0.47193200	1.27361000

IV-TS2

H	0.21658500	-0.48601600	0.24870000
N	0.86317200	1.51273200	-1.72152900
N	-1.34968300	1.85297600	-0.04379400
H	1.65057100	1.31880200	-1.09940700
S	-2.05841500	1.61406700	1.41129800
O	-2.66940600	0.25923800	1.39565300
O	-1.14790100	1.93119300	2.54675200
C	-3.45091200	2.75057700	1.59925400
C	-4.53856100	2.67462200	0.72675000
C	-3.45542600	3.66726100	2.65368300
C	-5.62749100	3.52654500	0.90980900
C	-4.54995100	4.51447200	2.82203200
C	-5.65252400	4.46076600	1.95591300
C	-0.32588600	2.93211600	-0.05478400
H	0.44580400	2.73562300	0.69968800
C	0.34694100	2.89955100	-1.44370100
H	-0.43374400	3.06219800	-2.19197700
C	-0.89364300	4.32415500	0.21656300
C	-0.38531800	5.09457300	1.26992300
C	-1.91635100	4.86195900	-0.57934100
C	-0.88685100	6.37348300	1.52797700
H	0.40406900	4.68774700	1.89708800
C	-2.41909100	6.13852900	-0.32447700
H	-2.33219100	4.27385400	-1.39318200
C	-1.90636100	6.89867800	0.73175300
H	-0.48211600	6.95526600	2.35234800
H	-3.21311300	6.54059700	-0.94880200
H	-2.29981600	7.89212200	0.93145200
C	1.40820100	3.97867600	-1.62138500
C	2.53799400	4.04855900	-0.79218300
C	1.26198700	4.93273200	-2.63792300
C	3.49610500	5.04689500	-0.97798800

H	2.67183000	3.32778700	0.00865800
C	2.21790200	5.93414200	-2.82387000
H	0.39106100	4.89246500	-3.28758800
C	3.33985900	5.99290800	-1.99477600
H	4.36453500	5.08723800	-0.32542300
H	2.08526400	6.66516000	-3.61729800
H	4.08617800	6.77000800	-2.13777500
C	1.36236800	1.44602700	-3.13351300
H	2.26566800	2.05837100	-3.19896400
H	-2.61437100	3.71574600	3.33597500
H	-6.47100800	3.46452100	0.22653400
H	-4.54677500	5.22823100	3.64272900
H	-4.54227200	1.96381600	-0.09160800
C	-6.83126000	5.37980900	2.15701100
H	-7.35457500	5.15438700	3.09548500
H	-6.51207800	6.42775000	2.21461500
H	-7.55501900	5.28994200	1.34068300
H	0.60254600	1.94337800	-3.74269500
C	1.66691500	0.09205100	-3.74536000
C	0.73268700	-0.97368100	-3.82416400
C	2.89762600	-0.04065100	-4.38809300
C	1.07756900	-2.10932000	-4.56828300
C	3.22659900	-1.18855600	-5.12439500
H	3.63019300	0.76063600	-4.34159000
C	2.30279200	-2.23127200	-5.22567200
H	0.36469700	-2.92542900	-4.64435700
H	2.51827500	-3.12822900	-5.79459800
O	4.46256200	-1.18260300	-5.70423900
C	4.84805500	-2.31173300	-6.49002300
H	5.85003800	-2.08500000	-6.85970000
H	4.88174700	-3.22609700	-5.88564400
H	4.17040200	-2.45623600	-7.33971700
C	-0.55202400	-0.96933600	-3.08753600
C	-1.66390900	-0.03421700	-3.23436600
C	-0.93041300	-1.95332800	-2.11557100
C	-2.67404000	-0.42708500	-2.30144200
C	-2.21845400	-1.57770400	-1.57610600
C	-1.83634800	1.00587400	-4.30185400
H	-0.98315400	1.01994100	-4.98489700
H	-2.72888900	0.77749000	-4.89958400
H	-1.97337700	2.01396000	-3.89437000
C	-4.01395500	0.22188700	-2.17910600
H	-4.69418600	-0.19905500	-2.93361400
H	-4.46387300	0.04395200	-1.19977700

H	-3.96046400	1.30024700	-2.35496500
C	-3.00261600	-2.39008700	-0.59321100
H	-2.38569400	-2.67211400	0.26547400
H	-3.87721600	-1.84680100	-0.22702900
H	-3.35884500	-3.31682800	-1.06497400
C	-0.21173300	-3.21774500	-1.77589500
H	-0.27195400	-3.42431400	-0.70444300
H	-0.69157400	-4.05362500	-2.30507300
H	0.83613600	-3.19006400	-2.07963200
Rh	-0.69208100	0.10768900	-1.18361800
C	1.91582000	-2.02230900	0.38345500
C	0.67666600	-1.55033000	1.21726900
O	-0.25947500	-2.40517300	1.33658900
C	2.58734900	-3.28471600	0.82543800
C	2.15693700	-4.04223900	1.92981300
C	3.71781600	-3.71007700	0.10245600
C	2.84514400	-5.19822200	2.29759700
H	1.28171400	-3.73742700	2.49098800
C	4.39948800	-4.86577700	0.47098900
H	4.04985100	-3.12264400	-0.74785600
C	3.96446000	-5.61254500	1.57155500
H	2.50384600	-5.77687500	3.15153800
H	5.26992500	-5.18610300	-0.09512100
H	4.49689200	-6.51451500	1.86184500
C	1.08085400	-0.73986600	2.46004800
C	0.22602400	-0.79167100	3.50616600
H	-0.66241100	-1.40090500	3.34842800
C	0.30467500	-0.16241600	4.83152700
C	0.99989500	1.03543800	5.09544500
C	-0.39766000	-0.76804900	5.89295700
C	1.01194000	1.58366600	6.37684600
H	1.51205400	1.55005000	4.29007800
C	-0.37470500	-0.22512900	7.17706800
H	-0.96066500	-1.67894800	5.70262800
C	0.33389200	0.95321600	7.42536300
H	1.54811900	2.51214000	6.55694300
H	-0.91587700	-0.71732600	7.98116900
H	0.34926000	1.38320300	8.42346600
C	2.45117900	-0.15281600	2.51711000
C	3.39221000	-0.69847700	3.41112800
C	2.86447500	0.89104000	1.67556500
C	4.69514000	-0.20370700	3.47122700
H	3.09743500	-1.52045200	4.05745800
C	4.16945800	1.38598100	1.73291600

H	2.15059200	1.32659000	0.98640800
C	5.08960400	0.84151700	2.63139400
H	5.40445700	-0.63998600	4.17011700
H	4.46704100	2.19728800	1.07345400
H	6.10558100	1.22526800	2.67452000
O	2.32885500	-1.41120800	-0.59668900

IV-TS3

C	5.25158800	0.59479700	0.70892400
O	5.13866900	1.64067200	1.34924000
C	3.98732600	-0.20026600	0.36536300
O	3.74978900	-0.29385200	-0.86468100
C	3.14174600	-0.71752500	1.40407300
C	2.11156600	-1.58419200	0.97677000
H	2.27131500	-1.96148800	-0.03032500
C	1.47478000	-2.57503700	1.89525500
C	1.81306500	-3.93002800	1.72150400
C	0.60101300	-2.23004300	2.93726900
C	1.30495800	-4.90985700	2.57626600
H	2.49399000	-4.21339300	0.92314300
C	0.08410100	-3.21359900	3.78360500
H	0.30857100	-1.19628300	3.07797900
C	0.43471800	-4.55496300	3.60982000
H	1.58671000	-5.94945400	2.42972800
H	-0.59701500	-2.92775200	4.58125600
H	0.03119200	-5.31666000	4.27193200
H	0.84738000	-0.77903900	0.40456200
C	6.55465100	0.14089000	0.16209800
C	6.70974600	-1.11654800	-0.44785300
C	7.67158100	0.98313500	0.30574800
C	7.96301300	-1.51898100	-0.90702700
H	5.85808900	-1.77876000	-0.56312500
C	8.91931700	0.58002000	-0.15928200
H	7.54403600	1.95022600	0.78268700
C	9.06658300	-0.67298800	-0.76548400
H	8.07949300	-2.49212900	-1.37570500
H	9.77790100	1.23669400	-0.04946300
H	10.04172300	-0.98913900	-1.12643300
C	3.47656800	-0.46469600	2.83780600
C	2.67724000	0.33910200	3.66659100
C	4.63974900	-1.03587200	3.38510300
C	3.02846500	0.56184400	5.00017800
H	1.77666700	0.79537200	3.26812800
C	4.99432300	-0.80857200	4.71654300

H	5.26738800	-1.66827000	2.76173000
C	4.18827800	-0.00898500	5.53026600
H	2.39492400	1.18752100	5.62422100
H	5.89699500	-1.26254300	5.11804800
H	4.46076600	0.16722300	6.56772100
N	1.07042200	1.30621300	-1.29031600
N	-0.96680700	1.27813900	0.61505800
H	1.96353900	1.04305000	-0.86685400
S	-1.65654500	0.83654600	2.02749800
O	-2.28574900	-0.49567700	1.83131000
O	-0.73350400	0.95595100	3.19511200
C	-3.03408900	1.94685000	2.38093100
C	-4.09589500	2.02847900	1.47688800
C	-3.06196800	2.67346000	3.57322200
C	-5.18102300	2.85446500	1.76684000
C	-4.15380300	3.49651000	3.84771600
C	-5.22894200	3.60337500	2.95226100
C	0.05410600	2.35574700	0.72654000
H	0.88881500	2.02001700	1.35519600
C	0.59979200	2.60259900	-0.69753500
H	-0.24021000	2.91732700	-1.32297800
C	-0.46432700	3.66972700	1.30901500
C	0.13021400	4.20549000	2.45837100
C	-1.51262100	4.37836000	0.70174000
C	-0.31133100	5.41806700	2.99516500
H	0.94530200	3.66946300	2.93791400
C	-1.95694400	5.58863600	1.23589200
H	-1.99472200	3.97892000	-0.18642900
C	-1.35808600	6.11260900	2.38606400
H	0.16275500	5.81609600	3.88887600
H	-2.77163100	6.12381800	0.75446400
H	-1.70461900	7.05514500	2.80225900
C	1.67421500	3.68097200	-0.74170600
C	2.86481300	3.54228300	-0.01392200
C	1.49014600	4.83256200	-1.51916500
C	3.84908400	4.53075500	-0.06288600
H	3.03107800	2.66337200	0.60085400
C	2.47181600	5.82602200	-1.56584200
H	0.57184600	4.95426500	-2.08883400
C	3.65507200	5.67735600	-0.83835700
H	4.76686500	4.39932800	0.50478000
H	2.31172900	6.71376800	-2.17242300
H	4.42044100	6.44836900	-0.87527000
C	1.30343500	1.48046600	-2.75817900

H	2.07254400	2.24704800	-2.89322300
H	-2.23841200	2.60197400	4.27496800
H	-6.00413100	2.91760900	1.05933500
H	-4.16943400	4.06383700	4.77559000
H	-4.07891000	1.46357500	0.55067900
C	-6.40105900	4.50098600	3.26252000
H	-6.89212500	4.20203400	4.19726900
H	-6.08052300	5.54280500	3.38946200
H	-7.14951900	4.47250700	2.46428300
H	0.37546500	1.88925100	-3.16753500
C	1.71918600	0.25759400	-3.55039400
C	0.95407800	-0.93499700	-3.62060400
C	2.87439300	0.36806700	-4.32394500
C	1.38079500	-1.94959600	-4.48693100
C	3.28643400	-0.66007700	-5.18436600
H	3.47816400	1.27002500	-4.27927500
C	2.52750700	-1.82937000	-5.27405100
H	0.79794400	-2.86377900	-4.55596800
H	2.81219200	-2.64122000	-5.93290600
O	4.43348000	-0.41920700	-5.88657000
C	4.90934200	-1.43471900	-6.77199900
H	5.81522300	-1.03012200	-7.22795500
H	5.15640000	-2.35393300	-6.22763400
H	4.17665500	-1.65614300	-7.55700700
C	-0.22982300	-1.17938300	-2.76201700
C	-1.49313900	-0.44910600	-2.75940900
C	-0.33930800	-2.26338600	-1.82370900
C	-2.31777300	-1.03932500	-1.76076400
C	-1.60743700	-2.13198200	-1.14658200
C	-1.92776900	0.61287600	-3.72575800
H	-1.13358800	0.85612600	-4.43621800
H	-2.78976800	0.26044700	-4.30822900
H	-2.23815700	1.53605400	-3.22309500
C	-3.72321100	-0.62501200	-1.45964800
H	-4.42233700	-1.20675900	-2.07761100
H	-3.98534300	-0.80326600	-0.41372100
H	-3.88787300	0.43254500	-1.68675800
C	-2.17182400	-3.12148600	-0.17066800
H	-1.41635400	-3.45688700	0.54664000
H	-3.00918300	-2.70132100	0.39153800
H	-2.54100500	-4.01141500	-0.70053300
C	0.61870800	-3.39670500	-1.64693500
H	0.59834400	-3.78420200	-0.62604100
H	0.33401200	-4.22510500	-2.31120500

H 1.64442000 -3.11537000 -1.90114200
Rh-0.29974900 -0.27739500 -0.77100100

7d’
C -1.89047600 0.30211500 1.15500700
O -2.13102000 1.32752800 1.78887100
C -0.51087500 -0.34745200 1.40124600
O -0.46398000 -1.54728600 1.61849400
C 0.67134400 0.60143300 1.52245500
C 2.00557100 -0.12060600 1.87220700
H 1.80106900 -0.79482100 2.71103300
C 2.70262900 -0.87986700 0.75938000
C 2.43372700 -2.23555900 0.51028300
C 3.66718100 -0.24009300 -0.03556000
C 3.09826100 -2.92454000 -0.50741000
H 1.70013500 -2.75468500 1.12000200
C 4.33400700 -0.92559600 -1.05368500
H 3.90398300 0.80488600 0.15090200
C 4.04936500 -2.27175100 -1.29574100
H 2.87552500 -3.97482800 -0.67990100
H 5.07919900 -0.40906800 -1.65385200
H 4.56852300 -2.80856000 -2.08577900
H 2.68319100 0.65421900 2.24723000
C -2.88245000 -0.35020300 0.27270900
C -2.56045700 -1.43766900 -0.56057900
C -4.18792400 0.17653400 0.25087700
C -3.53290600 -1.98506900 -1.39611700
H -1.56007300 -1.85499700 -0.56196000
C -5.15526200 -0.37882000 -0.57888500
H -4.42664800 1.01924400 0.89235500
C -4.82822300 -1.46080400 -1.40480300
H -3.27989800 -2.82223900 -2.04040000
H -6.16240200 0.02831400 -0.58699100
H -5.58357000 -1.89301200 -2.05571500
C 0.74520400 1.55624300 0.33823000
C 0.96493400 2.92419100 0.55610100
C 0.61113000 1.09814800 -0.98176900
C 1.05759900 3.81285500 -0.51630400
H 1.06304300 3.29404500 1.57420300
C 0.69357100 1.98819600 -2.05606200
H 0.44410200 0.04187200 -1.17730400
C 0.91995200 3.34690000 -1.82700200
H 1.23097600 4.86926000 -0.32756300
H 0.58364900 1.61654800 -3.07162500

H 0.98617900 4.03879000 -2.66264800
H 0.41759600 1.21155700 2.40163800

IV-TS4
H 0.17774800 -0.55883100 0.29738400
N 0.79298400 1.46601700 -1.21650400
N -1.61193300 1.58849800 0.16561000
H 1.45727000 1.21256500 -0.46577600
S -2.44326000 1.15868600 1.50273900
O -2.99865600 -0.20140900 1.27993400
O -1.65231800 1.35030600 2.75392200
C -3.88920700 2.22524000 1.67626900
C -4.86044500 2.24113500 0.66929000
C -4.07252900 2.96512600 2.84425900
C -6.01062800 3.00686100 0.83807500
C -5.23247700 3.72791200 2.99912000
C -6.21737400 3.76365800 2.00403800
C -0.64126500 2.69566200 0.39050900
H 0.03164600 2.44328900 1.21707400
C 0.21393900 2.81470000 -0.88912100
H -0.45302200 3.05712900 -1.72157900
C -1.29786700 4.03403100 0.71673900
C -0.95395600 4.70982100 1.89421500
C -2.24038400 4.61659200 -0.14381300
C -1.53653700 5.94048100 2.20989000
H -0.22884500 4.26578100 2.57184900
C -2.82439400 5.84489000 0.16853200
H -2.52980000 4.10192300 -1.05620200
C -2.47452300 6.51119900 1.34747400
H -1.25876900 6.44889400 3.12980700
H -3.55503400 6.28249700 -0.50726000
H -2.93167800 7.46676500 1.59147800
C 1.27465200 3.90498600 -0.78968100
C 2.30172800 3.84908000 0.16623500
C 1.23310100 4.99396200 -1.67091400
C 3.26089700 4.86144600 0.23491800
H 2.35830700 3.01122600 0.85531300
C 2.19179000 6.00776800 -1.60207700
H 0.44363300 5.04794800 -2.41692100
C 3.20838100 5.94481500 -0.64683400
H 4.05000600 4.80456400 0.98049700
H 2.14290100 6.84390100 -2.29504600
H 3.95627500 6.73167300 -0.59161300
C 1.50839400 1.52089000 -2.52950300

H	2.39092100	2.15489700	-2.40742000
H	-3.32451600	2.94125900	3.62859400
H	-6.76437800	3.01448100	0.05406100
H	-5.37287100	4.29887400	3.91361400
H	-4.72395800	1.66602200	-0.23984800
C	-7.46098600	4.60100200	2.16649400
H	-7.63213500	4.86445900	3.21532000
H	-7.38223600	5.53830100	1.59902800
H	-8.34786800	4.07504500	1.79476500
H	0.84010400	2.04611700	-3.21766200
C	1.95548300	0.21751600	-3.16995000
C	1.09613200	-0.87690600	-3.45872500
C	3.28393000	0.15992300	-3.59184700
C	1.61758300	-1.96029300	-4.17821100
C	3.79025100	-0.94066200	-4.29846400
H	3.96121800	0.98127900	-3.37537300
C	2.94548900	-2.00927700	-4.60671200
H	0.96569800	-2.79658300	-4.41452800
H	3.29905000	-2.87121600	-5.16066800
O	5.11254300	-0.86749200	-4.63361400
C	5.69924500	-1.97234600	-5.32384300
H	6.75048200	-1.71131800	-5.46143400
H	5.62732400	-2.89401800	-4.73448400
H	5.23200800	-2.12347100	-6.30423800
C	-0.29747800	-0.95403800	-2.95919800
C	-1.39531700	-0.03589400	-3.24549600
C	-0.81212300	-1.99911700	-2.10980300
C	-2.52699800	-0.48131100	-2.49991000
C	-2.17417200	-1.67228000	-1.77346100
C	-1.41404200	1.06958000	-4.25873400
H	-0.45005000	1.16769900	-4.76399400
H	-2.16564900	0.85183500	-5.02950200
H	-1.67766600	2.03864500	-3.82026500
C	-3.88079600	0.14919800	-2.54379900
H	-4.45816400	-0.27590700	-3.37742800
H	-4.44379900	-0.04162700	-1.62681600
H	-3.82058800	1.22967100	-2.70463900
C	-3.11538000	-2.55204800	-1.00778900
H	-2.60134300	-3.09493300	-0.20916300
H	-3.92957900	-1.97946400	-0.55707400
H	-3.56545000	-3.29934200	-1.67717800
C	-0.11511200	-3.25595500	-1.70358100
H	-0.38884100	-3.55862200	-0.68978100
H	-0.41586500	-4.07032500	-2.37841100

H	0.96993100	-3.16175800	-1.76239100
Rh	-0.74340900	0.00290700	-1.06049900
C	1.37626900	-0.58630500	1.27132100
C	0.52822000	-0.97067600	2.52684300
O	-0.38240400	-1.78049400	2.48040500
C	2.18953800	-1.73178400	0.68555800
C	1.93247700	-3.08566100	0.95746700
C	3.30752500	-1.40090000	-0.09821000
C	2.77664100	-4.07971100	0.45700800
H	1.08022200	-3.37197700	1.56225300
C	4.13684300	-2.39515200	-0.61384300
H	3.52919100	-0.35579500	-0.28474700
C	3.87605500	-3.74103600	-0.33408400
H	2.56926000	-5.12200900	0.68516600
H	4.99483600	-2.11976600	-1.22166200
H	4.52922500	-4.51688400	-0.72495100
C	0.96821400	-0.29662700	3.83087700
C	-0.02540500	-0.59729400	4.97995400
H	-1.03918900	-0.43331100	4.60198400
C	0.20903100	0.27363100	6.19621700
C	0.87226300	-0.21787600	7.32963900
C	-0.23114800	1.60723700	6.20751200
C	1.09250100	0.59819300	8.44310500
H	1.21559900	-1.24974500	7.34258500
C	-0.01244100	2.42565000	7.31712300
H	-0.75500600	2.00228100	5.33960400
C	0.65194200	1.92361600	8.44040800
H	1.60602700	0.19608400	9.31316700
H	-0.36522000	3.45414300	7.30707600
H	0.82058500	2.55866500	9.30646400
C	2.40717500	-0.68500200	4.16568800
C	2.73870100	-2.01596300	4.46454700
C	3.42219400	0.28140000	4.18738400
C	4.05084600	-2.36840000	4.78759300
H	1.96706800	-2.78187700	4.44476300
C	4.73489000	-0.06886900	4.51188400
H	3.18131400	1.31499800	3.95216000
C	5.05349700	-1.39519800	4.81308700
H	4.28964200	-3.40393900	5.01671300
H	5.50756400	0.69576500	4.52922900
H	6.07476500	-1.66872100	5.06556000
O	1.89107500	0.57635200	1.25812600
H	0.95780400	0.77832300	3.61947700
H	0.04322100	-1.65476100	5.25539800

IV-TS5

H	-0.09624800	-0.69916200	0.26642500
N	0.71308700	1.21273000	-1.32426400
N	-1.58137400	1.66893400	0.17647600
H	1.41445100	0.82486100	-0.68972000
S	-2.54629000	1.45067500	1.47555100
O	-3.30597000	0.18766300	1.27532100
O	-1.81663100	1.58139100	2.76707600
C	-3.79444100	2.75551800	1.49787700
C	-4.63626900	2.93223200	0.39789700
C	-3.94519300	3.55109900	2.63633900
C	-5.62520500	3.91441900	0.43932200
C	-4.93687500	4.53122600	2.66188500
C	-5.79244900	4.73091500	1.56772100
C	-0.43091900	2.58477700	0.42001800
H	0.19400800	2.18569700	1.22558900
C	0.41168700	2.61579900	-0.87350100
H	-0.20543400	3.04640600	-1.66676000
C	-0.82901700	4.01115800	0.79961100
C	-0.39917300	4.55413100	2.01712400
C	-1.60802300	4.80817900	-0.05309000
C	-0.74163900	5.85941100	2.38094000
H	0.20295500	3.94738100	2.68874200
C	-1.95290500	6.11144200	0.30735100
H	-1.95853300	4.40699500	-1.00032800
C	-1.52193400	6.64183600	1.52741600
H	-0.40066700	6.26144200	3.33177900
H	-2.55962300	6.71369600	-0.36440800
H	-1.79236900	7.65663600	1.80807300
C	1.67725300	3.45232300	-0.72465600
C	2.67424200	3.10548200	0.20170700
C	1.86577400	4.58802500	-1.52404400
C	3.83006700	3.87934600	0.32173900
H	2.55141700	2.22879900	0.83247400
C	3.02039400	5.36499300	-1.40157200
H	1.10220600	4.86725800	-2.24590000
C	4.00669500	5.01188100	-0.47814800
H	4.59270400	3.59745400	1.04314600
H	3.14882500	6.24308200	-2.02926400
H	4.90717800	5.61292500	-0.38203300
C	1.28600800	1.23918600	-2.70786900
H	2.22557700	1.79801700	-2.67588700
H	-3.29182300	3.40849000	3.48981600

H	-6.27647800	4.04898700	-0.42081700
H	-5.04750100	5.15028000	3.54925500
H	-4.51917100	2.32211000	-0.49044200
C	-6.85840500	5.79764300	1.61083000
H	-7.55152700	5.63141000	2.44516000
H	-6.41842100	6.79258500	1.75571900
H	-7.44239800	5.81887500	0.68525600
H	0.58942800	1.83036800	-3.30849800
C	1.55143300	-0.08395800	-3.40306300
C	0.56966100	-1.08913200	-3.61228700
C	2.81769400	-0.25328400	-3.96399000
C	0.90676600	-2.20038800	-4.39532100
C	3.13731600	-1.37527000	-4.74320700
H	3.58909100	0.49714200	-3.81424900
C	2.16870400	-2.35543400	-4.97196300
H	0.15827700	-2.96844600	-4.56918000
H	2.37739300	-3.23057800	-5.57613000
O	4.41165400	-1.41039600	-5.23018000
C	4.80064800	-2.52779700	-6.03171900
H	5.83901600	-2.34081700	-6.31244100
H	4.74008300	-3.46388200	-5.46409000
H	4.18660500	-2.60313100	-6.93680200
C	-0.77252700	-1.03950500	-2.98724100
C	-1.80994200	-0.03741000	-3.21119400
C	-1.30140900	-2.02570100	-2.08184700
C	-2.92225800	-0.38700300	-2.39051100
C	-2.60901800	-1.58368300	-1.65904400
C	-1.80769800	1.05508900	-4.23902000
H	-0.88015700	1.05944500	-4.81721300
H	-2.63351600	0.89981200	-4.94609600
H	-1.94663500	2.04809600	-3.79703300
C	-4.23077100	0.33331900	-2.37399100
H	-4.89686000	-0.10441500	-3.13172200
H	-4.73368600	0.24269700	-1.40829800
H	-4.11266100	1.39324800	-2.61630800
C	-3.55320600	-2.37394200	-0.80446400
H	-3.03801900	-2.84130700	0.03920400
H	-4.35721900	-1.75012100	-0.40641200
H	-4.01680800	-3.17657300	-1.39582000
C	-0.69093000	-3.33940100	-1.71161700
H	-0.92394000	-3.61208700	-0.68007100
H	-1.10338500	-4.12500100	-2.36076500
H	0.39241800	-3.33907800	-1.84183100
Rh	-1.00514000	-0.04476200	-1.07147100

C	-0.42677200	-1.62086900	2.26269200
C	0.75216200	-0.97969200	1.44483800
O	1.26907100	0.11442600	1.81736900
C	-0.64177800	-1.28174200	3.69955800
C	0.27056400	-0.54684300	4.47721000
C	-1.82740600	-1.75027800	4.29888000
C	0.00210700	-0.29761200	5.82335500
H	1.18257400	-0.17320200	4.03160700
C	-2.09605900	-1.48980400	5.63851800
H	-2.53283300	-2.31501700	3.69763600
C	-1.17895400	-0.76274700	6.40584500
H	0.71699600	0.26549600	6.41726300
H	-3.01757600	-1.85114800	6.08693400
H	-1.38565900	-0.55985400	7.45350000
C	1.70593400	-2.11598000	0.91792400
C	2.86321400	-1.53368500	0.07086400
H	2.42699900	-1.04812800	-0.80875200
C	3.87896500	-2.55928700	-0.39390200
C	5.12972100	-2.67096700	0.23238400
C	3.59936400	-3.41797200	-1.46875400
C	6.06681700	-3.61733800	-0.19112300
H	5.37383800	-2.00703500	1.05795900
C	4.53177800	-4.36667500	-1.89475700
H	2.64640700	-3.33788800	-1.98506000
C	5.77020300	-4.47196200	-1.25527600
H	7.02939200	-3.68378700	0.31032000
H	4.29179400	-5.02190400	-2.72884000
H	6.49801900	-5.20818600	-1.58696000
C	2.20205900	-2.90984600	2.12581600
C	3.06560100	-2.34020500	3.07676400
C	1.78635900	-4.23620500	2.31537000
C	3.50735400	-3.07958500	4.17558600
H	3.39314500	-1.31120200	2.96044300
C	2.22741500	-4.97871800	3.41391400
H	1.11301600	-4.69270300	1.59373900
C	3.09054800	-4.40263300	4.34840700
H	4.17757300	-2.62051800	4.89822800
H	1.89589900	-6.00673500	3.53776000
H	3.43524700	-4.97749100	5.20417500
O	-1.13689000	-2.46144800	1.71438100
H	1.11792400	-2.79563900	0.29901500
H	3.37107200	-0.75387100	0.64618900

7d

C	-1.82860600	-1.51007000	0.87747200
C	-0.74206800	-1.47262700	-0.21672000
O	-1.00220600	-1.95171600	-1.31088700
C	-3.09528000	-0.80327300	0.40483100
C	-3.35404200	0.52309900	0.77182100
C	-4.00494800	-1.47501200	-0.42273900
C	-4.50835500	1.16689000	0.31934000
H	-2.65680300	1.04779800	1.41754600
C	-5.15563000	-0.82867900	-0.87848200
H	-3.81709100	-2.50813300	-0.70255700
C	-5.41076300	0.49486200	-0.50878600
H	-4.70087600	2.19544300	0.61403200
H	-5.85676800	-1.36211500	-1.51535600
H	-6.30926600	0.99643600	-0.85886300
C	0.61396000	-0.84686800	0.09323700
C	1.69916500	-1.37756600	-0.88012900
H	1.65172500	-2.47325100	-0.87015200
C	3.09471500	-0.92415300	-0.50860100
C	3.70274500	0.15956800	-1.15898800
C	3.80839100	-1.57426000	0.51065600
C	4.98576600	0.58333700	-0.80215200
H	3.16870500	0.67311300	-1.95514700
C	5.08993900	-1.15342800	0.87140400
H	3.35734400	-2.42231700	1.02202700
C	5.68384900	-0.07130000	0.21527500
H	5.43994300	1.42312600	-1.32254300
H	5.62708000	-1.67358800	1.66087500
H	6.68309000	0.25549800	0.49187200
C	0.46604300	0.67509900	0.05812900
C	-0.01074300	1.32160100	-1.09287300
C	0.81635900	1.45154600	1.17122600
C	-0.12360900	2.71302500	-1.13202400
H	-0.29931800	0.73478800	-1.96167200
C	0.70440200	2.84326100	1.13297200
H	1.17979000	0.96244100	2.07143800
C	0.23459500	3.47876200	-0.01931300
H	-0.49514300	3.19773900	-2.03130900
H	0.98454100	3.43003500	2.00426400
H	0.14625100	4.56169200	-0.04905500
H	0.88260900	-1.13136500	1.11482500
H	1.45879100	-1.05934000	-1.89907000
O	-1.29969200	-0.97624700	2.08070400
H	-1.97109800	-1.08650500	2.77610600
H	-2.05365300	-2.58067500	1.00086200
